# Transcriptional profiles of crossbred embryos derived from yak oocytes *in vitro* fertilized with cattle sperm

**DOI:** 10.1038/s41598-018-29912-7

**Published:** 2018-08-01

**Authors:** Xiang-dong Zi, Shuang Liu, Wei Xia, Xian-rong Xiong, Bin Luo

**Affiliations:** 1 Key Laboratory of Animal Science of State Ethnic Affairs Commission, Southwest Minzu University, Chengdu, 610041 China; 2 Ministry of Education Key Laboratory of Conservation & Utilization of Qinghai-Tibetan Plateau Animal Genetic Resources, Southwest Minzu University, Chengdu, 610041 China

## Abstract

During mammalian pre-implantation embryonic development, dramatic and orchestrated changes occur in gene transcription. Pregnancy rates were low when yak females were crossbred with cattle breeds, but few studies exist to describe the unique molecular network regulation behind the pre-implantation development of these embryos. We determined the transcriptomes of crossbred embryos derived from yak oocytes *in vitro* fertilized with Jersey sperm using Illumina RNA-seq for the first time in this study. Embryos were sampled at the 2-, 4-, and 8-cell, morula and blastocyst stages. The results showed that in total, 291.9 million short reads were generated from the five libraries of 2-, 4-, and 8-cell, morula and blastocyst stages, with 276.2 million high-quality reads selected for further analysis. Eighty to 91% of the clean reads were aligned against the yak reference genome. A total of 19,072 transcripts were identified in five libraries, of which 7,785 transcripts were co-expressed in each stage and 2,013 transcripts were stage-specific. When a |log_2_ ratio| ≥1 and q-value ≤ 0.05 were set as thresholds for identifying differentially expressed genes (DEGs), we detected a total of 3,690 to 10,298 DEGs between any two consecutive stages. Based on the results of GO and KEGG enrichment, some of these DEGs potentially play an important role in regulating pre-implantation development, but they are most likely stage-specific. There were 2,960, 7,287, 6,420, 7,724 and 10,417 DEGs in 2-, 4-, 8-cell, morula and blastocyst stages between the crossbred embryos and purebred embryos of the yak, respectively, leading to a large difference in GO terms and pathways. In conclusion, we sequenced transcriptomes of *in vitro*-produced crossbred embryos of yak and cattle during pre-implantation and provided comprehensive examinations of gene activities. These will be helpful for development of assisted reproductive technology and better understanding the early maternal-fetal or maternal-embryonic dialog in inter-species crossbreeding.

## Introduction

The yak (*Bos grunniens*) is one of the world’s most remarkable domestic animals ‒ an herbivore living in and around the Himalayas and further north at altitudes ranging from 2,500 to 5,500 m with no frost-free period. They are very important to local people for providing milk and meat, as few other domestic animals can survive in such harsh conditions. However, the production performance of yak is inferior to that of improved cattle breeds^1,2^.

The economic traits of F1 hybrids derived from yak females crossbred with improved cattle breeds either by natural mating or artificial insemination are greatly improved. The F1 hybrids derived from dairy cattle breeds produce 100‒300% more milk than the yak, while those derived from beef cattle breeds grow faster and produce 50‒100% more meat than the yak^1^. However, there is a marked difference between the pregnancy rates of purebred service (>70%) compared with crossbred service (<30%)^3^, and the underlying causes of this difference have not been well studied. Mammalian pre-implantation embryonic development is a complex process including fertilization, cleavage divisions, compaction, and blastulation, governed by dramatic and orchestrated changes occurring in gene transcription. Our previous study indicated that the fertilization stage was normal when yak oocytes were fertilized with cattle sperm, but the cleavage rates and blastocyst rates were lower^4^. The development of RNA sequencing technologies permits the study of gene regulation at an unprecedented level. Such studies have been successfully conducted in mouse^5^, pig^6^, cattle^7,8^, human^9^ and yak^10^. However, these data have limited utility in crossbred embryogenesis of the yak due to the large differences in gene expression and genome sequences between different species^11–13^. Here, we provide the first comprehensive description of gene activities during the *in vitro* development of crossbred embryos of yak and cattle.

## Results

### Illumina HiSeq mRNA sequencing

**Figure 1:**
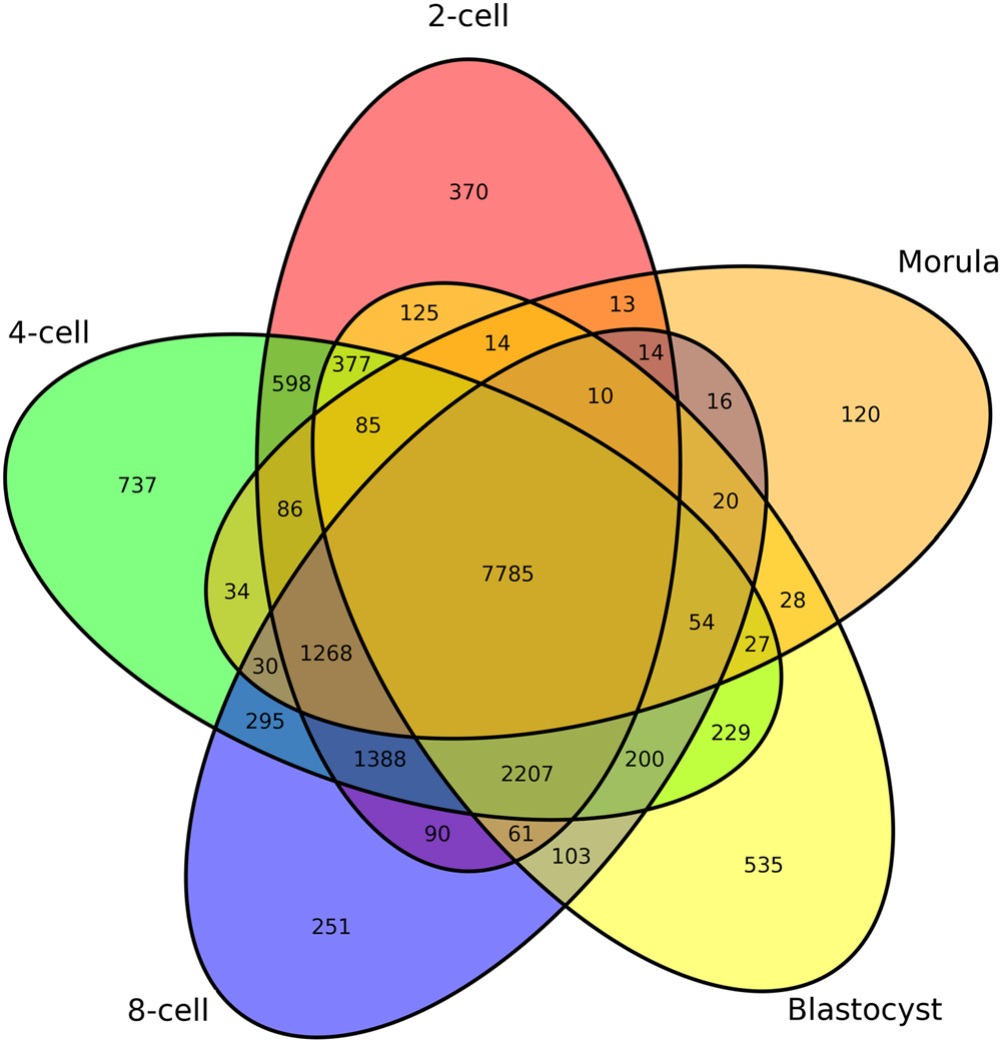
Venn diagram of gene expression during *in vitro* pre-implantation development of yak crossbred embryos.

**Figure 2:**
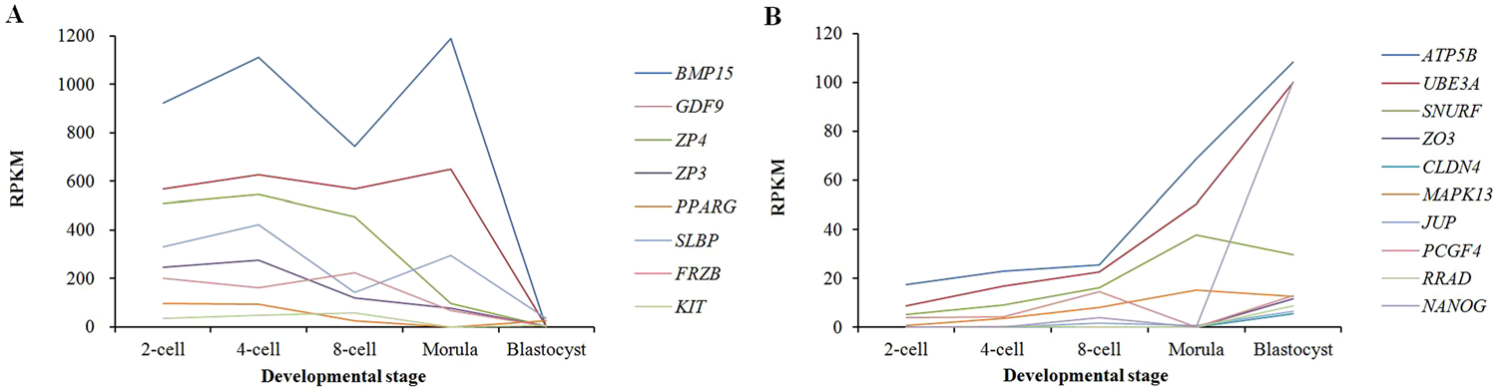
Gene transcripts with maternal or embryonic expression profiles. (**A**) Expression profile of maternal transcripts. (**B**) Expression profile of the embryonic genes.

The average cleavage rates and blastocyst rates after yak oocytes were *in vitro* fertilized (IVF) with Jersey sperm were 78.4% and 36.3%, respectively. In total, 291.9 million short reads were generated from the five libraries of yak crossbred embryos, i.e., the 2-, 4-, and 8-cell, morula and blastocyst stages throughout pre-implantation, with 276.2 million high-quality reads selected for further analysis. Eighty to 91% of the clean reads were aligned against the yak reference genome. A total of 19,072 transcripts were identified in five libraries, of which 7,785 transcripts were co-expressed in each stage. A total of 370, 737, 251, 120 and 535 transcripts were really stage-specific at the 2-, 4-, and 8-cell, morula and blastocyst stages, respectively, and the rest of the genes are common between two, three of four stages (Fig. 1). In total, 9,600 to 15,400 transcripts were detected in each *in vitro* stage, of which, 496, 564, 747, 441 and 519 novel transcripts were detected at the 2-, 4-, and 8-cell, morula and blastocyst stages, respectively. As embryo development proceeded, *BMP15*, *ZP3*, 4, *PPARG*, *SLBP*, *FRZB* and *KIT* (the maternal expression profiles) were decreased (Fig. 2A), whereas *ATP5B*, *UBE3A*, *SNURF*, *ZO3*, *CLDN*4, *MAPK13*, *JUP*, *PCGF4*, *RRAD* and *NANOG* (the embryonic expression profiles) were increased at specific stages (Fig. 2B).

### Differentially expressed genes (DEGs) during pre-implantation development

**Figure 3:**
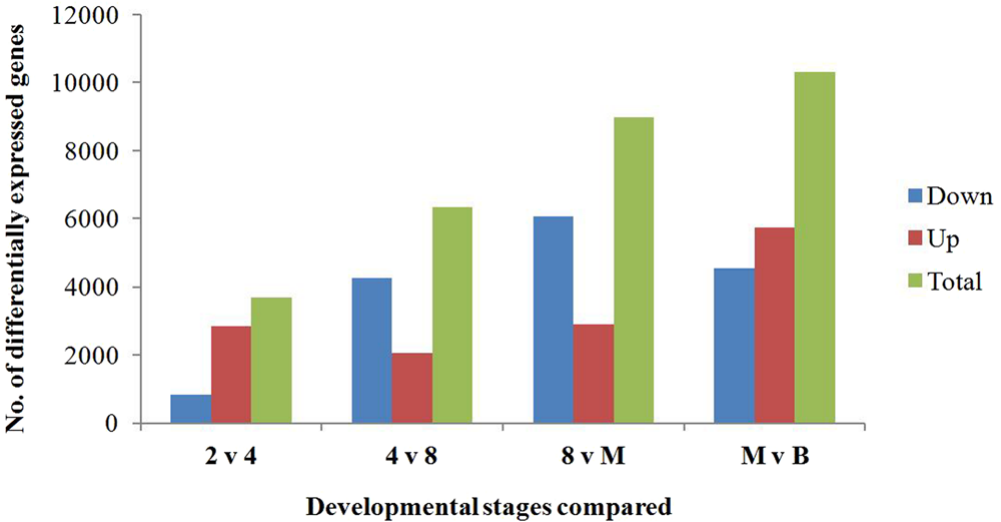
Number of differentially expressed genes during pre-implantation development of yak crossbred embryos. The X axis represents four different stage comparisons of pre-implantation development, including from 2-cell to 4-cell (2 v 4), from 4-cell to 8-cell (4 v 8), from 8-cell to morula (8 v M), and from morula to blastocyst (M v B).

Differences in gene expression at five stages during the pre-implantation development of crossbred embryos were examined, and DEGs were identified by pairwise comparisons of any two consecutive embryonic stages (Fig. 3, Supplementary Table 1). We detected a total of 3,690 to 10,298 DEGs between two consecutive stages. The number of DEGs identified in the comparison of 2- vs. 4-cell, 4- vs. 8-cell, 8-cell vs. morula, and morula vs. blastocyst stages increased with increasing the developmental stages. Four-cell vs. 8-cell stage of development presents the lower number of up-regulated genes, but it increases in the following stages of development. The number of down-regulated DEGs increased from the 2-cell stage to the morula stage but decreased from the morula stage to the blastocyst stage. The number of up-regulated DEGs was higher across the 2- vs. 4-cell stage than the 4- vs. 8-cell stage, but thereafter, it increased with increasing developmental stages.

**Figure 4:**
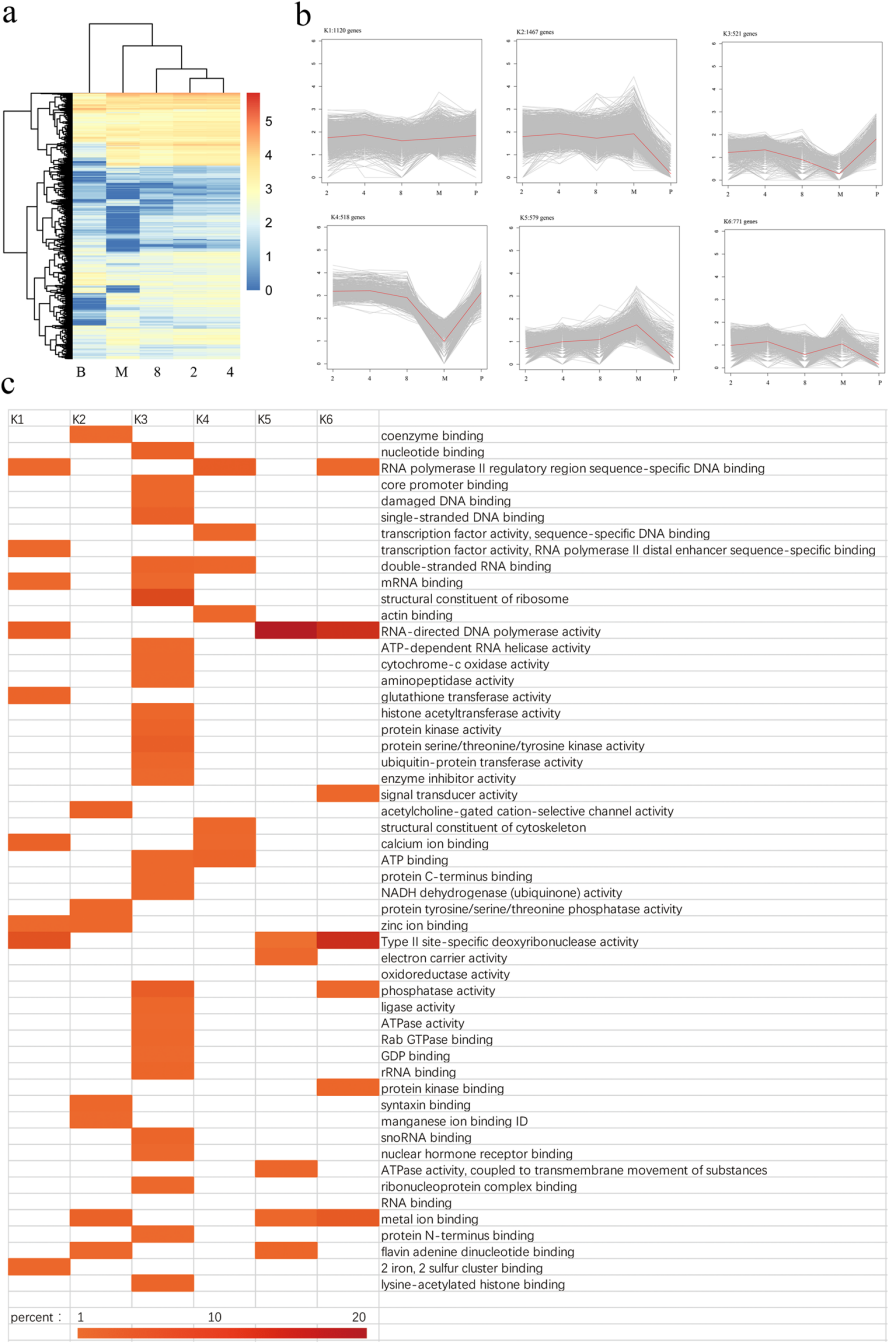
Overview of DEG analysis during the five consecutive stages of yak crossbred pre-implantation development. (**a**) Heat map of DEGs across five developmental stages, including the 2-cell (2), 4-cell (4), 8-cell (8), morula (M), and blastocyst (B) stages. (**b**) Expression patterns of the genes in the six main clusters, namely, K1-K6, corresponding to the heatmap. (**c**) GO-term function enrichment analysis of the different clusters.

We performed hierarchical clustering of all DEGs using the Euclidean distance method associated with complete linkage (Fig. 4a,b). We used the SOTA function in the clValid package to classify DEGs into 12 clusters. Finally, we chose the six clusters with the most significant variations, as shown in Fig. 4b. Six clusters were plotted from the expression patterns. The genes in clusters K1 (1,120 genes) and K2 (1,467 genes) contained most of the DEGs, but the genes in the two clusters had different expression patterns. The genes in K1 were expressed at a relatively steady level throughout the developmental stages, whereas the ones in K2 dramatically decreased at the blastocyst stage. The expression patterns of genes in clusters K3 (521 genes) and K4 (518 genes) were similar, but genes in K4 were expressed at higher levels than those in K3. They were down-regulated as the embryonic developmental stage increased from the 2-cell stage to the morula stage but were dramatically up-regulated at the blastocyst stage. Genes in cluster K5 (579 genes) were up-regulated between the 2-cell stage and the morula stage but dramatically down-regulated at the blastocyst stage. Genes in cluster K6 (771 genes) were slightly down-regulated at the 8-cell stage and up-regulated at the morula stage, then dramatically down-regulated at the blastocyst stage.

We further analysed the overrepresented GO functions within each cluster. The enriched GO terms of biological process are shown in Fig. 4c. The K3 cluster contained the most overrepresented GO terms among all of the clusters. Some gene GO terms were enriched in a particular cluster, such as the structural constituents of ribosomes and protein serine/threonine/tyrosine kinase activities, with protein kinase activities enriched specifically in K3.

### Significantly related GO terms and pathways

We used GO assignments to classify the functions of DEGs in pairwise comparisons of cDNA libraries between different developmental stages of the yak crossbred embryos. In the Biological Process (BP) category, there were 29, 10 and 34 GO terms significantly enriched in the comparisons of 2- vs. 4-cell, 4- vs. 8-cell, and 8-cell vs. morula stages, respectively, but there was no GO term significantly enriched in the morula vs. blastocyst stage (Table 1, Supplementary Table 2). In the 2- vs. 4-cell stage and 4- vs. 8-cell stage, the most significant GO terms were the same, i.e., detection of chemical stimulus and sensory perception of chemical stimulus, but in 8-cell vs. blastocyst stage, the most significant GO terms were multicellular organismal process and single-multicellular organism process. In the Cellular Component (CC) category, there were 19 GO terms significantly enriched in the comparison of 2- vs. 4-cell stage, and the most significant GO terms were extracellular space, intrinsic to membrane, and extracellular region. In the comparison of 4- vs. 8-cell stage, there were 10 GO terms significantly enriched, and the most significant GO terms were plasma membrane, cell periphery and intrinsic to plasma membrane. In the comparison of 8-cell vs. morula stage, there were 12 GO terms significantly enriched, and the most significant GO terms were intrinsic to membrane, integral to membrane and extracellular space. In the morula vs. blastocyst stage, there were 9 GO terms significantly enriched, and the most significant GO terms were cytosolic ribosome, plasma membrane and cell periphery (Table 2, Supplementary Table 3). In the Molecular Function (MF) category, there were 43 GO terms significantly enriched in the comparison of 2- vs. 4-cell stage, and the most significant GO terms were receptor activity, signalling receptor activity, and G-protein coupled receptor activity. In the comparison of 4- vs. 8-cell stage, there were 7 GO terms significantly enriched, and the most significant GO terms were G-protein coupled receptor activity, signalling receptor activity and transmembrane signalling receptor activity. In the comparison of 8-cell vs. morula stage, there were 7 GO terms significantly enriched, and the most significant GO terms were receptor activity, signalling receptor activity and transmembrane signalling receptor activity. In the comparison of morula vs. blastocyst stages, there was only one GO terms significantly enriched, i.e., signalling receptor activity (Table 3, Supplementary Table 4).

**Table 1:** Top significant GO terms of the Biological Process enriched for differentially expressed genes between subsequent stages of yak crossbred embryos.

Stage	GO terms	GO	q-value
2 v 4 (29)	Detection of chemical stimulus	0009593	2.29E-08
	Sensory perception of chemical stimulus	0007606	6.63E-08
	Detection of stimulus involved in sensory perception	0050906	6.63E-08
	Detection of chemical stimulus involved in sensory perception	0050907	6.63E-08
	Sensory perception	0007600	2.89E-07
	Detection of stimulus	0051606	4.82E-07
	System process	0003008	1.17E-05
	Biological adhesion	0022610	3.48E-05
	Cell adhesion	0007155	3.48E-05
	Homophilic cell adhesion	0007156	0.0020
4 v 8 (10)	Detection of chemical stimulus	0009593	0.0018
	Sensory perception of chemical stimulus	0007606	0.0193
	Immune system process	0002376	0.0265
	Detection of chemical stimulus involved in sensory perception	0050907	0.0265
	Inflammatory response	0006954	0.0265
	Defence response	0006952	0.0327
	Multicellular organismal process	0032501	0.0327
	Detection of stimulus involved in sensory perception	0050906	0.0388
	Detection of stimulus	0051606	0.0388
	Single-multicellular organism process	0044707	0.0470
8 v M (34)	Multicellular organismal process	0032501	1.11E-05
	Single-multicellular organism process	0044707	1.11E-05
	Locomotion	0040011	5.85E-05
	Multicellular organismal development	0007275	6.19E-05
	Signalling	0023052	6.19E-05
	Cell communication	0007154	6.19E-05
	Single organism signalling	0044700	6.19E-05
	Response to external stimulus	0009605	8.05E-05
	Cell surface receptor signalling pathway	0007166	0.0010
	System development	0048731	0.0010
M v B	none		

A q-value < 0.05 was identified as significant. The number in the bracket is the count of significant GO terms.

**Table 2:** Top significant GO terms of the Cellular Component enriched for differentially expressed genes between subsequent stages of yak crossbred embryos.

Stage	Description	GO	q-value
2 v 4 (19)	Extracellular space	0005615	7.00E-18
	Intrinsic to membrane	0031224	3.80E-14
	Extracellular region	0005576	5.60E-14
	Integral to membrane	0016021	2.70E-13
	Extracellular matrix	0031012	3.40E-12
	Intrinsic to plasma membrane	0031226	4.80E-10
	Proteinaceous extracellular matrix	0005578	1.20E-09
	Integral to plasma membrane	0005887	4.10E-09
	Extracellular region part	0044421	7.50E-09
	Membrane part	0044425	3.20E-07
4 v 8 (10)	Plasma membrane	0005886	1.00E-08
	Cell periphery	0071944	1.20E-08
	Intrinsic to plasma membrane	0031226	4.70E-08
	Intrinsic to membrane	0031224	8.50E-08
	Integral to plasma membrane	0005887	9.10E-08
	Integral to membrane	0016021	3.00E-07
	Plasma membrane part	0044459	8.40E-07
	Membrane part	0044425	1.10E-05
	Nuclear nucleosome	0000788	2.30E-05
	Extracellular space	0005615	7.00E-05
8 v M (12)	Intrinsic to membrane	0031224	1.60E-12
	Integral to membrane	0016021	1.00E-11
	Extracellular space	0005615	1.40E-09
	Plasma membrane	0005886	8.40E-09
	Membrane part	0044425	1.20E-08
	Cell periphery	0071944	1.70E-08
	Intrinsic to plasma membrane	0031226	2.50E-08
	Integral to plasma membrane	0005887	5.00E-08
	Plasma membrane part	0044459	1.30E-07
	Proteinaceous extracellular matrix	0005578	0.00014
M v B (9)	Cytosolic ribosome	0022626	4.90E-06
	Plasma membrane	0005886	1.10E-05
	Cell periphery	0071944	2.60E-05
	Cytosolic large ribosomal subunit	0022625	3.20E-05
	Intrinsic to membrane	0031224	3.90E-05
	Extracellular space	0005615	5.30E-05
	Extracellular matrix	0031012	8.50E-05
	Integral to membrane	0016021	0.00019
	Integral to plasma membrane	0005887	0.00028

A q-value < 0.05 was identified as significant. The number in the bracket is the count of significant GO terms.

**Table 3:** Top significant GO terms of the Molecular Function enriched for differentially expressed genes between subsequent stages of yak crossbred embryos.

Stage	Description	GO	p value
2 v 4 (43)	Receptor activity	0004872	1.00E-13
	Signalling receptor activity	0038023	1.10E-13
	G-protein coupled receptor activity	0004930	1.70E-13
	Transmembrane signalling receptor activity	0004888	6.80E-13
	Olfactory receptor activity	0004984	2.50E-12
	Molecular transducer activity	0060089	1.80E-11
	Signal transducer activity	0004871	1.80E-11
	Calcium ion binding	0005509	2.90E-07
	Peptidase inhibitor activity	0030414	6.70E-07
	Potassium channel activity	0005267	7.10E-07
4 v 8 (7)	G-protein coupled receptor activity	0004930	6.00E-14
	Signalling receptor activity	0038023	2.10E-11
	Transmembrane signalling receptor activity	0004888	4.60E-11
	Receptor activity	0004872	8.60E-11
	Signal transducer activity	0004871	5.50E-10
	Molecular transducer activity	0060089	5.50E-10
	Olfactory receptor activity	0004984	6.10E-06
8 v M (7)	Receptor activity	0004872	5.30E-14
	Signalling receptor activity	0038023	6.20E-13
	Transmembrane signalling receptor activity	0004888	4.70E-11
	Molecular transducer activity	0060089	4.00E-10
	Signal transducer activity	0004871	4.00E-10
	G-protein coupled receptor activity	0004930	4.90E-10
	Calcium ion binding	0005509	5.50E-05
M v B (1)	Signalling receptor activity	0038023	1.20E-05

A q-value < 0.05 was identified as significant. The number in the bracket is the count of significant GO terms.

Pathways enriched for differentially expressed genes are shown in Table 4. There were 15 significantly enriched pathways in the 2- vs. 4-cell stage. The most significantly enriched pathways were olfactory transduction, ubiquitin-mediated proteolysis, and protein processing in the endoplasmic reticulum. There were no significantly enriched pathways in the 4- vs. 8-cell stage. There were 18 significantly enriched pathways in the 8-cell vs. morula stage. The most significantly enriched pathways were associated with ribosomes, Parkinson’s disease, and oxidative phosphorylation. There was only one significantly enriched pathway in the morula vs. blastocyst stage, i.e., RNA transport.

**Table 4:** Pathways enriched for differentially expressed genes between subsequent stages of yak crossbred embryos.

Stage	Pathways	Map	q-value
2 v 4	Olfactory transduction	04740	1.74E-09
	Ubiquitin-mediated proteolysis	04120	0.0005
	Protein processing in the endoplasmic reticulum	04141	0.0081
	Carbon fixation in photosynthetic organisms	00710	0.0087
	Cell cycle	04110	0.0152
	Spliceosome	03040	0.0176
	RNA transport	03013	0.0236
	Progesterone-mediated oocyte maturation	04914	0.0236
	Epstein-Barr virus infection	05169	0.0236
	Viral carcinogenesis	05203	0.0236
	Cytokine-cytokine receptor interaction	04060	0.0258
	Chronic myeloid leukaemia	05220	0.0258
	Glycolysis / Gluconeogenesis	00010	0.0306
	Microbial metabolism in diverse environments	01120	0.0323
	Pyruvate metabolism	00620	0.0415
	Cytokine-cytokine receptor interaction	04060	0.0258
	Chronic myeloid leukemia	05220	0.0258
	Glycolysis / Gluconeogenesis	00010	0.0306
	Microbial metabolism in diverse environments	01120	0.0323
	Pyruvate metabolism	00620	0.0415
4 v 8	none		
8 v M	Ribosome	03010	1.63E-13
	Parkinson’s disease	05012	1.82E-06
	Oxidative phosphorylation	00190	1.88E-06
	Huntington’s disease	05016	1.52E-05
	RNA transport	03013	8.03E-05
	Neuroactive ligand-receptor interaction	04080	0.0025
	Alzheimer’s disease	05010	0.0034
	Transcriptional misregulation in cancer	05202	0.0036
	Cardiac muscle contraction	04260	0.0043
	Proteasome	03050	0.0062
	Ribosome biogenesis in eukaryotes	03008	0.0066
	Ubiquitin mediated proteolysis	04120	0.0185
	Basal cell carcinoma	05217	0.0185
	ABC transporters	02010	0.0185
	Systemic lupus erythematosus	05322	0.0195
	Amoebiasis	05146	0.0202
	ECM-receptor interaction	04512	0.0294
	Steroid hormone biosynthesis	00140	0.0406
	Ribosome biogenesis in eukaryotes	03008	0.0066
	Ubiquitin mediated proteolysis	04120	0.0185
	Basal cell carcinoma	05217	0.0185
	ABC transporters	02010	0.0185
	Systemic lupus erythematosus	05322	0.0195
	Amoebiasis	05146	0.0202
	ECM-receptor interaction	04512	0.0294
	Steroid hormone biosynthesis	00140	0.0406
M v B	RNA transport	03013	0.0387

A q-value < 0.05 was identified as significant.

### Validation of RNA-seq results by qRT-PCR

**Figure 5:**
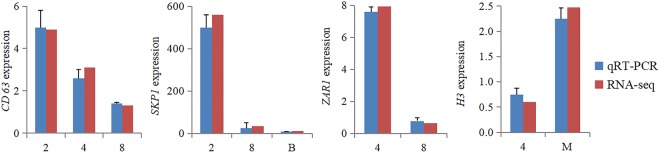
The validation of results of Smart-seq2 by qRT-PCR. The X axis represents the stages of pre-implantation development, including the 2-cell (2), 4-cell (4), 8-cell (8), morula (M), and blastocyst (B) stages.

To validate the findings of Smart-seq2 by qRT-PCR, we randomly selected four gene transcripts (*SKP1*, *CD63*, *ZAR1* and *H3*) that were expressed differentially during embryonic development as candidate genes. The qRT-PCR results revealed that the expression patterns of the studied transcripts were in agreement with those observed in the RNA-seq analysis (Fig. 5)

### Comparative analysis of transcriptome between the crossbred embryos and purebred embryos of the yak

**Figure 6:**
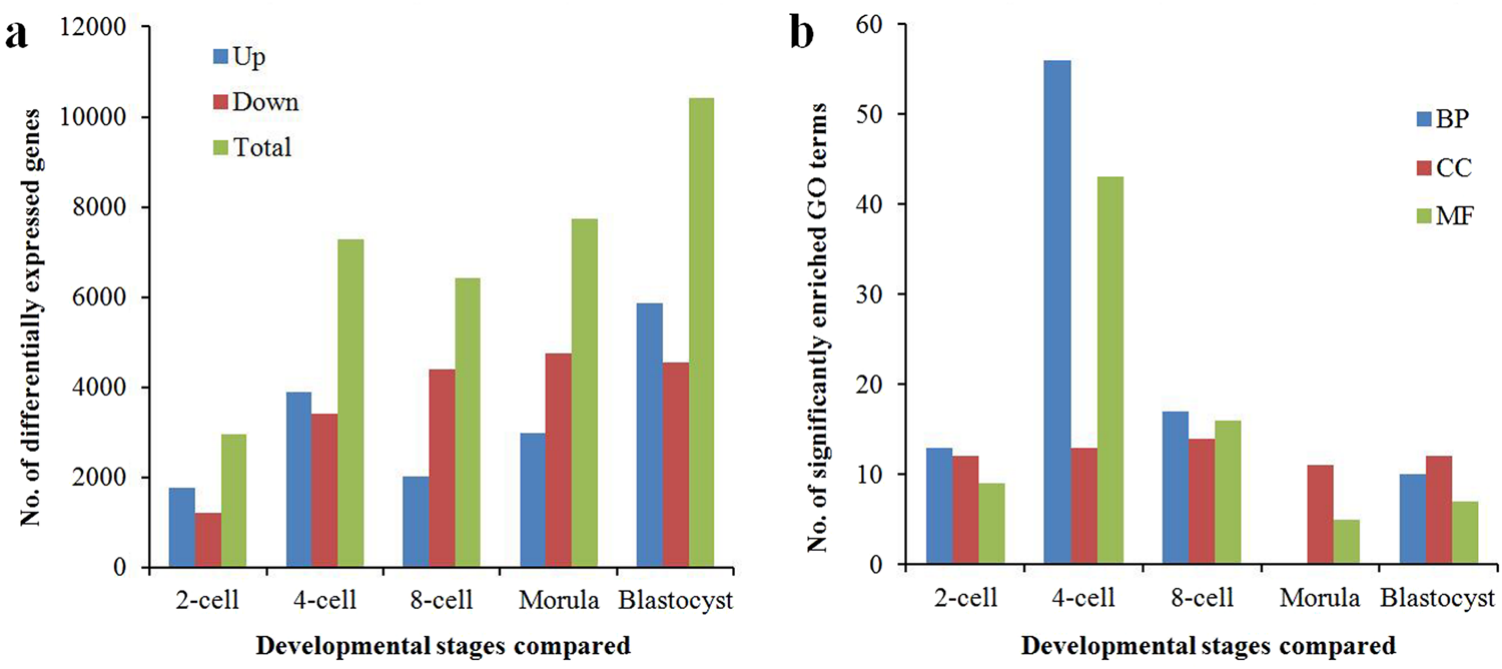
Number of DEGs (**a**) and significantly enriched GO terms (**b**) between crossbred embryos and purebred embryos at the particular stage. BP, CC and MF indicated Biological Process, Cellular Component and Molecular Function, respectively.

There were 2,960, 7,287, 6,420, 7,724 and 10,417 DEGs in 2-, 4-, 8-cell, morula and blastocyst stages between the crossbred embryos and purebred embryos of the yak, respectively (Fig. 6a). There were a number of significantly enriched GO terms (Fig. 6b) and KEGG pathways (Table 5) in each developmental stage between the two types of embryos. In BP category, the most significantly enriched GO term was cell surface receptor signalling pathway, defense response, detection of chemical stimulus, and locomotion in 2-, 4-, 8-cell and blastocyst stages, respectively, but there was no significantly enriched GO term in morula stage. In CC category, the most significantly enriched GO term was intrinsic to membrane in all stages compared. In MF category, the most significantly enriched GO term was receptor activity in 2- and 4-cell stages, but it was G-protein coupled receptor activity in 8-cell and morula stages, and transmembrane signalling receptor activity in blastocyst stage (Supplementary Table 5). The most significantly pathway was RNA transport in 2-cell and morula stages, but it was spliceosome, olfactory transduction, and ribosome biogenesis in eukaryote in 4-, 8-cell and blastocyst stages, respectively (Table 5).

**Table 5:** Pathways enriched for differentially expressed genes between crossbred embryos and yak embryos. A q-value < 0.05 was identified as significant.

Stage	Pathways	Map	q-value
2-cell	RNA transport	3013	2.42E-05
	Spliceosome	3040	0.0030
	Cell cycle	4110	0.0060
	Cell cycle - yeast	4111	0.0072
	Systemic lupus erythematosus	5322	0.0082
	Ubiquitin mediated proteolysis	4120	0.0109
4-cell	Spliceosome	3040	4.34E-07
	Neuroactive ligand-receptor interaction	4080	1.01E-05
	Protein processing in endoplasmic reticulum	4141	0.0010
	Cytokine-cytokine receptor interaction	4060	0.0015
	RNA transport	3013	0.0070
	Ribosome biogenesis in eukaryotes	3008	0.0166
	RNA degradation	3018	0.0224
	Olfactory transduction	4740	0.0394
8-cell	Olfactory transduction	4740	8.80E-15
	Neuroactive ligand-receptor interaction	4080	1.35E-08
	Ubiquitin mediated proteolysis	4120	0.0000
	Cell cycle - yeast	4111	0.0028
	Fatty acid elongation	62	0.0131
	Oocyte meiosis	4114	0.0131
	Cytokine-cytokine receptor interaction	4060	0.0131
	Complement and coagulation cascades	4610	0.0183
	Systemic lupus erythematosus	5322	0.0183
	Cell cycle	4110	0.0242
	Nucleotide excision repair	3420	0.0279
	RNA transport	3013	0.0497
Morula	RNA transport	3013	6.89E-06
	Ribosome biogenesis in eukaryotes	3008	0.0114
	Ubiquitin mediated proteolysis	4120	0.0385
Blastocyst	Ribosome biogenesis in eukaryotes	3008	0.0000
	Spliceosome	3040	0.0010
	RNA polymerase	3020	0.0326
	RNA transport	3013	0.0326

The number in the bracket is the count of significant pathways.

## Discussion

We used high-throughput sequencing to generate comprehensive transcriptome profiles of crossbred embryos of yak and cattle at the 2-, 4-, and 8-cell embryo, morula and blastocyst stages for the first time. This offers valuable information for investigating the causes of low pregnancy rates when yak females are mated with *Bos taurus* and the mechanism that regulates embryonic development of these crossbred embryos. However, *in vitro*-produced embryos are known to be developmentally less competent than *in vivo*-derived embryos, and the expression of genes involved in transcription and translation in *in vitro* cases is different from in *in vivo*-cultured bovine embryos^14–17^ and mouse embryos^18,19^. Therefore, future studies comparing the transcriptomes of *in vitro* vs. *in vivo* crossbred embryos of yak and cattle will provide essential information for improving assisted reproductive technology.

In the various developmental stages of the crossbred embryos of yak and cattle analysed in the present study, transcripts from 9,600 to 15,400 different genes per developmental stage were identified (Fig. 1). This was similar to the number of expressed genes detected in bovine embryos and human embryos^7,8,16,20^, except for the lower number of expressed genes in the morula stage detected in the present study. The lower number of transcripts detected at the morula stage may indicate that the current *in vitro* culture system is inferior. To provide information about the expression changes of individual genes over time, we further performed hierarchical clustering of all DEGs using the Euclidean distance method. Although the dynamics of actual gene expression changes in individual genes was very complex, the k-means clustering provided a good overview of the expression trends and formed a wave-like expression pattern. As a result, six clusters were identified during pre-implantation development (Fig. 4). Several regulated genes specific to each cluster were different from those of other mammals^9–13,16,20^, although early mammalian development encompasses dynamic cellular, molecular and epigenetic events that are largely conserved from mouse to man^21^.

Early embryonic development is controlled by maternal RNA and proteins accumulated during oogenesis and oocyte maturation^22,23^. As development proceeds, these maternally derived substances are degraded, while embryonic genome activation (EGA) gradually occurs^7,24,25^. The EGA is initiated at a species-specific time point. It occurs at the 2-cell stage in mice^26^, between the 4- and 8-cell stages in humans^27^ and pigs^6,26^, and between the 8- and 16-cell stages in bovines^23,26^. Previous reports have alluded to minor bovine EGA between the zygote and 4-cell stage^10,16,28–31^. However, our previous report in the yak^10^ and this data show that a high number of genes were significantly up-regulated between 2-cell and 4-cell stage, indicating that EGA occurs before the 8-cell stage in the yak and its crossbred embryos. *BMP15*, *ZP3*, *4*, *PPARG*, *SLBP*, *FRZB* and *KIT* are oocyte markers^27,32–37^, and they were down-regulated with the development of the embryos (Fig. 2A). The number of down-regulated DEGs increased from the 2-cell stage to the morula stage of these crossbred embryos (Fig. 3), also indicating that maternally derived transcripts and proteins are gradually degraded during this period. *ATP5B*, *UBE3A*, *SNURF*, *ZO3*, *CLDN4*, *MAPK13*, *JUP*, *PCGF4*, *RRAD* and *NANOG* are previously known to be embryonically expressed^7,23,36,38,39^. They were up-regulated with the development of the embryos or expressed at specific stages (Fig. 2B). They are important in regulating embryonic development. For example, *NANOG* transcripts were first observed at the eight-cell stage in the bovine embryo^7^, and its expression is required for the bovine embryonic development^40^. Mouse embryos cultured in the presence of *Clostridium perfringens* enterotoxin inhibitory to CLDN4 failed to form a mature blastocele cavity, demonstrating the importance of CLDN4 in the normal formation of blastocysts^41^. In addition, CLDN4 could be potentially involved in uterine implantation^42^.

Our previous study^10^ and qRT-PCR analyses of the amplified RNA in the present study (Fig. 5) verified that the Smart-seq2 accurately reflect the relative abundance of selected amplified transcripts (*SKP1*, *CD63*, *ZAR1* and *H3*) in the samples. *SKP1* mRNA synthesis was activated at early bovine embryonic stages, which suggests that these transcripts are necessary to prepare the embryo for EGA. The level of SKP1 protein significantly increased from MII oocytes to 4-cell embryos but then significantly decreased again^43^. Increasing importance for all aspects of inter-cell communications is attributed to extracellular vesicles (EVs) released by eukaryotic and prokaryotic cells^44^. Giacomini *et al*.^45^ showed EVs to be CD63, CD9 and ALIX, suggesting their predominant exosomal nature. The proteins mediate signal transduction events that play a role in the regulation of cell development, activation, growth and motility. *Zar1* was the first oocyte-specific maternal-effect gene identified to play an essential role during the oocyte-to-embryo transition in humans and mice, as elucidated by knockout experiments in mice^46^. H3.3-mediated paternal chromatin remodelling is essential for the development of pre-implantation embryos and the activation of the paternal genome during embryogenesis^47^. The results of both RNA-seq and qRT-PCR showed that *SKP1*, *CD63*, *ZAR1* and *H3* were expressed in the pre-implantation development of crossbred embryos of yak and cattle, and their expression patterns were similar to previous observations in other mammals^43–47^.

In the present study, we found that there were a large number of DEGs (Fig. 6a), different enriched GO terms (Fig. 6b) and pathways (Table 5) between each stage of crossbred embryos and purebred embryos of the yak. The most significantly enriched pathways for differentially expressed genes were olfactory transduction, ubiquitin-mediated proteolysis, and protein processing in the endoplasmic reticulum before the 4-cell stage, while ribosome, Parkinson’s disease, and oxidative phosphorylation pathways were highly represented after the 8-cell stage in crossbred embryos of yak and cattle (Table 5). This is different from the findings in bovine embryos reported by Jiang *et al*.^8^, except those for the ribosome pathway. The oxidative phosphorylation pathway is one of the obligatory energy metabolism pathways in most species throughout pre-implantation development^48^, and we also identified this pathway as enriched from the 8-cell to the morula stage of crossbred embryo of the yak. We identified ubiquitin-mediated proteolysis as enriched from the 2- to 4-cell and from the 8-cell to morula stages, and the most common pathway was RNA transport throughout the pre-implantation development of crossbred embryos of yak and cattle. In general, the pathways enriched by DEGs were not completely the same as those found in the bovine^8,16,49^, yak^10^, pig^6^, mouse and human^11,37,42^. Embryonic expression profiles across these mammalian species are different. Therefore, the regulatory pathways involved in pre-implantation development appear to be species-specific.

Our preliminary study indicated that the cleavage rates and blastocyst rates were lower when yak oocytes were *in vitro* fertilized with cattle sperm^4^. However, the report by Sun *et al*.^50^ and the present study showed that the developmental competence of the crossbred embryos is comparable to that of bovine embryos in the optimal *in vitro* culture conditions. Fertility defect is multifactorial origin. Among the possible origins of recurrent pregnancy loss are uterine structural defaults, defective immunological dialog between the embryo (or the fetus) and the uterus sometimes in relation with immunological disorders (such as autoimmune diseases), thrombophilia, and free radical metabolism imbalance. Numerous variants of genes are supposed to be intervening in the different facets of the early maternal-fetal or maternal-embryonic dialog, and eventually modify the outcome of fertilization, leading to success or failure of post-implantation development^51–53^. From the foregoing, there were a number of DEGs, and different GO terms and pathways between the crossbred embryos and the purebred embryos of the yak. It is worthy to further study on whether these differences effect on the early maternal (yak) - fetal (crossbred) or maternal (yak) - embryonic (crossbred) dialog, and eventually on pregnancy rate of female yaks borne crossbred embryos or fetal.

## Conclusions

We sequenced the transcriptomes of *in vitro*-produced crossbred embryos of yak and cattle during pre-implantation with the Illumina 2500 sequencing platform and provided a comprehensive examination of gene activities. This is the first report to investigate the mechanism that regulates embryonic development in crossbred embryos of yak and cattle using high-throughput sequencing, which will be helpful for development of assisted reproductive technology in yak crossbreeding. There were a number of DEGs, different GO terms and pathways between the crossbred embryos and the purebred embryos of the yak, however, there is a need to study whether these differences effect on the early maternal-fetal or maternal-embryonic dialog, and eventually on pregnancy rate of female yaks borne crossbred embryos or fetal.

## Methods

### IVM, IVF, IVC and embryo sample collection

All animal procedures were approved by the Institutional Animal Care and Use Committee of the Southwest Minzu University and all methods were performed in accordance with the relevant guidelines and regulations. Yak ovaries were collected at local slaughterhouses in October. IVM was performed as previously described by Xiao *et al*.^54^, and IVF and IVC were performed as previously described by Yao *et al*.^55^ with some modifications. Briefly, cumulus-oocyte complexes (COCs) were collected in Dulbecco’s phosphate buffered saline (DPBS) supplemented with 6 mg/ml BSA using a low-power (20×) stereomicroscope (Leica MZ75, Germany). The COCs were rinsed three times in DPBS containing 5% (v/v) foetal calf serum (FCS) and twice in TCM 199 (Gibco, Grand Island, NY, USA) supplemented with 20% (v/v) FCS, 5 μg/ml FSH, 5 μg/ml LH (Bioniche Inc, Belleville, Canada), 1 μg/ml oestradiol-17β, and 100 U/ml penicillin and 100 g/ml streptomycin (Sigma Chemical Co, St. Louis, MO, USA) (maturation medium). Oocytes with homogenous cytoplasm and several layers of cumulus cells were selected for IVM. Up to 30 COCs were placed in each culture well (Nunc Inc, Naperville IL, USA) containing 600 μl of maturation medium covered with 300 μl mineral oil (Sigma Chemical Co, St. Louis, MO, USA) and were matured at 38.5 °C for 24 h in a humidified incubator with 5% CO_2_. Motile spermatozoa were selected using a swim-up technique. Briefly, 200 μl of Jersey frozen/thawed semen was overlaid with 1.0 ml of SpermRinse™ (Vitrolife, Sweden) in a 1.5-ml EP tube and incubated at 38.5 °C. After 50 min, 600 μl of the supernatant was collected and centrifuged at 500 × g for 5 min, and then 50 μl of the concentrated sperm fraction was removed for IVF.

After IVM, the COCs were further washed twice in IVF^TM^ (Vitrolife, Sweden) before being transferred into 4-well plates (up to 30 per well) containing 500 μl IVF^TM^ covered with mineral oil per well. Motile spermatozoa were added to produce a final concentration of 2 × 10^6^ sperms/ml, and after a period of 22 h post-insemination (hpi), cumulus cells were removed by 0.2% hyaluronidase. Presumptive zygotes were cultured in 50-μl drops of G1^TM^ for 72 h and then cultured in G2^TM^ (Vitrolife, Sweden) covered with mineral oil in a humidified incubator with 90% N_2_ and 5% CO_2_ in air. Each culture droplet contained 10‒15 embryos. All embryos were carefully evaluated under a stereomicroscope, and only morphologically intact embryos scored as grade 1 according to the Manual of the International Embryo Transfer Society (IETS)^56^ were used. Embryos were pooled in groups of 10 in the case of 2-cell (42‒46 hpi), 4-cell (68‒72 hpi), and 8-cell (88‒96 hpi) stages or in singles for morula (120 hpi) and blastocyst (168 hpi) stages and were washed three times in saline, then immediately frozen and stored at −80 °C until use.

### Library preparation and sequencing

The collected embryos were lysed to release all RNA using cell lysis buffer (Sigma-Aldrich), and RNA was then reverse-transcribed by Smart-Seq2 into first cDNA. Smart-seq2 was carried out by Annoroad Gene Technology (Beijing, China) according to the method previously described^57–59^. Briefly, first-strand cDNA was synthesized from the RNA using oligo-dT, superscript II reverse transcriptase (Invitrogen), first-strand buffer (Invitrogen), RNase inhibitor (Clontech), and template-switching oligonucleotides (TSO) primers. Second-strand cDNAs were synthesized using IS PCR primers and KAPA HiFi HotStart ReadyMix (KAPA Biosystems) on a thermal cycler (S1000, Bio-Rad). cDNAs were consequently pre-amplified, purified and recovered. After purification, the distribution of fragments and the quality of amplified products were examined with the Agilent high-sensitivity DNA chip (Agilent Technologies, Palo Alto, CA, USA) using a Bioanalyzer 2100 (Agilent). The qualified library contained no fragment <500 bp, with peak value at 1.5–2 kb.

The cDNA samples were fragmented by Tagmentation (Tn5) to obtain cDNA fragments, ligated with a sequencing adapter, and then subjected to PCR amplification with KAPA HiFi DNA polymerase on a thermal cycler (S1000, Bio-Rad). The amplified cDNA was purified with AMPure XP beads (Beckman-Coulter). The cDNA concentration was determined on a Qubit 2.0 Fluorometer (Life Technologies, Carlsbad, CA, USA), and the distributions of the fragments of amplified products were examined with the Agilent high-sensitivity DNA chip (Agilent Technologies, Palo Alto, CA, USA) using a Bioanalyzer 2100 (Agilent). The peak value of DNA fragments was within the range of 300–800 bp. The qualified cDNA libraries were sequenced on the Illumina X-Ten platform (Illumina, San Diego, CA, USA) with read lengths of paired-ends at 125 bp.

### Analysis of sequencing results: mapping and differential expression

Raw reads obtained from the RNA-seq were cleaned by removing adapter sequences, reads containing ploy-N, and low-quality sequences (Q < 20) as previously described^60^. Clean reads were then aligned to the yak reference genome (BosGru_v2.0)^61^ using Tophat2 (v2.0.12). Novel transcripts were identified from TopHat alignment results using the Cufflinks (v2.2.1) (http://cufflinks.cbcb.umd.edu/howitworks.html) reference annotation-based transcript (RABT) assembly method, with lengths ≥180 bp, sequence depths ≥2, and distances within 200 bp of the annotated gene^62^.

Gene expression levels were normalized by considering the RPKM value (reads per kilobase of the exon model per million mapped reads)^63^. Differentially expressed gene (DEG) analysis was conducted using the R package DEGseq (v1.18.0). The P values were adjusted using the Benjamini & Hochberg method^64^. The significant DEGs between subsequent stages were identified with corrected ‘p < 0.05 and |log_2_ fold change| >1’ in this study. The heat maps were drawn using the R packages (v3.1.1) as follows: function ‘heatmap.2’ of the ‘gplots’ package and the ‘sota’ function in the ‘clValid’ package, with default Euclidean distance and the hierarchical clustering method.

### Gene ontology and pathway enrichment analysis of DEGs

The main functions of the differentially expressed genes were determined using GO analysis (http://www.geneontology.org/) provided by the NCBI, which can discover gene regulatory networks on the basis of biological processes, molecular functions and cellular components. A hypergeometric test was used to find significantly enriched GO terms or pathways in DEGs by comparison with the genome background. Multiple tests were adjusted by the FDR method^64^. The adjusted p-value ≤0.05 was set as the significant threshold. Pathway annotations of DEGs were performed using the Kyoto Encyclopedia of Genes and Genomes (KEGG) database (http://www.enome.jp/kegg/).

### Validation of the Smart-seq2 results by qRT-PCR

The total RNA from each pool of crossbred yak embryos (*n* = 3) was isolated using TRIzol reagent (Invitrogen) according to the manufacturer’s instructions. Then, RNA was dissolved in sterile water and stored at −80 °C until use. Quantitative real-time PCR (qRT-PCR) was performed to validate the expression of four selected genes (*SKP1*, *CD63*, *ZAR1* and *H3*). They were amplified with specific primers (Supplementary Table 6) on an ABI 7500 Fast instrument (Applied Biosystems). All values were normalized to the internal control, *H2A*. The efficiency of each primer pair was calculated over a 3.5 log dilution range, and the relative gene expression values were calculated using the 2^−△△Ct^ method^65^.

### Comparative analysis of transcriptome between the crossbred embryos and purebred embryos of the yak

*In vitro* production and mRNA sequencing of yak embryos were exactly as same as the above described. Raw sequencing data of yak embryos were stored in the NCBI Sequence Read Archive (Accession number SRP127024)^10^. The DEGs in each stage between these two types of embryos, and GO and KEGG pathway analysis were the same with the above described.

## Electronic supplementary material

Supplementary Dataset 1
