# Supplementary material for: Transcriptional profiles of crossbred embryos derived from yak oocytes in vitro fertilized with cattle sperm
Source: Sci Rep. 2018 Aug 1;8:11571. doi: 10.1038/s41598-018-29912-7 (PMC6070518; doi:10.1038/s41598-018-29912-7)
Supplement: Supplementary file 1 — Supplementary Dataset 1 [file 41598_2018_29912_MOESM1_ESM.pdf]

Supplementary Information

Transcriptional profiles of crossbred embryos derived from yak oocytes *in vitro* fertilized with cattle sperm

Xiang-dong Zi[1], Shuang Liu[2], Wei Xia[1], Xian-rong Xiong[2] & Bin Luo[2]

[1]Key Laboratory of Animal Science of State Ethnic Affairs Commission, Southwest Minzu University, Chengdu 610041, China. [2]Ministry of Education Key Laboratory of Conservation & Utilization of Qinghai-Tibetan Plateau Animal Genetic Resources, Southwest Minzu University, Chengdu 610041, China

Correspondence and requests for materials should be addressed to X.-d.Z. (zixd2000@yahoo.com)

Supplementary Table 1

Differentially expressed genes during pre-implantation development

Gene	log_2FC	adj-p	up/dn	Description
2-cell vs 4-cell				
ENSP00000351254-D1	-9.88	8.51E-77	↑	B. mutus methyl-CpG binding domain protein 3-like 3 (MBD3L3), mRNA
ENSBTAP00000023885-D5	-8.88	1.40E-44	↑	B. mutus tripartite motif-containing protein 64-like (LOC102282339), mRNA
ENSP00000292853-D5	-8.77	7.93E-42	↑	Bubalus bubalis uncharacterized LOC102396720 (LOC102396720), mRNA
ENSBTAP00000010968-D1	-8.72	1.12E-40	↑	B. mutus cytohesin 1 interacting protein (CYTIP), mRNA
ENSBTAP00000050537-D1	-8.61	2.39E-38	↑	B. mutus zinc finger and SCAN domain-containing protein 4-like (LOC102273489), mRNA
ENSP00000371553-D1	-8.57	1.68E-37	↑	Bubalus bubalis NANOG neighbor homeobox (NANOGNB), mRNA
ENSBTAP00000042698-D5	-8.49	8.49E-36	↑	Bubalus bubalis putative RNA polymerase II subunit A C-terminal domain phosphatase SSU72-like protein 1-like (LOC102405647), mRNA
ENSP00000365141-D9	-8.31	2.55E-32	↑	Bubalus bubalis tripartite motif-containing protein 64-like (LOC102410925), mRNA
ENSBTAP00000003296-D1	-8.14	1.81E-29	↑	Bubalus bubalis TATA-box-binding protein-like (LOC102408938), mRNA
yakA22750	-7.93	3.65E-26	↑	bovine 1.711 g/ml satellite dna, f fragment
ENSBTAP00000028766-D1	-7.93	3.65E-26	↑	B. mutus secretogranin-2-like (LOC102269011), mRNA
ENSP00000403967-D4	-7.93	3.65E-26	↑	Ovis aries putative upstream-binding factor 1-like protein 1 (LOC101111139), mRNA
ENSBTAP00000047795-D1	-7.92	5.59E-26	↑	B. mutus putative germ cell-less protein-like 1-like (LOC102270837), mRNA
ENSBTAP00000001562-D1	-7.78	4.30E-24	↑	B. mutus ubiquitin specific peptidase like 1 (USPL1), mRNA
ENSBTAP00000014774-D1	-7.54	5.37E-21	↑	Bubalus bubalis heterogeneous nuclear ribonucleoproteins A2/B1-like (LOC102406360), mRNA
ENSBTAP00000048101-D1	-7.48	4.70E-39	↑	B. mutus chemokine (C-X-C motif) receptor 2 (CXCR2), mRNA
ENSBTAP00000038062-D4	-7.36	5.29E-19	↑	B. taurus NADH dehydrogenase (ubiquinone) 1 alpha subcomplex, 4-like 2 (NDUFA4L2), mRNA
ENSP00000349931-D8	-7.27	5.47E-18	↑	Bubalus bubalis putative PRAME family member 24-like (LOC102393907), mRNA
ENSP00000359563-D1	-7.18	3.62E-17	↑	B. mutus protein ycf2-like (LOC102265747), mRNA
ENSBTAP00000033424-D1	-7.05	6.38E-16	↑	B. mutus epithelial chloride channel protein-like (LOC102271020), transcript variant X1, mRNA
ENSP00000403095-D3	-7.03	1.04E-15	↑	B. mutus putative upstream-binding factor 1-like protein 1-like (LOC102269715), mRNA
ENSBTAP00000050537-D2	-6.93	7.37E-15	↑	B. mutus zinc finger and SCAN domain-containing protein 4-like (LOC102280465), mRNA
ENSBTAP00000024724-D1	-6.90	1.20E-14	↑	B. mutus lysyl oxidase-like 3 (LOXL3), mRNA
ENSBTAP00000022815-D1	-6.80	8.74E-14	↑	B. mutus matrilin 2 (MATN2), mRNA
ENSP00000398079-D3	-6.77	1.45E-13	↑	B. mutus putative RNA polymerase II subunit A C-terminal domain phosphatase SSU72-like protein 1-like, mRNA
ENSBTAP00000048510-D1	-6.74	2.40E-13	↑	B. mutus negative elongation factor C/D-like (LOC102286519), mRNA
ENSBTAP00000050906-D7	-6.74	2.40E-13	↑	B. mutus uncharacterized LOC102265782 (LOC102265782), mRNA
ENSBTAP00000023885-D2	-6.71	3.95E-13	↑	B. mutus tripartite motif containing 64 (TRIM64), mRNA
ENSP00000398079-D1	-6.68	6.54E-13	↑	B. mutus putative RNA polymerase II subunit A C-terminal domain phosphatase SSU72-like protein 1-like, mRNA
ENSBTAP00000009523-D1	-6.68	1.73E-160	↑	B. mutus tumor necrosis factor, alpha-induced protein 6 (TNFAIP6), mRNA
yakG013325	-6.66	9.35E-69	↑	B. mutus bisphosphoglycerate mutase-like (LOC102275256), mRNA
ENSBTAP00000020078-D1	-6.65	1.09E-12	↑	B. mutus hydroxysteroid (11-beta) dehydrogenase 1 (HSD11B1), mRNA
ENSBTAP00000021833-D1	-6.59	3.02E-12	↑	B. mutus leukotriene A4 hydrolase (LTA4H), transcript variant X1, mRNA
ENSBTAP00000005307-D1	-6.49	1.42E-11	↑	B. mutus serpin peptidase inhibitor, clade A (alpha-1 antiproteinase, antitrypsin), member 5 (SERPINA5), mRNA
ENSP00000333813-D1	-6.42	4.01E-11	↑	B. mutus ALG12, alpha-1,6-mannosyltransferase (ALG12), mRNA
ENSBTAP00000049936-D1	-6.38	6.76E-11	↑	B. mutus heterogeneous nuclear ribonucleoproteins A2/B1-like (LOC102276625), mRNA
yakA18166	-6.38	6.76E-11	↑	Sus scrofa uncharacterized LOC102165801 (LOC102165801), mRNA
ENSBTAP00000023277-D2	-6.34	1.14E-10	↑	Bubalus bubalis cyclin D2 (CCND2), mRNA
ENSP00000350848-D1	-6.31	1.93E-10	↑	Bubalus bubalis high mobility group nucleosome binding domain 5 (HMGN5), mRNA
ENSBTAP00000023645-D1	-6.31	1.93E-10	↑	B. mutus BCDIN3 domain containing (BCDIN3D), mRNA
ENSBTAP00000012447-D1	-6.27	3.28E-10	↑	B. mutus serine/threonine/tyrosine interacting-like 1 (STYXL1), mRNA
yakA01168	-6.23	0.00E+00	↑	bovine 1.711 g/ml satellite dna, f fragment
ENSP00000383643-D14	-6.18	9.46E-10	↑	B. mutus zinc finger protein 41 (ZNF41), mRNA
ENSBTAP00000050581-D1	-6.18	9.46E-10	↑	B. mutus ribose-phosphate pyrophosphokinase 1-like (LOC102279419), mRNA
ENSBTAP00000044500-D1	-6.18	9.46E-10	↑	Bubalus bubalis SEBOX homeobox (SEBOX), mRNA
ENSP00000396648-D3	-6.18	9.46E-10	↑	B. mutus proline-rich protein 23C-like (LOC102280548), mRNA
ENSBTAP00000016441-D1	-6.12	1.60E-48	↑	B. mutus putative PRAME family member 24-like (LOC102265211), mRNA
ENSBTAP00000009568-D1	-6.10	2.78E-09	↑	B. mutus triggering receptor expressed on myeloid cells 2 (TREM2), mRNA
ENSBTAP00000017943-D1	-6.05	4.76E-09	↑	B. mutus 5-hydroxytryptamine (serotonin) receptor 2A, G protein-coupled (HTR2A), mRNA

ENSBTAP00000028431-D1	-6.00	8.17E-09	↑	B. mutus dynein assembly factor with WDR repeat domains 1 (DAW1), mRNA
ENSBTAP00000020343-D1	-6.00	8.17E-09	↑	B. mutus testis-expressed protein 19.2-like (LOC102276876), mRNA
ENSBTAP00000012865-D10	-5.95	1.40E-08	↑	B. mutus melanoma antigen preferentially expressed in tumors-like (LOC102267181), mRNA
ENSP00000366416-D1	-5.95	1.40E-08	↑	Bubalus bubalis endothelin receptor type B (EDNRB), mRNA
ENSBTAP00000047565-D1	-5.95	1.40E-08	↑	B. mutus uncharacterized LOC102270435 (LOC102270435), mRNA
ENSP00000393444-D1	-5.95	1.40E-08	↑	Bubalus bubalis pregnancy-associated plasma protein A, pappalysin 1 (PAPPA), partial mRNA
ENSP00000363545-D1	-5.90	2.42E-08	↑	B. mutus zinc finger, DBF-type containing 2 (ZDBF2), mRNA
ENSBTAP00000050211-D1	-5.85	4.21E-08	↑	.
ENSP00000408828-D1	-5.80	7.33E-08	↑	B. mutus leucine rich repeat containing 63 (LRRC63), mRNA
ENSP00000216862-D1	-5.74	1.28E-07	↑	B. mutus 1,25-dihydroxyvitamin D(3) 24-hydroxylase, mitochondrial-like, transcript variant X1, mRNA
ENSP00000389928-D6	-5.74	1.28E-07	↑	B. mutus tripartite motif-containing protein 43-like (LOC102274187), mRNA
ENSBTAP00000051958-D1	-5.74	1.28E-07	↑	B. taurus T cell receptor alpha (TCRA) gene, J segments and C region
ENSP00000384262-D1	-5.74	1.28E-07	↑	B. mutus heparanase (HPSE), mRNA
ENSBTAP00000029241-D1	-5.74	1.28E-07	↑	B. mutus retinol saturase (all-trans-retinol 13,14-reductase) (RETSAT), mRNA
ENSBTAP00000051195-D1	-5.74	1.28E-07	↑	B. mutus progesterone receptor-like (LOC102285168), partial mRNA
ENSBTAP00000018876-D1	-5.68	2.22E-07	↑	B. mutus solute carrier family 38, member 4 (SLC38A4), transcript variant X2, mRNA
ENSP00000332164-D2	-5.68	2.22E-07	↑	Bubalus bubalis slit homolog 2 (Drosophila) (SLIT2), transcript variant X3, mRNA
ENSP00000378431-D1	-5.68	2.22E-07	↑	B. mutus multimerin 1 (MMRN1), transcript variant X1, mRNA
ENSBTAP00000038873-D2	-5.62	3.88E-07	↑	B. mutus putative protein FAM90A12P-like (LOC102273546), mRNA
ENSP00000306776-D1	-5.62	3.88E-07	↑	B. mutus coiled-coil domain containing 110 (CCDC110), mRNA
ENSBTAP00000002469-D1	-5.62	3.88E-07	↑	B. mutus potassium voltage-gated channel, subfamily H (eag-related), member 7 (KCNH7), mRNA
ENSP00000384038-D1	-5.56	6.83E-07	↑	B. mutus phosphofructokinase, liver (PFKL), mRNA
ENSP00000382767-D1	-5.49	1.20E-06	↑	B. mutus sel-1 suppressor of lin-12-like 3 (C. elegans) (SEL1L3), mRNA
ENSP00000303802-D1	-5.49	1.20E-06	↑	B. mutus GTP-binding protein 8 (putative) (GTPBP8), mRNA
ENSP00000365363-D15	-5.49	1.20E-06	↑	B. mutus putative PRAME family member 24-like (LOC102270444), mRNA
ENSBTAP00000002745-D1	-5.49	1.20E-06	↑	B. mutus myosin IIIA (MYO3A), mRNA
ENSBTAP00000003384-D1	-5.49	1.20E-06	↑	B. taurus LON peptidase N-terminal domain and ring finger 3 (LONRF3), mRNA
ENSP00000257570-D1	-5.49	1.20E-06	↑	B. taurus 2'-5'-oligoadenylate synthetase-like (OASL), mRNA
ENSBTAP00000027514-D1	-5.49	1.20E-06	↑	B. mutus zinc finger CCCH-type containing 6 (ZC3H6), mRNA
ENSBTAP00000006089-D1	-5.49	1.20E-06	↑	B. mutus sperm adhesion molecule 1 (SPAM1), transcript variant X2, mRNA
ENSP00000410221-D1	-5.42	3.37E-11	↑	B. mutus stimulated by retinoic acid 6 (STRA6), transcript variant X1, mRNA
ENSBTAP00000036892-D1	-5.42	2.13E-06	↑	B. mutus mucin-19-like (LOC102286973), mRNA
ENSP00000410940-D5	-5.42	2.13E-06	↑	Pantholops hodgsonii putative tripartite motif-containing protein 64C-like (LOC102327758), mRNA
ENSBTAP00000036397-D1	-5.42	2.13E-06	↑	B. mutus R3H domain and coiled-coil containing 1 (R3HCC1), mRNA
ENSBTAP00000021898-D1	-5.42	2.13E-06	↑	B. mutus N-acetyltransferase 1 (arylamine N-acetyltransferase) (NAT1), transcript variant X2, mRNA
ENSP00000357130-D1	-5.42	2.13E-06	↑	B. mutus spectrin, alpha, erythrocytic 1 (elliptocytosis 2) (SPTA1), mRNA
ENSBTAP00000021570-D1	-5.42	2.13E-06	↑	B. mutus monoamine oxidase A (MAOA), mRNA
ENSP00000401313-D1	-5.42	2.13E-06	↑	B. mutus cysteine and tyrosine-rich protein 1-like (LOC102272880), mRNA
ENSBTAP00000043456-D1	-5.42	2.13E-06	↑	B. mutus hydroxysteroid (17-beta) dehydrogenase 1 (HSD17B1), mRNA
ENSBTAP00000028074-D1	-5.42	2.13E-06	↑	B. mutus chromosome unknown open reading frame, human C1orf210 (LOC102287438), mRNA
ENSP00000401856-D1	-5.39	1.26E-133	↑	B. mutus coiled-coil domain containing 80 (CCDC80), mRNA
ENSP00000297203-D1	-5.34	3.78E-06	↑	B. mutus uncharacterized LOC102273755 (LOC102273755), mRNA
ENSP00000358551-D1	-5.34	3.78E-06	↑	B. mutus adenosine monophosphate deaminase 1 (AMPD1), transcript variant X2, mRNA
ENSBTAP00000012262-D1	-5.34	3.78E-06	↑	B. taurus peroxisomal biogenesis factor 10 (PEX10), mRNA
ENSP00000340578-D1	-5.34	3.78E-06	↑	B. mutus Ras association (RalGDS/AF-6) domain family member 6 (RASSF6), mRNA
ENSBTAP00000016164-D1	-5.34	3.78E-06	↑	B. mutus dickkopf-like 1 (DKKL1), mRNA
ENSP00000358786-D1	-5.27	6.73E-06	↑	B. mutus potassium voltage-gated channel, shaker-related subfamily, member 10 (KCNA10), mRNA
ENSBTAP00000004136-D1	-5.27	6.73E-06	↑	B. mutus netrin 4 (NTN4), transcript variant X1, mRNA
ENSP00000264036-D1	-5.27	6.73E-06	↑	B. mutus melanoma cell adhesion molecule (MCAM), mRNA
ENSBTAP00000034409-D1	-5.27	6.73E-06	↑	B. mutus melanoma antigen preferentially expressed in tumors-like (LOC102269877), mRNA
ENSBTAP00000049101-D1	-5.27	6.73E-06	↑	B. mutus seizure threshold 2 homolog (mouse) (SZT2), mRNA
ENSP00000284425-D2	-5.27	6.73E-06	↑	Bubalus bubalis ATP-binding cassette, sub-family A (ABC1), member 10, transcript variant X4, misc_RNA
ENSP00000396648-D2	-5.27	6.73E-06	↑	B. mutus proline-rich protein 23B-like (LOC102280271), mRNA
ENSBTAP00000025591-D13	-5.18	1.06E-09	↑	B. mutus putative RNA polymerase II subunit A C-terminal domain phosphatase SSU72-like protein 1-like, mRNA
ENSBTAP00000048850-D42	-5.18	1.20E-05	↑	B.taurus DNA sequence from clone CH240-414K18, complete sequence

ENSBTAP00000022540-D1	-5.18	1.20E-05	↑	B. mutus angiotensin II receptor, type 1 (AGTR1), mRNA
ENSBTAP00000038660-D1	-5.18	1.20E-05	↑	B. mutus protein FAM32A-like (LOC102287030), mRNA
ENSP00000373194-D68	-5.18	1.20E-05	↑	B. mutus olfactory receptor 8H1-like (LOC102280886), mRNA
yakG026412	-5.18	1.20E-05	↑	Pantholops hodgsonii ribosomal protein L9 (RPL9), transcript variant X1, mRNA
ENSBTAP00000002390-D1	-5.18	1.20E-05	↑	B. mutus secernin 2 (SCRN2), mRNA
ENSBTAP00000049930-D1	-5.18	1.20E-05	↑	B. mutus transmembrane 4 L six family member 19 (TM4SF19), mRNA
ENSBTAP00000019765-D1	-5.18	1.20E-05	↑	B. mutus glucosidase, beta, acid (GBA), mRNA
ENSP00000329748-D1	-5.18	1.20E-05	↑	B. mutus copine VIII (CPNE8), mRNA
ENSBTAP00000004288-D1	-5.18	1.20E-05	↑	B. mutus tumor necrosis factor receptor superfamily, member 9 (TNFRSF9), mRNA
ENSBTAP00000001642-D1	-5.14	2.62E-17	↑	B. mutus plasminogen activator, tissue (PLAT), transcript variant X1, mRNA
ENSP00000328030-D3	-5.10	3.42E-09	↑	Pantholops hodgsonii protein BEX5-like (LOC102341393), misc_RNA
ENSBTAP00000002330-D1	-5.10	3.42E-09	↑	B. mutus nuclear factor of kappa light polypeptide gene enhancer in B-cells inhibitor, beta (NFKBIB), mRNA
ENSBTAP00000017140-D1	-5.10	2.15E-05	↑	B. taurus keratin 10, mRNA (cDNA clone MGC:151759 IMAGE:8284815), complete cds
ENSP00000310788-D53	-5.10	2.15E-05	↑	B. mutus olfactory receptor 5P3-like (LOC102269408), mRNA
ENSBTAP00000001547-D1	-5.10	2.15E-05	↑	B. mutus core histone macro-H2A.2-like (LOC102268089), mRNA
ENSBTAP00000052710-D1	-5.10	2.15E-05	↑	B. mutus peptidase D (PEPD), transcript variant X1, mRNA
ENSP00000216780-D1	-5.10	2.15E-05	↑	B. mutus phosphoenolpyruvate carboxykinase 2 (mitochondrial) (PCK2), mRNA
ENSBTAP00000001635-D1	-5.10	2.15E-05	↑	B. mutus cell surface glycoprotein CD200 receptor 1-like (LOC102279587), mRNA
ENSBTAP00000043215-D1	-5.10	2.15E-05	↑	B. mutus cadherin 17, LI cadherin (liver-intestine) (CDH17), mRNA
ENSP00000371497-D1	-5.10	2.15E-05	↑	B. mutus C1q and tumor necrosis factor related protein 3 (C1QTNF3), transcript variant X1, mRNA
ENSBTAP00000003472-D1	-5.05	6.10E-09	↑	B. mutus phosphofructokinase, platelet (PFKP), mRNA
ENSBTAP00000042366-D7	-5.00	8.68E-16	↑	B. mutus zinc finger protein 146 (ZNF146), mRNA
ENSBTAP00000040663-D1	-5.00	1.09E-08	↑	B. mutus ATP-binding cassette, sub-family C (CFTR/MRP), member 9 (ABCC9), mRNA
ENSBTAP00000020971-D1	-5.00	3.85E-05	↑	B. mutus ras-related protein Rab-38-like (LOC102267377), mRNA
ENSBTAP00000049139-D17	-5.00	3.85E-05	↑	Tursiops truncatus ribosomal protein S6 (RPS6), mRNA
ENSBTAP00000003598-D1	-5.00	3.85E-05	↑	B. taurus hypothetical protein LOC767878, mRNA (cDNA clone IMAGE:8051342), partial cds
ENSP00000378508-D1	-5.00	3.85E-05	↑	B. mutus DnaJ (Hsp40) homolog, subfamily C, member 22 (DNAJC22), mRNA
ENSBTAP00000051231-D3	-5.00	3.85E-05	↑	B. mutus 40S ribosomal protein S4-like (LOC102273649), mRNA
ENSP00000354571-D2	-5.00	3.85E-05	↑	B. mutus ring finger protein 175 (RNF175), mRNA
ENSBTAP00000012987-D1	-5.00	3.85E-05	↑	B. mutus placenta-specific gene 8 protein-like (LOC102276015), mRNA
ENSBTAP00000029677-D1	-5.00	3.85E-05	↑	B. mutus transmembrane protein 132B-like (LOC102279488), mRNA
ENSBTAP00000040134-D1	-5.00	3.85E-05	↑	B. mutus melanoma antigen preferentially expressed in tumors-like (LOC102270432), mRNA
ENSP00000217909-D1	-5.00	3.85E-05	↑	B. mutus solute carrier family 25, member 43 (SLC25A43), mRNA
ENSP00000302393-D1	-5.00	3.85E-05	↑	B. mutus L-lactate dehydrogenase A-like 6B-like (LOC102282411), transcript variant X1, mRNA
yakG040722	-5.00	3.85E-05	↑	B. mutus mastermind-like 3 (Drosophila) (MAML3), mRNA
ENSBTAP00000024208-D1	-4.99	8.22E-79	↑	B. mutus stefin-C-like (LOC102287480), mRNA
ENSP00000387264-D1	-4.90	2.37E-21	↑	B. mutus LIM and senescent cell antigen-like domains 1 (LIMS1), transcript variant X2, mRNA
ENSBTAP00000012944-D2	-4.90	2.37E-21	↑	B. mutus PRAME family member 12-like (LOC102269045), mRNA
ENSBTAP00000015947-D1	-4.90	3.54E-08	↑	B. mutus LIM homeobox transcription factor 1, alpha (LMX1A), mRNA
ENSBTAP00000012968-D1	-4.90	3.54E-08	↑	B. mutus cyclic nucleotide gated channel alpha 3 (CNGA3), mRNA
yakG025165	-4.90	6.92E-05	↑	Physeter catodon general transcription factor IIF, polypeptide 1, 74kDa (GTF2F1), mRNA
ENSBTAP00000015461-D1	-4.90	6.92E-05	↑	B. taurus zinc finger protein LOC768229 (LOC768229), mRNA
ENSBTAP00000004156-D1	-4.90	6.92E-05	↑	B. mutus chondroitin sulfate proteoglycan 4-like (LOC102282122), mRNA
ENSP00000304077-D23	-4.90	6.92E-05	↑	B. mutus olfactory receptor 4F3/4F16/4F29-like (LOC102286438), mRNA
ENSP00000409717-D1	-4.90	6.92E-05	↑	B. mutus LIM domain kinase 1 (LIMK1), mRNA
ENSP00000352167-D1	-4.90	6.92E-05	↑	B. taurus solute carrier family 25 (mitochondrial carnitine/acylcarnitine carrier), member 29 (SLC25A29), mRNA
ENSBTAP00000020290-D1	-4.90	6.92E-05	↑	B. mutus ST6 (alpha-N-acetyl-neuraminyl-2,3-beta-galactosyl-1,3)-N-acetylgalactosaminide alpha-2, 6-sialyltransferase 2 (ST6GALNAC2), transcript variant X1, mRNA
ENSP00000333915-D1	-4.90	6.92E-05	↑	B. mutus coiled-coil domain containing 42B (CCDC42B), mRNA
ENSBTAP00000053795-D1	-4.90	6.92E-05	↑	B. mutus EPH receptor A3 (EPHA3), transcript variant X1, mRNA
ENSBTAP00000032404-D1	-4.90	6.92E-05	↑	B. mutus melanocortin 2 receptor accessory protein (MRAP), mRNA
ENSP00000360858-D2	-4.90	6.92E-05	↑	B. mutus olfactomedin 3 (OLFM3), transcript variant X1, mRNA
ENSBTAP00000030712-D1	-4.90	6.92E-05	↑	B. mutus synaptoporin (SYNPR), transcript variant X2, mRNA
ENSP00000342848-D1	-4.90	6.92E-05	↑	B. mutus NADPH oxidase activator 1 (NOXA1), mRNA
ENSP00000382213-D1	-4.90	6.92E-05	↑	B. mutus dentin sialophosphoprotein (DSPP), mRNA

ENSP00000300504-D1	-4.90	6.92E-05	↑	B. mutus TBC1 domain family, member 21 (TBC1D21), transcript variant X1, mRNA
ENSP00000367635-D1	-4.90	6.92E-05	↑	B. mutus cytosolic thiouridylase subunit 2 homolog (S. pombe) (CTU2), mRNA
ENSBTAP00000000693-D1	-4.90	6.92E-05	↑	B. mutus glutamate decarboxylase 1-like (LOC102287268), mRNA
ENSP00000408987-D14	-4.90	6.92E-05	↑	Homo sapiens alpha satellite DNA
ENSP00000246841-D1	-4.90	6.92E-05	↑	B. mutus fibronectin leucine rich transmembrane protein 1 (FLRT1), mRNA
ENSBTAP00000027432-D1	-4.90	6.92E-05	↑	Capra hircus coiled-coil and C2 domain-containing protein 2A-like (LOC102172322), mRNA
ENSBTAP00000049401-D1	-4.90	6.92E-05	↑	B. mutus claudin 17 (CLDN17), mRNA
ENSP00000250056-D1	-4.90	6.92E-05	↑	B. mutus family with sequence similarity 64, member A (FAM64A), mRNA
ENSP00000334665-D2	-4.85	6.39E-08	↑	B. mutus fascin homolog 1, actin-bundling protein (Strongylocentrotus purpuratus) (FSCN1), partial mRNA
ENSBTAP00000041206-D1	-4.85	6.39E-08	↑	B. mutus myosin binding protein C, slow type (MYBPC1), transcript variant X1, mRNA
ENSBTAP00000011393-D1	-4.85	6.39E-08	↑	B. mutus tripartite motif family-like 1 (TRIML1), mRNA
ENSP00000364236-D1	-4.80	1.26E-04	↑	B. mutus transmembrane protein 136 (TMEM136), mRNA
ENSP00000407396-D1	-4.80	1.26E-04	↑	B. mutus transmembrane protein 40 (TMEM40), mRNA
ENSBTAP00000013588-D81	-4.80	1.26E-04	↑	Capra hircus zinc finger protein 347-like (LOC102174966), mRNA
ENSP00000303316-D1	-4.80	1.26E-04	↑	B. mutus piezo-type mechanosensitive ion channel component 2 (PIEZO2), mRNA
ENSBTAP00000049744-D1	-4.80	1.26E-04	↑	B. mutus ADP-ribosylarginine hydrolase (ADPRH), mRNA
ENSBTAP00000011219-D1	-4.80	1.26E-04	↑	B. mutus potassium voltage-gated channel subfamily V member 1-like (LOC102275313), mRNA
ENSP00000381036-D1	-4.80	1.26E-04	↑	B. mutus androglobin (ADGB), mRNA
ENSBTAP00000036630-D1	-4.80	1.26E-04	↑	B. mutus L-threonine 3-dehydrogenase, mitochondrial-like (LOC102283236), mRNA
ENSBTAP00000052694-D43	-4.80	1.26E-04	↑	B. mutus olfactory receptor 4K2-like (LOC102279421), mRNA
ENSBTAP00000025607-D1	-4.80	1.26E-04	↑	B. mutus prostaglandin E receptor 3 (subtype EP3) (PTGER3), transcript variant X1, mRNA
ENSBTAP00000027209-D2	-4.80	1.26E-04	↑	Bubalus bubalis death-associated protein kinase 2 (DAPK2), transcript variant X1, mRNA
ENSP00000260061-D1	-4.80	1.26E-04	↑	B. mutus leucine rich repeat containing 32 (LRRC32), mRNA
ENSBTAP00000021995-D1	-4.80	1.26E-04	↑	B. mutus calcium/calmodulin-dependent protein kinase IG (CAMK1G), mRNA
ENSBTAP00000048086-D33	-4.80	1.26E-04	↑	B. mutus olfactory receptor 2B2-like (LOC102269771), mRNA
ENSBTAP00000001526-D1	-4.77	1.77E-13	↑	B. mutus human immunodeficiency virus type I enhancer binding protein 2 (HIVEP2), mRNA
ENSP00000368959-D2	-4.76	2.71E-19	↑	B. mutus receptor accessory protein 5 (REEP5), mRNA
ENSP00000249356-D1	-4.74	2.46E-42	↑	B. mutus DnaJ (Hsp40) homolog, subfamily B, member 9 (DNAJB9), mRNA
ENSBTAP00000009604-D1	-4.74	2.09E-07	↑	B. mutus four and a half LIM domains 3 (FHL3), mRNA
ENSBTAP00000024392-D1	-4.74	2.09E-07	↑	B. mutus RAD9 homolog B (S. pombe) (RAD9B), mRNA
ENSBTAP00000016757-D1	-4.71	5.80E-13	↑	Pantholops hodgsonii Norrie disease (pseudoglioma) (NDP), transcript variant X3, mRNA
ENSP00000356259-D1	-4.68	3.77E-07	↑	B. mutus coiled-coil domain containing 170 (CCDC170), mRNA
ENSP00000382483-D4	-4.68	3.77E-07	↑	B. mutus interferon induced transmembrane protein 3 (IFITM3), transcript variant X2, mRNA
ENSBTAP00000014986-D1	-4.68	2.28E-04	↑	B. mutus matrilin 4 (MATN4), transcript variant X1, mRNA
ENSBTAP00000021458-D1	-4.68	2.28E-04	↑	B. mutus potassium voltage-gated channel, shaker-related subfamily, member 7 (KCNA7), mRNA
yakG014317	-4.68	2.28E-04	↑	.
yakG026351	-4.68	2.28E-04	↑	Pantholops hodgsonii putative PRAME family member 24-like (LOC102329037), mRNA
ENSP00000369553-D42	-4.68	2.28E-04	↑	Capra hircus interferon omega-1-like (LOC102178826), mRNA
ENSP00000256857-D1	-4.68	2.28E-04	↑	B. mutus uncharacterized LOC102265786 (LOC102265786), mRNA
ENSP00000302724-D1	-4.68	2.28E-04	↑	B. mutus insulin-like 5 (INSL5), mRNA
ENSBTAP00000024015-D1	-4.68	2.28E-04	↑	B. mutus proteasome (prosome, macropain) subunit, beta type, 10 (PSMB10), mRNA
ENSP00000377355-D3	-4.68	2.28E-04	↑	B. mutus PDZ domain containing ring finger 4 (PDZRN4), mRNA
ENSBTAP00000011333-D1	-4.68	2.28E-04	↑	B. mutus high affinity immunoglobulin gamma Fc receptor I-like (LOC102280754), mRNA
ENSBTAP00000017255-D1	-4.68	2.28E-04	↑	B. mutus catechol-O-methyltransferase domain containing 1 (COMTD1), mRNA
ENSBTAP00000014753-D1	-4.68	2.28E-04	↑	B. mutus acyl-CoA thioesterase 12 (ACOT12), mRNA
ENSBTAP00000007283-D8	-4.68	2.28E-04	↑	B. taurus chromobox homolog 3, mRNA (cDNA clone MGC:166015 IMAGE:8248349), complete cds
ENSP00000248598-D1	-4.68	2.28E-04	↑	B. mutus fibrinogen-like 2 (FGL2), mRNA
ENSP00000367345-D5	-4.68	2.28E-04	↑	B. mutus uncharacterized LOC102280142 (LOC102280142), misc_RNA
ENSBTAP00000003883-D1	-4.68	2.28E-04	↑	B. mutus PITPNM family member 3 (PITPNM3), mRNA
ENSBTAP00000053460-D1	-4.68	2.28E-04	↑	B. mutus spermatogenesis associated 16 (SPATA16), mRNA
ENSBTAP00000018207-D1	-4.68	2.28E-04	↑	B. mutus zinc finger protein 296 (ZNF296), mRNA
ENSBTAP00000048971-D1	-4.68	2.28E-04	↑	B. mutus follicle stimulating hormone receptor (FSHR), mRNA
ENSBTAP00000024333-D1	-4.68	2.28E-04	↑	B. mutus vesicle-associated membrane protein 5 (VAMP5), mRNA
ENSBTAP00000023397-D1	-4.68	2.28E-04	↑	B. mutus triggering receptor expressed on myeloid cells 1 (TREM1), mRNA
ENSBTAP00000004318-D1	-4.67	1.07E-34	↑	B. mutus follistatin (FST), mRNA

ENSP00000361813-D1	-4.66	5.29E-18	↑	B. taurus brain expressed X-linked 2 (BEX2), mRNA
ENSBTAP00000031485-D9	-4.62	6.84E-07	↑	Bubalus bubalis multidrug resistance-associated protein 4-like (LOC102398037), mRNA
ENSBTAP00000027731-D1	-4.62	6.84E-07	↑	B. mutus solute carrier family 36 (proton/amino acid symporter), member 2 (SLC36A2), mRNA
ENSP00000265018-D2	-4.62	6.84E-07	↑	B. mutus family with sequence similarity 184, member B (FAM184B), partial mRNA
ENSP00000282588-D1	-4.56	1.15E-11	↑	B. mutus integrin, alpha 1 (ITGA1), mRNA
ENSBTAP00000032984-D1	-4.56	1.24E-06	↑	B. mutus interleukin-37-like (LOC102287673), mRNA
ENSP00000408914-D1	-4.56	1.24E-06	↑	B. mutus RNA-binding protein Nova-1-like (LOC102288208), transcript variant X1, mRNA
ENSP00000349029-D3	-4.56	1.24E-06	↑	B. mutus inositol 1,4,5-trisphosphate receptor, type 3 (ITPR3), mRNA
ENSP00000219919-D1	-4.56	4.16E-04	↑	B. mutus aquaporin 9 (AQP9), transcript variant X1, mRNA
ENSBTAP00000024762-D1	-4.56	4.16E-04	↑	B. mutus thrombospondin-type laminin G domain and EAR repeats (TSPEAR), mRNA
ENSBTAP00000008171-D1	-4.56	4.16E-04	↑	B. mutus thymocyte selection associated family member 2 (THEMIS2), mRNA
ENSBTAP00000046304-D9	-4.56	4.16E-04	↑	B. taurus AFG3-like protein 1 (LOC532875), transcript variant X4, mRNA
ENSBTAP00000019284-D1	-4.56	4.16E-04	↑	B. mutus zinc finger protein 503 (ZNF503), mRNA
ENSP00000419260-D1	-4.56	4.16E-04	↑	B. mutus phosphatidylinositol-4,5-bisphosphate 3-kinase, catalytic subunit gamma (PIK3CG), mRNA
ENSBTAP00000006003-D1	-4.56	4.16E-04	↑	B. mutus paired related homeobox 1 (PRRX1), transcript variant X1, mRNA
ENSBTAP00000004317-D1	-4.56	4.16E-04	↑	B. mutus NLR family, apoptosis inhibitory protein (NAIP), mRNA
yakA19103	-4.56	4.16E-04	↑	B. mutus potassium channel tetramerization domain containing 8 (KCTD8), mRNA
ENSBTAP00000009155-D8	-4.56	4.16E-04	↑	Capra hircus 60S ribosomal protein L12-like (LOC102178143), mRNA
ENSBTAP00000007340-D1	-4.56	4.16E-04	↑	B. taurus decapping exoribonuclease (DXO), mRNA
ENSP00000264360-D1	-4.56	4.16E-04	↑	B. mutus protocadherin 10 (PCDH10), mRNA
ENSP00000378857-D1	-4.56	4.16E-04	↑	B. mutus WD repeat domain 6 (WDR6), mRNA
ENSP00000365118-D3	-4.56	4.16E-04	↑	Pantholops hodgsonii tripartite motif-containing protein 43-like (LOC102329169), partial mRNA
ENSBTAP00000033947-D3	-4.56	4.16E-04	↑	B. mutus shroom family member 4 (SHROOM4), mRNA
ENSP00000303129-D1	-4.56	4.16E-04	↑	B. mutus vesicle amine transport 1-like (VAT1L), mRNA
ENSBTAP00000037103-D1	-4.56	4.16E-04	↑	B. mutus sulfatase 1 (SULF1), mRNA
ENSBTAP00000012255-D1	-4.56	4.16E-04	↑	B. mutus solute carrier family 39 (zinc transporter), member 4 (SLC39A4), mRNA
ENSBTAP00000028657-D1	-4.56	4.16E-04	↑	B. mutus epithelial chloride channel protein-like (LOC102270735), mRNA
ENSBTAP00000018382-D1	-4.56	4.16E-04	↑	B. mutus desmoglein 1 (DSG1), mRNA
ENSBTAP00000034554-D1	-4.56	4.16E-04	↑	B. mutus protein FAM32A-like (LOC102265148), mRNA
ENSP00000407071-D1	-4.56	4.16E-04	↑	Bubalus bubalis leucine, glutamate and lysine rich 1 (LEKR1), transcript variant X1, mRNA
ENSBTAP00000011332-D1	-4.56	4.16E-04	↑	B. mutus inhibitor of CDK, cyclin A1 interacting protein 1 (INCA1), transcript variant X1, mRNA
yakG019336	-4.56	4.16E-04	↑	B. mutus potassium voltage-gated channel, subfamily G, member 2 (KCNG2), mRNA
ENSP00000331062-D1	-4.56	4.16E-04	↑	B. mutus V-set and transmembrane domain containing 4 (VSTM4), mRNA
ENSP00000002829-D1	-4.56	4.16E-04	↑	B. mutus sema domain, immunoglobulin domain, short basic domain, secreted, SEM), transcript variant X1, mRNA
ENSBTAP00000040534-D1	-4.56	4.16E-04	↑	B. mutus 40S ribosomal protein S6-like (LOC102269094), mRNA
yakA13537	-4.52	2.09E-11	↑	B. mutus protein phosphatase 1, regulatory (inhibitor) subunit 14A (PPP1R14A), mRNA
ENSP00000262095-D2	-4.52	2.09E-11	↑	B. mutus one cut homeobox 2 (ONECUT2), mRNA
ENSP00000372750-D1	-4.49	2.26E-06	↑	B. mutus cholinergic receptor, muscarinic 5 (CHRM5), mRNA
ENSP00000257290-D1	-4.43	1.44E-74	↑	B. mutus platelet-derived growth factor receptor, alpha polypeptide (PDGFRA), mRNA
ENSBTAP00000026699-D1	-4.42	1.26E-10	↑	B. mutus kelch-like family member 28 (KLHL28), mRNA
ENSP00000360942-D1	-4.42	4.12E-06	↑	B. mutus Fas cell surface death receptor (FAS), mRNA
ENSP00000316244-D1	-4.42	7.58E-04	↑	B. mutus 5-hydroxytryptamine receptor 1A-like (LOC102264766), mRNA
ENSBTAP00000046506-D1	-4.42	7.58E-04	↑	B. mutus sphingomyelin phosphodiesterase 3-like (LOC102275231), mRNA
ENSP00000365883-D1	-4.42	7.58E-04	↑	B. mutus nudix (nucleoside diphosphate linked moiety X)-type motif 8 (NUDT8), mRNA
ENSP00000365005-D1	-4.42	7.58E-04	↑	B. mutus mitogen-activated protein kinase kinase kinase 19 (MAP3K19), transcript variant X1, mRNA
ENSBTAP00000016263-D1	-4.42	7.58E-04	↑	B. mutus pyridoxamine 5'-phosphate oxidase (PNPO), mRNA
ENSBTAP00000022052-D1	-4.42	7.58E-04	↑	Capra hircus ankyrin repeat domain 26 (ANKRD26), mRNA
ENSP00000357336-D1	-4.42	7.58E-04	↑	B. mutus RUN and SH3 domain containing 1 (RUSC1), transcript variant X1, mRNA
ENSP00000295588-D1	-4.42	7.58E-04	↑	B. mutus protein O-glucosyltransferase 1 (POGLUT1), mRNA
yakG016733	-4.42	7.58E-04	↑	Pantholops hodgsonii heat shock transcription factor, X-linked-like (LOC102332041), misc_RNA
ENSBTAP00000004230-D1	-4.42	7.58E-04	↑	B. mutus zinc finger protein 132 (ZNF132), mRNA
ENSP00000306997-D1	-4.42	7.58E-04	↑	B. mutus myozenin 2 (MYOZ2), mRNA
ENSBTAP00000012751-D1	-4.42	7.58E-04	↑	Bubalus bubalis olfactory receptor 5-like (LOC102395191), mRNA
ENSBTAP00000025119-D1	-4.42	7.58E-04	↑	B. mutus immunoglobulin superfamily, member 6 (IGSF6), mRNA
ENSBTAP00000044542-D1	-4.42	7.58E-04	↑	B. mutus insulin-like growth factor binding protein, acid labile subunit (IGFALS), mRNA

ENSP00000388599-D1	-4.42	7.58E-04	↑	B. mutus prickle homolog 3 (Drosophila) (PRICKLE3), transcript variant X1, mRNA
ENSP00000303549-D1	-4.42	7.58E-04	↑	B. mutus G protein-coupled receptor 82 (GPR82), mRNA
ENSBTAP00000004195-D1	-4.42	7.58E-04	↑	B. mutus sialic acid binding Ig-like lectin 10 (SIGLEC10), mRNA
ENSP00000336812-D1	-4.42	7.58E-04	↑	B. mutus protein FAM19A5-like (LOC102273016), mRNA
ENSP00000276480-D1	-4.42	7.58E-04	↑	B. mutus suppression of tumorigenicity 18 (breast carcinoma) (zinc finger protein) (ST18), mRNA
ENSP00000305714-D1	-4.42	7.58E-04	↑	B. mutus bone morphogenetic protein 1 (BMP1), mRNA
ENSBTAP00000025498-D1	-4.42	7.58E-04	↑	B. mutus mex-3 RNA binding family member A (MEX3A), mRNA
ENSP00000220676-D1	-4.42	7.58E-04	↑	B. mutus retinitis pigmentosa 1 (autosomal dominant) (RP1), mRNA
ENSP00000296604-D1	-4.39	1.15E-23	↑	Bubalus bubalis RAN binding protein 3-like (RANBP3L), transcript variant X1, mRNA
ENSP00000352833-D1	-4.38	3.83E-19	↑	B. taurus protein tyrosine phosphatase, non-receptor type 22 (lymphoid) (PTPN22), mRNA
ENSBTAP00000049986-D1	-4.34	4.19E-10	↑	B. mutus tripartite motif-containing protein 64-like (LOC102273617), mRNA
ENSP00000358327-D1	-4.34	7.52E-06	↑	B. mutus caspase 7, apoptosis-related cysteine peptidase (CASP7), mRNA
ENSBTAP00000027556-D1	-4.28	7.74E-22	↑	B. mutus matrix metallopeptidase 9 (gelatinase B, 92kDa gelatinase, 92kDa type IV collagenase) (MMP9), mRNA
ENSP00000250863-D2	-4.27	6.32E-66	↑	B. mutus deleted in azoospermia-like (DAZL), mRNA
ENSP00000265968-D1	-4.27	1.15E-250	↑	B. mutus cysteine and glycine-rich protein 3 (cardiac LIM protein) (CSRP3), mRNA
ENSP00000231461-D1	-4.27	1.39E-09	↑	B. mutus CMP-N-acetylneuraminate-poly-alpha-2,8-sialyltransferase-like (LOC102280523), mRNA
ENSBTAP00000027359-D1	-4.27	1.38E-05	↑	B. mutus PYD and CARD domain containing (PYCARD), transcript variant X1, mRNA
ENSP00000363727-D1	-4.27	1.39E-03	↑	B. mutus StAR-related lipid transfer (START) domain containing 8 (STARD8), mRNA
ENSP00000314560-D1	-4.27	1.39E-03	↑	B. mutus MAP6 domain containing 1 (MAP6D1), mRNA
ENSBTAP00000020324-D1	-4.27	1.39E-03	↑	B. mutus solute carrier family 26 (anion exchanger), member 11 (SLC26A11), mRNA
ENSP00000316518-D30	-4.27	1.39E-03	↑	B. mutus olfactory receptor 6C74-like (LOC102266447), mRNA
ENSBTAP00000050199-D4	-4.27	1.39E-03	↑	Balaenoptera acutorostrata scammoni transcription elongation factor B polypeptide 2-like, mRNA
ENSBTAP00000053306-D1	-4.27	1.39E-03	↑	B. mutus protein phosphatase 1, regulatory (inhibitor) subunit 1C (PPP1R1C), transcript variant X1, mRNA
ENSP00000222249-D1	-4.27	1.39E-03	↑	B. mutus potassium intermediate/small conductance calcium-activated channel, subfamily N, member 1, mRNA
ENSBTAP00000017571-D1	-4.27	1.39E-03	↑	B. mutus glycoprotein V (platelet) (GP5), mRNA
ENSBTAP00000053088-D1	-4.27	1.39E-03	↑	B. mutus histone H1-like (LOC102272053), mRNA
ENSP00000216144-D1	-4.27	1.39E-03	↑	B. mutus calcium binding protein 7 (CABP7), mRNA
ENSP00000389175-D1	-4.27	1.39E-03	↑	B. mutus glycerate kinase (GLYCTK), transcript variant X1, mRNA
ENSP00000351632-D1	-4.27	1.39E-03	↑	B. mutus family with sequence similarity 3, member D (FAM3D), mRNA
ENSP00000313490-D1	-4.27	1.39E-03	↑	B. mutus phosphoribosylformylglycinamidine synthase (PFAS), mRNA
ENSP00000231021-D1	-4.27	1.39E-03	↑	B. mutus cadherin-9-like (LOC102285586), mRNA
ENSBTAP00000045610-D1	-4.27	1.39E-03	↑	B. mutus leucine-rich, glioma inactivated 1 (LGI1), transcript variant X1, mRNA
ENSP00000230588-D1	-4.27	1.39E-03	↑	B. mutus meprin A, alpha (PABA peptide hydrolase) (MEP1A), mRNA
ENSP00000369979-D1	-4.27	1.39E-03	↑	B. mutus carboxypeptidase X (M14 family), member 1 (CPXM1), transcript variant X1, mRNA
ENSP00000392568-D3	-4.27	1.39E-03	↑	Canis familiaris beta-defensin 109 (CBD109) mRNA, complete cds
ENSP00000325776-D1	-4.27	1.39E-03	↑	B. mutus serine-rich and transmembrane domain containing 1 (SERTM1), mRNA
ENSP00000370936-D2	-4.27	1.39E-03	↑	Bubalus bubalis transmembrane gamma-carboxyglutamic acid protein 1-like, transcript variant X1, mRNA
ENSP00000377303-D1	-4.27	1.39E-03	↑	B. mutus renin binding protein (RENBP), partial mRNA
ENSBTAP00000027848-D1	-4.27	1.39E-03	↑	B. mutus insulin-like 3 (Leydig cell) (INSL3), mRNA
ENSP00000360635-D1	-4.27	1.39E-03	↑	B. taurus dipeptidyl-peptidase 7 (DPP7), mRNA
ENSP00000374782-D82	-4.27	1.39E-03	↑	B. taurus hypothetical protein LOC785621, mRNA (cDNA clone MGC:152637 IMAGE:8436818), complete cds
ENSBTAP00000050702-D8	-4.27	1.39E-03	↑	B. mutus UDP-glucuronosyltransferase 2B31-like (LOC102276699), transcript variant X1, mRNA
ENSBTAP00000011456-D2	-4.27	1.39E-03	↑	B. mutus solute carrier family 6, member 9 (SLC6A9), transcript variant X1, mRNA
ENSBTAP00000048301-D1	-4.27	1.39E-03	↑	B. taurus cDNA clone IMAGE:8656618, partial cds
ENSBTAP00000052585-D1	-4.27	1.39E-03	↑	B. mutus olfactory receptor 4X2-like (LOC102273589), mRNA
ENSP00000366283-D1	-4.27	1.39E-03	↑	B. mutus SLIT and NTRK-like family, member 5 (SLITRK5), mRNA
ENSBTAP00000015039-D1	-4.27	1.39E-03	↑	B. mutus gap junction protein, alpha 10, 62kDa (GJA10), mRNA
ENSP00000366620-D1	-4.27	1.39E-03	↑	Balaenoptera acutorostrata scammoni hexose-6-phosphate dehydrogenase (H6PD), transcript variant X2, mRNA
ENSP00000389813-D2	-4.27	1.39E-03	↑	B. mutus acyl-CoA dehydrogenase family, member 10 (ACAD10), mRNA
ENSP00000333183-D3	-4.27	1.39E-03	↑	B. mutus methyl-CpG-binding domain protein 3-like 1-like (LOC102277844), mRNA
ENSBTAP00000017356-D7	-4.27	1.39E-03	↑	B. mutus cathepsin G-like (LOC102286039), mRNA
ENSBTAP00000044748-D1	-4.27	1.39E-03	↑	B. mutus syntrophin, gamma 1 (SNTG1), mRNA
ENSBTAP00000042635-D5	-4.23	4.70E-21	↑	B. mutus histone H2A.J-like (LOC102285000), mRNA
ENSBTAP00000011619-D1	-4.18	4.64E-09	↑	B. mutus polymerase (RNA) III (DNA directed) polypeptide F, 39 kDa (POLR3F), mRNA
yakA00390	-4.18	4.64E-09	↑	B. mutus 60S ribosomal protein L23a-like (LOC102266460), mRNA

ENSBTAP00000028636-D1	-4.18	4.64E-09	↑	B. mutus tetraspanin 18 (TSPAN18), mRNA
ENSP00000334181-D1	-4.18	4.64E-09	↑	B. mutus lysine (K)-specific demethylase 4D (KDM4D), mRNA
ENSBTAP00000050080-D12	-4.18	4.64E-09	↑	B. taurus BAC CH240-472P12 complete sequence
ENSP00000385213-D1	-4.18	2.50E-05	↑	Capra hircus piggyBac transposable element derived 1 (PGBD1), mRNA
ENSBTAP00000010571-D2	-4.18	2.50E-05	↑	B. mutus cadherin, EGF LAG seven-pass G-type receptor 1 (CELSR1), mRNA
yakA00814	-4.18	2.50E-05	↑	B. mutus exocyst complex component 1-like (LOC102287155), mRNA
yakG035477	-4.18	2.50E-05	↑	B. taurus GTF2I repeat domain containing 1 (GTF2IRD1), mRNA
ENSP00000357440-D1	-4.14	3.20E-23	↑	B. mutus heat shock transcription factor 2 (HSF2), transcript variant X1, mRNA
ENSBTAP00000051260-D1	-4.13	2.25E-267	↑	B. taurus clone 2 JY-1 mRNA, complete cds
ENSBTAP00000051312-D1	-4.10	5.18E-12	↑	B. mutus 3-hydroxybutyrate dehydrogenase, type 2 (BDH2), transcript variant X2, mRNA
ENSP00000392188-D52	-4.10	1.54E-08	↑	B. taurus growth hormone receptor gene, complete cds
ENSP00000330302-D1	-4.10	1.54E-08	↑	B. taurus protocadherin 7 (PCDH7), mRNA
ENSBTAP00000048539-D1	-4.10	4.56E-05	↑	B. mutus lysophosphatidylcholine acyltransferase 2B-like (LOC102268140), mRNA
ENSP00000320025-D2	-4.10	4.56E-05	↑	B. mutus calcium channel, voltage-dependent, beta 1 subunit (CACNB1), transcript variant X1, mRNA
ENSBTAP00000015064-D1	-4.10	4.56E-05	↑	B. mutus ankyrin repeat domain-containing protein 33B-like (LOC102269174), mRNA
yakG029673	-4.10	2.56E-03	↑	B. mutus fibrous sheath CABYR-binding protein-like (LOC102286260), mRNA
ENSP00000365351-D1	-4.10	2.56E-03	↑	B. mutus integrin, beta-like 1 (with EGF-like repeat domains) (ITGBL1), mRNA
ENSBTAP00000020696-D1	-4.10	2.56E-03	↑	Bubalus bubalis complement C3-like (LOC102408787), transcript variant X2, mRNA
ENSBTAP00000004658-D1	-4.10	2.56E-03	↑	B. mutus SET domain containing (lysine methyltransferase) 7 (SETD7), mRNA
ENSBTAP00000028105-D1	-4.10	2.56E-03	↑	B. mutus clavesin 2 (CLVS2), mRNA
ENSBTAP00000010670-D1	-4.10	2.56E-03	↑	B. mutus odd-skipped related transciption factor 1 (OSR1), mRNA
ENSBTAP00000013662-D1	-4.10	2.56E-03	↑	B. mutus uncharacterized LOC102281420 (LOC102281420), mRNA
yakA16304	-4.10	2.56E-03	↑	B. mutus multidrug resistance-associated protein 4-like (LOC102287016), partial mRNA
ENSP00000353557-D1	-4.10	2.56E-03	↑	B. mutus solute carrier family 35, member F1 (SLC35F1), mRNA
ENSBTAP00000011256-D1	-4.10	2.56E-03	↑	B. mutus solute carrier family 22 (organic cation transporter), member 3 (SLC22A3), mRNA
ENSBTAP00000041306-D1	-4.10	2.56E-03	↑	B. mutus family with sequence similarity 3, member B (FAM3B), mRNA
ENSBTAP00000041217-D1	-4.10	2.56E-03	↑	B. mutus family with sequence similarity 228, member A (FAM228A), mRNA
ENSP00000357583-D1	-4.10	2.56E-03	↑	B. mutus uncharacterized LOC102285484 (LOC102285484), mRNA
ENSP00000303158-D1	-4.10	2.56E-03	↑	B. mutus coiled-coil domain-containing protein 8-like (LOC102282935), mRNA
ENSP00000379108-D1	-4.10	2.56E-03	↑	B. mutus methylenetetrahydrofolate dehydrogenase (NADP+ dependent) 2-like (MTHFD2L), mRNA
ENSP00000320849-D1	-4.10	2.56E-03	↑	B. mutus chromosome unknown open reading frame, human C12orf68 (LOC102286603), mRNA
ENSBTAP00000003763-D1	-4.10	2.56E-03	↑	B. mutus tumor necrosis factor (ligand) superfamily, member 4 (TNFSF4), mRNA
ENSP00000404672-D1	-4.10	2.56E-03	↑	B. mutus doublecortin domain containing 5 (DCDC5), mRNA
ENSBTAP00000046490-D3	-4.10	2.56E-03	↑	B. mutus GTP-binding nuclear protein Ran-like (LOC102280145), mRNA
ENSP00000393664-D6	-4.10	2.56E-03	↑	Capra hircus ankyrin repeat domain 26 (ANKRD26), mRNA
ENSBTAP00000042029-D1	-4.10	2.56E-03	↑	B. mutus vascular cell adhesion protein 1-like (LOC102272413), transcript variant X1, mRNA
yakG045875	-4.10	2.56E-03	↑	B. mutus RAD52 motif-containing protein 1-like (LOC102281912), misc_RNA
ENSP00000297130-D1	-4.10	2.56E-03	↑	B. mutus myozenin 3 (MYOZ3), mRNA
ENSP00000351363-D1	-4.10	2.56E-03	↑	B. mutus uncharacterized LOC102279583 (LOC102279583), mRNA
ENSBTAP00000047883-D2	-4.10	2.56E-03	↑	B. mutus tubulin alpha-1D chain-like (LOC102281213), mRNA
ENSP00000323821-D1	-4.10	2.56E-03	↑	B. mutus putative uncharacterized protein FLJ37770-like (LOC102268031), mRNA
ENSP00000369395-D1	-4.10	2.56E-03	↑	B. mutus leucine rich repeat containing 19 (LRRC19), mRNA
ENSBTAP00000012836-D1	-4.10	2.56E-03	↑	B. mutus leukotriene C4 synthase (LTC4S), mRNA
ENSBTAP00000018930-D2	-4.10	2.56E-03	↑	B. taurus tracheal antimicrobial peptide (TAP), mRNA
ENSBTAP00000034061-D1	-4.10	2.56E-03	↑	B. mutus kynurenine 3-monooxygenase (kynurenine 3-hydroxylase) (KMO), mRNA
ENSBTAP00000047565-D2	-4.10	2.56E-03	↑	Pantholops hodgsonii uncharacterized LOC102340024 (LOC102340024), mRNA
ENSP00000368353-D1	-4.10	2.56E-03	↑	B. mutus solute carrier family 10, member 5 (SLC10A5), mRNA
ENSBTAP00000047569-D1	-4.10	2.56E-03	↑	B. taurus solute carrier family 39 (zinc transporter), member 13 (SLC39A13), mRNA
ENSBTAP00000032595-D1	-4.10	2.56E-03	↑	Capra hircus putative helicase Mov10l1-like (LOC102190943), mRNA
ENSBTAP00000028460-D1	-4.10	2.56E-03	↑	B. mutus acidic repeat containing (ACRC), mRNA
ENSP00000364000-D1	-4.08	3.57E-22	↑	B. mutus collagen, type V, alpha 2 (COL5A2), mRNA
ENSBTAP00000015740-D1	-4.08	1.06E-18	↑	B. taurus solute carrier family 38, member 5, mRNA (cDNA clone MGC:139490 IMAGE:8215650), complete cds
ENSBTAP00000002583-D1	-4.05	2.81E-08	↑	B. mutus MAP7 domain containing 1 (MAP7D1), mRNA
ENSP00000353013-D1	-4.05	2.81E-08	↑	B. mutus C-type lectin domain family 14, member A (CLEC14A), mRNA
ENSP00000360366-D1	-4.00	8.35E-05	↑	B. mutus acyl-CoA thioesterase 11 (ACOT11), mRNA

ENSBTAP00000004012-D1	-4.00	8.35E-05	↑	B. mutus solute carrier family 50 (sugar efflux transporter), member 1 (SLC50A1), mRNA
ENSP00000393599-D1	-3.97	6.69E-89	↑	Physeter catodon uncharacterized LOC102993959 (LOC102993959), ncRNA
ENSP00000222573-D1	-3.92	1.30E-16	↑	B. mutus integrin, beta 8 (ITGB8), mRNA
ENSBTAP00000026091-D1	-3.90	1.92E-10	↑	B. mutus peptidase inhibitor 15 (PI15), mRNA
ENSP00000362187-D1	-3.90	1.71E-07	↑	B. mutus family with sequence similarity 102, member A (FAM102A), mRNA
ENSBTAP00000028684-D1	-3.90	1.71E-07	↑	B. mutus phospholipase A2, group X (PLA2G10), mRNA
ENSP00000307479-D1	-3.90	1.71E-07	↑	B. mutus aryl-hydrocarbon receptor nuclear translocator 2 (ARNT2), mRNA
ENSP00000348081-D1	-3.90	1.54E-04	↑	B. mutus mindbomb E3 ubiquitin protein ligase 2 (MIB2), mRNA
ENSBTAP00000039733-D1	-3.90	1.54E-04	↑	B. mutus interferon beta-2-like (LOC102281065), mRNA
ENSBTAP00000008736-D1	-3.90	1.54E-04	↑	B. mutus zinc finger protein 668 (ZNF668), mRNA
ENSBTAP00000012465-D1	-3.90	1.54E-04	↑	B. mutus family with sequence similarity 159, member B (FAM159B), mRNA
ENSBTAP00000007879-D1	-3.90	1.54E-04	↑	Bubalus bubalis death inducer-obliterator 1 (DIDO1), transcript variant X6, mRNA
ENSBTAP00000025749-D2	-3.90	4.74E-03	↑	B. mutus putative olfactory receptor 2W6-like (LOC102286600), mRNA
yakG022620	-3.90	4.74E-03	↑	B. mutus PHD finger protein 11 (PHF11), mRNA
ENSBTAP00000008949-D1	-3.90	4.74E-03	↑	B. mutus kin of IRRE like 3 (Drosophila) (KIRREL3), mRNA
ENSBTAP00000007987-D1	-3.90	4.74E-03	↑	B. mutus interleukin 15 receptor, alpha (IL15RA), mRNA
ENSP00000350896-D1	-3.90	4.74E-03	↑	B. mutus EPH receptor B4 (EPHB4), transcript variant X1, mRNA
ENSBTAP00000010313-D1	-3.90	4.74E-03	↑	B. mutus OTU domain containing 6A (OTUD6A), mRNA
ENSBTAP00000019812-D1	-3.90	4.74E-03	↑	B. mutus DNA (cytosine-5-)-methyltransferase 3-like (DNMT3L), mRNA
ENSBTAP00000023551-D1	-3.90	4.74E-03	↑	B. mutus solute carrier family 39 (zinc transporter), member 3 (SLC39A3), transcript variant X2, mRNA
ENSP00000362238-D1	-3.90	4.74E-03	↑	B. mutus premature ovarian failure, 1B (POF1B), mRNA
ENSBTAP00000033826-D1	-3.90	4.74E-03	↑	B. mutus retinal degeneration 3 (RD3), mRNA
ENSBTAP00000047831-D29	-3.90	4.74E-03	↑	B. mutus olfactory receptor 6-like (LOC102267397), mRNA
ENSP00000407619-D3	-3.90	4.74E-03	↑	B. mutus prohibitin-like (LOC102265733), misc_RNA
ENSBTAP00000030474-D27	-3.90	4.74E-03	↑	B. mutus protocadherin gamma subfamily B, 2 (PCDHGB2), mRNA
ENSBTAP00000007883-D1	-3.90	4.74E-03	↑	B. mutus leucine rich repeat containing 69 (LRRC69), mRNA
ENSP00000350630-D1	-3.90	4.74E-03	↑	B. mutus tolloid-like 2 (TLL2), mRNA
ENSP00000383230-D1	-3.90	4.74E-03	↑	B. mutus inducible T-cell co-stimulator ligand (ICOSLG), mRNA
ENSP00000355736-D1	-3.90	4.74E-03	↑	B. mutus t-complex-associated-testis-expressed 3 (TCTE3), mRNA
ENSP00000261623-D1	-3.90	4.74E-03	↑	B. mutus cytochrome b-245 light chain-like (LOC102270937), mRNA
ENSBTAP00000015745-D1	-3.90	4.74E-03	↑	B. mutus protocadherin 20 (PCDH20), mRNA
ENSBTAP00000042009-D10	-3.90	4.74E-03	↑	B. mutus olfactory receptor 287-like (LOC102280282), mRNA
ENSBTAP00000038812-D1	-3.90	4.74E-03	↑	B. mutus piggyBac transposable element derived 5 (PGBD5), mRNA
ENSP00000378802-D1	-3.90	4.74E-03	↑	Bubalus bubalis transmembrane protein 71 (TMEM71), transcript variant X8, mRNA
ENSP00000191922-D1	-3.90	4.74E-03	↑	B. taurus s SLC9A3R2, transcript variant X1, mRNA
ENSP00000323928-D99	-3.90	4.74E-03	↑	Capra hircus olfactory receptor 1030-like (LOC102188490), mRNA
ENSP00000310594-D1	-3.90	4.74E-03	↑	B. mutus fibronectin type III domain containing 9 (FNDC9), mRNA
ENSP00000408288-D1	-3.90	4.74E-03	↑	Tupaia chinensis protein phosphatase 1, regulatory subunit 3E (PPP1R3E), mRNA
ENSBTAP00000034436-D1	-3.90	4.74E-03	↑	B. mutus uncharacterized LOC102279062 (LOC102279062), mRNA
ENSP00000345436-D1	-3.90	4.74E-03	↑	B. mutus CASK interacting protein 1 (CASKIN1), mRNA
ENSBTAP00000028765-D1	-3.90	4.74E-03	↑	B. mutus sphingomyelin phosphodiesterase, acid-like 3A (SMPDL3A), mRNA
ENSBTAP00000020079-D1	-3.90	4.74E-03	↑	B. mutus glycerophosphodiester phosphodiesterase domain containing 5 (GDPD5), transcript variant X1, mRNA
ENSP00000330393-D1	-3.90	4.74E-03	↑	B. mutus leptin receptor-like (LOC102269476), mRNA
ENSP00000263278-D1	-3.90	4.74E-03	↑	B. mutus hydroxysteroid (17-beta) dehydrogenase 14 (HSD17B14), mRNA
ENSBTAP00000044656-D1	-3.90	4.74E-03	↑	B. mutus NCK-associated protein 1-like (NCKAP1L), mRNA
ENSBTAP00000020205-D1	-3.90	4.74E-03	↑	B. mutus two pore segment channel 2 (TPCN2), mRNA
yakG014013	-3.90	4.74E-03	↑	B. taurus hCG1657980 (LOC100139318), mRNA
ENSBTAP00000051381-D6	-3.90	4.74E-03	↑	Bubalus bubalis 60S ribosomal protein L21-like (LOC102416141), mRNA
ENSP00000337140-D1	-3.90	4.74E-03	↑	Macaca mulatta transcription factor COE1-like (LOC694086), mRNA
ENSP00000222753-D1	-3.90	4.74E-03	↑	B. mutus homeobox A13 (HOXA13), mRNA
ENSBTAP00000011783-D1	-3.90	4.74E-03	↑	B. mutus alkaline phosphatase, liver/bone/kidney (ALPL), mRNA
ENSBTAP00000021606-D1	-3.90	4.74E-03	↑	B. mutus dual oxidase maturation factor 2 (DUOXA2), mRNA
ENSBTAP00000011478-D1	-3.90	4.74E-03	↑	B. mutus uncharacterized LOC102274784 (LOC102274784), mRNA
ENSP00000326018-D1	-3.90	4.74E-03	↑	B. mutus periaxin (PRX), mRNA
ENSBTAP00000039787-D1	-3.90	4.74E-03	↑	B. taurus gamma-aminobutyric-acid receptor beta 1-like (LOC536190), mRNA

ENSBTAP00000040897-D1	-3.90	4.74E-03	↑	Bubalus bubalis fibronectin type III domain containing 5 (FNDC5), transcript variant X2, mRNA
ENSP00000326572-D5	-3.90	4.74E-03	↑	B. mutus ankyrin repeat domain-containing protein 26-like (LOC102283266), mRNA
ENSP00000368983-D25	-3.90	4.74E-03	↑	B. mutus olfactory receptor 6C74-like (LOC102286280), mRNA
ENSP00000395583-D40	-3.90	4.74E-03	↑	Homo sapiens contig freeze2_12509 genomic sequence
ENSP00000307513-D1	-3.90	4.74E-03	↑	B. mutus mannose receptor, C type 2 (MRC2), mRNA
ENSBTAP00000000920-D1	-3.90	4.74E-03	↑	Bubalus bubalis zinc finger and SCAN domain-containing protein 5B-like (LOC102398387), partial mRNA
ENSP00000408494-D15	-3.90	4.74E-03	↑	.
ENSP00000365343-D15	-3.90	4.74E-03	↑	B. mutus uncharacterized LOC102265782 (LOC102265782), mRNA
ENSP00000246186-D3	-3.90	4.74E-03	↑	B. mutus matrix metallopeptidase 14 (membrane-inserted) (MMP14), mRNA
ENSP00000419199-D1	-3.90	4.74E-03	↑	B. mutus ankyrin repeat and SOCS box containing 14 (ASB14), mRNA
ENSP00000392188-D1	-3.90	4.74E-03	↑	Pan troglodytes BAC clone CH251-452L20 from chromosome 7, complete sequence
ENSBTAP00000050320-D53	-3.90	4.74E-03	↑	B. mutus olfactory receptor 2B2-like (LOC102269771), mRNA
yakG045446	-3.90	4.74E-03	↑	B. mutus protein kinase C epsilon type-like (LOC102283594), partial mRNA
ENSP00000400588-D1	-3.90	4.74E-03	↑	B. mutus kynurenine/alpha-aminoadipate aminotransferase, mitochondrial-like (LOC102270370), mRNA
ENSP00000407306-D1	-3.90	4.74E-03	↑	Lipotes vexillifer high mobility group AT-hook 2 (HMGA2), mRNA
ENSP00000419718-D1	-3.90	4.74E-03	↑	Bubalus bubalis uncharacterized LOC102406751 (LOC102406751), partial mRNA
ENSBTAP00000043951-D2	-3.90	4.74E-03	↑	B. taurus brain expressed X-linked 2 (BEX2), mRNA
ENSP00000378238-D1	-3.89	4.89E-19	↑	B. mutus ecotropic viral integration site 2A (EVI2A), mRNA
ENSP00000377542-D1	-3.85	3.10E-07	↑	Capra hircus WW domain binding protein 1 (WBP1), transcript variant X1, mRNA
ENSBTAP00000006351-D1	-3.82	1.30E-12	↑	B. mutus kinesin family member 21A (KIF21A), transcript variant X2, mRNA
ENSBTAP00000006118-D1	-3.82	1.30E-12	↑	Bubalus bubalis solute carrier family 16, member 12 (SLC16A12), mRNA
ENSP00000344609-D1	-3.82	2.51E-26	↑	B. mutus BTG family, member 3 (BTG3), transcript variant X3, mRNA
ENSP00000319635-D2	-3.82	5.40E-18	↑	B. mutus c-X-C chemokine receptor type 2-like (LOC102269794), mRNA
ENSBTAP00000043722-D1	-3.80	5.65E-07	↑	B. mutus family with sequence similarity 134, member B (FAM134B), mRNA
ENSBTAP00000006033-D2	-3.80	2.80E-04	↑	B. mutus transgelin 3 (TAGLN3), transcript variant X2, mRNA
ENSP00000226359-D1	-3.80	2.80E-04	↑	B. mutus alpha-fetoprotein-like (LOC102267570), mRNA
ENSBTAP00000049066-D1	-3.80	2.80E-04	↑	B. mutus potassium large conductance calcium-activated channel, subfamily M, beta member 1, mRNA
ENSP00000378396-D1	-3.80	2.80E-04	↑	B. mutus 60S ribosomal protein L23a-like (LOC102286665), mRNA
ENSP00000340434-D1	-3.80	2.80E-04	↑	B. mutus leucine zipper, down-regulated in cancer 1-like (LDOC1L), mRNA
ENSBTAP00000048983-D1	-3.80	2.80E-04	↑	B. mutus PQ loop repeat containing 1 (PQLC1), mRNA
ENSBTAP00000017670-D1	-3.80	2.80E-04	↑	B. mutus smoothened, frizzled family receptor (SMO), mRNA
ENSBTAP00000019142-D1	-3.77	4.33E-12	↑	B. taurus Kruppel-like factor 10, mRNA (cDNA clone IMAGE:8494660), partial cds
ENSBTAP00000050477-D1	-3.74	1.03E-06	↑	B. mutus ras-related GTP-binding protein B-like (LOC102281123), mRNA
ENSP00000418287-D1	-3.74	1.03E-06	↑	B. mutus cytosolic iron-sulfur protein assembly 1 (CIAO1), mRNA
ENSP00000351163-D1	-3.73	2.91E-14	↑	B. taurus collagen, type XI, alpha 1 (COL11A1), mRNA
ENSP00000281131-D1	-3.70	1.02E-33	↑	B. mutus ankyrin repeat domain 50 (ANKRD50), mRNA
ENSBTAP00000025164-D1	-3.68	2.62E-11	↑	B. mutus glucose-fructose oxidoreductase domain containing 2 (GFOD2), transcript variant X2, mRNA
ENSP00000325123-D1	-3.68	1.88E-06	↑	B. mutus zinc finger and SCAN domain containing 2 (ZSCAN2), mRNA
ENSBTAP00000045688-D1	-3.68	5.11E-04	↑	Bubalus bubalis zymogen granule protein 16 homolog B-like (LOC102394887), mRNA
ENSBTAP00000046249-D1	-3.68	5.11E-04	↑	Pantholops hodgsonii uncharacterized LOC102324023 (LOC102324023), misc_RNA
ENSP00000329499-D1	-3.68	5.11E-04	↑	B. mutus Ig-like V-type domain-containing protein FAM187A-like (LOC102276394), mRNA
ENSP00000363827-D1	-3.68	5.11E-04	↑	B. mutus heparan sulfate proteoglycan 2 (HSPG2), partial mRNA
ENSP00000319851-D1	-3.68	5.11E-04	↑	B. mutus choline dehydrogenase (CHDH), mRNA
ENSP00000350894-D1	-3.68	5.11E-04	↑	B. mutus serpin peptidase inhibitor, clade H, member 1, (collagen binding protein 1) (SERPINH1), mRNA
ENSP00000414649-D1	-3.68	8.73E-03	↑	B. taurus gene for bitter taste receptor, partial cds, clone: Bota-T2R39
ENSBTAP00000044908-D1	-3.68	8.73E-03	↑	B. mutus family with sequence similarity 84, member B (FAM84B), mRNA
ENSBTAP00000051608-D1	-3.68	8.73E-03	↑	B. mutus olfactory receptor 11H7-like (LOC102271454), mRNA
ENSP00000325203-D86	-3.68	8.73E-03	↑	B. mutus olfactory receptor 49-like (LOC102279217), mRNA
ENSBTAP00000008122-D1	-3.68	8.73E-03	↑	B. taurus DnaJ (Hsp40) homolog, subfamily B, member 3 (DNAJB3), mRNA
ENSP00000383047-D1	-3.68	8.73E-03	↑	Capra hircus CUB and Sushi multiple domains 1 (CSMD1), mRNA
ENSBTAP00000050983-D26	-3.68	8.73E-03	↑	B. mutus olfactory receptor 4K1-like (LOC102278859), mRNA
ENSBTAP00000000146-D1	-3.68	8.73E-03	↑	B. mutus CD68 molecule (CD68), mRNA
ENSBTAP00000000491-D1	-3.68	8.73E-03	↑	B. mutus chromosome unknown open reading frame, human C12orf39 (LOC102264850), mRNA
ENSBTAP00000007633-D1	-3.68	8.73E-03	↑	B. mutus epoxide hydrolase 4 (EPHX4), mRNA
ENSBTAP00000023275-D1	-3.68	8.73E-03	↑	B. taurus cysteinyl leukotriene receptor 2, mRNA (cDNA clone MGC:179430 IMAGE:8986550), complete cds

ENSP00000361125-D1	-3.68	8.73E-03	↑	Macaca fascicularis vascular endothelial growth factor A (VEGFA), transcript variant X1, mRNA
ENSP00000327687-D1	-3.68	8.73E-03	↑	Pantholops hodgsonii glutamate receptor, ionotropic, kainate 1 (GRIK1), transcript variant X4, mRNA
ENSBTAP00000049627-D1	-3.68	8.73E-03	↑	B. mutus left-right determination factor 2-like (LOC102267059), mRNA
ENSBTAP00000010825-D1	-3.68	8.73E-03	↑	B. mutus BEN domain containing 6 (BEND6), mRNA
ENSP00000338297-D1	-3.68	8.73E-03	↑	B. mutus insulin-like growth factor 2 (somatomedin A) (IGF2), mRNA
ENSP00000231130-D41	-3.68	8.73E-03	↑	B. mutus uncharacterized LOC102279859 (LOC102279859), misc_RNA
ENSP00000310335-D1	-3.68	8.73E-03	↑	B. mutus RAB15 effector protein (REP15), mRNA
ENSBTAP00000007819-D1	-3.68	8.73E-03	↑	B. mutus protein tyrosine kinase 2 beta (PTK2B), transcript variant X1, mRNA
ENSBTAP00000045314-D1	-3.68	8.73E-03	↑	B. mutus neuropeptide Y receptor Y5 (NPY5R), mRNA
ENSBTAP00000049067-D1	-3.68	8.73E-03	↑	B. taurus T cell receptor, alpha, mRNA (cDNA clone MGC:139847 IMAGE:8283961), complete cds
ENSBTAP00000028438-D1	-3.68	8.73E-03	↑	B. mutus renin (REN), transcript variant X1, mRNA
ENSBTAP00000020040-D1	-3.68	8.73E-03	↑	B. mutus retinol binding protein 2, cellular (RBP2), mRNA
ENSP00000310585-D1	-3.68	8.73E-03	↑	B. taurus Purkinje cell protein 2 (PCP2), mRNA
ENSP00000338532-D1	-3.68	8.73E-03	↑	B. mutus family with sequence similarity 53, member B (FAM53B), mRNA
ENSBTAP00000019292-D6	-3.68	8.73E-03	↑	Bubalus bubalis olfactory receptor 8H1-like (LOC102414683), mRNA
ENSBTAP00000040857-D1	-3.68	8.73E-03	↑	B. mutus IQ motif containing C (IQCC), mRNA
ENSP00000308361-D1	-3.68	8.73E-03	↑	B. mutus purinergic receptor P2Y, G-protein coupled, 14 (P2RY14), mRNA
ENSP00000367345-D4	-3.68	8.73E-03	↑	B. taurus protocadherin gamma subfamily A, 8 (PCDHGA8), mRNA
ENSP00000399863-D1	-3.68	8.73E-03	↑	B. mutus zinc finger protein 839 (ZNF839), mRNA
ENSP00000420211-D1	-3.68	8.73E-03	↑	B. mutus ral guanine nucleotide dissociation stimulator-like 2 (RGL2), mRNA
ENSBTAP00000053734-D1	-3.68	8.73E-03	↑	B. mutus Myb/SANT-like DNA-binding domain containing 2 (MSANTD2), mRNA
yakG033364	-3.68	8.73E-03	↑	Camelus ferus 40S ribosomal protein S23-like (LOC102517640), mRNA
ENSBTAP00000027792-D2	-3.68	8.73E-03	↑	Bubalus bubalis UPF3 regulator of nonsense transcripts homolog B (yeast) (UPF3B), transcript variant X2, mRNA
ENSBTAP00000048025-D1	-3.68	8.73E-03	↑	B. mutus NKG2-D type II integral membrane protein-like (LOC102285170), mRNA
ENSBTAP00000035880-D5	-3.68	8.73E-03	↑	B. mutus toll-like receptor 1 (TLR1), mRNA
ENSBTAP00000000176-D1	-3.68	8.73E-03	↑	B. mutus leucine rich repeat and fibronectin type III domain containing 3 (LRFN3), mRNA
ENSP00000324403-D1	-3.68	8.73E-03	↑	B. mutus PHD finger protein 21B (PHF21B), mRNA
ENSBTAP00000025291-D1	-3.68	8.73E-03	↑	B. mutus solute carrier family 2 (facilitated glucose transporter), member 12 (SLC2A12), mRNA
ENSBTAP00000024756-D1	-3.68	8.73E-03	↑	B. mutus lysozyme-like 4 (LYZL4), mRNA
ENSBTAP00000042828-D1	-3.68	8.73E-03	↑	B. mutus nephronophthisis 4 (NPHP4), mRNA
ENSBTAP00000017851-D1	-3.68	8.73E-03	↑	B. mutus cystatin 11 (CST11), mRNA
ENSP00000331302-D2	-3.68	8.73E-03	↑	B. mutus transcription elongation factor B polypeptide 3-like (LOC102282977), mRNA
ENSBTAP00000020444-D1	-3.68	8.73E-03	↑	B. mutus solute carrier family 16, member 4 (SLC16A4), mRNA
ENSBTAP00000025912-D1	-3.68	8.73E-03	↑	B. mutus prostaglandin E synthase (PTGES), mRNA
yakG007284	-3.68	8.73E-03	↑	B. mutus protocadherin-16-like (LOC102285931), mRNA
ENSBTAP00000006590-D1	-3.68	8.73E-03	↑	B. mutus uncharacterized LOC102282939 (LOC102282939), mRNA
ENSBTAP00000000306-D1	-3.68	8.73E-03	↑	B. mutus malic enzyme 3, NADP(+)-dependent, mitochondrial (ME3), mRNA
ENSP00000367373-D8	-3.68	8.73E-03	↑	Capra hircus protocadherin alpha-7-like (LOC102187401), mRNA
ENSBTAP00000005736-D1	-3.68	8.73E-03	↑	B. mutus epithelial splicing regulatory protein 2 (ESRP2), mRNA
ENSP00000372900-D1	-3.68	8.73E-03	↑	B. mutus piezo-type mechanosensitive ion channel component 2 (PIEZO2), mRNA
ENSBTAP00000006870-D1	-3.68	8.73E-03	↑	B. mutus interleukin 10 receptor, alpha (IL10RA), mRNA
ENSP00000335566-D2	-3.68	8.73E-03	↑	B. taurus keratin-associated protein 21-1 (LOC785105), mRNA
ENSBTAP00000037706-D1	-3.68	8.73E-03	↑	B. mutus sulfotransferase 6B1-like (LOC102273225), mRNA
ENSBTAP00000052960-D15	-3.68	8.73E-03	↑	B. mutus interferon omega-1-like (LOC102282615), mRNA
ENSBTAP00000008192-D1	-3.68	8.73E-03	↑	B. mutus glucosaminyl (N-acetyl) transferase family member 7 (GCNT7), mRNA
ENSP00000358045-D1	-3.68	8.73E-03	↑	B. mutus extracellular matrix protein 1 (ECM1), transcript variant X1, mRNA
ENSP00000250160-D1	-3.68	8.73E-03	↑	B. mutus WNT1 inducible signaling pathway protein 1 (WISP1), mRNA
ENSBTAP00000029915-D3	-3.68	8.73E-03	↑	Bubalus bubalis retinoblastoma binding protein 4 (RBBP4), transcript variant X1, mRNA
ENSBTAP00000013144-D1	-3.68	8.73E-03	↑	B. mutus enoyl-CoA delta isomerase 1 (ECI1), mRNA
ENSP00000384644-D1	-3.68	8.73E-03	↑	B. mutus kelch-like family member 31 (KLHL31), mRNA
ENSBTAP00000016346-D3	-3.68	8.73E-03	↑	B. mutus purine nucleoside phosphorylase-like (LOC102270686), mRNA
ENSBTAP00000049432-D3	-3.68	8.73E-03	↑	B. mutus chymotrypsinogen A-like (LOC102286947), mRNA
ENSP00000312273-D1	-3.68	8.73E-03	↑	B. taurus leucine-rich repeat LGI family, member 4 (LGI4), mRNA
ENSP00000321026-D1	-3.68	8.73E-03	↑	B. taurus leucine rich adaptor protein 1-like (LURAP1L), mRNA
ENSBTAP00000026223-D1	-3.68	8.73E-03	↑	B. mutus melanocortin receptor 4-like (LOC102286686), mRNA

ENSBTAP00000015756-D1	-3.68	8.73E-03	↑	B. taurus echinoderm microtubule associated protein like 3 (EML3), mRNA
ENSBTAP00000050251-D1	-3.68	8.73E-03	↑	B. mutus chondroadherin-like (CHADL), mRNA
ENSBTAP00000010273-D1	-3.68	8.73E-03	↑	Human mariner1 transposase gene, complete consensus sequence
ENSBTAP00000047568-D1	-3.68	8.73E-03	↑	B. mutus chromosome unknown open reading frame, human C11orf63, transcript variant X1, mRNA
ENSBTAP00000007323-D1	-3.68	8.73E-03	↑	B. mutus zinc finger protein 205 (ZNF205), transcript variant X2, mRNA
ENSP00000355510-D1	-3.68	8.73E-03	↑	B. mutus WD repeat domain 64 (WDR64), mRNA
ENSBTAP00000017584-D1	-3.68	8.73E-03	↑	B. mutus odd-skipped related transciption factor 2 (OSR2), transcript variant X3, mRNA
ENSBTAP00000003192-D1	-3.68	8.73E-03	↑	B. mutus tubulin, alpha 4a (TUBA4A), transcript variant X1, mRNA
ENSBTAP00000049839-D67	-3.68	8.73E-03	↑	B. mutus olfactory receptor 9K2-like (LOC102269265), misc_RNA
ENSP00000356429-D1	-3.68	8.73E-03	↑	B. mutus regulator of G-protein signaling 1 (RGS1), mRNA
ENSBTAP00000000066-D1	-3.68	8.73E-03	↑	B. mutus PR domain containing 14 (PRDM14), mRNA
ENSP00000384482-D39	-3.68	8.73E-03	↑	Bubalus bubalis zinc finger and SCAN domain-containing protein 5B-like (LOC102413599), partial mRNA
ENSP00000296452-D1	-3.68	8.73E-03	↑	B. mutus bassoon presynaptic cytomatrix protein (BSN), mRNA
ENSP00000232744-D1	-3.68	8.73E-03	↑	B. mutus ankyrin repeat and BTB (POZ) domain containing 1 (ABTB1), mRNA
ENSBTAP00000024449-D1	-3.68	8.73E-03	↑	B. mutus StAR-related lipid transfer (START) domain containing 7 (STARD7), mRNA
ENSBTAP00000050243-D4	-3.68	8.73E-03	↑	.
ENSBTAP00000009016-D3	-3.68	8.73E-03	↑	Bubalus bubalis C-type lectin domain family 2 member D11-like (LOC102402803), mRNA
ENSP00000409223-D1	-3.68	8.73E-03	↑	Bubalus bubalis interaptin-like (LOC102391435), mRNA
ENSBTAP00000021983-D1	-3.64	1.28E-08	↑	B. taurus mitogen-activated protein kinase-activated protein kinase 3 (MAPKAPK3), transcript variant X1, mRNA
ENSBTAP00000031689-D1	-3.63	3.21E-13	↑	B. taurus paternally expressed gene 3 (Peg3) mRNA, complete cds
ENSBTAP00000006849-D1	-3.62	8.67E-11	↑	B. mutus tRNA-yW synthesizing protein 3 homolog (S. cerevisiae) (TYW3), transcript variant X1, mRNA
ENSBTAP00000002867-D1	-3.61	8.99E-43	↑	B. mutus glutamine-fructose-6-phosphate transaminase 2 (GFPT2), mRNA
ENSP00000266718-D1	-3.60	0.00E+00	↑	B. mutus lumican (LUM), mRNA
ENSBTAP00000024623-D1	-3.60	2.31E-08	↑	B. mutus tubby like protein 1 (TULP1), mRNA
ENSBTAP00000046522-D2	-3.59	1.44E-39	↑	B. mutus serpin B8-like (LOC102277521), mRNA
ENSP00000250617-D1	-3.56	2.86E-10	↑	B. mutus Rac/Cdc42 guanine nucleotide exchange factor (GEF) 6 (ARHGEF6), mRNA
ENSP00000240587-D1	-3.56	6.22E-06	↑	B. mutus teashirt zinc finger homeobox 3 (TSHZ3), mRNA
ENSP00000331210-D1	-3.56	9.32E-04	↑	B. mutus brevican (BCAN), transcript variant X1, mRNA
ENSBTAP00000014051-D1	-3.56	9.32E-04	↑	B. mutus mucin 1, cell surface associated (MUC1), mRNA
ENSP00000305288-D2	-3.56	9.32E-04	↑	B. mutus neuroligin 2 (NLGN2), mRNA
ENSP00000385026-D1	-3.56	9.32E-04	↑	B. mutus cholinergic receptor, nicotinic, alpha 2 (neuronal) (CHRNA2), transcript variant X1, mRNA
ENSBTAP00000035589-D1	-3.56	9.32E-04	↑	B. mutus probable phospholipid-transporting ATPase IB-like (LOC102277904), mRNA
ENSBTAP00000034542-D1	-3.56	9.32E-04	↑	B. mutus collagen alpha-1(XVIII) chain-like (LOC102285924), mRNA
ENSP00000410938-D1	-3.56	9.32E-04	↑	B. mutus catechol-O-methyltransferase (COMT), mRNA
ENSBTAP00000003503-D1	-3.56	9.32E-04	↑	B. mutus glutathione S-transferase zeta 1 (GSTZ1), transcript variant X2, mRNA
ENSP00000375073-D1	-3.56	9.32E-04	↑	B. mutus EP300 interacting inhibitor of differentiation 2 (EID2), mRNA
ENSBTAP00000047588-D1	-3.56	9.32E-04	↑	B. mutus L-gulonolactone oxidase-like (LOC102268085), mRNA
ENSBTAP00000047813-D1	-3.56	9.32E-04	↑	B. mutus protease, serine, 50 (PRSS50), mRNA
ENSBTAP00000016092-D1	-3.56	9.32E-04	↑	B. mutus metalloproteinase inhibitor 1-like (LOC102275743), mRNA
ENSBTAP00000008907-D1	-3.54	1.57E-16	↑	B. mutus phosphoglycerate dehydrogenase (PHGDH), mRNA
ENSP00000353922-D1	-3.53	3.23E-31	↑	B. mutus zinc finger protein 711 (ZNF711), transcript variant X1, mRNA
ENSBTAP00000026804-D1	-3.51	4.25E-14	↑	B. taurus HtrA serine peptidase 2 (HTRA2), mRNA
ENSP00000389066-D1	-3.51	7.64E-08	↑	B. mutus DEAH (Asp-Glu-Ala-His) box polypeptide 33 (DHX33), transcript variant X1, mRNA
ENSP00000020945-D1	-3.50	5.16E-16	↑	B. mutus snail family zinc finger 2 (SNAI2), mRNA
ENSBTAP00000043833-D1	-3.50	2.44E-123	↑	B. mutus adipogenesis regulatory factor (ADIRF), transcript variant X1, mRNA
ENSP00000251127-D1	-3.49	1.13E-05	↑	B. mutus sodium leak channel, non-selective (NALCN), mRNA
ENSBTAP00000001371-D1	-3.49	1.13E-05	↑	B. mutus interleukin 18 receptor 1 (IL18R1), mRNA
ENSP00000314103-D1	-3.47	1.38E-07	↑	B. mutus ankyrin repeat domain 34A (ANKRD34A), mRNA
ENSP00000268603-D1	-3.45	1.70E-09	↑	B. mutus cadherin 11, type 2, OB-cadherin (osteoblast) (CDH11), mRNA
ENSBTAP00000012721-D1	-3.45	1.70E-09	↑	B. mutus lysophosphatidic acid receptor 1 (LPAR1), mRNA
ENSP00000338967-D1	-3.42	8.35E-19	↑	B. mutus Rho GTPase activating protein 6 (ARHGAP6), mRNA
ENSBTAP00000004291-D1	-3.42	3.77E-11	↑	B. taurus mitochondrial ribosomal protein L57 (MRPL57), mRNA
ENSBTAP00000030128-D13	-3.42	3.07E-09	↑	B. mutus AHNAK nucleoprotein (AHNAK), mRNA
ENSBTAP00000007733-D1	-3.42	3.07E-09	↑	Bubalus bubalis zinc finger protein 333 (ZNF333), transcript variant X4, mRNA
ENSP00000306396-D1	-3.42	2.04E-05	↑	B. taurus ring finger protein 187 (RNF187), mRNA

ENSP00000407031-D1	-3.42	2.04E-05	↑	B. mutus NUT midline carcinoma, family member 1 (NUTM1), mRNA
ENSP00000404334-D1	-3.42	2.04E-05	↑	B. mutus HemK methyltransferase family member 1 (HEMK1), mRNA
ENSBTAP00000044692-D2	-3.42	1.70E-03	↑	Bubalus bubalis solute carrier family 35, member F2 (SLC35F2), mRNA
ENSP00000284274-D1	-3.42	1.70E-03	↑	B. mutus family with sequence similarity 105, member B (FAM105B), mRNA
ENSBTAP00000017835-D2	-3.42	1.70E-03	↑	B. mutus cysteine and glycine-rich protein 1 (CSRP1), transcript variant X1, mRNA
ENSP00000341479-D1	-3.42	1.70E-03	↑	B. mutus potassium inwardly-rectifying channel, subfamily J, member 14 (KCNJ14), mRNA
ENSP00000386419-D1	-3.42	1.70E-03	↑	B. taurus mitotic spindle organizing protein 2B (MZT2B), transcript variant X1, mRNA
ENSP00000343331-D1	-3.42	1.70E-03	↑	B. mutus lipase, member I (LIPI), mRNA
ENSBTAP00000043710-D1	-3.42	1.61E-02	↑	B. mutus tuftelin 1 (TUFT1), transcript variant X1, mRNA
ENSP00000309504-D1	-3.42	1.61E-02	↑	B. mutus solute carrier family 26 (anion exchanger), member 7 (SLC26A7), mRNA
ENSBTAP00000017342-D1	-3.42	1.61E-02	↑	B. mutus glutamate receptor, metabotropic 7 (GRM7), transcript variant X3, mRNA
ENSBTAP00000051739-D1	-3.42	1.61E-02	↑	B. mutus olfactory receptor 52B4-like (LOC102273223), mRNA
ENSP00000416993-D2	-3.42	1.61E-02	↑	B. mutus glutathione S-transferase theta-4-like (LOC102276933), mRNA
ENSP00000315265-D1	-3.42	1.61E-02	↑	Pantholops hodgsonii mucin-2-like (LOC102327357), mRNA
ENSBTAP00000016647-D1	-3.42	1.61E-02	↑	B. mutus calcineurin-like EF-hand protein 2 (CHP2), mRNA
ENSP00000328190-D1	-3.42	1.61E-02	↑	B. mutus septin 5 (SEPT5), partial mRNA
ENSBTAP00000024371-D1	-3.42	1.61E-02	↑	B. mutus chemokine (C-X-C motif) ligand 16 (CXCL16), mRNA
ENSBTAP00000000977-D1	-3.42	1.61E-02	↑	B. mutus BCL2-related protein A1 (BCL2A1), mRNA
ENSBTAP00000026027-D1	-3.42	1.61E-02	↑	B. mutus androgen-dependent TFPI-regulating protein (ADTRP), mRNA
ENSP00000413540-D1	-3.42	1.61E-02	↑	Bubalus bubalis uncharacterized LOC102413129 (LOC102413129), mRNA
ENSP00000327890-D1	-3.42	1.61E-02	↑	B. mutus uncharacterized LOC102279846 (LOC102279846), mRNA
ENSBTAP00000027390-D1	-3.42	1.61E-02	↑	Bubalus bubalis beta-defensin 36-like (LOC102412939), mRNA
ENSP00000267845-D1	-3.42	1.61E-02	↑	B. mutus histidine decarboxylase (HDC), transcript variant X1, mRNA
ENSBTAP00000051802-D1	-3.42	1.61E-02	↑	B. taurus magnesium transporter 1-like pseudogene (LOC100138302), non-coding RNA
ENSBTAP00000029297-D1	-3.42	1.61E-02	↑	B. mutus single-strand-selective monofunctional uracil-DNA glycosylase 1, transcript variant X2, mRNA
ENSBTAP00000019930-D1	-3.42	1.61E-02	↑	B. mutus prostaglandin E receptor 4 (subtype EP4) (PTGER4), mRNA
ENSBTAP00000012813-D1	-3.42	1.61E-02	↑	Bubalus bubalis Rho GTPase activating protein 33 (ARHGAP33), transcript variant X2, mRNA
ENSBTAP00000003821-D1	-3.42	1.61E-02	↑	B. mutus egf-like module containing, mucin-like, hormone receptor-like 3 (EMR3), mRNA
ENSBTAP00000041537-D24	-3.42	1.61E-02	↑	B. mutus olfactory receptor 6C68-like (LOC102272803), mRNA
ENSP00000369560-D26	-3.42	1.61E-02	↑	B. mutus interferon alpha-2-like (LOC102281344), mRNA
ENSP00000167106-D1	-3.42	1.61E-02	↑	B. mutus vasohibin 1 (VASH1), mRNA
ENSBTAP00000012724-D1	-3.42	1.61E-02	↑	B. mutus pleckstrin (PLEK), mRNA
ENSBTAP00000003873-D1	-3.42	1.61E-02	↑	B. mutus flavin containing monooxygenase 2 (non-functional) (FMO2), mRNA
ENSBTAP00000025871-D1	-3.42	1.61E-02	↑	B. mutus dishevelled-binding antagonist of beta-catenin 1 (DACT1), mRNA
ENSP00000316395-D4	-3.42	1.61E-02	↑	B. mutus EPH receptor A5 (EPHA5), transcript variant X4, mRNA
ENSBTAP00000050009-D1	-3.42	1.61E-02	↑	Sus scrofa fibrous sheath-interacting protein 2-like (LOC100626097), misc_RNA
ENSP00000284311-D1	-3.42	1.61E-02	↑	B. mutus G protein-coupled receptor 15 (GPR15), mRNA
ENSBTAP00000004568-D1	-3.42	1.61E-02	↑	B. mutus heat shock transcription factor 4 (HSF4), mRNA
ENSP00000367345-D7	-3.42	1.61E-02	↑	Bubalus bubalis protocadherin gamma subfamily A, 7 (PCDHGA7), mRNA
ENSBTAP00000003307-D1	-3.42	1.61E-02	↑	B. mutus potassium inwardly-rectifying channel, subfamily J, member 8 (KCNJ8), mRNA
ENSBTAP00000007235-D1	-3.42	1.61E-02	↑	B. mutus tumor protein p53 inducible nuclear protein 2 (TP53INP2), mRNA
ENSBTAP00000045311-D1	-3.42	1.61E-02	↑	B. mutus solute carrier family 9, member C2 (putative) (SLC9C2), mRNA
ENSBTAP00000021785-D8	-3.42	1.61E-02	↑	B. mutus cytochrome P450 4X1-like (LOC102274990), mRNA
ENSP00000265708-D1	-3.42	1.61E-02	↑	B. mutus ADAM metallopeptidase domain 2 (ADAM2), mRNA
ENSBTAP00000001065-D1	-3.42	1.61E-02	↑	B. mutus IQ motif containing F1 (IQCF1), mRNA
ENSBTAP00000050036-D48	-3.42	1.61E-02	↑	B. taurus olfactory receptor 1030 (LOC618091), mRNA
ENSP00000361795-D1	-3.42	1.61E-02	↑	B. mutus dysbindin (dystrobrevin binding protein 1) domain containing 2 (DBNDD2), mRNA
yakG011588	-3.42	1.61E-02	↑	B. mutus hyaluronan synthase 2-like (LOC102284172), mRNA
ENSBTAP00000018404-D1	-3.42	1.61E-02	↑	Bubalus bubalis single-stranded DNA binding protein 2 (SSBP2), transcript variant X2, mRNA
ENSBTAP00000030053-D1	-3.42	1.61E-02	↑	B. mutus androgen receptor-like (LOC102270158), mRNA
ENSBTAP00000022462-D1	-3.42	1.61E-02	↑	B. mutus metallothionein-1A-like (LOC102271915), mRNA
ENSP00000361818-D1	-3.42	1.61E-02	↑	B. mutus syndecan 4 (SDC4), mRNA
ENSBTAP00000033767-D1	-3.42	1.61E-02	↑	Bubalus bubalis collagen, type XXVIII, alpha 1 (COL28A1), mRNA
ENSP00000318635-D5	-3.42	1.61E-02	↑	Bubalus bubalis small ubiquitin-related modifier 2-like (LOC102396148), mRNA
ENSP00000418169-D1	-3.42	1.61E-02	↑	B. mutus synaptophysin (SYP), mRNA

ENSBTAP00000042409-D3	-3.42	1.61E-02	↑	B. mutus histone H4-like (LOC102274007), mRNA
ENSBTAP00000024926-D1	-3.42	1.61E-02	↑	B. mutus filamin binding LIM protein 1 (FBLIM1), transcript variant X1, mRNA
ENSBTAP00000047083-D1	-3.42	1.61E-02	↑	B. mutus arylsulfatase E (chondrodysplasia punctata 1) (ARSE), mRNA
ENSP00000372649-D1	-3.42	1.61E-02	↑	B. mutus proline-rich protein 23C-like (LOC102281114), mRNA
ENSP00000368245-D1	-3.42	1.61E-02	↑	B. mutus chromosome unknown open reading frame, human CXorf21 (LOC102266006), mRNA
ENSP00000262041-D1	-3.42	1.61E-02	↑	B. mutus mesenchyme homeobox 2 (MEOX2), mRNA
yakG023494	-3.42	1.61E-02	↑	Bubalus bubalis methyltransferase like 7A (METTL7A), mRNA
yakG041951	-3.42	1.61E-02	↑	Pantholops hodgsonii ribosomal protein S7 (RPS7), transcript variant X1, mRNA
ENSBTAP00000049732-D1	-3.42	1.61E-02	↑	B. mutus olfactory receptor 8J2-like (LOC102280590), mRNA
ENSBTAP00000051952-D1	-3.42	1.61E-02	↑	Bubalus bubalis zinc finger protein 470-like (LOC102390690), transcript variant X11, mRNA
ENSP00000289957-D2	-3.42	1.61E-02	↑	Bubalus bubalis cholinergic receptor, nicotinic, beta 3 (neuronal) (CHRNB3), mRNA
ENSP00000334854-D1	-3.42	1.61E-02	↑	B. mutus zinc activated ligand-gated ion channel (ZACN), mRNA
ENSBTAP00000027968-D1	-3.42	1.61E-02	↑	B. mutus acid phosphatase 2, lysosomal (ACP2), transcript variant X1, mRNA
ENSBTAP00000005147-D1	-3.42	1.61E-02	↑	B. mutus chromosome unknown open reading frame, human C2orf62 (LOC102286249), mRNA
ENSBTAP00000037476-D1	-3.42	1.61E-02	↑	B. taurus neuronal growth regulator 1, mRNA (cDNA clone IMAGE:8299787)
ENSP00000402226-D1	-3.42	1.61E-02	↑	B. mutus relaxin/insulin-like family peptide receptor 1 (RXFP1), mRNA
yakG029468	-3.42	1.61E-02	↑	B. mutus potassium large conductance calcium-activated channel, subfamily M beta member 3 (KCNMB3), mRNA
ENSBTAP00000016984-D1	-3.42	1.61E-02	↑	B. mutus aquaporin 6, kidney specific (AQP6), mRNA
ENSBTAP00000046412-D1	-3.42	1.61E-02	↑	B. mutus collagen, type IX, alpha 1 (COL9A1), transcript variant X2, mRNA
ENSBTAP00000003421-D1	-3.42	1.61E-02	↑	B. mutus potassium voltage-gated channel, KQT-like subfamily, member 4 (KCNQ4), mRNA
ENSBTAP00000038807-D3	-3.42	1.61E-02	↑	B. mutus GTPase IMAP family member 7-like (LOC102287693), mRNA
ENSBTAP00000035695-D2	-3.42	1.61E-02	↑	B. mutus glutathione S-transferase A2-like (LOC102287938), partial mRNA
ENSBTAP00000025880-D5	-3.42	1.61E-02	↑	B. mutus chemokine (C-C motif) receptor 2 (CCR2), mRNA
ENSP00000378804-D1	-3.42	1.61E-02	↑	Bubalus bubalis spermidine/spermine N1-acetyl transferase-like 1 (SATL1), mRNA
ENSBTAP00000031243-D3	-3.42	1.61E-02	↑	B. mutus serpin peptidase inhibitor, clade B (ovalbumin), member 1 (SERPINB1), mRNA
ENSBTAP00000053422-D1	-3.42	1.61E-02	↑	B. mutus collagen, type IX, alpha 1 (COL9A1), transcript variant X1, mRNA
ENSBTAP00000037299-D1	-3.42	1.61E-02	↑	B. mutus stannin (SNN), mRNA
ENSP00000361024-D1	-3.42	1.61E-02	↑	B. mutus diencephalon/mesencephalon homeobox 1 (DMBX1), transcript variant X2, mRNA
ENSBTAP00000006532-D3	-3.42	1.61E-02	↑	B. taurus actin, alpha, cardiac muscle 1 (ACTC1), mRNA
ENSP00000382178-D1	-3.42	1.61E-02	↑	B. mutus MAM domain containing glycosylphosphatidylinositol anchor 2 (MDGA2), mRNA
ENSBTAP00000015694-D1	-3.42	1.61E-02	↑	B. mutus osteoglycin (OGN), transcript variant X2, mRNA
ENSP00000384982-D20	-3.42	1.61E-02	↑	B. mutus calphotin-like (LOC102282031), mRNA
ENSBTAP00000011232-D1	-3.42	1.61E-02	↑	B. mutus putative protein FAM90A12P-like (LOC102273546), mRNA
ENSP00000290510-D1	-3.42	1.61E-02	↑	B. mutus leprecan-like 2 (LEPREL2), mRNA
ENSBTAP00000024433-D1	-3.42	1.61E-02	↑	B. mutus transmembrane protein 132A (TMEM132A), transcript variant X2, mRNA
ENSBTAP00000044687-D1	-3.42	1.61E-02	↑	Bubalus bubalis prostate and testis expressed 2 (PATE2), mRNA
ENSBTAP00000007573-D1	-3.42	1.61E-02	↑	B. mutus Ly6/neurotoxin 1 (LYNX1), mRNA
ENSP00000328625-D1	-3.42	1.61E-02	↑	B. mutus negative regulator of reactive oxygen species (NRROS), mRNA
yakA16470	-3.42	1.61E-02	↑	B. taurus RALY RNA binding protein-like (RALYL), mRNA
ENSBTAP00000014791-D1	-3.42	1.61E-02	↑	Bubalus bubalis nuclear receptor subfamily 2, group F, member 6 (NR2F6), mRNA
ENSBTAP00000014806-D1	-3.42	1.61E-02	↑	B. mutus single immunoglobulin and toll-interleukin 1 receptor (TIR) domain (SIGIRR), mRNA
ENSP00000365441-D1	-3.42	1.61E-02	↑	B. mutus calcium channel, voltage-dependent, L type, alpha 1F subunit (CACNA1F), transcript variant X1, mRNA
ENSBTAP00000023918-D4	-3.42	1.61E-02	↑	B. mutus histone H2B type W-T-like (LOC102270856), mRNA
ENSBTAP00000052872-D1	-3.42	1.61E-02	↑	Human mariner1 transposase gene, complete consensus sequence
ENSP00000262648-D1	-3.42	1.61E-02	↑	B. mutus Kallmann syndrome 1 sequence (KAL1), mRNA
ENSBTAP00000040465-D4	-3.42	1.61E-02	↑	B. mutus protein FAM32A-like (LOC102287772), partial mRNA
ENSBTAP00000018701-D1	-3.42	1.61E-02	↑	B. mutus inter-alpha-trypsin inhibitor heavy chain 2 (ITIH2), mRNA
ENSBTAP00000008409-D3	-3.42	1.61E-02	↑	Bubalus bubalis KATNB1-like protein 1-like (LOC102391934), mRNA
ENSBTAP00000050450-D1	-3.42	1.61E-02	↑	B. mutus transmembrane protease serine 11E-like (LOC102277099), mRNA
yakA07452	-3.42	1.61E-02	↑	B. mutus olfactory receptor 10AG1-like (LOC102269521), mRNA
ENSBTAP00000047428-D2	-3.42	1.61E-02	↑	B. mutus odorant-binding protein-like (LOC102280567), partial mRNA
ENSP00000382575-D1	-3.42	1.61E-02	↑	B. mutus FLYWCH-type zinc finger 1 (FLYWCH1), mRNA
ENSBTAP00000022557-D1	-3.42	1.61E-02	↑	B. mutus PHD finger protein 19 (PHF19), mRNA
ENSP00000362562-D1	-3.42	1.61E-02	↑	B. mutus transmembrane protein 54 (TMEM54), transcript variant X1, mRNA
ENSP00000280481-D1	-3.42	1.61E-02	↑	B. mutus FRAS1 related extracellular matrix protein 2 (FREM2), mRNA

ENSBTAP00000052264-D1	-3.42	1.61E-02	↑	B. mutus olfactory receptor 8I2-like (LOC102274296), mRNA
ENSBTAP00000022976-D1	-3.42	1.61E-02	↑	B. mutus DNA-damage regulated autophagy modulator 1 (DRAM1), mRNA
ENSBTAP00000026454-D1	-3.42	1.61E-02	↑	B. mutus EGF-like module-containing mucin-like hormone receptor-like 4-like (LOC102277268), mRNA
ENSBTAP00000005975-D1	-3.41	2.86E-201	↑	B. mutus oxidized low density lipoprotein (lectin-like) receptor 1 (OLR1), transcript variant X1, mRNA
ENSBTAP00000048807-D1	-3.39	1.13E-53	↑	B. mutus solute carrier family 25, member 3 (SLC25A3), transcript variant X3, mRNA
ENSP00000360170-D1	-3.38	2.20E-16	↑	B. taurus angiopoietin-like 3 (ANGPTL3), mRNA
ENSBTAP00000036905-D1	-3.37	4.49E-07	↑	Bubalus bubalis RIMS binding protein 2 (RIMBP2), transcript variant X4, mRNA
ENSP00000413218-D1	-3.34	9.99E-09	↑	B. mutus family with sequence similarity 213, member B (FAM213B), mRNA
ENSBTAP00000014872-D1	-3.34	3.68E-05	↑	B. mutus inositol polyphosphate multikinase (IPMK), mRNA
ENSBTAP00000024072-D1	-3.34	3.68E-05	↑	B. mutus quinolinate phosphoribosyltransferase (QPRT), mRNA
ENSBTAP00000042309-D1	-3.34	3.68E-05	↑	B. mutus gametogenetin (GGN), mRNA
ENSP00000342295-D1	-3.33	5.90E-14	↑	B. mutus ankyrin repeat domain 55 (ANKRD55), transcript variant X1, mRNA
ENSP00000369071-D1	-3.33	4.38E-72	↑	B. mutus periostin, osteoblast specific factor (POSTN), transcript variant X1, mRNA
ENSBTAP00000005167-D1	-3.33	2.21E-10	↑	B. mutus zinc finger protein 316 (ZNF316), mRNA
ENSBTAP00000000821-D1	-3.32	7.77E-21	↑	B. mutus catsper channel auxiliary subunit gamma (CATSPERG), mRNA
ENSBTAP00000014749-D1	-3.32	7.24E-40	↑	B. mutus solute carrier family 38, member 2 (SLC38A2), transcript variant X1, mRNA
ENSP00000389989-D1	-3.31	1.80E-08	↑	B. mutus jerky homolog-like (mouse) (JRKL), mRNA
ENSBTAP00000003629-D4	-3.31	1.80E-08	↑	B. mutus sulfotransferase family, cytosolic, 1A, phenol-preferring, member 1 (SULT1A1), mRNA
ENSBTAP00000043674-D1	-3.30	7.15E-29	↑	B. mutus glutathione S-transferase omega-1-like (LOC102288314), transcript variant X1, mRNA
ENSP00000349785-D1	-3.28	3.31E-50	↑	B. mutus microtubule-associated proteins 1A/1B light chain 3C-like (LOC102288385), mRNA
ENSP00000006275-D1	-3.27	1.46E-06	↑	B. mutus trafficking protein particle complex 6A (TRAPPC6A), mRNA
ENSP00000364087-D1	-3.27	6.62E-05	↑	B. mutus SLA class II histocompatibility antigen, DQ haplotype D alpha chain-like (LOC102269978), mRNA
ENSBTAP00000013751-D1	-3.27	6.62E-05	↑	B. mutus protocadherin 17 (PCDH17), mRNA
ENSBTAP00000024714-D1	-3.27	6.62E-05	↑	B. mutus short chain dehydrogenase/reductase family 16C, member 5 (SDR16C5), mRNA
ENSP00000245932-D1	-3.27	6.62E-05	↑	B. mutus vasodilator-stimulated phosphoprotein (VASP), mRNA
ENSP00000408891-D1	-3.27	3.10E-03	↑	B. mutus pyruvate kinase, liver and RBC (PKLR), mRNA
ENSP00000294725-D1	-3.27	3.10E-03	↑	B. mutus potassium channel, subfamily T, member 2 (KCNT2), transcript variant X1, mRNA
ENSP00000292140-D1	-3.27	3.10E-03	↑	Bubalus bubalis pleckstrin homology-like domain, family B, member 3 (PHLDB3), transcript variant X1, mRNA
ENSP00000293379-D1	-3.27	3.10E-03	↑	B. mutus integrin, alpha 5 (fibronectin receptor, alpha polypeptide) (ITGA5), partial mRNA
ENSP00000271610-D1	-3.27	3.10E-03	↑	B. mutus protein tyrosine phosphatase, receptor type, C (PTPRC), partial mRNA
ENSBTAP00000001191-D1	-3.27	3.10E-03	↑	B. mutus coagulation factor II (thrombin) receptor-like 2 (F2RL2), mRNA
ENSBTAP00000041561-D1	-3.27	3.10E-03	↑	B. mutus interferon induced transmembrane protein 5 (IFITM5), mRNA
ENSBTAP00000002409-D1	-3.27	3.10E-03	↑	B. taurus EPS8-like 3 (EPS8L3), mRNA
ENSP00000416142-D2	-3.27	3.10E-03	↑	B. mutus amylase, alpha 2B (pancreatic) (AMY2B), mRNA
ENSP00000233638-D1	-3.27	3.10E-03	↑	B. mutus T-cell leukemia homeobox 2 (TLX2), mRNA
ENSBTAP00000027975-D1	-3.27	3.10E-03	↑	B. mutus tripartite motif containing 55 (TRIM55), transcript variant X1, mRNA
ENSP00000263379-D1	-3.27	3.10E-03	↑	B. mutus interleukin 27 receptor, alpha (IL27RA), mRNA
ENSP00000356792-D2	-3.25	7.01E-41	↑	B. mutus lymphotactin-like (LOC102285258), misc_RNA
ENSBTAP00000018852-D2	-3.23	1.28E-09	↑	B. mutus solute carrier family 12 (potassium/chloride transporter), member 7 (SLC12A7), mRNA
ENSBTAP00000051259-D76	-3.23	1.28E-09	↑	B.taurus DNA sequence from clone CH240-487I9, complete sequence
ENSP00000324857-D1	-3.23	1.04E-20	↑	B. mutus sema domain, transmembrane domain, and cytoplasmic domain, SEMA6D, transcript variant X2, mRNA
ENSP00000319914-D1	-3.22	7.86E-27	↑	B. mutus chromosome unknown open reading frame, human C8orf4 (LOC102288196), mRNA
ENSBTAP00000017619-D1	-3.21	2.62E-06	↑	B. mutus ES cell expressed Ras (ERAS), mRNA
ENSP00000286614-D1	-3.20	1.05E-32	↑	B. mutus matrix metallopeptidase 16 (membrane-inserted) (MMP16), mRNA
ENSP00000348706-D4	-3.20	2.31E-09	↑	B. mutus histone H2B type 1-like (LOC102279439), mRNA
ENSBTAP00000052380-D1	-3.20	2.31E-09	↑	Pantholops hodgsonii PRAME family member 9/15-like (LOC102327627), mRNA
ENSP00000409797-D1	-3.20	2.01E-12	↑	Bubalus bubalis protein phosphatase 1, regulatory subunit 42 (PPP1R42), transcript variant X1, mRNA
ENSP00000314897-D1	-3.18	9.11E-11	↑	B. mutus angiopoietin 2 (ANGPT2), transcript variant X1, mRNA
ENSP00000330074-D1	-3.18	1.20E-04	↑	B. mutus histone H1.5-like (LOC102271377), mRNA
ENSBTAP00000000461-D1	-3.18	1.20E-04	↑	B. mutus oxidoreductase-like domain containing 1 (OXLD1), mRNA
ENSP00000318868-D1	-3.18	1.20E-04	↑	B. mutus serine hydroxymethyltransferase 1 (soluble) (SHMT1), transcript variant X1, mRNA
ENSP00000372221-D1	-3.15	1.33E-60	↑	B. mutus serpin peptidase inhibitor, clade B (ovalbumin), member 5 (SERPINB5), transcript variant X1, mRNA
ENSBTAP00000018283-D1	-3.14	1.67E-61	↑	B. mutus brain expressed X-linked 2 (BEX2), transcript variant X2, mRNA
ENSP00000379279-D1	-3.13	5.37E-22	↑	B. mutus DnaJ (Hsp40) homolog, subfamily C, member 24 (DNAJC24), mRNA
ENSP00000371735-D1	-3.12	9.59E-22	↑	B. mutus spastic ataxia of Charlevoix-Saguenay (sacsin) (SACS), mRNA

ENSBTAP00000026725-D1	-3.10	8.77E-40	↑	B. taurus collagen, type XII, alpha 1 (COL12A1), mRNA
ENSP00000378079-D1	-3.10	2.46E-42	↑	B. mutus zinc finger protein 644 (ZNF644), transcript variant X1, mRNA
ENSP00000364946-D1	-3.10	4.74E-24	↑	B. mutus mohawk homeobox (MKX), mRNA
ENSBTAP00000004617-D1	-3.10	1.25E-15	↑	B. mutus chromosome unknown open reading frame, human C11orf54 (LOC102275785), mRNA
ENSP00000384360-D1	-3.10	5.20E-10	↑	B. mutus ubiquitin specific peptidase 26 (USP26), mRNA
ENSP00000370673-D1	-3.10	3.30E-07	↑	B. mutus MANSC domain containing 4 (MANSC4), mRNA
ENSBTAP00000024193-D1	-3.10	3.30E-07	↑	B. mutus chromosome unknown open reading frame, human C12orf50, transcript variant X1, mRNA
ENSBTAP00000050148-D1	-3.10	8.40E-06	↑	B. taurus CG2446-like (LOC516108), mRNA
ENSBTAP00000052619-D23	-3.10	8.40E-06	↑	B. taurus chromosome 9 clone BAC RPCI42-522F04, complete sequence
ENSBTAP00000006939-D1	-3.10	8.40E-06	↑	B. mutus WEE1 G2 checkpoint kinase (WEE1), mRNA
ENSP00000302120-D1	-3.10	2.14E-04	↑	B. mutus tripartite motif containing 8 (TRIM8), mRNA
ENSBTAP00000012081-D1	-3.10	2.14E-04	↑	B. mutus lipin 3 (LPIN3), mRNA
ENSBTAP00000028795-D1	-3.10	2.14E-04	↑	B. mutus G protein-coupled receptor 110 (GPR110), mRNA
ENSP00000361598-D1	-3.10	2.14E-04	↑	B. taurus zinc finger, SWIM-type containing 1 (ZSWIM1), mRNA
ENSBTAP00000049946-D17	-3.10	2.14E-04	↑	B.taurus DNA sequence from clone CH240-487I9, complete sequence
ENSBTAP00000023834-D1	-3.10	2.14E-04	↑	B. mutus dolichyldiphosphatase 1 (DOLPP1), transcript variant X1, mRNA
ENSP00000411847-D1	-3.10	5.61E-03	↑	B. mutus BAH domain and coiled-coil containing 1 (BAHCC1), mRNA
ENSBTAP00000051357-D1	-3.10	5.61E-03	↑	B. mutus vomeronasal type-1 receptor 1-like (LOC102274643), mRNA
ENSBTAP00000005212-D1	-3.10	5.61E-03	↑	B. mutus inositol 1,4,5-trisphosphate receptor interacting protein (ITPRIP), mRNA
ENSBTAP00000010892-D1	-3.10	5.61E-03	↑	B. mutus hepatocyte nuclear factor 4, gamma (HNF4G), mRNA
ENSBTAP00000026853-D1	-3.10	5.61E-03	↑	B. mutus kelch-like family member 22 (KLHL22), mRNA
ENSP00000310260-D2	-3.10	5.61E-03	↑	B. mutus DEAD/H (Asp-Glu-Ala-Asp/His) box polypeptide 26B (DDX26B), transcript variant X1, mRNA
ENSP00000359019-D1	-3.10	5.61E-03	↑	B. mutus paired-like homeodomain 3 (PITX3), mRNA
ENSP00000261483-D2	-3.10	5.61E-03	↑	B. mutus mannosidase, alpha, class 2A, member 2 (MAN2A2), mRNA
ENSBTAP00000023209-D1	-3.10	5.61E-03	↑	B. mutus solute carrier family 16 (monocarboxylate transporter), member 3 (SLC16A3), mRNA
ENSBTAP00000006985-D1	-3.10	5.61E-03	↑	B. mutus G protein-coupled receptor 180 (GPR180), mRNA
ENSBTAP00000004098-D1	-3.10	5.61E-03	↑	B. mutus interferon, alpha-inducible protein 27-like 2 (IFI27L2), mRNA
ENSBTAP00000008115-D1	-3.10	5.61E-03	↑	B. mutus cAMP responsive element binding protein 3-like 3 (CREB3L3), mRNA
ENSBTAP00000040256-D1	-3.10	5.61E-03	↑	B. mutus ADP-ribosylation factor-like 16 (ARL16), mRNA
ENSP00000006777-D1	-3.07	9.25E-10	↑	B. mutus rhomboid domain containing 2 (RHBDD2), mRNA
ENSBTAP00000004389-D1	-3.06	2.33E-08	↑	B. mutus suppressor of Ty 4 homolog 1 (S. cerevisiae) (SUPT4H1), mRNA
ENSP00000252137-D1	-3.05	5.88E-07	↑	B. mutus DiGeorge syndrome critical region gene 14 (DGCR14), transcript variant X1, mRNA
ENSBTAP00000026449-D1	-3.05	5.88E-07	↑	B. mutus serine/threonine-protein phosphatase 2A 65 kDa regulatory subunit A alpha isoform-like, mRNA
ENSBTAP00000007366-D3	-3.04	7.77E-19	↑	B. mutus tripartite motif-containing protein 5-like (LOC102273840), mRNA
ENSBTAP00000049167-D1	-3.04	2.52E-29	↑	B. mutus succinate receptor 1-like (LOC102287115), mRNA
ENSBTAP00000025719-D1	-3.04	2.15E-21	↑	B. mutus phospholipase A2, group VII (platelet-activating factor acetylhydrolase, plasma) (PLA2G7), mRNA
ENSP00000306817-D1	-3.04	1.65E-09	↑	B. mutus ribokinase (RBKS), mRNA
ENSBTAP00000007857-D1	-3.04	1.50E-05	↑	B. mutus SFT2 domain containing 3 (SFT2D3), mRNA
ENSBTAP00000021521-D1	-3.04	1.50E-05	↑	B. mutus inhibitor of DNA binding 1, dominant negative helix-loop-helix protein (ID1), mRNA
ENSBTAP00000017169-D1	-3.00	8.12E-12	↑	B. mutus transmembrane protein 70 (TMEM70), mRNA
ENSP00000338171-D1	-3.00	2.93E-09	↑	B. mutus src kinase associated phosphoprotein 1 (SKAP1), mRNA
ENSP00000295597-D1	-3.00	3.82E-04	↑	Lipotes vexillifer plakophilin 1 (PKP1), transcript variant X1, mRNA
yakG002137	-3.00	3.82E-04	↑	B. mutus eukaryotic translation elongation factor 1 gamma (EEF1G), mRNA
ENSBTAP00000042518-D1	-3.00	3.82E-04	↑	Pantholops hodgsonii early B-cell factor 4 (EBF4), mRNA
ENSP00000324175-D1	-3.00	3.82E-04	↑	B. mutus MEF2 activating motif and SAP domain containing transcriptional regulator, transcript variant X1, mRNA
ENSBTAP00000006879-D1	-3.00	3.82E-04	↑	B. mutus uncharacterized LOC102270742 (LOC102270742), mRNA
ENSBTAP00000005802-D1	-2.99	1.50E-304	↑	B. mutus histone H2A.Z-like (LOC102269063), mRNA
ENSBTAP00000006167-D1	-2.99	2.05E-10	↑	B. mutus alpha-2-macroglobulin (A2M), mRNA
ENSBTAP00000005558-D3	-2.98	7.37E-08	↑	B. mutus lysophospholipase I (LYPLA1), transcript variant X1, mRNA
ENSBTAP00000005487-D1	-2.97	2.65E-05	↑	B. mutus jumonji domain containing 4 (JMJD4), mRNA
ENSBTAP00000007505-D1	-2.96	3.64E-10	↑	Bubalus bubalis fucose mutarotase (FUOM), mRNA
ENSP00000410403-D1	-2.96	3.64E-10	↑	B. mutus RAB34, member RAS oncogene family (RAB34), mRNA
ENSP00000365548-D1	-2.95	1.85E-06	↑	B. mutus procollagen-lysine, 2-oxoglutarate 5-dioxygenase 1 (PLOD1), mRNA
ENSP00000362095-D1	-2.95	1.85E-06	↑	B. mutus sushi-repeat containing protein, X-linked 2 (SRPX2), mRNA

ENSBTAP00000026765-D1	-2.94	1.30E-07	↑	B. mutus epoxide hydrolase 3 (EPHX3), mRNA
ENSBTAP00000024188-D1	-2.94	1.30E-07	↑	B. mutus chromosome unknown open reading frame, human C17orf62 (LOC102276036), mRNA
ENSBTAP00000028958-D1	-2.94	1.30E-07	↑	B. mutus pyruvate dehydrogenase (lipoamide) beta (PDHB), transcript variant X1, mRNA
ENSBTAP00000028806-D1	-2.93	4.51E-11	↑	B. mutus zinc finger CCCH-type, antiviral 1 (ZC3HAV1), mRNA
ENSP00000363799-D1	-2.92	2.21E-13	↑	B. mutus actin-like 7B (ACTL7B), mRNA
ENSP00000333181-D1	-2.92	2.21E-13	↑	B. mutus membrane-associated ring finger (C3HC4) 11 (MARCH11), partial mRNA
ENSP00000238994-D1	-2.90	1.32E-16	↑	B. mutus protein phosphatase 1, regulatory subunit 3C (PPP1R3C), mRNA
ENSP00000268711-D1	-2.90	4.67E-05	↑	B. mutus mediator complex subunit 9 (MED9), mRNA
ENSBTAP00000013354-D1	-2.90	4.67E-05	↑	B. mutus apolipoprotein E (APOE), mRNA
ENSP00000301727-D1	-2.90	6.76E-04	↑	B. mutus E4F transcription factor 1 (E4F1), mRNA
ENSBTAP00000009267-D1	-2.90	6.76E-04	↑	B. mutus frizzled family receptor 10 (FZD10), mRNA
ENSBTAP00000036833-D1	-2.90	6.76E-04	↑	B. mutus A disintegrin and metalloproteinase with thrombospondin motifs 20-like (LOC102281259), mRNA
yakG045259	-2.90	6.76E-04	↑	B. mutus uncharacterized LOC102278095 (LOC102278095), mRNA
ENSP00000361383-D1	-2.90	6.76E-04	↑	B. mutus allograft inflammatory factor 1-like (AIF1L), mRNA
ENSP00000411948-D1	-2.90	6.76E-04	↑	B. mutus suppressor APC domain containing 1 (SAPCD1), mRNA
ENSP00000039007-D1	-2.90	1.01E-02	↑	B. mutus ornithine carbamoyltransferase (OTC), mRNA
ENSP00000296130-D1	-2.90	1.01E-02	↑	B. mutus C-type lectin domain family 3, member B (CLEC3B), mRNA
ENSP00000384515-D2	-2.90	1.01E-02	↑	B. taurus parvin, beta (PARVB), mRNA
ENSP00000289746-D1	-2.90	1.01E-02	↑	B. mutus cadherin 15, type 1, M-cadherin (myotubule) (CDH15), mRNA
ENSP00000216840-D1	-2.90	1.01E-02	↑	B. mutus Rab geranylgeranyltransferase, alpha subunit (RABGGTA), mRNA
ENSBTAP00000023151-D1	-2.90	1.01E-02	↑	B. mutus adenylate kinase 5 (AK5), mRNA
ENSP00000352284-D1	-2.90	1.01E-02	↑	B. mutus transmembrane protein 139 (TMEM139), mRNA
ENSP00000258200-D1	-2.90	1.01E-02	↑	B. mutus F-box and leucine-rich repeat protein 8 (FBXL8), mRNA
ENSP00000358696-D1	-2.90	1.01E-02	↑	B. mutus plexin A3 (PLXNA3), mRNA
ENSP00000332788-D1	-2.90	1.01E-02	↑	B. mutus anoctamin 9 (ANO9), mRNA
ENSBTAP00000010598-D1	-2.90	1.01E-02	↑	B. mutus glutamate decarboxylase 2 (pancreatic islets and brain, 65kDa) (GAD2), mRNA
ENSP00000353654-D1	-2.90	1.01E-02	↑	B. mutus collagen, type IV, alpha 2 (COL4A2), partial mRNA
ENSP00000364515-D1	-2.90	1.01E-02	↑	B. mutus BTB (POZ) domain containing 17 (BTBD17), mRNA
ENSBTAP00000019348-D1	-2.90	1.01E-02	↑	Bubalus bubalis Cas scaffolding protein family member 4 (CASS4), transcript variant X2, mRNA
ENSBTAP00000032695-D1	-2.90	1.01E-02	↑	Bubalus bubalis uncharacterized LOC102392385 (LOC102392385), mRNA
ENSP00000341289-D9	-2.90	1.01E-02	↑	B. mutus tubulin beta-2B chain-like (LOC102279506), misc_RNA
ENSBTAP00000002838-D1	-2.90	1.01E-02	↑	B. mutus chromosome unknown open reading frame, human C8orf46 (LOC102272650), mRNA
ENSP00000332601-D1	-2.88	6.83E-13	↑	B. mutus transcription initiation factor TFIID subunit 11-like (LOC102273510), mRNA
ENSBTAP00000018541-D1	-2.87	2.84E-08	↑	B. mutus UDP-N-acetyl-alpha-D-galactosamine:polypeptide N-acetylgalactosaminyltransferase 3, mRNA
ENSP00000367359-D1	-2.87	8.40E-14	↑	Bubalus bubalis chromosome unknown open reading frame, human C6orf62, transcript variant X1, mRNA
ENSBTAP00000027051-D1	-2.86	4.03E-07	↑	B. mutus PQ loop repeat containing 3 (PQLC3), transcript variant X1, mRNA
ENSBTAP00000050903-D1	-2.85	3.07E-20	↑	B. mutus Rho family GTPase 3 (RND3), transcript variant X2, mRNA
ENSBTAP00000052237-D1	-2.85	4.55E-52	↑	B. mutus biliverdin reductase A (BLVRA), mRNA
ENSP00000325285-D1	-2.85	5.78E-06	↑	B. mutus myotubularin related protein 4 (MTMR4), mRNA
ENSBTAP00000033750-D1	-2.85	5.78E-06	↑	B. mutus anterior gradient 2 (AGR2), transcript variant X2, mRNA
ENSBTAP00000007589-D1	-2.85	1.48E-13	↑	B. mutus phosphoglucomutase 2 (PGM2), mRNA
ENSBTAP00000029075-D1	-2.84	8.31E-43	↑	B. mutus alpha-methylacyl-CoA racemase (AMACR), mRNA
ENSBTAP00000025337-D1	-2.84	7.72E-19	↑	B. mutus Rho GTPase activating protein 28 (ARHGAP28), mRNA
ENSP00000295454-D2	-2.83	5.00E-08	↑	B. mutus gamma-aminobutyric acid (GABA) A receptor, beta 1 (GABRB1), transcript variant X1, mRNA
ENSBTAP00000048591-D2	-2.83	8.24E-05	↑	B. mutus zinc finger and SCAN domain-containing protein 5B-like (LOC102287491), mRNA
ENSBTAP00000028498-D1	-2.83	8.24E-05	↑	B. mutus S100 calcium binding protein A14 (S100A14), mRNA
ENSBTAP00000010554-D1	-2.83	8.24E-05	↑	B. mutus oxytocin/neurophysin I prepropeptide (OXT), mRNA
ENSBTAP00000020664-D1	-2.82	7.09E-07	↑	B. mutus deleted in liver cancer 1 (DLC1), transcript variant X2, mRNA
ENSP00000416247-D1	-2.81	6.15E-09	↑	B. mutus desmocollin 2 (DSC2), mRNA
ENSP00000357431-D1	-2.81	3.91E-15	↑	Pantholops hodgsonii protein kinase (cAMP-dependent, catalytic) inhibitor beta, transcript variant X2, mRNA
ENSP00000232219-D1	-2.80	1.22E-78	↑	B. mutus retinol binding protein 1, cellular (RBP1), mRNA
ENSP00000002596-D1	-2.80	1.02E-05	↑	B. mutus heparan sulfate (glucosamine) 3-O-sulfotransferase 1 (HS3ST1), mRNA
ENSP00000342576-D1	-2.80	1.02E-05	↑	B. mutus family with sequence similarity 221, member A (FAM221A), transcript variant X1, mRNA
ENSBTAP00000003797-D1	-2.80	1.20E-03	↑	B. mutus chemokine (C-X3-C motif) receptor 1 (CX3CR1), mRNA
ENSBTAP00000043756-D2	-2.80	1.20E-03	↑	B. mutus metallothionein 1B (MT1B), mRNA

ENSBTAP00000011966-D2	-2.80	1.20E-03	↑	B. mutus beta-adducin-like (LOC102266026), partial mRNA
ENSP00000333262-D1	-2.80	1.20E-03	↑	B. mutus inositol polyphosphate-5-phosphatase J (INPP5J), transcript variant X1, mRNA
ENSP00000330381-D1	-2.78	9.32E-11	↑	B. mutus WD repeat domain 5B (WDR5B), mRNA
ENSBTAP00000012118-D1	-2.78	7.27E-18	↑	B. mutus ankyrin repeat and SOCS box containing 9 (ASB9), transcript variant X1, mRNA
ENSP00000390147-D2	-2.78	8.39E-16	↑	B. mutus ribosomal protein L10a (RPL10A), mRNA
ENSP00000409597-D73	-2.78	1.08E-08	↑	B. mutus zinc finger protein 12 (ZNF12), transcript variant X1, mRNA
ENSP00000362111-D1	-2.78	8.25E-185	↑	B. mutus tetraspanin 6 (TSPAN6), transcript variant X1, mRNA
ENSP00000342235-D1	-2.77	1.24E-06	↑	Capra hircus v-erb-b2 avian erythroblastic leukemia viral oncogene homolog 4, transcript variant X3, mRNA
ENSP00000369014-D1	-2.77	1.32E-09	↑	B. mutus neuron-derived neurotrophic factor (NDNF), mRNA
ENSP00000337513-D1	-2.76	1.72E-13	↑	B. mutus steroid receptor RNA activator 1 (SRA1), mRNA
ENSP00000279146-D1	-2.76	1.53E-07	↑	B. mutus aryl hydrocarbon receptor interacting protein (AIP), mRNA
ENSBTAP00000040922-D1	-2.76	1.53E-07	↑	B. mutus coiled-coil domain containing 114 (CCDC114), mRNA
ENSP00000264187-D1	-2.76	1.53E-07	↑	B. mutus nidogen 1 (NID1), mRNA
ENSP00000359454-D1	-2.76	1.45E-04	↑	B. mutus HFM1, ATP-dependent DNA helicase homolog (S. cerevisiae) (HFM1), partial mRNA
ENSBTAP00000001147-D1	-2.75	3.72E-25	↑	B. mutus solute carrier family 38, member 1 (SLC38A1), transcript variant X1, mRNA
ENSBTAP00000017420-D1	-2.74	2.32E-09	↑	B. taurus collagen, type I, alpha 1 (COL1A1), mRNA
ENSBTAP00000022108-D1	-2.73	2.84E-10	↑	B. mutus dual serine/threonine and tyrosine protein kinase (DSTYK), mRNA
ENSBTAP00000008621-D1	-2.73	4.57E-101	↑	B. mutus proteasome (prosome, macropain) subunit, alpha type, 1 (PSMA1), transcript variant X1, mRNA
ENSBTAP00000020514-D1	-2.73	6.74E-17	↑	B. mutus N-terminal Xaa-Pro-Lys N-methyltransferase 1 (NTMT1), mRNA
ENSBTAP00000021305-D1	-2.72	1.02E-18	↑	B. mutus protein phosphatase 3, catalytic subunit, alpha isozyme (PPP3CA), transcript variant X1, mRNA
ENSP00000232975-D1	-2.72	2.66E-07	↑	B. mutus troponin C type 1 (slow) (TNNC1), mRNA
ENSP00000390279-D4	-2.72	1.25E-19	↑	B. mutus ribosomal protein L38 (RPL38), mRNA
ENSP00000377195-D1	-2.72	3.27E-08	↑	B. taurus drebrin 1 (DBN1), transcript variant X1, mRNA
ENSP00000334105-D1	-2.71	4.60E-55	↑	B. taurus phospholipase C, beta 4 (PLCB4), mRNA
ENSBTAP00000026534-D1	-2.69	3.46E-53	↑	B. taurus gelsolin (GSN), transcript variant 1, mRNA
ENSP00000338283-D1	-2.68	3.08E-05	↑	B. mutus neuronal PAS domain protein 2 (NPAS2), mRNA
ENSBTAP00000038143-D1	-2.68	3.08E-05	↑	B. mutus aldehyde oxidase 1 (AOX1), mRNA
ENSBTAP00000011438-D1	-2.68	2.53E-04	↑	B. mutus serine/arginine repetitive matrix 4 (SRRM4), partial mRNA
ENSBTAP00000032295-D1	-2.68	2.53E-04	↑	B. taurus popeye domain containing 2 (POPDC2), mRNA
ENSP00000384667-D1	-2.68	2.53E-04	↑	B. mutus chromosome unknown open reading frame, human C22orf23, transcript variant X1, mRNA
ENSBTAP00000009858-D1	-2.68	2.10E-03	↑	B. mutus ADP-ribosylation factor related protein 1 (ARFRP1), transcript variant X1, mRNA
ENSP00000393725-D1	-2.68	2.10E-03	↑	B. mutus GDNF family receptor alpha 1 (GFRA1), transcript variant X2, mRNA
ENSP00000355058-D1	-2.68	2.10E-03	↑	B. mutus mature T-cell proliferation 1 (MTCP1), mRNA
ENSP00000365318-D1	-2.68	2.10E-03	↑	B. mutus NFKBIL1, transcript variant X1, mRNA
ENSBTAP00000052592-D8	-2.68	2.10E-03	↑	B.taurus DNA sequence from clone CH240-487I9, complete sequence
ENSBTAP00000035294-D1	-2.68	2.10E-03	↑	B. mutus pre T-cell antigen receptor alpha (PTCRA), mRNA
ENSBTAP00000028542-D2	-2.68	2.10E-03	↑	B. mutus C1GALT1-specific chaperone 1-like (LOC102277714), mRNA
ENSP00000341528-D90	-2.68	1.79E-02	↑	Bubalus bubalis zinc finger protein 485-like (LOC102398414), transcript variant X4, mRNA
ENSBTAP00000020782-D1	-2.68	1.79E-02	↑	B. mutus transmembrane protein 156 (TMEM156), mRNA
ENSBTAP00000010773-D1	-2.68	1.79E-02	↑	B. taurus sulfatase modifying factor 2 (SUMF2), mRNA
ENSBTAP00000052766-D1	-2.68	1.79E-02	↑	B. mutus solute carrier family 25, member 42 (SLC25A42), mRNA
ENSBTAP00000012316-D2	-2.68	1.79E-02	↑	Balaenoptera acutorostrata scammoni metastasis suppressor 1-like (MTSS1L), partial mRNA
ENSBTAP00000027077-D1	-2.68	1.79E-02	↑	B. taurus dysbindin (dystrobrevin binding protein 1) domain containing 1 (DBNDD1), mRNA
ENSP00000410789-D1	-2.68	1.79E-02	↑	B. mutus EPH receptor B6 (EPHB6), mRNA
ENSP00000326424-D1	-2.68	1.79E-02	↑	B. mutus NAD synthetase 1 (NADSYN1), mRNA
ENSBTAP00000028368-D1	-2.68	1.79E-02	↑	B. mutus syntaxin-binding protein 6-like (LOC102273284), partial mRNA
ENSBTAP00000001727-D1	-2.68	1.79E-02	↑	B. mutus myeloid-associated differentiation marker-like (LOC102278806), mRNA
ENSP00000262430-D1	-2.68	1.79E-02	↑	B. mutus malonyl-CoA decarboxylase (MLYCD), mRNA
ENSP00000328274-D43	-2.68	1.79E-02	↑	B. mutus melanoma-associated antigen B1-like (LOC102274163), mRNA
ENSBTAP00000040441-D1	-2.68	1.79E-02	↑	B. taurus premature RNA for bovine serum amine oxidase
ENSP00000269601-D1	-2.68	1.79E-02	↑	B. mutus thioredoxin-like 4A (TXNL4A), mRNA
ENSP00000254325-D1	-2.68	1.79E-02	↑	B. mutus regulatory factor X, 1 (influences HLA class II expression) (RFX1), mRNA
ENSBTAP00000025079-D1	-2.68	1.79E-02	↑	B. mutus IQ and AAA domain-containing protein 1-like (LOC102266334), mRNA
ENSP00000360158-D1	-2.68	1.79E-02	↑	Pantholops hodgsonii polycystic kidney and hepatic disease 1 (autosomal recessive) (PKHD1), mRNA
ENSP00000322339-D1	-2.68	1.79E-02	↑	Bubalus bubalis vomeronasal type-1 receptor 1-like (LOC102408695), mRNA

ENSP00000352608-D3	-2.68	1.79E-02	↑	B. mutus ryanodine receptor 1-like (LOC102275567), partial mRNA
ENSBTAP00000014794-D1	-2.67	6.10E-16	↑	B. mutus butyrylcholinesterase (BCHE), mRNA
ENSBTAP00000052592-D54	-2.65	9.87E-08	↑	B. taurus Y Chr BAC CH240-278G19 complete sequence
ENSBTAP00000002289-D1	-2.65	9.87E-08	↑	B. mutus Bet1 golgi vesicular membrane trafficking protein-like (BET1L), mRNA
ENSBTAP00000005525-D1	-2.64	8.00E-07	↑	B. mutus mitogen-activated protein kinase kinase kinase 2 (MAP3K2), mRNA
ENSBTAP00000013674-D1	-2.64	3.88E-11	↑	B. mutus ATPase, Cu++ transporting, beta polypeptide (ATP7B), mRNA
ENSP00000360519-D1	-2.63	2.58E-09	↑	B. mutus retinol binding protein 4, plasma (RBP4), mRNA
ENSBTAP00000017759-D1	-2.62	5.32E-05	↑	Bubalus bubalis dystrophia myotonica-protein kinase (DMPK), transcript variant X1, mRNA
ENSP00000347088-D1	-2.62	5.32E-05	↑	B. mutus like-glycosyltransferase (LARGE), transcript variant X2, mRNA
ENSP00000350332-D1	-2.61	1.70E-07	↑	B. mutus myosin binding protein C, fast type (MYBPC2), mRNA
ENSP00000381282-D1	-2.60	5.60E-30	↑	B. mutus VCP-interacting membrane protein (VIMP), mRNA
ENSBTAP00000006382-D1	-2.60	4.46E-09	↑	B. mutus solute carrier family 24 (sodium/potassium/calcium exchanger), member 5 (SLC24A5), mRNA
ENSBTAP00000041294-D2	-2.60	4.38E-04	↑	B. mutus fatty acid desaturase 2-like protein FADS2P1-like (LOC102287215), partial mRNA
ENSP00000035307-D1	-2.60	4.38E-04	↑	B. mutus chondroitin polymerizing factor 2 (CHPF2), mRNA
ENSP00000364298-D1	-2.60	4.38E-04	↑	B.taurus DNA sequence from clone CH240-388C17, complete sequence
ENSP00000407950-D1	-2.59	2.49E-21	↑	B. mutus drebrin-like (DBNL), transcript variant X1, mRNA
ENSP00000263915-D1	-2.58	2.43E-16	↑	B. taurus growth factor receptor-bound protein 14 (GRB14), mRNA
ENSP00000392204-D1	-2.58	1.13E-05	↑	B. mutus killin, p53-regulated DNA replication inhibitor (KLLN), mRNA
yakG040936	-2.56	4.92E-19	↑	B. mutus ribosomal protein, large, P2 (RPLP2), mRNA
ENSBTAP00000003779-D1	-2.56	3.66E-03	↑	B. mutus enoyl-CoA hydratase domain-containing protein 2, mitochondrial-like (LOC102280117), mRNA
ENSP00000392188-D32	-2.56	3.66E-03	↑	B. taurus Y Chr BAC CH240-82K5 complete sequence
ENSBTAP00000024852-D1	-2.56	3.66E-03	↑	B. mutus tripartite motif containing 11 (TRIM11), mRNA
ENSBTAP00000004648-D1	-2.56	3.66E-03	↑	B. mutus coagulation factor XI (F11), mRNA
ENSP00000369833-D1	-2.56	3.66E-03	↑	B. mutus spermidine/spermine N1-acetyltransferase family member 2 (SAT2), mRNA
ENSP00000383760-D1	-2.56	3.66E-03	↑	B. mutus quinone oxidoreductase-like protein 2-like (LOC102276601), mRNA
ENSBTAP00000011390-D1	-2.56	3.66E-03	↑	B. taurus phosphodiesterase 4B, cAMP-specific (PDE4B), mRNA
ENSP00000390428-D2	-2.56	3.66E-03	↑	B. mutus creatine kinase, mitochondrial 2 (sarcomeric) (CKMT2), transcript variant X2, mRNA
ENSBTAP00000016228-D1	-2.56	3.66E-03	↑	B. mutus cyclin-dependent kinase 10 (CDK10), transcript variant X1, mRNA
ENSBTAP00000050194-D3	-2.56	3.66E-03	↑	B.taurus DNA sequence from clone CH240-487I9, complete sequence
ENSBTAP00000025923-D1	-2.56	3.66E-03	↑	B. mutus monooxygenase, DBH-like 1 (MOXD1), mRNA
ENSP00000362285-D1	-2.56	3.66E-03	↑	B. mutus solute carrier family 29 (equilibrative nucleoside transporter), member 3 (SLC29A3), mRNA
ENSP00000246190-D1	-2.56	3.66E-03	↑	Ceratotherium simum simum N-terminal EF-hand calcium binding protein 3 (NECAB3), mRNA
ENSP00000256495-D1	-2.54	3.23E-17	↑	B. mutus basic helix-loop-helix family, member e40 (BHLHE40), mRNA
ENSBTAP00000007079-D1	-2.54	6.84E-18	↑	B. mutus zinc finger, CCHC domain containing 10 (ZCCHC10), mRNA
ENSP00000367185-D1	-2.53	1.94E-05	↑	B. mutus LY6/PLAUR domain containing 5 (LYPD5), mRNA
ENSBTAP00000045968-D9	-2.53	1.94E-05	↑	B. mutus uncharacterized LOC102273059 (LOC102273059), mRNA
ENSP00000340083-D1	-2.53	1.94E-05	↑	B. mutus lysine-rich coiled-coil 1 (KRCC1), mRNA
ENSBTAP00000005354-D1	-2.52	1.07E-07	↑	B. mutus chromosome unknown open reading frame, human C6orf211 (LOC102285229), mRNA
ENSP00000350961-D1	-2.52	5.84E-10	↑	Bubalus bubalis uncharacterized LOC102410095 (LOC102410095), mRNA
ENSBTAP00000014053-D1	-2.51	4.60E-28	↑	B. mutus dynactin 2 (p50) (DCTN2), transcript variant X3, mRNA
ENSBTAP00000010182-D4	-2.51	1.23E-10	↑	B. mutus multidrug resistance-associated protein 4-like (LOC102286098), partial mRNA
ENSP00000321455-D1	-2.51	4.08E-06	↑	B. mutus mitochondrial calcium uptake family, member 3 (MICU3), mRNA
ENSP00000261058-D1	-2.51	7.56E-04	↑	B. mutus coiled-coil domain containing 54 (CCDC54), mRNA
ENSBTAP00000002092-D2	-2.51	7.56E-04	↑	B. mutus metallothionein-1A-like (LOC102278728), mRNA
ENSBTAP00000051671-D12	-2.49	1.58E-04	↑	B.taurus DNA sequence from clone CH240-487I9, complete sequence
ENSBTAP00000026669-D1	-2.49	7.80E-23	↑	B. mutus 5,10-methenyltetrahydrofolate synthetase (5-formyltetrahydrofolate cyclo-ligase) (MTHFS), mRNA
ENSP00000254667-D1	-2.48	4.44E-11	↑	B. mutus protein tyrosine phosphatase, receptor type, E (PTPRE), mRNA
ENSBTAP00000020989-D79	-2.48	2.61E-18	↑	Bubalus bubalis zinc finger protein 208-like (LOC102396279), partial mRNA
ENSBTAP00000011212-D1	-2.47	3.31E-05	↑	B. mutus sphingosine kinase 1 (SPHK1), mRNA
ENSP00000354855-D1	-2.47	2.42E-20	↑	B. mutus nuclear factor, erythroid 2-like 1 (NFE2L1), transcript variant X1, mRNA
ENSBTAP00000031070-D1	-2.47	1.70E-09	↑	B. mutus CNDP dipeptidase 2 (metallopeptidase M20 family) (CNDP2), transcript variant X1, mRNA
ENSP00000276410-D1	-2.47	6.97E-06	↑	B. mutus cholinergic receptor, nicotinic, alpha 6 (neuronal) (CHRNA6), mRNA
ENSBTAP00000017066-D1	-2.46	3.50E-60	↑	B. mutus stromal cell-derived factor 2 (SDF2), mRNA
ENSP00000381055-D1	-2.46	7.57E-11	↑	B. mutus dermatan sulfate epimerase-like (DSEL), mRNA
ENSP00000268129-D1	-2.46	3.34E-12	↑	B. mutus abhydrolase domain containing 2 (ABHD2), mRNA

ENSBTAP00000011884-D1	-2.45	3.08E-07	↑	B. mutus phosphofurin acidic cluster sorting protein 1 (PACS1), mRNA
ENSP00000275603-D3	-2.45	1.19E-38	↑	B. mutus chaperonin containing TCP1, subunit 6A (zeta 1) (CCT6A), mRNA
ENSP00000268053-D1	-2.44	6.08E-17	↑	B. mutus cholesterol side-chain cleavage enzyme, mitochondrial-like (LOC102282116), mRNA
ENSP00000318697-D2	-2.44	1.11E-21	↑	B. mutus tubulin, beta 6 class V (TUBB6), transcript variant X1, mRNA
ENSBTAP00000022702-D1	-2.43	1.03E-16	↑	B. mutus zinc finger protein 384 (ZNF384), transcript variant X1, mRNA
ENSP00000332287-D1	-2.42	1.03E-09	↑	B. mutus synaptogyrin 1 (SYNGR1), partial mRNA
ENSP00000304308-D1	-2.42	1.03E-09	↑	Bubalus bubalis zinc finger protein 217 (ZNF217), mRNA
ENSP00000292476-D1	-2.42	5.62E-05	↑	B. mutus cleavage and polyadenylation specific factor 4, 30kDa (CPSF4), transcript variant X1, mRNA
ENSBTAP00000027193-D1	-2.42	5.62E-05	↑	B. mutus formimidoyltransferase cyclodeaminase (FTCD), mRNA
ENSBTAP00000010762-D1	-2.42	2.69E-04	↑	B. taurus FBJ murine osteosarcoma viral oncogene homolog B (FOSB), mRNA
ENSBTAP00000027515-D1	-2.42	2.69E-04	↑	B. mutus RAS-like, family 11, member B (RASL11B), mRNA
ENSBTAP00000005250-D1	-2.42	2.69E-04	↑	B. mutus premelanosome protein (PMEL), transcript variant X2, mRNA
ENSBTAP00000042303-D1	-2.42	1.29E-03	↑	B. mutus RAS guanyl releasing protein 4 (RASGRP4), mRNA
ENSP00000371587-D1	-2.42	1.29E-03	↑	B. mutus Kv channel interacting protein 4 (KCNIP4), transcript variant X1, mRNA
ENSBTAP00000035394-D1	-2.42	1.29E-03	↑	B. mutus chorionic somatomammotropin hormone 1-like (LOC102274562), transcript variant X1, mRNA
ENSBTAP00000018383-D1	-2.42	1.29E-03	↑	B. mutus activin A receptor type II-like 1 (ACVRL1), mRNA
ENSBTAP00000019527-D1	-2.42	1.29E-03	↑	B. mutus poly(A) binding protein interacting protein 2B (PAIP2B), mRNA
ENSBTAP00000006089-D2	-2.42	6.32E-03	↑	B. mutus hyaluronidase PH-20-like (LOC102275634), partial mRNA
ENSP00000377545-D1	-2.42	6.32E-03	↑	B. mutus C1q and tumor necrosis factor related protein 2 (C1QTNF2), mRNA
ENSBTAP00000043151-D1	-2.42	6.32E-03	↑	B. mutus coiled-coil domain containing 159 (CCDC159), mRNA
ENSBTAP00000005969-D1	-2.42	6.32E-03	↑	B. taurus family with sequence similarity 78, member A (FAM78A), mRNA
ENSBTAP00000022785-D1	-2.42	6.32E-03	↑	B. mutus somatostatin receptor 2 (SSTR2), mRNA
yakG007721	-2.42	6.32E-03	↑	Bubalus bubalis cyclin-dependent kinase inhibitor 3 (CDKN3), transcript variant X2, mRNA
ENSBTAP00000027924-D1	-2.42	6.32E-03	↑	B. mutus ALX homeobox 3 (ALX3), mRNA
ENSBTAP00000030308-D1	-2.42	6.32E-03	↑	B. mutus RAB33A, member RAS oncogene family (RAB33A), mRNA
ENSBTAP00000053391-D1	-2.42	3.20E-02	↑	B. mutus potassium voltage-gated channel, Shaw-related subfamily, member 2 (KCNC2), partial mRNA
ENSBTAP00000031467-D2	-2.42	3.20E-02	↑	Bubalus bubalis cardiotrophin-2-like (LOC102402005), mRNA
ENSP00000332123-D1	-2.42	3.20E-02	↑	B. taurus ProSAPiP1 protein (PROSAPIP1), mRNA
ENSBTAP00000026100-D1	-2.42	3.20E-02	↑	B. mutus boLa class II histocompatibility antigen, DQB*0101 beta chain-like (LOC102277803), mRNA
ENSP00000291890-D7	-2.42	3.20E-02	↑	B. mutus glycoprotein VI (platelet) (GP6), mRNA
ENSBTAP00000019225-D1	-2.42	3.20E-02	↑	B. mutus family with sequence similarity 83, member H (FAM83H), mRNA
ENSBTAP00000018632-D1	-2.42	3.20E-02	↑	B. mutus IKAROS family zinc finger 1 (Ikaros) (IKZF1), transcript variant X2, mRNA
ENSBTAP00000005808-D1	-2.42	3.20E-02	↑	B. mutus sphingosine-1-phosphate phosphatase 1 (SGPP1), mRNA
ENSBTAP00000004371-D1	-2.42	3.20E-02	↑	B. mutus L-3-hydroxyproline dehydratase (trans-) (L3HYPDH), mRNA
ENSP00000295981-D1	-2.42	3.20E-02	↑	B. taurus interleukin 17 receptor C (IL17RC), transcript variant X1, mRNA
ENSBTAP00000006418-D1	-2.42	3.20E-02	↑	B. mutus forkhead box O4 (FOXO4), transcript variant X1, mRNA
ENSP00000335306-D1	-2.42	3.20E-02	↑	B. mutus developmental pluripotency-associated protein 4-like (LOC102264686), mRNA
ENSBTAP00000026467-D1	-2.42	3.20E-02	↑	B. mutus mitogen-activated protein kinase 15 (MAPK15), mRNA
ENSBTAP00000008354-D1	-2.42	3.20E-02	↑	B. mutus EPH receptor A1 (EPHA1), mRNA
ENSP00000361382-D1	-2.42	3.20E-02	↑	B. mutus pulmonary surfactant-associated protein A-like (LOC102285978), transcript variant X1, mRNA
ENSBTAP00000020767-D1	-2.42	3.20E-02	↑	B. mutus interleukin 20 receptor, alpha (IL20RA), transcript variant X1, mRNA
ENSP00000359096-D1	-2.42	3.20E-02	↑	B. mutus alpha-amylase 2B-like (LOC102275141), mRNA
ENSP00000327234-D1	-2.42	3.20E-02	↑	Orcinus orca transmembrane and coiled-coil domains 5A (TMCO5A), mRNA
ENSP00000416869-D1	-2.42	3.20E-02	↑	B. mutus pantothenate kinase 4 (PANK4), mRNA
yakA06652	-2.42	3.20E-02	↑	B. mutus 60S ribosomal protein L23a-like (LOC102285651), mRNA
ENSP00000282541-D1	-2.42	3.20E-02	↑	B. mutus glycerol-3-phosphate dehydrogenase 1-like protein-like (LOC102269290), mRNA
ENSP00000393299-D1	-2.42	3.20E-02	↑	B. mutus Src homology 2 domain containing F (SHF), transcript variant X3, mRNA
ENSP00000309794-D1	-2.42	3.20E-02	↑	B. mutus TBC1 domain family, member 16 (TBC1D16), mRNA
ENSBTAP00000002361-D1	-2.42	3.20E-02	↑	B. mutus twisted gastrulation BMP signaling modulator 1 (TWSG1), mRNA
ENSP00000341342-D1	-2.42	3.20E-02	↑	B. mutus kelch-like family member 6 (KLHL6), mRNA
ENSBTAP00000001704-D1	-2.42	3.20E-02	↑	B. mutus lactotransferrin (LTF), mRNA
ENSP00000355330-D1	-2.42	3.20E-02	↑	B. mutus transglutaminase 2 (TGM2), transcript variant X1, mRNA
ENSBTAP00000052592-D100	-2.42	3.20E-02	↑	B.taurus DNA sequence from clone CH240-487I9, complete sequence
ENSBTAP00000053555-D1	-2.42	3.20E-02	↑	B. mutus v-maf avian musculoaponeurotic fibrosarcoma oncogene homolog (MAF), mRNA
ENSP00000405527-D1	-2.42	3.20E-02	↑	B. taurus nuclear casein kinase and cyclin-dependent kinase substrate 1 (NUCKS1), transcript variant X1, mRNA

ENSP00000273067-D1	-2.42	3.20E-02	↑	B. mutus membrane-associated ring finger (C3HC4) 4, E3 ubiquitin protein ligase (MARCH4), mRNA
ENSBTAP00000024243-D1	-2.42	3.20E-02	↑	B. mutus shisa family member 2 (SHISA2), mRNA
ENSBTAP00000008890-D1	-2.42	3.20E-02	↑	B. mutus carbonic anhydrase XI (CA11), mRNA
ENSP00000395249-D1	-2.42	3.20E-02	↑	B. mutus family with sequence similarity 134, member A (FAM134A), mRNA
yakA02780	-2.42	3.20E-02	↑	B. mutus lipoma HMGIC fusion partner (LHFP), mRNA
ENSBTAP00000015657-D11	-2.42	3.20E-02	↑	B. taurus synaptotagmin XVII (SYT17), mRNA
ENSBTAP00000002315-D1	-2.40	7.78E-11	↑	B. mutus xylosylprotein beta 1,4-galactosyltransferase, polypeptide 7 (B4GALT7), mRNA
ENSBTAP00000053577-D1	-2.40	2.96E-37	↑	Bubalus bubalis protein kinase, AMP-activated, gamma 2 non-catalytic subunit, transcript variant X1, mRNA
ENSP00000407978-D3	-2.39	1.28E-45	↑	B. mutus homeobox D4 (HOXD4), mRNA
ENSBTAP00000009718-D1	-2.39	8.31E-09	↑	B. mutus uterine milk protein-like (LOC102281257), mRNA
ENSP00000378174-D1	-2.39	5.38E-14	↑	Bubalus bubalis signal peptide, CUB domain, EGF-like 3 (SCUBE3), transcript variant X1, mRNA
ENSP00000357681-D1	-2.39	0.00E+00	↑	B. mutus fibronectin type III and ankyrin repeat domains 1 (FANK1), mRNA
ENSBTAP00000025186-D1	-2.38	7.89E-18	↑	B. mutus liver expressed antimicrobial peptide 2 (LEAP2), mRNA
ENSBTAP00000012258-D1	-2.38	8.85E-07	↑	B. mutus MORN repeat containing 1 (MORN1), mRNA
ENSP00000297268-D1	-2.38	3.89E-50	↑	B. mutus collagen, type I, alpha 2 (COL1A2), mRNA
ENSBTAP00000032310-D1	-2.38	2.21E-38	↑	B. mutus protein S (alpha) (PROS1), mRNA
ENSP00000301819-D1	-2.38	1.31E-10	↑	Pantholops hodgsonii tetratricopeptide repeat domain 21A (TTC21A), transcript variant X1, mRNA
ENSP00000355649-D1	-2.38	4.21E-06	↑	B. mutus S-phase response (cyclin related) (SPHAR), mRNA
ENSP00000386227-D1	-2.38	4.21E-06	↑	B. mutus mutated in colorectal cancers (MCC), mRNA
ENSBTAP00000016110-D1	-2.37	2.01E-05	↑	B. mutus tectonin beta-propeller repeat containing 2 (TECPR2), mRNA
ENSBTAP00000052092-D1	-2.36	6.98E-22	↑	B. mutus malignant T-cell-amplified sequence 1-like (LOC102270612), mRNA
ENSBTAP00000000445-D1	-2.36	4.75E-18	↑	B. mutus activity-dependent neuroprotector homeobox (ADNP), mRNA
ENSP00000377865-D1	-2.36	3.30E-21	↑	B. mutus ribosomal protein L23 (RPL23), mRNA
ENSBTAP00000011592-D1	-2.36	9.54E-05	↑	B. mutus XK, Kell blood group complex subunit-related family, member 8 (XKR8), mRNA
ENSBTAP00000041854-D1	-2.36	1.06E-16	↑	B. mutus fibronectin type III domain containing 4 (FNDC4), mRNA
ENSBTAP00000031895-D1	-2.35	7.58E-89	↑	B. mutus procollagen-lysine, 2-oxoglutarate 5-dioxygenase 2 (PLOD2), mRNA
ENSBTAP00000025870-D1	-2.35	3.09E-30	↑	B. mutus NADH dehydrogenase Fe-S protein 7, 20kDa (NADH-coenzyme Q reductase) (NDUFS7), mRNA
ENSP00000301939-D1	-2.35	1.26E-19	↑	B. mutus transmembrane protein 256 (TMEM256), mRNA
ENSP00000281525-D1	-2.34	1.49E-06	↑	B. mutus tryptophan 2,3-dioxygenase (TDO2), mRNA
ENSP00000360748-D1	-2.34	1.49E-06	↑	B. mutus transmembrane protein 141 (TMEM141), mRNA
ENSBTAP00000025264-D1	-2.34	4.55E-04	↑	B. mutus transmembrane protein 236 (TMEM236), mRNA
ENSP00000350260-D1	-2.34	4.55E-04	↑	B. mutus family with sequence similarity 219, member B (FAM219B), mRNA
ENSP00000361708-D1	-2.34	2.35E-08	↑	B. mutus transcription elongation factor A (SII)-like 1 (TCEAL1), mRNA
ENSBTAP00000002297-D1	-2.33	7.09E-06	↑	B. mutus fucosidase, alpha-L- 2, plasma (FUCA2), mRNA
ENSP00000269190-D1	-2.33	7.09E-06	↑	B. mutus dystrobrevin, alpha (DTNA), transcript variant X1, mRNA
ENSP00000384712-D1	-2.33	1.12E-07	↑	B. mutus CUE domain containing 1 (CUEDC1), partial mRNA
ENSBTAP00000025389-D4	-2.33	1.44E-43	↑	B. mutus epithelial membrane protein 3 (EMP3), mRNA
ENSBTAP00000035146-D1	-2.32	8.78E-23	↑	B. mutus 60S ribosomal protein L23a-like (LOC102287861), mRNA
ENSBTAP00000041817-D1	-2.32	2.06E-12	↑	B. mutus selenoprotein M-like (LOC102284466), mRNA
ENSBTAP00000013198-D1	-2.32	1.32E-10	↑	B. mutus mitogen-activated protein kinase 13 (MAPK13), mRNA
ENSBTAP00000026577-D1	-2.32	3.36E-05	↑	B. mutus prenylcysteine oxidase 1 like (PCYOX1L), mRNA
ENSBTAP00000003205-D1	-2.32	2.20E-03	↑	B. taurus caspase 9, apoptosis-related cysteine peptidase (CASP9), mRNA
ENSP00000249330-D1	-2.32	2.20E-03	↑	B. mutus VGF nerve growth factor inducible (VGF), mRNA
ENSBTAP00000042600-D1	-2.32	2.20E-03	↑	B. mutus serpin peptidase inhibitor, clade A, member 10 (SERPINA10), mRNA
ENSBTAP00000053170-D1	-2.32	2.20E-03	↑	B. taurus pleckstrin homology domain containing, family A member 4 (PLEKHA4), transcript variant X1, mRNA
ENSP00000339175-D1	-2.32	2.20E-03	↑	B. mutus nudix (nucleoside diphosphate linked moiety X)-type motif 14 (NUDT14), mRNA
ENSP00000233609-D1	-2.32	6.50E-248	↑	B. mutus ribosomal protein S15 (RPS15), mRNA
ENSP00000406751-D1	-2.31	4.66E-11	↑	B. mutus cryptochrome 2 (photolyase-like) (CRY2), mRNA
ENSP00000334314-D1	-2.31	2.51E-06	↑	Bubalus bubalis echinoderm microtubule associated protein like 1 (EML1), transcript variant X3, mRNA
ENSBTAP00000023671-D1	-2.31	2.51E-06	↑	B. mutus RAB14, member RAS oncogene family (RAB14), mRNA
ENSBTAP00000001158-D1	-2.30	2.97E-09	↑	Bubalus bubalis tripartite motif containing 27 (TRIM27), mRNA
ENSBTAP00000023078-D1	-2.30	2.97E-09	↑	B. mutus excision repair cross-complementing rodent repair deficiency, complementation group 1, mRNA
ENSBTAP00000025963-D5	-2.30	3.72E-115	↑	Capra hircus ribosomal protein L10a (RPL10A), transcript variant X1, mRNA
ENSBTAP00000023192-D1	-2.30	4.02E-15	↑	B. mutus kelch-like family member 24 (KLHL24), mRNA
ENSBTAP00000015829-D1	-2.30	0.00E+00	↑	B. mutus CD63 molecule (CD63), transcript variant X1, mRNA

ENSBTAP00000012517-D1	-2.30	1.87E-07	↑	B. mutus EPS8-like 1 (EPS8L1), mRNA
ENSBTAP00000041757-D1	-2.30	1.61E-04	↑	B. mutus ribonuclease L (2',5'-oligoisoadenylate synthetase-dependent) (RNASEL), mRNA
ENSBTAP00000014000-D1	-2.29	1.65E-11	↑	B. mutus protein ZNF365-like (LOC102275040), mRNA
ENSBTAP00000024572-D1	-2.29	2.33E-194	↑	B. mutus vimentin (VIM), mRNA
ENSP00000398153-D1	-2.29	1.19E-05	↑	Gorilla gorilla gorilla uncharacterized LOC101146367 (LOC101146367), mRNA
ENSBTAP00000020046-D1	-2.28	2.63E-77	↑	B. mutus potassium channel, subfamily K, member 18 (KCNK18), mRNA
ENSBTAP00000029136-D1	-2.28	5.80E-12	↑	B. mutus R3H domain and coiled-coil containing 1-like (R3HCC1L), transcript variant X1, mRNA
ENSP00000375957-D1	-2.28	6.61E-08	↑	B. mutus mitochondrial chaperone BCS1-like (LOC102274064), transcript variant X3, mRNA
ENSP00000372445-D1	-2.28	4.95E-09	↑	B. mutus methyltransferase like 17 (METTL17), mRNA
ENSP00000359380-D1	-2.27	9.12E-21	↑	B. mutus stearoyl-CoA desaturase (delta-9-desaturase) (SCD), mRNA
ENSP00000294923-D1	-2.27	4.19E-06	↑	B. mutus desumoylating isopeptidase 2 (DESI2), transcript variant X2, mRNA
ENSP00000265348-D2	-2.27	5.62E-05	↑	Bubalus bubalis cullin 7 (CUL7), mRNA
yakG012203	-2.27	7.64E-04	↑	Capra hircus nitric oxide synthase 1 (neuronal) adaptor protein (NOS1AP), transcript variant X2, mRNA
ENSP00000377233-D1	-2.27	7.64E-04	↑	B. mutus ArfGAP with RhoGAP domain, ankyrin repeat and PH domain 1 (ARAP1), transcript variant X1, mRNA
ENSP00000362344-D1	-2.27	7.64E-04	↑	B. mutus folylpolyglutamate synthase (FPGS), mRNA
ENSBTAP00000003928-D1	-2.27	7.64E-04	↑	B. mutus scavenger receptor cysteine rich domain containing, group B (4 domains) (SRCRB4D), mRNA
ENSP00000310094-D1	-2.27	7.64E-04	↑	Bubalus bubalis TAO kinase 2 (TAOK2), transcript variant X1, mRNA
ENSP00000326813-D1	-2.27	7.64E-04	↑	B. mutus YOD1 deubiquitinase (YOD1), mRNA
yakA21441	-2.27	7.64E-04	↑	Pantholops hodgsonii serine incorporator 4 (SERINC4), mRNA
ENSP00000417928-D1	-2.27	7.64E-04	↑	B. mutus ORAI calcium release-activated calcium modulator 2 (ORAI2), mRNA
ENSBTAP00000037251-D1	-2.27	1.07E-02	↑	B. taurus maestro heat-like repeat family member 1 (MROH1), mRNA
ENSBTAP00000004042-D1	-2.27	1.07E-02	↑	B. mutus LIM homeobox 5 (LHX5), mRNA
ENSP00000164133-D1	-2.27	1.07E-02	↑	B. mutus protein phosphatase 2, regulatory subunit B', beta (PPP2R5B), mRNA
ENSBTAP00000049815-D1	-2.27	1.07E-02	↑	B. taurus olfactory receptor 4E1 (LOC522609), mRNA
ENSP00000351345-D1	-2.27	1.07E-02	↑	B. mutus family with sequence similarity 150, member A (FAM150A), mRNA
ENSP00000403636-D1	-2.27	1.07E-02	↑	B. taurus synaptic Ras GTPase activating protein 1 (SYNGAP1), mRNA
ENSP00000332967-D1	-2.27	1.07E-02	↑	Bubalus bubalis solute carrier family 22, member 31 (SLC22A31), mRNA
ENSBTAP00000021892-D1	-2.27	1.07E-02	↑	B. mutus 7-dehydrocholesterol reductase (DHCR7), mRNA
ENSP00000371219-D1	-2.27	1.07E-02	↑	B. mutus ras homolog family member H (RHOH), mRNA
ENSBTAP00000035133-D1	-2.26	2.57E-27	↑	B. mutus eukaryotic translation termination factor 1 (ETF1), mRNA
ENSP00000359521-D1	-2.25	1.11E-07	↑	B. mutus SLIT and NTRK-like family, member 2 (SLITRK2), transcript variant X3, mRNA
ENSP00000368104-D1	-2.25	1.11E-07	↑	B. mutus bone morphogenetic protein 2 (BMP2), mRNA
ENSBTAP00000038304-D1	-2.24	7.65E-11	↑	B. taurus protein phosphatase 2, regulatory subunit B', alpha (PPP2R5A), mRNA
ENSBTAP00000007315-D1	-2.23	2.68E-04	↑	B. mutus ceroid-lipofuscinosis, neuronal 6, late infantile, variant (CLN6), mRNA
ENSP00000393043-D1	-2.23	2.68E-04	↑	B. mutus ankyrin and armadillo repeat containing (ANKAR), transcript variant X2, mRNA
ENSP00000282470-D1	-2.23	7.00E-39	↑	B. mutus SPARC-like 1 (hevin) (SPARCL1), mRNA
ENSBTAP00000015413-D1	-2.23	2.69E-11	↑	B. mutus mitogen-activated protein kinase kinase kinase 8 (MAP3K8), mRNA
ENSP00000348849-D4	-2.23	3.61E-10	↑	B. mutus ribosomal protein S26 (RPS26), mRNA
ENSP00000362287-D4	-2.23	6.98E-06	↑	B. mutus argonaute RISC catalytic component 4 (AGO4), mRNA
ENSBTAP00000006862-D1	-2.23	6.98E-06	↑	B. mutus small nuclear RNA activating complex, polypeptide 5, 19kDa (SNAPC5), mRNA
ENSBTAP00000028238-D1	-2.22	3.30E-26	↑	B. mutus cadherin-2-like (LOC102266027), mRNA
ENSBTAP00000042184-D1	-2.21	3.03E-14	↑	B. mutus latexin (LXN), mRNA
ENSP00000400664-D1	-2.21	2.46E-06	↑	B. taurus receptor (G protein-coupled) activity modifying protein 2 (RAMP2), mRNA
ENSBTAP00000005621-D1	-2.21	9.40E-05	↑	B. mutus ellis-van Creveld syndrome protein-like (LOC102273071), partial mRNA
ENSP00000399808-D1	-2.21	3.70E-03	↑	B. mutus homeobox C4 (HOXC4), transcript variant X2, mRNA
ENSP00000310832-D1	-2.21	3.70E-03	↑	B. mutus cathepsin F (CTSF), transcript variant X1, mRNA
yakG004837	-2.21	3.70E-03	↑	B. mutus fer-1-like protein 4-like (LOC102288206), mRNA
ENSP00000162391-D1	-2.21	3.70E-03	↑	B. mutus forkhead box J2 (FOXJ2), mRNA
ENSP00000263125-D2	-2.21	3.70E-03	↑	B. mutus protein kinase C, delta (PRKCD), mRNA
ENSP00000188790-D1	-2.21	3.70E-03	↑	B. mutus fibroblast activation protein, alpha (FAP), transcript variant X2, mRNA
ENSP00000384983-D1	-2.21	3.70E-03	↑	B. mutus formin-2-like (LOC102266504), partial mRNA
ENSBTAP00000028661-D1	-2.21	1.58E-42	↑	B. mutus ADP-ribosylation-like factor 6 interacting protein 5 (ARL6IP5), mRNA
ENSBTAP00000019041-D1	-2.20	5.97E-10	↑	B. mutus solute carrier family 33 (acetyl-CoA transporter), member 1 (SLC33A1), transcript variant X2, mRNA
ENSBTAP00000011980-D1	-2.20	5.97E-10	↑	B. mutus DnaJ (Hsp40) homolog, subfamily C , member 25 (DNAJC25), mRNA
ENSBTAP00000000086-D1	-2.20	6.76E-44	↑	B. mutus GLI pathogenesis-related 2 (GLIPR2), mRNA

ENSBTAP00000028016-D1	-2.20	1.93E-12	↑	B. mutus cathepsin K (CTSK), mRNA
yakG044082	-2.20	3.29E-05	↑	B. mutus mitochondrial ribosomal protein S36 (MRPS36), mRNA
ENSBTAP00000051671-D33	-2.20	3.29E-05	↑	B. taurus Y Chr BAC CH240-278G19 complete sequence
ENSBTAP00000018697-D1	-2.20	3.29E-05	↑	B. mutus neurexin 1 (NRXN1), transcript variant X1, mRNA
ENSP00000340989-D1	-2.20	3.29E-05	↑	B. mutus stratifin (SFN), mRNA
ENSBTAP00000007036-D6	-2.19	1.63E-137	↑	Bubalus bubalis ribosomal protein L27a (RPL27A), mRNA
ENSBTAP00000009779-D1	-2.19	3.03E-07	↑	B. mutus tektin-1-like (LOC102276407), mRNA
ENSBTAP00000021516-D1	-2.18	1.16E-05	↑	B. mutus keratin 7 (KRT7), mRNA
ENSP00000376148-D1	-2.18	1.28E-03	↑	B. mutus armadillo repeat containing 6 (ARMC6), transcript variant X1, mRNA
ENSBTAP00000006032-D1	-2.18	1.28E-03	↑	B. mutus pim-2 oncogene (PIM2), mRNA
ENSBTAP00000010234-D2	-2.18	1.28E-03	↑	B. mutus N(alpha)-acetyltransferase 50, NatE catalytic subunit (NAA50), mRNA
ENSBTAP00000052415-D1	-2.18	1.28E-03	↑	Bubalus bubalis ovochymase 1 (OVCH1), mRNA
ENSBTAP00000015978-D1	-2.18	1.28E-03	↑	B. mutus peptidyl arginine deiminase, type III (PADI3), mRNA
ENSP00000369003-D1	-2.18	1.28E-03	↑	B. mutus transient receptor potential cation channel, subfamily C, member 4, transcript variant X1, mRNA
ENSP00000384177-D1	-2.18	1.28E-03	↑	Bubalus bubalis MNT, MAX dimerization protein (MNT), mRNA
ENSBTAP00000003943-D1	-2.18	1.28E-03	↑	B. mutus growth arrest and DNA damage-inducible protein GADD45 gamma-like (LOC102276116), mRNA
ENSBTAP00000008215-D1	-2.18	1.28E-03	↑	B. mutus KIAA1614 ortholog (KIAA1614), mRNA
ENSBTAP00000041324-D1	-2.18	2.66E-16	↑	B. mutus mitogen-activated protein kinase 1 interacting protein 1-like (MAPK1IP1L), mRNA
ENSP00000384586-D1	-2.18	9.44E-17	↑	B. mutus MpV17 mitochondrial inner membrane protein (MPV17), transcript variant X1, mRNA
ENSP00000390724-D1	-2.17	1.32E-08	↑	B. mutus ribonuclease/angiogenin inhibitor 1 (RNH1), mRNA
ENSP00000276654-D1	-2.17	1.32E-08	↑	B. mutus low density lipoprotein receptor-related protein 12 (LRP12), transcript variant X1, mRNA
ENSBTAP00000007048-D1	-2.17	1.32E-08	↑	B. mutus suppression of tumorigenicity 5 (ST5), transcript variant X2, mRNA
ENSBTAP00000005660-D1	-2.17	1.32E-08	↑	B. taurus v-fos FBJ murine osteosarcoma viral oncogene homolog (FOS), mRNA, complete cds
ENSP00000239597-D1	-2.17	4.45E-04	↑	Homo sapiens cDNA FLJ11867 fis, clone HEMBA1006976, weakly similar to H.sapiens mRNA for Gal-beta(1-3/1-4)GlcNAc alpha-2.3-sialyltransferase
ENSP00000265071-D3	-2.17	4.45E-04	↑	B. mutus cadherin 10, type 2 (T2-cadherin) (CDH10), mRNA
ENSP00000363979-D1	-2.16	3.25E-54	↑	B. taurus dynein, light chain, roadblock-type 1 (DYNLRB1), transcript variant X1, mRNA
ENSP00000389630-D1	-2.16	2.55E-16	↑	B. mutus dpy-19-like 4 (C. elegans) (DPY19L4), mRNA
ENSBTAP00000045387-D1	-2.16	1.56E-04	↑	B. mutus desmocollin 3 (DSC3), transcript variant X1, mRNA
ENSBTAP00000050773-D48	-2.16	1.56E-04	↑	TPA_exp: B. taurus MUC5AC gene for mucin-5AC, exons 1-52
ENSP00000368517-D1	-2.16	1.56E-04	↑	B. mutus enoyl CoA hydratase domain containing 3 (ECHDC3), mRNA
ENSBTAP00000048967-D86	-2.16	1.56E-04	↑	B. taurus Y Chr BAC CH240-49F14 complete sequence
ENSP00000307234-D1	-2.16	1.56E-04	↑	B. mutus protocadherin beta 1 (PCDHB1), mRNA
ENSBTAP00000001955-D1	-2.15	5.43E-05	↑	B. taurus ilvB (bacterial acetolactate synthase)-like, mRNA (cDNA clone IMAGE:8412101), partial cds
ENSBTAP00000024227-D1	-2.14	1.91E-05	↑	B. mutus dihydroxyacetone kinase 2 homolog (S. cerevisiae) (DAK), partial mRNA
ENSP00000223095-D1	-2.14	7.92E-19	↑	B. mutus serpin peptidase inhibitor, clade E, member 1 (SERPINE1), transcript variant X1, mRNA
ENSBTAP00000001425-D1	-2.13	3.09E-27	↑	B. mutus SAYSVFN motif domain containing 1 (SAYSD1), mRNA
ENSBTAP00000027443-D1	-2.13	8.22E-07	↑	B. mutus OCIA domain containing 2 (OCIAD2), mRNA
ENSP00000261023-D1	-2.12	1.04E-50	↑	B. mutus integrin, alpha V (ITGAV), mRNA
ENSP00000382423-D1	-2.12	2.14E-18	↑	B. mutus mitogen-activated protein kinase kinase kinase 1, E3 ubiquitin protein ligase (MAP3K1), mRNA
ENSBTAP00000015875-D1	-2.12	1.46E-44	↑	B. taurus ribosomal protein S19 (RPS19), mRNA
ENSP00000249363-D1	-2.11	1.23E-18	↑	B. mutus leucine rich repeat containing 4 (LRRC4), transcript variant X1, mRNA
ENSP00000397435-D1	-2.11	4.31E-19	↑	B. mutus TSC22 domain family, member 1 (TSC22D1), transcript variant X1, mRNA
ENSBTAP00000005733-D1	-2.10	2.97E-72	↑	B. mutus canopy FGF signaling regulator 2 (CNPY2), mRNA
ENSBTAP00000016879-D1	-2.10	8.67E-20	↑	B. mutus exosome component 1 (EXOSC1), mRNA
ENSP00000355884-D2	-2.10	3.12E-10	↑	B. mutus MAP/microtubule affinity-regulating kinase 1 (MARK1), mRNA
ENSBTAP00000015193-D1	-2.10	2.05E-08	↑	B. mutus cAMP responsive element binding protein 3 (CREB3), transcript variant X1, mRNA
ENSBTAP00000011678-D2	-2.10	2.05E-08	↑	B. taurus calpain 2, (m/II) large subunit (CAPN2), mRNA
ENSP00000360683-D1	-2.10	4.72E-07	↑	B. mutus protein tyrosine phosphatase, non-receptor type 1 (PTPN1), mRNA
ENSBTAP00000036503-D1	-2.10	1.34E-06	↑	B. mutus transmembrane protein ENSP00000340100 homolog (LOC102270784), mRNA
ENSP00000361827-D5	-2.10	3.84E-06	↑	Bubalus bubalis G protein-coupled receptor associated sorting protein 1 (GPRASP1), mRNA
ENSP00000302599-D1	-2.10	3.12E-05	↑	B. mutus fucosyltransferase 9 (alpha (1,3) fucosyltransferase) (FUT9), mRNA
ENSBTAP00000029703-D1	-2.10	2.56E-04	↑	Balaenoptera acutorostrata scammoni FtsJ RNA methyltransferase homolog 1, transcript variant X5, mRNA
ENSP00000294671-D8	-2.10	2.56E-04	↑	Lipotes vexillifer guanylate binding protein 7 (GBP7), mRNA
ENSP00000380315-D28	-2.10	2.56E-04	↑	Bubalus bubalis zinc finger protein 84 (ZNF84), transcript variant X6, mRNA

ENSBTAP00000008994-D1	-2.10	7.33E-04	↑	B. taurus acylphosphatase 2, muscle type (ACYP2), mRNA
ENSBTAP00000009334-D1	-2.10	2.12E-03	↑	B. mutus thiamine triphosphatase (THTPA), mRNA
ENSBTAP00000041408-D1	-2.10	2.12E-03	↑	B. mutus Leber congenital amaurosis 5-like (LCA5L), mRNA
ENSBTAP00000050273-D68	-2.10	2.12E-03	↑	B. taurus clone RP42-210F11, complete sequence
ENSP00000252520-D1	-2.10	6.16E-03	↑	Pantholops hodgsonii inverted formin, FH2 and WH2 domain containing (INF2), mRNA
ENSP00000218006-D1	-2.10	6.16E-03	↑	B. mutus guanylate cyclase 2F, retinal (GUCY2F), mRNA
ENSBTAP00000021935-D1	-2.10	6.16E-03	↑	B. mutus zinc finger protein 740 (ZNF740), mRNA
ENSP00000370522-D1	-2.10	6.16E-03	↑	B. mutus arylsulfatase family, member H (ARSH), mRNA
ENSP00000312649-D1	-2.10	6.16E-03	↑	B. mutus peroxisome proliferator-activated receptor gamma, coactivator 1 beta (PPARGC1B), mRNA
ENSP00000301050-D1	-2.10	6.16E-03	↑	B. mutus calcium channel, voltage-dependent, beta 3 subunit (CACNB3), transcript variant X1, mRNA
ENSBTAP00000023309-D1	-2.10	6.16E-03	↑	B. mutus fetuin B (FETUB), mRNA
ENSBTAP00000020522-D1	-2.10	1.79E-02	↑	B. mutus solute carrier family 27 (fatty acid transporter), member 4 (SLC27A4), mRNA
ENSBTAP00000020468-D2	-2.10	1.79E-02	↑	Pantholops hodgsonii growth regulation by estrogen in breast cancer 1 (GREB1), transcript variant X3, mRNA
ENSBTAP00000025485-D3	-2.10	1.79E-02	↑	B. mutus oxysterol binding protein 2 (OSBP2), mRNA
ENSBTAP00000046236-D2	-2.10	1.79E-02	↑	B. mutus histone H2A-Bbd type 1-like (LOC102264563), mRNA
ENSP00000331462-D1	-2.10	1.79E-02	↑	B. mutus zinc finger protein 704 (ZNF704), mRNA
ENSBTAP00000040682-D1	-2.10	1.79E-02	↑	B. mutus dihydrodiol dehydrogenase (dimeric) (DHDH), mRNA
ENSP00000322191-D1	-2.10	1.79E-02	↑	Capra hircus beta-defensin 104a mRNA, complete cds
ENSP00000300134-D1	-2.10	1.79E-02	↑	B. mutus signal transducer and activator of transcription 6, interleukin-4 induced (STAT6), mRNA
ENSP00000248668-D1	-2.10	1.79E-02	↑	B. mutus leucine rich repeat and fibronectin type III domain containing 1 (LRFN1), mRNA
ENSP00000290075-D1	-2.10	1.79E-02	↑	B. mutus solute carrier family 25 (mitochondrial iron transporter), member 37 (SLC25A37), mRNA
ENSP00000361110-D1	-2.10	1.79E-02	↑	B. mutus globoside alpha-1,3-N-acetylgalactosaminyltransferase 1 (GBGT1), transcript variant X1, mRNA
ENSBTAP00000053765-D1	-2.10	1.79E-02	↑	B. mutus receptor tyrosine kinase-like orphan receptor 2 (ROR2), mRNA
ENSBTAP00000009647-D1	-2.10	1.79E-02	↑	Pantholops hodgsonii NFKBID, transcript variant X2, mRNA
ENSP00000362888-D1	-2.10	1.79E-02	↑	B. mutus 2-aminoethanethiol (cysteamine) dioxygenase (ADO), mRNA
ENSP00000287957-D1	-2.10	1.79E-02	↑	B. taurus GATA zinc finger domain containing 1 (GATAD1), mRNA
ENSP00000385695-D1	-2.10	1.79E-02	↑	B. mutus SEC14-like 6 (S. cerevisiae) (SEC14L6), mRNA
ENSBTAP00000008298-D1	-2.09	1.10E-28	↑	B. mutus aldehyde dehydrogenase 1 family, member L2 (ALDH1L2), mRNA
ENSP00000343445-D1	-2.08	4.06E-14	↑	B. mutus serpin B4-like (LOC102283923), partial mRNA
ENSP00000379769-D1	-2.08	3.64E-37	↑	B. mutus aconitase 2, mitochondrial (ACO2), mRNA
yakG027741	-2.08	5.09E-10	↑	B. mutus uncharacterized LOC102273590 (LOC102273590), mRNA
ENSP00000260045-D1	-2.08	5.34E-18	↑	B. mutus protein-kinase, interferon-inducible double stranded RNA dependent inhibitor, repressor of, mRNA
ENSBTAP00000003377-D1	-2.07	2.72E-98	↑	B. mutus FK506 binding protein 3, 25kDa (FKBP3), mRNA
ENSP00000354826-D1	-2.07	7.96E-60	↑	Pantholops hodgsonii caldesmon 1 (CALD1), partial mRNA
ENSBTAP00000002200-D1	-2.07	2.19E-32	↑	B. mutus stanniocalcin 1 (STC1), mRNA
ENSP00000346032-D2	-2.07	4.72E-129	↑	B. mutus annexin A2 (ANXA2), mRNA
ENSP00000415424-D1	-2.06	6.39E-27	↑	B. mutus single-pass membrane protein with aspartate-rich tail 1 (SMDT1), transcript variant X2, mRNA
ENSP00000417651-D1	-2.06	1.62E-15	↑	B. mutus cytochrome c oxidase copper chaperone-like (LOC102276020), mRNA
ENSP00000222305-D1	-2.06	1.08E-13	↑	B. mutus upstream transcription factor 2, c-fos interacting (USF2), mRNA
ENSP00000287196-D1	-2.06	4.03E-17	↑	B. mutus poly (ADP-ribose) polymerase family, member 6 (PARP6), mRNA
ENSP00000291577-D1	-2.05	2.50E-12	↑	B. mutus chromosome unknown open reading frame, human C21orf33 (LOC102265001), mRNA
ENSBTAP00000013230-D3	-2.05	2.84E-18	↑	B. mutus cytochrome P450 2J2-like (LOC102283844), mRNA
ENSBTAP00000015277-D1	-2.05	5.82E-11	↑	B. mutus phosphorylase, glycogen, liver (PYGL), mRNA
ENSBTAP00000002094-D2	-2.04	1.55E-07	↑	B. mutus phosphatidylinositol transfer protein, membrane-associated 1 (PITPNM1), mRNA
ENSBTAP00000011732-D1	-2.04	1.55E-07	↑	B. mutus transmembrane protein 98 (TMEM98), mRNA
ENSP00000283228-D1	-2.04	1.46E-04	↑	B. mutus protein tyrosine phosphatase, receptor type, R (PTPRR), transcript variant X1, mRNA
ENSP00000356988-D1	-2.04	4.72E-10	↑	B. mutus nitrilase 1 (NIT1), transcript variant X2, mRNA
ENSP00000222008-D1	-2.04	8.78E-38	↑	B. mutus Rab acceptor 1 (prenylated) (RABAC1), mRNA
ENSBTAP00000003767-D1	-2.04	4.17E-04	↑	B. mutus family with sequence similarity 111, member B (FAM111B), mRNA
ENSBTAP00000043683-D1	-2.04	4.17E-04	↑	B. mutus leucine rich repeat and Ig domain containing 1 (LINGO1), mRNA
ENSP00000361740-D1	-2.04	4.17E-04	↑	B. mutus WW domain binding protein 5 (WBP5), mRNA
ENSP00000293760-D1	-2.04	4.17E-04	↑	B. mutus LEM domain containing 2 (LEMD2), mRNA
ENSP00000350667-D3	-2.03	2.24E-48	↑	B. mutus tropomyosin 4 (TPM4), transcript variant X1, mRNA
ENSBTAP00000017839-D1	-2.02	3.44E-289	↑	B. mutus enolase 1, (alpha) (ENO1), transcript variant X1, mRNA
ENSP00000401980-D1	-2.02	1.02E-05	↑	B. mutus mitochondrial antiviral signaling protein (MAVS), mRNA

ENSBTAP00000029367-D1	-2.02	1.20E-03	↑	B. mutus guanine nucleotide binding protein (G protein), beta polypeptide 1-like (GNB1L), mRNA
ENSP00000268864-D1	-2.02	1.20E-03	↑	B. mutus RAS-like, family 10, member B (RASL10B), mRNA
ENSP00000372199-D1	-2.02	1.20E-03	↑	B. mutus URB1 ribosome biogenesis 1 homolog (S. cerevisiae) (URB1), mRNA
ENSP00000390728-D1	-2.02	1.20E-03	↑	B. mutus ADAM metallopeptidase domain 8 (ADAM8), mRNA
ENSP00000359211-D1	-2.02	1.20E-03	↑	B. mutus dihydropyrimidine dehydrogenase [NADP(+)]-like (LOC102267734), mRNA
ENSBTAP00000048967-D99	-2.02	2.18E-09	↑	B. taurus Y Chr BAC CH240-246E9 complete sequence
ENSP00000407487-D1	-2.02	2.18E-09	↑	B. mutus unc-45 homolog A (C. elegans) (UNC45A), mRNA
ENSP00000409815-D2	-2.02	8.65E-83	↑	B. mutus proteasome (prosome, macropain) subunit, alpha type, 2 (PSMA2), mRNA
ENSBTAP00000030798-D1	-2.01	1.33E-55	↑	B. mutus zinc finger protein 385B (ZNF385B), transcript variant X4, mRNA
ENSBTAP00000020159-D1	-2.01	5.74E-40	↑	B. taurus myeloid cell leukemia 1 (MCL1), mRNA
ENSP00000358814-D1	-2.01	1.76E-08	↑	B. mutus adenosylhomocysteinase-like 1 (AHCYL1), transcript variant X2, mRNA
ENSBTAP00000001628-D1	-2.01	6.42E-15	↑	B. taurus cadherin-related family member 1 (CDHR1), mRNA
ENSP00000282928-D1	-2.00	8.25E-05	↑	Bubalus bubalis Zic family member 1 (ZIC1), mRNA
ENSP00000353393-D1	-2.00	8.25E-05	↑	B. mutus coagulation factor VIII, procoagulant component (F8), mRNA
ENSBTAP00000025343-D1	-2.00	8.25E-05	↑	B. mutus phospholipase B domain containing 2 (PLBD2), transcript variant X1, mRNA
ENSBTAP00000022157-D1	-2.00	3.47E-03	↑	B. taurus ubiquitin specific peptidase 18 (USP18), mRNA
ENSP00000391669-D2	-2.00	3.47E-03	↑	B. mutus neural precursor cell expressed, developmentally down-regulated 9 (NEDD9), mRNA
ENSBTAP00000022184-D1	-2.00	3.47E-03	↑	B. mutus zinc finger protein 202 (ZNF202), mRNA
ENSBTAP00000023987-D2	-2.00	3.47E-03	↑	Bubalus bubalis B-cell CLL/lymphoma 11A (zinc finger protein) (BCL11A), transcript variant X3, mRNA
ENSBTAP00000042158-D1	-2.00	3.47E-03	↑	B. mutus prostate transmembrane protein, androgen induced 1 (PMEPA1), mRNA
ENSP00000392188-D7	-2.00	3.47E-03	↑	B. taurus clone RP42-324C24, complete sequence
ENSBTAP00000042923-D2	-2.00	3.47E-03	↑	B. mutus phosphatidic acid phosphatase type 2 domain containing 1B (PPAPDC1B), mRNA
ENSP00000284770-D1	-1.99	5.78E-06	↑	B. mutus PDZ and LIM domain 3 (PDLIM3), transcript variant X1, mRNA
ENSP00000377102-D1	-1.99	2.63E-31	↑	B. mutus chromosome unknown open reading frame, human C11orf30, transcript variant X3, mRNA
ENSBTAP00000017908-D1	-1.99	1.73E-11	↑	B. mutus neurexophilin and PC-esterase domain family, member 3 (NXPE3), mRNA
ENSP00000378554-D1	-1.99	2.36E-04	↑	B. mutus armadillo repeat containing, X-linked 3 (ARMCX3), mRNA
ENSBTAP00000051893-D1	-1.98	2.00E-09	↑	B. mutus kynurenine--oxoglutarate transaminase 1-like (LOC102274277), partial mRNA
ENSP00000354703-D1	-1.98	1.65E-05	↑	B. mutus zinc finger protein 358 (ZNF358), mRNA
ENSP00000382237-D1	-1.98	1.65E-05	↑	B. mutus transmembrane protein 26 (TMEM26), mRNA
ENSBTAP00000008425-D1	-1.98	8.18E-17	↑	B. mutus troponin I type 3 (cardiac) (TNNI3), mRNA
ENSP00000262946-D4	-1.98	4.76E-14	↑	B. mutus cytochrome b-c1 complex subunit 10-like (LOC102273579), mRNA
ENSBTAP00000047226-D5	-1.98	8.08E-08	↑	B. taurus BAC CH240-319N17
ENSP00000377833-D1	-1.98	3.69E-40	↑	B. mutus annexin A4 (ANXA4), transcript variant X1, mRNA
ENSP00000367263-D1	-1.98	3.26E-27	↑	B. mutus AHNAK nucleoprotein (AHNAK), mRNA
ENSBTAP00000013466-D1	-1.97	8.27E-42	↑	B. mutus UDP-N-acteylglucosamine pyrophosphorylase 1 (UAP1), transcript variant X1, mRNA
ENSBTAP00000037593-D1	-1.97	7.92E-11	↑	B. mutus endothelin converting enzyme 1 (ECE1), mRNA
ENSP00000358879-D1	-1.97	2.29E-07	↑	B. mutus filamin A, alpha (FLNA), transcript variant X1, mRNA
ENSP00000394981-D1	-1.97	3.27E-06	↑	B. mutus D4, zinc and double PHD fingers family 1 (DPF1), transcript variant X2, mRNA
ENSP00000333633-D3	-1.97	4.66E-05	↑	B. mutus metastasis associated 1 family, member 2 (MTA2), mRNA
ENSBTAP00000005204-D1	-1.97	6.76E-04	↑	B. mutus CXXC finger protein 5 (CXXC5), mRNA
ENSBTAP00000047862-D1	-1.97	6.76E-04	↑	B. mutus NAD(P)H dehydrogenase, quinone 2 (NQO2), mRNA
ENSP00000319817-D1	-1.97	6.76E-04	↑	B. mutus optic atrophy 3 protein-like (LOC102281553), mRNA
ENSBTAP00000053865-D1	-1.97	6.76E-04	↑	Bubalus bubalis dachshund homolog 1-like (LOC102416212), mRNA
ENSBTAP00000009528-D88	-1.97	6.76E-04	↑	Bubalus bubalis zinc finger protein 584 (ZNF584), transcript variant X2, mRNA
ENSP00000400796-D1	-1.97	1.01E-02	↑	B. mutus isovaleryl-CoA dehydrogenase (IVD), mRNA
ENSP00000243349-D1	-1.97	1.01E-02	↑	B. mutus activin A receptor, type IC (ACVR1C), transcript variant X1, mRNA
ENSP00000406485-D1	-1.97	1.01E-02	↑	B. mutus smoothelin-like 1 (SMTNL1), mRNA
ENSBTAP00000014377-D1	-1.97	1.01E-02	↑	B. mutus cysteine-rich with EGF-like domains 1 (CRELD1), mRNA
ENSP00000243108-D1	-1.97	1.01E-02	↑	B. mutus homeobox C6 (HOXC6), mRNA
ENSP00000325941-D1	-1.97	1.01E-02	↑	B. mutus RIC8 guanine nucleotide exchange factor A (RIC8A), mRNA
ENSBTAP00000039202-D3	-1.97	1.01E-02	↑	B. mutus nuclear factor I/B (NFIB), mRNA
ENSBTAP00000053404-D1	-1.97	1.01E-02	↑	B. mutus mannosidase, alpha, class 2C, member 1 (MAN2C1), transcript variant X2, mRNA
ENSP00000352540-D1	-1.97	1.01E-02	↑	B. mutus epithelial membrane protein 2 (EMP2), mRNA
ENSBTAP00000012340-D1	-1.97	1.01E-02	↑	B. mutus diphthamide biosynthesis 1 (DPH1), mRNA
ENSBTAP00000019137-D1	-1.97	1.01E-02	↑	B. mutus myotubularin-related protein 9-like (LOC102264329), mRNA

ENSBTAP00000025322-D1	-1.97	1.01E-02	↑	B. mutus NADH dehydrogenase (ubiquinone) 1 alpha subcomplex, 11, 14.7kDa (NDUFA11), mRNA
ENSP00000323680-D1	-1.97	1.01E-02	↑	B. mutus DDB1 and CUL4 associated factor 11 (DCAF11), transcript variant X2, mRNA
ENSP00000389502-D1	-1.97	1.01E-02	↑	Bubalus bubalis DNA fragmentation factor, 40kDa, beta polypeptide (DFFB), transcript variant X4, mRNA
ENSBTAP00000029577-D1	-1.97	1.01E-02	↑	Capra hircus selectin P ligand (SELPLG), transcript variant X1, mRNA
ENSBTAP00000013862-D1	-1.96	3.22E-09	↑	B. mutus vacuolar protein sorting 4 homolog B (S. cerevisiae) (VPS4B), mRNA
ENSP00000301608-D1	-1.96	8.60E-15	↑	B. mutus acyl-CoA oxidase 1, palmitoyl (ACOX1), transcript variant X1, mRNA
ENSBTAP00000027459-D1	-1.95	1.33E-04	↑	B. mutus ATP-binding cassette, sub-family B (MDR/TAP), member 6 (ABCB6), mRNA
ENSBTAP00000003062-D1	-1.95	1.33E-04	↑	B. mutus transmembrane protein 217-like (LOC102287652), mRNA
ENSP00000361776-D4	-1.95	1.85E-06	↑	B. mutus brain expressed, X-linked 5 (BEX5), mRNA
ENSP00000401191-D1	-1.94	7.18E-24	↑	B. mutus Williams Beuren syndrome chromosome region 22 (WBSCR22), mRNA
ENSBTAP00000008393-D1	-1.94	2.64E-05	↑	B. mutus leucine-rich repeats and calponin homology (CH) domain containing 2, transcript variant X1, mRNA
ENSBTAP00000003094-D1	-1.94	1.94E-03	↑	B. mutus transforming growth factor beta 1 induced transcript 1 (TGFB1I1), mRNA
ENSP00000380594-D1	-1.94	1.94E-03	↑	B. mutus BPI fold containing family C (BPIFC), mRNA
ENSP00000411197-D1	-1.94	1.94E-03	↑	B. mutus TBC1 domain family member 9-like (LOC102266156), partial mRNA
ENSBTAP00000000922-D1	-1.94	1.94E-03	↑	B. mutus thioesterase superfamily member 5 (THEM5), mRNA
ENSBTAP00000002914-D1	-1.94	1.94E-03	↑	B. mutus apolipoprotein A-I (APOA1), partial mRNA
ENSP00000355827-D1	-1.94	5.39E-16	↑	B. taurus F-box protein 28 (FBXO28), mRNA
ENSBTAP00000021249-D1	-1.94	1.54E-15	↑	B. mutus glutaredoxin 2 (GLRX2), mRNA
ENSBTAP00000020148-D1	-1.93	0.00E+00	↑	Bubalus bubalis protein S100-A11-like (LOC102396947), mRNA
ENSP00000392188-D14	-1.93	3.82E-04	↑	B. taurus growth hormone receptor gene, complete cds
ENSP00000324205-D1	-1.93	1.91E-30	↑	B. mutus charged multivesicular body protein 4A (CHMP4A), mRNA
ENSP00000419194-D1	-1.93	2.08E-07	↑	B. mutus microseminoprotein, prostate associated (MSMP), mRNA
ENSP00000401645-D4	-1.93	6.82E-39	↑	B. mutus serpin peptidase inhibitor, clade B (ovalbumin), member 6 (SERPINB6), transcript variant X2, mRNA
ENSP00000341963-D1	-1.93	4.37E-29	↑	B. mutus regulatory solute carrier protein family 1 member 1-like (LOC102278947), mRNA
ENSBTAP00000035660-D1	-1.93	2.46E-15	↑	Bubalus bubalis signal peptide peptidase like 2B (SPPL2B), transcript variant X2, mRNA
ENSP00000404382-D1	-1.93	2.46E-15	↑	B. mutus chromosome unknown open reading frame, human C19orf57 (LOC102264996), mRNA
ENSBTAP00000000841-D1	-1.92	7.00E-15	↑	B. mutus adenine phosphoribosyltransferase (APRT), mRNA
ENSBTAP00000005899-D1	-1.92	2.96E-34	↑	B. mutus estrogen receptor 2 (ER beta) (ESR2), mRNA
ENSBTAP00000028828-D1	-1.92	2.95E-06	↑	B. mutus thymine-DNA glycosylase (TDG), mRNA
ENSBTAP00000009229-D1	-1.92	2.52E-55	↑	B. mutus target of myb1 (chicken)-like 1 (TOM1L1), mRNA
ENSBTAP00000021338-D1	-1.92	2.54E-12	↑	B. mutus exosome component 5 (EXOSC5), mRNA
ENSP00000210444-D1	-1.91	4.64E-09	↑	B. mutus N-acetylneuraminic acid synthase (NANS), mRNA
ENSP00000351100-D1	-1.91	9.21E-10	↑	B. mutus WD repeat domain 55 (WDR55), mRNA
ENSBTAP00000052348-D1	-1.90	1.95E-18	↑	B. taurus eukaryotic translation initiation factor 2B, subunit 2 beta, 39kDa, mRNA (cDNA clone MGC:143127 IMAGE:8284239), complete cds
ENSP00000386557-D1	-1.90	1.66E-06	↑	B. mutus uncharacterized LOC102283278 (LOC102283278), mRNA
ENSP00000410207-D1	-1.90	1.66E-06	↑	B. mutus methionine sulfoxide reductase B1 (MSRB1), mRNA
ENSBTAP00000016573-D5	-1.90	2.14E-04	↑	Pantholops hodgsonii allergen Bos d 2-like (LOC102336318), mRNA
ENSBTAP00000052128-D1	-1.90	2.14E-04	↑	B. mutus retrotransposon gag domain containing 1 (RGAG1), mRNA
yakG033567	-1.90	1.09E-03	↑	Chrysochloris asiatica poly(A) binding protein, nuclear 1 (PABPN1), mRNA
ENSBTAP00000002533-D60	-1.90	1.09E-03	↑	B. mutus zinc finger protein 543 (ZNF543), mRNA
ENSBTAP00000051671-D53	-1.90	1.09E-03	↑	B. taurus Y Chr BAC CH240-278G19 complete sequence
ENSBTAP00000020308-D1	-1.90	5.61E-03	↑	B. mutus N-sulfoglucosamine sulfohydrolase (SGSH), mRNA
ENSBTAP00000023305-D1	-1.90	5.61E-03	↑	B. mutus snail family zinc finger 3 (SNAI3), mRNA
ENSBTAP00000041235-D1	-1.90	5.61E-03	↑	B. mutus kinesin light chain 3 (KLC3), mRNA
ENSP00000361459-D1	-1.90	5.61E-03	↑	B. mutus TSC22 domain family, member 3 (TSC22D3), transcript variant X4, misc_RNA
ENSBTAP00000027172-D1	-1.90	5.61E-03	↑	B. mutus aldehyde dehydrogenase 1 family, member B1 (ALDH1B1), mRNA
ENSBTAP00000033586-D1	-1.90	3.01E-02	↑	Bubalus bubalis potassium voltage-gated channel, Shaw-related subfamily, member 4, transcript variant X2, mRNA
ENSBTAP00000050174-D65	-1.90	3.01E-02	↑	B. taurus cDNA clone IMAGE:8414372
ENSBTAP00000026209-D1	-1.90	3.01E-02	↑	B. mutus interleukin 1 receptor antagonist (IL1RN), transcript variant X1, mRNA
ENSBTAP00000015588-D1	-1.90	3.01E-02	↑	B. mutus transferrin receptor 2 (TFR2), mRNA
ENSBTAP00000032308-D1	-1.90	3.01E-02	↑	B. mutus synovial sarcoma translocation gene on chromosome 18-like 1 (SS18L1), transcript variant X1, mRNA
ENSBTAP00000053677-D1	-1.90	3.01E-02	↑	B. mutus contactin 4 (CNTN4), partial mRNA
ENSP00000392188-D34	-1.90	3.01E-02	↑	B. taurus Y Chr BAC CH240-82K5 complete sequence
ENSBTAP00000008848-D1	-1.90	3.01E-02	↑	B. mutus solute carrier family 7 (amino acid transporter light chain, L system), member 5 (SLC7A5), mRNA

ENSBTAP00000053211-D2	-1.90	3.01E-02	↑	B. mutus mitochondrial amidoxime reducing component 2 (MARC2), mRNA
ENSBTAP00000034269-D2	-1.90	3.01E-02	↑	B. mutus metallothionein 2A (MT2A), mRNA
ENSP00000355082-D1	-1.90	3.01E-02	↑	B. mutus protocadherin-18-like (LOC102266928), transcript variant X1, mRNA
ENSP00000300061-D1	-1.90	3.01E-02	↑	B. mutus sodium channel, non-voltage-gated 1, gamma subunit (SCNN1G), mRNA
ENSBTAP00000002098-D1	-1.90	3.01E-02	↑	B. mutus interleukin 4 receptor (IL4R), mRNA
ENSP00000273063-D1	-1.90	3.01E-02	↑	B. mutus solute carrier family 4 (anion exchanger), member 3 (SLC4A3), transcript variant X1, mRNA
yakA07029	-1.90	3.01E-02	↑	B. mutus TRPC5 opposite strand (TRPC5OS), mRNA
ENSP00000217254-D1	-1.90	3.01E-02	↑	B. mutus solute carrier family 52 (riboflavin transporter), member 3 (SLC52A3), mRNA
ENSP00000307852-D1	-1.90	3.01E-02	↑	B. mutus nudix (nucleoside diphosphate linked moiety X)-type motif 18 (NUDT18), mRNA
ENSBTAP00000001536-D1	-1.90	3.01E-02	↑	B. mutus diacylglycerol O-acyltransferase 2 (DGAT2), mRNA
ENSP00000320509-D2	-1.90	3.01E-02	↑	B. mutus B-cell scaffold protein with ankyrin repeats 1 (BANK1), mRNA
ENSBTAP00000053069-D4	-1.90	3.01E-02	↑	B. mutus mucin-19-like (LOC102275211), mRNA
ENSBTAP00000002404-D1	-1.90	3.01E-02	↑	B. mutus INO80 complex subunit B (INO80B), mRNA
ENSP00000084795-D3	-1.90	1.27E-134	↑	B. mutus ribosomal protein L18 (RPL18), mRNA
ENSP00000284981-D1	-1.90	1.77E-14	↑	B. mutus amyloid beta A4 protein-like (LOC102267321), mRNA
ENSP00000269290-D1	-1.90	2.92E-10	↑	B. mutus ribonuclease, RNase K (RNASEK), mRNA
ENSP00000366829-D1	-1.89	8.32E-25	↑	B. mutus RNA binding motif protein 10 (RBM10), transcript variant X3, mRNA
ENSBTAP00000028655-D1	-1.89	7.82E-17	↑	B. mutus prosaposin (PSAP), transcript variant X1, mRNA
ENSP00000364198-D1	-1.89	5.59E-30	↑	B. mutus melanoma-associated antigen D2-like (LOC102279651), transcript variant X2, mRNA
ENSBTAP00000016768-D1	-1.89	1.81E-78	↑	B. mutus NADH dehydrogenase (ubiquinone) 1 beta subcomplex, 7, 18kDa (NDUFB7), mRNA
ENSP00000331201-D1	-1.89	5.03E-14	↑	B. mutus hepatocyte growth factor-regulated tyrosine kinase substrate (HGS), mRNA
ENSBTAP00000051259-D89	-1.89	2.55E-13	↑	B. taurus BAC CH240-316E7 complete sequence
ENSP00000052754-D1	-1.89	1.86E-07	↑	B. mutus decorin (DCN), transcript variant X2, mRNA
ENSP00000285141-D1	-1.88	2.37E-05	↑	Homo sapiens cDNA: FLJ23403 fis, clone HEP18857
ENSBTAP00000053646-D1	-1.88	8.28E-26	↑	B. mutus zinc finger E-box binding homeobox 2 (ZEB2), transcript variant X2, mRNA
ENSBTAP00000051259-D7	-1.88	1.20E-04	↑	B. taurus Y Chr BAC CH240-278G19 complete sequence
ENSP00000296325-D1	-1.88	2.34E-09	↑	B. mutus low density lipoprotein receptor-related protein associated protein 1 (LRPAP1), mRNA
ENSBTAP00000004874-D1	-1.88	1.03E-11	↑	Bubalus bubalis lysine (K)-specific demethylase 6A (KDM6A), transcript variant X4, mRNA
ENSP00000415110-D3	-1.88	1.86E-84	↑	Bubalus bubalis ribosomal protein L23a (RPL23A), transcript variant X1, mRNA
ENSBTAP00000018534-D1	-1.88	3.74E-108	↑	B. mutus aprataxin (APTX), transcript variant X2, mRNA
ENSP00000354091-D1	-1.87	2.51E-14	↑	B. mutus guanine nucleotide binding protein-like 3 (nucleolar)-like (GNL3L), transcript variant X1, mRNA
ENSBTAP00000013429-D1	-1.87	6.56E-09	↑	B. mutus golgi transport 1A (GOLT1A), mRNA
ENSBTAP00000023595-D1	-1.86	3.90E-40	↑	B. mutus interleukin 6 signal transducer (gp130, oncostatin M receptor) (IL6ST), transcript variant X1, mRNA
ENSP00000351137-D1	-1.86	3.59E-13	↑	B. mutus XPA binding protein 2 (XAB2), mRNA
ENSBTAP00000001239-D1	-1.86	1.48E-06	↑	B. mutus zinc finger protein 746 (ZNF746), mRNA
ENSP00000267085-D1	-1.86	6.70E-05	↑	B. mutus cysteine sulfinic acid decarboxylase (CSAD), transcript variant X3, mRNA
ENSP00000367345-D3	-1.86	3.12E-03	↑	B. mutus protocadherin gamma subfamily A, 5 (PCDHGA5), mRNA
ENSP00000354590-D1	-1.86	3.12E-03	↑	B. mutus vesicle transport protein SFT2A-like (LOC102268502), mRNA
ENSP00000351327-D1	-1.86	3.12E-03	↑	B. mutus A kinase (PRKA) anchor protein 4 (AKAP4), transcript variant X2, mRNA
ENSBTAP00000048042-D2	-1.86	4.67E-70	↑	B. mutus 60S acidic ribosomal protein P2-like (LOC102275763), mRNA
ENSP00000362469-D1	-1.86	8.20E-11	↑	B. mutus solute carrier family 2, member 8 (SLC2A8), transcript variant X1, mRNA
ENSP00000333037-D1	-1.86	1.66E-07	↑	B. mutus actin-related protein T3 (ACTRT3), mRNA
ENSBTAP00000007436-D1	-1.86	1.76E-30	↑	B. mutus proteasome (prosome, macropain) 26S subunit, non-ATPase, 2 (PSMD2), mRNA
ENSP00000364574-D1	-1.86	1.95E-31	↑	B. mutus negative elongation factor complex member E (NELFE), mRNA
ENSBTAP00000004954-D1	-1.86	4.13E-10	↑	B. taurus methyl-CpG binding domain protein 1 (MBD1), mRNA
ENSP00000349699-D1	-1.86	7.46E-06	↑	Bubalus bubalis PWWP domain-containing protein MUM1L1-like (LOC102399718), transcript variant X7, mRNA
ENSBTAP00000028036-D1	-1.85	3.39E-22	↑	B. mutus adrenomedullin (ADM), transcript variant X2, mRNA
ENSP00000392490-D1	-1.85	1.85E-08	↑	B. mutus polycystic kidney and hepatic disease 1 (autosomal recessive)-like 1 (PKHD1L1), mRNA
ENSBTAP00000012602-D1	-1.85	8.30E-07	↑	B. mutus UBX domain protein 11 (UBXN11), mRNA
ENSBTAP00000048632-D1	-1.85	8.30E-07	↑	B. taurus zinc finger protein 177 (ZNF177), mRNA
ENSBTAP00000043674-D2	-1.85	3.41E-04	↑	B. mutus glutathione S-transferase omega-1-like (LOC102264698), transcript variant X1, mRNA
ENSP00000415836-D1	-1.85	3.41E-04	↑	B. taurus zinc finger protein 276 (ZNF276), transcript variant X3, mRNA
ENSP00000367284-D2	-1.85	2.91E-26	↑	B. mutus FUN14 domain containing 1 (FUNDC1), mRNA
ENSBTAP00000028048-D1	-1.85	3.21E-37	↑	B. mutus proteasome (prosome, macropain) 26S subunit, ATPase, 5 (PSMC5), mRNA
ENSBTAP00000027623-D1	-1.85	3.75E-05	↑	B. mutus dehydrogenase/reductase (SDR family) member 7 (DHRS7), mRNA

ENSBTAP00000008383-D1	-1.85	3.19E-13	↑	B. mutus tubulin, gamma complex associated protein 2 (TUBGCP2), transcript variant X2, mRNA
ENSBTAP00000003073-D1	-1.84	1.30E-10	↑	B. mutus nucleobindin 1 (NUCB1), mRNA
ENSBTAP00000001982-D1	-1.84	4.65E-07	↑	B. mutus PDLIM1 interacting kinase 1 like (PDIK1L), transcript variant X1, mRNA
ENSBTAP00000020193-D1	-1.83	4.07E-11	↑	B. mutus chromosome unknown open reading frame, human C6orf201 (LOC102278660), mRNA
ENSBTAP00000053178-D1	-1.83	3.27E-09	↑	B. mutus F-box protein 11 (FBXO11), mRNA
ENSP00000339381-D1	-1.83	2.34E-06	↑	B. mutus regulator of G-protein signaling 12 (RGS12), partial mRNA
ENSBTAP00000013173-D1	-1.83	2.11E-05	↑	B. mutus integrin, beta 3 (platelet glycoprotein IIIa, antigen CD61) (ITGB3), mRNA
ENSP00000296266-D1	-1.83	1.90E-04	↑	Bubalus bubalis intraflagellar transport 122 homolog (Chlamydomonas) (IFT122), transcript variant X1, mRNA
ENSP00000312082-D1	-1.83	1.90E-04	↑	B. mutus toll-like receptor 8 (TLR8), mRNA
ENSP00000407460-D1	-1.83	1.90E-04	↑	B. mutus secernin 1 (SCRN1), transcript variant X1, mRNA
ENSP00000296414-D1	-1.83	1.90E-04	↑	B. mutus dual adaptor of phosphotyrosine and 3-phosphoinositides (DAPP1), mRNA
ENSBTAP00000043168-D1	-1.83	1.73E-03	↑	B. mutus endonuclease, polyU-specific (ENDOU), transcript variant X1, mRNA
ENSP00000318585-D1	-1.83	1.73E-03	↑	B. mutus beta-site APP-cleaving enzyme 1 (BACE1), transcript variant X1, mRNA
ENSBTAP00000015491-D1	-1.83	1.62E-02	↑	Pantholops hodgsonii potassium channel tetramerization domain containing 14 (KCTD14), mRNA
ENSBTAP00000038868-D82	-1.83	1.62E-02	↑	Human mariner1 transposase gene, complete consensus sequence
ENSP00000403290-D1	-1.83	1.62E-02	↑	B. mutus idnK, gluconokinase homolog (E. coli) (IDNK), mRNA
ENSBTAP00000020860-D1	-1.83	1.62E-02	↑	Bubalus bubalis BTG family, member 2 (BTG2), mRNA
ENSP00000165698-D1	-1.83	1.62E-02	↑	B. mutus receptor accessory protein 1 (REEP1), mRNA
ENSBTAP00000018089-D1	-1.83	1.62E-02	↑	B. taurus mRNA for vascular endothelial growth factor B186 precursor, partial cds
ENSBTAP00000051259-D60	-1.83	1.62E-02	↑	B. taurus Y Chr BAC CH240-278G19 complete sequence
ENSBTAP00000016090-D1	-1.83	1.62E-02	↑	B. mutus aminoadipate-semialdehyde synthase (AASS), transcript variant X2, mRNA
ENSBTAP00000001373-D1	-1.83	1.62E-02	↑	B. mutus phosphorylase, glycogen, muscle (PYGM), transcript variant X1, mRNA
ENSBTAP00000010992-D1	-1.83	3.95E-37	↑	B. taurus hydroxy-delta-5-steroid dehydrogenase, 3 beta- and steroid delta-isomerase 1, mRNA complete cds
ENSP00000376228-D1	-1.83	2.28E-11	↑	Pantholops hodgsonii ADP-ribosylation-like factor 6 interacting protein 4 (ARL6IP4), transcript variant X2, mRNA
ENSBTAP00000049230-D5	-1.83	4.87E-29	↑	Bubalus bubalis ribosomal protein L27a (RPL27A), mRNA
ENSP00000250237-D1	-1.83	3.22E-25	↑	B. mutus queuine tRNA-ribosyltransferase 1 (QTRT1), mRNA
ENSP00000299466-D1	-1.83	2.22E-27	↑	Pantholops hodgsonii spalt-like transcription factor 3 (SALL3), mRNA
ENSP00000352005-D81	-1.82	1.31E-06	↑	Bubalus bubalis zinc finger protein 665-like (LOC102393772), mRNA
ENSP00000311905-D1	-1.82	1.18E-05	↑	B. mutus latent transforming growth factor beta binding protein 4 (LTBP4), mRNA
ENSP00000379773-D1	-1.82	6.80E-17	↑	B. mutus zinc finger CCCH-type containing 7A (ZC3H7A), mRNA
ENSBTAP00000050712-D1	-1.82	1.77E-26	↑	Balaenoptera acutorostrata scammoni trans-2,3-enoyl-CoA reductase (TECR), mRNA
ENSP00000379406-D1	-1.82	1.06E-04	↑	B. mutus lipin 1 (LPIN1), transcript variant X2, mRNA
ENSBTAP00000001440-D1	-1.82	1.06E-04	↑	B. mutus four and a half LIM domains 2 (FHL2), mRNA
ENSP00000402140-D1	-1.81	1.70E-15	↑	B. mutus nitric oxide synthase trafficking (NOSTRIN), transcript variant X3, mRNA
ENSBTAP00000040566-D1	-1.81	2.07E-86	↑	B. mutus coiled-coil domain containing 23 (CCDC23), mRNA
ENSBTAP00000029666-D1	-1.81	1.80E-10	↑	B. mutus Rab interacting lysosomal protein-like 1 (RILPL1), transcript variant X2, mRNA
ENSP00000361465-D1	-1.81	5.90E-05	↑	B. mutus polymerase (RNA) I polypeptide C, 30kDa (POLR1C), mRNA
ENSBTAP00000003200-D1	-1.80	3.69E-06	↑	B. mutus family with sequence similarity 173, member A (FAM173A), mRNA
ENSBTAP00000032539-D1	-1.80	3.69E-06	↑	B. mutus nudix (nucleoside diphosphate linked moiety X)-type motif 7 (NUDT7), transcript variant X1, mRNA
ENSBTAP00000025356-D1	-1.80	3.69E-06	↑	B. mutus apoptosis-associated tyrosine kinase (AATK), mRNA
ENSBTAP00000042500-D1	-1.80	7.16E-148	↑	B. taurus mesencephalic astrocyte-derived neurotrophic factor (MANF), mRNA
ENSP00000406027-D1	-1.80	9.00E-10	↑	B. mutus EPM2A (laforin) interacting protein 1 (EPM2AIP1), mRNA
ENSBTAP00000032097-D1	-1.80	2.18E-13	↑	B. mutus transmembrane emp24 protein transport domain containing 5 (TMED5), transcript variant X1, mRNA
ENSP00000363397-D1	-1.80	1.29E-07	↑	B. mutus UDP-glucose ceramide glucosyltransferase (UGCG), mRNA
ENSBTAP00000007468-D1	-1.80	2.06E-06	↑	B. mutus elaC ribonuclease Z 1 (ELAC1), mRNA
ENSBTAP00000037054-D1	-1.80	5.34E-04	↑	B. mutus brain and acute leukemia, cytoplasmic (BAALC), mRNA
ENSP00000245323-D1	-1.80	5.34E-04	↑	B. mutus ephrin-B2 (EFNB2), partial mRNA
ENSP00000358202-D1	-1.80	8.95E-03	↑	B. mutus empty spiracles homeobox 2 (EMX2), mRNA
ENSBTAP00000049230-D6	-1.80	8.95E-03	↑	Bubalus bubalis ribosomal protein L27a (RPL27A), mRNA
ENSBTAP00000020387-D1	-1.79	1.15E-06	↑	B. mutus brain-specific angiogenesis inhibitor 3 (BAI3), mRNA
ENSP00000334234-D1	-1.79	2.53E-09	↑	B. mutus septin 10 (SEPT10), mRNA
ENSP00000355865-D1	-1.79	1.85E-05	↑	B. mutus E3 ubiquitin-protein ligase parkin-like (LOC102284805), partial mRNA
ENSP00000259569-D1	-1.79	4.02E-08	↑	B. mutus RAN binding protein 6 (RANBP6), transcript variant X1, mRNA
ENSBTAP00000000088-D1	-1.78	6.42E-07	↑	B. mutus glutamate-rich WD repeat containing 1 (GRWD1), mRNA
ENSP00000394510-D1	-1.78	2.99E-04	↑	B. mutus proline rich 12 (PRR12), mRNA

ENSP00000204279-D4	-1.78	2.99E-04	↑	B. mutus yippee-like 1 (Drosophila) (YPEL1), mRNA
ENSP00000254454-D1	-1.78	4.51E-18	↑	B. mutus leucine rich repeat containing 41 (LRRC41), mRNA
ENSP00000350750-D3	-1.78	4.51E-18	↑	B. mutus ribosomal protein L26 (RPL26), mRNA
ENSBTAP00000020468-D1	-1.78	3.59E-07	↑	B. mutus growth regulation by estrogen in breast cancer-like (GREB1L), mRNA
ENSBTAP00000013139-D1	-1.78	3.05E-21	↑	B. mutus phosducin-like 3 (PDCL3), mRNA
ENSP00000341422-D1	-1.77	2.02E-07	↑	B. mutus prolyl 4-hydroxylase, transmembrane (endoplasmic reticulum) (P4HTM), mRNA
ENSP00000262992-D1	-1.77	2.02E-07	↑	B. mutus inositol polyphosphate-4-phosphatase, type II, 105kDa (INPP4B), mRNA
ENSBTAP00000050423-D1	-1.77	1.67E-04	↑	B. mutus succinate dehydrogenase complex assembly factor 1 (SDHAF1), mRNA
ENSP00000420778-D1	-1.77	4.94E-03	↑	B. mutus UDP-GlcNAc:betaGal beta-1,3-N-acetylglucosaminyltransferase 5 (B3GNT5), mRNA
ENSP00000365397-D1	-1.77	4.94E-03	↑	B. mutus dehydrogenase/reductase (SDR family) member 3 (DHRS3), transcript variant X2, mRNA
ENSBTAP00000018681-D1	-1.77	4.94E-03	↑	B. mutus U6 snRNA-associated Sm-like protein LSm6-like (LOC102270235), mRNA
ENSBTAP00000004087-D1	-1.77	4.94E-03	↑	B. mutus TBCC domain containing 1 (TBCCD1), mRNA
yakA12314	-1.77	4.79E-12	↑	B. mutus DNA-directed RNA polymerases I, II, and III subunit RPABC2-like (LOC102287366), mRNA
ENSBTAP00000027417-D1	-1.77	7.69E-11	↑	B. mutus protease, serine 27 (PRSS27), mRNA
ENSP00000251296-D1	-1.77	8.32E-22	↑	B. mutus immunoglobin superfamily, member 21 (IGSF21), mRNA
ENSBTAP00000002229-D1	-1.77	6.30E-08	↑	B. mutus arrestin domain containing 2 (ARRDC2), mRNA
ENSBTAP00000010180-D1	-1.77	9.28E-05	↑	B. mutus pseudouridylate synthase 7 homolog (S. cerevisiae) (PUS7), mRNA
ENSBTAP00000021752-D1	-1.77	9.28E-05	↑	B. mutus TEA domain family member 2 (TEAD2), transcript variant X3, mRNA
ENSBTAP00000024902-D1	-1.76	6.89E-32	↑	B. mutus PDZ and LIM domain 7 (enigma) (PDLIM7), transcript variant X1, mRNA
ENSP00000258415-D1	-1.76	2.73E-03	↑	B. mutus sterol 26-hydroxylase, mitochondrial-like (LOC102275770), mRNA
ENSP00000389415-D1	-1.76	2.73E-03	↑	Bubalus bubalis coiled-coil domain containing 17 (CCDC17), mRNA
ENSBTAP00000008106-D1	-1.76	2.73E-03	↑	B. mutus RAB11 family interacting protein 5 (class I) (RAB11FIP5), mRNA
ENSP00000357835-D1	-1.76	2.73E-03	↑	B. mutus phospholysine phosphohistidine inorganic pyrophosphate phosphatase (LHPP), mRNA
ENSP00000381970-D2	-1.76	2.73E-03	↑	B. mutus gypsy retrotransposon integrase 1 (GIN1), transcript variant X1, mRNA
ENSP00000256010-D1	-1.75	6.14E-09	↑	B. mutus neurotensin (NTS), mRNA
ENSP00000301457-D1	-1.75	2.44E-15	↑	B. mutus NADH dehydrogenase (ubiquinone) 1 alpha subcomplex, 7, 14.5kDa (NDUFA7), mRNA
yakA09460	-1.75	1.51E-03	↑	B. taurus coatomer protein complex, subunit gamma 2 (COPG2), mRNA
ENSBTAP00000000519-D1	-1.75	1.17E-11	↑	B. mutus vitamin K epoxide reductase complex, subunit 1 (VKORC1), transcript variant X1, mRNA
ENSBTAP00000006277-D1	-1.75	9.82E-08	↑	B. mutus dynein, axonemal, heavy chain 2 (DNAH2), mRNA
ENSBTAP00000028235-D1	-1.75	1.03E-40	↑	Bubalus bubalis inhibitor of DNA binding 2, dominant negative helix-loop-helix protein (ID2), mRNA
ENSP00000378669-D1	-1.75	1.50E-34	↑	B. mutus aldolase A, fructose-bisphosphate (ALDOA), transcript variant X1, mRNA
ENSP00000315334-D1	-1.74	1.41E-32	↑	B. mutus Rho GTPase activating protein 24 (ARHGAP24), transcript variant X3, mRNA
ENSBTAP00000002402-D1	-1.74	6.49E-12	↑	B. mutus leucine rich repeat containing 36 (LRRC36), mRNA
ENSP00000379011-D2	-1.74	9.01E-06	↑	B. mutus carnitine palmitoyltransferase 1A (liver) (CPT1A), mRNA
ENSBTAP00000019639-D1	-1.74	9.01E-06	↑	B. mutus interferon stimulated exonuclease gene 20kDa (ISG20), mRNA
ENSBTAP00000003725-D1	-1.74	2.32E-72	↑	B. mutus COP9 signalosome subunit 6 (COPS6), mRNA
ENSBTAP00000015208-D1	-1.74	2.02E-12	↑	B. mutus histidine triad nucleotide binding protein 2 (HINT2), mRNA
ENSP00000385063-D1	-1.74	4.52E-19	↑	B. mutus transforming growth factor beta regulator 4 (TBRG4), transcript variant X2, mRNA
ENSBTAP00000023515-D1	-1.74	8.36E-04	↑	B. mutus nitric oxide synthase 3 (endothelial cell) (NOS3), mRNA
ENSBTAP00000008629-D1	-1.74	8.36E-04	↑	B. mutus seizure related 6 homolog (mouse)-like (SEZ6L), mRNA
ENSBTAP00000009545-D1	-1.74	4.97E-25	↑	B. mutus dual-specificity tyrosine-(Y)-phosphorylation regulated kinase 2 (DYRK2), mRNA
ENSBTAP00000007664-D1	-1.73	1.25E-18	↑	B. mutus c-mer proto-oncogene tyrosine kinase (MERTK), mRNA
ENSP00000243189-D1	-1.73	4.66E-04	↑	B. mutus chromosome unknown open reading frame, human C1orf63 (LOC102287901), mRNA
ENSBTAP00000021043-D1	-1.73	4.66E-04	↑	B. mutus homeobox protein abdominal-A-like (LOC102284868), mRNA
ENSBTAP00000022716-D1	-1.73	2.98E-09	↑	Bubalus bubalis aspartylglucosaminidase (AGA), transcript variant X1, mRNA
ENSBTAP00000001465-D2	-1.73	7.31E-137	↑	B. mutus mitochondrial ribosomal protein S14 (MRPS14), mRNA
ENSBTAP00000027511-D4	-1.73	2.60E-04	↑	B. mutus guanine nucleotide binding protein, alpha transducing 3 (GNAT3), mRNA
ENSBTAP00000023676-D1	-1.73	2.60E-04	↑	B. mutus solute carrier family 39, member 9 (SLC39A9), transcript variant X1, mRNA
ENSBTAP00000053297-D1	-1.73	2.60E-04	↑	B. mutus uncharacterized LOC102270160 (LOC102270160), mRNA
ENSBTAP00000041055-D2	-1.73	2.60E-04	↑	B. mutus heterogeneous nuclear ribonucleoprotein A/B (HNRNPAB), mRNA
ENSBTAP00000020185-D1	-1.72	2.40E-102	↑	B. mutus lactase (LCT), mRNA
ENSP00000354525-D1	-1.72	5.56E-17	↑	B. mutus mitochondrial ribosomal protein L24 (MRPL24), mRNA
ENSBTAP00000027250-D1	-1.72	1.48E-58	↑	B. mutus electron-transfer-flavoprotein, beta polypeptide (ETFB), mRNA
ENSBTAP00000012182-D2	-1.72	8.02E-05	↑	B. mutus protein phosphatase 2, catalytic subunit, beta isozyme (PPP2CB), mRNA
ENSBTAP00000040605-D6	-1.72	0.00E+00	↑	B. mutus zinc finger protein 226 (ZNF226), mRNA

ENSBTAP00000012599-D1	-1.72	7.99E-132	↑	B. mutus SH3 domain binding glutamic acid-rich protein like 3 (SH3BGRL3), mRNA
ENSP00000230671-D1	-1.72	4.47E-05	↑	B. mutus solute carrier family 6 (neurotransmitter transporter), member 7 (SLC6A7), mRNA
ENSBTAP00000049567-D1	-1.72	8.26E-09	↑	B. mutus FLYWCH family member 2 (FLYWCH2), mRNA
ENSP00000296358-D1	-1.72	2.50E-05	↑	B. mutus otopetrin 1 (OTOP1), mRNA
ENSP00000210187-D1	-1.72	2.50E-05	↑	Bubalus bubalis RAB26, member RAS oncogene family (RAB26), transcript variant X1, mRNA
ENSBTAP00000011521-D1	-1.71	4.80E-17	↑	B. mutus aldehyde dehydrogenase 2 family (mitochondrial) (ALDH2), transcript variant X1, mRNA
ENSP00000301587-D3	-1.71	4.48E-118	↑	B. mutus ATP synthase, H+ transporting, mitochondrial Fo complex, subunit d (ATP5H), mRNA
ENSBTAP00000048643-D19	-1.71	1.37E-15	↑	B. mutus zinc finger protein 404 (ZNF404), mRNA
ENSBTAP00000043726-D1	-1.71	9.77E-73	↑	B. mutus transferrin receptor (TFRC), mRNA
ENSBTAP00000028364-D1	-1.71	2.31E-17	↑	B. mutus proteasome (prosome, macropain) subunit, beta type, 4 (PSMB4), mRNA
ENSBTAP00000000721-D1	-1.70	1.89E-32	↑	B. mutus ATP synthase, H+ transporting, mitochondrial F1 complex, delta subunit (ATP5D), mRNA
ENSBTAP00000006653-D1	-1.70	0.00E+00	↑	B. mutus TIMP metallopeptidase inhibitor 1 (TIMP1), mRNA
ENSP00000263468-D1	-1.70	2.23E-09	↑	B. mutus KIAA1377 ortholog (KIAA1377), mRNA
ENSP00000358939-D1	-1.70	3.02E-58	↑	B. mutus seryl-tRNA synthetase (SARS), mRNA
ENSBTAP00000015319-D1	-1.69	8.52E-79	↑	B. mutus small integral membrane protein 11 (SMIM11), mRNA
ENSBTAP00000050572-D1	-1.68	3.98E-268	↑	Bubalus bubalis acyl-CoA synthetase long-chain family member 4 (ACSL4), transcript variant X1, mRNA
ENSP00000196551-D1	-1.68	4.45E-297	↑	B. mutus ribosomal protein S5 (RPS5), mRNA
ENSP00000310219-D1	-1.68	3.54E-26	↑	B. mutus heat shock 70 kDa protein 6-like (LOC102273821), mRNA
ENSBTAP00000016131-D1	-1.68	3.01E-13	↑	B. mutus coiled-coil domain containing 158 (CCDC158), mRNA
ENSP00000378882-D1	-1.68	5.58E-12	↑	B. mutus zinc finger protein 484 (ZNF484), mRNA
ENSBTAP00000023996-D1	-1.68	1.96E-08	↑	B. mutus LanC lantibiotic synthetase component C-like 2 (bacterial) (LANCL2), mRNA
ENSBTAP00000051331-D74	-1.68	6.31E-08	↑	B. taurus growth hormone receptor gene, complete cds
ENSBTAP00000008370-D1	-1.68	1.13E-07	↑	Bubalus bubalis transcription factor SPT20 homolog (LOC102399634), mRNA
ENSBTAP00000002156-D1	-1.68	2.08E-06	↑	B. mutus AAR2 splicing factor homolog (S. cerevisiae) (AAR2), mRNA
ENSP00000360722-D1	-1.68	3.74E-06	↑	B. taurus phosphohistidine phosphatase 1 (PHPT1), mRNA
ENSP00000396157-D3	-1.68	6.71E-06	↑	B. mutus sialic acid binding Ig-like lectin 8 (SIGLEC8), mRNA
ENSBTAP00000018760-D1	-1.68	1.20E-05	↑	B. taurus importin 8 (IPO8), mRNA
ENSBTAP00000002334-D1	-1.68	2.15E-05	↑	B. mutus seryl-tRNA synthetase 2, mitochondrial (SARS2), mRNA
ENSBTAP00000020682-D1	-1.68	2.15E-05	↑	B. mutus enkurin, TRPC channel interacting protein (ENKUR), mRNA
ENSBTAP00000047840-D1	-1.68	3.85E-05	↑	B. taurus NADH dehydrogenase Fe-S protein 6, 13kDa NDUFS6, transcript variant X1, mRNA
ENSP00000361245-D1	-1.68	3.85E-05	↑	B. mutus HECT domain containing E3 ubiquitin protein ligase 3 (HECTD3), mRNA
ENSP00000265965-D1	-1.68	6.92E-05	↑	B. mutus secretion regulating guanine nucleotide exchange factor (SERGEF), mRNA
ENSBTAP00000015942-D1	-1.68	1.25E-04	↑	B. mutus lemur tyrosine kinase 3 (LMTK3), mRNA
ENSP00000345412-D1	-1.68	2.24E-04	↑	B. mutus cleavage and polyadenylation specific factor 7, 59kDa (CPSF7), transcript variant X1, mRNA
ENSBTAP00000037387-D1	-1.68	4.03E-04	↑	B. mutus 2,4-dienoyl CoA reductase 2, peroxisomal (DECR2), mRNA
ENSBTAP00000036773-D3	-1.68	7.22E-04	↑	B. mutus 40S ribosomal protein S29-like (LOC102269755), mRNA
ENSP00000310472-D5	-1.68	1.30E-03	↑	B. mutus zinc finger protein 383 (ZNF383), mRNA
ENSBTAP00000021234-D1	-1.68	1.30E-03	↑	B. mutus acyl-CoA synthetase family member 3 (ACSF3), mRNA
ENSBTAP00000051809-D2	-1.68	1.30E-03	↑	B. mutus 60S ribosomal protein L26-like (LOC102266867), mRNA
ENSP00000354677-D1	-1.68	2.36E-03	↑	B. mutus glutathione peroxidase 7 (GPX7), mRNA
yakG021684	-1.68	2.36E-03	↑	B. mutus calpain, small subunit 1 (CAPNS1), mRNA
ENSBTAP00000036259-D1	-1.68	4.28E-03	↑	B. mutus chromosome unknown open reading frame, human C14orf164 (LOC102285910), mRNA
ENSBTAP00000049268-D3	-1.68	4.28E-03	↑	B. mutus serpin peptidase inhibitor, clade B (ovalbumin), member 4 (SERPINB4), mRNA
ENSBTAP00000007490-D1	-1.68	7.76E-03	↑	B. taurus cyclic nucleotide gated channel beta 1 (CNGB1), transcript variant 1, mRNA
ENSBTAP00000013154-D1	-1.68	7.76E-03	↑	B. mutus inhibin, alpha (INHA), mRNA
ENSBTAP00000053328-D1	-1.68	7.76E-03	↑	B. mutus protoporphyrinogen oxidase (PPOX), mRNA
ENSP00000268763-D1	-1.68	7.76E-03	↑	B. mutus kinase suppressor of ras 1 (KSR1), mRNA
ENSP00000396551-D1	-1.68	7.76E-03	↑	.
ENSP00000251472-D1	-1.68	1.41E-02	↑	B. mutus microtubule associated serine/threonine kinase 1 (MAST1), mRNA
ENSBTAP00000002536-D1	-1.68	1.41E-02	↑	B. mutus prominin 2 (PROM2), mRNA
yakG005699	-1.68	1.41E-02	↑	B. taurus ring finger protein 149 (RNF149), transcript variant X3, mRNA
ENSBTAP00000022755-D1	-1.68	1.41E-02	↑	B. mutus family with sequence similarity 8, member A1 (FAM8A1), mRNA
ENSBTAP00000018523-D1	-1.68	1.41E-02	↑	B. mutus diacylglycerol lipase, alpha (DAGLA), mRNA
ENSP00000323633-D1	-1.68	1.41E-02	↑	B. mutus DIP2 disco-interacting protein 2 homolog A (Drosophila) (DIP2A), mRNA
ENSP00000377171-D87	-1.68	1.41E-02	↑	B. taurus zinc finger protein 548 (ZNF548), mRNA

ENSBTAP00000040772-D1	-1.68	1.41E-02	↑	Bubalus bubalis basic helix-loop-helix family, member e41 (BHLHE41), mRNA
ENSBTAP00000040546-D1	-1.68	1.41E-02	↑	B. mutus lactamase, beta-like 1 (LACTBL1), mRNA
ENSP00000372649-D3	-1.68	1.41E-02	↑	B. mutus proline-rich protein 23C-like (LOC102280834), mRNA
ENSBTAP00000049695-D1	-1.68	1.41E-02	↑	B. mutus tumor necrosis factor receptor superfamily member 26-like (LOC102281177), mRNA
ENSBTAP00000046305-D1	-1.68	2.59E-02	↑	B. mutus Rho GTPase activating protein 42 (ARHGAP42), mRNA
ENSBTAP00000009942-D1	-1.68	2.59E-02	↑	Bubalus bubalis dual-specificity tyrosine-(Y)-phosphorylation regulated kinase 4 (DYRK4), mRNA
ENSBTAP00000004058-D1	-1.68	2.59E-02	↑	B. mutus FERM and PDZ domain containing 3 (FRMPD3), mRNA
ENSBTAP00000001095-D1	-1.68	2.59E-02	↑	B. mutus sedoheptulokinase (SHPK), mRNA
ENSBTAP00000047142-D1	-1.68	2.59E-02	↑	B. mutus multidrug resistance-associated protein 4-like (LOC102281982), mRNA
ENSP00000416512-D2	-1.68	2.59E-02	↑	Bubalus bubalis retinitis pigmentosa 9 (autosomal dominant) (RP9), transcript variant X2, mRNA
ENSP00000319052-D1	-1.68	2.59E-02	↑	B. mutus centrin, EF-hand protein, 1 (CETN1), transcript variant X1, mRNA
ENSBTAP00000024186-D1	-1.68	2.59E-02	↑	B. taurus hexosaminidase (glycosyl hydrolase family 20, catalytic domain) containing (HEXDC), mRNA
ENSP00000394624-D1	-1.68	2.59E-02	↑	B. mutus opioid receptor, mu 1 (OPRM1), transcript variant X2, mRNA
ENSBTAP00000048041-D1	-1.68	2.59E-02	↑	Bubalus bubalis proline rich 24 (PRR24), mRNA
ENSP00000304410-D1	-1.68	4.96E-02	↑	B. mutus chromosome unknown open reading frame, human C2orf68, transcript variant X1, mRNA
ENSP00000239446-D5	-1.68	4.96E-02	↑	B. mutus protocadherin gamma-B1-like (LOC102280419), mRNA
ENSBTAP00000024811-D1	-1.68	4.96E-02	↑	B. mutus distal-less homeobox 5 (DLX5), mRNA
ENSP00000226432-D1	-1.68	4.96E-02	↑	B. mutus cell wall biogenesis 43 C-terminal homolog (S. cerevisiae) (CWH43), mRNA
ENSP00000325663-D1	-1.68	4.96E-02	↑	B. mutus NFKBIZ, transcript variant X2, mRNA
ENSBTAP00000010607-D1	-1.68	4.96E-02	↑	B. mutus vacuolar protein sorting 37 homolog C (S. cerevisiae) (VPS37C), mRNA
ENSP00000368666-D2	-1.68	4.96E-02	↑	B. mutus patched domain-containing protein 3-like (LOC102270365), partial mRNA
ENSBTAP00000022174-D1	-1.68	4.96E-02	↑	B. mutus phosphotriesterase related (PTER), transcript variant X1, mRNA
ENSP00000292114-D1	-1.68	4.96E-02	↑	B. mutus transmembrane protein 199 (TMEM199), mRNA
ENSBTAP00000007111-D1	-1.68	4.96E-02	↑	B. mutus hemicentin 2 (HMCN2), mRNA
ENSBTAP00000052257-D20	-1.68	4.96E-02	↑	B. taurus Y Chr BAC BTDAEX-299K14 (Amplicon Express Bovine BAC Library (male)) complete sequence
ENSBTAP00000044639-D18	-1.68	4.96E-02	↑	Lipotes vexillifer ribosomal protein S3A (RPS3A), mRNA
ENSP00000257868-D1	-1.68	4.96E-02	↑	B. mutus growth differentiation factor 11 (GDF11), mRNA
ENSBTAP00000043509-D1	-1.68	4.96E-02	↑	B. mutus corticotropin releasing hormone receptor 2 (CRHR2), transcript variant X4, mRNA
ENSP00000278829-D2	-1.68	4.96E-02	↑	B. mutus fatty acid desaturase 2 (FADS2), mRNA
ENSP00000378917-D1	-1.68	2.82E-28	↑	B. mutus ataxin 2-like (ATXN2L), transcript variant X1, mRNA
ENSP00000347170-D1	-1.68	6.40E-21	↑	B. mutus nei endonuclease VIII-like 1 (E. coli) (NEIL1), mRNA
ENSP00000371103-D4	-1.67	3.24E-32	↑	B. mutus ribosomal protein L37 (RPL37), mRNA
ENSBTAP00000004955-D1	-1.67	5.25E-09	↑	B. mutus RCD1 required for cell differentiation1 homolog (S. pombe) (RQCD1), mRNA
ENSBTAP00000007042-D1	-1.66	3.09E-07	↑	B. mutus vacuolar protein sorting 11 homolog (S. cerevisiae) (VPS11), transcript variant X2, mRNA
ENSP00000353846-D2	-1.66	5.54E-07	↑	Bubalus bubalis doublecortin-like kinase 1 (DCLK1), transcript variant X1, mRNA
ENSP00000300302-D1	-1.66	1.78E-06	↑	B. mutus homocysteine-inducible, endoplasmic reticulum stress-inducible, ubiquitin-like domain member 1, mRNA
ENSBTAP00000015353-D1	-1.66	4.24E-11	↑	B. mutus surfeit 6 (SURF6), mRNA
ENSP00000396929-D1	-1.66	1.73E-24	↑	B. mutus eukaryotic translation initiation factor 3, subunit F (EIF3F), transcript variant X1, mRNA
ENSP00000357304-D1	-1.65	2.44E-10	↑	Pantholops hodgsonii KIAA0907 ortholog (KIAA0907), transcript variant X1, mRNA
ENSP00000335094-D1	-1.65	4.36E-10	↑	B. mutus transmembrane protein 17 (TMEM17), mRNA
ENSBTAP00000002983-D1	-1.65	2.54E-17	↑	B. mutus pleiotrophin (PTN), transcript variant X2, mRNA
ENSP00000357384-D1	-1.65	3.38E-13	↑	B. mutus dolichyl-phosphate mannosyltransferase polypeptide 3 (DPM3), mRNA
ENSP00000386893-D2	-1.65	1.09E-12	↑	B. mutus peptidylprolyl isomerase (cyclophilin)-like 3 (PPIL3), mRNA
ENSP00000334314-D2	-1.65	3.30E-05	↑	B. mutus echinoderm microtubule associated protein like 2 (EML2), transcript variant X2, mRNA
ENSBTAP00000003276-D1	-1.65	3.49E-12	↑	B. mutus leydig cell tumor 10 kDa protein homolog (LOC102280028), mRNA
ENSP00000318472-D1	-1.65	4.62E-08	↑	B. mutus neural cell adhesion molecule 1 (NCAM1), transcript variant X2, mRNA
ENSP00000264198-D1	-1.64	1.07E-04	↑	B. mutus mitochondrial E3 ubiquitin protein ligase 1 (MUL1), mRNA
ENSP00000417628-D2	-1.64	1.92E-04	↑	B. mutus contactin-associated protein-like 3-like (LOC102267253), mRNA
ENSP00000240050-D1	-1.64	1.92E-04	↑	B. mutus MTERF domain containing 3 (MTERFD3), transcript variant X2, mRNA
ENSBTAP00000051852-D2	-1.64	9.28E-13	↑	B. mutus homeodomain interacting protein kinase 1 (HIPK1), mRNA
ENSBTAP00000035838-D1	-1.64	1.35E-40	↑	B. mutus erythrocyte membrane protein band 4.2 (EPB42), mRNA
ENSBTAP00000044620-D1	-1.64	4.73E-07	↑	B. mutus sphingosine-1-phosphate lyase 1 (SGPL1), mRNA
ENSP00000307449-D1	-1.64	3.44E-04	↑	B. mutus N-terminal EF-hand calcium binding protein 2 (NECAB2), mRNA
ENSP00000269391-D1	-1.64	3.44E-04	↑	B. mutus ring finger protein 157 (RNF157), mRNA
ENSP00000322439-D1	-1.63	1.16E-50	↑	B. mutus Tu translation elongation factor, mitochondrial (TUFM), mRNA

ENSBTAP00000048347-D1	-1.63	1.23E-08	↑	B. mutus quiescin Q6 sulfhydryl oxidase 1 (QSOX1), mRNA
ENSBTAP00000024795-D1	-1.63	6.17E-04	↑	B. mutus HD domain containing 3 (HDDC3), mRNA
ENSP00000351657-D25	-1.63	6.17E-04	↑	B. mutus zinc finger protein 814 (ZNF814), mRNA
ENSBTAP00000048664-D1	-1.63	0.00E+00	↑	B. mutus developmental pluripotency-associated protein 3-like (LOC102269367), mRNA
yakG015429	-1.63	1.11E-03	↑	Bubalus bubalis myosin, heavy chain 14, non-muscle (MYH14), transcript variant X5, mRNA
ENSBTAP00000053450-D1	-1.63	1.11E-03	↑	B. mutus histone deacetylase 5 (HDAC5), transcript variant X2, mRNA
ENSBTAP00000015066-D1	-1.62	1.83E-09	↑	B. mutus neuronal regeneration related protein (NREP), transcript variant X2, mRNA
ENSBTAP00000000447-D1	-1.62	4.03E-07	↑	B. mutus procollagen-lysine, 2-oxoglutarate 5-dioxygenase 3 (PLOD3), mRNA
ENSP00000258081-D1	-1.62	2.81E-05	↑	Bubalus bubalis ancient ubiquitous protein 1 (AUP1), mRNA
ENSBTAP00000050519-D1	-1.62	2.81E-05	↑	B. mutus histone deacetylase 6 (HDAC6), mRNA
ENSP00000058691-D1	-1.62	2.01E-03	↑	B. mutus uncharacterized LOC102268128 (LOC102268128), mRNA
ENSBTAP00000027999-D1	-1.62	2.01E-03	↑	B. mutus leprecan-like 1 (LEPREL1), transcript variant X2, mRNA
ENSP00000373477-D3	-1.62	1.65E-19	↑	B. mutus glutathione peroxidase 1 (GPX1), mRNA
ENSBTAP00000023743-D1	-1.62	6.67E-31	↑	B. mutus serglycin (SRGN), mRNA
ENSP00000252102-D3	-1.62	1.28E-70	↑	B. mutus NADH dehydrogenase (ubiquinone) 1 alpha subcomplex, 2, 8kDa (NDUFA2), mRNA
ENSBTAP00000032874-D1	-1.62	5.04E-05	↑	B. mutus arginine vasopressin-induced 1 (AVPI1), mRNA
ENSP00000222247-D1	-1.61	1.45E-78	↑	B. mutus ribosomal protein L18a (RPL18A), mRNA
ENSBTAP00000018997-D1	-1.61	8.38E-45	↑	B. mutus Mov10, Moloney leukemia virus 10, homolog (mouse) (MOV10), transcript variant X2, mRNA
ENSBTAP00000001558-D1	-1.61	1.55E-09	↑	B. mutus plexin A2 (PLXNA2), mRNA
ENSBTAP00000016639-D1	-1.61	3.64E-03	↑	B. mutus BEN domain containing 3 (BEND3), mRNA
ENSP00000241305-D1	-1.61	3.64E-03	↑	B. mutus carboxypeptidase X (M14 family), member 2 (CPXM2), mRNA
ENSBTAP00000000356-D1	-1.61	1.84E-12	↑	B. mutus GUF1 GTPase homolog (S. cerevisiae) (GUF1), mRNA
ENSBTAP00000042051-D1	-1.61	1.08E-07	↑	B. mutus ras responsive element binding protein 1 (RREB1), mRNA
ENSBTAP00000000448-D1	-1.61	9.95E-17	↑	B. mutus zinc finger, HIT-type containing 1 (ZNHIT1), mRNA
ENSBTAP00000007149-D1	-1.61	1.63E-04	↑	B. mutus LIM and cysteine-rich domains 1 (LMCD1), transcript variant X1, mRNA
ENSP00000247970-D1	-1.60	1.24E-14	↑	B. taurus peptidylprolyl cis/trans isomerase, NIMA-interacting 1 (PIN1), mRNA
ENSBTAP00000048457-D1	-1.60	1.90E-210	↑	B. taurus general transcription factor IIF, polypeptide 1, 74kDa, mRNA complete cds
ENSP00000350667-D1	-1.60	1.55E-48	↑	B. mutus tropomyosin alpha-1 chain-like (LOC102270744), transcript variant X1, mRNA
ENSBTAP00000042356-D1	-1.60	2.22E-14	↑	B. mutus mitochondrial inner membrane protease ATP23 homolog (LOC102280287), mRNA
ENSBTAP00000014306-D5	-1.60	2.71E-189	↑	B. mutus myosin, light chain 6, alkali, smooth muscle and non-muscle (MYL6), transcript variant X2, mRNA
ENSBTAP00000021397-D1	-1.60	1.48E-20	↑	Bubalus bubalis cytochrome c oxidase subunit 4 isoform 1, mitochondrial-like (LOC102407037), mRNA
ENSBTAP00000051635-D2	-1.60	2.78E-12	↑	B. mutus histone H2A-Bbd type 1-like (LOC102270543), mRNA
ENSP00000260356-D1	-1.60	6.08E-11	↑	B. mutus thrombospondin 1 (THBS1), mRNA
ENSBTAP00000008917-D1	-1.60	1.34E-05	↑	B. mutus napsin A aspartic peptidase (NAPSA), mRNA
ENSP00000341083-D1	-1.60	2.92E-04	↑	B. mutus natriuretic peptide receptor B/guanylate cyclase B (atrionatriuretic peptide receptor B) (NPR2), mRNA
ENSP00000268042-D1	-1.60	2.92E-04	↑	B. mutus arrestin domain-containing protein 4-like (LOC102267107), mRNA
ENSBTAP00000003218-D1	-1.60	2.92E-04	↑	B. mutus nudix (nucleoside diphosphate linked moiety X)-type motif 1 (NUDT1), transcript variant X1, mRNA
ENSP00000378792-D69	-1.60	6.59E-03	↑	B. mutus zinc finger protein 304-like (LOC102264621), mRNA
ENSP00000343290-D1	-1.60	6.59E-03	↑	B. mutus chromosome unknown open reading frame, human C9orf171 (LOC102282858), mRNA
ENSP00000311447-D1	-1.60	6.59E-03	↑	B. mutus aspartate beta-hydroxylase domain containing 1 (ASPHD1), transcript variant X2, mRNA
ENSBTAP00000008725-D1	-1.60	6.59E-03	↑	B. mutus chromosome unknown open reading frame, human C4orf19 (LOC102279576), mRNA
ENSBTAP00000020147-D1	-1.60	1.06E-14	↑	B. mutus GEM interacting protein (GMIP), mRNA
ENSP00000401371-D1	-1.60	5.00E-12	↑	B. mutus TIA1 cytotoxic granule-associated RNA binding protein (TIA1), transcript variant X1, mRNA
ENSBTAP00000020263-D3	-1.60	1.22E-24	↑	Balaenoptera acutorostrata scammoni phospholipase A2, group XIIB (PLA2G12B), transcript variant X3, mRNA
ENSP00000399681-D3	-1.59	2.39E-05	↑	B. mutus dynamin 3 (DNM3), transcript variant X4, mRNA
ENSP00000361725-D1	-1.59	1.61E-11	↑	B. mutus endonuclease G (ENDOG), mRNA
ENSP00000356809-D1	-1.59	9.12E-08	↑	B. mutus G protein-coupled receptor 161-like (LOC102285066), transcript variant X1, mRNA
ENSBTAP00000013968-D1	-1.59	9.12E-08	↑	B. mutus dehydrogenase/reductase (SDR family) member 7B (DHRS7B), mRNA
ENSP00000233143-D4	-1.59	3.86E-118	↑	B. mutus thymosin beta 10 (TMSB10), mRNA
ENSBTAP00000032431-D1	-1.59	1.97E-06	↑	B. mutus spastic paraplegia 7 (pure and complicated autosomal recessive) (SPG7), partial mRNA
ENSP00000414147-D1	-1.59	1.97E-06	↑	B. mutus spastic paraplegia 20 (Troyer syndrome) (SPG20), mRNA
ENSP00000412663-D1	-1.59	5.22E-04	↑	B. mutus glycogen synthase kinase 3 alpha (GSK3A), mRNA
ENSBTAP00000003115-D1	-1.59	4.26E-05	↑	B. mutus uncharacterized LOC102271444 (LOC102271444), mRNA
ENSP00000288937-D1	-1.59	2.33E-22	↑	B. mutus mitochondrial ribosomal protein L17 (MRPL17), mRNA
ENSP00000379653-D1	-1.59	8.11E-118	↑	B. mutus BCL2-like 14 (apoptosis facilitator) (BCL2L14), transcript variant X2, mRNA

ENSP00000415243-D1	-1.59	2.90E-07	↑	B. mutus SKI-like oncogene (SKIL), transcript variant X1, mRNA
ENSBTAP00000016466-D1	-1.58	2.38E-21	↑	B. mutus peptidylglycine alpha-amidating monooxygenase (PAM), transcript variant X2, mRNA
ENSP00000309548-D1	-1.58	5.17E-20	↑	B. mutus pyruvate dehyrogenase phosphatase catalytic subunit 2 (PDP2), mRNA
ENSBTAP00000027504-D1	-1.58	7.64E-05	↑	B. mutus metalloproteinase inhibitor 3-like (LOC102277890), transcript variant X1, mRNA
ENSP00000309338-D1	-1.58	9.39E-04	↑	B. mutus cardiotrophin-like cytokine factor 1 (CLCF1), mRNA
ENSBTAP00000004504-D24	-1.58	9.39E-04	↑	B. mutus putative uncharacterized zinc finger protein 814-like (LOC102265916), mRNA
ENSP00000253303-D1	-1.58	1.19E-02	↑	B. mutus regucalcin (RGN), transcript variant X2, mRNA
ENSBTAP00000012705-D1	-1.58	1.19E-02	↑	B. mutus A kinase (PRKA) anchor protein 5 (AKAP5), mRNA
ENSBTAP00000022119-D1	-1.58	1.19E-02	↑	B. mutus mex-3 RNA binding family member B (MEX3B), partial mRNA
ENSP00000371376-D1	-1.58	1.19E-02	↑	B. mutus toll-like receptor 6 (TLR6), mRNA
ENSBTAP00000008613-D1	-1.58	1.19E-02	↑	B. mutus VANGL planar cell polarity protein 2 (VANGL2), mRNA
ENSP00000327650-D1	-1.58	7.71E-08	↑	B. mutus vacuolar protein sorting 33 homolog B (yeast) (VPS33B), transcript variant X1, mRNA
ENSP00000355963-D1	-1.58	9.30E-07	↑	B. mutus lysophosphatidylglycerol acyltransferase 1 (LPGAT1), mRNA
ENSP00000362207-D1	-1.57	2.03E-08	↑	B. mutus carbohydrate (chondroitin 6) sulfotransferase 3 (CHST3), mRNA
ENSP00000253108-D1	-1.57	2.91E-44	↑	B. taurus eukaryotic translation initiation factor 3, subunit G (EIF3G), mRNA
ENSP00000366702-D1	-1.57	3.68E-11	↑	B. mutus ERBB receptor feedback inhibitor 1 (ERRFI1), mRNA
ENSP00000363614-D1	-1.57	2.03E-05	↑	Capra hircus 3-hydroxymethyl-3-methylglutaryl-CoA lyase (HMGCL), transcript variant X1, mRNA
ENSP00000346398-D1	-1.57	1.70E-03	↑	B. mutus uncharacterized LOC102275418 (LOC102275418), mRNA
ENSBTAP00000023909-D1	-1.57	4.45E-10	↑	B. mutus carbonic anhydrase IV (CA4), mRNA
ENSBTAP00000051282-D1	-1.57	2.98E-06	↑	B. mutus magnesium-dependent phosphatase 1 (MDP1), transcript variant X1, mRNA
ENSBTAP00000000795-D1	-1.57	5.38E-09	↑	B. mutus junctional adhesion molecule 2 (JAM2), mRNA
ENSBTAP00000043279-D1	-1.57	2.47E-04	↑	Bubalus bubalis chromosome unknown open reading frame, human C19orf38, transcript variant X3, mRNA
ENSP00000312141-D1	-1.57	2.47E-04	↑	B. mutus zinc finger protein 654 (ZNF654), mRNA
ENSP00000336687-D4	-1.56	3.08E-14	↑	B. mutus chromobox homolog 3 (CBX3), mRNA
ENSBTAP00000020385-D1	-1.56	3.61E-05	↑	B. mutus tetraspanin 12 (TSPAN12), mRNA
ENSBTAP00000019763-D1	-1.56	5.33E-06	↑	B. mutus metaxin 1 (MTX1), mRNA
ENSP00000322016-D1	-1.56	1.03E-29	↑	B. mutus poly-U binding splicing factor 60KDa (PUF60), transcript variant X1, mRNA
ENSP00000231061-D1	-1.56	3.88E-18	↑	B. mutus secreted protein, acidic, cysteine-rich (osteonectin) (SPARC), mRNA
ENSBTAP00000011581-D2	-1.56	2.55E-09	↑	B. mutus SWI/SNF-related matrix-associated actin-dependent regulator of chromatin subfamily E member 1-related-like (LOC102268603), misc_RNA
ENSP00000392379-D1	-1.56	8.72E-30	↑	B. mutus alanyl-tRNA synthetase (AARS), mRNA
ENSBTAP00000003986-D1	-1.56	1.89E-41	↑	B. taurus PAX3 and PAX7 binding protein 1 (PAXBP1), mRNA
ENSBTAP00000053381-D1	-1.56	2.08E-07	↑	B. mutus KIAA0556 ortholog (KIAA0556), mRNA
ENSBTAP00000040758-D1	-1.56	6.46E-05	↑	Sus scrofa pro-neuregulin-2, membrane-bound isoform-like (LOC100626493), mRNA
ENSP00000355454-D1	-1.56	6.46E-05	↑	B. mutus zinc finger protein 496 (ZNF496), mRNA
ENSBTAP00000010277-D1	-1.56	6.46E-05	↑	B. mutus WW domain containing transcription regulator 1 (WWTR1), mRNA
ENSP00000420502-D17	-1.56	4.42E-04	↑	Bubalus bubalis olfactory receptor 2A2-like (LOC102401687), mRNA
ENSBTAP00000033956-D1	-1.56	3.06E-03	↑	B. mutus Fc fragment of IgE, high affinity I, receptor for; gamma polypeptide (FCER1G), mRNA
ENSBTAP00000022672-D1	-1.56	3.06E-03	↑	B. mutus olfactory receptor 51G1-like (LOC102268776), mRNA
ENSP00000367459-D1	-1.56	3.06E-03	↑	B. mutus KIAA0319 ortholog (KIAA0319), transcript variant X1, mRNA
ENSP00000334805-D1	-1.56	3.06E-03	↑	B. mutus testis-specific H1 histone-like (LOC102265640), mRNA
ENSBTAP00000034988-D1	-1.56	2.17E-02	↑	B. mutus gamma-glutamyltransferase 6 (GGT6), mRNA
ENSP00000398736-D1	-1.56	2.17E-02	↑	B. mutus cysteine-rich, angiogenic inducer, 61 (CYR61), mRNA
ENSP00000334113-D1	-1.56	2.17E-02	↑	B. mutus dehydrogenase/reductase SDR family member on chromosome X-like (LOC102285450), mRNA
ENSP00000303102-D1	-1.56	2.17E-02	↑	B. mutus zinc finger and BTB domain containing 46 (ZBTB46), mRNA
ENSBTAP00000043930-D1	-1.56	2.17E-02	↑	B. mutus DPY30 domain containing 2 (DYDC2), transcript variant X2, mRNA
ENSP00000359505-D1	-1.55	8.93E-29	↑	Bubalus bubalis chromosome unknown open reading frame, human C6orf57 (LOC102393034), mRNA
ENSP00000416650-D1	-1.55	4.93E-21	↑	B. mutus proteasome (prosome, macropain) activator subunit 2 (PA28 beta) (PSME2), mRNA
ENSBTAP00000030505-D3	-1.55	3.70E-07	↑	Bubalus bubalis kelch-like family member 7 (KLHL7), transcript variant X2, mRNA
ENSBTAP00000004892-D1	-1.55	1.25E-16	↑	B. mutus transketolase (TKT), transcript variant X1, mRNA
ENSBTAP00000021879-D1	-1.55	2.51E-06	↑	B. mutus CDC42 binding protein kinase beta (DMPK-like) (CDC42BPB), mRNA
ENSBTAP00000018910-D4	-1.55	2.52E-42	↑	B. mutus ribosomal protein L34 (RPL34), mRNA
ENSBTAP00000049526-D81	-1.55	1.71E-05	↑	B. taurus Y Chr BAC CH240-278G19 complete sequence
ENSP00000299001-D1	-1.55	1.71E-05	↑	B. mutus piwi-like RNA-mediated gene silencing 4 (PIWIL4), mRNA
ENSBTAP00000052537-D1	-1.55	1.16E-04	↑	B. mutus endothelin 3 (EDN3), partial mRNA

ENSP00000346927-D1	-1.55	1.16E-04	↑	B. mutus N-acetyltransferase 6 (GCN5-related) (NAT6), mRNA
ENSP00000311121-D1	-1.55	5.65E-10	↑	Capra hircus proteasome (prosome, macropain) subunit, alpha type, 8 (PSMA8), transcript variant X1, mRNA
ENSBTAP00000022381-D1	-1.55	2.95E-24	↑	B. mutus WD repeat domain 46 (WDR46), transcript variant X1, mRNA
ENSBTAP00000035964-D1	-1.54	7.90E-04	↑	B. mutus zinc finger, AN1-type domain 3 (ZFAND3), mRNA
ENSP00000385328-D1	-1.54	7.90E-04	↑	B. mutus ring finger protein 43 (RNF43), mRNA
ENSBTAP00000022457-D1	-1.54	1.18E-29	↑	B. mutus coiled-coil domain containing 101 (CCDC101), transcript variant X1, mRNA
ENSP00000372295-D1	-1.54	3.04E-05	↑	B. mutus gap junction protein, beta 2, 26kDa (GJB2), mRNA
ENSP00000276062-D1	-1.54	6.78E-104	↑	B. mutus NADH dehydrogenase 1 beta subcomplex, 11, 17.3kDa (NDUFB11), transcript variant X1, mRNA
ENSBTAP00000024658-D1	-1.54	5.88E-14	↑	B. mutus tripartite motif containing 38 (TRIM38), mRNA
ENSBTAP00000031777-D1	-1.54	1.18E-06	↑	B. mutus asteroid homolog 1 (Drosophila) (ASTE1), mRNA
ENSP00000334974-D1	-1.54	1.22E-08	↑	B. mutus chromosome unknown open reading frame, human C3orf70 (LOC102286219), mRNA
ENSBTAP00000018838-D1	-1.54	2.08E-04	↑	Vicugna pacos zinc finger and SCAN domain-containing protein 20-like (LOC102541682), mRNA
ENSBTAP00000042136-D2	-1.54	5.54E-03	↑	B. mutus spindlin family, member 2B (SPIN2B), transcript variant X1, mRNA
ENSP00000341165-D1	-1.54	5.54E-03	↑	Capra hircus zinc finger protein 772 (ZNF772), transcript variant X2, mRNA
ENSP00000333122-D1	-1.54	5.54E-03	↑	B. mutus nuclear receptor subfamily 4, group A, member 3 (NR4A3), mRNA
ENSBTAP00000006290-D1	-1.54	1.37E-51	↑	B. mutus ubiquitin-conjugating enzyme E2T (putative) (UBE2T), mRNA
ENSBTAP00000027627-D2	-1.54	1.34E-42	↑	Bubalus bubalis 40S ribosomal protein S15a-like (LOC102410001), transcript variant X2, mRNA
ENSBTAP00000016889-D1	-1.53	3.23E-09	↑	B. mutus receptor (chemosensory) transporter protein 1 (RTP1), mRNA
ENSP00000340662-D1	-1.53	1.28E-31	↑	B. mutus NOP16 nucleolar protein (NOP16), mRNA
ENSP00000331258-D1	-1.53	5.43E-05	↑	B. mutus protein O-mannose kinase-like (LOC102270881), mRNA
ENSP00000391637-D1	-1.53	5.73E-09	↑	B. mutus heme oxygenase (decycling) 2 (HMOX2), mRNA
ENSBTAP00000026470-D1	-1.53	1.71E-19	↑	B. mutus copper metabolism (Murr1) domain containing 1 (COMMD1), partial mRNA
ENSP00000203166-D1	-1.53	1.44E-05	↑	Bubalus bubalis HAUS augmin-like complex, subunit 5 (HAUS5), mRNA
ENSBTAP00000015255-D1	-1.53	1.42E-03	↑	B. mutus elongator acetyltransferase complex subunit 6 (ELP6), mRNA
ENSP00000309007-D1	-1.53	1.42E-03	↑	B. mutus THAP domain containing 6 (THAP6), partial mRNA
ENSBTAP00000007090-D1	-1.53	9.93E-07	↑	B. mutus glia maturation factor, gamma (GMFG), mRNA
ENSP00000221459-D1	-1.53	9.93E-07	↑	B. mutus lin-7 homolog B (C. elegans) (LIN7B), mRNA
ENSP00000402831-D1	-1.53	3.72E-04	↑	B. mutus cyclin M4 (CNNM4), mRNA
ENSBTAP00000008975-D1	-1.52	1.92E-12	↑	B. mutus chromosome unknown open reading frame, human C9orf85 (LOC102279221), mRNA
ENSBTAP00000040493-D1	-1.52	2.62E-07	↑	B. mutus solute carrier family 2 (facilitated glucose transporter), member 3 (SLC2A3), mRNA
ENSP00000384209-D1	-1.52	2.55E-05	↑	B. mutus grainyhead-like 1 (Drosophila) (GRHL1), transcript variant X1, mRNA
ENSP00000225430-D1	-1.52	2.17E-40	↑	B. mutus ribosomal protein L19 (RPL19), mRNA
ENSBTAP00000029167-D1	-1.52	3.35E-10	↑	B. taurus aarF domain containing kinase 3 (ADCK3), mRNA
ENSBTAP00000045674-D2	-1.52	1.18E-34	↑	B. mutus small EDRK-rich factor 1A (telomeric) (SERF1A), mRNA
ENSBTAP00000045061-D1	-1.52	2.33E-11	↑	B. mutus interferon, gamma-inducible protein 30 (IFI30), mRNA
ENSP00000380116-D1	-1.52	4.66E-07	↑	B. mutus chromosome unknown open reading frame, human C19orf55 (LOC102269601), mRNA
ENSBTAP00000014206-D1	-1.52	1.24E-07	↑	B. mutus KRI1 homolog (S. cerevisiae) (KRI1), mRNA
ENSBTAP00000000927-D1	-1.52	1.24E-07	↑	B. mutus integrin-linked kinase (ILK), transcript variant X3, mRNA
ENSP00000346112-D1	-1.52	1.26E-188	↑	B. mutus solute carrier family 12, member 6 (SLC12A6), transcript variant X1, mRNA
ENSP00000160827-D1	-1.52	7.43E-159	↑	B. mutus kinesin family member 22 (KIF22), mRNA
ENSP00000361891-D1	-1.51	9.38E-27	↑	B. mutus CAP, adenylate cyclase-associated protein 1 (yeast) (CAP1), transcript variant X2, mRNA
ENSBTAP00000028999-D1	-1.51	3.55E-13	↑	B. mutus zinc finger, DHHC-type containing 17 (ZDHHC17), mRNA
ENSBTAP00000001467-D1	-1.51	4.55E-05	↑	B. mutus solute carrier family 16 (monocarboxylate transporter), member 5 (SLC16A5), mRNA
ENSBTAP00000000436-D1	-1.51	1.75E-04	↑	B. mutus aldehyde dehydrogenase 16 family, member A1 (ALDH16A1), mRNA
ENSP00000228468-D1	-1.51	2.56E-03	↑	Physeter catodon acid-sensing (proton-gated) ion channel 1 (ASIC1), transcript variant X1, mRNA
ENSP00000379057-D1	-1.51	1.00E-02	↑	B. mutus mitochondrial elongation factor 2 (MIEF2), mRNA
ENSP00000289805-D1	-1.51	1.00E-02	↑	B. mutus spermatogenesis associated 2-like (SPATA2L), mRNA
ENSBTAP00000033255-D1	-1.51	1.00E-02	↑	B. mutus WD repeat domain 60 (WDR60), mRNA
ENSP00000403403-D1	-1.51	1.00E-02	↑	Bubalus bubalis papilin, proteoglycan-like sulfated glycoprotein (PAPLN), transcript variant X3, mRNA
ENSBTAP00000028688-D1	-1.51	4.11E-02	↑	B. mutus protein reprimo-like (LOC102282346), mRNA
ENSBTAP00000039071-D1	-1.51	4.11E-02	↑	B. mutus dual specificity phosphatase 22 (DUSP22), mRNA
ENSBTAP00000013854-D1	-1.51	4.11E-02	↑	B. mutus LRRN4 C-terminal like (LRRN4CL), mRNA
ENSBTAP00000031210-D1	-1.51	4.11E-02	↑	B. taurus sodium/glucose cotransporter 1-like (LOC531152), mRNA
ENSP00000312753-D1	-1.51	4.11E-02	↑	B. mutus family with sequence similarity 109, member B (FAM109B), mRNA
ENSP00000390787-D1	-1.51	4.11E-02	↑	B. mutus family with sequence similarity 170, member A (FAM170A), mRNA

ENSP00000355153-D1	-1.51	4.11E-02	↑	Sus scrofa p14ARF protein (P14ARF), mRNA
ENSP00000333537-D1	-1.51	4.11E-02	↑	B. mutus spalt-like transcription factor 2 (SALL2), mRNA
ENSBTAP00000050270-D1	-1.51	4.11E-02	↑	B. mutus DDB1- and CUL4-associated factor 12-like protein 2-like (LOC102282386), mRNA
ENSP00000216445-D1	-1.51	4.11E-02	↑	B. mutus chromosome unknown open reading frame, human C14orf105, transcript variant X1, mRNA
ENSBTAP00000004406-D1	-1.51	4.11E-02	↑	B. mutus potassium voltage-gated channel, subfamily G, member 1 (KCNG1), mRNA
ENSBTAP00000013313-D1	-1.51	4.11E-02	↑	Bubalus bubalis FANCD2 opposite strand (FANCD2OS), mRNA
ENSP00000309189-D1	-1.51	4.11E-02	↑	Bubalus bubalis spermatid associated (SPERT), mRNA
ENSBTAP00000010973-D1	-1.51	4.11E-02	↑	B. mutus ets variant 4 (ETV4), transcript variant X2, mRNA
ENSP00000370224-D1	-1.51	7.66E-25	↑	B. mutus prostate androgen-regulated mucin-like protein 1 (PARM1), partial mRNA
ENSBTAP00000010212-D1	-1.51	3.89E-241	↑	Bubalus bubalis cyclin-dependent kinase 5 (CDK5), transcript variant X1, mRNA
ENSP00000371462-D1	-1.50	1.64E-11	↑	B. mutus microtubule-associated protein 1A (MAP1A), mRNA
ENSP00000297632-D1	-1.50	5.65E-06	↑	B. mutus uncharacterized LOC102278395 (LOC102278395), mRNA
ENSBTAP00000003691-D1	-1.50	2.15E-05	↑	B. mutus TRAF3 interacting protein 3 (TRAF3IP3), mRNA
ENSBTAP00000006917-D1	-1.50	4.86E-08	↑	B. mutus peroxisomal biogenesis factor 13 (PEX13), mRNA
ENSP00000284878-D1	-1.50	8.12E-05	↑	B. mutus coxsackie virus and adenovirus receptor (CXADR), transcript variant X1, mRNA
ENSBTAP00000042269-D1	-1.50	1.84E-07	↑	B. mutus chromosome unknown open reading frame, human C1orf174 (LOC102271556), mRNA
ENSBTAP00000028319-D1	-1.50	1.84E-07	↑	B. mutus transmembrane protein 35 (TMEM35), mRNA
ENSP00000246117-D1	-1.50	3.11E-04	↑	B. mutus nicalin (NCLN), mRNA
ENSBTAP00000020029-D1	-1.50	7.37E-48	↑	B. mutus ECSIT signalling integrator (ECSIT), transcript variant X1, mRNA
ENSBTAP00000041860-D1	-1.50	0.00E+00	↑	B. mutus thioredoxin (TXN), transcript variant X1, mRNA
ENSBTAP00000013082-D2	-1.50	0.00E+00	↑	B. mutus aldo-keto reductase family 1, member B1 (aldose reductase) (AKR1B1), mRNA
ENSP00000307292-D1	-1.49	1.01E-05	↑	B. mutus protein kinase C, delta binding protein (PRKCDBP), mRNA
ENSP00000306410-D1	-1.49	1.19E-03	↑	B. mutus armadillo repeat containing 4 (ARMC4), mRNA
ENSP00000320176-D1	-1.49	3.81E-05	↑	B. mutus hematopoietic cell-specific Lyn substrate 1 (HCLS1), mRNA
ENSP00000412582-D2	-1.49	1.78E-51	↑	B. mutus DEAD (Asp-Glu-Ala-Asp) box polypeptide 39A (DDX39A), mRNA
ENSP00000409548-D1	-1.49	1.12E-44	↑	B. mutus solute carrier family 35, member B1 (SLC35B1), mRNA
ENSP00000379505-D1	-1.49	5.17E-20	↑	B. mutus SAMM50 sorting and assembly machinery component (SAMM50), mRNA
ENSP00000390160-D1	-1.49	1.54E-07	↑	B. mutus family with sequence similarity 3, member C (FAM3C), mRNA
ENSBTAP00000048820-D1	-1.49	4.72E-06	↑	B. mutus protocadherin-9-like (LOC102273237), misc_RNA
ENSP00000366549-D1	-1.49	4.65E-03	↑	B. mutus ST3 beta-galactoside alpha-2,3-sialyltransferase 5 (ST3GAL5), mRNA
ENSP00000263665-D1	-1.49	4.65E-03	↑	B. mutus contactin 3 (plasmacytoma associated) (CNTN3), mRNA
ENSP00000345229-D1	-1.49	4.65E-03	↑	B. mutus delta/notch-like EGF repeat containing (DNER), partial mRNA
ENSP00000344393-D2	-1.49	4.73E-68	↑	B. mutus FCF1 rRNA-processing protein (FCF1), mRNA
ENSBTAP00000013663-D3	-1.48	2.37E-18	↑	B. mutus moesin (MSN), mRNA
ENSBTAP00000025471-D2	-1.48	5.54E-04	↑	B. mutus serine/threonine-protein kinase Nek5-like (LOC102283783), partial mRNA
ENSBTAP00000041594-D1	-1.48	2.23E-36	↑	B. mutus transient receptor potential cation channel, subfamily M, member 7 (TRPM7), mRNA
ENSBTAP00000016009-D2	-1.48	1.11E-87	↑	B. mutus coiled-coil domain containing 167 (CCDC167), mRNA
ENSBTAP00000035894-D1	-1.48	6.80E-05	↑	Bubalus bubalis cyclin D3 (CCND3), transcript variant X3, mRNA
ENSP00000358727-D3	-1.48	6.80E-05	↑	B. mutus glutathione S-transferase omega 2 (GSTO2), mRNA
ENSBTAP00000015861-D1	-1.48	1.98E-18	↑	B. mutus mRNA turnover 4 homolog (S. cerevisiae) (MRTO4), mRNA
ENSP00000301396-D1	-1.48	1.04E-06	↑	B. mutus proline, glutamate and leucine rich protein 1 (PELP1), transcript variant X1, mRNA
ENSBTAP00000012908-D1	-1.48	1.49E-14	↑	B. mutus general transcription factor II-I repeat domain-containing protein 2-like (LOC102266843), mRNA
ENSP00000360163-D1	-1.48	2.79E-49	↑	B. mutus SWI/SNF related, matrix associated, actin dependent regulator of chromatin, subfamily a, member 1 (SMARCA1), transcript variant X2, mRNA
ENSBTAP00000017155-D1	-1.48	1.65E-18	↑	B. mutus heat shock protein 70kDa family, member 13 (HSPA13), mRNA
ENSP00000400644-D1	-1.47	3.18E-05	↑	Bubalus bubalis ADP-ribosylation factor 3 (ARF3), mRNA
ENSP00000416558-D1	-1.47	2.60E-04	↑	B. mutus F-box protein 24 (FBXO24), transcript variant X1, mRNA
ENSBTAP00000023779-D1	-1.47	2.14E-03	↑	B. mutus zinc finger, matrin-type 5 (ZMAT5), mRNA
ENSBTAP00000009103-D1	-1.47	2.14E-03	↑	Bubalus bubalis kinesin family member 7 (KIF7), transcript variant X3, mRNA
ENSP00000314151-D1	-1.47	2.14E-03	↑	B. mutus kallikrein 1 (KLK1), mRNA
ENSBTAP00000002176-D1	-1.47	2.14E-03	↑	B. mutus peptide deformylase (mitochondrial) (PDF), mRNA
ENSBTAP00000007444-D1	-1.47	1.80E-02	↑	B. mutus leucine rich repeat containing 20 (LRRC20), mRNA
ENSP00000414068-D1	-1.47	1.80E-02	↑	B. mutus pyrroline-5-carboxylate reductase 1 (PYCR1), transcript variant X2, mRNA
ENSP00000393854-D1	-1.47	1.80E-02	↑	B. mutus tigger transposable element-derived protein 1-like (LOC102276729), mRNA
ENSBTAP00000007333-D1	-1.47	1.80E-02	↑	B. mutus seizure threshold 2 homolog (mouse) (SZT2), mRNA

ENSBTAP00000018863-D2	-1.47	1.80E-02	↑	B. mutus catenin (cadherin-associated protein), alpha 2 (CTNNA2), transcript variant X5, mRNA
ENSP00000278385-D1	-1.47	1.89E-16	↑	B. taurus CD44 molecule (Indian blood group) (CD44), transcript variant X4, mRNA
ENSBTAP00000018959-D1	-1.47	1.50E-05	↑	B. mutus scavenger receptor class B, member 1 (SCARB1), mRNA
ENSBTAP00000007953-D1	-1.47	1.01E-21	↑	B. mutus acyl-CoA binding domain containing 4 (ACBD4), mRNA
ENSBTAP00000050534-D1	-1.47	1.22E-04	↑	B. mutus forkhead box I3 (FOXI3), partial mRNA
ENSBTAP00000015716-D1	-1.47	4.07E-140	↑	B. mutus eukaryotic translation elongation factor 1 gamma (EEF1G), mRNA
ENSBTAP00000038178-D2	-1.47	8.65E-07	↑	Panthera tigris altaica dynein, axonemal, heavy chain 12 (DNAH12), mRNA
ENSP00000325919-D1	-1.47	3.49E-25	↑	B. mutus proteasome (prosome, macropain) assembly chaperone 2 (PSMG2), mRNA
yakA09424	-1.47	9.90E-04	↑	B. mutus Ral GEF with PH domain and SH3 binding motif 1 (RALGPS1), mRNA
ENSP00000381607-D2	-1.46	5.50E-40	↑	B. mutus glutathione S-transferase P-like (LOC102272205), mRNA
ENSBTAP00000035742-D1	-1.46	1.22E-12	↑	B. mutus myelin expression factor 2 (MYEF2), mRNA
yakG020512	-1.46	5.65E-05	↑	Pantholops hodgsonii family with sequence similarity 96, member A (FAM96A), transcript variant X3, mRNA
ENSP00000221852-D1	-1.46	5.65E-05	↑	B. mutus Src homology 2 domain containing transforming protein D (SHD), mRNA
ENSBTAP00000019643-D1	-1.46	2.35E-54	↑	B. mutus CD9 molecule (CD9), mRNA
ENSP00000253047-D1	-1.46	1.11E-08	↑	B. mutus transmembrane protein 160 (TMEM160), mRNA
ENSBTAP00000004835-D1	-1.46	6.43E-10	↑	B. mutus Sjogren syndrome/scleroderma autoantigen 1 (SSSCA1), mRNA
ENSBTAP00000032801-D1	-1.46	4.15E-16	↑	B. mutus peroxisome proliferator-activated receptor gamma, coactivator-related 1 (PPRC1), mRNA
ENSP00000352956-D1	-1.46	1.76E-11	↑	B. mutus RNA binding motif protein 23 (RBM23), transcript variant X1, mRNA
ENSBTAP00000018459-D1	-1.46	1.54E-06	↑	B. mutus tripartite motif containing 21 (TRIM21), mRNA
ENSBTAP00000010721-D1	-1.46	2.65E-05	↑	B. mutus Sad1 and UNC84 domain containing 3 (SUN3), mRNA
ENSBTAP00000029616-D1	-1.46	2.65E-05	↑	B. taurus SH2B adaptor protein 3 (SH2B3), mRNA
ENSP00000412272-D1	-1.46	4.62E-04	↑	.
ENSBTAP00000012633-D1	-1.46	4.62E-04	↑	B. mutus STAM binding protein (STAMBP), transcript variant X2, mRNA
ENSBTAP00000030281-D1	-1.46	8.38E-03	↑	B. mutus transmembrane protein 218 (TMEM218), mRNA
ENSP00000216133-D1	-1.46	8.38E-03	↑	B. mutus chromobox homolog 7 (CBX7), mRNA
ENSP00000320510-D1	-1.46	8.38E-03	↑	B. mutus protein phosphatase 1, regulatory subunit 9B (PPP1R9B), mRNA
ENSBTAP00000024209-D1	-1.46	2.29E-31	↑	B. mutus NADH dehydrogenase (ubiquinone) 1, subcomplex unknown, 2, 14.5kDa (NDUFC2), mRNA
ENSBTAP00000050674-D1	-1.46	7.21E-07	↑	B. mutus solute carrier family 39 (zinc transporter), member 7 (SLC39A7), mRNA
ENSBTAP00000020482-D1	-1.46	1.62E-16	↑	B. mutus transglutaminase 7 (TGM7), mRNA
ENSBTAP00000005523-D1	-1.45	2.56E-114	↑	B. mutus retinoic acid receptor responder (tazarotene induced) 2 (RARRES2), mRNA
ENSP00000396960-D1	-1.45	9.10E-108	↑	B. mutus 26S proteasome complex subunit DSS1-like (LOC102268542), mRNA
ENSP00000229270-D1	-1.45	1.25E-29	↑	B. mutus triosephosphate isomerase 1 (TPI1), mRNA
ENSBTAP00000012400-D1	-1.45	2.17E-04	↑	B. mutus NIPA-like domain containing 1 (NIPAL1), mRNA
ENSBTAP00000045373-D8	-1.45	2.17E-04	↑	B. taurus ribosomal protein S6 (RPS6), mRNA
ENSBTAP00000018925-D1	-1.45	7.74E-94	↑	B. mutus ATPase, H+ transporting, lysosomal 31kDa, V1 subunit E1 (ATP6V1E1), transcript variant X1, mRNA
ENSBTAP00000044540-D1	-1.45	1.77E-27	↑	B. mutus fumarylacetoacetate hydrolase domain containing 1 (FAHD1), mRNA
ENSBTAP00000025691-D1	-1.45	6.31E-119	↑	B. mutus malate dehydrogenase 1, NAD (soluble) (MDH1), transcript variant X1, mRNA
ENSBTAP00000004272-D1	-1.45	7.46E-08	↑	B. mutus milk fat globule-EGF factor 8 protein (MFGE8), mRNA
ENSBTAP00000039689-D1	-1.45	2.73E-06	↑	Physeter catodon pleckstrin homology domain containing, family G member 3, transcript variant X2, mRNA
ENSBTAP00000050586-D1	-1.45	3.83E-03	↑	B. mutus metallo-beta-lactamase domain containing 1 (MBLAC1), mRNA
ENSP00000304077-D18	-1.45	3.83E-03	↑	B. mutus olfactory receptor 4F15-like (LOC102282982), mRNA
ENSP00000334294-D1	-1.45	3.83E-03	↑	B. mutus transcription factor-like 5 (basic helix-loop-helix) (TCFL5), partial mRNA
ENSP00000215909-D1	-1.45	4.81E-70	↑	Bubalus bubalis galectin-1-like (LOC102391045), mRNA
ENSP00000386759-D1	-1.44	1.99E-16	↑	B. mutus SET domain containing 2 (SETD2), mRNA
ENSBTAP00000024034-D1	-1.44	1.82E-92	↑	B. taurus RNA binding motif (RNP1, RRM) protein 3, mRNA (cDNA clone MGC:128854 IMAGE:8119702), complete cds
ENSP00000361418-D1	-1.44	4.85E-06	↑	B. mutus importin 13 (IPO13), mRNA
ENSP00000311827-D1	-1.44	6.15E-22	↑	B. mutus male-specific lethal 2 homolog (Drosophila) (MSL2), transcript variant X1, mRNA
ENSBTAP00000052443-D1	-1.44	3.24E-54	↑	B. mutus vacuole membrane protein 1 (VMP1), transcript variant X1, mRNA
ENSP00000363668-D1	-1.44	2.27E-06	↑	B. mutus death-domain associated protein (DAXX), mRNA
ENSBTAP00000022140-D1	-1.44	2.27E-06	↑	B. mutus aminopeptidase Q-like (LOC102273815), mRNA
ENSP00000397645-D1	-1.44	1.38E-23	↑	B. mutus U2 small nuclear RNA auxiliary factor 1-like 4 (U2AF1L4), transcript variant X1, mRNA
ENSBTAP00000050787-D1	-1.44	2.93E-16	↑	B. mutus chromosome unknown open reading frame, human C8orf59 (LOC102281394), mRNA
ENSBTAP00000000718-D1	-1.44	1.80E-11	↑	B. mutus kelch domain containing 10 (KLHDC10), mRNA
ENSP00000309934-D1	-1.44	1.09E-29	↑	B. mutus CTD small phosphatase 2 (CTDSP2), mRNA

ENSBTAP00000002327-D1	-1.44	1.06E-06	↑	B. mutus sirtuin 2 (SIRT2), mRNA
ENSP00000388446-D1	-1.44	1.80E-04	↑	Pantholops hodgsonii reelin (RELN), mRNA
ENSP00000259470-D1	-1.44	1.80E-04	↑	B. mutus cathepsin L1-like (LOC102269419), mRNA
ENSP00000362085-D1	-1.43	1.21E-25	↑	B. mutus chromosome unknown open reading frame, human C9orf16 (LOC102266060), mRNA
ENSBTAP00000052430-D14	-1.43	5.66E-26	↑	Bubalus bubalis 40S ribosomal protein S27-like (LOC102415253), mRNA
ENSP00000403265-D1	-1.43	3.61E-59	↑	B. mutus pyruvate kinase, muscle (PKM), transcript variant X5, mRNA
ENSP00000355924-D1	-1.43	2.72E-27	↑	B. mutus SET and MYND domain containing 2 (SMYD2), mRNA
ENSBTAP00000005590-D1	-1.43	1.26E-19	↑	B. mutus trafficking protein particle complex 5 (TRAPPC5), mRNA
ENSP00000403172-D3	-1.43	3.23E-256	↑	B. mutus ribosomal protein L6 (RPL6), mRNA
ENSBTAP00000033144-D2	-1.43	4.50E-17	↑	B. mutus thioredoxin 2 (TXN2), mRNA
ENSP00000402537-D1	-1.43	1.84E-05	↑	B. mutus myotubularin related protein 10 (MTMR10), mRNA
ENSBTAP00000044312-D1	-1.43	1.89E-06	↑	B. mutus family with sequence similarity 114, member A1 (FAM114A1), mRNA
ENSBTAP00000026776-D1	-1.43	2.36E-21	↑	Bubalus bubalis stathmin-like 2 (STMN2), transcript variant X1, mRNA
ENSBTAP00000004644-D2	-1.43	8.84E-07	↑	B. taurus DEAD (Asp-Glu-Ala-As) box polypeptide 19B (DDX19B), mRNA
ENSBTAP00000010982-D1	-1.43	1.37E-39	↑	B. mutus antioxidant 1 copper chaperone (ATOX1), mRNA
ENSBTAP00000011042-D1	-1.43	3.48E-29	↑	B. mutus HtrA serine peptidase 1 (HTRA1), partial mRNA
ENSBTAP00000005323-D1	-1.43	3.25E-166	↑	B. mutus isopentenyl-diphosphate delta isomerase 1 (IDI1), mRNA
ENSP00000311596-D9	-1.43	1.95E-07	↑	B. mutus uncharacterized LOC102287194 (LOC102287194), misc_RNA
ENSP00000283684-D1	-1.43	4.18E-21	↑	B. mutus ATPase, aminophospholipid transporter, class I, type 8B, member 1 (ATP8B1), mRNA
ENSP00000347919-D1	-1.43	2.93E-15	↑	B. mutus cytochrome c oxidase assembly factor 4 homolog, mitochondrial-like (LOC102283861), mRNA
ENSBTAP00000002179-D1	-1.42	4.65E-25	↑	B. mutus component of oligomeric golgi complex 8 (COG8), mRNA
ENSP00000361190-D1	-1.42	2.13E-41	↑	B. mutus mitochondrial ribosomal protein S18A (MRPS18A), transcript variant X2, mRNA
ENSBTAP00000053408-D4	-1.42	7.41E-21	↑	B. mutus myosin, heavy chain 2, skeletal muscle, adult (MYH2), mRNA
ENSP00000374171-D1	-1.42	1.05E-12	↑	Pantholops hodgsonii tetratricopeptide repeat, ankyrin repeat and coiled-coil containing 2 (TANC2), transcript variant X1, mRNA
ENSBTAP00000023395-D1	-1.42	8.29E-11	↑	B. mutus heparan sulfate 2-O-sulfotransferase 1 (HS2ST1), mRNA
ENSP00000326737-D1	-1.42	1.53E-05	↑	B. mutus tumor necrosis factor receptor superfamily, member 12A (TNFRSF12A), transcript variant X1, mRNA
ENSP00000286713-D1	-1.42	6.92E-05	↑	B. mutus stomatin (STOM), mRNA
ENSBTAP00000020094-D1	-1.42	1.50E-04	↑	Bubalus bubalis ATPase, H+ transporting, lysosomal 9kDa, V0 subunit e1 (ATP6V0E1), mRNA
ENSBTAP00000050194-D37	-1.42	3.19E-04	↑	B.taurus DNA sequence from clone CH240-487I9, complete sequence
ENSP00000366493-D1	-1.42	6.83E-04	↑	Bubalus bubalis luteinizing hormone beta polypeptide (LHB), transcript variant X1, mRNA
ENSP00000365678-D1	-1.42	6.83E-04	↑	B. mutus OTU domain containing 1 (OTUD1), mRNA
ENSBTAP00000015317-D1	-1.42	1.47E-03	↑	B. mutus 5', 3'-nucleotidase, cytosolic (NT5C), mRNA
ENSP00000253382-D1	-1.42	3.18E-03	↑	B. mutus acyl-CoA synthetase short-chain family member 2 (ACSS2), transcript variant X1, mRNA
ENSP00000400184-D1	-1.42	3.18E-03	↑	B. taurus chromosome 19 open reading frame, human C17orf89 (C19H17orf89), mRNA
ENSBTAP00000049790-D39	-1.42	6.88E-03	↑	B. taurus clone rp42-147e22, complete sequence
ENSBTAP00000017991-D1	-1.42	6.88E-03	↑	B. mutus dimethylarginine dimethylaminohydrolase 2 (DDAH2), mRNA
ENSBTAP00000020785-D1	-1.42	6.88E-03	↑	B. mutus tenascin N (TNN), mRNA
ENSBTAP00000016559-D1	-1.42	6.88E-03	↑	B. mutus TBC1 domain family, member 13 (TBC1D13), mRNA
ENSP00000324821-D1	-1.42	6.88E-03	↑	B. mutus chitinase domain containing 1 (CHID1), transcript variant X1, mRNA
ENSBTAP00000043307-D1	-1.42	1.51E-02	↑	B. mutus formin homology 2 domain containing 1 (FHOD1), mRNA
ENSBTAP00000014661-D1	-1.42	1.51E-02	↑	B. mutus melanocortin 2 receptor (adrenocorticotropic hormone) (MC2R), mRNA
ENSP00000375809-D1	-1.42	1.51E-02	↑	B. mutus excision repair cross-complementing rodent repair deficiency, complementation group 2, mRNA
ENSBTAP00000024749-D1	-1.42	3.35E-02	↑	B. taurus heat shock protein, alpha-crystallin-related, B6, mRNA, complete cds
ENSBTAP00000023106-D1	-1.42	3.35E-02	↑	B. mutus UDP-N-acetyl-alpha-D-galactosamine:polypeptide N-acetylgalactosaminyltransferase 8, mRNA
ENSBTAP00000016890-D1	-1.42	3.35E-02	↑	B. mutus serpin peptidase inhibitor, clade I (neuroserpin), member 1 (SERPINI1), transcript variant X2, mRNA
ENSP00000397718-D1	-1.42	3.35E-02	↑	B. mutus uncharacterized LOC102270894 (LOC102270894), mRNA
ENSBTAP00000015555-D1	-1.42	3.35E-02	↑	B. mutus RecQ protein-like 5 (RECQL5), transcript variant X1, mRNA
ENSP00000216487-D1	-1.42	3.35E-02	↑	B. mutus Ras and Rab interactor 3 (RIN3), partial mRNA
ENSBTAP00000040788-D1	-1.42	3.35E-02	↑	B. mutus solute carrier family 35, member A4 (SLC35A4), mRNA
ENSBTAP00000019112-D1	-1.42	3.35E-02	↑	B. mutus caspase recruitment domain family, member 6 (CARD6), mRNA
ENSP00000361048-D1	-1.42	3.35E-02	↑	B. mutus leucine rich adaptor protein 1 (LURAP1), mRNA
ENSBTAP00000052597-D1	-1.42	3.35E-02	↑	B. mutus rhabdoid tumor deletion region gene 1 (RTDR1), mRNA
ENSP00000407436-D3	-1.42	3.35E-02	↑	Bubalus bubalis ArfGAP with GTPase domain, ankyrin repeat and PH domain 3, transcript variant X1, mRNA
ENSBTAP00000024327-D1	-1.42	3.35E-02	↑	B. mutus spermatogenesis associated 5-like 1 (SPATA5L1), mRNA

ENSBTAP00000043403-D4	-1.42	3.35E-02	↑	B. mutus multidrug resistance-associated protein 4-like (LOC102268001), partial mRNA
ENSBTAP00000014796-D1	-1.42	8.88E-162	↑	B. mutus family with sequence similarity 162, member A (FAM162A), mRNA
ENSBTAP00000021613-D1	-1.41	1.59E-13	↑	B. mutus retinoblastoma-like 2 (p130) (RBL2), transcript variant X1, mRNA
ENSP00000398026-D1	-1.41	8.74E-43	↑	B. mutus hexosaminidase A (alpha polypeptide) (HEXA), transcript variant X1, mRNA
ENSP00000364536-D3	-1.41	8.30E-92	↑	Bubalus bubalis sodium channel, voltage gated, type VIII, alpha subunit (SCN8A), transcript variant X2, mRNA
ENSBTAP00000047796-D1	-1.41	3.22E-11	↑	B. mutus excision repair cross-complementing rodent repair deficiency, complementation group 8, mRNA
ENSBTAP00000005688-D1	-1.41	7.30E-20	↑	B. mutus mitochondrial ribosomal protein L21 (MRPL21), mRNA
ENSBTAP00000029147-D1	-1.41	6.88E-18	↑	B. mutus thioredoxin domain containing 17 (TXNDC17), mRNA
ENSP00000370883-D1	-1.41	2.82E-13	↑	Bubalus bubalis NODAL modulator 1 (NOMO1), mRNA
ENSBTAP00000000230-D1	-1.41	5.87E-19	↑	B. mutus tRNA methyltransferase 10 homolog A (S. cerevisiae) (TRMT10A), mRNA
ENSBTAP00000008363-D1	-1.41	1.07E-172	↑	B. mutus NHP2 ribonucleoprotein (NHP2), mRNA
ENSBTAP00000028983-D1	-1.41	8.34E-41	↑	B. mutus proteasome (prosome, macropain) 26S subunit, ATPase, 3 (PSMC3), mRNA
ENSP00000354688-D1	-1.41	5.71E-11	↑	B. mutus family with sequence similarity 45, member A (FAM45A), transcript variant X1, mRNA
ENSBTAP00000016108-D1	-1.41	2.77E-06	↑	B. mutus RAD9 homolog A (S. pombe) (RAD9A), transcript variant X1, mRNA
ENSBTAP00000041677-D1	-1.41	4.74E-18	↑	B. mutus chondroitin sulfate synthase 3 (CHSY3), mRNA
ENSP00000320147-D1	-1.41	1.07E-42	↑	B. taurus enhancer of zeste 2 polycomb repressive complex 2 subunit (EZH2), mRNA
ENSP00000334003-D1	-1.40	4.84E-12	↑	B. mutus inturned planar cell polarity protein (INTU), mRNA
ENSP00000258457-D1	-1.40	1.78E-40	↑	B. mutus chromosome unknown open reading frame, human C2orf49, transcript variant X1, mRNA
ENSP00000304923-D1	-1.40	3.66E-16	↑	B. mutus leucine rich repeat containing 28 (LRRC28), mRNA
ENSBTAP00000009273-D1	-1.40	5.03E-07	↑	Balaenoptera acutorostrata scammoni zinc finger homeobox 2 (ZFHX2), mRNA
ENSP00000396577-D1	-1.40	2.64E-04	↑	B. mutus WD repeat domain 37 (WDR37), mRNA
ENSP00000398476-D1	-1.40	3.24E-32	↑	B. mutus YTH domain family, member 3 (YTHDF3), transcript variant X1, mRNA
ENSBTAP00000011257-D1	-1.40	1.07E-06	↑	B. mutus microsomal glutathione S-transferase 1 (MGST1), mRNA
yakG015168	-1.40	5.64E-04	↑	Pantholops hodgsonii uncharacterized LOC102329963 (LOC102329963), mRNA
ENSBTAP00000011048-D1	-1.40	5.64E-04	↑	B. mutus amyloid beta (A4) precursor protein-binding, family A, member 3 (APBA3), mRNA
ENSBTAP00000001069-D1	-1.40	3.94E-25	↑	Bubalus bubalis cytochrome c oxidase assembly protein COX14-like, transcript variant X6, mRNA
ENSBTAP00000014217-D1	-1.40	1.39E-15	↑	B. mutus biogenesis of lysosome-related organelles complex-1 subunit 2-like (LOC102281125), mRNA
ENSBTAP00000006808-D1	-1.40	1.22E-03	↑	B. mutus ATPase, aminophospholipid transporter, class I, type 8B, member 2, transcript variant X1, mRNA
ENSP00000364324-D1	-1.39	2.23E-05	↑	B. mutus family with sequence similarity 120C (FAM120C), mRNA
ENSP00000306625-D1	-1.39	1.67E-08	↑	B. mutus alveolar soft part sarcoma chromosome region, candidate 1 (ASPSCR1), mRNA
ENSBTAP00000008193-D1	-1.39	2.62E-03	↑	B. mutus mannosidase, alpha, class 2B, member 1 (MAN2B1), transcript variant X1, mRNA
ENSBTAP00000000944-D1	-1.39	2.62E-03	↑	B. mutus ADAM metallopeptidase with thrombospondin type 1 motif, 1 (ADAMTS1), mRNA
ENSBTAP00000009158-D3	-1.39	1.26E-11	↑	B. mutus tubulin beta-5 chain-like (LOC102278004), transcript variant X2, mRNA
ENSBTAP00000030636-D1	-1.39	5.68E-03	↑	Bubalus bubalis T-cell leukemia translocation altered (TCTA), mRNA
ENSBTAP00000007726-D1	-1.39	5.68E-03	↑	B. mutus family with sequence similarity 83, member A (FAM83A), mRNA
ENSP00000358964-D1	-1.39	5.68E-03	↑	B. mutus chromosome unknown open reading frame, human C1orf194 (LOC102265216), mRNA
ENSBTAP00000017416-D3	-1.39	5.68E-03	↑	B. mutus aldolase B, fructose-bisphosphate (ALDOB), transcript variant X2, mRNA
ENSBTAP00000016103-D2	-1.39	5.68E-03	↑	B. mutus netrin 1 (NTN1), mRNA
ENSBTAP00000016106-D1	-1.39	5.68E-03	↑	B. mutus G protein-coupled receptor 128 (GPR128), mRNA
ENSP00000357801-D1	-1.39	5.68E-03	↑	B. mutus S100 calcium binding protein A10 (S100A10), mRNA
ENSP00000328169-D1	-1.39	5.68E-03	↑	B. mutus jagged 2 (JAG2), partial mRNA
ENSBTAP00000006787-D6	-1.39	5.68E-03	↑	B. taurus MHC class I heavy chain (BOLA), mRNA
ENSBTAP00000007083-D1	-1.39	5.68E-03	↑	B. mutus processing of precursor 5, ribonuclease P/MRP subunit (S. cerevisiae) (POP5), mRNA
ENSBTAP00000017856-D1	-1.38	2.49E-54	↑	B. mutus HIV-1 Tat interactive protein 2, 30kDa (HTATIP2), transcript variant X1, mRNA
ENSP00000286794-D2	-1.38	1.36E-14	↑	B. mutus N(alpha)-acetyltransferase 10, NatA catalytic subunit (NAA10), transcript variant X1, mRNA
ENSP00000379566-D1	-1.38	1.85E-05	↑	Pantholops hodgsonii coiled-coil alpha-helical rod protein 1 (CCHCR1), mRNA
ENSBTAP00000053247-D1	-1.38	6.30E-08	↑	B. mutus methionine sulfoxide reductase B3 (MSRB3), transcript variant X2, mRNA
ENSP00000417867-D1	-1.38	4.67E-04	↑	B. mutus TAF8 RNA polymerase II, TATA box binding protein (TBP)-associated factor, 43kDa (TAF8), transcript variant X2, mRNA
ENSBTAP00000053364-D1	-1.38	4.59E-10	↑	B. mutus zinc finger, SWIM-type containing 7 (ZSWIM7), mRNA
ENSBTAP00000014220-D1	-1.38	3.26E-18	↑	B. mutus clathrin, light chain B (CLTB), transcript variant X1, mRNA
ENSBTAP00000050430-D85	-1.38	2.03E-15	↑	B. taurus cDNA clone IMAGE:8414372
yakG024011	-1.38	7.78E-177	↑	B. mutus glutathione peroxidase 4 (GPX4), mRNA
ENSP00000368405-D1	-1.38	4.46E-09	↑	B. mutus GDP-D-glucose phosphorylase 1 (GDPGP1), mRNA
ENSP00000333125-D1	-1.38	1.00E-03	↑	B. taurus mediator complex subunit 12 (MED12), mRNA

ENSP00000386609-D1	-1.38	1.23E-02	↑	B. mutus WD repeat domain 81 (WDR81), transcript variant X1, mRNA
ENSP00000331691-D1	-1.38	1.23E-02	↑	B. mutus chromosome unknown open reading frame, human C12orf60 (LOC102288219), mRNA
ENSBTAP00000051767-D1	-1.38	1.23E-02	↑	B. mutus armadillo repeat containing, X-linked 1 (ARMCX1), mRNA
ENSP00000305403-D28	-1.38	1.23E-02	↑	B. mutus olfactory receptor 481-like (LOC102266893), mRNA
ENSP00000384718-D1	-1.38	1.23E-02	↑	B. mutus 40S ribosomal protein S25-like (LOC102282755), mRNA
ENSBTAP00000011265-D1	-1.37	1.26E-11	↑	B. mutus activating transcription factor 3 (ATF3), mRNA
ENSP00000224784-D2	-1.37	1.38E-15	↑	B. mutus actin, gamma 2, smooth muscle, enteric (ACTG2), transcript variant X2, mRNA
ENSBTAP00000042852-D1	-1.37	2.02E-08	↑	B. mutus uncharacterized LOC102271040 (LOC102271040), misc_RNA
ENSP00000384718-D9	-1.37	1.40E-193	↑	B. mutus ribosomal protein S25 (RPS25), mRNA
ENSBTAP00000011924-D1	-1.37	1.81E-04	↑	B. mutus matrix metallopeptidase 19 (MMP19), mRNA
ENSBTAP00000042334-D2	-1.37	3.69E-09	↑	B. mutus TAF13 RNA polymerase II, TATA box binding protein (TBP)-associated factor, 18kDa (TAF13), mRNA
ENSP00000374226-D1	-1.37	2.77E-06	↑	B. mutus patched domain containing 2 (PTCHD2), mRNA
ENSBTAP00000007228-D1	-1.37	4.04E-34	↑	B. mutus squalene epoxidase (SQLE), transcript variant X1, mRNA
ENSP00000391053-D1	-1.37	3.26E-05	↑	Bubalus bubalis V-set and immunoglobulin domain containing 1 (VSIG1), transcript variant X1, mRNA
ENSBTAP00000016955-D1	-1.37	2.16E-03	↑	Capra hircus kinesin light chain 4 (KLC4), mRNA
ENSP00000341610-D1	-1.37	2.16E-03	↑	B. mutus chromosome unknown open reading frame, human C15orf48 (LOC102273674), mRNA
ENSBTAP00000007349-D1	-1.37	3.39E-13	↑	B. mutus insulin-like growth factor binding protein 2, 36kDa (IGFBP2), partial mRNA
ENSBTAP00000024322-D1	-1.37	9.18E-08	↑	B. mutus Fas (TNFRSF6)-associated via death domain (FADD), mRNA
ENSBTAP00000001074-D1	-1.37	1.12E-14	↑	B. mutus DEAD (Asp-Glu-Ala-Asp) box polypeptide 49 (DDX49), mRNA
ENSBTAP00000023206-D1	-1.37	8.81E-20	↑	B. mutus EGF containing fibulin-like extracellular matrix protein 1 (EFEMP1), mRNA
ENSP00000387056-D1	-1.37	8.23E-32	↑	Bubalus bubalis methyltransferase like 5 (METTL5), transcript variant X1, mRNA
ENSBTAP00000037032-D1	-1.37	9.63E-31	↑	B. mutus zinc finger protein 3 (ZNF3), mRNA
ENSBTAP00000050921-D50	-1.37	3.87E-04	↑	B. taurus NOVECTOR CH240-248M14 complete sequence
ENSBTAP00000037975-D1	-1.37	3.87E-04	↑	B. mutus solute carrier family 19 (thiamine transporter), member 3 (SLC19A3), mRNA
ENSP00000353850-D3	-1.37	9.20E-46	↑	B. mutus integrin beta 1 binding protein 1 (ITGB1BP1), transcript variant X1, mRNA
ENSP00000037243-D5	-1.36	3.99E-26	↑	Bubalus bubalis GABA(A) receptor-associated protein (GABARAP), mRNA
ENSBTAP00000027619-D1	-1.36	5.97E-13	↑	Bubalus bubalis Rho guanine nucleotide exchange factor (GEF) 7 (ARHGEF7), transcript variant X7, mRNA
ENSP00000376110-D55	-1.36	2.13E-10	↑	B. mutus zinc finger protein 555 (ZNF555), mRNA
ENSBTAP00000019607-D1	-1.36	2.25E-34	↑	B. mutus NOP2 nucleolar protein (NOP2), mRNA
ENSBTAP00000023705-D1	-1.36	5.30E-16	↑	B. mutus RNA binding motif, single stranded interacting protein 2 (RBMS2), mRNA
ENSBTAP00000039864-D1	-1.36	1.51E-51	↑	B. mutus retinoic acid receptor responder (tazarotene induced) 1 (RARRES1), mRNA
ENSP00000306752-D1	-1.36	1.05E-12	↑	B. taurus bolA family member 2B (BOLA2B), transcript variant X2, mRNA
ENSBTAP00000022087-D1	-1.36	5.33E-09	↑	B. mutus uncharacterized LOC102278877 (LOC102278877), mRNA
ENSP00000265537-D1	-1.36	4.70E-03	↑	B. mutus leucyl-tRNA synthetase 2, mitochondrial (LARS2), partial mRNA
ENSP00000249887-D1	-1.36	2.72E-02	↑	B. mutus atypical chemokine receptor 4 (ACKR4), mRNA
ENSP00000330523-D1	-1.36	2.72E-02	↑	B. mutus collagen triple helix repeat containing 1 (CTHRC1), mRNA
ENSBTAP00000019045-D1	-1.36	2.72E-02	↑	B. mutus KIAA1467 ortholog (KIAA1467), mRNA
ENSP00000416922-D1	-1.36	2.72E-02	↑	B. mutus glutamyl-tRNA synthetase 2, mitochondrial (EARS2), mRNA
ENSBTAP00000003926-D1	-1.36	2.72E-02	↑	B. mutus methyl-CpG binding domain protein 3 (MBD3), mRNA
ENSBTAP00000024520-D1	-1.36	2.72E-02	↑	B. mutus gamma-secretase activating protein (GSAP), mRNA
ENSP00000327168-D1	-1.36	2.72E-02	↑	B. taurus proline rich 7 (synaptic) (PRR7), mRNA
ENSBTAP00000029107-D1	-1.36	2.72E-02	↑	B. taurus transcription elongation factor A (SII), 2 (TCEA2), mRNA
ENSP00000364550-D1	-1.36	2.07E-09	↑	B. mutus lysine (K)-specific demethylase 5C (KDM5C), transcript variant X2, mRNA
yakG017947	-1.36	3.42E-07	↑	B. mutus histone H2A.v-like (LOC102271296), mRNA
ENSBTAP00000050581-D2	-1.36	1.71E-31	↑	B. mutus phosphoribosyl pyrophosphate synthetase 1 (PRPS1), transcript variant X1, mRNA
ENSP00000367086-D1	-1.35	1.86E-12	↑	Bubalus bubalis acyl-CoA thioesterase 7 (ACOT7), transcript variant X2, mRNA
ENSP00000381304-D19	-1.35	7.15E-13	↑	B. mutus zinc finger protein 26 (ZNF26), mRNA
ENSP00000363680-D1	-1.35	7.30E-07	↑	B. mutus ectodysplasin A (EDA), transcript variant X1, mRNA
ENSBTAP00000028978-D1	-1.35	3.19E-04	↑	B. mutus UDP-N-acetyl-alpha-D-galactosamine:polypeptide N-acetylgalactosaminyltransferase 14, mRNA
ENSP00000399831-D1	-1.35	3.19E-04	↑	B. mutus nucleolar protein 3 (apoptosis repressor with CARD domain) (NOL3), mRNA
ENSP00000356655-D1	-1.35	9.38E-09	↑	B. mutus epithelial cell transforming sequence 2 oncogene-like (ECT2L), transcript variant X1, mRNA
ENSBTAP00000021469-D1	-1.35	2.78E-13	↑	B. mutus golgi-associated, gamma adaptin ear containing, ARF binding protein 3, transcript variant X1, mRNA
yakG044243	-1.35	2.13E-61	↑	B. mutus karyopherin alpha 5 (importin alpha 6) (KPNA5), transcript variant X2, mRNA
ENSBTAP00000051910-D1	-1.35	2.22E-05	↑	B.taurus DNA sequence from clone CH240-487I9, complete sequence
ENSBTAP00000016697-D1	-1.35	1.78E-03	↑	Bubalus bubalis UV-stimulated scaffold protein A (UVSSA), transcript variant X1, mRNA

ENSBTAP00000006356-D1	-1.35	1.78E-03	↑	B. mutus iroquois homeobox 5 (IRX5), mRNA
ENSP00000297596-D1	-1.35	1.78E-03	↑	B. mutus GTP binding protein overexpressed in skeletal muscle (GEM), mRNA
ENSP00000379680-D1	-1.35	1.78E-03	↑	B. mutus N-acetylgalactosaminidase, alpha- (NAGA), mRNA
ENSBTAP00000018862-D1	-1.35	7.87E-17	↑	B. mutus nuclear receptor coactivator 5 (NCOA5), transcript variant X1, mRNA
ENSP00000364699-D1	-1.35	3.92E-19	↑	B. mutus succinate dehydrogenase complex, subunit D, integral membrane protein, transcript variant X1, mRNA
ENSP00000363162-D1	-1.35	5.84E-36	↑	B. mutus ATPase, H+ transporting, lysosomal 13kDa, V1 subunit G1 (ATP6V1G1), mRNA
ENSP00000237536-D1	-1.35	4.86E-13	↑	B. mutus suppressor of glucose, autophagy associated 1 (SOGA1), mRNA
ENSBTAP00000047533-D1	-1.35	8.60E-06	↑	Bubalus bubalis transmembrane protein 175 (TMEM175), transcript variant X1, mRNA
ENSP00000364476-D1	-1.35	1.26E-27	↑	B. mutus RIB43A domain with coiled-coils 1 (RIBC1), mRNA
ENSP00000351619-D1	-1.34	3.32E-56	↑	B. mutus phenazine biosynthesis-like protein domain containing (PBLD), mRNA
ENSP00000340010-D1	-1.34	2.33E-07	↑	B. mutus insulin induced gene 1 (INSIG1), mRNA
ENSP00000358154-D7	-1.34	1.01E-02	↑	B. mutus histone H3.3-like (LOC102268079), mRNA
ENSBTAP00000022045-D1	-1.34	1.01E-02	↑	B. mutus integrin alpha FG-GAP repeat containing 3 (ITFG3), mRNA
ENSBTAP00000021482-D1	-1.34	5.96E-14	↑	B. mutus transmembrane protein 120A (TMEM120A), mRNA
ENSP00000391203-D2	-1.34	1.28E-06	↑	Bubalus bubalis uncharacterized LOC102404524 (LOC102404524), partial mRNA
ENSP00000262577-D1	-1.34	1.83E-05	↑	B. mutus zinc finger CCCH-type containing 3 (ZC3H3), mRNA
ENSP00000264977-D1	-1.34	7.08E-06	↑	B. mutus protein phosphatase 2, regulatory subunit B", alpha (PPP2R3A), transcript variant X1, mRNA
ENSBTAP00000043335-D1	-1.34	5.26E-09	↑	B. mutus density-regulated protein (DENR), mRNA
ENSBTAP00000002607-D3	-1.34	1.16E-33	↑	B. mutus family with sequence similarity 96, member A (FAM96A), transcript variant X1, mRNA
ENSP00000234816-D1	-1.34	7.46E-08	↑	B. mutus angiopoietin-like 1 (ANGPTL1), transcript variant X2, mRNA
ENSP00000410533-D1	-1.34	3.83E-03	↑	B. mutus ring finger protein 223 (RNF223), mRNA
ENSP00000408309-D1	-1.34	1.23E-19	↑	B. mutus lin-54 homolog (C. elegans) (LIN54), transcript variant X1, mRNA
ENSP00000408994-D1	-1.33	1.51E-05	↑	B. mutus de-etiolated homolog 1 (Arabidopsis) (DET1), mRNA
ENSBTAP00000010389-D1	-1.33	1.18E-68	↑	B. mutus fission 1 (mitochondrial outer membrane) homolog (S. cerevisiae) (FIS1), mRNA
ENSBTAP00000014520-D3	-1.33	1.60E-63	↑	B. mutus single-stranded DNA binding protein 1, mitochondrial (SSBP1), mRNA
ENSBTAP00000003272-D1	-1.33	5.59E-04	↑	B. mutus synaptotagmin V (SYT5), transcript variant X1, mRNA
ENSP00000408631-D1	-1.33	5.59E-04	↑	B. mutus cell death-inducing DFFA-like effector c (CIDEC), transcript variant X2, mRNA
ENSBTAP00000010077-D1	-1.33	4.24E-33	↑	B. mutus catenin, beta like 1 (CTNNBL1), mRNA
ENSBTAP00000006328-D2	-1.33	4.25E-43	↑	B. mutus polymerase (RNA) II (DNA directed) polypeptide F (POLR2F), mRNA
ENSP00000369757-D1	-1.33	1.47E-60	↑	B. mutus ribosomal protein S6 (RPS6), mRNA
ENSP00000385114-D1	-1.33	5.35E-10	↑	B. mutus ADP-ribose/CDP-alcohol diphosphatase, manganese-dependent (ADPRM), mRNA
ENSBTAP00000020296-D1	-1.33	2.16E-04	↑	B. mutus beta-galactosidase-like (LOC102285166), mRNA
ENSP00000395281-D1	-1.33	1.96E-08	↑	B. mutus chromosome unknown open reading frame, human C9orf116 (LOC102276682), mRNA
ENSP00000380679-D1	-1.32	6.60E-11	↑	B. mutus coiled-coil domain containing 40 (CCDC40), mRNA
ENSBTAP00000017844-D1	-1.32	1.85E-06	↑	B. mutus coiled-coil domain containing 51 (CCDC51), transcript variant X2, mRNA
ENSBTAP00000015221-D1	-1.32	1.88E-15	↑	B. mutus polymerase (DNA directed), alpha 2, accessory subunit (POLA2), transcript variant X1, mRNA
ENSBTAP00000041555-D1	-1.32	1.61E-08	↑	B. mutus peroxisome proliferator-activated receptor gamma (PPARG), transcript variant X1, mRNA
ENSBTAP00000012486-D1	-1.32	1.23E-41	↑	B. mutus nuclear RNA export factor 1 (NXF1), mRNA
ENSBTAP00000050442-D5	-1.32	0.00E+00	↑	B. mutus ribosomal protein S3A (RPS3A), mRNA
ENSP00000216252-D1	-1.32	1.67E-18	↑	B. mutus PHD finger protein 5A (PHF5A), mRNA
ENSP00000266719-D1	-1.32	6.83E-05	↑	B. mutus keratocan (KERA), mRNA
ENSBTAP00000025683-D2	-1.32	3.15E-03	↑	B. mutus protein kinase C and casein kinase substrate in neurons 1 (PACSIN1), mRNA
ENSBTAP00000000071-D1	-1.32	3.15E-03	↑	B. mutus flap structure-specific endonuclease 1 (FEN1), mRNA
yakG029197	-1.32	3.15E-03	↑	Lipotes vexillifer 40S ribosomal protein SA-like (LOC103075883), mRNA
ENSP00000370770-D1	-1.32	3.15E-03	↑	B. mutus chromosome unknown open reading frame, human C17orf107 (LOC102265750), mRNA
ENSP00000392188-D9	-1.32	3.15E-03	↑	B. taurus growth hormone receptor gene, complete cds
ENSP00000415471-D1	-1.32	8.36E-03	↑	B. mutus PR domain containing 15 (PRDM15), transcript variant X2, mRNA
ENSBTAP00000025341-D1	-1.32	8.36E-03	↑	B. mutus aquaporin 4 (AQP4), transcript variant X1, mRNA
ENSP00000252483-D1	-1.32	8.36E-03	↑	B. mutus poliovirus receptor-related 2 (herpesvirus entry mediator B) (PVRL2), mRNA
ENSBTAP00000027138-D1	-1.32	2.20E-02	↑	B. mutus solute carrier family 35, member F3 (SLC35F3), mRNA
ENSBTAP00000001550-D1	-1.32	2.20E-02	↑	B. mutus apoptosis-inducing factor, mitochondrion-associated, 2 (AIFM2), transcript variant X2, mRNA
ENSP00000223214-D1	-1.32	2.20E-02	↑	B. mutus secreted frizzled-related protein 4 (SFRP4), mRNA
ENSP00000281581-D1	-1.32	2.20E-02	↑	B. mutus coiled-coil domain containing 175 (CCDC175), mRNA
ENSP00000299853-D1	-1.32	2.20E-02	↑	B. mutus polymerase (RNA) III (DNA directed) polypeptide E (80kD) (POLR3E), transcript variant X1, mRNA
ENSBTAP00000045767-D2	-1.32	2.20E-02	↑	B. mutus melanoma-associated antigen B1-like (LOC102266865), mRNA

ENSP00000383234-D1	-1.32	2.20E-02	↑	B. mutus mitogen-activated protein kinase 4 (MAPK4), mRNA
ENSBTAP00000021683-D1	-1.32	2.20E-02	↑	B. mutus glycerol-3-phosphate dehydrogenase 1 (soluble) (GPD1), transcript variant X1, mRNA
ENSBTAP00000026370-D1	-1.32	4.14E-36	↑	B. mutus grainyhead-like 3 (Drosophila) (GRHL3), transcript variant X2, mRNA
ENSBTAP00000038763-D5	-1.32	8.19E-13	↑	B. mutus ribosomal protein L12 (RPL12), transcript variant X1, mRNA
ENSP00000368119-D1	-1.32	6.33E-10	↑	B. mutus galactose-1-phosphate uridylyltransferase (GALT), transcript variant X1, mRNA
ENSP00000361158-D1	-1.32	4.26E-09	↑	B. mutus testis-specific kinase 2 (TESK2), mRNA
ENSP00000373340-D1	-1.31	2.08E-30	↑	B. mutus bromodomain and PHD finger containing, 1 (BRPF1), mRNA
ENSBTAP00000039142-D1	-1.31	5.61E-05	↑	B. mutus chromosome unknown open reading frame, human C20orf196 (LOC102271125), mRNA
ENSP00000269886-D3	-1.31	5.61E-05	↑	B. mutus SH3-domain GRB2-like 3 (SH3GL3), transcript variant X1, mRNA
ENSBTAP00000017058-D1	-1.31	1.15E-20	↑	B. mutus retinol dehydrogenase 12 (all-trans/9-cis/11-cis) (RDH12), mRNA
ENSBTAP00000045413-D1	-1.31	1.66E-10	↑	B. mutus mitochondria-localized glutamic acid-rich protein (MGARP), mRNA
ENSBTAP00000014112-D1	-1.31	1.46E-04	↑	B. mutus gem (nuclear organelle) associated protein 7 (GEMIN7), mRNA
ENSBTAP00000000497-D1	-1.31	1.46E-04	↑	B. mutus zinc finger and BTB domain containing 18 (ZBTB18), mRNA
ENSP00000245503-D3	-1.31	3.80E-04	↑	B. mutus myosin, heavy chain 1, skeletal muscle, adult (MYH1), mRNA
ENSP00000385610-D1	-1.31	9.83E-04	↑	B. mutus mex-3 RNA binding family member C (MEX3C), mRNA
ENSBTAP00000011959-D1	-1.30	3.28E-07	↑	B. mutus zinc finger protein 572 (ZNF572), mRNA
ENSP00000314499-D1	-1.30	4.61E-05	↑	B. mutus cyclin G associated kinase (GAK), mRNA
ENSBTAP00000047910-D1	-1.30	2.58E-03	↑	B. mutus gonadotropin-releasing hormone II receptor-like (LOC102266467), mRNA
ENSBTAP00000024505-D1	-1.30	5.97E-29	↑	B. mutus proteasome (prosome, macropain) inhibitor subunit 1 (PI31) (PSMF1), mRNA
ENSP00000257899-D1	-1.30	1.20E-14	↑	B. mutus biogenesis of lysosomal organelles complex-1, subunit 1 (BLOC1S1), mRNA
ENSP00000363587-D1	-1.30	2.35E-82	↑	Bubalus bubalis sushi, von Willebrand factor type A, EGF and pentraxin domain containing 1 (SVEP1), mRNA
ENSP00000259939-D1	-1.30	5.65E-16	↑	B. mutus ring finger protein 144B (RNF144B), mRNA
ENSP00000256246-D1	-1.30	3.57E-11	↑	B. mutus testis expressed 15 (TEX15), mRNA
ENSBTAP00000040745-D1	-1.30	1.05E-07	↑	B. mutus Fc fragment of IgG, receptor, transporter, alpha (FCGRT), transcript variant X1, mRNA
ENSP00000389046-D34	-1.30	4.38E-12	↑	B. mutus olfactory receptor 2A2-like (LOC102284678), mRNA
ENSBTAP00000000201-D1	-1.30	3.13E-15	↑	B. mutus Sp3 transcription factor (SP3), mRNA
ENSP00000317985-D1	-1.30	4.15E-20	↑	B. taurus Rho-associated, coiled-coil containing protein kinase 2 (ROCK2), mRNA
ENSBTAP00000013665-D1	-1.30	6.79E-03	↑	B. mutus interleukin 1, alpha (IL1A), mRNA
ENSBTAP00000051780-D1	-1.30	6.79E-03	↑	B. mutus cytochrome c-like (LOC102277261), mRNA
ENSBTAP00000051069-D1	-1.30	6.79E-03	↑	B. mutus 60S ribosomal protein L35-like (LOC102267011), mRNA
ENSBTAP00000008733-D1	-1.30	3.33E-08	↑	B. mutus regulator of chromosome condensation (RCC1) and BTB (POZ) domain containing protein 2, mRNA
ENSP00000341538-D1	-1.30	1.37E-226	↑	B. mutus protein transport protein Sec61 subunit gamma-like (LOC102269051), transcript variant X2, mRNA
ENSBTAP00000025068-D1	-1.30	8.61E-08	↑	B. mutus D-amino-acid oxidase (DAO), mRNA
ENSP00000283357-D1	-1.30	1.80E-06	↑	B. mutus family with sequence similarity 81, member B (FAM81B), mRNA
ENSBTAP00000017481-D1	-1.30	2.22E-07	↑	B. mutus nipsnap homolog 1 (C. elegans) (NIPSNAP1), transcript variant X1, mRNA
ENSP00000265881-D1	-1.29	0.00E+00	↑	B. mutus RNA exonuclease 2 (REXO2), mRNA
ENSBTAP00000048266-D1	-1.29	4.66E-06	↑	B. mutus mitochondrial ribosomal protein L12 (MRPL12), mRNA
ENSBTAP00000050721-D43	-1.29	4.66E-06	↑	B. taurus BAC CH240-448P19 complete sequence
ENSP00000264263-D1	-1.29	8.24E-17	↑	B. mutus G elongation factor, mitochondrial 1 (GFM1), transcript variant X1, mRNA
ENSBTAP00000001507-D1	-1.29	2.98E-13	↑	B. mutus serrate RNA effector molecule homolog (Arabidopsis) (SRRT), transcript variant X1, mRNA
ENSBTAP00000000876-D1	-1.29	9.85E-05	↑	B. mutus GH3 domain containing (GHDC), transcript variant X1, mRNA
ENSBTAP00000020579-D1	-1.29	1.21E-05	↑	B. mutus CD2 (cytoplasmic tail) binding protein 2 (CD2BP2), mRNA
ENSP00000342307-D1	-1.29	3.11E-05	↑	B. mutus forkhead box M1 (FOXM1), transcript variant X1, mRNA
ENSP00000329452-D1	-1.29	2.11E-03	↑	B. mutus solute carrier family 25, member 21 (SLC25A21), transcript variant X1, mRNA
ENSBTAP00000001990-D1	-1.29	2.11E-03	↑	B. mutus ribosomal RNA adenine dimethylase domain containing 1 (RRNAD1), mRNA
ENSP00000413245-D1	-1.29	1.79E-02	↑	B. mutus family with sequence similarity 178, member B (FAM178B), mRNA
ENSP00000370531-D4	-1.29	1.79E-02	↑	B. mutus acyl-coenzyme A thioesterase 4-like (LOC102286093), mRNA
ENSBTAP00000030954-D1	-1.29	1.79E-02	↑	B. mutus nose resistant to fluoxetine protein 6-like (LOC102266072), partial mRNA
ENSBTAP00000009911-D1	-1.29	1.79E-02	↑	B. mutus neutrophil cytosolic factor 4, 40kDa (NCF4), mRNA
ENSP00000376037-D4	-1.29	1.79E-02	↑	B. mutus cytochrome c oxidase subunit 6B1-like (LOC102275847), mRNA
ENSP00000375952-D1	-1.29	1.79E-02	↑	Pantholops hodgsonii SPARC related modular calcium binding 2 (SMOC2), mRNA
ENSP00000284031-D1	-1.29	1.79E-02	↑	B. mutus dimethylarginine dimethylaminohydrolase 1 (DDAH1), transcript variant X3, mRNA
ENSBTAP00000047385-D4	-1.29	1.79E-02	↑	Bubalus bubalis multidrug resistance-associated protein 4-like (LOC102412565), transcript variant X1, mRNA
ENSP00000246229-D1	-1.29	1.79E-02	↑	B. mutus pleiomorphic adenoma gene-like 2 (PLAGL2), mRNA
ENSBTAP00000024318-D1	-1.29	2.81E-10	↑	B. mutus nuclear factor of activated T-cells, cytoplasmic, calcineurin-dependent 2 (NFATC2), mRNA

ENSBTAP00000001006-D1	-1.29	2.81E-10	↑	B. mutus transmembrane protein 206 (TMEM206), mRNA
ENSP00000341551-D1	-1.29	4.69E-19	↑	B. mutus SMAD family member 4 (SMAD4), mRNA
ENSBTAP00000005622-D1	-1.28	1.84E-21	↑	B. mutus DEAD (Asp-Glu-Ala-Asp) box polypeptide 28 (DDX28), mRNA
ENSP00000344223-D1	-1.28	2.56E-05	↑	B. mutus membrane-bound transcription factor peptidase, site 1 (MBTPS1), mRNA
ENSP00000410731-D1	-1.28	1.89E-10	↑	B. mutus uncharacterized LOC102273470 (LOC102273470), misc_RNA
ENSBTAP00000006040-D1	-1.28	5.36E-32	↑	B. mutus coiled-coil domain containing 124 (CCDC124), mRNA
ENSBTAP00000008580-D1	-1.28	7.46E-51	↑	B. mutus NSFL1 (p97) cofactor (p47) (NSFL1C), transcript variant X1, mRNA
ENSBTAP00000023476-D1	-1.28	4.65E-41	↑	Bubalus bubalis sex comb on midleg-like 1 (Drosophila) (SCML1), mRNA
ENSBTAP00000001081-D1	-1.28	1.25E-08	↑	B. mutus PR domain containing 1, with ZNF domain (PRDM1), mRNA
ENSBTAP00000036113-D1	-1.28	5.57E-03	↑	B. mutus latrophilin 1 (LPHN1), transcript variant X1, mRNA
ENSP00000283027-D1	-1.28	8.75E-229	↑	B. mutus nucleotide binding protein 1 (NUBP1), mRNA
ENSP00000221265-D1	-1.28	9.12E-58	↑	B. mutus Paf1, RNA polymerase II associated factor, homolog (S. cerevisiae) (PAF1), mRNA
ENSBTAP00000010191-D1	-1.28	1.57E-11	↑	B. mutus tonsoku-like, DNA repair protein (TONSL), mRNA
ENSBTAP00000053609-D1	-1.28	3.21E-08	↑	B. mutus nuclear receptor coactivator 7 (NCOA7), transcript variant X1, mRNA
ENSBTAP00000022675-D1	-1.28	1.30E-28	↑	B. mutus acid phosphatase 6, lysophosphatidic (ACP6), transcript variant X1, mRNA
ENSBTAP00000013659-D1	-1.28	5.53E-123	↑	B. mutus ALG11, alpha-1,2-mannosyltransferase (ALG11), mRNA
ENSP00000411115-D1	-1.28	1.23E-48	↑	Pantholops hodgsonii cytochrome c oxidase subunit 6C-like (LOC102321458), mRNA
ENSBTAP00000053539-D3	-1.28	1.94E-14	↑	B. mutus mannosidase, alpha, class 1C, member 1 (MAN1C1), mRNA
ENSP00000258772-D1	-1.28	5.00E-13	↑	B. mutus DEAD (Asp-Glu-Ala-Asp) box helicase 56 (DDX56), transcript variant X2, mRNA
ENSBTAP00000053695-D1	-1.28	1.73E-03	↑	B. mutus v-ski avian sarcoma viral oncogene homolog (SKI), partial mRNA
ENSP00000234923-D1	-1.28	1.73E-03	↑	B. mutus KIAA1324 ortholog (KIAA1324), transcript variant X1, mRNA
ENSBTAP00000015682-D1	-1.28	3.81E-22	↑	Bubalus bubalis transportin 2 (TNPO2), transcript variant X6, mRNA
ENSP00000261813-D1	-1.28	0.00E+00	↑	B. mutus prefoldin subunit 1 (PFDN1), mRNA
ENSP00000307541-D1	-1.28	6.68E-06	↑	B. mutus BCL6 corepressor-like 1 (BCORL1), mRNA
ENSBTAP00000012142-D1	-1.27	8.40E-09	↑	B. taurus translocase of outer mitochondrial membrane 40 homolog (yeast)-like (TOMM40L), mRNA
ENSP00000360860-D1	-1.27	3.22E-26	↑	B. mutus interferon-induced protein with tetratricopeptide repeats 5 (IFIT5), mRNA
ENSP00000341289-D3	-1.27	1.06E-12	↑	B. taurus tubulin, beta 4B class IVb (TUBB4B), mRNA
ENSBTAP00000008595-D1	-1.27	1.72E-04	↑	B. mutus rabaptin, RAB GTPase binding effector protein 2 (RABEP2), mRNA
ENSBTAP00000053454-D1	-1.27	1.73E-05	↑	B. mutus fibroblast growth factor 20 (FGF20), mRNA
ENSBTAP00000004233-D1	-1.27	3.57E-19	↑	B. mutus pelota homolog (Drosophila) (PELO), mRNA
ENSBTAP00000004611-D1	-1.27	2.24E-11	↑	B. mutus TATA box binding protein (TBP)-associated factor, RNA polymerase I, D, 41kDa (TAF1D), mRNA
ENSBTAP00000013862-D2	-1.27	7.27E-16	↑	B. mutus vacuolar protein sorting 4 homolog A (S. cerevisiae) (VPS4A), mRNA
ENSBTAP00000039412-D1	-1.27	1.80E-09	↑	B. mutus uncharacterized LOC102281069 (LOC102281069), mRNA
ENSP00000389365-D1	-1.27	1.53E-14	↑	B. mutus TAF4b RNA polymerase II, TATA box binding protein (TBP)-associated factor, 105kDa, mRNA
ENSP00000400142-D1	-1.27	4.83E-14	↑	B. mutus EWS RNA-binding protein 1 (EWSR1), mRNA
ENSP00000354579-D1	-1.27	4.45E-04	↑	B. mutus CKLF-like MARVEL transmembrane domain containing 3 (CMTM3), mRNA
ENSBTAP00000033821-D1	-1.27	4.57E-03	↑	B. mutus teashirt homolog 2-like (LOC102269390), mRNA
ENSP00000382708-D1	-1.27	4.57E-03	↑	B. mutus chromosome unknown open reading frame, human C5orf49 (LOC102273827), mRNA
ENSP00000267540-D1	-1.27	4.57E-03	↑	B. mutus stonin 2 (STON2), mRNA
ENSBTAP00000035161-D1	-1.27	1.48E-02	↑	B. mutus solute carrier family 46 (folate transporter), member 1 (SLC46A1), transcript variant X1, mRNA
ENSBTAP00000026930-D1	-1.27	1.48E-02	↑	B. mutus wingless-type MMTV integration site family, member 5A (WNT5A), mRNA
ENSP00000349364-D1	-1.27	1.34E-21	↑	Bubalus bubalis intermediate filament family orphan 1 (IFFO1), transcript variant X5, mRNA
ENSBTAP00000015397-D1	-1.26	8.58E-17	↑	B. mutus cell division cycle 14B (CDC14B), mRNA
ENSP00000381010-D1	-1.26	3.25E-91	↑	B. mutus PRKR interacting protein 1 (IL11 inducible) (PRKRIP1), mRNA
ENSBTAP00000047771-D1	-1.26	2.66E-14	↑	B. mutus olfactory receptor 5M9-like (LOC102268920), mRNA
ENSP00000351255-D1	-1.26	1.16E-05	↑	B. mutus signal transducer and activator of transcription 4 (STAT4), transcript variant X2, mRNA
ENSP00000291552-D2	-1.26	1.34E-27	↑	B. mutus U2 small nuclear RNA auxiliary factor 1 (U2AF1), transcript variant X1, mRNA
ENSP00000366755-D1	-1.26	3.65E-05	↑	B. mutus period circadian clock 3 (PER3), mRNA
ENSBTAP00000037577-D6	-1.26	0.00E+00	↑	B. mutus glyceraldehyde-3-phosphate dehydrogenase (GAPDH), mRNA
ENSBTAP00000008496-D1	-1.26	1.79E-14	↑	B. mutus polymerase (RNA) I polypeptide A, 194kDa (POLR1A), mRNA
ENSBTAP00000000576-D1	-1.26	1.77E-12	↑	B. mutus transmembrane protein 126A (TMEM126A), mRNA
ENSBTAP00000042915-D2	-1.26	9.53E-06	↑	Bubalus bubalis PET100 homolog (S. cerevisiae) (PET100), transcript variant X1, mRNA
ENSBTAP00000020870-D1	-1.26	1.16E-03	↑	Bubalus bubalis acrosin inhibitor 1-like (LOC102401452), transcript variant X3, mRNA
ENSBTAP00000009708-D1	-1.26	2.27E-31	↑	B. mutus cytohesin 3 (CYTH3), mRNA
ENSBTAP00000041770-D8	-1.26	2.99E-05	↑	B. mutus tubulin, beta 4A class IVa (TUBB4A), mRNA

ENSBTAP00000017556-D1	-1.26	7.85E-07	↑	B. mutus low density lipoprotein receptor-related protein 1 (LRP1), mRNA
ENSP00000258888-D1	-1.25	2.48E-06	↑	B. mutus alpha-kinase 3 (ALPK3), mRNA
ENSBTAP00000004093-D1	-1.25	4.82E-252	↑	B. mutus interferon alpha-inducible protein 27-like protein 2B-like (LOC102279357), mRNA
ENSP00000255175-D3	-1.25	1.14E-106	↑	B. mutus serine incorporator 1 (SERINC1), mRNA
ENSBTAP00000020613-D33	-1.25	2.99E-04	↑	B. mutus zinc finger protein 696 (ZNF696), mRNA
ENSBTAP00000006353-D1	-1.25	2.45E-05	↑	B. mutus homeobox D3 (HOXD3), mRNA
ENSBTAP00000051799-D1	-1.25	2.45E-05	↑	B. mutus uncharacterized LOC102271688 (LOC102271688), mRNA
ENSBTAP00000010854-D1	-1.25	9.45E-04	↑	B. mutus glypican 6 (GPC6), mRNA
ENSBTAP00000022253-D1	-1.25	1.20E-02	↑	B. taurus deltex 1, E3 ubiquitin ligase (DTX1), mRNA
ENSP00000316262-D1	-1.25	1.20E-02	↑	B. taurus chromosome 14 open reading frame, human C8orf82 (C14H8orf82), mRNA
ENSP00000404220-D1	-1.25	1.20E-02	↑	B. mutus coiled-coil domain containing 155 (CCDC155), mRNA
ENSP00000217455-D1	-1.25	1.20E-02	↑	B. mutus acyl-CoA thioesterase 8 (ACOT8), mRNA
ENSBTAP00000005097-D95	-1.25	1.20E-02	↑	B. taurus clone RP42-139G14, complete sequence
ENSBTAP00000026877-D1	-1.25	1.20E-02	↑	B. mutus arylsulfatase family, member K (ARSK), mRNA
ENSBTAP00000024108-D1	-1.25	6.53E-23	↑	B. mutus activin A receptor, type IIA (ACVR2A), transcript variant X2, mRNA
ENSBTAP00000009988-D1	-1.25	1.88E-44	↑	B. mutus CWF19-like 1, cell cycle control (S. pombe) (CWF19L1), transcript variant X1, mRNA
ENSP00000294168-D1	-1.25	2.45E-04	↑	B. mutus TAF6-like RNA polymerase II, p300/CBP-associated factor (PCAF)-associated factor, 65kDa (TAF6L), transcript variant X2, mRNA
ENSBTAP00000005789-D1	-1.25	2.00E-32	↑	B. mutus nudC nuclear distribution protein (NUDC), transcript variant X1, mRNA
ENSBTAP00000021140-D1	-1.24	5.24E-06	↑	Bubalus bubalis zinc finger protein 354C (ZNF354C), mRNA
ENSBTAP00000004044-D1	-1.24	4.56E-123	↑	B. mutus integral membrane protein 2B-like (LOC102280075), mRNA
ENSBTAP00000015917-D1	-1.24	6.33E-05	↑	B. taurus zinc finger, MIZ-type containing 2 (ZMIZ2), mRNA
ENSP00000350989-D1	-1.24	6.33E-05	↑	B. mutus thioredoxin domain containing 5 (endoplasmic reticulum) (TXNDC5), mRNA
ENSP00000345001-D1	-1.24	3.03E-03	↑	B. mutus CREB regulated transcription coactivator 1 (CRTC1), mRNA
ENSP00000325398-D1	-1.24	8.98E-88	↑	B. mutus TP53RK binding protein (TPRKB), transcript variant X2, mRNA
ENSP00000373347-D1	-1.24	3.51E-06	↑	B. mutus SLIT-ROBO Rho GTPase activating protein 3 (SRGAP3), mRNA
ENSP00000362413-D2	-1.24	2.19E-48	↑	B. mutus phosphoglycerate kinase 1 (PGK1), mRNA
ENSBTAP00000053453-D1	-1.24	1.55E-19	↑	B. mutus Meis homeobox 2 (MEIS2), transcript variant X1, mRNA
ENSP00000280886-D1	-1.23	6.33E-04	↑	B. mutus DIP2 disco-interacting protein 2 homolog C (Drosophila) (DIP2C), mRNA
ENSBTAP00000022546-D1	-1.23	6.33E-04	↑	B. mutus beta-1,3-glucuronyltransferase 3 (glucuronosyltransferase I) (B3GAT3), mRNA
ENSBTAP00000045918-D1	-1.23	6.33E-04	↑	B. mutus nucleosome assembly protein 1-like 3 (NAP1L3), mRNA
ENSBTAP00000041529-D2	-1.23	2.48E-03	↑	B. mutus EP300 interacting inhibitor of differentiation 1 (EID1), mRNA
ENSP00000246794-D1	-1.23	9.78E-03	↑	B. mutus proline rich Gla (G-carboxyglutamic acid) 2 (PRRG2), mRNA
ENSP00000356140-D2	-1.23	9.78E-03	↑	B. mutus neuronal cell adhesion molecule (NRCAM), transcript variant X5, mRNA
ENSP00000244799-D1	-1.23	4.05E-02	↑	B. mutus opsin 5 (OPN5), mRNA
ENSP00000363832-D1	-1.23	4.05E-02	↑	B. mutus aldehyde oxidase-like (LOC102272565), mRNA
ENSP00000413635-D1	-1.23	4.05E-02	↑	B. mutus solute carrier family 43, member 3 (SLC43A3), mRNA
ENSBTAP00000017753-D1	-1.23	4.05E-02	↑	B. mutus SIX homeobox 5 (SIX5), partial mRNA
ENSP00000358789-D1	-1.23	4.05E-02	↑	Bubalus bubalis SH3 and PX domains 2A (SH3PXD2A), transcript variant X1, mRNA
ENSP00000361993-D2	-1.23	4.05E-02	↑	B. mutus mitochondrial import inner membrane translocase subunit Tim8 A-like (LOC102287497), mRNA
ENSP00000335185-D1	-1.23	4.05E-02	↑	B. mutus prickle homolog 4 (Drosophila) (PRICKLE4), mRNA
ENSP00000393066-D1	-1.23	4.05E-02	↑	B. taurus solute carrier family 4, sodium bicarbonate transporter, member 10 (SLC4A10), mRNA
ENSBTAP00000001300-D1	-1.23	1.92E-10	↑	B. mutus chromosome unknown open reading frame, human C3orf84 (LOC102278960), mRNA
ENSP00000303252-D1	-1.23	4.68E-20	↑	B. mutus zinc finger protein 804A (ZNF804A), mRNA
ENSP00000403117-D1	-1.23	1.21E-95	↑	B. mutus major facilitator superfamily domain-containing protein 1-like, transcript variant X2, mRNA
ENSBTAP00000015375-D1	-1.23	1.34E-04	↑	B. mutus ectonucleotide pyrophosphatase/phosphodiesterase 6 (ENPP6), mRNA
ENSP00000360431-D1	-1.23	1.34E-04	↑	B. mutus phospholipase C, epsilon 1 (PLCE1), mRNA
ENSP00000246077-D1	-1.23	1.91E-09	↑	B. mutus TBC1 domain family, member 20 (TBC1D20), mRNA
ENSP00000302913-D1	-1.23	7.29E-09	↑	B. mutus SH3 domain containing 19 (SH3D19), transcript variant X1, mRNA
ENSP00000344041-D1	-1.23	7.41E-06	↑	B. mutus synaptogyrin 4 (SYNGR4), mRNA
ENSBTAP00000024595-D1	-1.23	7.41E-06	↑	B. mutus kelch repeat and BTB (POZ) domain containing 4 (KBTBD4), transcript variant X2, mRNA
ENSP00000254663-D1	-1.23	4.10E-07	↑	B. mutus selenocysteine lyase (SCLY), mRNA
ENSP00000293805-D1	-1.23	4.10E-07	↑	B. mutus B-cell CLL/lymphoma 6, member B (BCL6B), mRNA
ENSBTAP00000032868-D1	-1.23	2.85E-05	↑	B. mutus EFR3 homolog B (S. cerevisiae) (EFR3B), mRNA
ENSP00000281950-D1	-1.23	1.46E-33	↑	B. mutus gem (nuclear organelle) associated protein 6 (GEMIN6), transcript variant X2, mRNA

ENSP00000337393-D1	-1.22	1.80E-66	↑	B. mutus ubiquitously-expressed, prefoldin-like chaperone (UXT), mRNA
ENSP00000412582-D1	-1.22	9.95E-68	↑	B. mutus DEAD (Asp-Glu-Ala-Asp) box polypeptide 39B (DDX39B), transcript variant X2, mRNA
ENSP00000218432-D1	-1.22	1.85E-11	↑	B. mutus protein (peptidylprolyl cis/trans isomerase) NIMA-interacting, 4 (parvulin) (PIN4), mRNA
ENSP00000300026-D1	-1.22	5.00E-48	↑	B. mutus peptidylprolyl isomerase B (cyclophilin B) (PPIB), mRNA
ENSP00000393527-D1	-1.22	1.80E-52	↑	B. mutus P450 (cytochrome) oxidoreductase (POR), mRNA
ENSP00000293677-D1	-1.22	4.24E-04	↑	B. mutus ribonucleoprotein, PTB-binding 1 (RAVER1), transcript variant X1, mRNA
ENSP00000262241-D1	-1.22	4.24E-04	↑	B. mutus REST corepressor 1 (RCOR1), partial mRNA
ENSP00000370013-D1	-1.22	1.46E-13	↑	B. mutus pentatricopeptide repeat domain 2 (PTCD2), mRNA
ENSBTAP00000020289-D1	-1.22	8.55E-10	↑	B. mutus cytochrome c oxidase assembly protein COX19-like (LOC102265679), mRNA
ENSP00000333664-D1	-1.22	1.54E-08	↑	B. mutus acetyl-CoA acyltransferase 1 (ACAA1), transcript variant X1, mRNA
ENSBTAP00000025044-D1	-1.22	1.06E-06	↑	B. mutus isocitrate dehydrogenase 3 (NAD+) beta (IDH3B), transcript variant X3, misc_RNA
ENSP00000332530-D1	-1.22	2.26E-07	↑	B. mutus renalase, FAD-dependent amine oxidase (RNLS), mRNA
ENSP00000392341-D1	-1.22	2.26E-07	↑	B. taurus ERGIC and golgi 3, mRNA (cDNA clone MGC:127511 IMAGE:7951207), complete cds
ENSBTAP00000001970-D1	-1.22	1.65E-03	↑	B. mutus G protein-coupled receptor kinase 4 (GRK4), mRNA
ENSBTAP00000019665-D1	-1.21	6.89E-09	↑	B. mutus 5'-nucleotidase domain containing 2 (NT5DC2), mRNA
ENSP00000410452-D1	-1.21	4.45E-153	↑	Bubalus bubalis stathmin 1 (STMN1), mRNA
ENSBTAP00000003267-D1	-1.21	1.35E-03	↑	Balaenoptera acutorostrata scammoni dynein, axonemal, assembly factor 3, transcript variant X1, mRNA
ENSP00000317333-D1	-1.21	1.35E-03	↑	B. mutus neurogenin 2 (NEUROG2), mRNA
ENSP00000301067-D1	-1.21	1.35E-03	↑	B. mutus lysine (K)-specific methyltransferase 2D (KMT2D), mRNA
ENSBTAP00000009222-D1	-1.21	1.35E-03	↑	B. mutus MOK protein kinase (MOK), mRNA
ENSBTAP00000009823-D1	-1.21	6.49E-03	↑	B. mutus gremlin 1, DAN family BMP antagonist (GREM1), transcript variant X1, mRNA
ENSBTAP00000051692-D9	-1.21	3.24E-02	↑	.
ENSBTAP00000020239-D1	-1.21	3.24E-02	↑	B. mutus interferon (alpha, beta and omega) receptor 2 (IFNAR2), mRNA
ENSP00000219660-D1	-1.21	3.24E-02	↑	B. mutus aquaporin 8 (AQP8), mRNA
ENSP00000401831-D1	-1.21	3.24E-02	↑	B. mutus acyl-CoA synthetase family member 2 (ACSF2), transcript variant X1, mRNA
yakA08541	-1.21	3.24E-02	↑	B. mutus Ellis van Creveld syndrome (EVC), mRNA
ENSP00000355089-D1	-1.21	3.24E-02	↑	B. mutus CUGBP, Elav-like family member 4 (CELF4), transcript variant X1, mRNA
ENSBTAP00000009187-D1	-1.21	3.24E-02	↑	B. mutus myelin regulatory factor (MYRF), mRNA
ENSBTAP00000021694-D1	-1.21	3.24E-02	↑	B. mutus ATPase type 13A4 (ATP13A4), mRNA
ENSP00000287152-D1	-1.21	3.24E-02	↑	B. mutus kinesin family member 6 (KIF6), mRNA
ENSBTAP00000025304-D1	-1.21	1.27E-69	↑	B. mutus eukaryotic translation initiation factor 2B, subunit 3 gamma, 58kDa (EIF2B3), mRNA
ENSP00000395021-D1	-1.21	4.90E-05	↑	B. mutus cytochrome c oxidase subunit 7A1, mitochondrial-like (LOC102282043), mRNA
ENSP00000265343-D1	-1.21	8.30E-08	↑	B. taurus AF4/FMR2 family, member 4 (AFF4), mRNA
ENSBTAP00000028079-D1	-1.21	1.84E-108	↑	B. mutus RecQ protein-like (DNA helicase Q1-like) (RECQL), mRNA
ENSP00000377385-D1	-1.21	2.23E-132	↑	Balaenoptera acutorostrata scammoni purinergic receptor P2Y, G-protein coupled, 11, transcript variant X1, mRNA
ENSP00000367024-D1	-1.21	8.56E-06	↑	B. taurus N(alpha)-acetyltransferase 40, NatD catalytic subunit (NAA40), mRNA
ENSBTAP00000004834-D1	-1.21	1.10E-03	↑	B. mutus family with sequence similarity 89, member B (FAM89B), mRNA
ENSP00000264027-D1	-1.21	1.73E-18	↑	B. mutus sterol-C5-desaturase (SC5D), transcript variant X1, mRNA
ENSP00000323858-D1	-1.20	3.49E-12	↑	B. mutus DEAD (Asp-Glu-Ala-Asp) box polypeptide 54 (DDX54), transcript variant X1, mRNA
ENSP00000324511-D1	-1.20	2.89E-122	↑	B. mutus septin 1 (SEPT1), mRNA
ENSBTAP00000050082-D1	-1.20	2.60E-07	↑	B. mutus NADH dehydrogenase [ubiquinone] 1 alpha subcomplex subunit 10, mitochondrial-like (LOC102271415), transcript variant X1, mRNA
ENSP00000405068-D1	-1.20	2.60E-07	↑	B. mutus NOP14 nucleolar protein (NOP14), mRNA
yakA12524	-1.20	5.28E-03	↑	B. mutus F-box protein 6 (FBXO6), mRNA
ENSP00000410059-D1	-1.20	5.01E-21	↑	B. mutus eukaryotic translation elongation factor 1 delta EEF1D, transcript variant X2, mRNA
ENSP00000324173-D1	-1.20	5.25E-261	↑	B. mutus heat shock 70kDa protein 5 (glucose-regulated protein, 78kDa) (HSPA5), mRNA
ENSP00000412574-D2	-1.20	5.73E-06	↑	B. mutus torsin family 1, member B (torsin B) (TOR1B), mRNA
ENSBTAP00000007841-D1	-1.20	8.94E-04	↑	B. mutus hydroxysteroid (17-beta) dehydrogenase 7 (HSD17B7), mRNA
ENSBTAP00000029531-D1	-1.20	7.35E-12	↑	B. mutus solute carrier family 8 (sodium/lithium/calcium exchanger), member B1 (SLC8B1), mRNA
ENSBTAP00000015026-D1	-1.20	1.50E-21	↑	B. mutus coiled-coil domain containing 12 (CCDC12), mRNA
ENSP00000370724-D1	-1.20	2.68E-05	↑	B. mutus family with sequence similarity 49, member A (FAM49A), mRNA
ENSBTAP00000007973-D1	-1.20	8.14E-07	↑	B. mutus coiled-coil and C2 domain containing 1A (CC2D1A), mRNA
ENSP00000270281-D1	-1.20	1.43E-07	↑	B. mutus cleft lip and palate associated transmembrane protein 1 (CLPTM1), transcript variant X2, mRNA
ENSBTAP00000006331-D1	-1.20	8.64E-34	↑	B. mutus mitochondrial ribosomal protein L35 (MRPL35), mRNA
ENSBTAP00000028928-D1	-1.20	3.68E-15	↑	Bubalus bubalis ubiquitin-fold modifier conjugating enzyme 1 (UFC1), transcript variant X2, mRNA

ENSP00000400899-D1	-1.20	3.82E-06	↑	B. mutus leucine rich repeat containing 9 (LRRC9), mRNA
ENSP00000246747-D1	-1.20	1.26E-04	↑	B. mutus ADP-ribosylation factor-like 2 (ARL2), transcript variant X1, mRNA
ENSBTAP00000005747-D1	-1.20	7.31E-04	↑	B. mutus ethylmalonic encephalopathy 1 (ETHE1), mRNA
ENSP00000356218-D1	-1.20	4.32E-03	↑	B. mutus NADH-cytochrome b5 reductase 1-like (LOC102275956), mRNA
ENSBTAP00000050234-D1	-1.20	2.60E-02	↑	B. mutus adenylate kinase 1 (AK1), transcript variant X1, mRNA
ENSP00000378865-D1	-1.20	2.60E-02	↑	B. mutus receptor accessory protein 6 (REEP6), mRNA
ENSP00000393854-D3	-1.20	2.60E-02	↑	B. taurus tigger transposable element-derived protein 1-like (LOC101903649), transcript variant X14, mRNA
ENSP00000310241-D3	-1.20	2.60E-02	↑	B. mutus solute carrier family 22, member 20 (SLC22A20), mRNA
ENSBTAP00000000683-D1	-1.19	0.00E+00	↑	B. mutus cystatin B (stefin B) (CSTB), mRNA
ENSBTAP00000004852-D1	-1.19	2.92E-09	↑	B. mutus myotubularin related protein 9 (MTMR9), mRNA
ENSP00000257336-D1	-1.19	3.82E-13	↑	B. mutus basic, immunoglobulin-like variable motif containing (BIVM), mRNA
ENSBTAP00000000639-D1	-1.19	3.13E-06	↑	B. mutus kelch-like protein 13-like (LOC102281723), transcript variant X2, mRNA
ENSBTAP00000045723-D1	-1.19	4.16E-10	↑	B. mutus bromodomain containing 9 (BRD9), mRNA
ENSBTAP00000041338-D1	-1.19	2.27E-30	↑	B. mutus transmembrane protein 176B (TMEM176B), transcript variant X1, mRNA
ENSBTAP00000006928-D1	-1.19	1.03E-04	↑	B. mutus kelch-like family member 2 (KLHL2), transcript variant X3, mRNA
ENSBTAP00000006428-D1	-1.19	1.47E-12	↑	B. mutus solute carrier family 10, member 4 (SLC10A4), mRNA
ENSBTAP00000007584-D1	-1.19	5.97E-04	↑	B. mutus nephronophthisis 3 (adolescent) (NPHP3), mRNA
ENSP00000265838-D1	-1.19	9.67E-32	↑	B. mutus acetyl-CoA acetyltransferase 1 (ACAT1), mRNA
yakG026461	-1.19	9.91E-25	↑	B. mutus HAUS augmin-like complex, subunit 8 (HAUS8), mRNA
ENSP00000286298-D1	-1.19	3.51E-03	↑	B. mutus solute carrier family 26 (anion exchanger), member 2 (SLC26A2), mRNA
ENSBTAP00000004579-D1	-1.19	3.51E-03	↑	B. mutus KIAA0895-like ortholog (KIAA0895L), mRNA
ENSBTAP00000023798-D1	-1.19	2.84E-48	↑	B. mutus WD repeat domain 91 (WDR91), mRNA
ENSBTAP00000027728-D1	-1.19	2.68E-199	↑	B. mutus Finkel-Biskis-Reilly murine sarcoma virus ubiquitously expressed (FAU), transcript variant X1, mRNA
ENSBTAP00000021595-D1	-1.19	4.87E-04	↑	B. mutus protein O-fucosyltransferase 1 (POFUT1), transcript variant X1, mRNA
ENSP00000367890-D1	-1.19	4.87E-04	↑	B. mutus NAD kinase (NADK), mRNA
ENSP00000404833-D1	-1.19	4.87E-04	↑	B. mutus TAP binding protein (tapasin) (TAPBP), mRNA
ENSBTAP00000015248-D3	-1.19	6.01E-27	↑	B. mutus myosin, light chain 12B, regulatory (MYL12B), transcript variant X1, mRNA
ENSBTAP00000003609-D1	-1.19	2.42E-25	↑	B. mutus COP9 signalosome subunit 5 (COPS5), mRNA
ENSBTAP00000015669-D1	-1.19	6.89E-05	↑	B. mutus perilipin 3 (PLIN3), transcript variant X1, mRNA
ENSBTAP00000047345-D1	-1.19	6.89E-05	↑	B. mutus F-box and WD repeat domain containing 8 (FBXW8), mRNA
yakG028134	-1.19	4.00E-37	↑	B. mutus chromosome unknown open reading frame, human C8orf48 (LOC102276626), mRNA
ENSP00000327179-D1	-1.19	1.17E-58	↑	B. mutus cirrhosis, autosomal recessive 1A (cirhin) (CIRH1A), mRNA
ENSP00000294742-D1	-1.18	5.90E-26	↑	B. mutus actin related protein 2/3 complex, subunit 5, 16kDa (ARPC5), transcript variant X2, mRNA
ENSBTAP00000042447-D1	-1.18	5.62E-05	↑	B. mutus placenta-specific gene 8 protein-like (LOC102276301), mRNA
ENSBTAP00000021041-D1	-1.18	2.85E-03	↑	B. mutus Rho guanine nucleotide exchange factor (GEF) 4 (ARHGEF4), mRNA
ENSP00000262765-D1	-1.18	2.85E-03	↑	B. mutus glutamine rich 2 (QRICH2), mRNA
ENSBTAP00000006659-D1	-1.18	2.09E-02	↑	B. mutus B lymphoid tyrosine kinase (BLK), mRNA
ENSBTAP00000003618-D1	-1.18	2.09E-02	↑	B. mutus leucine-rich alpha-2-glycoprotein 1 (LRG1), mRNA
ENSP00000204517-D1	-1.18	2.09E-02	↑	B. mutus transcription factor AP-4 (activating enhancer binding protein 4) (TFAP4), mRNA
ENSBTAP00000013156-D1	-1.18	2.09E-02	↑	B. taurus t-SNARE domain containing 1 (TSNARE1), mRNA
ENSBTAP00000041141-D1	-1.18	2.09E-02	↑	B. taurus phosphatidylethanolamine N-methyltransferase (PEMT), mRNA
ENSP00000382441-D1	-1.18	3.88E-21	↑	B. mutus ubiquitin fusion degradation 1 like (yeast) (UFD1L), mRNA
ENSBTAP00000032948-D1	-1.18	1.51E-25	↑	B. mutus t-complex 11, testis-specific-like 1 (TCP11L1), transcript variant X2, mRNA
ENSP00000361635-D1	-1.18	1.25E-94	↑	Capra hircus voltage-dependent anion channel 2 (VDAC2), transcript variant X1, mRNA
ENSBTAP00000012699-D1	-1.18	2.03E-22	↑	B. mutus protein arginine methyltransferase 7 (PRMT7), mRNA
ENSBTAP00000037826-D1	-1.18	3.49E-67	↑	B. mutus WNT1 inducible signaling pathway protein 3 (WISP3), mRNA
ENSP00000334409-D2	-1.18	3.45E-18	↑	B. mutus guanosine monophosphate reductase (GMPR), mRNA
ENSBTAP00000001208-D1	-1.18	3.45E-18	↑	B. mutus cytochrome b-c1 complex subunit 9-like (LOC102270524), mRNA
ENSBTAP00000003881-D1	-1.18	2.12E-10	↑	B. taurus glutamic pyruvate transaminase (alanine aminotransferase) 2 (GPT2), mRNA
ENSBTAP00000052439-D3	-1.18	1.61E-17	↑	B. mutus pyruvate dehydrogenase (lipoamide) alpha 1 (PDHA1), mRNA
ENSP00000222803-D1	-1.18	1.61E-17	↑	B. mutus FK506 binding protein 14, 22 kDa (FKBP14), mRNA
ENSP00000397900-D2	-1.18	2.93E-44	↑	B. mutus maestro (MRO), transcript variant X2, mRNA
ENSBTAP00000006962-D1	-1.18	2.33E-03	↑	B. mutus fms-related tyrosine kinase 3 ligand (FLT3LG), transcript variant X1, mRNA
ENSP00000316933-D95	-1.18	2.33E-03	↑	B. mutus zinc finger protein 175 (ZNF175), mRNA
ENSP00000344816-D1	-1.18	5.09E-07	↑	B. mutus BSD domain containing 1 (BSDC1), transcript variant X2, mRNA

ENSBTAP00000015059-D1	-1.18	4.22E-26	↑	B. mutus 5'-nucleotidase, ecto (CD73) (NT5E), mRNA
ENSBTAP00000009087-D1	-1.18	1.13E-20	↑	B. mutus ATP-binding cassette, sub-family F (GCN20), member 3 (ABCF3), transcript variant X1, mRNA
ENSBTAP00000053136-D1	-1.18	1.67E-37	↑	B. mutus binder of sperm protein homolog 1 (BSPH1), mRNA
ENSBTAP00000027893-D1	-1.18	4.89E-08	↑	B. taurus annexin A11 (ANXA11), mRNA
ENSBTAP00000041398-D1	-1.18	5.36E-101	↑	B. mutus tubulin alpha-1A chain-like (LOC102270684), mRNA
ENSBTAP00000010711-D1	-1.17	5.44E-10	↑	Pantholops hodgsonii protein kinase inhibitor alpha (PKIA), transcript variant X2, mRNA
ENSBTAP00000043360-D1	-1.17	4.68E-09	↑	B. mutus bolA family member 1 (BOLA1), mRNA
ENSBTAP00000016475-D1	-1.17	2.93E-06	↑	B. mutus zinc finger protein 511 (ZNF511), mRNA
ENSP00000360425-D1	-1.17	2.17E-04	↑	B. mutus regulator of calcineurin 2 (RCAN2), transcript variant X2, mRNA
ENSBTAP00000053512-D1	-1.17	1.70E-02	↑	B. mutus chromobox homolog 2 (CBX2), mRNA
ENSBTAP00000053074-D1	-1.17	1.70E-02	↑	B. taurus cDNA clone IMAGE:8163664, **** WARNING: chimeric clone ****
ENSP00000242210-D2	-1.17	1.70E-02	↑	B. mutus 5'-nucleotidase, cytosolic IIIA (NT5C3A), transcript variant X1, mRNA
ENSBTAP00000023908-D2	-1.17	3.11E-43	↑	B. mutus prostaglandin E synthase 3 (cytosolic) (PTGES3), transcript variant X1, mRNA
ENSBTAP00000038799-D1	-1.17	1.00E-57	↑	B. mutus apolipoprotein B (APOB), mRNA
ENSBTAP00000004401-D1	-1.17	5.43E-14	↑	B. mutus zinc finger, RAN-binding domain containing 1 (ZRANB1), mRNA
ENSP00000346437-D1	-1.17	3.82E-13	↑	B. mutus autophagy related 7 (ATG7), mRNA
yakG041221	-1.17	2.28E-07	↑	B. mutus aldose reductase-like (LOC102286471), mRNA
ENSBTAP00000013678-D1	-1.17	2.28E-07	↑	B. mutus tRNA-histidine guanylyltransferase 1-like (S. cerevisiae) (THG1L), transcript variant X1, mRNA
ENSBTAP00000033002-D2	-1.17	2.28E-07	↑	Capra hircus ribosomal protein S12 (RPS12), mRNA
ENSP00000298119-D1	-1.17	1.54E-03	↑	B. mutus leucine rich repeat and fibronectin type III domain containing 5 (LRFN5), mRNA
ENSBTAP00000016063-D1	-1.17	1.54E-03	↑	B. taurus solute carrier family 25 (mitochondrial iron transporter), member 28 (SLC25A28), mRNA
ENSBTAP00000006347-D1	-1.17	1.60E-06	↑	B. mutus nitrilase family, member 2 (NIT2), mRNA
ENSBTAP00000008072-D2	-1.17	1.98E-14	↑	B. mutus microtubule-associated protein 1 light chain 3 alpha (MAP1LC3A), partial mRNA
ENSP00000369129-D1	-1.17	1.70E-09	↑	B. mutus desmoplakin (DSP), mRNA
ENSP00000356174-D1	-1.17	1.80E-12	↑	B. mutus SRY (sex determining region Y)-box 13 (SOX13), mRNA
ENSBTAP00000020586-D1	-1.17	1.38E-05	↑	Bubalus bubalis lysine (K)-specific demethylase 4B (KDM4B), transcript variant X1, mRNA
ENSP00000356702-D1	-1.17	1.67E-122	↑	B. mutus phosphatidylinositol glycan anchor biosynthesis, class C (PIGC), transcript variant X3, mRNA
ENSP00000264712-D1	-1.17	4.41E-25	↑	B. mutus kinesin family member 3C (KIF3C), mRNA
ENSBTAP00000041351-D1	-1.17	8.44E-12	↑	B. mutus ubiquitin interaction motif containing 1 (UIMC1), mRNA
ENSP00000377112-D1	-1.17	1.26E-03	↑	B. mutus solute carrier family 12, member 8 (SLC12A8), partial mRNA
ENSBTAP00000002199-D1	-1.17	1.39E-02	↑	B. mutus leucine rich repeat containing 52 (LRRC52), mRNA
ENSP00000320081-D1	-1.17	1.39E-02	↑	B. taurus chromosome 1 open reading frame, human C3orf58 (C1H3orf58), mRNA
ENSBTAP00000019133-D1	-1.17	1.39E-02	↑	Pantholops hodgsonii natural resistance-associated macrophage protein 2-like (LOC102329220), misc_RNA
ENSBTAP00000044999-D1	-1.17	1.39E-02	↑	B. mutus jumonji domain containing 8 (JMJD8), mRNA
ENSBTAP00000016742-D1	-1.17	1.39E-02	↑	B. mutus protein SCO2 homolog, mitochondrial-like (LOC102268175), mRNA
ENSBTAP00000044757-D1	-1.17	3.37E-51	↑	B. mutus family with sequence similarity 229, member B (FAM229B), mRNA
ENSP00000276658-D1	-1.17	4.15E-14	↑	B. mutus estrogen receptor binding site associated, antigen, 9 (EBAG9), mRNA
ENSP00000386563-D1	-1.17	9.18E-06	↑	B. mutus MORN repeat containing 2 (MORN2), mRNA
ENSBTAP00000006343-D1	-1.17	9.18E-06	↑	B. mutus Yip1 interacting factor homolog A (S. cerevisiae) (YIF1A), mRNA
ENSP00000388311-D100	-1.16	6.50E-09	↑	Bubalus bubalis zinc finger protein 850 (ZNF850), transcript variant X2, mRNA
ENSBTAP00000029271-D1	-1.16	9.66E-05	↑	B. mutus Niemann-Pick disease, type C2 (NPC2), mRNA
ENSP00000379767-D2	-1.16	7.72E-42	↑	B. mutus mitochondrial ribosomal protein S36 (MRPS36), mRNA
ENSBTAP00000023204-D1	-1.16	1.02E-03	↑	B. mutus mitochondrial elongation factor 1 (MIEF1), mRNA
ENSBTAP00000007959-D1	-1.16	3.91E-26	↑	B. mutus translin (TSN), mRNA
ENSBTAP00000020766-D1	-1.16	2.16E-11	↑	B. mutus inositol monophosphatase domain containing 1 (IMPAD1), mRNA
ENSBTAP00000034279-D1	-1.16	4.75E-07	↑	B. mutus translocase of inner mitochondrial membrane 10 homolog (yeast) (TIMM10), mRNA
ENSBTAP00000019848-D1	-1.16	2.33E-105	↑	B. mutus versican (VCAN), transcript variant X1, mRNA
yakG004246	-1.16	5.00E-06	↑	Balaenoptera acutorostrata scammoni RNA polymerase-associated protein LEO1-like (LOC102998321), mRNA
ENSBTAP00000045378-D1	-1.16	8.34E-04	↑	B. mutus up-regulated during skeletal muscle growth protein 5-like (LOC102273004), mRNA
ENSP00000386711-D1	-1.16	1.13E-02	↑	B. mutus janus kinase and microtubule interacting protein 1 (JAKMIP1), mRNA
ENSP00000257414-D1	-1.16	1.13E-02	↑	B. mutus sema domain, transmembrane domain (TM), and cytoplasmic domain, (semaphorin) 6A (SEMA6A), transcript variant X1, mRNA
ENSP00000337653-D1	-1.16	1.13E-02	↑	B. mutus tetratricopeptide repeat domain 8 (TTC8), mRNA
ENSBTAP00000019603-D1	-1.16	1.51E-10	↑	B. mutus non-SMC condensin I complex, subunit D2 (NCAPD2), mRNA
ENSBTAP00000021223-D1	-1.16	4.58E-65	↑	B. mutus methylmalonic aciduria and homocystinuria type D homolog, mitochondrial-like, mRNA

ENSP00000366280-D1	-1.16	8.25E-11	↑	B. mutus lectin, mannose-binding 2-like (LMAN2L), transcript variant X1, mRNA
ENSBTAP00000017665-D1	-1.16	6.63E-18	↑	B. mutus NECAP endocytosis associated 2 (NECAP2), transcript variant X1, mRNA
ENSBTAP00000025255-D1	-1.16	1.51E-152	↑	B. mutus COP9 signalosome subunit 3 (COPS3), mRNA
ENSP00000194118-D1	-1.16	1.74E-07	↑	B. mutus KIAA0141 ortholog (KIAA0141), mRNA
ENSP00000317872-D1	-1.16	4.96E-35	↑	B. mutus retinoblastoma binding protein 6 (RBBP6), transcript variant X1, mRNA
ENSP00000335147-D1	-1.16	9.14E-03	↑	B. mutus ankyrin repeat domain 37 (ANKRD37), mRNA
ENSP00000356382-D1	-1.16	9.14E-03	↑	B. mutus coagulation factor XIII, B polypeptide (F13B), mRNA
ENSBTAP00000006264-D1	-1.16	9.14E-03	↑	B. mutus retinoic acid induced 1 (RAI1), mRNA
ENSP00000378298-D1	-1.15	4.72E-10	↑	B. mutus solute carrier family 6 (neurotransmitter transporter), member 4 (SLC6A4), mRNA
ENSBTAP00000023552-D1	-1.15	1.35E-51	↑	B. mutus ATP-binding cassette, sub-family G (WHITE), member 2 (ABCG2), transcript variant X2, mRNA
ENSBTAP00000007532-D1	-1.15	4.87E-46	↑	B. mutus 1-acylglycerol-3-phosphate O-acyltransferase 6 (AGPAT6), transcript variant X2, mRNA
ENSP00000358374-D1	-1.15	5.87E-19	↑	B. mutus SR-related CTD-associated factor 11 (SCAF11), mRNA
ENSBTAP00000048623-D1	-1.15	4.54E-04	↑	B. mutus protein unc-13 homolog C-like (LOC102285566), mRNA
ENSBTAP00000002392-D1	-1.15	3.04E-20	↑	B. mutus leucine rich repeat containing 46 (LRRC46), mRNA
ENSP00000391106-D1	-1.15	5.50E-58	↑	B. mutus myosin X (MYO10), mRNA
ENSBTAP00000047883-D15	-1.15	2.34E-05	↑	B. mutus tubulin beta chain-like (LOC102285043), mRNA
ENSP00000200181-D1	-1.15	2.34E-05	↑	B. mutus integrin, beta 4 (ITGB4), transcript variant X3, mRNA
ENSBTAP00000016048-D1	-1.15	6.33E-08	↑	B. mutus symplekin (SYMPK), mRNA
ENSP00000319062-D1	-1.15	1.51E-15	↑	B. mutus Bardet-Biedl syndrome 12 (BBS12), mRNA
ENSP00000282486-D1	-1.15	3.77E-13	↑	Bubalus bubalis muscleblind-like splicing regulator 1 (MBNL1), transcript variant X2, mRNA
ENSP00000307870-D1	-1.15	1.92E-05	↑	B. mutus copper chaperone for superoxide dismutase (CCS), mRNA
ENSBTAP00000021507-D1	-1.15	7.45E-03	↑	B. mutus mitogen-activated protein kinase 3 (MAPK3), mRNA
ENSP00000366513-D2	-1.15	7.45E-03	↑	B. mutus calsyntenin 2 (CLSTN2), mRNA
ENSBTAP00000005375-D1	-1.15	7.45E-03	↑	B. mutus guanidinoacetate N-methyltransferase (GAMT), mRNA
ENSP00000215061-D1	-1.15	1.30E-14	↑	Bubalus bubalis occludin/ELL domain containing 1 (OCEL1), transcript variant X1, mRNA
ENSP00000312773-D1	-1.15	8.10E-07	↑	B. mutus NFKB inhibitor interacting Ras-like 2 (NKIRAS2), transcript variant X1, mRNA
ENSP00000376721-D1	-1.15	1.57E-05	↑	B. mutus zinc finger and BTB domain containing 16 (ZBTB16), mRNA
ENSBTAP00000022652-D1	-1.15	1.57E-05	↑	B. mutus protein kinase N1 (PKN1), mRNA
ENSP00000353094-D1	-1.15	6.61E-07	↑	B. mutus stromal cell derived factor 4 (SDF4), mRNA
ENSP00000286371-D6	-1.15	2.64E-12	↑	B. mutus ATPase, Na+/K+ transporting, beta 3 polypeptide (ATP1B3), mRNA
ENSBTAP00000044311-D1	-1.15	1.28E-05	↑	B. mutus cytochrome c oxidase protein 20 homolog (LOC102271041), mRNA
ENSBTAP00000020982-D1	-1.15	1.88E-08	↑	Canis lupus familiaris serine/threonine-protein phosphatase 4 regulatory subunit 1-like, transcript variant X5, mRNA
ENSP00000386867-D1	-1.15	6.06E-03	↑	B. mutus GULP, engulfment adaptor PTB domain containing 1 (GULP1), transcript variant X1, mRNA
ENSBTAP00000026034-D1	-1.15	6.06E-03	↑	B. mutus microfibrillar-associated protein 3-like (MFAP3L), mRNA
ENSP00000371512-D1	-1.15	6.06E-03	↑	B. mutus sarcoglycan, zeta (SGCZ), mRNA
ENSP00000370620-D1	-1.15	6.06E-03	↑	B. mutus zinc finger BED domain-containing protein 1-like (LOC102267452), mRNA
ENSBTAP00000021600-D1	-1.15	1.81E-26	↑	B. mutus ribonuclease H1 (RNASEH1), partial mRNA
ENSBTAP00000052867-D1	-1.15	4.52E-105	↑	B. mutus transforming, acidic coiled-coil containing protein 3 (TACC3), mRNA
ENSBTAP00000006105-D1	-1.15	2.02E-04	↑	B. mutus lysophosphatidic acid receptor 2 (LPAR2), mRNA
ENSP00000347802-D1	-1.15	2.02E-04	↑	B. mutus ankyrin repeat domain 35 (ANKRD35), mRNA
ENSP00000374989-D1	-1.14	6.96E-06	↑	B. taurus IgM (LOC524810), mRNA
ENSP00000350311-D1	-1.14	2.62E-20	↑	Bubalus bubalis BCL2-like 11 (apoptosis facilitator) (BCL2L11), transcript variant X1, mRNA
ENSP00000362264-D1	-1.14	1.65E-04	↑	B. mutus apolipoprotein O-like (APOOL), mRNA
ENSBTAP00000024319-D1	-1.14	4.91E-03	↑	B. mutus solute carrier family 38, member 10 (SLC38A10), mRNA
ENSP00000366982-D1	-1.14	2.10E-41	↑	B. mutus tRNA methyltransferase 10 homolog B (S. cerevisiae) (TRMT10B), mRNA
ENSP00000356700-D1	-1.14	5.61E-09	↑	B. mutus chromosome unknown open reading frame, human C1orf105 (LOC102280804), mRNA
ENSBTAP00000020929-D1	-1.14	2.47E-31	↑	B. mutus coenzyme Q5 homolog, methyltransferase (S. cerevisiae) (COQ5), mRNA
ENSP00000259467-D1	-1.14	1.59E-10	↑	B. taurus phosducin-like (PDCL), mRNA
ENSP00000386310-D1	-1.14	4.64E-06	↑	B. mutus spermatogenesis associated, serine-rich 2-like (SPATS2L), transcript variant X1, mRNA
ENSBTAP00000047102-D1	-1.14	1.35E-04	↑	B. mutus acyl-CoA dehydrogenase family, member 9 (ACAD9), mRNA
ENSP00000300022-D1	-1.14	4.00E-03	↑	B. mutus yippee-like 4 (Drosophila) (YPEL4), mRNA
ENSBTAP00000050584-D1	-1.14	4.00E-03	↑	B. mutus NEDD4 binding protein 2-like 1 (N4BP2L1), mRNA
ENSP00000300128-D1	-1.14	2.11E-11	↑	B. mutus transmembrane protein 194A (TMEM194A), transcript variant X1, mRNA
ENSBTAP00000037960-D1	-1.14	3.20E-08	↑	B. mutus phospholipase A2, group IB (pancreas) (PLA2G1B), mRNA

ENSBTAP00000027076-D3	-1.14	1.37E-06	↑	B. mutus adaptor-related protein complex 1, beta 1 subunit (AP1B1), transcript variant X1, mRNA
ENSP00000375632-D1	-1.14	6.96E-82	↑	B. mutus ribosomal protein S9 (RPS9), mRNA
ENSP00000368278-D1	-1.14	2.13E-08	↑	B. mutus interleukin 1 receptor accessory protein-like 1 (IL1RAPL1), mRNA
yakG038243	-1.14	2.65E-03	↑	B. mutus lactoylglutathione lyase-like (LOC102279164), mRNA
ENSP00000313875-D1	-1.14	1.42E-08	↑	B. mutus membrane cofactor protein-like (LOC102279542), mRNA
ENSP00000366844-D66	-1.14	4.16E-12	↑	B. mutus zinc finger protein 112 homolog (mouse) (ZFP112), mRNA
ENSP00000350353-D1	-1.13	6.12E-07	↑	B. mutus methyltransferase like 20 (METTL20), mRNA
ENSBTAP00000002742-D2	-1.13	2.16E-03	↑	B. mutus sesquipedalian-1-like (LOC102265287), mRNA
ENSBTAP00000024993-D1	-1.13	2.16E-03	↑	B. mutus hydroxysteroid dehydrogenase like 1 (HSDL1), mRNA
ENSBTAP00000007216-D1	-1.13	4.53E-18	↑	B. mutus ariadne RBR E3 ubiquitin protein ligase 2 (ARIH2), transcript variant X2, mRNA
ENSP00000377725-D1	-1.13	6.12E-31	↑	B. mutus zinc finger with KRAB and SCAN domains 5 (ZKSCAN5), mRNA
ENSBTAP00000018411-D1	-1.13	2.65E-05	↑	B. taurus dual specificity phosphatase 1 (DUSP1), mRNA
ENSBTAP00000042466-D4	-1.13	2.65E-05	↑	B. mutus histone H4-like (LOC102271941), mRNA
ENSBTAP00000052925-D1	-1.13	1.27E-14	↑	B. mutus BH3 interacting domain death agonist (BID), mRNA
ENSP00000297459-D1	-1.13	8.23E-13	↑	B. mutus transmembrane protein 74 (TMEM74), mRNA
ENSBTAP00000009038-D1	-1.13	1.85E-33	↑	B. mutus RasGEF domain family, member 1A (RASGEF1A), mRNA
ENSP00000365840-D1	-1.13	4.26E-09	↑	B. mutus ectonucleoside triphosphate diphosphohydrolase 6 (putative) (ENTPD6), mRNA
ENSP00000355568-D1	-1.13	2.73E-07	↑	B. mutus interferon regulatory factor 2 binding protein 2 (IRF2BP2), partial mRNA
ENSP00000222339-D1	-1.13	2.73E-07	↑	B. mutus zinc finger protein 574 (ZNF574), mRNA
yakG033344	-1.13	3.58E-11	↑	B. mutus TBC1 domain family member 7-like (LOC102280443), mRNA
ENSBTAP00000039471-D1	-1.13	1.43E-03	↑	B. mutus latrophilin-3-like (LOC102280206), transcript variant X3, mRNA
ENSBTAP00000012443-D1	-1.13	1.33E-13	↑	B. mutus methyltransferase-like protein 2-like (LOC102268373), mRNA
ENSP00000305918-D1	-1.13	1.76E-30	↑	B. mutus bromodomain containing 3 (BRD3), mRNA
ENSBTAP00000012287-D1	-1.13	1.24E-18	↑	B. mutus NADH dehydrogenase (ubiquinone) 1 alpha subcomplex, 5 (NDUFA5), transcript variant X1, mRNA
ENSBTAP00000048537-D40	-1.13	9.48E-04	↑	B.taurus DNA sequence from clone CH240-271N5, complete sequence
ENSBTAP00000007855-D2	-1.13	9.48E-04	↑	B. mutus dishevelled associated activator of morphogenesis 2 (DAAM2), mRNA
ENSP00000218348-D1	-1.13	1.94E-17	↑	B. mutus ubiquitin specific peptidase 11 (USP11), mRNA
ENSP00000405218-D25	-1.12	7.83E-144	↑	B. mutus zinc finger protein 347-like (LOC102269944), mRNA
ENSBTAP00000002398-D1	-1.12	3.30E-11	↑	B. taurus gap junction protein, alpha 1, 43kDa (GJA1), mRNA
ENSP00000265085-D1	-1.12	0.00E+00	↑	B. mutus cytoplasmic polyadenylation element binding protein 4 (CPEB4), mRNA
ENSBTAP00000017239-D1	-1.12	2.14E-09	↑	B. mutus secernin 3 (SCRN3), mRNA
ENSP00000395337-D3	-1.12	6.68E-115	↑	B. mutus lactate dehydrogenase B (LDHB), mRNA
ENSBTAP00000046356-D2	-1.12	4.78E-15	↑	B. mutus malignant T cell amplified sequence 1 (MCTS1), transcript variant X1, mRNA
ENSBTAP00000048061-D1	-1.12	0.00E+00	↑	B. mutus NLR family, pyrin domain containing 8 (NLRP8), mRNA
ENSP00000333551-D1	-1.12	1.87E-04	↑	B. mutus proline synthetase co-transcribed homolog (bacterial) (PROSC), mRNA
ENSBTAP00000017999-D1	-1.12	1.87E-04	↑	B. mutus DNA fragmentation factor, 45kDa, alpha polypeptide (DFFA), mRNA
ENSBTAP00000042643-D1	-1.12	2.30E-10	↑	B. mutus inositol-tetrakisphosphate 1-kinase (ITPK1), mRNA
ENSBTAP00000002274-D1	-1.12	1.03E-22	↑	B. mutus ubinuclein 1 (UBN1), mRNA
ENSBTAP00000023162-D1	-1.12	2.47E-17	↑	B. mutus transmembrane protein 214 (TMEM214), transcript variant X1, mRNA
ENSP00000249750-D1	-1.12	5.19E-31	↑	B. mutus aldehyde dehydrogenase 1 family, member A2 (ALDH1A2), transcript variant X1, mRNA
ENSBTAP00000037474-D1	-1.12	5.49E-05	↑	B. mutus l(3)mbt-like 2 (Drosophila) (L3MBTL2), mRNA
ENSBTAP00000000862-D1	-1.12	1.90E-67	↑	B. mutus family with sequence similarity 83, member D (FAM83D), mRNA
ENSBTAP00000039462-D1	-1.12	4.48E-05	↑	B. mutus small integral membrane protein 20 (SMIM20), mRNA
ENSP00000398181-D1	-1.11	4.71E-16	↑	B. mutus alkB, alkylation repair homolog 2 (E. coli) (ALKBH2), mRNA
ENSBTAP00000041411-D1	-1.11	1.07E-30	↑	B. mutus eukaryotic translation initiation factor 6 (EIF6), mRNA
ENSP00000239830-D1	-1.11	2.00E-05	↑	Bubalus bubalis coiled-coil domain containing 77 (CCDC77), transcript variant X2, mRNA
ENSBTAP00000013718-D1	-1.11	1.31E-09	↑	B. mutus tRNA selenocysteine 1 associated protein 1 (TRNAU1AP), mRNA
ENSP00000344285-D1	-1.11	4.41E-22	↑	B. mutus brain-specific homeobox (BSX), mRNA
ENSP00000372368-D1	-1.11	9.85E-31	↑	Bubalus bubalis chromosome unknown open reading frame, human C18orf5), transcript variant X2, mRNA
ENSP00000252453-D1	-1.11	7.25E-06	↑	B. mutus chromosome unknown open reading frame, human C19orf80 (LOC102267376), mRNA
ENSP00000321636-D1	-1.11	4.83E-06	↑	B. mutus topoisomerase (DNA) III alpha (TOP3A), mRNA
ENSP00000346635-D1	-1.11	3.94E-06	↑	B. mutus ERO1-like beta (S. cerevisiae) (ERO1LB), mRNA
ENSP00000385899-D1	-1.11	3.94E-06	↑	B. mutus sidekick cell adhesion molecule 1 (SDK1), mRNA
ENSBTAP00000009410-D1	-1.11	2.63E-06	↑	B. mutus osteosarcoma amplified 9, endoplasmic reticulum lectin (OS9), transcript variant X1, mRNA
ENSBTAP00000006995-D1	-1.11	1.64E-47	↑	B. mutus ring finger protein (C3H2C3 type) 6 (RNF6), mRNA

ENSBTAP00000019583-D1	-1.11	7.79E-07	↑	B. mutus cystatin E/M (CST6), mRNA
ENSBTAP00000039615-D1	-1.11	2.83E-07	↑	B. mutus eukaryotic translation initiation factor 4E binding protein 1 (EIF4EBP1), mRNA
ENSP00000234396-D1	-1.11	2.04E-08	↑	B. mutus ATPase, H+ transporting, lysosomal 56/58kDa, V1 subunit B1, transcript variant X1, mRNA
ENSP00000352608-D2	-1.11	1.10E-15	↑	Pantholops hodgsonii ryanodine receptor 3 (RYR3), mRNA
ENSBTAP00000004027-D1	-1.10	2.87E-17	↑	B. mutus squamous cell carcinoma antigen recognized by T cells (SART1), mRNA
ENSBTAP00000005013-D1	-1.10	1.43E-81	↑	B. taurus SWI/SNF related, matrix associated, actin dependent regulator of chromatin, subfamily a-like 1, mRNA
ENSBTAP00000012088-D6	-1.10	1.10E-129	↑	B. mutus ribosomal protein L7 (RPL7), transcript variant X2, mRNA
ENSBTAP00000027808-D1	-1.10	1.64E-105	↑	B. mutus methionine adenosyltransferase II, alpha (MAT2A), mRNA
ENSBTAP00000020084-D1	-1.10	7.31E-21	↑	B. mutus nucleolar protein 12 (NOL12), mRNA
ENSP00000363458-D1	-1.10	1.13E-15	↑	B. mutus low density lipoprotein receptor adaptor protein 1 (LDLRAP1), mRNA
ENSBTAP00000015698-D1	-1.10	1.35E-09	↑	B. mutus AT rich interactive domain 3B (BRIGHT-like) (ARID3B), mRNA
ENSBTAP00000042442-D1	-1.10	5.16E-08	↑	B. mutus solute carrier family 35 member E2-like (LOC102271485), mRNA
ENSP00000269886-D2	-1.10	7.73E-08	↑	B. mutus SH3-domain GRB2-like 2 (SH3GL2), mRNA
ENSP00000414002-D1	-1.10	2.12E-07	↑	B. mutus Berardinelli-Seip congenital lipodystrophy 2 (seipin) (BSCL2), transcript variant X2, mRNA
ENSBTAP00000020116-D1	-1.10	5.83E-07	↑	Bubalus bubalis SIK family kinase 3 (SIK3), transcript variant X3, mRNA
ENSBTAP00000041782-D1	-1.10	1.72E-04	↑	B. mutus cytochrome P450 19A1-like (LOC102277416), mRNA
ENSBTAP00000025446-D1	-1.10	1.72E-04	↑	B. mutus CDC42 effector protein (Rho GTPase binding) 4 (CDC42EP4), mRNA
ENSP00000281631-D1	-1.10	3.87E-04	↑	B. mutus poly (ADP-ribose) polymerase family, member 8 (PARP8), transcript variant X1, mRNA
ENSP00000356370-D1	-1.10	7.10E-04	↑	B. taurus crumbs homolog 1 (Drosophila) (CRB1), mRNA
ENSP00000377910-D1	-1.10	8.71E-04	↑	B. mutus dual specificity phosphatase 14 (DUSP14), mRNA
ENSP00000359665-D1	-1.10	1.99E-03	↑	B. taurus phosphatidylinositol 4-kinase type 2 alpha (PI4K2A), mRNA
yakG038680	-1.10	2.44E-03	↑	Bubalus bubalis formimidoyltransferase-cyclodeaminase-like (LOC102390644), mRNA
ENSBTAP00000013338-D1	-1.10	2.99E-03	↑	B. mutus zinc finger and BTB domain containing 24 (ZBTB24), transcript variant X2, misc_RNA
ENSBTAP00000011420-D1	-1.10	2.99E-03	↑	Pantholops hodgsonii chromosome unknown open reading frame, human C11orf52, transcript variant X2, mRNA
ENSP00000222462-D1	-1.10	2.99E-03	↑	B. mutus wingless-type MMTV integration site family, member 16 (WNT16), mRNA
ENSBTAP00000025570-D1	-1.10	3.68E-03	↑	B. mutus potassium voltage-gated channel, delayed-rectifier, subfamily S, member 3 (KCNS3), mRNA
ENSP00000262464-D1	-1.10	3.68E-03	↑	B. mutus fibrillin 2 (FBN2), mRNA
ENSBTAP00000049946-D28	-1.10	3.68E-03	↑	B.taurus DNA sequence from clone CH240-487I9, complete sequence
ENSBTAP00000000043-D1	-1.10	4.55E-03	↑	B. mutus sirtuin 7 (SIRT7), mRNA
ENSBTAP00000007341-D1	-1.10	4.55E-03	↑	B. mutus serine/threonine kinase 19 (STK19), mRNA
ENSBTAP00000031650-D9	-1.10	4.55E-03	↑	Capra hircus multidrug resistance-associated protein 4-like (LOC102172427), mRNA
ENSBTAP00000001564-D1	-1.10	5.57E-03	↑	B. mutus telomerase-associated protein 1 (TEP1), mRNA
ENSP00000311684-D1	-1.10	5.57E-03	↑	B. taurus SPEG complex locus (SPEG), mRNA
ENSBTAP00000000314-D1	-1.10	1.28E-02	↑	B. mutus haloacid dehalogenase-like hydrolase domain containing 3 (HDHD3), mRNA
ENSBTAP00000003564-D1	-1.10	1.28E-02	↑	Bubalus bubalis V-set and transmembrane domain containing 5 (VSTM5), partial mRNA
ENSBTAP00000025092-D1	-1.10	1.58E-02	↑	B. mutus DND microRNA-mediated repression inhibitor 1 (DND1), mRNA
ENSP00000394849-D1	-1.10	1.58E-02	↑	Orycteropus afer afer rhomboid, veinlet-like 3 (Drosophila) (RHBDL3), transcript variant X3, misc_RNA
ENSP00000384666-D1	-1.10	1.58E-02	↑	B. mutus peptidyl-prolyl cis-trans isomerase A-like (LOC102281186), mRNA
ENSP00000419153-D1	-1.10	1.58E-02	↑	B. mutus zinc finger and BTB domain containing 20 (ZBTB20), transcript variant X4, mRNA
ENSBTAP00000022753-D1	-1.10	1.93E-02	↑	B. mutus RASD family, member 2 (RASD2), mRNA
ENSP00000413501-D1	-1.10	1.93E-02	↑	Bos indicus breed Vechur growth hormone precursor (GH) gene, complete cds
ENSP00000341214-D4	-1.10	1.93E-02	↑	B. mutus testis-specific H1 histone-like (LOC102287131), partial mRNA
ENSP00000343348-D1	-1.10	1.93E-02	↑	B. mutus vimentin-type intermediate filament associated coiled-coil protein (VMAC), mRNA
ENSBTAP00000008240-D1	-1.10	2.40E-02	↑	B. mutus RNA binding protein, fox-1 homolog (C. elegans) 3 (RBFOX3), transcript variant X3, mRNA
ENSP00000395668-D1	-1.10	2.40E-02	↑	B. mutus coiled-coil domain containing 149 (CCDC149), mRNA
ENSBTAP00000051454-D1	-1.10	2.40E-02	↑	B. mutus TATA box binding protein (TBP)-associated factor, RNA polymerase I, C, 110kDa (TAF1C), mRNA
ENSBTAP00000002244-D1	-1.10	3.00E-02	↑	B. mutus autophagy related 2B (ATG2B), mRNA
ENSP00000378561-D1	-1.10	3.74E-02	↑	B. mutus Ras association (RalGDS/AF-6) domain family member 1 (RASSF1), transcript variant X2, mRNA
ENSBTAP00000023093-D1	-1.10	3.74E-02	↑	B. mutus RELT-like 2 (RELL2), mRNA
ENSBTAP00000020299-D2	-1.10	3.74E-02	↑	B. mutus lectin, galactoside-binding, soluble, 16 (LGALS16), mRNA
ENSBTAP00000001503-D1	-1.10	3.74E-02	↑	B. mutus thyroid hormone receptor interactor 6 (TRIP6), mRNA
ENSP00000251808-D1	-1.10	3.74E-02	↑	B. mutus grainyhead-like 2 (Drosophila) (GRHL2), mRNA
ENSBTAP00000023840-D1	-1.10	4.69E-02	↑	Bubalus bubalis ornithine decarboxylase 1 (ODC1), transcript variant X2, mRNA
ENSP00000221166-D1	-1.10	4.69E-02	↑	B. mutus neurofilament, medium polypeptide (NEFM), mRNA
ENSP00000341187-D1	-1.10	4.69E-02	↑	B. mutus phosphodiesterase 10A (PDE10A), mRNA

ENSP00000238647-D1	-1.10	4.69E-02	↑	B. mutus interferon regulatory factor 2 binding protein-like (IRF2BPL), partial mRNA
ENSBTAP00000031960-D1	-1.10	4.69E-02	↑	B. mutus solute carrier family 7, member 14 (SLC7A14), transcript variant X2, mRNA
yakG013753	-1.10	1.81E-263	↑	B. mutus U3 small nucleolar RNA-associated protein 14 homolog A-like (LOC102275867), mRNA
ENSBTAP00000041906-D1	-1.10	2.40E-138	↑	B. mutus malate dehydrogenase 2, NAD (mitochondrial) (MDH2), transcript variant X1, mRNA
ENSBTAP00000015248-D1	-1.09	0.00E+00	↑	B. mutus myosin regulatory light polypeptide 9-like (LOC102269444), transcript variant X2, mRNA
ENSBTAP00000029505-D1	-1.09	1.42E-29	↑	B. mutus THO complex 5 (THOC5), transcript variant X1, mRNA
ENSP00000387123-D1	-1.09	6.65E-35	↑	B. mutus aldehyde dehydrogenase 7 family, member A1 (ALDH7A1), mRNA
ENSBTAP00000019184-D3	-1.09	4.84E-11	↑	B. mutus ribosomal protein L22 (RPL22), mRNA
ENSP00000352040-D1	-1.09	3.67E-10	↑	.
ENSBTAP00000025153-D1	-1.09	8.00E-36	↑	Physeter catodon RGD motif, leucine rich repeats, tropomodulin domain and proline-rich containing, mRNA
ENSBTAP00000012065-D1	-1.09	1.68E-24	↑	B. mutus solute carrier family 35 (adenosine 3'-phospho 5'-phosphosulfate transporter), member B3, mRNA
ENSBTAP00000043655-D1	-1.09	9.34E-09	↑	B. mutus transcription factor A, mitochondrial-like (LOC102274331), mRNA
yakG029112	-1.08	5.42E-11	↑	B. mutus LON peptidase N-terminal domain and ring finger 2 (LONRF2), mRNA
yakG036397	-1.08	1.19E-59	↑	Bubalus bubalis oligosaccharyltransferase 4 homolog (S. cerevisiae) (OST4), mRNA
ENSBTAP00000006739-D2	-1.08	1.82E-10	↑	B. mutus bone morphogenetic protein 4 (BMP4), transcript variant X4, mRNA
ENSBTAP00000043974-D1	-1.08	2.74E-10	↑	B. mutus tyrosine-protein kinase Tec-like (LOC102264510), mRNA
ENSBTAP00000043290-D1	-1.08	6.13E-10	↑	B. mutus DCN1, defective in cullin neddylation 1, domain containing 3 (DCUN1D3), mRNA
ENSBTAP00000015090-D1	-1.08	4.08E-26	↑	B. mutus nudix (nucleoside diphosphate linked moiety X)-type motif 9 (NUDT9), mRNA
ENSP00000251363-D1	-1.08	1.13E-09	↑	B. mutus ceramide synthase 4 (CERS4), mRNA
ENSBTAP00000028797-D1	-1.08	9.63E-89	↑	B. mutus chromosome unknown open reading frame, human C11orf74 (LOC102286452), mRNA
ENSBTAP00000052626-D8	-1.08	4.65E-13	↑	B. mutus ribosomal protein L30 (RPL30), mRNA
ENSBTAP00000020194-D1	-1.08	1.56E-16	↑	B. mutus protease, serine, 23 (PRSS23), mRNA
ENSBTAP00000012334-D1	-1.08	5.69E-09	↑	B. mutus coiled-coil domain containing 36 (CCDC36), mRNA
ENSBTAP00000036040-D2	-1.08	8.25E-30	↑	B. mutus NOP10 ribonucleoprotein (NOP10), mRNA
ENSP00000381840-D1	-1.08	2.76E-53	↑	B. mutus AE binding protein 2 (AEBP2), transcript variant X2, mRNA
ENSBTAP00000024450-D1	-1.08	5.63E-05	↑	B. mutus ataxia, cerebellar, Cayman type (ATCAY), mRNA
ENSP00000264157-D1	-1.08	3.20E-28	↑	B. mutus cyclin T2 (CCNT2), transcript variant X1, mRNA
ENSP00000264079-D1	-1.08	6.92E-05	↑	B. mutus mucolipin 1 (MCOLN1), transcript variant X1, mRNA
ENSP00000380779-D1	-1.08	2.43E-27	↑	B. mutus tectonic family member 1 (TCTN1), partial mRNA
ENSP00000262188-D1	-1.08	1.26E-17	↑	B. mutus SWI/SNF related, matrix associated, actin dependent regulator of chromatin, subfamily d, member 1 (SMARCD1), transcript variant X1, mRNA
ENSP00000259486-D1	-1.08	6.45E-08	↑	Bubalus bubalis ectonucleotide pyrophosphatase/phosphodiesterase 2 (ENPP2), transcript variant X2, mRNA
ENSBTAP00000027614-D1	-1.08	3.22E-20	↑	B. mutus mitochondrial ribosomal protein L55 (MRPL55), transcript variant X1, mRNA
ENSP00000276708-D1	-1.08	9.06E-11	↑	B. mutus gasdermin-C-like (LOC102274018), mRNA
ENSBTAP00000011379-D1	-1.08	6.15E-200	↑	B. mutus mitochondrial fission regulator 1 (MTFR1), mRNA
ENSP00000340510-D1	-1.08	1.45E-07	↑	Bubalus bubalis periplakin (PPL), mRNA
ENSP00000388647-D1	-1.08	4.92E-70	↑	B. mutus minichromosome maintenance complex component 3 (MCM3), mRNA
ENSBTAP00000018734-D1	-1.07	8.80E-144	↑	B. mutus Rho guanine nucleotide exchange factor (GEF) 3 (ARHGEF3), transcript variant X2, mRNA
ENSBTAP00000029667-D1	-1.07	1.50E-14	↑	B. mutus transmembrane emp24 domain trafficking protein 2 (TMED2), transcript variant X1, mRNA
ENSBTAP00000031803-D2	-1.07	3.53E-04	↑	B. mutus interferon alpha-inducible protein 27-like protein 2-like (LOC102279077), mRNA
ENSBTAP00000021699-D3	-1.07	6.29E-37	↑	B. mutus ATPase, H+ transporting, lysosomal 34kDa, V1 subunit D (ATP6V1D), mRNA
ENSP00000390232-D1	-1.07	5.28E-04	↑	B. mutus zinc finger, B-box domain containing (ZBBX), mRNA
ENSBTAP00000049089-D1	-1.07	5.28E-04	↑	B. mutus ZFP62 zinc finger protein (ZFP62), mRNA
ENSBTAP00000017322-D1	-1.07	3.11E-18	↑	B. mutus leucine rich repeat containing 8 family, member A (LRRC8A), transcript variant X2, mRNA
ENSP00000265138-D1	-1.07	4.03E-86	↑	B. mutus arrestin domain containing 3 (ARRDC3), mRNA
ENSBTAP00000004817-D1	-1.07	2.58E-26	↑	B. mutus ZFP3 zinc finger protein (ZFP3), mRNA
ENSP00000368401-D1	-1.07	4.52E-06	↑	B. mutus paired box 6 (PAX6), transcript variant X4, mRNA
ENSBTAP00000005441-D1	-1.07	7.92E-04	↑	B. mutus spermatogenesis associated 20 (SPATA20), transcript variant X1, mRNA
ENSP00000264144-D1	-1.07	9.74E-04	↑	B. mutus laminin, gamma 2 (LAMC2), mRNA
ENSBTAP00000036700-D1	-1.07	9.74E-04	↑	B. mutus SH3 domain binding glutamic acid-rich protein (SH3BGR), mRNA
ENSBTAP00000031700-D1	-1.07	8.33E-06	↑	B. mutus 60S ribosomal protein L28-like (LOC102287469), mRNA
ENSBTAP00000024965-D1	-1.07	2.59E-47	↑	B. mutus protein kinase, AMP-activated, alpha 2 catalytic subunit (PRKAA2), mRNA
ENSP00000351310-D1	-1.07	1.02E-05	↑	B. mutus collagen, type VI, alpha 6 (COL6A6), mRNA
ENSP00000326671-D1	-1.07	1.20E-03	↑	B. mutus solute carrier family 47 (multidrug and toxin extrusion), member 2 (SLC47A2), mRNA
ENSBTAP00000003340-D1	-1.07	7.29E-16	↑	B. mutus fibrillarin (FBL), transcript variant X2, mRNA

ENSBTAP00000050243-D2	-1.07	1.87E-05	↑	B. mutus S-phase kinase-associated protein 1 (SKP1), mRNA
ENSP00000385865-D3	-1.07	2.42E-07	↑	B. mutus retinoic acid receptor, beta (RARB), mRNA
ENSP00000396552-D1	-1.06	2.29E-05	↑	B. mutus inositol 1,4,5-trisphosphate receptor interacting protein-like 1 (ITPRIPL1), transcript variant X2, mRNA
ENSP00000388311-D31	-1.06	6.81E-24	↑	B. mutus zinc finger protein 845-like (LOC102275476), mRNA
ENSP00000356520-D1	-1.06	5.78E-18	↑	B. mutus DEAH (Asp-Glu-Ala-His) box helicase 9 (DHX9), mRNA
ENSP00000221482-D1	-1.06	1.78E-12	↑	B. mutus DOT1-like histone H3K79 methyltransferase (DOT1L), mRNA
ENSP00000360222-D1	-1.06	5.24E-45	↑	B. mutus TM2 domain containing 1 (TM2D1), mRNA
ENSP00000373272-D1	-1.06	3.43E-05	↑	B. mutus tripartite motif containing 71, E3 ubiquitin protein ligase (TRIM71), mRNA
ENSBTAP00000043485-D1	-1.06	2.22E-03	↑	B. mutus ras homolog family member F (in filopodia) (RHOF), mRNA
ENSBTAP00000035049-D1	-1.06	2.22E-03	↑	B. mutus serine/threonine kinase 32A (STK32A), transcript variant X1, mRNA
ENSP00000346300-D1	-1.06	2.22E-03	↑	B. mutus v-crk avian sarcoma virus CT10 oncogene homolog-like (CRKL), mRNA
ENSP00000384763-D1	-1.06	5.81E-178	↑	B. mutus solute carrier family 8 (sodium/calcium exchanger), member 1 (SLC8A1), transcript variant X4, mRNA
ENSP00000370737-D1	-1.06	1.06E-08	↑	B. mutus glycine dehydrogenase (decarboxylating) (GLDC), mRNA
ENSP00000362803-D1	-1.06	2.07E-10	↑	B. mutus peptidylprolyl isomerase (cyclophilin)-like 1 (PPIL1), mRNA
ENSBTAP00000022249-D1	-1.06	2.53E-10	↑	B. mutus zinc finger and BTB domain containing 48 (ZBTB48), mRNA
ENSBTAP00000024514-D1	-1.06	0.00E+00	↑	B. mutus DEAD (Asp-Glu-Ala-Asp) box helicase 5 (DDX5), transcript variant X1, mRNA
ENSP00000361366-D1	-1.06	2.72E-03	↑	B. mutus collectin-43-like (LOC102277122), mRNA
ENSP00000373288-D1	-1.06	7.69E-05	↑	B. mutus biotinidase (BTD), transcript variant X1, mRNA
ENSP00000225171-D1	-1.06	3.35E-03	↑	B. mutus DnaJ (Hsp40) homolog, subfamily C, member 12 (DNAJC12), transcript variant X2, mRNA
ENSP00000338769-D1	-1.06	2.35E-09	↑	Bubalus bubalis ArfGAP with SH3 domain, ankyrin repeat and PH domain 3 (ASAP3), mRNA
ENSBTAP00000018765-D1	-1.06	1.16E-04	↑	B. taurus coiled-coil domain containing 9 (CCDC9), mRNA
ENSBTAP00000006828-D1	-1.06	1.16E-04	↑	B. mutus mevalonate kinase (MVK), mRNA
ENSP00000265104-D1	-1.06	4.12E-03	↑	B. mutus dynein, axonemal, heavy chain 5 (DNAH5), mRNA
ENSP00000370713-D2	-1.06	4.12E-03	↑	B. mutus adenosine deaminase, RNA-specific, B1 (ADARB1), transcript variant X2, mRNA
ENSBTAP00000019338-D1	-1.06	4.11E-06	↑	B. mutus family with sequence similarity 129, member A (FAM129A), mRNA
ENSBTAP00000002979-D1	-1.06	1.42E-04	↑	B. mutus chondroitin sulfate proteoglycan 5 (neuroglycan C) (CSPG5), mRNA
ENSP00000364405-D1	-1.06	5.04E-06	↑	B. mutus zinc finger protein 367 (ZNF367), mRNA
ENSP00000321406-D76	-1.06	5.05E-03	↑	B. mutus zinc finger protein 768 (ZNF768), mRNA
ENSP00000377530-D1	-1.06	5.05E-03	↑	B. mutus pyruvate carboxylase (PC), mRNA
ENSBTAP00000006410-D1	-1.06	9.64E-09	↑	B. mutus N(alpha)-acetyltransferase 60, NatF catalytic subunit (NAA60), mRNA
ENSBTAP00000019842-D1	-1.05	3.29E-07	↑	B. mutus sirtuin 5 (SIRT5), transcript variant X1, mRNA
ENSP00000403491-D6	-1.05	7.02E-32	↑	B. mutus calmodulin 3 (phosphorylase kinase, delta) (CALM3), transcript variant X2, mRNA
ENSP00000265433-D1	-1.05	5.31E-18	↑	B. mutus nibrin (NBN), mRNA
ENSP00000338093-D1	-1.05	2.61E-04	↑	Bubalus bubalis threonyl-tRNA synthetase-like 2 (TARSL2), transcript variant X1, mRNA
ENSP00000278829-D3	-1.05	2.61E-04	↑	B. mutus fatty acid desaturase 3 (FADS3), partial mRNA
ENSBTAP00000013600-D1	-1.05	6.23E-03	↑	B. taurus dehydrogenase/reductase (SDR family) member 11 (DHRS11), mRNA
ENSBTAP00000003750-D2	-1.05	6.23E-03	↑	B. mutus DNA-damage inducible 1 homolog 2 (S. cerevisiae) (DDI2), mRNA
ENSP00000351040-D1	-1.05	6.23E-03	↑	B. mutus family with sequence similarity 217, member B (FAM217B), mRNA
ENSP00000349705-D1	-1.05	6.23E-03	↑	B. mutus topoisomerase (DNA) III beta (TOP3B), mRNA
ENSP00000299339-D1	-1.05	1.90E-50	↑	B. mutus claudin-10-like (LOC102277558), partial mRNA
ENSBTAP00000050174-D14	-1.05	1.16E-09	↑	B. taurus BAC CH240-5F6 complete sequence
ENSP00000418082-D6	-1.05	1.20E-48	↑	B. mutus ribosomal protein L37a (RPL37A), mRNA
ENSBTAP00000010654-D1	-1.05	1.43E-27	↑	B. mutus golgin A7 family, member B (GOLGA7B), mRNA
ENSBTAP00000026875-D1	-1.05	4.86E-08	↑	B. mutus chromosome unknown open reading frame, human C12orf10 (LOC102283703), mRNA
ENSP00000353427-D1	-1.05	4.86E-08	↑	B. mutus nuclear receptor subfamily 4, group A, member 1 (NR4A1), transcript variant X2, mRNA
ENSBTAP00000037133-D1	-1.05	2.61E-09	↑	B. mutus NOP2/Sun domain family, member 5 (NSUN5), transcript variant X1, mRNA
ENSP00000280193-D1	-1.05	7.67E-03	↑	B. mutus vascular endothelial growth factor C (VEGFC), mRNA
ENSP00000341483-D1	-1.05	2.57E-19	↑	B. mutus RAN binding protein 3 (RANBP3), mRNA
ENSP00000356595-D1	-1.05	2.08E-10	↑	B. taurus ABL proto-oncogene 2, non-receptor tyrosine kinase (ABL2), mRNA
ENSBTAP00000006938-D1	-1.05	7.28E-08	↑	B. mutus interleukin 1 receptor, type I (IL1R1), mRNA
ENSBTAP00000004187-D1	-1.05	1.35E-06	↑	B. mutus coiled-coil domain containing 115 (CCDC115), mRNA
ENSP00000394008-D1	-1.05	2.54E-05	↑	B. mutus GABA(A) receptor-associated protein like 1 (GABARAPL1), mRNA
ENSBTAP00000023886-D1	-1.05	4.06E-41	↑	B. mutus leucine-rich repeat-containing protein 51-like (LOC102274281), transcript variant X3, mRNA
ENSP00000379457-D1	-1.05	1.98E-22	↑	B. mutus Fas (TNFRSF6) associated factor 1 (FAF1), mRNA
ENSP00000359098-D5	-1.05	1.19E-66	↑	B. mutus cytochrome c oxidase subunit 7A2, mitochondrial-like (LOC102278351), mRNA

ENSP00000311399-D1	-1.05	1.30E-13	↑	B. mutus Down syndrome critical region gene 3 (DSCR3), transcript variant X1, mRNA
ENSP00000376472-D1	-1.05	1.33E-82	↑	B. mutus STT3A, subunit of the oligosaccharyltransferase complex (STT3A), transcript variant X2, mRNA
yakG045754	-1.05	9.41E-03	↑	.
ENSP00000294811-D1	-1.05	9.41E-03	↑	B. mutus chromosome unknown open reading frame, human C1orf74 (LOC102275820), mRNA
ENSBTAP00000003559-D2	-1.05	1.63E-164	↑	B. mutus APEX nuclease (multifunctional DNA repair enzyme) 1 (APEX1), mRNA
ENSP00000240423-D1	-1.05	4.06E-118	↑	B. mutus non-SMC condensin I complex, subunit H (NCAPH), mRNA
ENSP00000294008-D1	-1.04	2.93E-08	↑	B. mutus SLX4 structure-specific endonuclease subunit (SLX4), mRNA
ENSP00000276282-D1	-1.04	1.52E-11	↑	B. mutus malignant fibrous histiocytoma amplified sequence 1 (MFHAS1), partial mRNA
ENSBTAP00000053633-D1	-1.04	3.17E-19	↑	Bubalus bubalis ectonucleoside triphosphate diphosphohydrolase 4 (ENTPD4), transcript variant X2, mRNA
ENSBTAP00000009602-D1	-1.04	6.96E-05	↑	B. mutus testis expressed 29 (TEX29), mRNA
ENSBTAP00000053410-D1	-1.04	1.16E-02	↑	B. taurus ribosomal protein S6 kinase, 90kDa, polypeptide 2 (RPS6KA2), mRNA
ENSBTAP00000050872-D1	-1.04	1.16E-02	↑	B. taurus chromodomain helicase DNA binding protein 2, mRNA (cDNA clone IMAGE:8246925), complete cds
ENSP00000349962-D1	-1.04	1.16E-02	↑	B. mutus growth arrest-specific 6 (GAS6), mRNA
ENSP00000389130-D1	-1.04	4.75E-19	↑	B. taurus regulatory factor X, 5 (influences HLA class II expression) (RFX5), mRNA
ENSP00000362169-D1	-1.04	2.21E-12	↑	B. mutus family with sequence similarity 133, member A (FAM133A), mRNA
ENSP00000412461-D1	-1.04	2.17E-13	↑	B. mutus Rho GTPase activating protein 22 (ARHGAP22), transcript variant X1, mRNA
ENSBTAP00000052520-D51	-1.04	4.97E-26	↑	Bubalus bubalis zinc finger protein 624 (ZNF624), transcript variant X4, mRNA
ENSBTAP00000004433-D1	-1.04	1.09E-03	↑	B. mutus phosphorylase kinase, gamma 2 (testis) (PHKG2), mRNA
ENSBTAP00000022527-D1	-1.04	2.79E-40	↑	B. mutus chromosome unknown open reading frame, human C19orf67 (LOC102287370), mRNA
ENSP00000385642-D1	-1.04	1.96E-27	↑	B. mutus aryl hydrocarbon receptor-like (LOC102265507), mRNA
ENSP00000380293-D13	-1.04	1.28E-51	↑	B. taurus zinc finger protein 420 (ZNF420), mRNA
ENSP00000274459-D1	-1.04	2.86E-36	↑	B. mutus autophagy related 12 (ATG12), mRNA
ENSP00000372335-D1	-1.04	1.29E-04	↑	B. taurus negative elongation factor complex member A (NELFA), mRNA
ENSBTAP00000016040-D1	-1.04	1.43E-02	↑	B. mutus fibulin 1 (FBLN1), mRNA
ENSBTAP00000039420-D1	-1.04	1.54E-05	↑	B. mutus zinc finger protein 408 (ZNF408), mRNA
ENSBTAP00000016363-D1	-1.04	1.58E-04	↑	B. mutus zinc finger protein 524-like (LOC102264468), partial mRNA
ENSP00000399364-D1	-1.04	1.64E-03	↑	B. mutus ADAMTS-like 5 (ADAMTSL5), mRNA
ENSP00000335618-D1	-1.04	1.64E-03	↑	B. mutus phosphatidylinositol transfer protein, cytoplasmic 1 (PITPNC1), transcript variant X1, mRNA
ENSBTAP00000016549-D1	-1.04	1.88E-40	↑	B. mutus interleukin enhancer binding factor 2 (ILF2), transcript variant X1, mRNA
ENSBTAP00000005008-D1	-1.04	3.97E-08	↑	B. mutus cell adhesion molecule 1 (CADM1), mRNA
ENSP00000327309-D1	-1.04	6.91E-11	↑	B. mutus ADP-ribosylation factor GTPase activating protein 2 (ARFGAP2), transcript variant X1, mRNA
ENSBTAP00000025945-D1	-1.04	2.37E-04	↑	B. mutus N-acylethanolamine acid amidase (NAAA), mRNA
ENSBTAP00000031937-D2	-1.04	1.75E-02	↑	B. mutus enoyl CoA hydratase 1, peroxisomal (ECH1), mRNA
ENSBTAP00000044383-D1	-1.04	1.75E-02	↑	B. mutus SPRY domain containing 3 (SPRYD3), mRNA
ENSBTAP00000013971-D1	-1.03	3.44E-05	↑	B. mutus transmembrane protein 11 (TMEM11), mRNA
ENSP00000380362-D1	-1.03	3.04E-21	↑	B. mutus eukaryotic translation initiation factor 3, subunit D (EIF3D), mRNA
ENSBTAP00000012611-D1	-1.03	8.32E-16	↑	B. grunniens cell division cycle 25A (Cdc25A) mRNA, complete cds
ENSP00000366477-D1	-1.03	1.02E-15	↑	Bubalus bubalis chromosome unknown open reading frame, human C10orf112 (LOC102410644), partial mRNA
ENSBTAP00000014602-D1	-1.03	2.47E-03	↑	B. mutus histone deacetylase 10 (HDAC10), mRNA
ENSBTAP00000026387-D1	-1.03	0.00E+00	↑	B. mutus small nuclear ribonucleoprotein 25kDa (U11/U12) (SNRNP25), mRNA
ENSBTAP00000008947-D1	-1.03	3.56E-04	↑	B. mutus zinc finger protein 180 (ZNF180), mRNA
ENSBTAP00000006178-D1	-1.03	3.56E-04	↑	B. mutus suppressor of variegation 3-9 homolog 1 (Drosophila) (SUV39H1), mRNA
ENSBTAP00000021217-D1	-1.03	1.34E-07	↑	B. mutus golgi SNAP receptor complex member 2 (GOSR2), mRNA
ENSBTAP00000015764-D1	-1.03	1.96E-08	↑	B. mutus chromatin assembly factor 1, subunit B (p60) (CHAF1B), mRNA
ENSP00000376135-D63	-1.03	9.33E-11	↑	B. mutus zinc finger protein 614 (ZNF614), mRNA
ENSP00000309772-D1	-1.03	9.29E-06	↑	B. mutus cadherin-like and PC-esterase domain containing 1 (CPED1), mRNA
ENSP00000356954-D1	-1.03	4.35E-04	↑	B. mutus connective tissue growth factor-like (LOC102287187), mRNA
ENSP00000416177-D1	-1.03	3.03E-03	↑	B. mutus membrane-associated ring finger (C3HC4) 10, E3 ubiquitin protein ligase (MARCH10), mRNA
ENSBTAP00000042467-D5	-1.03	2.16E-02	↑	Bubalus bubalis histone H2A type 1-like (LOC102392571), mRNA
ENSP00000284601-D1	-1.03	2.16E-02	↑	B. mutus protein phosphatase 1, regulatory subunit 3A (PPP1R3A), mRNA
ENSBTAP00000000557-D1	-1.03	2.16E-02	↑	B. mutus tumor necrosis factor, alpha-induced protein 3 (TNFAIP3), mRNA
ENSP00000386105-D1	-1.03	5.29E-09	↑	B. mutus polymerase (DNA directed), delta 2, accessory subunit (POLD2), mRNA
ENSBTAP00000014131-D1	-1.03	1.58E-43	↑	B. mutus nuclear receptor subfamily 1, group H, member 3 (NR1H3), transcript variant X1, mRNA
ENSBTAP00000027625-D1	-1.03	9.49E-10	↑	Bubalus bubalis proline rich Gla (G-carboxyglutamic acid) 4 (transmembrane) (PRRG4), mRNA
ENSP00000265080-D1	-1.03	3.00E-16	↑	B. mutus Ras protein-specific guanine nucleotide-releasing factor 2 (RASGRF2), mRNA

ENSP00000328813-D1	-1.03	1.71E-10	↑	B. mutus potassium voltage-gated channel subfamily H member 8-like (LOC102270491), mRNA
ENSBTAP00000012029-D1	-1.03	3.07E-11	↑	B. mutus stromal antigen 2 (STAG2), transcript variant X1, mRNA
ENSBTAP00000002346-D1	-1.03	5.31E-04	↑	B. mutus hydroxysteroid (11-beta) dehydrogenase 1-like (HSD11B1L), transcript variant X1, mRNA
ENSBTAP00000049655-D1	-1.03	9.87E-13	↑	Bubalus bubalis nucleosome assembly protein 1-like 1 (NAP1L1), transcript variant X5, misc_RNA
ENSP00000326432-D1	-1.03	1.22E-16	↑	B. mutus chemokine (C-C motif) receptor 8 (CCR8), mRNA
ENSBTAP00000007903-D1	-1.02	6.53E-04	↑	B. mutus GTP binding protein 2 (GTPBP2), mRNA
ENSBTAP00000032330-D1	-1.02	8.03E-35	↑	B. mutus 2-oxoglutarate and iron-dependent oxygenase domain containing 1 (OGFOD1), mRNA
ENSP00000396538-D1	-1.02	7.08E-14	↑	B. mutus nuclear factor of activated T-cells 5, tonicity-responsive (NFAT5), mRNA
ENSBTAP00000007115-D1	-1.02	4.30E-19	↑	B. mutus CDC-like kinase 1 (CLK1), mRNA
ENSP00000394164-D1	-1.02	2.71E-82	↑	B. mutus general transcription factor IIH, polypeptide 2, 44kDa (GTF2H2), transcript variant X1, mRNA
ENSBTAP00000028113-D1	-1.02	2.77E-11	↑	B. mutus solute carrier family 35, member F5 (SLC35F5), mRNA
ENSP00000206595-D1	-1.02	8.19E-07	↑	B. mutus G2/M-phase specific E3 ubiquitin protein ligase (G2E3), mRNA
ENSBTAP00000006504-D1	-1.02	4.59E-03	↑	B. mutus annexin A10 (ANXA10), mRNA
ENSBTAP00000053593-D1	-1.02	6.27E-15	↑	B. mutus calcium channel, voltage-dependent, R type, alpha 1E subunit (CACNA1E), transcript variant X1, mRNA
ENSBTAP00000015719-D1	-1.02	6.07E-12	↑	B. mutus arsA arsenite transporter, ATP-binding, homolog 1 (bacterial) (ASNA1), mRNA
ENSBTAP00000027669-D1	-1.02	1.81E-07	↑	Bubalus bubalis F-box only protein 27-like (LOC102402343), mRNA
ENSP00000377751-D1	-1.02	9.76E-18	↑	B. mutus short coiled-coil protein (SCOC), transcript variant X1, mRNA
ENSBTAP00000025136-D1	-1.02	1.15E-14	↑	B. mutus dynactin 3 (p22) (DCTN3), transcript variant X1, mRNA
ENSP00000265689-D1	-1.02	9.78E-04	↑	B. mutus choline kinase alpha (CHKA), partial mRNA
ENSBTAP00000049457-D14	-1.02	7.14E-64	↑	B. mutus zinc finger and SCAN domain containing 12 (ZSCAN12), mRNA
ENSP00000349770-D2	-1.02	1.48E-16	↑	B. mutus interferon regulatory factor 6 (IRF6), transcript variant X3, mRNA
ENSP00000005905-D1	-1.02	1.07E-08	↑	B. mutus KIAA0100 ortholog (KIAA0100), mRNA
ENSBTAP00000024060-D1	-1.02	2.14E-04	↑	B. mutus translocator protein (18kDa) (TSPO), mRNA
ENSBTAP00000014649-D1	-1.02	5.61E-03	↑	B. mutus prostaglandin reductase 1 (PTGR1), mRNA
ENSBTAP00000016560-D1	-1.02	4.03E-07	↑	B. mutus CUB domain containing protein 1 (CDCP1), mRNA
ENSP00000266534-D1	-1.02	1.20E-03	↑	B. mutus transmembrane protein 117-like (LOC102277991), partial mRNA
ENSP00000196489-D14	-1.02	1.99E-16	↑	B. mutus zinc finger protein 211 (ZNF211), mRNA
ENSP00000361900-D1	-1.01	2.04E-15	↑	B. mutus translocase of outer mitochondrial membrane 34 (TOMM34), mRNA
ENSP00000263187-D1	-1.01	3.36E-06	↑	B. mutus mutS homolog 4 (MSH4), mRNA
ENSP00000251289-D1	-1.01	3.20E-04	↑	B. mutus WD repeat domain 18 (WDR18), mRNA
ENSP00000371175-D1	-1.01	6.88E-03	↑	B. mutus golgi-associated, gamma adaptin ear containing, ARF binding protein 1, transcript variant X1, mRNA
ENSP00000370242-D1	-1.01	6.88E-03	↑	B. mutus WWC family member 3 (WWC3), mRNA
ENSBTAP00000010198-D1	-1.01	6.88E-03	↑	B. mutus pre-mRNA processing factor 31 (PRPF31), mRNA
ENSP00000381047-D1	-1.01	3.35E-02	↑	B. mutus chromosome unknown open reading frame, human C21orf2 (LOC102265558), mRNA
ENSBTAP00000011670-D1	-1.01	3.35E-02	↑	B. mutus lymphocyte antigen 96 (LY96), mRNA
ENSBTAP00000025895-D1	-1.01	2.91E-13	↑	B. mutus mediator complex subunit 28 (MED28), mRNA
ENSBTAP00000004655-D1	-1.01	9.12E-25	↑	B. mutus deleted in primary ciliary dyskinesia homolog (mouse) (DPCD), mRNA
ENSBTAP00000018540-D1	-1.01	1.52E-118	↑	B. taurus heterogeneous nuclear ribonucleoprotein D (HNRNPD), transcript variant X2, misc_RNA
ENSP00000223167-D2	-1.01	1.81E-03	↑	B. mutus myosin light chain, phosphorylatable, fast skeletal muscle (MYLPF), mRNA
ENSP00000399970-D3	-1.01	1.23E-24	↑	B. mutus protein phosphatase 2, regulatory subunit B, beta (PPP2R2B), partial mRNA
ENSP00000274068-D2	-1.01	1.79E-51	↑	Bubalus bubalis ring finger protein 121 (RNF121), transcript variant X2, mRNA
ENSBTAP00000027191-D1	-1.01	3.11E-142	↑	B. mutus NADH dehydrogenase (ubiquinone) 1 beta subcomplex, 9, 22kDa (NDUFB9), mRNA
ENSBTAP00000021852-D1	-1.01	2.96E-21	↑	B. mutus Fanconi anemia, complementation group B (FANCB), mRNA
ENSP00000363391-D1	-1.01	8.52E-03	↑	B. mutus sperm associated antigen 4 (SPAG4), transcript variant X1, mRNA
ENSBTAP00000041953-D1	-1.01	7.09E-09	↑	B. mutus leucine rich repeat containing 2 (LRRC2), mRNA
ENSP00000302843-D1	-1.01	5.95E-225	↑	B. mutus DnaJ (Hsp40) homolog, subfamily C, member 18 (DNAJC18), transcript variant X1, mRNA
ENSP00000294904-D1	-1.01	2.06E-56	↑	B. mutus RNA binding motif, single stranded interacting protein 1 (RBMS1), transcript variant X2, mRNA
ENSP00000376794-D1	-1.01	2.22E-03	↑	B. mutus solute carrier family 35, member G2 (SLC35G2), mRNA
ENSP00000258411-D1	-1.01	2.22E-03	↑	B. mutus wingless-type MMTV integration site family, member 10A (WNT10A), mRNA
ENSP00000310042-D1	-1.01	6.22E-53	↑	B. mutus zinc finger protein 622 (ZNF622), mRNA
ENSBTAP00000046060-D1	-1.01	5.86E-04	↑	B. mutus SURP and G patch domain containing 1 (SUGP1), mRNA
ENSBTAP00000025950-D1	-1.01	1.57E-04	↑	B. mutus claudin-12-like (LOC102274827), mRNA
ENSBTAP00000043609-D1	-1.00	2.39E-21	↑	Bubalus bubalis exocyst complex component 1 (EXOC1), transcript variant X5, mRNA
ENSP00000354718-D1	-1.00	1.82E-11	↑	B. mutus A kinase (PRKA) anchor protein 13 (AKAP13), mRNA
ENSP00000296043-D1	-1.00	9.41E-14	↑	B. mutus shroom family member 3 (SHROOM3), mRNA

ENSP00000226319-D1	-1.00	3.84E-34	↑	Bubalus bubalis jade family PHD finger 1 (JADE1), transcript variant X7, mRNA
ENSP00000400799-D1	-1.00	1.93E-08	↑	B. mutus solute carrier family 45, member 4 (SLC45A4), mRNA
ENSP00000289269-D1	-1.00	2.72E-03	↑	B. mutus protocadherin alpha subfamily C, 2 (PCDHAC2), mRNA
ENSP00000293925-D1	-1.00	2.72E-03	↑	B. mutus Crm, cramped-like (Drosophila) (CRAMP1L), mRNA
ENSBTAP00000014258-D1	-1.00	1.04E-02	↑	B. mutus tubulin polyglutamylase complex subunit 1 (TPGS1), partial mRNA
ENSBTAP00000028210-D1	-1.00	1.04E-02	↑	B. mutus sirtuin 4 (SIRT4), mRNA
ENSBTAP00000019966-D1	-1.00	1.04E-02	↑	B. mutus WD repeat domain 62 (WDR62), mRNA
ENSBTAP00000024412-D1	-1.00	4.19E-02	↑	B. mutus interleukin 33 (IL33), mRNA
ENSBTAP00000010835-D1	-1.00	4.19E-02	↑	B. mutus toll interacting protein (TOLLIP), mRNA
ENSP00000311038-D89	-1.00	4.19E-02	↑	B. mutus olfactory receptor 4X2-like (LOC102273298), mRNA
ENSBTAP00000020019-D1	-1.00	4.19E-02	↑	Bubalus bubalis family with sequence similarity 203, member A (FAM203A), mRNA
ENSBTAP00000030408-D1	-1.00	4.19E-02	↑	B. mutus tocopherol (alpha) transfer protein (TTPA), mRNA
ENSBTAP00000029970-D1	-1.00	4.19E-02	↑	B. taurus nudix (nucleoside diphosphate linked moiety X)-type motif 10 (NUDT10), mRNA
ENSBTAP00000017304-D1	-1.00	4.19E-02	↑	B. mutus coiled-coil domain containing 13 (CCDC13), mRNA
ENSP00000364126-D1	-1.00	6.42E-13	↑	B. mutus APEX nuclease (apurinic/apyrimidinic endonuclease) 2 (APEX2), transcript variant X1, mRNA
ENSBTAP00000020228-D1	-1.00	2.85E-22	↑	B. mutus ATP synthase, H+ transporting, mitochondrial Fo complex, subunit s (factor B) (ATP5S), mRNA
ENSP00000269829-D55	-1.00	1.69E-05	↑	B. mutus zinc finger protein 789 (ZNF789), transcript variant X2, mRNA
ENSBTAP00000051337-D10	-1.00	2.36E-29	↑	B. mutus hemoglobin subunit alpha-like (LOC102268919), mRNA
ENSP00000351446-D2	-1.00	1.41E-143	↑	B. mutus WD repeat domain 5 (WDR5), mRNA
ENSP00000307604-D1	-1.00	5.82E-17	↑	B. mutus zinc finger and BTB domain containing 5 (ZBTB5), mRNA
ENSP00000295101-D1	-1.00	5.54E-06	↑	B. mutus potassium inwardly-rectifying channel, subfamily J, member 3 (KCNJ3), transcript variant X1, mRNA
ENSBTAP00000005575-D1	1.00	0.00E+00	↓	B. mutus ornithine decarboxylase-like (LOC102276634), mRNA
ENSP00000311224-D5	1.00	1.01E-03	↓	Pantholops hodgsonii bile acid CoA: amino acid N-acyltransferase (glycine N-choloyltransferase) (BAAT), mRNA
ENSP00000301924-D1	1.00	4.62E-02	↓	Bubalus bubalis tripartite motif containing 35 (TRIM35), mRNA
ENSBTAP00000001499-D1	1.00	4.62E-02	↓	B. mutus ring finger protein 165 (RNF165), mRNA
ENSBTAP00000051198-D1	1.00	4.62E-02	↓	B. mutus hyaluronoglucosaminidase 2 (HYAL2), mRNA
ENSP00000362465-D1	1.00	4.62E-02	↓	B. mutus zinc finger, DHHC-type containing 15 (ZDHHC15), transcript variant X1, mRNA
ENSBTAP00000026114-D1	1.00	0.00E+00	↓	B. mutus oocyte expressed protein (OOEP), mRNA
ENSBTAP00000009974-D1	1.01	1.16E-10	↓	B. mutus adenosine A2b receptor (ADORA2B), mRNA
ENSP00000366384-D1	1.01	1.26E-05	↓	B. mutus zinc finger protein 311 (ZNF311), mRNA
ENSBTAP00000027362-D1	1.01	1.26E-05	↓	B. mutus HECT and RLD domain containing E3 ubiquitin protein ligase family member 6 (HERC6), mRNA
ENSP00000233997-D1	1.01	1.43E-02	↓	B. mutus azurocidin 1 (AZU1), mRNA
ENSBTAP00000041951-D1	1.01	1.43E-02	↓	B. taurus zinc finger, FYVE domain containing 21 (ZFYVE21), mRNA
ENSBTAP00000047583-D1	1.01	1.43E-02	↓	B. mutus complement component 7 (C7), mRNA
ENSBTAP00000050715-D1	1.01	1.43E-02	↓	B. mutus chromosome unknown open reading frame, human C15orf43 (LOC102284958), partial mRNA
ENSP00000383938-D1	1.01	2.19E-31	↓	B. mutus cysteine-rich with EGF-like domains 2 (CRELD2), mRNA
ENSP00000306344-D1	1.01	5.43E-07	↓	B. mutus transmembrane protein 135-like (LOC102283432), mRNA
ENSBTAP00000016562-D1	1.01	1.53E-03	↓	B. mutus Ran GTPase activating protein 1 (RANGAP1), mRNA
ENSBTAP00000000364-D1	1.02	1.30E-17	↓	B. mutus dehydrogenase E1 and transketolase domain containing 1 (DHTKD1), mRNA
ENSBTAP00000024022-D2	1.02	1.76E-03	↓	B. mutus UDP-GlcNAc:betaGal beta-1,3-N-acetylglucosaminyltransferase 2 (B3GNT2), mRNA
ENSBTAP00000041190-D1	1.02	5.36E-03	↓	B. mutus coiled-coil-helix-coiled-coil-helix domain containing 6 (CHCHD6), mRNA
ENSBTAP00000036828-D1	1.02	1.63E-02	↓	B. mutus sodium/hydrogen exchanger 2-like (LOC102284519), mRNA
ENSP00000320445-D1	1.02	2.49E-05	↓	B. mutus CUB and Sushi multiple domains 1 (CSMD1), mRNA
ENSBTAP00000025159-D1	1.02	4.64E-08	↓	B. taurus adrenocortical dysplasia homolog (mouse), mRNA (cDNA clone IMAGE:8290235), partial cds
ENSP00000344820-D1	1.02	2.85E-05	↓	B. mutus potassium channel, subfamily K, member 7 (KCNK7), mRNA
ENSBTAP00000018635-D1	1.02	2.02E-03	↓	B. mutus diphosphoinositol pentakisphosphate kinase 1 (PPIP5K1), transcript variant X1, mRNA
ENSBTAP00000025389-D1	1.03	1.40E-06	↓	B. mutus peripheral myelin protein 22 (PMP22), mRNA
ENSBTAP00000005416-D1	1.03	5.37E-07	↓	Bubalus bubalis BTB and CNC homology 1, basic leucine zipper transcription factor 1 (BACH1), mRNA
ENSBTAP00000007496-D1	1.03	7.86E-42	↓	B. mutus abhydrolase domain containing 3 (ABHD3), mRNA
ENSBTAP00000040579-D10	1.03	1.88E-02	↓	B. mutus zinc finger protein 829-like (LOC102269603), mRNA
ENSBTAP00000028257-D1	1.03	1.88E-02	↓	B. mutus phosphatidic acid phosphatase type 2 domain containing 3 (PPAPDC3), mRNA
ENSP00000323901-D1	1.03	1.88E-02	↓	B. mutus frizzled family receptor 2 (FZD2), partial mRNA
ENSP00000377298-D1	1.03	1.12E-04	↓	B. taurus nuclear mitotic apparatus protein 1 (NUMA1), transcript variant X4, mRNA
ENSP00000335620-D3	1.03	7.39E-17	↓	B. mutus glutathione S-transferase A1-like (LOC102274807), partial mRNA
ENSBTAP00000018079-D1	1.04	4.57E-14	↓	B. mutus nuclear receptor subfamily 1, group H, member 4 (NR1H4), transcript variant X1, mRNA

ENSP00000259698-D1	1.04	2.46E-05	↓	B. mutus protein FAM65B-like (LOC102273075), mRNA
ENSBTAP00000004133-D12	1.04	3.05E-03	↓	Capra hircus vomeronasal type-1 receptor 2-like (LOC102191644), mRNA
ENSBTAP00000009880-D1	1.04	2.17E-02	↓	B. taurus fatty acid amide hydrolase (FAAH), mRNA
ENSBTAP00000002191-D1	1.04	0.00E+00	↓	B. mutus Rtf1, Paf1/RNA polymerase II complex component, homolog (S. cerevisiae) (RTF1), mRNA
ENSBTAP00000048702-D1	1.05	1.73E-08	↓	B. mutus adaptor-related protein complex 1, sigma 3 subunit (AP1S3), mRNA
ENSBTAP00000037502-D1	1.05	1.73E-08	↓	B. mutus insulin-like growth factor 2 receptor (IGF2R), mRNA
ENSBTAP00000017147-D1	1.05	5.74E-04	↓	B. mutus interferon gamma receptor 2 (interferon gamma transducer 1) (IFNGR2), mRNA
ENSP00000340465-D1	1.05	9.30E-03	↓	B. mutus solute carrier family 20 (phosphate transporter), member 2 (SLC20A2), mRNA
ENSBTAP00000025929-D1	1.05	9.30E-03	↓	B. mutus aarF domain containing kinase 4 (ADCK4), transcript variant X2, mRNA
ENSBTAP00000053780-D1	1.05	9.30E-03	↓	B. mutus dedicator of cytokinesis 3 (DOCK3), partial mRNA
ENSP00000218056-D1	1.05	2.44E-92	↓	B. mutus WD repeat domain 13 (WDR13), transcript variant X1, mRNA
ENSP00000349923-D1	1.05	8.02E-12	↓	B. mutus L antigen family, member 3 (LAGE3), mRNA
ENSBTAP00000014880-D1	1.05	1.55E-52	↓	B. mutus ninjurin 1 (NINJ1), mRNA
ENSBTAP00000023221-D1	1.05	2.00E-277	↓	B. mutus nucleobindin 2 (NUCB2), transcript variant X2, mRNA
ENSBTAP00000044044-D1	1.05	7.07E-06	↓	B. mutus abhydrolase domain containing 17C (ABHD17C), mRNA
ENSBTAP00000053503-D1	1.06	7.13E-11	↓	B. mutus colorectal cancer associated 2 (COLCA2), mRNA
ENSP00000280758-D1	1.06	4.01E-03	↓	B. mutus BTB (POZ) domain containing 11 (BTBD11), mRNA
ENSBTAP00000005162-D1	1.06	2.50E-02	↓	B. mutus transmembrane protein 220 (TMEM220), mRNA
ENSP00000343507-D1	1.06	2.50E-02	↓	B. mutus transmembrane protein 198 (TMEM198), transcript variant X2, mRNA
ENSBTAP00000012628-D1	1.06	4.00E-104	↓	B. mutus neuroguidin, EIF4E binding protein (NGDN), mRNA
ENSP00000372527-D1	1.06	2.11E-05	↓	B. mutus transmembrane 4 L6 family member 1-like (LOC102285510), mRNA
ENSBTAP00000028292-D1	1.06	5.06E-08	↓	B. mutus HLA class II histocompatibility antigen, DRB1-4 beta chain-like (LOC102271117), misc_RNA
ENSP00000348216-D1	1.06	4.62E-03	↓	Bubalus bubalis tripartite motif containing 4 (TRIM4), transcript variant X1, mRNA
ENSBTAP00000052934-D1	1.07	2.89E-08	↓	B. mutus WAP four-disulfide core domain 3 (WFDC3), mRNA
ENSP00000245812-D1	1.07	5.05E-97	↓	B. mutus alkB, alkylation repair homolog 7 (E. coli) (ALKBH7), mRNA
ENSBTAP00000016791-D1	1.07	2.90E-02	↓	B. mutus ovo-like zinc finger 1 (OVOL1), mRNA
ENSP00000355089-D2	1.07	2.90E-02	↓	B. mutus CUGBP, Elav-like family member 3 (CELF3), transcript variant X1, mRNA
ENSP00000353074-D37	1.07	2.90E-02	↓	B. mutus uncharacterized LOC102265545 (LOC102265545), mRNA
ENSBTAP00000016718-D4	1.07	8.92E-15	↓	B. mutus heat-responsive protein 12 (HRSP12), mRNA
ENSP00000359640-D1	1.08	9.19E-10	↓	B. mutus mucolipin 2 (MCOLN2), mRNA
ENSBTAP00000007030-D1	1.08	1.01E-10	↓	B. mutus tryptophan hydroxylase 1 (TPH1), mRNA
ENSBTAP00000009938-D1	1.08	3.33E-07	↓	B. mutus potassium voltage-gated channel, shaker-related subfamily, member 5 (KCNA5), mRNA
ENSP00000350881-D1	1.08	2.15E-04	↓	B. mutus GRAM domain containing 1C (GRAMD1C), transcript variant X2, mRNA
ENSP00000064778-D1	1.08	4.91E-04	↓	Capra hircus family with sequence similarity 168, member A (FAM168A), transcript variant X1, mRNA
ENSBTAP00000011702-D1	1.08	1.13E-03	↓	B. mutus progestin and adipoQ receptor family member III (PAQR3), mRNA
ENSBTAP00000010864-D1	1.08	3.00E-18	↓	Bubalus bubalis exophilin 5 (EXPH5), transcript variant X6, mRNA
ENSP00000376021-D1	1.08	6.04E-03	↓	B. mutus THAP domain containing 8 (THAP8), mRNA
ENSP00000395100-D1	1.08	2.23E-52	↓	B. mutus TNFAIP3 interacting protein 3 (TNIP3), mRNA
yakA06437	1.09	1.41E-02	↓	B. mutus late histone H2B.2.2-like (LOC102285091), mRNA
ENSBTAP00000014920-D1	1.09	2.49E-28	↓	B. mutus forkhead box O3 (FOXO3), transcript variant X1, mRNA
ENSP00000367330-D1	1.10	2.37E-58	↓	B. mutus chromosome unknown open reading frame, human C12orf73, transcript variant X2, mRNA
ENSP00000398560-D1	1.10	3.20E-04	↓	B. mutus spermatogenesis-associated protein 13-like (LOC102279512), partial mRNA
ENSP00000325827-D1	1.10	1.47E-03	↓	B. mutus SEC16 homolog A (S. cerevisiae) (SEC16A), mRNA
ENSBTAP00000015586-D1	1.10	3.35E-02	↓	B. mutus insulin-like growth factor 2 mRNA binding protein 1 (IGF2BP1), transcript variant X1, mRNA
ENSBTAP00000006543-D1	1.10	3.35E-02	↓	B. mutus leucine rich repeat containing 24 (LRRC24), mRNA
ENSBTAP00000052197-D2	1.10	2.58E-47	↓	B. mutus C-x(9)-C motif containing 2 (CMC2), mRNA
ENSP00000161863-D1	1.10	2.88E-13	↓	B. mutus probable ATP-dependent RNA helicase YTHDC2-like (LOC102271076), mRNA
ENSBTAP00000041952-D1	1.10	4.11E-44	↓	B. mutus synaptonemal complex protein 2-like (SYCP2L), mRNA
ENSP00000300057-D1	1.10	4.37E-06	↓	B. mutus mesoderm posterior 1 homolog (mouse) (MESP1), partial mRNA
ENSP00000264552-D1	1.10	1.87E-13	↓	B. taurus ubiquitin-conjugating enzyme E2S (UBE2S), mRNA
yakG015969	1.11	1.61E-02	↓	Bubalus bubalis protocadherin-related 15 (PCDH15), mRNA
ENSBTAP00000044555-D1	1.11	5.34E-100	↓	Capra hircus coiled-coil domain-containing protein 162-like (LOC102179006), mRNA
ENSP00000395774-D1	1.11	7.91E-03	↓	B. mutus kelch-like family member 35 (KLHL35), mRNA
ENSP00000270112-D1	1.11	2.08E-04	↓	B. mutus hormonally up-regulated Neu-associated kinase (HUNK), partial mRNA
ENSP00000405818-D1	1.12	2.62E-46	↓	B. mutus NUAK family, SNF1-like kinase, 2 (NUAK2), mRNA

ENSP00000383958-D2	1.12	4.68E-13	↓	B. taurus nuclear factor I/A (NFIA), mRNA
ENSP00000310303-D1	1.12	4.32E-14	↓	Bubalus bubalis testis and ovary specific PAZ domain containing 1 (TOPAZ1), mRNA
ENSP00000304108-D1	1.13	2.19E-03	↓	B. mutus family with sequence similarity 171, member B (FAM171B), mRNA
ENSP00000257408-D1	1.13	2.19E-03	↓	B. mutus klotho beta (KLB), mRNA
ENSBTAP00000052271-D1	1.13	1.83E-02	↓	B. mutus transketolase-like 2 (TKTL2), transcript variant X1, mRNA
ENSBTAP00000001851-D1	1.13	1.83E-02	↓	B. mutus hemK methyltransferase family member 2-like (LOC102272049), transcript variant X1, mRNA
ENSBTAP00000022073-D1	1.13	1.83E-02	↓	B. mutus phosphatidylinositol glycan anchor biosynthesis, class Q (PIGQ), mRNA
ENSBTAP00000018774-D1	1.13	3.89E-02	↓	B. mutus prostaglandin-endoperoxide synthase 2 (PTGS2), transcript variant X1, mRNA
ENSP00000400153-D2	1.13	3.89E-02	↓	B. mutus uncharacterized LOC102284826 (LOC102284826), mRNA
ENSBTAP00000011846-D1	1.13	3.89E-02	↓	B. mutus endoglin (ENG), mRNA
ENSP00000238508-D4	1.13	3.89E-02	↓	B. mutus serpin peptidase inhibitor, clade B (ovalbumin), member 9 (SERPINB9), transcript variant X1, mRNA
ENSP00000409016-D1	1.14	1.92E-80	↓	B. mutus transforming growth factor beta regulator 1 (TBRG1), mRNA
ENSBTAP00000016739-D1	1.14	7.43E-22	↓	B. mutus ganglioside induced differentiation associated protein 1 (GDAP1), transcript variant X1, mRNA
ENSBTAP00000040741-D1	1.14	1.75E-04	↓	Bubalus bubalis ATP/GTP binding protein-like 4 (AGBL4), mRNA
yakG006826	1.14	1.13E-09	↓	B. mutus empty spiracles homeobox 1 (EMX1), mRNA
ENSP00000264029-D1	1.15	1.41E-03	↓	B. mutus trehalase (brush-border membrane glycoprotein) (TREH), mRNA
ENSBTAP00000008068-D2	1.15	1.41E-03	↓	B. mutus dynein, light chain, roadblock-type 2 (DYNLRB2), mRNA
yakA09427	1.15	2.09E-02	↓	B. mutus Ral GEF with PH domain and SH3 binding motif 1 (RALGPS1), mRNA
ENSP00000357076-D1	1.15	1.13E-04	↓	B. mutus transgelin 2 (TAGLN2), mRNA
ENSP00000350415-D1	1.16	7.95E-04	↓	B. mutus gap junction protein, alpha 9, 59kDa (GJA9), mRNA
ENSBTAP00000047915-D1	1.16	5.00E-19	↓	B. mutus histone H3.1-like (LOC102280563), mRNA
ENSP00000363925-D1	1.16	0.00E+00	↓	B. mutus structural maintenance of chromosomes protein 2-like (LOC102264480), mRNA
ENSP00000071281-D1	1.17	2.07E-05	↓	B. mutus four and a half LIM domains 1 (FHL1), transcript variant X1, mRNA
ENSBTAP00000034763-D1	1.17	2.57E-04	↓	B. mutus methyltransferase-like protein 7A-like (LOC102270564), mRNA
ENSBTAP00000041125-D1	1.17	3.24E-03	↓	B. mutus trefoil factor 2-like (LOC102282879), mRNA
ENSBTAP00000004192-D1	1.17	1.17E-02	↓	B. mutus RAB7, member RAS oncogene family-like 1 (RAB7L1), transcript variant X1, mRNA
ENSBTAP00000029033-D1	1.17	1.17E-02	↓	B. mutus selenoprotein N, 1 (SEPN1), mRNA
ENSBTAP00000005415-D1	1.17	4.47E-02	↓	Bubalus bubalis glioma tumor suppressor candidate region gene 1 (GLTSCR1), mRNA
ENSBTAP00000013171-D1	1.17	4.47E-02	↓	B. mutus calcium activated nucleotidase 1 (CANT1), mRNA
ENSP00000281523-D2	1.17	4.47E-02	↓	B. mutus zinc finger protein 385D (ZNF385D), mRNA
ENSBTAP00000023347-D1	1.17	4.47E-02	↓	B. mutus HHIP-like 2 (HHIPL2), mRNA
ENSBTAP00000050011-D1	1.17	4.11E-05	↓	B. mutus zinc finger protein 135-like (LOC102268393), mRNA
ENSBTAP00000040669-D1	1.17	5.13E-04	↓	Capra hircus coiled-coil domain-containing protein 136-like (LOC102183655), mRNA
ENSBTAP00000016098-D1	1.17	1.83E-03	↓	B. mutus diphthamide biosynthesis 7 (DPH7), mRNA
ENSBTAP00000005160-D1	1.18	6.59E-03	↓	B. taurus F-box and leucine-rich repeat protein 2 (FBXL2), mRNA
ENSBTAP00000006736-D3	1.18	6.59E-03	↓	B. mutus slit homolog 1 (Drosophila) (SLIT1), mRNA
ENSBTAP00000050085-D1	1.18	1.03E-03	↓	B. mutus regulator of G-protein signaling 21 (RGS21), mRNA
ENSP00000261692-D1	1.18	6.42E-59	↓	B. mutus cyclin-dependent kinase 2 associated protein 1 (CDK2AP1), mRNA
ENSP00000245796-D1	1.18	9.36E-05	↓	B. mutus pleckstrin and Sec7 domain containing 4 (PSD4), mRNA
ENSP00000222139-D1	1.18	3.69E-03	↓	B. mutus erythropoietin receptor (EPOR), mRNA
ENSP00000322594-D2	1.18	2.40E-02	↓	B. mutus RAB39B, member RAS oncogene family (RAB39B), mRNA
ENSBTAP00000025435-D1	1.18	2.40E-02	↓	B. mutus chromosome unknown open reading frame, human C17orf70 (LOC102265744), mRNA
ENSP00000401466-D1	1.19	8.59E-06	↓	Pantholops hodgsonii tripartite motif containing 66 (TRIM66), mRNA
ENSP00000382084-D1	1.19	2.08E-03	↓	B. taurus THAP domain containing 7 (THAP7), mRNA
ENSBTAP00000037377-D1	1.19	3.28E-40	↓	B. mutus N-methylpurine-DNA glycosylase (MPG), mRNA
ENSBTAP00000048026-D1	1.20	6.00E-05	↓	B. mutus CD3e molecule, epsilon (CD3-TCR complex) (CD3E), mRNA
ENSP00000288840-D1	1.20	6.00E-05	↓	B. taurus SMAD family member 6 (SMAD6), mRNA
ENSBTAP00000043140-D1	1.20	7.48E-03	↓	B. mutus transmembrane protein 64 (TMEM64), mRNA
ENSP00000419204-D1	1.20	7.48E-03	↓	B. mutus tetratricopeptide repeat domain 6 (TTC6), mRNA
ENSBTAP00000009887-D1	1.20	9.98E-36	↓	B. mutus phosphoglycerate mutase family member 5 (PGAM5), mRNA
ENSP00000378495-D1	1.20	8.86E-13	↓	B. mutus chromosome unknown open reading frame, human C10orf35 (LOC102267331), mRNA
ENSBTAP00000053832-D1	1.21	1.14E-06	↓	B. mutus sterile alpha motif domain containing 9 (SAMD9), mRNA
ENSBTAP00000023059-D1	1.21	2.36E-03	↓	B. mutus kinesin-like protein KIF2B-like (LOC102272172), mRNA
ENSBTAP00000040176-D1	1.21	2.36E-03	↓	B. mutus apolipoprotein H (beta-2-glycoprotein I) (APOH), mRNA
yakA06234	1.21	2.36E-03	↓	.

ENSP00000290776-D1	1.21	9.86E-16	↓	B. mutus copine II (CPNE2), mRNA
ENSP00000331741-D1	1.21	7.49E-04	↓	B. mutus homeobox B2 (HOXB2), mRNA
ENSBTAP00000053020-D5	1.22	1.30E-68	↓	B. mutus histone H3.1-like (LOC102280001), mRNA
ENSBTAP00000053708-D1	1.22	8.38E-23	↓	Bubalus bubalis chromosome unknown open reading frame, human C11orf31 (LOC102393693), mRNA
ENSP00000335463-D1	1.22	2.37E-208	↓	B. mutus cancer susceptibility candidate 5 (CASC5), mRNA
ENSBTAP00000024424-D4	1.22	2.79E-26	↓	B. mutus ribosomal protein S23 (RPS23), mRNA
ENSP00000248673-D1	1.23	3.56E-11	↓	B. mutus ZFP36 ring finger protein (ZFP36), mRNA
ENSP00000363537-D1	1.23	1.72E-07	↓	B. mutus ADAM metallopeptidase domain 23 (ADAM23), mRNA
ENSP00000272771-D1	1.23	1.54E-04	↓	B. mutus transmembrane protein with EGF-like and two follistatin-like domains 2 (TMEFF2), mRNA
ENSP00000207870-D1	1.23	4.74E-03	↓	B. mutus xylulokinase homolog (H. influenzae) (XYLB), mRNA
ENSP00000258381-D1	1.23	8.52E-03	↓	Pantholops hodgsonii SP110 nuclear body protein (SP110), mRNA
ENSBTAP00000013281-D1	1.23	1.52E-02	↓	B. taurus receptor accessory protein 2 (REEP2), mRNA
ENSP00000396214-D1	1.23	1.52E-02	↓	B. mutus STEAP family member 3, metalloreductase (STEAP3), mRNA
ENSP00000308725-D1	1.23	1.52E-02	↓	B. mutus gamma-aminobutyric acid (GABA) A receptor, beta 3 (GABRB3), mRNA
ENSBTAP00000023729-D1	1.23	2.74E-02	↓	B. mutus retinoid X receptor, alpha (RXRA), mRNA
ENSBTAP00000009404-D1	1.23	2.74E-02	↓	B. mutus carboxypeptidase, vitellogenic-like (CPVL), mRNA
ENSBTAP00000018740-D5	1.23	2.74E-02	↓	B. mutus crystallin, gamma B (CRYGB), mRNA
ENSBTAP00000016414-D1	1.23	2.74E-02	↓	B. mutus matrix Gla protein (MGP), mRNA
ENSBTAP00000024586-D1	1.23	2.74E-02	↓	B. mutus macrophage receptor with collagenous structure (MARCO), mRNA
ENSBTAP00000021843-D1	1.23	1.25E-10	↓	B. mutus matrix metallopeptidase 25 (MMP25), mRNA
ENSP00000349932-D1	1.24	9.84E-05	↓	Bubalus bubalis protein tyrosine phosphatase, receptor type, S (PTPRS), transcript variant X7, mRNA
ENSBTAP00000028278-D2	1.25	7.63E-07	↓	B. mutus zinc finger, SWIM-type containing 5 (ZSWIM5), mRNA
ENSBTAP00000009939-D1	1.25	1.51E-123	↓	B. mutus interferon, alpha-inducible protein 6 (IFI6), mRNA
ENSBTAP00000010776-D1	1.25	5.37E-03	↓	Bubalus bubalis lipid phosphate phosphatase-related protein type 2-like, transcript variant X1, mRNA
ENSP00000286657-D1	1.25	5.37E-03	↓	B. mutus ADAM metallopeptidase with thrombospondin type 1 motif, 3 (ADAMTS3), mRNA
ENSP00000320672-D1	1.26	4.00E-05	↓	B. mutus cytochrome c oxidase subunit 6B2-like (LOC102286900), mRNA
ENSBTAP00000000499-D1	1.26	9.58E-03	↓	B. taurus zinc finger protein 212 (ZNF212), transcript variant X1, mRNA
ENSBTAP00000008061-D1	1.26	4.70E-06	↓	B. mutus general transcription factor IIIC, polypeptide 1, alpha 220kDa (GTF3C1), partial mRNA
ENSBTAP00000019111-D1	1.26	0.00E+00	↓	B. mutus centrin, EF-hand protein, 3 (CETN3), mRNA
ENSP00000264031-D1	1.27	1.70E-02	↓	B. mutus uroplakin 2 (UPK2), mRNA
ENSBTAP00000038865-D1	1.27	1.70E-02	↓	B. mutus PC-esterase domain containing 1B (PCED1B), mRNA
ENSP00000407761-D1	1.27	3.89E-04	↓	B. mutus uncharacterized LOC102287687 (LOC102287687), mRNA
ENSBTAP00000018498-D1	1.28	6.86E-04	↓	B. mutus WAP four-disulfide core domain protein 2-like (LOC102266833), mRNA
ENSP00000361285-D1	1.28	3.13E-02	↓	B. taurus uridine-cytidine kinase 1 (UCK1), mRNA
ENSBTAP00000040770-D1	1.28	3.13E-02	↓	B. mutus tetratricopeptide repeat domain 16 (TTC16), mRNA
ENSP00000349297-D1	1.28	3.13E-02	↓	B. mutus very long-chain specific acyl-CoA dehydrogenase, mitochondrial-like (LOC102273950), partial mRNA
ENSBTAP00000053704-D2	1.28	3.13E-02	↓	B. mutus neurotrophic tyrosine kinase, receptor, type 2 (NTRK2), transcript variant X1, mRNA
ENSBTAP00000051287-D1	1.29	1.37E-06	↓	B. mutus ornithine decarboxylase 2-like (LOC102273833), mRNA
ENSP00000304290-D1	1.29	4.36E-04	↓	B. mutus cholinergic receptor, nicotinic, beta 1 (muscle) (CHRNB1), transcript variant X1, mRNA
ENSP00000260264-D1	1.29	4.36E-04	↓	B. mutus POU class 2 homeobox 3 (POU2F3), mRNA
ENSBTAP00000003099-D1	1.29	1.31E-17	↓	B. mutus mediator complex subunit 19 (MED19), mRNA
ENSP00000384264-D1	1.30	5.67E-05	↓	B. taurus cyclic nucleotide gated channel alpha 1 (CNGA1), mRNA
ENSBTAP00000053659-D1	1.30	1.08E-02	↓	B. mutus cornichon family AMPA receptor auxiliary protein 3 (CNIH3), mRNA
ENSP00000263160-D1	1.30	1.08E-02	↓	B. mutus solute carrier family 17 (vesicular glutamate transporter), member 6 (SLC17A6), mRNA
ENSP00000323183-D1	1.30	3.82E-03	↓	B. mutus zinc finger and BTB domain containing 2 (ZBTB2), mRNA
ENSBTAP00000012842-D1	1.30	7.09E-12	↓	B. mutus tubulin tyrosine ligase-like family, member 9 (TTLL9), mRNA
ENSBTAP00000026199-D1	1.32	2.41E-03	↓	B. mutus transmembrane and ubiquitin-like domain containing 2 (TMUB2), transcript variant X1, mRNA
ENSP00000381891-D1	1.32	2.41E-03	↓	B. mutus v-ets avian erythroblastosis virus E26 oncogene homolog (ERG), transcript variant X1, mRNA
ENSP00000410007-D1	1.32	6.79E-03	↓	B. mutus F-box protein 46 (FBXO46), mRNA
ENSP00000361266-D1	1.32	1.92E-02	↓	B. mutus patched 2 (PTCH2), mRNA
ENSP00000254260-D1	1.32	1.92E-02	↓	B. mutus rhophilin, Rho GTPase binding protein 2 (RHPN2), mRNA
ENSP00000274181-D1	1.32	1.92E-02	↓	B. mutus ADAM metallopeptidase with thrombospondin type 1 motif, 16 (ADAMTS16), mRNA
ENSP00000085068-D1	1.32	1.92E-02	↓	Capra hircus isochorismatase domain containing 2 (ISOC2), transcript variant X1, mRNA
ENSP00000347620-D1	1.33	7.08E-05	↓	Bubalus bubalis transmembrane protein 116 (TMEM116), transcript variant X3, mRNA
ENSBTAP00000015763-D1	1.33	7.78E-07	↓	B. mutus mal, T-cell differentiation protein-like (MALL), mRNA

ENSBTAP00000026055-D1	1.33	6.40E-108	↓	B. taurus splicing factor, arginine/serine-rich 17A, mRNA (cDNA clone IMAGE:8272031), partial cds
ENSP00000307705-D1	1.33	0.00E+00	↓	B. mutus histone H1.4-like (LOC102278600), mRNA
yakG041387	1.34	3.77E-06	↓	B. mutus alkylglycerol monooxygenase (AGMO), mRNA
ENSP00000366995-D1	1.34	6.63E-06	↓	B. mutus FERM and PDZ domain containing 1 (FRMPD1), mRNA
ENSBTAP00000001496-D1	1.34	6.63E-06	↓	B. mutus ADAM-like, decysin 1 (ADAMDEC1), mRNA
ENSBTAP00000003130-D1	1.34	9.62E-04	↓	B. mutus malonyl CoA:ACP acyltransferase (mitochondrial) (MCAT), mRNA
ENSP00000381086-D1	1.34	1.21E-02	↓	B. mutus chromosome unknown open reading frame, human CXorf36 (LOC102273300), mRNA
ENSP00000323036-D1	1.34	1.21E-02	↓	B. mutus prostatic acid phosphatase-like (LOC102273416), transcript variant X1, mRNA
ENSP00000366234-D1	1.35	7.34E-44	↓	Pantholops hodgsonii uncharacterized LOC102317578 (LOC102317578), mRNA
ENSBTAP00000047401-D1	1.36	8.82E-05	↓	B. mutus pleckstrin homology domain containing, family G (with RhoGef domain) member 4 (PLEKHG4), mRNA
ENSP00000322304-D2	1.36	8.82E-05	↓	B. mutus porcupine homolog (Drosophila) (PORCN), transcript variant X1, mRNA
ENSBTAP00000001461-D1	1.36	3.87E-04	↓	B. taurus CKLF-like MARVEL transmembrane domain containing 4 (CMTM4), mRNA
ENSP00000360437-D1	1.36	1.70E-03	↓	B. mutus family with sequence similarity 210, member B (FAM210B), mRNA
ENSBTAP00000026477-D1	1.36	1.70E-03	↓	B. mutus phosphoinositide-3-kinase adaptor protein 1 (PIK3AP1), mRNA
ENSP00000349805-D1	1.36	7.61E-03	↓	B. taurus family with sequence similarity 212, member B (FAM212B), mRNA
ENSBTAP00000000488-D1	1.36	3.53E-02	↓	B. mutus BMX non-receptor tyrosine kinase (BMX), mRNA
ENSBTAP00000004024-D1	1.36	3.53E-02	↓	B. mutus testis specific, 10 interacting protein (TSGA10IP), mRNA
ENSP00000282091-D1	1.36	3.53E-02	↓	B. mutus parathyroid hormone (PTH), mRNA
ENSP00000333917-D1	1.36	3.53E-02	↓	B. mutus dual specificity phosphatase 18 (DUSP18), mRNA
ENSP00000222482-D1	1.36	3.53E-02	↓	B. mutus carboxypeptidase A4 (CPA4), transcript variant X1, mRNA
ENSBTAP00000006131-D1	1.36	3.53E-02	↓	B. mutus dynein, axonemal, heavy chain 9 (DNAH9), mRNA
yakG044571	1.36	3.53E-02	↓	Mus musculus cDNA, clone:Y1G0145O05, strand:minus, reference: ENSEMBL: Mouse-Transcript-ENST: ENSMUST00000057024, based on BLAT search
ENSBTAP00000041391-D1	1.36	3.53E-02	↓	B. mutus nucleoporin 210kDa (NUP210), mRNA
ENSP00000362160-D2	1.38	4.76E-03	↓	B. mutus solute carrier family 25 (mitochondrial carrier; phosphate carrier), member 23 (SLC25A23), mRNA
yakG001435	1.38	5.40E-105	↓	B. mutus theg spermatid protein-like (THEGL), mRNA
ENSP00000381968-D1	1.39	4.69E-12	↓	B. mutus serine palmitoyltransferase, long chain base subunit 3 (SPTLC3), mRNA
ENSBTAP00000031886-D2	1.39	4.17E-09	↓	B. mutus phospholipase A2 inhibitor and Ly6/PLAUR domain-containing protein-like (LOC102275986), mRNA
ENSP00000415860-D1	1.39	4.28E-04	↓	Pantholops hodgsonii protocadherin-related 15 (PCDH15), transcript variant X3, mRNA
ENSP00000266503-D1	1.39	3.00E-03	↓	B. mutus aryl hydrocarbon receptor nuclear translocator-like 2 (ARNTL2), mRNA
ENSBTAP00000018888-D1	1.39	2.15E-02	↓	B. mutus 60S ribosomal protein L35a-like (LOC102281180), mRNA
ENSP00000356094-D1	1.39	2.15E-02	↓	B. mutus arginine vasopressin receptor 1B (AVPR1B), mRNA
yakG032601	1.39	2.15E-02	↓	Bubalus bubalis anaplastic lymphoma receptor tyrosine kinase (ALK), mRNA
ENSBTAP00000027594-D1	1.39	2.15E-02	↓	B. mutus neurotrypsin-like (LOC102288178), partial mRNA
ENSP00000417184-D1	1.39	2.15E-02	↓	B. mutus phospholipid scramblase family, member 5 (PLSCR5), mRNA
ENSP00000357484-D1	1.40	1.45E-269	↓	B. mutus golgi-associated PDZ and coiled-coil motif containing (GOPC), transcript variant X1, mRNA
ENSBTAP00000007891-D1	1.40	1.72E-04	↓	B. mutus syntrophin, gamma 2 (SNTG2), mRNA
ENSP00000403879-D20	1.40	4.18E-22	↓	B. mutus zinc finger protein 845-like (LOC102286134), partial mRNA
ENSBTAP00000022621-D1	1.41	8.61E-08	↓	B. mutus coiled-coil domain containing 125 (CCDC125), mRNA
ENSP00000382390-D1	1.41	1.19E-03	↓	B. mutus neuralized homolog 4 (Drosophila) (NEURL4), mRNA
yakG045552	1.41	1.19E-03	↓	Pantholops hodgsonii UDP-glucuronate decarboxylase 1 pseudogene (LOC102316691), misc_RNA
ENSP00000395738-D1	1.41	1.35E-02	↓	B. mutus tRNA methyltransferase 2 homolog A (S. cerevisiae) (TRMT2A), mRNA
ENSBTAP00000050583-D2	1.41	1.35E-02	↓	B. taurus BAC CH240-472P12 complete sequence
ENSBTAP00000028522-D1	1.41	1.35E-02	↓	B. mutus NK2 homeobox 3 (NKX2-3), mRNA
ENSP00000202816-D1	1.41	0.00E+00	↓	B. mutus ESF1, nucleolar pre-rRNA processing protein, homolog (S. cerevisiae) (ESF1), mRNA
ENSP00000353410-D11	1.41	2.36E-07	↓	B. taurus cDNA clone IMAGE:8228065
ENSP00000368438-D1	1.41	0.00E+00	↓	B. mutus proliferating cell nuclear antigen (PCNA), mRNA
ENSBTAP00000002834-D1	1.42	8.47E-03	↓	B. mutus chromosome unknown open reading frame, human C16orf89 (LOC102275173), mRNA
ENSBTAP00000049408-D1	1.42	8.47E-03	↓	B. taurus centromere protein A (CENPA), mRNA
ENSP00000244096-D2	1.42	2.99E-04	↓	B. mutus melanoma-associated antigen 10-like (LOC102277366), mRNA
ENSBTAP00000010911-D1	1.43	5.28E-03	↓	B. mutus protein C receptor, endothelial (PROCR), mRNA
ENSBTAP00000049395-D1	1.43	5.28E-03	↓	B. mutus 1-phosphatidylinositol 4,5-bisphosphate phosphodiesterase delta-1-like (LOC102270277), mRNA
ENSP00000403888-D1	1.43	1.20E-04	↓	B. mutus disrupted in schizophrenia 1 (DISC1), mRNA
ENSP00000228945-D1	1.43	3.32E-03	↓	B. mutus Rho GDP dissociation inhibitor (GDI) beta (ARHGDIB), mRNA
ENSBTAP00000007339-D1	1.44	2.09E-03	↓	B. mutus superkiller viralicidic activity 2-like (S. cerevisiae) (SKIV2L), mRNA

ENSBTAP00000028662-D1	1.44	0.00E+00	↓	B. mutus leiomodin 3 (fetal) (LMOD3), mRNA
ENSBTAP00000048708-D1	1.45	8.23E-04	↓	Bubalus bubalis casein kinase I isoform alpha-like (LOC102405585), mRNA
ENSBTAP00000024453-D12	1.45	3.25E-26	↓	B. mutus prostaglandin F synthase 1-like (LOC102271733), mRNA
ENSBTAP00000051871-D3	1.45	2.98E-09	↓	Bubalus bubalis ankyrin repeat domain-containing protein 26-like (LOC102410310), mRNA
ENSBTAP00000016300-D1	1.45	3.29E-04	↓	B. taurus aldo-keto reductase family 7, member A2 (aflatoxin aldehyde reductase) (AKR7A2), mRNA
ENSP00000362125-D1	1.45	2.14E-47	↓	Bubalus bubalis protocadherin 19 (PCDH19), transcript variant X1, mRNA
ENSP00000273371-D1	1.46	1.13E-17	↓	B. mutus phospholipase A1 member A (PLA1A), transcript variant X1, mRNA
ENSP00000376910-D1	1.46	8.12E-09	↓	B. mutus leucine-rich repeats and IQ motif containing 1 (LRRIQ1), mRNA
ENSP00000329403-D1	1.47	8.78E-26	↓	B. mutus Purkinje cell protein 4 (PCP4), mRNA
ENSBTAP00000000774-D1	1.47	3.61E-28	↓	Bubalus bubalis chromosome unknown open reading frame, human C5orf42, transcript variant X3, mRNA
ENSP00000263238-D1	1.48	6.13E-15	↓	B. mutus ARP3 actin-related protein 3 homolog B (yeast) (ACTR3B), transcript variant X2, mRNA
yakG012084	1.48	2.47E-16	↓	B. mutus small kinetochore-associated protein-like (LOC102266133), mRNA
ENSBTAP00000005079-D1	1.49	1.26E-33	↓	Bubalus bubalis cytidine monophosphate-N-acetylneuraminic acid hydroxylase-like, transcript variant X3, mRNA
ENSP00000373698-D1	1.49	1.43E-03	↓	B. mutus F-box and WD repeat domain containing 4 (FBXW4), partial mRNA
ENSP00000407569-D1	1.49	1.43E-03	↓	B. mutus serine/arginine repetitive matrix protein 2-like (LOC102282263), misc_RNA
ENSP00000305193-D1	1.49	2.28E-03	↓	B. mutus HCLS1 binding protein 3 (HS1BP3), mRNA
ENSBTAP00000053606-D1	1.49	3.64E-03	↓	B. mutus frizzled family receptor 4 (FZD4), mRNA
ENSP00000404429-D3	1.49	3.64E-03	↓	Pantholops hodgsonii BCL2/adenovirus E1B 19 kDa protein-interacting protein 3-like (LOC102336418), mRNA
ENSP00000366923-D1	1.49	5.79E-03	↓	B. mutus insulin-like growth factor binding protein-like 1 (IGFBPL1), mRNA
ENSBTAP00000041215-D1	1.49	5.79E-03	↓	B. mutus TNF receptor-associated factor 1 (TRAF1), mRNA
ENSBTAP00000011254-D1	1.49	9.25E-03	↓	B. mutus dynein, axonemal, intermediate chain 1 (DNAI1), mRNA
ENSP00000295304-D1	1.49	1.49E-02	↓	Bubalus bubalis ChaC, cation transport regulator homolog 2 (E. coli) (CHAC2), transcript variant X1, mRNA
ENSBTAP00000006320-D1	1.49	1.49E-02	↓	B. mutus class I histocompatibility antigen, Gogo-C*0202 alpha chain-like (LOC102288356), mRNA
ENSBTAP00000053812-D1	1.49	2.38E-02	↓	.
ENSP00000329632-D1	1.49	2.38E-02	↓	B. mutus F-box and leucine-rich repeat protein 7 (FBXL7), mRNA
ENSBTAP00000026537-D1	1.49	2.38E-02	↓	B. mutus coiled-coil domain containing 64B (CCDC64B), mRNA
ENSBTAP00000045355-D1	1.49	2.38E-02	↓	B. mutus fragile X mental retardation 1 neighbor (FMR1NB), mRNA
ENSP00000386167-D18	1.49	2.38E-02	↓	B. taurus olfactory receptor 2A2 (LOC511400), mRNA
ENSBTAP00000028224-D1	1.49	3.94E-02	↓	B. taurus ADAM metallopeptidase with thrombospondin type 1 motif, 14 (ADAMTS14), mRNA
ENSBTAP00000018887-D1	1.49	3.94E-02	↓	B. mutus ADAM metallopeptidase with thrombospondin type 1 motif, 10 (ADAMTS10), mRNA
ENSP00000372193-D1	1.49	3.94E-02	↓	B. mutus chloride channel, voltage-sensitive 7 (CLCN7), mRNA
ENSP00000382941-D1	1.49	3.94E-02	↓	B. mutus signal-regulatory protein alpha (SIRPA), mRNA
ENSP00000221347-D1	1.49	3.94E-02	↓	Bubalus bubalis Fc fragment of IgG binding protein (FCGBP), transcript variant X1, mRNA
ENSBTAP00000026215-D1	1.49	3.94E-02	↓	B. mutus scavenger receptor cysteine-rich type 1 protein M130-like (LOC102266058), mRNA
ENSBTAP00000032110-D3	1.49	3.94E-02	↓	B. taurus heterogeneous nuclear ribonucleoprotein A1 (HNRNPA1), mRNA
ENSP00000263431-D1	1.49	3.94E-02	↓	B. mutus protein kinase C, gamma (PRKCG), mRNA
ENSP00000416445-D1	1.49	3.94E-02	↓	B. mutus coiled-coil domain containing 120 (CCDC120), transcript variant X1, mRNA
ENSBTAP00000044058-D1	1.49	3.94E-02	↓	B. mutus dynein heavy chain 3, axonemal-like (LOC102278029), misc_RNA
ENSP00000364621-D1	1.50	5.47E-40	↓	B. mutus sushi domain containing 3 (SUSD3), mRNA
ENSP00000410996-D1	1.51	4.05E-07	↓	Bubalus bubalis six transmembrane epithelial antigen of the prostate 1 (STEAP1), transcript variant X1, mRNA
ENSP00000351280-D100	1.52	2.49E-05	↓	B. mutus zinc finger protein 546 (ZNF546), mRNA
yakG025883	1.52	3.92E-05	↓	B. mutus src kinase associated phosphoprotein 1 (SKAP1), mRNA
ENSBTAP00000033286-D7	1.52	2.84E-08	↓	B. mutus histone H2A type 2-B-like (LOC102276911), mRNA
ENSBTAP00000002681-D1	1.52	9.86E-05	↓	B. mutus neprilysin-like (LOC102276645), transcript variant X3, mRNA
ENSBTAP00000053295-D1	1.52	1.12E-07	↓	B. mutus homogentisate 1,2-dioxygenase (HGD), mRNA
ENSBTAP00000027579-D2	1.52	1.56E-04	↓	Bubalus bubalis putative uncharacterized protein ZNRD1-AS1 homolog, transcript variant X1, mRNA
ENSBTAP00000050302-D1	1.53	7.20E-51	↓	B. mutus mitochondrial ribosomal protein L38 (MRPL38), mRNA
ENSBTAP00000036380-D1	1.53	6.52E-36	↓	B. mutus calmodulin-like 4 (CALML4), transcript variant X1, mRNA
ENSP00000264218-D1	1.54	9.78E-04	↓	B. mutus neuromedin U (NMU), mRNA
ENSBTAP00000010730-D1	1.54	9.78E-04	↓	B. mutus chloride channel accessory 1 (CLCA1), mRNA
ENSBTAP00000047628-D1	1.54	9.78E-04	↓	B. taurus homeobox A9, mRNA (cDNA clone MGC:155055 IMAGE:8468673), complete cds
ENSBTAP00000036314-D1	1.55	2.48E-03	↓	B. mutus tumor necrosis factor (ligand) superfamily, member 8 (TNFSF8), mRNA
ENSBTAP00000045511-D1	1.55	5.25E-08	↓	B. mutus endomucin (EMCN), mRNA
ENSBTAP00000017531-D1	1.56	1.07E-04	↓	B. mutus septin 3 (SEPT3), transcript variant X1, mRNA
ENSBTAP00000009659-D1	1.56	1.07E-04	↓	B. mutus FERM domain containing 7 (FRMD7), transcript variant X1, mRNA

ENSP00000265707-D1	1.56	3.94E-03	↓	B. mutus ADAM metallopeptidase domain 18 (ADAM18), mRNA
ENSBTAP00000025041-D1	1.56	3.94E-03	↓	B. mutus transmembrane channel-like 2 (TMC2), partial mRNA
ENSBTAP00000011776-D1	1.56	3.94E-03	↓	B. mutus serine dehydratase-like (SDSL), mRNA
yakG032823	1.56	3.94E-03	↓	B. mutus putative deoxyribonuclease TATDN2-like (LOC102279628), mRNA
ENSBTAP00000000607-D1	1.57	2.66E-04	↓	B. mutus interferon-related developmental regulator 2 (IFRD2), mRNA
ENSP00000229332-D1	1.57	2.66E-04	↓	B. taurus C-type lectin domain family 4, member A (CLEC4A), mRNA
ENSBTAP00000011949-D1	1.57	6.29E-03	↓	B. mutus mitogen-activated protein kinase kinase kinase 5 (MAP3K5), mRNA
ENSBTAP00000042293-D1	1.57	6.29E-03	↓	B. mutus deltex homolog 3 (Drosophila) (DTX3), mRNA
ENSBTAP00000030514-D1	1.57	6.29E-03	↓	B. mutus C-type lectin domain family 12, member B (CLEC12B), mRNA
ENSBTAP00000012923-D1	1.57	6.29E-03	↓	Bubalus bubalis doublecortin domain containing 2 (DCDC2), transcript variant X1, mRNA
ENSBTAP00000039023-D2	1.57	1.12E-303	↓	B. mutus oocyte secreted protein 1 (OOSP1), mRNA
ENSBTAP00000019517-D1	1.57	1.27E-06	↓	B. mutus chromosome unknown open reading frame, human C14orf39 (LOC102285496), mRNA
ENSBTAP00000040815-D1	1.58	4.96E-99	↓	B. mutus SPARC related modular calcium binding 1 (SMOC1), transcript variant X2, mRNA
ENSBTAP00000013688-D1	1.58	6.67E-04	↓	B. mutus chromosome unknown open reading frame, human C14orf79 (LOC102272442), mRNA
ENSP00000377141-D1	1.58	6.67E-04	↓	B. mutus arrestin, beta 1 (ARRB1), partial mRNA
ENSBTAP00000013902-D1	1.58	1.00E-02	↓	Bubalus bubalis poliovirus receptor related immunoglobulin domain containing (PVRIG), mRNA
ENSBTAP00000039011-D1	1.58	1.00E-02	↓	B. taurus protease, serine, 1 (trypsin 1), mRNA (cDNA clone IMAGE:8050015), partial cds
ENSBTAP00000006291-D1	1.59	7.19E-05	↓	B. mutus charged multivesicular body protein 4C (CHMP4C), mRNA
ENSBTAP00000053268-D1	1.60	2.36E-07	↓	B. mutus astrotactin 1 (ASTN1), transcript variant X1, mRNA
ENSBTAP00000025660-D1	1.60	1.81E-04	↓	B. mutus DMRT-like family C2 (DMRTC2), transcript variant X1, mRNA
ENSBTAP00000052911-D65	1.60	1.61E-02	↓	B. taurus similar to T-cell receptor beta chain V region CTL-F3 precursor, mRNA (cDNA clone IMAGE:8189587)
ENSP00000404232-D1	1.61	7.73E-15	↓	B. mutus EF-hand domain (C-terminal) containing 2 (EFHC2), mRNA
ENSP00000357392-D1	1.62	2.65E-03	↓	B. taurus ephrin-A1 (EFNA1), mRNA
ENSBTAP00000005528-D1	1.62	2.65E-03	↓	Bubalus bubalis 60S ribosomal protein L27-like (LOC102411130), mRNA
ENSP00000404826-D1	1.62	2.75E-08	↓	B. mutus polycystic kidney disease 1-like 2 (PKD1L2), mRNA
ENSP00000007264-D1	1.63	4.49E-04	↓	B. mutus RNA pseudouridylate synthase domain containing 1 (RPUSD1), mRNA
ENSBTAP00000042083-D1	1.63	4.49E-04	↓	Pantholops hodgsonii ATPase, H+ transporting, lysosomal accessory protein 2 pseudogene, misc_RNA
ENSP00000362139-D1	1.63	2.69E-12	↓	B. taurus EPH receptor A10 (EPHA10), mRNA
yakA01362	1.63	7.67E-05	↓	.
ENSP00000352252-D18	1.63	1.33E-05	↓	B. taurus histone H3.2 (LOC504599), mRNA
ENSP00000409937-D1	1.64	4.90E-13	↓	Balaenoptera acutorostrata scammoni putative leucine-rich repeat-containing protein DDB_G0290503-like, mRNA
ENSBTAP00000048073-D1	1.64	2.09E-05	↓	B. mutus brain protein I3 (BRI3), partial mRNA
ENSP00000390600-D7	1.64	7.08E-04	↓	B. mutus sodium channel, voltage-gated, type XI, alpha subunit (SCN11A), mRNA
ENSBTAP00000000932-D1	1.64	2.58E-02	↓	B. mutus myosin XVIIIB (MYO18B), mRNA
ENSBTAP00000016638-D2	1.64	2.58E-02	↓	B. mutus ferritin heavy chain-like (LOC102264724), mRNA
ENSP00000337915-D3	1.64	2.58E-02	↓	B. mutus cytochrome P450 3A24-like (LOC102284132), mRNA
ENSBTAP00000013791-D1	1.64	2.58E-02	↓	B. mutus copine IV (CPNE4), mRNA
ENSBTAP00000001758-D1	1.65	3.74E-10	↓	B. mutus growth hormone receptor (GHR), mRNA
ENSBTAP00000020053-D1	1.66	2.74E-19	↓	B. mutus transmembrane protease, serine 7 (TMPRSS7), mRNA
ENSP00000325296-D1	1.66	1.12E-03	↓	B. mutus polycystic kidney disease 2-like 1 (PKD2L1), mRNA
ENSP00000273784-D1	1.67	5.33E-09	↓	B. mutus alpha-2-HS-glycoprotein (AHSG), mRNA
ENSBTAP00000019617-D1	1.67	3.01E-04	↓	B. mutus leucine-rich repeats and WD repeat domain containing 1 (LRWD1), mRNA
ENSP00000392637-D1	1.67	6.68E-03	↓	Bubalus bubalis CD99 molecule-like 2 (CD99L2), transcript variant X1, mRNA
ENSBTAP00000036858-D1	1.67	3.79E-06	↓	Bubalus bubalis limb bud and heart development (LBH), mRNA
ENSBTAP00000039906-D3	1.68	1.77E-03	↓	B. mutus epididymis-specific alpha-mannosidase-like (LOC102271787), partial mRNA
ENSBTAP00000004942-D1	1.69	4.73E-04	↓	B. mutus tektin 3 (TEKT3), mRNA
ENSP00000350136-D1	1.70	1.87E-07	↓	B. mutus ribosomal modification protein rimK-like family member B (RIMKLB), mRNA
ENSP00000367715-D1	1.71	4.31E-10	↓	B. mutus doublecortin domain containing 2 (DCDC2), mRNA
ENSP00000362601-D1	1.71	1.07E-06	↓	B. mutus cysteine-rich hydrophobic domain 1 (CHIC1), transcript variant X1, mRNA
ENSP00000254457-D1	1.71	1.07E-06	↓	B. mutus LIM homeobox 1 (LHX1), mRNA
ENSP00000317534-D1	1.71	2.79E-03	↓	B. mutus zinc finger and BTB domain containing 34 (ZBTB34), mRNA
ENSBTAP00000053651-D1	1.71	1.06E-02	↓	B. mutus lin-28 homolog B (C. elegans) (LIN28B), mRNA
ENSBTAP00000042418-D1	1.71	1.06E-02	↓	B. mutus 2'-5'-oligoadenylate synthase 1-like (LOC102282949), mRNA
ENSBTAP00000009516-D1	1.71	1.06E-02	↓	B. mutus netrin G2 (NTNG2), partial mRNA
ENSP00000367062-D17	1.71	1.06E-02	↓	B. mutus histone H3.1-like (LOC102275502), mRNA

ENSBTAP00000051022-D1	1.71	4.23E-02	↓	B. mutus proproteinase E-like (LOC102267066), mRNA
ENSBTAP00000005589-D3	1.71	4.23E-02	↓	B. mutus V-type proton ATPase 116 kDa subunit a isoform 4-like (LOC102270383), mRNA
ENSBTAP00000028780-D1	1.71	4.23E-02	↓	Bubalus bubalis zinc and ring finger 3 (ZNRF3), mRNA
ENSP00000385357-D1	1.71	4.23E-02	↓	Bubalus bubalis coiled-coil domain containing 157 (CCDC157), transcript variant X1, mRNA
ENSBTAP00000032783-D1	1.71	4.23E-02	↓	Lipotes vexillifer collagen, type XVII, alpha 1 (COL17A1), mRNA
ENSBTAP00000009466-D1	1.71	4.23E-02	↓	B. mutus guanylate cyclase activator 2A (guanylin) (GUCA2A), mRNA
ENSBTAP00000025132-D1	1.71	4.23E-02	↓	B. mutus synaptotagmin VI (SYT6), mRNA
ENSBTAP00000019149-D1	1.71	4.23E-02	↓	B. taurus sorbin and SH3 domain containing 3 (SORBS3), transcript variant X8, mRNA
yakG001024	1.71	4.23E-02	↓	bovine 1.711 g/ml satellite dna, f fragment
ENSP00000350265-D1	1.71	4.23E-02	↓	B. mutus ectonucleotide pyrophosphatase/phosphodiesterase 3 (ENPP3), mRNA
yakG017852	1.71	4.23E-02	↓	B. mutus histone H2A type 1-D-like (LOC102270329), transcript variant X2, mRNA
ENSP00000297537-D1	1.71	4.23E-02	↓	Pantholops hodgsonii gastrulation brain homeobox 1 (GBX1), mRNA
ENSP00000339637-D1	1.71	4.23E-02	↓	B. mutus BRCA1-associated ATM activator 1 (BRAT1), mRNA
ENSBTAP00000007078-D1	1.71	4.23E-02	↓	B. mutus solute carrier family 41 (magnesium transporter), member 1 (SLC41A1), mRNA
ENSP00000299543-D1	1.73	2.29E-05	↓	B. mutus CTD (carboxy-terminal domain, RNA polymerase II, polypeptide A) phosphatase, subunit 1, mRNA
ENSP00000219599-D2	1.74	6.07E-09	↓	B. mutus ketimine reductase mu-crystallin-like (LOC102287512), partial mRNA
ENSP00000298295-D1	1.74	1.17E-03	↓	B. mutus chromosome unknown open reading frame, human C10orf10 (LOC102275935), mRNA
ENSP00000378524-D1	1.74	3.02E-07	↓	B. mutus leucine-rich repeats and IQ motif containing 3 (LRRIQ3), mRNA
ENSBTAP00000004304-D1	1.75	1.29E-07	↓	B. mutus tubulin tyrosine ligase-like family, member 7 (TTLL7), mRNA
ENSP00000263196-D1	1.75	4.43E-03	↓	Bubalus bubalis DiGeorge syndrome critical region gene 2 (DGCR2), partial mRNA
ENSBTAP00000053754-D1	1.77	6.44E-06	↓	B. mutus WD repeat domain 72 (WDR72), mRNA
ENSP00000392091-D1	1.78	1.67E-02	↓	B. mutus carbohydrate (N-acetylgalactosamine 4-0) sulfotransferase 9 (CHST9), mRNA
ENSBTAP00000022890-D1	1.78	1.67E-02	↓	B. mutus ring finger protein 135 (RNF135), mRNA
ENSBTAP00000003878-D1	1.78	1.67E-02	↓	B. mutus phosphoinositide-3-kinase, regulatory subunit 3 (gamma) (PIK3R3), transcript variant X2, mRNA
ENSP00000303394-D1	1.78	1.67E-02	↓	B. mutus heat shock 27kDa protein 3 (HSPB3), mRNA
ENSBTAP00000012416-D1	1.78	1.67E-02	↓	B. mutus transmembrane protein 79 (TMEM79), mRNA
ENSBTAP00000020668-D1	1.78	1.67E-02	↓	B. mutus deleted in liver cancer 1 (DLC1), transcript variant X1, mRNA
ENSP00000365198-D1	1.79	1.00E-05	↓	Bubalus bubalis kazrin, periplakin interacting protein (KAZN), mRNA
ENSBTAP00000053791-D1	1.80	7.67E-04	↓	B. mutus protein tyrosine phosphatase, receptor type, M (PTPRM), partial mRNA
ENSP00000362807-D1	1.80	7.67E-04	↓	B. mutus penta-EF-hand domain containing 1 (PEF1), mRNA
ENSBTAP00000018420-D1	1.81	6.92E-03	↓	B. mutus SH3-domain binding protein 4 (SH3BP4), mRNA
ENSBTAP00000026682-D1	1.81	6.92E-03	↓	B. mutus polycystic kidney disease 2 (autosomal dominant) (PKD2), mRNA
ENSP00000396145-D1	1.81	6.92E-03	↓	B. mutus serine hydrolase-like 2 (SERHL2), mRNA
ENSBTAP00000020679-D1	1.82	6.58E-06	↓	B. mutus DLA class II histocompatibility antigen, DR-1 beta chain-like (LOC102282150), misc_RNA
ENSBTAP00000013193-D1	1.82	1.35E-10	↓	B. mutus interferon regulatory factor 2 (IRF2), mRNA
ENSP00000244537-D6	1.83	2.18E-97	↓	B. mutus histone H4-like (LOC102279902), mRNA
ENSBTAP00000042479-D9	1.83	1.17E-06	↓	B. mutus histone H2A type 3-like (LOC102280294), mRNA
ENSBTAP00000040707-D4	1.84	8.82E-11	↓	B. mutus keratin, type I cytoskeletal 25-like (LOC102266759), partial mRNA
ENSBTAP00000045034-D1	1.84	2.41E-05	↓	B. mutus interferon beta-2-like (LOC102280497), mRNA
ENSBTAP00000011612-D1	1.84	5.67E-14	↓	B. mutus laminin, alpha 4 (LAMA4), transcript variant X2, mRNA
ENSP00000264037-D1	1.84	7.64E-10	↓	B. mutus tectorin alpha (TECTA), mRNA
ENSP00000362762-D1	1.86	2.12E-04	↓	B. mutus brain-specific angiogenesis inhibitor 2 (BAI2), transcript variant X1, mRNA
ENSBTAP00000017827-D1	1.86	3.07E-92	↓	B. mutus vasoactive intestinal peptide (VIP), mRNA
ENSP00000334100-D1	1.90	7.63E-07	↓	Pteropus alecto exocyst complex component 7 (EXOC7), transcript variant X1, mRNA
ENSP00000407097-D1	1.90	1.02E-05	↓	.
ENSP00000397297-D1	1.90	5.72E-05	↓	B. taurus neurotrophin 3 (NTF3), mRNA
ENSBTAP00000053558-D1	1.90	7.72E-04	↓	B. mutus microtubule associated serine/threonine kinase 3 (MAST3), transcript variant X2, mRNA
ENSBTAP00000026928-D1	1.90	1.08E-02	↓	B. mutus MSS51 mitochondrial translational activator (MSS51), mRNA
ENSP00000289228-D1	1.90	1.08E-02	↓	B. mutus ARP1 actin-related protein 1 homolog B, centractin beta (yeast) (ACTR1B), mRNA
ENSP00000418259-D1	1.90	1.08E-02	↓	B. mutus ZFP57 zinc finger protein (ZFP57), mRNA
ENSP00000295190-D1	1.90	1.08E-02	↓	B. mutus solute carrier family 16, member 14 (SLC16A14), mRNA
ENSBTAP00000043397-D11	1.90	2.66E-02	↓	Pantholops hodgsonii multidrug resistance-associated protein 4-like (LOC102336500), misc_RNA
ENSBTAP00000048897-D1	1.90	2.66E-02	↓	B. mutus solute carrier family 9, subfamily A (NHE5, cation proton antiporter 5), member 5 (SLC9A5), mRNA
ENSP00000340811-D1	1.90	2.66E-02	↓	B. mutus gap junction protein, beta 5, 31.1kDa (GJB5), mRNA
ENSBTAP00000033974-D1	1.90	2.66E-02	↓	B. mutus calmodulin-like 6 (CALML6), mRNA

ENSBTAP00000043988-D1	1.90	2.66E-02	↓	Bubalus bubalis transmembrane protein 253 (TMEM253), mRNA
ENSP00000316426-D1	1.90	2.66E-02	↓	B. mutus myeloid/lymphoid or mixed-lineage leukemia; translocated to, 6 (MLLT6), mRNA
ENSBTAP00000006298-D1	1.90	2.66E-02	↓	B. mutus tRNA methyltransferase 44 homolog (S. cerevisiae) (TRMT44), mRNA
ENSBTAP00000045070-D1	1.91	1.31E-79	↓	B. mutus interleukin 18 receptor accessory protein (IL18RAP), mRNA
ENSBTAP00000049368-D4	1.93	2.08E-07	↓	Capra hircus multidrug resistance-associated protein 4-like (LOC102172427), mRNA
ENSP00000370343-D1	1.93	2.77E-06	↓	B. mutus interferon regulatory factor 4 (IRF4), transcript variant X1, mRNA
ENSP00000289473-D1	1.93	2.77E-06	↓	B. mutus neutrophil cytosolic factor 1 (NCF1), mRNA
ENSBTAP00000051307-D1	1.93	2.77E-06	↓	B. taurus septin 4 (SEPT4), mRNA, incomplete 5' cds
ENSBTAP00000019855-D1	1.94	6.59E-06	↓	B. mutus steroid 5 alpha-reductase 3 (SRD5A3), mRNA
ENSBTAP00000002357-D1	1.94	8.54E-42	↓	B. mutus synaptotagmin IV (SYT4), transcript variant X1, mRNA
ENSP00000244534-D2	1.95	0.00E+00	↓	B. mutus histone H1.2-like (LOC102281127), mRNA
ENSBTAP00000029961-D1	1.95	2.87E-31	↓	B. mutus transmembrane protein 134 (TMEM134), transcript variant X1, mRNA
ENSBTAP00000019761-D1	1.95	2.10E-04	↓	B. mutus vacuolar protein sorting 26 homolog B (S. pombe) (VPS26B), mRNA
ENSBTAP00000040327-D1	1.95	2.10E-04	↓	B. mutus platelet/endothelial cell adhesion molecule 1 (PECAM1), mRNA
ENSP00000249499-D1	1.97	1.19E-03	↓	Capra hircus homeobox C9 (Hoxc9) gene, complete cds
ENSP00000341094-D2	1.98	3.59E-11	↓	B. mutus histone H2A type 1-like (LOC102276344), mRNA
ENSP00000358320-D1	1.99	2.33E-08	↓	B. mutus polymerase (RNA) III (DNA directed) polypeptide G (32kD)-like (POLR3GL), mRNA
ENSP00000298820-D1	1.99	2.85E-03	↓	B. mutus otogelin-like protein-like (LOC102282101), partial mRNA
ENSP00000358997-D1	1.99	2.85E-03	↓	B. mutus interleukin-1 receptor-associated kinase 1 (IRAK1), mRNA
ENSP00000416776-D1	2.01	3.08E-07	↓	B. mutus cysteine-serine-rich nuclear protein 1 (CSRNP1), mRNA
ENSP00000297313-D1	2.02	6.87E-03	↓	B. mutus regulator of G-protein signaling 20 (RGS20), transcript variant X1, mRNA
ENSBTAP00000021263-D1	2.02	6.87E-03	↓	B. mutus cell adhesion molecule 4 (CADM4), mRNA
ENSP00000340211-D1	2.02	6.87E-03	↓	B. mutus coronin, actin binding protein, 1B (CORO1B), mRNA
ENSP00000364150-D1	2.02	6.87E-03	↓	B. mutus UDP-N-acetyl-alpha-D-galactosamine:polypeptide N-acetylgalactosaminyltransferase 12, partial mRNA
ENSBTAP00000007448-D1	2.03	1.73E-06	↓	B. mutus solute carrier family 39 (zinc transporter), member 8 (SLC39A8), transcript variant X2, mRNA
ENSP00000293774-D1	2.04	1.97E-07	↓	B. mutus keratin 4 (KRT4), mRNA
ENSP00000359506-D1	2.05	3.66E-70	↓	B. mutus fragile X mental retardation 1 (FMR1), transcript variant X1, mRNA
ENSBTAP00000042923-D1	2.07	3.93E-16	↓	B. mutus phosphatidic acid phosphatase type 2 domain containing 1A (PPAPDC1A), mRNA
ENSBTAP00000037042-D1	2.07	2.28E-05	↓	B. mutus zinc-alpha-2-glycoprotein-like (LOC102285241), mRNA
ENSP00000334153-D1	2.07	1.80E-03	↓	B. mutus raftlin, lipid raft linker 1 (RFTN1), mRNA
ENSBTAP00000001298-D1	2.07	1.65E-02	↓	B. mutus kelch domain containing 8B (KLHDC8B), mRNA
yakG021933	2.07	1.65E-02	↓	Capra hircus uncharacterized LOC102191729 (LOC102191729), transcript variant X1, misc_RNA
ENSP00000399221-D2	2.07	1.65E-02	↓	B. mutus ribosomal protein L3 pseudogene (LOC102265321), misc_RNA
ENSP00000323777-D3	2.07	1.65E-02	↓	Balaenoptera acutorostrata scammoni Nanog homeobox (NANOG), mRNA
yakG002358	2.07	1.65E-02	↓	B. mutus transmembrane protein 132B (TMEM132B), mRNA
ENSBTAP00000048404-D1	2.07	1.65E-02	↓	B. mutus THAP domain containing 10 (THAP10), mRNA
ENSBTAP00000028359-D1	2.07	1.65E-02	↓	Bubalus bubalis radial spoke head 1 homolog (Chlamydomonas) (RSPH1), mRNA
ENSBTAP00000007338-D1	2.07	1.65E-02	↓	B. mutus glycine amidinotransferase (L-arginine:glycine amidinotransferase) (GATM), mRNA
ENSP00000292174-D1	2.07	1.65E-02	↓	B. mutus chemokine (C-X-C motif) receptor 5 (CXCR5), mRNA
ENSBTAP00000016986-D1	2.07	1.65E-02	↓	B. mutus lactoperoxidase (LPO), transcript variant X1, mRNA
ENSP00000315564-D1	2.11	4.76E-04	↓	B. mutus galectin-9-like (LOC102265423), transcript variant X1, mRNA
ENSBTAP00000047500-D1	2.11	4.76E-04	↓	B. mutus coiled-coil domain containing 160 (CCDC160), mRNA
ENSP00000248444-D1	2.13	1.27E-04	↓	B. mutus villin 1 (VIL1), mRNA
ENSBTAP00000000619-D1	2.13	4.33E-03	↓	B. mutus rhotekin (RTKN), transcript variant X1, mRNA
ENSP00000320869-D1	2.13	4.33E-03	↓	B. mutus tripartite motif containing 41 (TRIM41), mRNA
ENSP00000400168-D1	2.13	4.33E-03	↓	B. mutus pentatricopeptide repeat domain 1 (PTCD1), mRNA
ENSP00000299092-D1	2.13	4.33E-03	↓	B. mutus G protein-coupled receptor 176 (GPR176), mRNA
ENSP00000313699-D1	2.14	3.38E-05	↓	B. mutus hephaestin-like 1 (HEPHL1), mRNA
ENSBTAP00000010703-D1	2.14	6.92E-11	↓	B. mutus interferon induced with helicase C domain 1 (IFIH1), mRNA
ENSP00000416453-D1	2.15	1.13E-03	↓	B. mutus solute carrier family 4 (sodium bicarbonate cotransporter), member 5 (SLC4A5), mRNA
ENSBTAP00000045934-D1	2.15	1.13E-03	↓	B. mutus transporter 1, ATP-binding cassette, sub-family B (MDR/TAP) (TAP1), mRNA
ENSBTAP00000025512-D1	2.15	1.13E-03	↓	B. taurus paired-like homeobox 2a (PHOX2A), mRNA
ENSP00000361929-D1	2.15	1.13E-03	↓	B. mutus cerebral endothelial cell adhesion molecule (CERCAM), transcript variant X3, mRNA
ENSBTAP00000029525-D1	2.15	1.13E-03	↓	B. mutus chromosome unknown open reading frame, human C12orf52 (LOC102285101), mRNA
ENSP00000357354-D1	2.17	2.99E-04	↓	B. mutus family with sequence similarity 189, member B (FAM189B), transcript variant X1, mRNA

ENSP00000272570-D1	2.17	0.00E+00	↓	B. mutus zinc finger CCCH-type containing 8 (ZC3H8), mRNA
ENSP00000385862-D1	2.23	1.33E-05	↓	Human DNA sequence from clone RP1-308I13 on chromosome 1p35-36.3, complete sequence
ENSP00000366119-D1	2.23	2.67E-03	↓	B. mutus ER membrane protein complex subunit 10 (EMC10), mRNA
ENSP00000369121-D1	2.23	2.67E-03	↓	B. mutus tectonin beta-propeller repeat containing 1 (TECPR1), mRNA
ENSBTAP00000036019-D15	2.23	2.67E-03	↓	B. mutus protein kinase C-binding protein NELL1-like (LOC102271177), partial mRNA
ENSBTAP00000016342-D1	2.23	1.03E-02	↓	B. mutus low density lipoprotein receptor (LDLR), mRNA
ENSP00000263317-D1	2.23	1.03E-02	↓	B. mutus NADPH oxidase 4 (NOX4), transcript variant X1, mRNA
ENSBTAP00000041163-D1	2.23	1.03E-02	↓	B. mutus chromosome unknown open reading frame, human C19orf25 (LOC102276976), mRNA
yakA24149	2.23	1.03E-02	↓	B. mutus collagen, type XXV, alpha 1 (COL25A1), mRNA
ENSBTAP00000047637-D1	2.23	1.03E-02	↓	B. taurus olfactory receptor 10T2 (LOC782645), mRNA
yakA05801	2.23	1.03E-02	↓	B. mutus solute carrier family 24 (sodium/potassium/calcium exchanger), member 2 (SLC24A2), mRNA
ENSBTAP00000049286-D1	2.23	1.03E-02	↓	B. taurus phosphatidylinositol glycan anchor biosynthesis, class P pseudogene (LOC782120) on chromosome 1
ENSP00000375470-D1	2.23	4.16E-02	↓	.
ENSP00000386413-D1	2.23	4.16E-02	↓	B. mutus LY6/PLAUR domain containing 6 (LYPD6), mRNA
ENSP00000384666-D2	2.23	4.16E-02	↓	B. mutus peptidyl-prolyl cis-trans isomerase A-like (LOC102284231), mRNA
ENSBTAP00000006339-D1	2.23	4.16E-02	↓	B. mutus acid phosphatase 5, tartrate resistant (ACP5), mRNA
ENSBTAP00000052903-D1	2.23	4.16E-02	↓	B. mutus zinc finger protein 184-like (LOC102266607), mRNA
ENSP00000333266-D1	2.23	4.16E-02	↓	B. mutus ubiquitin-like modifier activating enzyme 7 (UBA7), transcript variant X2, mRNA
ENSP00000270061-D2	2.23	4.16E-02	↓	B. mutus single stranded DNA binding protein 4 (SSBP4), mRNA
ENSP00000242462-D1	2.23	4.16E-02	↓	B. mutus neurogenin 3 (NEUROG3), mRNA
ENSP00000392252-D1	2.23	4.16E-02	↓	B. mutus tubulin tyrosine ligase-like family, member 8 (TTLL8), mRNA
ENSP00000357889-D1	2.23	4.16E-02	↓	B. mutus uncharacterized LOC102267221 (LOC102267221), misc_RNA
ENSBTAP00000006765-D1	2.23	4.16E-02	↓	B. mutus ankyrin repeat and sterile alpha motif domain containing 6 (ANKS6), mRNA
ENSBTAP00000018027-D1	2.23	4.16E-02	↓	B. mutus unc-13 homolog D (C. elegans) (UNC13D), mRNA
ENSBTAP00000040632-D1	2.23	4.16E-02	↓	B. mutus junction plakoglobin (JUP), transcript variant X3, mRNA
ENSBTAP00000000874-D1	2.23	4.16E-02	↓	B. mutus solute carrier family 22, member 15 (SLC22A15), partial mRNA
ENSP00000363817-D1	2.23	4.16E-02	↓	Pantholops hodgsonii retinoid X receptor, beta (RXRB), transcript variant X1, mRNA
ENSBTAP00000015772-D1	2.23	4.16E-02	↓	B. mutus coiled-coil domain containing 24 (CCDC24), mRNA
ENSBTAP00000011164-D1	2.23	4.16E-02	↓	B. mutus c-X-C motif chemokine 13-like (LOC102265246), mRNA
ENSBTAP00000041148-D1	2.23	4.16E-02	↓	B. mutus uromodulin-like 1 (UMODL1), mRNA
ENSP00000340402-D1	2.23	4.16E-02	↓	B. mutus solute carrier family 15, member 5 (SLC15A5), mRNA
yakG034753	2.23	4.16E-02	↓	B. mutus histone H2A type 1-like (LOC102276065), mRNA
ENSP00000365668-D1	2.27	5.00E-10	↓	B. mutus alpha tubulin acetyltransferase 1 (ATAT1), transcript variant X1, mRNA
ENSBTAP00000049865-D24	2.28	1.16E-04	↓	Pantholops hodgsonii uncharacterized LOC102329181 (LOC102329181), misc_RNA
ENSBTAP00000037662-D1	2.31	6.03E-08	↓	B. mutus 5-hydroxytryptamine (serotonin) receptor 1E, G protein-coupled (HTR1E), mRNA
ENSBTAP00000001770-D1	2.32	1.64E-03	↓	B. mutus protein-lysine methyltransferase METTL21E-like (LOC102287829), mRNA
ENSBTAP00000030051-D1	2.32	1.64E-03	↓	B. mutus casein kinase I isoform beta-like (LOC102283373), mRNA
yakG038373	2.32	1.64E-03	↓	B. mutus uncharacterized LOC102285429 (LOC102285429), mRNA
ENSBTAP00000048522-D2	2.32	1.64E-03	↓	B. mutus Harvey rat sarcoma viral oncogene homolog (HRAS), mRNA
ENSP00000244751-D1	2.34	7.08E-05	↓	B. mutus copine V (CPNE5), mRNA
ENSP00000367193-D1	2.34	3.13E-06	↓	B. taurus F-box protein 48 (FBXO48), mRNA
ENSP00000317128-D1	2.36	2.67E-04	↓	B. mutus plexin D1 (PLXND1), mRNA
ENSP00000292431-D2	2.36	6.30E-03	↓	Vicugna pacos NACC family member 2, BEN and BTB (POZ) domain containing (NACC2), mRNA
ENSBTAP00000009302-D1	2.36	6.30E-03	↓	B. mutus solute carrier family 7 (amino acid transporter light chain, L system), member 8 (SLC7A8), mRNA
ENSP00000304078-D1	2.39	4.34E-05	↓	B. mutus family with sequence similarity 83, member B (FAM83B), mRNA
ENSBTAP00000039441-D1	2.40	3.15E-07	↓	B. mutus cholinergic receptor, nicotinic, alpha 9 (neuronal) (CHRNA9), mRNA
ENSBTAP00000002668-D1	2.43	1.63E-04	↓	B. mutus transient receptor potential cation channel, subfamily A, member 1 (TRPA1), mRNA
ENSBTAP00000029067-D1	2.44	1.03E-30	↓	B. mutus tachykinin 3 (TAC3), mRNA
yakG013051	2.46	1.18E-07	↓	.
ENSP00000371085-D1	2.49	2.65E-06	↓	B. mutus BAI1-associated protein 2-like 2 (BAIAP2L2), mRNA
ENSP00000327652-D1	2.49	1.62E-05	↓	B. mutus D(1A) dopamine receptor-like (LOC102277347), mRNA
ENSP00000337103-D1	2.49	1.62E-05	↓	B. mutus choline O-acetyltransferase (CHAT), mRNA
ENSP00000274705-D20	2.49	6.07E-04	↓	B. mutus uncharacterized LOC102280142 (LOC102280142), misc_RNA
ENSBTAP00000014527-D1	2.49	6.07E-04	↓	B. mutus chromosome unknown open reading frame, human C1orf64 (LOC102276592), mRNA
ENSBTAP00000051139-D1	2.49	3.79E-03	↓	B. mutus zinc finger and BTB domain containing 3 (ZBTB3), mRNA

ENSBTAP00000052852-D1	2.49	2.43E-02	↓	B. mutus gap junction protein, beta 6, 30kDa (GJB6), mRNA
ENSBTAP00000014308-D1	2.49	2.43E-02	↓	B. mutus carboxymethylenebutenolidase homolog (Pseudomonas) (CMBL), mRNA
ENSBTAP00000002568-D1	2.49	2.43E-02	↓	Bubalus bubalis TAF7-like RNA polymerase II, TATA box binding protein (TBP)-associated factor, 50kDa (TAF7L), mRNA
ENSBTAP00000011919-D1	2.49	2.43E-02	↓	B. mutus T-box 6 (TBX6), mRNA
ENSP00000313318-D1	2.49	2.43E-02	↓	B. mutus solute carrier family 35 (GDP-fucose transporter), member C1 (SLC35C1), mRNA
ENSBTAP00000023979-D1	2.49	2.43E-02	↓	B. taurus interleukin 27 (IL27), mRNA
ENSBTAP00000010946-D1	2.49	2.43E-02	↓	B. mutus ATPase type 13A2 (ATP13A2), mRNA
ENSBTAP00000026739-D7	2.49	2.43E-02	↓	B. taurus ankyrin repeat domain 26 (ANKRD26), mRNA
ENSP00000263372-D1	2.49	2.43E-02	↓	B. mutus potassium channel, subfamily K, member 6 (KCNK6), mRNA
ENSBTAP00000010179-D1	2.49	2.43E-02	↓	Bubalus bubalis cytokine receptor-like factor 1 (CRLF1), transcript variant X4, mRNA
ENSBTAP00000014977-D1	2.49	2.43E-02	↓	B. mutus leucine rich repeat containing 23 (LRRC23), mRNA
ENSBTAP00000025295-D1	2.49	2.43E-02	↓	B. mutus PR domain containing 10 (PRDM10), transcript variant X1, mRNA
ENSBTAP00000008918-D5	2.49	2.43E-02	↓	B. mutus cytochrome P450 2C9-like (LOC102285833), transcript variant X1, mRNA
ENSBTAP00000027111-D1	2.53	4.95E-28	↓	B. mutus myocilin, trabecular meshwork inducible glucocorticoid response (MYOC), mRNA
ENSP00000320560-D92	2.54	9.81E-06	↓	B. mutus olfactory receptor 10A3-like (LOC102276857), mRNA
yakG028895	2.55	5.96E-05	↓	B. mutus probable ergosterol biosynthetic protein 28-like (LOC102283838), mRNA
ENSP00000420560-D1	2.57	3.67E-04	↓	B. mutus leucine rich repeat containing 61 (LRRC61), mRNA
ENSBTAP00000010295-D1	2.57	5.49E-79	↓	B. mutus thyroglobulin (TG), mRNA
ENSBTAP00000051821-D1	2.59	7.69E-22	↓	B. mutus histone H3.1-like (LOC102280012), mRNA
ENSBTAP00000011067-D1	2.60	3.59E-05	↓	B. mutus ovo-like zinc finger 2 (OVOL2), mRNA
ENSP00000418593-D1	2.60	2.27E-03	↓	B. mutus sorting nexin family member 21 (SNX21), mRNA
ENSP00000399018-D8	2.60	2.27E-03	↓	B. mutus keratin-associated protein 10-12-like (LOC102288366), mRNA
ENSBTAP00000022905-D1	2.60	2.27E-03	↓	B. mutus diacylglycerol kinase, theta 110kDa (DGKQ), mRNA
ENSP00000372135-D1	2.60	2.27E-03	↓	B. mutus chromosome unknown open reading frame, human C15orf52 (LOC102275565), mRNA
ENSBTAP00000037835-D1	2.60	2.27E-03	↓	B. mutus collagen, type XVI, alpha 1 (COL16A1), mRNA
ENSBTAP00000016933-D1	2.64	5.64E-10	↓	B. mutus zinc finger protein 827 (ZNF827), partial mRNA
ENSBTAP00000018376-D1	2.66	5.38E-13	↓	B. mutus allantoicase (ALLC), mRNA
ENSBTAP00000022973-D1	2.66	3.38E-10	↓	Bubalus bubalis uncharacterized LOC102414862 (LOC102414862), mRNA
ENSP00000339952-D1	2.71	1.31E-04	↓	Jaculus jaculus kinase suppressor of ras 2 (Ksr2), mRNA
ENSP00000347169-D2	2.71	1.35E-03	↓	B. mutus numb homolog (Drosophila)-like (NUMBL), mRNA
ENSBTAP00000010033-D1	2.71	1.35E-03	↓	B. mutus interleukin 2 receptor, gamma (IL2RG), transcript variant X1, mRNA
ENSP00000265154-D1	2.71	1.44E-02	↓	B. mutus Rho guanine nucleotide exchange factor (GEF) 38 (ARHGEF38), mRNA
ENSP00000360210-D1	2.71	1.44E-02	↓	B. mutus chromosome unknown open reading frame, human C20orf85 (LOC102272329), mRNA
ENSP00000322649-D1	2.71	1.44E-02	↓	B. mutus solute carrier family 25, member 41 (SLC25A41), mRNA
ENSP00000264833-D3	2.71	1.44E-02	↓	B. mutus noelin-like (LOC102276162), mRNA
ENSP00000368698-D1	2.71	1.44E-02	↓	B. mutus human immunodeficiency virus type I enhancer binding protein 1 (HIVEP1), mRNA
ENSBTAP00000025388-D1	2.71	1.44E-02	↓	B. mutus synaptogyrin 2 (SYNGR2), mRNA
ENSBTAP00000013292-D1	2.71	1.44E-02	↓	B. taurus collagen, type XV, alpha 1 (COL15A1), mRNA
ENSBTAP00000002174-D1	2.71	1.44E-02	↓	B. mutus EH-domain containing 3 (EHD3), mRNA
ENSP00000292431-D1	2.71	1.44E-02	↓	B. mutus nucleus accumbens associated 1, BEN and BTB (POZ) domain containing (NACC1), mRNA
ENSBTAP00000017950-D1	2.71	1.44E-02	↓	B. mutus dehydrogenase/reductase (SDR family) member 13 (DHRS13), mRNA
ENSP00000358823-D1	2.76	7.64E-06	↓	B. mutus deoxyribonuclease I-like 1 (DNASE1L1), mRNA
ENSP00000256649-D1	2.78	7.74E-05	↓	B. mutus tripartite motif containing 45 (TRIM45), transcript variant X1, mRNA
ENSBTAP00000008918-D2	2.78	7.74E-05	↓	B. mutus cytochrome P450 2C19-like (LOC102280731), transcript variant X1, mRNA
ENSP00000382659-D1	2.79	4.45E-07	↓	B. mutus transient receptor potential cation channel, subfamily V, member 1 (TRPV1), mRNA
ENSBTAP00000046985-D1	2.81	7.92E-04	↓	Pantholops hodgsonii ankyrin repeat domain-containing protein 26-like (LOC102332545), misc_RNA
ENSBTAP00000016638-D1	2.81	7.92E-04	↓	B. mutus ferritin heavy chain-like (LOC102271334), mRNA
ENSBTAP00000002194-D1	2.81	7.92E-04	↓	B. mutus inositol-trisphosphate 3-kinase A (ITPKA), mRNA
ENSP00000391363-D1	2.81	7.92E-04	↓	B. mutus bone marrow stromal antigen 2-like (LOC102264642), mRNA
ENSBTAP00000008893-D1	2.81	7.92E-04	↓	B. mutus orthodenticle homeobox 1 (OTX1), mRNA
ENSP00000358765-D1	2.81	7.92E-04	↓	B. mutus trophoblast glycoprotein (TPBG), mRNA
ENSP00000357079-D1	2.81	7.92E-04	↓	B. mutus coiled-coil domain containing 19 (CCDC19), mRNA
ENSBTAP00000037784-D1	2.90	1.57E-06	↓	Bubalus bubalis transducin-like enhancer of split 3 (TLE3), transcript variant X1, mRNA
ENSP00000397380-D1	2.90	4.67E-04	↓	B. mutus lysosomal protein transmembrane 5 (LAPTM5), mRNA

ENSBTAP00000032393-D2	2.90	8.42E-03	↓	B. mutus liver carboxylesterase-like (LOC102281437), mRNA
ENSP00000219271-D1	2.90	8.42E-03	↓	B. taurus matrix metallopeptidase 15 (membrane-inserted) (MMP15), mRNA
ENSP00000350818-D2	2.90	8.42E-03	↓	B. mutus chromosome unknown open reading frame, human C4orf22 (LOC102281592), mRNA
ENSBTAP00000013977-D1	2.90	8.42E-03	↓	B. taurus N-acetyltransferase domain containing 1 (NATD1), mRNA
ENSBTAP00000038578-D1	2.90	8.42E-03	↓	Bubalus bubalis mesoderm posterior 2 homolog (mouse) (MESP2), transcript variant X2, mRNA
ENSBTAP00000029925-D9	2.90	8.42E-03	↓	Pantholops hodgsonii ankyrin repeat domain-containing protein 26-like (LOC102332545), misc_RNA
ENSP00000402682-D3	2.90	8.42E-03	↓	B. mutus protein tyrosine phosphatase, receptor type, M (PTPRM), partial mRNA
ENSP00000178638-D1	2.90	8.42E-03	↓	Bubalus bubalis carbonic anhydrase XII (CA12), transcript variant X2, mRNA
ENSBTAP00000008602-D1	2.90	8.42E-03	↓	B. mutus family with sequence similarity 26, member D (FAM26D), mRNA
ENSP00000309762-D1	2.92	6.26E-13	↓	B. mutus collagen, type VI, alpha 5 (COL6A5), mRNA
ENSBTAP00000041185-D1	2.96	1.59E-05	↓	B. taurus uncharacterized protein MGC137454 (MGC137454), mRNA
ENSBTAP00000023928-D1	3.01	2.51E-15	↓	B. mutus GLIS family zinc finger 1 (GLIS1), mRNA
ENSP00000359375-D1	3.07	1.85E-07	↓	B. mutus fetal and adult testis expressed 1 (FATE1), mRNA
ENSBTAP00000009810-D1	3.07	5.42E-06	↓	B. mutus guanylate cyclase soluble subunit beta-2-like (LOC102285947), mRNA
ENSBTAP00000017182-D1	3.07	4.83E-03	↓	Bubalus bubalis phospholamban (PLN), transcript variant X2, mRNA
ENSBTAP00000035318-D1	3.07	4.83E-03	↓	B. mutus shroom family member 1 (SHROOM1), mRNA
ENSP00000384488-D1	3.07	4.83E-03	↓	B. mutus tetraspanin 9 (TSPAN9), mRNA
ENSBTAP00000020043-D1	3.07	4.83E-03	↓	B. mutus guanylate binding protein 5 (GBP5), mRNA
ENSP00000389863-D1	3.07	4.83E-03	↓	Bubalus bubalis neuregulin 1 (NRG1), transcript variant X6, mRNA
ENSBTAP00000001070-D1	3.07	4.83E-03	↓	B. mutus homeobox protein caupolican-like (LOC102266448), mRNA
ENSP00000230361-D1	3.07	4.83E-03	↓	B. mutus guanylate cyclase activator 1B (retina) (GUCA1B), mRNA
ENSBTAP00000015493-D1	3.07	4.83E-03	↓	B. mutus peptidase M20 domain containing 2 (PM20D2), mRNA
ENSBTAP00000015905-D1	3.07	4.83E-03	↓	B. mutus phospholipid scramblase 4 (PLSCR4), mRNA
ENSBTAP00000026111-D1	3.23	2.11E-08	↓	B. taurus EGF-like, fibronectin type III and laminin G domains (EGFLAM), mRNA
ENSP00000403181-D1	3.23	2.78E-03	↓	Sus scrofa uncharacterized LOC102159665 (LOC102159665), partial mRNA
ENSBTAP00000028930-D1	3.23	2.78E-03	↓	Bubalus bubalis T-box 3 (TBX3), transcript variant X2, mRNA
ENSP00000357448-D1	3.23	2.78E-03	↓	B. mutus pre-B-cell leukemia homeobox interacting protein 1 (PBXIP1), transcript variant X2, mRNA
ENSP00000256953-D1	3.23	2.78E-03	↓	B. mutus RAS-like, estrogen-regulated, growth inhibitor (RERG), partial mRNA
ENSP00000296350-D1	3.23	2.06E-02	↓	B. mutus antigen p97 (melanoma associated) identified by monoclonal antibodies 133.2 and 96.5 (MFI2), mRNA
ENSBTAP00000041073-D1	3.23	2.06E-02	↓	Bubalus bubalis solute carrier family 37 (glucose-6-phosphate transporter), member 1, transcript variant X2, mRNA
ENSP00000298110-D1	3.23	2.06E-02	↓	B. mutus G protein-coupled receptor 101 (GPR101), mRNA
ENSBTAP00000015241-D1	3.23	2.06E-02	↓	B. mutus NXPE family member 3-like (LOC102268328), mRNA
ENSBTAP00000022893-D1	3.23	2.06E-02	↓	B. mutus DEAD (Asp-Glu-Ala-Asp) box polypeptide 51 (DDX51), mRNA
ENSP00000357922-D1	3.23	2.06E-02	↓	Capra hircus chromosome 3 open reading frame, human C1orf56 (C3H1orf56), mRNA
ENSBTAP00000046134-D1	3.23	2.06E-02	↓	B. mutus coiled-coil domain containing 122 (CCDC122), mRNA
ENSP00000384887-D1	3.23	2.06E-02	↓	Bubalus bubalis carcinoembryonic antigen-related cell adhesion molecule 19 (CEACAM19), mRNA
ENSBTAP00000041053-D1	3.23	2.06E-02	↓	B. mutus kaptin (actin binding protein) (KPTN), mRNA
ENSBTAP00000026867-D1	3.23	2.06E-02	↓	B. mutus inositol polyphosphate-5-phosphatase, 145kDa (INPP5D), mRNA
ENSP00000409542-D1	3.23	2.06E-02	↓	Sus scrofa uncharacterized LOC100153473 (LOC100153473), transcript variant X1, mRNA
ENSBTAP00000043123-D1	3.23	2.06E-02	↓	B. mutus basic salivary proline-rich protein 1-like (LOC102282563), mRNA
ENSBTAP00000034717-D1	3.23	2.06E-02	↓	B. mutus heat shock protein, alpha-crystallin-related, B9 (HSPB9), mRNA
ENSP00000392610-D60	3.23	2.06E-02	↓	Homo sapiens 47kB DNA fragment from Xq28, proximal to MTM1 gene
ENSP00000230643-D1	3.23	2.06E-02	↓	Bubalus bubalis polycystic kidney disease 2-like 2 (PKD2L2), transcript variant X1, mRNA
ENSP00000230050-D6	3.23	2.06E-02	↓	Lipotes vexillifer ribosomal protein S12 (RPS12), mRNA
ENSBTAP00000015995-D2	3.23	2.06E-02	↓	B. mutus aldehyde dehydrogenase family 3 member B1-like (LOC102273352), partial mRNA
ENSBTAP00000022428-D1	3.23	2.06E-02	↓	B. mutus lipopolysaccharide-binding protein-like (LOC102281707), mRNA
ENSP00000389924-D19	3.23	2.06E-02	↓	.
ENSBTAP00000017899-D1	3.23	2.06E-02	↓	Bubalus bubalis transmembrane protein 207 (TMEM207), mRNA
ENSP00000266395-D1	3.23	2.06E-02	↓	B. mutus phosphodiesterase 6H, cGMP-specific, cone, gamma (PDE6H), mRNA
yakA21555	3.23	2.06E-02	↓	B. mutus histone H2B type 1-like (LOC102277470), mRNA
ENSP00000355492-D1	3.23	2.06E-02	↓	B. mutus chromosome unknown open reading frame, human C1orf101 (LOC102271330), mRNA
ENSBTAP00000006510-D1	3.23	2.06E-02	↓	B. mutus brain ribonuclease-like (LOC102286301), mRNA
ENSBTAP00000012128-D1	3.23	2.06E-02	↓	B. mutus forkhead box S1 (FOXS1), mRNA
ENSP00000266581-D1	3.23	2.06E-02	↓	B. mutus adhesion molecule with Ig-like domain 2 (AMIGO2), transcript variant X2, mRNA
ENSBTAP00000003759-D1	3.23	2.06E-02	↓	B. mutus 5-hydroxytryptamine (serotonin) receptor 5A, G protein-coupled (HTR5A), mRNA

ENSBTAP00000043637-D8	3.23	2.06E-02	↓	Orycteropus afer afer glyceraldehyde-3-phosphate dehydrogenase-like (LOC103211275), mRNA
ENSBTAP00000019434-D1	3.23	2.06E-02	↓	B. taurus adenylate cyclase 8 (brain) (ADCY8), mRNA
ENSP00000295898-D1	3.23	2.06E-02	↓	B. mutus chromosome unknown open reading frame, human C4orf36 (LOC102272709), mRNA
ENSBTAP00000043466-D1	3.23	2.06E-02	↓	B. mutus signaling threshold regulating transmembrane adaptor 1 (SIT1), mRNA
yakG029857	3.23	2.06E-02	↓	.
ENSP00000246070-D1	3.23	2.06E-02	↓	B. mutus lysosomal-associated membrane protein family, member 5 (LAMP5), transcript variant X1, mRNA
ENSP00000392650-D1	3.23	2.06E-02	↓	.
ENSP00000401894-D21	3.23	2.06E-02	↓	.
ENSP00000336627-D1	3.23	2.06E-02	↓	B. mutus SLIT and NTRK-like family, member 4 (SLITRK4), transcript variant X2, mRNA
ENSP00000305847-D1	3.23	2.06E-02	↓	B. mutus sperm acrosome associated 5 (SPACA5), mRNA
ENSBTAP00000024720-D1	3.23	2.06E-02	↓	B. mutus dihydropyrimidinase-like 5 (DPYSL5), mRNA
ENSP00000361274-D1	3.23	2.06E-02	↓	B. mutus Tctex1 domain containing 4 (TCTEX1D4), mRNA
ENSBTAP00000042943-D1	3.23	2.06E-02	↓	Bubalus bubalis succinate--hydroxymethylglutarate CoA-transferase-like (LOC102405632), mRNA
ENSBTAP00000041356-D1	3.23	2.06E-02	↓	B. mutus autophagy related 9B (ATG9B), mRNA
ENSBTAP00000019750-D1	3.23	2.06E-02	↓	B. mutus protein phosphatase 1, regulatory subunit 32 (PPP1R32), mRNA
ENSBTAP00000048348-D23	3.23	2.06E-02	↓	B. mutus ferritin heavy chain-like (LOC102264775), mRNA
ENSBTAP00000021009-D1	3.23	2.06E-02	↓	B. mutus complement factor properdin (CFP), mRNA
ENSBTAP00000023763-D51	3.23	2.06E-02	↓	B. taurus T cell receptor, beta cluster, mRNA (cDNA clone MGC:133478 IMAGE:8067041), complete cds
ENSP00000311427-D1	3.23	2.06E-02	↓	B. mutus sorting nexin 33 (SNX33), mRNA
ENSP00000402649-D1	3.23	2.06E-02	↓	B. mutus uncharacterized LOC102274235 (LOC102274235), mRNA
ENSBTAP00000011887-D1	3.30	3.09E-05	↓	B. mutus uncharacterized LOC102276152 (LOC102276152), mRNA
ENSBTAP00000012917-D2	3.33	4.63E-28	↓	B. mutus interferon-induced protein 44 (IFI44), mRNA
ENSBTAP00000052860-D1	3.36	1.59E-03	↓	B. mutus thyroid stimulating hormone receptor (TSHR), mRNA
ENSBTAP00000026897-D1	3.36	1.59E-03	↓	B. mutus fibroblast growth factor 13-like (LOC102286664), transcript variant X1, mRNA
ENSBTAP00000027551-D1	3.36	1.59E-03	↓	B. mutus tripartite motif containing 62 (TRIM62), mRNA
ENSBTAP00000053424-D1	3.43	2.88E-20	↓	B. mutus heparanase 2 (HPSE2), mRNA
ENSP00000264128-D5	3.49	9.08E-04	↓	B. mutus calcium-binding mitochondrial carrier protein SCaMC-1-like (LOC102272537), mRNA
ENSBTAP00000015350-D1	3.49	1.17E-02	↓	B. taurus otoferlin (OTOF), mRNA
ENSP00000380061-D8	3.49	1.17E-02	↓	Capra hircus zinc finger with KRAB and SCAN domains 2 (ZKSCAN2), mRNA
ENSP00000257951-D1	3.49	1.17E-02	↓	B. mutus keratin 84 (KRT84), mRNA
ENSP00000249504-D1	3.49	1.17E-02	↓	Ovis aries homeobox D11 (hoxd11) gene, complete cds
yakG032657	3.49	1.17E-02	↓	B. mutus SET binding protein 1 (SETBP1), mRNA
ENSBTAP00000018021-D3	3.49	1.17E-02	↓	B. mutus cytochrome b-245 heavy chain-like (LOC102288244), mRNA
ENSBTAP00000020822-D1	3.49	1.17E-02	↓	B. mutus retinal G protein coupled receptor (RGR), mRNA
ENSBTAP00000001250-D1	3.49	1.17E-02	↓	B. mutus lysophosphatidylcholine acyltransferase 1 (LPCAT1), mRNA
ENSP00000258682-D1	3.49	1.17E-02	↓	Chlorocebus sabaeus calcium/calmodulin-dependent protein kinase II beta, transcript variant X14, mRNA
ENSP00000302274-D3	3.49	1.17E-02	↓	B. mutus complement component 1, q subcomponent, C chain (C1QC), mRNA
ENSP00000347310-D1	3.49	1.17E-02	↓	B. mutus arylsulfatase G (ARSG), mRNA
ENSBTAP00000015212-D1	3.49	1.17E-02	↓	Capra hircus transmembrane protein 8B (TMEM8B), transcript variant X2, mRNA
ENSP00000414287-D1	3.49	1.17E-02	↓	B. mutus macrophage stimulating 1 (hepatocyte growth factor-like) (MST1), mRNA
ENSBTAP00000051603-D2	3.49	1.17E-02	↓	B. mutus olfactory receptor 4C12-like (LOC102270176), mRNA
ENSBTAP00000016038-D1	3.49	1.17E-02	↓	B. mutus radial spoke head 6 homolog A (Chlamydomonas) (RSPH6A), mRNA
ENSP00000332723-D1	3.49	1.17E-02	↓	B. mutus collectin sub-family member 10 (C-type lectin) (COLEC10), mRNA
ENSBTAP00000023500-D1	3.49	1.17E-02	↓	B. mutus interferon-induced guanylate-binding protein 1-like (LOC102279350), mRNA
ENSBTAP00000016291-D1	3.49	1.17E-02	↓	B. mutus chloride channel, voltage-sensitive 2 (CLCN2), transcript variant X1, mRNA
ENSP00000293280-D1	3.49	1.17E-02	↓	B. mutus chemokine (C-C motif) ligand 23 (CCL23), mRNA
ENSBTAP00000041330-D1	3.49	1.17E-02	↓	Bubalus bubalis Down syndrome cell adhesion molecule (DSCAM), mRNA
ENSBTAP00000046511-D1	3.49	1.17E-02	↓	B. mutus transmembrane protein 249 (TMEM249), mRNA
ENSBTAP00000012385-D1	3.49	1.17E-02	↓	B. mutus pannexin 3 (PANX3), mRNA
ENSBTAP00000036928-D1	3.49	1.17E-02	↓	B. mutus UDP glucuronosyltransferase 1 family, polypeptide A6 (UGT1A6), transcript variant X1, mRNA
ENSP00000364110-D2	3.49	1.17E-02	↓	B. mutus P antigen family member 3-like (LOC102274503), mRNA
ENSBTAP00000001650-D1	3.49	1.17E-02	↓	B. mutus sulfotransferase family, cytosolic, 1B, member 1 (SULT1B1), mRNA
ENSBTAP00000017854-D1	3.49	1.17E-02	↓	B. mutus glycosylation-dependent cell adhesion molecule 1-like (LOC102268168), mRNA
ENSBTAP00000040913-D1	3.49	1.17E-02	↓	B. mutus family with sequence similarity 71, member D (FAM71D), mRNA
ENSP00000367922-D1	3.49	1.17E-02	↓	Bubalus bubalis uncharacterized LOC102411542 (LOC102411542), transcript variant X1, mRNA

ENSBTAP00000027181-D1	3.49	1.17E-02	↓	B. mutus methyltransferase like 21C (METTL21C), mRNA
ENSP00000347929-D1	3.49	1.17E-02	↓	B. mutus benzodiazapine receptor (peripheral) associated protein 1 (BZRAP1), mRNA
ENSBTAP00000034611-D1	3.49	1.17E-02	↓	B. mutus transmembrane protein 235 (TMEM235), mRNA
ENSP00000370531-D1	3.49	1.17E-02	↓	B. mutus acyl-coenzyme A thioesterase 6-like (LOC102285809), mRNA
ENSBTAP00000047843-D6	3.49	1.17E-02	↓	B. mutus trace amine-associated receptor 7a-like (LOC102288268), mRNA
ENSBTAP00000053745-D1	3.49	1.17E-02	↓	B. mutus anthrax toxin receptor 1 (ANTXR1), mRNA
ENSP00000263686-D1	3.49	1.17E-02	↓	Bubalus bubalis selectin P (granule membrane protein 140kDa, antigen CD62) (SELP), mRNA
ENSP00000308165-D2	3.56	2.95E-20	↓	B. mutus platelet glycoprotein 4-like (LOC102266632), transcript variant X2, mRNA
ENSBTAP00000051517-D2	3.57	1.28E-08	↓	B. taurus phosphoribosylaminoimidazole carboxylase pseudogene (PAICSP) on chromosome 13
ENSBTAP00000026998-D1	3.60	5.17E-04	↓	Bubalus bubalis synapsin II (SYN2), mRNA
ENSP00000359778-D1	3.60	5.17E-04	↓	B. mutus EGF, latrophilin and seven transmembrane domain containing 1 (ELTD1), mRNA
ENSBTAP00000042963-D1	3.60	5.17E-04	↓	B. mutus tumor necrosis factor ligand superfamily member 12-like (LOC102267096), mRNA
ENSBTAP00000021161-D1	3.60	5.17E-04	↓	B. mutus dermokine (DMKN), mRNA
ENSP00000263405-D1	3.61	6.19E-31	↓	B. mutus FYN binding protein (FYB), mRNA
ENSBTAP00000044174-D1	3.66	1.10E-06	↓	B. mutus cytotoxic and regulatory T cell molecule (CRTAM), mRNA
ENSBTAP00000044658-D1	3.67	1.93E-14	↓	B. taurus methyltransferase like 24 (METTL24), mRNA
ENSBTAP00000030826-D25	3.71	2.96E-04	↓	Human mariner1 transposase gene, complete consensus sequence
ENSBTAP00000030094-D1	3.71	2.96E-04	↓	B. mutus chromosome unknown open reading frame, human C1orf192, transcript variant X1, mRNA
ENSBTAP00000026782-D1	3.71	6.61E-03	↓	B. mutus aquaporin 7 (AQP7), mRNA
ENSP00000324534-D6	3.71	6.61E-03	↓	B. mutus olfactory receptor 5V1-like (LOC102267686), mRNA
ENSBTAP00000047785-D10	3.71	6.61E-03	↓	B. taurus olfactory receptor 1L8 (LOC509074), mRNA
ENSBTAP00000024898-D1	3.71	6.61E-03	↓	B. mutus phosphatidylcholine transfer protein-like (LOC102268322), mRNA
ENSP00000290823-D1	3.71	6.61E-03	↓	B. mutus G protein-coupled receptor 114 (GPR114), mRNA
ENSP00000311291-D1	3.71	6.61E-03	↓	B. mutus solute carrier organic anion transporter family, member 2A1 (SLCO2A1), mRNA
ENSP00000381935-D1	3.71	6.61E-03	↓	B. mutus ovostatin homolog 2-like (LOC102268072), mRNA
ENSP00000295943-D1	3.71	6.61E-03	↓	B. mutus doublecortin-like kinase 3 (DCLK3), mRNA
ENSP00000299345-D4	3.71	6.61E-03	↓	B. mutus cadherin 8, type 2 (CDH8), partial mRNA
ENSP00000297350-D1	3.71	6.61E-03	↓	B. mutus tumor necrosis factor receptor superfamily, member 11b (TNFRSF11B), mRNA
ENSBTAP00000050479-D4	3.71	6.61E-03	↓	.
ENSP00000307831-D1	3.71	6.61E-03	↓	B. mutus G protein-coupled receptor 113 (GPR113), mRNA
ENSBTAP00000015578-D1	3.71	6.61E-03	↓	B. mutus StAR-related lipid transfer (START) domain containing 3 (STARD3), transcript variant X1, mRNA
ENSBTAP00000052397-D1	3.71	6.61E-03	↓	B. mutus guanylate binding protein 2, interferon-inducible (GBP2), mRNA
ENSBTAP00000030518-D1	3.71	6.61E-03	↓	B. mutus protein kinase, cGMP-dependent, type I (PRKG1), transcript variant X1, mRNA
ENSP00000291901-D1	3.71	6.61E-03	↓	Bubalus bubalis troponin T type 1 (skeletal, slow) (TNNT1), transcript variant X1, mRNA
ENSBTAP00000020701-D1	3.71	6.61E-03	↓	B. grunniens breed Tianzhu White heme oxygenase 1 (HMOX1) mRNA, complete cds
ENSBTAP00000012159-D1	3.71	6.61E-03	↓	B. mutus fibulin 7 (FBLN7), mRNA
ENSBTAP00000007132-D1	3.71	6.61E-03	↓	B. mutus potassium voltage-gated channel, Shaw-related subfamily, member 1 (KCNC1), mRNA
ENSBTAP00000024680-D1	3.71	6.61E-03	↓	B. mutus kallikrein-related peptidase 15 (KLK15), mRNA
ENSBTAP00000017217-D1	3.71	6.61E-03	↓	B. mutus 60S ribosomal protein L22-like 1-like (LOC102287535), mRNA
ENSBTAP00000011320-D1	3.71	6.61E-03	↓	B. mutus phosphatase domain containing, paladin 1 (PALD1), mRNA
ENSP00000361287-D1	3.71	6.61E-03	↓	B. mutus S-adenosylmethionine synthase isoform type-1-like (LOC102286442), mRNA
ENSBTAP00000016480-D1	3.71	6.61E-03	↓	B. mutus L-amino-acid oxidase-like (LOC102281829), partial mRNA
ENSBTAP00000050331-D1	3.71	6.61E-03	↓	B. mutus C-type lectin domain family 1, member B (CLEC1B), mRNA
ENSP00000310405-D9	3.71	6.61E-03	↓	B. taurus C-C chemokine receptor type 1-like (LOC529196), mRNA
ENSBTAP00000050020-D1	3.71	6.61E-03	↓	B. mutus sodium channel, non-voltage-gated 1 alpha subunit (SCNN1A), mRNA
ENSBTAP00000032106-D1	3.71	6.61E-03	↓	B. mutus MOB kinase activator 3C (MOB3C), mRNA
ENSP00000408979-D1	3.71	6.61E-03	↓	Bubalus bubalis kinesin family member 5A (KIF5A), mRNA
ENSP00000373964-D1	3.71	6.61E-03	↓	B. mutus smoothelin-like protein 2-like (LOC102286145), mRNA
ENSBTAP00000001818-D1	3.71	6.61E-03	↓	B. mutus solute carrier family 26 (anion exchanger), member 9 (SLC26A9), mRNA
ENSP00000356433-D1	3.81	1.69E-04	↓	B. mutus uronyl-2-sulfotransferase (UST), mRNA
ENSBTAP00000050952-D8	3.85	3.91E-80	↓	B. mutus histone H3.1-like (LOC102275786), mRNA
ENSP00000347205-D1	3.90	9.58E-05	↓	Bubalus bubalis ATP-binding cassette, sub-family A (ABC1), member 2 (ABCA2), partial mRNA
ENSBTAP00000028574-D1	3.90	3.73E-03	↓	B. mutus v-maf avian musculoaponeurotic fibrosarcoma oncogene homolog F (MAFF), mRNA
ENSBTAP00000053425-D1	3.90	3.73E-03	↓	B. mutus thrombospondin, type I, domain containing 4 (THSD4), mRNA
ENSBTAP00000016201-D1	3.90	3.73E-03	↓	B. mutus dipeptidase 1 (renal) (DPEP1), mRNA

ENSBTAP00000018307-D2	3.90	3.73E-03	↓	B. taurus keratin 86 (KRT86), mRNA
ENSBTAP00000009183-D1	3.90	3.73E-03	↓	Bubalus bubalis meiosis 1 associated protein (M1AP), transcript variant X2, mRNA
ENSP00000322546-D32	3.90	3.73E-03	↓	B. mutus olfactory receptor 51Q1-like (LOC102273545), partial mRNA
ENSBTAP00000042331-D2	3.90	3.73E-03	↓	B. mutus potassium voltage-gated channel, Shal-related subfamily, member 2 (KCND2), mRNA
ENSP00000290390-D1	3.90	3.73E-03	↓	B. mutus chromosome unknown open reading frame, human C2orf81 (LOC102267548), mRNA
yakG042071	3.90	3.73E-03	↓	Bubalus bubalis neuregulin 1 (NRG1), transcript variant X9, mRNA
ENSP00000330264-D1	3.99	5.43E-05	↓	B. mutus transmembrane protease, serine 9 (TMPRSS9), mRNA
ENSP00000343948-D1	4.07	3.09E-05	↓	B. mutus transcription factor EB (TFEB), transcript variant X1, mRNA
ENSBTAP00000023983-D1	4.07	2.11E-03	↓	Bubalus bubalis ovostatin homolog 2-like (LOC102391019), mRNA
ENSP00000381329-D1	4.07	2.11E-03	↓	Bubalus bubalis uncharacterized LOC102398014 (LOC102398014), misc_RNA
ENSP00000329735-D1	4.07	2.11E-03	↓	B. mutus family with sequence similarity 212, member A (FAM212A), mRNA
ENSP00000248244-D1	4.07	2.11E-03	↓	B. mutus toll-like receptor adaptor molecule 1 (TICAM1), mRNA
ENSBTAP00000049972-D6	4.07	2.11E-03	↓	B. mutus serpin peptidase inhibitor, clade B (ovalbumin), member 10 (SERPINB10), mRNA
ENSBTAP00000021364-D1	4.07	2.11E-03	↓	B. mutus arylsulfatase A (ARSA), mRNA
ENSBTAP00000001651-D3	4.07	2.11E-03	↓	B. mutus transcription factor AP-2 gamma (activating enhancer binding protein 2 gamma) (TFAP2C), mRNA
ENSBTAP00000025891-D1	4.07	2.11E-03	↓	B. mutus schlafen family member 11 (SLFN11), mRNA
ENSBTAP00000016440-D1	4.07	2.11E-03	↓	B. mutus angiotensinogen (serpin peptidase inhibitor, clade A, member 8) (AGT), mRNA
ENSBTAP00000053589-D6	4.07	2.11E-03	↓	B. mutus N-acetyllactosaminide alpha-1,3-galactosyltransferase-like (LOC102282898), mRNA
ENSBTAP00000005549-D1	4.07	2.11E-03	↓	B. mutus betacellulin (BTC), mRNA
ENSBTAP00000051123-D27	4.07	2.11E-03	↓	B. grunniens interferon-alpha 1 (IFN-alpha 1) mRNA, complete cds
ENSP00000391885-D1	4.07	2.11E-03	↓	B. mutus prostate and testis expressed 3 (PATE3), mRNA
ENSBTAP00000018799-D1	4.15	1.77E-05	↓	B. mutus ankyrin repeat and SOCS box containing 5 (ASB5), mRNA
ENSP00000301061-D1	4.15	1.77E-05	↓	B. mutus wingless-type MMTV integration site family, member 10B (WNT10B), mRNA
ENSBTAP00000052521-D1	4.23	1.19E-03	↓	B. mutus tetratricopeptide repeat domain 34 (TTC34), mRNA
ENSBTAP00000007489-D1	4.23	1.19E-03	↓	B. mutus interleukin 1 receptor-like 2 (IL1RL2), mRNA
ENSBTAP00000051834-D1	4.23	1.19E-03	↓	B. mutus ets homologous factor (EHF), transcript variant X1, mRNA
ENSBTAP00000015041-D1	4.23	1.19E-03	↓	B. mutus zinc finger CCCH-type containing 12A (ZC3H12A), mRNA
ENSP00000270824-D1	4.23	1.19E-03	↓	B. taurus eva-1 homolog B (C. elegans) (EVA1B), mRNA
ENSP00000363345-D1	4.23	1.19E-03	↓	B. mutus solute carrier family 46, member 2 (SLC46A2), mRNA
ENSP00000303482-D1	4.23	1.19E-03	↓	B. mutus receptor accessory protein 4 (REEP4), mRNA
ENSBTAP00000036564-D11	4.23	1.19E-03	↓	Capra hircus SET nuclear oncogene (SET), transcript variant X2, mRNA
ENSP00000266643-D1	4.23	1.19E-03	↓	B. mutus membrane-associated ring finger (C3HC4) 9 (MARCH9), partial mRNA
ENSBTAP00000019461-D1	4.30	5.72E-06	↓	B. mutus actin, alpha 2, smooth muscle, aorta (ACTA2), transcript variant X2, mRNA
ENSP00000420662-D1	4.36	7.86E-11	↓	B. mutus dopamine receptor D3 (DRD3), mRNA
ENSBTAP00000011214-D1	4.36	3.26E-06	↓	B. mutus solute carrier family 38, member 3 (SLC38A3), mRNA
ENSBTAP00000043361-D6	4.36	6.75E-04	↓	B. mutus olfactory receptor-like protein DTMT-like (LOC102283062), mRNA
yakG008778	4.36	6.75E-04	↓	B. mutus aryl hydrocarbon receptor-like (LOC102279195), mRNA
ENSBTAP00000040628-D1	4.36	6.75E-04	↓	B. mutus RERG/RAS-like (RERGL), mRNA
ENSBTAP00000021201-D1	4.36	6.75E-04	↓	B. mutus transmembrane protein 81 (TMEM81), mRNA
ENSBTAP00000006656-D1	4.36	6.75E-04	↓	B. mutus gastric inhibitory polypeptide (GIP), mRNA
ENSBTAP00000012860-D1	4.36	6.75E-04	↓	B. mutus CD70 molecule (CD70), mRNA
ENSP00000389891-D1	4.46	1.46E-11	↓	B. taurus TNF receptor-associated factor 5 (TRAF5), mRNA
ENSBTAP00000023700-D1	4.49	3.85E-04	↓	B. mutus involucrin (IVL), mRNA
ENSP00000362056-D1	4.49	3.85E-04	↓	B. mutus guanylate cyclase activator 1A (retina) (GUCA1A), mRNA
ENSP00000351591-D1	4.49	3.85E-04	↓	B. mutus neuroligin 3 (NLGN3), transcript variant X1, mRNA
ENSBTAP00000043950-D1	4.49	3.85E-04	↓	B. mutus ATP-binding cassette, sub-family C (CFTR/MRP), member 8 (ABCC8), mRNA
ENSP00000377616-D1	4.49	3.85E-04	↓	B. mutus keratin 222 (KRT222), mRNA
ENSP00000264613-D2	4.49	3.85E-04	↓	B. mutus ceruloplasmin-like (LOC102267159), mRNA
ENSBTAP00000022113-D1	4.49	3.85E-04	↓	B. mutus 5-hydroxytryptamine receptor 3C-like (LOC102281219), mRNA
ENSBTAP00000029287-D1	4.60	3.46E-07	↓	B. mutus probable E3 ubiquitin-protein ligase MID2-like (LOC102288161), mRNA
ENSP00000383456-D1	4.60	3.46E-07	↓	B. mutus chromosome unknown open reading frame, human C9orf152 (LOC102266162), mRNA
ENSBTAP00000025557-D1	4.60	2.19E-04	↓	B. mutus BPI fold containing family B, member 2 (BPIFB2), mRNA
ENSBTAP00000034797-D1	4.71	1.25E-04	↓	B. mutus arachidonate lipoxygenase 3 (ALOXE3), mRNA
ENSBTAP00000034009-D1	4.71	1.25E-04	↓	B. mutus S100 calcium binding protein A12 (S100A12), transcript variant X2, mRNA
ENSP00000415638-D1	4.71	1.25E-04	↓	B. mutus leupaxin (LPXN), mRNA

ENSP00000402388-D1	4.71	1.25E-04	↓	B. mutus transmembrane protein 213 (TMEM213), mRNA
ENSP00000293303-D1	4.71	1.25E-04	↓	B. mutus kelch-like family member 10 (KLHL10), transcript variant X1, mRNA
ENSBTAP00000025329-D1	4.71	1.25E-04	↓	Pantholops hodgsonii lipid phosphate phosphatase-related protein type 4-like, transcript variant X1, mRNA
ENSP00000359899-D1	4.76	6.54E-08	↓	B. mutus hypocretin (orexin) receptor 2 (HCRTR2), mRNA
ENSBTAP00000044019-D1	4.81	7.12E-05	↓	B. mutus cadherin-related family member 3 (CDHR3), mRNA
ENSP00000001146-D1	4.81	7.12E-05	↓	B. mutus cytochrome P450 26B1-like (LOC102272258), transcript variant X1, mRNA
ENSP00000290943-D3	4.81	7.12E-05	↓	Pantholops hodgsonii ankyrin repeat domain-containing protein 26-like (LOC102332545), misc_RNA
ENSP00000233616-D1	4.81	7.12E-05	↓	B. mutus mannosyl-oligosaccharide glucosidase (MOGS), mRNA
ENSBTAP00000052020-D2	4.87	3.12E-22	↓	B. mutus bisphosphoglycerate mutase-like (LOC102275256), mRNA
ENSBTAP00000014299-D1	4.90	4.10E-05	↓	B. mutus uncharacterized LOC102267969 (LOC102267969), mRNA
ENSBTAP00000003409-D1	4.90	4.10E-05	↓	B. mutus casein beta (CSN2), mRNA
yakG039836	4.90	4.10E-05	↓	Pteropus alecto stress-induced phosphoprotein 1 (STIP1), mRNA
ENSP00000392763-D1	4.99	2.37E-05	↓	B. mutus solute carrier organic anion transporter family, member 1B3 (SLCO1B3), mRNA
ENSP00000366977-D1	5.07	1.38E-05	↓	B. mutus pleckstrin homology domain containing, family G member 5 (PLEKHG5), transcript variant X1, mRNA
ENSBTAP00000031988-D1	5.15	7.96E-06	↓	B. mutus non-specific cytotoxic cell receptor protein 1 homolog (zebrafish) (NCCRP1), mRNA
ENSBTAP00000009227-D1	5.19	2.72E-10	↓	Bubalus bubalis testis expressed 36 (TEX36), mRNA
ENSP00000356037-D2	5.23	4.62E-06	↓	B. mutus C4b-binding protein alpha chain-like (LOC102272644), mRNA
ENSP00000395916-D1	5.36	1.57E-06	↓	B. mutus eyes absent homolog 4 (Drosophila) (EYA4), transcript variant X1, mRNA
ENSBTAP00000000078-D1	5.36	1.57E-06	↓	Bubalus bubalis complement factor H-related 5 (CFHR5), transcript variant X1, mRNA
ENSP00000284384-D1	5.43	9.20E-07	↓	B. mutus protein kinase C, alpha (PRKCA), mRNA
ENSBTAP00000039155-D1	5.55	3.17E-07	↓	B. mutus calmodulin-like 3 (CALML3), mRNA
ENSP00000395590-D1	5.66	1.12E-07	↓	B. mutus interferon-induced protein 35 (IFI35), mRNA
ENSBTAP00000049737-D52	5.71	6.59E-08	↓	Balaenoptera acutorostrata scammoni zinc finger protein 814 (ZNF814), mRNA
ENSP00000305469-D82	5.81	2.33E-08	↓	B. mutus olfactory receptor 1L8-like (LOC102280006), mRNA
ENSP00000249883-D1	5.95	5.00E-09	↓	B. mutus angiomotin like 2 (AMOTL2), transcript variant X2, mRNA
ENSBTAP00000050287-D1	6.07	1.09E-09	↓	B. mutus protein C19orf12 homolog (LOC102281521), mRNA
ENSBTAP00000021429-D1	8.33	6.43E-35	↓	Bubalus bubalis probable protein BRICK1-like (LOC102411996), mRNA
4-cell vs 8-cell				
ENSBTAP00000042576-D1	-10.68	5.63E-106	↑	protein SSX2-like, partial [Bison bison bison]
yakA26736	-10.49	6.23E-190	↑	beclin-2-like [B. mutus]
ENSBTAP00000001053-D3	-9.63	6.43E-61	↑	folate receptor alpha precursor [B. taurus]
ENSP00000403095-D1	-9.54	5.03E-114	↑	putative upstream-binding factor 1-like protein 1-like [B. mutus]
ENSP00000335578-D1	-9.50	0.00E+00	↑	arginine-fifty homeobox [Bison bison bison]
ENSBTAP00000053629-D1	-9.36	1.25E-52	↑	zinc finger protein 42 homolog [Bison bison bison]
ENSBTAP00000044913-D22	-9.16	2.84E-47	↑	spermatid nuclear transition protein 3-like [B. mutus]
ENSP00000340083-D3	-8.78	6.76E-148	↑	hypothetical protein M91_04855 [B. mutus]
ENSBTAP00000016441-D2	-8.52	3.17E-33	↑	hypothetical protein M91_17770, partial [B. mutus]
ENSBTAP00000048235-D3	-8.37	1.19E-30	↑	hypothetical protein M91_04682, partial [B. mutus]
ENSP00000296486-D1	-8.33	4.74E-30	↑	ethanolamine-phosphate phospho-lyase isoform X1 [B. mutus]
ENSP00000292853-D2	-8.13	8.78E-52	↑	F-box only protein 27, partial [B. mutus]
ENSP00000308575-D1	-7.84	5.87E-23	↑	protein FAM46D [B. mutus]
ENSBTAP00000049813-D1	-7.79	0.00E+00	↑	Double homeobox protein A, partial [B. mutus]
ENSP00000266581-D1	-7.66	9.13E-21	↑	amphoterin-induced protein 2 isoform X1 [B. mutus]
yakG029673	-7.61	3.16E-293	↑	hypothetical protein M91_15046 [B. mutus]
ENSBTAP00000049936-D1	-7.44	0.00E+00	↑	hypothetical protein M91_04889, partial [B. mutus]
ENSP00000265643-D1	-7.40	4.80E-18	↑	galanin peptides preproprotein [B. taurus]
ENSP00000392763-D1	-7.28	7.00E-17	↑	solute carrier organic anion transporter family member 1B3 [B. mutus]
ENSBTAP00000023823-D2	-7.18	6.15E-16	↑	cationic amino acid transporter 3-like [B. mutus]
ENSP00000226382-D1	-7.15	1.06E-15	↑	paired mesoderm homeobox protein 2B [Camelus ferus]
ENSP00000377616-D1	-7.12	1.84E-15	↑	keratin-like protein KRT222 [B. taurus]
ENSBTAP00000050011-D1	-7.06	0.00E+00	↑	zinc finger protein 135-like [B. mutus]
ENSBTAP00000050906-D5	-7.01	2.10E-102	↑	PRAME family member 9/15, partial [B. mutus]
ENSBTAP00000053745-D1	-7.00	1.70E-14	↑	anthrax toxin receptor 1 isoform X1 [Odobenus rosmarus divergens]
ENSP00000336627-D1	-6.94	5.27E-14	↑	SLIT and NTRK-like protein 4-like isoform X1 [B. mutus]
ENSBTAP00000017158-D1	-6.91	9.29E-14	↑	Cellular retinoic acid-binding protein 1, partial [B. mutus]

ENSP00000344361-D18	-6.91	9.29E-14	↑	ras-related protein Rap-1b, partial [Ictidomys tridecemlineatus]
ENSBTAP00000027863-D2	-6.88	1.64E-13	↑	homeobox protein NANOG [B. mutus]
ENSBTAP00000039269-D1	-6.88	1.64E-13	↑	hypothetical protein M91_09989, partial [B. mutus]
ENSBTAP00000001651-D3	-6.84	2.91E-13	↑	transcription factor AP-2 gamma isoform X3 [Ovis aries musimon]
ENSBTAP00000046053-D2	-6.84	2.91E-13	↑	Guanine nucleotide-binding protein G(I)/G(S)/G(O) subunit gamma-11 [B. mutus]
ENSBTAP00000048801-D1	-6.77	9.14E-13	↑	Developmental pluripotency-associated protein 2, partial [B. mutus]
ENSBTAP00000036194-D2	-6.60	1.61E-79	↑	Krueppel-like factor 17, partial [B. mutus]
ENSBTAP00000019461-D1	-6.60	2.66E-21	↑	alpha 2 actin [B. taurus]
ENSBTAP00000013262-D1	-6.49	5.56E-11	↑	Granzyme B, partial [B. mutus]
ENSBTAP00000043209-D1	-6.49	5.56E-11	↑	Chordin, partial [B. mutus]
ENSBTAP00000038553-D1	-6.49	5.56E-11	↑	hypothetical protein M91_03828 [B. mutus]
ENSP00000356037-D2	-6.45	1.01E-10	↑	hypothetical protein M91_02271 [B. mutus]
ENSBTAP00000052463-D1	-6.43	3.08E-19	↑	transcription factor 21 [B. taurus]
ENSBTAP00000036565-D23	-6.31	6.16E-10	↑	PRAME family member 20/21, partial [B. mutus]
ENSBTAP00000031988-D1	-6.31	6.16E-10	↑	Non-specific cytotoxic cell receptor protein 1-like protein, partial [B. mutus]
ENSBTAP00000030173-D3	-6.31	6.16E-10	↑	Pregnancy-associated glycoprotein 2, partial [B. mutus]
ENSP00000367197-D1	-6.31	6.16E-10	↑	secretagogin [B. mutus]
ENSBTAP00000020025-D2	-6.26	1.14E-09	↑	Putative serine protease 41, partial [B. mutus]
ENSBTAP00000047904-D1	-6.20	2.09E-09	↑	hypothetical protein M91_09836, partial [B. mutus]
yakA08242	-6.20	2.09E-09	↑	hypothetical protein M91_18051 [B. mutus]
ENSBTAP00000000213-D1	-6.20	2.09E-09	↑	heparan sulfate glucosamine 3-O-sulfotransferase 5 [B. taurus]
ENSBTAP00000009796-D1	-6.20	2.09E-09	↑	beta-nerve growth factor-like [B. mutus]
ENSP00000396586-D3	-6.20	2.09E-09	↑	hypothetical protein M91_07654 [B. mutus]
ENSBTAP00000007924-D1	-6.20	2.09E-09	↑	Lysozyme C-1 [B. mutus]
ENSBTAP00000049972-D6	-6.20	2.09E-09	↑	serpin B10 [B. mutus]
ENSP00000335008-D2	-6.17	1.37E-45	↑	spindlin-2B-like [B. mutus]
yakG008778	-6.15	3.88E-09	↑	Aryl hydrocarbon receptor [B. mutus]
ENSBTAP00000016462-D1	-6.15	3.88E-09	↑	Z-DNA-binding protein 1, partial [B. mutus]
ENSBTAP00000032595-D1	-6.14	8.09E-116	↑	hypothetical protein M91_03891 [B. mutus]
ENSBTAP00000051834-D1	-6.09	7.22E-09	↑	ETS homologous factor isoform X1 [B. mutus]
ENSP00000403333-D2	-6.04	1.35E-08	↑	Hepatitis A virus cellular receptor 1, partial [B. mutus]
ENSBTAP00000046543-D4	-6.04	1.35E-08	↑	hypothetical protein M91_16213 [B. mutus]
ENSP00000331556-D1	-6.04	1.35E-08	↑	Cylicin-1, partial [B. mutus]
ENSBTAP00000013015-D1	-5.97	2.51E-08	↑	Protein FAM71F1, partial [B. mutus]
ENSBTAP00000006255-D1	-5.97	2.51E-08	↑	olfactory receptor 51I1-like [B. mutus]
ENSBTAP00000005618-D1	-5.97	2.51E-08	↑	hypothetical protein M91_15871 [B. mutus]
ENSP00000321627-D1	-5.97	2.51E-08	↑	Ankyrin repeat and fibronectin type-III domain-containing protein 1, partial [B. mutus]
ENSBTAP00000027520-D1	-5.97	2.51E-08	↑	testis-specific serine/threonine-protein kinase 3 [B. taurus]
ENSBTAP00000040632-D1	-5.91	3.62E-14	↑	Junction plakoglobin, partial [B. mutus]
ENSBTAP00000049914-D6	-5.91	4.72E-08	↑	hypothetical protein M91_12293, partial [B. mutus]
ENSP00000395590-D1	-5.91	4.72E-08	↑	interferon-induced 35 kDa protein [B. mutus]
ENSP00000412673-D1	-5.91	4.72E-08	↑	Gamma-aminobutyric acid receptor subunit rho-1, partial [B. mutus]
ENSBTAP00000038660-D2	-5.84	1.29E-13	↑	protein FAM32A-like [B. mutus]
ENSBTAP00000009227-D1	-5.84	1.29E-13	↑	hypothetical protein M91_11567, partial [B. mutus]
ENSBTAP00000017854-D1	-5.84	8.94E-08	↑	glycosylation-dependent cell adhesion molecule 1-like [B. mutus]
ENSBTAP00000004673-D1	-5.84	8.94E-08	↑	protein FAM216B [B. taurus]
ENSBTAP00000044174-D1	-5.83	5.48E-25	↑	Cytotoxic and regulatory T-cell molecule, partial [B. mutus]
ENSP00000382780-D2	-5.79	1.96E-24	↑	60S ribosomal protein L10a isoform X1 [Colobus angolensis palliatus]
ENSBTAP00000021222-D1	-5.77	1.69E-07	↑	Tetraspanin-2, partial [B. mutus]
yakG039836	-5.77	1.69E-07	↑	Stress-induced-phosphoprotein 1 [B. mutus]
ENSBTAP00000000375-D1	-5.77	1.69E-07	↑	protein KIAA2022 homolog isoform X1 [B. taurus]
yakG034321	-5.77	1.69E-07	↑	hypothetical protein M91_04058 [B. mutus]
ENSBTAP00000008808-D1	-5.77	1.69E-07	↑	prostaglandin D2 receptor-like [B. mutus]
ENSBTAP00000009776-D1	-5.77	1.69E-07	↑	Protein KIAA1199 [B. mutus]
ENSBTAP00000048785-D1	-5.77	1.69E-07	↑	Olfactory receptor 52I2, partial [B. mutus]

ENSP00000384982-D14	-5.77	1.69E-07	↑	hypothetical protein M91_08008, partial [B. mutus]
ENSP00000383643-D14	-5.74	0.00E+00	↑	Zinc finger protein 41, partial [B. mutus]
ENSBTAP00000015493-D1	-5.74	8.77E-13	↑	peptidase M20 domain-containing protein 2 [B. mutus]
ENSP00000322276-D2	-5.70	3.22E-07	↑	Pantetheinase [B. mutus]
ENSP00000302855-D1	-5.70	3.22E-07	↑	Zinc finger protein 280A, partial [B. mutus]
ENSBTAP00000000078-D1	-5.70	3.22E-07	↑	hypothetical protein M91_03590, partial [B. mutus]
ENSP00000270233-D1	-5.70	3.22E-07	↑	basal cell adhesion molecule [B. mutus]
ENSBTAP00000039446-D1	-5.70	3.22E-07	↑	V(D)J recombination-activating protein 2 [B. taurus]
ENSBTAP00000052791-D8	-5.70	3.22E-07	↑	Hemoglobin fetal subunit beta, partial [B. mutus]
ENSBTAP00000052397-D1	-5.62	6.18E-07	↑	Interferon-induced guanylate-binding protein 2, partial [B. mutus]
ENSBTAP00000016291-D1	-5.62	6.18E-07	↑	Chloride channel protein 2, partial [B. mutus]
ENSBTAP00000026668-D1	-5.62	6.18E-07	↑	interleukin-13 receptor subunit alpha-2 [B. mutus]
ENSBTAP00000026275-D1	-5.62	6.18E-07	↑	interleukin-8 [Pantholops hodgsonii]
ENSBTAP00000008743-D1	-5.62	6.18E-07	↑	prolactin-inducible protein homolog [B. mutus]
ENSBTAP00000022103-D1	-5.62	6.18E-07	↑	Fc receptor-like protein 2, partial [B. mutus]
ENSBTAP00000046675-D7	-5.62	6.18E-07	↑	putative ankyrin repeat domain-containing protein ENSP00000383069 [Ovis aries musimon]
ENSBTAP00000048801-D2	-5.58	1.17E-11	↑	hypothetical protein M91_06760, partial [B. mutus]
ENSBTAP00000052590-D2	-5.58	1.17E-11	↑	protein FAM127 [B. taurus]
ENSBTAP00000049212-D4	-5.57	8.91E-31	↑	hypothetical protein M91_02395, partial [B. mutus]
ENSBTAP00000027772-D1	-5.54	1.20E-06	↑	Kallikrein-5 [B. mutus]
ENSBTAP00000049429-D1	-5.54	1.20E-06	↑	Serine protease inhibitor Kazal-type 4, partial [B. mutus]
ENSBTAP00000006187-D1	-5.54	1.20E-06	↑	Mast cell-expressed membrane protein 1, partial [B. mutus]
yakG035046	-5.45	2.31E-06	↑	Alpha-N-acetylgalactosaminide alpha-2,6-sialyltransferase 3 [B. mutus]
ENSP00000417515-D1	-5.45	2.31E-06	↑	maltase-glucoamylase, intestinal isoform X4 [B. taurus]
ENSBTAP00000042513-D1	-5.45	2.31E-06	↑	WD repeat-containing protein 88, partial [B. mutus]
yakA21555	-5.45	2.31E-06	↑	histone H2B type 1-like [B. mutus]
ENSBTAP00000045361-D1	-5.45	2.31E-06	↑	hypothetical protein M91_20371, partial [B. mutus]
ENSP00000257632-D1	-5.45	2.31E-06	↑	uroplakin-3b isoform X3 [Bubalus bubalis]
ENSBTAP00000035220-D1	-5.45	2.31E-06	↑	Oligodendrocyte myelin glycoprotein [B. taurus]
ENSP00000335332-D1	-5.45	2.31E-06	↑	Transmembrane protease serine 12 [B. mutus]
ENSBTAP00000004059-D1	-5.45	2.31E-06	↑	Solute carrier family 22 member 1, partial [B. mutus]
ENSBTAP00000044861-D1	-5.45	2.31E-06	↑	hypothetical protein M91_05965 [B. mutus]
ENSP00000335008-D3	-5.39	2.27E-27	↑	spindlin-2 [B. taurus]
ENSP00000388566-D1	-5.36	3.09E-10	↑	Caspase-13, partial [B. mutus]
ENSBTAP00000008930-D1	-5.36	4.48E-06	↑	hypothetical protein M91_13886 [B. mutus]
ENSP00000324633-D4	-5.36	4.48E-06	↑	Beta-defensin 103A, partial [B. mutus]
ENSBTAP00000034629-D1	-5.36	4.48E-06	↑	zinc finger protein 750 [B. mutus]
ENSP00000368077-D1	-5.36	4.48E-06	↑	C-C motif chemokine 19, partial [B. mutus]
ENSBTAP00000021191-D1	-5.36	4.48E-06	↑	iodotyrosine dehalogenase 1 [B. mutus]
ENSBTAP00000004881-D1	-5.31	6.01E-10	↑	calcium-activated potassium channel subunit beta-4 [Pteropus alecto]
ENSBTAP00000010674-D1	-5.30	1.42E-73	↑	CD99 antigen, partial [B. mutus]
ENSP00000301061-D1	-5.26	1.17E-09	↑	protein Wnt-10b [B. mutus]
ENSBTAP00000032422-D53	-5.26	1.17E-09	↑	mariner transposase [Homo sapiens]
ENSBTAP00000037843-D2	-5.26	8.75E-06	↑	hypothetical protein M91_07312, partial [B. mutus]
ENSBTAP00000034991-D3	-5.26	8.75E-06	↑	Zinc finger protein 322A, partial [B. mutus]
ENSP00000303325-D1	-5.26	8.75E-06	↑	neuromedin-K receptor [B. mutus]
ENSBTAP00000049437-D1	-5.26	8.75E-06	↑	vomeronasal type-1 receptor 2-like [B. mutus]
ENSBTAP00000034579-D1	-5.26	8.75E-06	↑	Alpha-N-acetylgalactosaminide alpha-2,6-sialyltransferase 1, partial [B. mutus]
yakA23725	-5.26	8.75E-06	↑	AP-5 complex subunit beta-1 [B. mutus]
ENSBTAP00000037596-D17	-5.26	8.75E-06	↑	Trace amine-associated receptor 6, partial [B. mutus]
ENSP00000263686-D1	-5.26	8.75E-06	↑	P-selectin [Bubalus bubalis]
ENSBTAP00000030565-D1	-5.16	3.40E-138	↑	zinc finger protein 596-like isoform X1 [Bubalus bubalis]
ENSP00000359693-D2	-5.15	1.72E-05	↑	peptidyl-prolyl cis-trans isomerase FKBP1A-like [B. mutus]
ENSP00000324633-D2	-5.15	1.72E-05	↑	beta-defensin 103A-like [B. mutus]
ENSBTAP00000051035-D78	-5.15	1.72E-05	↑	olfactory receptor 51G2-like [Galeopterus variegatus]

ENSBTAP00000020121-D1	-5.15	1.72E-05	↑	Kallikrein-6 [B. mutus]
ENSP00000295559-D1	-5.15	1.72E-05	↑	kinesin-like protein KIF21B [Bubalus bubalis]
ENSBTAP00000007294-D1	-5.15	1.72E-05	↑	hyaluronidase-4 [B. mutus]
ENSBTAP00000001004-D1	-5.15	1.72E-05	↑	Zinc finger RNA-binding protein 2, partial [B. mutus]
ENSP00000317691-D1	-5.15	1.72E-05	↑	ribonuclease P protein subunit p25 [B. mutus]
ENSP00000409384-D1	-5.15	1.72E-05	↑	sperm-associated antigen 11-like [B. taurus]
ENSBTAP00000022924-D1	-5.15	1.72E-05	↑	RPE-spondin, partial [B. mutus]
yakG044517	-5.15	1.72E-05	↑	Putative overexpressed in colon carcinoma 1 protein-like protein [B. mutus]
ENSBTAP00000041185-D1	-5.09	1.82E-22	↑	hypothetical protein M91_04650, partial [B. mutus]
ENSBTAP00000015648-D2	-5.09	8.72E-09	↑	Iron-sulfur cluster assembly enzyme ISCU, mitochondrial, partial [B. mutus]
ENSBTAP00000029287-D1	-5.04	1.71E-08	↑	Putative E3 ubiquitin-protein ligase MID2, partial [B. mutus]
ENSBTAP00000050441-D1	-5.04	3.40E-05	↑	hypothetical protein M91_01920, partial [B. mutus]
ENSBTAP00000043123-D1	-5.04	3.40E-05	↑	Receptor-transporting protein 4 [B. mutus]
ENSBTAP00000011564-D1	-5.04	3.40E-05	↑	Retinal guanylyl cyclase 1, partial [B. mutus]
ENSBTAP00000049051-D1	-5.04	3.40E-05	↑	surfactant-associated protein 2 precursor [B. taurus]
ENSP00000347310-D1	-5.04	3.40E-05	↑	arylsulfatase G [B. mutus]
ENSP00000337014-D1	-5.04	3.40E-05	↑	Hemojuvelin [B. mutus]
ENSBTAP00000021495-D1	-5.04	3.40E-05	↑	Complement component C9, partial [B. mutus]
ENSBTAP00000046047-D1	-5.04	3.40E-05	↑	Carbonic anhydrase-related protein 10, partial [B. mutus]
ENSP00000348478-D1	-5.04	3.40E-05	↑	Katanin p60 ATPase-containing subunit A-like 2, partial [B. mutus]
ENSBTAP00000015434-D1	-5.04	3.40E-05	↑	izumo sperm-egg fusion protein 1 isoform X1 [B. taurus]
ENSBTAP00000051999-D1	-5.02	1.73E-124	↑	ankyrin repeat domain-containing protein 26-like isoform X2 [B. taurus]
ENSP00000219197-D1	-4.97	3.36E-08	↑	cerebellin-1 [Octodon degus]
ENSBTAP00000043129-D1	-4.91	7.84E-20	↑	kynureninase isoform X1 [Bison bison bison]
ENSBTAP00000006079-D4	-4.91	6.63E-08	↑	Arylacetyl-CoA N-acyltransferase, partial [B. mutus]
ENSBTAP00000024226-D1	-4.91	6.79E-05	↑	interleukin-15 precursor [B. taurus]
ENSBTAP00000008748-D1	-4.91	6.79E-05	↑	hypothetical protein M91_00263, partial [B. mutus]
ENSBTAP00000005144-D2	-4.91	6.79E-05	↑	hypothetical protein M91_07077, partial [B. mutus]
ENSP00000306662-D1	-4.91	6.79E-05	↑	Alpha-1B adrenergic receptor, partial [B. mutus]
ENSP00000395040-D1	-4.91	6.79E-05	↑	Transcription elongation factor SPT5, partial [B. mutus]
ENSBTAP00000003860-D1	-4.91	6.79E-05	↑	Protein phosphatase 1M, partial [B. mutus]
ENSBTAP00000031584-D1	-4.91	6.79E-05	↑	Calcium-binding tyrosine phosphorylation-regulated protein [B. mutus]
ENSBTAP00000031114-D11	-4.91	6.79E-05	↑	putative RNA polymerase II subunit A C-terminal domain phosphatase SSU72-like protein 1-like [Lipotes vexillifer]
ENSBTAP00000048408-D1	-4.91	6.79E-05	↑	hypothetical protein M91_05048, partial [B. mutus]
ENSP00000371711-D1	-4.91	6.79E-05	↑	Doublesex- and mab-3-related transcription factor 1 [B. mutus]
ENSP00000368347-D1	-4.91	6.79E-05	↑	Heat shock protein HSP 90-beta, partial [B. mutus]
ENSP00000292672-D1	-4.91	6.79E-05	↑	CUGBP Elav-like family member 5 isoform X1 [Odobenus rosmarus divergens]
ENSBTAP00000025292-D1	-4.91	6.79E-05	↑	spermatid nuclear transition protein 1 [B. mutus]
ENSP00000314543-D1	-4.91	6.79E-05	↑	melanoma-associated antigen B10-like [B. mutus]
ENSP00000368698-D1	-4.77	2.60E-07	↑	zinc finger protein 40 [B. mutus]
ENSBTAP00000024803-D2	-4.77	1.36E-04	↑	RCC1 domain-containing protein 1, partial [B. mutus]
ENSBTAP00000010444-D1	-4.77	1.36E-04	↑	peroxisomal sarcosine oxidase [B. mutus]
ENSBTAP00000047512-D1	-4.77	1.36E-04	↑	proteasome (prosome, macropain) 26S subunit, ATPase, 6, partial [Danio rerio]
ENSP00000332444-D1	-4.77	1.36E-04	↑	cleavage stimulation factor, 3' pre-RNA, subunit 2, 64kDa, tau variant [B. mutus]
ENSBTAP00000053344-D1	-4.77	1.36E-04	↑	Armadillo repeat-containing protein 3, partial [B. mutus]
ENSBTAP00000005418-D1	-4.77	1.36E-04	↑	probable inactive protein kinase-like protein SgK071 [B. mutus]
ENSBTAP00000036381-D1	-4.77	1.36E-04	↑	LAG1 longevity assurance-like protein 1, partial [B. mutus]
ENSBTAP00000052334-D1	-4.77	1.36E-04	↑	hypothetical protein M91_15088, partial [B. mutus]
ENSBTAP00000045461-D2	-4.77	1.36E-04	↑	POTE ankyrin domain family member A, partial [B. mutus]
ENSP00000323555-D18	-4.77	1.36E-04	↑	olfactory receptor 8K3-like isoform X2 [Ovis aries musimon]
ENSBTAP00000048590-D2	-4.77	2.75E-162	↑	transcription elongation factor B polypeptide 2-like [B. mutus]
ENSP00000363344-D74	-4.75	0.00E+00	↑	Zinc finger imprinted 3, partial [B. mutus]
ENSBTAP00000052860-D1	-4.70	5.17E-07	↑	Thyrotropin receptor, partial [B. mutus]
ENSP00000384611-D1	-4.70	5.17E-07	↑	Potassium voltage-gated channel subfamily G member 3, partial [B. mutus]

ENSP00000334225-D1	-4.70	5.17E-07	↑	UPF0258 protein KIAA1024-like protein, partial [B. mutus]
ENSP00000406035-D1	-4.70	5.17E-07	↑	Antigen peptide transporter 2, partial [B. mutus]
ENSBTAP00000049986-D1	-4.67	2.23E-190	↑	hypothetical protein M91_19384, partial [B. mutus]
ENSBTAP00000007079-D1	-4.66	0.00E+00	↑	zinc finger CCHC domain-containing protein 10 [Oryctolagus cuniculus]
ENSP00000371553-D1	-4.66	0.00E+00	↑	NANOG neighbor homeobox, partial [B. mutus]
ENSBTAP00000023885-D2	-4.64	4.87E-239	↑	hypothetical protein M91_19385, partial [B. mutus]
ENSP00000216513-D1	-4.62	1.04E-06	↑	Homeobox protein SIX4, partial [B. mutus]
ENSBTAP00000021161-D1	-4.62	1.04E-06	↑	Dermokine [B. mutus]
ENSBTAP00000001331-D1	-4.62	2.73E-04	↑	Endothelial cell-selective adhesion molecule [B. mutus]
ENSP00000358160-D2	-4.62	2.73E-04	↑	histone H3.1-like [B. mutus]
ENSP00000382146-D1	-4.62	2.73E-04	↑	C-type lectin domain family 19 member A-like [B. mutus]
yakG014383	-4.62	2.73E-04	↑	hypothetical protein M91_06300 [B. mutus]
ENSBTAP00000049500-D14	-4.62	2.73E-04	↑	hypothetical protein M91_19872, partial [B. mutus]
ENSBTAP00000010289-D1	-4.62	2.73E-04	↑	nephrin [B. mutus]
ENSBTAP00000013535-D1	-4.62	2.73E-04	↑	Growth hormone-regulated TBC protein 1, partial [B. mutus]
ENSBTAP00000000139-D1	-4.62	2.73E-04	↑	Fibroblast growth factor 18, partial [B. mutus]
ENSBTAP00000042187-D4	-4.62	2.73E-04	↑	60S ribosomal protein L39 [Jaculus jaculus]
ENSBTAP00000041545-D1	-4.62	2.73E-04	↑	1-phosphatidylinositol 4,5-bisphosphate phosphodiesterase delta-4 [B. mutus]
ENSP00000342162-D1	-4.62	2.73E-04	↑	Transmembrane protein 221, partial [B. mutus]
ENSP00000231004-D1	-4.62	2.73E-04	↑	protein-lysine 6-oxidase isoform X1 [B. taurus]
ENSBTAP00000021364-D1	-4.62	2.73E-04	↑	Arylsulfatase A, partial [B. mutus]
ENSBTAP00000032976-D1	-4.62	2.73E-04	↑	SH2 domain-containing protein 6, partial [B. mutus]
ENSP00000374205-D1	-4.62	2.73E-04	↑	T-box brain protein 1 [B. mutus]
yakG022501	-4.62	2.73E-04	↑	E3 ubiquitin-protein ligase MARCH1 [B. mutus]
ENSP00000270824-D1	-4.62	2.73E-04	↑	Protein FAM176B, partial [B. mutus]
ENSBTAP00000053809-D1	-4.62	2.73E-04	↑	disintegrin and metalloproteinase domain-containing protein 7 isoform X2 [Ovis aries]
ENSBTAP00000030207-D2	-4.62	2.73E-04	↑	hypothetical protein M91_08980, partial [B. mutus]
ENSBTAP00000050537-D1	-4.62	0.00E+00	↑	hypothetical protein M91_02062, partial [B. mutus]
ENSP00000263405-D1	-4.58	2.19E-57	↑	FYN-binding protein, partial [B. mutus]
ENSBTAP00000005986-D1	-4.54	2.08E-06	↑	Dehydrogenase/reductase SDR family member 9, partial [B. mutus]
ENSBTAP00000052032-D1	-4.54	2.08E-06	↑	natural cytotoxicity triggering receptor 3 [B. mutus]
ENSP00000252813-D1	-4.54	2.08E-06	↑	Pyroglutamyl-peptidase 1, partial [B. mutus]
ENSBTAP00000012030-D1	-4.54	2.08E-06	↑	Aldehyde dehydrogenase family 1 member A3, partial [B. mutus]
ENSP00000397197-D1	-4.54	2.08E-06	↑	Inhibin beta A chain [B. mutus]
ENSBTAP00000015499-D1	-4.54	2.08E-06	↑	histone H1.4-like [B. mutus]
ENSP00000390478-D1	-4.54	2.08E-06	↑	three prime repair exonuclease 1 [B. mutus]
ENSBTAP00000031240-D1	-4.49	1.33E-10	↑	Anthrax toxin receptor-like protein, partial [B. mutus]
ENSBTAP00000015745-D1	-4.45	3.65E-31	↑	protocadherin-20-like [B. mutus]
ENSP00000379689-D96	-4.45	4.17E-06	↑	hypothetical protein M91_11867, partial [B. mutus]
ENSBTAP00000029684-D1	-4.45	5.56E-04	↑	Glycosyltransferase 1 domain-containing protein 1, partial [B. mutus]
ENSP00000312099-D1	-4.45	5.56E-04	↑	Beta-crystallin S, partial [B. mutus]
ENSP00000402929-D1	-4.45	5.56E-04	↑	Tumor necrosis factor receptor superfamily member 27 [B. mutus]
ENSBTAP00000049713-D4	-4.45	5.56E-04	↑	protocadherin beta-6 [B. mutus]
ENSP00000359663-D1	-4.45	5.56E-04	↑	CD40 ligand [B. taurus]
ENSP00000369738-D70	-4.45	5.56E-04	↑	hypothetical protein M91_01591, partial [B. mutus]
ENSBTAP00000032154-D17	-4.45	5.56E-04	↑	Zinc finger protein 550, partial [B. mutus]
yakG040942	-4.45	5.56E-04	↑	D(4) dopamine receptor [B. mutus]
ENSP00000334042-D1	-4.45	5.56E-04	↑	Tachykinin-4, partial [B. mutus]
ENSP00000368207-D6	-4.45	5.56E-04	↑	Ferritin heavy chain [B. mutus]
ENSBTAP00000013951-D1	-4.45	5.56E-04	↑	sodium/potassium-transporting ATPase subunit alpha-4 [B. mutus]
ENSBTAP00000025090-D1	-4.45	5.56E-04	↑	Serpin A11, partial [B. mutus]
ENSP00000218008-D1	-4.45	5.56E-04	↑	protein ATP1B4 [B. taurus]
ENSBTAP00000010124-D1	-4.45	5.56E-04	↑	transmembrane protein 59-like precursor [B. taurus]
ENSBTAP00000027836-D1	-4.45	5.56E-04	↑	Matrilin-3, partial [B. mutus]
ENSBTAP00000006849-D1	-4.43	1.31E-190	↑	tRNA wybutosine-synthesizing protein 3 homolog [B. taurus]

ENSBTAP00000000066-D1	-4.42	2.14E-26	↑	PR domain zinc finger protein 14 [B. mutus]
ENSBTAP00000052020-D2	-4.42	3.49E-14	↑	hypothetical protein M91_16189 [B. mutus]
ENSP00000262041-D1	-4.39	6.27E-22	↑	homeobox protein MOX-2-like [Sus scrofa]
ENSBTAP00000050518-D4	-4.36	8.35E-06	↑	hypothetical protein M91_11552, partial [B. mutus]
ENSP00000368727-D1	-4.32	2.80E-13	↑	Xanthine dehydrogenase/oxidase, partial [B. mutus]
ENSP00000242719-D1	-4.27	2.04E-45	↑	RING finger protein 11 [Ceratotherium simum simum]
ENSBTAP00000013990-D1	-4.26	4.25E-09	↑	SH3 domain-binding glutamic acid-rich-like protein [B. taurus]
ENSBTAP00000009013-D1	-4.26	1.69E-05	↑	Homeobox protein Meis3, partial [B. mutus]
ENSBTAP00000026008-D1	-4.26	1.69E-05	↑	integrin alpha-D [B. mutus]
ENSP00000334448-D5	-4.26	1.69E-05	↑	Guanine nucleotide-binding protein G(I)/G(S)/G(O) subunit gamma-5, partial [B. mutus]
ENSBTAP00000006339-D1	-4.26	1.69E-05	↑	Tartrate-resistant acid phosphatase type 5, partial [B. mutus]
ENSBTAP00000001766-D1	-4.26	1.13E-03	↑	protein Wnt-5b isoform X1 [B. taurus]
ENSBTAP00000048923-D1	-4.26	1.13E-03	↑	hypothetical protein M91_18609, partial [B. mutus]
ENSBTAP00000016978-D1	-4.26	1.13E-03	↑	histone H2B subacrosomal variant-like [B. mutus]
ENSP00000402649-D1	-4.26	1.13E-03	↑	hypothetical protein M91_07472, partial [B. mutus]
ENSP00000366977-D1	-4.26	1.13E-03	↑	Pleckstrin-like protein domain-containing family G member 5, partial [B. mutus]
ENSP00000317818-D1	-4.26	1.13E-03	↑	PILR alpha-associated neural protein precursor [B. taurus]
ENSBTAP00000028950-D1	-4.26	1.13E-03	↑	Poly(ADP-ribose) glycohydrolase ARH3, partial [B. mutus]
yakA25805	-4.26	1.13E-03	↑	Lipopolysaccharide-binding protein [B. mutus]
ENSP00000410106-D1	-4.26	1.13E-03	↑	Tenascin-R, partial [B. mutus]
ENSBTAP00000050468-D1	-4.26	1.13E-03	↑	.
ENSP00000382434-D1	-4.26	1.13E-03	↑	cytosolic phospholipase A2 epsilon [B. mutus]
ENSP00000369252-D1	-4.26	1.13E-03	↑	leucine-rich repeat, immunoglobulin-like domain and transmembrane domain-containing protein 3 [B. mutus]
ENSBTAP00000051204-D1	-4.26	1.13E-03	↑	calcium/calmodulin-dependent 3',5'-cyclic nucleotide phosphodiesterase 1A isoform X1 [B. mutus]
ENSP00000299824-D1	-4.26	1.13E-03	↑	protein phosphatase 1 regulatory inhibitor subunit 16B [B. taurus]
ENSP00000377347-D1	-4.26	1.13E-03	↑	unnamed protein product [Homo sapiens]
ENSBTAP00000017105-D59	-4.26	1.13E-03	↑	zinc finger protein 83-like [B. mutus]
ENSBTAP00000051584-D52	-4.26	1.13E-03	↑	endogenous retrovirus group K member 11 Pol protein-like [Bison bison bison]
ENSP00000303482-D1	-4.26	1.13E-03	↑	receptor expression-enhancing protein 4 [B. mutus]
ENSBTAP00000017244-D1	-4.26	1.13E-03	↑	E3 ubiquitin-protein ligase RNF167, partial [B. mutus]
ENSBTAP00000038763-D1	-4.26	1.13E-03	↑	hypothetical protein M91_03153, partial [B. mutus]
ENSBTAP00000014630-D45	-4.26	1.13E-03	↑	mariner transposase [Homo sapiens]
ENSBTAP00000047173-D19	-4.26	1.13E-03	↑	PRAME family member 12 isoform X2 [B. taurus]
ENSBTAP00000042213-D5	-4.26	1.13E-03	↑	hypothetical protein M91_00741, partial [B. mutus]
ENSP00000365838-D1	-4.26	1.13E-03	↑	divergent paired-related homeobox-like [B. taurus]
ENSBTAP00000017815-D1	-4.26	1.13E-03	↑	Protein FAM57A, partial [B. mutus]
ENSP00000392188-D5	-4.26	1.13E-03	↑	hypothetical protein M91_20590, partial [B. mutus]
ENSBTAP00000034822-D2	-4.26	1.13E-03	↑	solute carrier family 22 member 9 [B. mutus]
ENSBTAP00000006851-D1	-4.26	1.13E-03	↑	fibroblast growth factor 1 [B. taurus]
ENSBTAP00000010033-D1	-4.20	8.52E-09	↑	cytokine receptor common subunit gamma precursor [B. taurus]
ENSBTAP00000026288-D1	-4.15	1.70E-08	↑	ankyrin repeat and EF-hand domain-containing protein 1 [B. mutus]
ENSBTAP00000052476-D1	-4.15	1.70E-08	↑	Serpin B7 [B. mutus]
ENSBTAP00000025927-D1	-4.15	3.40E-05	↑	T-complex protein 1 subunit zeta isoform X1 [Ovis aries]
ENSBTAP00000033782-D1	-4.15	3.40E-05	↑	leucine-rich repeat and death domain-containing protein 1 [B. mutus]
ENSP00000302569-D1	-4.15	3.40E-05	↑	C-type lectin domain family 7 member A [B. mutus]
ENSBTAP00000046780-D2	-4.15	3.40E-05	↑	hypothetical protein M91_13119, partial [B. mutus]
ENSP00000358817-D1	-4.15	3.40E-05	↑	Macrophage colony-stimulating factor 1, partial [B. mutus]
ENSBTAP00000002568-D1	-4.15	3.40E-05	↑	Transcription initiation factor TFIID subunit 7-like protein, partial [B. mutus]
ENSBTAP00000048201-D5	-4.15	3.40E-05	↑	histone H2B type 1-J-like [Sorex araneus]
ENSBTAP00000030514-D1	-4.13	2.68E-21	↑	C-type lectin domain family 12 member B [B. mutus]
ENSBTAP00000028634-D1	-4.09	3.40E-08	↑	carbonic anhydrase 14 [B. mutus]
ENSP00000351254-D1	-4.04	0.00E+00	↑	putative methyl-CpG-binding domain protein 3-like 3 [B. mutus]
ENSP00000411217-D1	-4.04	7.20E-11	↑	protein CC2D2B isoform X31 [B. taurus]
ENSP00000302289-D1	-4.04	6.84E-08	↑	Embigin, partial [B. mutus]
ENSBTAP00000005603-D1	-4.04	6.90E-05	↑	Adipocyte plasma membrane-associated protein, partial [B. mutus]

ENSBTAP00000003353-D1	-4.04	6.90E-05	↑	mRNA-decapping enzyme 1B [B. mutus]
ENSP00000283309-D1	-4.04	6.90E-05	↑	FERM domain-containing protein 1, partial [B. mutus]
ENSP00000403181-D1	-4.04	6.90E-05	↑	hypothetical protein M91_00185, partial [B. mutus]
ENSBTAP00000019834-D1	-4.04	2.33E-03	↑	hexokinase-3 [B. mutus]
ENSBTAP00000044787-D1	-4.04	2.33E-03	↑	Protein FAM162B, partial [B. mutus]
yakG002415	-4.04	2.33E-03	↑	hypothetical protein M91_01478 [B. mutus]
ENSBTAP00000051494-D1	-4.04	2.33E-03	↑	hypothetical protein M91_17997, partial [B. mutus]
ENSP00000309087-D1	-4.04	2.33E-03	↑	PLAC8-like protein 1 [B. taurus]
ENSP00000413405-D1	-4.04	2.33E-03	↑	GPI mannosyltransferase 4, partial [B. mutus]
ENSP00000413517-D1	-4.04	2.33E-03	↑	putative uncharacterized protein FLJ13197-like [Tursiops truncatus]
ENSP00000264613-D2	-4.04	2.33E-03	↑	Ceruloplasmin [B. mutus]
ENSP00000285402-D1	-4.04	2.33E-03	↑	outer dense fiber protein 1 [B. mutus]
ENSP00000290953-D1	-4.04	2.33E-03	↑	agouti-related protein isoform X1 [B. taurus]
ENSP00000401306-D1	-4.04	2.33E-03	↑	fibrous sheath-interacting protein 2-like, partial [B. mutus]
ENSP00000266068-D1	-4.04	2.33E-03	↑	glucocorticoid modulatory element-binding protein 2 [B. mutus]
ENSP00000365392-D2	-4.04	2.33E-03	↑	major histocompatibility class I related protein isoform X2 [B. taurus]
ENSP00000299441-D1	-4.04	2.33E-03	↑	Protocadherin-16 [B. mutus]
ENSP00000283025-D1	-4.04	2.33E-03	↑	tektin-5 [B. mutus]
ENSP00000311030-D1	-4.04	2.33E-03	↑	Multidrug resistance-associated protein 9 [B. mutus]
ENSBTAP00000044019-D1	-4.04	2.33E-03	↑	Cadherin-related family member 3, partial [B. mutus]
ENSBTAP00000051598-D38	-4.04	2.33E-03	↑	Interferon tau-1, partial [B. mutus]
ENSP00000256324-D1	-4.04	2.33E-03	↑	UPF0317 protein C14orf159 homolog, mitochondrial [Balaenoptera acutorostrata scammoni]
ENSBTAP00000035721-D65	-4.04	2.33E-03	↑	hypothetical protein M91_16788, partial [B. mutus]
ENSBTAP00000051671-D48	-4.04	2.33E-03	↑	hypothetical protein M91_03766 [B. mutus]
ENSP00000307911-D1	-4.04	2.33E-03	↑	Carbohydrate sulfotransferase 2 [Mus musculus]
ENSBTAP00000018244-D1	-4.04	2.33E-03	↑	Glutamate receptor delta-2 subunit, partial [B. mutus]
ENSBTAP00000042136-D2	-4.00	7.03E-57	↑	spindlin-like [B. taurus]
ENSP00000366953-D1	-3.97	1.37E-07	↑	DDB1- and CUL4-associated factor 10, partial [B. mutus]
ENSP00000389891-D1	-3.97	1.37E-07	↑	TNF receptor-associated factor 5 [B. mutus]
yakG041951	-3.96	6.42E-16	↑	hypothetical protein M91_01555 [B. mutus]
ENSBTAP00000050581-D2	-3.91	0.00E+00	↑	Ribose-phosphate pyrophosphokinase 1, partial [B. mutus]
ENSP00000355142-D1	-3.91	2.75E-07	↑	Membrane progestin receptor beta, partial [B. mutus]
ENSP00000380523-D1	-3.91	1.40E-04	↑	calcyphosin-like protein isoform X2 [Ovis aries]
ENSBTAP00000030094-D1	-3.91	1.40E-04	↑	hypothetical protein M91_20772 [B. mutus]
ENSP00000333980-D11	-3.91	1.40E-04	↑	Zinc finger protein 416, partial [B. mutus]
ENSBTAP00000053257-D1	-3.89	1.10E-17	↑	alpha-protein kinase 1 [B. mutus]
ENSP00000415860-D1	-3.88	4.22E-41	↑	protocadherin-15 isoform X3 [Capra hircus]
ENSBTAP00000026562-D1	-3.87	1.15E-09	↑	UDP-N-acetylglucosamine/UDP-glucose/GDP-mannose transporter, partial [B. mutus]
ENSBTAP00000038660-D1	-3.86	1.55E-45	↑	TPA: hypothetical protein BOS_18285 [B. taurus]
ENSP00000367193-D1	-3.85	9.33E-20	↑	F-box only protein 48 [B. taurus]
ENSP00000362748-D1	-3.85	4.81E-60	↑	methylcytosine dioxygenase TET1 [B. mutus]
ENSBTAP00000053188-D1	-3.84	4.00E-22	↑	adenylate kinase 7 [B. mutus]
ENSP00000318770-D1	-3.84	4.85E-12	↑	aquaporin-11 isoform X1 [B. taurus]
ENSP00000243189-D1	-3.81	2.15E-70	↑	UPF0471 protein C1orf63 homolog [B. mutus]
ENSP00000261170-D1	-3.81	1.58E-21	↑	heat-stable enterotoxin receptor [B. mutus]
ENSP00000343706-D1	-3.77	8.18E-14	↑	melanoma-associated antigen H1 [B. taurus]
ENSBTAP00000043397-D11	-3.77	1.11E-06	↑	hypothetical protein M91_02194 [B. mutus]
ENSP00000384193-D1	-3.77	1.11E-06	↑	cell adhesion molecule 2 isoform X1 [B. taurus]
ENSBTAP00000051904-D1	-3.77	2.83E-04	↑	uncharacterized protein C16orf52 homolog [Jaculus jaculus]
ENSBTAP00000025295-D1	-3.77	2.83E-04	↑	PR domain zinc finger protein 10 [B. mutus]
ENSBTAP00000047643-D68	-3.77	2.83E-04	↑	uncharacterized protein LOC105605376 [Ovis aries]
ENSBTAP00000044518-D1	-3.77	2.83E-04	↑	RING finger protein 151, partial [B. mutus]
ENSBTAP00000014308-D1	-3.77	2.83E-04	↑	carboxymethylenebutenolidase homolog [B. mutus]
ENSBTAP00000006236-D1	-3.77	2.83E-04	↑	B-lymphocyte antigen CD20 [B. mutus]
ENSBTAP00000034009-D1	-3.77	4.84E-03	↑	protein S100-A12 isoform X1 [B. mutus]

ENSBTAP00000017274-D1	-3.77	4.84E-03	↑	cadherin-6 precursor [B. taurus]
ENSBTAP00000011569-D1	-3.77	4.84E-03	↑	Potassium-transporting ATPase alpha chain 1, partial [B. mutus]
ENSBTAP00000019695-D1	-3.77	4.84E-03	↑	Carboxypeptidase Z, partial [B. mutus]
ENSP00000249883-D1	-3.77	4.84E-03	↑	angiomotin-like protein 2 isoform X2 [B. mutus]
ENSP00000225823-D1	-3.77	4.84E-03	↑	Amiloride-sensitive cation channel 1, neuronal, partial [B. mutus]
ENSBTAP00000028547-D1	-3.77	4.84E-03	↑	transmembrane and coiled-coil domain-containing protein 5B-like [B. mutus]
ENSP00000397695-D1	-3.77	4.84E-03	↑	Zinc finger protein ZIC 4, partial [B. mutus]
ENSBTAP00000018130-D1	-3.77	4.84E-03	↑	killer cell lectin-like receptor subfamily G member 1 [B. mutus]
ENSP00000290871-D1	-3.77	4.84E-03	↑	Testis, prostate and placenta-expressed protein, partial [B. mutus]
ENSBTAP00000045681-D1	-3.77	4.84E-03	↑	Immunoglobulin superfamily DCC subclass member 3, partial [B. mutus]
ENSP00000330812-D1	-3.77	4.84E-03	↑	Putative short-chain dehydrogenase/reductase family 42E member 2, partial [B. mutus]
ENSBTAP00000038597-D1	-3.77	4.84E-03	↑	Synaptic vesicle glycoprotein 2B, partial [B. mutus]
ENSBTAP00000023232-D1	-3.77	4.84E-03	↑	Krueppel-like factor 3 isoform X1 [Physeter catodon]
ENSP00000042931-D1	-3.77	4.84E-03	↑	bestrophin-2 [B. mutus]
ENSBTAP00000016726-D1	-3.77	4.84E-03	↑	tektin-4-like [B. mutus]
ENSBTAP00000016860-D82	-3.77	4.84E-03	↑	zinc finger protein 485, partial [Capra hircus]
ENSP00000349770-D1	-3.77	4.84E-03	↑	interferon regulatory factor 5 isoform X1 [B. mutus]
ENSBTAP00000053057-D3	-3.77	4.84E-03	↑	Complement factor H-related protein 3, partial [B. mutus]
ENSBTAP00000015578-D1	-3.77	4.84E-03	↑	stAR-related lipid transfer protein 3 isoform X1 [B. mutus]
ENSBTAP00000006958-D1	-3.77	4.84E-03	↑	GDNF family receptor alpha-3, partial [B. mutus]
ENSP00000411354-D1	-3.77	4.84E-03	↑	hypothetical protein M91_16574 [B. mutus]
ENSBTAP00000037863-D1	-3.77	4.84E-03	↑	Protein FAM26F [B. mutus]
ENSP00000288048-D1	-3.77	4.84E-03	↑	uncharacterized protein C1orf158 homolog [B. mutus]
ENSP00000321744-D1	-3.77	4.84E-03	↑	histone H2B type 1-like [Propithecus coquereli]
ENSBTAP00000024720-D1	-3.77	4.84E-03	↑	dihydropyrimidinase-related protein 5 isoform X2 [Ovis aries musimon]
ENSP00000292853-D5	-3.77	0.00E+00	↑	hypothetical protein M91_07075 [B. mutus]
ENSBTAP00000006879-D1	-3.73	2.63E-36	↑	hypothetical protein M91_13902, partial [B. mutus]
ENSBTAP00000045803-D1	-3.70	1.56E-26	↑	splicing regulator RBM11 [B. mutus]
ENSP00000386167-D18	-3.70	7.75E-11	↑	hypothetical protein M91_01396 [B. mutus]
ENSBTAP00000006298-D1	-3.70	2.24E-06	↑	Putative tRNA (uracil-O(2)-)-methyltransferase, partial [B. mutus]
ENSP00000265431-D1	-3.67	1.64E-34	↑	Calbindin [B. mutus]
ENSBTAP00000051517-D2	-3.67	1.83E-08	↑	multifunctional protein ADE2 isoform X1 [Bubalus bubalis]
ENSBTAP00000012280-D1	-3.67	1.83E-08	↑	septin-14 [B. mutus]
ENSP00000340211-D1	-3.67	1.83E-08	↑	Coronin-1B [B. mutus]
ENSBTAP00000050537-D2	-3.65	1.14E-122	↑	zinc finger and SCAN domain-containing protein 4 [Bison bison bison]
ENSP00000390428-D2	-3.62	9.71E-25	↑	Creatine kinase S-type, mitochondrial [B. mutus]
ENSP00000327890-D1	-3.62	2.57E-12	↑	Interleukin-3 receptor subunit alpha, partial [B. mutus]
ENSP00000347041-D1	-3.62	3.65E-08	↑	fibromodulin [B. mutus]
ENSBTAP00000045034-D4	-3.62	4.47E-06	↑	interferon beta-2-like [B. mutus]
ENSBTAP00000007794-D1	-3.62	4.47E-06	↑	Meiotic recombination protein DMC1/LIM15-like protein, partial [B. mutus]
ENSP00000296098-D1	-3.62	5.77E-04	↑	Tripartite motif-containing protein 54, partial [B. mutus]
ENSBTAP00000023012-D1	-3.62	5.77E-04	↑	AT-hook-containing transcription factor [B. mutus]
ENSBTAP00000028930-D1	-3.62	5.77E-04	↑	T-box transcription factor TBX3-like [Balaenoptera acutorostrata scammoni]
ENSBTAP00000045721-D1	-3.62	5.77E-04	↑	angiotensin-converting enzyme 2 [B. mutus]
ENSP00000361569-D1	-3.62	5.77E-04	↑	Immediate early response 5-like protein, partial [B. mutus]
ENSP00000370483-D2	-3.62	5.77E-04	↑	Neuroligin-4, X-linked, partial [B. mutus]
ENSBTAP00000053754-D1	-3.60	2.36E-30	↑	WD repeat-containing protein 72, partial [B. mutus]
ENSBTAP00000026699-D1	-3.58	4.43E-82	↑	kelch-like protein 28 [B. taurus]
ENSP00000364429-D1	-3.58	8.82E-132	↑	glutamate-rich protein 2 [Propithecus coquereli]
ENSBTAP00000026537-D1	-3.54	1.22E-09	↑	bicaudal D-related protein 2 [B. mutus]
ENSBTAP00000009302-D1	-3.54	8.92E-06	↑	large neutral amino acids transporter small subunit 2 [B. mutus]
ENSP00000362258-D1	-3.54	8.92E-06	↑	UNC5C-like protein [B. mutus]
yakG001076	-3.54	8.92E-06	↑	hypothetical protein M91_12772 [B. mutus]
ENSP00000396918-D1	-3.52	6.87E-21	↑	Up-regulator of cell proliferation, partial [B. mutus]
ENSP00000365118-D3	-3.51	1.17E-22	↑	Putative tripartite motif-containing protein 43C, partial [B. mutus]

ENSBTAP00000011067-D1	-3.49	2.41E-09	↑	Transcription factor Ovo-like 2 [B. mutus]
ENSP00000315289-D1	-3.48	4.58E-22	↑	synaptopodin [B. mutus]
ENSBTAP00000015396-D1	-3.48	5.42E-33	↑	glioma pathogenesis-related protein 1 [B. mutus]
ENSBTAP00000007469-D1	-3.48	6.73E-13	↑	N-arachidonyl glycine receptor [Capra hircus]
ENSP00000359504-D4	-3.45	1.33E-12	↑	interferon-induced guanylate-binding protein 2-like, partial [B. mutus]
ENSBTAP00000024371-D1	-3.45	8.01E-11	↑	c-X-C motif chemokine 16 [B. mutus]
ENSP00000377437-D1	-3.45	2.88E-07	↑	ELMO domain-containing protein 3 [B. mutus]
ENSBTAP00000013576-D1	-3.45	1.79E-05	↑	O(6)-methylguanine-induced apoptosis 2 [B. mutus]
ENSBTAP00000049636-D2	-3.45	1.79E-05	↑	myosin light chain 4 [Pantholops hodgsonii]
ENSBTAP00000018269-D1	-3.45	1.79E-05	↑	rho-related GTP-binding protein RhoQ isoform X1 [Condylura cristata]
ENSP00000408119-D3	-3.45	1.79E-05	↑	putative RNA polymerase II subunit A C-terminal domain phosphatase SSU72-like protein 2-like [B. mutus]
ENSP00000380903-D1	-3.45	1.17E-03	↑	roundabout homolog 3 [B. mutus]
ENSP00000376260-D1	-3.45	1.17E-03	↑	Transmembrane channel-like protein 6 [B. mutus]
ENSP00000295213-D1	-3.45	1.17E-03	↑	Spermatogenesis-associated protein 18, partial [B. mutus]
ENSBTAP00000026897-D1	-3.45	1.17E-03	↑	fibroblast growth factor 13 isoform X1 [Orcinus orca]
ENSBTAP00000011575-D1	-3.45	1.17E-03	↑	5-hydroxytryptamine receptor 7 isoform X3 [B. taurus]
ENSP00000368298-D1	-3.45	1.17E-03	↑	Uroplakin-1a [B. mutus]
ENSBTAP00000039405-D1	-3.45	1.17E-03	↑	RE1-silencing transcription factor [B. mutus]
ENSBTAP00000026214-D1	-3.45	1.17E-03	↑	ADP-ribosylation factor-like protein 4C-like protein [Cricetulus griseus]
ENSBTAP00000003831-D1	-3.45	1.17E-03	↑	probable D-tyrosyl-tRNA(Tyr) deacylase 2 [B. taurus]
ENSP00000382770-D1	-3.45	1.17E-03	↑	Potassium channel subfamily U member 1, partial [B. mutus]
ENSP00000300035-D1	-3.42	5.18E-38	↑	PCNA-associated factor, partial [B. mutus]
ENSBTAP00000045378-D2	-3.41	9.91E-69	↑	up-regulated during skeletal muscle growth protein 5 [B. taurus]
yakA19277	-3.40	9.47E-09	↑	Putative uncharacterized protein encoded by NCRNA00116 [B. mutus]
ENSBTAP00000052701-D2	-3.39	5.20E-12	↑	hypothetical protein M91_09636, partial [B. mutus]
ENSP00000399801-D1	-3.37	1.73E-13	↑	beta-crystallin B1 [B. mutus]
ENSBTAP00000050583-D2	-3.36	1.03E-11	↑	endogenous retrovirus group K member 11 Pol protein-like [Bison bison bison]
ENSP00000327487-D1	-3.36	1.87E-08	↑	tRNA-splicing endonuclease subunit Sen54 [B. mutus]
ENSP00000396732-D3	-3.36	3.56E-05	↑	histone-lysine N-methyltransferase PRDM9-like [Bubalus bubalis]
ENSBTAP00000010971-D1	-3.36	3.56E-05	↑	neuron-specific calcium-binding protein hippocalcin isoformX2 [Canis lupus familiaris]
ENSBTAP00000009466-D1	-3.36	3.56E-05	↑	guanylin precursor [B. taurus]
ENSBTAP00000013791-D1	-3.32	1.14E-06	↑	copine-4 isoform X1 [Ovis aries]
ENSBTAP00000018838-D1	-3.32	2.34E-61	↑	Galanin-like peptide, partial [B. mutus]
ENSP00000219599-D2	-3.31	1.11E-40	↑	Mu-crystallin-like protein, partial [B. mutus]
ENSBTAP00000005802-D1	-3.29	0.00E+00	↑	histone H2A.Z-like [Falco cherrug]
ENSP00000263368-D1	-3.27	1.74E-21	↑	Flavin reductase [B. mutus]
ENSP00000418778-D1	-3.27	1.74E-21	↑	Schwannomin-interacting protein 1, partial [B. mutus]
ENSP00000356792-D2	-3.27	0.00E+00	↑	lymphotactin [Capra hircus]
ENSP00000322191-D1	-3.26	8.57E-14	↑	beta-defensin 104A isoform X1 [B. taurus]
ENSBTAP00000031013-D1	-3.26	2.26E-06	↑	mitochondrial enolase superfamily member 1 [B. mutus]
ENSBTAP00000049197-D1	-3.26	2.26E-06	↑	Chemokine-like factor [B. mutus]
ENSP00000263301-D1	-3.26	7.11E-05	↑	mth938 domain-containing protein [B. mutus]
ENSBTAP00000040290-D1	-3.26	7.11E-05	↑	Ras-related protein Rab-17, partial [B. mutus]
ENSBTAP00000030479-D16	-3.26	2.36E-03	↑	Chain A, Crystal Structure Of Up1 Complexed With D(Ttagggttag(6-Mi) G); A Human Telomeric Repeat Containing 6-Methyl-8-(2- Deoxy-Beta-Ribofuranosyl)isoxanthopteridine (6-Mi)
ENSP00000375470-D1	-3.26	2.36E-03	↑	hypothetical protein M91_11983, partial [B. mutus]
ENSBTAP00000034409-D3	-3.26	2.36E-03	↑	hypothetical protein M91_13901, partial [B. mutus]
ENSBTAP00000008662-D1	-3.26	2.36E-03	↑	olfactory receptor 5W2 [B. taurus]
ENSP00000389912-D11	-3.26	2.36E-03	↑	hypothetical protein M91_13311, partial [B. mutus]
ENSBTAP00000019925-D1	-3.26	2.36E-03	↑	yjeF N-terminal domain-containing protein 3 [B. mutus]
ENSBTAP00000013292-D1	-3.26	2.36E-03	↑	Collagen alpha-1(XV) chain, partial [B. mutus]
ENSBTAP00000042356-D1	-3.26	2.65E-241	↑	Mitochondrial inner membrane protease ATP23-like protein, partial [B. mutus]
ENSP00000390984-D1	-3.22	1.55E-10	↑	Nuclear protein 1 [B. mutus]
ENSBTAP00000012917-D2	-3.20	1.67E-21	↑	Interferon-induced protein 44, partial [B. mutus]
ENSBTAP00000022174-D1	-3.19	3.03E-10	↑	phosphotriesterase-related protein [B. taurus]

ENSP00000418259-D1	-3.19	4.44E-06	↑	Zinc finger protein 57-like protein, partial [B. mutus]
ENSBTAP00000026585-D1	-3.19	4.44E-06	↑	Transmembrane 4 L6 family member 18, partial [B. mutus]
ENSP00000380855-D1	-3.19	4.44E-06	↑	programmed cell death 1 ligand 2 [B. mutus]
ENSBTAP00000013285-D1	-3.17	1.56E-26	↑	gasdermin-A [B. mutus]
ENSBTAP00000001560-D1	-3.16	4.17E-14	↑	leucine-rich repeat neuronal protein 1 precursor [B. taurus]
ENSP00000265191-D1	-3.15	5.93E-10	↑	nucleoside diphosphate kinase homolog 5 isoform X1 [Ovis aries]
ENSP00000358823-D1	-3.15	2.80E-07	↑	deoxyribonuclease-1-like 1 [B. mutus]
ENSBTAP00000049878-D1	-3.15	2.80E-07	↑	T-cell-specific surface glycoprotein CD28, partial [B. mutus]
ENSP00000407118-D1	-3.15	1.41E-04	↑	Syntabulin [B. mutus]
ENSBTAP00000000561-D1	-3.15	1.41E-04	↑	heat shock protein beta-2 [B. mutus]
ENSBTAP00000046985-D1	-3.15	1.41E-04	↑	TPA: ankyrin repeat domain 26-like [B. taurus]
ENSBTAP00000005345-D1	-3.15	1.41E-04	↑	adenylate kinase 8 [B. mutus]
ENSP00000368124-D1	-3.15	1.41E-04	↑	Calcium/calmodulin-dependent protein kinase type 1D, partial [B. mutus]
ENSP00000294947-D1	-3.15	1.41E-04	↑	uncharacterized protein C2orf61 homolog isoform X2 [B. taurus]
ENSBTAP00000053602-D2	-3.15	1.41E-04	↑	zinc finger protein 41 homolog [Bison bison bison]
ENSBTAP00000006983-D1	-3.15	1.41E-04	↑	angiopoietin-related protein 5 [B. mutus]
ENSP00000414669-D1	-3.15	1.41E-04	↑	putative uncharacterized protein PNAS-138-like [Ceratotherium simum simum]
ENSBTAP00000008730-D1	-3.15	2.48E-145	↑	lysophosphatidic acid receptor 6 [B. mutus]
ENSBTAP00000005239-D1	-3.15	8.34E-30	↑	fibroblast growth factor 7 precursor [B. taurus]
ENSP00000216727-D1	-3.14	0.00E+00	↑	Polyadenylate-binding protein 2, partial [B. mutus]
ENSBTAP00000014774-D1	-3.13	1.91E-115	↑	heterogeneous nuclear ribonucleoproteins A2/B1-like isoform X2 [B. taurus]
ENSP00000248935-D2	-3.11	3.08E-21	↑	glutathione S-transferase theta-1-like, partial [B. mutus]
ENSP00000215838-D1	-3.11	8.72E-06	↑	transcobalamin-2 [B. mutus]
ENSBTAP00000006996-D1	-3.11	8.72E-06	↑	Nuclear RNA export factor 2, partial [B. mutus]
ENSP00000295190-D1	-3.11	8.72E-06	↑	monocarboxylate transporter 14 [B. mutus]
ENSP00000261861-D1	-3.11	8.72E-06	↑	Coronin-2B, partial [B. mutus]
ENSBTAP00000005354-D1	-3.11	4.44E-57	↑	UPF0364 protein C6orf211 homolog [B. mutus]
yakG033476	-3.11	1.02E-14	↑	Microtubule-associated proteins 1A/1B light chain 3B [B. mutus]
ENSBTAP00000021969-D1	-3.09	3.59E-27	↑	hypothetical protein M91_21008, partial [B. mutus]
ENSP00000307567-D1	-3.09	1.66E-24	↑	Glutaminyl-tRNA synthetase [B. mutus]
ENSP00000282541-D1	-3.08	3.50E-08	↑	glycerol-3-phosphate dehydrogenase 1-like protein-like [B. mutus]
ENSP00000367882-D1	-3.07	2.26E-20	↑	lanC-like protein 3 [Canis lupus familiaris]
ENSP00000361740-D1	-3.07	3.71E-30	↑	WW domain-binding protein 5 [Bubalus bubalis]
ENSP00000308165-D2	-3.06	9.35E-12	↑	platelet glycoprotein 4-like isoform X1 [B. mutus]
ENSBTAP00000004611-D1	-3.06	3.15E-217	↑	TATA box-binding protein-associated factor RNA polymerase I subunit D, partial [B. mutus]
ENSP00000225171-D1	-3.05	3.72E-49	↑	dnaJ homolog subfamily C member 12 isoform X1 [B. mutus]
ENSP00000324882-D1	-3.04	8.50E-39	↑	IQ and ubiquitin-like domain-containing protein [B. mutus]
ENSP00000357057-D1	-3.04	5.53E-21	↑	calsequestrin-1 [B. mutus]
ENSBTAP00000002838-D1	-3.04	2.82E-10	↑	hypothetical protein M91_16539, partial [B. mutus]
ENSBTAP00000016414-D1	-3.04	4.39E-09	↑	hypothetical protein M91_04351, partial [B. mutus]
ENSBTAP00000012983-D1	-3.04	1.71E-05	↑	probable G-protein coupled receptor 171 [B. mutus]
ENSP00000369816-D1	-3.04	1.71E-05	↑	Sex hormone-binding globulin [B. mutus]
ENSBTAP00000002720-D1	-3.04	1.71E-05	↑	frizzled-1-like [Physeter catodon]
ENSBTAP00000050580-D1	-3.04	2.78E-04	↑	hypothetical protein M91_05510, partial [B. mutus]
ENSP00000377582-D23	-3.04	2.78E-04	↑	hypothetical protein M91_00511, partial [B. mutus]
ENSBTAP00000001298-D1	-3.04	2.78E-04	↑	kelch domain-containing protein 8B [B. mutus]
ENSBTAP00000017188-D1	-3.04	2.78E-04	↑	Isobutyryl-CoA dehydrogenase, mitochondrial, partial [B. mutus]
yakA05801	-3.04	2.78E-04	↑	hypothetical protein M91_03567 [B. mutus]
ENSP00000360028-D5	-3.04	4.81E-03	↑	glutathione S-transferase A4 isoform X1 [B. taurus]
ENSBTAP00000003394-D2	-3.04	4.81E-03	↑	SAM domain-containing protein SAMSN-1, partial [B. mutus]
ENSP00000371594-D1	-3.04	4.81E-03	↑	guanine nucleotide-binding protein G(I)/G(S)/G(O) subunit gamma-7 isoform 1 [Canis lupus familiaris]
ENSBTAP00000050773-D76	-3.04	4.81E-03	↑	hypothetical protein M91_00245, partial [B. mutus]
ENSBTAP00000051525-D1	-3.04	4.81E-03	↑	C4b-binding protein beta chain [B. mutus]
ENSP00000264833-D3	-3.04	4.81E-03	↑	Noelin [B. mutus]
ENSBTAP00000011190-D1	-3.04	4.81E-03	↑	hypothetical protein M91_10367, partial [B. mutus]

ENSBTAP00000029925-D9	-3.04	4.81E-03	↑	ankyrin repeat domain-containing protein 26-like isoform X1 [B. taurus]
ENSBTAP00000011474-D1	-3.04	4.81E-03	↑	Sulfotransferase 6B1, partial [B. mutus]
ENSBTAP00000001648-D1	-3.04	4.81E-03	↑	leukocyte cell-derived chemotaxin-2 [B. mutus]
ENSBTAP00000023971-D1	-3.04	4.81E-03	↑	leucine-, glutamate- and lysine-rich protein 1 [B. mutus]
ENSBTAP00000016164-D1	-3.02	2.96E-24	↑	Dickkopf-like protein 1, partial [B. mutus]
ENSP00000416142-D2	-3.01	2.26E-12	↑	alpha-amylase 2B [B. mutus]
ENSP00000372527-D1	-3.00	3.16E-52	↑	Transmembrane 4 L6 family member 1 [B. mutus]
ENSBTAP00000035695-D2	-2.99	1.32E-07	↑	Glutathione S-transferase A2, partial [B. mutus]
ENSBTAP00000023743-D1	-2.98	0.00E+00	↑	Serglycin, partial [B. mutus]
ENSP00000360676-D1	-2.97	2.10E-06	↑	protein KTI12 homolog isoform X1 [B. mutus]
ENSBTAP00000033443-D1	-2.96	2.85E-16	↑	Cytosolic carboxypeptidase 3 [B. mutus]
ENSP00000317872-D1	-2.95	0.00E+00	↑	E3 ubiquitin-protein ligase RBBP6 isoform X1 [B. mutus]
ENSP00000307954-D1	-2.95	8.49E-12	↑	membrane protein FAM174A [Cavia porcellus]
ENSP00000350230-D1	-2.95	8.49E-12	↑	transmembrane protein 170A isoform X1 [Propithecus coquereli]
ENSBTAP00000047422-D1	-2.95	3.34E-05	↑	general transcription factor IIF subunit 2-like [B. mutus]
ENSBTAP00000044395-D1	-2.94	2.56E-07	↑	hypothetical protein M91_03817, partial [B. mutus]
ENSP00000407583-D1	-2.92	2.09E-173	↑	Putative leucine-twenty homeobox, partial [B. mutus]
ENSBTAP00000007339-D1	-2.91	2.03E-12	↑	Helicase SKI2W, partial [B. mutus]
ENSBTAP00000028761-D1	-2.91	3.17E-08	↑	tubulin polyglutamylase complex subunit 2 [B. mutus]
ENSBTAP00000026322-D1	-2.91	4.06E-06	↑	hypothetical protein M91_16323 [B. mutus]
ENSP00000260191-D1	-2.91	4.06E-06	↑	5-hydroxytryptamine receptor 3B, partial [B. mutus]
ENSP00000382267-D6	-2.91	4.06E-06	↑	cytochrome c-like [B. mutus]
ENSBTAP00000002485-D1	-2.91	4.06E-06	↑	Transient receptor putative cation channel subfamily V member 5 [B. mutus]
ENSP00000324617-D1	-2.91	5.50E-04	↑	Heparan-sulfate 6-O-sulfotransferase 2, partial [B. mutus]
ENSBTAP00000051022-D1	-2.91	5.50E-04	↑	Proproteinase E, partial [B. mutus]
ENSBTAP00000028359-D1	-2.91	5.50E-04	↑	radial spoke head 1 homolog isoform X1 [B. taurus]
ENSBTAP00000032783-D1	-2.91	5.50E-04	↑	collagen alpha-1(XVII) chain [B. mutus]
ENSP00000392996-D1	-2.91	5.50E-04	↑	Ribonuclease-like protein 10, partial [B. mutus]
ENSP00000269829-D55	-2.89	8.36E-108	↑	Zinc finger protein 789, partial [B. mutus]
ENSBTAP00000019527-D1	-2.88	3.16E-17	↑	Polyadenylate-binding protein-interacting protein 2B, partial [B. mutus]
ENSBTAP00000012865-D10	-2.88	2.72E-31	↑	melanoma antigen preferentially expressed in tumors-like [Bison bison bison]
ENSP00000304553-D1	-2.87	2.88E-43	↑	M-phase-specific PLK1-interacting protein [B. mutus]
ENSP00000381047-D1	-2.87	4.90E-19	↑	hypothetical protein M91_12155 [B. mutus]
ENSBTAP00000029915-D3	-2.87	6.09E-08	↑	histone-binding protein RBBP4 [Anolis carolinensis]
ENSP00000333534-D1	-2.87	6.51E-05	↑	cytochrome P450 2F3-like [B. mutus]
ENSBTAP00000038104-D1	-2.87	6.51E-05	↑	apomucin-like, partial [Bison bison bison]
ENSBTAP00000047675-D2	-2.87	6.51E-05	↑	Solute carrier family 7 member 13, partial [B. mutus]
ENSP00000366416-D1	-2.85	1.90E-30	↑	endothelin B receptor isoform X2 [Ovis aries musimon]
ENSP00000320560-D92	-2.83	9.54E-07	↑	hypothetical protein M91_01389, partial [B. mutus]
ENSBTAP00000031831-D1	-2.81	3.50E-67	↑	cytochrome c 2 [B. taurus]
ENSP00000229769-D1	-2.81	1.45E-08	↑	Fanconi anemia group E protein, partial [B. mutus]
ENSP00000281631-D1	-2.80	7.90E-56	↑	Poly [ADP-ribose] polymerase 8, partial [B. mutus]
ENSP00000371587-D1	-2.79	8.02E-16	↑	Kv channel-interacting protein 4, partial [B. mutus]
ENSBTAP00000028460-D1	-2.77	3.41E-09	↑	Acidic repeat-containing protein, partial [B. mutus]
ENSP00000263160-D1	-2.77	3.41E-09	↑	vesicular glutamate transporter 2 [B. taurus]
ENSBTAP00000037365-D1	-2.77	2.75E-08	↑	hypothetical protein M91_08744, partial [B. mutus]
ENSP00000261623-D1	-2.77	2.75E-08	↑	Cytochrome b-245 light chain [B. mutus]
ENSBTAP00000049865-D24	-2.77	1.83E-06	↑	spermatid nuclear transition protein 3-like, partial [Bison bison bison]
ENSBTAP00000026111-D1	-2.77	1.51E-05	↑	Pikachurin, partial [B. mutus]
ENSBTAP00000040185-D1	-2.77	1.51E-05	↑	Glutamate [NMDA] receptor subunit epsilon-4, partial [B. mutus]
ENSBTAP00000014031-D1	-2.77	1.26E-04	↑	ADP-ribosylation factor-like protein 9, partial [B. mutus]
ENSBTAP00000003504-D1	-2.77	1.26E-04	↑	Meiotic recombination protein REC8-like protein [B. mutus]
ENSP00000396652-D1	-2.77	1.26E-04	↑	hypothetical protein EGK_14167 [Macaca mulatta]
ENSBTAP00000027857-D1	-2.77	1.26E-04	↑	tyrosine-protein kinase JAK3 [B. mutus]
ENSBTAP00000028221-D1	-2.77	1.07E-03	↑	cysteine-rich secretory protein LCCL domain-containing 2 [B. mutus]

ENSP00000387907-D1	-2.77	9.69E-03	↑	protein ANKUB1 [B. mutus]
ENSP00000297508-D1	-2.77	9.69E-03	↑	MICAL-like protein 2 [B. taurus]
ENSBTAP00000011856-D1	-2.77	9.69E-03	↑	Dual specificity protein phosphatase 15, partial [B. mutus]
ENSP00000274764-D9	-2.77	9.69E-03	↑	histone H2B type 1-like [B. mutus]
ENSBTAP00000017690-D1	-2.77	9.69E-03	↑	Zinc finger and BTB domain-containing protein 7C, partial [B. mutus]
ENSP00000262219-D1	-2.77	9.69E-03	↑	Annexin A13, partial [B. mutus]
ENSP00000335320-D1	-2.77	9.69E-03	↑	B-cell CLL/lymphoma 9-like protein isoform X1 [B. mutus]
ENSBTAP00000050857-D1	-2.77	9.69E-03	↑	Double C2-like domain-containing protein gamma [B. mutus]
ENSP00000402050-D2	-2.77	9.69E-03	↑	hypothetical protein M91_12177, partial [B. mutus]
ENSBTAP00000037794-D1	-2.77	9.69E-03	↑	cytochrome c oxidase subunit 6B1-like [B. mutus]
ENSBTAP00000032393-D2	-2.77	9.69E-03	↑	liver carboxylesterase-like [B. mutus]
ENSP00000414215-D1	-2.77	9.69E-03	↑	Transmembrane channel-like protein 3 [B. mutus]
ENSBTAP00000040053-D1	-2.77	9.69E-03	↑	sorting nexin-18 [B. mutus]
ENSBTAP00000052350-D1	-2.77	9.69E-03	↑	mitochondrial import inner membrane translocase subunit TIM14-like [B. mutus]
ENSBTAP00000023208-D1	-2.76	6.29E-60	↑	prolyl-tRNA synthetase associated domain-containing protein 1 [B. taurus]
ENSP00000382450-D1	-2.75	6.63E-119	↑	zinc finger protein 131 isoform X1 [B. mutus]
ENSBTAP00000019855-D1	-2.75	1.23E-11	↑	polyprenol reductase [B. mutus]
ENSP00000266503-D1	-2.74	9.96E-11	↑	hypothetical protein M91_13238 [B. mutus]
ENSBTAP00000010081-D1	-2.73	5.23E-08	↑	Myosin-If, partial [B. mutus]
ENSP00000416993-D2	-2.71	3.49E-06	↑	Glutathione S-transferase theta-4, partial [B. mutus]
ENSBTAP00000049917-D1	-2.71	3.49E-06	↑	Glypican-6, partial [B. mutus]
ENSP00000270593-D1	-2.70	2.89E-05	↑	testicular acid phosphatase [B. mutus]
ENSP00000332643-D1	-2.70	2.89E-05	↑	necdin [B. taurus]
ENSBTAP00000004389-D1	-2.70	1.77E-34	↑	Transcription elongation factor SPT4, partial [B. mutus]
ENSP00000352540-D1	-2.69	4.43E-11	↑	epithelial membrane protein 2 [B. mutus]
ENSBTAP00000048510-D1	-2.68	9.10E-43	↑	negative elongation factor C/D-like [B. mutus]
ENSBTAP00000030023-D1	-2.67	8.10E-07	↑	mariner transposase [Homo sapiens]
ENSP00000254260-D1	-2.67	8.10E-07	↑	rhophilin-2 [B. mutus]
ENSBTAP00000022366-D1	-2.67	2.43E-04	↑	growth-regulated protein homolog alpha precursor [B. taurus]
ENSP00000297313-D1	-2.67	2.43E-04	↑	Regulator of G-protein signaling 20 [B. mutus]
ENSBTAP00000013665-D1	-2.67	1.44E-20	↑	interleukin-1 alpha [B. mutus]
ENSP00000384428-D1	-2.66	8.36E-11	↑	Regulator of G-protein signaling 7 [B. mutus]
ENSBTAP00000053200-D1	-2.65	6.64E-06	↑	peroxisomal bifunctional enzyme [B. mutus]
ENSBTAP00000047500-D1	-2.65	6.64E-06	↑	Coiled-coil domain-containing protein 160, partial [B. mutus]
ENSBTAP00000027390-D1	-2.65	6.64E-06	↑	beta-defensin 123 precursor [B. taurus]
ENSP00000285518-D1	-2.65	1.21E-122	↑	1-acyl-sn-glycerol-3-phosphate acyltransferase epsilon, partial [B. mutus]
ENSBTAP00000001989-D1	-2.62	1.53E-06	↑	interferon-stimulated 20 kDa exonuclease-like 2 [B. taurus]
ENSBTAP00000003608-D1	-2.62	5.48E-05	↑	zinc finger CCCH domain-containing protein 7B isoform X2 [Bison bison bison]
ENSBTAP00000016301-D1	-2.62	2.07E-03	↑	Amiloride-sensitive sodium channel subunit beta, partial [B. mutus]
ENSBTAP00000026619-D1	-2.62	2.07E-03	↑	DNA replication complex GINS protein PSF1, partial [B. mutus]
ENSP00000278426-D1	-2.62	2.07E-03	↑	large neutral amino acids transporter small subunit 3 [B. taurus]
ENSBTAP00000004726-D1	-2.62	2.07E-03	↑	Putative DBH-like monooxygenase protein 2, partial [B. mutus]
ENSP00000358765-D1	-2.62	2.07E-03	↑	trophoblast glycoprotein [B. taurus]
ENSP00000242210-D2	-2.61	2.11E-16	↑	Cytosolic 5'-nucleotidase 3, partial [B. mutus]
ENSBTAP00000053306-D1	-2.60	1.02E-08	↑	protein phosphatase 1 regulatory subunit 1C isoform X1 [B. mutus]
ENSP00000368959-D2	-2.60	7.23E-59	↑	receptor expression-enhancing protein 5 [B. mutus]
ENSBTAP00000023151-D1	-2.60	3.52E-07	↑	Adenylate kinase isoenzyme 5, partial [B. mutus]
ENSBTAP00000000920-D1	-2.60	3.52E-07	↑	zinc finger and SCAN domain-containing protein 5B-like, partial [Bison bison bison]
ENSBTAP00000036255-D1	-2.59	1.25E-05	↑	synaptonemal complex protein SC65, partial [B. mutus]
ENSBTAP00000007770-D1	-2.59	1.25E-05	↑	Scm-like with four MBT domains protein 2 [B. mutus]
ENSBTAP00000017380-D1	-2.58	4.61E-46	↑	ELL-associated factor 2 [B. taurus]
ENSBTAP00000010911-D1	-2.58	8.18E-08	↑	Endothelial protein C receptor [B. mutus]
ENSP00000350750-D3	-2.57	1.13E-132	↑	60S ribosomal protein L26-like 1 isoform X1 [Pan troglodytes]
ENSBTAP00000051799-D1	-2.57	5.20E-52	↑	uncharacterized protein LOC613444 [B. taurus]
ENSBTAP00000050872-D1	-2.57	1.87E-21	↑	CHD2 protein [B. taurus]

ENSP00000410996-D1	-2.57	1.87E-21	↑	similar to Six transmembrane epithelial antigen of prostate [Homo sapiens]
ENSP00000366549-D1	-2.57	4.02E-17	↑	Lactosylceramide alpha-2,3-sialyltransferase, partial [B. mutus]
ENSP00000365363-D15	-2.57	4.02E-17	↑	TPA: preferentially expressed antigen in melanoma-like [B. taurus]
ENSBTAP00000035880-D5	-2.57	2.88E-06	↑	toll-like receptor 1 [B. mutus]
ENSP00000226432-D1	-2.57	2.88E-06	↑	PGAP2-interacting protein [B. mutus]
ENSP00000376400-D1	-2.57	2.88E-06	↑	protein S100-A13 isoform X1 [B. taurus]
ENSBTAP00000030051-D1	-2.57	4.61E-04	↑	casein kinase I isoform beta-like [B. mutus]
ENSBTAP00000009810-D1	-2.57	4.61E-04	↑	Guanylate cyclase soluble subunit beta-2, partial [B. mutus]
ENSBTAP00000021263-D1	-2.57	4.61E-04	↑	Cell adhesion molecule 4, partial [B. mutus]
ENSBTAP00000049176-D5	-2.57	4.61E-04	↑	hypothetical protein M91_04382, partial [B. mutus]
ENSBTAP00000003296-D1	-2.56	2.16E-96	↑	TATA-box-binding protein, partial [B. mutus]
ENSP00000253247-D1	-2.56	3.16E-94	↑	nucleolar protein 11 [B. mutus]
ENSP00000328030-D3	-2.55	1.32E-24	↑	hypothetical protein M91_02917, partial [B. mutus]
ENSP00000363533-D1	-2.55	1.03E-09	↑	Putative malate dehydrogenase 1B, partial [B. mutus]
ENSBTAP00000007563-D1	-2.54	1.16E-28	↑	protein phosphatase 1K, mitochondrial isoform X1 [B. mutus]
ENSBTAP00000004994-D1	-2.54	1.04E-04	↑	Microfibrillar-associated protein 2, partial [B. mutus]
ENSBTAP00000016033-D1	-2.54	1.04E-04	↑	N-acetyllactosaminide alpha-1,3-galactosyltransferase isoform X4 [B. taurus]
ENSBTAP00000041573-D5	-2.54	1.04E-04	↑	hypothetical protein M91_17490, partial [B. mutus]
ENSBTAP00000004943-D1	-2.54	1.04E-04	↑	Leucine-rich repeat-containing protein 27 [B. mutus]
ENSP00000384420-D1	-2.53	1.39E-16	↑	Factor XIIa inhibitor, partial [B. mutus]
ENSBTAP00000031879-D1	-2.53	7.79E-126	↑	plastin-3-like isoform X1 [Capra hircus]
ENSBTAP00000001851-D1	-2.53	3.54E-08	↑	hemK methyltransferase family member 2 [B. taurus]
ENSBTAP00000029136-D1	-2.53	1.34E-58	↑	R3H and coiled-coil domain-containing protein 1-like isoform X1 [B. mutus]
ENSBTAP00000019112-D1	-2.52	8.26E-09	↑	caspase recruitment domain-containing protein 6 [B. mutus]
ENSBTAP00000002361-D1	-2.52	2.35E-05	↑	Twisted gastrulation protein-like protein 1, partial [B. mutus]
ENSBTAP00000042158-D1	-2.52	3.00E-12	↑	protein TMEPAI [B. taurus]
ENSBTAP00000000146-D1	-2.51	5.39E-06	↑	Macrosialin, partial [B. mutus]
ENSP00000409815-D2	-2.50	0.00E+00	↑	Proteasome subunit alpha type-2, partial [B. mutus]
ENSBTAP00000044349-D1	-2.49	1.30E-12	↑	Leucine-rich repeat-containing protein 58, partial [B. mutus]
ENSBTAP00000018153-D13	-2.49	1.45E-91	↑	60S ribosomal protein L22-like 1 [Ovis aries]
ENSBTAP00000036892-D1	-2.49	3.80E-15	↑	hypothetical protein M91_18898, partial [B. mutus]
ENSBTAP00000012287-D1	-2.48	9.68E-244	↑	NADH dehydrogenase [ubiquinone] 1 alpha subcomplex subunit 5, partial [B. mutus]
ENSP00000379279-D1	-2.47	1.61E-76	↑	dnaJ homolog subfamily C member 24 [B. taurus]
ENSBTAP00000034409-D1	-2.47	1.30E-13	↑	Melanoma antigen preferentially expressed in tumors, partial [B. mutus]
ENSP00000402050-D8	-2.47	1.64E-15	↑	hypothetical protein M91_20981, partial [B. mutus]
ENSP00000340662-D1	-2.46	8.40E-263	↑	HSPC185 [Homo sapiens]
ENSBTAP00000009389-D2	-2.45	2.82E-08	↑	Lithostathine, partial [B. mutus]
ENSBTAP00000011776-D1	-2.45	2.31E-06	↑	serine dehydratase-like [B. mutus]
ENSP00000420211-D1	-2.45	1.00E-05	↑	Ral guanine nucleotide dissociation stimulator-like 2, partial [B. mutus]
ENSBTAP00000043782-D1	-2.45	4.40E-05	↑	CAV1 protein [B. taurus]
ENSBTAP00000028109-D1	-2.45	1.95E-04	↑	tumor necrosis factor receptor superfamily member 6B precursor [B. taurus]
ENSP00000246792-D1	-2.45	1.95E-04	↑	ras-related protein R-Ras [B. taurus]
ENSP00000332613-D1	-2.45	8.70E-04	↑	Out at first protein-like protein, partial [B. mutus]
ENSBTAP00000020428-D2	-2.45	8.70E-04	↑	hypothetical protein M91_16259, partial [B. mutus]
ENSBTAP00000028562-D1	-2.45	3.98E-03	↑	complexin-3 [B. mutus]
ENSP00000373354-D32	-2.45	3.98E-03	↑	mariner transposase [Homo sapiens]
ENSBTAP00000025032-D1	-2.45	3.98E-03	↑	ankyrin repeat and SOCS box protein 13 [B. taurus]
ENSBTAP00000041163-D1	-2.45	3.98E-03	↑	UPF0449 protein C19orf25 homolog [B. taurus]
ENSBTAP00000042806-D12	-2.45	1.92E-02	↑	hypothetical protein M91_15908 [B. mutus]
ENSP00000344967-D1	-2.45	1.92E-02	↑	Protein sprouty-like protein 4, partial [B. mutus]
ENSP00000265154-D1	-2.45	1.92E-02	↑	Rho guanine nucleotide exchange factor 38 [B. mutus]
ENSP00000384604-D1	-2.45	1.92E-02	↑	Protein FAM150B, partial [B. mutus]
ENSP00000219271-D1	-2.45	1.92E-02	↑	Matrix metalloproteinase-14, partial [B. mutus]
ENSBTAP00000047790-D43	-2.45	1.92E-02	↑	endogenous retrovirus group K member 18 Pol protein-like [B. taurus]
ENSBTAP00000049852-D2	-2.45	1.92E-02	↑	interferon-induced very large GTPase 1-like [B. taurus]

ENSP00000230361-D1	-2.45	1.92E-02	↑	guanylyl cyclase-activating protein 2 [B. taurus]
ENSBTAP00000045403-D1	-2.45	1.92E-02	↑	leucine-rich repeat-containing protein 72 [B. mutus]
ENSBTAP00000047817-D40	-2.45	1.92E-02	↑	hypothetical protein M91_11424, partial [B. mutus]
ENSBTAP00000010179-D1	-2.45	1.92E-02	↑	Cytokine receptor-like factor 1, partial [B. mutus]
ENSBTAP00000007931-D1	-2.45	1.92E-02	↑	WNT1-inducible-signaling pathway protein 2 [B. mutus]
ENSP00000289373-D4	-2.45	8.55E-55	↑	thymosin beta-15A [B. mutus]
ENSP00000384411-D1	-2.44	5.88E-279	↑	mitochondrial import receptor subunit TOM5 homolog [Propithecus coquereli]
ENSP00000336687-D4	-2.44	7.44E-106	↑	chromobox protein homolog 3-like [B. taurus]
ENSBTAP00000019573-D1	-2.43	1.62E-31	↑	Ubiquitin-like protein ISG15, partial [B. mutus]
ENSBTAP00000004065-D1	-2.43	3.53E-11	↑	Peroxisomal membrane protein PEX16, partial [B. mutus]
ENSBTAP00000001503-D1	-2.42	1.52E-10	↑	thyroid hormone receptor interactor 6 [B. mutus]
ENSP00000295992-D1	-2.42	1.52E-10	↑	Procollagen C-endopeptidase enhancer 2 [B. mutus]
ENSBTAP00000006653-D1	-2.42	0.00E+00	↑	metalloproteinase inhibitor 1 precursor [B. taurus]
ENSBTAP00000014377-D1	-2.42	1.21E-08	↑	cysteine-rich with EGF-like domain protein 1 precursor [B. taurus]
ENSBTAP00000043140-D1	-2.42	1.21E-08	↑	Transmembrane protein 64, partial [B. mutus]
ENSBTAP00000023551-D1	-2.40	4.25E-06	↑	zinc transporter ZIP3 isoform X1 [B. mutus]
ENSP00000340083-D1	-2.40	5.50E-18	↑	lysine-rich coiled-coil protein 1 isoform X1 [Bison bison bison]
ENSBTAP00000047583-D1	-2.39	1.20E-09	↑	complement component C7 [B. mutus]
ENSP00000325663-D1	-2.39	1.85E-05	↑	NF-kappa-B inhibitor zeta, partial [B. mutus]
ENSP00000386125-D1	-2.39	1.85E-05	↑	armadillo repeat-containing protein 5 isoform X2 [Ovis aries musimon]
ENSBTAP00000003377-D1	-2.39	0.00E+00	↑	peptidyl-prolyl cis-trans isomerase FKBP3 isoform X2 [Ovis aries musimon]
ENSP00000350667-D2	-2.38	0.00E+00	↑	Tropomyosin beta chain [B. mutus]
ENSBTAP00000027888-D1	-2.38	4.07E-101	↑	snRNA-activating protein complex subunit 1 [B. mutus]
ENSBTAP00000015489-D1	-2.38	9.55E-08	↑	SHC-transforming protein 4 [B. mutus]
ENSBTAP00000033875-D1	-2.38	8.18E-05	↑	ephrin-A4 [B. mutus]
ENSBTAP00000013139-D1	-2.37	2.51E-126	↑	phosducin-like protein 3 [B. mutus]
ENSBTAP00000024352-D1	-2.37	5.10E-10	↑	Phosphatidylinositol N-acetylglucosaminyltransferase subunit H [B. mutus]
ENSP00000409797-D1	-2.37	1.78E-36	↑	Leucine-rich repeat-containing protein 67, partial [B. mutus]
ENSBTAP00000027359-D1	-2.37	2.71E-12	↑	apoptosis-associated speck-like protein containing a CARD [B. taurus]
ENSP00000407726-D1	-2.37	4.12E-07	↑	von Willebrand factor A domain-containing protein 5A, partial [B. mutus]
ENSBTAP00000042029-D1	-2.36	1.80E-06	↑	vascular cell adhesion protein 1-like isoform X1 [B. mutus]
ENSP00000386190-D1	-2.36	1.80E-06	↑	hypothetical protein M91_00186, partial [B. mutus]
ENSBTAP00000049395-D1	-2.36	1.80E-06	↑	1-phosphatidylinositol 4,5-bisphosphate phosphodiesterase delta-1-like [B. mutus]
ENSBTAP00000019348-D1	-2.34	7.81E-06	↑	Cas scaffolding protein family member 4, partial [B. mutus]
ENSP00000289316-D1	-2.34	7.81E-06	↑	histone H2B type 1-M-like [Mustela putorius furo]
ENSP00000282030-D1	-2.34	1.75E-07	↑	SET-binding protein, partial [B. mutus]
ENSP00000258381-D1	-2.34	1.75E-07	↑	sp110 nuclear body protein isoform X3 [Bison bison bison]
ENSBTAP00000008068-D2	-2.33	2.10E-12	↑	hypothetical protein M91_21375, partial [B. mutus]
ENSBTAP00000038164-D1	-2.33	2.66E-79	↑	Voltage-dependent calcium channel gamma-like subunit [B. mutus]
ENSBTAP00000013354-D1	-2.32	2.09E-13	↑	apolipoprotein E [B. mutus]
ENSP00000355510-D1	-2.32	3.41E-05	↑	WD repeat-containing protein 64 [B. mutus]
ENSBTAP00000023784-D1	-2.32	1.63E-03	↑	Alpha-2,8-sialyltransferase 8B, partial [B. mutus]
ENSP00000317980-D1	-2.32	1.63E-03	↑	Protein turtle-like protein B, partial [B. mutus]
ENSBTAP00000034537-D1	-2.32	1.63E-03	↑	N-acetyllactosaminide beta-1,6-N-acetylglucosaminyl-transferase, isoform B-like [B. mutus]
ENSBTAP00000053750-D2	-2.32	1.63E-03	↑	neurabin-1 [B. mutus]
ENSBTAP00000000032-D1	-2.32	1.63E-03	↑	Lysozyme-like protein 6, partial [B. mutus]
ENSBTAP00000006857-D1	-2.32	4.56E-107	↑	transmembrane protein 14A isoform X2 [B. taurus]
ENSP00000379978-D1	-2.32	6.20E-41	↑	Beta-2-microglobulin [B. mutus]
ENSBTAP00000049167-D1	-2.32	4.44E-89	↑	Succinate receptor 1, partial [B. mutus]
ENSP00000361383-D1	-2.32	1.69E-09	↑	Allograft inflammatory factor 1-like protein, partial [B. mutus]
ENSP00000326737-D1	-2.31	3.86E-34	↑	tumor necrosis factor receptor superfamily member 12A [Ovis aries]
ENSBTAP00000015449-D1	-2.31	0.00E+00	↑	microtubule-associated proteins 1A/1B light chain 3B [B. taurus]
ENSP00000362264-D1	-2.30	6.93E-35	↑	Apolipoprotein O-like protein, partial [B. mutus]
ENSBTAP00000012447-D1	-2.30	4.51E-21	↑	Serine/threonine/tyrosine-interacting-like protein 1 [B. mutus]
ENSP00000203407-D1	-2.30	1.61E-12	↑	Cytochrome b-c1 complex subunit 1, mitochondrial [B. mutus]

ENSP00000401704-D1	-2.30	1.50E-04	↑	PH and SEC7 domain-containing protein 3, partial [B. mutus]
ENSBTAP00000026999-D1	-2.30	1.50E-04	↑	metalloproteinase inhibitor 4 [B. mutus]
ENSP00000365668-D1	-2.29	3.07E-09	↑	alpha-tubulin N-acetyltransferase 1 [B. taurus]
ENSBTAP00000015248-D2	-2.29	3.07E-09	↑	Myosin regulatory light chain 2, smooth muscle major isoform, partial [B. mutus]
ENSP00000331719-D1	-2.29	1.43E-05	↑	ethanolamine-phosphate cytidylyltransferase [B. mutus]
ENSP00000348849-D4	-2.28	3.94E-38	↑	40S ribosomal protein S26 [Myotis brandtii]
ENSP00000390600-D7	-2.28	1.38E-06	↑	Sodium channel protein type 11 subunit alpha, partial [B. mutus]
ENSBTAP00000016757-D1	-2.28	3.31E-27	↑	norrin [B. mutus]
ENSP00000363260-D1	-2.27	1.32E-08	↑	GPI mannosyltransferase 2 [B. mutus]
ENSBTAP00000006703-D2	-2.27	1.92E-164	↑	regulation of nuclear pre-mRNA domain-containing protein 1A [B. mutus]
ENSBTAP00000025636-D1	-2.26	1.17E-16	↑	alpha-galactosidase A [B. mutus]
ENSBTAP00000041566-D1	-2.26	5.75E-07	↑	RNA-binding protein Nova-1 [B. taurus]
ENSP00000379255-D1	-2.26	6.69E-04	↑	transcriptional activator GLI3 isoform X6 [B. taurus]
ENSP00000231173-D8	-2.26	6.69E-04	↑	Protocadherin gamma-A9, partial [B. mutus]
ENSP00000368450-D1	-2.26	6.69E-04	↑	CD83 antigen isoform X1 [B. mutus]
ENSP00000369962-D1	-2.26	6.69E-04	↑	Immunoglobulin superfamily member 5, partial [B. mutus]
ENSP00000358798-D1	-2.26	7.53E-03	↑	calcium homeostasis modulator protein 3 [B. taurus]
ENSP00000307598-D55	-2.26	7.53E-03	↑	hypothetical protein M91_06797, partial [B. mutus]
ENSP00000320232-D1	-2.26	7.53E-03	↑	hypothetical protein M91_15732 [B. mutus]
ENSBTAP00000016342-D1	-2.26	7.53E-03	↑	Low-density lipoprotein receptor, partial [B. mutus]
ENSP00000351657-D16	-2.26	7.53E-03	↑	Zinc finger protein 256 [B. mutus]
ENSBTAP00000042880-D2	-2.26	7.53E-03	↑	RNA-binding motif protein, X chromosome-like [B. mutus]
ENSP00000350265-D1	-2.26	7.53E-03	↑	ectonucleotide pyrophosphatase/phosphodiesterase family member 3 [B. mutus]
ENSP00000358795-D1	-2.26	7.53E-03	↑	neuralized-like protein 1A [B. mutus]
ENSP00000399221-D2	-2.26	7.53E-03	↑	Rpl13 [B. taurus]
ENSBTAP00000008986-D1	-2.26	7.53E-03	↑	Putative L-aspartate dehydrogenase, partial [B. mutus]
ENSP00000378238-D1	-2.25	1.67E-43	↑	protein EVI2A [B. mutus]
ENSP00000350881-D1	-2.25	2.09E-17	↑	GRAM domain-containing protein 1C isoform X2 [Ovis aries musimon]
ENSP00000382948-D1	-2.25	3.09E-129	↑	GA-binding protein alpha chain [Bubalus bubalis]
ENSP00000338967-D1	-2.24	2.23E-46	↑	Rho GTPase-activating protein 6, partial [B. mutus]
ENSBTAP00000012987-D1	-2.24	2.33E-09	↑	placenta-specific gene 8 protein-like [B. mutus]
ENSBTAP00000034554-D1	-2.24	2.41E-07	↑	TPA: hypothetical protein BOS_18285 [B. taurus]
ENSBTAP00000008621-D1	-2.24	0.00E+00	↑	Proteasome subunit alpha type-1, partial [B. mutus]
ENSP00000338218-D1	-2.24	9.50E-12	↑	E3 ubiquitin-protein ligase RNF213 isoform X1, partial [B. taurus]
ENSP00000365505-D1	-2.24	2.03E-92	↑	proteolipid protein 2 [B. taurus]
ENSP00000415203-D1	-2.23	2.59E-05	↑	GPI ethanolamine phosphate transferase 2 isoform X1 [Bison bison bison]
ENSP00000359454-D1	-2.22	4.07E-11	↑	probable ATP-dependent DNA helicase HFM1-like, partial [B. mutus]
ENSBTAP00000002681-D1	-2.22	4.18E-09	↑	neprilysin [B. taurus]
ENSBTAP00000041125-D1	-2.22	4.18E-09	↑	trefoil factor 2-like [B. mutus]
ENSP00000295304-D1	-2.22	2.74E-04	↑	Cation transport regulator-like protein 2, partial [B. mutus]
ENSBTAP00000000841-D1	-2.21	2.68E-64	↑	Adenine phosphoribosyltransferase [B. mutus]
ENSBTAP00000014852-D1	-2.21	4.24E-08	↑	Tetratricopeptide repeat protein 23, partial [B. mutus]
ENSBTAP00000025607-D1	-2.21	4.24E-08	↑	prostaglandin E2 receptor EP3 subtype isoform X1 [B. mutus]
ENSBTAP00000043977-D1	-2.21	1.21E-15	↑	Bile salt export pump [B. mutus]
ENSBTAP00000012108-D1	-2.21	4.10E-48	↑	Trifunctional purine biosynthetic protein adenosine-3, partial [B. mutus]
ENSP00000324821-D1	-2.19	3.05E-11	↑	Chitinase domain-containing protein 1, partial [B. mutus]
ENSBTAP00000011598-D1	-2.19	1.59E-18	↑	Ubiquitin carboxyl-terminal hydrolase 3, partial [B. mutus]
ENSBTAP00000024333-D1	-2.19	1.81E-07	↑	vesicle-associated membrane protein 5 [B. taurus]
yakG040301	-2.19	1.13E-04	↑	SH3-containing GRB2-like protein 3-interacting protein 1 [B. mutus]
ENSP00000386439-D1	-2.19	1.13E-04	↑	Syncoilin, partial [B. mutus]
ENSBTAP00000001037-D1	-2.19	1.13E-04	↑	protransforming growth factor alpha [Balaenoptera acutorostrata scammoni]
ENSBTAP00000017013-D1	-2.19	3.02E-03	↑	mannan-binding lectin serine protease 2 [B. mutus]
ENSBTAP00000002023-D1	-2.19	3.02E-03	↑	sodium-dependent phosphate transport protein 2B [B. mutus]
ENSBTAP00000050623-D44	-2.19	3.02E-03	↑	hypothetical protein M91_11336, partial [B. mutus]
ENSBTAP00000001770-D1	-2.19	3.02E-03	↑	protein-lysine methyltransferase METTL21E-like [B. mutus]

yakG011015	-2.19	3.02E-03	↑	hypothetical protein M91_07947 [B. mutus]
ENSP00000382941-D1	-2.19	3.02E-03	↑	Tyrosine-protein phosphatase non-receptor type substrate 1, partial [B. mutus]
ENSP00000367486-D1	-2.19	3.02E-03	↑	meiosis expressed gene 1 protein homolog isoform 1 [B. taurus]
ENSBTAP00000004639-D1	-2.19	3.02E-03	↑	hypothetical protein M91_19703, partial [B. mutus]
ENSBTAP00000010263-D3	-2.19	3.02E-03	↑	hypothetical protein M91_20410 [B. mutus]
ENSP00000335307-D1	-2.19	3.02E-03	↑	beta-defensin 15 isoform X2 [B. taurus]
ENSP00000400168-D1	-2.19	3.02E-03	↑	Pentatricopeptide repeat-containing protein 1, partial [B. mutus]
yakG042468	-2.19	3.02E-03	↑	60S ribosomal protein L9-like [B. mutus]
ENSBTAP00000017898-D1	-2.18	7.40E-47	↑	Speckle-type POZ protein-like protein, partial [B. mutus]
ENSBTAP00000052237-D1	-2.18	2.42E-144	↑	Biliverdin reductase A, partial [B. mutus]
ENSP00000409912-D1	-2.18	7.60E-08	↑	hypothetical protein M91_09062, partial [B. mutus]
ENSBTAP00000042083-D1	-2.17	1.87E-06	↑	renin receptor isoform X5 [Ovis aries musimon]
ENSBTAP00000049324-D1	-2.17	1.87E-06	↑	endothelin-1 receptor [Bison bison bison]
ENSBTAP00000036700-D1	-2.17	6.42E-25	↑	SH3 domain-binding glutamic acid-rich protein, partial [B. mutus]
ENSP00000303802-D1	-2.17	2.28E-11	↑	GTP-binding protein 8 isoform X4 [B. taurus]
ENSP00000420140-D1	-2.17	4.67E-05	↑	Protein FAM71F2 [B. mutus]
ENSBTAP00000043655-D1	-2.17	7.88E-76	↑	transcription factor A, mitochondrial-like [B. mutus]
ENSBTAP00000021256-D2	-2.17	0.00E+00	↑	annexin A1 [B. mutus]
ENSBTAP00000005625-D2	-2.16	3.97E-13	↑	Dihydropyrimidinase-related protein 2 [B. mutus]
ENSBTAP00000007115-D1	-2.15	4.16E-198	↑	dual specificity protein kinase CLK1 isoform X1 [B. taurus]
ENSP00000259470-D1	-2.15	6.49E-21	↑	cathepsin L1-like [B. mutus]
ENSP00000379108-D1	-2.15	1.93E-05	↑	Putative bifunctional methylenetetrahydrofolate dehydrogenase/cyclohydrolase 2, partial [B. mutus]
ENSBTAP00000025597-D25	-2.15	1.93E-05	↑	Pepsin A, partial [B. mutus]
ENSBTAP00000044315-D1	-2.15	1.21E-03	↑	leucine-rich repeat-containing protein 3B precursor [B. taurus]
ENSP00000336931-D1	-2.14	7.73E-36	↑	proline-rich nuclear receptor coactivator 1 [B. mutus]
ENSBTAP00000009523-D1	-2.14	2.31E-293	↑	tumor necrosis factor-inducible gene 6 protein precursor [B. taurus]
ENSP00000357076-D4	-2.14	2.10E-57	↑	Transgelin, partial [B. mutus]
ENSBTAP00000040583-D73	-2.14	8.01E-06	↑	hypothetical protein M91_05670, partial [B. mutus]
ENSBTAP00000034581-D19	-2.14	1.95E-27	↑	histone H3.3 [Mytilus galloprovincialis]
ENSP00000282470-D1	-2.13	7.43E-132	↑	SPARC-like protein 1 [B. mutus]
ENSBTAP00000011042-D1	-2.13	8.10E-180	↑	serine protease HTRA1 isoform X2 [Ovis aries musimon]
ENSBTAP00000023928-D1	-2.13	3.33E-06	↑	Zinc finger protein GLIS1, partial [B. mutus]
ENSP00000378804-D1	-2.13	4.97E-04	↑	Spermidine/spermine N(1)-acetyltransferase-like protein 1, partial [B. mutus]
ENSP00000352119-D8	-2.12	1.59E-15	↑	histone H2A type 1-E-like [Leptonychotes weddellii]
ENSP00000216780-D1	-2.12	9.90E-09	↑	phosphoenolpyruvate carboxykinase [GTP], mitochondrial [B. mutus]
ENSBTAP00000021717-D1	-2.12	4.22E-70	↑	UPF0690 protein C1orf52 homolog [B. taurus]
ENSBTAP00000017827-D1	-2.12	2.81E-109	↑	VIP peptides [B. mutus]
ENSBTAP00000007032-D1	-2.12	4.30E-219	↑	GPN-loop GTPase 3, partial [B. mutus]
ENSP00000361465-D1	-2.11	4.94E-17	↑	DNA-directed RNA polymerases I and III subunit RPAC1 [B. mutus]
ENSBTAP00000033686-D1	-2.11	5.75E-07	↑	Chloride anion exchanger, partial [B. mutus]
ENSP00000409223-D1	-2.11	2.03E-04	↑	hypothetical protein M91_19169, partial [B. mutus]
ENSBTAP00000020444-D1	-2.11	2.03E-04	↑	Monocarboxylate transporter 5, partial [B. mutus]
ENSP00000306425-D1	-2.11	3.73E-131	↑	coiled-coil-helix-coiled-coil-helix domain-containing protein 7 isoform X1 [Bison bison bison]
ENSBTAP00000003645-D1	-2.11	2.39E-07	↑	Sterile alpha and TIR motif-containing protein 1, partial [B. mutus]
ENSP00000261721-D1	-2.11	3.03E-10	↑	TPA: BTB (POZ) domain containing 1 [B. taurus]
ENSP00000338711-D2	-2.10	1.27E-10	↑	Tripartite motif-containing protein 5, partial [B. mutus]
ENSP00000381282-D1	-2.10	3.43E-81	↑	selenoprotein S [B. mutus]
ENSBTAP00000036040-D2	-2.09	3.57E-270	↑	H/ACA ribonucleoprotein complex subunit 3 [B. taurus]
ENSBTAP00000052271-D1	-2.09	1.43E-05	↑	transketolase-like protein 2 isoform X1 [B. mutus]
ENSBTAP00000051032-D1	-2.09	1.41E-24	↑	Cullin-2, partial [B. mutus]
ENSBTAP00000044811-D1	-2.08	7.90E-100	↑	DNA-directed RNA polymerase I subunit RPA43 [B. mutus]
ENSP00000233121-D1	-2.08	2.46E-06	↑	Microtubule-associated protein RP/EB family member 3 [Fukomys damarensis]
ENSBTAP00000007448-D1	-2.08	2.46E-06	↑	zinc transporter ZIP8 precursor [B. taurus]
ENSBTAP00000009487-D1	-2.07	1.86E-70	↑	RNA polymerase II elongation factor ELL2, partial [B. mutus]
ENSBTAP00000018919-D1	-2.07	7.36E-08	↑	hypothetical protein M91_16328 [B. mutus]

ENSP00000266718-D1	-2.06	0.00E+00	↑	lumican [B. mutus]
ENSP00000350314-D1	-2.06	5.37E-09	↑	Beta,beta-carotene 9',10'-oxygenase, partial [B. mutus]
ENSBTAP00000051809-D2	-2.05	2.87E-11	↑	60S ribosomal protein L26-like [B. mutus]
ENSBTAP00000042184-D1	-2.05	3.25E-42	↑	latexin [B. mutus]
ENSP00000358183-D2	-2.05	0.00E+00	↑	protein FAM204A [B. mutus]
ENSBTAP00000009821-D1	-2.05	4.70E-15	↑	UDP-GalNAc:beta-1,3-N-acetylgalactosaminyltransferase 1 [B. taurus]
ENSBTAP00000011257-D1	-2.05	2.73E-32	↑	microsomal glutathione S-transferase 1 [B. mutus]
ENSP00000410731-D1	-2.04	5.53E-62	↑	hypothetical protein M91_01439 [B. mutus]
ENSP00000216420-D1	-2.04	2.31E-23	↑	cell growth regulator with RING finger domain protein 1 isoform X1 [Pantholops hodgsonii]
ENSBTAP00000024424-D4	-2.04	9.63E-79	↑	40S ribosomal protein S23, partial [B. mutus]
ENSP00000387264-D1	-2.04	1.07E-33	↑	LIM and senescent cell antigen-like-containing domain protein 1 isoform X4 [B. taurus]
ENSBTAP00000042923-D1	-2.04	1.12E-13	↑	Phosphatidate phosphatase PPAPDC1A, partial [B. mutus]
ENSP00000314407-D1	-2.04	5.03E-11	↑	carbonic anhydrase-related protein [B. taurus]
ENSBTAP00000004304-D1	-2.04	1.64E-09	↑	tubulin polyglutamylase TTLL7 [B. mutus]
ENSBTAP00000007033-D1	-2.04	9.40E-09	↑	Regulatory factor X-associated protein, partial [B. mutus]
ENSBTAP00000040579-D10	-2.04	4.32E-06	↑	Putative protein ZNF720, partial [B. mutus]
yakG007721	-2.04	1.04E-05	↑	Cyclin-dependent kinase inhibitor 3 [B. mutus]
ENSBTAP00000037662-D1	-2.04	1.04E-05	↑	5-hydroxytryptamine receptor 1E, partial [B. mutus]
ENSBTAP00000030308-D1	-2.04	1.04E-05	↑	ras-related protein Rab-33A [Sus scrofa]
ENSBTAP00000017423-D1	-2.04	2.52E-05	↑	hypothetical protein M91_05472, partial [B. mutus]
ENSBTAP00000006985-D1	-2.04	6.10E-05	↑	integral membrane protein GPR180 precursor [B. taurus]
ENSP00000353074-D37	-2.04	6.10E-05	↑	hypothetical protein M91_19007, partial [B. mutus]
ENSP00000326424-D1	-2.04	3.62E-04	↑	glutamine-dependent NAD(+) synthetase [B. mutus]
ENSBTAP00000018774-D1	-2.04	3.62E-04	↑	prostaglandin G/H synthase 2 isoform X1 [B. mutus]
ENSP00000244751-D1	-2.04	8.87E-04	↑	copine-5 [B. taurus]
ENSP00000382178-D1	-2.04	8.87E-04	↑	MAM domain-containing glycosylphosphatidylinositol anchor protein 2 isoform X2 [B. taurus]
ENSBTAP00000042806-D28	-2.04	2.19E-03	↑	hypothetical protein M91_09013, partial [B. mutus]
ENSP00000264705-D1	-2.04	2.19E-03	↑	CAD protein [B. mutus]
ENSBTAP00000053812-D1	-2.04	2.19E-03	↑	Matrix metalloproteinase-21, partial [B. mutus]
ENSBTAP00000039977-D1	-2.04	2.19E-03	↑	hypothetical protein M91_15761, partial [B. mutus]
ENSP00000375647-D1	-2.04	2.19E-03	↑	voltage-dependent calcium channel gamma-7 subunit [Homo sapiens]
ENSP00000301873-D1	-2.04	2.19E-03	↑	Latent-transforming growth factor beta-binding protein 3, partial [B. mutus]
ENSBTAP00000005303-D1	-2.04	5.50E-03	↑	hypothetical protein M91_19816, partial [B. mutus]
ENSP00000379023-D1	-2.04	5.50E-03	↑	TPA: ATP synthase, H+ transporting, mitochondrial F1 complex, epsilon subunit-like [B. taurus]
ENSP00000358001-D1	-2.04	5.50E-03	↑	Transforming acidic coiled-coil-containing protein 2, partial [B. mutus]
ENSP00000366091-D1	-2.04	5.50E-03	↑	myelin-oligodendrocyte glycoprotein isoform X1 [Pantholops hodgsonii]
ENSBTAP00000002345-D1	-2.04	5.50E-03	↑	protein QIL1 isoform X2 [B. taurus]
ENSP00000236709-D1	-2.04	5.50E-03	↑	alpha-1,4-N-acetylglucosaminyltransferase [B. mutus]
ENSP00000367815-D1	-2.04	5.50E-03	↑	Leucine-rich repeat-containing protein 50, partial [B. mutus]
ENSBTAP00000023866-D1	-2.04	1.41E-02	↑	DNA polymerase delta subunit 4 [B. mutus]
ENSP00000376419-D1	-2.04	1.41E-02	↑	kelch-like protein 23 [Bubalus bubalis]
ENSBTAP00000048404-D1	-2.04	1.41E-02	↑	THAP domain-containing protein 10 [B. mutus]
ENSBTAP00000028633-D1	-2.04	1.41E-02	↑	S-arrestin, partial [B. mutus]
ENSP00000376946-D1	-2.04	1.41E-02	↑	Bardet-Biedl syndrome 10 protein, partial [B. mutus]
yakG017852	-2.04	1.41E-02	↑	Histone H4 [Heterocephalus glaber]
ENSBTAP00000043988-D1	-2.04	1.41E-02	↑	hypothetical protein M91_12651 [B. mutus]
ENSBTAP00000004038-D7	-2.04	1.41E-02	↑	hypothetical protein M91_12045, partial [B. mutus]
ENSBTAP00000025829-D8	-2.04	1.41E-02	↑	Sodium channel protein type 7 subunit alpha, partial [B. mutus]
ENSBTAP00000052319-D3	-2.04	3.88E-02	↑	hypothetical protein M91_08009, partial [B. mutus]
ENSBTAP00000014977-D1	-2.04	3.88E-02	↑	Leucine-rich repeat-containing protein 23 [B. mutus]
ENSP00000263717-D1	-2.04	3.88E-02	↑	Spermatogenesis-associated protein 1, partial [B. mutus]
ENSBTAP00000000851-D1	-2.04	3.88E-02	↑	selenoprotein O [B. taurus]
ENSP00000369976-D1	-2.04	3.88E-02	↑	epidermal growth factor-like protein 6 isoform X1 [B. taurus]
ENSP00000408451-D4	-2.04	3.88E-02	↑	60S ribosomal protein L36-like [B. mutus]
ENSBTAP00000046387-D3	-2.04	3.88E-02	↑	hypothetical protein M91_01929, partial [B. mutus]

ENSBTAP00000042963-D1	-2.04	3.88E-02	↑	Tumor necrosis factor ligand superfamily member 12, partial [B. mutus]
ENSBTAP00000049587-D1	-2.04	3.88E-02	↑	lymphocyte antigen 6 complex locus protein G5b [B. mutus]
ENSBTAP00000013977-D1	-2.04	3.88E-02	↑	protein GTLF3B [Physeter catodon]
ENSP00000352565-D1	-2.04	3.88E-02	↑	Heart- and neural crest derivatives-expressed protein 2, partial [B. mutus]
ENSBTAP00000049103-D4	-2.04	3.88E-02	↑	Histone H2B type 1-B, partial [B. mutus]
ENSBTAP00000047717-D1	-2.04	3.88E-02	↑	thiosulfate sulfurtransferase/rhodanese-like domain-containing protein 3 [B. mutus]
ENSBTAP00000014522-D1	-2.04	3.88E-02	↑	chromobox protein homolog 6 isoform 1 [Macaca mulatta]
ENSBTAP00000020075-D5	-2.04	3.88E-02	↑	Chloride intracellular channel protein 3, partial [B. mutus]
ENSP00000231173-D17	-2.04	3.88E-02	↑	Protocadherin gamma-A6, partial [B. mutus]
ENSBTAP00000045962-D1	-2.04	3.88E-02	↑	Testis-specific serine/threonine-protein kinase 6, partial [B. mutus]
ENSBTAP00000025671-D4	-2.04	3.88E-02	↑	Gamma-crystallin F, partial [B. mutus]
ENSBTAP00000011214-D1	-2.04	3.88E-02	↑	sodium-coupled neutral amino acid transporter 3 [B. mutus]
ENSP00000389414-D1	-2.04	3.88E-02	↑	Thromboxane-A synthase [B. mutus]
ENSBTAP00000019639-D1	-2.02	1.75E-19	↑	interferon-stimulated gene 20 kDa protein [B. mutus]
ENSP00000390279-D4	-2.02	1.97E-45	↑	60S ribosomal protein L38, partial [Oryzias melastigma]
ENSBTAP00000019517-D1	-2.01	5.00E-10	↑	Protein SIX6OS1 [B. mutus]
ENSBTAP00000020148-D1	-2.01	0.00E+00	↑	Protein S100-A11, partial [B. mutus]
ENSBTAP00000012944-D2	-2.01	1.72E-32	↑	hypothetical protein M91_13894, partial [B. mutus]
ENSBTAP00000012238-D1	-2.01	4.52E-56	↑	Membrane-spanning 4-domains subfamily A member 8A, partial [B. mutus]
ENSBTAP00000015401-D1	-2.00	2.38E-280	↑	KRR1 small subunit processome component homolog [B. mutus]
ENSP00000404232-D1	-2.00	8.71E-22	↑	EF-hand domain-containing family member C2, partial [B. mutus]
ENSP00000309181-D1	-1.99	2.40E-66	↑	G1/S-specific cyclin-E2 [B. taurus]
ENSP00000362111-D1	-1.99	0.00E+00	↑	tetraspanin-6 [B. taurus]
ENSP00000325919-D1	-1.99	2.37E-123	↑	proteasome assembly chaperone 2 [B. taurus]
ENSBTAP00000025923-D1	-1.99	7.55E-06	↑	DBH-like monooxygenase protein 1 [B. mutus]
ENSBTAP00000017147-D1	-1.98	8.06E-12	↑	Interferon gamma receptor 2, partial [B. mutus]
ENSP00000374782-D82	-1.98	4.41E-05	↑	hypothetical protein M91_06182, partial [B. mutus]
ENSBTAP00000010973-D1	-1.97	1.07E-04	↑	ETS translocation variant 4 isoform X2 [B. taurus]
ENSP00000310260-D2	-1.97	1.07E-04	↑	protein DDX26B-like isoform X1 [B. mutus]
ENSP00000281131-D1	-1.97	7.58E-57	↑	ankyrin repeat domain-containing protein 50 [B. mutus]
ENSBTAP00000023725-D1	-1.97	2.74E-18	↑	junctional adhesion molecule A precursor [B. taurus]
ENSP00000224784-D2	-1.97	2.98E-79	↑	hypothetical protein CB1_000252031 [Camelus ferus]
ENSBTAP00000006351-D1	-1.97	2.56E-20	↑	Kinesin-like protein KIF21A, partial [B. mutus]
ENSP00000370443-D1	-1.97	4.33E-33	↑	A disintegrin and metalloproteinase with thrombospondin motifs 6 precursor [B. taurus]
ENSBTAP00000019481-D1	-1.96	2.61E-04	↑	Visual system homeobox 2, partial [B. mutus]
ENSP00000216840-D1	-1.96	2.61E-04	↑	Geranylgeranyl transferase type-2 subunit alpha, partial [B. mutus]
ENSP00000257264-D1	-1.96	4.84E-56	↑	transcobalamin-1 [B. mutus]
ENSP00000332164-D2	-1.95	4.55E-10	↑	slit homolog 2 protein isoform X3 [B. taurus]
ENSP00000328553-D1	-1.95	5.45E-06	↑	collagen alpha-4(IV) chain [B. mutus]
ENSBTAP00000014753-D1	-1.95	5.45E-06	↑	Acyl-coenzyme A thioesterase 12, partial [B. mutus]
ENSP00000386649-D1	-1.95	5.45E-06	↑	small membrane A-kinase anchor protein [B. taurus]
ENSBTAP00000028426-D1	-1.95	6.39E-04	↑	Transmembrane 4 L6 family member 20, partial [B. mutus]
ENSBTAP00000053247-D1	-1.95	1.11E-35	↑	Methionine-R-sulfoxide reductase B3, mitochondrial, partial [B. mutus]
yakG038480	-1.95	9.35E-14	↑	histone H2A.J-like [B. mutus]
ENSBTAP00000042467-D5	-1.94	6.22E-09	↑	histone H4-like [Alligator sinensis]
ENSBTAP00000018910-D4	-1.94	5.63E-182	↑	60S ribosomal protein L34, partial [B. mutus]
ENSP00000417651-D1	-1.94	3.19E-43	↑	Cytochrome c oxidase copper chaperone [B. mutus]
ENSBTAP00000005097-D95	-1.94	1.48E-08	↑	uncharacterized protein LOC103282962 [Anolis carolinensis]
ENSP00000388599-D1	-1.94	3.17E-05	↑	Prickle-like protein 3 [B. mutus]
ENSBTAP00000014593-D1	-1.94	1.57E-03	↑	Cathepsin H, partial [B. mutus]
ENSBTAP00000016476-D2	-1.93	0.00E+00	↑	ARL14 effector protein [B. taurus]
ENSP00000346032-D2	-1.93	0.00E+00	↑	NMDA receptor-regulated protein 2 [Pteropus alecto]
ENSP00000376315-D3	-1.92	1.48E-82	↑	cysteine-rich protein 1 [B. taurus]
ENSBTAP00000024297-D1	-1.92	5.20E-12	↑	cyclin-dependent kinase inhibitor 1B [B. taurus]
ENSBTAP00000004247-D1	-1.92	9.91E-210	↑	DET1- and DDB1-associated protein 1-like [B. mutus]

ENSP00000354842-D1	-1.92	2.36E-10	↑	Thymocyte selection-associated high mobility group box protein TOX, partial [B. mutus]
ENSBTAP00000014794-D1	-1.92	1.27E-32	↑	cholinesterase [B. mutus]
ENSBTAP00000006382-D1	-1.92	2.20E-18	↑	sodium/potassium/calcium exchanger 5 [B. mutus]
ENSBTAP00000011789-D1	-1.91	1.34E-09	↑	proteasome subunit beta type-9 [B. mutus]
ENSP00000333537-D1	-1.91	1.87E-04	↑	sal-like protein 2 [B. mutus]
ENSBTAP00000048682-D1	-1.91	3.89E-03	↑	interleukin-12 receptor subunit beta-2 isoform X1 [B. mutus]
ENSP00000324343-D1	-1.91	3.89E-03	↑	beta-ureidopropionase [B. mutus]
ENSP00000302077-D1	-1.91	3.89E-03	↑	Schlafen family member 12, partial [B. mutus]
ENSP00000361531-D1	-1.91	3.89E-03	↑	hypothetical protein M91_07524, partial [B. mutus]
ENSBTAP00000007187-D1	-1.91	3.89E-03	↑	adenylate cyclase type 6 [B. mutus]
ENSBTAP00000004411-D3	-1.91	3.89E-03	↑	protein-arginine deiminase type-1 [B. mutus]
ENSP00000218432-D1	-1.91	1.39E-61	↑	Peptidyl-prolyl cis-trans isomerase NIMA-interacting 4, partial [B. mutus]
ENSBTAP00000043674-D2	-1.90	1.69E-10	↑	glutathione S-transferase omega-1-like isoform X1 [B. mutus]
ENSBTAP00000031886-D2	-1.90	4.00E-16	↑	Putative protein ENSP00000244321-like protein [B. mutus]
ENSBTAP00000007083-D1	-1.90	7.64E-09	↑	Ribonuclease P/MRP protein subunit POP5 [B. mutus]
ENSBTAP00000005975-D1	-1.89	0.00E+00	↑	oxidized low-density lipoprotein receptor 1 isoform X1 [B. mutus]
ENSBTAP00000050891-D1	-1.89	2.80E-06	↑	WD repeat-containing protein 38, partial [B. mutus]
ENSP00000334100-D1	-1.89	2.80E-06	↑	exocyst complex component 7 isoformX2 [Equus caballus]
ENSP00000385862-D1	-1.89	4.55E-04	↑	hypothetical protein M91_10965, partial [B. mutus]
ENSP00000320509-D2	-1.89	4.55E-04	↑	B-cell scaffold protein with ankyrin repeats, partial [B. mutus]
ENSBTAP00000004044-D1	-1.89	0.00E+00	↑	integral membrane protein 2B-like [B. mutus]
ENSP00000306344-D1	-1.89	2.66E-23	↑	transmembrane protein 135, partial [Bison bison bison]
ENSBTAP00000020801-D1	-1.89	2.29E-09	↑	interphotoreceptor matrix proteoglycan 2 [B. mutus]
ENSP00000396960-D1	-1.89	0.00E+00	↑	26S proteasome complex subunit DSS1-like [B. mutus]
ENSBTAP00000017066-D1	-1.88	5.54E-132	↑	Stromal cell-derived factor 2, partial [B. mutus]
ENSP00000335384-D1	-1.88	5.79E-13	↑	Zona pellucida-binding protein 2, partial [B. mutus]
ENSBTAP00000047693-D1	-1.88	6.72E-06	↑	RAD51-associated protein 2 [B. mutus]
ENSBTAP00000028955-D1	-1.88	6.72E-06	↑	corneodesmosin [Bison bison bison]
ENSBTAP00000035133-D1	-1.87	2.02E-64	↑	eukaryotic peptide chain release factor subunit 1 [Anolis carolinensis]
ENSBTAP00000023885-D5	-1.87	6.56E-69	↑	hypothetical protein M91_21298, partial [B. mutus]
ENSBTAP00000033017-D1	-1.87	1.87E-132	↑	Mitochondrial ribonuclease P protein 1 [B. mutus]
ENSBTAP00000012599-D1	-1.87	0.00E+00	↑	Chain A, Nmr Structure And Regulated Expression In Apl Cell Of Human Sh3bgrl3
ENSBTAP00000026470-D1	-1.87	4.16E-75	↑	COMM domain-containing protein 1, partial [B. mutus]
ENSBTAP00000003010-D4	-1.87	2.04E-171	↑	U6 snRNA-associated Sm-like protein LSm5 isoform X1 [Cavia porcellus]
ENSBTAP00000044597-D1	-1.87	3.39E-21	↑	hypothetical protein M91_01312 [B. mutus]
ENSBTAP00000036380-D1	-1.87	2.05E-50	↑	Calmodulin-like protein 4, partial [B. mutus]
ENSBTAP00000010703-D1	-1.87	2.47E-07	↑	interferon-induced helicase C domain-containing protein 1 [B. mutus]
ENSP00000349687-D1	-1.87	2.00E-06	↑	ganglioside GM2 activator [B. mutus]
ENSP00000378871-D1	-1.87	1.11E-03	↑	KN motif and ankyrin repeat domain-containing protein 4 [B. mutus]
ENSP00000352288-D1	-1.87	9.79E-03	↑	plexin-B2 precursor [B. taurus]
ENSBTAP00000052774-D5	-1.87	9.79E-03	↑	hCG1645834 [Homo sapiens]
ENSP00000391093-D1	-1.87	9.79E-03	↑	Neurobeachin, partial [B. mutus]
ENSP00000397659-D1	-1.87	9.79E-03	↑	hypothetical protein M91_18895, partial [B. mutus]
ENSBTAP00000028224-D1	-1.87	9.79E-03	↑	A disintegrin and metalloproteinase with thrombospondin motifs 14, partial [B. mutus]
ENSBTAP00000017302-D1	-1.87	9.79E-03	↑	probable G-protein coupled receptor 61 [B. mutus]
yakA00220	-1.87	9.79E-03	↑	NADH dehydrogenase [ubiquinone] 1 alpha subcomplex subunit 1-like [B. mutus]
ENSP00000417202-D2	-1.86	1.30E-297	↑	programmed cell death protein 10 [Mus musculus]
ENSBTAP00000010982-D1	-1.86	7.80E-171	↑	copper transport protein ATOX1 [Ovis aries]
ENSBTAP00000025591-D13	-1.85	5.55E-12	↑	putative RNA polymerase II subunit A C-terminal domain phosphatase SSU72-like protein 1-like [B. mutus]
ENSP00000329748-D1	-1.85	5.91E-07	↑	copine-8 [B. mutus]
ENSBTAP00000053170-D1	-1.85	4.77E-06	↑	Pleckstrin-like protein domain-containing family A member 4 [B. mutus]
ENSBTAP00000015829-D1	-1.85	0.00E+00	↑	CD63 antigen, partial [B. mutus]
ENSP00000295666-D1	-1.85	1.49E-95	↑	Insulin-like growth factor-binding protein 7, partial [B. mutus]
ENSBTAP00000021579-D1	-1.85	2.21E-08	↑	RNA-binding protein 43, partial [B. mutus]
ENSBTAP00000005069-D1	-1.84	3.21E-04	↑	seminal plasma protein A3-like [B. mutus]

ENSP00000384718-D9	-1.84	0.00E+00	↑	40S ribosomal protein S25, partial [Charadrius vociferus]
ENSBTAP00000005558-D3	-1.84	1.19E-12	↑	Acyl-protein thioesterase 1, partial [B. mutus]
ENSP00000317684-D1	-1.83	4.09E-23	↑	sperm acrosomal protein FSA-ACR.1 isoform X1 [B. mutus]
ENSBTAP00000026242-D1	-1.83	1.53E-16	↑	Nicotinamide riboside kinase 1, partial [B. mutus]
ENSBTAP00000030490-D1	-1.83	6.64E-20	↑	DNA-directed RNA polymerases I, II, and III subunit RPABC4 [Fukomys damarensis]
ENSP00000367715-D1	-1.83	1.78E-10	↑	Doublecortin domain-containing protein 2, partial [B. mutus]
ENSBTAP00000023851-D1	-1.83	9.36E-05	↑	DOMON domain-containing protein FRRS1L [Capra hircus]
ENSBTAP00000049943-D1	-1.83	2.74E-03	↑	UPF0452 protein C7orf41 homolog [Ceratotherium simum simum]
ENSP00000368312-D1	-1.83	2.74E-03	↑	ETS translocation variant 2 [B. mutus]
ENSBTAP00000023918-D2	-1.83	2.74E-03	↑	Histone H2B type F-M, partial [B. mutus]
ENSBTAP00000041296-D5	-1.82	2.26E-14	↑	membrane cofactor protein isoform X1 [B. taurus]
ENSBTAP00000047871-D2	-1.82	5.70E-74	↑	28S ribosomal protein S17, mitochondrial isoform X1 [B. mutus]
ENSP00000229332-D1	-1.82	2.75E-05	↑	C-type lectin domain family 4 member A [B. mutus]
ENSBTAP00000029107-D1	-1.82	2.75E-05	↑	Transcription elongation factor A protein 2, partial [B. mutus]
ENSBTAP00000047776-D1	-1.82	2.75E-05	↑	granulins [B. mutus]
ENSBTAP00000050903-D1	-1.81	6.15E-34	↑	rho-related GTP-binding protein RhoE [Rhinopithecus roxellana]
ENSP00000344609-D1	-1.81	5.54E-35	↑	Protein BTG3 [B. mutus]
ENSP00000371376-D1	-1.81	8.11E-06	↑	toll-like receptor 6 [B. mutus]
ENSP00000298295-D1	-1.81	7.80E-04	↑	protein DEPP [B. mutus]
ENSBTAP00000040327-D1	-1.81	7.80E-04	↑	Platelet endothelial cell adhesion molecule, partial [B. mutus]
ENSBTAP00000020705-D1	-1.81	5.07E-21	↑	polycomb complex protein BMI-1 [B. taurus]
ENSBTAP00000051780-D1	-1.81	2.65E-08	↑	cytochrome c [B. taurus]
ENSBTAP00000040566-D1	-1.81	4.00E-250	↑	coiled-coil domain-containing protein 23 [B. taurus]
ENSBTAP00000041843-D1	-1.81	2.09E-56	↑	hydroxysteroid dehydrogenase-like protein 2 [B. mutus]
ENSP00000389928-D6	-1.81	7.95E-09	↑	tripartite motif-containing protein 43-like [B. mutus]
ENSP00000385636-D1	-1.80	2.27E-04	↑	obscurin-like protein 1, partial [B. mutus]
ENSBTAP00000053424-D1	-1.80	2.27E-04	↑	inactive heparanase-2 [Bubalus bubalis]
ENSBTAP00000024658-D1	-1.80	2.27E-47	↑	tripartite motif-containing protein 38 [B. mutus]
ENSBTAP00000001452-D1	-1.80	1.05E-185	↑	peptidyl-prolyl cis-trans isomerase FKBP7 isoform X1 [B. mutus]
ENSBTAP00000010676-D1	-1.80	0.00E+00	↑	ribosome production factor 1 [B. taurus]
ENSBTAP00000000086-D1	-1.80	1.13E-96	↑	Golgi-associated plant pathogenesis-related protein 1, partial [B. mutus]
ENSBTAP00000020872-D2	-1.80	1.88E-08	↑	cysteine-rich PDZ-binding protein [Homo sapiens]
ENSP00000399808-D1	-1.79	1.93E-05	↑	homeobox protein Hox-C4 [Mus musculus]
ENSP00000239891-D1	-1.79	2.32E-60	↑	dolichyl-phosphate beta-glucosyltransferase isoform 1 [B. taurus]
ENSBTAP00000021501-D1	-1.79	5.04E-10	↑	Disabled-like protein 2 [B. mutus]
ENSP00000381970-D2	-1.79	5.03E-07	↑	gypsy retrotransposon integrase-like protein 1 isoform X1 [B. mutus]
ENSBTAP00000005845-D1	-1.79	9.90E-18	↑	transmembrane protein 45A-like [B. mutus]
ENSP00000391457-D1	-1.78	1.16E-27	↑	INO80 complex subunit C [B. mutus]
ENSBTAP00000012182-D2	-1.78	9.53E-12	↑	serine/threonine-protein phosphatase 2A catalytic subunit beta isoform [Heterocephalus glaber]
ENSBTAP00000041860-D1	-1.78	0.00E+00	↑	thioredoxin [B. taurus]
ENSBTAP00000005747-D1	-1.78	7.63E-14	↑	Protein ETHE1, mitochondrial, partial [B. mutus]
ENSP00000363162-D1	-1.78	1.44E-150	↑	V-type proton ATPase subunit G 1 [B. taurus]
ENSP00000367284-D2	-1.78	2.48E-69	↑	FUN14 domain-containing protein 1 [Ovis aries]
ENSP00000415243-D1	-1.78	3.59E-21	↑	ski-like protein isoform X1 [B. mutus]
ENSBTAP00000052063-D1	-1.78	2.66E-24	↑	Biotin--protein ligase, partial [B. mutus]
ENSBTAP00000018760-D1	-1.77	1.61E-14	↑	importin-8 [B. taurus]
ENSP00000262059-D1	-1.77	2.26E-11	↑	calcium-binding and coiled-coil domain-containing protein 1 isoform X1 [B. mutus]
yakA21441	-1.77	1.20E-06	↑	Serine incorporator 4 [B. mutus]
ENSBTAP00000039681-D2	-1.77	1.20E-06	↑	tetratricopeptide repeat protein 30B [B. mutus]
ENSP00000358320-D1	-1.77	4.05E-06	↑	DNA-directed RNA polymerase III subunit RPC7-like [B. taurus]
ENSP00000367356-D1	-1.77	1.59E-04	↑	Protein FAM171A1, partial [B. mutus]
ENSBTAP00000005969-D1	-1.77	1.59E-04	↑	Protein FAM78A, partial [B. mutus]
ENSBTAP00000025681-D1	-1.77	1.59E-04	↑	F-box/LRR-repeat protein 17, partial [B. mutus]
ENSBTAP00000053659-D1	-1.77	5.49E-04	↑	protein cornichon homolog 3 [B. taurus]
ENSBTAP00000030588-D1	-1.77	1.91E-03	↑	death domain-containing membrane protein NRADD-like [B. mutus]

ENSBTAP00000052592-D18	-1.77	1.91E-03	↑	hypothetical protein M91_12176, partial [B. mutus]
yakG029257	-1.77	6.78E-03	↑	hypothetical protein M91_15713 [B. mutus]
ENSBTAP00000004546-D2	-1.77	6.78E-03	↑	Androgen-induced 1 protein, partial [B. mutus]
ENSBTAP00000015001-D1	-1.77	6.78E-03	↑	leucine-zipper-like transcriptional regulator 1 [Equus przewalskii]
ENSBTAP00000048593-D12	-1.77	6.78E-03	↑	endogenous retrovirus group K member 7 Gag polyprotein-like [B. taurus]
ENSBTAP00000018203-D1	-1.77	6.78E-03	↑	tetratricopeptide repeat protein 25 [B. mutus]
ENSBTAP00000009510-D1	-1.77	6.78E-03	↑	spermatogenesis-associated protein 9 [B. mutus]
yakA24149	-1.77	2.58E-02	↑	Collagen alpha-1(XXV) chain [B. mutus]
ENSP00000386167-D15	-1.77	2.58E-02	↑	hypothetical protein M91_02341 [B. mutus]
ENSBTAP00000048897-D1	-1.77	2.58E-02	↑	Sodium/hydrogen exchanger 5, partial [B. mutus]
ENSBTAP00000002699-D1	-1.77	2.58E-02	↑	protein FAM217A [B. mutus]
ENSBTAP00000041536-D57	-1.77	2.58E-02	↑	olfactory receptor 6C1-like [B. taurus]
ENSP00000301327-D1	-1.77	2.58E-02	↑	major facilitator superfamily domain-containing protein 3 [B. mutus]
ENSBTAP00000006372-D1	-1.77	2.58E-02	↑	Olfactomedin-like protein 2A, partial [B. mutus]
ENSP00000264651-D2	-1.77	2.58E-02	↑	keratin, type I cytoskeletal 24 [B. mutus]
ENSP00000278385-D1	-1.77	2.63E-57	↑	CD44 antigen [B. mutus]
ENSP00000386557-D1	-1.77	1.14E-14	↑	hypothetical protein M91_07134, partial [B. mutus]
ENSP00000233143-D4	-1.76	0.00E+00	↑	thymosin beta-10 [B. taurus]
ENSBTAP00000011613-D1	-1.76	9.02E-14	↑	selenide, water dikinase 1-like isoform X1 [B. mutus]
ENSP00000345229-D1	-1.76	2.50E-07	↑	Delta and Notch-like epidermal growth factor-related receptor, partial [B. mutus]
ENSBTAP00000052226-D1	-1.76	0.00E+00	↑	Protein DBF4 like protein B [Pteropus alecto]
ENSBTAP00000020870-D1	-1.76	1.13E-11	↑	acrosin inhibitor 1 [B. taurus]
ENSBTAP00000003618-D1	-1.75	2.85E-06	↑	leucine-rich alpha-2-glycoprotein [B. mutus]
ENSBTAP00000007891-D1	-1.75	2.85E-06	↑	Gamma-2-syntrophin, partial [B. mutus]
ENSBTAP00000023987-D2	-1.75	9.62E-06	↑	B-cell lymphoma/leukemia 11A [B. mutus]
ENSBTAP00000003238-D1	-1.75	0.00E+00	↑	Synaptonemal complex protein 3, partial [B. mutus]
ENSP00000263334-D1	-1.75	1.76E-07	↑	TPA: paired box 8 [B. taurus]
ENSP00000281156-D2	-1.75	1.76E-07	↑	KH domain-containing, RNA-binding, signal transduction-associated protein 2, partial [B. mutus]
ENSP00000244799-D1	-1.75	1.12E-04	↑	opsin-5 [B. mutus]
ENSBTAP00000003529-D1	-1.74	8.27E-26	↑	HORMA domain-containing protein 1 isoform X1 [B. mutus]
ENSP00000368920-D1	-1.74	3.32E-09	↑	NHL repeat-containing protein 3 [B. mutus]
ENSBTAP00000024562-D1	-1.74	4.33E-50	↑	ufm1-specific protease 2 [B. mutus]
ENSBTAP00000018677-D1	-1.74	2.01E-06	↑	hypothetical protein M91_07407 [B. mutus]
ENSBTAP00000010726-D1	-1.74	3.84E-04	↑	Protein FAM164B, partial [B. mutus]
ENSBTAP00000043674-D1	-1.74	1.62E-38	↑	glutathione S-transferase omega-1-like isoform X1 [B. mutus]
ENSBTAP00000046522-D2	-1.73	3.96E-49	↑	Serpin B8 [B. mutus]
ENSP00000240050-D1	-1.73	1.48E-10	↑	mTERF domain-containing protein 3, mitochondrial isoform X1 [B. mutus]
ENSP00000355082-D1	-1.73	1.32E-03	↑	Protocadherin-18, partial [B. mutus]
ENSBTAP00000025264-D1	-1.73	1.41E-06	↑	transmembrane protein 236 [B. mutus]
ENSBTAP00000008920-D1	-1.72	5.05E-56	↑	coiled-coil domain-containing protein 174 [B. mutus]
ENSBTAP00000015208-D1	-1.72	1.18E-31	↑	histidine triad nucleotide-binding protein 2, mitochondrial [B. mutus]
ENSP00000398079-D1	-1.72	1.24E-13	↑	putative RNA polymerase II subunit A C-terminal domain phosphatase SSU72-like protein 1-like [B. mutus]
ENSP00000377355-D3	-1.72	7.82E-05	↑	PDZ domain-containing RING finger protein 4, partial [B. mutus]
ENSP00000360916-D1	-1.72	7.82E-05	↑	Guanine nucleotide exchange factor VAV2, partial [B. mutus]
ENSP00000341538-D1	-1.72	0.00E+00	↑	protein transport protein Sec61 subunit gamma-like [Bubalus bubalis]
ENSP00000401856-D1	-1.72	5.09E-134	↑	Coiled-coil domain-containing protein 80, partial [B. mutus]
ENSP00000366636-D98	-1.72	0.00E+00	↑	hypothetical protein M91_13120, partial [B. mutus]
ENSBTAP00000009568-D1	-1.72	1.16E-09	↑	triggering receptor expressed on myeloid cells 2 [B. mutus]
ENSP00000357440-D1	-1.72	9.31E-26	↑	heat shock factor protein 2 [B. taurus]
ENSBTAP00000051820-D2	-1.71	1.61E-05	↑	serum amyloid A protein [B. taurus]
ENSP00000380959-D1	-1.71	2.68E-04	↑	hypothetical protein M91_11123, partial [B. mutus]
ENSP00000407569-D1	-1.71	2.68E-04	↑	transmembrane protein 80, partial [Lipotes vexillifer]
ENSBTAP00000050194-D98	-1.71	4.71E-03	↑	hypothetical protein M91_04059 [B. mutus]
ENSBTAP00000002231-D1	-1.71	4.71E-03	↑	transmembrane protein 107 [B. taurus]
ENSP00000271331-D1	-1.71	4.71E-03	↑	prokineticin-1 [B. mutus]

ENSP00000354091-D1	-1.71	7.17E-33	↑	guanine nucleotide-binding protein-like 3-like protein isoform X1 [B. mutus]
ENSP00000312141-D1	-1.71	1.72E-10	↑	Zinc finger protein 654, partial [B. mutus]
ENSBTAP00000025963-D5	-1.71	6.13E-212	↑	60S ribosomal protein L10a, partial [B. mutus]
ENSP00000397123-D1	-1.71	2.73E-09	↑	Bifunctional 3'-phosphoadenosine 5'-phosphosulfate synthase 2, partial [B. mutus]
ENSBTAP00000001208-D1	-1.70	4.47E-81	↑	cytochrome b-c1 complex subunit 9 [Ovis aries]
ENSBTAP00000043335-D1	-1.70	6.19E-31	↑	density-regulated protein isoform X1 [Ovis aries]
ENSBTAP00000004701-D1	-1.70	0.00E+00	↑	protein PBDC1 isoform X1 [B. taurus]
ENSBTAP00000003059-D1	-1.70	1.46E-22	↑	host cell factor C1 regulator 1 isoform X2 [B. taurus]
ENSBTAP00000029280-D1	-1.70	1.13E-05	↑	sepiapterin reductase isoform X1 [B. mutus]
ENSBTAP00000001107-D1	-1.70	9.23E-04	↑	Breast carcinoma-amplified sequence 1-like protein [B. mutus]
ENSP00000287957-D1	-1.70	9.23E-04	↑	GATA zinc finger domain-containing protein 1, partial [B. mutus]
ENSBTAP00000053765-D1	-1.70	9.23E-04	↑	Tyrosine-protein kinase transmembrane receptor ROR2, partial [B. mutus]
ENSBTAP00000025389-D4	-1.70	1.16E-77	↑	epithelial membrane protein 3 [B. taurus]
ENSBTAP00000023485-D1	-1.70	3.04E-08	↑	Palmdelphin, partial [B. mutus]
ENSP00000392852-D1	-1.69	4.00E-19	↑	hypothetical protein M91_16290 [B. mutus]
ENSBTAP00000002200-D1	-1.69	1.26E-65	↑	stanniocalcin-1 precursor [B. taurus]
ENSBTAP00000035589-D1	-1.69	1.87E-04	↑	Putative phospholipid-transporting ATPase IB, partial [B. mutus]
ENSBTAP00000052922-D1	-1.69	1.87E-04	↑	Kelch repeat and BTB domain-containing protein 5, partial [B. mutus]
ENSBTAP00000053178-D1	-1.69	1.78E-20	↑	F-box only protein 11, partial [B. mutus]
ENSP00000317985-D1	-1.69	2.85E-76	↑	Rho-associated protein kinase 2, partial [B. mutus]
ENSBTAP00000024392-D1	-1.69	1.02E-07	↑	Cell cycle checkpoint control protein RAD9B [B. mutus]
ENSP00000350961-D1	-1.69	1.56E-15	↑	hypothetical protein M91_02012 [B. mutus]
ENSBTAP00000017525-D1	-1.69	0.00E+00	↑	replication protein A 14 kDa subunit [B. mutus]
ENSBTAP00000048590-D1	-1.69	4.57E-79	↑	Transcription elongation factor B polypeptide 2, partial [B. mutus]
ENSP00000276062-D1	-1.68	0.00E+00	↑	NADH dehydrogenase [ubiquinone] 1 beta subcomplex subunit 11, mitochondrial isoform X1 [B. mutus]
ENSP00000410083-D1	-1.68	2.50E-26	↑	vezatin-like, partial [B. mutus]
ENSBTAP00000010856-D1	-1.68	6.86E-20	↑	Small nuclear ribonucleoprotein-associated protein N, partial [B. mutus]
ENSBTAP00000048387-D1	-1.67	5.00E-08	↑	Glycosyltransferase 8 domain-containing protein 2, partial [B. mutus]
ENSP00000368648-D1	-1.67	5.52E-06	↑	Zinc transporter 6, partial [B. mutus]
ENSBTAP00000007071-D1	-1.67	6.43E-04	↑	Calcineurin-like phosphoesterase domain-containing protein 1 [B. mutus]
ENSP00000332287-D1	-1.67	1.80E-15	↑	Synaptogyrin-1, partial [B. mutus]
ENSP00000414649-D1	-1.67	3.25E-03	↑	Taste receptor type 2 member 39, partial [B. mutus]
ENSBTAP00000008159-D1	-1.67	1.72E-02	↑	Lysyl oxidase-like protein 2 [B. mutus]
ENSP00000379326-D1	-1.67	1.72E-02	↑	sodium-coupled monocarboxylate transporter 2 [B. mutus]
ENSBTAP00000011718-D1	-1.67	1.72E-02	↑	Chymotrypsin-like elastase family member 1 [B. mutus]
ENSP00000317579-D1	-1.67	1.72E-02	↑	UPF0420 protein C16orf58 homolog, partial [B. mutus]
ENSP00000369121-D1	-1.67	1.72E-02	↑	tectonin beta-propeller repeat-containing protein 1 [B. mutus]
ENSP00000320869-D1	-1.67	1.72E-02	↑	E3 ubiquitin-protein ligase TRIM41 [Bubalus bubalis]
ENSBTAP00000010176-D2	-1.67	1.29E-84	↑	Ubiquitin-60S ribosomal protein L40, partial [B. mutus]
yakG001435	-1.67	1.91E-141	↑	Testicular haploid expressed protein [B. mutus]
ENSBTAP00000026851-D2	-1.67	2.40E-108	↑	E3 ubiquitin-protein ligase RNF138 isoform X2 [B. mutus]
ENSP00000419057-D1	-1.67	7.39E-09	↑	hypothetical protein M91_08248, partial [B. mutus]
ENSBTAP00000005204-D1	-1.66	8.03E-07	↑	CXXC-type zinc finger protein 5 isoform X1 [Bubalus bubalis]
ENSBTAP00000030740-D1	-1.66	5.54E-277	↑	Synaptonemal complex protein 1 [B. mutus]
ENSBTAP00000024072-D1	-1.66	3.86E-06	↑	nicotinate-nucleotide pyrophosphorylase [carboxylating] [Bison bison bison]
ENSBTAP00000012523-D2	-1.66	0.00E+00	↑	acyl-CoA-binding protein [B. taurus]
ENSBTAP00000001891-D1	-1.66	1.86E-05	↑	Solute carrier family 15 member 2, partial [B. mutus]
ENSBTAP00000004093-D1	-1.66	0.00E+00	↑	Interferon alpha-inducible protein 27-like protein 1 [B. mutus]
ENSP00000056233-D1	-1.66	2.22E-13	↑	Nuclear factor erythroid 2-related factor 3, partial [B. mutus]
ENSP00000378554-D1	-1.66	1.17E-07	↑	armadillo repeat-containing X-linked protein 3 [B. mutus]
ENSBTAP00000019617-D1	-1.65	4.46E-04	↑	Leucine-rich repeat and WD repeat-containing protein 1, partial [B. mutus]
ENSBTAP00000004724-D51	-1.65	4.46E-04	↑	zinc finger protein 501 [Lipotes vexillifer]
ENSP00000403290-D1	-1.65	4.46E-04	↑	Putative gluconokinase, partial [B. mutus]
ENSP00000361776-D4	-1.65	3.49E-12	↑	protein BEX5 isoform X2 [B. taurus]
ENSBTAP00000051854-D1	-1.65	5.35E-10	↑	Leucine-rich repeat transmembrane protein FLRT2, partial [B. mutus]

ENSBTAP00000005375-D1	-1.65	8.20E-08	↑	guanidinoacetate N-methyltransferase [B. taurus]
ENSBTAP00000010324-D1	-1.65	2.65E-89	↑	Polycomb protein EED, partial [B. mutus]
ENSP00000276590-D1	-1.65	2.08E-32	↑	beta-lactamase-like protein 2 [B. mutus]
ENSP00000377865-D1	-1.64	4.55E-34	↑	60S ribosomal protein L23 [Nipponia nippon]
ENSBTAP00000002785-D1	-1.64	2.23E-03	↑	cytochrome P450 4V2-like [B. mutus]
ENSBTAP00000002976-D1	-1.64	0.00E+00	↑	probable U3 small nucleolar RNA-associated protein 11 [B. mutus]
ENSBTAP00000037080-D1	-1.64	8.47E-09	↑	calcipressin-1 isoform X2 [Orycteropus afer afer]
ENSBTAP00000046618-D2	-1.64	4.72E-62	↑	Zinc finger CW-type PWWP domain protein 2, partial [B. mutus]
ENSBTAP00000050555-D1	-1.64	5.52E-15	↑	RAB6-interacting golgin [B. mutus]
ENSP00000294923-D1	-1.64	1.25E-09	↑	desumoylating isopeptidase 2 [Aotus nancymaae]
ENSBTAP00000003094-D1	-1.64	9.04E-06	↑	transforming growth factor beta-1-induced transcript 1 protein isoform X1 [Bubalus bubalis]
ENSBTAP00000051619-D1	-1.64	2.31E-90	↑	cytochrome c oxidase subunit 7C, mitochondrial [B. taurus]
ENSP00000262776-D1	-1.64	1.84E-10	↑	galectin-3-binding protein [B. mutus]
ENSBTAP00000010178-D1	-1.64	1.84E-10	↑	midkine isoform X2 [Bison bison bison]
ENSP00000415424-D1	-1.63	4.05E-50	↑	essential MCU regulator, mitochondrial precursor [B. taurus]
ENSP00000410885-D7	-1.63	3.09E-04	↑	40S ribosomal protein S27-like [B. mutus]
ENSP00000365141-D9	-1.63	2.81E-33	↑	Putative tripartite motif-containing protein 64B, partial [B. mutus]
ENSBTAP00000023779-D1	-1.63	1.91E-07	↑	zinc finger matrin-type protein 5 [B. taurus]
ENSBTAP00000024572-D1	-1.63	0.00E+00	↑	Vimentin, partial [B. mutus]
ENSBTAP00000026776-D1	-1.63	2.57E-64	↑	stathmin-2 [Mus musculus]
ENSP00000375909-D1	-1.62	2.90E-09	↑	A-kinase anchor protein SPHKAP [B. mutus]
ENSP00000371103-D4	-1.62	3.81E-78	↑	60S ribosomal protein L37-like [Elephantulus edwardii]
ENSP00000317110-D1	-1.62	1.35E-114	↑	Centromere protein S, partial [B. mutus]
ENSP00000371886-D1	-1.62	3.06E-05	↑	Cytosolic phospholipase A2 beta [B. mutus]
ENSP00000356793-D1	-1.62	1.54E-03	↑	lymphotactin [B. taurus]
ENSBTAP00000024042-D1	-1.62	1.16E-02	↑	T-lymphocyte activation antigen CD80 [B. mutus]
ENSP00000333917-D1	-1.62	1.16E-02	↑	Dual specificity protein phosphatase 18 [B. mutus]
ENSBTAP00000016176-D1	-1.62	1.16E-02	↑	Putative protein KIAA0415, partial [B. mutus]
ENSBTAP00000045934-D1	-1.62	1.16E-02	↑	Antigen peptide transporter 1, partial [B. mutus]
ENSBTAP00000012247-D1	-1.62	1.16E-02	↑	Xylosyltransferase 2, partial [B. mutus]
ENSP00000328137-D1	-1.62	1.16E-02	↑	hypothetical protein M91_00034, partial [B. mutus]
ENSBTAP00000046309-D1	-1.62	1.16E-02	↑	Butyrophilin-like protein 2 [B. mutus]
ENSBTAP00000025512-D1	-1.62	1.16E-02	↑	Paired mesoderm homeobox protein 2A, partial [B. mutus]
ENSP00000381861-D1	-1.62	1.16E-02	↑	tetratricopeptide repeat protein 29 isoform X1 [B. taurus]
ENSBTAP00000005369-D1	-1.62	1.54E-19	↑	Centromere protein X, partial [B. mutus]
ENSBTAP00000037698-D1	-1.62	3.20E-16	↑	LYR motif-containing protein 2 [B. taurus]
ENSP00000360425-D1	-1.62	9.91E-14	↑	Calcipressin-2, partial [B. mutus]
ENSP00000260956-D1	-1.62	0.00E+00	↑	lupus La protein homolog [B. mutus]
ENSP00000386867-D1	-1.61	6.54E-08	↑	PTB domain-containing engulfment adapter protein 1 isoform X1 [B. mutus]
ENSBTAP00000021984-D1	-1.61	4.46E-07	↑	carbonic anhydrase 5B, mitochondrial [B. mutus]
ENSBTAP00000008725-D1	-1.61	2.12E-05	↑	uncharacterized protein C4orf19 homolog [B. mutus]
ENSP00000410146-D1	-1.61	3.38E-14	↑	Glutamate receptor 2, partial [B. mutus]
ENSBTAP00000007841-D1	-1.61	1.02E-10	↑	3-keto-steroid reductase [B. taurus]
ENSBTAP00000050952-D8	-1.61	3.11E-07	↑	histone H3.1-like [B. mutus]
ENSBTAP00000007955-D1	-1.61	1.48E-04	↑	protein HEXIM1 [B. taurus]
ENSBTAP00000024880-D1	-1.61	4.71E-09	↑	Acyl-CoA synthetase short-chain family member 3, mitochondrial, partial [B. mutus]
ENSP00000304229-D2	-1.61	1.81E-284	↑	histidine triad nucleotide-binding protein 1 [B. mutus]
ENSBTAP00000008272-D1	-1.60	1.13E-46	↑	estradiol 17-beta-dehydrogenase 11 [B. mutus]
ENSBTAP00000041289-D1	-1.60	1.69E-31	↑	interferon-induced GTP-binding protein Mx1 isoform X1 [B. mutus]
ENSBTAP00000038273-D2	-1.60	3.81E-63	↑	uncharacterized protein LOC105612963 [Ovis aries musimon]
ENSBTAP00000044311-D1	-1.60	4.03E-19	↑	protein FAM36A [B. taurus]
ENSP00000326813-D1	-1.60	1.48E-05	↑	ubiquitin thioesterase OTU1 [B. mutus]
ENSP00000369979-D1	-1.60	1.06E-03	↑	probable carboxypeptidase X1 precursor [B. taurus]
ENSBTAP00000028278-D2	-1.60	3.39E-10	↑	Zinc finger SWIM domain-containing protein 5, partial [B. mutus]
ENSBTAP00000018283-D9	-1.60	8.35E-16	↑	Protein BEX3, partial [B. mutus]

ENSBTAP00000007149-D1	-1.60	2.30E-09	↑	LIM and cysteine-rich domains protein 1, partial [B. mutus]
ENSP00000387209-D1	-1.60	1.08E-22	↑	hypothetical protein M91_13856, partial [B. mutus]
ENSP00000259569-D1	-1.59	4.10E-16	↑	TPA: karyopherin beta 3-like [B. taurus]
ENSBTAP00000050787-D1	-1.59	2.25E-45	↑	uncharacterized protein C8orf59 homolog [B. taurus]
ENSBTAP00000017599-D1	-1.59	1.06E-44	↑	checkpoint protein HUS1 isoform X1 [B. taurus]
ENSBTAP00000023192-D1	-1.59	5.30E-23	↑	kelch-like protein 24 [B. taurus]
ENSBTAP00000019184-D3	-1.59	5.18E-45	↑	60S ribosomal protein L22-like, partial [Bubalus bubalis]
ENSP00000244217-D1	-1.59	3.07E-18	↑	Methylmalonyl-CoA epimerase, mitochondrial, partial [B. mutus]
ENSBTAP00000029970-D1	-1.59	7.17E-05	↑	nudix (nucleoside diphosphate linked moiety X)-type motif 11 [B. taurus]
ENSBTAP00000050234-D1	-1.59	7.17E-05	↑	adenylate kinase isoenzyme 1 isoform X1 [B. mutus]
ENSBTAP00000003031-D1	-1.59	7.32E-04	↑	Syntaxin-1A, partial [B. mutus]
ENSP00000361818-D1	-1.59	7.92E-03	↑	Syndecan-4, partial [B. mutus]
ENSBTAP00000053704-D2	-1.59	7.92E-03	↑	BDNF/NT-3 growth factors receptor-like isoform X1 [B. mutus]
ENSP00000249499-D1	-1.59	7.92E-03	↑	homeobox protein Hox-C9 [Homo sapiens]
ENSBTAP00000024433-D1	-1.59	7.92E-03	↑	Transmembrane protein 132A, partial [B. mutus]
yakG009841	-1.59	7.92E-03	↑	V-type proton ATPase subunit F [B. taurus]
ENSBTAP00000008890-D1	-1.59	7.92E-03	↑	Carbonic anhydrase-related protein 11 [B. mutus]
ENSBTAP00000008042-D1	-1.59	0.00E+00	↑	arginine/serine-rich coiled-coil protein 2 isoform X1 [Microcebus murinus]
ENSP00000339659-D1	-1.58	3.83E-10	↑	Tripartite motif-containing protein 2, partial [B. mutus]
ENSBTAP00000025066-D1	-1.58	4.99E-06	↑	CKLF-like MARVEL transmembrane domain-containing protein 2-like [B. mutus]
ENSP00000314099-D1	-1.58	4.99E-05	↑	carbonic anhydrase 5B, mitochondrial [B. mutus]
ENSBTAP00000014306-D5	-1.58	0.00E+00	↑	myosin light polypeptide 6 isoform X2 [Capra hircus]
ENSBTAP00000043450-D1	-1.58	1.38E-12	↑	hypothetical protein M91_11909, partial [B. mutus]
ENSP00000334113-D1	-1.58	5.08E-04	↑	Dehydrogenase/reductase SDR family member on chromosome X, partial [B. mutus]
ENSP00000264360-D1	-1.58	5.08E-04	↑	Protocadherin-10, partial [B. mutus]
ENSBTAP00000023994-D1	-1.58	5.08E-04	↑	Nuclear receptor subfamily 1 group D member 2, partial [B. mutus]
ENSBTAP00000010696-D1	-1.57	4.76E-46	↑	SRA stem-loop-interacting RNA-binding protein, mitochondrial [B. taurus]
ENSBTAP00000041017-D5	-1.57	1.27E-89	↑	cytochrome c oxidase subunit 7B, mitochondrial precursor [B. taurus]
ENSBTAP00000036858-D1	-1.57	3.45E-05	↑	protein LBH [B. taurus]
ENSBTAP00000025895-D1	-1.57	7.07E-60	↑	mediator of RNA polymerase II transcription subunit 28 [Orcinus orca]
ENSBTAP00000001847-D1	-1.57	1.19E-15	↑	CB1 cannabinoid receptor-interacting protein 1 [B. taurus]
ENSP00000380988-D1	-1.57	5.41E-03	↑	Protein FAM188B2, partial [B. mutus]
ENSBTAP00000013144-D1	-1.57	5.41E-03	↑	3,2-trans-enoyl-CoA isomerase, mitochondrial, partial [B. mutus]
ENSBTAP00000009404-D1	-1.57	5.41E-03	↑	probable serine carboxypeptidase CPVL [B. mutus]
ENSBTAP00000019111-D1	-1.56	0.00E+00	↑	centrin-3 [Ovis aries]
ENSBTAP00000005523-D1	-1.56	0.00E+00	↑	retinoic acid receptor responder protein 2 [B. mutus]
ENSP00000367415-D1	-1.56	4.30E-10	↑	glutathione S-transferase kappa 1 isoform X1 [B. mutus]
ENSP00000297268-D1	-1.56	6.35E-71	↑	collagen alpha-2(I) chain isoform X1 [B. taurus]
ENSBTAP00000021457-D1	-1.56	2.16E-11	↑	GPI transamidase component PIG-T, partial [B. mutus]
ENSBTAP00000042008-D1	-1.56	4.17E-09	↑	prenylcysteine oxidase-like [B. mutus]
ENSBTAP00000022140-D1	-1.56	2.74E-15	↑	Aminopeptidase Q, partial [B. mutus]
ENSP00000401831-D1	-1.56	2.43E-04	↑	Acyl-CoA synthetase family member 2, mitochondrial [B. mutus]
ENSBTAP00000011678-D2	-1.55	3.69E-13	↑	calpain-2 catalytic subunit precursor [B. taurus]
ENSP00000363979-D1	-1.55	5.56E-85	↑	hypothetical protein M91_20167, partial [B. mutus]
ENSP00000217893-D1	-1.55	2.71E-302	↑	transcription initiation factor TFIID subunit 9-like isoform X1 [B. mutus]
ENSBTAP00000028235-D1	-1.55	1.75E-83	↑	DNA-binding protein inhibitor ID-2 [B. taurus]
ENSBTAP00000024453-D12	-1.55	1.98E-26	↑	Prostaglandin F synthase 1, partial [B. mutus]
ENSBTAP00000040669-D1	-1.55	5.60E-06	↑	hypothetical protein M91_21619 [B. mutus]
ENSBTAP00000053865-D1	-1.55	5.60E-06	↑	dachshund homolog 1 isoform X2 [Ovis aries]
ENSBTAP00000042500-D1	-1.54	2.21E-289	↑	mesencephalic astrocyte-derived neurotrophic factor precursor [B. taurus]
ENSBTAP00000050340-D2	-1.54	1.51E-73	↑	cytochrome c oxidase subunit 7C, mitochondrial [B. taurus]
ENSP00000326806-D1	-1.54	3.76E-38	↑	nuclear cap-binding protein subunit 2 isoform X2 [Chlorocebus sabaeus]
yakG033319	-1.54	3.90E-06	↑	hypothetical protein M91_19059 [B. mutus]
ENSP00000319914-D1	-1.54	2.55E-27	↑	uncharacterized protein C8orf4 homolog [B. taurus]
ENSBTAP00000047796-D1	-1.54	9.08E-29	↑	DNA excision repair protein ERCC-8 [B. mutus]

ENSBTAP00000042601-D1	-1.54	1.33E-07	↑	NKAP-like protein [B. mutus]
ENSP00000321468-D1	-1.54	2.53E-03	↑	hypothetical protein M91_20309, partial [B. mutus]
ENSP00000390147-D2	-1.54	2.97E-18	↑	TPA: ribosomal protein L10a-like [B. taurus]
yakA00390	-1.53	4.51E-08	↑	hypothetical protein M91_21712 [B. mutus]
ENSBTAP00000004617-D1	-1.53	2.76E-16	↑	hypothetical protein M91_18005, partial [B. mutus]
ENSBTAP00000023277-D1	-1.53	9.11E-07	↑	G1/S-specific cyclin-D1 [B. mutus]
ENSBTAP00000004942-D1	-1.53	1.74E-03	↑	tektin-3 [B. mutus]
ENSBTAP00000045378-D1	-1.53	3.73E-10	↑	up-regulated during skeletal muscle growth protein 5 [B. taurus]
ENSP00000350848-D1	-1.53	1.53E-08	↑	High mobility group nucleosome-binding domain-containing protein 5, partial [B. mutus]
ENSBTAP00000018160-D1	-1.52	1.81E-10	↑	uncharacterized protein C3orf26 homolog [B. mutus]
ENSP00000341610-D1	-1.52	4.40E-07	↑	normal mucosa of esophagus-specific gene 1 protein [B. mutus]
ENSBTAP00000014563-D1	-1.52	1.19E-03	↑	Transmembrane emp24 domain-containing protein 1, partial [B. mutus]
ENSP00000264350-D1	-1.52	1.50E-12	↑	E3 ISG15--protein ligase HERC5, partial [B. mutus]
ENSBTAP00000017169-D1	-1.52	1.50E-12	↑	transmembrane protein 70, mitochondrial precursor [B. taurus]
ENSBTAP00000016957-D1	-1.52	1.62E-123	↑	NADH dehydrogenase [ubiquinone] 1 beta subcomplex subunit 3 [Ovis aries]
ENSP00000360826-D1	-1.52	1.29E-05	↑	cyclin-dependent kinase 4 inhibitor C [B. taurus]
ENSP00000270645-D2	-1.52	1.49E-20	↑	reticulocalbin-1 isoform X2 [Ovis aries musimon]
ENSBTAP00000046591-D23	-1.52	3.55E-13	↑	hypothetical protein M91_12051, partial [B. mutus]
ENSBTAP00000043789-D1	-1.52	1.75E-09	↑	Apolipoprotein D, partial [B. mutus]
ENSP00000310749-D3	-1.51	8.95E-06	↑	neurocalcin-delta [B. taurus]
ENSBTAP00000024209-D1	-1.51	1.94E-77	↑	NADH dehydrogenase [ubiquinone] 1 subunit C2 [B. mutus]
ENSP00000322579-D1	-1.51	2.38E-12	↑	PHD finger protein 23 isoform X1 [B. mutus]
ENSBTAP00000022755-D1	-1.51	5.68E-04	↑	Protein FAM8A1, partial [B. mutus]
ENSBTAP00000007711-D2	-1.51	1.66E-12	↑	Heme-binding protein 1 [B. mutus]
ENSBTAP00000026202-D1	-1.50	1.05E-29	↑	2,4-dienoyl-CoA reductase, mitochondrial [B. mutus]
ENSBTAP00000003636-D1	-1.50	1.24E-115	↑	Proteasome subunit alpha type-3, partial [B. mutus]
ENSP00000347919-D1	-1.50	3.48E-37	↑	coiled-coil-helix-coiled-coil-helix domain-containing protein 8 [B. taurus]
ENSP00000358879-D1	-1.50	2.31E-11	↑	Filamin-A [B. mutus]
ENSBTAP00000020299-D2	-1.50	2.70E-04	↑	galectin-16 [B. mutus]
ENSBTAP00000027579-D2	-1.50	2.70E-04	↑	hypothetical protein M91_16352, partial [B. mutus]
ENSBTAP00000000693-D1	-1.50	2.70E-04	↑	Glutamate decarboxylase 1, partial [B. mutus]
ENSP00000233609-D1	-1.50	0.00E+00	↑	40S ribosomal protein S15, partial [B. mutus]
ENSBTAP00000036773-D1	-1.50	4.51E-57	↑	40S ribosomal protein S29 [Pteropus vampyrus]
ENSBTAP00000041338-D1	-1.50	1.13E-98	↑	transmembrane protein 176B isoform X1 [B. mutus]
ENSP00000349785-D1	-1.50	1.71E-46	↑	microtubule-associated proteins 1A/1B light chain 3C [B. taurus]
ENSP00000385946-D2	-1.49	4.58E-10	↑	midkine [Bison bison bison]
ENSP00000330302-D1	-1.49	2.36E-07	↑	Protocadherin-7, partial [B. mutus]
yakG003629	-1.49	7.57E-73	↑	26S protease regulatory subunit 10B [B. mutus]
ENSP00000257700-D1	-1.49	2.32E-21	↑	RAD50-interacting protein 1, partial [B. mutus]
ENSP00000280987-D1	-1.49	7.47E-69	↑	protein FAM177A1 isoform X1 [B. mutus]
ENSP00000262946-D4	-1.49	7.90E-22	↑	cytochrome b-c1 complex subunit 10-like [Capra hircus]
ENSP00000324444-D1	-1.49	1.08E-122	↑	MAP3K12-binding inhibitory protein 1 isoform X1 [B. mutus]
ENSP00000286371-D6	-1.49	6.51E-40	↑	Sodium/potassium-transporting ATPase subunit beta-3, partial [B. mutus]
ENSBTAP00000015763-D1	-1.49	7.96E-08	↑	MAL-like protein [B. mutus]
ENSBTAP00000002916-D1	-1.49	6.20E-05	↑	betaine--homocysteine S-methyltransferase 1 isoform X1 [B. mutus]
ENSBTAP00000050877-D1	-1.49	2.17E-23	↑	pyridine nucleotide-disulfide oxidoreductase domain-containing protein 1 [B. mutus]
ENSP00000376097-D1	-1.49	1.88E-118	↑	Unconventional prefoldin RPB5 interactor, partial [B. mutus]
ENSBTAP00000008907-D1	-1.49	1.17E-14	↑	D-3-phosphoglycerate dehydrogenase [B. mutus]
ENSP00000378524-D1	-1.49	4.29E-05	↑	leucine-rich repeat and IQ domain-containing protein 3 [B. mutus]
ENSP00000306396-D1	-1.49	2.98E-05	↑	Protein RNF187, partial [B. mutus]
ENSP00000360942-D1	-1.49	2.98E-05	↑	Tumor necrosis factor receptor superfamily member 6, partial [B. mutus]
ENSBTAP00000020053-D1	-1.48	3.34E-13	↑	Transmembrane protease serine 7 [B. mutus]
ENSP00000253382-D1	-1.48	4.78E-06	↑	Acetyl-coenzyme A synthetase, cytoplasmic [B. mutus]
ENSBTAP00000050477-D1	-1.48	4.78E-06	↑	ras-related GTP-binding protein B-like [B. mutus]
ENSBTAP00000029091-D1	-1.48	1.42E-27	↑	protein ARV1 [B. mutus]

ENSP00000276201-D1	-1.48	1.02E-136	↑	regulator of nonsense transcripts 3B isoform X4 [B. taurus]
ENSBTAP00000027728-D1	-1.47	0.00E+00	↑	Ubiquitin-like protein FUBI, partial [B. mutus]
ENSBTAP00000025691-D1	-1.46	1.46E-277	↑	Malate dehydrogenase, cytoplasmic, partial [B. mutus]
ENSBTAP00000052370-D12	-1.46	1.81E-119	↑	40S ribosomal protein S27-like isoform X4 [Bison bison bison]
ENSBTAP00000052430-D14	-1.46	6.24E-60	↑	40S ribosomal protein S27 isoform X2 [Larimichthys crocea]
ENSP00000301587-D3	-1.46	1.27E-214	↑	ATP synthase subunit d, mitochondrial [B. taurus]
ENSP00000252102-D3	-1.46	1.66E-138	↑	NADH dehydrogenase [ubiquinone] 1 alpha subcomplex subunit 2 [B. taurus]
ENSP00000291458-D2	-1.45	1.16E-272	↑	Coiled-coil domain-containing protein 58, partial [B. mutus]
ENSBTAP00000042698-D5	-1.45	2.83E-28	↑	putative RNA polymerase II subunit A C-terminal domain phosphatase SSU72-like protein 1 [Bison bison bison]
ENSP00000356832-D1	-1.45	1.51E-21	↑	serine/threonine-protein kinase Sgk1 isoform X1 [B. taurus]
ENSP00000295220-D1	-1.45	1.79E-09	↑	Primary ciliary dyskinesia protein 1, partial [B. mutus]
ENSP00000306817-D1	-1.45	3.70E-09	↑	ribokinase-like [B. mutus]
ENSP00000280354-D1	-1.45	3.23E-08	↑	mitochondrial import inner membrane translocase subunit Tim8 B [B. taurus]
ENSP00000262547-D1	-1.45	1.38E-07	↑	double zinc ribbon and ankyrin repeat-containing protein 1 [Vicugna pacos]
ENSBTAP00000029451-D1	-1.45	1.98E-07	↑	HORMA domain-containing protein 2 [B. mutus]
ENSBTAP00000053784-D1	-1.45	1.22E-06	↑	Dynein heavy chain 14, axonemal, partial [B. mutus]
ENSBTAP00000053067-D4	-1.45	2.53E-06	↑	histone H3.3 [Felis catus]
ENSP00000307423-D1	-1.45	1.57E-05	↑	alpha-1,6-mannosyl-glycoprotein 2-beta-N-acetylglucosaminyltransferase [Bison bison bison]
ENSP00000320081-D1	-1.45	3.26E-05	↑	hypothetical protein M91_10250, partial [B. mutus]
ENSP00000362684-D1	-1.45	4.32E-04	↑	protein FAM167B [B. mutus]
ENSP00000330161-D1	-1.45	6.27E-04	↑	Receptor-interacting serine/threonine-protein kinase 4, partial [B. mutus]
ENSBTAP00000019752-D1	-1.45	9.07E-04	↑	voltage-dependent P/Q-type calcium channel subunit alpha-1A [B. mutus]
ENSP00000370526-D3	-1.45	9.07E-04	↑	Arylsulfatase D, partial [B. mutus]
ENSP00000221166-D1	-1.45	9.07E-04	↑	neurofilament medium polypeptide [B. mutus]
ENSP00000262188-D3	-1.45	9.07E-04	↑	SWI/SNF-related matrix-associated actin-dependent regulator of chromatin subfamily D member 3 [B. mutus]
ENSP00000360899-D1	-1.45	1.93E-03	↑	BEN domain-containing protein 5, partial [B. mutus]
ENSP00000410007-D1	-1.45	2.81E-03	↑	F-box protein 46 [B. mutus]
ENSP00000265825-D1	-1.45	2.81E-03	↑	Fascin-3 [B. mutus]
ENSBTAP00000040682-D1	-1.45	4.10E-03	↑	trans-1,2-dihydrobenzene-1,2-diol dehydrogenase [B. taurus]
ENSP00000360916-D2	-1.45	4.10E-03	↑	Proto-oncogene vav [B. mutus]
yakG029648	-1.45	5.98E-03	↑	Nuclear factor 1 A-type [B. mutus]
ENSBTAP00000034540-D1	-1.45	8.86E-03	↑	serine/threonine-protein kinase MAK isoform X2 [B. mutus]
ENSBTAP00000052592-D80	-1.45	8.86E-03	↑	hypothetical protein M91_02702, partial [B. mutus]
ENSBTAP00000050413-D1	-1.45	1.31E-02	↑	hypothetical protein LOC91289 [B. taurus]
ENSBTAP00000018404-D1	-1.45	1.31E-02	↑	single-stranded DNA-binding protein 2 isoform X2 [Chrysochloris asiatica]
ENSP00000357354-D1	-1.45	1.31E-02	↑	Protein FAM189B [B. mutus]
ENSP00000388762-D1	-1.45	1.31E-02	↑	tripeptidyl-peptidase 1 [B. mutus]
ENSBTAP00000026454-D1	-1.45	1.31E-02	↑	EGF-like module-containing mucin-like hormone receptor-like 4, partial [B. mutus]
ENSBTAP00000044658-D1	-1.45	1.31E-02	↑	methyltransferase-like protein 24 precursor [B. taurus]
ENSBTAP00000043710-D1	-1.45	1.31E-02	↑	tuftelin isoform X1 [B. mutus]
ENSP00000301057-D1	-1.45	1.31E-02	↑	Tumor protein p53-inducible protein 13, partial [B. mutus]
ENSBTAP00000050176-D13	-1.45	1.95E-02	↑	histone H2B type 1-J-like [Camelus ferus]
ENSP00000378204-D1	-1.45	1.95E-02	↑	zinc finger and BTB domain-containing protein 25 [B. mutus]
ENSBTAP00000042049-D1	-1.45	2.99E-02	↑	phospholemman precursor [B. taurus]
ENSP00000339952-D1	-1.45	2.99E-02	↑	Kinase suppressor of Ras 2, partial [B. mutus]
ENSP00000289228-D1	-1.45	2.99E-02	↑	beta-centractin [B. taurus]
ENSP00000410076-D1	-1.45	2.99E-02	↑	caspase-1-like [B. mutus]
ENSBTAP00000044382-D1	-1.45	2.99E-02	↑	mammalian ependymin-related protein 1 [Bison bison bison]
ENSP00000388723-D1	-1.45	2.99E-02	↑	kelch repeat and BTB domain-containing protein 13 [Bubalus bubalis]
ENSP00000386435-D1	-1.45	2.99E-02	↑	Protein FAM176A, partial [B. mutus]
ENSP00000392188-D8	-1.45	2.99E-02	↑	hypothetical protein M91_18372, partial [B. mutus]
ENSBTAP00000007806-D1	-1.45	2.99E-02	↑	urokinase-type plasminogen activator [B. mutus]
yakG000522	-1.45	2.99E-02	↑	hypothetical protein M91_00584 [B. mutus]
ENSP00000420560-D1	-1.45	2.99E-02	↑	leucine-rich repeat-containing protein 61 [B. mutus]
ENSP00000415299-D1	-1.45	2.99E-02	↑	hypothetical protein M91_01548 [B. mutus]

ENSP00000391968-D2	-1.45	2.99E-02	↑	hypothetical protein LOC100428091 isoform 1 [Macaca mulatta]
ENSBTAP00000007338-D1	-1.45	4.68E-02	↑	Glycine amidinotransferase, mitochondrial, partial [B. mutus]
ENSP00000328928-D1	-1.45	4.68E-02	↑	Homeobox protein Hox-B4 [B. mutus]
ENSBTAP00000050179-D67	-1.45	4.68E-02	↑	olfactory receptor 51L1-like isoform X1 [Ovis aries]
ENSP00000278886-D2	-1.45	4.68E-02	↑	ninein-like protein isoform X4 [B. taurus]
ENSP00000344218-D1	-1.45	4.68E-02	↑	hypothetical protein M91_07487, partial [B. mutus]
ENSBTAP00000015284-D1	-1.45	4.68E-02	↑	hypothetical protein M91_18522, partial [B. mutus]
ENSP00000392188-D50	-1.45	4.68E-02	↑	hypothetical protein M91_04089, partial [B. mutus]
ENSP00000256861-D1	-1.45	4.68E-02	↑	Inter-alpha-trypsin inhibitor heavy chain H5, partial [B. mutus]
ENSBTAP00000035484-D1	-1.45	4.68E-02	↑	Fibrillin-3, partial [B. mutus]
yakA09141	-1.45	4.68E-02	↑	hypothetical protein M91_05413 [B. mutus]
ENSBTAP00000046324-D1	-1.45	4.68E-02	↑	uncharacterized protein LOC102264979 [B. mutus]
ENSBTAP00000007078-D1	-1.45	4.68E-02	↑	solute carrier family 41 member 1 [B. taurus]
ENSBTAP00000033405-D1	-1.45	4.68E-02	↑	uricase [Bison bison bison]
ENSBTAP00000002194-D1	-1.45	4.68E-02	↑	Inositol-trisphosphate 3-kinase A, partial [B. mutus]
ENSBTAP00000010706-D1	-1.45	4.68E-02	↑	sphingomyelin phosphodiesterase 2 [B. taurus]
ENSBTAP00000012735-D1	-1.44	5.30E-19	↑	DNA-binding protein A, partial [B. mutus]
ENSBTAP00000014217-D1	-1.44	2.30E-35	↑	biogenesis of lysosome-related organelles complex-1 subunit 2 [Rattus norvegicus]
ENSBTAP00000027091-D2	-1.44	8.63E-12	↑	protein BTG1-like [Elephantulus edwardii]
ENSBTAP00000015248-D1	-1.44	0.00E+00	↑	Myosin regulatory light polypeptide 9 [Myotis brandtii]
ENSBTAP00000052140-D1	-1.44	1.04E-17	↑	ubiquitin-like protein 5 isoform X1 [Physeter catodon]
ENSP00000260746-D1	-1.43	2.91E-71	↑	ADP-ribosylation factor-like protein 3 [B. taurus]
ENSBTAP00000026534-D1	-1.43	2.48E-53	↑	Gelsolin, partial [B. mutus]
ENSP00000161863-D1	-1.43	1.18E-20	↑	probable ATP-dependent RNA helicase YTHDC2 [B. taurus]
ENSBTAP00000051131-D1	-1.43	2.54E-13	↑	coiled-coil domain-containing protein 68 [B. mutus]
ENSBTAP00000014975-D1	-1.43	1.50E-07	↑	Sorting nexin-31 [B. mutus]
ENSBTAP00000005903-D1	-1.43	3.28E-24	↑	pro-neuropeptide Y precursor [B. taurus]
ENSBTAP00000012118-D1	-1.42	2.52E-17	↑	ankyrin repeat and SOCS box protein 9 isoform X1 [B. mutus]
ENSP00000253233-D1	-1.42	2.29E-54	↑	hypothetical protein M91_19035, partial [B. mutus]
ENSBTAP00000007160-D1	-1.42	1.71E-05	↑	WD repeat-containing protein 78 [B. taurus]
ENSP00000356879-D1	-1.42	6.30E-09	↑	Regulator of G-protein signaling 5, partial [B. mutus]
ENSP00000358964-D1	-1.42	3.55E-05	↑	uncharacterized protein C1orf194 homolog [B. mutus]
ENSP00000315017-D1	-1.42	0.00E+00	↑	39S ribosomal protein L1, mitochondrial [B. mutus]
ENSP00000260619-D1	-1.42	2.50E-21	↑	THUMP domain-containing protein 2 [B. mutus]
ENSP00000381992-D1	-1.42	1.64E-42	↑	septin-7 isoform X1 [B. mutus]
ENSP00000414321-D1	-1.42	1.71E-160	↑	40S ribosomal protein S24, partial [B. mutus]
ENSBTAP00000008938-D1	-1.41	7.41E-05	↑	E3 ubiquitin-protein ligase MARCH3 [B. taurus]
ENSBTAP00000010276-D1	-1.41	1.32E-21	↑	NADH dehydrogenase [ubiquinone] 1 alpha subcomplex subunit 13, partial [B. mutus]
ENSBTAP00000044412-D1	-1.41	1.00E-15	↑	E3 ubiquitin-protein ligase RNF170 isoform X1 [Ovis aries]
ENSP00000354703-D1	-1.41	2.34E-07	↑	zinc finger protein 358 [B. mutus]
ENSP00000416268-D2	-1.41	1.55E-04	↑	hypothetical protein M91_15086, partial [B. mutus]
ENSP00000301457-D1	-1.41	2.40E-24	↑	NADH dehydrogenase [ubiquinone] 1 alpha subcomplex subunit 7, partial [B. mutus]
ENSBTAP00000028238-D1	-1.41	3.97E-31	↑	Cadherin-2, partial [B. mutus]
ENSBTAP00000021123-D2	-1.40	1.24E-20	↑	NADH dehydrogenase [ubiquinone] 1 beta subcomplex subunit 4, partial [B. mutus]
ENSBTAP00000039615-D1	-1.40	2.55E-20	↑	eukaryotic translation initiation factor 4E-binding protein 1 [B. taurus]
ENSP00000390625-D1	-1.40	7.57E-18	↑	heterogeneous nuclear ribonucleoprotein L-like isoform X1 [B. mutus]
ENSBTAP00000006309-D1	-1.40	1.41E-08	↑	T-cell immunomodulatory protein precursor [B. taurus]
ENSBTAP00000012029-D1	-1.40	4.30E-37	↑	Cohesin subunit SA-2, partial [B. mutus]
ENSBTAP00000042915-D2	-1.40	6.66E-13	↑	hypothetical protein M91_04874, partial [B. mutus]
ENSP00000332225-D1	-1.40	1.05E-53	↑	pro-MCH [B. mutus]
ENSP00000375651-D1	-1.40	4.70E-04	↑	myeloid-associated differentiation marker [B. mutus]
ENSP00000390724-D1	-1.40	1.99E-10	↑	Ribonuclease inhibitor, partial [B. mutus]
ENSBTAP00000053832-D1	-1.40	4.14E-08	↑	sterile alpha motif domain-containing protein 9 [B. mutus]
ENSBTAP00000027504-D1	-1.40	5.95E-08	↑	metalloproteinase inhibitor 3-like isoform X1 [B. mutus]
ENSBTAP00000020069-D1	-1.40	6.83E-04	↑	hypothetical protein M91_20563, partial [B. mutus]

ENSP00000416650-D1	-1.40	1.04E-38	↑	Proteasome activator complex subunit 2 [B. mutus]
ENSBTAP00000051734-D1	-1.40	8.91E-06	↑	hypothetical protein M91_18687, partial [B. mutus]
ENSBTAP00000019734-D1	-1.39	2.92E-81	↑	myosin light chain kinase 3 [B. mutus]
ENSBTAP00000020679-D1	-1.39	9.89E-04	↑	DLA class II histocompatibility antigen, DR-1 beta chain-like isoform X1 [Bison bison bison]
ENSP00000233997-D1	-1.39	9.89E-04	↑	azurocidin [B. taurus]
ENSBTAP00000008072-D2	-1.39	4.10E-39	↑	Microtubule-associated proteins 1A/1B light chain 3A, partial [B. mutus]
ENSBTAP00000040453-D1	-1.39	6.82E-115	↑	39S ribosomal protein L22, mitochondrial, partial [B. mutus]
ENSBTAP00000002542-D1	-1.39	1.43E-03	↑	histidine triad nucleotide-binding protein 3 [B. mutus]
ENSP00000218006-D1	-1.39	1.43E-03	↑	retinal guanylyl cyclase 2 [B. mutus]
ENSBTAP00000031895-D1	-1.39	5.26E-95	↑	Procollagen-lysine,2-oxoglutarate 5-dioxygenase 2, partial [B. mutus]
ENSP00000359730-D3	-1.39	2.17E-08	↑	Sodium/hydrogen exchanger 9, partial [B. mutus]
ENSBTAP00000051821-D1	-1.39	3.85E-05	↑	hypothetical protein M91_05551, partial [B. mutus]
ENSP00000382134-D1	-1.39	1.09E-06	↑	Putative protein FAM177A2, partial [B. mutus]
ENSP00000294390-D1	-1.38	2.25E-06	↑	lipid phosphate phosphohydrolase 3 [B. taurus]
ENSBTAP00000027024-D1	-1.38	9.23E-08	↑	15-hydroxyprostaglandin dehydrogenase [NAD(+)] isoform X1 [B. mutus]
yakG021684	-1.38	8.03E-05	↑	Calpain small subunit 1 [B. mutus]
ENSP00000398079-D3	-1.38	5.53E-09	↑	putative RNA polymerase II subunit A C-terminal domain phosphatase SSU72-like protein 1-like [B. mutus]
ENSBTAP00000037960-D1	-1.38	1.57E-21	↑	Chain A, Crystallographic Refinement Of Bovine Pro-Phospholipase A2 At 1.6 Angstroms Resolution
ENSBTAP00000027439-D1	-1.38	1.90E-07	↑	BTB/POZ domain-containing protein KCTD20 [B. mutus]
ENSBTAP00000007925-D1	-1.38	4.65E-06	↑	NADH dehydrogenase [ubiquinone] 1 alpha subcomplex subunit 1-like [B. mutus]
ENSBTAP00000024161-D3	-1.38	2.05E-44	↑	28S ribosomal protein S18c, mitochondrial [B. taurus]
ENSP00000052754-D1	-1.37	4.76E-10	↑	decorin precursor [B. taurus]
ENSBTAP00000024453-D7	-1.37	6.83E-10	↑	hypothetical protein M91_01964, partial [B. mutus]
ENSBTAP00000023707-D1	-1.37	1.63E-08	↑	glutaredoxin-like protein C5orf63 homolog [B. taurus]
ENSBTAP00000003666-D1	-1.37	2.32E-35	↑	Porimin, partial [B. mutus]
ENSBTAP00000028928-D1	-1.37	2.17E-38	↑	ubiquitin-fold modifier-conjugating enzyme 1 isoform X2 [B. taurus]
ENSBTAP00000028828-D1	-1.37	3.35E-08	↑	G/T mismatch-specific thymine DNA glycosylase, partial [B. mutus]
ENSBTAP00000006215-D1	-1.37	2.61E-14	↑	G1/S-specific cyclin-E1 [B. taurus]
ENSBTAP00000009939-D1	-1.37	5.99E-130	↑	interferon alpha-inducible protein 6 precursor [B. taurus]
ENSBTAP00000021249-D1	-1.37	2.10E-20	↑	glutaredoxin-2, mitochondrial [B. mutus]
ENSBTAP00000026930-D1	-1.37	2.43E-04	↑	Protein Wnt-5a, partial [B. mutus]
ENSP00000351632-D1	-1.37	4.46E-03	↑	Protein FAM3D, partial [B. mutus]
ENSBTAP00000044748-D1	-1.37	4.46E-03	↑	gamma-1-syntrophin [B. mutus]
ENSBTAP00000041428-D1	-1.37	1.96E-95	↑	GPI-anchor transamidase [B. mutus]
ENSBTAP00000015875-D1	-1.36	3.70E-52	↑	ribosomal protein S19, partial [Homo sapiens]
yakA09846	-1.36	3.50E-04	↑	U6 snRNA-associated Sm-like protein LSm6-like [B. mutus]
ENSP00000366024-D4	-1.36	3.50E-04	↑	BOLA class I histocompatibility antigen, alpha chain BL3-7-like [Bubalus bubalis]
ENSBTAP00000008351-D1	-1.36	3.50E-04	↑	Lysosomal protein NCU-G1, partial [B. mutus]
ENSBTAP00000050442-D5	-1.36	0.00E+00	↑	40S ribosomal protein S3a [Myotis brandtii]
ENSP00000384656-D27	-1.36	0.00E+00	↑	ferritin heavy chain [B. mutus]
ENSP00000375563-D1	-1.36	2.11E-16	↑	PRO0845 [Homo sapiens]
ENSP00000376037-D4	-1.36	5.07E-04	↑	hypothetical protein M91_17386, partial [B. mutus]
ENSBTAP00000004098-D1	-1.36	6.51E-03	↑	Interferon alpha-inducible protein 27-like protein 2, partial [B. mutus]
ENSP00000351363-D1	-1.36	6.51E-03	↑	Beta-microseminoprotein, partial [B. mutus]
ENSP00000386170-D1	-1.36	6.51E-03	↑	T-box transcription factor TBX20 [B. mutus]
ENSP00000330937-D1	-1.35	1.72E-28	↑	NADH dehydrogenase [ubiquinone] 1 alpha subcomplex subunit 6 [B. mutus]
ENSBTAP00000016314-D1	-1.35	7.32E-04	↑	TBC1 domain family member 22A [B. mutus]
ENSP00000376829-D1	-1.35	1.26E-18	↑	Kelch repeat and BTB domain-containing protein 3, partial [B. mutus]
ENSP00000280357-D1	-1.35	3.79E-38	↑	interleukin-18 isoform X2 [B. mutus]
ENSBTAP00000046356-D2	-1.35	1.49E-40	↑	malignant T-cell-amplified sequence 1 isoform X2 [B. taurus]
ENSP00000378191-D1	-1.35	7.80E-97	↑	Aminoacyl tRNA synthase complex-interacting multifunctional protein 1, partial [B. mutus]
ENSBTAP00000051754-D1	-1.35	4.33E-30	↑	39S ribosomal protein L33, mitochondrial, partial [B. mutus]
ENSBTAP00000030207-D1	-1.34	1.39E-47	↑	.
ENSP00000418684-D1	-1.34	3.32E-09	↑	Zinc finger protein Aiolos, partial [B. mutus]
ENSBTAP00000015461-D1	-1.34	1.07E-03	↑	zinc finger and SCAN domain-containing protein 5B-like [Bison bison bison]

ENSBTAP00000015210-D1	-1.34	9.67E-03	↑	Semaphorin-7A, partial [B. mutus]
ENSBTAP00000051671-D14	-1.34	9.67E-03	↑	hypothetical protein M91_04365 [B. mutus]
ENSP00000360803-D1	-1.34	9.67E-03	↑	DNL-type zinc finger protein, partial [B. mutus]
ENSBTAP00000031126-D6	-1.34	9.67E-03	↑	hypothetical protein M91_05882, partial [B. mutus]
ENSBTAP00000041271-D1	-1.34	9.67E-03	↑	membrane-spanning 4-domains subfamily A member 13 [B. taurus]
ENSBTAP00000039787-D1	-1.34	9.67E-03	↑	uncharacterized protein LOC536190 precursor [B. taurus]
ENSBTAP00000035184-D3	-1.34	0.00E+00	↑	S-phase kinase-associated protein 1 [Gorilla gorilla gorilla]
ENSBTAP00000042635-D5	-1.34	2.80E-13	↑	histone H2A.J [Homo sapiens]
ENSBTAP00000036773-D4	-1.34	1.83E-11	↑	40S ribosomal protein S29, partial [Myotis lucifugus]
ENSBTAP00000052092-D1	-1.34	1.00E-21	↑	uncharacterized protein LOC515358 [B. taurus]
ENSP00000354611-D1	-1.34	1.80E-04	↑	pleckstrin homology-like domain family B member 1 [B. mutus]
ENSBTAP00000001319-D1	-1.34	9.76E-09	↑	Protein FAM46A [Myotis brandtii]
ENSBTAP00000028874-D1	-1.34	8.21E-13	↑	Methyltransferase-like protein 4 [B. mutus]
ENSP00000378396-D1	-1.33	1.54E-03	↑	60S ribosomal protein L23a, partial [B. mutus]
ENSP00000358202-D1	-1.33	1.54E-03	↑	homeobox protein EMX2 [B. mutus]
ENSP00000355802-D1	-1.33	1.54E-03	↑	epoxide hydrolase 1 isoform X1 [B. mutus]
ENSP00000037243-D5	-1.33	2.97E-52	↑	Chain A, Crystal Structure Of The Gaba(A) Receptor Associated Protein, Gabarap
ENSP00000265838-D1	-1.33	6.24E-78	↑	Acetyl-CoA acetyltransferase, mitochondrial, partial [B. mutus]
ENSBTAP00000051195-D1	-1.33	4.43E-05	↑	RecName: Full=Progesterone receptor; Short=PR; AltName: Full=Nuclear receptor subfamily 3 group C member 3, partial [Ovis aries]
ENSP00000243346-D1	-1.33	6.39E-21	↑	N-myc-interactor [B. mutus]
ENSBTAP00000001164-D1	-1.33	1.34E-06	↑	Endoplasmic reticulum aminopeptidase 2, partial [B. mutus]
ENSP00000358838-D1	-1.33	7.27E-09	↑	Glutathione S-transferase Mu 1 [B. mutus]
ENSBTAP00000005222-D1	-1.33	2.31E-36	↑	histone deacetylase complex subunit SAP30L [B. taurus]
ENSBTAP00000021699-D3	-1.33	3.59E-106	↑	V-type proton ATPase subunit D [Trichechus manatus latirostris]
ENSP00000334234-D1	-1.32	1.79E-12	↑	Septin-10, partial [B. mutus]
ENSP00000380594-D1	-1.32	3.76E-04	↑	BPI fold-containing family C protein [B. mutus]
ENSP00000257408-D1	-1.32	3.76E-04	↑	Beta-klotho, partial [B. mutus]
ENSP00000400153-D2	-1.32	1.42E-02	↑	Interleukin-32, partial [B. mutus]
ENSP00000395738-D1	-1.32	1.42E-02	↑	tRNA (uracil-5-)-methyltransferase-like protein A, partial [B. mutus]
ENSP00000260983-D1	-1.32	1.42E-02	↑	E3 ubiquitin-protein ligase HECW2 [B. mutus]
ENSBTAP00000047568-D1	-1.32	1.42E-02	↑	uncharacterized protein C11orf63 homolog isoform X1 [B. mutus]
ENSBTAP00000053558-D1	-1.32	1.42E-02	↑	Microtubule-associated serine/threonine-protein kinase 3 [B. mutus]
ENSBTAP00000006500-D1	-1.32	1.42E-02	↑	testis-expressed sequence 35 protein [B. mutus]
ENSBTAP00000010389-D1	-1.32	1.41E-141	↑	mitochondrial fission 1 protein [B. taurus]
ENSP00000269214-D1	-1.32	1.09E-88	↑	N-acetyltransferase ESCO1 [B. mutus]
ENSBTAP00000035742-D1	-1.32	6.31E-22	↑	myelin expression factor 2 [B. mutus]
ENSBTAP00000043889-D1	-1.32	4.09E-29	↑	hypothetical protein M91_21321 [B. mutus]
ENSBTAP00000007584-D1	-1.32	8.44E-08	↑	Nephrocystin-3 [B. mutus]
ENSBTAP00000032144-D1	-1.32	1.18E-10	↑	dol-P-Man:Man(5)GlcNAc(2)-PP-Dol alpha-1,3-mannosyltransferase [B. taurus]
ENSBTAP00000043009-D1	-1.32	6.52E-47	↑	GTP-binding protein 10 [B. mutus]
ENSBTAP00000011702-D1	-1.32	9.20E-05	↑	Progestin and adipoQ receptor family member 3, partial [B. mutus]
ENSBTAP00000004850-D1	-1.32	1.13E-133	↑	Bromodomain testis-specific protein [B. mutus]
ENSBTAP00000013355-D1	-1.32	2.87E-163	↑	Histone acetyltransferase type B catalytic subunit, partial [B. mutus]
ENSBTAP00000002557-D1	-1.32	1.73E-07	↑	Coiled-coil domain-containing protein 39 [B. mutus]
ENSP00000370373-D3	-1.32	1.73E-16	↑	Peptidyl-prolyl cis-trans isomerase FKBP1B [Myotis brandtii]
ENSBTAP00000047570-D7	-1.31	1.25E-129	↑	ribosomal protein L9 [B. taurus]
ENSBTAP00000011390-D1	-1.31	3.28E-03	↑	cAMP-specific 3',5'-cyclic phosphodiesterase 4B, partial [B. mutus]
ENSBTAP00000015436-D1	-1.31	3.27E-43	↑	UPF0711 protein C18orf21 homolog [B. mutus]
ENSBTAP00000045968-D9	-1.31	3.28E-05	↑	hypothetical protein M91_02087, partial [B. mutus]
ENSP00000300302-D1	-1.31	4.01E-09	↑	Homocysteine-responsive endoplasmic reticulum-resident ubiquitin-like domain member 1 protein, partial [B. mutus]
ENSBTAP00000047976-D1	-1.31	2.26E-08	↑	E3 ubiquitin-protein ligase SIAH2, partial [B. mutus]
ENSBTAP00000035146-D1	-1.31	8.03E-22	↑	hypothetical protein M91_08369 [B. mutus]
ENSP00000351506-D2	-1.31	1.72E-144	↑	39S ribosomal protein L42, mitochondrial, partial [B. mutus]

ENSP00000420331-D1	-1.31	0.00E+00	↑	translocation protein SEC62 isoform X1 [Mustela putorius furo]
ENSP00000327539-D2	-1.31	1.10E-119	↑	heterogeneous nuclear ribonucleoprotein H isoform X1 [Camelus dromedarius]
ENSP00000346045-D1	-1.30	1.87E-195	↑	hypothetical protein PANDA_008513, partial [Ailuropoda melanoleuca]
ENSBTAP00000017943-D1	-1.30	1.17E-05	↑	5-hydroxytryptamine receptor 2A [B. mutus]
ENSBTAP00000027559-D1	-1.30	1.57E-15	↑	Contactin-1, partial [B. mutus]
ENSBTAP00000044267-D1	-1.30	7.33E-07	↑	interferon regulatory factor 9 [B. mutus]
ENSBTAP00000010668-D1	-1.30	3.05E-272	↑	intraflagellar transport protein 20 homolog [B. mutus]
ENSBTAP00000053592-D1	-1.30	8.11E-16	↑	frizzled-6 isoform X1 [B. mutus]
ENSBTAP00000020789-D92	-1.30	5.25E-17	↑	mariner transposase [Homo sapiens]
ENSBTAP00000006862-D1	-1.30	4.20E-06	↑	snRNA-activating protein complex subunit 5 [B. mutus]
ENSP00000408464-D1	-1.30	1.69E-05	↑	Dedicator of cytokinesis protein 8, partial [B. mutus]
ENSBTAP00000016040-D1	-1.30	6.80E-05	↑	Fibulin-1, partial [B. mutus]
ENSP00000322304-D2	-1.30	2.75E-04	↑	protein-cysteine N-palmitoyltransferase porcupine isoform X1 [B. taurus]
ENSP00000377575-D1	-1.30	2.75E-04	↑	basal body-orientation factor 1 isoform X1 [B. taurus]
ENSBTAP00000040134-D1	-1.30	1.13E-03	↑	hypothetical protein M91_00819, partial [B. mutus]
ENSBTAP00000013688-D1	-1.30	4.79E-03	↑	uncharacterized protein C14orf79 homolog [B. mutus]
yakG024169	-1.30	2.13E-02	↑	hypothetical protein M91_16460 [B. mutus]
ENSBTAP00000024638-D1	-1.30	2.13E-02	↑	Phakinin, partial [B. mutus]
ENSP00000382659-D1	-1.30	2.13E-02	↑	transient receptor potential cation channel subfamily V member 1 [B. mutus]
ENSP00000392637-D1	-1.30	2.13E-02	↑	CD99 antigen-like protein 2, partial [B. mutus]
ENSP00000356906-D1	-1.30	2.13E-02	↑	SH2 domain-containing protein 1B [B. taurus]
yakA16470	-1.30	2.13E-02	↑	hypothetical protein M91_21672 [B. mutus]
ENSBTAP00000039011-D1	-1.30	2.13E-02	↑	Trypsin, partial [B. mutus]
ENSP00000084795-D3	-1.30	3.47E-159	↑	60S ribosomal protein L18, partial [B. mutus]
ENSBTAP00000028661-D1	-1.29	8.55E-42	↑	PRA1 family protein 3, partial [B. mutus]
ENSP00000365019-D4	-1.29	1.51E-06	↑	spindlin-1 isoform X1 [Ovis aries]
ENSP00000408184-D1	-1.29	2.09E-24	↑	putative tetraspanin-19 isoform X1 [B. mutus]
ENSP00000419124-D1	-1.29	8.25E-13	↑	Protein FAM107A, partial [B. mutus]
ENSP00000361708-D1	-1.29	4.91E-08	↑	transcription elongation factor A protein-like 1 [Camelus ferus]
ENSBTAP00000013356-D1	-1.29	4.58E-20	↑	cysteine protease ATG4C isoform X1 [B. mutus]
ENSBTAP00000010281-D1	-1.29	1.64E-03	↑	GDP-fucose protein O-fucosyltransferase 2, partial [B. mutus]
ENSP00000340465-D1	-1.29	1.64E-03	↑	Sodium-dependent phosphate transporter 2 [B. mutus]
ENSBTAP00000006291-D1	-1.29	1.64E-03	↑	charged multivesicular body protein 4c [Ovis aries]
ENSBTAP00000009419-D1	-1.29	4.41E-80	↑	tetraspanin-31 [B. taurus]
ENSBTAP00000040465-D5	-1.29	2.42E-64	↑	protein FAM32A [Homo sapiens]
ENSBTAP00000033059-D1	-1.28	1.41E-04	↑	cyclin-dependent kinase-like 4 [B. mutus]
ENSBTAP00000045234-D1	-1.28	4.86E-150	↑	transcription elongation factor A protein-like 8 [B. taurus]
ENSBTAP00000033117-D1	-1.28	6.96E-03	↑	sodium/hydrogen exchanger 4 [B. mutus]
ENSP00000397297-D1	-1.28	6.96E-03	↑	Neurotrophin-3, partial [B. mutus]
ENSP00000377303-D1	-1.28	6.96E-03	↑	N-acylglucosamine 2-epimerase, partial [B. mutus]
ENSP00000408891-D1	-1.28	6.96E-03	↑	Pyruvate kinase isozymes R/L, partial [B. mutus]
ENSP00000319052-D1	-1.28	6.96E-03	↑	centrin-1 [B. taurus]
ENSBTAP00000050189-D1	-1.28	6.57E-17	↑	hypothetical protein M91_13247, partial [B. mutus]
ENSP00000354916-D1	-1.28	1.78E-05	↑	dual specificity protein phosphatase CDC14A isoform X5 [Bison bison bison]
ENSP00000384304-D1	-1.28	2.90E-88	↑	Nuclear nucleic acid-binding protein C1D, partial [B. mutus]
ENSBTAP00000008936-D7	-1.28	4.59E-22	↑	Glycine cleavage system H protein, mitochondrial, partial [B. mutus]
ENSP00000331720-D2	-1.28	6.38E-06	↑	UPF0472 protein C16orf72-like [Physeter catodon]
ENSP00000352833-D1	-1.27	8.15E-11	↑	tyrosine-protein phosphatase non-receptor type 22 [B. taurus]
ENSP00000310832-D1	-1.27	2.38E-03	↑	cathepsin F isoform X1 [B. mutus]
ENSBTAP00000000573-D1	-1.27	2.38E-03	↑	D-beta-hydroxybutyrate dehydrogenase, mitochondrial [B. mutus]
ENSP00000297001-D1	-1.27	2.38E-03	↑	uncharacterized protein C7orf72 homolog [B. taurus]
ENSP00000399229-D1	-1.27	8.30E-04	↑	matrix-remodeling-associated protein 8 [Tursiops truncatus]
ENSBTAP00000006275-D1	-1.27	6.59E-14	↑	protein S100-B [B. taurus]
ENSBTAP00000011076-D1	-1.27	0.00E+00	↑	vacuolar protein sorting-associated protein 29 isoform X1 [Ornithorhynchus anatinus]
ENSBTAP00000029060-D1	-1.27	2.02E-26	↑	migration and invasion enhancer 1 [B. taurus]

ENSP00000398064-D7	-1.26	2.12E-150	↑	HIG1 domain family member 1A, partial [B. mutus]
ENSBTAP00000029147-D1	-1.26	8.64E-30	↑	thioredoxin domain-containing protein 17 [B. taurus]
ENSP00000370557-D1	-1.26	1.10E-36	↑	protein MIS12 homolog [B. taurus]
ENSBTAP00000042058-D1	-1.26	3.28E-15	↑	Sorting nexin-7, partial [B. mutus]
ENSBTAP00000044190-D45	-1.26	1.68E-06	↑	hypothetical protein M91_15880, partial [B. mutus]
ENSBTAP00000013956-D1	-1.26	1.68E-06	↑	leucine-rich single-pass membrane protein 1 [B. mutus]
ENSBTAP00000012693-D1	-1.26	0.00E+00	↑	la-related protein 7 isoform X2 [Bison bison bison]
ENSBTAP00000004418-D2	-1.26	0.00E+00	↑	splicing factor 3B subunit 6 [Homo sapiens]
ENSBTAP00000018681-D1	-1.26	1.19E-03	↑	U6 snRNA-associated Sm-like protein LSm6-like [B. mutus]
ENSP00000248598-D1	-1.26	3.46E-03	↑	fibroleukin [Bison bison bison]
yakG005699	-1.26	3.46E-03	↑	E3 ubiquitin-protein ligase RNF149 [B. mutus]
yakG038008	-1.26	3.46E-03	↑	Cytosolic beta-glucosidase [B. mutus]
ENSBTAP00000018207-D1	-1.26	3.46E-03	↑	zinc finger protein 296 [B. mutus]
ENSP00000223366-D1	-1.26	1.02E-02	↑	Glucokinase, partial [B. mutus]
ENSBTAP00000013662-D1	-1.26	1.02E-02	↑	Parkinson disease 7 domain-containing protein 1, partial [B. mutus]
ENSBTAP00000032393-D1	-1.26	1.02E-02	↑	Liver carboxylesterase, partial [B. mutus]
ENSBTAP00000053531-D8	-1.26	1.02E-02	↑	Ephrin type-A receptor 5, partial [B. mutus]
yakG040511	-1.26	1.02E-02	↑	ras-related protein Rab-27A-like [B. mutus]
ENSBTAP00000020034-D1	-1.26	1.02E-02	↑	DNA-directed RNA polymerase, mitochondrial [B. mutus]
yakG044571	-1.26	3.25E-02	↑	hypothetical protein M91_15201 [B. mutus]
ENSP00000404456-D1	-1.26	3.25E-02	↑	MAP7 domain-containing protein 1-like [Bubalus bubalis]
ENSP00000354612-D1	-1.26	3.25E-02	↑	Prostaglandin G/H synthase 1, partial [B. mutus]
ENSBTAP00000004485-D1	-1.26	3.25E-02	↑	Cyclic AMP-dependent transcription factor ATF-5 [B. mutus]
ENSP00000332706-D1	-1.26	3.25E-02	↑	transcriptional activator protein Pur-alpha isoform X3 [Sus scrofa]
ENSBTAP00000045382-D1	-1.26	3.25E-02	↑	agouti-signaling protein-like [B. mutus]
ENSP00000040584-D1	-1.26	3.25E-02	↑	homeobox protein Hox-C8 [Trichechus manatus latirostris]
ENSBTAP00000045355-D1	-1.26	3.25E-02	↑	fragile X mental retardation 1 neighbor protein [B. mutus]
ENSBTAP00000051011-D15	-1.26	3.25E-02	↑	histone H2B type 1-N-like [B. mutus]
ENSP00000377567-D3	-1.26	3.25E-02	↑	keratin, type I microfibrillar, 47.6 kDa-like [B. mutus]
ENSBTAP00000022716-D1	-1.26	1.68E-11	↑	N(4)-(beta-N-acetylglucosaminyl)-L-asparaginase, partial [B. mutus]
ENSBTAP00000043604-D1	-1.25	1.15E-28	↑	neuroendocrine protein 7B2 isoform X1 [B. taurus]
ENSP00000392892-D1	-1.25	0.00E+00	↑	SAP domain-containing ribonucleoprotein, partial [B. mutus]
ENSBTAP00000016993-D2	-1.25	0.00E+00	↑	cytochrome c oxidase subunit 6A1, mitochondrial precursor [B. taurus]
ENSP00000369014-D1	-1.25	1.60E-07	↑	protein NDNF precursor [B. taurus]
ENSP00000386563-D1	-1.25	2.40E-11	↑	MORN repeat-containing protein 2 isoform X1 [Physeter catodon]
ENSBTAP00000051420-D1	-1.25	9.61E-06	↑	Transmembrane emp24 domain-containing protein 9, partial [B. mutus]
ENSBTAP00000027226-D1	-1.25	5.87E-13	↑	Mediator of RNA polymerase II transcription subunit 29, partial [B. mutus]
ENSP00000268668-D1	-1.25	7.12E-89	↑	NADH dehydrogenase [ubiquinone] 1 beta subcomplex subunit 10 [B. taurus]
ENSBTAP00000021223-D1	-1.25	8.65E-145	↑	methylmalonic aciduria and homocystinuria type D homolog, mitochondrial-like [B. mutus]
ENSP00000341094-D2	-1.25	2.14E-04	↑	histone H2A type 1-like [Cavia porcellus]
ENSBTAP00000048272-D1	-1.25	0.00E+00	↑	Protein FAM133B, partial [B. mutus]
ENSBTAP00000045674-D2	-1.25	7.63E-51	↑	Small EDRK-rich factor 1, partial [B. mutus]
ENSP00000285141-D1	-1.25	4.93E-06	↑	Protein FAM38B [B. mutus]
ENSP00000364107-D2	-1.25	2.23E-13	↑	Putative G antigen family D member 1, partial [B. mutus]
ENSP00000256429-D1	-1.25	2.23E-13	↑	Methyl-CpG-binding domain protein 2, partial [B. mutus]
ENSBTAP00000000683-D1	-1.24	0.00E+00	↑	Cystatin-B, partial [B. mutus]
ENSBTAP00000005810-D1	-1.24	1.74E-03	↑	Chordin-like protein 1, partial [B. mutus]
ENSBTAP00000053328-D1	-1.24	1.74E-03	↑	protoporphyrinogen oxidase [B. mutus]
ENSBTAP00000050535-D1	-1.24	1.74E-03	↑	ADP-ribosylation factor-like protein 10 [B. mutus]
ENSP00000202816-D1	-1.24	3.25E-250	↑	ESF1 homolog [B. mutus]
ENSBTAP00000046387-D4	-1.24	4.84E-84	↑	hypothetical protein M91_21441, partial [B. mutus]
ENSBTAP00000017146-D1	-1.24	8.18E-21	↑	40S ribosomal protein S27-like protein, partial [B. mutus]
ENSBTAP00000008804-D1	-1.24	1.95E-30	↑	metaxin-2 [B. taurus]
ENSP00000233607-D1	-1.24	3.07E-04	↑	adenomatous polyposis coli protein 2 [B. mutus]
ENSBTAP00000011009-D1	-1.24	2.85E-16	↑	serum paraoxonase/arylesterase 2 [B. mutus]

ENSBTAP00000000013-D1	-1.24	2.73E-24	↑	tetratricopeptide repeat protein 33 [B. taurus]
ENSBTAP00000025852-D1	-1.24	2.54E-12	↑	Interleukin-10 receptor subunit beta, partial [B. mutus]
ENSBTAP00000031029-D1	-1.24	6.30E-28	↑	Interferon-induced protein 44-like protein [B. mutus]
ENSP00000304108-D1	-1.23	8.73E-04	↑	protein FAM171B [Bison bison bison]
ENSBTAP00000002914-D1	-1.23	8.73E-04	↑	Apolipoprotein A-I, partial [B. mutus]
ENSBTAP00000043281-D1	-1.23	5.22E-247	↑	Selenoprotein K, partial [B. mutus]
ENSP00000249356-D1	-1.23	2.48E-20	↑	dnaJ homolog subfamily B member 9 precursor [B. taurus]
ENSP00000386893-D2	-1.23	4.25E-16	↑	peptidyl-prolyl cis-trans isomerase-like 3 [Elephantulus edwardii]
ENSBTAP00000051924-D7	-1.23	1.10E-70	↑	histone H3.3-like [B. mutus]
ENSP00000257899-D1	-1.23	1.49E-25	↑	biogenesis of lysosome-related organelles complex 1 subunit 1-like isoform X7 [Macaca fascicularis]
ENSP00000358541-D1	-1.23	3.23E-39	↑	suppressor of IKBKE 1 isoform X1 [B. taurus]
ENSP00000383392-D1	-1.23	2.65E-06	↑	neural cell adhesion molecule 2-like isoform X1 [B. mutus]
ENSP00000311224-D5	-1.23	7.95E-05	↑	bile acid-CoA:amino acid N-acyltransferase [Pantholops hodgsonii]
ENSP00000360035-D1	-1.23	2.51E-03	↑	Protein phosphatase 1 regulatory subunit 3D, partial [B. mutus]
ENSP00000361459-D1	-1.23	2.51E-03	↑	TSC22 domain family protein 3 isoform X1 [Condylura cristata]
yakG022852	-1.23	1.51E-02	↑	hypothetical protein M91_06050 [B. mutus]
yakG017263	-1.23	1.51E-02	↑	Leukemia NUP98 fusion partner 1 [B. mutus]
ENSBTAP00000031700-D1	-1.22	2.77E-12	↑	60S ribosomal protein L28 [B. taurus]
ENSBTAP00000007067-D1	-1.22	3.95E-12	↑	disks large-associated protein 1 isoform X4 [B. taurus]
ENSBTAP00000028638-D1	-1.22	3.56E-07	↑	uncharacterized protein C1orf54 homolog precursor [B. taurus]
ENSP00000259470-D2	-1.22	8.95E-54	↑	cathepsin L1-like isoform X1 [B. mutus]
ENSP00000355557-D2	-1.22	7.93E-205	↑	Chain A, Crystal Structure Of Human Neutrophil Peptide 2, Hnp-2 (variant Gly16- >gi|75765495|pdb|1XHM|B Chain B, The Crystal Structure Of A Biologically Active Peptide (Sigk) Bound To A G Protein Beta:gamma Heterodimer
ENSBTAP00000025719-D1	-1.22	1.01E-13	↑	platelet-activating factor acetylhydrolase [B. mutus]
ENSP00000418194-D1	-1.22	9.82E-24	↑	poly [ADP-ribose] polymerase 14 [B. mutus]
ENSBTAP00000027308-D4	-1.22	2.50E-16	↑	histone H2B type 1-like [B. mutus]
ENSBTAP00000053277-D1	-1.22	0.00E+00	↑	centromere protein K isoform X1 [Bubalus bubalis]
ENSP00000215909-D1	-1.22	4.81E-104	↑	Galectin-1, partial [B. mutus]
ENSBTAP00000036391-D1	-1.22	3.44E-17	↑	uncharacterized protein C19orf60 homolog isoform X1 [B. mutus]
ENSBTAP00000010554-D1	-1.22	6.37E-04	↑	oxytocin-neurophysin 1 [B. mutus]
ENSBTAP00000002339-D1	-1.22	7.34E-03	↑	hydroxylysine kinase [B. mutus]
ENSBTAP00000040365-D1	-1.22	7.34E-03	↑	hypothetical protein M91_04484, partial [B. mutus]
ENSP00000264218-D1	-1.22	7.34E-03	↑	Neuromedin-U, partial [B. mutus]
ENSBTAP00000047439-D1	-1.21	0.00E+00	↑	thyroid transcription factor 1-associated protein 26-like [B. mutus]
ENSP00000403400-D1	-1.21	3.87E-96	↑	cell cycle progression protein 1 isoform X1 [B. mutus]
ENSBTAP00000005323-D1	-1.21	4.07E-251	↑	Isopentenyl-diphosphate Delta-isomerase 1 [B. mutus]
ENSP00000357431-D1	-1.21	1.69E-10	↑	cAMP-dependent protein kinase inhibitor beta, partial [B. mutus]
ENSP00000262265-D1	-1.21	1.54E-24	↑	PIH1 domain-containing protein 1 [B. mutus]
ENSP00000415464-D1	-1.21	4.07E-60	↑	la-related protein 4 [B. taurus]
ENSBTAP00000012485-D1	-1.21	1.14E-12	↑	transmembrane protein 223 [B. mutus]
ENSP00000257262-D1	-1.21	4.00E-97	↑	putative nef1 variant 1 [Taeniopygia guttata]
ENSP00000350667-D1	-1.21	3.12E-61	↑	tropomyosin alpha-1 chain isoform X17 [Ovis aries]
ENSP00000356541-D1	-1.21	4.30E-62	↑	splicing factor 3B subunit 5 [Homo sapiens]
ENSBTAP00000022514-D1	-1.21	8.31E-05	↑	heterogeneous nuclear ribonucleoprotein A0 [B. taurus]
ENSP00000264263-D1	-1.21	2.08E-28	↑	elongation factor G, mitochondrial isoform X1 [B. mutus]
ENSP00000222008-D1	-1.21	9.17E-35	↑	prenylated Rab acceptor protein 1 [B. taurus]
ENSBTAP00000028718-D1	-1.21	6.28E-24	↑	nucleolar protein 7 [B. taurus]
ENSBTAP00000008319-D1	-1.21	6.17E-261	↑	ATPase inhibitor, mitochondrial isoform X1 [B. mutus]
ENSBTAP00000010180-D1	-1.20	2.15E-05	↑	pseudouridylate synthase 7 homolog [B. mutus]
ENSBTAP00000022876-D1	-1.20	0.00E+00	↑	LOC100138230 protein [B. taurus]
ENSP00000359098-D5	-1.20	6.20E-159	↑	cytochrome c oxidase subunit 7A2, mitochondrial [Ailuropoda melanoleuca]
ENSP00000339723-D1	-1.20	0.00E+00	↑	hypothetical protein PANDA_008997, partial [Ailuropoda melanoleuca]
ENSBTAP00000029018-D1	-1.20	1.20E-70	↑	cullin-3 [Vicugna pacos]
ENSBTAP00000026841-D1	-1.20	1.98E-184	↑	prefoldin subunit 2 [Homo sapiens]

ENSBTAP00000014520-D3	-1.20	1.54E-103	↑	Single-stranded DNA-binding protein, mitochondrial, partial [B. mutus]
ENSBTAP00000052626-D8	-1.20	3.20E-28	↑	60S ribosomal protein L30-like protein [Cricetulus griseus]
ENSBTAP00000021534-D1	-1.20	5.07E-09	↑	fibrinogen-like protein 1 isoform X1 [B. mutus]
ENSBTAP00000002055-D4	-1.20	0.00E+00	↑	Chain A, Crystal Structure Of Calmodulin Complexed With A Peptide
ENSBTAP00000011448-D1	-1.20	1.19E-04	↑	Solute carrier family 46 member 3, partial [B. mutus]
ENSBTAP00000019351-D2	-1.20	7.24E-197	↑	nucleolar RNA helicase 2 [Bison bison bison]
ENSP00000368931-D1	-1.20	4.77E-29	↑	Coiled-coil domain-containing protein 112, partial [B. mutus]
ENSP00000263245-D1	-1.20	7.22E-09	↑	ADP-ribosylation factor GTPase-activating protein 3 isoform X1 [B. mutus]
ENSP00000410221-D1	-1.19	8.04E-06	↑	Stimulated by retinoic acid 6 protein-like protein, partial [B. mutus]
ENSP00000349156-D1	-1.19	4.68E-152	↑	E3 ubiquitin-protein ligase SIAH1 [B. taurus]
ENSBTAP00000023306-D1	-1.19	4.54E-121	↑	plasminogen receptor (KT) [B. mutus]
ENSBTAP00000023098-D1	-1.19	2.44E-56	↑	Serine beta-lactamase-like protein LACTB, mitochondrial, partial [B. mutus]
ENSBTAP00000005590-D1	-1.19	5.33E-28	↑	trafficking protein particle complex subunit 5 [Pan troglodytes]
ENSBTAP00000048503-D1	-1.19	6.44E-16	↑	pejvakin [B. mutus]
ENSBTAP00000002597-D2	-1.19	2.44E-62	↑	uncharacterized protein C12orf31 homolog [B. mutus]
ENSBTAP00000042277-D1	-1.19	8.65E-05	↑	Transmembrane and immunoglobulin domain-containing protein 2 [B. mutus]
ENSP00000384264-D1	-1.19	3.35E-04	↑	cGMP-gated cation channel alpha-1, partial [B. mutus]
ENSBTAP00000041190-D1	-1.19	1.31E-03	↑	Coiled-coil-helix-coiled-coil-helix domain-containing protein 6, partial [B. mutus]
ENSP00000361982-D1	-1.19	2.62E-03	↑	probable tRNA pseudouridine synthase 2 [B. mutus]
ENSBTAP00000002024-D1	-1.19	2.62E-03	↑	alpha-1,3-mannosyl-glycoprotein 2-beta-N-acetylglucosaminyltransferase isoform X1 [Pantholops hodgsonii]
ENSBTAP00000046769-D1	-1.19	5.28E-03	↑	synaptonemal complex central element protein 1 [B. mutus]
ENSP00000382390-D1	-1.19	5.28E-03	↑	Neuralized-like protein 4, partial [B. mutus]
ENSP00000376910-D2	-1.19	1.07E-02	↑	leucine-rich repeat and IQ domain-containing protein 1-like, partial [B. mutus]
ENSP00000401258-D1	-1.19	1.07E-02	↑	Beta-soluble NSF attachment protein [B. mutus]
ENSP00000418668-D1	-1.19	1.07E-02	↑	muscle-related coiled-coil protein [B. mutus]
ENSBTAP00000053140-D1	-1.19	1.07E-02	↑	60S ribosomal protein L36a-like [B. mutus]
ENSBTAP00000013784-D1	-1.19	2.24E-02	↑	Deoxyribonuclease-2-beta, partial [B. mutus]
ENSBTAP00000050520-D73	-1.19	2.24E-02	↑	hypothetical protein M91_07727, partial [B. mutus]
ENSBTAP00000007819-D1	-1.19	2.24E-02	↑	protein-tyrosine kinase 2-beta isoform X1 [B. mutus]
ENSBTAP00000005939-D1	-1.19	1.57E-26	↑	U6 snRNA-associated Sm-like protein LSm7 [Lipotes vexillifer]
ENSBTAP00000038763-D5	-1.19	3.26E-20	↑	60S ribosomal protein L12, partial [B. mutus]
ENSP00000403117-D1	-1.18	5.54E-171	↑	Major facilitator superfamily domain-containing protein 1, partial [B. mutus]
ENSBTAP00000023407-D1	-1.18	2.81E-38	↑	Ras-related protein Rab-13, partial [B. mutus]
ENSBTAP00000007036-D6	-1.18	6.65E-109	↑	ORF [B. taurus]
ENSP00000261180-D1	-1.18	4.28E-31	↑	thyrotropin-releasing hormone-degrading ectoenzyme [Bison bison bison]
ENSBTAP00000018595-D1	-1.18	7.47E-45	↑	acylphosphatase-1 isoform X1 [Bison bison bison]
ENSP00000288937-D1	-1.18	3.29E-27	↑	39S ribosomal protein L17, mitochondrial precursor [B. taurus]
ENSBTAP00000053852-D1	-1.18	4.28E-06	↑	platelet-derived growth factor C isoform X1 [Bubalus bubalis]
ENSBTAP00000019969-D1	-1.18	1.90E-55	↑	mCG22814 [Mus musculus]
ENSBTAP00000022090-D1	-1.18	3.55E-296	↑	polycomb group RING finger protein 1 [Ovis aries]
ENSP00000366845-D3	-1.18	9.96E-68	↑	TPA: COMM domain-containing protein 6-like [B. taurus]
ENSP00000362863-D1	-1.18	6.43E-35	↑	Receptor expression-enhancing protein 3, partial [B. mutus]
ENSP00000269290-D1	-1.18	4.00E-10	↑	hypothetical protein M91_06864, partial [B. mutus]
ENSP00000296597-D1	-1.17	6.43E-53	↑	mimitin, mitochondrial [B. mutus]
ENSBTAP00000018493-D1	-1.17	1.31E-105	↑	glutaminyl-peptide cyclotransferase [B. mutus]
ENSP00000415246-D1	-1.17	1.85E-116	↑	E3 ubiquitin-protein ligase RNF181 isoform X1 [Macaca nemestrina]
ENSP00000348314-D1	-1.17	1.57E-07	↑	Sperm flagellar protein 2 [B. mutus]
ENSP00000350576-D49	-1.17	9.50E-04	↑	Zinc finger protein 709, partial [B. mutus]
ENSP00000358554-D1	-1.17	0.00E+00	↑	pre-mRNA-splicing factor SPF27 [Felis catus]
ENSP00000331691-D1	-1.17	1.89E-03	↑	uncharacterized protein C12orf60 homolog [B. taurus]
ENSP00000400843-D1	-1.17	1.89E-03	↑	cytosolic phospholipase A2 zeta [B. mutus]
ENSBTAP00000052478-D1	-1.17	1.34E-98	↑	40S ribosomal protein S16-like isoform X1 [B. mutus]
ENSBTAP00000016768-D1	-1.17	3.90E-73	↑	NADH dehydrogenase [ubiquinone] 1 beta subcomplex subunit 7 [B. taurus]
ENSBTAP00000007391-D1	-1.17	8.95E-05	↑	lipopolysaccharide-induced tumor necrosis factor-alpha factor isoform X1 [Bison bison bison]
ENSBTAP00000052443-D1	-1.17	8.13E-74	↑	vacuole membrane protein 1 [B. taurus]

ENSBTAP00000025136-D1	-1.17	1.12E-32	↑	dynactin subunit 3 [B. taurus]
ENSBTAP00000021643-D2	-1.17	0.00E+00	↑	Small nuclear ribonucleoprotein F, partial [B. mutus]
ENSBTAP00000051891-D1	-1.17	4.28E-233	↑	NADH dehydrogenase [ubiquinone] 1 subunit C1, mitochondrial precursor [B. taurus]
ENSBTAP00000048042-D2	-1.17	1.63E-66	↑	60S acidic ribosomal protein P2, partial [B. mutus]
ENSBTAP00000016536-D1	-1.17	1.76E-04	↑	fibrous sheath-interacting protein 2-like isoform X2 [Ovis aries musimon]
ENSBTAP00000017531-D1	-1.17	3.78E-03	↑	neuronal-specific septin-3 isoform X1 [B. taurus]
ENSP00000357927-D1	-1.17	8.49E-07	↑	bcl-2/adenovirus E1B 19 kDa-interacting protein 2-like protein [B. mutus]
ENSBTAP00000048266-D1	-1.16	4.30E-09	↑	MRPL12 protein [B. taurus]
ENSBTAP00000020832-D1	-1.16	7.14E-128	↑	cytochrome c oxidase assembly protein COX16 homolog, mitochondrial [B. taurus]
ENSP00000418082-D6	-1.16	3.78E-106	↑	60S ribosomal protein L37a [Falco cherrug]
ENSP00000419357-D1	-1.16	3.38E-44	↑	NADH dehydrogenase [ubiquinone] 1 beta subcomplex subunit 2, mitochondrial precursor [B. taurus]
ENSBTAP00000015942-D1	-1.16	3.32E-05	↑	lemur tyrosine kinase 3 [B. mutus]
ENSP00000369055-D1	-1.16	1.18E-11	↑	Beta-1,4-galactosyltransferase 1, partial [B. mutus]
ENSBTAP00000012183-D1	-1.16	1.01E-128	↑	Protein dpy-30-like protein, partial [B. mutus]
ENSBTAP00000029075-D1	-1.16	4.65E-25	↑	alpha-methylacyl-CoA racemase [B. mutus]
ENSBTAP00000010295-D1	-1.16	4.44E-10	↑	thyroglobulin [B. mutus]
ENSBTAP00000016021-D1	-1.16	1.24E-21	↑	transmembrane protein 176A [B. mutus]
ENSBTAP00000039319-D1	-1.15	0.00E+00	↑	U2 small nuclear ribonucleoprotein B'', partial [B. mutus]
ENSBTAP00000014801-D4	-1.15	2.95E-40	↑	cytochrome c oxidase subunit NDUFA4 [B. taurus]
yakG040933	-1.15	4.57E-14	↑	DNA-directed RNA polymerases I, II, and III subunit RPABC5 [B. mutus]
ENSP00000064778-D1	-1.15	2.52E-04	↑	protein FAM168A isoform X2 [Canis lupus familiaris]
ENSP00000389998-D1	-1.15	1.69E-07	↑	meckelin [B. mutus]
ENSP00000356233-D1	-1.15	8.94E-06	↑	Lysine-specific demethylase 5B, partial [B. mutus]
ENSBTAP00000008411-D1	-1.15	1.80E-19	↑	ER membrane protein complex subunit 4 isoform X1 [Otolemur garnettii]
ENSP00000361238-D1	-1.15	1.72E-06	↑	Radial spoke head protein 9-like protein, partial [B. mutus]
ENSP00000315757-D1	-1.15	6.47E-14	↑	plastin-2 [B. mutus]
ENSP00000372221-D1	-1.15	2.13E-31	↑	Serpin B5 [B. mutus]
ENSP00000298386-D1	-1.15	4.60E-09	↑	relaxin receptor 2 isoform X1 [B. mutus]
ENSBTAP00000051312-D1	-1.15	3.34E-06	↑	3-hydroxybutyrate dehydrogenase type 2, partial [B. mutus]
yakG013051	-1.15	1.58E-02	↑	hypothetical protein M91_06530 [B. mutus]
ENSP00000274773-D15	-1.15	1.58E-02	↑	E3 ubiquitin-protein ligase TRIM31, partial [B. mutus]
ENSBTAP00000018053-D1	-1.15	6.99E-161	↑	Leptin receptor overlapping transcript-like 1, partial [B. mutus]
ENSP00000384357-D1	-1.15	1.88E-43	↑	serine/arginine-rich splicing factor 11-like, partial [Tursiops truncatus]
ENSP00000332103-D1	-1.15	5.11E-20	↑	N(alpha)-acetyltransferase 38, NatC auxiliary subunit isoform X2 [B. taurus]
ENSBTAP00000022730-D1	-1.15	1.77E-12	↑	Glutamate-rich protein 1, partial [B. mutus]
ENSP00000364000-D1	-1.15	2.44E-10	↑	collagen alpha-2(V) chain-like, partial [Leptonychotes weddellii]
ENSP00000369757-D1	-1.15	1.05E-88	↑	40S ribosomal protein S6 [B. taurus]
ENSP00000333277-D10	-1.15	4.28E-265	↑	histone H3.3 [Felis catus]
ENSBTAP00000017969-D1	-1.14	1.39E-17	↑	protein asunder homolog [B. taurus]
ENSP00000345494-D1	-1.14	7.47E-20	↑	Phospholipid scramblase 2 [B. mutus]
ENSP00000245615-D1	-1.14	6.74E-05	↑	Lysophospholipid acyltransferase 7 [B. mutus]
ENSP00000206595-D1	-1.14	1.34E-13	↑	G2/M phase-specific E3 ubiquitin-protein ligase [B. mutus]
ENSBTAP00000037054-D1	-1.14	3.60E-04	↑	Brain and acute leukemia cytoplasmic protein, partial [B. mutus]
ENSBTAP00000004892-D1	-1.14	2.05E-19	↑	Transketolase, partial [B. mutus]
ENSP00000304290-D1	-1.14	1.96E-03	↑	acetylcholine receptor subunit beta isoform X1 [B. mutus]
ENSP00000374268-D1	-1.14	1.96E-03	↑	Glucoside xylosyltransferase 2, partial [B. mutus]
ENSP00000301939-D1	-1.14	7.32E-14	↑	transmembrane protein 256 precursor [B. taurus]
ENSBTAP00000008107-D1	-1.14	1.51E-32	↑	peroxiredoxin-4 precursor [B. taurus]
ENSBTAP00000019142-D1	-1.14	2.51E-06	↑	Krueppel-like factor 10, partial [B. mutus]
ENSBTAP00000033320-D1	-1.13	3.09E-97	↑	Complement component C8 beta chain [B. mutus]
ENSBTAP00000020461-D1	-1.13	1.94E-12	↑	fructosamine-3-kinase isoform X1 [B. mutus]
ENSP00000322218-D1	-1.13	9.57E-05	↑	Membrane-associated phosphatidylinositol transfer protein 2, partial [B. mutus]
ENSBTAP00000050222-D22	-1.13	1.23E-252	↑	60S ribosomal protein L17, partial [B. mutus]
ENSP00000376910-D1	-1.13	3.53E-05	↑	Leucine-rich repeat and IQ domain-containing protein 1, partial [B. mutus]
ENSP00000376159-D1	-1.13	3.31E-152	↑	Bcl-2-associated transcription factor 1, partial [B. mutus]

ENSBTAP00000028640-D1	-1.13	1.03E-26	↑	glutathione S-transferase A4 [B. taurus]
ENSBTAP00000017155-D1	-1.13	1.29E-22	↑	heat shock 70 kDa protein 13 precursor [B. taurus]
ENSBTAP00000000519-D1	-1.13	1.42E-11	↑	vitamin K epoxide reductase complex subunit 1 isoform X1 [Camelus ferus]
ENSP00000350667-D3	-1.13	2.78E-38	↑	Tropomyosin alpha-1 chain [B. mutus]
ENSBTAP00000045387-D1	-1.13	5.13E-04	↑	desmocollin-3 isoform X1 [B. mutus]
ENSBTAP00000014749-D1	-1.13	2.37E-19	↑	Sodium-coupled neutral amino acid transporter 2, partial [B. mutus]
ENSP00000389630-D1	-1.13	1.46E-12	↑	Protein dpy-19-like protein 4, partial [B. mutus]
yakG039136	-1.13	1.40E-03	↑	Laminin subunit alpha-5 [B. mutus]
ENSBTAP00000037325-D1	-1.13	1.11E-02	↑	ATP-binding cassette sub-family A member 3, partial [B. mutus]
ENSBTAP00000038868-D82	-1.13	1.11E-02	↑	mariner transposase [Homo sapiens]
ENSBTAP00000005370-D1	-1.13	3.40E-02	↑	hypothetical protein M91_16566, partial [B. mutus]
ENSP00000335306-D1	-1.13	3.40E-02	↑	Developmental pluripotency-associated protein 4, partial [B. mutus]
ENSBTAP00000007080-D1	-1.13	3.40E-02	↑	hepatitis delta antigen interacting protein A [Homo sapiens]
ENSBTAP00000007812-D1	-1.13	3.40E-02	↑	ESX homeobox 1 [B. mutus]
ENSBTAP00000049290-D1	-1.13	3.40E-02	↑	SLIT and NTRK-like protein 1, partial [B. mutus]
yakG032601	-1.13	3.40E-02	↑	ALK tyrosine kinase receptor [B. mutus]
ENSP00000263196-D1	-1.13	3.40E-02	↑	Integral membrane protein DGCR2/IDD, partial [B. mutus]
ENSP00000228928-D2	-1.13	6.07E-17	↑	2'-5'-oligoadenylate synthase 3 [Ceratotherium simum simum]
ENSBTAP00000007030-D1	-1.12	2.00E-10	↑	tryptophan 5-hydroxylase 1 isoform X1 [B. taurus]
ENSBTAP00000049118-D1	-1.12	7.28E-78	↑	Centrin-1, partial [B. mutus]
ENSBTAP00000018626-D1	-1.12	0.00E+00	↑	proteasome maturation protein [B. taurus]
ENSP00000347852-D1	-1.12	5.08E-07	↑	Sorbin and SH3 domain-containing protein 2, partial [B. mutus]
ENSP00000284811-D2	-1.12	0.00E+00	↑	RNA polymerase II transcription factor SIII subunit C, partial [Macaca mulatta]
ENSBTAP00000019583-D1	-1.12	1.09E-11	↑	Cystatin-M [B. mutus]
ENSP00000396377-D1	-1.12	7.90E-23	↑	cAMP-regulated phosphoprotein 19 isoform 1 [B. taurus]
ENSBTAP00000033878-D2	-1.12	0.00E+00	↑	cyclin-dependent kinases regulatory subunit 2 [Condylura cristata]
ENSBTAP00000049655-D1	-1.12	2.33E-25	↑	nucleosome assembly protein 1-like 1 isoform X4 [Balaenoptera acutorostrata scammoni]
ENSBTAP00000011619-D1	-1.12	9.85E-05	↑	DNA-directed RNA polymerase III subunit RPC6 isoform X2 [Ovis aries]
ENSBTAP00000003218-D1	-1.12	9.85E-05	↑	7,8-dihydro-8-oxoguanine triphosphatase, partial [B. mutus]
ENSBTAP00000012088-D6	-1.12	5.00E-239	↑	60S ribosomal protein L7, partial [B. mutus]
ENSP00000306410-D1	-1.12	2.68E-04	↑	Armadillo repeat-containing protein 4 [B. mutus]
ENSBTAP00000024520-D1	-1.11	7.91E-03	↑	Protein pigeon-like protein, partial [B. mutus]
ENSBTAP00000011305-D5	-1.11	0.00E+00	↑	40S ribosomal protein S14, partial [B. mutus]
ENSBTAP00000005416-D1	-1.11	1.95E-07	↑	transcription regulator protein BACH1 [Bison bison bison]
yakG002790	-1.11	2.53E-118	↑	Ribosome biogenesis protein NSA2-like protein [B. mutus]
ENSBTAP00000031693-D1	-1.11	1.91E-05	↑	Zinc finger protein 322A, partial [B. mutus]
ENSBTAP00000023908-D2	-1.11	2.66E-71	↑	prostaglandin E synthase 3 isoform X1 [Mustela putorius furo]
ENSP00000262288-D1	-1.11	4.60E-14	↑	retinoid-inducible serine carboxypeptidase [B. mutus]
ENSBTAP00000050944-D4	-1.11	1.25E-152	↑	hypothetical protein M91_11864, partial [B. mutus]
ENSP00000337513-D1	-1.11	3.87E-08	↑	steroid receptor RNA activator 1 [B. mutus]
ENSP00000365625-D1	-1.11	1.40E-06	↑	putative pre-mRNA-splicing factor ATP-dependent RNA helicase DHX16 [B. mutus]
ENSBTAP00000016624-D1	-1.11	2.01E-03	↑	cytochrome c oxidase subunit 7B, mitochondrial-like [B. mutus]
ENSBTAP00000027890-D1	-1.11	8.18E-11	↑	Placenta-specific protein 9, partial [B. mutus]
ENSBTAP00000019802-D1	-1.11	5.60E-44	↑	cytochrome c oxidase assembly factor 5 [B. taurus]
ENSP00000392341-D1	-1.11	5.96E-11	↑	Endoplasmic reticulum-Golgi intermediate compartment protein 3 [B. mutus]
ENSBTAP00000003986-D1	-1.11	1.57E-44	↑	PAX3- and PAX7-binding protein 1 [B. taurus]
ENSBTAP00000018283-D1	-1.11	3.23E-29	↑	protein BEX2 isoform X1 [B. mutus]
ENSBTAP00000025572-D1	-1.10	0.00E+00	↑	Ribosome biogenesis protein BRX1-like protein, partial [B. mutus]
ENSBTAP00000052755-D1	-1.10	1.16E-27	↑	Cancer susceptibility candidate protein 1-like protein, partial [B. mutus]
ENSP00000339051-D2	-1.10	2.94E-91	↑	hypothetical protein M91_03677, partial [B. mutus]
ENSBTAP00000001886-D1	-1.10	1.84E-138	↑	Homeobox expressed in ES cells 1, partial [B. mutus]
ENSP00000393599-D1	-1.10	8.27E-35	↑	Metastasis-associated lung adenocarcinoma transcript 1, partial [B. mutus]
ENSBTAP00000023995-D1	-1.10	1.20E-35	↑	IQ domain-containing protein G [B. mutus]
ENSBTAP00000050575-D6	-1.10	1.45E-03	↑	.
ENSBTAP00000021683-D1	-1.10	5.65E-03	↑	Glycerol-3-phosphate dehydrogenase [NAD+], cytoplasmic, partial [B. mutus]

ENSP00000311257-D1	-1.10	5.65E-03	↑	storkhead-box protein 2 [B. mutus]
ENSBTAP00000007879-D1	-1.10	5.65E-03	↑	Death-inducer obliterator 1 [B. mutus]
ENSBTAP00000029148-D1	-1.10	2.32E-02	↑	mediator of RNA polymerase II transcription subunit 31 isoform X1 [Papio anubis]
ENSP00000350630-D1	-1.10	2.32E-02	↑	tolloid-like protein 2 [B. mutus]
ENSP00000189978-D1	-1.10	2.32E-02	↑	muscle, skeletal receptor tyrosine-protein kinase isoform X3 [B. mutus]
ENSBTAP00000028988-D1	-1.10	3.75E-125	↑	Annexin A5 [B. mutus]
ENSP00000358222-D4	-1.10	4.05E-47	↑	cyclin C [Capra hircus]
ENSP00000401823-D1	-1.10	3.14E-55	↑	RNA polymerase II-associated protein 3 isoform X1 [B. mutus]
ENSBTAP00000001172-D1	-1.10	1.62E-40	↑	Katanin p60 ATPase-containing subunit A1 [B. mutus]
ENSP00000381607-D2	-1.10	5.74E-46	↑	Glutathione S-transferase P, partial [B. mutus]
ENSP00000324729-D1	-1.10	4.18E-17	↑	protein salvador homolog 1 [B. taurus]
ENSBTAP00000011798-D1	-1.10	1.42E-43	↑	39S ribosomal protein L50, mitochondrial [B. mutus]
ENSBTAP00000045313-D1	-1.10	5.78E-31	↑	3-hydroxyisobutyryl-CoA hydrolase, mitochondrial [B. mutus]
ENSP00000265339-D2	-1.09	7.72E-186	↑	ubiquitin-conjugating enzyme E2 B isoform X1 [Rattus norvegicus]
ENSBTAP00000028364-D1	-1.09	2.14E-16	↑	Proteasome subunit beta type-4 [B. mutus]
ENSBTAP00000001465-D2	-1.09	1.43E-122	↑	28S ribosomal protein S14, mitochondrial [B. taurus]
ENSBTAP00000035017-D1	-1.09	5.94E-66	↑	ER membrane protein complex subunit 6 [Mus musculus]
ENSBTAP00000046644-D1	-1.09	1.47E-06	↑	1-phosphatidylinositol-4,5-bisphosphate phosphodiesterase beta-1, partial [B. mutus]
ENSBTAP00000024631-D1	-1.09	2.78E-05	↑	LisH domain-containing protein ARMC9, partial [B. mutus]
ENSBTAP00000019650-D1	-1.09	7.27E-172	↑	nudC domain-containing protein 2 [B. taurus]
ENSBTAP00000013198-D1	-1.09	2.10E-07	↑	mitogen-activated protein kinase 13 [B. taurus]
ENSP00000407834-D1	-1.09	3.23E-30	↑	serine/threonine-protein phosphatase 4 regulatory subunit 3A isoform X1 [Ovis aries]
ENSBTAP00000005162-D1	-1.09	1.63E-02	↑	Transmembrane protein 220, partial [B. mutus]
ENSBTAP00000026613-D1	-1.09	1.63E-02	↑	UMP-CMP kinase 2, mitochondrial, partial [B. mutus]
ENSBTAP00000053088-D1	-1.09	1.63E-02	↑	Histone H1x, partial [B. mutus]
ENSP00000350479-D1	-1.09	8.42E-303	↑	60S ribosomal protein L14 isoform X1 [Ovis aries]
ENSBTAP00000007718-D1	-1.09	0.00E+00	↑	Sentrin-specific protease 6, partial [B. mutus]
ENSP00000272444-D1	-1.09	5.37E-52	↑	RNA/RNP complex-1-interacting phosphatase, partial [B. mutus]
yakG029799	-1.09	1.78E-43	↑	hypothetical protein M91_15455 [B. mutus]
ENSBTAP00000004835-D1	-1.09	2.52E-11	↑	Sjoegren syndrome/scleroderma autoantigen 1 [Capra hircus]
ENSBTAP00000002462-D1	-1.09	1.83E-135	↑	hypothetical protein M91_08640, partial [B. mutus]
yakG036397	-1.08	1.24E-105	↑	Dolichyl-diphosphooligosaccharide--protein glycosyltransferase subunit 4 [B. mutus]
ENSP00000221852-D1	-1.08	1.06E-05	↑	SH2 domain-containing adapter protein D [Pantholops hodgsonii]
ENSBTAP00000012484-D1	-1.08	2.95E-07	↑	transmembrane protein 179B [B. mutus]
ENSBTAP00000034848-D1	-1.08	2.51E-60	↑	Chromodomain-helicase-DNA-binding protein 1, partial [B. mutus]
ENSBTAP00000014112-D1	-1.08	7.65E-06	↑	gem-associated protein 7 isoform X1 [B. taurus]
ENSBTAP00000029284-D1	-1.08	0.00E+00	↑	Activated RNA polymerase II transcriptional coactivator p15, partial [B. mutus]
ENSBTAP00000025760-D1	-1.08	3.23E-188	↑	nuclear speckle splicing regulatory protein 1 [B. mutus]
ENSP00000376956-D1	-1.08	3.36E-40	↑	39S ribosomal protein L27, mitochondrial, partial [B. mutus]
ENSP00000388311-D78	-1.08	4.36E-15	↑	hypothetical protein M91_15016, partial [B. mutus]
ENSBTAP00000050194-D85	-1.08	2.83E-90	↑	hypothetical protein M91_06506 [B. mutus]
ENSBTAP00000009564-D1	-1.08	2.03E-04	↑	serotransferrin [Bison bison bison]
ENSP00000347620-D1	-1.08	1.48E-03	↑	Transmembrane protein 116, partial [B. mutus]
ENSBTAP00000011391-D1	-1.08	1.14E-02	↑	Putative E3 ubiquitin-protein ligase TRIML2, partial [B. mutus]
ENSBTAP00000053813-D1	-1.08	8.68E-09	↑	hypothetical protein M91_18338, partial [B. mutus]
ENSBTAP00000040527-D2	-1.08	2.16E-66	↑	Beta-2-microglobulin [B. mutus]
ENSBTAP00000023996-D1	-1.08	4.35E-08	↑	lanC-like protein 2 [B. mutus]
ENSP00000378079-D1	-1.08	1.54E-19	↑	Zinc finger protein 644, partial [B. mutus]
ENSBTAP00000016524-D1	-1.07	1.07E-03	↑	Disintegrin and metalloproteinase domain-containing protein 12, partial [B. mutus]
ENSBTAP00000028556-D1	-1.07	2.72E-40	↑	translocon-associated protein subunit delta isoform X1 [Ovis aries]
ENSP00000308021-D1	-1.07	1.09E-187	↑	centrosomal protein of 290 kDa isoform X5 [B. taurus]
ENSP00000420448-D1	-1.07	2.86E-18	↑	zinc finger protein 148 isoform X1 [Orcinus orca]
ENSBTAP00000000576-D1	-1.07	7.15E-17	↑	transmembrane protein 126A [B. mutus]
ENSBTAP00000016718-D4	-1.07	5.76E-13	↑	ribonuclease UK114 [B. mutus]
ENSP00000358210-D1	-1.07	5.67E-06	↑	single-minded homolog 1 [B. mutus]

ENSP00000366915-D1	-1.07	5.53E-05	↑	Progesterone-induced-blocking factor 1 [Pteropus alecto]
ENSP00000356632-D1	-1.07	1.29E-234	↑	costars family protein ABRACL [B. taurus]
ENSP00000296734-D1	-1.07	1.11E-133	↑	probable glutathione peroxidase 8 [B. mutus]
ENSP00000340568-D1	-1.07	2.98E-06	↑	Cytochrome c oxidase subunit 8C, mitochondrial, partial [B. mutus]
ENSBTAP00000027443-D1	-1.07	2.90E-05	↑	OCIA domain-containing protein 2 [B. taurus]
ENSBTAP00000010601-D1	-1.07	3.99E-04	↑	muscarinic acetylcholine receptor M3-like [B. mutus]
ENSBTAP00000024412-D1	-1.07	5.76E-03	↑	interleukin-33 [B. mutus]
yakG025493	-1.06	1.93E-21	↑	hypothetical protein M91_12911 [B. mutus]
ENSBTAP00000022463-D1	-1.06	4.20E-28	↑	transcription initiation factor TFIID subunit 11 [B. taurus]
ENSP00000405133-D1	-1.06	4.11E-03	↑	ATP-binding cassette sub-family A member 12 [B. mutus]
ENSP00000210633-D1	-1.06	4.11E-03	↑	Semaphorin-4G [B. mutus]
ENSP00000363894-D1	-1.06	1.33E-09	↑	protein NipSnap homolog 3A [B. mutus]
ENSBTAP00000050194-D37	-1.06	7.82E-05	↑	hypothetical protein M91_11778 [B. mutus]
ENSP00000356520-D1	-1.06	1.96E-30	↑	ATP-dependent RNA helicase A isoform X1 [Bison bison bison]
ENSP00000309772-D1	-1.06	1.36E-09	↑	cadherin-like and PC-esterase domain-containing protein 1 [B. mutus]
ENSBTAP00000019133-D1	-1.06	2.10E-03	↑	Natural resistance-associated macrophage protein 2, partial [B. mutus]
ENSP00000311010-D1	-1.06	2.10E-03	↑	ETS-related transcription factor Elf-5, partial [B. mutus]
ENSP00000350415-D1	-1.06	2.10E-03	↑	gap junction alpha-9 protein [B. mutus]
ENSBTAP00000036999-D1	-1.06	1.52E-16	↑	Copine-3 [B. mutus]
ENSBTAP00000050243-D2	-1.06	1.29E-08	↑	hypothetical protein M91_05021, partial [B. mutus]
ENSBTAP00000019041-D1	-1.06	6.09E-07	↑	Acetyl-coenzyme A transporter 1, partial [B. mutus]
ENSP00000202677-D1	-1.06	1.51E-03	↑	Ral GTPase-activating protein subunit alpha-2, partial [B. mutus]
ENSP00000301178-D1	-1.05	1.09E-03	↑	Tyrosine-protein kinase receptor UFO, partial [B. mutus]
ENSP00000282406-D1	-1.05	1.09E-03	↑	pleckstrin homology domain-containing family H member 2, partial [B. mutus]
ENSBTAP00000051492-D13	-1.05	0.00E+00	↑	histone H3.3 [Felis catus]
ENSBTAP00000049230-D5	-1.05	8.90E-23	↑	TPA: ribosomal protein L27a-like [B. taurus]
ENSP00000272570-D1	-1.05	2.62E-71	↑	Zinc finger CCCH domain-containing protein 8 [B. mutus]
ENSP00000404826-D1	-1.05	7.80E-04	↑	hypothetical protein M91_21383, partial [B. mutus]
ENSBTAP00000011393-D1	-1.05	7.80E-04	↑	Putative E3 ubiquitin-protein ligase TRIML1, partial [B. mutus]
ENSBTAP00000000790-D1	-1.05	2.73E-29	↑	cystatin-C-like [Bubalus bubalis]
ENSP00000372857-D1	-1.05	8.25E-12	↑	Serine/threonine-protein kinase Nek11, partial [B. mutus]
ENSBTAP00000045864-D1	-1.05	5.89E-06	↑	uncharacterized protein LOC100126544 [B. taurus]
ENSBTAP00000025615-D2	-1.05	0.00E+00	↑	hypothetical protein M91_12348, partial [B. mutus]
ENSP00000351737-D1	-1.05	3.94E-96	↑	transmembrane protein 126B [B. mutus]
ENSBTAP00000001045-D1	-1.05	1.89E-14	↑	protein phosphatase 1 regulatory subunit 7 [B. mutus]
ENSP00000298705-D1	-1.05	2.92E-04	↑	hypothetical protein M91_07555, partial [B. mutus]
ENSP00000384372-D1	-1.05	2.91E-43	↑	Microsomal glutathione S-transferase 3, partial [B. mutus]
ENSBTAP00000027713-D3	-1.05	5.27E-33	↑	40S ribosomal protein S21 isoform X2 [Loxodonta africana]
ENSBTAP00000027627-D2	-1.05	2.58E-41	↑	40S ribosomal protein S15a, partial [B. mutus]
ENSBTAP00000023197-D1	-1.05	2.85E-10	↑	Disintegrin and metalloproteinase domain-containing protein 9 [B. mutus]
ENSP00000379888-D1	-1.05	1.99E-92	↑	40S ribosomal protein S8 [Homo sapiens]
ENSBTAP00000041817-D1	-1.05	6.59E-08	↑	Selenoprotein M [B. mutus]
ENSBTAP00000007254-D1	-1.05	0.00E+00	↑	Nucleolar protein 58, partial [B. mutus]
ENSBTAP00000004555-D1	-1.04	4.24E-47	↑	UPF0547 protein C16orf87 homolog [B. taurus]
ENSBTAP00000006227-D1	-1.04	1.27E-07	↑	runt-related transcription factor 2 isoform X3 [Bison bison bison]
ENSP00000324948-D1	-1.04	2.95E-10	↑	transcription factor SOX-6 isoform X3 [B. taurus]
ENSBTAP00000045096-D1	-1.04	1.53E-66	↑	translocon-associated protein subunit beta isoform X1 [B. taurus]
ENSBTAP00000007436-D1	-1.04	1.46E-22	↑	26S proteasome non-ATPase regulatory subunit 2, partial [B. mutus]
ENSBTAP00000004433-D1	-1.04	1.60E-05	↑	phosphorylase b kinase gamma catalytic chain, liver/testis isoform [B. taurus]
ENSP00000379301-D1	-1.04	4.14E-04	↑	GTPase KRas isoform X1 [Ovis aries]
ENSP00000357844-D1	-1.04	5.74E-04	↑	Monocarboxylate transporter 10, partial [B. mutus]
ENSBTAP00000002176-D1	-1.04	1.11E-03	↑	Peptide deformylase, mitochondrial, partial [B. mutus]
ENSP00000384667-D1	-1.04	4.19E-03	↑	UPF0193 protein EVG1 isoform X1 [B. mutus]
ENSBTAP00000004192-D1	-1.04	1.66E-02	↑	Ras-related protein Rab-7L1, partial [B. mutus]
ENSBTAP00000034061-D1	-1.04	2.38E-02	↑	kynurenine 3-monooxygenase [B. mutus]

ENSP00000377372-D1	-1.04	2.38E-02	↑	Neuromodulin, partial [B. mutus]
ENSP00000216445-D1	-1.04	2.38E-02	↑	uncharacterized protein C14orf105 homolog isoform X1 [B. mutus]
ENSBTAP00000024705-D1	-1.04	2.38E-02	↑	Zinc finger FYVE domain-containing protein 27 [B. mutus]
ENSBTAP00000040470-D1	-1.04	3.49E-02	↑	Collagen alpha-1(V) chain, partial [B. mutus]
yakA15909	-1.04	3.49E-02	↑	hypothetical protein M91_21668 [B. mutus]
ENSBTAP00000048832-D1	-1.04	3.49E-02	↑	hypothetical protein M91_21269, partial [B. mutus]
ENSBTAP00000002617-D7	-1.04	3.49E-02	↑	hypothetical protein M91_09584, partial [B. mutus]
ENSP00000371085-D1	-1.04	3.49E-02	↑	brain-specific angiogenesis inhibitor 1-associated protein 2-like protein 2 [B. mutus]
ENSBTAP00000011949-D1	-1.04	3.49E-02	↑	Mitogen-activated protein kinase kinase kinase 5, partial [B. mutus]
ENSP00000415032-D1	-1.03	5.70E-112	↑	General transcription factor IIH subunit 5 [B. mutus]
ENSBTAP00000035234-D1	-1.03	1.99E-13	↑	hypothetical protein M91_15734, partial [B. mutus]
ENSBTAP00000044670-D1	-1.03	2.17E-72	↑	NADH dehydrogenase [ubiquinone] iron-sulfur protein 4, mitochondrial precursor [B. taurus]
ENSBTAP00000018489-D1	-1.03	2.89E-59	↑	Motile sperm domain-containing protein 1, partial [B. mutus]
ENSBTAP00000033879-D1	-1.03	6.81E-21	↑	Coiled-coil domain-containing protein 90B, mitochondrial, partial [B. mutus]
ENSP00000415939-D1	-1.03	4.50E-11	↑	Zinc finger BED domain-containing protein 5 [B. mutus]
ENSBTAP00000053264-D1	-1.03	5.75E-10	↑	Voltage-dependent calcium channel subunit alpha-2/delta-1, partial [B. mutus]
ENSP00000387180-D1	-1.03	1.42E-17	↑	Peptidyl-tRNA hydrolase 2, mitochondrial, partial [B. mutus]
ENSBTAP00000024326-D1	-1.03	3.13E-216	↑	ATP synthase subunit O, mitochondrial [B. mutus]
ENSBTAP00000031873-D1	-1.03	1.44E-17	↑	fas apoptotic inhibitory molecule 1 [B. taurus]
ENSP00000305230-D1	-1.03	2.83E-70	↑	signal recognition particle 9kDa [Canis lupus familiaris]
ENSBTAP00000002406-D1	-1.03	0.00E+00	↑	Glutathione S-transferase Mu 5 [B. mutus]
ENSP00000286424-D1	-1.03	6.24E-92	↑	Transmembrane BAX inhibitor motif-containing protein 4 [B. mutus]
ENSBTAP00000041678-D1	-1.03	8.34E-19	↑	LYR motif-containing protein 7, partial [B. mutus]
ENSBTAP00000007399-D1	-1.02	3.69E-08	↑	CAMPATH-1 antigen precursor [B. taurus]
ENSP00000359505-D1	-1.02	3.07E-26	↑	succinate dehydrogenase assembly factor 4, mitochondrial [Bison bison bison]
ENSP00000346839-D1	-1.02	1.02E-28	↑	fibronectin isoform X1 [B. mutus]
ENSP00000262293-D1	-1.02	9.64E-08	↑	proline-rich protein 11 [B. mutus]
ENSBTAP00000004426-D1	-1.02	1.33E-07	↑	Protein FAM154B, partial [B. mutus]
ENSP00000346627-D1	-1.02	1.57E-04	↑	tRNA wybutosine-synthesizing protein 5 [B. mutus]
ENSP00000377107-D1	-1.02	8.65E-54	↑	F-actin-capping protein subunit alpha-2, partial [B. mutus]
ENSP00000334040-D1	-1.02	1.67E-22	↑	Round spermatid basic protein 1-like protein [B. mutus]
ENSBTAP00000035023-D42	-1.02	4.69E-11	↑	Zinc finger protein 674, partial [B. mutus]
ENSP00000332979-D1	-1.02	4.19E-04	↑	Beta-secretase 2, partial [B. mutus]
ENSBTAP00000004334-D1	-1.02	7.44E-186	↑	HD domain-containing protein 2, partial [B. mutus]
ENSBTAP00000002165-D1	-1.02	0.00E+00	↑	pre-mRNA-splicing factor SYF2 [B. mutus]
ENSP00000392376-D2	-1.02	6.00E-10	↑	hypothetical protein M91_12181, partial [B. mutus]
ENSBTAP00000013248-D1	-1.02	0.00E+00	↑	Kinetochore protein Spc25, partial [B. mutus]
ENSBTAP00000010428-D1	-1.02	2.83E-24	↑	probable sodium-coupled neutral amino acid transporter 6 isoform X1 [B. taurus]
ENSP00000395463-D14	-1.02	0.00E+00	↑	hypothetical protein M91_18730, partial [B. mutus]
ENSBTAP00000018079-D1	-1.02	5.13E-12	↑	bile acid receptor isoform X1 [B. taurus]
ENSBTAP00000032331-D1	-1.01	0.00E+00	↑	Soma ferritin [B. mutus]
ENSP00000265018-D2	-1.01	2.17E-03	↑	hypothetical protein M91_14807, partial [B. mutus]
ENSP00000348501-D27	-1.01	2.17E-03	↑	zinc finger protein 565 [B. mutus]
ENSP00000375899-D1	-1.01	8.33E-05	↑	nuclear body protein SP140 isoform X29 [B. taurus]
ENSP00000390232-D1	-1.01	1.21E-05	↑	zinc finger B-box domain-containing protein 1 [B. mutus]
ENSBTAP00000005874-D1	-1.01	7.99E-28	↑	hydroxyacid-oxoacid transhydrogenase, mitochondrial isoform X1 [B. mutus]
ENSP00000336673-D1	-1.01	4.17E-09	↑	transmembrane protein 106B [B. taurus]
ENSBTAP00000026765-D1	-1.01	3.05E-04	↑	epoxide hydrolase 3 [B. taurus]
ENSP00000408451-D5	-1.01	6.86E-44	↑	60S ribosomal protein L36 [B. taurus]
ENSP00000355649-D1	-1.01	4.24E-04	↑	S-phase response (cyclin related) [Equus caballus]
ENSBTAP00000010234-D2	-1.01	5.92E-03	↑	N-alpha-acetyltransferase 50-like [B. mutus]
ENSP00000409199-D1	-1.01	5.92E-03	↑	transmembrane glycoprotein NMB [B. mutus]
ENSBTAP00000017816-D1	-1.01	2.05E-08	↑	proteasome subunit beta type-6 isoform X1 [Bubalus bubalis]
ENSP00000264065-D1	-1.00	4.52E-75	↑	dnaJ homolog subfamily C member 10 [Bison bison bison]
ENSP00000026464-D1	-1.00	4.37E-17	↑	Coiled-coil domain-containing protein 28A, partial [B. mutus]

ENSBTAP00000006331-D1	-1.00	9.03E-43	↑	39S ribosomal protein L35, mitochondrial [B. taurus]
ENSP00000222124-D1	-1.00	1.96E-15	↑	transmembrane protein 205 [B. mutus]
ENSP00000369400-D1	1.00	5.37E-47	↓	Nance-Horan syndrome protein, partial [B. mutus]
ENSBTAP00000018650-D1	1.00	3.05E-266	↓	FYVE, RhoGEF and PH domain-containing protein 6, partial [B. mutus]
ENSBTAP00000012582-D1	1.00	4.30E-40	↓	nesprin-2 [B. mutus]
ENSP00000340330-D1	1.00	4.93E-10	↓	histone acetyltransferase KAT5 isoform X1 [B. taurus]
ENSP00000267102-D1	1.00	5.67E-06	↓	protein LMBR1L [B. taurus]
ENSBTAP00000023563-D1	1.00	2.29E-32	↓	RNA-binding Raly-like protein, partial [B. mutus]
ENSBTAP00000051366-D1	1.00	9.55E-11	↓	polycomb group RING finger protein 3 [B. taurus]
ENSP00000392709-D1	1.00	7.34E-80	↓	NADH-ubiquinone oxidoreductase 75 kDa subunit, mitochondrial, partial [B. mutus]
ENSBTAP00000022372-D1	1.00	5.56E-152	↓	U5 small nuclear ribonucleoprotein 40 kDa protein [B. mutus]
ENSBTAP00000018514-D1	1.01	4.56E-19	↓	hypothetical protein M91_16219, partial [B. mutus]
ENSP00000395910-D1	1.01	9.09E-08	↓	protein tyrosine phosphatase domain-containing protein 1 isoform X1 [B. mutus]
ENSP00000378897-D1	1.01	5.29E-13	↓	Neutral ceramidase, partial [B. mutus]
ENSP00000216489-D1	1.01	3.37E-28	↓	alkylated DNA repair protein alkB homolog 1 isoform X1 [B. taurus]
ENSBTAP00000013678-D1	1.01	9.91E-06	↓	probable tRNA(His) guanylyltransferase [B. taurus]
ENSBTAP00000019537-D1	1.01	0.00E+00	↓	transcriptional adapter 2-alpha [B. taurus]
ENSP00000384048-D1	1.01	4.49E-14	↓	PAX-interacting protein 1, partial [B. mutus]
ENSP00000288757-D1	1.01	3.12E-18	↓	uncharacterized protein C12orf43 homolog isoform X1 [B. mutus]
ENSP00000260187-D1	1.01	2.07E-54	↓	ubiquitin carboxyl-terminal hydrolase 2 [B. taurus]
ENSBTAP00000025616-D2	1.01	3.05E-08	↓	hypothetical protein M91_18977, partial [B. mutus]
ENSBTAP00000007075-D1	1.01	5.70E-86	↓	F-box only protein 30 [B. mutus]
ENSP00000196489-D14	1.01	2.62E-14	↓	hypothetical protein M91_05127, partial [B. mutus]
ENSP00000337313-D1	1.01	1.47E-30	↓	Zinc finger CCHC domain-containing protein 8 [B. mutus]
ENSP00000298288-D1	1.01	4.50E-21	↓	leucine-rich repeat protein 1 isoform X1 [B. mutus]
ENSP00000359172-D1	1.01	1.53E-51	↓	UDP-N-acetylglucosamine transporter, partial [B. mutus]
ENSP00000371710-D1	1.01	2.13E-04	↓	Cadherin-18, partial [B. mutus]
ENSBTAP00000053793-D1	1.01	2.71E-03	↓	methionine sulfoxide reductase B2 [B. mutus]
ENSBTAP00000000051-D1	1.01	6.42E-03	↓	hypothetical protein M91_19667, partial [B. mutus]
ENSBTAP00000026423-D1	1.01	1.58E-02	↓	TGF-beta receptor type-2, partial [B. mutus]
ENSBTAP00000025092-D1	1.01	1.58E-02	↓	Dead end protein-like protein 1 [B. mutus]
ENSP00000407071-D1	1.01	4.13E-02	↓	hypothetical protein M91_20231 [B. mutus]
ENSBTAP00000010776-D1	1.01	4.13E-02	↓	Lipid phosphate phosphatase-related protein type 2 [B. mutus]
ENSP00000378857-D1	1.01	4.13E-02	↓	WD repeat-containing protein 6 [B. mutus]
ENSP00000227471-D1	1.01	4.13E-02	↓	Protein unc-93-like protein B1, partial [B. mutus]
ENSBTAP00000028657-D1	1.01	4.13E-02	↓	epithelial chloride channel protein-like [B. mutus]
ENSBTAP00000043930-D1	1.01	4.13E-02	↓	DPY30 domain-containing protein 2 isoform X1 [B. mutus]
ENSBTAP00000020677-D1	1.01	4.13E-02	↓	hypothetical protein [B. taurus]
ENSBTAP00000022654-D1	1.01	4.13E-02	↓	Lymphocyte antigen 6E, partial [B. mutus]
ENSP00000417207-D1	1.01	4.13E-02	↓	Homeobox protein Hox-B3 [B. mutus]
ENSBTAP00000041558-D1	1.01	1.00E-22	↓	tRNA-splicing endonuclease subunit Sen2 isoform X1 [B. mutus]
ENSP00000259512-D1	1.01	1.86E-18	↓	derlin-1 [B. taurus]
ENSP00000276326-D1	1.01	3.46E-37	↓	F-box only protein 25 [B. mutus]
ENSBTAP00000020923-D1	1.01	2.48E-25	↓	U6 snRNA phosphodiesterase isoform X1 [B. mutus]
ENSBTAP00000015592-D1	1.01	1.22E-19	↓	Ubiquitin carboxyl-terminal hydrolase 31, partial [B. mutus]
ENSBTAP00000021080-D1	1.01	3.61E-35	↓	pre-rRNA-processing protein TSR2 homolog [B. mutus]
ENSBTAP00000015645-D1	1.01	3.45E-09	↓	Secretogranin-1, partial [B. mutus]
ENSP00000320949-D1	1.01	4.14E-164	↓	CCR4-NOT transcription complex subunit 1 [B. taurus]
ENSP00000369162-D1	1.01	3.85E-100	↓	serine/threonine-protein kinase RIO1 [B. mutus]
ENSBTAP00000020161-D1	1.01	4.81E-07	↓	GRB2-related adapter protein 2 [B. taurus]
ENSBTAP00000041402-D1	1.01	4.95E-65	↓	A-kinase anchor protein 10, mitochondrial, partial [B. mutus]
ENSP00000351524-D1	1.01	3.32E-50	↓	probable helicase with zinc finger domain [B. mutus]
ENSBTAP00000011379-D1	1.01	2.47E-152	↓	mitochondrial fission regulator 1 [B. mutus]
ENSP00000310561-D1	1.01	9.00E-15	↓	rho GTPase-activating protein 32 [B. mutus]
ENSBTAP00000028405-D1	1.01	1.89E-18	↓	Solute carrier family 25 member 40, partial [B. mutus]

ENSBTAP00000017341-D1	1.01	1.32E-05	↓	metabotropic glutamate receptor 7 isoform X3 [Ovis aries]
ENSP00000349824-D1	1.01	1.32E-05	↓	ralBP1-associated Eps domain-containing protein 2 isoform X5 [B. taurus]
ENSBTAP00000035972-D1	1.01	1.32E-05	↓	Protein YIPF2 [B. mutus]
ENSBTAP00000004475-D1	1.01	1.39E-29	↓	ferric-chelate reductase 1 [B. mutus]
ENSP00000413684-D1	1.01	1.33E-15	↓	dnaJ homolog subfamily B member 5 isoform X1 [Capra hircus]
ENSP00000384638-D1	1.01	0.00E+00	↓	Catenin alpha-1 [B. mutus]
ENSP00000344666-D1	1.01	3.05E-05	↓	merlin isoform X1 [Bubalus bubalis]
ENSP00000249910-D1	1.01	3.59E-71	↓	Methyl-CpG-binding domain protein 4, partial [B. mutus]
ENSP00000417038-D1	1.01	7.01E-05	↓	phosphatase and actin regulator 2 isoform X3 [Bison bison bison]
ENSBTAP00000051869-D1	1.01	1.61E-07	↓	unnamed protein product [Mus musculus]
ENSBTAP00000009098-D1	1.01	9.36E-65	↓	ATP-binding cassette sub-family F member 1 [B. taurus]
ENSBTAP00000024502-D1	1.02	2.19E-28	↓	Tubulin polyglutamylase TTLL4 [B. mutus]
ENSP00000372024-D1	1.02	2.00E-25	↓	hypothetical protein M91_10246, partial [B. mutus]
ENSP00000405387-D1	1.02	3.70E-112	↓	protein SMG7 isoform X2 [Bison bison bison]
ENSP00000316840-D1	1.02	4.39E-36	↓	TNF receptor-associated factor 6 [B. mutus]
ENSBTAP00000013621-D1	1.02	2.49E-29	↓	exportin-6 isoform X1 [B. taurus]
ENSP00000303844-D1	1.02	0.00E+00	↓	cellular nucleic acid-binding protein isoform X1 [Balaenoptera acutorostrata scammoni]
ENSP00000318944-D1	1.02	4.74E-23	↓	E3 ubiquitin-protein ligase TRAF7, partial [B. mutus]
ENSP00000362674-D1	1.02	9.63E-13	↓	TPA: histone deacetylase 8 [B. taurus]
ENSP00000385253-D1	1.02	2.04E-03	↓	egl nine homolog 2 isoform X2 [B. taurus]
ENSP00000266732-D1	1.02	1.28E-48	↓	lamina-associated polypeptide 2, isoforms beta/delta/epsilon/gamma isoform X1 [B. mutus]
ENSP00000369325-D1	1.02	2.61E-36	↓	Cyclin-dependent kinase-like 5, partial [B. mutus]
ENSP00000247207-D4	1.02	9.39E-08	↓	Heat shock 70 kDa protein 1B [B. mutus]
ENSBTAP00000020635-D1	1.02	9.39E-08	↓	Mitochondrial intermembrane space import and assembly protein 40 [B. mutus]
ENSBTAP00000002350-D1	1.02	6.11E-09	↓	Lon protease-like protein, mitochondrial, partial [B. mutus]
ENSP00000360891-D1	1.02	2.14E-07	↓	interferon-induced protein with tetratricopeptide repeats 2 [B. mutus]
ENSP00000411418-D1	1.02	1.54E-108	↓	Rab3 GTPase-activating protein catalytic subunit, partial [B. mutus]
ENSBTAP00000014523-D1	1.02	0.00E+00	↓	mRNA export factor isoform X1 [Capra hircus]
ENSBTAP00000050665-D1	1.02	1.33E-10	↓	syntaxin-binding protein 1 [Cricetulus griseus]
ENSBTAP00000015633-D1	1.02	5.49E-24	↓	protein FAM124B-like [B. mutus]
ENSP00000401112-D1	1.02	1.76E-05	↓	DNA-binding protein SATB2 [B. mutus]
ENSP00000398365-D1	1.02	1.76E-05	↓	Dickkopf-related protein 3, partial [B. mutus]
ENSBTAP00000014649-D1	1.02	4.85E-03	↓	prostaglandin reductase 1 [B. mutus]
ENSBTAP00000044758-D1	1.02	1.99E-215	↓	SHC SH2 domain-binding protein 1 [B. mutus]
ENSBTAP00000027858-D1	1.02	1.20E-16	↓	Formin-binding protein 4, partial [B. mutus]
ENSP00000350854-D1	1.02	2.46E-24	↓	vacuolar protein sorting-associated protein 13D [B. mutus]
ENSBTAP00000020222-D1	1.02	6.64E-04	↓	Zinc finger protein DZIP1L [B. mutus]
ENSP00000299413-D1	1.02	6.64E-04	↓	tripartite motif-containing protein 44 [B. taurus]
ENSP00000417132-D1	1.02	9.35E-05	↓	ubiquitin carboxyl-terminal hydrolase BAP1 isoform X1 [B. mutus]
yakG034094	1.02	2.55E-22	↓	High affinity cAMP-specific 3',5'-cyclic phosphodiesterase 7A [B. mutus]
ENSBTAP00000020126-D1	1.02	4.46E-113	↓	Arginine and glutamate-rich protein 1 [B. mutus]
ENSBTAP00000026055-D1	1.02	2.83E-25	↓	A-kinase anchor protein 17B, partial [B. mutus]
ENSBTAP00000039752-D1	1.02	8.65E-282	↓	BTB/POZ domain-containing protein 10 [B. taurus]
ENSP00000361310-D1	1.02	4.18E-08	↓	DNA polymerase eta isoform X1 [B. mutus]
ENSBTAP00000053621-D1	1.02	6.60E-98	↓	nuclear cap-binding protein subunit 1 [B. mutus]
ENSP00000155840-D1	1.02	1.55E-03	↓	potassium voltage-gated channel subfamily KQT member 1 [B. mutus]
ENSBTAP00000012406-D1	1.02	1.17E-02	↓	gigaxonin [B. taurus]
ENSBTAP00000019875-D1	1.02	1.17E-02	↓	Myosin light chain kinase 2, skeletal/cardiac muscle [B. mutus]
ENSBTAP00000046883-D1	1.02	2.56E-35	↓	Tryptophanyl-tRNA synthetase, cytoplasmic [B. mutus]
ENSP00000342876-D1	1.02	6.53E-25	↓	Zinc finger protein Helios [Tupaia chinensis]
ENSP00000344215-D1	1.02	9.81E-150	↓	tumor protein p53-inducible nuclear protein 1 isoform X1 [B. mutus]
ENSBTAP00000042937-D2	1.02	3.63E-29	↓	rhombotin-1 [B. taurus]
ENSBTAP00000040330-D1	1.02	7.61E-13	↓	Dolichyl-diphosphooligosaccharide--protein glycosyltransferase 48 kDa subunit, partial [B. mutus]
ENSBTAP00000019692-D1	1.02	1.49E-06	↓	armadillo repeat-containing protein 7 [B. taurus]
ENSP00000265036-D1	1.03	1.79E-176	↓	DEP domain-containing protein 1B isoform X1 [B. mutus]

ENSBTAP00000048150-D1	1.03	1.08E-08	↓	ATR-interacting protein [B. taurus]
ENSP00000346762-D1	1.03	3.98E-34	↓	staphylococcal nuclease domain-containing protein 1 [B. taurus]
ENSBTAP00000024318-D1	1.03	4.96E-07	↓	nuclear factor of activated T-cells, cytoplasmic 2 [B. mutus]
ENSBTAP00000019823-D1	1.03	5.03E-04	↓	cytochrome P450 19A1-like [B. mutus]
ENSBTAP00000019422-D1	1.03	3.62E-267	↓	ribosomal L1 domain-containing protein 1 [B. mutus]
ENSBTAP00000002687-D1	1.03	8.39E-29	↓	lysine-specific demethylase 4A isoform X1 [B. mutus]
ENSBTAP00000053827-D1	1.03	1.76E-73	↓	Forkhead box protein J3, partial [B. mutus]
ENSP00000244040-D1	1.03	1.38E-19	↓	ras-related protein Rab-22A isoform X1 [Ovis aries]
ENSP00000265062-D1	1.03	3.48E-22	↓	ras-related protein Rab-7a isoform X1 [B. taurus]
ENSBTAP00000041555-D1	1.03	7.81E-06	↓	peroxisome proliferator-activated receptor gamma [B. taurus]
ENSBTAP00000004644-D2	1.03	1.64E-04	↓	ATP-dependent RNA helicase DDX19B, partial [B. mutus]
ENSBTAP00000012210-D1	1.03	3.64E-03	↓	UHRF1-binding protein 1, partial [B. mutus]
ENSP00000268206-D1	1.03	1.02E-23	↓	elongation factor Tu GTP-binding domain-containing protein 1 isoform X1 [B. mutus]
ENSP00000259406-D1	1.03	3.12E-10	↓	Regulator of G-protein signaling 3, partial [B. mutus]
ENSP00000358404-D1	1.03	1.75E-12	↓	Transcription factor 7-like 2 [B. mutus]
ENSP00000345789-D3	1.03	1.62E-09	↓	PWWP domain-containing protein MUM1, partial [B. mutus]
ENSP00000264649-D1	1.03	2.50E-62	↓	V-type proton ATPase 116 kDa subunit a isoform 1 isoform X1 [Capra hircus]
ENSP00000246789-D1	1.03	2.05E-24	↓	protein arginine N-methyltransferase 1 isoform 1 [Homo sapiens]
ENSBTAP00000007273-D1	1.03	3.82E-04	↓	glycine N-methyltransferase [B. mutus]
ENSBTAP00000034443-D1	1.03	1.07E-53	↓	Serine/threonine-protein phosphatase 6 regulatory ankyrin repeat subunit B, partial [B. mutus]
ENSP00000278319-D1	1.03	0.00E+00	↓	Zinc finger protein 215 [B. mutus]
ENSP00000337512-D1	1.03	7.83E-13	↓	ftsJ methyltransferase domain-containing protein 1 [B. mutus]
ENSBTAP00000018279-D1	1.03	6.19E-22	↓	Transcriptional repressor CTCF [B. mutus]
ENSP00000364133-D1	1.03	4.14E-21	↓	TGF-beta receptor type-1 [Bison bison bison]
ENSBTAP00000026774-D1	1.03	1.36E-05	↓	p53 apoptosis effector related to PMP-22 [B. taurus]
ENSP00000264156-D1	1.03	0.00E+00	↓	DNA replication licensing factor MCM6 [B. mutus]
ENSBTAP00000004547-D1	1.03	1.07E-10	↓	Ectonucleotide pyrophosphatase/phosphodiesterase family member 4, partial [B. mutus]
ENSBTAP00000018554-D1	1.03	5.37E-25	↓	phosphoserine aminotransferase [Bison bison bison]
ENSBTAP00000035840-D1	1.03	1.69E-07	↓	snRNA-activating protein complex subunit 2 [B. mutus]
ENSBTAP00000021186-D1	1.03	3.85E-108	↓	tetratricopeptide repeat protein 4 isoform X1 [B. mutus]
ENSBTAP00000021556-D1	1.03	7.94E-45	↓	histone acetyltransferase p300 isoform X2 [Bubalus bubalis]
ENSBTAP00000018956-D1	1.03	3.08E-12	↓	sterol regulatory element-binding protein 2 [B. mutus]
ENSBTAP00000027271-D1	1.03	2.50E-19	↓	fermitin family homolog 1 [B. taurus]
ENSP00000356984-D1	1.03	1.89E-102	↓	Death effector domain-containing protein [B. mutus]
ENSP00000377473-D1	1.04	8.76E-03	↓	protein KIBRA [B. mutus]
ENSP00000415034-D1	1.04	8.76E-03	↓	neurobeachin-like protein 2 [B. mutus]
ENSP00000392204-D1	1.04	8.76E-03	↓	Killin, partial [B. mutus]
ENSP00000221561-D1	1.04	8.76E-03	↓	amino-terminal enhancer of split [B. mutus]
ENSBTAP00000008196-D1	1.04	3.14E-05	↓	WD repeat domain-containing protein 83 isoform X1 [B. mutus]
ENSP00000377112-D1	1.04	2.76E-03	↓	Solute carrier family 12 member 8, partial [B. mutus]
ENSBTAP00000041372-D1	1.04	2.98E-02	↓	Sushi domain-containing protein 1, partial [B. mutus]
ENSP00000249330-D1	1.04	2.98E-02	↓	Neurosecretory protein VGF, partial [B. mutus]
ENSBTAP00000010194-D1	1.04	2.98E-02	↓	Kinesin-like protein KIFC2 [B. mutus]
ENSBTAP00000014236-D1	1.04	2.98E-02	↓	thyroid hormone receptor alpha [B. taurus]
ENSBTAP00000042304-D2	1.04	2.98E-02	↓	signal transducer CD24 [B. taurus]
ENSBTAP00000015994-D1	1.04	2.98E-02	↓	Diphosphomevalonate decarboxylase, partial [B. mutus]
ENSBTAP00000043333-D1	1.04	2.98E-02	↓	glutaryl-CoA dehydrogenase, mitochondrial [B. mutus]
ENSP00000370949-D1	1.04	2.15E-22	↓	NF-X1-type zinc finger protein NFXL1 isoform X1 [Bison bison bison]
ENSP00000327436-D1	1.04	6.71E-16	↓	histone-lysine N-methyltransferase setd3 [B. mutus]
ENSBTAP00000021623-D1	1.04	1.08E-10	↓	Pleckstrin-2, partial [B. mutus]
ENSBTAP00000023618-D1	1.04	3.20E-48	↓	nuclear factor interleukin-3-regulated protein [B. mutus]
ENSBTAP00000001974-D1	1.04	1.67E-20	↓	ETS domain-containing protein Elk-3 [B. taurus]
ENSP00000409466-D1	1.04	8.47E-09	↓	Retrograde Golgi transport protein RGP1-like protein, partial [B. mutus]
ENSBTAP00000030687-D1	1.04	7.43E-127	↓	PX domain-containing protein kinase-like protein [B. taurus]
ENSP00000371221-D1	1.04	1.03E-14	↓	Myotubularin-related protein 6, partial [B. mutus]

ENSBTAP00000032679-D1	1.04	7.22E-05	↓	hypothetical protein M91_05894, partial [B. mutus]
ENSBTAP00000026305-D1	1.04	5.16E-60	↓	nuclear protein MDM1 [B. mutus]
ENSP00000351933-D1	1.04	2.17E-09	↓	kelch-like protein 9 [Ailuropoda melanoleuca]
ENSBTAP00000013726-D1	1.04	6.79E-16	↓	dedicator of cytokinesis protein 9 isoform X4 [Bison bison bison]
ENSBTAP00000023201-D1	1.04	2.02E-06	↓	Calcitonin receptor, partial [B. mutus]
ENSBTAP00000030166-D1	1.04	2.56E-131	↓	Protein FAM50A, partial [B. mutus]
ENSBTAP00000007065-D1	1.04	1.87E-10	↓	UDP-N-acetylglucosamine--dolichyl-phosphate N-acetylglucosaminephosphotransferase [B. mutus]
ENSBTAP00000025160-D1	1.04	6.73E-04	↓	enkurin domain-containing protein 1 [B. mutus]
ENSP00000287322-D3	1.04	4.58E-15	↓	BAG family molecular chaperone regulator 4, partial [B. mutus]
ENSBTAP00000017657-D1	1.04	5.10E-07	↓	coiled-coil domain-containing protein 22 isoform X1 [B. taurus]
ENSBTAP00000022726-D1	1.04	2.96E-47	↓	cap-specific mRNA (nucleoside-2'-O-)-methyltransferase 1 [Bubalus bubalis]
ENSP00000375700-D1	1.04	4.36E-08	↓	nuclear pore glycoprotein p62 [Bubalus bubalis]
ENSP00000354855-D1	1.04	1.53E-06	↓	Nuclear factor erythroid 2-related factor 1, partial [B. mutus]
ENSBTAP00000021833-D1	1.04	2.07E-03	↓	leukotriene A-4 hydrolase [B. taurus]
ENSP00000419740-D1	1.04	2.07E-03	↓	5-methylcytosine rRNA methyltransferase NSUN4 [B. taurus]
ENSP00000337572-D1	1.04	5.61E-56	↓	TPA: diacylglycerol kinase, eta-like [B. taurus]
ENSP00000376703-D2	1.04	3.99E-43	↓	anoctamin-4 isoform X1 [Ovis aries]
ENSBTAP00000016954-D1	1.04	7.40E-85	↓	T-complex protein 1 subunit eta, partial [B. mutus]
ENSP00000256658-D1	1.04	4.10E-14	↓	AP-4 complex subunit beta-1 isoform X1 [B. mutus]
ENSP00000253457-D1	1.04	4.16E-39	↓	ER membrane protein complex subunit 8 [Sus scrofa]
ENSBTAP00000010129-D1	1.04	4.17E-05	↓	elongation of very long chain fatty acids protein 7 [B. taurus]
ENSP00000252137-D1	1.04	6.53E-03	↓	protein DGCR14 [B. taurus]
ENSP00000321326-D1	1.04	6.53E-03	↓	Proteinase-activated receptor 1, partial [B. mutus]
ENSP00000264448-D1	1.04	2.21E-143	↓	Alstrom syndrome protein 1, partial [B. mutus]
ENSP00000391106-D1	1.04	2.10E-42	↓	Myosin-X, partial [B. mutus]
ENSBTAP00000022338-D1	1.05	6.75E-24	↓	peroxisomal biogenesis factor 7 [B. taurus]
ENSP00000252677-D1	1.05	0.00E+00	↓	bone morphogenetic protein 15 [B. mutus]
ENSBTAP00000042742-D1	1.05	5.00E-09	↓	cyclic AMP-dependent transcription factor ATF-6 beta isoform X2 [B. mutus]
ENSP00000261556-D1	1.05	1.39E-14	↓	hypothetical protein M91_11980, partial [B. mutus]
ENSP00000346809-D1	1.05	1.76E-24	↓	GPI inositol-deacylase [B. mutus]
ENSBTAP00000012242-D1	1.05	1.57E-03	↓	Ena/VASP-like protein, partial [B. mutus]
ENSBTAP00000048457-D1	1.05	1.35E-92	↓	General transcription factor IIF subunit 1, partial [B. mutus]
ENSP00000384832-D1	1.05	3.92E-233	↓	Protein FAM104A [B. mutus]
ENSBTAP00000042161-D1	1.05	1.65E-54	↓	Zinc finger protein 256 [B. mutus]
ENSBTAP00000046417-D1	1.05	2.57E-08	↓	uncharacterized protein C7orf57 homolog [Pantholops hodgsonii]
ENSBTAP00000034418-D1	1.05	1.58E-23	↓	kinetochore-associated protein DSN1 homolog [B. mutus]
ENSP00000396755-D1	1.05	1.44E-12	↓	cyclin N-terminal domain-containing protein 2 [B. mutus]
ENSP00000403343-D1	1.05	1.97E-08	↓	angio-associated migratory cell protein isoform X1 [B. taurus]
ENSP00000229268-D1	1.05	4.65E-06	↓	ubiquitin carboxyl-terminal hydrolase 5 [B. mutus]
ENSBTAP00000051966-D1	1.05	1.84E-05	↓	DENN domain-containing protein 2D [B. mutus]
ENSBTAP00000017344-D1	1.05	7.31E-05	↓	NIPA-like protein 3 [B. taurus]
ENSBTAP00000001667-D1	1.05	7.31E-05	↓	histidine-rich glycoprotein [Bubalus bubalis]
ENSBTAP00000031777-D1	1.05	2.92E-04	↓	Protein asteroid-like protein 1 [B. mutus]
ENSBTAP00000008078-D1	1.05	2.16E-02	↓	peroxisomal membrane protein 11A [B. taurus]
ENSP00000262843-D1	1.05	3.02E-36	↓	Midline-1, partial [B. mutus]
ENSP00000229268-D2	1.05	4.14E-160	↓	Ubiquitin carboxyl-terminal hydrolase 13, partial [B. mutus]
yakG009020	1.05	2.02E-262	↓	hypothetical protein M91_08913 [B. mutus]
ENSP00000285398-D1	1.05	8.93E-40	↓	TFIIH basal transcription factor complex helicase XPB subunit [B. mutus]
ENSBTAP00000021955-D1	1.05	1.94E-10	↓	Carboxypeptidase E, partial [B. mutus]
ENSP00000360305-D1	1.06	5.23E-186	↓	PDZ and LIM domain protein 1 [B. mutus]
ENSP00000373522-D1	1.06	4.09E-32	↓	Lysine-specific histone demethylase 1B [B. mutus]
ENSBTAP00000016204-D1	1.06	8.05E-35	↓	complement C5-like [B. mutus]
ENSBTAP00000018375-D1	1.06	1.71E-09	↓	coiled-coil domain-containing protein 170-like [B. mutus]
ENSP00000350945-D2	1.06	2.38E-50	↓	Myosin-Vb, partial [B. mutus]
ENSP00000394817-D1	1.06	2.50E-301	↓	Calnexin, partial [B. mutus]

ENSBTAP00000023798-D1	1.06	1.84E-34	↓	WD repeat-containing protein 91 [B. mutus]
ENSP00000301764-D1	1.06	8.41E-15	↓	DNA damage-binding protein 1 [B. mutus]
ENSP00000390595-D1	1.06	6.31E-29	↓	TPA: sprouty-related, EVH1 domain containing 2-like [B. taurus]
ENSP00000370372-D1	1.06	4.25E-16	↓	Neurolysin, mitochondrial, partial [B. mutus]
ENSBTAP00000034918-D1	1.06	1.96E-10	↓	Tetratricopeptide repeat protein 19, partial [B. mutus]
ENSBTAP00000024487-D1	1.06	2.48E-16	↓	methionine--tRNA ligase, cytoplasmic [B. mutus]
ENSP00000306968-D1	1.06	2.31E-82	↓	Cell division cycle-associated protein 7, partial [B. mutus]
ENSP00000351664-D1	1.06	1.25E-19	↓	NEDD9-interacting protein with calponin-like protein and LIM domains [B. mutus]
ENSBTAP00000008135-D1	1.06	1.90E-16	↓	interleukin-1 receptor-associated kinase-like 2 [B. mutus]
ENSP00000420213-D1	1.06	1.52E-08	↓	filamin-B isoform X1 [B. mutus]
ENSP00000262995-D1	1.06	1.25E-47	↓	GRB2-associated-binding protein 1 isoform X1 [B. mutus]
ENSP00000219139-D1	1.06	1.88E-30	↓	hypothetical protein M91_18290 [B. mutus]
ENSBTAP00000018746-D1	1.06	2.40E-68	↓	Muscleblind-like protein 3, partial [B. mutus]
ENSBTAP00000001445-D1	1.06	6.81E-04	↓	vesicular glutamate transporter 3 isoform X1 [B. mutus]
ENSP00000391521-D1	1.06	1.26E-53	↓	solute carrier family 25 member 36 [B. taurus]
ENSP00000219240-D1	1.06	1.11E-22	↓	Dihydroorotate dehydrogenase, mitochondrial, partial [B. mutus]
ENSP00000384763-D1	1.06	8.01E-150	↓	Sodium/calcium exchanger 1, partial [B. mutus]
ENSP00000372689-D1	1.06	4.36E-42	↓	Cohesin subunit SA-1, partial [B. mutus]
ENSBTAP00000049387-D4	1.06	2.79E-03	↓	Dimethylaniline monooxygenase [N-oxide-forming] 1, partial [B. mutus]
ENSBTAP00000001633-D1	1.06	2.79E-03	↓	myelin protein zero-like protein 2 precursor [B. taurus]
ENSP00000407031-D1	1.06	1.60E-02	↓	NUT family member 1 [B. mutus]
ENSBTAP00000001038-D1	1.07	4.40E-44	↓	vascular endothelial growth factor receptor 2 precursor [B. taurus]
ENSP00000265381-D1	1.07	2.86E-21	↓	Amyloid beta A4 precursor protein-binding family A member 1, partial [B. mutus]
ENSBTAP00000041888-D1	1.07	1.70E-13	↓	Selenoprotein T [B. mutus]
ENSP00000317123-D1	1.07	1.15E-10	↓	U5 small nuclear ribonucleoprotein 200 kDa helicase [B. mutus]
ENSBTAP00000003257-D1	1.07	2.72E-06	↓	guanine nucleotide-binding protein G(I)/G(S)/G(O) subunit gamma-3-like protein [Camelus ferus]
ENSP00000194118-D1	1.07	2.72E-06	↓	death ligand signal enhancer [B. mutus]
ENSP00000354927-D1	1.07	6.62E-13	↓	mitogen-activated protein kinase kinase kinase 3 isoform X1 [B. taurus]
ENSBTAP00000018739-D1	1.07	1.41E-05	↓	HAUS augmin-like complex subunit 7 [B. mutus]
ENSP00000358458-D1	1.07	1.93E-22	↓	protein FAM46C [B. mutus]
ENSBTAP00000007349-D1	1.07	1.53E-08	↓	Insulin-like growth factor-binding protein 2, partial [B. mutus]
ENSBTAP00000023206-D1	1.07	2.57E-12	↓	EGF-containing fibulin-like extracellular matrix protein 1 [B. mutus]
ENSP00000309262-D1	1.07	1.18E-159	↓	Ubiquitin carboxyl-terminal hydrolase 48, partial [B. mutus]
ENSP00000296292-D1	1.07	8.59E-17	↓	protein RFT1 homolog [B. mutus]
ENSBTAP00000017535-D1	1.07	8.89E-49	↓	uncharacterized protein KIAA0355 homolog [B. mutus]
ENSBTAP00000011513-D1	1.07	0.00E+00	↓	cullin-1-like [B. mutus]
ENSP00000339992-D1	1.07	2.61E-10	↓	transcriptional activator Myb isoform X1 [B. mutus]
ENSBTAP00000011617-D1	1.07	1.91E-136	↓	Next to BRCA1 1 protein [B. mutus]
ENSBTAP00000047800-D1	1.07	1.57E-84	↓	Nuclear pore complex protein Nup133, partial [B. mutus]
ENSBTAP00000002733-D1	1.07	5.81E-12	↓	zinc finger CCHC domain-containing protein 3 [B. taurus]
ENSP00000364277-D1	1.07	6.81E-09	↓	FYVE, RhoGEF and PH domain-containing protein 1, partial [B. mutus]
ENSBTAP00000035361-D5	1.07	2.27E-11	↓	hypothetical protein M91_15298 [B. mutus]
ENSBTAP00000047345-D1	1.07	2.95E-04	↓	F-box/WD repeat-containing protein 8 [B. mutus]
ENSP00000258302-D1	1.07	8.67E-19	↓	Regulator of G-protein signaling 8, partial [B. mutus]
ENSBTAP00000010219-D1	1.07	6.74E-27	↓	Equilibrative nucleoside transporter 4, partial [B. mutus]
ENSP00000360108-D1	1.07	4.51E-10	↓	Putative tyrosine-protein phosphatase auxilin, partial [B. mutus]
ENSP00000362993-D1	1.07	8.02E-44	↓	Protein bicaudal C-like protein 1, partial [B. mutus]
ENSP00000346693-D1	1.07	1.18E-02	↓	elongation of very long chain fatty acids protein 2 [B. taurus]
ENSP00000276326-D2	1.07	2.25E-15	↓	F-box only protein 32 [B. mutus]
ENSBTAP00000025659-D2	1.07	2.83E-142	↓	asparagine--tRNA ligase, cytoplasmic [B. mutus]
ENSBTAP00000043649-D1	1.07	5.15E-23	↓	hypothetical protein M91_20809, partial [B. mutus]
ENSP00000376785-D1	1.07	1.93E-81	↓	E3 ubiquitin-protein ligase TRIM37 [B. mutus]
ENSBTAP00000051259-D89	1.07	3.61E-06	↓	hypothetical protein M91_14419, partial [B. mutus]
ENSBTAP00000028036-D1	1.08	1.34E-09	↓	ADM isoform X1 [B. mutus]
ENSP00000359603-D1	1.08	8.76E-15	↓	Collagen alpha-1(XXIV) chain, partial [B. mutus]

ENSBTAP00000026808-D1	1.08	1.57E-36	↓	RNA binding protein fox-1 homolog 2 isoform X1 [Capra hircus]
ENSBTAP00000017653-D1	1.08	1.99E-12	↓	N-acetylglucosaminyl-phosphatidylinositol de-N-acetylase [B. mutus]
ENSP00000170564-D1	1.08	4.41E-71	↓	G patch domain-containing protein 1, partial [B. mutus]
ENSBTAP00000026311-D1	1.08	1.19E-03	↓	chromosome transmission fidelity protein 18 homolog [B. mutus]
ENSBTAP00000050375-D1	1.08	2.60E-71	↓	General transcription factor IIE subunit 1 [B. mutus]
ENSBTAP00000013153-D3	1.08	2.36E-31	↓	UV excision repair protein RAD23-like protein A [B. mutus]
ENSP00000346464-D1	1.08	1.54E-226	↓	vacuolar protein sorting-associated protein 72 homolog [Camelus ferus]
ENSP00000318185-D1	1.08	5.02E-64	↓	leucine-rich repeat-containing protein 42 isoform X1 [Bubalus bubalis]
ENSP00000337991-D1	1.08	4.51E-77	↓	protein SPT2 homolog [B. mutus]
ENSBTAP00000008766-D1	1.08	4.63E-30	↓	transcriptional repressor p66-beta [B. taurus]
ENSBTAP00000053475-D1	1.08	8.68E-62	↓	scm-like with four MBT domains protein 1 [B. taurus]
ENSBTAP00000010387-D1	1.08	1.04E-27	↓	Cytochrome b reductase 1, partial [B. mutus]
ENSP00000385120-D1	1.08	1.04E-27	↓	ornithine decarboxylase antizyme 2 [Otolemur garnettii]
ENSBTAP00000027398-D1	1.08	1.74E-21	↓	probable ATP-dependent RNA helicase DDX59 [B. mutus]
ENSP00000376234-D1	1.08	8.85E-03	↓	ATP-binding cassette sub-family B member 9 isoform X2 [B. mutus]
ENSP00000342576-D1	1.08	8.85E-03	↓	hypothetical protein M91_01497, partial [B. mutus]
ENSBTAP00000021336-D3	1.08	8.85E-03	↓	Homeobox protein Hox-B9 [B. mutus]
ENSP00000265814-D1	1.08	1.36E-25	↓	protein CBFA2T1 isoform X1 [Bison bison bison]
ENSP00000398476-D1	1.08	3.94E-19	↓	YTH domain-containing family protein 3 isoform X2 [Bison bison bison]
ENSP00000301831-D1	1.08	1.73E-214	↓	serine/threonine-protein kinase ULK4 [B. mutus]
ENSBTAP00000007860-D1	1.08	6.94E-74	↓	cytosol aminopeptidase [B. mutus]
ENSP00000333664-D1	1.08	9.23E-07	↓	3-ketoacyl-CoA thiolase B, peroxisomal isoform X1 [B. mutus]
ENSP00000358062-D1	1.08	2.45E-104	↓	absent in melanoma 1 protein [B. mutus]
ENSBTAP00000013082-D2	1.08	0.00E+00	↓	Aldose reductase, partial [B. mutus]
ENSBTAP00000027508-D1	1.08	3.28E-148	↓	Palmitoyltransferase ZDHHC13, partial [B. mutus]
ENSBTAP00000022075-D1	1.08	6.27E-06	↓	Formin-like protein 3, partial [B. mutus]
ENSP00000344816-D1	1.08	6.27E-06	↓	BSD domain-containing protein 1, partial [B. mutus]
ENSBTAP00000022675-D1	1.08	1.76E-19	↓	lysophosphatidic acid phosphatase type 6 isoform X1 [B. mutus]
ENSP00000334008-D1	1.09	1.75E-15	↓	alpha-parvin [Camelus dromedarius]
ENSBTAP00000024203-D1	1.09	3.09E-07	↓	Pyridoxal kinase, partial [B. mutus]
ENSP00000377813-D1	1.09	3.63E-06	↓	Protein phosphatase 1 regulatory subunit 1B, partial [B. mutus]
ENSP00000351657-D25	1.09	6.58E-03	↓	putative uncharacterized zinc finger protein 814-like [B. mutus]
ENSP00000225512-D1	1.09	6.58E-03	↓	proto-oncogene Wnt-3 precursor [B. taurus]
ENSBTAP00000047165-D3	1.09	6.58E-03	↓	hypothetical protein M91_00797, partial [B. mutus]
ENSP00000358016-D1	1.09	6.58E-03	↓	sex comb on midleg-like protein 4 isoform X2 [Bison bison bison]
ENSP00000258991-D1	1.09	5.22E-19	↓	Testis-expressed sequence 2 protein [B. mutus]
ENSBTAP00000009179-D1	1.09	5.05E-101	↓	transmembrane 7 superfamily member 3 [B. mutus]
ENSBTAP00000023200-D1	1.09	1.20E-57	↓	DCC-interacting protein 13-beta, partial [B. mutus]
ENSP00000361967-D1	1.09	6.26E-71	↓	tubulin-specific chaperone C [B. taurus]
ENSBTAP00000008839-D1	1.09	2.36E-07	↓	Fibroblast growth factor 16, partial [B. mutus]
ENSP00000419760-D1	1.09	3.25E-05	↓	transcription initiation factor TFIID subunit 6 isoform X1 [Pantholops hodgsonii]
ENSBTAP00000006221-D1	1.09	1.47E-41	↓	Synaptic vesicular amine transporter, partial [B. mutus]
ENSBTAP00000036132-D1	1.09	1.17E-49	↓	DnaJ-like protein subfamily B member 1, partial [B. mutus]
ENSP00000388381-D1	1.09	6.90E-11	↓	A-kinase anchor protein 1, mitochondrial, partial [B. mutus]
ENSP00000325313-D1	1.09	3.94E-04	↓	Microtubule-associated protein 1S [B. mutus]
ENSBTAP00000025453-D1	1.09	4.72E-198	↓	WD repeat and HMG-box DNA-binding protein 1 [B. mutus]
ENSBTAP00000005534-D1	1.09	1.54E-118	↓	Pre-rRNA-processing protein TSR1-like protein, partial [B. mutus]
ENSP00000374552-D1	1.09	2.50E-76	↓	E3 ubiquitin-protein ligase RNF216 [B. mutus]
ENSBTAP00000006868-D1	1.09	1.37E-07	↓	Lactase-like protein [B. mutus]
ENSP00000388373-D1	1.09	1.29E-83	↓	Liprin-alpha-2, partial [B. mutus]
ENSP00000387089-D1	1.09	6.85E-17	↓	NGFI-A-binding protein 1 isoform X1 [B. mutus]
ENSP00000401477-D1	1.09	1.05E-07	↓	Transmembrane protein 232, partial [B. mutus]
ENSBTAP00000013636-D1	1.09	1.05E-07	↓	Hypoxia up-regulated protein 1 [B. mutus]
ENSP00000223073-D1	1.09	6.72E-46	↓	RNA-binding protein 28 isoform X1 [B. mutus]
ENSBTAP00000013270-D1	1.09	5.29E-09	↓	short transient receptor potential channel 5 [B. mutus]

ENSP00000312770-D1	1.09	1.88E-05	↓	UPF0565 protein C2orf69 homolog [B. mutus]
ENSBTAP00000001564-D1	1.09	4.96E-03	↓	Telomerase protein component 1 [B. mutus]
ENSP00000389039-D2	1.09	4.03E-09	↓	synaptotagmin-16 isoform X3 [B. taurus]
ENSBTAP00000009038-D1	1.09	3.17E-27	↓	Ras-GEF domain-containing family member 1A, partial [B. mutus]
ENSP00000361027-D1	1.09	2.48E-33	↓	angiomotin [B. mutus]
ENSP00000355966-D1	1.09	1.40E-131	↓	serine/threonine-protein kinase Nek2 isoform X1 [B. taurus]
ENSBTAP00000017450-D1	1.09	9.66E-35	↓	Golgi resident protein GCP60 [B. taurus]
ENSBTAP00000047415-D1	1.09	2.33E-48	↓	LOC535754 protein [B. taurus]
ENSP00000334564-D1	1.09	7.22E-62	↓	DNA-directed RNA polymerase III subunit RPC3 [B. mutus]
ENSP00000283027-D1	1.09	6.53E-150	↓	cytosolic Fe-S cluster assembly factor NUBP1 [B. mutus]
ENSP00000344424-D1	1.09	2.02E-14	↓	uncharacterized protein KIAA0247 homolog isoform X1 [B. taurus]
ENSBTAP00000006568-D1	1.09	3.72E-32	↓	NTF2-related export protein 1 [B. mutus]
ENSBTAP00000035035-D1	1.09	7.27E-29	↓	HMG domain-containing protein 3, partial [B. mutus]
ENSBTAP00000001451-D1	1.09	4.45E-78	↓	interferon-inducible double-stranded RNA-dependent protein kinase activator A [B. taurus]
ENSP00000362287-D4	1.09	3.72E-03	↓	hypothetical protein PANDA_005372, partial [Ailuropoda melanoleuca]
ENSBTAP00000009255-D1	1.09	3.72E-03	↓	vesicular glutamate transporter 1 [B. taurus]
ENSP00000399341-D1	1.09	9.59E-127	↓	apoptosis inhibitor 5 [B. taurus]
ENSBTAP00000037315-D1	1.10	0.00E+00	↓	ZAR1-like protein [B. mutus]
ENSBTAP00000042386-D1	1.10	4.28E-62	↓	Enoyl-CoA hydratase, mitochondrial, partial [B. mutus]
ENSBTAP00000049055-D1	1.10	2.37E-07	↓	inhibitor of nuclear factor kappa-B kinase subunit beta [B. taurus]
ENSBTAP00000016607-D1	1.10	2.37E-07	↓	dual specificity tyrosine-phosphorylation-regulated kinase 1B [Pantholops hodgsonii]
ENSP00000301396-D1	1.10	1.30E-04	↓	PELP1 [Homo sapiens]
ENSP00000377390-D1	1.10	3.41E-259	↓	serine/threonine-protein phosphatase 6 regulatory subunit 3 isoform X1 [B. mutus]
ENSP00000370047-D1	1.10	8.72E-40	↓	Zinc finger protein basonuclin-2 [B. mutus]
ENSP00000354891-D1	1.10	4.59E-16	↓	protein FAM163A [B. mutus]
ENSP00000260283-D1	1.10	5.30E-09	↓	Rho GTPase-activating protein 20, partial [B. mutus]
ENSP00000373214-D1	1.10	5.30E-09	↓	Calcium-dependent secretion activator 1, partial [Heterocephalus glaber]
ENSBTAP00000040402-D1	1.10	9.83E-05	↓	T-cell immunoglobulin and mucin domain-containing protein 4 [B. mutus]
ENSBTAP00000001374-D1	1.10	9.04E-46	↓	3-hydroxyisobutyrate dehydrogenase, mitochondrial [B. mutus]
ENSP00000360275-D1	1.10	1.65E-28	↓	Disabled-like protein 1, partial [B. mutus]
ENSP00000332791-D1	1.10	5.30E-11	↓	kelch-like protein 15 [B. taurus]
ENSP00000322804-D1	1.10	2.21E-65	↓	metastasis suppressor protein 1 [B. taurus]
ENSBTAP00000044543-D1	1.10	4.03E-17	↓	homeobox protein notochord isoform X1 [B. taurus]
ENSP00000312419-D1	1.10	4.29E-58	↓	Chromodomain-helicase-DNA-binding protein 4 [B. mutus]
ENSP00000211998-D1	1.10	9.00E-44	↓	vinculin-like [B. mutus]
ENSBTAP00000011140-D1	1.10	4.30E-05	↓	E3 ubiquitin-protein ligase CCNB1IP1 [B. taurus]
ENSP00000379531-D1	1.10	4.30E-05	↓	Transmembrane channel-like protein 5, partial [B. mutus]
ENSP00000312435-D1	1.10	4.02E-13	↓	dystroglycan [B. mutus]
ENSP00000334409-D1	1.10	1.62E-24	↓	GMP reductase 2 isoform X2 [Homo sapiens]
ENSP00000295958-D1	1.10	2.37E-21	↓	small integral membrane protein 14 isoform X2 [B. taurus]
ENSP00000228922-D1	1.10	1.59E-03	↓	2-oxoglutarate and iron-dependent oxygenase domain-containing protein 2 [B. mutus]
ENSP00000264229-D1	1.10	1.45E-256	↓	Putative protein KIAA1211 [B. mutus]
ENSBTAP00000020074-D1	1.10	5.62E-29	↓	F-box only protein 9-like, partial [B. mutus]
ENSP00000378917-D1	1.10	7.91E-14	↓	ataxin-2-like protein isoform X1 [B. mutus]
ENSP00000315931-D1	1.10	1.20E-80	↓	Putative adenosylhomocysteinase 3, partial [B. mutus]
ENSP00000007699-D1	1.10	3.17E-23	↓	Y-box-binding protein 2, partial [B. mutus]
ENSBTAP00000024812-D1	1.10	2.24E-169	↓	V-type proton ATPase subunit B, brain isoform, partial [B. mutus]
ENSBTAP00000011642-D1	1.10	6.95E-11	↓	DDB1- and CUL4-associated factor 15, partial [B. mutus]
ENSBTAP00000001963-D1	1.11	1.81E-07	↓	Cytohesin-2 [B. mutus]
ENSBTAP00000008586-D1	1.11	2.85E-28	↓	CDK5 regulatory subunit-associated protein 2, partial [B. mutus]
ENSP00000301744-D1	1.11	2.35E-11	↓	TPA: zinc finger and SCAN domain containing 2-like [B. taurus]
ENSBTAP00000007488-D1	1.11	9.08E-04	↓	Mitogen-activated protein kinase kinase kinase 9, partial [B. mutus]
ENSBTAP00000050906-D7	1.11	9.08E-04	↓	Putative PRAME family member 24, partial [B. mutus]
ENSBTAP00000025255-D1	1.11	6.78E-120	↓	COP9 signalosome complex subunit 3 [B. taurus]
ENSBTAP00000008791-D60	1.11	8.28E-06	↓	zinc finger protein 271-like isoform X1 [B. taurus]

ENSP00000356848-D1	1.11	0.00E+00	↓	transcriptional adapter 1 [Bison bison bison]
ENSBTAP00000006361-D1	1.11	5.64E-29	↓	RING finger and SPRY domain-containing protein 1 [B. mutus]
ENSP00000313644-D1	1.11	4.25E-27	↓	Mitogen-activated protein kinase kinase kinase kinase 4, partial [B. mutus]
ENSP00000363116-D1	1.11	5.20E-04	↓	tyrosine-protein kinase Fgr [B. taurus]
ENSP00000376952-D1	1.11	5.20E-04	↓	crossover junction endonuclease EME1 [B. mutus]
ENSBTAP00000020032-D1	1.11	5.20E-04	↓	potassium/sodium hyperpolarization-activated cyclic nucleotide-gated channel 2 isoform X2 [Ovis aries musimon]
ENSBTAP00000017067-D1	1.11	2.87E-55	↓	E3 ubiquitin-protein ligase LRSAM1 [B. mutus]
ENSP00000260363-D1	1.11	0.00E+00	↓	Kinesin-like protein KIF23 [B. mutus]
ENSP00000376352-D1	1.11	1.66E-28	↓	[Pyruvate dehydrogenase [lipoamide]] kinase isozyme 1, mitochondrial, partial [B. mutus]
ENSBTAP00000007945-D1	1.11	2.12E-06	↓	dephospho-CoA kinase domain-containing protein [B. mutus]
ENSP00000276654-D1	1.11	3.95E-04	↓	low-density lipoprotein receptor-related protein 12 isoform X1 [B. mutus]
ENSP00000303525-D1	1.11	1.04E-44	↓	carbonyl reductase family member 4 [B. taurus]
ENSBTAP00000013484-D1	1.11	7.01E-21	↓	protein artemis [B. mutus]
ENSP00000339484-D1	1.11	3.52E-16	↓	AN1-type zinc finger and ubiquitin domain-containing protein 1 [B. mutus]
ENSBTAP00000024447-D1	1.11	1.09E-25	↓	Wiskott-Aldrich syndrome protein family member 2, partial [B. mutus]
ENSP00000344847-D1	1.11	1.04E-11	↓	A disintegrin and metalloproteinase with thrombospondin motifs 12 isoform X2 [B. mutus]
ENSP00000365931-D1	1.11	7.94E-12	↓	Transcription factor Sp2, partial [B. mutus]
ENSBTAP00000006129-D2	1.11	3.02E-179	↓	Ezrin, partial [B. mutus]
ENSBTAP00000020539-D1	1.11	1.27E-28	↓	Serine/threonine-protein phosphatase with EF-hands 2, partial [B. mutus]
ENSBTAP00000013304-D1	1.11	7.97E-45	↓	Low affinity cationic amino acid transporter 2 [B. mutus]
ENSBTAP00000053632-D1	1.11	4.30E-150	↓	Microtubule-associated tumor suppressor 1-like protein, partial [B. mutus]
ENSBTAP00000052274-D1	1.11	9.07E-13	↓	Transketolase-like protein 1 [B. mutus]
ENSP00000386401-D2	1.11	1.30E-04	↓	MAGUK p55 subfamily member 2 [B. mutus]
ENSBTAP00000013100-D1	1.11	1.30E-04	↓	POLR2A protein [Homo sapiens]
ENSBTAP00000032681-D1	1.12	1.81E-07	↓	Zinc finger protein Pegasus, partial [B. mutus]
ENSP00000345216-D1	1.12	2.48E-27	↓	Inactive ubiquitin carboxyl-terminal hydrolase 54 [B. mutus]
ENSBTAP00000019468-D1	1.12	1.63E-24	↓	Wiskott-Aldrich syndrome protein family member 3 [B. mutus]
ENSP00000380083-D1	1.12	4.62E-16	↓	nuclear receptor subfamily 2 group C member 2 [B. taurus]
ENSBTAP00000053335-D1	1.12	1.28E-30	↓	Myomegalin, partial [B. mutus]
ENSBTAP00000008906-D1	1.12	6.60E-34	↓	Mdm2-binding protein, partial [B. mutus]
ENSP00000349571-D1	1.12	2.01E-109	↓	LIM domain only protein 7, partial [B. mutus]
ENSBTAP00000042051-D1	1.12	7.47E-05	↓	ras-responsive element-binding protein 1 [B. mutus]
ENSP00000348429-D1	1.12	4.17E-23	↓	Long-chain-fatty-acid--CoA ligase 5, partial [B. mutus]
ENSBTAP00000018545-D1	1.12	3.27E-05	↓	Chain A, Crystal Structure Of The Human P21-Activated Kinase 4
ENSBTAP00000020395-D1	1.12	7.97E-45	↓	Spectrin alpha chain, brain [B. mutus]
ENSBTAP00000020711-D1	1.12	6.98E-09	↓	serine/threonine-protein kinase ICK isoform X2 [Bison bison bison]
ENSBTAP00000010661-D1	1.12	6.79E-219	↓	retinal dehydrogenase 1 [B. mutus]
ENSP00000362153-D1	1.12	1.97E-64	↓	Nucleolar GTP-binding protein 2 [B. mutus]
ENSP00000305426-D1	1.12	4.80E-24	↓	tubby protein homolog [B. mutus]
ENSBTAP00000024108-D1	1.12	4.05E-17	↓	activin receptor type-2A isoform X1 [Camelus ferus]
ENSP00000349029-D1	1.12	2.59E-37	↓	inositol 1,4,5-trisphosphate receptor type 1 isoform X3 [Ovis aries]
ENSP00000367721-D1	1.12	7.71E-39	↓	nuclear pore complex protein Nup160 [B. mutus]
ENSP00000382582-D1	1.12	1.20E-18	↓	Transmembrane protein ENSP00000382582, partial [B. mutus]
ENSBTAP00000020842-D1	1.12	9.05E-15	↓	elongation of very long chain fatty acids protein 3 [B. taurus]
ENSBTAP00000012819-D1	1.12	4.29E-29	↓	Protein RRP5-like protein, partial [B. mutus]
ENSP00000296484-D1	1.12	3.61E-84	↓	POC1 centriolar protein homolog A [B. taurus]
ENSBTAP00000022956-D1	1.12	2.89E-59	↓	Coilin, partial [B. mutus]
ENSP00000340510-D1	1.12	2.37E-07	↓	periplakin [Pantholops hodgsonii]
ENSBTAP00000003433-D1	1.12	1.29E-61	↓	zinc finger protein ZPR1 [B. taurus]
yakG028808	1.12	2.70E-18	↓	Microtubule-associated serine/threonine-protein kinase 4 [B. mutus]
ENSBTAP00000025170-D1	1.12	3.08E-79	↓	Ras-related protein Rab-25, partial [B. mutus]
ENSBTAP00000016302-D1	1.12	2.13E-24	↓	cip1-interacting zinc finger protein isoform X1 [B. mutus]
ENSBTAP00000013086-D1	1.13	4.33E-101	↓	protein N-terminal glutamine amidohydrolase isoform X1 [B. taurus]
ENSP00000366783-D1	1.13	2.93E-108	↓	probable E3 ubiquitin-protein ligase HECTD4 [B. mutus]
ENSP00000256682-D1	1.13	4.47E-39	↓	ADP-ribosylation factor 1 isoformX1 [Equus caballus]

ENSBTAP00000039583-D1	1.13	3.53E-08	↓	ribosomal RNA-processing protein 8 [B. mutus]
ENSP00000385276-D1	1.13	1.18E-14	↓	Teneurin-3, partial [B. mutus]
ENSP00000362909-D1	1.13	6.90E-15	↓	Protein NDRG3, partial [B. mutus]
ENSP00000310244-D1	1.13	2.06E-08	↓	RAS guanyl-releasing protein 1, partial [B. mutus]
ENSP00000351446-D2	1.13	2.40E-146	↓	WD repeat-containing protein 5 isoform X1 [Papio anubis]
ENSP00000377571-D1	1.13	1.20E-08	↓	dynactin subunit 1 isoform X3 [B. mutus]
ENSP00000381897-D1	1.13	4.49E-129	↓	Putative Polycomb group protein ASXL2, partial [B. mutus]
ENSP00000222005-D1	1.13	4.05E-09	↓	hsp90 co-chaperone Cdc37 [B. taurus]
ENSBTAP00000042180-D1	1.13	3.09E-46	↓	scaffold attachment factor B2 [Orcinus orca]
ENSP00000378294-D1	1.13	5.50E-25	↓	Nucleotide exchange factor SIL1, partial [B. mutus]
ENSP00000407130-D1	1.13	1.79E-17	↓	Zinc finger protein 410, partial [B. mutus]
ENSP00000371310-D1	1.13	4.28E-166	↓	Ubiquitin carboxyl-terminal hydrolase 7, partial [B. mutus]
ENSBTAP00000014699-D1	1.13	4.24E-166	↓	tRNA-splicing ligase RtcB-like protein [Camelus ferus]
ENSBTAP00000053340-D1	1.13	4.01E-142	↓	Transcription factor COE1, partial [B. mutus]
ENSP00000346500-D1	1.13	1.40E-50	↓	Limb region 1 protein-like protein, partial [B. mutus]
ENSP00000225972-D1	1.13	1.03E-15	↓	leucine-rich repeat-containing protein 59 [B. mutus]
ENSP00000367787-D1	1.13	2.08E-49	↓	DNA ligase 3 isoform X1 [B. mutus]
ENSBTAP00000029886-D1	1.13	4.63E-20	↓	follistatin-related protein 1 isoform X2 [Ovis aries musimon]
ENSBTAP00000006613-D2	1.13	8.57E-71	↓	Glutaredoxin-3, partial [B. mutus]
ENSP00000272418-D1	1.13	4.29E-33	↓	28S ribosomal protein S5, mitochondrial [B. taurus]
ENSBTAP00000010858-D1	1.13	2.68E-10	↓	dystrophin isoform X17 [Ovis aries musimon]
ENSP00000258772-D1	1.13	6.04E-10	↓	probable ATP-dependent RNA helicase DDX56 [B. taurus]
ENSBTAP00000051161-D1	1.13	7.92E-10	↓	zinc finger protein 346 [B. taurus]
ENSBTAP00000035807-D1	1.13	7.92E-10	↓	hypothetical protein LOC100423469 [Macaca mulatta]
ENSBTAP00000017032-D1	1.13	1.36E-09	↓	Syntaxin-3, partial [B. mutus]
ENSBTAP00000032740-D1	1.13	4.03E-09	↓	Zinc finger protein 79 [B. mutus]
ENSP00000378298-D1	1.13	6.95E-09	↓	Sodium-dependent serotonin transporter, partial [B. mutus]
ENSP00000367086-D1	1.13	6.95E-09	↓	Cytosolic acyl coenzyme A thioester hydrolase [B. mutus]
ENSP00000276204-D1	1.13	9.11E-09	↓	Dedicator of cytokinesis protein 11, partial [B. mutus]
ENSBTAP00000000857-D1	1.13	3.52E-08	↓	hypothetical protein M91_01746, partial [B. mutus]
ENSP00000365002-D1	1.13	4.63E-08	↓	abhydrolase domain-containing protein 16A [B. mutus]
ENSP00000301042-D1	1.13	2.77E-06	↓	TPA: zinc finger protein 286-like [B. taurus]
ENSP00000306625-D1	1.13	3.64E-06	↓	tether containing UBX domain for GLUT4 [B. mutus]
ENSP00000287773-D1	1.13	1.09E-05	↓	transmembrane protein 171 isoform X1 [B. mutus]
ENSP00000371230-D1	1.13	3.26E-05	↓	GTP:AMP phosphotransferase AK3, mitochondrial [B. taurus]
ENSP00000287996-D1	1.13	7.45E-05	↓	Inositol-pentakisphosphate 2-kinase [B. mutus]
ENSP00000407487-D1	1.13	9.82E-05	↓	protein unc-45 homolog A [B. mutus]
ENSP00000278829-D3	1.13	1.71E-04	↓	Fatty acid desaturase 3, partial [B. mutus]
ENSP00000370808-D2	1.13	9.07E-04	↓	ADP/ATP translocase 3 [B. taurus]
ENSP00000286353-D1	1.13	9.07E-04	↓	acid phosphatase-like protein 2 [B. mutus]
ENSP00000230732-D1	1.13	1.19E-03	↓	POU domain, class 4, transcription factor 3 [B. taurus]
ENSP00000409937-D1	1.13	1.19E-03	↓	Amyotrophic lateral sclerosis 2 chromosomal region candidate 11 protein [B. mutus]
ENSBTAP00000027366-D1	1.13	4.96E-03	↓	chondroitin sulfate synthase 2 [B. mutus]
ENSP00000367185-D1	1.13	6.59E-03	↓	ly6/PLAUR domain-containing protein 5 [B. mutus]
ENSP00000331625-D1	1.13	8.86E-03	↓	AP20 region protein 1, partial [B. mutus]
ENSP00000391692-D1	1.13	8.86E-03	↓	Coiled-coil domain-containing protein 103 [B. mutus]
ENSP00000419739-D1	1.13	1.18E-02	↓	Mitochondrial carrier-like protein 1, partial [B. mutus]
ENSP00000325827-D1	1.13	1.18E-02	↓	protein transport protein Sec16A [B. mutus]
ENSBTAP00000002745-D1	1.13	1.18E-02	↓	myosin-IIIa [B. mutus]
ENSBTAP00000020990-D1	1.13	1.18E-02	↓	sigma non-opioid intracellular receptor 1 isoform X2 [Ovis aries musimon]
ENSBTAP00000013931-D1	1.13	1.18E-02	↓	Thioredoxin domain-containing protein 8 [B. mutus]
ENSP00000312397-D1	1.13	1.61E-02	↓	Kelch-like protein 3, partial [B. mutus]
ENSBTAP00000031488-D1	1.13	1.61E-02	↓	nucleoredoxin-like protein 2 [B. mutus]
yakA06234	1.13	2.17E-02	↓	Growth hormone-regulated TBC protein 1 [B. mutus]
ENSBTAP00000001278-D1	1.13	2.17E-02	↓	serine/threonine-protein kinase TNNI3K [B. taurus]

ENSP00000276055-D1	1.13	2.17E-02	↓	carbohydrate sulfotransferase 7 [Ailuropoda melanoleuca]
ENSP00000375809-D1	1.13	2.17E-02	↓	TFIIH basal transcription factor complex helicase XPD subunit isoform X1 [Ovis aries]
ENSBTAP00000031442-D1	1.13	2.99E-02	↓	disintegrin and metalloproteinase domain-containing protein 32 [B. mutus]
ENSP00000320431-D1	1.13	2.99E-02	↓	sia-alpha-2,3-Gal-beta-1,4-GlcNAc-R:alpha 2,8-sialyltransferase [B. mutus]
ENSP00000338019-D1	1.13	2.99E-02	↓	DnaJ-like protein subfamily B member 2 [B. mutus]
ENSBTAP00000053460-D1	1.13	2.99E-02	↓	Spermatogenesis-associated protein 16, partial [B. mutus]
ENSBTAP00000040523-D1	1.13	4.17E-02	↓	Serine/threonine-protein phosphatase 6 regulatory subunit 1 [B. mutus]
ENSBTAP00000011456-D2	1.13	4.17E-02	↓	Sodium- and chloride-dependent glycine transporter 1, partial [B. mutus]
ENSP00000231021-D1	1.13	4.17E-02	↓	Cadherin-9 [B. mutus]
ENSBTAP00000012350-D1	1.13	4.17E-02	↓	uncharacterized protein C1orf167 homolog isoform X1 [B. taurus]
ENSP00000378605-D1	1.13	4.17E-02	↓	dnaJ homolog subfamily C member 30 [B. taurus]
ENSP00000233638-D1	1.13	4.17E-02	↓	T-cell leukemia homeobox protein 2 [Bubalus bubalis]
ENSP00000417303-D1	1.13	4.17E-02	↓	Krueppel-like factor 8 isoform X1 [B. taurus]
ENSP00000332967-D1	1.13	4.17E-02	↓	Putative solute carrier family 22 member ENSG00000182157, partial [B. mutus]
ENSP00000346659-D1	1.13	4.17E-02	↓	P protein, partial [B. mutus]
ENSBTAP00000012801-D1	1.14	0.00E+00	↓	Cat eye syndrome critical region protein 2, partial [B. mutus]
ENSP00000386992-D1	1.14	2.37E-52	↓	Importin-11, partial [B. mutus]
ENSBTAP00000007146-D1	1.14	0.00E+00	↓	Bcl-2-like protein 10 [B. mutus]
ENSP00000266987-D1	1.14	5.26E-19	↓	RISC-loading complex subunit TARBP2 [B. taurus]
ENSBTAP00000033778-D1	1.14	2.78E-65	↓	Peroxisome biogenesis factor 1, partial [B. mutus]
ENSBTAP00000004003-D1	1.14	1.18E-16	↓	5'-nucleotidase domain-containing protein 1 [B. mutus]
ENSP00000374147-D1	1.14	4.09E-56	↓	MBT domain-containing protein 1 [B. mutus]
ENSP00000343690-D1	1.14	2.63E-14	↓	dihydropyrimidinase-related protein 3 isoform X1 [B. mutus]
ENSP00000303909-D1	1.14	1.82E-25	↓	Active breakpoint cluster region-related protein, partial [B. mutus]
ENSP00000415682-D1	1.14	1.60E-222	↓	RING finger protein 10 [B. taurus]
ENSBTAP00000017259-D1	1.14	1.83E-27	↓	Phosphopantothenate--cysteine ligase, partial [B. mutus]
ENSP00000411825-D1	1.14	6.16E-26	↓	programmed cell death 6-interacting protein-like isoform X1 [B. mutus]
ENSBTAP00000045723-D1	1.14	1.19E-08	↓	Bromodomain-containing protein 9, partial [B. mutus]
ENSP00000358552-D1	1.14	6.02E-22	↓	DENN domain-containing protein 2C isoform X1 [B. taurus]
ENSP00000408617-D1	1.14	1.56E-08	↓	Histone deacetylase 9, partial [B. mutus]
ENSP00000364493-D1	1.14	8.91E-15	↓	hippocampus abundant transcript-like protein 1-like [B. mutus]
ENSP00000274008-D1	1.14	4.47E-134	↓	spermatogenesis-associated protein 5 [B. mutus]
ENSBTAP00000019878-D1	1.14	5.48E-78	↓	Trafficking kinesin-binding protein 2 [B. mutus]
ENSP00000369600-D1	1.15	3.77E-123	↓	Protein furry-like protein [B. mutus]
ENSBTAP00000051635-D2	1.15	2.35E-07	↓	histone H2A-Bbd type 1-like [B. mutus]
ENSBTAP00000038058-D1	1.15	2.56E-134	↓	ubiquitin carboxyl-terminal hydrolase 37 [B. taurus]
ENSBTAP00000022442-D1	1.15	0.00E+00	↓	DNA polymerase delta subunit 3, partial [B. mutus]
ENSP00000338160-D1	1.15	3.71E-99	↓	Peptidyl-prolyl cis-trans isomerase FKBP5 [B. mutus]
ENSP00000298923-D1	1.15	5.18E-21	↓	Sodium- and chloride-dependent glycine transporter 2, partial [B. mutus]
ENSP00000368797-D1	1.15	5.23E-11	↓	kelch repeat and BTB domain-containing protein 7 [B. taurus]
ENSP00000372924-D1	1.15	2.05E-75	↓	ralA-binding protein 1 [B. taurus]
ENSBTAP00000021144-D1	1.15	2.78E-99	↓	Nuclear receptor ROR-alpha, partial [B. mutus]
ENSBTAP00000011616-D1	1.15	1.19E-73	↓	Breast cancer type 1 susceptibility protein-like protein [B. mutus]
ENSP00000222145-D1	1.15	2.00E-18	↓	ras-interacting protein 1 [Otolemur garnettii]
ENSP00000323421-D1	1.15	2.65E-22	↓	structural maintenance of chromosomes protein 1A [B. taurus]
ENSP00000361699-D1	1.15	2.15E-42	↓	CTP synthase 1 isoform X2 [B. taurus]
ENSP00000274897-D1	1.15	7.84E-10	↓	hypothetical protein M91_00211, partial [B. mutus]
ENSBTAP00000002020-D1	1.15	1.42E-05	↓	BRI3-binding protein, partial [B. mutus]
ENSP00000222584-D1	1.15	1.87E-05	↓	Transcription factor Sp4, partial [B. mutus]
ENSP00000273612-D1	1.15	8.17E-224	↓	Protein VPRBP, partial [B. mutus]
ENSP00000331105-D1	1.15	1.18E-08	↓	thyroid adenoma-associated protein homolog [B. mutus]
ENSBTAP00000028683-D1	1.15	3.41E-18	↓	bifunctional apoptosis regulator [B. mutus]
ENSBTAP00000001744-D1	1.15	4.47E-18	↓	vacuolar protein sorting-associated protein 16 homolog isoform X1 [B. mutus]
yakA05597	1.15	2.64E-109	↓	Band 4.1-like protein 5 [B. mutus]
ENSP00000412045-D1	1.15	4.43E-71	↓	thioredoxin reductase 1, cytoplasmic [Canis lupus familiaris]

ENSP00000329127-D1	1.15	1.27E-54	↓	protein kinase C eta type [B. mutus]
ENSP00000246077-D1	1.15	6.00E-08	↓	TBC1 domain family member 20 [Bubalus bubalis]
ENSBTAP00000012393-D1	1.15	3.77E-58	↓	Putative protein KIAA1704, partial [B. mutus]
ENSP00000343435-D1	1.15	7.89E-08	↓	Protein YIF1B, partial [B. mutus]
ENSP00000356230-D1	1.15	1.73E-19	↓	Kelch-like protein 12 [B. mutus]
ENSBTAP00000042980-D1	1.16	4.03E-33	↓	sororin-like isoform X1 [B. mutus]
ENSBTAP00000006956-D1	1.16	9.76E-05	↓	serpin E3 [B. mutus]
ENSP00000388647-D1	1.16	1.66E-66	↓	DNA replication licensing factor MCM3 isoform X2 [Ovis aries]
ENSP00000267415-D1	1.16	2.21E-110	↓	TERF1-interacting nuclear factor 2 [B. taurus]
ENSP00000358921-D1	1.16	4.52E-26	↓	Alpha-centractin [Pteropus alecto]
ENSP00000357037-D1	1.16	4.52E-26	↓	Mediator of RNA polymerase II transcription subunit 23 [B. mutus]
ENSP00000312617-D1	1.16	2.35E-39	↓	nuclear transcription factor Y subunit gamma isoform X1 [B. mutus]
ENSBTAP00000001246-D3	1.16	7.76E-26	↓	pleckstrin homology domain-containing family B member 2 [B. mutus]
ENSP00000368972-D1	1.16	1.31E-17	↓	MAP7 domain-containing protein 2 [B. mutus]
ENSBTAP00000028680-D1	1.16	8.28E-66	↓	Calcium signal-modulating cyclophilin ligand, partial [B. mutus]
ENSP00000364621-D1	1.16	1.34E-09	↓	Sushi domain-containing protein 3, partial [B. mutus]
ENSBTAP00000027619-D1	1.16	2.30E-09	↓	Rho guanine nucleotide exchange factor 7, partial [B. mutus]
ENSP00000310170-D1	1.16	2.24E-04	↓	fos-related antigen 1 [B. taurus]
ENSBTAP00000040563-D1	1.16	1.01E-11	↓	Solute carrier family 2, facilitated glucose transporter member 1, partial [B. mutus]
ENSBTAP00000044440-D1	1.16	2.45E-101	↓	Rho guanine nucleotide exchange factor 12, partial [B. mutus]
ENSP00000324064-D75	1.16	3.48E-38	↓	Zinc finger protein 436, partial [B. mutus]
ENSP00000345629-D1	1.16	3.43E-30	↓	Mitogen-activated protein kinase kinase kinase 15, partial [B. mutus]
ENSBTAP00000047807-D1	1.16	3.13E-53	↓	Junctional adhesion molecule C, partial [B. mutus]
ENSBTAP00000019269-D1	1.16	2.52E-256	↓	Activating signal cointegrator 1, partial [B. mutus]
ENSP00000352541-D1	1.16	1.74E-31	↓	ubiquitin-associated protein 1 isoform X1 [B. mutus]
ENSP00000296003-D1	1.16	7.56E-18	↓	myotubularin-related protein 14 isoform X1 [B. taurus]
ENSBTAP00000036960-D1	1.16	5.76E-22	↓	mismatch repair endonuclease PMS2 [B. mutus]
ENSP00000363027-D1	1.16	8.85E-11	↓	mannose-1-phosphate guanyltransferase alpha isoform X2 [Pan paniscus]
ENSBTAP00000002877-D1	1.16	4.04E-188	↓	E3 ubiquitin-protein ligase UHRF1 [B. mutus]
ENSP00000418356-D1	1.16	2.73E-06	↓	ras-related protein M-Ras [Myotis davidii]
ENSP00000327154-D1	1.16	1.52E-10	↓	Transmembrane protein 63B [B. mutus]
ENSP00000265342-D1	1.16	2.64E-08	↓	Follistatin-related protein 4, partial [B. mutus]
ENSP00000357000-D1	1.16	3.46E-08	↓	upstream transcription factor 1 [Pantholops hodgsonii]
ENSP00000362144-D1	1.16	3.33E-16	↓	calcium uniporter protein, mitochondrial-like isoform X1 [Bubalus bubalis]
ENSBTAP00000018734-D1	1.16	7.21E-138	↓	Rho guanine nucleotide exchange factor 3, partial [B. mutus]
ENSBTAP00000014776-D1	1.17	3.95E-49	↓	Serine/threonine-protein kinase 38 [B. mutus]
ENSP00000334974-D1	1.17	8.15E-06	↓	hypothetical protein M91_13831, partial [B. mutus]
ENSP00000349351-D1	1.17	9.01E-04	↓	Protein bicaudal D-like protein 2 [B. mutus]
ENSBTAP00000018815-D1	1.17	9.01E-04	↓	Tubulin--tyrosine ligase, partial [B. mutus]
ENSP00000261349-D1	1.17	1.73E-53	↓	Low-density lipoprotein receptor-related protein 6, partial [B. mutus]
ENSP00000351284-D1	1.17	9.69E-22	↓	DNA repair protein RAD52-like protein [B. mutus]
ENSP00000363680-D1	1.17	1.85E-05	↓	Chain A, Crystal Structure Of Eda-A1
ENSP00000311905-D1	1.17	1.18E-03	↓	Latent-transforming growth factor beta-binding protein 4, partial [B. mutus]
ENSBTAP00000024112-D1	1.17	1.18E-03	↓	mitochondrial fission process protein 1 [B. taurus]
ENSBTAP00000014742-D1	1.17	3.73E-21	↓	Echinoderm microtubule-associated protein-like 6, partial [B. mutus]
ENSP00000222339-D1	1.17	5.24E-07	↓	Zinc finger protein 574, partial [B. mutus]
ENSP00000311344-D1	1.17	1.46E-30	↓	serine/threonine-protein phosphatase 2A 65 kDa regulatory subunit A beta isoform isoform X1 [B. mutus]
ENSBTAP00000026353-D1	1.17	6.10E-154	↓	cell division cycle-associated protein 3-like [B. mutus]
ENSP00000267012-D1	1.17	1.25E-63	↓	Bridging integrator 2, partial [B. mutus]
ENSBTAP00000003240-D6	1.17	1.99E-08	↓	cadherin-7-like, partial [B. mutus]
ENSBTAP00000023778-D1	1.17	1.19E-138	↓	T-complex protein 1 subunit epsilon, partial [B. mutus]
ENSBTAP00000003210-D1	1.17	1.90E-24	↓	Mitotic spindle assembly checkpoint protein MAD1 [B. mutus]
ENSBTAP00000032546-D1	1.17	1.70E-11	↓	Arrestin domain-containing protein 1, partial [B. mutus]
ENSBTAP00000006171-D1	1.17	6.53E-13	↓	Zinc finger protein 211, partial [B. mutus]
ENSBTAP00000026859-D1	1.17	1.00E-09	↓	NFX1-type zinc finger-containing protein 1 [B. mutus]

ENSP00000254190-D1	1.17	2.14E-51	↓	Chondroitin sulfate synthase 1, partial [B. mutus]
ENSP00000264932-D1	1.17	4.49E-08	↓	succinate dehydrogenase complex, subunit A, flavoprotein precursor [B. taurus]
ENSP00000351255-D1	1.17	5.57E-05	↓	Signal transducer and activator of transcription 4, partial [B. mutus]
ENSBTAP00000029455-D1	1.17	2.09E-03	↓	Serine/threonine-protein kinase PINK1, mitochondrial, partial [B. mutus]
ENSBTAP00000016216-D1	1.17	0.00E+00	↓	importin subunit alpha-1 [B. taurus]
ENSP00000361813-D1	1.18	7.34E-05	↓	protein BEX2 [B. taurus]
ENSBTAP00000010763-D1	1.18	1.29E-235	↓	Chromatin assembly factor 1 subunit A [B. mutus]
ENSP00000273980-D1	1.18	2.73E-45	↓	TBC domain-containing protein kinase-like protein isoform X1 [B. mutus]
ENSBTAP00000004002-D2	1.18	9.66E-05	↓	Segment polarity protein dishevelled-like protein DVL-3, partial [B. mutus]
ENSBTAP00000024623-D1	1.18	2.78E-03	↓	Tubby-related protein 1 [B. mutus]
ENSP00000212355-D1	1.18	5.23E-56	↓	transforming growth factor beta receptor type 3 [B. mutus]
ENSP00000346634-D1	1.18	1.13E-69	↓	thyroid hormone receptor-associated protein 3 isoform X1 [Capra hircus]
ENSBTAP00000051991-D1	1.18	1.19E-49	↓	consortin [B. mutus]
ENSP00000354227-D1	1.18	2.72E-33	↓	hypothetical protein M91_14016, partial [B. mutus]
ENSP00000324842-D1	1.18	8.44E-13	↓	acetoacetyl-CoA synthetase [B. mutus]
ENSP00000414712-D1	1.18	2.11E-19	↓	Ensconsin, partial [B. mutus]
ENSBTAP00000006004-D2	1.18	3.27E-170	↓	U4/U6 small nuclear ribonucleoprotein Prp4 isoform X2 [Trichechus manatus latirostris]
ENSBTAP00000015973-D1	1.18	3.01E-07	↓	ARF GTPase-activating protein GIT1, partial [B. mutus]
ENSBTAP00000019540-D1	1.18	2.67E-90	↓	putative ATP-dependent RNA helicase DHX57 [B. mutus]
ENSBTAP00000040765-D1	1.18	3.49E-119	↓	presenilin-1 isoform X1 [Bubalus bubalis]
ENSP00000414921-D1	1.18	1.34E-50	↓	polypyrimidine tract-binding protein 3 isoform X1 [B. taurus]
ENSP00000217121-D1	1.18	1.50E-94	↓	tumor protein D54 isoform X1 [B. taurus]
ENSP00000384406-D1	1.18	9.90E-10	↓	interferon regulatory factor 1 [B. taurus]
ENSBTAP00000017270-D1	1.18	3.94E-07	↓	tRNA pseudouridine synthase A, mitochondrial isoform X1 [B. taurus]
ENSBTAP00000005002-D1	1.18	1.68E-04	↓	R-spondin-1 [Bison bison bison]
ENSBTAP00000025116-D1	1.18	6.78E-42	↓	leucine-rich repeat and calponin homology domain-containing protein 3 [B. mutus]
ENSBTAP00000014941-D1	1.18	3.08E-30	↓	nuclear-interacting partner of ALK [B. mutus]
ENSBTAP00000020983-D100	1.18	5.33E-26	↓	zinc finger protein 583 isoform X3 [Ovis aries musimon]
ENSP00000365071-D1	1.18	6.66E-50	↓	G patch domain and ankyrin repeat-containing protein 1 [B. taurus]
ENSP00000399970-D3	1.18	4.63E-27	↓	serine/threonine-protein phosphatase 2A 55 kDa regulatory subunit B beta isoform isoform X2 [Cricetulus griseus]
ENSBTAP00000002792-D1	1.18	5.60E-12	↓	Zinc finger protein 771, partial [B. mutus]
ENSP00000370736-D1	1.18	3.38E-08	↓	Transmembrane protein 165, partial [B. mutus]
ENSBTAP00000004472-D1	1.18	1.48E-51	↓	Ubiquitin carboxyl-terminal hydrolase 46, partial [B. mutus]
ENSBTAP00000018349-D1	1.18	2.22E-04	↓	Pre-B-cell leukemia transcription factor 1, partial [Columba livia]
ENSBTAP00000013799-D1	1.19	2.92E-09	↓	Podocalyxin, partial [B. mutus]
ENSBTAP00000027006-D1	1.19	5.64E-53	↓	thiamine transporter 1 [B. mutus]
ENSBTAP00000020690-D1	1.19	5.96E-31	↓	glutamate--cysteine ligase catalytic subunit isoform X1 [B. taurus]
ENSBTAP00000053322-D1	1.19	1.83E-16	↓	DNA-directed RNA polymerase III subunit RPC2 [B. mutus]
ENSP00000357220-D1	1.19	1.66E-11	↓	Tetratricopeptide repeat protein 24 [B. mutus]
ENSP00000310126-D1	1.19	1.98E-74	↓	leucine-rich repeat and immunoglobulin-like domain-containing nogo receptor-interacting protein 2-like [B. mutus]
ENSBTAP00000053313-D1	1.19	7.65E-08	↓	major facilitator superfamily domain-containing protein 8 [B. mutus]
ENSP00000362014-D1	1.19	4.91E-03	↓	Dynamin-1 [B. mutus]
ENSBTAP00000050388-D1	1.19	4.91E-03	↓	Coiled-coil domain-containing protein 97, partial [B. mutus]
ENSP00000394472-D1	1.19	4.91E-03	↓	BTB/POZ domain-containing protein 18 [B. mutus]
ENSBTAP00000017626-D1	1.19	3.02E-26	↓	hepatoma-derived growth factor-related protein 2 [B. taurus]
ENSP00000383901-D2	1.19	4.42E-33	↓	Lysosomal acid lipase/cholesteryl ester hydrolase, partial [B. mutus]
ENSP00000256190-D1	1.19	3.09E-88	↓	Myotubularin-related protein 13, partial [B. mutus]
ENSBTAP00000045626-D1	1.19	8.47E-40	↓	putative sodium-coupled neutral amino acid transporter 8 [B. mutus]
ENSP00000251241-D1	1.19	9.07E-88	↓	probable ATP-dependent RNA helicase DHX40 isoform X1 [Bison bison bison]
ENSBTAP00000022240-D1	1.19	1.32E-07	↓	polynucleotide 5'-hydroxyl-kinase NOL9 [Bison bison bison]
ENSBTAP00000025969-D1	1.19	1.81E-16	↓	Immunoglobulin-like domain-containing receptor 2, partial [B. mutus]
ENSBTAP00000043469-D1	1.19	1.70E-30	↓	DNA excision repair protein ERCC-6 [B. mutus]
ENSBTAP00000006301-D1	1.19	1.94E-08	↓	protein Smaug homolog 2 isoform X1 [Bubalus bubalis]
ENSBTAP00000044604-D1	1.19	2.20E-09	↓	Charged multivesicular body protein 1a, partial [B. mutus]
ENSP00000319139-D1	1.19	2.54E-08	↓	Dual specificity mitogen-activated protein kinase kinase 3, partial [B. mutus]

ENSP00000313875-D1	1.19	2.54E-08	↓	Membrane cofactor protein, partial [B. mutus]
ENSBTAP00000043233-D1	1.19	3.50E-06	↓	major prion protein precursor [B. taurus]
ENSP00000222345-D1	1.19	4.17E-05	↓	Signal-induced proliferation-associated 1-like protein 3 [B. mutus]
ENSBTAP00000004887-D1	1.19	5.10E-04	↓	StAR-related lipid transfer protein 9, partial [B. mutus]
ENSBTAP00000017386-D1	1.19	1.84E-12	↓	Sorting nexin-19 [B. mutus]
ENSP00000372035-D2	1.20	1.75E-18	↓	serine/threonine-protein kinase LATS2 isoform X1 [B. taurus]
ENSP00000304151-D1	1.20	6.74E-04	↓	Ribosome biogenesis protein BOP1, partial [B. mutus]
ENSP00000388756-D1	1.20	5.04E-49	↓	C2 domain-containing protein 5 isoform X2 [B. mutus]
ENSBTAP00000000431-D1	1.20	6.49E-09	↓	ENTH domain-containing protein 1 [B. mutus]
ENSP00000288139-D1	1.20	1.29E-22	↓	voltage-dependent L-type calcium channel subunit alpha-1D isoform X1 [B. mutus]
ENSP00000221130-D2	1.20	3.61E-32	↓	glutathione reductase, mitochondrial-like [B. mutus]
ENSBTAP00000029285-D1	1.20	4.05E-227	↓	nesprin-2 [B. mutus]
ENSBTAP00000019737-D1	1.20	1.92E-21	↓	Y+L amino acid transporter 1 [B. mutus]
ENSP00000333363-D1	1.20	9.61E-10	↓	dynein heavy chain 8, axonemal isoform X5 [B. taurus]
ENSBTAP00000018177-D1	1.20	4.29E-25	↓	Oxidative stress-induced growth inhibitor 2, partial [B. mutus]
ENSP00000353427-D1	1.20	1.12E-08	↓	Nuclear receptor subfamily 4 group A member 1, partial [B. mutus]
ENSP00000380006-D1	1.20	1.54E-36	↓	Protein unc-13-like protein B, partial [B. mutus]
ENSP00000299866-D1	1.20	7.61E-40	↓	Protein FAM18A, partial [B. mutus]
ENSBTAP00000015257-D1	1.20	1.65E-09	↓	Inositol polyphosphate 5-phosphatase K, partial [B. mutus]
ENSP00000392466-D1	1.20	1.46E-08	↓	LIM domain-binding protein 1 isoform X1 [Cricetulus griseus]
ENSP00000406027-D1	1.20	1.04E-05	↓	EPM2A-interacting protein 1 [B. taurus]
ENSP00000282588-D1	1.20	8.88E-04	↓	integrin alpha-1 [B. mutus]
ENSBTAP00000006787-D6	1.20	8.76E-03	↓	hypothetical protein M91_20047, partial [B. mutus]
ENSBTAP00000002589-D1	1.20	8.76E-03	↓	HEAT repeat-containing protein 7B2, partial [B. mutus]
ENSBTAP00000033956-D1	1.20	8.76E-03	↓	high affinity immunoglobulin epsilon receptor subunit gamma precursor [B. taurus]
ENSP00000240587-D1	1.20	8.76E-03	↓	Teashirt-like protein 3, partial [B. mutus]
ENSBTAP00000001963-D2	1.20	3.39E-110	↓	Cytohesin-1, partial [B. mutus]
ENSBTAP00000017322-D1	1.20	7.55E-19	↓	volume-regulated anion channel subunit LRRC8A [B. taurus]
ENSP00000381821-D1	1.20	6.73E-16	↓	Protein yippee-like 3, partial [B. mutus]
ENSP00000377914-D1	1.20	6.82E-71	↓	anamorsin [B. mutus]
ENSP00000373657-D1	1.20	2.40E-216	↓	uncharacterized protein C17orf85 homolog [B. taurus]
ENSP00000276533-D1	1.20	2.49E-93	↓	DNA replication complex GINS protein SLD5 [B. mutus]
ENSP00000362330-D1	1.21	1.60E-45	↓	Chromodomain-helicase-DNA-binding protein 6, partial [B. mutus]
ENSBTAP00000020371-D1	1.21	1.26E-04	↓	non-syndromic hearing impairment protein 5 homolog [B. mutus]
ENSBTAP00000049093-D62	1.21	1.26E-04	↓	Olfactory receptor 4S1, partial [B. mutus]
ENSBTAP00000040146-D1	1.21	1.04E-48	↓	anaphase-promoting complex subunit 1 isoform X1 [Bison bison bison]
ENSBTAP00000032337-D1	1.21	1.04E-12	↓	uncharacterized protein LOC102275278 [B. mutus]
ENSBTAP00000002861-D1	1.21	9.55E-21	↓	Protein LAS1-like protein [B. mutus]
ENSP00000219476-D1	1.21	3.28E-08	↓	tuberin [B. mutus]
ENSP00000366509-D10	1.21	1.17E-03	↓	zinc finger protein 160-like isoform X1 [Ovis aries]
ENSBTAP00000010013-D1	1.21	1.61E-90	↓	Bardet-Biedl syndrome 4 protein homolog [B. mutus]
ENSBTAP00000020850-D1	1.21	9.71E-19	↓	transmembrane and coiled-coil domain-containing protein 3 [B. mutus]
ENSBTAP00000006514-D1	1.21	1.50E-32	↓	ETS domain-containing protein Elk-4, partial [B. mutus]
ENSBTAP00000021257-D1	1.21	6.38E-09	↓	Seizure protein 6-like protein, partial [B. mutus]
ENSP00000238018-D1	1.21	9.87E-17	↓	guanine deaminase [B. mutus]
ENSP00000359290-D1	1.21	6.26E-22	↓	protein Dr1 [Jaculus jaculus]
ENSBTAP00000043465-D1	1.21	2.75E-58	↓	Protein MRVI1 [B. mutus]
ENSP00000383623-D1	1.21	1.47E-17	↓	afadin-like [B. mutus]
ENSP00000312999-D5	1.21	1.65E-04	↓	guanine nucleotide-binding protein G(o) subunit alpha isoform X1 [B. mutus]
ENSBTAP00000042066-D1	1.21	2.32E-76	↓	protein FAM126B isoform X1 [Bison bison bison]
ENSBTAP00000024222-D1	1.21	8.36E-09	↓	zinc finger and BTB domain-containing protein 8A [B. mutus]
ENSP00000350704-D1	1.21	2.37E-05	↓	uncharacterized protein C1orf109 homolog [B. mutus]
ENSBTAP00000027371-D1	1.21	4.56E-13	↓	B-cell CLL/lymphoma 7 protein family member B, partial [B. mutus]
ENSP00000351310-D1	1.21	3.45E-06	↓	collagen alpha-6(VI) chain isoform X2 [B. taurus]
ENSBTAP00000053506-D1	1.21	4.15E-81	↓	DNA mismatch repair protein Mlh3 [B. mutus]

ENSP00000362904-D1	1.21	0.00E+00	↓	protein 4.1 isoform X1 [B. mutus]
ENSBTAP00000022977-D1	1.21	4.54E-41	↓	General transcription factor 3C polypeptide 2 [B. mutus]
ENSBTAP00000007484-D1	1.21	2.05E-24	↓	Protein DBF4-like protein B, partial [B. mutus]
ENSBTAP00000017205-D1	1.21	3.33E-15	↓	protein prune homolog 2 [B. mutus]
ENSBTAP00000009343-D1	1.21	3.14E-10	↓	Periodic tryptophan protein 2-like protein, partial [B. mutus]
ENSP00000384932-D1	1.21	2.18E-04	↓	G-protein coupled receptor 12 [B. taurus]
ENSBTAP00000001982-D1	1.21	2.18E-04	↓	Serine/threonine-protein kinase PDIK1L, partial [B. mutus]
ENSP00000395886-D3	1.21	1.55E-03	↓	40S ribosomal protein S28-like [B. mutus]
ENSBTAP00000008106-D1	1.21	1.17E-02	↓	Rab11 family-interacting protein 5, partial [B. mutus]
ENSBTAP00000010628-D1	1.21	1.99E-13	↓	protein sel-1 homolog 1 [B. mutus]
ENSBTAP00000006739-D2	1.21	6.12E-11	↓	bone morphogenetic protein 4 precursor [Bubalus bubalis]
ENSP00000343204-D1	1.22	6.10E-166	↓	tyrosine-protein kinase JAK1 [B. taurus]
ENSBTAP00000018248-D1	1.22	6.84E-90	↓	uridine 5'-monophosphate synthase [B. mutus]
ENSP00000398026-D1	1.22	2.20E-29	↓	Beta-hexosaminidase subunit alpha [B. mutus]
ENSBTAP00000015397-D1	1.22	5.05E-14	↓	dual specificity protein phosphatase CDC14B isoform X3 [Bubalus bubalis]
ENSBTAP00000021843-D1	1.22	4.10E-05	↓	matrix metalloproteinase-25 [B. mutus]
ENSP00000391440-D1	1.22	4.10E-05	↓	polyhomeotic-like protein 2 [B. mutus]
ENSP00000381044-D1	1.22	1.63E-70	↓	Ubiquitin carboxyl-terminal hydrolase 10, partial [B. mutus]
ENSBTAP00000025331-D1	1.22	2.87E-04	↓	ras-associating and dilute domain-containing protein [B. mutus]
ENSP00000216605-D1	1.22	3.37E-198	↓	C-1-tetrahydrofolate synthase, cytoplasmic, partial [B. mutus]
ENSBTAP00000006322-D1	1.22	2.66E-11	↓	Type I inositol-3,4-bisphosphate 4-phosphatase [B. mutus]
ENSP00000401512-D55	1.22	1.49E-13	↓	Zinc finger protein 37A, partial [B. mutus]
ENSP00000343741-D1	1.22	1.10E-39	↓	serine/threonine-protein kinase ATR [B. mutus]
ENSBTAP00000025762-D1	1.22	3.80E-155	↓	RFK protein [B. taurus]
ENSBTAP00000009395-D1	1.22	1.01E-34	↓	WD repeat and SOCS box-containing protein 2 isoform X1 [B. mutus]
ENSBTAP00000006686-D1	1.22	2.86E-07	↓	Zinc finger HIT domain-containing protein 2 [B. mutus]
ENSBTAP00000046521-D1	1.22	5.78E-141	↓	THO complex subunit 3 [B. mutus]
ENSBTAP00000010863-D1	1.22	3.08E-10	↓	Dystrophin, partial [B. mutus]
ENSBTAP00000027892-D1	1.22	1.86E-15	↓	RING finger protein 166, partial [B. mutus]
ENSBTAP00000004398-D1	1.22	8.39E-49	↓	Splicing factor 3A subunit 1, partial [B. mutus]
ENSBTAP00000009341-D1	1.22	0.00E+00	↓	tissue factor precursor [B. taurus]
ENSP00000336528-D1	1.22	7.85E-11	↓	nuclear receptor subfamily 1 group I member 2, partial [Capra hircus]
ENSBTAP00000049510-D1	1.22	7.10E-05	↓	Phospholipid scramblase 3, partial [B. mutus]
ENSBTAP00000011396-D1	1.22	2.21E-34	↓	hypothetical protein M91_04433, partial [B. mutus]
ENSP00000184956-D1	1.22	1.41E-08	↓	HEAT repeat-containing protein 6-like [B. mutus]
ENSBTAP00000004559-D1	1.22	1.41E-08	↓	glutathione synthetase [B. mutus]
ENSBTAP00000018852-D1	1.22	2.62E-42	↓	Solute carrier family 12 member 5, partial [B. mutus]
ENSBTAP00000008020-D1	1.22	2.09E-80	↓	X-ray repair cross-complementing protein 6 [B. mutus]
ENSBTAP00000011104-D1	1.23	5.49E-15	↓	AP-5 complex subunit sigma-1 [B. taurus]
ENSP00000394394-D1	1.23	4.48E-100	↓	anaphase-promoting complex subunit 7 [B. mutus]
ENSP00000350558-D1	1.23	3.27E-55	↓	CDK5 regulatory subunit-associated protein 1 [B. mutus]
ENSP00000278412-D2	1.23	2.09E-36	↓	FACT complex subunit SSRP1 isoform X2 [Bubalus bubalis]
ENSP00000311697-D1	1.23	1.91E-13	↓	fibroblast growth factor 5 [B. mutus]
ENSP00000368848-D1	1.23	5.31E-17	↓	Synphilin-1, partial [B. mutus]
ENSBTAP00000017878-D1	1.23	6.10E-27	↓	hypothetical protein M91_14072, partial [B. mutus]
ENSP00000293677-D1	1.23	5.00E-04	↓	ribonucleoprotein PTB-binding 1 isoform X1 [B. mutus]
ENSP00000262731-D1	1.23	1.58E-02	↓	Rho GTPase-activating protein 8, partial [B. mutus]
ENSBTAP00000001547-D1	1.23	1.58E-02	↓	core histone macro-H2A.2-like isoform X1 [Ictidomys tridecemlineatus]
ENSBTAP00000052650-D1	1.23	1.58E-02	↓	Alkylglycerol monooxygenase, partial [B. mutus]
ENSP00000242576-D1	1.23	1.38E-46	↓	uracil-DNA glycosylase [B. mutus]
yakG028650	1.23	1.26E-21	↓	Heterogeneous nuclear ribonucleoprotein U-like protein 1 [B. mutus]
ENSBTAP00000034708-D1	1.23	4.48E-67	↓	protein HEXIM2 isoform X1 [B. mutus]
yakG041173	1.23	8.66E-39	↓	Protein phosphatase 1 regulatory subunit 15A [B. mutus]
ENSBTAP00000051129-D1	1.23	2.19E-12	↓	Nuclear receptor subfamily 6 group A member 1, partial [B. mutus]
ENSP00000295050-D1	1.23	7.38E-40	↓	sprT-like domain-containing protein Spartan isoform X1 [B. mutus]

ENSP00000378320-D1	1.23	2.32E-05	↓	Tetratricopeptide repeat protein 7A, partial [B. mutus]
ENSP00000309431-D15	1.23	3.06E-204	↓	Tubulin alpha-1C chain, partial [B. mutus]
ENSP00000351646-D1	1.23	1.60E-150	↓	Stress-induced-phosphoprotein 1, partial [B. mutus]
ENSP00000265168-D2	1.23	1.95E-56	↓	hydroxyacyl-coenzyme A dehydrogenase, mitochondrial isoform X2 [B. mutus]
ENSBTAP00000016093-D1	1.23	1.23E-04	↓	Rab GDP dissociation inhibitor alpha, partial [B. mutus]
ENSBTAP00000053783-D1	1.23	1.69E-263	↓	Kinesin-like protein KIF15, partial [B. mutus]
ENSP00000374218-D1	1.23	4.93E-12	↓	extended synaptotagmin-3 [B. mutus]
ENSBTAP00000022720-D1	1.23	6.61E-04	↓	Growth factor receptor-bound protein 10, partial [B. mutus]
ENSBTAP00000019756-D1	1.24	4.30E-270	↓	zona pellucida sperm-binding protein 3 [B. mutus]
ENSP00000373772-D1	1.24	3.31E-242	↓	E3 ubiquitin-protein ligase BRE1A [B. mutus]
ENSP00000321874-D1	1.24	1.80E-22	↓	UDP-GlcNAc:betaGal beta-1,3-N-acetylglucosaminyltransferase 3 [B. mutus]
ENSBTAP00000005281-D1	1.24	1.07E-29	↓	Putative ATP-dependent RNA helicase DHX35, partial [B. mutus]
ENSBTAP00000000929-D1	1.24	3.49E-09	↓	dual specificity protein phosphatase 16 isoform X1 [Ovis aries]
ENSP00000398244-D1	1.24	4.90E-19	↓	hsp70-binding protein 1 [B. taurus]
ENSBTAP00000005760-D1	1.24	4.19E-70	↓	poly(A) RNA polymerase, mitochondrial, partial [B. mutus]
ENSBTAP00000008887-D1	1.24	7.61E-06	↓	6-phosphofructo-2-kinase/fructose-2,6-biphosphatase 4, partial [B. mutus]
ENSBTAP00000022364-D1	1.24	7.61E-06	↓	Pyrroline-5-carboxylate reductase 3, partial [B. mutus]
ENSBTAP00000003434-D1	1.24	3.61E-03	↓	methyltransferase-like protein 7A-like [B. mutus]
ENSP00000369739-D1	1.24	1.11E-11	↓	valacyclovir hydrolase [B. mutus]
ENSBTAP00000013140-D1	1.24	1.69E-17	↓	MORC family CW-type zinc finger protein 2 isoform X2 [Bison bison bison]
ENSBTAP00000020579-D1	1.24	4.00E-05	↓	CD2 antigen cytoplasmic tail-binding protein 2, partial [B. mutus]
ENSP00000346112-D1	1.24	1.81E-117	↓	Solute carrier family 12 member 6 [B. mutus]
ENSBTAP00000018481-D1	1.24	3.04E-91	↓	Putative glutamyl-tRNA(Gln) amidotransferase subunit B, mitochondrial [B. mutus]
ENSBTAP00000019429-D1	1.24	4.80E-07	↓	EF-hand domain-containing protein D1 isoform X1 [Bubalus bubalis]
ENSBTAP00000027029-D1	1.24	4.61E-14	↓	dual specificity phosphatase 5 [B. taurus]
ENSP00000415822-D1	1.24	8.69E-04	↓	ataxin-1-like [B. taurus]
ENSP00000363689-D1	1.24	4.40E-294	↓	DNA-binding protein inhibitor ID-3 [B. mutus]
ENSBTAP00000053647-D1	1.24	3.85E-10	↓	Iron-sulfur protein NUBPL, partial [B. mutus]
ENSBTAP00000007976-D1	1.24	4.84E-49	↓	src substrate protein p85 isoform X1 [B. mutus]
ENSP00000378090-D2	1.24	2.51E-06	↓	DNA repair protein RAD51-like protein 4, partial [B. mutus]
ENSBTAP00000044021-D1	1.24	1.26E-17	↓	Tetratricopeptide repeat protein 23-like protein, partial [B. mutus]
ENSP00000399734-D1	1.24	1.26E-17	↓	unnamed protein product [Homo sapiens]
ENSP00000298428-D2	1.24	6.71E-22	↓	protein transport protein Sec61 subunit alpha isoform 1-like protein [Cricetulus griseus]
ENSP00000297990-D1	1.24	6.29E-07	↓	nucleolar protein 6 isoform X1 [B. mutus]
ENSP00000355648-D1	1.24	1.34E-20	↓	hypothetical protein M91_02777 [B. mutus]
ENSBTAP00000021469-D1	1.24	1.28E-10	↓	ADP-ribosylation factor-binding protein GGA3, partial [B. mutus]
ENSP00000318247-D1	1.24	1.85E-25	↓	Activating molecule in BECN1-regulated autophagy protein 1 [B. mutus]
ENSBTAP00000015194-D1	1.25	5.27E-05	↓	Non-lysosomal glucosylceramidase, partial [B. mutus]
ENSP00000286307-D1	1.25	3.29E-06	↓	U7 snRNA-associated Sm-like protein LSm11 [B. mutus]
ENSBTAP00000006875-D1	1.25	6.40E-24	↓	serine/threonine-protein kinase WNK1, partial [Bison bison bison]
ENSP00000404545-D3	1.25	2.50E-65	↓	Scaffold attachment factor B1, partial [B. mutus]
ENSBTAP00000052414-D1	1.25	1.08E-11	↓	hypothetical protein M91_03971, partial [B. mutus]
ENSBTAP00000015785-D1	1.25	2.08E-07	↓	Glutamate receptor-interacting protein 2, partial [B. mutus]
ENSBTAP00000001378-D1	1.25	2.08E-07	↓	Mitogen-activated protein kinase kinase kinase 2, partial [B. mutus]
ENSBTAP00000022087-D1	1.25	2.08E-07	↓	uncharacterized protein LOC786914 [B. taurus]
ENSP00000387546-D1	1.25	2.08E-07	↓	zinc finger protein 438 [B. mutus]
ENSBTAP00000022223-D1	1.25	1.14E-14	↓	Centrosomal protein of 68 kDa, partial [B. mutus]
ENSBTAP00000014187-D1	1.25	0.00E+00	↓	39S ribosomal protein L37, mitochondrial [B. mutus]
ENSP00000362578-D1	1.25	0.00E+00	↓	E3 ubiquitin-protein ligase RNF8 [B. mutus]
ENSBTAP00000026919-D1	1.25	4.02E-46	↓	leucine-rich repeat-containing protein 8B [B. mutus]
ENSP00000394485-D1	1.25	6.93E-05	↓	Na(+)/H(+) exchange regulatory cofactor NHE-RF3 [B. mutus]
ENSBTAP00000009823-D1	1.25	4.82E-03	↓	gremlin-1 isoform X2 [Ovis aries musimon]
ENSP00000298440-D1	1.25	4.82E-03	↓	2-oxoglutarate receptor 1 [B. mutus]
ENSP00000016171-D1	1.25	4.44E-09	↓	cytochrome c oxidase assembly protein COX15 homolog [B. mutus]
ENSP00000307260-D1	1.25	4.31E-06	↓	Transducin beta-like protein 2 [B. mutus]

ENSBTAP00000010606-D1	1.25	1.72E-05	↓	peroxisome proliferator-activated receptor alpha [B. taurus]
ENSP00000316674-D10	1.25	1.15E-03	↓	Actin-like protein 8, partial [B. mutus]
ENSP00000365198-D1	1.25	2.13E-02	↓	kazrin isoform X4 [B. taurus]
ENSP00000270474-D1	1.25	2.13E-02	↓	cAMP-specific 3',5'-cyclic phosphodiesterase 4A [B. mutus]
ENSBTAP00000004354-D1	1.25	2.13E-02	↓	Sodium-dependent noradrenaline transporter, partial [B. mutus]
ENSBTAP00000016858-D1	1.25	2.13E-02	↓	insulin receptor isoform X8 [B. taurus]
ENSBTAP00000043987-D1	1.25	2.13E-02	↓	Mothers against decapentaplegic-like protein 7, partial [B. mutus]
ENSP00000303316-D1	1.25	2.13E-02	↓	piezo-type mechanosensitive ion channel component 2 isoform X1 [B. taurus]
ENSP00000299543-D1	1.25	2.13E-02	↓	RNA polymerase II subunit A C-terminal domain phosphatase isoformX1 [Canis lupus familiaris]
ENSBTAP00000024963-D1	1.25	5.41E-44	↓	Protein FAM117B, partial [B. mutus]
ENSP00000397107-D1	1.25	2.42E-11	↓	LETM1 domain-containing protein 1 [B. mutus]
ENSBTAP00000028046-D1	1.25	7.00E-63	↓	ATP-dependent RNA helicase DDX42 [B. mutus]
ENSP00000352272-D1	1.25	1.37E-30	↓	Myozenin-1 [B. mutus]
ENSP00000333367-D2	1.25	2.03E-57	↓	DNA-binding protein SATB1, partial [B. mutus]
ENSP00000356595-D1	1.25	8.03E-12	↓	Abelson tyrosine-protein kinase 2 isoform X1 [Bison bison bison]
ENSP00000365210-D1	1.25	0.00E+00	↓	G2/mitotic-specific cyclin-B3, partial [B. mutus]
ENSBTAP00000013429-D1	1.26	2.26E-05	↓	Vesicle transport protein GOT1A, partial [B. mutus]
ENSBTAP00000053301-D1	1.26	5.18E-18	↓	Protein CLEC16A, partial [B. mutus]
ENSP00000363036-D1	1.26	2.99E-08	↓	F-box/WD repeat-containing protein 2 [Pteropus alecto]
ENSP00000190165-D1	1.26	9.08E-05	↓	Doublesex- and mab-3-related transcription factor 3, partial [B. mutus]
ENSBTAP00000014256-D1	1.26	6.78E-18	↓	Opa interacting protein 5 [B. mutus]
ENSP00000361701-D1	1.26	4.13E-28	↓	HSPC109 [Homo sapiens]
ENSP00000343445-D1	1.26	4.67E-07	↓	Serpin B4, partial [B. mutus]
ENSP00000335486-D1	1.26	1.10E-79	↓	G patch domain-containing protein 8, partial [B. mutus]
ENSP00000317786-D1	1.26	1.05E-16	↓	Myosin phosphatase Rho-interacting protein, partial [B. mutus]
ENSP00000310729-D1	1.26	3.15E-21	↓	homocysteine-responsive endoplasmic reticulum-resident ubiquitin-like domain member 2 protein [B. taurus]
ENSBTAP00000018029-D1	1.26	3.70E-04	↓	Guanine nucleotide exchange factor MSS4, partial [B. mutus]
ENSBTAP00000001142-D1	1.26	3.70E-04	↓	solute carrier family 52, riboflavin transporter, member 2 [B. mutus]
ENSBTAP00000002653-D1	1.26	1.86E-160	↓	Tyrosine-protein phosphatase non-receptor type 11, partial [B. mutus]
ENSP00000281527-D1	1.26	2.13E-10	↓	Cathepsin O, partial [B. mutus]
ENSP00000358314-D1	1.26	8.38E-10	↓	LIX1-like protein [B. taurus]
ENSBTAP00000000107-D1	1.26	5.69E-77	↓	Histone-lysine N-methyltransferase SETDB1, partial [B. mutus]
ENSP00000374118-D1	1.26	5.39E-106	↓	Serine/threonine-protein kinase SMG1, partial [B. mutus]
ENSP00000345656-D1	1.26	7.12E-178	↓	Vesicle-associated membrane protein-associated protein A, partial [B. mutus]
ENSBTAP00000025179-D1	1.26	3.30E-09	↓	Inosine-5'-monophosphate dehydrogenase 2, partial [B. mutus]
ENSP00000387128-D1	1.26	1.76E-74	↓	Nck-associated protein 5, partial [B. mutus]
ENSP00000368976-D1	1.26	2.77E-37	↓	kinesin-like protein KIFC3 isoform X1 [B. mutus]
ENSP00000406218-D1	1.26	2.00E-39	↓	protein PROCA1 [B. mutus]
ENSP00000302898-D1	1.26	1.11E-145	↓	Serine/threonine-protein kinase 13, partial [B. mutus]
ENSBTAP00000002373-D1	1.26	3.64E-10	↓	Rho guanine nucleotide exchange factor 5, partial [B. mutus]
ENSBTAP00000027497-D1	1.26	7.63E-83	↓	RNA-binding protein NOB1 [B. mutus]
ENSBTAP00000019665-D1	1.26	1.70E-08	↓	5'-nucleotidase domain-containing protein 2 [B. mutus]
yakG015983	1.26	4.47E-22	↓	AMY-1-associating protein expressed in testis 1 [B. mutus]
ENSP00000408263-D1	1.26	2.48E-88	↓	selenium-binding protein 1 [B. mutus]
ENSBTAP00000030362-D1	1.26	0.00E+00	↓	Synaptotagmin-like protein 2 [B. mutus]
ENSBTAP00000041778-D1	1.26	1.56E-15	↓	Glutamine synthetase, partial [B. mutus]
ENSP00000293805-D1	1.26	8.05E-07	↓	B-cell CLL/lymphoma 6 member B protein [B. mutus]
ENSBTAP00000007277-D1	1.26	6.70E-08	↓	Serine/threonine-protein phosphatase 2A 56 kDa regulatory subunit delta isoform, partial [B. mutus]
ENSP00000271628-D1	1.26	6.63E-28	↓	splicing factor 3B subunit 4 [Oryzias latipes]
yakG039592	1.27	7.39E-56	↓	Eukaryotic translation initiation factor 3 subunit B [B. mutus]
ENSP00000309751-D1	1.27	4.03E-11	↓	monocarboxylate transporter 13 [B. mutus]
ENSP00000333374-D1	1.27	3.68E-40	↓	coiled-coil domain-containing protein 60 [Bison bison bison]
ENSBTAP00000006505-D1	1.27	4.00E-26	↓	Bardet-Biedl syndrome 7 protein [B. taurus]
ENSP00000405275-D1	1.27	3.89E-05	↓	proline-rich protein 3 isoform X2 [Pantholops hodgsonii]
ENSBTAP00000053800-D1	1.27	3.89E-05	↓	testis development-related protein-like [B. mutus]

ENSBTAP00000041167-D1	1.27	4.86E-04	↓	Extended synaptotagmin-2, partial [B. mutus]
ENSP00000405798-D1	1.27	4.86E-04	↓	Cell division protein kinase 16, partial [B. mutus]
ENSP00000395892-D1	1.27	4.86E-04	↓	protein FAM118A isoform X1 [B. mutus]
ENSBTAP00000040188-D1	1.27	6.36E-03	↓	BTB/POZ domain-containing protein KCTD2 [Ceratotherium simum simum]
ENSBTAP00000021621-D1	1.27	6.36E-03	↓	Glucose-6-phosphatase 3, partial [B. mutus]
ENSBTAP00000040537-D1	1.27	6.36E-03	↓	epsin-1 isoform X2 [B. taurus]
ENSP00000416040-D1	1.27	1.05E-14	↓	UPF0606 protein KIAA1549, partial [B. mutus]
ENSBTAP00000042659-D1	1.27	1.05E-06	↓	Nucleoside diphosphate-linked moiety X motif 19, mitochondrial, partial [B. mutus]
ENSBTAP00000013968-D1	1.27	1.27E-05	↓	Dehydrogenase/reductase SDR family member 7B, partial [B. mutus]
ENSBTAP00000011371-D1	1.27	2.98E-122	↓	cAMP-dependent protein kinase type I-alpha regulatory subunit, partial [B. mutus]
ENSBTAP00000000686-D1	1.27	8.43E-96	↓	DNA topoisomerase 2-binding protein 1 [B. taurus]
ENSBTAP00000040972-D1	1.27	1.57E-04	↓	Mesoderm induction early response protein 2, partial [B. mutus]
ENSP00000359956-D1	1.27	3.82E-31	↓	elongation of very long chain fatty acids protein 5-like [B. mutus]
ENSP00000349588-D1	1.27	2.20E-84	↓	Ankyrin-2, partial [B. mutus]
ENSP00000353410-D11	1.27	2.01E-03	↓	zinc finger protein 347 isoform X1 [B. taurus]
ENSBTAP00000002648-D1	1.27	4.85E-41	↓	NAD-dependent protein deacetylase sirtuin-3, mitochondrial [B. mutus]
ENSBTAP00000017058-D1	1.27	2.43E-17	↓	Retinol dehydrogenase 12, partial [B. mutus]
ENSP00000222305-D1	1.27	4.53E-07	↓	Upstream stimulatory factor 2, partial [B. mutus]
ENSBTAP00000003467-D1	1.28	2.07E-36	↓	SUMO-activating enzyme subunit 1 [B. taurus]
ENSP00000367462-D1	1.28	6.41E-04	↓	S-acyl fatty acid synthase thioesterase, medium chain, partial [B. mutus]
ENSBTAP00000027737-D1	1.28	0.00E+00	↓	E3 ubiquitin-protein ligase UHRF2 [B. mutus]
ENSBTAP00000015698-D1	1.28	1.53E-10	↓	AT-rich interactive domain-containing protein 3B isoform X1 [Bison bison bison]
ENSP00000361433-D1	1.28	4.84E-80	↓	exosome complex component RRP4 [Ovis aries]
ENSBTAP00000029531-D1	1.28	1.69E-11	↓	Sodium/potassium/calcium exchanger 6 [B. mutus]
ENSBTAP00000035818-D1	1.28	3.02E-116	↓	TPA: scaffold attachment factor B [B. taurus]
ENSBTAP00000017015-D1	1.28	7.17E-06	↓	protein KIAA1045 homolog [B. taurus]
ENSBTAP00000004550-D1	1.28	2.37E-06	↓	DDB1- and CUL4-associated factor 7 isoform X1 [Chinchilla lanigera]
ENSBTAP00000044633-D1	1.28	1.67E-146	↓	steroidogenic acute regulatory protein, mitochondrial [B. mutus]
ENSP00000341380-D1	1.28	2.56E-07	↓	hypothetical protein M91_15523, partial [B. mutus]
ENSBTAP00000016027-D1	1.28	4.26E-49	↓	CCR4-NOT transcription complex subunit 8 isoform X2 [Monodelphis domestica]
ENSBTAP00000021109-D1	1.28	3.38E-84	↓	ubiquitin-conjugating enzyme E2 J2 isoform X2 [Capra hircus]
ENSP00000379474-D1	1.28	1.25E-11	↓	Tyrosine-protein phosphatase non-receptor type 5, partial [B. mutus]
ENSBTAP00000021600-D1	1.29	4.91E-27	↓	Ribonuclease H1, partial [B. mutus]
ENSBTAP00000029169-D2	1.29	1.93E-26	↓	AP-3 complex subunit mu-2 isoform X2 [B. mutus]
ENSBTAP00000031483-D1	1.29	6.41E-27	↓	Tribbles-like protein 1 [B. mutus]
ENSBTAP00000020219-D1	1.29	2.63E-29	↓	Niemann-Pick C1 protein, partial [B. mutus]
ENSBTAP00000000160-D1	1.29	1.11E-07	↓	N-lysine methyltransferase SETD8 [Bison bison bison]
ENSP00000300127-D22	1.29	9.39E-06	↓	Olfactory receptor 4B1 [B. mutus]
ENSBTAP00000032868-D1	1.29	2.88E-05	↓	Protein EFR3-like protein B, partial [B. mutus]
ENSP00000303507-D1	1.29	2.65E-03	↓	Breakpoint cluster region protein, partial [B. mutus]
ENSP00000340595-D1	1.29	8.52E-03	↓	G-protein coupled receptor family C group 5 member C, partial [B. mutus]
ENSBTAP00000016639-D1	1.29	8.52E-03	↓	BEN domain-containing protein 3 [B. mutus]
ENSP00000407436-D3	1.29	2.93E-02	↓	Arf-GAP with GTPase, ANK repeat and PH domain-containing protein 3, partial [B. mutus]
ENSP00000394135-D1	1.29	2.93E-02	↓	unnamed protein product [Homo sapiens]
ENSP00000359557-D1	1.29	2.93E-02	↓	Protein LDOC1 [B. mutus]
ENSBTAP00000025631-D1	1.29	2.93E-02	↓	uncharacterized protein C1orf172 homolog [B. mutus]
ENSP00000320658-D1	1.29	2.93E-02	↓	tyrosine--tRNA ligase, mitochondrial [B. mutus]
ENSP00000349892-D1	1.29	3.88E-65	↓	E3 ubiquitin-protein ligase MYCBP2 [Bison bison bison]
ENSBTAP00000007619-D1	1.29	3.53E-91	↓	Putative pre-mRNA-splicing factor ATP-dependent RNA helicase DHX32, partial [B. mutus]
ENSBTAP00000017880-D1	1.29	3.83E-41	↓	RNA-binding protein 42 isoform X2 [Ovis aries musimon]
ENSBTAP00000005491-D1	1.29	1.75E-09	↓	Ral guanine nucleotide dissociation stimulator, partial [B. mutus]
ENSBTAP00000008609-D1	1.29	5.29E-09	↓	HDGF protein [B. taurus]
ENSP00000358563-D1	1.29	0.00E+00	↓	H/ACA ribonucleoprotein complex subunit 4 isoform X1 [B. mutus]
ENSP00000412500-D2	1.29	5.73E-75	↓	unconventional myosin-VI isoform X1 [Bison bison bison]
ENSP00000381102-D1	1.29	1.45E-07	↓	type 1 phosphatidylinositol 4,5-bisphosphate 4-phosphatase isoform X1 [Vicugna pacos]

ENSBTAP00000001658-D1	1.29	1.14E-70	↓	ankyrin repeat domain-containing protein 46 isoform X1 [Sus scrofa]
ENSP00000300896-D1	1.29	7.91E-106	↓	ubiquitin carboxyl-terminal hydrolase 32 [B. mutus]
ENSP00000295324-D1	1.29	1.23E-05	↓	cdc42 effector protein 3 [B. mutus]
ENSP00000356988-D1	1.29	1.23E-05	↓	nitrilase homolog 1 isoform X1 [B. mutus]
ENSP00000407509-D1	1.29	1.23E-05	↓	Neuropathy target esterase [B. mutus]
ENSP00000350676-D1	1.29	9.04E-23	↓	Histone lysine demethylase PHF8, partial [B. mutus]
ENSP00000349616-D1	1.29	9.11E-134	↓	adiponectin receptor protein 2 [B. mutus]
ENSP00000348730-D1	1.30	5.96E-19	↓	dual specificity protein phosphatase 13 isoform X2 [Bison bison bison]
ENSP00000390948-D1	1.30	1.57E-201	↓	cytoplasmic FMR1-interacting protein 2 isoform X1 [B. mutus]
ENSP00000313854-D1	1.30	2.59E-19	↓	Cleft lip and palate transmembrane protein 1-like protein, partial [B. mutus]
ENSP00000311679-D1	1.30	2.58E-102	↓	Zinc finger protein 483, partial [B. mutus]
ENSP00000369335-D1	1.30	2.99E-09	↓	F-box only protein 18, partial [B. mutus]
ENSP00000227524-D1	1.30	7.77E-19	↓	Pre-mRNA-processing factor 19, partial [B. mutus]
ENSBTAP00000003073-D1	1.30	1.74E-06	↓	nucleobindin-1 [B. mutus]
ENSP00000338272-D1	1.30	7.54E-88	↓	hairy/enhancer-of-split related with YRPW motif protein 1 isoform X1 [B. mutus]
ENSP00000294053-D1	1.30	7.51E-13	↓	caseinolytic peptidase B protein homolog isoform X1 [B. mutus]
ENSP00000240731-D4	1.30	1.91E-16	↓	zinc finger protein 211 [B. taurus]
ENSBTAP00000008074-D1	1.30	1.91E-16	↓	cytidine deaminase [B. taurus]
ENSBTAP00000012699-D1	1.30	2.61E-22	↓	protein arginine N-methyltransferase 7 [B. mutus]
ENSBTAP00000008383-D1	1.30	8.17E-08	↓	gamma-tubulin complex component 2 isoform X2 [B. mutus]
ENSBTAP00000011735-D2	1.30	2.20E-48	↓	Cytoplasmic FMR1-interacting protein 1, partial [B. mutus]
ENSP00000373937-D1	1.30	2.75E-17	↓	Protogenin, partial [B. mutus]
ENSP00000230449-D1	1.30	2.73E-53	↓	Exocyst complex component 2 [B. mutus]
ENSBTAP00000022005-D1	1.30	1.11E-03	↓	Laminin subunit beta-3 [B. mutus]
ENSP00000246117-D1	1.30	1.11E-03	↓	nicalin [B. taurus]
ENSBTAP00000053539-D3	1.30	4.25E-13	↓	Mannosyl-oligosaccharide 1,2-alpha-mannosidase IC, partial [B. mutus]
ENSP00000336747-D1	1.30	1.93E-22	↓	Huntingtin-interacting protein 1, partial [B. mutus]
ENSBTAP00000019822-D1	1.30	3.53E-08	↓	discoidin, CUB and LCCL domain-containing protein 2 [B. mutus]
ENSBTAP00000051202-D1	1.30	4.94E-05	↓	Lambda-crystallin-like protein, partial [B. mutus]
ENSP00000361014-D1	1.30	6.11E-114	↓	MAP kinase-interacting serine/threonine-protein kinase 1, partial [B. mutus]
ENSBTAP00000045293-D1	1.30	8.37E-208	↓	dual specificity mitogen-activated protein kinase kinase 1 [Homo sapiens]
ENSP00000370543-D1	1.30	1.21E-23	↓	sodium/myo-inositol cotransporter [B. taurus]
ENSBTAP00000018637-D1	1.30	2.10E-40	↓	Rhotekin-2, partial [B. mutus]
ENSBTAP00000021306-D1	1.30	9.46E-30	↓	Ankyrin repeat domain-containing protein 11, partial [B. mutus]
ENSP00000319248-D1	1.30	5.54E-13	↓	Zinc finger E-box-binding homeobox 1, partial [B. mutus]
ENSBTAP00000026388-D1	1.30	1.51E-04	↓	Rhomboid family member 1 [B. mutus]
ENSP00000415836-D1	1.30	3.49E-03	↓	zinc finger protein 276 isoform X1 [B. taurus]
ENSP00000265348-D2	1.30	3.49E-03	↓	cullin-7 [B. taurus]
ENSBTAP00000053799-D1	1.31	1.98E-51	↓	Tetratricopeptide repeat protein 17, partial [B. mutus]
ENSP00000370883-D1	1.31	1.05E-10	↓	nodal modulator 1 [B. taurus]
ENSBTAP00000002972-D1	1.31	7.66E-48	↓	golgi glycoprotein 1 [B. mutus]
ENSP00000307304-D1	1.31	1.00E-27	↓	chromodomain-helicase-DNA-binding protein 7 [B. mutus]
ENSBTAP00000013868-D1	1.31	1.85E-22	↓	solute carrier family 40 member 1 isoform X1 [B. mutus]
ENSP00000283684-D1	1.31	2.39E-16	↓	probable phospholipid-transporting ATPase IC [B. mutus]
ENSP00000294016-D1	1.31	9.80E-07	↓	adenylate cyclase type 9 [B. mutus]
ENSBTAP00000038425-D1	1.31	1.88E-184	↓	Myogenic factor 5, partial [B. mutus]
ENSP00000311596-D9	1.31	2.97E-06	↓	zinc finger protein 420 isoform X3 [Ovis aries musimon]
ENSBTAP00000022546-D1	1.31	4.69E-04	↓	galactosylgalactosylxylosylprotein 3-beta-glucuronosyltransferase 3 [Bubalus bubalis]
ENSBTAP00000012742-D1	1.31	6.01E-08	↓	Transmembrane protein 184B, partial [B. mutus]
ENSP00000297954-D1	1.31	6.47E-05	↓	Serine/threonine-protein kinase WNK2, partial [B. mutus]
ENSP00000393471-D1	1.31	1.79E-25	↓	transcriptional adapter 3 [Ictidomys tridecemlineatus]
ENSBTAP00000028599-D1	1.31	1.22E-12	↓	Transcription factor E2-alpha, partial [B. mutus]
ENSP00000344087-D1	1.31	2.53E-14	↓	autism susceptibility candidate 2 [B. mutus]
ENSP00000387836-D83	1.31	7.64E-20	↓	Zinc finger protein 248, partial [B. mutus]
ENSBTAP00000007769-D1	1.31	1.10E-17	↓	ADNP homeobox protein 2, partial [B. mutus]

ENSBTAP00000009672-D1	1.31	1.82E-07	↓	homeobox protein DLX-4 [B. taurus]
ENSP00000256194-D1	1.31	2.01E-177	↓	Protein MICAL-2 [B. mutus]
ENSBTAP00000007135-D1	1.32	4.83E-78	↓	26S protease regulatory subunit 4, partial [B. mutus]
ENSBTAP00000021778-D1	1.32	2.76E-05	↓	opioid growth factor receptor-like protein 1-like [B. mutus]
ENSBTAP00000048364-D3	1.32	1.46E-03	↓	vesicle transport protein SFT2A [B. taurus]
ENSP00000399075-D1	1.32	1.46E-03	↓	hypothetical protein M91_08470, partial [B. mutus]
ENSBTAP00000025471-D2	1.32	1.46E-03	↓	Serine/threonine-protein kinase Nek5, partial [B. mutus]
ENSP00000364298-D1	1.32	1.13E-02	↓	FK506-binding protein-like protein [B. mutus]
ENSP00000351975-D1	1.32	1.13E-02	↓	chromosome 16 open reading frame 5 [Homo sapiens]
ENSP00000344244-D1	1.32	1.13E-02	↓	N-acetylated-alpha-linked acidic dipeptidase-like protein [B. mutus]
ENSP00000392188-D60	1.32	1.13E-02	↓	hypothetical protein M91_08847, partial [B. mutus]
ENSP00000357656-D1	1.32	2.99E-34	↓	Tyrosine-protein kinase Fyn [B. mutus]
ENSP00000365477-D1	1.32	3.57E-192	↓	coiled-coil domain-containing protein 93 [B. taurus]
ENSP00000344582-D1	1.32	3.01E-49	↓	NHS-like protein 1 isoform X2 [B. taurus]
ENSP00000310015-D1	1.32	1.01E-10	↓	tRNA-specific adenosine deaminase 1 isoform X1 [Bison bison bison]
ENSBTAP00000008572-D1	1.32	2.44E-35	↓	bcl-2-like protein 1 [B. taurus]
ENSP00000366035-D1	1.32	6.75E-55	↓	Alpha-actinin-1 [B. mutus]
ENSP00000381932-D1	1.32	1.88E-90	↓	Dual specificity tyrosine-phosphorylation-regulated kinase 1A, partial [B. mutus]
ENSBTAP00000027126-D1	1.32	1.57E-132	↓	GON-4-like protein [B. mutus]
ENSP00000380116-D1	1.32	1.18E-05	↓	hypothetical protein M91_17395, partial [B. mutus]
ENSBTAP00000028964-D1	1.32	2.71E-12	↓	2-oxoglutarate dehydrogenase-like, mitochondrial, partial [B. mutus]
yakG002104	1.32	1.15E-24	↓	TBC1 domain family member 1 [B. mutus]
ENSP00000401018-D1	1.32	2.79E-40	↓	DNA replication complex GINS protein PSF3 [B. taurus]
ENSP00000304769-D1	1.32	8.46E-05	↓	Zinc finger protein 467 [B. mutus]
ENSP00000307272-D1	1.32	7.18E-07	↓	regulatory-associated protein of mTOR isoform X1 [B. mutus]
yakG013753	1.32	2.01E-297	↓	hypothetical protein M91_21518 [B. mutus]
ENSBTAP00000020586-D1	1.32	5.07E-06	↓	lysine-specific demethylase 4B isoform X1 [B. taurus]
yakG042006	1.32	6.16E-04	↓	hypothetical protein M91_01569 [B. mutus]
ENSBTAP00000028809-D3	1.32	0.00E+00	↓	hypothetical protein M91_02237, partial [B. mutus]
ENSBTAP00000007681-D1	1.32	3.34E-55	↓	secretory carrier-associated membrane protein 2 [B. taurus]
ENSP00000307214-D1	1.32	1.20E-22	↓	leucine carboxyl methyltransferase 2 [B. mutus]
ENSBTAP00000007039-D1	1.32	1.54E-31	↓	BAG family molecular chaperone regulator 2, partial [B. mutus]
ENSBTAP00000016449-D1	1.33	3.55E-75	↓	Deoxycytidine kinase [B. mutus]
ENSBTAP00000028690-D1	1.33	3.60E-22	↓	Insulin-like growth factor 1 receptor [B. mutus]
ENSBTAP00000017780-D2	1.33	1.01E-17	↓	Palmitoyl-protein thioesterase 1, partial [B. mutus]
ENSP00000367476-D1	1.33	5.15E-10	↓	centrosomal protein of 104 kDa, partial [B. mutus]
ENSP00000267064-D1	1.33	2.51E-59	↓	SWI/SNF complex subunit SMARCC2, partial [B. mutus]
ENSP00000374171-D1	1.33	2.23E-10	↓	Protein TANC2, partial [B. mutus]
ENSP00000338283-D1	1.33	4.64E-03	↓	neuronal PAS domain-containing protein 2 [B. mutus]
ENSP00000378001-D1	1.33	4.64E-03	↓	bifunctional heparan sulfate N-deacetylase/N-sulfotransferase 3-like [B. mutus]
ENSP00000386420-D1	1.33	5.39E-30	↓	Calmodulin-regulated spectrin-associated protein 1, partial [B. mutus]
ENSBTAP00000008631-D1	1.33	3.74E-13	↓	vesicle-trafficking protein SEC22c [B. taurus]
ENSP00000409204-D1	1.33	2.65E-22	↓	SUN domain-containing protein 2 [B. mutus]
ENSP00000307854-D1	1.33	9.41E-62	↓	protein-L-isoaspartate O-methyltransferase domain-containing protein 2 isoform X1 [B. mutus]
ENSP00000318472-D1	1.33	6.63E-06	↓	Neural cell adhesion molecule 1, partial [B. mutus]
ENSP00000350708-D1	1.33	1.19E-16	↓	UV excision repair protein RAD23-like protein B, partial [B. mutus]
ENSP00000310722-D1	1.33	0.00E+00	↓	proto-oncogene serine/threonine-protein kinase mos [Bison bison bison]
ENSBTAP00000009123-D1	1.33	1.81E-11	↓	GTPase-activating Rap/Ran-GAP domain-like protein 3 [B. mutus]
ENSBTAP00000022510-D1	1.33	4.88E-13	↓	adenylyl cyclase-associated protein 2 isoform X1 [B. mutus]
ENSBTAP00000051218-D3	1.33	1.11E-04	↓	hypothetical protein M91_11273, partial [B. mutus]
ENSBTAP00000001083-D1	1.33	4.45E-154	↓	Pleckstrin-like protein domain-containing family G member 7, partial [B. mutus]
ENSP00000350989-D1	1.33	4.72E-05	↓	Thioredoxin domain-containing protein 5, partial [B. mutus]
ENSP00000169551-D1	1.33	4.83E-59	↓	mitochondrial import inner membrane translocase subunit Tim21 [B. mutus]
ENSP00000390107-D1	1.33	5.27E-34	↓	Adenylosuccinate lyase [B. mutus]
ENSP00000368389-D1	1.34	1.81E-28	↓	neugrin-like [B. mutus]

ENSBTAP00000012233-D1	1.34	2.07E-56	↓	Glutaminase liver isoform, mitochondrial [B. mutus]
ENSBTAP00000032197-D1	1.34	9.39E-82	↓	DNA-directed RNA polymerase I subunit RPA34 [B. mutus]
ENSP00000343054-D1	1.34	2.43E-215	↓	RNA-binding protein 5, partial [B. mutus]
ENSP00000371419-D1	1.34	4.21E-08	↓	poly [ADP-ribose] polymerase 4-like [B. mutus]
ENSP00000321746-D1	1.34	2.79E-123	↓	PDZ and LIM domain protein 5 isoform X1 [B. mutus]
ENSBTAP00000035273-D1	1.34	3.56E-57	↓	RNA polymerase-associated protein CTR9-like protein [B. mutus]
ENSBTAP00000014880-D1	1.34	3.13E-32	↓	Ninjurin-1, partial [B. mutus]
ENSP00000363288-D1	1.34	1.83E-08	↓	delta-aminolevulinic acid dehydratase isoform X1 [Pongo abelii]
ENSP00000217901-D1	1.34	1.41E-19	↓	isocitrate dehydrogenase [NAD] subunit gamma, mitochondrial isoform X1 [B. taurus]
ENSP00000376007-D1	1.34	7.64E-164	↓	poly(A)-specific ribonuclease PARN-like domain-containing protein 1 [B. mutus]
ENSBTAP00000009890-D3	1.34	7.00E-25	↓	Copine-9, partial [B. mutus]
ENSP00000379503-D1	1.34	3.72E-45	↓	pyruvate dehyrogenase phosphatase catalytic subunit 1 isoform X1 [Bison bison bison]
ENSP00000368464-D1	1.34	9.42E-120	↓	Kinesin-like protein KIF24 [B. mutus]
ENSP00000337127-D1	1.34	1.05E-139	↓	Superoxide dismutase [Mn], mitochondrial, partial [B. mutus]
ENSBTAP00000005823-D1	1.35	3.72E-35	↓	xaa-Pro aminopeptidase 1 isoform X2 [B. taurus]
ENSBTAP00000049024-D2	1.35	4.33E-23	↓	protein zyg-11 homolog B isoform X2 [Ovis aries]
ENSBTAP00000026137-D1	1.35	1.43E-93	↓	src-like-adapter 2 [B. taurus]
ENSP00000355866-D1	1.35	1.27E-167	↓	dual specificity protein phosphatase 10 [B. taurus]
ENSP00000358576-D1	1.35	2.37E-08	↓	5' exonuclease Apollo, partial [B. mutus]
ENSBTAP00000027659-D1	1.35	3.97E-15	↓	Zinc finger protein 526 [B. mutus]
ENSP00000362900-D1	1.35	5.77E-58	↓	serine/arginine-rich splicing factor 4 [Mesocricetus auratus]
ENSBTAP00000019160-D1	1.35	1.43E-51	↓	Oxysterol-binding protein-related protein 6, partial [B. mutus]
ENSBTAP00000048673-D1	1.35	1.96E-293	↓	Clusterin-associated protein 1 [B. mutus]
ENSBTAP00000046384-D1	1.35	1.13E-05	↓	zinc finger CCHC domain-containing protein 12-like [B. mutus]
ENSP00000382717-D1	1.35	7.82E-242	↓	WD repeat-containing protein 19, partial [B. mutus]
ENSP00000398181-D1	1.35	2.14E-18	↓	Alpha-ketoglutarate-dependent dioxygenase alkB-like protein 2 [B. mutus]
ENSBTAP00000006538-D1	1.35	1.20E-46	↓	Kinesin-like protein KIF1B [B. mutus]
yakG015170	1.35	8.94E-07	↓	Kielin/chordin-like protein [B. mutus]
ENSBTAP00000040666-D1	1.35	5.56E-41	↓	target of EGR1 protein 1 [B. taurus]
ENSBTAP00000021018-D1	1.35	8.82E-40	↓	AMP deaminase 3 [B. mutus]
ENSBTAP00000017171-D1	1.35	1.66E-07	↓	lysine-specific demethylase PHF2-like [Bubalus bubalis]
ENSP00000358266-D1	1.35	7.18E-88	↓	Actin-binding LIM protein 1 [B. mutus]
ENSP00000232564-D3	1.35	6.34E-25	↓	guanine nucleotide-binding protein G(I)/G(S)/G(T) subunit beta-1 [Mus musculus]
ENSBTAP00000021104-D1	1.35	5.45E-16	↓	RAD9, HUS1, RAD1-interacting nuclear orphan protein 1 [B. mutus]
ENSP00000363587-D1	1.35	7.23E-74	↓	sushi, von Willebrand factor type A, EGF and pentraxin domain-containing protein 1, partial [B. mutus]
ENSBTAP00000011840-D1	1.35	0.00E+00	↓	Eukaryotic translation initiation factor 4E transporter [B. mutus]
ENSBTAP00000024228-D1	1.35	6.80E-19	↓	cytochrome b ascorbate-dependent protein 3 [B. taurus]
ENSBTAP00000006848-D1	1.35	1.94E-102	↓	Tyrosine-protein kinase BAZ1B, partial [B. mutus]
ENSP00000357643-D1	1.35	3.84E-131	↓	antigen KI-67 [B. mutus]
ENSBTAP00000020894-D1	1.35	0.00E+00	↓	Importin subunit alpha-8, partial [B. mutus]
ENSP00000402009-D1	1.35	0.00E+00	↓	Nuclear pore complex protein Nup153, partial [B. mutus]
ENSP00000347134-D1	1.36	6.36E-55	↓	neuroepithelial cell-transforming gene 1 protein isoform X1 [B. taurus]
ENSBTAP00000053572-D1	1.36	5.85E-149	↓	Ankyrin-3, partial [B. mutus]
ENSP00000372358-D1	1.36	1.40E-13	↓	OTU domain-containing protein 7A [Ovis aries]
ENSP00000233154-D1	1.36	4.93E-11	↓	cytoplasmic protein NCK2 [B. taurus]
ENSP00000350583-D1	1.36	2.15E-07	↓	Dipeptidyl peptidase 9, partial [B. mutus]
yakG017947	1.36	1.17E-06	↓	hypothetical protein M91_20063 [B. mutus]
ENSP00000408176-D1	1.36	6.31E-06	↓	A/G-specific adenine DNA glycosylase, partial [B. mutus]
ENSBTAP00000014478-D1	1.36	1.47E-05	↓	PAXIP1-associated glutamate-rich protein 1 [B. mutus]
ENSP00000369131-D1	1.36	8.06E-05	↓	paired amphipathic helix protein Sin3b isoform X1 [B. taurus]
ENSBTAP00000044680-D1	1.36	4.47E-04	↓	Putative hexokinase HKDC1, partial [B. mutus]
ENSBTAP00000037296-D1	1.36	1.06E-03	↓	RecQ-mediated genome instability protein 2, partial [B. mutus]
ENSBTAP00000041788-D1	1.36	2.53E-03	↓	LIM domain kinase 2, partial [B. mutus]
yakG027366	1.36	1.52E-02	↓	hypothetical protein M91_04468 [B. mutus]
ENSP00000228245-D1	1.36	1.52E-02	↓	Tubby-related protein 3, partial [B. mutus]

ENSBTAP00000023093-D1	1.36	1.52E-02	↓	RELT-like protein 2 [B. taurus]
ENSP00000315872-D1	1.36	1.52E-02	↓	putative uncharacterized protein FLJ40606 [Ovis aries musimon]
ENSBTAP00000018512-D1	1.36	4.05E-02	↓	coiled-coil domain-containing protein 71 [B. mutus]
ENSP00000350896-D1	1.36	4.05E-02	↓	ephrin type-B receptor 4 isoform X1 [B. mutus]
ENSBTAP00000008949-D1	1.36	4.05E-02	↓	Kin of IRRE-like protein 3, partial [B. mutus]
ENSP00000341289-D9	1.36	4.05E-02	↓	Tubulin beta-2 chain, partial [Corvus brachyrhynchos]
ENSBTAP00000053069-D4	1.36	4.05E-02	↓	hypothetical protein M91_02365, partial [B. mutus]
ENSP00000356145-D1	1.36	4.05E-02	↓	leucine-rich repeat neuronal protein 2 [B. mutus]
ENSP00000296130-D1	1.36	4.05E-02	↓	tetranectin [B. mutus]
ENSP00000384515-D2	1.36	4.05E-02	↓	Beta-parvin, partial [B. mutus]
ENSBTAP00000041215-D1	1.36	4.05E-02	↓	TNF receptor-associated factor 1 [B. mutus]
ENSP00000307513-D1	1.36	4.05E-02	↓	C-type mannose receptor 2 [B. mutus]
ENSBTAP00000045651-D1	1.36	4.05E-02	↓	regulator of G-protein signaling 18 [B. taurus]
ENSBTAP00000007185-D1	1.36	1.75E-39	↓	fragile X mental retardation syndrome-related protein 2 isoform X1 [Bison bison bison]
ENSBTAP00000004141-D1	1.36	3.72E-53	↓	arf-GAP with SH3 domain, ANK repeat and PH domain-containing protein 1 [B. taurus]
ENSP00000344801-D2	1.36	4.78E-42	↓	sodium/potassium/calcium exchanger 2-like [B. mutus]
ENSP00000267620-D1	1.36	7.89E-10	↓	thyroid receptor-interacting protein 11-like protein [Camelus ferus]
ENSBTAP00000013009-D1	1.36	1.82E-09	↓	Cell death activator CIDE-A, partial [B. mutus]
ENSBTAP00000005255-D1	1.36	8.62E-35	↓	UPF0577 protein KIAA1324-like protein, partial [B. mutus]
ENSBTAP00000035548-D1	1.36	4.81E-66	↓	tubulin polyglutamylase TTLL5 [B. mutus]
ENSP00000263026-D1	1.36	1.21E-07	↓	eukaryotic elongation factor 2 kinase [Bison bison bison]
ENSBTAP00000009298-D11	1.36	8.29E-27	↓	Zinc finger protein with KRAB and SCAN domains 1, partial [B. mutus]
ENSBTAP00000028704-D1	1.37	2.15E-20	↓	WD repeat-containing protein 11 [B. mutus]
ENSP00000358865-D1	1.37	1.92E-10	↓	Alpha-internexin, partial [B. mutus]
ENSBTAP00000009697-D1	1.37	3.53E-06	↓	PDZ and LIM domain protein 2 [B. mutus]
ENSBTAP00000052857-D64	1.37	2.68E-31	↓	hypothetical protein M91_12604, partial [B. mutus]
ENSBTAP00000002986-D1	1.37	0.00E+00	↓	E3 ubiquitin-protein ligase RNF34, partial [B. mutus]
ENSBTAP00000012410-D1	1.37	2.15E-48	↓	solute carrier family 25 member 44 [B. taurus]
ENSP00000262539-D1	1.37	1.48E-47	↓	Tyrosine-protein phosphatase non-receptor type 3 [B. mutus]
ENSBTAP00000053701-D1	1.37	4.22E-18	↓	zinc transporter 4 [B. taurus]
ENSP00000338360-D1	1.37	1.11E-23	↓	transcription factor E3 [B. taurus]
ENSBTAP00000050302-D1	1.37	3.21E-14	↓	39S ribosomal protein L38, mitochondrial [B. mutus]
ENSBTAP00000016000-D1	1.37	5.39E-46	↓	zinc finger protein 473 [B. mutus]
ENSBTAP00000026715-D1	1.37	1.35E-30	↓	Malectin [B. mutus]
ENSP00000342144-D1	1.37	1.05E-04	↓	Limbin, partial [B. mutus]
ENSP00000351416-D1	1.37	0.00E+00	↓	Ankyrin repeat domain-containing protein 17, partial [B. mutus]
ENSP00000348031-D1	1.37	9.14E-38	↓	histone-lysine N-methyltransferase, H3 lysine-36 and H4 lysine-20 specific isoform X1 [B. mutus]
ENSP00000368842-D1	1.37	3.08E-09	↓	motile sperm domain-containing protein 3 [B. taurus]
ENSP00000344976-D1	1.37	2.48E-04	↓	X-linked interleukin-1 receptor accessory protein-like 2 [B. mutus]
ENSP00000331258-D1	1.37	2.48E-04	↓	protein O-mannose kinase-like [B. mutus]
ENSP00000358884-D1	1.37	2.48E-04	↓	Cytochrome b561 domain-containing protein 1 [B. mutus]
ENSBTAP00000034997-D1	1.37	1.65E-08	↓	Protein spinster-like protein 3, partial [B. mutus]
ENSP00000240423-D1	1.37	1.90E-152	↓	Condensin complex subunit 2, partial [B. mutus]
ENSBTAP00000003498-D1	1.37	2.21E-190	↓	Mast/stem cell growth factor receptor, partial [B. mutus]
ENSBTAP00000007404-D1	1.38	1.19E-12	↓	Valyl-tRNA synthetase [B. mutus]
ENSP00000364240-D1	1.38	5.85E-04	↓	UBX domain-containing protein 10 [B. mutus]
ENSP00000301067-D1	1.38	5.85E-04	↓	histone-lysine N-methyltransferase 2D [B. mutus]
ENSBTAP00000007216-D1	1.38	8.33E-21	↓	E3 ubiquitin-protein ligase ARIH2 [B. taurus]
ENSBTAP00000048486-D1	1.38	1.07E-05	↓	DNA replication factor Cdt1, partial [B. mutus]
ENSP00000160373-D1	1.38	7.69E-24	↓	Cortactin-binding protein 2, partial [B. mutus]
ENSBTAP00000024378-D2	1.38	0.00E+00	↓	melanoma-associated antigen 10-like isoform X2 [B. taurus]
ENSBTAP00000034568-D1	1.38	1.31E-14	↓	Na(+)/H(+) exchange regulatory cofactor NHE-RF1, partial [B. mutus]
ENSP00000370648-D1	1.38	3.56E-12	↓	Retinol dehydrogenase 14, partial [B. mutus]
ENSP00000399384-D1	1.38	1.38E-03	↓	AP-2 complex subunit alpha-1, partial [B. mutus]
ENSP00000376390-D1	1.38	1.38E-03	↓	Ras-related protein Rab-37, partial [B. mutus]

ENSP00000347930-D1	1.38	1.38E-03	↓	cell cycle control protein 50B [B. taurus]
ENSP00000218348-D1	1.38	2.49E-20	↓	ubiquitin specific peptidase 11 [B. mutus]
ENSP00000354730-D1	1.38	8.45E-115	↓	chromosome alignment-maintaining phosphoprotein 1 [B. mutus]
ENSP00000408478-D2	1.38	3.20E-15	↓	Mastermind-like protein 1, partial [B. mutus]
ENSP00000234396-D1	1.38	4.19E-10	↓	V-type proton ATPase subunit B, kidney isoform isoform X1 [B. mutus]
ENSBTAP00000002494-D1	1.38	1.37E-37	↓	hypothetical protein M91_05378, partial [B. mutus]
ENSP00000288071-D1	1.38	9.60E-28	↓	ATP-dependent RNA helicase DDX19A [B. taurus]
ENSP00000366876-D1	1.38	1.10E-06	↓	PHD finger protein 13, partial [B. mutus]
ENSP00000303992-D1	1.38	5.84E-05	↓	Transmembrane protein 43, partial [B. mutus]
ENSP00000256460-D1	1.38	5.84E-05	↓	Calcium/calmodulin-dependent protein kinase type 1 [B. mutus]
ENSBTAP00000031070-D1	1.38	5.84E-05	↓	Cytosolic non-specific dipeptidase, partial [B. mutus]
ENSBTAP00000030993-D1	1.38	8.59E-40	↓	AFG3-like protein 2 [B. mutus]
ENSP00000325546-D1	1.38	1.79E-15	↓	zinc finger protein PLAG1 [Pantholops hodgsonii]
ENSBTAP00000042970-D1	1.38	9.97E-38	↓	cadherin-3 isoform X1 [B. taurus]
ENSP00000255674-D1	1.38	1.02E-10	↓	rotatin [B. mutus]
ENSP00000419879-D1	1.38	2.56E-06	↓	Phosphatidate cytidylyltransferase 2, partial [B. mutus]
yakG043453	1.38	2.56E-06	↓	Hydroxyindole O-methyltransferase [B. mutus]
ENSBTAP00000023588-D2	1.38	7.25E-60	↓	tubulin alpha-1 chain-like [B. mutus]
ENSBTAP00000028179-D1	1.38	2.00E-17	↓	Proline-serine-threonine phosphatase-interacting protein 1, partial [B. mutus]
ENSP00000222388-D1	1.39	3.05E-121	↓	ATP-binding cassette sub-family F member 2 [B. taurus]
ENSP00000256996-D1	1.39	3.79E-32	↓	DNA damage-binding protein 2 [B. mutus]
ENSBTAP00000002772-D1	1.39	1.18E-120	↓	Protein-arginine deiminase type-6, partial [B. mutus]
ENSBTAP00000012720-D1	1.39	2.64E-07	↓	T-cell leukemia/lymphoma protein 1B [B. mutus]
ENSBTAP00000050426-D1	1.39	5.96E-06	↓	solute carrier family 10 member 6 [B. mutus]
ENSP00000383145-D1	1.39	1.37E-04	↓	Protein SFI1-like protein [B. mutus]
ENSBTAP00000029144-D1	1.39	3.33E-03	↓	Negative elongation factor B, partial [B. mutus]
ENSBTAP00000012208-D1	1.39	3.33E-03	↓	CD226 antigen, partial [B. mutus]
ENSP00000269554-D1	1.39	3.33E-03	↓	phosphatidylinositol 5-phosphate 4-kinase type-2 beta [B. taurus]
ENSBTAP00000036113-D1	1.39	3.33E-03	↓	latrophilin-1 isoform X1 [B. mutus]
ENSP00000382441-D1	1.39	4.98E-23	↓	Ubiquitin fusion degradation protein 1-like protein, partial [B. mutus]
ENSP00000378056-D1	1.39	4.56E-40	↓	High affinity cAMP-specific and IBMX-insensitive 3',5'-cyclic phosphodiesterase 8A, partial [B. mutus]
ENSBTAP00000022552-D1	1.39	5.49E-76	↓	WD repeat and FYVE domain-containing protein 1, partial [B. mutus]
ENSP00000406037-D1	1.39	5.71E-44	↓	Putative histone acetyltransferase MYST1 [B. mutus]
ENSBTAP00000053437-D1	1.39	6.92E-85	↓	Neuron navigator 3, partial [B. mutus]
ENSP00000246194-D1	1.39	1.57E-60	↓	RNA-binding protein Raly [B. mutus]
ENSP00000411372-D1	1.39	1.39E-05	↓	Protein FAM200A [B. mutus]
ENSBTAP00000035932-D1	1.39	1.54E-56	↓	hypothetical protein M91_12753, partial [B. mutus]
ENSBTAP00000016002-D1	1.39	4.05E-25	↓	WD repeat domain phosphoinositide-interacting protein 2, partial [B. mutus]
ENSBTAP00000052975-D1	1.39	1.43E-06	↓	Arf-GAP with dual PH domain-containing protein 1, partial [B. mutus]
ENSP00000409610-D1	1.39	3.22E-04	↓	Protein TSSC4, partial [B. mutus]
ENSP00000220562-D1	1.39	2.54E-27	↓	exostosin-like 3-like [B. mutus]
ENSBTAP00000004187-D1	1.39	1.55E-08	↓	coiled-coil domain-containing protein 115 [B. mutus]
ENSP00000376564-D1	1.39	3.24E-05	↓	Na(+)/H(+) exchange regulatory cofactor NHE-RF4 [B. mutus]
ENSBTAP00000013879-D1	1.39	4.35E-17	↓	cohesin subunit SA-3 [B. taurus]
ENSBTAP00000019311-D1	1.39	1.63E-09	↓	Carbonyl reductase [NADPH] 1, partial [B. mutus]
ENSBTAP00000017463-D1	1.39	2.21E-155	↓	Tyrosyl-DNA phosphodiesterase 1, partial [B. mutus]
ENSP00000246112-D1	1.39	3.64E-53	↓	Transducin-like enhancer protein 6, partial [B. mutus]
ENSP00000356809-D1	1.39	3.33E-06	↓	G-protein coupled receptor 161 isoform X2 [Bison bison bison]
ENSP00000291577-D1	1.39	3.43E-07	↓	ES1 protein-like protein, mitochondrial [B. mutus]
ENSBTAP00000053582-D1	1.40	1.56E-99	↓	protein dopey-1 [B. mutus]
ENSBTAP00000015445-D1	1.40	3.57E-08	↓	epidermal growth factor receptor [Bison bison bison]
ENSP00000299201-D1	1.40	3.57E-08	↓	tRNA (adenine-N(1)-)-methyltransferase catalytic subunit TRMT61A, partial [B. mutus]
ENSP00000258198-D1	1.40	5.64E-261	↓	cytoplasmic dynein 1 light intermediate chain 2 [Bison bison bison]
ENSP00000338769-D1	1.40	4.34E-12	↓	arf-GAP with SH3 domain, ANK repeat and PH domain-containing protein 3 [Bison bison bison]
ENSP00000386105-D1	1.40	4.34E-12	↓	DNA polymerase delta subunit 2 [B. mutus]

ENSP00000409514-D37	1.40	9.27E-26	↓	Zinc finger protein 23, partial [B. mutus]
ENSBTAP00000029911-D2	1.40	7.61E-04	↓	motile sperm domain-containing protein 2 isoform X2 [Bubalus bubalis]
ENSBTAP00000006962-D1	1.40	7.61E-04	↓	fms-related tyrosine kinase 3 ligand isoform X1 [B. mutus]
ENSBTAP00000036408-D1	1.40	8.08E-03	↓	serine protease 38 [B. mutus]
ENSP00000264036-D1	1.40	8.08E-03	↓	Cell surface glycoprotein MUC18, partial [B. mutus]
ENSBTAP00000011438-D1	1.40	8.08E-03	↓	serine/arginine repetitive matrix protein 4, partial [B. mutus]
ENSP00000349486-D1	1.40	8.08E-03	↓	protein scribble homolog [B. mutus]
ENSBTAP00000003928-D1	1.40	8.08E-03	↓	Scavenger receptor cysteine-rich domain-containing group B protein, partial [B. mutus]
ENSBTAP00000015695-D2	1.40	8.08E-03	↓	uncharacterized protein C19orf43 homolog [Camelus bactrianus]
ENSP00000262442-D2	1.40	1.01E-22	↓	dynein heavy chain 17, axonemal isoform X1 [B. taurus]
ENSP00000408429-D1	1.40	2.21E-10	↓	hypothetical protein M91_04827, partial [B. mutus]
ENSP00000263331-D1	1.40	2.66E-98	↓	DNA-directed RNA polymerase I subunit RPA2 isoform X1 [B. mutus]
ENSBTAP00000016037-D1	1.40	1.73E-58	↓	Gem-associated protein 5 [B. mutus]
ENSBTAP00000005677-D1	1.40	4.47E-29	↓	nicastrin isoform X1 [B. mutus]
ENSBTAP00000027991-D1	1.40	1.51E-14	↓	far upstream element-binding protein 2 isoform X2, partial [B. taurus]
ENSBTAP00000053586-D1	1.40	1.36E-12	↓	.
ENSP00000414088-D1	1.40	4.43E-45	↓	disintegrin and metalloproteinase domain-containing protein 19 [B. mutus]
ENSBTAP00000001360-D1	1.40	2.24E-27	↓	AT-rich interactive domain-containing protein 1A [B. mutus]
ENSBTAP00000023162-D1	1.40	6.53E-21	↓	Transmembrane protein 214, partial [B. mutus]
ENSP00000224721-D1	1.40	1.57E-47	↓	Cadherin-23, partial [B. mutus]
ENSBTAP00000019595-D1	1.40	1.78E-04	↓	CD27 antigen [B. mutus]
ENSBTAP00000025878-D1	1.41	1.27E-49	↓	Putative protein KIAA0232 [B. mutus]
ENSBTAP00000027071-D1	1.41	3.33E-147	↓	nuclear receptor coactivator 2 [B. mutus]
ENSBTAP00000003427-D1	1.41	7.28E-12	↓	Vacuolar protein sorting-associated protein 52-like protein [B. mutus]
ENSBTAP00000002392-D1	1.41	2.32E-23	↓	Leucine-rich repeat-containing protein 46, partial [B. mutus]
ENSBTAP00000025153-D1	1.41	5.37E-45	↓	leucine-rich repeat-containing protein 16C [Bison bison bison]
ENSP00000414859-D1	1.41	2.00E-76	↓	CUGBP Elav-like family member 1 isoform X1 [B. mutus]
ENSP00000413362-D1	1.41	1.04E-06	↓	TPA: tubulin tyrosine ligase-like family, member 6 [B. taurus]
ENSP00000297135-D1	1.41	2.60E-100	↓	conserved oligomeric Golgi complex subunit 5 [B. mutus]
ENSBTAP00000010532-D1	1.41	1.60E-10	↓	Folliculin [B. mutus]
ENSBTAP00000026945-D1	1.41	4.57E-26	↓	RNA exonuclease 1-like protein, partial [B. mutus]
ENSBTAP00000002572-D2	1.41	2.48E-07	↓	Small cell adhesion glycoprotein, partial [B. mutus]
ENSP00000347325-D1	1.41	6.20E-192	↓	Histone-lysine N-methyltransferase MLL3, partial [B. mutus]
ENSBTAP00000010424-D1	1.41	6.89E-93	↓	Nuclear receptor coactivator 6, partial [B. mutus]
ENSBTAP00000020714-D1	1.41	1.55E-62	↓	Putative protein KIAA0753, partial [B. mutus]
ENSBTAP00000019726-D1	1.41	9.55E-53	↓	GLTSCR1-like protein isoform X1 [B. mutus]
ENSP00000249363-D1	1.41	3.70E-10	↓	leucine-rich repeat-containing protein 4 isoform X1 [B. mutus]
ENSBTAP00000043277-D1	1.41	1.39E-37	↓	serine/threonine-protein kinase haspin, partial [B. mutus]
ENSBTAP00000002852-D1	1.41	2.20E-52	↓	NADH dehydrogenase [ubiquinone] iron-sulfur protein 2, mitochondrial, partial [B. mutus]
ENSP00000358160-D3	1.41	4.20E-04	↓	histone H3.1-like [B. mutus]
ENSBTAP00000014034-D1	1.41	4.20E-04	↓	methyl-CpG-binding domain protein 6 [Bison bison bison]
ENSP00000292205-D1	1.41	2.65E-86	↓	double-stranded RNA-specific adenosine deaminase isoform X1 [Bison bison bison]
ENSBTAP00000028236-D1	1.41	5.26E-12	↓	39S ribosomal protein L16, mitochondrial [B. mutus]
ENSP00000377385-D1	1.41	4.21E-142	↓	Suppressor of SWI4 1-like protein, partial [B. mutus]
ENSP00000413130-D1	1.41	2.47E-125	↓	TPA: glucosamine (N-acetyl)-6-sulfatase precursor [B. taurus]
ENSP00000410403-D1	1.41	9.86E-05	↓	ras-related protein Rab-34 [B. mutus]
ENSBTAP00000013946-D1	1.42	1.57E-23	↓	centrosomal protein of 76 kDa [B. taurus]
ENSP00000386765-D1	1.42	4.02E-163	↓	PERQ amino acid-rich with GYF domain-containing protein 2, partial [B. mutus]
ENSBTAP00000009938-D1	1.42	2.34E-05	↓	potassium voltage-gated channel subfamily A member 5 [B. mutus]
ENSP00000180173-D1	1.42	1.34E-50	↓	Myotubularin-related protein 7, partial [B. mutus]
ENSBTAP00000053533-D1	1.42	6.39E-17	↓	gamma-tubulin complex component 3 [B. mutus]
yakG023620	1.42	4.62E-47	↓	RING finger protein 38 [B. mutus]
ENSBTAP00000023803-D1	1.42	1.16E-10	↓	Conserved oligomeric Golgi complex subunit 3, partial [B. mutus]
ENSBTAP00000024607-D1	1.42	4.41E-91	↓	AF4/FMR2 family member 1, partial [B. mutus]
ENSBTAP00000020731-D1	1.42	3.99E-240	↓	Endoplasmic reticulum metallopeptidase 1, partial [B. mutus]

ENSP00000263274-D1	1.42	0.00E+00	↓	DNA ligase 1, partial [B. mutus]
ENSP00000319979-D1	1.42	7.76E-08	↓	UDP-GlcNAc:betaGal beta-1,3-N-acetylglucosaminyltransferase-like protein 1, partial [B. mutus]
ENSP00000296824-D1	1.42	1.39E-15	↓	coiled-coil domain-containing protein 127 [B. taurus]
ENSBTAP00000053583-D1	1.42	6.08E-36	↓	rho GTPase-activating protein 26 isoform X2 [B. mutus]
ENSBTAP00000005936-D1	1.42	2.88E-19	↓	probable methyltransferase TARBP1, partial [B. mutus]
ENSBTAP00000024059-D1	1.42	1.57E-11	↓	phenylalanine--tRNA ligase, mitochondrial [B. mutus]
ENSBTAP00000025697-D1	1.42	7.84E-108	↓	Glyoxylate reductase/hydroxypyruvate reductase, partial [B. mutus]
ENSP00000364805-D1	1.42	1.39E-22	↓	heat shock 70 kDa protein 1-like [B. taurus]
ENSP00000384817-D1	1.42	7.06E-27	↓	Paired box protein Pax-9, partial [B. mutus]
yakA28237	1.42	2.15E-222	↓	Mediator of RNA polymerase II transcription subunit 1 [B. mutus]
ENSP00000260102-D1	1.42	6.41E-65	↓	39S ribosomal protein L15, mitochondrial precursor [B. taurus]
ENSBTAP00000037356-D1	1.42	5.79E-76	↓	Serine/arginine repetitive matrix protein 2 [B. mutus]
ENSP00000306129-D1	1.42	8.25E-31	↓	D(1B) dopamine receptor [B. mutus]
ENSP00000356057-D1	1.42	3.04E-14	↓	Transmembrane protein 181, partial [B. mutus]
ENSP00000280886-D1	1.42	2.32E-04	↓	disco-interacting protein 2 homolog C [B. taurus]
ENSBTAP00000010385-D1	1.42	9.95E-04	↓	N-acetylglucosamine-1-phosphodiester alpha-N-acetylglucosaminidase [B. mutus]
ENSBTAP00000016106-D1	1.42	4.36E-03	↓	probable G-protein coupled receptor 128 [B. mutus]
ENSBTAP00000007726-D1	1.42	4.36E-03	↓	protein FAM83A [B. mutus]
ENSP00000393043-D1	1.42	4.36E-03	↓	ankyrin and armadillo repeat-containing protein isoform X1 [B. mutus]
yakG027591	1.42	2.02E-02	↓	histone H2B type 1-like [Propithecus coquereli]
ENSBTAP00000034988-D1	1.42	2.02E-02	↓	gamma-glutamyltransferase 6 [B. mutus]
ENSBTAP00000052227-D1	1.42	2.02E-02	↓	hypothetical protein M91_00465, partial [B. mutus]
ENSP00000289932-D1	1.42	2.02E-02	↓	Sodium/myo-inositol cotransporter 2, partial [B. mutus]
ENSP00000366581-D9	1.42	2.02E-02	↓	hypothetical protein M91_12052, partial [B. mutus]
ENSP00000374855-D30	1.43	2.30E-282	↓	hypothetical protein M91_09155, partial [B. mutus]
ENSBTAP00000008406-D1	1.43	8.57E-19	↓	THAP domain-containing protein 11 [B. mutus]
ENSBTAP00000010347-D1	1.43	1.77E-84	↓	glucosamine-6-phosphate isomerase 1 [Camelus ferus]
ENSP00000327536-D1	1.43	4.14E-15	↓	Roundabout-like protein 2, partial [B. mutus]
ENSP00000300965-D1	1.43	4.58E-73	↓	hypothetical protein EGK_10294 [Macaca mulatta]
ENSBTAP00000027917-D1	1.43	2.03E-11	↓	tumor suppressor candidate 2 [B. taurus]
ENSP00000248975-D2	1.43	2.10E-47	↓	14-3-3 protein eta, partial [B. mutus]
ENSBTAP00000018659-D1	1.43	0.00E+00	↓	Ras GTPase-activating protein-binding protein 2, partial [B. mutus]
ENSP00000370719-D1	1.43	2.31E-15	↓	intersectin-1 isoform X1 [B. mutus]
yakG020864	1.43	3.85E-21	↓	Protein piccolo [B. mutus]
ENSP00000403163-D1	1.43	1.96E-145	↓	VPS33B-interacting protein, partial [B. mutus]
ENSP00000417470-D1	1.43	1.29E-15	↓	Zinc finger protein 786, partial [B. mutus]
ENSBTAP00000000479-D1	1.43	2.17E-87	↓	epidermal growth factor receptor kinase substrate 8 isoform X2 [Bubalus bubalis]
ENSBTAP00000024147-D1	1.43	2.95E-207	↓	Denticleless protein-like protein, partial [B. mutus]
ENSBTAP00000003261-D1	1.43	1.43E-25	↓	Heterogeneous nuclear ribonucleoprotein U-like protein 2, partial [B. mutus]
ENSP00000391641-D15	1.43	3.56E-71	↓	Zinc finger protein 2, partial [B. mutus]
ENSP00000329034-D1	1.43	4.61E-239	↓	atlastin-3 [B. taurus]
ENSBTAP00000029015-D1	1.43	7.30E-295	↓	proheparin-binding EGF-like growth factor [B. mutus]
ENSP00000331327-D1	1.43	3.04E-05	↓	Wilms tumor protein isoform X1 [Otolemur garnettii]
ENSBTAP00000011134-D1	1.43	6.67E-221	↓	Large subunit GTPase 1-like protein [B. mutus]
ENSP00000266269-D1	1.43	5.59E-08	↓	POZ-, AT hook-, and zinc finger-containing protein 1 [B. taurus]
ENSP00000379430-D1	1.43	4.07E-16	↓	NADH dehydrogenase (ubiquinone) complex I, assembly factor 6 [B. mutus]
ENSBTAP00000020811-D1	1.43	2.38E-32	↓	Transmembrane protein 194B, partial [B. mutus]
ENSP00000366280-D1	1.43	8.61E-13	↓	VIP36-like protein precursor [B. taurus]
ENSBTAP00000022381-D1	1.44	4.47E-19	↓	WD repeat-containing protein 46 isoform X1 [B. mutus]
ENSBTAP00000009250-D1	1.44	1.02E-89	↓	3-oxoacyl-[acyl-carrier-protein] synthase, mitochondrial [B. mutus]
ENSBTAP00000025183-D1	1.44	4.83E-13	↓	Glutamine-rich protein 1 [B. mutus]
ENSP00000386040-D1	1.44	4.89E-103	↓	ATP-citrate synthase isoform X2 [B. mutus]
ENSP00000384899-D1	1.44	1.74E-81	↓	Transcriptional repressor p66-alpha, partial [B. mutus]
ENSP00000379795-D1	1.44	5.48E-04	↓	CKLF-like MARVEL transmembrane domain-containing protein 7, partial [B. mutus]
ENSBTAP00000010226-D1	1.44	1.03E-09	↓	N-glycosylase/DNA lyase [B. taurus]

ENSBTAP00000046684-D1	1.44	6.74E-16	↓	Ankyrin repeat domain-containing protein 16, partial [B. mutus]
ENSBTAP00000050620-D1	1.44	2.24E-17	↓	Guanine nucleotide-binding protein subunit alpha-11, partial [B. mutus]
ENSP00000415430-D1	1.44	2.69E-57	↓	G2 and S phase-expressed protein 1, partial [B. mutus]
ENSP00000245323-D1	1.44	2.36E-03	↓	ephrin-B2, partial [B. mutus]
ENSBTAP00000008961-D1	1.44	3.52E-49	↓	E3 ubiquitin-protein ligase CBL, partial [B. mutus]
ENSP00000394117-D1	1.44	0.00E+00	↓	F-box only protein 34 [B. mutus]
ENSP00000261722-D1	1.45	1.43E-12	↓	AP-3 complex subunit beta-2 isoform X2 [B. mutus]
ENSBTAP00000025736-D1	1.45	1.61E-17	↓	N(2),N(2)-dimethylguanosine tRNA methyltransferase, partial [B. mutus]
ENSP00000343676-D1	1.45	2.71E-219	↓	gap junction alpha-4 protein [B. mutus]
ENSBTAP00000052082-D1	1.45	3.00E-04	↓	integrator complex subunit 11 [B. mutus]
ENSP00000235372-D1	1.45	4.00E-107	↓	PR domain zinc finger protein 2, partial [B. mutus]
ENSBTAP00000013096-D46	1.45	4.80E-35	↓	hypothetical protein M91_18033, partial [B. mutus]
ENSP00000347427-D1	1.45	1.01E-10	↓	Misshapen-like kinase 1, partial [B. mutus]
ENSBTAP00000041245-D1	1.45	2.25E-08	↓	multiple epidermal growth factor-like domains protein 9, partial [B. mutus]
ENSP00000263062-D1	1.45	0.00E+00	↓	enhancer of polycomb homolog 1 isoform X1 [B. mutus]
ENSP00000399258-D1	1.45	6.00E-44	↓	Laminin subunit alpha-3, partial [B. mutus]
ENSBTAP00000004174-D1	1.45	2.08E-17	↓	Kinesin-like protein KIF3B, partial [B. mutus]
ENSP00000010299-D1	1.45	5.12E-80	↓	Protein FAM76A, partial [B. mutus]
ENSBTAP00000053622-D1	1.45	1.73E-21	↓	Echinoderm microtubule-associated protein-like 5, partial [B. mutus]
ENSBTAP00000010384-D1	1.45	2.85E-172	↓	sodium-dependent phosphate transporter 1 [B. mutus]
ENSP00000359827-D1	1.45	1.03E-12	↓	protein FAM73A [Bison bison bison]
ENSBTAP00000000541-D1	1.45	1.78E-134	↓	RNA-binding protein 4 [B. taurus]
ENSP00000366190-D1	1.45	1.22E-30	↓	Dr1-associated corepressor, partial [B. mutus]
ENSBTAP00000004027-D1	1.45	3.06E-22	↓	U4/U6.U5 tri-snRNP-associated protein 1 [B. mutus]
ENSBTAP00000052925-D1	1.46	3.63E-18	↓	BH3-interacting domain death agonist, partial [B. mutus]
ENSBTAP00000053355-D1	1.46	1.58E-63	↓	Myosin-Ie, partial [B. mutus]
ENSBTAP00000022419-D1	1.46	1.58E-186	↓	BUD13 homolog isoform X1 [B. mutus]
ENSBTAP00000015061-D1	1.46	6.18E-28	↓	E3 ubiquitin-protein ligase TRIM68 [B. mutus]
ENSBTAP00000008682-D1	1.46	3.22E-13	↓	hepatic leukemia factor [Ovis aries]
ENSBTAP00000016912-D1	1.46	1.75E-11	↓	HAUS augmin-like complex subunit 4 isoform X1 [B. mutus]
ENSBTAP00000003176-D1	1.46	2.89E-06	↓	hypothetical protein M91_13779 [B. mutus]
ENSBTAP00000020343-D1	1.46	1.29E-03	↓	testis-expressed protein 19.2-like [B. mutus]
ENSP00000392093-D1	1.46	1.29E-03	↓	rhomboid domain-containing protein 3 [B. mutus]
ENSBTAP00000018697-D1	1.46	1.29E-03	↓	Neurexin-1-alpha, partial [B. mutus]
ENSP00000262593-D1	1.46	1.06E-02	↓	docking protein 5-like [B. mutus]
ENSBTAP00000009350-D1	1.46	1.06E-02	↓	Transmembrane protein 143 [B. mutus]
ENSBTAP00000042479-D9	1.46	1.06E-02	↓	histone H2A type 1-like [Ursus maritimus]
ENSBTAP00000011195-D1	1.46	1.06E-02	↓	Zinc finger MYM-type protein 3 [B. mutus]
ENSP00000311038-D89	1.46	1.06E-02	↓	olfactory receptor 4X2-like [B. mutus]
ENSBTAP00000018533-D1	1.46	0.00E+00	↓	U4/U6 small nuclear ribonucleoprotein Prp3 [Homo sapiens]
ENSP00000262384-D1	1.46	7.34E-24	↓	NEDD4-binding protein 1, partial [B. mutus]
ENSBTAP00000044540-D1	1.46	7.34E-24	↓	acylpyruvase FAHD1, mitochondrial [B. taurus]
ENSBTAP00000016102-D1	1.46	8.16E-75	↓	la-related protein 1 isoform X2 [Ovis aries musimon]
ENSP00000401574-D1	1.46	2.43E-14	↓	hypothetical protein M91_14931 [B. mutus]
ENSBTAP00000027347-D1	1.46	2.40E-37	↓	Procollagen C-endopeptidase enhancer 1, partial [B. mutus]
ENSP00000361512-D3	1.46	2.77E-93	↓	ribose-phosphate pyrophosphokinase 2 isoform X2 [B. mutus]
ENSBTAP00000020641-D1	1.46	2.89E-08	↓	nuclear receptor subfamily 0 group B member 1 [B. taurus]
ENSP00000294129-D1	1.46	2.89E-08	↓	NCK-interacting protein with SH3 domain, partial [B. mutus]
ENSP00000376065-D1	1.46	2.06E-44	↓	Zinc finger protein 181, partial [B. mutus]
ENSBTAP00000002369-D1	1.46	0.00E+00	↓	Histone H1oo [B. mutus]
ENSBTAP00000026451-D2	1.46	3.19E-51	↓	E3 ubiquitin-protein ligase SMURF1, partial [B. mutus]
ENSP00000366477-D1	1.46	1.58E-22	↓	MAM and LDL-receptor class A domain-containing protein 1 [Bison bison bison]
ENSP00000416696-D1	1.46	5.74E-69	↓	protein RIC1 homolog isoform X1 [B. mutus]
ENSBTAP00000026674-D2	1.47	1.05E-18	↓	uncharacterized protein C1orf43 homolog [B. mutus]
ENSBTAP00000022217-D1	1.47	7.71E-18	↓	ribosome biogenesis regulatory protein homolog [B. mutus]

ENSBTAP00000003661-D1	1.47	6.69E-06	↓	E3 ubiquitin-protein ligase RNF185 [Otolemur garnettii]
ENSBTAP00000053695-D1	1.47	7.06E-04	↓	ski oncogene, partial [B. mutus]
ENSBTAP00000011888-D1	1.47	5.32E-13	↓	NADPH oxidase 5, partial [B. mutus]
ENSP00000403954-D2	1.47	4.31E-18	↓	Teneurin-4 [B. mutus]
ENSBTAP00000048712-D1	1.47	3.56E-23	↓	Nuclear receptor-binding factor 2 [B. mutus]
ENSBTAP00000012421-D1	1.47	8.20E-289	↓	erythroid differentiation-related factor 1 [B. mutus]
ENSBTAP00000002157-D1	1.47	1.99E-38	↓	Midasin [B. mutus]
ENSP00000263576-D1	1.47	2.64E-234	↓	ATP-dependent RNA helicase DDX25, partial [B. mutus]
ENSBTAP00000039445-D1	1.47	4.85E-55	↓	LIM and calponin homology domains-containing protein 1 [B. taurus]
ENSBTAP00000004636-D1	1.47	3.71E-08	↓	Tripartite motif-containing protein 3, partial [B. mutus]
ENSBTAP00000027119-D1	1.47	5.30E-62	↓	Astacin-like metalloendopeptidase, partial [B. mutus]
ENSP00000254480-D1	1.47	8.02E-39	↓	SWI/SNF complex subunit SMARCC1, partial [B. mutus]
ENSBTAP00000015405-D1	1.47	1.25E-14	↓	Inositol hexakisphosphate kinase 1, partial [B. mutus]
ENSBTAP00000024485-D1	1.47	9.26E-14	↓	TSC22 domain family protein 2, partial [B. mutus]
ENSP00000306522-D1	1.47	3.09E-18	↓	Calmodulin-binding transcription activator 1, partial [B. mutus]
ENSP00000215980-D1	1.47	6.82E-13	↓	centromere protein M [B. taurus]
ENSP00000362469-D1	1.48	1.54E-07	↓	Solute carrier family 2, facilitated glucose transporter member 8 [B. mutus]
ENSP00000268719-D1	1.48	2.07E-06	↓	glucose-induced degradation protein 4 homolog [B. taurus]
ENSP00000246914-D1	1.48	2.07E-06	↓	Serine/threonine-protein kinase WNK4, partial [B. mutus]
ENSBTAP00000011449-D1	1.48	5.71E-03	↓	Tetratricopeptide repeat protein 15, partial [B. mutus]
ENSP00000358154-D7	1.48	5.71E-03	↓	hypothetical protein M91_02497, partial [B. mutus]
ENSBTAP00000049224-D1	1.48	5.71E-03	↓	protein FAM136A-like [Bubalus bubalis]
yakG010089	1.48	5.71E-03	↓	hypothetical protein M91_11573 [B. mutus]
yakG013905	1.48	2.61E-137	↓	Rab3 GTPase-activating protein non-catalytic subunit [B. mutus]
yakG034077	1.48	6.94E-38	↓	Deubiquitinating protein VCIP135 [B. mutus]
ENSBTAP00000014517-D1	1.48	5.02E-15	↓	cysteine/serine-rich nuclear protein 2 [B. mutus]
ENSBTAP00000006175-D1	1.48	6.45E-57	↓	Immunoglobulin-like domain-containing receptor 1, partial [B. mutus]
ENSBTAP00000046380-D1	1.48	1.63E-17	↓	Histone H3-like centromeric protein A, partial [B. mutus]
ENSP00000347538-D1	1.48	1.55E-05	↓	Synaptotagmin-17, partial [B. mutus]
ENSP00000297770-D1	1.48	1.55E-05	↓	Carboxypeptidase A6, partial [B. mutus]
ENSP00000261819-D1	1.48	1.57E-15	↓	Anaphase-promoting complex subunit 5 [B. mutus]
ENSP00000304292-D1	1.48	8.54E-11	↓	ankyrin repeat domain-containing protein 27 isoform X1 [B. mutus]
ENSP00000381779-D1	1.48	2.24E-109	↓	exportin-5 isoform X2 [Bubalus bubalis]
ENSBTAP00000029302-D1	1.48	6.37E-68	↓	protein PRRC1 [B. mutus]
ENSBTAP00000003135-D1	1.49	3.37E-143	↓	ras-related protein Rab-30 [Homo sapiens]
ENSP00000328203-D1	1.49	1.74E-29	↓	TRAF-interacting protein [B. mutus]
ENSBTAP00000026195-D1	1.49	1.29E-178	↓	Serine/threonine-protein kinase 24, partial [B. mutus]
ENSBTAP00000026122-D1	1.49	9.13E-167	↓	intraflagellar transport protein 52 homolog [B. taurus]
ENSBTAP00000053453-D1	1.49	5.03E-22	↓	homeobox protein Meis2-like isoform X1 [B. mutus]
ENSP00000272930-D3	1.49	1.79E-35	↓	NEDD8-conjugating enzyme UBE2F [B. taurus]
ENSBTAP00000020766-D1	1.49	4.74E-14	↓	Inositol monophosphatase 3, partial [B. mutus]
ENSP00000379861-D1	1.49	3.64E-18	↓	MKL/myocardin-like protein 1, partial [B. mutus]
ENSP00000261530-D1	1.49	8.09E-46	↓	G patch domain-containing protein 2-like isoform X1 [B. mutus]
ENSBTAP00000000639-D1	1.49	1.97E-07	↓	kelch-like protein 13 isoform X3 [Ailuropoda melanoleuca]
ENSBTAP00000000862-D1	1.49	1.47E-88	↓	Protein FAM83D, partial [B. mutus]
ENSP00000258455-D1	1.49	7.48E-38	↓	28S ribosomal protein S9, mitochondrial [B. mutus]
ENSP00000281243-D1	1.49	2.05E-21	↓	Dihydropteridine reductase, partial [B. mutus]
ENSBTAP00000004037-D1	1.49	1.14E-63	↓	Eukaryotic translation initiation factor 4E type 1B, partial [B. mutus]
ENSBTAP00000053633-D1	1.49	6.69E-28	↓	Ectonucleoside triphosphate diphosphohydrolase 4, partial [B. mutus]
ENSBTAP00000018809-D1	1.49	4.93E-140	↓	homeobox protein PKNOX1 [B. mutus]
ENSBTAP00000044312-D1	1.49	2.65E-06	↓	protein NOXP20 [B. mutus]
ENSP00000377665-D1	1.49	2.57E-12	↓	GTPase-activating protein and VPS9 domain-containing protein 1 [Capra hircus]
ENSBTAP00000006410-D1	1.49	2.57E-12	↓	N-acetyltransferase 15 [B. mutus]
ENSP00000324804-D2	1.49	2.60E-18	↓	serine/threonine-protein phosphatase 2A 65 kDa regulatory subunit A alpha isoform-like isoform X1 [P.hodgsonii]
ENSP00000418397-D1	1.49	6.53E-05	↓	Isovaleryl-CoA dehydrogenase, mitochondrial, partial [B. mutus]

ENSP00000327145-D1	1.49	6.53E-05	↓	Filamin-C [B. mutus]
ENSBTAP00000022154-D1	1.49	1.44E-09	↓	abhydrolase domain-containing protein 4 [B. mutus]
ENSBTAP00000018985-D1	1.50	1.89E-14	↓	protein Wnt-2b [B. mutus]
ENSBTAP00000027893-D1	1.50	8.04E-10	↓	Annexin A11 [B. mutus]
ENSP00000398837-D1	1.50	8.22E-26	↓	Histone-lysine N-methyltransferase MLL4, partial [B. mutus]
ENSP00000261692-D1	1.50	1.49E-32	↓	cyclin-dependent kinase 2-associated protein 1 [Sus scrofa]
ENSP00000309463-D1	1.50	1.20E-53	↓	putative lipoyltransferase 2, mitochondrial [B. mutus]
ENSBTAP00000029071-D1	1.50	8.31E-33	↓	probable ATP-dependent RNA helicase DDX23 isoform X2 [Pteropus vampyrus]
ENSBTAP00000036773-D3	1.50	1.67E-03	↓	40S ribosomal protein S29-like [B. mutus]
ENSP00000347464-D1	1.50	1.57E-52	↓	ARF GTPase-activating protein GIT2, partial [B. mutus]
ENSP00000324205-D1	1.50	5.80E-19	↓	charged multivesicular body protein 4a [B. mutus]
ENSBTAP00000006685-D1	1.50	3.28E-12	↓	Disrupted in renal carcinoma protein 2-like protein [B. mutus]
ENSP00000366527-D1	1.50	4.54E-07	↓	Zinc finger and SCAN domain-containing protein 16 [B. mutus]
ENSBTAP00000038279-D1	1.50	1.37E-17	↓	Roquin [B. mutus]
ENSP00000262878-D1	1.50	1.81E-19	↓	SAM domain and HD domain-containing protein 1 [B. mutus]
ENSBTAP00000050033-D1	1.50	1.99E-05	↓	Putative prolyl-tRNA synthetase, mitochondrial, partial [B. mutus]
ENSBTAP00000001064-D1	1.50	1.35E-14	↓	zinc finger protein 667 [B. taurus]
ENSP00000274849-D1	1.50	1.79E-16	↓	activator of basal transcription 1 [B. taurus]
ENSP00000407163-D1	1.50	3.31E-09	↓	pleckstrin homology domain-containing family M member 2 [B. mutus]
ENSBTAP00000024965-D1	1.50	1.79E-67	↓	5'-AMP-activated protein kinase catalytic subunit alpha-2, partial [B. mutus]
ENSP00000254301-D1	1.50	2.40E-62	↓	galectin-3 [Bison bison bison]
ENSBTAP00000005830-D1	1.50	1.76E-20	↓	diacylglycerol kinase epsilon [B. mutus]
ENSBTAP00000038799-D1	1.50	4.08E-71	↓	apolipoprotein B-100 [B. mutus]
ENSP00000411465-D1	1.50	1.10E-05	↓	cystinosin isoform X2 [B. taurus]
ENSBTAP00000047683-D1	1.50	9.14E-04	↓	Putative carboxypeptidase PM20D1 [B. mutus]
ENSP00000412663-D1	1.50	9.14E-04	↓	glycogen synthase kinase-3 alpha [B. mutus]
ENSBTAP00000040974-D1	1.50	9.14E-04	↓	mucosal addressin cell adhesion molecule 1 [B. mutus]
ENSP00000371682-D1	1.50	9.14E-04	↓	DDB1- and CUL4-associated factor 16 [Lipotes vexillifer]
ENSBTAP00000053253-D1	1.51	1.60E-75	↓	golgi-specific brefeldin A-resistance guanine nucleotide exchange factor 1 isoform X1 [B. mutus]
ENSBTAP00000022271-D1	1.51	0.00E+00	↓	N-acetyltransferase 10, partial [B. mutus]
ENSP00000399013-D3	1.51	7.85E-08	↓	Contactin-associated protein-like 5, partial [B. mutus]
ENSBTAP00000004631-D1	1.51	7.14E-28	↓	Apoptosis-stimulating of p53 protein 1, partial [B. mutus]
ENSBTAP00000019080-D2	1.51	2.90E-42	↓	RING finger protein 126, partial [B. mutus]
ENSBTAP00000053525-D1	1.51	5.02E-04	↓	EVI5-like protein [B. mutus]
ENSP00000243389-D1	1.51	1.64E-23	↓	proton-coupled amino acid transporter 1 isoform X2 [B. taurus]
ENSBTAP00000023149-D1	1.51	1.23E-21	↓	Heat shock factor 2-binding protein, partial [B. mutus]
ENSP00000404121-D1	1.51	9.07E-88	↓	Interleukin enhancer-binding factor 3, partial [B. mutus]
ENSP00000369274-D1	1.51	3.52E-183	↓	phosphorylase b kinase regulatory subunit alpha, liver isoform [B. mutus]
ENSP00000329360-D1	1.51	1.67E-15	↓	coiled-coil domain-containing protein 137 [B. mutus]
ENSBTAP00000036625-D1	1.51	0.00E+00	↓	Hydroxymethylglutaryl-CoA synthase, cytoplasmic, partial [B. mutus]
ENSP00000259737-D1	1.51	8.99E-25	↓	protein YIPF3 [B. mutus]
ENSP00000217173-D1	1.51	2.28E-13	↓	RING finger protein 37 isoform X1 [B. mutus]
ENSP00000264515-D1	1.51	0.00E+00	↓	retinoblastoma-binding protein 5 isoform X3 [Microcebus murinus]
ENSP00000343155-D2	1.51	1.20E-68	↓	X-box-binding protein 1, partial [B. mutus]
ENSBTAP00000006412-D2	1.51	2.92E-16	↓	rab-like protein 2A-like [B. mutus]
ENSP00000263228-D2	1.51	1.73E-11	↓	ubiquitin-conjugating enzyme E2 R1 [B. taurus]
ENSP00000359729-D1	1.51	1.05E-06	↓	Sodium/hydrogen exchanger 6, partial [B. mutus]
ENSP00000392549-D1	1.51	2.75E-04	↓	tubulin monoglycylase TTLL3 [B. mutus]
ENSP00000363708-D1	1.51	1.22E-101	↓	bone morphogenetic protein receptor type-2 [B. mutus]
ENSBTAP00000053635-D1	1.51	2.36E-153	↓	DENN domain-containing protein 5A, partial [B. mutus]
ENSP00000378363-D1	1.52	5.36E-12	↓	DnaJ-like protein subfamily B member 12, partial [B. mutus]
ENSP00000263734-D1	1.52	1.80E-82	↓	Endothelial PAS domain-containing protein 1 [B. mutus]
ENSP00000413737-D1	1.52	1.34E-42	↓	tafazzin isoform X4 [Bubalus bubalis]
ENSBTAP00000001081-D1	1.52	1.31E-09	↓	PR domain zinc finger protein 1 [B. mutus]
ENSP00000366702-D1	1.52	1.31E-09	↓	ERBB receptor feedback inhibitor 1 [B. taurus]

ENSP00000265334-D1	1.52	1.51E-04	↓	cyclin-dependent kinase-like 3 [B. mutus]
ENSBTAP00000039471-D1	1.52	1.51E-04	↓	latrophilin-3-like isoform X3 [B. mutus]
ENSBTAP00000020194-D1	1.52	2.68E-23	↓	protease, serine, 23 [B. mutus]
ENSBTAP00000027370-D1	1.52	1.55E-18	↓	SH2 domain-containing adapter protein E, partial [B. mutus]
ENSBTAP00000022527-D1	1.52	1.44E-60	↓	hypothetical protein M91_00849 [B. mutus]
ENSP00000215061-D1	1.52	8.62E-19	↓	Occludin/ELL domain-containing protein 1, partial [B. mutus]
ENSP00000357048-D1	1.52	2.47E-186	↓	coatomer subunit alpha isoform X1 [B. mutus]
ENSBTAP00000016104-D1	1.52	7.70E-31	↓	Homeobox protein SIX1, partial [B. mutus]
ENSP00000398778-D1	1.52	1.00E-07	↓	RELT-like protein 1 [B. mutus]
ENSBTAP00000029247-D1	1.52	8.37E-05	↓	SH3 domain-binding protein 1, partial [B. mutus]
ENSBTAP00000027115-D1	1.52	0.00E+00	↓	calponin-3 isoform X1 [B. mutus]
ENSP00000321706-D1	1.52	5.05E-14	↓	gem-associated protein 4 [B. mutus]
ENSBTAP00000000364-D1	1.52	1.57E-14	↓	Putative 2-oxoglutarate dehydrogenase E1 component DHKTD1, mitochondrial, partial [B. mutus]
ENSBTAP00000042527-D1	1.52	6.32E-18	↓	Inner centromere protein [B. mutus]
ENSP00000300584-D1	1.52	1.23E-11	↓	TBC1 domain family member 2B, partial [B. mutus]
ENSBTAP00000004811-D1	1.52	0.00E+00	↓	WD repeat domain phosphoinositide-interacting protein 3, partial [B. mutus]
ENSBTAP00000014625-D1	1.52	1.11E-269	↓	Protein phosphatase Slingshot-like protein 2 [B. mutus]
ENSBTAP00000003493-D1	1.53	3.08E-27	↓	ubiquitin-protein ligase E3B isoform X1 [B. mutus]
ENSP00000320543-D1	1.53	1.68E-09	↓	epsin-2 isoform X2 [B. mutus]
ENSP00000348128-D1	1.53	3.18E-123	↓	supervillin [B. mutus]
ENSP00000322016-D1	1.53	7.42E-25	↓	Poly(U)-binding-splicing factor PUF60, partial [B. mutus]
ENSBTAP00000009440-D1	1.53	1.96E-98	↓	aspartate aminotransferase, mitochondrial isoform X1 [B. mutus]
ENSP00000339834-D1	1.53	3.45E-60	↓	U11/U12 small nuclear ribonucleoprotein 48 kDa protein [B. mutus]
yakG018989	1.53	6.73E-53	↓	Ankyrin repeat and FYVE domain-containing protein 1 [B. mutus]
ENSP00000376394-D1	1.53	1.67E-49	↓	unknown [Homo sapiens]
ENSBTAP00000019817-D1	1.53	4.35E-06	↓	Myomesin-3 [B. mutus]
ENSBTAP00000028744-D1	1.53	3.42E-178	↓	DDB1- and CUL4-associated factor 12, partial [B. mutus]
ENSP00000328547-D1	1.53	1.73E-23	↓	DNA (cytosine-5)-methyltransferase 3B [B. mutus]
ENSBTAP00000000541-D2	1.53	9.68E-24	↓	RNA-binding protein 4B isoform X1 [Bubalus bubalis]
ENSP00000384776-D1	1.53	3.21E-59	↓	ataxin-1 [B. mutus]
ENSBTAP00000025396-D1	1.53	2.42E-06	↓	serine/threonine-protein kinase PAK 6 isoform X1 [B. mutus]
ENSP00000354964-D1	1.53	1.89E-73	↓	Zinc finger protein 318, partial [B. mutus]
ENSBTAP00000011251-D1	1.53	1.32E-20	↓	Suppressor of cytokine signaling 7, partial [B. mutus]
ENSBTAP00000018564-D1	1.53	1.34E-06	↓	uncharacterized protein C19orf52 homolog [B. taurus]
ENSBTAP00000020055-D1	1.53	2.59E-151	↓	LanC-like protein 1, partial [B. mutus]
ENSP00000319635-D2	1.53	7.43E-07	↓	c-X-C chemokine receptor type 2-like [B. mutus]
ENSP00000390427-D1	1.53	4.87E-12	↓	peptidyl-prolyl cis-trans isomerase-like 2 [B. mutus]
ENSBTAP00000007704-D1	1.53	4.82E-45	↓	Cyclic AMP-dependent transcription factor ATF-7, partial [B. mutus]
ENSP00000355623-D1	1.53	2.29E-07	↓	hypothetical protein M91_14831, partial [B. mutus]
ENSP00000355343-D1	1.53	0.00E+00	↓	Ubiquitin-associated protein 2-like protein [B. mutus]
ENSP00000259467-D1	1.53	8.19E-14	↓	Phosducin-like protein [B. mutus]
ENSP00000378110-D1	1.54	2.32E-149	↓	septin-6 isoform X1 [Trichechus manatus latirostris]
ENSP00000377904-D1	1.54	2.05E-154	↓	Synergin gamma, partial [B. mutus]
ENSBTAP00000031555-D1	1.54	3.85E-66	↓	Microcephalin, partial [B. mutus]
ENSP00000347170-D1	1.54	2.40E-16	↓	endonuclease 8-like 1 [B. mutus]
ENSP00000269385-D1	1.54	1.36E-45	↓	chromobox protein homolog 8 [B. mutus]
ENSBTAP00000005929-D1	1.54	6.64E-10	↓	potassium channel subfamily K member 1 [B. mutus]
ENSP00000395220-D1	1.54	1.41E-109	↓	synaptotagmin-like protein 5 isoform X1 [B. mutus]
ENSBTAP00000022213-D1	1.54	4.01E-61	↓	Peptidyl-prolyl cis-trans isomerase F, mitochondrial [B. mutus]
ENSBTAP00000010675-D1	1.54	4.54E-81	↓	polymerase delta-interacting protein 2 [Microcebus murinus]
ENSP00000311399-D1	1.54	2.14E-20	↓	Down syndrome critical region protein 3 isoform X1 [B. mutus]
ENSBTAP00000038030-D1	1.54	6.05E-67	↓	Bromodomain adjacent to zinc finger domain protein 2A, partial [B. mutus]
ENSBTAP00000008985-D1	1.54	7.05E-32	↓	ubiquitin-like modifier-activating enzyme 6 [B. mutus]
ENSBTAP00000029505-D1	1.54	4.26E-42	↓	THO complex subunit 5 homolog isoform X1 [B. taurus]
ENSBTAP00000029400-D1	1.54	2.51E-53	↓	cytospin-A-like, partial [B. mutus]

ENSBTAP00000019217-D1	1.54	4.40E-54	↓	RecName: Full=Serine/threonine-protein kinase PLK1; AltName: Full=Polo-like kinase 1; Short=PLK-1
ENSBTAP00000018722-D1	1.54	1.93E-12	↓	amphoterin-induced protein 1 precursor [B. taurus]
ENSP00000256646-D1	1.54	6.54E-48	↓	neurogenic locus notch homolog protein 2 [B. mutus]
ENSP00000338487-D1	1.54	1.80E-14	↓	alpha-1,3-mannosyl-glycoprotein 4-beta-N-acetylglucosaminyltransferase B [B. mutus]
ENSBTAP00000020651-D1	1.54	9.76E-29	↓	rho GTPase-activating protein 35 [B. taurus]
ENSP00000295640-D1	1.54	2.88E-31	↓	aminopeptidase B [B. taurus]
ENSBTAP00000003956-D1	1.54	2.97E-17	↓	ral GTPase-activating protein subunit beta isoform X1 [B. mutus]
ENSP00000401946-D1	1.54	1.72E-293	↓	trinucleotide repeat-containing gene 6B protein isoform X1 [Bubalus bubalis]
ENSBTAP00000042728-D1	1.55	1.31E-26	↓	ras-GEF domain-containing family member 1B isoform X1 [Bubalus bubalis]
ENSP00000339145-D1	1.55	3.35E-113	↓	Ribosomal RNA processing protein 1-like protein B, partial [B. mutus]
ENSBTAP00000032838-D1	1.55	1.01E-66	↓	Fos-related antigen 2 [B. mutus]
ENSP00000405818-D1	1.55	8.32E-31	↓	NUAK family SNF1-like kinase 2, partial [B. mutus]
ENSP00000383911-D1	1.55	3.29E-22	↓	ubiquitin-associated domain-containing protein 2 [B. mutus]
ENSP00000262144-D1	1.55	3.35E-21	↓	WD repeat-containing protein 59 [B. mutus]
ENSP00000254454-D1	1.55	2.38E-13	↓	hypothetical protein [Pongo abelii]
ENSBTAP00000025006-D1	1.55	4.27E-13	↓	Negative elongation factor D, partial [B. mutus]
ENSP00000331242-D1	1.55	1.57E-08	↓	Kremen protein 1, partial [B. mutus]
ENSP00000358677-D1	1.55	1.57E-08	↓	T-box transcription factor TBX18 [B. mutus]
ENSP00000393952-D1	1.55	9.06E-08	↓	tetratricopeptide repeat protein 39A [B. mutus]
ENSP00000293328-D1	1.55	5.25E-07	↓	Signal transducer and activator of transcription 5B [B. mutus]
ENSP00000305138-D1	1.55	5.25E-07	↓	Protein FAM195A, partial [B. mutus]
ENSP00000342307-D1	1.55	5.54E-06	↓	Forkhead box protein M1 [B. mutus]
ENSBTAP00000016673-D2	1.55	1.80E-05	↓	ORM1-like protein 3 [Pteropus alecto]
ENSBTAP00000011810-D1	1.55	3.26E-05	↓	Ankyrin repeat and SOCS box protein 6, partial [B. mutus]
ENSP00000380785-D1	1.55	1.94E-04	↓	Protein Hook-like protein 2, partial [B. mutus]
ENSP00000319756-D1	1.55	3.53E-04	↓	tensin-like C1 domain-containing phosphatase isoform X4 [B. taurus]
ENSP00000390475-D1	1.55	1.17E-03	↓	CpG-binding protein, partial [B. mutus]
ENSP00000307265-D1	1.55	1.17E-03	↓	interferon regulatory factor 2-binding protein 1-like [B. mutus]
ENSP00000338785-D1	1.55	2.15E-03	↓	stAR-related lipid transfer protein 13 [B. mutus]
ENSBTAP00000041865-D1	1.55	3.97E-03	↓	proline-rich protein 5-like [Capra hircus]
ENSP00000343348-D1	1.55	3.97E-03	↓	vimentin-type intermediate filament-associated coiled-coil protein [B. mutus]
ENSBTAP00000022431-D1	1.55	3.97E-03	↓	testosterone 17-beta-dehydrogenase 3 [B. mutus]
ENSBTAP00000022753-D1	1.55	3.97E-03	↓	GTP-binding protein Rhes, partial [B. mutus]
ENSP00000258775-D1	1.55	3.97E-03	↓	NAC-alpha domain-containing protein 1, partial [B. mutus]
ENSP00000320083-D1	1.55	3.97E-03	↓	N-acetylated-alpha-linked acidic dipeptidase 2, partial [B. mutus]
ENSBTAP00000050273-D68	1.55	7.39E-03	↓	hypothetical protein M91_01434, partial [B. mutus]
ENSBTAP00000041523-D1	1.55	7.39E-03	↓	CMT1A duplicated region transcript 4 protein homolog [B. mutus]
ENSBTAP00000052710-D1	1.55	7.39E-03	↓	Xaa-Pro dipeptidase, partial [B. mutus]
ENSBTAP00000023834-D1	1.55	7.39E-03	↓	dolichyldiphosphatase 1 [B. taurus]
ENSBTAP00000052592-D8	1.55	1.39E-02	↓	hypothetical protein M91_03393, partial [B. mutus]
ENSP00000341187-D1	1.55	1.39E-02	↓	cAMP and cAMP-inhibited cGMP 3',5'-cyclic phosphodiesterase 10A, partial [B. mutus]
ENSBTAP00000005160-D1	1.55	1.39E-02	↓	F-box/LRR-repeat protein 2, partial [B. mutus]
ENSBTAP00000048708-D1	1.55	1.39E-02	↓	Casein kinase I isoform alpha, partial [B. mutus]
ENSP00000355058-D1	1.55	1.39E-02	↓	cx9C motif-containing protein 4 [B. taurus]
ENSP00000381950-D2	1.55	1.39E-02	↓	Transmembrane protein 216, partial [B. mutus]
ENSP00000370343-D1	1.55	1.39E-02	↓	interferon regulatory factor 4 isoform X1 [B. mutus]
ENSP00000393725-D1	1.55	1.39E-02	↓	GDNF family receptor alpha-1 isoform X1 [B. mutus]
ENSBTAP00000037042-D1	1.55	2.71E-02	↓	Zinc-alpha-2-glycoprotein, partial [B. mutus]
ENSBTAP00000049914-D1	1.55	2.71E-02	↓	hypothetical protein M91_12293, partial [B. mutus]
ENSP00000349805-D1	1.55	2.71E-02	↓	hypothetical protein M91_07484, partial [B. mutus]
ENSP00000257935-D3	1.55	2.71E-02	↓	keratin, type II microfibrillar, component 7C-like [B. mutus]
ENSP00000334781-D9	1.55	2.71E-02	↓	olfactory receptor 5D13, partial [B. taurus]
ENSBTAP00000025485-D3	1.55	2.71E-02	↓	Oxysterol-binding protein 2, partial [B. mutus]
ENSP00000297130-D1	1.55	2.71E-02	↓	Myozenin-3, partial [B. mutus]
ENSBTAP00000028687-D1	1.55	2.93E-279	↓	Signal transducer and activator of transcription 3 [B. mutus]

ENSBTAP00000003291-D1	1.55	2.42E-64	↓	hypothetical protein M91_10077, partial [B. mutus]
ENSBTAP00000053365-D1	1.55	4.15E-163	↓	WD repeat and FYVE domain-containing protein 3, partial [B. mutus]
ENSP00000377059-D1	1.55	1.52E-38	↓	sorting nexin-11 [B. taurus]
ENSBTAP00000019607-D1	1.55	1.64E-35	↓	Putative ribosomal RNA methyltransferase NOP2 [B. mutus]
ENSP00000411459-D1	1.56	9.02E-215	↓	Rho-guanine nucleotide exchange factor, partial [B. mutus]
ENSBTAP00000018730-D1	1.56	4.79E-17	↓	phosphopantothenoylcysteine decarboxylase [B. mutus]
ENSBTAP00000047824-D1	1.56	8.58E-17	↓	ADP-ribosylation factor GTPase-activating protein 1 [B. mutus]
ENSP00000182527-D2	1.56	2.39E-30	↓	hypothetical protein M91_11384, partial [B. mutus]
ENSP00000355884-D4	1.56	2.39E-30	↓	Serine/threonine-protein kinase MARK2 [B. mutus]
ENSP00000281923-D1	1.56	1.85E-100	↓	alpha-1,6-mannosylglycoprotein 6-beta-N-acetylglucosaminyltransferase A [B. mutus]
ENSBTAP00000010239-D1	1.56	2.88E-23	↓	mediator complex subunit 12-like [Sus scrofa]
ENSP00000264758-D1	1.56	8.92E-60	↓	Alpha-adducin [B. mutus]
ENSP00000386921-D1	1.56	4.77E-87	↓	TBC1 domain family member 14 isoform X1 [B. mutus]
ENSP00000413971-D1	1.56	1.04E-10	↓	Transmembrane protein 233, partial [B. mutus]
yakG014377	1.56	3.74E-22	↓	hypothetical protein M91_11177 [B. mutus]
ENSP00000294409-D1	1.56	8.45E-40	↓	Glucocorticoid modulatory element-binding protein 1 [B. mutus]
ENSP00000376268-D1	1.57	1.10E-131	↓	SEC14-like protein 1 [B. mutus]
ENSP00000382004-D1	1.57	1.59E-210	↓	catenin delta-1 [B. mutus]
ENSBTAP00000025159-D1	1.57	3.70E-07	↓	Adrenocortical dysplasia protein-like protein, partial [B. mutus]
ENSBTAP00000041415-D1	1.57	1.55E-52	↓	formin-2 isoform X2 [B. taurus]
ENSP00000394869-D1	1.57	5.84E-32	↓	Translation initiation factor eIF-2B subunit delta [B. mutus]
ENSBTAP00000001071-D1	1.57	1.51E-21	↓	Coatomer subunit epsilon [B. mutus]
ENSBTAP00000005076-D1	1.57	3.81E-35	↓	Period circadian protein-like protein 1, partial [B. mutus]
ENSP00000368278-D1	1.57	2.28E-11	↓	interleukin-1 receptor accessory protein-like 1 isoform X2 [B. taurus]
ENSBTAP00000016363-D1	1.57	1.21E-06	↓	Zinc finger protein 524, partial [B. mutus]
ENSP00000416673-D1	1.57	2.77E-95	↓	Protein PAT1-like protein 1, partial [B. mutus]
ENSBTAP00000012776-D1	1.57	8.14E-90	↓	F-box only protein 15 [B. mutus]
ENSBTAP00000024328-D1	1.57	4.10E-11	↓	Solute carrier family 28 member 3 [B. mutus]
ENSBTAP00000016560-D1	1.57	4.10E-11	↓	CUB domain-containing protein 1, partial [B. mutus]
ENSBTAP00000007834-D1	1.57	1.31E-10	↓	Tolloid-like protein 1, partial [B. mutus]
ENSP00000387303-D1	1.57	1.31E-10	↓	Putative ATP-dependent RNA helicase TDRD9 [B. mutus]
ENSBTAP00000048061-D1	1.57	0.00E+00	↓	NACHT, LRR and PYD domains-containing protein 8, partial [B. mutus]
ENSBTAP00000007532-D1	1.57	5.45E-62	↓	Glycerol-3-phosphate acyltransferase 4, partial [B. mutus]
ENSBTAP00000042791-D1	1.57	2.99E-40	↓	trichoplein keratin filament-binding protein [B. taurus]
ENSBTAP00000021081-D1	1.57	2.35E-10	↓	WD repeat-containing protein WRAP73 [B. mutus]
ENSBTAP00000012348-D1	1.57	3.91E-06	↓	Type-1 angiotensin II receptor-associated protein, partial [B. mutus]
ENSP00000316490-D1	1.57	0.00E+00	↓	histone RNA hairpin-binding protein [B. mutus]
ENSBTAP00000034433-D1	1.57	7.03E-06	↓	B(0,+)-type amino acid transporter 1 [B. mutus]
ENSP00000370055-D1	1.57	4.95E-48	↓	ubiquitin-like protein 3 [Aotus nancymaae]
ENSP00000311713-D1	1.57	1.36E-09	↓	serine/threonine-protein kinase OSR1 isoform X1 [Ovis aries]
ENSP00000340883-D1	1.58	1.09E-20	↓	SWI/SNF-related matrix-associated actin-dependent regulator of chromatin subfamily B member 1 [B. mutus]
ENSBTAP00000010506-D1	1.58	1.27E-05	↓	hyaluronan synthase 3 [B. taurus]
ENSP00000400459-D1	1.58	3.52E-20	↓	Clathrin coat assembly protein AP180 [B. mutus]
ENSP00000259006-D1	1.58	2.29E-05	↓	LIM domain-containing protein 2 [B. mutus]
ENSP00000316649-D1	1.58	1.03E-14	↓	oxysterol-binding protein-related protein 2 isoform X1 [B. mutus]
ENSP00000385395-D1	1.58	5.11E-24	↓	Glutamate receptor delta-1 subunit, partial [B. mutus]
ENSP00000177742-D1	1.58	1.84E-14	↓	28S ribosomal protein S34, mitochondrial, partial [B. mutus]
ENSP00000334409-D2	1.58	9.14E-24	↓	GMP reductase 1 [B. mutus]
ENSBTAP00000004229-D1	1.58	3.30E-98	↓	oxysterols receptor LXR-beta [B. taurus]
ENSP00000336127-D1	1.58	2.88E-11	↓	Tetratricopeptide repeat protein 7B, partial [B. mutus]
ENSP00000385861-D1	1.58	3.84E-17	↓	Tuftelin-interacting protein 11 [B. mutus]
ENSP00000355014-D1	1.58	7.51E-05	↓	E3 ubiquitin-protein ligase Praja-1 isoform X1 [B. mutus]
ENSP00000371734-D1	1.58	7.53E-59	↓	KN motif and ankyrin repeat domain-containing protein 1, partial [B. mutus]
ENSBTAP00000013251-D1	1.58	2.63E-152	↓	DNA-directed RNA polymerase III subunit RPC4 [B. taurus]
ENSP00000258301-D1	1.58	2.63E-18	↓	Syntaxin-6 [B. mutus]

ENSP00000297440-D1	1.59	5.34E-10	↓	HEAT repeat-containing protein 2, partial [B. mutus]
ENSP00000268603-D1	1.59	1.36E-04	↓	cadherin-11 precursor [B. taurus]
ENSP00000346927-D1	1.59	1.36E-04	↓	N-acetyltransferase 6, partial [B. mutus]
ENSP00000311122-D1	1.59	2.25E-15	↓	transmembrane protein 52 [B. mutus]
ENSP00000376246-D1	1.59	1.48E-36	↓	hypothetical protein M91_13482, partial [B. mutus]
ENSBTAP00000048343-D1	1.59	9.58E-10	↓	AP-2 complex subunit alpha-2, partial [B. mutus]
ENSP00000260324-D1	1.59	3.77E-22	↓	sulfide:quinone oxidoreductase, mitochondrial [B. mutus]
ENSBTAP00000029961-D1	1.59	8.47E-07	↓	transmembrane protein 134 isoform X3 [B. taurus]
ENSBTAP00000052270-D1	1.59	2.48E-04	↓	cyclin-dependent kinase 5 activator 1 [B. mutus]
ENSBTAP00000001978-D1	1.59	2.48E-04	↓	B-cell lymphoma 6 protein [B. mutus]
ENSP00000363617-D1	1.59	1.59E-91	↓	ras-associated and pleckstrin homology domains-containing protein 1 isoform X1 [Homo sapiens]
ENSP00000417354-D1	1.59	1.90E-35	↓	Golgi integral membrane protein 4 isoform X1 [B. mutus]
ENSP00000331983-D1	1.59	5.58E-48	↓	leucine-rich repeat-containing protein 16A isoform X3 [B. taurus]
ENSP00000335615-D1	1.59	4.75E-45	↓	BTB/POZ domain-containing protein 7 [B. mutus]
ENSBTAP00000006363-D2	1.59	1.27E-42	↓	ADP-ribosylation factor-like protein 2-binding protein [B. taurus]
ENSP00000252530-D1	1.59	9.89E-09	↓	protein FAM98C [B. mutus]
ENSP00000370532-D1	1.59	7.10E-20	↓	transcription factor IIIA [B. mutus]
ENSP00000253814-D1	1.59	7.70E-134	↓	NEDD4 family-interacting protein 1 isoform X1 [Ovis aries]
ENSP00000293362-D1	1.59	3.86E-71	↓	proteasome activator complex subunit 3 isoform X2 [Odobenus rosmarus divergens]
ENSP00000387262-D1	1.59	8.98E-141	↓	Mitochondrial inner membrane protein, partial [B. mutus]
ENSP00000400939-D1	1.59	2.58E-28	↓	treacle protein isoform X2 [Ovis aries musimon]
ENSBTAP00000006153-D1	1.59	4.50E-04	↓	delta(24)-sterol reductase [B. mutus]
ENSP00000368651-D1	1.60	9.15E-15	↓	Folliculin-interacting protein 2 [B. mutus]
ENSP00000419045-D1	1.60	5.28E-36	↓	hypothetical protein M91_18587, partial [B. mutus]
ENSBTAP00000051318-D1	1.60	3.28E-23	↓	E3 ubiquitin-protein ligase NRDP1 [B. taurus]
ENSP00000258428-D1	1.60	9.55E-199	↓	DNA repair protein REV1 [B. mutus]
ENSP00000252453-D1	1.60	3.17E-08	↓	betatrophin [B. mutus]
ENSBTAP00000034759-D1	1.60	7.27E-161	↓	Integrator complex subunit 10, partial [B. mutus]
ENSP00000285848-D1	1.60	2.08E-36	↓	mitochondrial inner membrane protein OXA1L-like [Lipotes vexillifer]
ENSBTAP00000021613-D1	1.60	5.27E-14	↓	Retinoblastoma-like protein 2 [B. mutus]
ENSP00000286827-D1	1.60	3.16E-91	↓	T-lymphoma invasion and metastasis-inducing protein 1-like isoform X1 [B. mutus]
ENSP00000278951-D1	1.60	8.95E-20	↓	SID1 transmembrane family member 2, partial [B. mutus]
ENSBTAP00000009887-D1	1.60	3.41E-21	↓	Serine/threonine-protein phosphatase PGAM5, mitochondrial, partial [B. mutus]
ENSBTAP00000018768-D1	1.60	8.90E-06	↓	protein kinase C zeta type [B. taurus]
ENSP00000393444-D1	1.60	8.19E-04	↓	protein PAPPAS-like [Macaca mulatta]
ENSP00000341289-D3	1.60	3.59E-15	↓	tubulin beta-4B chain [Heterocephalus glaber]
ENSP00000405068-D1	1.60	2.16E-09	↓	Nucleolar protein 14, partial [B. mutus]
ENSBTAP00000022652-D1	1.60	1.84E-07	↓	serine/threonine-protein kinase N1 [B. mutus]
ENSP00000276282-D1	1.60	2.94E-18	↓	Malignant fibrous histiocytoma-amplified sequence 1, partial [B. mutus]
ENSBTAP00000001141-D1	1.60	9.71E-13	↓	F-box/LRR-repeat protein 6 [B. mutus]
ENSBTAP00000053503-D1	1.60	3.88E-09	↓	uncharacterized protein C11orf93 homolog [B. mutus]
ENSBTAP00000019774-D1	1.60	1.61E-05	↓	GPN-loop GTPase 2, partial [B. mutus]
ENSBTAP00000029616-D1	1.60	1.61E-05	↓	SH2B adapter protein 3, partial [B. mutus]
ENSBTAP00000032931-D1	1.61	0.00E+00	↓	rho GTPase-activating protein 25 isoform X1 [Bison bison bison]
ENSP00000324587-D1	1.61	6.63E-14	↓	Protein FAM193A, partial [B. mutus]
ENSP00000398704-D1	1.61	1.37E-25	↓	Short stature homeobox protein 2, partial [B. mutus]
ENSP00000348234-D1	1.61	5.95E-54	↓	tyrosine aminotransferase isoform X1 [B. mutus]
ENSBTAP00000009769-D1	1.61	1.02E-63	↓	trafficking protein particle complex subunit 11 isoform X2 [B. mutus]
ENSP00000328813-D1	1.61	5.41E-17	↓	Potassium voltage-gated channel subfamily H member 8, partial [B. mutus]
ENSBTAP00000053594-D1	1.61	5.41E-17	↓	Zinc finger ZZ-type and EF-hand domain-containing protein 1, partial [B. mutus]
ENSBTAP00000004291-D1	1.61	2.91E-05	↓	hypothetical protein M91_11543 [B. mutus]
ENSBTAP00000029353-D1	1.61	1.50E-03	↓	Tripartite motif-containing protein 65, partial [B. mutus]
ENSBTAP00000023500-D2	1.61	1.50E-03	↓	Interferon-induced guanylate-binding protein 1, partial [B. mutus]
ENSBTAP00000008629-D1	1.61	1.50E-03	↓	seizure 6-like protein [B. mutus]
ENSBTAP00000029309-D1	1.61	1.50E-03	↓	Transmembrane protein 18, partial [B. mutus]

ENSP00000261822-D1	1.61	2.14E-13	↓	B-cell CLL/lymphoma 7 protein family member A, partial [B. mutus]
ENSBTAP00000022288-D1	1.61	3.27E-289	↓	DNA mismatch repair protein Mlh1 [B. taurus]
ENSP00000385888-D1	1.61	1.28E-121	↓	Histone acetyltransferase MYST3 [B. mutus]
ENSBTAP00000007814-D1	1.61	3.10E-16	↓	radial spoke head protein 3 homolog [B. mutus]
ENSP00000385699-D1	1.61	8.48E-10	↓	Fatty aldehyde dehydrogenase [B. mutus]
ENSP00000383958-D2	1.61	8.48E-10	↓	nuclear factor 1 A-type-like, partial [Leptonychotes weddellii]
ENSP00000265978-D1	1.61	6.84E-13	↓	FTS and Hook-interacting protein [B. mutus]
ENSBTAP00000037931-D1	1.61	5.54E-16	↓	Ubiquitin-conjugating enzyme E2C-binding protein, partial [B. mutus]
ENSBTAP00000005899-D1	1.61	1.44E-23	↓	estrogen receptor beta [B. mutus]
ENSBTAP00000020843-D1	1.61	5.26E-05	↓	Vacuolar fusion protein MON1-like protein B, partial [B. mutus]
ENSP00000343899-D1	1.62	3.81E-17	↓	FERM domain-containing protein 6 isoform X1 [B. mutus]
ENSP00000332668-D1	1.62	1.77E-15	↓	tsukushin [B. mutus]
ENSBTAP00000041770-D8	1.62	1.93E-06	↓	Tubulin beta-4 chain [B. mutus]
ENSBTAP00000016693-D1	1.62	2.16E-25	↓	Macrophage erythroblast attacher, partial [B. mutus]
ENSBTAP00000012334-D1	1.62	1.50E-13	↓	Coiled-coil domain-containing protein 36, partial [B. mutus]
ENSBTAP00000042643-D1	1.62	5.69E-15	↓	inositol-tetrakisphosphate 1-kinase [B. mutus]
ENSBTAP00000010451-D1	1.62	2.81E-215	↓	major vault protein [B. mutus]
ENSP00000378364-D3	1.62	2.66E-26	↓	Natural resistance-associated macrophage protein 2 [B. mutus]
ENSBTAP00000042804-D1	1.62	1.02E-14	↓	Acetylcholine receptor subunit epsilon [B. mutus]
ENSP00000404328-D10	1.62	4.88E-09	↓	hypothetical protein M91_18968 [B. mutus]
ENSBTAP00000005055-D1	1.62	3.47E-06	↓	syntaxin-16 isoform X3 [B. taurus]
ENSP00000414232-D1	1.62	9.50E-05	↓	gamma-aminobutyric acid receptor subunit alpha-1 precursor [B. taurus]
ENSP00000284601-D1	1.62	2.76E-03	↓	protein phosphatase 1 regulatory subunit 3A-like [B. mutus]
ENSBTAP00000020081-D1	1.62	2.14E-138	↓	Small glutamine-rich tetratricopeptide repeat-containing protein alpha [B. mutus]
ENSBTAP00000020325-D1	1.62	8.85E-68	↓	outer dense fiber protein 2 isoform X1 [B. mutus]
ENSP00000416951-D1	1.62	8.64E-34	↓	zinc finger CCCH domain-containing protein 18 isoform X2 [B. taurus]
ENSP00000365637-D1	1.62	8.75E-09	↓	Sickle tail protein-like protein, partial [B. mutus]
ENSP00000385899-D1	1.62	8.75E-09	↓	Protein sidekick-1, partial [B. mutus]
ENSBTAP00000026432-D1	1.62	3.26E-18	↓	SHC-transforming protein 1 isoform X3 [B. taurus]
ENSP00000268835-D1	1.62	2.31E-07	↓	phosphoribosyl pyrophosphate synthase-associated protein 2 [B. taurus]
ENSP00000341988-D1	1.63	6.24E-06	↓	Protein FAM75D1 [B. mutus]
ENSP00000290866-D1	1.63	6.24E-06	↓	Angiotensin-converting enzyme, partial [B. mutus]
ENSP00000356297-D1	1.63	7.97E-307	↓	Pleckstrin-like protein domain-containing family G member 1 [B. mutus]
ENSBTAP00000043290-D1	1.63	3.99E-15	↓	DCN1-like protein 3 [B. taurus]
ENSP00000360821-D1	1.63	4.06E-11	↓	Ubiquitin-associated domain-containing protein 1, partial [B. mutus]
ENSP00000356240-D1	1.63	1.07E-09	↓	Protein phosphatase 1 regulatory subunit 12B, partial [B. mutus]
ENSP00000404464-D1	1.63	1.72E-04	↓	TPA: KIAA1983 protein-like [B. taurus]
ENSBTAP00000004079-D1	1.63	2.34E-26	↓	Creatine kinase B-type, partial [B. mutus]
ENSP00000363794-D1	1.63	1.28E-18	↓	Estradiol 17-beta-dehydrogenase 8, partial [B. mutus]
ENSP00000262367-D1	1.63	2.03E-134	↓	CREB-binding protein [B. mutus]
ENSP00000349828-D1	1.63	2.80E-08	↓	Transcriptional enhancer factor TEF-5, partial [B. mutus]
ENSP00000219638-D1	1.63	3.61E-114	↓	Gene product with similarity to Ubiquitin binding enzyme [Homo sapiens]
ENSBTAP00000009715-D1	1.63	1.12E-05	↓	Synaptic vesicle membrane protein VAT-1-like protein, partial [B. mutus]
ENSP00000361725-D1	1.63	1.30E-10	↓	endonuclease G [B. mutus]
ENSBTAP00000053746-D1	1.63	2.70E-123	↓	Tescalcin, partial [B. mutus]
ENSBTAP00000052857-D1	1.63	3.31E-120	↓	zinc finger protein 616-like isoform X1 [Bison bison bison]
ENSBTAP00000014910-D1	1.63	5.02E-08	↓	Anion exchange protein 2, partial [B. mutus]
ENSBTAP00000051403-D14	1.63	5.02E-08	↓	uncharacterized protein LOC100076456 [Ornithorhynchus anatinus]
ENSP00000389000-D1	1.63	4.00E-49	↓	Transcription initiation factor TFIID subunit 1, partial [B. mutus]
ENSP00000388725-D1	1.64	0.00E+00	↓	ankycorbin isoform X1 [B. mutus]
ENSBTAP00000021414-D1	1.64	9.02E-08	↓	Ectoderm-neural cortex protein 2, partial [B. mutus]
ENSP00000280057-D1	1.64	2.02E-05	↓	Protein FAM124A, partial [B. mutus]
ENSBTAP00000046530-D1	1.64	3.12E-04	↓	Sharpin, partial [B. mutus]
ENSBTAP00000021041-D1	1.64	3.12E-04	↓	uncharacterized protein LOC102395856 isoform X3 [Bubalus bubalis]
ENSP00000371175-D1	1.64	3.12E-04	↓	ADP-ribosylation factor-binding protein GGA1, partial [B. mutus]

ENSP00000306138-D1	1.64	3.12E-04	↓	metabotropic glutamate receptor 5 isoform X2 [Ovis aries musimon]
ENSBTAP00000041785-D1	1.64	5.08E-03	↓	frizzled-5 [B. mutus]
ENSBTAP00000052415-D1	1.64	5.08E-03	↓	hypothetical protein M91_00451, partial [B. mutus]
ENSP00000261797-D2	1.64	5.08E-03	↓	Bifunctional heparan sulfate N-deacetylase/N-sulfotransferase 1, partial [B. mutus]
ENSBTAP00000000579-D1	1.64	5.08E-03	↓	CREB/ATF bZIP transcription factor, partial [B. mutus]
ENSBTAP00000043534-D3	1.64	5.08E-03	↓	C1q-related factor-like protein [Cricetulus griseus]
ENSBTAP00000016529-D1	1.64	5.08E-03	↓	HEPACAM family member 2 isoform X1 [B. mutus]
ENSP00000364536-D3	1.64	5.20E-96	↓	Sodium channel protein type 8 subunit alpha, partial [B. mutus]
ENSP00000315664-D1	1.64	1.64E-21	↓	Zinc finger protein 18 [B. mutus]
ENSP00000302640-D1	1.64	2.26E-39	↓	nucleolar transcription factor 1 isoform X1 [B. mutus]
ENSBTAP00000006262-D1	1.64	1.25E-35	↓	endonuclease 8-like 2 [B. mutus]
ENSP00000266880-D1	1.64	1.95E-15	↓	E3 ubiquitin-protein ligase CHFR isoform X1 [B. mutus]
ENSP00000262188-D1	1.64	9.84E-28	↓	SWI/SNF-related matrix-associated actin-dependent regulator of chromatin subfamily D member 1, partial [B. mutus]
ENSBTAP00000021265-D1	1.64	1.62E-07	↓	nuclear distribution protein nudE homolog 1 [B. taurus]
ENSP00000296318-D1	1.64	9.10E-11	↓	Interleukin-17 receptor D [B. mutus]
ENSP00000341422-D1	1.64	2.42E-06	↓	transmembrane prolyl 4-hydroxylase isoform X2 [Rhinopithecus roxellana]
ENSP00000384479-D1	1.64	2.65E-29	↓	tetratricopeptide repeat protein 18 [B. mutus]
ENSBTAP00000025143-D1	1.65	1.11E-11	↓	diphthamide biosynthesis protein 2 [B. mutus]
ENSBTAP00000018617-D1	1.65	1.40E-43	↓	UbiA prenyltransferase domain-containing protein 1, partial [B. mutus]
ENSBTAP00000008028-D1	1.65	7.63E-16	↓	exocyst complex component 8 [B. taurus]
ENSBTAP00000014279-D1	1.65	2.20E-27	↓	Protein phosphatase 1E, partial [B. mutus]
ENSBTAP00000020185-D1	1.65	3.38E-81	↓	Lactase-phlorizin hydrolase [B. mutus]
ENSBTAP00000014839-D1	1.65	3.50E-08	↓	Protein FAM189A2, partial [B. mutus]
ENSP00000296444-D1	1.65	5.69E-04	↓	Protein shisa-5, partial [B. mutus]
ENSP00000220509-D1	1.65	5.69E-04	↓	vacuolar protein sorting-associated protein 18 homolog [B. taurus]
ENSBTAP00000023194-D1	1.65	2.72E-129	↓	calcipressin-3 isoform X1 [B. mutus]
ENSBTAP00000026003-D1	1.65	5.34E-137	↓	Protein phosphatase 1G [B. mutus]
ENSBTAP00000027098-D1	1.65	1.68E-80	↓	Ectonucleoside triphosphate diphosphohydrolase 5 [B. mutus]
ENSP00000265094-D1	1.65	1.70E-56	↓	KIAA0696 protein [Homo sapiens]
ENSP00000409445-D1	1.65	4.24E-32	↓	integrator complex subunit 3 [Orcinus orca]
ENSBTAP00000001294-D1	1.65	4.63E-133	↓	Set1/Ash2 histone methyltransferase complex subunit ASH2 [B. mutus]
ENSBTAP00000042286-D1	1.65	7.45E-49	↓	mesoderm-specific transcript homolog protein isoform X1 [Capra hircus]
ENSP00000376127-D1	1.65	6.54E-17	↓	Cohesin loading complex subunit SCC4-like protein, partial [B. mutus]
ENSBTAP00000015764-D1	1.65	6.41E-14	↓	chromatin assembly factor 1 subunit B [B. mutus]
ENSP00000260147-D1	1.66	5.43E-75	↓	protocadherin Fat 1 [B. mutus]
ENSBTAP00000000301-D1	1.66	1.27E-204	↓	Double-stranded RNA-binding protein Staufen-like protein 1, partial [B. mutus]
ENSP00000314396-D1	1.66	2.14E-19	↓	putative protein arginine N-methyltransferase 10 isoform X1 [B. mutus]
ENSBTAP00000048203-D1	1.66	4.02E-130	↓	selenophosphate synthetase 2 [B. mutus]
ENSP00000379769-D1	1.66	4.15E-24	↓	Aconitate hydratase, mitochondrial, partial [B. mutus]
ENSBTAP00000001376-D1	1.66	7.71E-58	↓	splicing factor 1 isoform X1 [Bison bison bison]
ENSP00000258711-D1	1.67	2.45E-08	↓	carbohydrate sulfotransferase 12 [B. mutus]
ENSP00000366035-D3	1.67	1.41E-05	↓	Alpha-actinin-2, partial [B. mutus]
ENSBTAP00000047510-D1	1.67	1.04E-03	↓	transmembrane protein 245 [Elephantulus edwardii]
ENSBTAP00000045122-D1	1.67	1.04E-03	↓	tissue factor pathway inhibitor [B. mutus]
ENSP00000162391-D1	1.67	9.52E-03	↓	Forkhead box protein J2 [B. mutus]
ENSP00000416776-D1	1.67	9.52E-03	↓	Cysteine/serine-rich nuclear protein 1 [B. mutus]
ENSBTAP00000017670-D1	1.67	9.52E-03	↓	smoothened homolog precursor [B. taurus]
ENSBTAP00000008660-D1	1.67	9.52E-03	↓	ankyrin repeat and SAM domain-containing protein 3 [B. mutus]
ENSBTAP00000012605-D1	1.67	2.89E-24	↓	Focal adhesion kinase 1, partial [B. mutus]
ENSBTAP00000004744-D1	1.67	1.82E-95	↓	cyclin-J isoform X1 [B. mutus]
ENSBTAP00000043848-D1	1.67	9.62E-12	↓	syntaxin-5 [B. taurus]
ENSBTAP00000021048-D1	1.67	7.92E-11	↓	Protein FAM168B, partial [B. mutus]
ENSBTAP00000013169-D1	1.67	1.80E-247	↓	Sodium/potassium-transporting ATPase subunit alpha-1, partial [B. mutus]
ENSBTAP00000016395-D1	1.67	4.20E-23	↓	E3 ubiquitin-protein ligase RNF220, partial [B. mutus]

ENSBTAP00000009087-D1	1.67	3.12E-29	↓	ATP-binding cassette sub-family F member 3, partial [B. mutus]
ENSBTAP00000017963-D1	1.67	2.65E-178	↓	Torsin-2A, partial [B. mutus]
ENSP00000416015-D1	1.67	6.28E-52	↓	protein-methionine sulfoxide oxidase MICAL3 [B. mutus]
ENSP00000356319-D1	1.67	1.98E-81	↓	Kinesin-like protein KIF14, partial [B. mutus]
ENSBTAP00000013635-D1	1.67	1.52E-18	↓	GTP-binding protein 1, partial [B. mutus]
ENSP00000368686-D1	1.67	3.61E-07	↓	Transcription factor E2F4 [B. mutus]
ENSP00000378624-D2	1.67	8.51E-269	↓	probable E3 ubiquitin-protein ligase HERC3 [B. mutus]
ENSP00000328187-D1	1.67	1.34E-22	↓	F-box only protein 21 isoform X3 [Ovis aries musimon]
yakG029112	1.67	2.22E-17	↓	hypothetical protein M91_15025 [B. mutus]
ENSBTAP00000015199-D1	1.68	4.54E-97	↓	puromycin-sensitive aminopeptidase [Bison bison bison]
ENSBTAP00000005065-D1	1.68	3.73E-12	↓	Constitutive coactivator of peroxisome proliferator-activated receptor gamma, partial [B. mutus]
ENSBTAP00000011820-D2	1.68	9.53E-09	↓	Ubiquitin carboxyl-terminal hydrolase 49, partial [B. mutus]
ENSBTAP00000017150-D1	1.68	2.52E-10	↓	ADP-ribosylation factor-like protein 8B [Homo sapiens]
ENSBTAP00000028655-D1	1.68	8.15E-13	↓	Proactivator polypeptide [B. mutus]
ENSBTAP00000018773-D1	1.68	2.17E-04	↓	putative E3 ubiquitin-protein ligase UNKL isoform X1 [B. taurus]
ENSP00000369129-D1	1.68	1.78E-13	↓	Desmoplakin, partial [B. mutus]
ENSBTAP00000023702-D1	1.68	0.00E+00	↓	plasma membrane calcium-transporting ATPase 2 isoform 2 [Cricetulus griseus]
ENSBTAP00000028806-D1	1.68	5.43E-06	↓	Zinc finger CCCH-type antiviral protein 1 [B. mutus]
ENSBTAP00000010247-D1	1.68	7.96E-24	↓	GRIP1-associated protein 1 [B. mutus]
ENSP00000264276-D1	1.68	1.04E-40	↓	Alsin, partial [B. mutus]
ENSP00000331845-D1	1.68	1.47E-257	↓	protein PRR14L [B. mutus]
ENSP00000252996-D1	1.69	3.76E-47	↓	Transcription initiation factor TFIID subunit 4, partial [B. mutus]
ENSP00000356331-D1	1.69	1.31E-18	↓	Nuclear receptor subfamily 5 group A member 2 [B. mutus]
ENSBTAP00000001347-D1	1.69	4.57E-05	↓	Mitogen-activated protein kinase 7 [B. mutus]
ENSP00000357292-D1	1.69	4.57E-05	↓	ubiquilin-4 [B. mutus]
ENSBTAP00000028056-D1	1.69	1.89E-03	↓	Suppressor of fused-like protein [B. mutus]
ENSBTAP00000019482-D1	1.69	1.89E-03	↓	ATP-binding cassette sub-family D member 4 [B. mutus]
yakA09460	1.69	1.89E-03	↓	hypothetical protein M91_00902 [B. mutus]
ENSBTAP00000040060-D1	1.69	7.08E-27	↓	Adiponectin receptor protein 1, partial [B. mutus]
ENSBTAP00000041074-D1	1.69	4.64E-12	↓	probable ATP-dependent RNA helicase DDX41 [Ovis aries]
ENSP00000367991-D1	1.69	1.11E-30	↓	Septin-8, partial [B. mutus]
ENSP00000297761-D1	1.69	1.75E-10	↓	protein LCHN [Bubalus bubalis]
ENSBTAP00000005228-D1	1.69	6.63E-09	↓	protein FAM199X [B. taurus]
ENSP00000329419-D1	1.69	0.00E+00	↓	Coatomer subunit beta', partial [B. mutus]
ENSP00000361158-D1	1.69	3.83E-11	↓	dual specificity testis-specific protein kinase 2 isoform X4 [B. taurus]
ENSBTAP00000002169-D1	1.69	1.67E-42	↓	solute carrier organic anion transporter family member 3A1 [B. taurus]
ENSP00000347672-D1	1.69	4.91E-27	↓	vang-like protein 1 [Capra hircus]
ENSBTAP00000022801-D1	1.69	7.45E-18	↓	E3 ubiquitin-protein ligase TRIM32 [B. mutus]
ENSP00000297450-D1	1.69	5.43E-08	↓	Angiopoietin-1, partial [B. mutus]
ENSP00000264245-D1	1.69	5.43E-08	↓	rho GTPase-activating protein 31 [B. mutus]
ENSP00000295148-D1	1.70	9.45E-42	↓	WD repeat-containing protein C2orf44 homolog [B. mutus]
ENSP00000380038-D1	1.70	4.49E-285	↓	DNA replication licensing factor MCM4, partial [B. mutus]
ENSBTAP00000025389-D1	1.70	2.10E-06	↓	peripheral myelin protein 22 [B. taurus]
ENSBTAP00000013207-D1	1.70	3.93E-04	↓	DnaJ-like protein subfamily A member 3, mitochondrial, partial [B. mutus]
ENSP00000297596-D1	1.70	3.93E-04	↓	GTP-binding protein GEM isoform X2 [Bison bison bison]
ENSP00000269197-D1	1.70	6.84E-34	↓	Putative Polycomb group protein ASXL3, partial [B. mutus]
ENSBTAP00000015158-D1	1.70	2.56E-32	↓	Centrosomal protein of 72 kDa, partial [B. mutus]
ENSP00000301738-D1	1.70	1.48E-11	↓	BTB/POZ domain-containing protein KCTD5, partial [B. mutus]
ENSBTAP00000016778-D1	1.70	2.57E-09	↓	uroporphyrinogen decarboxylase [B. taurus]
ENSBTAP00000022068-D1	1.70	2.57E-09	↓	rab11 family-interacting protein 3 isoform X5 [B. taurus]
ENSP00000337332-D1	1.70	4.49E-07	↓	NAD-dependent protein deacetylase sirtuin-6 isoform X1 [B. mutus]
ENSP00000316032-D1	1.70	4.48E-153	↓	nuclear pore complex protein Nup98-Nup96 isoform X2 [B. taurus]
ENSBTAP00000053642-D1	1.70	4.41E-50	↓	RING finger protein 31, partial [B. mutus]
ENSBTAP00000012949-D1	1.70	7.78E-40	↓	Serine/threonine-protein phosphatase 4 regulatory subunit 1, partial [B. mutus]
ENSBTAP00000053705-D1	1.70	1.22E-10	↓	Protein Daple, partial [B. mutus]

ENSBTAP00000016063-D1	1.70	8.25E-05	↓	mitoferrin-2 [B. taurus]
ENSP00000250863-D2	1.70	6.90E-23	↓	Deleted in azoospermia-like protein, partial [B. mutus]
ENSBTAP00000005581-D1	1.70	6.62E-41	↓	Elongation factor 2, partial [B. mutus]
ENSP00000250237-D1	1.70	5.37E-20	↓	queuine tRNA-ribosyltransferase [B. mutus]
ENSBTAP00000012486-D1	1.70	6.70E-51	↓	Nuclear RNA export factor 1 [B. mutus]
ENSBTAP00000033176-D1	1.70	7.91E-161	↓	type II inositol 1,4,5-trisphosphate 5-phosphatase isoform X7 [Ovis aries musimon]
ENSBTAP00000018338-D1	1.70	2.55E-21	↓	phosphoribosyl pyrophosphate synthase-associated protein 1 [B. taurus]
ENSBTAP00000002936-D1	1.70	2.11E-08	↓	Protein ENL, partial [B. mutus]
ENSP00000352608-D2	1.70	7.23E-25	↓	ryanodine receptor 3 [B. mutus]
ENSBTAP00000042855-D1	1.70	5.61E-22	↓	N-terminal kinase-like protein isoform X1 [B. mutus]
ENSBTAP00000004035-D1	1.71	4.70E-105	↓	Sal-like protein 4, partial [B. mutus]
ENSBTAP00000002549-D1	1.71	1.18E-69	↓	28S ribosomal protein S27, mitochondrial isoform X1 [Bison bison bison]
ENSP00000356174-D1	1.71	3.58E-18	↓	Transcription factor SOX-13, partial [B. mutus]
ENSBTAP00000039142-D1	1.71	3.75E-06	↓	uncharacterized protein C20orf196 homolog [B. mutus]
ENSBTAP00000013208-D1	1.71	8.90E-35	↓	nmrA-like family domain-containing protein 1 [B. mutus]
ENSBTAP00000032471-D1	1.71	9.44E-152	↓	Embryonic polyadenylate-binding protein 2, partial [B. mutus]
ENSBTAP00000019620-D1	1.71	1.39E-18	↓	Thioredoxin domain-containing protein 15, partial [B. mutus]
ENSBTAP00000038059-D1	1.71	6.45E-66	↓	serine hydroxymethyltransferase, mitochondrial precursor [B. taurus]
ENSBTAP00000043763-D1	1.71	7.91E-76	↓	Pyridoxal-dependent decarboxylase domain-containing protein 1 [B. mutus]
ENSBTAP00000015985-D1	1.71	5.71E-69	↓	Histone-arginine methyltransferase CARM1, partial [B. mutus]
ENSBTAP00000033611-D1	1.71	3.87E-10	↓	interleukin-1 receptor-associated kinase 3 [Bison bison bison]
ENSP00000320309-D1	1.71	5.62E-21	↓	phenylalanine--tRNA ligase alpha subunit isoform X1 [B. mutus]
ENSP00000328998-D1	1.71	1.83E-11	↓	trafficking kinesin-binding protein 1 isoform X1 [B. mutus]
ENSP00000319104-D1	1.72	3.08E-28	↓	Transcription elongation factor SPT6 [B. mutus]
ENSP00000396052-D1	1.72	1.94E-15	↓	Breast cancer metastasis-suppressor 1-like protein, partial [B. mutus]
ENSBTAP00000002665-D1	1.72	4.59E-20	↓	DNA replication licensing factor MCM9, partial [B. mutus]
ENSP00000400010-D1	1.72	2.17E-96	↓	receptor-type tyrosine-protein phosphatase eta [B. mutus]
ENSBTAP00000005074-D1	1.72	6.50E-45	↓	EMILIN-2 protein, partial [B. mutus]
ENSBTAP00000005218-D1	1.72	3.61E-17	↓	hydrocephalus-inducing protein homolog isoform X1 [B. taurus]
ENSBTAP00000001076-D1	1.72	1.66E-16	↓	Homer protein-like protein 3, partial [B. mutus]
ENSP00000299192-D1	1.72	3.47E-15	↓	HEAT repeat-containing protein 3 [B. taurus]
ENSBTAP00000001080-D1	1.72	3.10E-07	↓	ephrin type-A receptor 2 [B. mutus]
ENSP00000350718-D1	1.72	1.48E-04	↓	Transmembrane and coiled-coil domains protein 2 [B. mutus]
ENSBTAP00000000944-D1	1.72	7.08E-04	↓	A disintegrin and metalloproteinase with thrombospondin motifs 1 [B. mutus]
ENSP00000335261-D1	1.72	7.08E-04	↓	trimethyllysine dioxygenase, mitochondrial [B. taurus]
ENSP00000280758-D1	1.72	3.46E-03	↓	Ankyrin repeat and BTB/POZ domain-containing protein BTBD11, partial [B. mutus]
ENSBTAP00000006646-D1	1.72	3.46E-03	↓	Serine/threonine-protein kinase A-Raf [B. mutus]
ENSBTAP00000008240-D1	1.72	3.46E-03	↓	Fox-1-like protein C, partial [B. mutus]
ENSBTAP00000016933-D1	1.72	1.78E-02	↓	Zinc finger protein 827, partial [B. mutus]
ENSBTAP00000022073-D1	1.72	1.78E-02	↓	phosphatidylinositol N-acetylglucosaminyltransferase subunit Q [B. mutus]
ENSBTAP00000000141-D1	1.72	1.78E-02	↓	Tumor necrosis factor ligand superfamily member 13, partial [B. mutus]
ENSP00000389813-D2	1.72	1.78E-02	↓	Acyl-CoA dehydrogenase family member 10 [B. mutus]
ENSBTAP00000020013-D1	1.72	1.78E-02	↓	GRB2-associated and regulator of MAPK protein-like isoform X1 [B. taurus]
ENSBTAP00000026369-D1	1.72	1.78E-02	↓	monocarboxylate transporter 9 [B. mutus]
ENSBTAP00000048631-D1	1.72	6.96E-165	↓	protein Tob1 [B. taurus]
ENSBTAP00000009691-D1	1.72	1.67E-80	↓	Eukaryotic translation initiation factor 2-alpha kinase 1, partial [B. mutus]
ENSBTAP00000002420-D1	1.72	6.03E-68	↓	DNA-directed RNA polymerase II subunit RPB3 [Tupaia chinensis]
ENSBTAP00000008644-D1	1.72	3.08E-27	↓	prolyl 4-hydroxylase subunit alpha-3 precursor [B. taurus]
ENSP00000262525-D1	1.73	2.68E-10	↓	zinc finger protein 629 [B. mutus]
ENSP00000391200-D1	1.73	1.23E-09	↓	Zinc finger protein 692, partial [B. mutus]
ENSP00000271657-D1	1.73	9.45E-43	↓	Phosphatidylinositol 4-kinase beta, partial [B. mutus]
ENSP00000398105-D1	1.73	2.30E-22	↓	Cation transport regulator-like protein 1, partial [B. mutus]
ENSP00000317442-D1	1.73	9.75E-36	↓	kelch-like protein 36 [B. taurus]
ENSP00000307863-D1	1.73	4.91E-83	↓	Splicing factor U2AF 65 kDa subunit, partial [B. mutus]
ENSP00000307541-D1	1.73	1.20E-07	↓	BCL-6 corepressor-like protein 1 [B. mutus]

ENSBTAP00000038062-D4	1.73	1.20E-07	↓	NADH dehydrogenase [ubiquinone] 1 alpha subcomplex subunit 4-like 2 [B. mutus]
ENSP00000402109-D1	1.73	1.07E-12	↓	Protein GPR107, partial [B. mutus]
ENSP00000388996-D1	1.73	4.90E-12	↓	AP-1 complex subunit mu-1 [Capra hircus]
ENSBTAP00000010937-D1	1.73	2.75E-91	↓	1-phosphatidylinositol 4,5-bisphosphate phosphodiesterase eta-1 [B. mutus]
ENSBTAP00000015136-D1	1.73	9.58E-106	↓	stomatin-like protein 2, mitochondrial [Bison bison bison]
ENSP00000387266-D1	1.73	1.62E-75	↓	Protein spire-like protein 1, partial [B. mutus]
ENSBTAP00000022158-D1	1.73	1.74E-26	↓	Carbamoyl-phosphate synthase [ammonia], mitochondrial, partial [B. mutus]
ENSP00000370215-D1	1.73	7.93E-26	↓	hypothetical protein M91_01973 [B. mutus]
ENSP00000305918-D1	1.73	2.36E-48	↓	bromodomain-containing protein 3 isoform X2 [Balaenoptera acutorostrata scammoni]
ENSBTAP00000017907-D1	1.73	4.39E-50	↓	centrosomal protein of 97 kDa [B. mutus]
ENSP00000343890-D1	1.73	2.58E-06	↓	ceramide-1-phosphate transfer protein [B. mutus]
ENSP00000361219-D1	1.73	1.21E-05	↓	general transcription factor 3C polypeptide 4 [B. mutus]
ENSBTAP00000024807-D1	1.73	1.35E-23	↓	E3 ubiquitin-protein ligase PDZRN3, partial [B. mutus]
ENSBTAP00000024887-D1	1.74	1.70E-251	↓	Histone acetyltransferase MYST2 [B. mutus]
ENSBTAP00000007621-D1	1.74	3.04E-58	↓	DDB1- and CUL4-associated factor 8 [B. taurus]
ENSBTAP00000036650-D1	1.74	1.82E-87	↓	cathepsin B isoform X1 [B. mutus]
ENSP00000344456-D1	1.74	5.20E-258	↓	Catenin beta-1, partial [B. mutus]
ENSP00000371201-D1	1.74	1.49E-43	↓	putative methyltransferase NSUN7 [B. mutus]
ENSBTAP00000027058-D1	1.74	1.67E-40	↓	Mitochondrial GTPase 1 [B. mutus]
ENSP00000363458-D1	1.74	6.73E-26	↓	Low density lipoprotein receptor adapter protein 1, partial [B. mutus]
ENSBTAP00000002247-D1	1.74	1.56E-11	↓	major facilitator superfamily domain-containing protein 9 [B. mutus]
ENSBTAP00000027092-D1	1.74	3.46E-22	↓	Protein FAM161B [B. mutus]
ENSP00000241416-D2	1.74	7.17E-11	↓	Activin receptor type-2B [Tupaia chinensis]
ENSP00000252015-D1	1.74	1.57E-21	↓	Short transient receptor putative channel 4-associated protein, partial [B. mutus]
ENSBTAP00000009750-D1	1.74	1.80E-08	↓	protein arginine N-methyltransferase 6 [Bubalus bubalis]
ENSP00000267460-D1	1.75	9.71E-17	↓	E3 ubiquitin-protein ligase pellino homolog 2 [Pantholops hodgsonii]
ENSP00000381049-D1	1.75	3.09E-33	↓	neuron-specific protein family member 1 [B. taurus]
ENSP00000412798-D1	1.75	4.63E-06	↓	Kinesin-like protein KIF18B [B. mutus]
ENSP00000355385-D1	1.75	4.63E-06	↓	hypothetical protein M91_15535, partial [B. mutus]
ENSP00000335193-D1	1.75	5.79E-76	↓	vam6/Vps39-like protein isoform X1 [B. mutus]
ENSBTAP00000014000-D1	1.75	8.27E-08	↓	protein ZNF365-like [B. mutus]
ENSBTAP00000038002-D1	1.75	2.03E-15	↓	diacylglycerol kinase alpha isoform X1 [Bubalus bubalis]
ENSBTAP00000028266-D1	1.75	0.00E+00	↓	F-box/LRR-repeat protein 5 isoform X2 [Bison bison bison]
ENSBTAP00000024505-D1	1.75	1.60E-37	↓	Proteasome inhibitor PI31 subunit, partial [B. mutus]
ENSP00000369461-D1	1.75	4.74E-211	↓	Peroxisomal 3,2-trans-enoyl-CoA isomerase, partial [B. mutus]
ENSBTAP00000005252-D1	1.75	1.80E-34	↓	cyclin-dependent kinase 2 [B. taurus]
ENSP00000262414-D1	1.75	1.55E-51	↓	116 kDa U5 small nuclear ribonucleoprotein component [Dipodomys ordii]
ENSP00000418232-D1	1.75	3.34E-84	↓	Nischarin, partial [B. mutus]
ENSP00000377725-D1	1.76	1.03E-49	↓	zinc finger protein with KRAB and SCAN domains 5 [B. mutus]
yakG021436	1.76	3.50E-13	↓	Zinc finger protein 142 [B. mutus]
ENSP00000370713-D2	1.76	1.02E-04	↓	Double-stranded RNA-specific editase 1, partial [B. mutus]
ENSP00000280979-D1	1.76	5.83E-107	↓	A-kinase anchor protein 6 [B. mutus]
ENSBTAP00000041598-D1	1.76	1.45E-104	↓	dual specificity mitogen-activated protein kinase kinase 4 [Ursus maritimus]
ENSP00000217964-D2	1.76	1.72E-31	↓	F-box-like/WD repeat-containing protein TBL1X, partial [B. mutus]
ENSBTAP00000014357-D1	1.76	1.24E-08	↓	PI-PLC X domain-containing protein 3, partial [B. mutus]
ENSP00000292180-D1	1.76	1.15E-53	↓	FAD synthase, partial [B. mutus]
ENSBTAP00000013256-D1	1.76	5.45E-200	↓	T-lymphoma invasion and metastasis-inducing protein 2, partial [B. mutus]
ENSBTAP00000003061-D1	1.76	8.26E-06	↓	Tubby-related protein 2, partial [B. mutus]
ENSP00000264144-D1	1.76	8.26E-06	↓	Laminin subunit gamma-2, partial [B. mutus]
ENSP00000222382-D3	1.76	7.26E-43	↓	cytochrome P450 3A24-like [B. mutus]
ENSBTAP00000009093-D1	1.76	1.14E-14	↓	Kelch domain-containing protein 4, partial [B. mutus]
ENSP00000345268-D2	1.76	7.37E-12	↓	Palmitoyltransferase ZDHHC3 [B. mutus]
ENSBTAP00000024626-D1	1.77	5.67E-08	↓	little elongation complex subunit 1 [Bison bison bison]
ENSBTAP00000041750-D1	1.77	1.63E-260	↓	Cyclin-I2 [B. mutus]
ENSP00000359991-D1	1.77	9.03E-75	↓	phosphoglycerate mutase 1 [B. taurus]

ENSP00000330965-D1	1.77	2.62E-07	↓	uncharacterized protein LOC101125104 isoform 1 [Gorilla gorilla gorilla]
ENSBTAP00000022108-D1	1.77	3.17E-06	↓	Dual serine/threonine and tyrosine protein kinase [B. mutus]
ENSP00000309338-D1	1.77	4.85E-04	↓	Cardiotrophin-like cytokine factor 1, partial [B. mutus]
ENSP00000262891-D1	1.77	4.85E-04	↓	MAP/microtubule affinity-regulating kinase 4, partial [B. mutus]
ENSBTAP00000047305-D1	1.77	4.85E-04	↓	hypothetical protein M91_11706, partial [B. mutus]
ENSP00000367394-D1	1.77	4.85E-04	↓	rho guanine nucleotide exchange factor 1 isoform X6 [B. taurus]
ENSBTAP00000049968-D1	1.77	6.35E-03	↓	A disintegrin and metalloproteinase with thrombospondin motifs 4 [B. mutus]
ENSBTAP00000020290-D1	1.77	6.35E-03	↓	alpha-N-acetylgalactosaminide alpha-2,6-sialyltransferase 2 isoform X5 [B. taurus]
ENSP00000365630-D1	1.77	6.35E-03	↓	Ras and EF-hand domain-containing protein, partial [B. mutus]
ENSBTAP00000008613-D1	1.77	6.35E-03	↓	vang-like protein 2 [B. mutus]
ENSP00000300504-D1	1.77	6.35E-03	↓	TBC1 domain family member 21 isoform X3 [B. taurus]
yakG025165	1.77	6.35E-03	↓	General transcription factor IIF subunit 1 [B. mutus]
ENSBTAP00000009659-D1	1.77	6.35E-03	↓	FERM domain-containing protein 7, partial [B. mutus]
ENSBTAP00000009267-D1	1.77	6.35E-03	↓	Frizzled-10, partial [B. mutus]
ENSBTAP00000018145-D1	1.77	0.00E+00	↓	transcription initiation factor IIA subunit 1 [Condylura cristata]
ENSP00000310042-D1	1.77	1.27E-104	↓	zinc finger protein 622 [Bison bison bison]
ENSP00000226319-D1	1.77	1.66E-67	↓	Protein Jade-1 [B. mutus]
ENSBTAP00000007703-D1	1.77	4.96E-181	↓	cyclin-T1 [B. taurus]
ENSBTAP00000042343-D2	1.78	6.03E-11	↓	catenin alpha-2 [B. taurus]
ENSBTAP00000012731-D1	1.78	7.13E-19	↓	ribonuclease H2 subunit A [B. taurus]
ENSP00000361705-D1	1.78	7.14E-10	↓	deoxynucleotidyltransferase terminal-interacting protein 1 [B. taurus]
ENSP00000248142-D1	1.78	7.14E-10	↓	WD repeat-containing protein 24, partial [B. mutus]
ENSBTAP00000027374-D1	1.78	1.69E-22	↓	arginine decarboxylase isoform X1 [B. mutus]
ENSBTAP00000011327-D1	1.78	8.50E-09	↓	Rho guanine nucleotide exchange factor 10, partial [B. mutus]
ENSBTAP00000007904-D1	1.78	4.89E-34	↓	Nuclear factor NF-kappa-B p100 subunit [B. mutus]
ENSBTAP00000002523-D1	1.78	1.71E-192	↓	Selenocysteine insertion sequence-binding protein 2 [B. mutus]
ENSP00000356744-D1	1.78	3.18E-127	↓	Protein-associating with the carboxyl-terminal domain of ezrin [B. mutus]
ENSP00000393633-D1	1.78	4.55E-16	↓	H(+)/Cl(-) exchange transporter 5, partial [B. mutus]
ENSP00000364080-D2	1.78	1.22E-06	↓	hypothetical protein M91_05095 [B. mutus]
ENSP00000317614-D1	1.78	4.55E-126	↓	zinc finger protein 518B [B. mutus]
ENSP00000254654-D1	1.78	4.85E-41	↓	integrin-linked kinase-associated serine/threonine phosphatase 2C [B. mutus]
ENSBTAP00000018050-D1	1.78	3.96E-33	↓	Neutral amino acid transporter B(0), partial [B. mutus]
ENSP00000237536-D1	1.78	1.77E-16	↓	protein SOGA1 [B. mutus]
ENSBTAP00000035894-D1	1.78	1.48E-05	↓	G1/S-specific cyclin-D3, partial [B. mutus]
ENSP00000320445-D1	1.78	1.48E-05	↓	CUB and sushi domain-containing protein 1 [B. mutus]
ENSP00000339299-D1	1.78	9.77E-92	↓	triple functional domain protein isoform X2 [Ovis aries musimon]
ENSBTAP00000014920-D1	1.78	3.83E-24	↓	Forkhead box protein O3, partial [B. mutus]
ENSBTAP00000040846-D1	1.79	8.04E-16	↓	Zinc finger FYVE domain-containing protein 26 [B. mutus]
ENSBTAP00000040521-D1	1.79	1.96E-65	↓	Putative ATP-dependent RNA helicase DDX31, partial [B. mutus]
ENSBTAP00000027473-D1	1.79	4.45E-30	↓	diacylglycerol kinase delta [B. taurus]
ENSP00000351040-D1	1.79	1.83E-04	↓	protein FAM217B [B. mutus]
ENSP00000389716-D1	1.79	1.83E-04	↓	unnamed protein product [Homo sapiens]
ENSP00000385865-D3	1.79	1.34E-12	↓	Retinoic acid receptor beta, partial [B. mutus]
ENSP00000221265-D1	1.79	2.29E-79	↓	RNA polymerase II-associated factor 1 homolog [Fukomys damarensis]
ENSP00000320340-D1	1.79	5.83E-09	↓	diacylglycerol kinase zeta isoform X3 [Ovis aries musimon]
ENSBTAP00000028094-D1	1.79	1.89E-10	↓	Multidrug resistance-associated protein 1, partial [B. mutus]
ENSBTAP00000015344-D1	1.79	2.09E-30	↓	short transient receptor potential channel 2-like [B. mutus]
ENSBTAP00000008055-D1	1.80	1.35E-65	↓	serine/threonine-protein kinase PLK3 [B. mutus]
ENSBTAP00000001247-D1	1.80	3.64E-44	↓	Zinc finger protein 286A, partial [B. mutus]
ENSBTAP00000020327-D1	1.80	2.75E-211	↓	golgin subfamily A member 1 [B. mutus]
ENSBTAP00000000697-D1	1.80	6.94E-05	↓	zinc finger protein 395 [B. mutus]
ENSP00000279146-D1	1.80	6.94E-05	↓	AH receptor-interacting protein [B. taurus]
ENSP00000370849-D1	1.80	2.33E-03	↓	inositol 1,4,5-trisphosphate receptor-interacting protein-like 2 [B. taurus]
ENSBTAP00000006804-D1	1.80	2.33E-03	↓	syntaxin-19 [B. taurus]
ENSP00000399565-D1	1.80	2.33E-03	↓	Splicing factor, arginine/serine-rich 2 [Pteropus alecto]

ENSP00000326630-D1	1.80	2.33E-03	↓	zinc finger protein, FOG family member 1 [B. mutus]
ENSP00000289865-D1	1.80	3.74E-23	↓	ubiquitin carboxyl-terminal hydrolase 21 isoform X1 [B. mutus]
ENSBTAP00000053317-D1	1.80	4.39E-22	↓	hypothetical protein M91_04617, partial [B. mutus]
ENSP00000337518-D1	1.80	1.60E-56	↓	pre-rRNA processing protein FTSJ3 [B. mutus]
ENSBTAP00000023648-D1	1.80	8.31E-07	↓	testis-specific Y-encoded-like protein 2 [B. taurus]
ENSBTAP00000006928-D1	1.80	8.31E-07	↓	Kelch-like protein 2, partial [B. mutus]
ENSP00000261723-D1	1.80	0.00E+00	↓	cytoplasmic polyadenylation element-binding protein 1 isoform X4 [Bubalus bubalis]
ENSP00000361009-D1	1.80	3.35E-10	↓	phosphatidylinositol 3,4,5-trisphosphate-dependent Rac exchanger 1 protein [B. mutus]
ENSP00000261888-D1	1.81	2.64E-05	↓	mono [ADP-ribose] polymerase PARP16 [B. mutus]
ENSP00000346300-D1	1.81	2.64E-05	↓	crk-like protein [B. mutus]
ENSBTAP00000014725-D1	1.81	2.64E-05	↓	fatty acyl-CoA reductase 2 [B. mutus]
yakG038520	1.81	5.93E-102	↓	calponin-3-like [B. mutus]
ENSBTAP00000022588-D1	1.81	7.91E-15	↓	Tyrosine-protein phosphatase non-receptor type 9, partial [B. mutus]
ENSBTAP00000018252-D1	1.81	1.00E-16	↓	Prominin-1, partial [B. mutus]
ENSP00000413520-D1	1.81	4.92E-19	↓	hypothetical protein M91_03306, partial [B. mutus]
ENSP00000364397-D1	1.81	2.43E-13	↓	uncharacterized protein KIAA0195 homolog [B. mutus]
ENSBTAP00000031858-D1	1.81	8.71E-04	↓	branched-chain-amino-acid aminotransferase, mitochondrial isoform X1 [B. mutus]
ENSBTAP00000011592-D1	1.81	8.71E-04	↓	XK-related protein 8 [B. mutus]
ENSP00000360060-D1	1.81	8.71E-04	↓	proto-oncogene FRAT1, partial [B. mutus]
ENSBTAP00000010671-D1	1.81	8.71E-04	↓	BTB/POZ domain-containing adapter for CUL3-mediated RhoA degradation protein 2 [B. mutus]
ENSBTAP00000003035-D1	1.81	1.18E-15	↓	ADP-ribosylation factor-binding protein GGA2, partial [B. mutus]
ENSP00000371634-D1	1.82	7.77E-44	↓	insulin-like growth factor 2 mRNA-binding protein 2 [B. taurus]
ENSBTAP00000018426-D1	1.82	2.24E-18	↓	protein phosphatase Slingshot homolog 3 isoform X1 [Bison bison bison]
ENSP00000323858-D1	1.82	2.24E-18	↓	ATP-dependent RNA helicase DDX54 isoform X1 [B. mutus]
ENSBTAP00000013741-D1	1.82	6.48E-67	↓	transmembrane protein 169 [B. mutus]
ENSBTAP00000011546-D1	1.82	6.85E-17	↓	protease-associated domain-containing protein 1 [Sus scrofa]
ENSBTAP00000015070-D1	1.82	1.11E-12	↓	kinetochore-associated protein NSL1 homolog [B. taurus]
ENSP00000368102-D1	1.82	1.64E-21	↓	coiled-coil domain-containing protein 3 isoform X1 [Ovis aries]
ENSBTAP00000037502-D1	1.82	1.82E-08	↓	Cation-independent mannose-6-phosphate receptor, partial [B. mutus]
ENSBTAP00000021770-D1	1.82	3.02E-37	↓	ATP-binding cassette sub-family G member 8, partial [B. mutus]
ENSP00000369666-D1	1.82	3.27E-04	↓	F-box/LRR-repeat protein 19, partial [B. mutus]
ENSBTAP00000026034-D1	1.82	3.27E-04	↓	microfibrillar-associated protein 3-like [B. mutus]
ENSP00000365899-D1	1.82	3.27E-04	↓	visual system homeobox 1 [B. mutus]
ENSBTAP00000040952-D1	1.82	7.11E-26	↓	ribosomal RNA processing protein 1 homolog A isoform X1 [B. mutus]
ENSBTAP00000039205-D2	1.82	6.70E-31	↓	sorting nexin 5 [B. mutus]
ENSBTAP00000017999-D1	1.83	5.65E-07	↓	DNA fragmentation factor subunit alpha [B. mutus]
ENSBTAP00000013522-D1	1.83	3.16E-39	↓	dehydrogenase/reductase SDR family member 1 [B. mutus]
ENSP00000252037-D1	1.83	9.93E-202	↓	peptidyl-prolyl cis-trans isomerase FKBP6 isoform X1 [B. mutus]
ENSBTAP00000015873-D1	1.83	1.58E-64	↓	Aspartate aminotransferase, cytoplasmic, partial [B. mutus]
ENSBTAP00000007042-D1	1.83	2.17E-07	↓	vacuolar protein sorting-associated protein 11 homolog isoform X1 [B. mutus]
ENSBTAP00000023918-D3	1.83	1.24E-04	↓	histone H2B type F-M-like [B. mutus]
ENSBTAP00000031650-D9	1.83	1.24E-04	↓	multidrug resistance-associated protein 4-like isoform X1 [B. taurus]
ENSBTAP00000028636-D1	1.83	1.24E-04	↓	tetraspanin-18 [B. mutus]
ENSP00000358147-D1	1.83	4.04E-19	↓	CDK2-associated and cullin domain-containing protein 1 [B. mutus]
ENSBTAP00000003078-D1	1.83	4.22E-28	↓	Putative palmitoyltransferase ZDHHC5 [B. mutus]
ENSP00000343785-D1	1.83	1.31E-72	↓	protein sprouty homolog 1 [B. taurus]
ENSP00000268053-D1	1.83	6.04E-11	↓	Cholesterol side-chain cleavage enzyme, mitochondrial [B. mutus]
ENSBTAP00000038069-D1	1.84	1.23E-17	↓	serine/threonine-protein kinase 36 [B. mutus]
ENSP00000316329-D1	1.84	4.70E-05	↓	stearoyl-CoA desaturase 5 [Pantholops hodgsonii]
ENSBTAP00000010823-D1	1.84	4.70E-05	↓	solute carrier family 23 member 1 isoform X1 [B. mutus]
ENSP00000354558-D1	1.84	1.26E-55	↓	serine/threonine-protein kinase mTOR [B. mutus]
ENSP00000308546-D1	1.84	2.55E-53	↓	7SK snRNA methylphosphate capping enzyme [Bubalus bubalis]
ENSBTAP00000006978-D1	1.84	1.35E-21	↓	spermidine synthase [B. taurus]
ENSP00000384996-D1	1.84	8.96E-12	↓	TBC1 domain family member 10A [B. mutus]
ENSP00000395465-D1	1.84	1.53E-242	↓	Nuclear receptor coactivator 4, partial [B. mutus]

ENSBTAP00000033789-D1	1.84	2.37E-50	↓	Pogo transposable element with ZNF domain [B. mutus]
ENSBTAP00000029470-D1	1.84	3.53E-51	↓	Coiled-coil domain-containing protein 117, partial [B. mutus]
ENSBTAP00000053216-D1	1.84	1.07E-19	↓	protein kinase C theta type isoform X2 [B. mutus]
ENSP00000349640-D1	1.84	3.75E-16	↓	microspherule protein 1 isoform X1 [Otolemur garnettii]
ENSBTAP00000024427-D1	1.84	4.31E-59	↓	transmembrane protein 109 precursor [B. taurus]
ENSP00000302777-D1	1.84	5.07E-28	↓	Tubulin beta-3 chain [B. mutus]
ENSP00000359001-D1	1.84	5.59E-32	↓	Host cell factor 1 [B. mutus]
ENSBTAP00000053756-D1	1.84	3.29E-146	↓	phosphodiesterase 3A, cGMP-inhibited [B. mutus]
ENSBTAP00000021730-D1	1.84	2.00E-13	↓	nucleoplasmin-3 isoform X1 [Bison bison bison]
ENSP00000221482-D1	1.84	5.38E-23	↓	Histone-lysine N-methyltransferase, H3 lysine-79 specific, partial [B. mutus]
ENSBTAP00000023660-D1	1.85	4.11E-11	↓	coiled-coil domain-containing protein 153 [B. mutus]
ENSP00000382863-D1	1.85	1.13E-52	↓	Chromodomain-helicase-DNA-binding protein 8 [B. mutus]
ENSBTAP00000013674-D1	1.85	3.83E-07	↓	Copper-transporting ATPase 2, partial [B. mutus]
ENSBTAP00000027683-D1	1.85	1.19E-95	↓	adenylate kinase 4, mitochondrial [B. mutus]
ENSP00000362979-D1	1.85	5.22E-14	↓	homeobox protein TGIF2 [B. mutus]
ENSBTAP00000001120-D1	1.85	5.67E-08	↓	Myosin-3 [B. mutus]
ENSBTAP00000010538-D1	1.85	7.79E-15	↓	Serine/threonine-protein phosphatase 5 [B. mutus]
ENSBTAP00000010961-D1	1.85	5.91E-35	↓	synaptosomal-associated protein 25 isoform a [Mus musculus]
ENSBTAP00000025111-D1	1.85	8.46E-09	↓	Eukaryotic translation initiation factor 4E type 3, partial [B. mutus]
ENSP00000362207-D1	1.86	3.26E-09	↓	carbohydrate sulfotransferase 3 [B. mutus]
ENSP00000327349-D1	1.86	1.04E-99	↓	transmembrane and coiled-coil domains protein 1 isoform X2 [B. taurus]
ENSP00000312066-D1	1.86	9.15E-61	↓	signal recognition particle subunit SRP68 [B. taurus]
ENSBTAP00000020316-D1	1.86	6.89E-42	↓	casein kinase I isoform epsilon-like isoform X1 [B. mutus]
ENSP00000246505-D1	1.86	2.77E-36	↓	PCI domain-containing protein 2, partial [B. mutus]
ENSP00000379144-D1	1.87	3.68E-64	↓	Trinucleotide repeat-containing 6A protein, partial [B. mutus]
ENSP00000282572-D1	1.87	1.57E-44	↓	cyclin-O [B. mutus]
ENSP00000371607-D1	1.87	1.30E-28	↓	Mitochondrial intermediate peptidase, partial [B. mutus]
ENSBTAP00000050902-D1	1.87	2.45E-39	↓	Zinc finger protein 699, partial [B. mutus]
ENSBTAP00000021483-D1	1.87	6.95E-202	↓	Liprin-beta-2, partial [B. mutus]
ENSBTAP00000028884-D1	1.87	1.63E-304	↓	E3 ubiquitin-protein ligase Praja-2, partial [B. mutus]
ENSBTAP00000009850-D1	1.87	0.00E+00	↓	nuclear receptor coactivator 3 [B. mutus]
ENSBTAP00000044223-D1	1.87	2.40E-14	↓	Protein slowmo-like protein 1, partial [B. mutus]
ENSBTAP00000043096-D1	1.87	4.64E-58	↓	Helicase ARIP4 [B. mutus]
ENSP00000265080-D1	1.87	1.71E-32	↓	ras-specific guanine nucleotide-releasing factor 2 [B. mutus]
ENSP00000340010-D1	1.87	8.52E-10	↓	Insulin-induced 1 protein, partial [B. mutus]
ENSP00000239440-D1	1.87	8.52E-10	↓	arf-GAP with Rho-GAP domain, ANK repeat and PH domain-containing protein 3 [B. mutus]
ENSBTAP00000006178-D1	1.87	2.60E-07	↓	histone-lysine N-methyltransferase SUV39H1 isoform X2 [Orcinus orca]
ENSP00000284878-D1	1.87	1.21E-05	↓	Coxsackievirus and adenovirus receptor-like protein, partial [B. mutus]
ENSP00000379852-D97	1.87	1.21E-05	↓	Zinc finger protein 271, partial [B. mutus]
ENSP00000294244-D1	1.87	3.17E-05	↓	hypothetical protein M91_13022, partial [B. mutus]
ENSP00000360103-D1	1.87	3.17E-05	↓	Palmitoyltransferase ZDHHC9 [B. mutus]
ENSBTAP00000003272-D1	1.87	3.17E-05	↓	synaptotagmin-5 [B. taurus]
ENSBTAP00000005525-D1	1.87	8.37E-05	↓	Mitogen-activated protein kinase kinase kinase 2, partial [B. mutus]
ENSBTAP00000004849-D1	1.87	8.37E-05	↓	leucine-rich repeat-containing protein 37A-like [B. mutus]
ENSP00000282928-D1	1.87	2.21E-04	↓	zinc finger protein ZIC 1 [Trichechus manatus latirostris]
ENSBTAP00000019129-D2	1.87	2.21E-04	↓	5'-AMP-activated protein kinase subunit beta-1 [B. mutus]
ENSBTAP00000025343-D1	1.87	2.21E-04	↓	Putative phospholipase B-like 2, partial [B. mutus]
ENSBTAP00000024049-D1	1.87	5.87E-04	↓	Spermatogenesis-associated protein 2, partial [B. mutus]
ENSP00000378472-D1	1.87	5.87E-04	↓	transcription factor 7 isoform X1 [B. taurus]
ENSP00000292476-D1	1.87	5.87E-04	↓	cleavage and polyadenylation specificity factor subunit 4 isoform X2 [Chinchilla lanigera]
ENSP00000335147-D1	1.87	5.87E-04	↓	ankyrin repeat domain-containing protein 37 [B. taurus]
ENSP00000361304-D1	1.87	1.57E-03	↓	solute carrier family 35 member C2 isoform X1 [B. mutus]
ENSP00000404220-D1	1.87	1.57E-03	↓	Coiled-coil domain-containing protein 155, partial [B. mutus]
ENSBTAP00000020622-D1	1.87	1.57E-03	↓	keratin, type I cytoskeletal 12 [B. mutus]
ENSBTAP00000016559-D1	1.87	1.57E-03	↓	TBC1 domain family member 13 [B. taurus]

ENSP00000394033-D1	1.87	4.25E-03	↓	potassium channel subfamily K member 2 isoform X3 [Ovis aries musimon]
ENSP00000347931-D1	1.87	4.25E-03	↓	Pecanex-like protein 3 [B. mutus]
yakG030921	1.87	4.25E-03	↓	Fer-1-like protein 5 [B. mutus]
ENSP00000391669-D2	1.87	4.25E-03	↓	Enhancer of filamentation 1, partial [B. mutus]
ENSBTAP00000022157-D1	1.87	4.25E-03	↓	ubl carboxyl-terminal hydrolase 18 [B. taurus]
ENSP00000407281-D1	1.87	4.25E-03	↓	WD repeat-containing protein 24, partial [Ovis aries]
ENSP00000257068-D1	1.87	1.17E-02	↓	Melatonin receptor type 1B, partial [B. mutus]
ENSP00000331965-D1	1.87	1.17E-02	↓	TLC domain-containing protein 2 [B. mutus]
ENSBTAP00000029033-D1	1.87	1.17E-02	↓	Selenoprotein N, partial [B. mutus]
ENSP00000386029-D1	1.87	1.17E-02	↓	Gamma-aminobutyric acid receptor subunit rho-2 [B. mutus]
ENSP00000217260-D1	1.87	1.17E-02	↓	R-spondin-4 [B. mutus]
ENSP00000373698-D1	1.87	1.17E-02	↓	F-box/WD repeat-containing protein 4 isoform X1 [Bison bison bison]
ENSBTAP00000022052-D1	1.87	1.17E-02	↓	hypothetical protein M91_03970, partial [B. mutus]
ENSBTAP00000001373-D1	1.87	1.17E-02	↓	Glycogen phosphorylase, muscle form, partial [B. mutus]
ENSP00000377545-D1	1.87	1.17E-02	↓	Complement C1q tumor necrosis factor-related protein 2, partial [B. mutus]
ENSP00000284274-D1	1.87	1.17E-02	↓	Protein FAM105B, partial [B. mutus]
ENSBTAP00000029870-D1	1.87	1.17E-02	↓	epidermal growth factor receptor kinase substrate 8-like protein 2 [B. mutus]
ENSBTAP00000024749-D1	1.87	1.17E-02	↓	Heat shock protein beta-6 [B. mutus]
ENSP00000262915-D1	1.87	1.17E-02	↓	CMP-N-acetylneuraminate-beta-1,4-galactoside alpha-2,3-sialyltransferase isoform a [Homo sapiens]
ENSBTAP00000048024-D1	1.87	1.17E-02	↓	Putative arylformamidase [B. mutus]
ENSP00000274565-D1	1.87	1.17E-02	↓	Serine protease inhibitor Kazal-type 7 [B. mutus]
ENSBTAP00000051910-D56	1.87	3.51E-02	↓	hypothetical protein M91_06993, partial [B. mutus]
ENSP00000309504-D1	1.87	3.51E-02	↓	Anion exchange transporter, partial [B. mutus]
ENSBTAP00000031467-D2	1.87	3.51E-02	↓	cardiotrophin-2-like [Bubalus bubalis]
ENSBTAP00000023918-D4	1.87	3.51E-02	↓	histone H2B type W-T-like [B. mutus]
ENSP00000268720-D2	1.87	3.51E-02	↓	copine-6 isoform X1 [B. mutus]
ENSP00000261326-D1	1.87	3.51E-02	↓	Molybdenum cofactor sulfurase [B. mutus]
ENSBTAP00000006320-D1	1.87	3.51E-02	↓	hypothetical protein M91_04687, partial [B. mutus]
ENSP00000332123-D1	1.87	3.51E-02	↓	ProSAP-interacting protein 1 [B. mutus]
ENSBTAP00000030053-D1	1.87	3.51E-02	↓	androgen receptor [Capreolus capreolus]
yakA02780	1.87	3.51E-02	↓	Lipoma HMGIC fusion partner [B. mutus]
ENSP00000349297-D1	1.87	3.51E-02	↓	Very long-chain specific acyl-CoA dehydrogenase, mitochondrial, partial [B. mutus]
yakA27206	1.87	3.51E-02	↓	hypothetical protein M91_10365 [B. mutus]
ENSBTAP00000053343-D1	1.87	3.51E-02	↓	armadillo repeat protein deleted in velo-cardio-facial syndrome homolog [B. mutus]
ENSP00000329697-D1	1.87	3.51E-02	↓	Lipid phosphate phosphohydrolase 2, partial [B. mutus]
ENSBTAP00000026100-D1	1.87	3.51E-02	↓	boLa class II histocompatibility antigen, DQB*0101 beta chain-like [B. mutus]
ENSP00000323678-D1	1.87	3.51E-02	↓	Zinc-binding alcohol dehydrogenase domain-containing protein 2, partial [B. mutus]
ENSP00000337103-D1	1.87	3.51E-02	↓	choline O-acetyltransferase [B. mutus]
ENSBTAP00000042409-D3	1.87	3.51E-02	↓	hypothetical protein M91_14590 [B. mutus]
ENSP00000328190-D1	1.87	3.51E-02	↓	septin-5 [B. taurus]
ENSP00000372319-D2	1.87	3.51E-02	↓	Chromodomain Y-like protein 2, partial [B. mutus]
ENSBTAP00000039021-D1	1.87	6.79E-164	↓	3-phosphoinositide-dependent protein kinase 1, partial [B. mutus]
ENSBTAP00000024191-D1	1.87	2.42E-55	↓	Nuclear prelamin A recognition factor [B. mutus]
ENSP00000307604-D1	1.88	1.49E-35	↓	zinc finger and BTB domain-containing protein 5 [B. mutus]
ENSBTAP00000001491-D1	1.88	8.34E-60	↓	Coronin-6, partial [B. mutus]
ENSBTAP00000043672-D1	1.88	4.51E-111	↓	DNA repair protein complementing XP-G cells [B. mutus]
ENSBTAP00000005927-D1	1.88	3.15E-77	↓	Putative E3 ubiquitin-protein ligase makorin-2, partial [B. mutus]
ENSP00000309934-D1	1.88	1.03E-36	↓	OS-4 protein [Homo sapiens]
ENSP00000383600-D1	1.88	6.20E-23	↓	transmembrane protein 51 [B. taurus]
ENSBTAP00000009376-D1	1.88	2.80E-81	↓	Brefeldin A-inhibited guanine nucleotide-exchange protein 2 [B. mutus]
ENSP00000393248-D3	1.88	5.26E-41	↓	Polyadenylate-binding protein 4, partial [B. mutus]
ENSBTAP00000024937-D1	1.88	2.65E-97	↓	UPF0687 protein C20orf27 homolog isoform X2 [B. mutus]
ENSP00000318604-D1	1.88	1.83E-20	↓	repressor of RNA polymerase III transcription MAF1 homolog isoform X1 [Balaenoptera acutorostrata scammoni]
ENSP00000355428-D1	1.89	1.56E-46	↓	SH3 domain-binding protein 5-like [B. mutus]
ENSP00000273395-D1	1.89	5.77E-10	↓	Brother of CDO [B. mutus]

ENSBTAP00000002274-D1	1.89	9.22E-41	↓	ubinuclein-1 isoform X1 [Bison bison bison]
ENSP00000317891-D1	1.89	3.88E-09	↓	TNFAIP3-interacting protein 1 [B. mutus]
ENSBTAP00000052213-D1	1.89	6.95E-72	↓	Protein transport protein Sec16B [B. mutus]
ENSBTAP00000024133-D1	1.89	2.44E-50	↓	vacuolar protein sorting-associated protein 53 homolog [B. taurus]
ENSBTAP00000053117-D1	1.89	7.40E-14	↓	crossover junction endonuclease MUS81 [B. mutus]
ENSBTAP00000001000-D1	1.89	6.39E-25	↓	E3 SUMO-protein ligase PIAS4, partial [B. mutus]
ENSBTAP00000048532-D1	1.89	1.76E-07	↓	Zinc finger and BTB domain-containing protein 9, partial [B. mutus]
ENSP00000248929-D1	1.89	1.76E-07	↓	Small G protein signaling modulator 3, partial [B. mutus]
ENSP00000321346-D1	1.89	1.76E-07	↓	Tubulin polyglutamylase TTLL11, partial [B. mutus]
ENSP00000375586-D1	1.89	2.47E-34	↓	Zinc finger protein 304, partial [B. mutus]
ENSP00000335040-D1	1.90	9.64E-18	↓	Niban-like protein 2, partial [B. mutus]
ENSBTAP00000002844-D1	1.90	9.64E-18	↓	RRP12-like protein [B. mutus]
ENSBTAP00000012945-D1	1.90	1.78E-100	↓	cell division cycle protein 20 homolog isoform X1 [B. taurus]
ENSBTAP00000003273-D1	1.90	1.67E-60	↓	Methylthioribose-1-phosphate isomerase, partial [B. mutus]
ENSBTAP00000027093-D1	1.90	1.12E-50	↓	Ubiquinone biosynthesis monooxygenase COQ6, partial [B. mutus]
ENSBTAP00000011217-D1	1.90	1.20E-06	↓	F-box only protein 42 [B. mutus]
ENSP00000395389-D1	1.90	5.83E-11	↓	lysophosphatidic acid receptor 3 [B. taurus]
ENSP00000411761-D1	1.90	5.83E-11	↓	dual specificity mitogen-activated protein kinase kinase 7 isoform X1 [Nannospalax galili]
ENSP00000350085-D20	1.90	1.71E-140	↓	Zinc finger protein 208, partial [B. mutus]
ENSBTAP00000005656-D1	1.90	8.26E-240	↓	Vigilin [B. mutus]
ENSP00000349259-D1	1.90	3.58E-291	↓	Spectrin beta chain, brain 1 [B. mutus]
ENSBTAP00000001239-D1	1.90	3.12E-06	↓	zinc finger protein 746 [B. taurus]
ENSBTAP00000026370-D1	1.90	1.98E-51	↓	Grainyhead-like protein 3-like protein, partial [B. mutus]
ENSBTAP00000008069-D1	1.90	5.01E-14	↓	DENN domain-containing protein 3, partial [B. mutus]
ENSBTAP00000037204-D1	1.90	2.57E-37	↓	Protein argonaute-2, partial [B. mutus]
ENSP00000375693-D1	1.90	8.15E-06	↓	DNA polymerase delta catalytic subunit [B. mutus]
ENSP00000384651-D1	1.91	1.84E-168	↓	myotubularin-related protein 3 isoform X4 [Bison bison bison]
ENSBTAP00000029084-D1	1.91	2.54E-19	↓	CD97 antigen isoform X1 [B. mutus]
ENSBTAP00000027206-D1	1.91	5.76E-22	↓	probable ATP-dependent RNA helicase DHX37 [B. mutus]
ENSBTAP00000027280-D1	1.91	1.62E-102	↓	lysine-specific demethylase 5A [B. mutus]
ENSBTAP00000010654-D1	1.91	1.40E-55	↓	Golgin subfamily A member 7B, partial [B. mutus]
ENSP00000295902-D2	1.91	2.04E-276	↓	prickle-like protein 1 isoform X1 [B. mutus]
ENSBTAP00000051325-D1	1.91	7.72E-27	↓	Katanin p80 WD40-containing subunit B1, partial [B. mutus]
ENSP00000411132-D5	1.91	2.95E-17	↓	hypothetical protein M91_15431, partial [B. mutus]
ENSBTAP00000028637-D1	1.91	4.56E-08	↓	Olfactomedin-like protein 1 [B. mutus]
ENSP00000412272-D1	1.91	5.61E-05	↓	TPA: Putative uncharacterized protein C10orf41-like [B. taurus]
ENSP00000262464-D1	1.91	5.61E-05	↓	fibrillin-2 precursor [B. taurus]
ENSBTAP00000029529-D1	1.92	8.91E-25	↓	Two pore calcium channel protein 1, partial [B. mutus]
ENSBTAP00000007766-D1	1.92	2.63E-10	↓	DNA methyltransferase 1-associated protein 1 [B. mutus]
ENSBTAP00000012142-D1	1.92	5.88E-13	↓	mitochondrial import receptor subunit TOM40B [B. taurus]
ENSBTAP00000002220-D1	1.92	5.60E-50	↓	RNA polymerase II-associated protein 1 [B. mutus]
ENSP00000263847-D1	1.92	3.07E-07	↓	Oxysterol-binding protein 1, partial [B. mutus]
ENSBTAP00000023781-D4	1.92	3.07E-07	↓	Bone morphogenetic protein 6, partial [B. mutus]
ENSBTAP00000022295-D1	1.92	1.48E-04	↓	uncharacterized protein C7orf61 homolog [B. mutus]
ENSBTAP00000024319-D1	1.92	1.48E-04	↓	putative sodium-coupled neutral amino acid transporter 10 [B. mutus]
ENSP00000354201-D1	1.92	1.48E-04	↓	ubiquitin-conjugating enzyme E2 Z [B. mutus]
ENSP00000362076-D1	1.92	1.48E-04	↓	Rhomboid-related protein 2, partial [B. mutus]
ENSP00000349770-D2	1.92	1.06E-34	↓	Interferon regulatory factor 6, partial [B. mutus]
ENSP00000380386-D1	1.93	1.03E-64	↓	ELKS/Rab6-interacting/CAST family member 1 [B. taurus]
ENSBTAP00000025703-D1	1.93	1.19E-08	↓	BTB/POZ domain-containing protein 3, partial [B. mutus]
ENSBTAP00000039748-D1	1.93	3.93E-04	↓	hypothetical protein M91_07905, partial [B. mutus]
ENSP00000233575-D2	1.93	3.93E-04	↓	sorting nexin-17 [B. taurus]
ENSP00000232888-D1	1.93	1.77E-10	↓	U3 small nucleolar RNA-interacting protein 2 [B. mutus]
ENSP00000321636-D1	1.93	1.77E-10	↓	DNA topoisomerase 3-alpha [B. mutus]
ENSBTAP00000027246-D1	1.93	1.79E-54	↓	Thimet oligopeptidase, partial [B. mutus]

ENSP00000378227-D1	1.93	3.07E-08	↓	Rab11 family-interacting protein 4, partial [B. mutus]
ENSBTAP00000003936-D1	1.93	3.97E-14	↓	Transmembrane protein 86A [B. mutus]
ENSP00000261800-D1	1.93	4.59E-10	↓	protocadherin Fat 2 [B. mutus]
ENSBTAP00000025560-D1	1.94	7.10E-26	↓	ELL-associated factor 1, partial [B. mutus]
ENSBTAP00000001113-D1	1.94	7.94E-22	↓	poly [ADP-ribose] polymerase 1-like, partial [B. mutus]
ENSP00000263733-D1	1.94	7.94E-22	↓	glycosaminoglycan xylosylkinase isoform X1 [B. taurus]
ENSP00000160827-D1	1.94	3.58E-188	↓	KIF22 protein [B. taurus]
ENSBTAP00000005622-D1	1.94	2.49E-32	↓	probable ATP-dependent RNA helicase DDX28 [B. mutus]
ENSP00000351352-D1	1.94	3.17E-95	↓	signal-induced proliferation-associated 1-like protein 1 isoform X1 [B. mutus]
ENSBTAP00000001725-D1	1.94	1.48E-36	↓	Frataxin, mitochondrial [B. mutus]
ENSBTAP00000023029-D2	1.94	6.89E-13	↓	Small ubiquitin-related modifier 3 [B. mutus]
ENSBTAP00000001624-D1	1.94	2.07E-07	↓	mitochondrial glutamate carrier 1 [B. mutus]
ENSBTAP00000016742-D1	1.94	1.05E-03	↓	protein SCO2 homolog, mitochondrial-like [B. mutus]
ENSP00000265071-D3	1.94	1.05E-03	↓	cadherin-10 [Capra hircus]
ENSBTAP00000014120-D1	1.94	2.53E-34	↓	Propionyl-CoA carboxylase alpha chain, mitochondrial, partial [B. mutus]
ENSBTAP00000043056-D1	1.94	9.15E-20	↓	Conserved oligomeric Golgi complex subunit 7 [B. mutus]
ENSP00000244051-D1	1.95	1.19E-10	↓	adenylyltransferase and sulfurtransferase MOCS3 [B. mutus]
ENSP00000376923-D1	1.95	1.40E-22	↓	Nuclear respiratory factor 1 [B. mutus]
ENSBTAP00000023578-D1	1.95	7.98E-09	↓	Zinc finger protein ZFAT, partial [B. mutus]
ENSBTAP00000013278-D1	1.95	7.98E-09	↓	lysine-specific demethylase 3B isoform X2 [B. taurus]
ENSP00000376306-D1	1.95	1.77E-113	↓	Citron Rho-interacting kinase, partial [B. mutus]
ENSBTAP00000018765-D1	1.95	2.06E-08	↓	coiled-coil domain-containing protein 9 [B. taurus]
ENSBTAP00000052162-D1	1.95	3.75E-05	↓	Interleukin-2 receptor subunit alpha, partial [B. mutus]
ENSBTAP00000001704-D2	1.95	3.75E-05	↓	Inhibitor of carbonic anhydrase, partial [B. mutus]
yakA22442	1.95	3.75E-05	↓	Serine/threonine-protein kinase PRKX [B. mutus]
ENSBTAP00000017425-D1	1.95	4.64E-13	↓	stromal interaction molecule 1 isoform X2 [Bison bison bison]
ENSBTAP00000006118-D1	1.96	1.41E-06	↓	Monocarboxylate transporter 12 [B. mutus]
ENSP00000401494-D1	1.96	6.33E-23	↓	autophagy-related protein 101 [B. taurus]
ENSBTAP00000027498-D1	1.96	6.58E-51	↓	F-box only protein 7 [B. mutus]
ENSBTAP00000000786-D1	1.96	7.44E-48	↓	Ankyrin repeat and LEM domain-containing protein 2, partial [B. mutus]
ENSBTAP00000027868-D1	1.96	1.39E-07	↓	Regulator of nonsense transcripts 1 [B. mutus]
ENSP00000362222-D1	1.96	1.39E-07	↓	Serine/threonine-protein kinase 40 [B. mutus]
ENSBTAP00000010350-D1	1.96	1.39E-07	↓	heparan sulfate glucosamine 3-O-sulfotransferase 3B1 [B. mutus]
ENSBTAP00000023559-D1	1.96	1.39E-07	↓	attractin-like protein 1 isoform X4 [Ovis aries]
ENSBTAP00000001467-D1	1.96	3.67E-06	↓	monocarboxylate transporter 6 [B. mutus]
ENSBTAP00000001990-D1	1.96	9.86E-05	↓	protein RRNAD1 [B. mutus]
ENSBTAP00000006808-D1	1.96	9.86E-05	↓	Putative phospholipid-transporting ATPase ID [B. mutus]
ENSBTAP00000023605-D1	1.96	9.86E-05	↓	Regulator of G-protein signaling 9 [B. mutus]
ENSP00000389066-D1	1.96	9.86E-05	↓	Putative ATP-dependent RNA helicase DHX33, partial [B. mutus]
ENSP00000302120-D1	1.96	2.81E-03	↓	tripartite motif containing 8 [Macaca mulatta]
ENSBTAP00000001456-D1	1.96	2.81E-03	↓	Interleukin-28 receptor subunit alpha, partial [B. mutus]
ENSBTAP00000009513-D1	1.96	2.81E-03	↓	WD repeat-containing protein 25 [B. mutus]
ENSP00000355060-D1	1.96	2.81E-03	↓	Rho guanine nucleotide exchange factor 10-like protein, partial [B. mutus]
ENSBTAP00000021091-D1	1.97	1.55E-34	↓	Calpain-14, partial [B. mutus]
ENSBTAP00000053245-D1	1.97	1.90E-132	↓	PH domain leucine-rich repeat-containing protein phosphatase 2 [B. taurus]
ENSP00000338807-D1	1.97	4.73E-18	↓	zinc finger and BTB domain-containing protein 49 [B. mutus]
ENSP00000395538-D1	1.97	5.35E-10	↓	trinucleotide repeat-containing gene 18 protein [B. mutus]
ENSBTAP00000000872-D1	1.97	5.29E-55	↓	transcription factor 20 [B. mutus]
ENSBTAP00000020456-D1	1.97	1.12E-25	↓	Ketosamine-3-kinase, partial [B. mutus]
ENSBTAP00000025683-D1	1.97	3.07E-109	↓	protein kinase C and casein kinase substrate in neurons protein 2 [B. taurus]
ENSBTAP00000044801-D1	1.97	3.59E-07	↓	RING finger protein 26 [B. mutus]
ENSP00000377272-D2	1.97	3.81E-46	↓	protein FAM19A2-like [Tupaia chinensis]
ENSP00000367764-D1	1.98	3.16E-17	↓	FERM domain-containing protein 4A [B. mutus]
ENSBTAP00000026394-D1	1.98	9.55E-06	↓	Protein FAM195B, partial [B. mutus]
ENSBTAP00000011906-D1	1.98	8.08E-16	↓	AP-3 complex subunit delta-1 [B. mutus]

ENSP00000367705-D1	1.98	7.40E-90	↓	BCL-6 corepressor [B. mutus]
ENSP00000408186-D1	1.98	4.08E-54	↓	testis-expressed sequence 264 protein isoform X1 [B. mutus]
ENSBTAP00000045877-D1	1.98	1.28E-89	↓	SH3 domain-binding glutamic acid-rich-like protein 2, partial [B. mutus]
ENSBTAP00000008507-D1	1.98	1.71E-188	↓	polyribonucleotide 5'-hydroxyl-kinase Clp1 isoform X1 [Balaenoptera acutorostrata scammoni]
ENSP00000346255-D1	1.98	1.30E-24	↓	protein phosphatase PTC7 homolog [Trichechus manatus latirostris]
ENSP00000259939-D1	1.99	3.34E-24	↓	E3 ubiquitin-protein ligase RNF144B [B. mutus]
ENSP00000354742-D1	1.99	5.42E-16	↓	Importin-9, partial [B. mutus]
ENSBTAP00000019113-D1	1.99	3.30E-42	↓	Transmembrane and coiled-coil domains protein 3, partial [B. mutus]
ENSBTAP00000036035-D1	1.99	9.27E-09	↓	Ankyrin repeat and SAM domain-containing protein 1A, partial [B. mutus]
ENSP00000331201-D1	1.99	3.60E-13	↓	hepatocyte growth factor-regulated tyrosine kinase substrate isoform X1 [B. taurus]
ENSP00000313419-D1	1.99	3.61E-15	↓	B-lymphocyte antigen CD19 [Bison bison bison]
ENSP00000349929-D1	2.00	2.41E-53	↓	Serologically defined colon cancer antigen 3, partial [B. mutus]
ENSP00000300850-D1	2.00	3.63E-16	↓	zinc finger protein 646 [B. mutus]
ENSBTAP00000020429-D1	2.00	5.83E-26	↓	GA-binding protein subunit beta-2 [B. mutus]
ENSBTAP00000016866-D1	2.00	1.45E-21	↓	hypothetical protein M91_01616, partial [B. mutus]
ENSBTAP00000034758-D1	2.00	9.27E-13	↓	Post-GPI attachment to proteins factor 3 [B. mutus]
ENSP00000310933-D1	2.00	9.27E-13	↓	MAP kinase-activating death domain protein isoform X1 [B. mutus]
ENSP00000376500-D1	2.00	1.50E-24	↓	TNF receptor-associated factor 3 [B. mutus]
ENSP00000419457-D1	2.00	3.70E-20	↓	Homeobox protein NOBOX, partial [B. mutus]
ENSBTAP00000015998-D1	2.00	0.00E+00	↓	1-aminocyclopropane-1-carboxylate synthase-like protein 2, partial [B. mutus]
ENSP00000338266-D1	2.00	6.73E-66	↓	Rap guanine nucleotide exchange factor 1, partial [B. mutus]
ENSBTAP00000029559-D1	2.00	2.39E-09	↓	acetyl-CoA carboxylase 2-like [B. mutus]
ENSP00000276440-D1	2.00	1.94E-108	↓	Dedicator of cytokinesis protein 5, partial [B. mutus]
ENSBTAP00000017794-D1	2.00	2.68E-48	↓	hypothetical protein M91_03667, partial [B. mutus]
ENSBTAP00000029514-D1	2.00	2.40E-11	↓	Ras-related protein Rab-35, partial [B. mutus]
ENSP00000307023-D1	2.00	4.45E-44	↓	Krueppel-like factor 11, partial [B. mutus]
ENSP00000393313-D1	2.01	5.49E-186	↓	Epidermal growth factor receptor substrate 15-like 1, partial [B. mutus]
ENSBTAP00000006899-D1	2.01	2.73E-125	↓	Ras-like protein family member 11A, partial [B. mutus]
ENSBTAP00000014899-D1	2.01	7.60E-43	↓	DnaJ-like protein subfamily C member 11, partial [B. mutus]
ENSP00000389913-D1	2.01	6.55E-23	↓	Pleckstrin-like protein domain-containing family M member 1, partial [B. mutus]
ENSBTAP00000041434-D1	2.01	6.17E-12	↓	Fatty acid desaturase 2-like protein, partial [B. mutus]
ENSP00000352819-D1	2.01	6.23E-07	↓	PR domain zinc finger protein 13 [B. mutus]
ENSBTAP00000010022-D1	2.01	6.23E-07	↓	Cathepsin D, partial [B. mutus]
ENSBTAP00000002297-D1	2.01	6.56E-05	↓	Plasma alpha-L-fucosidase, partial [B. mutus]
ENSBTAP00000047671-D1	2.01	6.91E-04	↓	placenta growth factor isoform X1 [B. taurus]
ENSP00000371800-D1	2.01	6.91E-04	↓	RING finger protein 215, partial [B. mutus]
ENSP00000398736-D1	2.01	7.66E-03	↓	protein CYR61 precursor [B. taurus]
ENSP00000243108-D1	2.01	7.66E-03	↓	homeobox protein Hox-C6 isoform X1 [Mustela putorius furo]
ENSP00000246190-D1	2.01	7.66E-03	↓	N-terminal EF-hand calcium-binding protein 3 [Ceratotherium simum simum]
ENSBTAP00000021895-D1	2.01	7.66E-03	↓	Pyruvate dehydrogenase phosphatase regulatory subunit, mitochondrial, partial [B. mutus]
ENSP00000258400-D1	2.01	7.66E-03	↓	5-hydroxytryptamine receptor 2B [B. mutus]
ENSP00000331062-D1	2.01	7.66E-03	↓	hypothetical protein M91_06234, partial [B. mutus]
ENSBTAP00000004411-D1	2.01	7.66E-03	↓	protein-arginine deiminase type-2 isoform X4 [B. taurus]
ENSBTAP00000053815-D1	2.01	7.66E-03	↓	Renalase, partial [B. mutus]
ENSP00000369833-D1	2.01	7.66E-03	↓	Diamine acetyltransferase 2, partial [B. mutus]
ENSBTAP00000019561-D1	2.01	7.66E-03	↓	Cytochrome b5 domain-containing protein 1 [B. mutus]
ENSBTAP00000053404-D1	2.01	7.66E-03	↓	Alpha-mannosidase 2C1, partial [B. mutus]
ENSBTAP00000005930-D1	2.01	0.00E+00	↓	RAF proto-oncogene serine/threonine-protein kinase [B. taurus]
ENSBTAP00000008367-D1	2.01	5.56E-130	↓	receptor-interacting serine/threonine-protein kinase 1 [B. mutus]
yakG021104	2.01	1.40E-50	↓	Lethal(2) giant larvae protein-like protein 2 [B. mutus]
ENSBTAP00000037377-D1	2.02	1.21E-32	↓	DNA-3-methyladenine glycosylase, partial [B. mutus]
ENSBTAP00000018459-D1	2.02	1.60E-08	↓	E3 ubiquitin-protein ligase TRIM21, partial [B. mutus]
ENSP00000332698-D1	2.02	3.00E-27	↓	Heat shock factor protein 1 [B. mutus]
ENSBTAP00000009843-D1	2.02	1.09E-18	↓	protein unc-119 homolog B isoform X1 [Ovis aries]
ENSP00000330813-D1	2.02	9.04E-169	↓	BRCA1-associated protein [B. mutus]

ENSBTAP00000053528-D1	2.02	2.85E-56	↓	Myotubularin-related protein 12, partial [B. mutus]
ENSP00000410257-D1	2.02	2.00E-65	↓	Sodium channel protein type 5 subunit alpha [B. mutus]
ENSP00000414921-D3	2.03	3.85E-107	↓	Polypyrimidine tract-binding protein 1, partial [B. mutus]
ENSBTAP00000014885-D1	2.03	1.06E-12	↓	Synaptotagmin-11, partial [B. mutus]
ENSBTAP00000017829-D1	2.03	4.12E-09	↓	Rho guanine nucleotide exchange factor 40, partial [B. mutus]
ENSP00000379065-D1	2.03	1.66E-05	↓	Zinc finger transcription factor Trps1, partial [B. mutus]
ENSP00000381523-D1	2.03	1.66E-05	↓	zinc finger and BTB domain-containing protein 21 [B. mutus]
ENSBTAP00000047712-D1	2.03	7.27E-19	↓	Synaptosomal-associated protein 47, partial [B. mutus]
ENSP00000389130-D1	2.03	1.69E-41	↓	DNA-binding protein RFX5 [B. mutus]
ENSP00000386310-D1	2.03	1.06E-10	↓	SPATS2-like protein, partial [B. mutus]
ENSP00000296043-D1	2.03	2.36E-31	↓	protein Shroom3 [B. mutus]
ENSBTAP00000041432-D1	2.04	4.14E-07	↓	Hormone-sensitive lipase, partial [B. mutus]
ENSBTAP00000004504-D24	2.04	1.72E-04	↓	hypothetical protein M91_02901, partial [B. mutus]
ENSBTAP00000016944-D1	2.04	9.79E-59	↓	testis intracellular mediator protein [B. taurus]
ENSBTAP00000009273-D1	2.04	1.06E-09	↓	zinc finger homeobox protein 2 [Camelus bactrianus]
ENSBTAP00000000184-D1	2.04	2.74E-11	↓	cystathionine beta-synthase [B. taurus]
ENSBTAP00000038956-D1	2.04	1.44E-158	↓	RING finger protein 114, partial [B. mutus]
ENSP00000319152-D1	2.04	4.09E-33	↓	Equilibrative nucleoside transporter 1, partial [B. mutus]
ENSP00000289968-D1	2.04	8.64E-24	↓	Rho GTPase-activating protein 17, partial [B. mutus]
ENSP00000338171-D1	2.04	4.22E-06	↓	Src kinase-associated phosphoprotein 1, partial [B. mutus]
ENSP00000293925-D1	2.04	4.22E-06	↓	Protein cramped-like protein, partial [B. mutus]
ENSBTAP00000002387-D1	2.04	4.86E-39	↓	hypothetical protein M91_18932, partial [B. mutus]
ENSP00000381590-D1	2.05	1.87E-79	↓	Ataxin-7, partial [B. mutus]
ENSBTAP00000009771-D1	2.05	1.24E-79	↓	Myb-binding protein 1A [B. mutus]
ENSP00000358131-D1	2.05	5.55E-21	↓	OTU domain-containing protein 7B [B. mutus]
ENSBTAP00000049569-D6	2.05	3.16E-16	↓	melanoma-associated antigen B2-like [B. taurus]
ENSP00000239830-D1	2.05	7.06E-10	↓	Coiled-coil domain-containing protein 77 [B. mutus]
ENSP00000254667-D1	2.05	2.73E-08	↓	Receptor-type tyrosine-protein phosphatase epsilon [B. mutus]
yakG007403	2.05	1.84E-03	↓	hypothetical protein M91_15425 [B. mutus]
ENSP00000204517-D1	2.05	1.84E-03	↓	transcription factor AP-4 [B. taurus]
ENSBTAP00000048850-D42	2.05	1.84E-03	↓	hypothetical protein M91_14973, partial [B. mutus]
ENSBTAP00000041141-D1	2.05	1.84E-03	↓	Phosphatidylethanolamine N-methyltransferase, partial [B. mutus]
ENSBTAP00000008215-D1	2.05	1.84E-03	↓	uncharacterized protein KIAA1614 homolog [B. mutus]
ENSP00000303192-D1	2.05	9.70E-62	↓	ral guanine nucleotide dissociation stimulator-like 1 [B. taurus]
ENSBTAP00000028227-D1	2.06	8.18E-17	↓	Mitogen-activated protein kinase kinase kinase MLK4, partial [B. mutus]
ENSBTAP00000004368-D1	2.06	7.06E-09	↓	patatin-like phospholipase domain-containing protein 3 isoform X3 [B. taurus]
ENSBTAP00000006828-D1	2.06	7.06E-09	↓	mevalonate kinase [B. taurus]
ENSP00000357465-D2	2.06	5.29E-34	↓	Tudor domain-containing protein 10, partial [B. mutus]
ENSP00000297293-D1	2.06	5.45E-18	↓	Serine/threonine-protein kinase LMTK2, partial [B. mutus]
ENSP00000414552-D1	2.06	2.74E-07	↓	mitogen-activated protein kinase kinase kinase 12 isoform X1 [B. taurus]
ENSP00000216268-D1	2.06	2.74E-07	↓	zinc finger BED domain-containing protein 4 [B. mutus]
ENSBTAP00000007733-D1	2.06	1.10E-05	↓	zinc finger protein 333 isoform X1 [B. taurus]
ENSBTAP00000019460-D2	2.07	1.40E-17	↓	hypothetical protein M91_20028, partial [B. mutus]
ENSP00000376436-D1	2.07	8.68E-48	↓	Transforming protein p68/c-ets-1 [B. mutus]
ENSBTAP00000049853-D1	2.07	3.10E-12	↓	WD repeat-containing protein 1, partial [B. mutus]
ENSBTAP00000015242-D1	2.07	5.35E-15	↓	Cysteinyl-tRNA synthetase, mitochondrial, partial [B. mutus]
ENSBTAP00000025945-D1	2.07	1.81E-08	↓	N-acylethanolamine-hydrolyzing acid amidase, partial [B. mutus]
ENSBTAP00000017554-D1	2.07	2.61E-112	↓	Disks large-like protein 5, partial [B. mutus]
ENSP00000354718-D1	2.07	1.93E-26	↓	A-kinase anchor protein 13 [B. mutus]
ENSBTAP00000028777-D1	2.08	0.00E+00	↓	tudor and KH domain-containing protein [B. mutus]
ENSBTAP00000023199-D1	2.08	1.61E-20	↓	serine/threonine-protein kinase 10 [B. mutus]
ENSBTAP00000052877-D1	2.08	2.26E-116	↓	GATS-like protein 3 [B. mutus]
ENSP00000372319-D1	2.08	5.13E-54	↓	Chromodomain Y-like protein, partial [B. mutus]
ENSBTAP00000041322-D1	2.08	2.06E-170	↓	hypothetical protein M91_00923 [B. mutus]
ENSBTAP00000040456-D2	2.08	1.71E-225	↓	Tubulin gamma-2 chain, partial [B. mutus]

ENSBTAP00000024595-D1	2.08	1.20E-09	↓	kelch repeat and BTB domain-containing protein 4 isoform X1 [B. mutus]
ENSP00000296328-D1	2.08	1.92E-78	↓	UBX domain-containing protein 7 [B. mutus]
ENSBTAP00000008501-D1	2.09	9.11E-16	↓	tRNA-splicing endonuclease subunit Sen34 [B. mutus]
ENSBTAP00000053836-D1	2.09	3.09E-10	↓	run domain Beclin-1 interacting and cysteine-rich containing protein-like isoform X1 [B. mutus]
ENSP00000380401-D1	2.09	1.13E-04	↓	FAD-dependent oxidoreductase domain-containing protein 2 [B. mutus]
ENSBTAP00000024795-D1	2.09	1.13E-04	↓	Guanosine-3',5'-bis(diphosphate) 3'-pyrophosphohydrolase MESH1, partial [B. mutus]
ENSP00000204279-D4	2.09	1.13E-04	↓	Protein yippee-like 1 [B. mutus]
ENSBTAP00000034529-D1	2.09	1.13E-04	↓	hypothetical protein M91_18808, partial [B. mutus]
ENSP00000311657-D1	2.09	5.24E-39	↓	GRAM domain-containing protein 2 [Pantholops hodgsonii]
ENSBTAP00000008101-D1	2.09	2.36E-16	↓	sulfite oxidase, mitochondrial [B. taurus]
ENSP00000366453-D1	2.09	5.66E-28	↓	tight junction protein ZO-2 isoform X6 [Bison bison bison]
ENSBTAP00000018609-D1	2.09	1.85E-22	↓	torsin-3A [B. mutus]
ENSP00000380679-D1	2.09	6.11E-17	↓	Coiled-coil domain-containing protein 40, partial [B. mutus]
ENSP00000363349-D1	2.09	3.79E-29	↓	Sorting nexin-30, partial [B. mutus]
ENSBTAP00000050075-D1	2.09	1.34E-38	↓	TNF receptor-associated factor 4 [B. taurus]
ENSP00000366829-D1	2.09	3.22E-24	↓	RNA-binding protein 10, partial [B. mutus]
ENSP00000347498-D1	2.10	1.36E-12	↓	Zinc finger FYVE domain-containing protein 19, partial [B. mutus]
ENSP00000284770-D1	2.10	7.21E-06	↓	PDZ and LIM domain protein 3 isoform X1 [B. taurus]
ENSP00000381797-D1	2.10	7.21E-06	↓	zinc finger protein DPF3 isoform X1 [B. taurus]
ENSBTAP00000032458-D1	2.10	3.86E-221	↓	protein BANP isoform X1 [Bubalus bubalis]
ENSBTAP00000025728-D1	2.10	1.84E-06	↓	COP9 signalosome complex subunit 1, partial [B. mutus]
ENSBTAP00000005338-D1	2.10	1.10E-184	↓	histone chaperone ASF1B [B. taurus]
ENSBTAP00000019948-D1	2.11	3.27E-67	↓	regulation of nuclear pre-mRNA domain-containing protein 2 isoform X1 [B. mutus]
ENSP00000286067-D1	2.11	4.02E-49	↓	uncharacterized protein C10orf12 homolog isoform X1 [B. mutus]
ENSP00000364324-D1	2.11	1.20E-07	↓	constitutive coactivator of PPAR-gamma-like protein 2 [B. mutus]
ENSBTAP00000007903-D1	2.11	1.20E-07	↓	GTP-binding protein 2, partial [B. mutus]
ENSP00000372326-D1	2.11	1.20E-07	↓	ferrochelatase, mitochondrial [Bubalus bubalis]
ENSP00000396032-D1	2.11	1.20E-07	↓	Putative protein KIAA0802, partial [B. mutus]
ENSP00000216492-D1	2.11	1.20E-07	↓	Chromogranin-A [B. mutus]
ENSBTAP00000032231-D1	2.11	2.73E-106	↓	transducin-like enhancer protein 4 [B. taurus]
ENSP00000338352-D1	2.11	1.24E-132	↓	diphosphoinositol polyphosphate phosphohydrolase 2 isoform X1 [B. mutus]
ENSBTAP00000009046-D1	2.11	1.51E-46	↓	ras GTPase-activating-like protein IQGAP3 [B. mutus]
ENSP00000387292-D1	2.12	6.26E-146	↓	transmembrane protein 150A precursor [B. taurus]
ENSBTAP00000002448-D1	2.12	2.04E-09	↓	Period circadian protein-like protein 2, partial [B. mutus]
ENSBTAP00000001972-D1	2.12	3.48E-11	↓	Huntingtin, partial [B. mutus]
ENSBTAP00000022529-D1	2.12	8.45E-104	↓	uncharacterized protein C2orf42 homolog isoform X1 [B. mutus]
ENSP00000397925-D1	2.12	7.20E-38	↓	Calcium-transporting ATPase type 2C member 2, partial [B. mutus]
ENSP00000377612-D1	2.13	3.91E-64	↓	Extended synaptotagmin-1 [B. mutus]
ENSP00000390342-D1	2.13	0.00E+00	↓	hypothetical protein M91_18721, partial [B. mutus]
ENSP00000276708-D1	2.13	5.97E-23	↓	Gasdermin-C [B. mutus]
ENSP00000308122-D1	2.13	3.99E-24	↓	V-type proton ATPase 116 kDa subunit a isoform 4 [B. mutus]
ENSBTAP00000020417-D1	2.13	1.34E-129	↓	CDC42 small effector protein 1 [Homo sapiens]
ENSBTAP00000009708-D1	2.13	2.87E-55	↓	Cytohesin-3, partial [B. mutus]
ENSP00000253925-D1	2.13	2.42E-214	↓	Liprin-alpha-1 [B. mutus]
ENSBTAP00000030064-D1	2.13	1.00E-244	↓	zinc finger protein ubi-d4 isoform X1 [Loxodonta africana]
ENSP00000227520-D1	2.13	3.38E-20	↓	coiled-coil domain-containing protein 86 [B. mutus]
ENSBTAP00000019591-D1	2.13	1.51E-12	↓	Mucin-4, partial [B. mutus]
ENSBTAP00000017306-D1	2.13	8.90E-11	↓	AMP deaminase 2 isoform X4 [B. mutus]
ENSP00000327889-D1	2.13	1.34E-09	↓	integrator complex subunit 5 [B. mutus]
ENSP00000386183-D1	2.13	1.34E-09	↓	hypothetical protein PANDA_004034, partial [Ailuropoda melanoleuca]
ENSBTAP00000025446-D1	2.13	2.03E-08	↓	Cdc42 effector protein 4 [B. mutus]
ENSBTAP00000002094-D2	2.13	3.07E-07	↓	Membrane-associated phosphatidylinositol transfer protein 1, partial [B. mutus]
ENSBTAP00000042192-D1	2.13	1.21E-06	↓	Major facilitator superfamily domain-containing protein 7, partial [B. mutus]
ENSP00000360261-D1	2.13	7.40E-05	↓	Tectonic-3, partial [B. mutus]
ENSP00000316786-D1	2.13	7.40E-05	↓	corticosteroid 11-beta-dehydrogenase isozyme 2 [Bison bison bison]

ENSBTAP00000053241-D1	2.13	7.40E-05	↓	glucose-fructose oxidoreductase domain-containing protein 1 isoform X1 [B. mutus]
ENSBTAP00000005794-D1	2.13	2.95E-04	↓	Bromo adjacent-like protein domain-containing 1 protein, partial [B. mutus]
ENSBTAP00000042196-D1	2.13	2.95E-04	↓	Serine protease 53, partial [B. mutus]
ENSBTAP00000047821-D1	2.13	2.95E-04	↓	beta-1,3-galactosyltransferase 6 [Bubalus bubalis]
ENSBTAP00000002419-D1	2.13	1.19E-03	↓	ubiquinone biosynthesis protein COQ9, mitochondrial [B. mutus]
ENSP00000377233-D1	2.13	1.19E-03	↓	Arf-GAP with Rho-GAP domain, ANK repeat and PH domain-containing protein 1, partial [B. mutus]
ENSBTAP00000003926-D1	2.13	4.95E-03	↓	Methyl-CpG-binding domain protein 3, partial [B. mutus]
yakG022792	2.13	4.95E-03	↓	hypothetical protein M91_04385 [B. mutus]
ENSP00000316193-D1	2.13	4.95E-03	↓	deoxyribonuclease gamma isoform X1 [B. mutus]
ENSP00000256857-D1	2.13	4.95E-03	↓	Gastrin-releasing peptide, partial [B. mutus]
ENSP00000327168-D1	2.13	4.95E-03	↓	proline-rich protein 7 [Tupaia chinensis]
ENSP00000381891-D1	2.13	4.95E-03	↓	transcriptional regulator ERG isoform X1 [B. mutus]
ENSBTAP00000030983-D1	2.13	4.95E-03	↓	hypothetical protein M91_19668, partial [B. mutus]
ENSP00000289473-D1	2.13	4.95E-03	↓	neutrophil cytosol factor 1 [B. mutus]
ENSBTAP00000007767-D3	2.13	4.95E-03	↓	olfactory receptor 4B1-like [Bison bison bison]
ENSP00000330382-D1	2.13	2.16E-02	↓	Platelet-derived growth factor subunit B, partial [B. mutus]
ENSBTAP00000038860-D1	2.13	2.16E-02	↓	Myelin transcription factor 1, partial [B. mutus]
ENSBTAP00000014838-D2	2.13	2.16E-02	↓	TPA: family with sequence similarity 38, member A-like [B. taurus]
ENSP00000262207-D1	2.13	2.16E-02	↓	cysteine-rich secretory protein LCCL domain-containing 1 [B. mutus]
yakG033364	2.13	2.16E-02	↓	hypothetical protein M91_21693 [B. mutus]
ENSP00000367345-D4	2.13	2.16E-02	↓	Protocadherin gamma-A8, partial [B. mutus]
ENSP00000410789-D1	2.13	2.16E-02	↓	ephrin type-B receptor 6 [B. mutus]
ENSP00000275730-D1	2.13	2.16E-02	↓	Solute carrier family 12 member 9 [B. mutus]
ENSBTAP00000001772-D1	2.13	2.16E-02	↓	stimulated by retinoic acid 8 [B. grunniens]
ENSBTAP00000044639-D18	2.13	2.16E-02	↓	40S ribosomal protein S3a-like [Cercocebus atys]
ENSP00000232744-D1	2.13	2.16E-02	↓	Ankyrin repeat and BTB/POZ domain-containing protein 1, partial [B. mutus]
ENSP00000279247-D1	2.13	2.16E-02	↓	Calpain-1 catalytic subunit [B. mutus]
ENSBTAP00000006590-D1	2.13	2.16E-02	↓	Alpha-S2-casein, partial [B. mutus]
ENSBTAP00000028155-D1	2.13	2.16E-02	↓	major facilitator superfamily domain-containing protein 10 [B. taurus]
ENSBTAP00000049627-D1	2.13	2.16E-02	↓	left-right determination factor 2-like [B. mutus]
ENSBTAP00000017429-D1	2.13	2.16E-02	↓	FERM, RhoGEF and pleckstrin domain-containing protein 2 isoform X3 [Bubalus bubalis]
ENSP00000389015-D1	2.13	2.16E-02	↓	Serine/threonine-protein kinase SBK2, partial [B. mutus]
ENSBTAP00000023729-D1	2.13	2.16E-02	↓	Retinoic acid receptor RXR-alpha, partial [B. mutus]
ENSBTAP00000040441-D1	2.13	2.16E-02	↓	primary amine oxidase, lung isozyme-like [Bison bison bison]
ENSBTAP00000024756-D1	2.13	2.16E-02	↓	lysozyme-like protein 4 [B. mutus]
ENSBTAP00000023275-D1	2.13	2.16E-02	↓	Cysteinyl leukotriene receptor 2 [B. mutus]
ENSP00000302108-D1	2.14	8.26E-66	↓	pantothenate kinase 1 [Nomascus leucogenys]
ENSP00000368880-D1	2.14	6.30E-60	↓	RecName: Full=Forkhead box protein O1; AltName: Full=Forkhead box protein O1A; AltName: Full=Forkhead in rhabdomyosarcoma
ENSP00000364550-D1	2.15	1.70E-14	↓	lysine-specific demethylase 5C isoform X2 [B. mutus]
ENSBTAP00000027421-D1	2.15	1.70E-14	↓	putative histone-lysine N-methyltransferase PRDM6 isoform X6 [Ovis aries musimon]
ENSP00000358323-D1	2.15	2.31E-186	↓	thioredoxin-interacting protein [B. taurus]
ENSP00000333926-D1	2.15	2.20E-19	↓	Multidrug resistance-associated protein 5 [B. mutus]
ENSP00000268138-D1	2.15	1.57E-157	↓	Treslin [B. mutus]
ENSP00000200181-D1	2.15	8.81E-10	↓	integrin beta-4 isoform X5 [Ovis aries musimon]
ENSP00000228705-D1	2.16	7.42E-16	↓	Protein phosphatase 1H, partial [B. mutus]
ENSBTAP00000018957-D1	2.16	5.75E-63	↓	Zinc finger CCHC domain-containing protein 14 [B. mutus]
ENSP00000312773-D1	2.16	6.55E-13	↓	NF-kappa-B inhibitor-interacting Ras-like protein 2 [B. mutus]
ENSP00000303252-D1	2.16	9.52E-37	↓	Zinc finger protein 804A, partial [B. mutus]
ENSBTAP00000006074-D1	2.16	1.26E-16	↓	cartilage oligomeric matrix protein precursor [B. taurus]
ENSP00000406305-D1	2.16	7.91E-07	↓	galactokinase [B. mutus]
ENSP00000282041-D1	2.17	4.89E-16	↓	UPF0493 protein KIAA1632 [B. mutus]
ENSP00000380144-D1	2.17	3.84E-11	↓	Zinc fingers and homeoboxes protein 3, partial [B. mutus]
ENSBTAP00000027232-D1	2.17	7.75E-126	↓	NACHT, LRR and PYD domains-containing protein 1 [B. mutus]
ENSBTAP00000035049-D1	2.17	3.10E-06	↓	Serine/threonine-protein kinase 32A, partial [B. mutus]

ENSBTAP00000020029-D1	2.17	8.81E-67	↓	evolutionarily conserved signaling intermediate in Toll pathway, mitochondrial isoform X1 [B. mutus]
ENSBTAP00000030443-D1	2.17	2.25E-09	↓	Receptor-type tyrosine-protein phosphatase O, partial [B. mutus]
ENSBTAP00000025071-D1	2.17	1.22E-05	↓	Leucine-rich repeat-containing protein 68, partial [B. mutus]
ENSP00000347755-D89	2.17	1.22E-05	↓	hypothetical protein M91_06004, partial [B. mutus]
ENSBTAP00000000626-D1	2.17	1.22E-05	↓	hypothetical protein M91_16804, partial [B. mutus]
ENSP00000376110-D55	2.18	3.20E-16	↓	Zinc finger protein 555, partial [B. mutus]
ENSP00000367265-D1	2.18	1.24E-15	↓	Cytoskeleton-associated protein 4, partial [B. mutus]
ENSBTAP00000034238-D1	2.18	1.24E-136	↓	hypothetical protein M91_04532, partial [B. mutus]
ENSBTAP00000016977-D1	2.18	1.05E-20	↓	Macrophage colony-stimulating factor 1 receptor, partial [B. mutus]
ENSBTAP00000015917-D1	2.18	3.38E-08	↓	Zinc finger MIZ domain-containing protein 2, partial [B. mutus]
ENSBTAP00000023023-D1	2.18	4.83E-05	↓	DNA polymerase nu [B. mutus]
ENSP00000364126-D1	2.18	5.22E-32	↓	DNA-(apurinic or apyrimidinic site) lyase 2 isoform X1 [B. mutus]
ENSP00000305687-D1	2.19	9.78E-11	↓	chromosome transmission fidelity protein 8 homolog isoform 2-like [B. mutus]
ENSP00000380309-D1	2.19	9.78E-11	↓	AP-1 complex subunit gamma-like 2 isoform X1 [B. mutus]
ENSBTAP00000013737-D1	2.19	1.37E-24	↓	Myosin-9, partial [B. mutus]
ENSP00000395535-D1	2.19	6.12E-19	↓	methyl-CpG-binding protein 2 [B. mutus]
ENSP00000261534-D1	2.19	1.09E-12	↓	Protein O-mannosyl-transferase 2, partial [B. mutus]
ENSBTAP00000027109-D1	2.19	8.95E-25	↓	putative helicase Mov10l1 [B. mutus]
ENSP00000377204-D1	2.19	1.22E-14	↓	G protein-coupled receptor kinase 6, partial [B. mutus]
ENSP00000309878-D1	2.19	4.22E-12	↓	fibroblast growth factor receptor 2 [B. taurus]
ENSBTAP00000019866-D1	2.19	1.47E-09	↓	Semaphorin-3E, partial [B. mutus]
ENSP00000339381-D1	2.19	5.15E-07	↓	Regulator of G-protein signaling 12, partial [B. mutus]
ENSP00000418287-D1	2.19	1.92E-04	↓	probable cytosolic iron-sulfur protein assembly protein CIAO1 [B. mutus]
ENSP00000307234-D1	2.19	1.92E-04	↓	protocadherin beta-1 [B. mutus]
ENSP00000356382-D1	2.19	1.92E-04	↓	coagulation factor XIII B chain-like [B. mutus]
ENSBTAP00000006264-D1	2.19	1.92E-04	↓	retinoic acid-induced protein 1 [B. mutus]
ENSP00000300571-D1	2.19	1.92E-04	↓	G-protein coupled receptor family C group 5 member B, partial [B. mutus]
ENSBTAP00000037133-D1	2.20	4.51E-21	↓	putative methyltransferase NSUN5 isoform X1 [B. mutus]
ENSBTAP00000004465-D1	2.20	3.74E-244	↓	zinc finger and BTB domain-containing protein 43 [B. taurus]
ENSP00000264249-D1	2.20	5.71E-09	↓	Carbohydrate sulfotransferase 10 [B. mutus]
ENSP00000397669-D1	2.20	5.71E-09	↓	Phosphatase and actin regulator 1, partial [B. mutus]
ENSP00000344914-D1	2.20	8.15E-161	↓	dynamin-binding protein [B. mutus]
ENSBTAP00000028663-D1	2.20	1.28E-231	↓	Ubiquitin carboxyl-terminal hydrolase 36 [B. mutus]
ENSBTAP00000030100-D1	2.20	2.03E-06	↓	Integral membrane protein GPR137, partial [B. mutus]
ENSBTAP00000023319-D1	2.20	7.66E-22	↓	peroxisome proliferator-activated receptor delta [B. taurus]
ENSP00000419923-D2	2.20	3.60E-39	↓	Krueppel-like factor 7, partial [B. mutus]
ENSP00000378414-D3	2.21	7.98E-15	↓	SWI/SNF-related matrix-associated actin-dependent regulator of chromatin subfamily D member 2, partial [B. mutus]
ENSBTAP00000045377-D2	2.21	3.10E-36	↓	hypothetical protein M91_12935 [B. mutus]
ENSBTAP00000028783-D1	2.21	4.43E-20	↓	Huntingtin-interacting protein 1-related protein, partial [B. mutus]
ENSBTAP00000022249-D1	2.21	2.21E-23	↓	Zinc finger and BTB domain-containing protein 48 [B. mutus]
ENSP00000311202-D1	2.21	1.72E-19	↓	BTB/POZ domain-containing adapter for CUL3-mediated RhoA degradation protein 1 [B. mutus]
ENSP00000253968-D1	2.21	1.07E-11	↓	Homeobox protein BarH-like 1, partial [B. mutus]
ENSP00000370299-D1	2.21	8.61E-08	↓	Protein Shroom2, partial [B. mutus]
ENSBTAP00000015375-D1	2.21	8.61E-08	↓	ectonucleotide pyrophosphatase/phosphodiesterase family member 6 [B. mutus]
ENSP00000374376-D1	2.21	8.61E-08	↓	hypothetical protein M91_01232 [B. mutus]
ENSBTAP00000031486-D11	2.21	7.93E-06	↓	hypothetical protein M91_01311, partial [B. mutus]
ENSP00000264198-D1	2.21	7.93E-06	↓	mitochondrial ubiquitin ligase activator of NFKB 1 [B. mutus]
ENSBTAP00000046628-D1	2.21	7.93E-06	↓	pogo transposable element with KRAB domain [B. mutus]
ENSP00000350260-D1	2.21	7.71E-04	↓	hypothetical protein M91_06827 [B. mutus]
ENSP00000378912-D1	2.21	7.71E-04	↓	Protein cordon-bleu, partial [B. mutus]
yakA04478	2.21	7.71E-04	↓	chemokine (C-C motif) ligand 27 [B. taurus]
ENSP00000403323-D1	2.21	7.71E-04	↓	rho GTPase-activating protein 27 isoform X3 [Ovis aries musimon]
ENSP00000359698-D1	2.21	7.71E-04	↓	ubiquitin domain-containing protein 1 isoform X1 [Condylura cristata]
ENSP00000375873-D1	2.21	8.83E-134	↓	Autophagy-related protein 16-1, partial [B. mutus]

ENSBTAP00000032948-D1	2.21	2.14E-51	↓	T-complex protein 11-like protein 1 [B. mutus]
ENSP00000416175-D1	2.22	7.82E-36	↓	KAT8 regulatory NSL complex subunit 3 isoform X1 [B. mutus]
ENSBTAP00000028313-D1	2.22	4.66E-13	↓	uncharacterized protein C7orf26 homolog [B. mutus]
ENSBTAP00000014385-D1	2.22	2.88E-20	↓	dimethylaniline monooxygenase [N-oxide-forming] 5-like [B. mutus]
ENSP00000350928-D1	2.22	9.12E-52	↓	glutamate decarboxylase 1 [B. taurus]
ENSBTAP00000007505-D1	2.22	3.35E-07	↓	Protein fucU-like protein, partial [B. mutus]
ENSBTAP00000001356-D1	2.22	3.35E-07	↓	zinc finger protein 598, partial [B. mutus]
ENSBTAP00000002602-D1	2.22	3.35E-07	↓	Kidney mitochondrial carrier protein 1, partial [B. mutus]
ENSP00000342481-D1	2.23	3.84E-17	↓	Putative phospholipid-transporting ATPase IIA, partial [B. mutus]
ENSP00000371345-D1	2.23	7.30E-20	↓	hypothetical protein M91_14892, partial [B. mutus]
ENSP00000166345-D1	2.23	1.19E-279	↓	Thyroid receptor-interacting protein 13, partial [B. mutus]
ENSP00000404382-D1	2.23	5.73E-16	↓	hypothetical protein M91_00857 [B. mutus]
ENSBTAP00000018151-D1	2.23	1.32E-06	↓	neuronal acetylcholine receptor subunit alpha-5 [B. mutus]
ENSBTAP00000048482-D1	2.24	0.00E+00	↓	cadherin-1 [B. mutus]
ENSBTAP00000006308-D1	2.24	9.77E-164	↓	RNA polymerase I-specific transcription initiation factor RRN3 [B. mutus]
ENSP00000229201-D1	2.24	5.53E-91	↓	Protein timeless-like protein, partial [B. mutus]
ENSP00000252992-D1	2.24	0.00E+00	↓	Coiled-coil domain-containing protein 21, partial [B. mutus]
ENSP00000306760-D1	2.24	1.05E-10	↓	Leucine-rich repeat-containing protein 45 [B. mutus]
ENSBTAP00000010364-D1	2.24	1.05E-10	↓	ras-related protein Rab-40C isoform X1 [Orcinus orca]
ENSP00000403802-D1	2.24	1.54E-23	↓	F-box only protein 10 isoform X1 [Bison bison bison]
ENSP00000265634-D1	2.25	3.73E-16	↓	neuronal pentraxin-2 [Bubalus bubalis]
ENSP00000365806-D1	2.25	1.63E-17	↓	guanine nucleotide-binding protein-like 1 [B. mutus]
ENSP00000289166-D1	2.25	9.53E-30	↓	protein FAM46B [B. mutus]
ENSP00000354608-D1	2.25	2.18E-07	↓	lysosomal thioesterase PPT2 precursor [B. taurus]
ENSBTAP00000015922-D1	2.25	2.18E-07	↓	Breast cancer anti-estrogen resistance protein 1, partial [B. mutus]
ENSP00000409227-D1	2.25	1.24E-04	↓	Runt-related transcription factor 1, partial [B. mutus]
ENSP00000222718-D1	2.25	1.24E-04	↓	homeobox protein Hox-A2 [B. mutus]
ENSP00000271555-D1	2.25	1.24E-04	↓	myocyte-specific enhancer factor 2D isoform X1 [B. mutus]
ENSBTAP00000045400-D1	2.25	1.24E-04	↓	hypothetical protein M91_12682, partial [B. mutus]
ENSP00000355614-D1	2.25	9.41E-09	↓	Protein FAM89A, partial [B. mutus]
ENSBTAP00000049559-D1	2.25	5.15E-06	↓	TBC1 domain family member 24, partial [B. mutus]
ENSBTAP00000011219-D1	2.25	3.17E-03	↓	potassium voltage-gated channel subfamily V member 1 [B. taurus]
ENSBTAP00000010814-D1	2.25	3.17E-03	↓	NLR family CARD domain-containing protein 4, partial [B. mutus]
ENSP00000296425-D1	2.25	3.17E-03	↓	membrane-associated progesterone receptor component 2 [B. taurus]
ENSBTAP00000036305-D1	2.25	3.17E-03	↓	ER membrane protein complex subunit 9 [B. taurus]
ENSP00000335651-D1	2.25	3.17E-03	↓	fibronectin type III and SPRY domain-containing protein 2 isoform X1 [B. mutus]
ENSP00000366036-D1	2.26	1.25E-140	↓	TBC1 domain family member 8 isoform X2 [Bison bison bison]
ENSP00000268638-D1	2.27	5.80E-154	↓	interferon regulatory factor 8 isoform X2 [Ovis aries musimon]
ENSP00000402325-D1	2.27	2.65E-10	↓	Putative ATP-dependent RNA helicase DDX11, partial [B. mutus]
ENSP00000357794-D1	2.27	6.33E-23	↓	Trichohyalin, partial [B. mutus]
ENSBTAP00000001896-D1	2.27	1.92E-12	↓	histamine H1 receptor isoform X1 [B. mutus]
ENSP00000317904-D1	2.27	2.02E-05	↓	glycogen [starch] synthase, muscle isoform X1 [B. mutus]
ENSP00000385899-D2	2.27	2.02E-05	↓	Protein sidekick-2, partial [B. mutus]
ENSP00000398366-D1	2.27	4.48E-18	↓	histone H4 transcription factor isoform X1 [Bison bison bison]
ENSP00000230859-D1	2.28	2.43E-22	↓	non-canonical poly(A) RNA polymerase PAPD7 isoform X1 [B. taurus]
ENSBTAP00000015459-D1	2.28	5.52E-40	↓	Serine/threonine-protein kinase 11 [B. mutus]
ENSP00000403673-D1	2.28	5.11E-26	↓	P3 protein, partial [B. mutus]
ENSBTAP00000003653-D1	2.28	5.41E-14	↓	Myelin protein zero-like protein 1, partial [B. mutus]
ENSP00000309644-D2	2.29	9.08E-15	↓	biorientation of chromosomes in cell division protein 1-like [Balaenoptera acutorostrata scammoni]
ENSP00000267202-D1	2.29	1.72E-10	↓	Vacuolar protein sorting-associated protein 37B, partial [B. mutus]
ENSP00000355536-D1	2.29	3.33E-06	↓	Methionine synthase, partial [B. mutus]
ENSP00000302251-D1	2.29	3.33E-06	↓	Homeobox protein GBX-2, partial [B. mutus]
ENSBTAP00000052456-D2	2.29	4.97E-04	↓	peroxisomal membrane protein 11A isoform X1 [B. mutus]
ENSP00000372348-D1	2.29	2.45E-28	↓	histone-lysine N-methyltransferase NSD2 isoform X1 [B. taurus]
ENSBTAP00000001861-D1	2.29	3.31E-26	↓	39S ribosomal protein L4, mitochondrial [B. mutus]

ENSBTAP00000033568-D1	2.29	3.61E-46	↓	ataxin-7-like protein 2 [B. taurus]
ENSP00000364476-D1	2.29	1.03E-47	↓	RIB43A-like with coiled-coils protein 1 [B. mutus]
ENSBTAP00000006746-D1	2.29	6.95E-55	↓	FK506-binding protein 15, partial [B. mutus]
ENSP00000322628-D1	2.29	3.96E-09	↓	ADP-ribosylation factor-like protein 4D [B. taurus]
ENSP00000245564-D1	2.30	4.81E-12	↓	protein misato homolog 1-like isoform X1 [B. mutus]
ENSBTAP00000009779-D1	2.30	5.50E-07	↓	tektin-1-like [B. mutus]
ENSP00000308258-D1	2.30	5.50E-07	↓	Phosphatidylserine synthase 2, partial [B. mutus]
ENSP00000320147-D1	2.30	8.47E-70	↓	histone-lysine N-methyltransferase EZH2 isoform X1 [Bison bison bison]
ENSP00000356623-D1	2.30	7.27E-18	↓	cbp/p300-interacting transactivator 2 [B. mutus]
ENSBTAP00000035660-D1	2.30	1.67E-16	↓	Signal peptide peptidase-like 2B, partial [B. mutus]
ENSP00000311984-D1	2.30	9.15E-08	↓	ATP-dependent (S)-NAD(P)H-hydrate dehydratase isoform X5 [Ovis aries musimon]
ENSP00000353806-D1	2.30	9.15E-08	↓	core histone macro-H2A.1 isoform X1 [B. taurus]
ENSBTAP00000009438-D1	2.30	9.15E-08	↓	Putative sodium-coupled neutral amino acid transporter 7, partial [B. mutus]
ENSP00000294543-D1	2.30	8.00E-05	↓	Transmembrane and coiled-coil domain-containing protein 4, partial [B. mutus]
ENSBTAP00000026497-D1	2.30	8.00E-05	↓	Protein HIRA, partial [B. mutus]
ENSBTAP00000041206-D1	2.30	8.00E-05	↓	Myosin-binding protein C, slow-type, partial [B. mutus]
ENSBTAP00000042582-D1	2.30	8.00E-05	↓	TPA: transmembrane protein 119 [B. taurus]
ENSBTAP00000021939-D1	2.31	1.40E-32	↓	Transmembrane channel-like protein 7, partial [B. mutus]
ENSP00000216101-D1	2.31	1.53E-08	↓	ras-like protein family member 10A [B. mutus]
ENSBTAP00000004948-D1	2.31	3.11E-12	↓	Threonyl-tRNA synthetase, mitochondrial [B. mutus]
ENSBTAP00000007890-D1	2.32	5.24E-13	↓	endophilin-A2 isoform X2 [B. mutus]
ENSP00000264712-D1	2.32	2.93E-54	↓	kinesin-like protein KIF3C [B. mutus]
ENSP00000328854-D1	2.32	2.16E-06	↓	nucleolar complex protein 4 homolog [B. mutus]
ENSP00000369071-D1	2.32	7.89E-43	↓	periostin isoform X1 [B. taurus]
ENSP00000376838-D1	2.33	6.97E-17	↓	Transmembrane protein 108, partial [B. mutus]
ENSBTAP00000003424-D1	2.33	1.17E-17	↓	probable small intestine urate exporter isoform X1 [B. taurus]
ENSBTAP00000046715-D1	2.33	1.58E-27	↓	BEN domain-containing protein 4, partial [B. mutus]
ENSP00000268676-D1	2.34	1.65E-09	↓	Differentially expressed in FDCP 8-like protein, partial [B. mutus]
ENSBTAP00000027245-D1	2.34	2.14E-25	↓	zinc finger protein 398 [B. taurus]
ENSP00000262764-D1	2.34	3.60E-26	↓	CDP-diacylglycerol--glycerol-3-phosphate 3-phosphatidyltransferase, mitochondrial, partial [B. mutus]
ENSP00000361823-D1	2.34	1.72E-28	↓	Protein BHLHb9, partial [B. mutus]
ENSBTAP00000020995-D1	2.34	1.38E-37	↓	Zinc finger protein 609, partial [B. mutus]
ENSBTAP00000053387-D1	2.34	4.65E-11	↓	Zinc finger protein 335, partial [B. mutus]
ENSBTAP00000008203-D1	2.35	1.73E-16	↓	protein CASC3 [B. mutus]
ENSBTAP00000004413-D1	2.35	1.87E-23	↓	Quinone oxidoreductase PIG3, partial [B. mutus]
ENSBTAP00000027964-D1	2.35	1.50E-26	↓	RuvB-like 1, partial [B. mutus]
ENSBTAP00000048313-D1	2.35	2.53E-27	↓	Tripartite motif-containing protein 26 [B. mutus]
ENSBTAP00000023854-D1	2.36	1.45E-61	↓	Nuclear envelope pore membrane protein POM 121C, partial [B. mutus]
ENSP00000409936-D1	2.36	1.41E-13	↓	unconventional myosin-XIX [B. mutus]
ENSP00000374989-D1	2.36	4.99E-12	↓	Ig alpha-1 chain C region, partial [B. mutus]
ENSBTAP00000008149-D1	2.36	2.99E-11	↓	probable Xaa-Pro aminopeptidase 3 [B. mutus]
ENSBTAP00000004002-D1	2.36	1.07E-09	↓	segment polarity protein dishevelled homolog DVL-2 isoform X2 [B. mutus]
ENSP00000376860-D1	2.36	6.38E-09	↓	protein unc-79 homolog isoform X7 [Ovis aries musimon]
ENSBTAP00000011392-D1	2.36	3.80E-08	↓	protein FAM110A [B. mutus]
ENSBTAP00000009888-D1	2.36	2.28E-07	↓	group XV phospholipase A2 [B. mutus]
ENSBTAP00000006601-D1	2.36	2.28E-07	↓	Sideroflexin-3, partial [B. mutus]
ENSBTAP00000014152-D1	2.36	1.38E-06	↓	Segment polarity protein dishevelled-like protein DVL-1, partial [B. mutus]
ENSP00000416177-D1	2.36	1.38E-06	↓	Putative E3 ubiquitin-protein ligase MARCH10, partial [B. mutus]
ENSBTAP00000021752-D1	2.36	8.36E-06	↓	Transcriptional enhancer factor TEF-4 [B. mutus]
ENSBTAP00000031108-D1	2.36	5.13E-05	↓	TPA: E3 ubiquitin-protein ligase RNF128 precursor [B. taurus]
ENSBTAP00000007668-D1	2.36	3.17E-04	↓	Striatin-4, partial [B. mutus]
ENSP00000298854-D1	2.36	3.17E-04	↓	43 kDa receptor-associated protein of the synapse isoform X1 [B. mutus]
ENSP00000299853-D1	2.36	2.01E-03	↓	DNA-directed RNA polymerase III subunit RPC5 isoform X1 [B. mutus]
ENSBTAP00000053780-D1	2.36	2.01E-03	↓	Dedicator of cytokinesis protein 3, partial [B. mutus]
ENSBTAP00000051012-D2	2.36	2.01E-03	↓	60S ribosomal protein L21-like [B. mutus]

ENSP00000383234-D1	2.36	2.01E-03	↓	Mitogen-activated protein kinase 4 [B. mutus]
ENSP00000395583-D40	2.36	1.34E-02	↓	hypothetical protein M91_04029 [B. mutus]
ENSP00000381086-D1	2.36	1.34E-02	↓	TPA: hypothetical protein LOC617391 precursor [B. taurus]
ENSBTAP00000008153-D1	2.36	1.34E-02	↓	Adenylate cyclase type 7 [B. mutus]
ENSP00000322594-D2	2.36	1.34E-02	↓	ras-related protein Rab-39B [B. taurus]
ENSP00000310594-D1	2.36	1.34E-02	↓	fibronectin type III domain-containing protein 9 isoform X1 [B. taurus]
ENSBTAP00000047831-D29	2.36	1.34E-02	↓	Olfactory receptor 6, partial [B. mutus]
ENSP00000417517-D1	2.36	1.34E-02	↓	toll-like receptor 9 [B. mutus]
ENSP00000315635-D1	2.36	1.34E-02	↓	Transmembrane protein 25 [B. mutus]
ENSBTAP00000045884-D1	2.36	1.34E-02	↓	Betaine--homocysteine S-methyltransferase 2, partial [B. mutus]
ENSP00000407619-D3	2.36	1.34E-02	↓	prohibitin isoform X1 [Tupaia chinensis]
ENSBTAP00000053677-D1	2.36	1.34E-02	↓	Contactin-4, partial [B. mutus]
yakG032823	2.36	1.34E-02	↓	hypothetical protein M91_08195 [B. mutus]
ENSP00000419718-D1	2.36	1.34E-02	↓	hypothetical protein M91_00021, partial [B. mutus]
ENSP00000313699-D1	2.36	1.34E-02	↓	Hephaestin-like protein 1 [B. mutus]
ENSP00000354652-D1	2.36	1.34E-02	↓	neuropeptide Y receptor type 1 [B. mutus]
ENSBTAP00000025041-D1	2.36	1.34E-02	↓	transmembrane channel-like protein 2 [B. taurus]
yakA07029	2.36	1.34E-02	↓	putative uncharacterized protein TRPC5OS [B. taurus]
ENSP00000408288-D1	2.36	1.34E-02	↓	unnamed protein product [Homo sapiens]
ENSBTAP00000042009-D10	2.36	1.34E-02	↓	olfactory receptor 287-like [B. mutus]
ENSBTAP00000039906-D3	2.36	1.34E-02	↓	epididymis-specific alpha-mannosidase-like, partial [B. mutus]
ENSBTAP00000002513-D1	2.36	1.05E-27	↓	Carnitine O-acetyltransferase [B. mutus]
ENSP00000310180-D1	2.37	1.10E-63	↓	Transmembrane protein 110 [B. mutus]
ENSP00000323511-D1	2.37	2.03E-18	↓	bifunctional polynucleotide phosphatase/kinase [B. taurus]
ENSBTAP00000012932-D1	2.37	1.14E-28	↓	inversin [B. mutus]
ENSP00000263373-D3	2.37	6.37E-39	↓	spectrin beta chain, erythrocytic [B. mutus]
ENSBTAP00000008416-D1	2.37	1.76E-22	↓	Enhancer of mRNA-decapping protein 4 [B. mutus]
ENSP00000391753-D1	2.37	3.21E-12	↓	Myeloid differentiation primary response protein MyD88, partial [B. mutus]
ENSP00000315713-D1	2.37	2.08E-30	↓	Protein phosphatase Slingshot-like protein 1, partial [B. mutus]
ENSP00000352995-D1	2.38	1.52E-61	↓	TPA: Rho guanine nucleotide exchange factor 18-like [B. taurus]
ENSBTAP00000012611-D1	2.38	8.12E-42	↓	M-phase inducer phosphatase 1, partial [B. mutus]
ENSBTAP00000018959-D1	2.38	2.45E-08	↓	Scavenger receptor class B member 1 [B. mutus]
ENSBTAP00000013006-D1	2.38	2.37E-20	↓	Fibroblast growth factor receptor substrate 3 [B. mutus]
ENSBTAP00000008112-D1	2.39	1.23E-23	↓	Zinc finger protein 76 [B. mutus]
ENSBTAP00000036097-D1	2.39	1.58E-109	↓	Tetratricopeptide repeat protein 5, partial [B. mutus]
ENSBTAP00000007623-D1	2.39	2.06E-12	↓	E3 ubiquitin-protein ligase TRIM50 isoform X1 [B. mutus]
ENSBTAP00000018791-D1	2.39	6.37E-27	↓	WD repeat-containing protein 17 isoform X2 [B. taurus]
ENSBTAP00000027209-D1	2.39	2.55E-21	↓	death-associated protein kinase 3 [B. mutus]
ENSP00000210187-D1	2.39	8.85E-07	↓	Ras-related protein Rab-26, partial [B. mutus]
ENSBTAP00000015162-D1	2.40	5.36E-06	↓	B-cell differentiation antigen CD72 [B. mutus]
ENSP00000352933-D1	2.40	1.64E-21	↓	Sodium- and chloride-dependent creatine transporter 1, partial [B. mutus]
ENSBTAP00000018227-D1	2.40	1.56E-61	↓	protein FAM5B [B. mutus]
ENSBTAP00000020600-D1	2.40	2.63E-09	↓	Protein fat-free-like protein, partial [B. mutus]
ENSBTAP00000007896-D1	2.40	6.73E-16	↓	stabilin-2 [B. mutus]
ENSBTAP00000029240-D1	2.41	1.79E-22	↓	arachidonate 12-lipoxygenase, 12S-type [B. mutus]
ENSP00000262518-D1	2.41	3.39E-83	↓	hypothetical protein PANDA_014226, partial [Ailuropoda melanoleuca]
ENSBTAP00000005681-D1	2.41	2.48E-32	↓	coiled-coil domain-containing protein 87 [B. mutus]
ENSP00000261650-D1	2.41	2.74E-92	↓	RNA demethylase ALKBH5 isoform X2 [Ovis aries musimon]
ENSBTAP00000016859-D1	2.41	2.85E-28	↓	zinc finger protein 454 [B. mutus]
ENSBTAP00000026200-D1	2.41	1.57E-08	↓	AT-rich interactive domain-containing protein 5A, partial [B. mutus]
ENSP00000384209-D1	2.41	9.39E-08	↓	Grainyhead-like protein 1, partial [B. mutus]
ENSBTAP00000025438-D1	2.42	2.57E-15	↓	Growth arrest-specific protein 7, partial [B. mutus]
ENSBTAP00000017416-D3	2.42	2.01E-04	↓	Fructose-bisphosphate aldolase B, partial [B. mutus]
ENSP00000319817-D1	2.42	2.01E-04	↓	Optic atrophy 3 protein-like protein, partial [B. mutus]
ENSP00000408914-D1	2.42	2.01E-04	↓	RNA-binding protein Nova-1, partial [Buceros rhinoceros silvestris]

ENSBTAP00000025702-D2	2.43	1.37E-30	↓	transcription factor MafG isoform X2 [Cavia porcellus]
ENSP00000354361-D1	2.43	4.46E-28	↓	E3 ubiquitin-protein ligase RFWD3 [B. mutus]
ENSP00000379078-D1	2.43	1.45E-25	↓	Histone-lysine N-methyltransferase, H3 lysine-9 specific 3 [B. mutus]
ENSBTAP00000023476-D1	2.43	3.18E-82	↓	Sex comb on midleg-like protein 1, partial [B. mutus]
ENSP00000308957-D1	2.43	8.33E-42	↓	Multiple C2 and transmembrane domain-containing protein 1, partial [B. mutus]
ENSBTAP00000000429-D2	2.44	8.60E-167	↓	hypothetical protein M91_14802, partial [B. mutus]
ENSBTAP00000043718-D1	2.44	1.81E-10	↓	homeodomain leucine zipper protein [B. taurus]
ENSBTAP00000011048-D1	2.44	3.42E-06	↓	amyloid beta A4 precursor protein-binding family A member 3 [B. mutus]
ENSBTAP00000018573-D1	2.44	3.42E-06	↓	low-density lipoprotein receptor-related protein 1B isoform X1, partial [B. taurus]
ENSBTAP00000009921-D1	2.44	5.54E-25	↓	peroxisomal biogenesis factor 19 isoform X1 [B. mutus]
yakA05610	2.44	3.24E-12	↓	adenylate kinase 2, mitochondrial-like [B. mutus]
ENSBTAP00000014447-D1	2.44	6.73E-116	↓	homeobox protein MSX-1 [B. mutus]
ENSBTAP00000008080-D1	2.44	6.00E-08	↓	WD repeat-containing protein 93 [B. mutus]
ENSBTAP00000024840-D1	2.44	1.10E-33	↓	RNA 3'-terminal phosphate cyclase-like protein [B. mutus]
ENSBTAP00000025033-D1	2.45	1.94E-11	↓	cadherin EGF LAG seven-pass G-type receptor 2 [B. mutus]
ENSP00000310219-D1	2.45	1.27E-35	↓	heat shock 70 kDa protein 6-like [B. mutus]
ENSP00000264895-D1	2.46	6.44E-09	↓	extracellular matrix protein FRAS1 [B. mutus]
ENSP00000381854-D1	2.46	6.44E-09	↓	Tensin-3 [B. mutus]
ENSBTAP00000028052-D1	2.46	2.07E-05	↓	Transmembrane protein 180, partial [B. mutus]
ENSBTAP00000003278-D1	2.46	2.07E-05	↓	zinc finger SWIM domain-containing protein 4 isoform X1 [B. taurus]
ENSBTAP00000040663-D1	2.46	2.07E-05	↓	ATP-binding cassette sub-family C member 9 [B. mutus]
ENSP00000340989-D1	2.46	2.07E-05	↓	14-3-3 protein sigma [Ovis aries]
ENSBTAP00000014402-D1	2.46	2.07E-05	↓	SERTA domain-containing protein 4 [B. mutus]
ENSBTAP00000037291-D1	2.46	1.26E-03	↓	ectoderm-neural cortex protein 1 isoform X1 [Odobenus rosmarus divergens]
ENSBTAP00000010294-D1	2.47	1.24E-11	↓	REST corepressor 2 [Bison bison bison]
ENSBTAP00000012550-D1	2.47	1.09E-33	↓	Proliferation-associated protein 2G4 [Pteropus alecto]
ENSP00000254101-D1	2.47	2.38E-14	↓	5'-AMP-activated protein kinase subunit beta-2 [B. taurus]
ENSP00000402760-D1	2.47	5.90E-26	↓	Solute carrier family 22 member 5 [B. mutus]
ENSP00000333711-D2	2.48	2.55E-15	↓	hypothetical protein M91_15279 [B. mutus]
ENSP00000377298-D1	2.48	2.18E-06	↓	Nuclear mitotic apparatus protein 1, partial [B. mutus]
ENSBTAP00000000263-D1	2.48	2.18E-06	↓	glutamate receptor ionotropic, NMDA 2B [B. mutus]
ENSP00000300176-D1	2.48	4.10E-09	↓	Arf-GAP domain and FG repeats-containing protein 2 [B. mutus]
ENSP00000225688-D1	2.48	5.37E-19	↓	dexamethasone-induced Ras-related protein 1 [B. taurus]
ENSP00000228284-D1	2.48	2.07E-24	↓	Squamous cell carcinoma antigen recognized by T-cells 3 [B. mutus]
ENSBTAP00000027076-D3	2.48	1.52E-14	↓	AP-1 complex subunit beta-1 [B. mutus]
ENSP00000268699-D1	2.49	2.29E-07	↓	growth arrest-specific protein 8 [B. mutus]
ENSBTAP00000022042-D2	2.49	1.28E-04	↓	hypothetical protein M91_21237, partial [B. mutus]
ENSBTAP00000048264-D1	2.49	1.28E-04	↓	small integral membrane protein 17 isoform X1 [B. taurus]
ENSP00000328403-D1	2.49	1.28E-04	↓	mitochondrial dicarboxylate carrier [B. taurus]
ENSP00000388548-D1	2.49	1.28E-04	↓	Cbp/p300-interacting transactivator 1, partial [B. mutus]
ENSBTAP00000026769-D1	2.49	8.70E-43	↓	cullin-4A [B. taurus]
ENSBTAP00000003325-D1	2.49	1.62E-56	↓	G patch domain and KOW motifs-containing protein [B. mutus]
ENSP00000295101-D1	2.50	1.04E-15	↓	G protein-activated inward rectifier potassium channel 1 [Bison bison bison]
ENSP00000328216-D1	2.50	1.32E-05	↓	calcium release-activated calcium channel protein 1 [Bubalus bubalis]
ENSBTAP00000007184-D1	2.50	1.32E-05	↓	protein SOX-15 [B. mutus]
yakA09424	2.50	1.32E-05	↓	hypothetical protein M91_05661 [B. mutus]
ENSBTAP00000009480-D1	2.51	1.20E-17	↓	striatin-interacting protein 2 [Bison bison bison]
ENSBTAP00000019773-D1	2.51	1.20E-17	↓	G patch domain-containing protein 3 [B. mutus]
ENSBTAP00000009042-D1	2.51	2.72E-22	↓	protein FAM117A [B. mutus]
ENSBTAP00000001074-D1	2.51	6.22E-27	↓	Putative ATP-dependent RNA helicase DDX49, partial [B. mutus]
ENSBTAP00000016762-D1	2.51	6.61E-16	↓	Excitatory amino acid transporter 2, partial [B. mutus]
ENSP00000364310-D6	2.51	2.99E-11	↓	histone H2A.x [B. taurus]
ENSBTAP00000005623-D1	2.51	2.99E-11	↓	cell division control protein 45 homolog isoform X1 [B. mutus]
ENSBTAP00000001940-D1	2.51	1.38E-06	↓	Protein turtle-like protein A, partial [B. mutus]
ENSP00000268624-D1	2.51	1.38E-06	↓	adenosine deaminase domain-containing protein 2 isoform X2 [B. taurus]

ENSBTAP00000026124-D1	2.52	8.22E-19	↓	Retinoic acid-induced protein 3, partial [B. mutus]
ENSBTAP00000020078-D1	2.52	1.46E-07	↓	corticosteroid 11-beta-dehydrogenase isozyme 1 [B. taurus]
ENSBTAP00000027922-D1	2.52	0.00E+00	↓	striatin-interacting protein 1 [Bubalus bubalis]
ENSP00000362606-D2	2.53	1.11E-40	↓	Proto-oncogene serine/threonine-protein kinase pim-1, partial [B. mutus]
ENSBTAP00000024450-D1	2.53	1.90E-11	↓	Caytaxin, partial [B. mutus]
ENSBTAP00000021723-D1	2.54	2.31E-14	↓	protein rogdi homolog [Bison bison bison]
ENSBTAP00000027531-D1	2.54	1.25E-72	↓	Ligand of Numb protein X 2, partial [B. mutus]
ENSP00000375986-D1	2.54	1.58E-33	↓	TPA: MTK1/MEKK4 homolog family member (mtk-1)-like [B. taurus]
ENSBTAP00000020263-D3	2.54	2.12E-37	↓	Group XIIB secretory phospholipase A2-like protein, partial [B. mutus]
ENSBTAP00000019489-D1	2.54	8.07E-31	↓	Zinc finger homeobox protein 3, partial [B. mutus]
ENSBTAP00000011353-D1	2.54	1.78E-65	↓	Exocyst complex component 3, partial [B. mutus]
ENSP00000325612-D1	2.55	1.13E-10	↓	Mediator of RNA polymerase II transcription subunit 16, partial [B. mutus]
ENSBTAP00000002979-D1	2.55	1.13E-10	↓	chondroitin sulfate proteoglycan 5 precursor [B. taurus]
ENSBTAP00000025999-D1	2.55	9.85E-09	↓	zinc finger protein 513 [B. taurus]
ENSP00000322453-D8	2.55	9.23E-08	↓	Tripartite motif-containing protein 7 [B. mutus]
ENSP00000405932-D1	2.55	9.23E-08	↓	Prospero homeobox protein 2, partial [B. mutus]
ENSBTAP00000019400-D1	2.55	8.73E-07	↓	collagen alpha-5(IV) chain-like, partial [Tursiops truncatus]
ENSBTAP00000010854-D1	2.55	8.73E-07	↓	Glypican-6, partial [B. mutus]
ENSBTAP00000006727-D1	2.55	8.31E-06	↓	platelet-activating factor acetylhydrolase 2, cytoplasmic isoform X1 [B. mutus]
ENSBTAP00000027070-D1	2.55	8.31E-06	↓	ubiquitin carboxyl-terminal hydrolase 20 [B. mutus]
ENSP00000160740-D1	2.55	8.31E-06	↓	Protein capicua-like protein, partial [B. mutus]
ENSBTAP00000021234-D1	2.55	8.03E-05	↓	acyl-CoA synthetase family member 3, mitochondrial [B. mutus]
ENSBTAP00000000314-D1	2.55	8.03E-05	↓	haloacid dehalogenase-like hydrolase domain-containing protein 3 [B. mutus]
ENSBTAP00000012081-D1	2.55	7.90E-04	↓	Phosphatidate phosphatase LPIN3 [B. mutus]
ENSBTAP00000001782-D1	2.55	7.90E-04	↓	Mitochondrial-processing peptidase subunit alpha, partial [B. mutus]
ENSP00000399588-D1	2.55	7.90E-04	↓	GRB2-associated-binding protein 3 isoform X1 [B. mutus]
ENSP00000351379-D1	2.55	7.90E-04	↓	profilin-3 [B. taurus]
ENSBTAP00000017140-D1	2.55	7.90E-04	↓	keratin, type I cytoskeletal 10 isoform X2 [B. taurus]
ENSBTAP00000049946-D17	2.55	7.90E-04	↓	hypothetical protein M91_08673 [B. mutus]
ENSBTAP00000041768-D1	2.55	8.24E-03	↓	regulator of G-protein signaling protein-like [B. mutus]
ENSBTAP00000045695-D1	2.55	8.24E-03	↓	hypothetical protein M91_09397, partial [B. mutus]
ENSP00000308725-D1	2.55	8.24E-03	↓	Gamma-aminobutyric acid receptor subunit beta-3, partial [B. mutus]
ENSBTAP00000018933-D1	2.55	8.24E-03	↓	Protein FAM83F, partial [B. mutus]
yakG045875	2.55	8.24E-03	↓	hypothetical protein M91_11329 [B. mutus]
ENSBTAP00000039071-D1	2.55	8.24E-03	↓	Dual specificity protein phosphatase 22, partial [B. mutus]
ENSP00000299641-D1	2.55	8.24E-03	↓	bifunctional heparan sulfate N-deacetylase/N-sulfotransferase 2 [B. taurus]
ENSBTAP00000052062-D1	2.55	8.24E-03	↓	hypothetical protein M91_08288, partial [B. mutus]
ENSP00000417383-D1	2.55	8.24E-03	↓	TPA: paraneoplastic antigen Ma2 homolog [B. taurus]
ENSBTAP00000041130-D1	2.55	8.24E-03	↓	Transmembrane protein adipocyte-associated 1, partial [B. mutus]
ENSP00000355153-D1	2.55	8.24E-03	↓	Cyclin-dependent kinase inhibitor 2A, isoform 4, partial [B. mutus]
ENSP00000331462-D1	2.55	8.24E-03	↓	zinc finger protein 704 [Bison bison bison]
ENSP00000300101-D1	2.57	1.07E-53	↓	zinc finger and BTB domain-containing protein 39 [B. mutus]
ENSP00000379915-D1	2.58	6.25E-09	↓	Atrophin-1, partial [B. mutus]
ENSBTAP00000002156-D1	2.58	6.25E-09	↓	protein AAR2 homolog isoform X1 [B. taurus]
ENSBTAP00000014779-D1	2.58	5.75E-21	↓	Myosin-IXb, partial [B. mutus]
ENSBTAP00000020613-D33	2.58	5.83E-08	↓	hypothetical protein M91_04723, partial [B. mutus]
ENSP00000372295-D1	2.58	5.83E-08	↓	gap junction beta-2 protein [B. mutus]
ENSBTAP00000028921-D1	2.59	5.50E-07	↓	Nuclear receptor ROR-beta, partial [B. mutus]
ENSBTAP00000026911-D1	2.59	4.52E-11	↓	Zinc finger protein 263, partial [B. mutus]
ENSP00000388743-D1	2.59	2.47E-28	↓	SUN domain-containing protein 1, partial [B. mutus]
ENSBTAP00000020514-D1	2.59	3.48E-14	↓	hypothetical protein M91_13526, partial [B. mutus]
ENSP00000336775-D1	2.59	3.48E-14	↓	Synemin, partial [B. mutus]
ENSP00000379406-D1	2.59	5.24E-06	↓	Phosphatidate phosphatase LPIN1, partial [B. mutus]
ENSP00000264555-D1	2.60	6.02E-47	↓	PHD and RING finger domain-containing protein 1 [B. mutus]
ENSP00000377027-D1	2.60	3.94E-09	↓	transforming growth factor-beta receptor-associated protein 1 [B. mutus]

ENSP00000373340-D1	2.61	7.34E-63	↓	Peregrin [B. mutus]
ENSP00000362294-D1	2.61	3.56E-40	↓	TPA: protein tyrosine phosphatase, receptor type, T-like [B. taurus]
ENSBTAP00000017198-D1	2.61	2.20E-14	↓	TPA: ubiquitin specific protease 35-like [B. taurus]
ENSBTAP00000037474-D1	2.61	2.85E-11	↓	lethal(3)malignant brain tumor-like protein 2 [B. taurus]
ENSBTAP00000027193-D1	2.61	5.04E-05	↓	formimidoyltransferase-cyclodeaminase [B. mutus]
ENSP00000342411-D1	2.61	5.04E-05	↓	neuritin-like protein [B. taurus]
ENSBTAP00000043629-D1	2.61	5.04E-05	↓	Taste receptor cell protein 1, partial [B. mutus]
ENSBTAP00000023373-D1	2.61	6.26E-29	↓	prelamin-A/C isoform X1 [B. mutus]
ENSP00000359337-D1	2.61	1.22E-76	↓	gamma-aminobutyric acid receptor subunit alpha-3 isoform X1 [B. mutus]
ENSBTAP00000048101-D1	2.61	9.99E-23	↓	chemokine (C-X-C motif) receptor 2 [B. mutus]
ENSP00000368119-D1	2.61	1.26E-19	↓	Galactose-1-phosphate uridylyltransferase, partial [B. mutus]
ENSP00000332258-D1	2.62	3.46E-07	↓	Diacylglycerol O-acyltransferase 1, partial [B. mutus]
ENSP00000262919-D1	2.62	5.21E-46	↓	attractin, partial [B. mutus]
ENSP00000337226-D1	2.63	4.30E-24	↓	Cell division cycle-associated protein 4 [B. mutus]
ENSP00000332204-D1	2.63	2.49E-09	↓	semaphorin-4B [B. mutus]
ENSBTAP00000025981-D1	2.63	1.33E-33	↓	Alpha-1D adrenergic receptor [B. mutus]
ENSBTAP00000013366-D1	2.63	6.24E-155	↓	Protein CBFA2T2, partial [B. mutus]
ENSP00000402457-D1	2.63	1.46E-67	↓	GRAM domain-containing protein 1B, partial [B. mutus]
ENSBTAP00000003297-D1	2.63	5.02E-20	↓	TRIO and F-actin-binding protein, partial [B. mutus]
ENSP00000299466-D1	2.64	3.89E-36	↓	Sal-like protein 3, partial [B. mutus]
ENSP00000368405-D1	2.64	6.40E-17	↓	hypothetical protein M91_16071, partial [B. mutus]
ENSP00000359563-D1	2.64	1.68E-10	↓	cerebellar degeneration-related antigen 1 [Pantholops hodgsonii]
ENSP00000381597-D1	2.64	2.32E-08	↓	Putative G-protein coupled receptor 179 [B. mutus]
ENSP00000396157-D3	2.64	2.32E-08	↓	sialic acid-binding Ig-like lectin 8 [B. mutus]
ENSBTAP00000020845-D1	2.64	3.30E-06	↓	FYVE and coiled-coil domain-containing protein 1, partial [B. mutus]
ENSBTAP00000021516-D1	2.64	3.30E-06	↓	keratin, type II cytoskeletal 7 [B. mutus]
ENSP00000386227-D1	2.64	3.30E-06	↓	Colorectal mutant cancer protein, partial [B. mutus]
ENSBTAP00000022042-D1	2.64	4.94E-04	↓	Integrin alpha-9, partial [B. mutus]
ENSP00000297375-D1	2.64	4.94E-04	↓	Homeobox protein engrailed-2, partial [B. mutus]
yakG025883	2.64	4.94E-04	↓	hypothetical protein M91_15640 [B. mutus]
ENSBTAP00000008640-D1	2.64	4.94E-04	↓	sperm flagellar protein 1 [B. mutus]
ENSBTAP00000012863-D1	2.64	3.29E-175	↓	Golgin subfamily A member 3 [B. mutus]
ENSBTAP00000002136-D1	2.64	4.34E-18	↓	Kinesin-like protein KIFC1, partial [B. mutus]
ENSBTAP00000028723-D1	2.64	6.24E-30	↓	Protein LSM14-like protein B, partial [B. mutus]
ENSP00000337452-D1	2.64	5.93E-16	↓	magnesium transporter NIPA1 [B. taurus]
ENSP00000361949-D2	2.65	2.14E-21	↓	Polyadenylate-binding protein 1-like protein, partial [B. mutus]
ENSP00000352040-D1	2.65	7.90E-27	↓	hypothetical protein M91_18803, partial [B. mutus]
ENSBTAP00000032574-D1	2.65	2.18E-07	↓	Protein VAC14-like protein [B. mutus]
ENSP00000296327-D1	2.66	1.06E-10	↓	organic solute transporter subunit alpha isoform X1 [B. taurus]
ENSP00000215730-D1	2.67	1.46E-08	↓	Synaptosomal-associated protein 29, partial [B. mutus]
ENSP00000420608-D1	2.67	3.16E-05	↓	Putative protein KIAA0090 [B. mutus]
ENSBTAP00000053093-D1	2.67	3.16E-05	↓	zinc finger and BTB domain-containing protein 17 isoform X1 [B. taurus]
ENSBTAP00000027416-D1	2.67	3.16E-05	↓	Signal peptide, CUB and EGF-like domain-containing protein 2, partial [B. mutus]
ENSBTAP00000018572-D1	2.68	2.07E-06	↓	Phosphatidylinositol 4-kinase alpha, partial [B. mutus]
ENSP00000413073-D1	2.68	1.74E-53	↓	zinc finger protein 445 [B. mutus]
ENSBTAP00000041513-D1	2.68	4.45E-40	↓	transcription cofactor vestigial-like protein 4 [B. taurus]
ENSBTAP00000042087-D1	2.69	1.37E-07	↓	UPF0669 protein C6orf120 homolog [Bubalus bubalis]
ENSP00000363241-D1	2.69	1.37E-15	↓	Zinc finger protein 618, partial [B. mutus]
ENSP00000363136-D1	2.69	9.18E-09	↓	G-protein coupled receptor 3, partial [B. mutus]
ENSBTAP00000015161-D1	2.70	6.17E-10	↓	dual specificity testis-specific protein kinase 1 [B. mutus]
ENSP00000301180-D1	2.70	2.51E-53	↓	disco-interacting protein 2 homolog B, partial [B. mutus]
ENSP00000392490-D1	2.70	4.17E-11	↓	Fibrocystin-L [B. mutus]
ENSBTAP00000053560-D1	2.71	0.00E+00	↓	Nuclear factor, partial [B. mutus]
ENSBTAP00000019510-D1	2.72	3.87E-10	↓	Protein spinster-like protein 1, partial [B. mutus]
ENSP00000263578-D1	2.72	3.06E-04	↓	FAD-dependent oxidoreductase domain-containing protein 1, partial [B. mutus]

ENSP00000332163-D2	2.72	3.06E-04	↓	Small proline-rich protein 4 [B. mutus]
ENSP00000402861-D1	2.72	3.06E-04	↓	Inactive phospholipase C-like protein 1, partial [B. mutus]
ENSBTAP00000049268-D3	2.72	3.06E-04	↓	hypothetical protein M91_09555, partial [B. mutus]
ENSBTAP00000009942-D1	2.72	5.03E-03	↓	Dual specificity tyrosine-phosphorylation-regulated kinase 4 [B. mutus]
ENSP00000385437-D1	2.72	5.03E-03	↓	transcription factor MafK [B. taurus]
ENSP00000398817-D1	2.72	5.03E-03	↓	uncharacterized protein LOC105082221 [Camelus bactrianus]
ENSP00000342993-D2	2.72	5.03E-03	↓	Synapsin-1, partial [B. mutus]
ENSBTAP00000002386-D1	2.72	5.03E-03	↓	Transcription factor Sp6 [B. mutus]
ENSP00000403636-D1	2.72	5.03E-03	↓	ras/Rap GTPase-activating protein SynGAP [B. taurus]
ENSP00000164133-D1	2.72	5.03E-03	↓	serine/threonine-protein phosphatase 2A 56 kDa regulatory subunit beta isoform [B. taurus]
ENSBTAP00000044054-D1	2.72	5.03E-03	↓	SKI family transcriptional corepressor 2, partial [B. mutus]
ENSBTAP00000020226-D1	2.72	5.03E-03	↓	l-2-hydroxyglutarate dehydrogenase, mitochondrial [B. mutus]
ENSP00000316518-D30	2.72	5.03E-03	↓	olfactory receptor 6C74-like [B. mutus]
ENSP00000228945-D1	2.72	5.03E-03	↓	rho GDP-dissociation inhibitor 2 [B. taurus]
ENSP00000375745-D1	2.73	1.56E-18	↓	Presenilin-2, partial [B. mutus]
ENSP00000366918-D1	2.75	3.60E-09	↓	protein FAM220A [Bubalus bubalis]
ENSP00000330808-D1	2.75	5.34E-08	↓	otolin-1 [B. mutus]
yakG015168	2.76	8.05E-07	↓	hypothetical protein M91_00881 [B. mutus]
ENSP00000401466-D1	2.76	8.05E-07	↓	Tripartite motif-containing protein 66, partial [B. mutus]
ENSBTAP00000003345-D1	2.76	8.05E-07	↓	monocarboxylate transporter 8 [Bubalus bubalis]
ENSP00000376827-D1	2.76	3.38E-64	↓	Meckel syndrome type 1 protein [B. mutus]
ENSP00000328083-D1	2.76	9.98E-28	↓	rho guanine nucleotide exchange factor 37 [B. mutus]
ENSBTAP00000005861-D1	2.77	1.09E-21	↓	hypothetical protein M91_07977, partial [B. mutus]
ENSBTAP00000010191-D1	2.77	2.16E-25	↓	NF-kappa-B inhibitor-like protein 2 [B. mutus]
ENSBTAP00000021634-D1	2.77	1.22E-05	↓	Peptidyl-prolyl cis-trans isomerase FKBP8 [B. mutus]
ENSP00000362187-D1	2.77	1.22E-05	↓	protein FAM102A [B. mutus]
ENSP00000417980-D1	2.78	3.28E-49	↓	Histone-lysine N-methyltransferase, H3 lysine-9 specific 5, partial [B. mutus]
ENSBTAP00000040882-D1	2.78	3.33E-08	↓	uncharacterized protein KIAA1522 homolog [B. mutus]
ENSBTAP00000007855-D2	2.78	3.33E-08	↓	disheveled-associated activator of morphogenesis 2 [B. mutus]
ENSP00000367384-D1	2.79	9.48E-11	↓	probable G-protein coupled receptor 34 [B. taurus]
ENSBTAP00000052206-D1	2.79	7.62E-16	↓	retinol dehydrogenase 12-like [B. mutus]
ENSP00000307041-D1	2.80	6.45E-30	↓	Zinc finger protein basonuclin-1, partial [B. mutus]
ENSP00000381430-D1	2.80	4.99E-07	↓	MAGUK p55 subfamily member 3, partial [B. mutus]
ENSBTAP00000015532-D1	2.80	1.89E-04	↓	Non-receptor tyrosine-protein kinase TYK2, partial [B. mutus]
ENSBTAP00000031486-D18	2.80	1.89E-04	↓	hypothetical protein M91_05519, partial [B. mutus]
ENSP00000358551-D1	2.80	1.89E-04	↓	AMP deaminase 1 isoform X2 [B. mutus]
ENSBTAP00000050640-D1	2.80	1.89E-04	↓	E3 ubiquitin-protein ligase NEURL3 [B. mutus]
ENSP00000359211-D1	2.80	1.89E-04	↓	Dihydropyrimidine dehydrogenase [NADP+], partial [B. mutus]
ENSP00000365840-D1	2.80	1.14E-23	↓	ectonucleoside triphosphate diphosphohydrolase 6 [B. mutus]
ENSP00000361180-D1	2.80	2.78E-40	↓	general transcription factor 3C polypeptide 5 [B. mutus]
ENSBTAP00000040177-D1	2.81	1.68E-13	↓	Axin-2 [B. mutus]
ENSBTAP00000015608-D1	2.81	7.04E-15	↓	Williams-Beuren syndrome chromosomal region 16 protein-like protein, partial [B. mutus]
ENSP00000408903-D1	2.81	2.08E-08	↓	Immunoglobulin superfamily member 2, partial [B. mutus]
ENSBTAP00000049323-D1	2.82	1.95E-43	↓	P2Y purinoceptor 3-like [Bison bison bison]
ENSP00000388613-D1	2.82	3.43E-37	↓	gamma-parvin [B. mutus]
ENSP00000375413-D3	2.82	8.73E-10	↓	hypothetical protein M91_12914, partial [B. mutus]
ENSP00000363640-D1	2.82	7.57E-06	↓	PHD finger protein 1 isoform X1 [B. mutus]
ENSP00000361423-D1	2.83	9.02E-76	↓	Tyrosine-protein kinase ABL1, partial [B. mutus]
ENSBTAP00000028559-D1	2.83	2.58E-32	↓	tyrosine-protein kinase CSK [B. taurus]
ENSP00000373288-D1	2.84	1.54E-12	↓	biotinidase isoform X1 [B. mutus]
ENSP00000363277-D1	2.84	3.09E-07	↓	ribosomal protein S6 kinase alpha-1 isoform X1 [B. mutus]
ENSBTAP00000009222-D1	2.84	3.09E-07	↓	MAPK/MAK/MRK overlapping kinase, partial [B. mutus]
ENSBTAP00000037593-D1	2.84	6.48E-14	↓	endothelin-converting enzyme 1 [B. mutus]
ENSBTAP00000023771-D1	2.84	6.79E-34	↓	leucine-rich repeat-containing protein 38 isoform X1 [B. taurus]
ENSBTAP00000014893-D1	2.85	4.87E-18	↓	THAP domain-containing protein 3 [Bison bison bison]

ENSP00000273600-D1	2.85	5.43E-10	↓	Dynein heavy chain 1, axonemal [B. mutus]
ENSP00000372445-D1	2.85	5.43E-10	↓	methyltransferase-like protein 17, mitochondrial precursor [B. taurus]
ENSBTAP00000039132-D1	2.85	2.06E-19	↓	zinc finger protein 512B [B. mutus]
ENSP00000234739-D1	2.86	2.83E-26	↓	B-cell CLL/lymphoma 9 protein isoform X1 [Bison bison bison]
ENSP00000264670-D1	2.86	1.24E-38	↓	tRNA (cytosine(34)-C(5))-methyltransferase isoform X2 [B. taurus]
ENSP00000265056-D1	2.87	5.67E-32	↓	DNA replication licensing factor MCM2 isoform X2 [Ovis aries musimon]
ENSP00000320176-D1	2.87	8.05E-09	↓	hematopoietic lineage cell-specific protein [B. mutus]
ENSP00000341274-D1	2.87	1.92E-07	↓	hypothetical protein M91_09600, partial [B. mutus]
ENSBTAP00000037521-D1	2.87	1.92E-07	↓	85/88 kDa calcium-independent phospholipase A2 [B. mutus]
ENSBTAP00000050407-D4	2.87	1.92E-07	↓	Serine/threonine-protein phosphatase 6 regulatory ankyrin repeat subunit A, partial [B. mutus]
ENSBTAP00000014258-D1	2.87	4.68E-06	↓	tubulin polyglutamylase complex subunit 1, partial [B. mutus]
ENSP00000291182-D94	2.87	4.68E-06	↓	zinc finger protein OZF isoform X1 [B. taurus]
ENSBTAP00000026956-D1	2.87	1.17E-04	↓	Neurexin-2-alpha, partial [B. mutus]
ENSBTAP00000027649-D1	2.87	1.17E-04	↓	Argininosuccinate synthase, partial [B. mutus]
ENSBTAP00000046506-D1	2.87	3.06E-03	↓	Sphingomyelin phosphodiesterase 3, partial [B. mutus]
ENSP00000386419-D1	2.87	3.06E-03	↓	Mitotic-spindle organizing protein 2, partial [B. mutus]
ENSBTAP00000053502-D3	2.87	3.06E-03	↓	low-density lipoprotein receptor class A domain-containing protein 3 precursor [B. taurus]
ENSP00000397718-D1	2.87	3.06E-03	↓	hypothetical protein M91_04947, partial [B. mutus]
ENSP00000284311-D1	2.87	1.66E-02	↓	G-protein coupled receptor 15 [B. mutus]
ENSP00000280481-D1	2.87	1.66E-02	↓	FRAS1-related extracellular matrix protein 2 [B. mutus]
ENSBTAP00000047428-D2	2.87	1.66E-02	↓	hypothetical protein M91_08561, partial [B. mutus]
ENSP00000361382-D1	2.87	1.66E-02	↓	pulmonary surfactant-associated protein A-like isoform X1 [B. mutus]
ENSP00000267845-D1	2.87	1.66E-02	↓	histidine decarboxylase isoform X1 [B. mutus]
ENSBTAP00000020767-D1	2.87	1.66E-02	↓	TPA: interleukin 20 receptor, alpha [B. taurus]
ENSP00000220772-D1	2.87	1.66E-02	↓	secreted frizzled-related protein 1 [B. mutus]
ENSP00000361024-D1	2.87	1.66E-02	↓	Diencephalon/mesencephalon homeobox protein 1 [B. mutus]
ENSBTAP00000003550-D1	2.87	1.66E-02	↓	sphingosine 1-phosphate receptor 2 [B. taurus]
ENSBTAP00000004568-D1	2.87	1.66E-02	↓	Heat shock factor protein 4, partial [B. mutus]
ENSBTAP00000026467-D1	2.87	1.66E-02	↓	Mitogen-activated protein kinase 15 [B. mutus]
ENSP00000237449-D1	2.87	1.66E-02	↓	dynein heavy chain 6, axonemal-like, partial [B. mutus]
ENSBTAP00000027594-D1	2.87	1.66E-02	↓	neurotrypsin-like, partial [B. mutus]
ENSBTAP00000029297-D1	2.87	1.66E-02	↓	single-strand selective monofunctional uracil DNA glycosylase [Bison bison bison]
ENSP00000373669-D1	2.87	1.66E-02	↓	uncharacterized protein C17orf59 homolog [B. mutus]
ENSBTAP00000001065-D1	2.87	1.66E-02	↓	IQ domain-containing protein F1, partial [B. mutus]
ENSBTAP00000051802-D1	2.87	1.66E-02	↓	Magnesium transporter protein 1, partial [B. mutus]
ENSBTAP00000024243-D1	2.87	1.66E-02	↓	protein shisa-2 homolog [B. mutus]
ENSBTAP00000001784-D1	2.87	1.66E-02	↓	72 kDa inositol polyphosphate 5-phosphatase [B. mutus]
ENSBTAP00000044687-D1	2.87	1.66E-02	↓	hypothetical protein M91_02461, partial [B. mutus]
ENSBTAP00000050450-D1	2.87	1.66E-02	↓	hypothetical protein M91_02228, partial [B. mutus]
ENSBTAP00000053555-D1	2.87	1.66E-02	↓	Transcription factor Maf, partial [B. mutus]
ENSBTAP00000008409-D3	2.87	1.66E-02	↓	hypothetical protein M91_15210, partial [B. mutus]
ENSP00000327652-D1	2.87	1.66E-02	↓	D(1A) dopamine receptor [B. taurus]
ENSBTAP00000005893-D1	2.87	1.66E-02	↓	beta-1,4 N-acetylgalactosaminyltransferase 1 isoform X3 [B. taurus]
ENSP00000354777-D1	2.87	1.66E-02	↓	TANK-binding kinase 1-binding protein 1 [Bison bison bison]
ENSBTAP00000034490-D6	2.87	1.66E-02	↓	multidrug resistance-associated protein 4-like isoform X1 [B. taurus]
ENSBTAP00000003873-D1	2.87	1.66E-02	↓	dimethylaniline monooxygenase [N-oxide-forming] 2 [B. mutus]
ENSBTAP00000053536-D1	2.87	1.66E-02	↓	ALK tyrosine kinase receptor, partial [B. mutus]
ENSBTAP00000050009-D1	2.87	1.66E-02	↓	fibrous sheath-interacting protein 2-like isoform X2 [Ovis aries musimon]
ENSP00000315564-D1	2.87	1.66E-02	↓	galectin-9 isoform 1 [B. taurus]
ENSBTAP00000052592-D100	2.87	1.66E-02	↓	hypothetical protein M91_11495, partial [B. mutus]
ENSBTAP00000026552-D1	2.87	1.66E-02	↓	Williams-Beuren syndrome chromosomal region 27 protein, partial [B. mutus]
ENSBTAP00000012724-D1	2.87	1.66E-02	↓	Pleckstrin, partial [B. mutus]
ENSP00000385631-D1	2.87	1.66E-02	↓	small G protein signaling modulator 1 isoform X4 [Vicugna pacos]
ENSP00000315265-D1	2.87	1.66E-02	↓	dynactin-associated protein isoform X2 [B. taurus]
ENSP00000381580-D1	2.87	1.66E-02	↓	uncharacterized protein C17orf98 homolog [B. mutus]

ENSP00000411029-D1	2.87	1.66E-02	↓	CBP80/20-dependent translation initiation factor isoform X2 [B. mutus]
ENSP00000281523-D2	2.87	1.66E-02	↓	Zinc finger protein 385D, partial [B. mutus]
ENSBTAP00000013171-D1	2.87	1.66E-02	↓	soluble calcium-activated nucleotidase 1 [B. mutus]
ENSP00000167106-D1	2.87	1.66E-02	↓	Vasohibin-1 [B. mutus]
ENSBTAP00000007235-D1	2.87	1.66E-02	↓	tumor protein p53-inducible nuclear protein 2 isoform X1 [Bubalus bubalis]
ENSBTAP00000019399-D2	2.87	1.66E-02	↓	Tripartite motif-containing protein 15, partial [B. mutus]
ENSP00000374306-D1	2.87	1.66E-02	↓	hypothetical protein M91_21314 [B. mutus]
ENSP00000328625-D1	2.87	1.66E-02	↓	negative regulator of reactive oxygen species precursor [B. taurus]
ENSBTAP00000043881-D1	2.87	1.66E-02	↓	growth hormone-inducible transmembrane protein [B. taurus]
ENSBTAP00000010231-D1	2.87	1.66E-02	↓	myotilin isoform X1 [B. mutus]
ENSP00000327234-D1	2.87	1.66E-02	↓	Transmembrane and coiled-coil domain-containing protein 5A [B. mutus]
ENSBTAP00000021899-D1	2.87	1.66E-02	↓	ras-like protein family member 12 [B. mutus]
ENSP00000304078-D1	2.87	1.66E-02	↓	protein FAM83B [B. mutus]
ENSBTAP00000037476-D1	2.87	1.66E-02	↓	Neuronal growth regulator 1 [B. mutus]
ENSBTAP00000005147-D1	2.87	1.66E-02	↓	hypothetical protein M91_13925, partial [B. mutus]
ENSP00000362562-D1	2.87	1.66E-02	↓	transmembrane protein 54 isoform X1 [B. mutus]
ENSBTAP00000051739-D1	2.87	1.66E-02	↓	hypothetical protein M91_04681, partial [B. mutus]
ENSBTAP00000040657-D1	2.87	1.66E-02	↓	Endothelial cell-specific chemotaxis regulator, partial [B. mutus]
ENSBTAP00000041537-D24	2.87	1.66E-02	↓	Olfactory receptor 6C68, partial [B. mutus]
ENSP00000417184-D1	2.87	1.66E-02	↓	phospholipid scramblase family member 5 [B. mutus]
ENSP00000402226-D1	2.87	1.66E-02	↓	Relaxin receptor 1, partial [B. mutus]
ENSP00000384982-D20	2.87	1.66E-02	↓	MAGE-like protein 2, partial [B. mutus]
ENSBTAP00000036278-D1	2.87	1.66E-02	↓	2',3'-cyclic-nucleotide 3'-phosphodiesterase isoform X2 [B. taurus]
ENSP00000356278-D1	2.87	1.66E-02	↓	pleckstrin homology-like domain family A member 3, partial [B. mutus]
ENSP00000318128-D1	2.87	1.66E-02	↓	biogenesis of lysosome-related organelles complex 1 subunit 4-like, partial [B. mutus]
ENSBTAP00000006532-D3	2.87	1.66E-02	↓	Actin, alpha cardiac muscle 1 [B. mutus]
ENSP00000361795-D1	2.87	1.66E-02	↓	dysbindin (dystrobrevin binding protein 1) domain containing 2 [B. mutus]
ENSBTAP00000024926-D1	2.87	1.66E-02	↓	filamin-binding LIM protein 1 isoform X2 [B. taurus]
ENSP00000295981-D1	2.87	1.66E-02	↓	interleukin-17 receptor C isoform X1 [B. taurus]
ENSBTAP00000009960-D1	2.87	1.66E-02	↓	tumor necrosis factor receptor superfamily, member 11a, NFKB activator [B. mutus]
ENSP00000318635-D5	2.87	1.66E-02	↓	small ubiquitin-related modifier 2-like [Bubalus bubalis]
ENSBTAP00000004371-D1	2.87	1.66E-02	↓	Putative proline racemase, partial [B. mutus]
ENSBTAP00000014109-D1	2.87	1.66E-02	↓	Phosphoinositide-3-kinase-interacting protein 1, partial [B. mutus]
ENSBTAP00000027603-D1	2.87	1.66E-02	↓	transcription regulator protein BACH2 [B. mutus]
ENSP00000367965-D1	2.87	1.66E-02	↓	Ankyrin repeat domain-containing protein 43 [B. mutus]
ENSBTAP00000022557-D1	2.87	1.66E-02	↓	PHD finger protein 19, partial [B. mutus]
ENSBTAP00000017342-D1	2.87	1.66E-02	↓	Metabotropic glutamate receptor 7, partial [B. mutus]
ENSP00000418169-D1	2.87	1.66E-02	↓	synaptophysin [B. mutus]
ENSBTAP00000040770-D1	2.87	1.66E-02	↓	Tetratricopeptide repeat protein 16, partial [B. mutus]
ENSP00000359096-D1	2.87	1.66E-02	↓	alpha-amylase 2B-like [B. mutus]
ENSBTAP00000013902-D1	2.87	1.66E-02	↓	Transmembrane protein PVRIG, partial [B. mutus]
ENSBTAP00000019892-D1	2.87	1.66E-02	↓	thromboxane A2 receptor [B. mutus]
ENSBTAP00000053422-D1	2.87	1.66E-02	↓	Collagen alpha-1(IX) chain, partial [B. mutus]
ENSP00000372649-D1	2.87	1.66E-02	↓	proline-rich protein 23C-like [B. mutus]
ENSBTAP00000050036-D48	2.87	1.66E-02	↓	olfactory receptor 1030-like [Microcebus murinus]
ENSBTAP00000023593-D1	2.87	1.66E-02	↓	non-receptor tyrosine-protein kinase TNK1 isoform X2 [B. mutus]
ENSP00000346586-D1	2.87	1.66E-02	↓	G-protein coupled estrogen receptor 1 isoform X1 [Bubalus bubalis]
ENSBTAP00000014806-D1	2.87	1.66E-02	↓	single Ig IL-1-related receptor [B. mutus]
ENSBTAP00000026027-D1	2.87	1.66E-02	↓	hypothetical protein M91_06189, partial [B. mutus]
ENSBTAP00000008325-D1	2.87	1.66E-02	↓	GPI-linked NAD(P)(+)--arginine ADP-ribosyltransferase 1 [B. mutus]
ENSBTAP00000026482-D1	2.87	1.66E-02	↓	desmocollin-1 isoform X1 [B. mutus]
ENSBTAP00000037299-D1	2.87	1.66E-02	↓	stannin [B. taurus]
ENSP00000265708-D1	2.87	1.66E-02	↓	Disintegrin and metalloproteinase domain-containing protein 2, partial [B. mutus]
ENSBTAP00000005965-D1	2.87	1.66E-02	↓	uncharacterized protein C6orf15 homolog [B. mutus]
ENSP00000382575-D1	2.87	1.66E-02	↓	FLYWCH-type zinc finger-containing protein 1 [B. mutus]

ENSP00000273067-D1	2.87	1.66E-02	↓	E3 ubiquitin-protein ligase MARCH4 precursor [B. taurus]
ENSP00000290510-D1	2.87	1.66E-02	↓	Prolyl 3-hydroxylase 3, partial [B. mutus]
ENSP00000257013-D1	2.87	1.66E-02	↓	protein FAM127 [B. taurus]
ENSBTAP00000001704-D1	2.87	1.66E-02	↓	Lactotransferrin, partial [B. mutus]
ENSP00000368332-D1	2.87	1.66E-02	↓	Homeobox protein ARX [B. mutus]
ENSBTAP00000039840-D1	2.87	1.66E-02	↓	Melanopsin, partial [B. mutus]
ENSBTAP00000025880-D5	2.87	1.66E-02	↓	C-C chemokine receptor type 2, partial [B. mutus]
ENSP00000355330-D1	2.87	1.66E-02	↓	protein-glutamine gamma-glutamyltransferase 2 isoform X1 [B. mutus]
ENSBTAP00000031243-D3	2.87	1.66E-02	↓	Leukocyte elastase inhibitor [B. mutus]
ENSP00000369560-D26	2.87	1.66E-02	↓	interferon alpha-2-like [B. mutus]
ENSBTAP00000049732-D1	2.87	1.66E-02	↓	Olfactory receptor 8J2, partial [B. mutus]
ENSBTAP00000021785-D8	2.87	1.66E-02	↓	cytochrome P450 4X1-like [B. mutus]
ENSBTAP00000007573-D1	2.87	1.66E-02	↓	ly-6/neurotoxin-like protein 1-like isoform X1 [Leptonychotes weddellii]
ENSBTAP00000012813-D1	2.87	1.66E-02	↓	Rho GTPase-activating protein 33, partial [B. mutus]
ENSP00000309794-D1	2.87	1.66E-02	↓	TBC1 domain family member 16 [B. mutus]
ENSBTAP00000008354-D1	2.87	1.66E-02	↓	Ephrin type-A receptor 1, partial [B. mutus]
ENSBTAP00000021196-D1	2.87	1.66E-02	↓	hypothetical protein M91_09700, partial [B. mutus]
ENSBTAP00000041233-D1	2.87	1.66E-02	↓	Dipeptidyl aminopeptidase-like protein 6, partial [B. mutus]
ENSP00000352028-D1	2.87	1.66E-02	↓	putative E3 ubiquitin-protein ligase SH3RF2 [B. mutus]
ENSBTAP00000022806-D1	2.87	1.66E-02	↓	hypothetical protein M91_15997, partial [B. mutus]
ENSP00000341342-D1	2.87	1.66E-02	↓	Kelch-like protein 6, partial [B. mutus]
ENSBTAP00000011220-D1	2.87	1.66E-02	↓	hypothetical protein M91_04609, partial [B. mutus]
ENSP00000289957-D2	2.87	1.66E-02	↓	Neuronal acetylcholine receptor subunit beta-3, partial [B. mutus]
ENSP00000361202-D1	2.87	1.66E-02	↓	insulin receptor substrate 4 [B. mutus]
ENSBTAP00000051952-D1	2.87	1.66E-02	↓	zinc finger protein 664-like [Bison bison bison]
ENSBTAP00000038807-D3	2.87	1.66E-02	↓	GTPase IMAP family member 7-like [B. mutus]
yakG023494	2.87	1.66E-02	↓	hypothetical protein M91_15134 [B. mutus]
ENSP00000393299-D1	2.87	1.66E-02	↓	TPA: Src homology 2 domain containing F [B. taurus]
ENSP00000356094-D1	2.87	1.66E-02	↓	Vasopressin V1b receptor [B. mutus]
ENSBTAP00000016984-D1	2.87	1.66E-02	↓	Aquaporin-6, partial [B. mutus]
ENSBTAP00000022976-D1	2.87	1.66E-02	↓	DNA damage-regulated autophagy modulator protein 1 [B. mutus]
yakG011588	2.87	1.66E-02	↓	hyaluronan synthase 2-like [B. mutus]
ENSBTAP00000025122-D1	2.87	1.66E-02	↓	Coagulation factor XII, partial [B. mutus]
ENSBTAP00000046412-D1	2.87	1.66E-02	↓	hypothetical protein M91_06384, partial [B. mutus]
ENSBTAP00000003821-D1	2.87	1.66E-02	↓	EGF-like module-containing mucin-like hormone receptor-like 3, partial [B. mutus]
ENSBTAP00000052264-D1	2.87	1.66E-02	↓	Olfactory receptor 8I2, partial [B. mutus]
ENSBTAP00000022462-D1	2.87	1.66E-02	↓	.
ENSP00000324000-D1	2.87	1.66E-02	↓	putative uncharacterized protein FLJ13224 [Ovis aries]
ENSBTAP00000025423-D1	2.87	1.66E-02	↓	Notchless protein-like protein 1, partial [B. mutus]
ENSP00000416869-D1	2.87	1.66E-02	↓	Pantothenate kinase 4, partial [B. mutus]
ENSBTAP00000052872-D1	2.87	1.66E-02	↓	mariner transposase [Homo sapiens]
ENSBTAP00000000977-D1	2.87	1.66E-02	↓	bcl-2-related protein A1 [B. mutus]
ENSP00000391404-D1	2.87	1.66E-02	↓	hypothetical protein M91_06509, partial [B. mutus]
ENSBTAP00000027968-D1	2.87	1.66E-02	↓	Lysosomal acid phosphatase, partial [B. mutus]
ENSBTAP00000011232-D1	2.87	1.66E-02	↓	putative protein FAM90A8P-like [B. mutus]
ENSP00000368245-D1	2.87	1.66E-02	↓	uncharacterized protein CXorf21 homolog [B. mutus]
ENSBTAP00000023347-D1	2.87	1.66E-02	↓	HHIP-like protein 2 [B. mutus]
ENSBTAP00000053391-D1	2.87	1.66E-02	↓	potassium voltage-gated channel, Shaw-related subfamily, member 2, partial [B. mutus]
ENSP00000387100-D1	2.87	1.66E-02	↓	Taperin, partial [B. mutus]
ENSP00000365441-D1	2.87	1.66E-02	↓	Voltage-dependent L-type calcium channel subunit alpha-1F [B. mutus]
yakA07452	2.87	1.66E-02	↓	hypothetical protein M91_15785 [B. mutus]
ENSBTAP00000020476-D1	2.87	1.66E-02	↓	interleukin-22 [B. mutus]
ENSP00000381364-D1	2.89	8.77E-12	↓	calcineurin-binding protein cabin-1 isoform X6 [B. taurus]
ENSP00000255152-D1	2.89	1.11E-28	↓	zinc finger SWIM domain-containing protein 3 [B. mutus]
ENSBTAP00000022347-D1	2.90	2.09E-10	↓	Serine/threonine-protein kinase/endoribonuclease IRE1, partial [B. mutus]

ENSBTAP00000020709-D1	2.90	1.30E-183	↓	Sequestosome-1, partial [B. mutus]
ENSP00000291823-D1	2.90	9.61E-15	↓	homeodomain-interacting protein kinase 4 [B. mutus]
ENSBTAP00000028319-D1	2.91	2.29E-13	↓	transmembrane protein 35 [Ovis aries]
ENSP00000361366-D1	2.91	1.19E-07	↓	Collectin-43, partial [B. mutus]
ENSP00000340625-D1	2.91	5.42E-12	↓	hypothetical protein M91_11830, partial [B. mutus]
ENSBTAP00000021731-D1	2.92	2.79E-19	↓	acyl-coenzyme A thioesterase 13 [B. taurus]
ENSBTAP00000024727-D1	2.92	2.89E-06	↓	docking protein 1 isoform X1 [B. mutus]
ENSP00000332565-D1	2.92	2.89E-06	↓	sulfotransferase 4A1 [B. taurus]
ENSBTAP00000014612-D1	2.92	2.89E-06	↓	Coiled-coil domain-containing protein 136, partial [B. mutus]
ENSP00000297107-D1	2.92	2.89E-06	↓	polypeptide N-acetylgalactosaminyltransferase 10 [B. mutus]
ENSP00000333602-D1	2.93	2.03E-35	↓	E1A-binding protein p400 [B. mutus]
ENSP00000356370-D1	2.93	3.09E-09	↓	Crumbs-like protein 1, partial [B. mutus]
ENSP00000356155-D1	2.93	3.69E-15	↓	phosphatidylinositol 4-phosphate 3-kinase C2 domain-containing subunit beta [B. mutus]
ENSP00000346348-D1	2.94	7.36E-08	↓	zinc finger protein 774 [B. mutus]
ENSP00000359665-D1	2.94	7.36E-08	↓	phosphatidylinositol 4-kinase type 2-alpha [Bubalus bubalis]
ENSBTAP00000026265-D1	2.94	7.15E-05	↓	endothelial transcription factor GATA-2 [B. taurus]
ENSP00000377435-D1	2.94	7.15E-05	↓	T-box transcription factor TBX4 isoform X2 [B. taurus]
ENSBTAP00000020895-D1	2.94	7.15E-05	↓	PERQ amino acid-rich with GYF domain-containing protein 1, partial [B. mutus]
ENSP00000309163-D1	2.95	2.79E-21	↓	CAAX prenyl protease 2-like isoform X1 [B. mutus]
ENSP00000327309-D1	2.95	1.14E-34	↓	ADP-ribosylation factor GTPase-activating protein 2 [B. taurus]
ENSP00000311977-D1	2.95	2.27E-15	↓	tRNA-dihydrouridine(47) synthase [NAD(P)(+)]-like [B. mutus]
ENSBTAP00000040863-D2	2.95	2.07E-12	↓	BTB/POZ domain-containing protein 2 isoform X2 [Ovis aries musimon]
ENSP00000005905-D1	2.97	8.29E-28	↓	UPF0378 protein KIAA0100, partial [B. mutus]
ENSBTAP00000029167-D1	2.97	9.67E-19	↓	atypical kinase ADCK3, mitochondrial [B. taurus]
ENSP00000332407-D1	2.98	1.44E-46	↓	short-chain dehydrogenase/reductase family 42E member 1 [B. mutus]
ENSBTAP00000011848-D1	2.98	2.61E-51	↓	phosphatidylserine decarboxylase proenzyme [B. mutus]
ENSP00000233969-D1	2.99	5.13E-30	↓	solute carrier family 9, subfamily A (NHE2, cation proton antiporter 2), member 2 [B. mutus]
ENSBTAP00000009437-D1	3.00	3.69E-19	↓	P2X purinoceptor 1 [B. mutus]
ENSBTAP00000009631-D1	3.00	8.19E-98	↓	protein jagged-1 [Bubalus bubalis]
ENSP00000386869-D1	3.01	4.24E-57	↓	Putative methylcytosine dioxygenase TET3, partial [B. mutus]
ENSBTAP00000040758-D1	3.01	2.79E-08	↓	Pro-neuregulin-2, membrane-bound isoform [Pteropus alecto]
ENSP00000400490-D1	3.01	1.10E-06	↓	probable JmjC domain-containing histone demethylation protein 2C isoform X6 [B. taurus]
ENSBTAP00000014144-D1	3.01	4.38E-05	↓	Pro-neuregulin-2, membrane-bound isoform, partial [B. mutus]
ENSP00000334805-D1	3.01	4.38E-05	↓	hypothetical protein M91_21479, partial [B. mutus]
ENSBTAP00000046375-D1	3.01	4.38E-05	↓	lipase member J-like, partial [B. mutus]
ENSP00000397269-D1	3.01	4.38E-05	↓	protein FAM136A isoform 2 [Gorilla gorilla gorilla]
ENSP00000323183-D1	3.01	1.85E-03	↓	zinc finger and BTB domain-containing protein 2 [B. taurus]
ENSP00000286657-D1	3.01	1.85E-03	↓	A disintegrin and metalloproteinase with thrombospondin motifs 3 [B. mutus]
ENSBTAP00000012255-D1	3.01	1.85E-03	↓	Zinc transporter ZIP4 [B. mutus]
ENSP00000363832-D1	3.01	1.85E-03	↓	Aldehyde oxidase [B. mutus]
yakG009244	3.01	1.85E-03	↓	hypothetical protein M91_11139 [B. mutus]
ENSP00000346291-D1	3.02	5.12E-34	↓	unconventional myosin-XVIIIa [B. mutus]
ENSBTAP00000022024-D1	3.03	3.02E-13	↓	SPRY domain-containing SOCS box protein 3 [B. taurus]
ENSBTAP00000026057-D1	3.03	5.98E-23	↓	Zinc finger and SCAN domain-containing protein 20, partial [B. mutus]
ENSBTAP00000036200-D1	3.03	5.98E-23	↓	collagen alpha-1(XXI) chain isoform X2 [B. taurus]
ENSP00000362638-D1	3.04	4.47E-10	↓	MARCKS-related protein [B. taurus]
ENSP00000253458-D1	3.04	1.87E-52	↓	genetic suppressor element 1 [B. mutus]
ENSP00000407950-D1	3.04	1.39E-21	↓	Drebrin-like protein [B. mutus]
ENSP00000400899-D1	3.04	4.82E-15	↓	leucine-rich repeat-containing protein 9-like [B. mutus]
ENSP00000353695-D1	3.04	1.72E-08	↓	hypothetical protein M91_16426, partial [B. mutus]
ENSBTAP00000042002-D1	3.04	1.72E-08	↓	Amyotrophic lateral sclerosis 2 chromosomal region candidate 4 protein [B. mutus]
ENSP00000378350-D1	3.05	1.86E-13	↓	cell division cycle protein 23 homolog isoform X1 [Ovis aries]
ENSBTAP00000027417-D1	3.05	7.79E-17	↓	Serine protease 27, partial [B. mutus]
ENSBTAP00000016383-D1	3.05	6.70E-07	↓	hypothetical protein M91_20921 [B. mutus]
ENSP00000406585-D1	3.06	2.79E-35	↓	uncharacterized protein LOC102327815 [Pantholops hodgsonii]

ENSP00000419194-D1	3.06	2.75E-10	↓	prostate-associated microseminoprotein [B. taurus]
ENSBTAP00000039689-D1	3.07	4.38E-12	↓	pleckstrin homology domain-containing family G member 3 isoform X2 [B. taurus]
ENSBTAP00000033928-D1	3.07	2.68E-05	↓	DENN domain-containing protein 4B, partial [B. mutus]
ENSBTAP00000043683-D1	3.07	2.68E-05	↓	Leucine-rich repeat and immunoglobulin-like domain-containing nogo receptor-interacting protein 1, partial [B. mutus]
ENSBTAP00000013435-D1	3.09	4.10E-07	↓	Unhealthy ribosome biogenesis protein 2-like protein [B. mutus]
ENSBTAP00000006434-D1	3.09	4.10E-07	↓	Oxidoreductase NAD-binding domain-containing protein 1, partial [B. mutus]
ENSP00000376794-D1	3.10	6.51E-09	↓	solute carrier family 35 member G2 [B. mutus]
ENSP00000358925-D1	3.11	6.26E-81	↓	Proline/serine-rich coiled-coil protein 1 [B. mutus]
ENSBTAP00000043531-D1	3.11	3.56E-72	↓	presequence protease, mitochondrial [Bison bison bison]
ENSP00000219172-D1	3.12	9.10E-43	↓	centromere protein T [B. mutus]
ENSP00000321556-D1	3.13	8.97E-47	↓	Dedicator of cytokinesis protein 6, partial [B. mutus]
ENSP00000386331-D1	3.13	1.20E-30	↓	Myosin-VIIa, partial [B. mutus]
yakA13557	3.13	1.61E-14	↓	Protein FAM170A [B. mutus]
ENSBTAP00000024188-D1	3.13	2.51E-07	↓	uncharacterized protein C17orf62 homolog [B. taurus]
ENSBTAP00000002536-D1	3.13	1.12E-03	↓	Prominin-2, partial [B. mutus]
ENSP00000312649-D1	3.13	1.12E-03	↓	Peroxisome proliferator-activated receptor gamma coactivator 1-beta, partial [B. mutus]
ENSBTAP00000019045-D1	3.13	1.12E-03	↓	Putative protein KIAA1467, partial [B. mutus]
ENSBTAP00000045688-D1	3.13	1.12E-03	↓	hypothetical protein M91_18799, partial [B. mutus]
ENSP00000379657-D1	3.13	1.12E-03	↓	calcitonin-like [B. mutus]
ENSBTAP00000016486-D1	3.13	1.12E-03	↓	transport and Golgi organization protein 2 homolog isoform X1 [B. mutus]
ENSP00000377171-D87	3.13	1.12E-03	↓	zinc finger protein 548-like [Bison bison bison]
ENSBTAP00000024015-D1	3.13	1.12E-03	↓	proteasome subunit beta type-10 [B. mutus]
ENSP00000238647-D1	3.13	1.12E-03	↓	interferon regulatory factor 2 binding protein-like, partial [B. mutus]
ENSP00000363827-D1	3.13	1.12E-03	↓	basement membrane-specific heparan sulfate proteoglycan core protein, partial [B. mutus]
ENSP00000360437-D1	3.13	1.12E-03	↓	hypothetical protein M91_17506, partial [B. mutus]
ENSP00000323633-D1	3.13	1.12E-03	↓	Disco-interacting protein 2-like protein A, partial [B. mutus]
ENSP00000368666-D2	3.13	9.79E-03	↓	patched domain-containing protein 3-like, partial [B. mutus]
ENSP00000296452-D1	3.13	9.79E-03	↓	protein bassoon [B. mutus]
ENSBTAP00000024934-D1	3.13	9.79E-03	↓	heat shock 70 kDa protein 12B isoform X1 [B. mutus]
ENSBTAP00000026223-D1	3.13	9.79E-03	↓	melanocortin receptor 4-like [B. mutus]
ENSBTAP00000008192-D1	3.13	9.79E-03	↓	beta-1,3-galactosyl-O-glycosyl-glycoprotein beta-1,6-N-acetylglucosaminyltransferase 7 [B. mutus]
ENSP00000341528-D90	3.13	9.79E-03	↓	Zinc finger protein 34, partial [B. mutus]
ENSP00000317534-D1	3.13	9.79E-03	↓	zinc finger and BTB domain-containing protein 34 isoform X1 [B. taurus]
ENSBTAP00000015756-D1	3.13	9.79E-03	↓	Echinoderm microtubule-associated protein-like 3, partial [B. mutus]
ENSBTAP00000023313-D1	3.13	9.79E-03	↓	Prostacyclin synthase, partial [B. mutus]
ENSP00000299237-D1	3.13	9.79E-03	↓	Zinc finger protein 319, partial [B. mutus]
ENSBTAP00000009016-D3	3.13	9.79E-03	↓	C-type lectin domain family 2 member D11 isoform X1 [B. taurus]
ENSBTAP00000050983-D26	3.13	9.79E-03	↓	hypothetical protein M91_09309, partial [B. mutus]
ENSP00000287538-D1	3.13	9.79E-03	↓	zinc finger protein ZIC 3-like [B. mutus]
ENSBTAP00000050251-D1	3.13	9.79E-03	↓	chondroadherin-like protein [B. mutus]
ENSBTAP00000005736-D1	3.13	9.79E-03	↓	Epithelial splicing regulatory protein 2, partial [B. mutus]
ENSBTAP00000016346-D3	3.13	9.79E-03	↓	Purine nucleoside phosphorylase, partial [B. mutus]
ENSBTAP00000028438-D1	3.13	9.79E-03	↓	Renin, partial [B. mutus]
ENSBTAP00000004838-D1	3.13	9.79E-03	↓	disintegrin and metalloproteinase domain-containing protein 21 [B. mutus]
ENSP00000321026-D1	3.13	9.79E-03	↓	leucine rich adaptor protein 1-like [B. taurus]
ENSP00000384482-D39	3.13	9.79E-03	↓	hypothetical protein M91_11423, partial [B. mutus]
ENSP00000254325-D1	3.13	9.79E-03	↓	MHC class II regulatory factor RFX1, partial [B. mutus]
ENSBTAP00000009492-D1	3.13	9.79E-03	↓	Arginyl aminopeptidase-like 1, partial [B. mutus]
ENSBTAP00000013209-D1	3.13	9.79E-03	↓	hypothetical protein M91_19064 [B. mutus]
ENSP00000361768-D1	3.13	9.79E-03	↓	regulating synaptic membrane exocytosis protein 3 [B. taurus]
ENSBTAP00000019292-D6	3.13	9.79E-03	↓	olfactory receptor 8H1-like [Bubalus bubalis]
ENSP00000407057-D1	3.13	9.79E-03	↓	ankyrin-1 isoform X2 [B. taurus]
ENSP00000361266-D1	3.13	9.79E-03	↓	protein patched homolog 2 [B. mutus]
ENSBTAP00000045224-D1	3.13	9.79E-03	↓	protein disulfide-isomerase A2 [B. mutus]

ENSP00000238508-D4	3.13	9.79E-03	↓	Serpin B9, partial [B. mutus]
ENSBTAP00000037706-D1	3.13	9.79E-03	↓	Sulfotransferase 6B1, partial [B. mutus]
ENSP00000261883-D1	3.13	9.79E-03	↓	cartilage intermediate layer protein 1 [B. mutus]
ENSBTAP00000012923-D1	3.13	9.79E-03	↓	Doublecortin domain-containing protein 2, partial [B. mutus]
ENSP00000325203-D86	3.13	9.79E-03	↓	hypothetical protein M91_00408, partial [B. mutus]
ENSBTAP00000042828-D1	3.13	9.79E-03	↓	nephrocystin-4 [B. mutus]
ENSP00000269601-D1	3.13	9.79E-03	↓	thioredoxin-like 4A isoform X1 [Equus caballus]
ENSP00000307822-D1	3.13	9.79E-03	↓	Neuropeptide FF receptor 2, partial [B. mutus]
ENSBTAP00000000306-D1	3.13	9.79E-03	↓	NADP-dependent malic enzyme, mitochondrial, partial [B. mutus]
yakG033540	3.13	9.79E-03	↓	hypothetical protein M91_18148 [B. mutus]
ENSP00000278976-D1	3.13	9.79E-03	↓	TOX high mobility group box family member 2 [B. mutus]
ENSBTAP00000021348-D1	3.13	9.79E-03	↓	guanine nucleotide-binding protein G(I)/G(S)/G(T) subunit beta-3 [B. taurus]
ENSP00000331302-D2	3.13	9.79E-03	↓	Transcription elongation factor B polypeptide 3, partial [B. mutus]
ENSP00000310335-D1	3.13	9.79E-03	↓	rab15 effector protein [B. mutus]
ENSP00000399863-D1	3.13	9.79E-03	↓	Zinc finger protein 839, partial [B. mutus]
ENSBTAP00000020040-D1	3.13	9.79E-03	↓	retinol-binding protein 2 [B. taurus]
yakG007284	3.13	9.79E-03	↓	hypothetical protein M91_16181 [B. mutus]
ENSBTAP00000040857-D1	3.13	9.79E-03	↓	IQ domain-containing protein C [B. mutus]
ENSBTAP00000052766-D1	3.13	9.79E-03	↓	Solute carrier family 25 member 42, partial [B. mutus]
ENSBTAP00000024449-D1	3.13	9.79E-03	↓	StAR-related lipid transfer protein 7, mitochondrial, partial [B. mutus]
ENSP00000372900-D1	3.13	9.79E-03	↓	piezo-type mechanosensitive ion channel component 2 [Capra hircus]
ENSBTAP00000049067-D1	3.13	9.79E-03	↓	T-cell receptor alpha chain C region, partial [B. mutus]
ENSBTAP00000007323-D1	3.13	9.79E-03	↓	zinc finger protein 205 isoform X1 [B. mutus]
ENSP00000405974-D1	3.13	9.79E-03	↓	EGF-like module-containing mucin-like hormone receptor-like 1, partial [B. mutus]
ENSBTAP00000049839-D67	3.13	9.79E-03	↓	olfactory receptor-like [B. taurus]
ENSBTAP00000007525-D1	3.13	9.79E-03	↓	Indoleamine 2,3-dioxygenase 2 [B. mutus]
ENSBTAP00000052257-D20	3.13	9.79E-03	↓	hypothetical protein M91_11521, partial [B. mutus]
ENSBTAP00000007111-D1	3.13	9.79E-03	↓	Hemicentin-2, partial [B. mutus]
ENSBTAP00000044908-D1	3.13	9.79E-03	↓	Protein FAM84B [B. mutus]
ENSBTAP00000010773-D1	3.13	9.79E-03	↓	Sulfatase-modifying factor 2, partial [B. mutus]
ENSBTAP00000015904-D1	3.13	9.79E-03	↓	probable ergosterol biosynthetic protein 28-like [B. mutus]
ENSBTAP00000027792-D2	3.13	9.79E-03	↓	Chain A, The Structural Basis Of The Interaction Between Nonsense Mediated Decay Factors Upf2 And Upf3
ENSP00000299308-D3	3.13	9.79E-03	↓	transmembrane protein 132D [B. mutus]
ENSP00000354669-D1	3.13	9.79E-03	↓	Protein FAM63A, partial [B. mutus]
ENSBTAP00000046319-D16	3.13	9.79E-03	↓	hypothetical protein M91_19205, partial [B. mutus]
ENSBTAP00000053259-D1	3.13	9.79E-03	↓	Myosin-VIIb, partial [B. mutus]
ENSBTAP00000022376-D2	3.13	9.79E-03	↓	allergen Bos d 2-like [B. mutus]
ENSP00000384644-D1	3.13	9.79E-03	↓	kelch-like protein 31 [B. mutus]
ENSBTAP00000049432-D3	3.13	9.79E-03	↓	Chymotrypsinogen A [B. mutus]
ENSP00000326022-D1	3.13	9.79E-03	↓	four and a half LIM domains protein 5 [B. mutus]
ENSBTAP00000000176-D1	3.13	9.79E-03	↓	Leucine-rich repeat and fibronectin type-III domain-containing protein 3 [B. mutus]
ENSBTAP00000019419-D1	3.13	9.79E-03	↓	Cancer-associated 1 protein-like protein, partial [B. mutus]
ENSBTAP00000028564-D1	3.13	9.79E-03	↓	Beta-Ala-His dipeptidase, partial [B. mutus]
ENSBTAP00000008122-D1	3.13	9.79E-03	↓	dnaJ homolog subfamily B member 3-like [B. mutus]
ENSP00000295759-D1	3.13	9.79E-03	↓	beta-galactosidase-1-like protein isoform X1 [B. mutus]
ENSP00000296140-D3	3.13	9.79E-03	↓	C-C chemokine receptor type 5, partial [B. mutus]
ENSP00000257868-D1	3.13	9.79E-03	↓	growth/differentiation factor 11 isoform X2 [Ovis aries musimon]
ENSP00000335566-D2	3.13	9.79E-03	↓	.
ENSBTAP00000051608-D1	3.13	9.79E-03	↓	Olfactory receptor 11H7, partial [B. mutus]
ENSBTAP00000025079-D1	3.13	9.79E-03	↓	Putative IQ and AAA domain-containing protein 1-like protein, partial [B. mutus]
ENSP00000231130-D41	3.13	9.79E-03	↓	Protocadherin gamma-B5, partial [B. mutus]
ENSP00000310585-D1	3.13	9.79E-03	↓	Purkinje cell protein 2, partial [B. mutus]
ENSBTAP00000025291-D1	3.13	9.79E-03	↓	solute carrier family 2, facilitated glucose transporter member 12 [B. mutus]
ENSBTAP00000052960-D15	3.13	9.79E-03	↓	interferon omega-1-like [B. mutus]
ENSBTAP00000024586-D1	3.13	9.79E-03	↓	Macrophage receptor MARCO, partial [B. mutus]

ENSBTAP00000043509-D1	3.13	9.79E-03	↓	corticotropin-releasing factor receptor 2 isoform X4 [B. mutus]
ENSP00000348461-D2	3.13	9.79E-03	↓	ras-related C3 botulinum toxin substrate 2 isoform X1 [B. mutus]
ENSBTAP00000018740-D5	3.13	9.79E-03	↓	Gamma-crystallin B, partial [B. mutus]
ENSP00000325618-D1	3.13	9.79E-03	↓	poly [ADP-ribose] polymerase 10 [Bison bison bison]
ENSP00000356429-D1	3.13	9.79E-03	↓	regulator of G-protein signaling 1 [B. mutus]
ENSBTAP00000009010-D1	3.13	9.79E-03	↓	hypothetical protein M91_13491, partial [B. mutus]
ENSP00000307858-D1	3.13	9.79E-03	↓	zinc finger and BTB domain-containing protein 4 [B. taurus]
ENSBTAP00000027077-D1	3.13	9.79E-03	↓	Dysbindin domain-containing protein 1, partial [B. mutus]
ENSBTAP00000011254-D1	3.13	9.79E-03	↓	Dynein intermediate chain 1, axonemal [B. mutus]
ENSBTAP00000012316-D2	3.13	9.79E-03	↓	MTSS1-like protein, partial [B. mutus]
ENSBTAP00000007633-D1	3.13	9.79E-03	↓	Epoxide hydrolase 4, partial [B. mutus]
ENSP00000322339-D1	3.13	9.79E-03	↓	vomeronasal type-1 receptor 1 [B. taurus]
ENSP00000367373-D8	3.13	9.79E-03	↓	Protocadherin alpha-7, partial [B. mutus]
ENSP00000386414-D1	3.13	9.79E-03	↓	double-strand-break repair protein rad21-like protein 1 [B. mutus]
ENSBTAP00000041521-D1	3.13	9.79E-03	↓	Low-density lipoprotein receptor-related protein 4, partial [B. mutus]
ENSBTAP00000016520-D1	3.13	9.79E-03	↓	HLA class II histocompatibility antigen, DM beta chain, partial [B. mutus]
ENSP00000278829-D2	3.13	9.79E-03	↓	Fatty acid desaturase 2, partial [B. mutus]
ENSP00000327687-D1	3.13	9.79E-03	↓	glutamate receptor ionotropic, kainate 1 isoform X2 [Ovis aries]
ENSP00000352608-D3	3.13	9.79E-03	↓	hypothetical protein M91_17444, partial [B. mutus]
ENSBTAP00000003192-D1	3.13	9.79E-03	↓	Tubulin alpha-4A chain, partial [B. mutus]
ENSBTAP00000017851-D1	3.13	9.79E-03	↓	cystatin-11 [B. mutus]
ENSBTAP00000010825-D1	3.13	9.79E-03	↓	BEN domain-containing protein 6 [B. mutus]
ENSP00000338297-D1	3.13	9.79E-03	↓	insulin-like growth factor II isoform X2 [B. taurus]
ENSBTAP00000048014-D1	3.13	9.79E-03	↓	Putative G-protein coupled receptor 111, partial [B. mutus]
ENSBTAP00000024811-D1	3.13	9.79E-03	↓	homeobox protein DLX-5 [B. taurus]
ENSBTAP00000050243-D4	3.13	9.79E-03	↓	hypothetical protein M91_06266, partial [B. mutus]
ENSP00000371212-D1	3.13	9.79E-03	↓	RNA-binding protein 47 [B. taurus]
ENSP00000384182-D1	3.13	9.79E-03	↓	sec1 family domain-containing protein 2-like [B. mutus]
ENSP00000324403-D1	3.13	9.79E-03	↓	PHD finger protein 21B, partial [B. mutus]
ENSP00000239446-D5	3.13	9.79E-03	↓	Protocadherin gamma-A11, partial [B. mutus]
ENSBTAP00000010273-D1	3.13	9.79E-03	↓	mariner transposase [Homo sapiens]
ENSP00000328274-D43	3.13	9.79E-03	↓	Melanoma-associated antigen B2, partial [B. mutus]
ENSBTAP00000046843-D1	3.13	9.79E-03	↓	hypothetical protein M91_21445, partial [B. mutus]
ENSBTAP00000017584-D1	3.13	9.79E-03	↓	protein odd-skipped-related 2 [B. taurus]
ENSBTAP00000053679-D1	3.13	9.79E-03	↓	Transmembrane protein 154, partial [B. mutus]
ENSP00000312273-D1	3.13	9.79E-03	↓	Leucine-rich repeat LGI family member 4 [B. mutus]
ENSP00000412461-D1	3.15	1.28E-45	↓	rho GTPase-activating protein 22 isoform X4 [Bison bison bison]
ENSBTAP00000000407-D1	3.16	3.90E-11	↓	nucleoside diphosphate-linked moiety X motif 13 [B. taurus]
ENSBTAP00000013862-D2	3.16	1.14E-40	↓	Vacuolar protein sorting-associated protein 4A, partial [B. mutus]
ENSBTAP00000018628-D1	3.17	3.80E-13	↓	Phosphoprotein associated with glycosphingolipid-enriched microdomains 1, partial [B. mutus]
ENSP00000395253-D1	3.17	1.54E-07	↓	submaxillary mucin-like protein isoform X2 [B. taurus]
ENSBTAP00000026411-D1	3.17	1.54E-07	↓	A disintegrin and metalloproteinase with thrombospondin motifs 17, partial [B. mutus]
ENSBTAP00000008496-D1	3.18	1.64E-37	↓	DNA-directed RNA polymerase I subunit RPA1 [B. mutus]
ENSBTAP00000053826-D1	3.19	9.89E-40	↓	RNA polymerase II elongation factor ELL, partial [B. mutus]
ENSBTAP00000023515-D1	3.19	9.91E-06	↓	nitric oxide synthase, endothelial [B. mutus]
ENSP00000414721-D1	3.19	9.91E-06	↓	WW domain binding protein 1-like [B. mutus]
ENSP00000254584-D1	3.20	5.79E-23	↓	arfaptin-2 isoform 1 [Ceratotherium simum simum]
ENSBTAP00000012415-D1	3.21	2.24E-17	↓	protein SMG5 [B. mutus]
ENSBTAP00000026052-D1	3.21	9.37E-08	↓	TBC1 domain family member 2A [B. mutus]
ENSP00000257894-D1	3.21	9.37E-08	↓	Protein FAM186B, partial [B. mutus]
ENSP00000356282-D1	3.21	9.37E-08	↓	Ladinin-1, partial [B. mutus]
ENSP00000404438-D1	3.23	1.35E-19	↓	Spermatogenesis- and oogenesis-specific basic helix-loop-helix-containing protein 1 [B. mutus]
ENSP00000352336-D1	3.23	2.07E-31	↓	1-phosphatidylinositol-4,5-bisphosphate phosphodiesterase gamma-2, partial [B. mutus]
ENSP00000262577-D1	3.23	8.88E-12	↓	Zinc finger CCCH domain-containing protein 3 [B. mutus]
ENSBTAP00000016505-D1	3.23	8.68E-14	↓	pyridoxal phosphate phosphatase, partial [B. mutus]

ENSBTAP00000016889-D1	3.24	8.37E-18	↓	receptor-transporting protein 1 [B. mutus]
ENSBTAP00000007973-D1	3.24	8.37E-18	↓	Coiled-coil and C2 domain-containing protein 1A, partial [B. mutus]
ENSBTAP00000015786-D2	3.25	5.56E-10	↓	SLIT-ROBO Rho GTPase-activating protein 2 isoform X4 [B. taurus]
ENSBTAP00000015696-D1	3.25	5.56E-10	↓	Peroxisome assembly protein 26 [B. mutus]
ENSP00000253055-D1	3.25	6.04E-06	↓	mitogen-activated protein kinase kinase kinase 10 isoform X2 [Ovis aries musimon]
ENSBTAP00000005146-D1	3.25	5.19E-16	↓	kinesin family member 5A [Camelus ferus]
ENSBTAP00000009187-D1	3.25	6.73E-04	↓	myelin regulatory factor isoform X1 [B. taurus]
ENSP00000355089-D1	3.25	6.73E-04	↓	CUGBP Elav-like family member 4 [B. mutus]
ENSP00000357362-D1	3.25	6.73E-04	↓	thrombospondin-3 isoform X1 [B. mutus]
ENSP00000332900-D1	3.25	6.73E-04	↓	Putative G-protein coupled receptor 97, partial [B. mutus]
ENSBTAP00000050715-D1	3.25	6.73E-04	↓	uncharacterized protein C15orf43 homolog, partial [B. mutus]
ENSBTAP00000043771-D1	3.27	3.17E-16	↓	Activated CDC42 kinase 1 [B. mutus]
ENSBTAP00000026474-D1	3.27	1.87E-20	↓	microprocessor complex subunit DGCR8 [B. mutus]
ENSP00000266534-D1	3.28	3.39E-10	↓	transmembrane protein 117-like, partial [B. mutus]
ENSP00000307235-D1	3.28	1.94E-16	↓	Eukaryotic translation initiation factor 2-alpha kinase 3, partial [B. mutus]
ENSP00000349364-D1	3.29	1.81E-58	↓	Intermediate filament family orphan 1, partial [B. mutus]
ENSP00000367024-D1	3.32	7.31E-15	↓	N-alpha-acetyltransferase 40, NatD catalytic subunit, partial [B. mutus]
ENSBTAP00000011884-D1	3.32	2.12E-08	↓	phosphofurin acidic cluster sorting protein 1 [B. mutus]
ENSBTAP00000018862-D1	3.35	1.67E-41	↓	Nuclear receptor coactivator 5, partial [B. mutus]
ENSBTAP00000025683-D2	3.36	2.22E-06	↓	Protein kinase C and casein kinase substrate in neurons protein 1 [B. mutus]
ENSBTAP00000015947-D1	3.36	2.22E-06	↓	LIM homeobox transcription factor 1-alpha, partial [B. mutus]
ENSP00000403208-D1	3.36	2.22E-06	↓	Putative protein KIAA1383, partial [B. mutus]
ENSBTAP00000041235-D1	3.36	4.05E-04	↓	kinesin light chain 3 [Bison bison bison]
ENSP00000403459-D1	3.36	4.05E-04	↓	growth factor receptor-bound protein 7 [B. mutus]
ENSBTAP00000025929-D1	3.36	4.05E-04	↓	Putative aarF domain-containing protein kinase 4 [B. mutus]
ENSP00000291672-D1	3.36	4.05E-04	↓	speriolin-like protein isoform X1 [B. mutus]
ENSBTAP00000004193-D1	3.36	5.76E-03	↓	estradiol 17-beta-dehydrogenase 2 [B. mutus]
ENSBTAP00000053791-D1	3.36	5.76E-03	↓	protein tyrosine phosphatase, receptor type, M, partial [B. mutus]
ENSBTAP00000051198-D1	3.36	5.76E-03	↓	hyaluronidase-2 [B. mutus]
ENSBTAP00000028870-D1	3.36	5.76E-03	↓	Substance-K receptor [B. mutus]
ENSBTAP00000004937-D6	3.36	5.76E-03	↓	guanine nucleotide-binding protein G(t) subunit alpha-2 [B. mutus]
ENSBTAP00000045416-D1	3.36	5.76E-03	↓	Chemokine-binding protein 2, partial [B. mutus]
ENSP00000039007-D1	3.36	5.76E-03	↓	Ornithine carbamoyltransferase, mitochondrial, partial [B. mutus]
ENSP00000366923-D1	3.36	5.76E-03	↓	Insulin-like growth factor-binding protein-like 1, partial [B. mutus]
ENSBTAP00000013544-D1	3.36	5.76E-03	↓	Synaptotagmin-like protein 1 [B. mutus]
ENSP00000264031-D1	3.36	5.76E-03	↓	uroplakin-2 precursor [B. taurus]
ENSBTAP00000013017-D1	3.36	5.76E-03	↓	transcription cofactor HES-6 [B. taurus]
ENSBTAP00000026209-D1	3.36	5.76E-03	↓	Interleukin-1 receptor antagonist protein, partial [B. mutus]
ENSBTAP00000025435-D1	3.36	5.76E-03	↓	Fanconi anemia-associated protein of 100 kDa, partial [B. mutus]
ENSP00000217254-D1	3.36	5.76E-03	↓	solute carrier family 52, riboflavin transporter, member 3 [B. mutus]
ENSBTAP00000010313-D1	3.36	5.76E-03	↓	OTU domain-containing protein 6A [B. mutus]
ENSBTAP00000028765-D1	3.36	5.76E-03	↓	acid sphingomyelinase-like phosphodiesterase 3a [B. mutus]
ENSBTAP00000021606-D1	3.36	5.76E-03	↓	dual oxidase maturation factor 2 [B. mutus]
ENSP00000307852-D1	3.36	5.76E-03	↓	Nucleoside diphosphate-linked moiety X motif 18, partial [B. mutus]
yakA08283	3.36	5.76E-03	↓	Rho-related GTP-binding protein RhoU [B. mutus]
ENSBTAP00000051169-D1	3.36	5.76E-03	↓	insulin-like growth factor-binding protein 6 isoform X2 [Bison bison bison]
ENSBTAP00000033586-D1	3.36	5.76E-03	↓	Potassium voltage-gated channel subfamily C member 4 [B. mutus]
ENSP00000318898-D1	3.36	5.76E-03	↓	Zinc finger protein 114, partial [B. mutus]
ENSBTAP00000053211-D2	3.36	5.76E-03	↓	MOSC domain-containing protein 2, mitochondrial [B. mutus]
ENSBTAP00000034436-D1	3.36	5.76E-03	↓	hypothetical protein M91_01927, partial [B. mutus]
ENSBTAP00000021357-D1	3.36	5.76E-03	↓	carnitine O-palmitoyltransferase 1, muscle isoform isoform X1 [B. mutus]
ENSBTAP00000001499-D1	3.36	5.76E-03	↓	RING finger protein 165, partial [B. mutus]
ENSBTAP00000038865-D1	3.36	5.76E-03	↓	PC-esterase domain-containing protein 1B [B. mutus]
ENSP00000323928-D99	3.36	5.76E-03	↓	olfactory receptor 5M11-like isoform X1 [B. taurus]
ENSBTAP00000015588-D1	3.36	5.76E-03	↓	transferrin receptor protein 2 [B. mutus]

ENSBTAP00000044656-D1	3.36	5.76E-03	↓	Nck-associated protein 1-like protein [B. mutus]
ENSP00000330572-D1	3.36	5.76E-03	↓	c-Jun-amino-terminal kinase-interacting protein 2 [Pantholops hodgsonii]
ENSP00000326018-D1	3.36	5.76E-03	↓	Periaxin [B. mutus]
yakG014013	3.36	5.76E-03	↓	hypothetical protein M91_00371 [B. mutus]
ENSP00000326572-D5	3.36	5.76E-03	↓	hypothetical protein M91_10244, partial [B. mutus]
ENSP00000273063-D1	3.36	5.76E-03	↓	Anion exchange protein 3 [B. mutus]
ENSP00000362807-D1	3.36	5.76E-03	↓	peflin [B. mutus]
ENSBTAP00000015155-D1	3.36	5.76E-03	↓	TPA: testis flippase-like [B. taurus]
ENSBTAP00000050320-D53	3.36	5.76E-03	↓	hypothetical protein M91_12986, partial [B. mutus]
ENSBTAP00000031506-D1	3.36	5.76E-03	↓	plasminogen activator inhibitor 2 [B. mutus]
ENSBTAP00000000510-D1	3.36	5.76E-03	↓	Protein canopy-like protein 1, partial [B. mutus]
ENSBTAP00000032308-D1	3.36	5.76E-03	↓	calcium-responsive transactivator isoform X1 [B. mutus]
ENSBTAP00000051460-D1	3.36	5.76E-03	↓	DNA nucleotidylexotransferase, partial [B. mutus]
ENSBTAP00000000580-D1	3.36	5.76E-03	↓	carboxypeptidase B [B. mutus]
ENSP00000378802-D1	3.36	5.76E-03	↓	Transmembrane protein 71 [B. mutus]
ENSBTAP00000002378-D1	3.36	5.76E-03	↓	Homeobox protein CDX-2, partial [B. mutus]
ENSP00000376565-D1	3.36	5.76E-03	↓	transmembrane and ubiquitin-like domain-containing protein 1-like [Camelus ferus]
ENSP00000330393-D1	3.36	5.76E-03	↓	Leptin receptor [B. mutus]
ENSP00000263278-D1	3.36	5.76E-03	↓	17-beta-hydroxysteroid dehydrogenase 14 [B. mutus]
ENSBTAP00000011783-D1	3.36	5.76E-03	↓	Alkaline phosphatase, tissue-nonspecific isozyme, partial [B. mutus]
ENSBTAP00000033826-D1	3.36	5.76E-03	↓	protein RD3 [B. mutus]
ENSP00000402081-D3	3.36	5.76E-03	↓	Disintegrin and metalloproteinase domain-containing protein 29, partial [B. mutus]
ENSP00000357615-D1	3.36	5.76E-03	↓	tyrosine-protein kinase FRK [B. mutus]
ENSBTAP00000029061-D1	3.36	5.76E-03	↓	GRAM domain-containing protein 4, partial [B. mutus]
ENSBTAP00000005528-D1	3.36	5.76E-03	↓	hypothetical protein M91_20669, partial [B. mutus]
ENSBTAP00000019812-D1	3.36	5.76E-03	↓	DNA (cytosine-5)-methyltransferase 3-like protein, partial [B. mutus]
ENSP00000323036-D1	3.36	5.76E-03	↓	prostatic acid phosphatase-like isoform X1 [B. mutus]
ENSBTAP00000007987-D1	3.36	5.76E-03	↓	Interleukin-15 receptor subunit alpha, partial [B. mutus]
ENSP00000371397-D1	3.36	5.76E-03	↓	protein FAM43A [B. taurus]
ENSP00000364515-D1	3.36	5.76E-03	↓	BTB/POZ domain-containing protein 17 [B. mutus]
ENSP00000340378-D1	3.36	5.76E-03	↓	Coiled-coil domain-containing protein 30 [B. mutus]
ENSBTAP00000020909-D1	3.36	5.76E-03	↓	Guanine nucleotide exchange factor for Rab-3A, partial [B. mutus]
ENSP00000265707-D1	3.36	5.76E-03	↓	disintegrin and metalloproteinase domain-containing protein 18 [B. mutus]
ENSBTAP00000051381-D6	3.36	5.76E-03	↓	TPA: ribosomal protein L21-like [B. taurus]
ENSBTAP00000008848-D1	3.36	5.76E-03	↓	Large neutral amino acids transporter small subunit 1, partial [B. mutus]
ENSBTAP00000053553-D1	3.36	5.76E-03	↓	Polymerase I and transcript release factor [B. mutus]
ENSP00000222753-D1	3.36	5.76E-03	↓	homeobox protein Hox-A13 isoform X2 [Ovis aries musimon]
ENSP00000355736-D1	3.36	5.76E-03	↓	Tctex1 domain-containing protein 3, partial [B. mutus]
ENSP00000383230-D1	3.36	5.76E-03	↓	ICOS ligand, partial [B. mutus]
ENSP00000329165-D1	3.36	5.76E-03	↓	Keratin, type I cuticular Ha6 [B. mutus]
ENSP00000408494-D15	3.36	5.76E-03	↓	high mobility group protein B3-like protein [Cricetulus griseus]
ENSP00000352284-D1	3.36	5.76E-03	↓	transmembrane protein 139 [B. taurus]
ENSBTAP00000053814-D1	3.36	5.76E-03	↓	Protein FAM179A, partial [B. mutus]
ENSP00000419199-D1	3.36	5.76E-03	↓	Ankyrin repeat and SOCS box protein 14 [B. mutus]
ENSBTAP00000019761-D1	3.36	5.76E-03	↓	Vacuolar protein sorting-associated protein 26B, partial [B. mutus]
ENSP00000277491-D1	3.36	5.76E-03	↓	hemicentin-2, partial [Bison bison bison]
ENSP00000191922-D1	3.36	5.76E-03	↓	Na(+)/H(+) exchange regulatory cofactor NHE-RF2, partial [B. mutus]
ENSBTAP00000045034-D2	3.36	5.76E-03	↓	interferon beta-2-like [B. mutus]
ENSP00000332788-D1	3.36	5.76E-03	↓	anoctamin-9 [B. mutus]
ENSBTAP00000024853-D1	3.36	5.76E-03	↓	E3 ubiquitin-protein ligase TRIM17 [B. mutus]
ENSP00000291715-D1	3.36	5.76E-03	↓	claudin domain-containing protein 2 [B. mutus]
yakG036742	3.36	5.76E-03	↓	hypothetical protein M91_21642 [B. mutus]
ENSBTAP00000025749-D2	3.36	5.76E-03	↓	putative olfactory receptor 2W6-like [B. mutus]
ENSP00000360973-D1	3.36	5.76E-03	↓	type-2 angiotensin II receptor-like [B. mutus]
ENSBTAP00000023764-D1	3.36	5.76E-03	↓	Rho GTPase-activating protein 30, partial [B. mutus]

yakG015401	3.36	5.76E-03	↓	hypothetical protein M91_17538 [B. mutus]
ENSBTAP00000045246-D1	3.36	5.76E-03	↓	hypothetical protein M91_08307, partial [B. mutus]
ENSBTAP00000032695-D1	3.36	5.76E-03	↓	nanos homolog 1 isoform X1, partial [Ovis aries]
ENSBTAP00000029010-D1	3.36	5.76E-03	↓	glycine receptor subunit beta [B. mutus]
ENSBTAP00000011478-D1	3.36	5.76E-03	↓	Bcl-2-related ovarian killer protein, partial [B. mutus]
ENSBTAP00000053788-D1	3.36	5.76E-03	↓	cyclin-dependent kinase 6 [B. mutus]
ENSBTAP00000007883-D1	3.36	5.76E-03	↓	leucine-rich repeat-containing protein 69 [B. mutus]
ENSBTAP00000042640-D2	3.36	5.76E-03	↓	Metabotropic glutamate receptor 4, partial [B. mutus]
ENSBTAP00000041296-D2	3.36	5.76E-03	↓	hypothetical protein M91_09914, partial [B. mutus]
ENSBTAP00000002834-D1	3.36	5.76E-03	↓	UPF0764 protein C16orf89 homolog [B. mutus]
ENSBTAP00000040512-D1	3.36	5.76E-03	↓	putative hydroxypyruvate isomerase isoform X1 [B. mutus]
ENSP00000378074-D1	3.36	5.76E-03	↓	sodium/nucleoside cotransporter 1 [B. mutus]
ENSP00000309285-D1	3.36	5.76E-03	↓	coiled-coil domain-containing protein 96 [B. mutus]
ENSP00000419361-D1	3.36	5.76E-03	↓	Adenylate cyclase type 5, partial [B. mutus]
ENSP00000410885-D18	3.36	5.76E-03	↓	hypothetical protein M91_09980, partial [B. mutus]
ENSBTAP00000018241-D1	3.36	5.76E-03	↓	probable G-protein coupled receptor 37 [B. mutus]
ENSP00000358696-D1	3.36	5.76E-03	↓	plexin-A3 [B. mutus]
ENSBTAP00000008304-D1	3.39	7.84E-09	↓	nudC domain-containing protein 3 isoform X1 [B. mutus]
ENSP00000362139-D1	3.39	7.84E-09	↓	Ephrin type-A receptor 10 [B. mutus]
ENSP00000352956-D1	3.40	4.88E-25	↓	probable RNA-binding protein 23 isoform X1 [Bison bison bison]
ENSBTAP00000016108-D1	3.40	1.68E-13	↓	cell cycle checkpoint control protein RAD9A isoform X1 [B. mutus]
ENSBTAP00000004376-D1	3.40	3.65E-18	↓	UDP-xylose and UDP-N-acetylglucosamine transporter [B. mutus]
ENSBTAP00000027459-D1	3.41	1.34E-06	↓	ATP-binding cassette sub-family B member 6, mitochondrial [B. taurus]
ENSP00000357076-D1	3.41	1.34E-06	↓	Transgelin-2, partial [B. mutus]
ENSP00000366184-D1	3.42	1.29E-51	↓	ETS domain-containing protein Elk-1, partial [B. mutus]
ENSBTAP00000014602-D1	3.42	4.75E-09	↓	Histone deacetylase 10, partial [B. mutus]
ENSBTAP00000018112-D2	3.42	4.75E-09	↓	Zinc finger protein 805, partial [B. mutus]
ENSBTAP00000015051-D2	3.44	2.97E-21	↓	Homeodomain-interacting protein kinase 2 [B. mutus]
ENSBTAP00000009698-D1	3.46	1.80E-21	↓	lipoma HMGIC fusion partner-like 2 protein [B. taurus]
ENSP00000255641-D1	3.46	3.74E-14	↓	casein kinase I isoform gamma-2 [B. taurus]
ENSP00000375085-D2	3.46	8.11E-07	↓	unnamed protein product [Homo sapiens]
ENSP00000348602-D1	3.46	8.11E-07	↓	amphiphysin [Bison bison bison]
ENSBTAP00000042518-D1	3.46	2.43E-04	↓	transcription factor COE4 [Pantholops hodgsonii]
ENSBTAP00000018649-D3	3.46	2.43E-04	↓	conglutinin-like isoform X2 [B. taurus]
ENSP00000290759-D1	3.46	2.43E-04	↓	insulin gene enhancer protein ISL-2 [Orcinus orca]
ENSP00000376021-D1	3.46	2.43E-04	↓	THAP domain-containing protein 8 [B. mutus]
ENSP00000378508-D1	3.46	2.43E-04	↓	DnaJ-like protein subfamily C member 22, partial [B. mutus]
ENSP00000327442-D1	3.48	2.26E-14	↓	formin-like protein 1 [Camelus bactrianus]
ENSBTAP00000026019-D1	3.49	5.02E-17	↓	CKLF-like MARVEL transmembrane domain-containing protein 6, partial [B. mutus]
ENSP00000352685-D1	3.49	1.75E-09	↓	TNF receptor-associated factor 2 [B. mutus]
ENSBTAP00000019989-D1	3.49	1.75E-09	↓	hypothetical protein M91_05948, partial [B. mutus]
ENSP00000380288-D1	3.49	1.37E-14	↓	hypothetical protein M91_00213 [B. mutus]
ENSP00000351163-D1	3.50	3.80E-12	↓	Collagen alpha-1(XI) chain [B. mutus]
ENSP00000339353-D1	3.50	3.80E-12	↓	Cleavage and polyadenylation specificity factor subunit 1, partial [B. mutus]
ENSP00000351650-D6	3.52	1.06E-09	↓	diphosphoinositol polyphosphate phosphohydrolase 2 isoform X1 [Cavia porcellus]
ENSP00000258411-D1	3.52	1.06E-09	↓	protein Wnt-10a [B. mutus]
ENSP00000399364-D1	3.55	6.39E-10	↓	ADAMTS-like protein 5 isoform X3 [Bubalus bubalis]
ENSP00000302599-D1	3.55	2.95E-07	↓	alpha-(1,3)-fucosyltransferase 9 [Ovis aries]
ENSBTAP00000028212-D1	3.55	2.95E-07	↓	Meiosis inhibitor protein 1 [B. mutus]
ENSP00000379057-D1	3.55	1.45E-04	↓	Smith-Magenis syndrome chromosomal region candidate 7 protein, partial [B. mutus]
ENSBTAP00000006547-D1	3.55	1.45E-04	↓	palmitoyltransferase ZDHHC23 [B. taurus]
ENSP00000255858-D1	3.55	1.45E-04	↓	SEC14-like protein 4 isoform X1 [B. mutus]
ENSBTAP00000008462-D1	3.55	1.45E-04	↓	T-cell surface glycoprotein CD3 delta chain [B. mutus]
ENSP00000310696-D1	3.55	1.45E-04	↓	beta-1,4-galactosyltransferase 2 [B. taurus]
ENSP00000372160-D1	3.55	1.45E-04	↓	docking protein 6 isoform X2 [B. taurus]

ENSP00000006526-D1	3.55	1.45E-04	↓	WD repeat-containing protein 54 isoform X1 [Bison bison bison]
ENSBTAP00000013313-D1	3.55	3.41E-03	↓	FANCD2 opposite strand protein [Ovis aries]
ENSP00000398108-D1	3.55	3.41E-03	↓	zinc finger protein 777 [B. taurus]
ENSBTAP00000026853-D1	3.55	3.41E-03	↓	kelch-like protein 22 [B. taurus]
ENSBTAP00000014083-D1	3.55	3.41E-03	↓	DC-STAMP domain-containing protein 1, partial [B. mutus]
ENSBTAP00000023124-D1	3.55	3.41E-03	↓	leucine-rich repeat LGI family member 2 [Bison bison bison]
ENSP00000396214-D1	3.55	3.41E-03	↓	Metalloreductase STEAP3, partial [B. mutus]
ENSP00000331298-D1	3.55	3.41E-03	↓	UBA-like domain-containing protein 2 [B. mutus]
ENSP00000396290-D1	3.55	3.41E-03	↓	sodium/glucose cotransporter 2 isoform X1 [B. mutus]
ENSP00000357583-D1	3.55	3.41E-03	↓	Type I inositol-1,4,5-trisphosphate 5-phosphatase, partial [B. mutus]
yakG032360	3.55	3.41E-03	↓	V-set and immunoglobulin domain-containing protein 10-like protein [B. mutus]
ENSBTAP00000047883-D2	3.55	3.41E-03	↓	Tubulin alpha-1D chain, partial [B. mutus]
ENSBTAP00000041306-D1	3.55	3.41E-03	↓	Protein FAM3B [B. mutus]
ENSBTAP00000048040-D1	3.55	3.41E-03	↓	Calmodulin, partial [B. mutus]
ENSBTAP00000011314-D1	3.55	3.41E-03	↓	protein RCC2 [B. taurus]
ENSBTAP00000046490-D3	3.55	3.41E-03	↓	GTP-binding nuclear protein Ran-like [B. mutus]
ENSBTAP00000003763-D1	3.55	3.41E-03	↓	tumor necrosis factor ligand superfamily member 4 [B. taurus]
ENSP00000404429-D3	3.55	3.41E-03	↓	BCL2/adenovirus E1B 19 kDa protein-interacting protein 3-like [Pantholops hodgsonii]
ENSBTAP00000006005-D1	3.55	3.41E-03	↓	RING finger protein 183 [B. mutus]
ENSBTAP00000009286-D1	3.55	3.41E-03	↓	glycoprotein hormone alpha-2 [B. mutus]
ENSBTAP00000008115-D1	3.55	3.41E-03	↓	cyclic AMP-responsive element-binding protein 3-like protein 3 [B. mutus]
ENSBTAP00000047569-D1	3.55	3.41E-03	↓	Zinc transporter ZIP13 [B. mutus]
ENSP00000252797-D55	3.55	3.41E-03	↓	hypothetical protein M91_19685, partial [B. mutus]
ENSP00000252244-D8	3.55	3.41E-03	↓	Keratin, type II cytoskeletal 5, partial [B. mutus]
ENSBTAP00000013281-D1	3.55	3.41E-03	↓	Receptor expression-enhancing protein 2, partial [B. mutus]
ENSP00000293215-D1	3.55	3.41E-03	↓	Cell division protein kinase 3, partial [B. mutus]
ENSP00000338627-D1	3.55	3.41E-03	↓	Orphan sodium- and chloride-dependent neurotransmitter transporter NTT5, partial [B. mutus]
ENSP00000353557-D1	3.55	3.41E-03	↓	Solute carrier family 35 member F1, partial [B. mutus]
ENSBTAP00000013145-D1	3.55	3.41E-03	↓	DNA repair protein XRCC3 [B. mutus]
ENSBTAP00000004406-D1	3.55	3.41E-03	↓	potassium voltage-gated channel subfamily G member 1 [B. taurus]
ENSBTAP00000023209-D1	3.55	3.41E-03	↓	Monocarboxylate transporter 4 [B. mutus]
ENSP00000363189-D1	3.55	3.41E-03	↓	transmembrane protein 222 [B. mutus]
ENSP00000279230-D1	3.55	3.41E-03	↓	1-phosphatidylinositol 4,5-bisphosphate phosphodiesterase beta-3 [B. taurus]
ENSP00000265340-D2	3.55	3.41E-03	↓	Pituitary homeobox 1, partial [B. mutus]
ENSBTAP00000023402-D1	3.55	3.41E-03	↓	Corticosteroid-binding globulin [B. mutus]
ENSBTAP00000041217-D1	3.55	3.41E-03	↓	hypothetical protein M91_00129, partial [B. mutus]
ENSP00000303158-D1	3.55	3.41E-03	↓	coiled-coil domain-containing protein 8-like [B. mutus]
ENSBTAP00000017906-D1	3.55	3.41E-03	↓	neural cell adhesion molecule L1 isoform X1 [B. mutus]
ENSP00000409021-D1	3.55	3.41E-03	↓	hypothetical protein M91_05071, partial [B. mutus]
ENSBTAP00000031485-D13	3.55	3.41E-03	↓	hypothetical protein M91_02593, partial [B. mutus]
ENSBTAP00000046236-D2	3.55	3.41E-03	↓	testis-specific histone 2a-like [B. taurus]
ENSP00000261483-D2	3.55	3.41E-03	↓	alpha-mannosidase 2x [B. mutus]
ENSBTAP00000028688-D1	3.55	3.41E-03	↓	protein reprimo [B. taurus]
ENSP00000361110-D1	3.55	3.41E-03	↓	globoside alpha-1,3-N-acetylgalactosaminyltransferase 1 isoform X1 [B. mutus]
ENSBTAP00000053606-D1	3.55	3.41E-03	↓	Frizzled-4, partial [B. mutus]
ENSBTAP00000051357-D1	3.55	3.41E-03	↓	hypothetical protein M91_16622, partial [B. mutus]
ENSP00000362888-D1	3.55	3.41E-03	↓	2-aminoethanethiol dioxygenase, partial [B. mutus]
ENSP00000166139-D1	3.55	3.41E-03	↓	Follistatin-related protein 3, partial [B. mutus]
ENSP00000309189-D1	3.55	3.41E-03	↓	Spermatid-associated protein [B. mutus]
ENSBTAP00000013854-D1	3.55	3.41E-03	↓	hypothetical protein M91_07635, partial [B. mutus]
ENSBTAP00000025779-D1	3.55	3.41E-03	↓	Potassium channel subfamily K member 10, partial [B. mutus]
yakA16304	3.55	3.41E-03	↓	hypothetical protein M91_21671 [B. mutus]
ENSP00000393742-D7	3.55	3.41E-03	↓	Collagen alpha-4(VI) chain, partial [B. mutus]
ENSP00000323821-D1	3.55	3.41E-03	↓	hypothetical protein M91_09003, partial [B. mutus]
ENSBTAP00000026263-D1	3.55	3.41E-03	↓	Nuclear RNA export factor 3, partial [B. mutus]

ENSBTAP00000020522-D1	3.55	3.41E-03	↓	Long-chain fatty acid transport protein 4, partial [B. mutus]
ENSBTAP00000016606-D1	3.55	3.41E-03	↓	Oxidative stress-induced growth inhibitor 1, partial [B. mutus]
ENSBTAP00000050270-D1	3.55	3.41E-03	↓	DDB1- and CUL4-associated factor 12-like protein 2-like [B. mutus]
ENSP00000401565-D1	3.55	3.41E-03	↓	ectonucleoside triphosphate diphosphohydrolase 3 [B. mutus]
ENSBTAP00000022032-D1	3.55	3.41E-03	↓	nitrogen permease regulator 3-like protein [B. mutus]
ENSP00000248668-D1	3.55	3.41E-03	↓	Leucine-rich repeat and fibronectin type III domain-containing protein 1, partial [B. mutus]
ENSBTAP00000023564-D1	3.55	3.41E-03	↓	c-C motif chemokine 22 [B. mutus]
ENSP00000390787-D1	3.55	3.41E-03	↓	protein FAM170A [B. mutus]
ENSP00000411847-D1	3.55	3.41E-03	↓	BAH and coiled-coil domain-containing protein 1 [B. mutus]
ENSP00000262032-D1	3.55	3.41E-03	↓	zinc finger protein Eos [B. taurus]
ENSP00000320849-D1	3.55	3.41E-03	↓	coiled-coil domain-containing protein 184 [B. taurus]
ENSP00000325296-D1	3.55	3.41E-03	↓	Polycystic kidney disease 2-like 1 protein, partial [B. mutus]
ENSP00000369395-D1	3.55	3.41E-03	↓	leucine-rich repeat-containing protein 19 precursor [B. taurus]
ENSBTAP00000047565-D2	3.55	3.41E-03	↓	hypothetical protein M91_06254, partial [B. mutus]
ENSBTAP00000008999-D1	3.55	3.41E-03	↓	Putative ATP-dependent RNA helicase DHX34 [B. mutus]
ENSP00000385695-D1	3.55	3.41E-03	↓	putative SEC14-like protein 6 [B. mutus]
ENSBTAP00000025356-D1	3.58	3.87E-10	↓	Serine/threonine-protein kinase LMTK1, partial [B. mutus]
ENSBTAP00000023285-D1	3.58	7.42E-24	↓	Zinc finger protein 236, partial [B. mutus]
ENSBTAP00000035832-D1	3.59	1.11E-15	↓	NF-kappa-B inhibitor epsilon, partial [B. mutus]
ENSP00000259216-D1	3.59	1.78E-07	↓	Cryptic protein, partial [B. mutus]
ENSP00000375080-D1	3.61	2.34E-10	↓	serine/threonine-protein phosphatase 2A regulatory subunit B" subunit beta [B. mutus]
ENSBTAP00000011594-D1	3.62	5.47E-19	↓	ATP synthase mitochondrial F1 complex assembly factor 2 isoform X1 [Bison bison bison]
ENSP00000290776-D1	3.64	4.44E-22	↓	copine-2 [B. mutus]
ENSP00000326846-D1	3.64	1.87E-13	↓	uncharacterized protein C14orf28 homolog isoform X1 [Ovis aries]
ENSBTAP00000022486-D1	3.64	1.41E-10	↓	WD repeat-containing protein 65, partial [B. mutus]
ENSP00000234923-D1	3.64	1.07E-07	↓	UPF0577 protein KIAA1324 [B. mutus]
ENSP00000262765-D1	3.64	1.07E-07	↓	glutamine-rich protein 2 [B. mutus]
ENSP00000415252-D1	3.64	1.07E-07	↓	KN motif and ankyrin repeat domain-containing protein 2 [B. mutus]
ENSBTAP00000019765-D1	3.64	8.67E-05	↓	Glucosylceramidase [B. mutus]
ENSBTAP00000010571-D2	3.64	8.67E-05	↓	cadherin EGF LAG seven-pass G-type receptor 1 isoform X1 [B. taurus]
ENSBTAP00000006080-D1	3.64	8.67E-05	↓	Lethal(2) giant larvae protein-like protein 1, partial [B. mutus]
ENSBTAP00000006353-D1	3.66	1.13E-13	↓	homeobox protein Hox-D3 [B. mutus]
ENSP00000381652-D1	3.68	8.75E-32	↓	Protein TSSC1, partial [B. mutus]
ENSP00000417628-D2	3.68	6.47E-08	↓	contactin-associated protein-like 3-like [B. mutus]
ENSP00000402831-D1	3.68	6.47E-08	↓	Metal transporter CNNM4, partial [B. mutus]
ENSBTAP00000041430-D1	3.71	1.03E-41	↓	proline-rich protein 19 [B. taurus]
ENSBTAP00000015662-D1	3.72	3.89E-08	↓	Thioredoxin reductase 3, partial [B. mutus]
ENSBTAP00000048973-D1	3.72	3.89E-08	↓	Prostaglandin F2 receptor negative regulator, partial [B. mutus]
ENSBTAP00000025570-D1	3.72	3.89E-08	↓	potassium voltage-gated channel subfamily S member 3 [B. mutus]
ENSBTAP00000034724-D1	3.72	5.19E-05	↓	F-box only protein 47 isoform X1 [B. mutus]
ENSP00000216133-D1	3.72	5.19E-05	↓	DNA dC->gi|440903021|gb|ELR53735.1| DNA dC->gi|555975049|ref|XP_005899509.1| chromobox protein homolog 7 [B. mutus]
ENSBTAP00000026608-D1	3.72	5.19E-05	↓	interleukin-7 receptor subunit alpha [B. mutus]
ENSP00000370936-D2	3.72	2.01E-03	↓	Transcription factor E2F6 [B. mutus]
ENSBTAP00000048041-D1	3.72	2.01E-03	↓	.
ENSBTAP00000048301-D1	3.72	2.01E-03	↓	TPA: retinoic acid receptor, beta-like [B. taurus]
ENSBTAP00000025148-D1	3.72	2.01E-03	↓	Peroxisomal membrane protein 11C, partial [B. mutus]
ENSP00000394624-D1	3.72	2.01E-03	↓	mu-type opioid receptor [Ursus maritimus]
ENSBTAP00000050199-D4	3.72	2.01E-03	↓	transcription elongation factor B polypeptide 2-like isoform X1 [Pan troglodytes]
ENSBTAP00000012054-D1	3.72	2.01E-03	↓	Cell surface A33 antigen, partial [B. mutus]
ENSP00000216144-D1	3.72	2.01E-03	↓	calcium-binding protein 7 [Mus musculus]
ENSP00000371219-D1	3.72	2.01E-03	↓	rho-related GTP-binding protein RhoH [Ovis aries]
ENSP00000392568-D3	3.72	2.01E-03	↓	hypothetical protein M91_08022, partial [B. mutus]
ENSBTAP00000006031-D1	3.72	2.01E-03	↓	glycosyl-phosphatidylinositol-anchored molecule-like protein-like [B. mutus]
ENSBTAP00000004653-D1	3.72	2.01E-03	↓	DNA polymerase lambda isoform X1 [B. mutus]

ENSBTAP00000004287-D1	3.72	2.01E-03	↓	Carbohydrate sulfotransferase 15 [B. mutus]
ENSP00000222249-D1	3.72	2.01E-03	↓	Small conductance calcium-activated potassium channel protein 1, partial [B. mutus]
ENSBTAP00000052585-D1	3.72	2.01E-03	↓	olfactory receptor 4X2-like [B. mutus]
ENSP00000271610-D1	3.72	2.01E-03	↓	receptor-type tyrosine-protein phosphatase C, partial [B. mutus]
ENSBTAP00000002409-D1	3.72	2.01E-03	↓	Epidermal growth factor receptor kinase substrate 8-like protein 3, partial [B. mutus]
ENSBTAP00000001095-D1	3.72	2.01E-03	↓	Sedoheptulokinase, partial [B. mutus]
ENSBTAP00000004058-D1	3.72	2.01E-03	↓	FERM and PDZ domain-containing protein 3, partial [B. mutus]
ENSBTAP00000000510-D2	3.72	2.01E-03	↓	hypothetical protein M91_18032, partial [B. mutus]
ENSP00000325776-D1	3.72	2.01E-03	↓	serine-rich and transmembrane domain-containing protein 1 [B. taurus]
ENSBTAP00000001840-D1	3.72	2.01E-03	↓	Proton-associated sugar transporter A, partial [B. mutus]
ENSBTAP00000020324-D1	3.72	2.01E-03	↓	Sodium-independent sulfate anion transporter [B. mutus]
ENSP00000307650-D1	3.72	2.01E-03	↓	metastasis-suppressor KiSS-1 [B. mutus]
ENSP00000296145-D2	3.72	2.01E-03	↓	Teratocarcinoma-derived growth factor 1, partial [B. mutus]
ENSBTAP00000034555-D8	3.72	2.01E-03	↓	hypothetical protein M91_15076, partial [B. mutus]
ENSP00000314560-D1	3.72	2.01E-03	↓	MAP6 domain-containing protein 1 isoform X2 [Ovis aries musimon]
ENSBTAP00000004042-D1	3.72	2.01E-03	↓	LIM/homeobox protein Lhx5, partial [B. mutus]
ENSP00000293379-D1	3.72	2.01E-03	↓	integrin alpha-5, partial [B. mutus]
ENSP00000389175-D1	3.72	2.01E-03	↓	glycerate kinase isoform X1 [B. mutus]
ENSBTAP00000009885-D1	3.72	2.01E-03	↓	Peroxisomal membrane protein 2 [B. mutus]
ENSBTAP00000047142-D1	3.72	2.01E-03	↓	hypothetical protein M91_05764, partial [B. mutus]
ENSBTAP00000001191-D1	3.72	2.01E-03	↓	proteinase-activated receptor 3 [B. mutus]
ENSBTAP00000015039-D1	3.72	2.01E-03	↓	Gap junction alpha-10 protein, partial [B. mutus]
ENSBTAP00000049815-D1	3.72	2.01E-03	↓	Olfactory receptor 4E1, partial [B. mutus]
ENSBTAP00000020285-D1	3.72	2.01E-03	↓	Delta-1-pyrroline-5-carboxylate dehydrogenase, mitochondrial, partial [B. mutus]
ENSBTAP00000000499-D1	3.72	2.01E-03	↓	Zinc finger protein 212, partial [B. mutus]
ENSP00000385591-D1	3.72	2.01E-03	↓	coiled-coil domain-containing protein 178 [B. mutus]
ENSP00000379815-D1	3.72	2.01E-03	↓	transmembrane protein 102 [B. mutus]
ENSP00000294725-D1	3.72	2.01E-03	↓	Potassium channel subfamily T member 2, partial [B. mutus]
ENSP00000313490-D1	3.72	2.01E-03	↓	Phosphoribosylformylglycinamidine synthase [B. mutus]
ENSP00000366620-D1	3.72	2.01E-03	↓	GDH/6PGL endoplasmic bifunctional protein, partial [B. mutus]
ENSP00000269856-D1	3.72	2.01E-03	↓	protein fem-1 homolog A isoform X1 [B. mutus]
ENSBTAP00000037251-D1	3.72	2.01E-03	↓	HEAT repeat-containing protein 7A [B. mutus]
ENSP00000333183-D3	3.72	2.01E-03	↓	putative methyl-CpG-binding domain protein 3-like 3 [Pantholops hodgsonii]
ENSP00000261937-D1	3.72	2.01E-03	↓	Vascular endothelial growth factor receptor 3 [B. mutus]
ENSP00000351345-D1	3.72	2.01E-03	↓	protein FAM150A [Bubalus bubalis]
ENSBTAP00000017571-D1	3.72	2.01E-03	↓	Platelet glycoprotein V, partial [B. mutus]
ENSP00000315383-D1	3.72	2.01E-03	↓	Retroviral-like aspartic protease 1, partial [B. mutus]
ENSBTAP00000021892-D1	3.72	2.01E-03	↓	7-dehydrocholesterol reductase [B. mutus]
ENSBTAP00000027848-D1	3.72	2.01E-03	↓	insulin-like 3 [B. mutus]
ENSBTAP00000048068-D1	3.72	2.01E-03	↓	hypothetical protein M91_10369, partial [B. mutus]
ENSP00000313158-D1	3.72	2.01E-03	↓	zinc finger protein 366 [B. mutus]
ENSP00000305193-D1	3.72	2.01E-03	↓	HCLS1-binding protein 3, partial [B. mutus]
ENSP00000368952-D1	3.72	2.01E-03	↓	Stomatin-like protein 3, partial [B. mutus]
ENSBTAP00000050702-D8	3.72	2.01E-03	↓	hypothetical protein M91_13705 [B. mutus]
ENSP00000366283-D1	3.72	2.01E-03	↓	SLIT and NTRK-like protein 5 [B. mutus]
ENSP00000363727-D1	3.72	2.01E-03	↓	StAR-related lipid transfer protein 8, partial [B. mutus]
ENSBTAP00000036940-D1	3.72	2.01E-03	↓	Golgi to ER traffic protein 4-like protein, partial [B. mutus]
ENSBTAP00000001730-D1	3.72	2.01E-03	↓	Rhodopsin, partial [B. mutus]
ENSBTAP00000002302-D2	3.72	2.01E-03	↓	transcription factor jun-D [Balaenoptera acutorostrata scammoni]
ENSBTAP00000049195-D1	3.72	2.01E-03	↓	Zinc finger protein 446, partial [B. mutus]
ENSP00000329117-D1	3.74	1.11E-77	↓	APC membrane recruitment protein 1 [B. mutus]
ENSBTAP00000051893-D1	3.76	8.99E-15	↓	kynurenine--oxoglutarate transaminase 1-like, partial [B. mutus]
ENSP00000297239-D1	3.78	1.70E-86	↓	synaptotagmin-like protein 3 isoform X1 [B. mutus]
ENSBTAP00000013230-D3	3.79	3.12E-28	↓	hypothetical protein M91_15035, partial [B. mutus]
ENSP00000393821-D1	3.80	1.59E-18	↓	GRAM domain-containing protein 1A [B. mutus]

ENSP00000378492-D1	3.80	1.42E-08	↓	metabotropic glutamate receptor 2 precursor [B. taurus]
ENSP00000401980-D1	3.80	1.42E-08	↓	Mitochondrial antiviral-signaling protein [B. mutus]
ENSBTAP00000051670-D1	3.80	3.11E-05	↓	G-protein coupled receptor 56 isoform X2 [B. mutus]
ENSBTAP00000053381-D1	3.82	1.96E-15	↓	Putative protein KIAA0556, partial [B. mutus]
ENSBTAP00000021132-D1	3.82	4.08E-12	↓	protein canopy homolog 3 [Bubalus bubalis]
ENSBTAP00000018110-D1	3.84	2.50E-29	↓	lamin-B2 [B. mutus]
ENSBTAP00000042384-D1	3.84	8.54E-09	↓	tRNA pseudouridine synthase-like 1, partial [B. mutus]
ENSBTAP00000003881-D1	3.86	2.68E-33	↓	alanine aminotransferase 2 isoform X1 [B. taurus]
ENSP00000364016-D1	3.87	1.18E-03	↓	proteasome subunit beta type-8 [B. mutus]
ENSP00000336812-D1	3.87	1.18E-03	↓	Protein FAM19A5, partial [B. mutus]
ENSP00000305714-D1	3.87	1.18E-03	↓	Bone morphogenetic protein 1, partial [B. mutus]
ENSP00000287766-D1	3.87	1.18E-03	↓	sodium- and chloride-dependent GABA transporter 1 [B. mutus]
ENSBTAP00000052597-D1	3.87	1.18E-03	↓	Rhabdoid tumor deletion region protein 1, partial [B. mutus]
ENSP00000007264-D1	3.87	1.18E-03	↓	RNA pseudouridylate synthase domain-containing protein 1 [B. mutus]
ENSBTAP00000004230-D1	3.87	1.18E-03	↓	zinc finger protein 132 isoform X2 [Bison bison bison]
ENSBTAP00000025119-D1	3.87	1.18E-03	↓	immunoglobulin superfamily member 6 [B. mutus]
ENSP00000377725-D60	3.87	1.18E-03	↓	Zinc finger protein with KRAB and SCAN domains 4, partial [B. mutus]
ENSP00000345096-D1	3.87	1.18E-03	↓	Inosine-5'-monophosphate dehydrogenase 1, partial [B. mutus]
ENSBTAP00000022785-D1	3.87	1.18E-03	↓	somatostatin receptor type 2 [B. mutus]
ENSP00000409007-D1	3.87	1.18E-03	↓	Glial cell line-derived neurotrophic factor, partial [B. mutus]
ENSBTAP00000043151-D1	3.87	1.18E-03	↓	Coiled-coil domain-containing protein 159, partial [B. mutus]
ENSBTAP00000018089-D1	3.87	1.18E-03	↓	vascular endothelial growth factor B186 precursor [B. taurus]
ENSP00000322249-D1	3.87	1.18E-03	↓	protein orai-3 [B. mutus]
ENSBTAP00000021753-D1	3.87	1.18E-03	↓	Syntaxin-10 [B. mutus]
ENSP00000220676-D1	3.87	1.18E-03	↓	Oxygen-regulated protein 1 [B. mutus]
ENSP00000316244-D1	3.87	1.18E-03	↓	5-hydroxytryptamine receptor 1A [B. taurus]
ENSP00000278865-D1	3.87	1.18E-03	↓	Membrane-spanning 4-domains subfamily A member 3 [B. mutus]
ENSBTAP00000032180-D1	3.87	1.18E-03	↓	Geminin coiled-coil domain-containing protein 1, partial [B. mutus]
ENSBTAP00000040788-D1	3.87	1.18E-03	↓	probable UDP-sugar transporter protein SLC35A4 [B. taurus]
ENSBTAP00000027924-D1	3.87	1.18E-03	↓	Homeobox protein aristaless-like 3, partial [B. mutus]
ENSP00000356694-D1	3.87	1.18E-03	↓	tumor necrosis factor ligand superfamily member 6 [B. mutus]
ENSP00000303549-D1	3.87	1.18E-03	↓	probable G-protein coupled receptor 82 [B. taurus]
ENSP00000376544-D1	3.87	1.18E-03	↓	B-cell antigen receptor complex-associated protein beta chain isoform X1 [B. mutus]
ENSP00000414253-D1	3.87	1.18E-03	↓	Cancer/testis antigen 47A, partial [B. mutus]
ENSP00000344637-D1	3.87	1.18E-03	↓	hypothetical protein M91_07480, partial [B. mutus]
ENSBTAP00000015555-D1	3.87	1.18E-03	↓	ATP-dependent DNA helicase Q5 isoform X1 [B. mutus]
ENSBTAP00000010730-D1	3.87	1.18E-03	↓	calcium-activated chloride channel regulator 1 [B. mutus]
ENSBTAP00000023106-D1	3.87	1.18E-03	↓	Putative polypeptide N-acetylgalactosaminyltransferase 8, partial [B. mutus]
ENSP00000365883-D1	3.87	1.18E-03	↓	nucleoside diphosphate-linked moiety X motif 8, mitochondrial [B. taurus]
ENSBTAP00000012751-D1	3.87	1.18E-03	↓	olfactory receptor 5 [B. taurus]
ENSBTAP00000023267-D1	3.87	1.18E-03	↓	Fas apoptotic inhibitory molecule 2, partial [B. mutus]
ENSP00000216487-D1	3.87	1.18E-03	↓	Ras and Rab interactor 3, partial [B. mutus]
ENSBTAP00000004195-D1	3.87	1.18E-03	↓	Sialic acid-binding Ig-like lectin 10 [B. mutus]
ENSBTAP00000051259-D60	3.87	1.18E-03	↓	hypothetical protein M91_20110 [B. mutus]
ENSP00000276480-D1	3.87	1.18E-03	↓	Suppression of tumorigenicity 18 protein, partial [B. mutus]
yakG016733	3.87	1.18E-03	↓	hypothetical protein M91_08440 [B. mutus]
ENSBTAP00000006222-D1	3.87	1.18E-03	↓	interleukin-12 subunit beta isoform X1 [B. mutus]
ENSBTAP00000043403-D4	3.87	1.18E-03	↓	hypothetical protein M91_16321, partial [B. mutus]
ENSP00000306496-D1	3.87	1.18E-03	↓	ras-related protein Rab-33B [Vicugna pacos]
ENSP00000270530-D1	3.87	1.18E-03	↓	EVI5-like protein, partial [B. mutus]
ENSBTAP00000006089-D2	3.87	1.18E-03	↓	Hyaluronidase PH-20 [B. mutus]
ENSBTAP00000025498-D1	3.87	1.18E-03	↓	RNA-binding protein MEX3A [B. mutus]
ENSBTAP00000031862-D1	3.87	1.18E-03	↓	bile salt sulfotransferase-like [B. mutus]
ENSP00000284425-D3	3.87	1.18E-03	↓	ATP-binding cassette sub-family A member 9, partial [B. mutus]
ENSP00000341479-D1	3.87	1.18E-03	↓	ATP-sensitive inward rectifier potassium channel 14 [B. mutus]

ENSP00000268679-D1	3.87	1.18E-03	↓	Protein CBFA2T3, partial [B. mutus]
ENSP00000345195-D1	3.87	1.85E-05	↓	ubiquilin-2 isoform X1 [Trichechus manatus latirostris]
ENSP00000379712-D1	3.87	1.85E-05	↓	IQ motif and SEC7 domain-containing protein 2 [Pan paniscus]
ENSP00000401313-D1	3.87	1.85E-05	↓	cysteine and tyrosine-rich protein 1-like isoform X2 [Bubalus bubalis]
ENSP00000308549-D1	3.87	1.85E-05	↓	adenosine receptor A1 isoform X1 [B. mutus]
ENSP00000357130-D1	3.87	1.85E-05	↓	spectrin alpha chain, erythrocytic 1 [B. mutus]
ENSP00000294964-D1	3.87	1.85E-05	↓	protein kinase domain-containing protein, cytoplasmic [Bubalus bubalis]
ENSP00000379566-D1	3.90	8.92E-13	↓	Coiled-coil alpha-helical rod protein 1, partial [B. mutus]
ENSP00000389128-D1	3.92	4.19E-57	↓	Leucine-rich repeat-containing protein 15, partial [B. mutus]
ENSP00000307235-D2	3.92	5.38E-13	↓	hypothetical protein M91_09390, partial [B. mutus]
ENSBTAP00000032431-D1	3.92	5.38E-13	↓	paraplegin, partial [B. mutus]
ENSP00000267484-D1	3.92	5.38E-13	↓	Reticulon-1, partial [B. mutus]
ENSP00000264607-D1	3.94	1.86E-09	↓	ankyrin repeat and SOCS box protein 1 [Pantholops hodgsonii]
ENSP00000356257-D1	3.94	1.86E-09	↓	leiomodin-1 [B. mutus]
ENSP00000382675-D1	3.94	1.11E-05	↓	TPA: hypothetical protein BOS_9159 [B. taurus]
ENSBTAP00000044999-D1	3.94	1.11E-05	↓	jmjC domain-containing protein 8 [Bubalus bubalis]
ENSBTAP00000002533-D60	3.94	1.11E-05	↓	Zinc finger protein 543, partial [B. mutus]
ENSP00000345868-D1	3.96	1.95E-13	↓	gap junction beta-4 protein [B. mutus]
ENSP00000262095-D2	3.98	1.12E-09	↓	One cut domain family member 2, partial [B. mutus]
ENSBTAP00000028978-D1	3.98	1.12E-09	↓	polypeptide N-acetylgalactosaminyltransferase 14 [Bubalus bubalis]
ENSBTAP00000015167-D1	3.99	5.67E-45	↓	hypothetical protein M91_01537 [B. mutus]
ENSP00000346508-D1	4.01	6.74E-10	↓	Platelet-derived growth factor subunit A, partial [B. mutus]
ENSBTAP00000046239-D3	4.01	6.74E-10	↓	hypothetical protein M91_20376, partial [B. mutus]
ENSBTAP00000045493-D1	4.01	6.64E-06	↓	Putative protein KIAA2012-like protein, partial [B. mutus]
ENSP00000328169-D1	4.01	6.64E-06	↓	protein jagged-2 isoform X1 [Bubalus bubalis]
ENSBTAP00000009155-D8	4.01	7.03E-04	↓	60S ribosomal protein L12-like [Capra hircus]
ENSBTAP00000001420-D1	4.01	7.03E-04	↓	cellular tumor antigen p53 [B. mutus]
ENSP00000358789-D1	4.01	7.03E-04	↓	SH3 and PX domain-containing protein 2A [B. mutus]
ENSP00000375053-D1	4.01	7.03E-04	↓	Transmembrane protein 203, partial [B. mutus]
ENSP00000327525-D14	4.01	7.03E-04	↓	olfactory receptor 4F21-like [B. mutus]
ENSP00000002829-D1	4.01	7.03E-04	↓	semaphorin-3F isoform X1 [B. taurus]
ENSBTAP00000012340-D1	4.01	7.03E-04	↓	diphthamide biosynthesis protein 1 [B. mutus]
ENSBTAP00000029577-D1	4.01	7.03E-04	↓	P-selectin glycoprotein ligand 1, partial [B. mutus]
ENSP00000219919-D1	4.01	7.03E-04	↓	aquaporin-9 isoform X1 [B. mutus]
ENSP00000324767-D1	4.01	7.03E-04	↓	calponin homology domain-containing protein 2 [B. mutus]
ENSP00000413635-D1	4.01	7.03E-04	↓	Solute carrier family 43 member 3 [B. mutus]
ENSBTAP00000017320-D1	4.01	7.03E-04	↓	cholecystokinin [B. mutus]
ENSBTAP00000019137-D1	4.01	7.03E-04	↓	myotubularin-related protein 9 [B. taurus]
ENSP00000370739-D1	4.01	7.03E-04	↓	beta-enolase isoform X3 [B. mutus]
ENSBTAP00000033947-D3	4.01	7.03E-04	↓	Protein Shroom4, partial [B. mutus]
ENSBTAP00000003779-D1	4.01	7.03E-04	↓	Enoyl-CoA hydratase domain-containing protein 2, mitochondrial [B. mutus]
ENSP00000416478-D1	4.01	7.03E-04	↓	Molybdenum cofactor biosynthesis protein 1, partial [B. mutus]
ENSBTAP00000017753-D1	4.01	7.03E-04	↓	homeobox protein SIX5 [B. taurus]
ENSP00000410938-D1	4.01	7.03E-04	↓	catechol O-methyltransferase [B. mutus]
ENSBTAP00000036449-D1	4.01	7.03E-04	↓	Protein FAM160B2, partial [B. mutus]
ENSBTAP00000004648-D1	4.01	7.03E-04	↓	coagulation factor XI [B. mutus]
ENSBTAP00000037103-D1	4.01	7.03E-04	↓	Extracellular sulfatase Sulf-1, partial [B. mutus]
ENSBTAP00000034542-D1	4.01	7.03E-04	↓	collagen alpha-1(XVIII) chain-like [B. mutus]
ENSP00000303102-D1	4.01	7.03E-04	↓	zinc finger and BTB domain-containing protein 46 [B. taurus]
ENSP00000389502-D1	4.01	7.03E-04	↓	DNA fragmentation factor subunit beta [B. mutus]
ENSBTAP00000002749-D1	4.01	7.03E-04	↓	metallophosphoesterase 1 [B. taurus]
ENSP00000390424-D1	4.01	7.03E-04	↓	EF-hand calcium-binding domain-containing protein 8, partial [B. mutus]
ENSP00000317905-D1	4.01	7.03E-04	↓	hypothetical protein M91_05252 [B. mutus]
ENSP00000360806-D1	4.01	7.03E-04	↓	Potassium voltage-gated channel subfamily B member 1, partial [B. mutus]
ENSBTAP00000047588-D1	4.01	7.03E-04	↓	L-gulonolactone oxidase, partial [B. mutus]

ENSBTAP00000011332-D1	4.01	7.03E-04	↓	Protein INCA1, partial [B. mutus]
ENSBTAP00000016092-D1	4.01	7.03E-04	↓	Metalloproteinase inhibitor 1, partial [B. mutus]
ENSBTAP00000024762-D1	4.01	7.03E-04	↓	Protein TSPEAR, partial [B. mutus]
ENSBTAP00000014051-D1	4.01	7.03E-04	↓	Mucin-1 [B. mutus]
ENSP00000406485-D1	4.01	7.03E-04	↓	smoothelin-like protein 1 [B. mutus]
ENSP00000331210-D1	4.01	7.03E-04	↓	brevican core protein isoform X1 [B. mutus]
ENSBTAP00000008171-D1	4.01	7.03E-04	↓	Protein THEMIS2, partial [B. mutus]
ENSP00000325941-D1	4.01	7.03E-04	↓	Synembryn-A [B. mutus]
ENSP00000292778-D1	4.01	7.03E-04	↓	UPF0249 protein ydjC-like protein, partial [B. mutus]
ENSP00000375073-D1	4.01	7.03E-04	↓	EP300-interacting inhibitor of differentiation 2 [B. mutus]
ENSBTAP00000040534-D1	4.01	7.03E-04	↓	40S ribosomal protein S6-like [B. mutus]
ENSP00000243349-D1	4.01	7.03E-04	↓	activin receptor type-1C precursor [B. taurus]
ENSP00000323680-D1	4.01	7.03E-04	↓	DDB1- and CUL4-associated factor 11 isoform X1 [B. mutus]
ENSP00000419204-D1	4.01	7.03E-04	↓	Tetratricopeptide repeat protein 6, partial [B. mutus]
ENSBTAP00000019284-D1	4.01	7.03E-04	↓	zinc finger protein 503 [B. mutus]
ENSBTAP00000003315-D1	4.01	7.03E-04	↓	inositol oxygenase [B. mutus]
ENSBTAP00000024852-D1	4.01	7.03E-04	↓	E3 ubiquitin-protein ligase TRIM11 [B. mutus]
ENSBTAP00000046304-D9	4.01	7.03E-04	↓	AFG3-like protein 1, partial [B. mutus]
ENSP00000282276-D1	4.01	7.03E-04	↓	methionine--tRNA ligase, mitochondrial isoform X1 [B. mutus]
ENSP00000393066-D1	4.01	7.03E-04	↓	Sodium-driven chloride bicarbonate exchanger [B. mutus]
ENSBTAP00000045596-D39	4.01	7.03E-04	↓	interferon alpha-2 [B. taurus]
ENSP00000296626-D1	4.01	7.03E-04	↓	solute carrier family 25 member 46 [Bison bison bison]
ENSP00000332530-D1	4.02	4.83E-22	↓	hypothetical protein M91_00091, partial [B. mutus]
ENSP00000301819-D1	4.05	2.54E-14	↓	tetratricopeptide repeat protein 21A isoform X1 [B. taurus]
ENSP00000238994-D1	4.06	1.64E-18	↓	Protein phosphatase 1 regulatory subunit 3C, partial [B. mutus]
ENSBTAP00000023791-D1	4.07	2.44E-10	↓	Layilin, partial [B. mutus]
ENSP00000400409-D1	4.07	2.44E-10	↓	Protein unc-13-like protein A, partial [B. mutus]
ENSBTAP00000013022-D1	4.07	2.44E-10	↓	Zinc finger protein ZXDC, partial [B. mutus]
ENSP00000303740-D1	4.12	3.29E-28	↓	beta-1,3-galactosyltransferase 1 [B. taurus]
ENSBTAP00000024829-D1	4.13	3.00E-37	↓	Serine/threonine-protein phosphatase 6 regulatory subunit 2, partial [B. mutus]
ENSP00000360683-D1	4.13	8.84E-11	↓	tyrosine-protein phosphatase non-receptor type 1 [B. mutus]
ENSP00000376103-D1	4.13	2.39E-06	↓	hypothetical protein M91_20423, partial [B. mutus]
ENSP00000382483-D4	4.13	2.39E-06	↓	interferon-induced transmembrane protein 3 isoform X2 [B. mutus]
ENSP00000360266-D1	4.13	2.39E-06	↓	Transcription factor AP-1 [B. mutus]
ENSBTAP00000020028-D1	4.13	2.39E-06	↓	Macrophage-stimulating protein receptor [B. mutus]
ENSBTAP00000003564-D1	4.13	2.39E-06	↓	hypothetical protein M91_18007, partial [B. mutus]
ENSBTAP00000046249-D1	4.13	4.19E-04	↓	Response to complement 32 protein, partial [B. mutus]
ENSP00000319851-D1	4.13	4.19E-04	↓	choline dehydrogenase, mitochondrial [B. taurus]
ENSBTAP00000023840-D1	4.13	4.19E-04	↓	ornithine decarboxylase isoform X1 [Ovis aries]
ENSBTAP00000053379-D1	4.13	4.19E-04	↓	Inositol monophosphatase 2, partial [B. mutus]
ENSP00000356140-D1	4.13	4.19E-04	↓	Neurofascin, partial [B. mutus]
yakG014317	4.13	4.19E-04	↓	Proteasome subunit beta type-6 [B. mutus]
ENSBTAP00000003689-D1	4.13	4.19E-04	↓	Liprin-alpha-4, partial [B. mutus]
ENSP00000300119-D1	4.13	4.19E-04	↓	Myosin-Ia [B. mutus]
ENSBTAP00000018376-D1	4.13	4.19E-04	↓	probable allantoicase [B. mutus]
ENSBTAP00000014986-D1	4.13	4.19E-04	↓	Matrilin-4 [B. mutus]
ENSBTAP00000040772-D1	4.13	4.19E-04	↓	Class E basic helix-loop-helix protein 41 [B. mutus]
ENSP00000302724-D1	4.13	4.19E-04	↓	insulin-like peptide INSL5 [B. mutus]
ENSBTAP00000023309-D1	4.13	4.19E-04	↓	fetuin-B [B. mutus]
yakG045552	4.13	4.19E-04	↓	UDP-glucuronic acid decarboxylase 1 [B. mutus]
ENSBTAP00000035294-D1	4.13	4.19E-04	↓	pre T-cell antigen receptor alpha [B. mutus]
ENSBTAP00000011333-D1	4.13	4.19E-04	↓	High affinity immunoglobulin gamma Fc receptor I, partial [B. mutus]
ENSBTAP00000026477-D1	4.13	4.19E-04	↓	Phosphoinositide 3-kinase adapter protein 1, partial [B. mutus]
ENSP00000380703-D1	4.13	4.19E-04	↓	bcl-2-modifying factor isoform X1 [B. mutus]
ENSBTAP00000002878-D1	4.13	4.19E-04	↓	F-box only protein 31, partial [B. mutus]

ENSP00000254846-D1	4.13	4.19E-04	↓	lysine-specific demethylase 6B, partial [B. mutus]
ENSP00000207870-D1	4.13	4.19E-04	↓	Xylulose kinase, partial [B. mutus]
ENSP00000252520-D1	4.13	4.19E-04	↓	Inverted formin-2 [B. mutus]
ENSP00000301050-D1	4.13	4.19E-04	↓	voltage-dependent L-type calcium channel subunit beta-3 [B. taurus]
ENSP00000249887-D1	4.13	4.19E-04	↓	c-C chemokine receptor type 11 [B. mutus]
ENSBTAP00000021458-D1	4.13	4.19E-04	↓	TPA: potassium voltage-gated channel, shaker-related subfamily, member 7-like [B. taurus]
ENSBTAP00000025660-D1	4.13	4.19E-04	↓	doublesex- and mab-3-related transcription factor C2 isoform X1 [B. mutus]
ENSBTAP00000022311-D1	4.13	4.19E-04	↓	Long-chain fatty acid transport protein 1 [B. mutus]
ENSP00000369553-D42	4.13	4.19E-04	↓	interferon tau-11-like [Bubalus bubalis]
ENSBTAP00000018523-D1	4.13	4.19E-04	↓	Sn1-specific diacylglycerol lipase alpha [B. mutus]
ENSP00000370522-D1	4.13	4.19E-04	↓	arylsulfatase H [B. mutus]
ENSP00000216862-D1	4.19	1.43E-06	↓	1,25-dihydroxyvitamin D(3) 24-hydroxylase, mitochondrial-like isoform X1 [B. mutus]
ENSP00000384262-D1	4.19	1.43E-06	↓	heparanase [Bison bison bison]
ENSP00000419313-D1	4.19	1.43E-06	↓	short transient receptor potential channel 1 isoformX1 [Canis lupus familiaris]
ENSP00000386538-D1	4.21	1.02E-20	↓	TBC1 domain family member 10B, partial [B. mutus]
ENSP00000390849-D1	4.21	3.43E-30	↓	1-acylglycerol-3-phosphate O-acyltransferase ABHD5 [B. mutus]
ENSP00000411197-D2	4.22	3.85E-49	↓	TBC1 domain family member 9B, partial [B. mutus]
ENSP00000286452-D1	4.22	1.92E-11	↓	Kinesin heavy chain isoform 5A [B. mutus]
ENSP00000341681-D1	4.22	1.92E-11	↓	Palmitoyltransferase ZDHHC7 [B. mutus]
ENSBTAP00000003637-D1	4.25	8.56E-07	↓	calpain-10 [B. mutus]
ENSP00000348489-D1	4.25	8.56E-07	↓	Putative fibrosin-1 long transcript protein, partial [B. mutus]
ENSBTAP00000020807-D1	4.25	8.56E-07	↓	transmembrane protein 127 [Camelus ferus]
ENSBTAP00000049744-D1	4.25	2.48E-04	↓	[Protein ADP-ribosylarginine] hydrolase, partial [B. mutus]
ENSBTAP00000048086-D33	4.25	2.48E-04	↓	hypothetical protein M91_12924, partial [B. mutus]
ENSBTAP00000024690-D1	4.25	2.48E-04	↓	von Willebrand factor C domain-containing protein 2-like protein, partial [B. mutus]
ENSBTAP00000053455-D1	4.25	2.48E-04	↓	Zinc finger protein 498, partial [B. mutus]
ENSBTAP00000006203-D1	4.25	2.48E-04	↓	progestin and adipoQ receptor family member 4 [B. mutus]
ENSBTAP00000039684-D1	4.25	2.48E-04	↓	T-cell activation Rho GTPase-activating protein, partial [B. mutus]
ENSP00000340434-D1	4.25	2.48E-04	↓	protein LDOC1L [B. taurus]
ENSBTAP00000025271-D1	4.25	2.48E-04	↓	CMRF35-like molecule 1, partial [B. mutus]
ENSBTAP00000006033-D2	4.25	2.48E-04	↓	transgelin-3 [B. taurus]
ENSP00000381036-D1	4.25	2.48E-04	↓	Calpain-7-like protein [B. mutus]
ENSP00000411848-D1	4.25	2.48E-04	↓	E3 ubiquitin-protein ligase MARCH8 isoform X1 [B. mutus]
ENSP00000350467-D1	4.25	2.48E-04	↓	N-acetyltransferase 9 [B. mutus]
ENSBTAP00000036630-D1	4.25	2.48E-04	↓	L-threonine 3-dehydrogenase, mitochondrial-like [B. mutus]
ENSP00000260061-D1	4.25	2.48E-04	↓	leucine-rich repeat-containing protein 32 [B. mutus]
ENSBTAP00000028598-D1	4.25	2.48E-04	↓	TPR and ankyrin repeat-containing protein 1-like [B. mutus]
ENSBTAP00000053385-D1	4.25	2.48E-04	↓	Dachshund-like protein 2, partial [B. mutus]
ENSP00000219660-D1	4.25	2.48E-04	↓	aquaporin-8 [B. mutus]
ENSP00000333262-D1	4.25	2.48E-04	↓	phosphatidylinositol 4,5-bisphosphate 5-phosphatase A isoform X1 [B. mutus]
ENSBTAP00000000549-D1	4.25	2.48E-04	↓	Bcl-2-like protein antagonist/killer [B. mutus]
ENSBTAP00000048983-D1	4.25	2.48E-04	↓	PQ-loop repeat-containing protein 1 [B. mutus]
ENSBTAP00000012212-D1	4.25	2.48E-04	↓	paraneoplastic antigen Ma1 homolog [B. mutus]
ENSP00000393854-D1	4.25	2.48E-04	↓	CENPB DNA-binding domain-containing protein 1, partial [B. mutus]
ENSP00000373073-D1	4.25	2.48E-04	↓	Putative urocanate hydratase, partial [B. mutus]
ENSP00000293774-D1	4.25	2.48E-04	↓	keratin, type II cytoskeletal 4 [Vicugna pacos]
ENSP00000188790-D1	4.25	2.48E-04	↓	prolyl endopeptidase FAP [B. taurus]
ENSBTAP00000027209-D2	4.25	2.48E-04	↓	hypothetical protein M91_14379, partial [B. mutus]
ENSBTAP00000049066-D1	4.25	2.48E-04	↓	Calcium-activated potassium channel subunit beta-1, partial [B. mutus]
ENSBTAP00000021694-D1	4.25	2.48E-04	↓	Putative cation-transporting ATPase 13A4, partial [B. mutus]
ENSBTAP00000013588-D81	4.25	2.48E-04	↓	Zinc finger protein 184, partial [B. mutus]
ENSP00000246635-D1	4.25	2.48E-04	↓	keratin, type I cytoskeletal 13 isoform X1 [B. mutus]
ENSBTAP00000052694-D43	4.25	2.48E-04	↓	Olfactory receptor 4K2, partial [B. mutus]
ENSP00000364163-D1	4.25	2.48E-04	↓	neurogenic locus notch homolog protein 4 [B. mutus]
ENSBTAP00000049230-D6	4.25	2.48E-04	↓	60S ribosomal protein L27a [B. taurus]

ENSP00000364236-D1	4.25	2.48E-04	↓	transmembrane protein 136 [B. taurus]
ENSP00000309092-D1	4.25	2.48E-04	↓	mannose-1-phosphate guanyltransferase beta isoform X1 [Ovis aries]
ENSP00000356761-D1	4.25	2.48E-04	↓	cAMP-specific 3',5'-cyclic phosphodiesterase 7B [B. mutus]
ENSP00000226359-D1	4.25	2.48E-04	↓	alpha-fetoprotein-like [B. mutus]
ENSBTAP00000022279-D1	4.25	2.48E-04	↓	Kelch-like protein 38, partial [B. mutus]
ENSP00000336888-D1	4.26	2.74E-31	↓	choline transporter-like protein 2 [B. taurus]
ENSBTAP00000032435-D1	4.28	6.95E-12	↓	partitioning defective 6 homolog alpha [B. taurus]
ENSP00000245304-D1	4.29	5.82E-17	↓	ras-related protein Rap-2a [Equus caballus]
ENSBTAP00000023734-D1	4.30	5.12E-07	↓	Integral membrane protein 2C, partial [B. mutus]
ENSP00000377854-D1	4.30	5.12E-07	↓	LIM/homeobox protein Lhx6, partial [B. mutus]
ENSP00000247977-D1	4.30	5.12E-07	↓	F-box/LRR-repeat protein 12 [B. taurus]
ENSP00000334665-D2	4.30	5.12E-07	↓	Fascin, partial [B. mutus]
ENSP00000366513-D2	4.30	5.12E-07	↓	Calsyntenin-2, partial [B. mutus]
ENSBTAP00000011275-D1	4.36	3.07E-07	↓	N-acetyl-beta-glucosaminyl-glycoprotein 4-beta-N-acetylgalactosaminyltransferase 2, partial [B. mutus]
ENSBTAP00000048857-D2	4.36	3.07E-07	↓	Pyrin, partial [B. mutus]
ENSP00000363545-D1	4.36	3.07E-07	↓	DBF4-type zinc finger-containing protein 2 [B. mutus]
ENSP00000366278-D1	4.36	3.07E-07	↓	mediator of RNA polymerase II transcription subunit 25 isoform X1 [Bubalus bubalis]
ENSBTAP00000033917-D1	4.36	1.48E-04	↓	Zinc transporter ZIP1, partial [B. mutus]
ENSP00000353582-D1	4.36	1.48E-04	↓	neuropilin-2 isoform X1 [B. mutus]
ENSP00000333915-D1	4.36	1.48E-04	↓	Coiled-coil domain-containing protein 42B [B. mutus]
ENSBTAP00000040697-D1	4.36	1.48E-04	↓	Phosphatidylinositol-4-phosphate 3-kinase C2 domain-containing subunit gamma [B. mutus]
ENSP00000360858-D2	4.36	1.48E-04	↓	Noelin-3 [B. mutus]
ENSP00000332413-D1	4.36	1.48E-04	↓	Bestrophin-3, partial [B. mutus]
ENSBTAP00000023305-D1	4.36	1.48E-04	↓	zinc finger protein SNAI3 [B. mutus]
ENSP00000281581-D1	4.36	1.48E-04	↓	hypothetical protein M91_05626, partial [B. mutus]
ENSBTAP00000027172-D1	4.36	1.48E-04	↓	aldehyde dehydrogenase 1 family, member B1 [B. mutus]
ENSBTAP00000007561-D1	4.36	1.48E-04	↓	Codanin-1, partial [B. mutus]
ENSBTAP00000004156-D1	4.36	1.48E-04	↓	hypothetical protein M91_20206, partial [B. mutus]
ENSP00000294064-D1	4.36	1.48E-04	↓	Sialidase-3, partial [B. mutus]
ENSBTAP00000003205-D1	4.36	1.48E-04	↓	Caspase-9, partial [B. mutus]
ENSP00000246841-D1	4.36	1.48E-04	↓	leucine-rich repeat transmembrane protein FLRT1 [B. taurus]
ENSBTAP00000027138-D1	4.36	1.48E-04	↓	Solute carrier family 35 member F3, partial [B. mutus]
ENSBTAP00000030712-D1	4.36	1.48E-04	↓	synaptoporin precursor [B. taurus]
ENSBTAP00000003130-D1	4.36	1.48E-04	↓	Malonyl-CoA-acyl carrier protein transacylase, mitochondrial, partial [B. mutus]
ENSP00000304077-D23	4.36	1.48E-04	↓	hypothetical protein M91_08365, partial [B. mutus]
ENSBTAP00000029564-D1	4.36	1.48E-04	↓	synaptic vesicle 2-related protein [B. taurus]
ENSP00000223210-D1	4.36	1.48E-04	↓	Zinc finger protein 862, partial [B. mutus]
ENSP00000409717-D1	4.36	1.48E-04	↓	LIM domain kinase 1 [B. mutus]
ENSP00000352167-D1	4.36	1.48E-04	↓	Mitochondrial carnitine/acylcarnitine carrier protein CACL [B. mutus]
ENSBTAP00000032404-D1	4.36	1.48E-04	↓	melanocortin-2 receptor accessory protein [B. mutus]
ENSP00000301727-D1	4.36	1.48E-04	↓	Transcription factor E4F1 [B. mutus]
ENSP00000342848-D1	4.36	1.48E-04	↓	NADPH oxidase activator 1, partial [B. mutus]
ENSBTAP00000000752-D1	4.36	1.48E-04	↓	Tenascin, partial [B. mutus]
ENSBTAP00000042600-D1	4.36	1.48E-04	↓	protein Z-dependent protease inhibitor [B. mutus]
ENSBTAP00000022119-D1	4.36	1.48E-04	↓	RNA-binding protein MEX3B, partial [B. mutus]
ENSP00000250056-D1	4.36	1.48E-04	↓	Protein FAM64A, partial [B. mutus]
ENSP00000397428-D3	4.36	1.48E-04	↓	hypothetical protein M91_09988, partial [B. mutus]
ENSBTAP00000053795-D1	4.36	1.48E-04	↓	Ephrin type-A receptor 3, partial [B. mutus]
ENSBTAP00000012465-D1	4.36	1.48E-04	↓	membrane protein FAM159B [B. taurus]
ENSBTAP00000026403-D1	4.37	4.62E-18	↓	ras-related protein Rab-9B [B. taurus]
ENSBTAP00000023352-D1	4.40	8.70E-24	↓	Cingulin, partial [B. mutus]
ENSBTAP00000004843-D1	4.41	1.68E-18	↓	2-hydroxyacyl-CoA lyase 1 isoform X1 [B. mutus]
ENSP00000370620-D1	4.41	1.85E-07	↓	zinc finger BED domain-containing protein 1-like [B. mutus]
ENSP00000309689-D1	4.41	1.85E-07	↓	Leucine-rich repeat and calponin-like protein domain-containing protein 4, partial [B. mutus]
ENSP00000394338-D1	4.41	1.85E-07	↓	Serine/threonine-protein phosphatase 2A activator, partial [B. mutus]

ENSP00000252100-D1	4.44	1.16E-24	↓	transmembrane and coiled-coil domain-containing protein 6 [B. mutus]
ENSBTAP00000010835-D1	4.46	8.81E-05	↓	Toll-interacting protein, partial [B. mutus]
ENSBTAP00000049368-D4	4.46	8.81E-05	↓	hypothetical protein M91_13791, partial [B. mutus]
ENSBTAP00000051231-D3	4.46	8.81E-05	↓	TPA: ribosomal protein S4, X-linked X-like [B. taurus]
ENSP00000295597-D1	4.46	8.81E-05	↓	Plakophilin-1, partial [B. mutus]
ENSBTAP00000042303-D1	4.46	8.81E-05	↓	RAS guanyl-releasing protein 4, partial [B. mutus]
ENSP00000266546-D1	4.46	8.81E-05	↓	calsyntenin-3 isoform X1 [B. mutus]
ENSP00000244096-D2	4.46	8.81E-05	↓	melanoma-associated antigen 10-like [B. mutus]
ENSP00000339802-D1	4.46	8.81E-05	↓	ankyrin repeat domain-containing protein 34B [B. mutus]
ENSBTAP00000017086-D1	4.46	8.81E-05	↓	lipoprotein lipase precursor [B. taurus]
ENSBTAP00000012805-D1	4.46	8.81E-05	↓	SET and MYND domain-containing protein 4 [B. mutus]
ENSBTAP00000003598-D1	4.46	8.81E-05	↓	hypothetical protein M91_13241, partial [B. mutus]
ENSBTAP00000010919-D1	4.46	8.81E-05	↓	interleukin-20 receptor subunit beta [B. mutus]
ENSP00000393854-D3	4.46	8.81E-05	↓	hypothetical protein M91_01358, partial [B. mutus]
ENSP00000302393-D1	4.46	8.81E-05	↓	L-lactate dehydrogenase A-like 6B-like isoform X1 [B. mutus]
ENSBTAP00000049139-D17	4.46	8.81E-05	↓	40S ribosomal protein S6 isoform X5 [Mandrillus leucophaeus]
ENSBTAP00000025305-D1	4.46	8.81E-05	↓	BTB/POZ domain-containing protein KCTD16, partial [B. mutus]
ENSBTAP00000040176-D1	4.46	8.81E-05	↓	Beta-2-glycoprotein 1, partial [B. mutus]
ENSP00000378865-D1	4.46	8.81E-05	↓	hypothetical protein M91_07124 [B. mutus]
yakG002137	4.46	8.81E-05	↓	Elongation factor 1-gamma [B. mutus]
ENSBTAP00000020971-D1	4.46	8.81E-05	↓	ras-related protein Rab-38 [B. taurus]
ENSBTAP00000044507-D1	4.46	8.81E-05	↓	C3 and PZP-like alpha-2-macroglobulin domain-containing protein 8 [B. mutus]
ENSBTAP00000007490-D1	4.46	8.81E-05	↓	cyclic nucleotide-gated cation channel beta-1 isoform a [B. taurus]
ENSBTAP00000020313-D1	4.46	8.81E-05	↓	Prolactin, partial [B. mutus]
ENSP00000268763-D1	4.46	8.81E-05	↓	Kinase suppressor of Ras 1, partial [B. mutus]
ENSP00000342059-D1	4.48	1.21E-13	↓	mitogen-activated protein kinase kinase kinase 14 [B. taurus]
ENSBTAP00000052498-D1	4.50	7.30E-14	↓	Aspartate beta-hydroxylase domain-containing protein 2, partial [B. mutus]
ENSBTAP00000000084-D1	4.55	4.04E-08	↓	Reversion-inducing cysteine-rich protein with Kazal motifs, partial [B. mutus]
ENSBTAP00000019293-D1	4.55	5.27E-05	↓	galactoside 2-alpha-L-fucosyltransferase 2-like [B. mutus]
ENSBTAP00000008785-D1	4.55	5.27E-05	↓	Proprotein convertase subtilisin/kexin type 6, partial [B. mutus]
yakG007106	4.55	5.27E-05	↓	Microtubule-associated tumor suppressor candidate 2 [B. mutus]
ENSBTAP00000022029-D1	4.55	5.27E-05	↓	cytosolic Fe-S cluster assembly factor NUBP2-like isoform X1 [B. mutus]
ENSP00000365397-D1	4.55	5.27E-05	↓	short-chain dehydrogenase/reductase 3 [Macaca mulatta]
ENSP00000407346-D1	4.55	5.27E-05	↓	hypothetical protein M91_04456, partial [B. mutus]
ENSBTAP00000030954-D1	4.55	5.27E-05	↓	Nose resistant to fluoxetine protein 6, partial [B. mutus]
ENSBTAP00000043215-D1	4.55	5.27E-05	↓	cadherin-17 [B. mutus]
ENSP00000261058-D1	4.55	5.27E-05	↓	coiled-coil domain-containing protein 54 [B. mutus]
ENSP00000403403-D1	4.55	5.27E-05	↓	Papilin, partial [B. mutus]
ENSBTAP00000041408-D1	4.55	5.27E-05	↓	lebercilin-like protein [B. mutus]
ENSBTAP00000033255-D1	4.55	5.27E-05	↓	WD repeat-containing protein 60 [B. mutus]
ENSBTAP00000009911-D1	4.55	5.27E-05	↓	Neutrophil cytosol factor 4 [B. mutus]
ENSBTAP00000002244-D1	4.55	5.27E-05	↓	autophagy-related protein 2 homolog B isoform X3 [Bubalus bubalis]
ENSBTAP00000000293-D1	4.55	5.27E-05	↓	PH and SEC7 domain-containing protein 2 [B. mutus]
ENSP00000284031-D1	4.55	5.27E-05	↓	N(G),N(G)-dimethylarginine dimethylaminohydrolase 1, partial [B. mutus]
ENSBTAP00000041520-D2	4.55	5.27E-05	↓	probable E3 ubiquitin-protein ligase MID2 isoform X1 [B. taurus]
ENSP00000323927-D1	4.55	5.27E-05	↓	plexin domain-containing protein 1 precursor [B. taurus]
ENSP00000310788-D53	4.55	5.27E-05	↓	Olfactory receptor 508 [B. mutus]
ENSBTAP00000012285-D1	4.55	5.27E-05	↓	hypothetical protein M91_15628, partial [B. mutus]
ENSBTAP00000028795-D1	4.55	5.27E-05	↓	probable G-protein coupled receptor 110-like [B. mutus]
ENSP00000289805-D1	4.55	5.27E-05	↓	spermatogenesis-associated protein 2-like protein [B. mutus]
ENSP00000370531-D4	4.55	5.27E-05	↓	hypothetical protein M91_00655, partial [B. mutus]
ENSBTAP00000048539-D1	4.55	5.27E-05	↓	TPA: acyltransferase like 1B-like [B. taurus]
ENSBTAP00000041104-D1	4.58	2.74E-27	↓	F-box/LRR-repeat protein 18, partial [B. mutus]
ENSP00000334474-D1	4.59	2.44E-08	↓	Zinc finger MIZ domain-containing protein 1 [B. mutus]
ENSP00000216962-D1	4.59	2.44E-08	↓	Glycogen phosphorylase, brain form, partial [B. mutus]

ENSBTAP00000047637-D2	4.64	3.55E-15	↓	hypothetical protein M91_02990, partial [B. mutus]
ENSBTAP00000006659-D1	4.64	3.17E-05	↓	tyrosine-protein kinase Blk [B. mutus]
ENSP00000354590-D1	4.64	3.17E-05	↓	hypothetical protein M91_02667, partial [B. mutus]
ENSP00000384177-D1	4.64	3.17E-05	↓	Max-binding protein MNT [B. mutus]
ENSBTAP00000011670-D1	4.64	3.17E-05	↓	lymphocyte antigen 96 precursor [B. taurus]
ENSBTAP00000002338-D2	4.64	3.17E-05	↓	Protein-glutamine gamma-glutamyltransferase 5, partial [B. mutus]
ENSP00000351327-D1	4.64	3.17E-05	↓	A-kinase anchor protein 4, partial [B. mutus]
ENSP00000367345-D3	4.64	3.17E-05	↓	Protocadherin gamma-A5, partial [B. mutus]
yakA00136	4.64	3.17E-05	↓	Zinc finger protein 385B [B. mutus]
ENSP00000386609-D1	4.64	3.17E-05	↓	WD repeat-containing protein 81 isoform X1 [B. mutus]
ENSBTAP00000051767-D1	4.64	3.17E-05	↓	armadillo repeat-containing X-linked protein 1 [B. mutus]
ENSBTAP00000022540-D1	4.64	3.17E-05	↓	type-1 angiotensin II receptor [B. mutus]
ENSP00000334329-D1	4.64	3.17E-05	↓	netrin receptor UNC5B isoform X1 [B. mutus]
ENSP00000285311-D1	4.64	3.17E-05	↓	dickkopf-related protein 2 [B. mutus]
ENSBTAP00000022679-D1	4.64	3.17E-05	↓	potassium/sodium hyperpolarization-activated cyclic nucleotide-gated channel 3 [B. mutus]
ENSBTAP00000000461-D1	4.64	3.17E-05	↓	oxidoreductase-like domain-containing protein 1 [B. taurus]
ENSP00000369003-D1	4.64	3.17E-05	↓	short transient receptor potential channel 4 isoform X1 [B. mutus]
yakG026412	4.64	3.17E-05	↓	hypothetical protein M91_09194 [B. mutus]
ENSBTAP00000018834-D1	4.64	3.17E-05	↓	FYVE, RhoGEF and PH domain-containing protein 2 [B. mutus]
ENSP00000311447-D1	4.64	3.17E-05	↓	aspartate beta-hydroxylase domain-containing protein 1 isoform X1 [B. mutus]
ENSP00000378792-D69	4.64	3.17E-05	↓	hypothetical protein M91_18036, partial [B. mutus]
ENSP00000345771-D1	4.66	2.14E-15	↓	6-phosphofructokinase, muscle type isoform X1 [B. mutus]
ENSP00000257927-D1	4.68	8.94E-09	↓	Coenzyme Q-binding protein COQ10-like protein A, mitochondrial, partial [B. mutus]
ENSBTAP00000040745-D1	4.69	1.19E-22	↓	IgG receptor FcRn large subunit p51 isoform X1 [B. mutus]
ENSBTAP00000002179-D1	4.70	4.95E-71	↓	conserved oligomeric Golgi complex subunit 8 [B. mutus]
yakG040728	4.72	5.41E-09	↓	hypothetical protein M91_09212 [B. mutus]
ENSBTAP00000049973-D1	4.72	5.41E-09	↓	BTB/POZ domain-containing protein 19 [Balaenoptera acutorostrata scammoni]
ENSBTAP00000016981-D1	4.72	1.90E-05	↓	serum response factor [Equus caballus]
ENSBTAP00000051454-D1	4.72	1.90E-05	↓	TATA box-binding protein-associated factor RNA polymerase I subunit C [B. mutus]
ENSBTAP00000036259-D1	4.72	1.90E-05	↓	RING finger protein C14orf164 homolog [B. mutus]
ENSP00000280200-D1	4.72	1.90E-05	↓	hypothetical protein M91_13300, partial [B. mutus]
yakG012203	4.72	1.90E-05	↓	Carboxyl-terminal PDZ ligand of neuronal nitric oxide synthase protein [B. mutus]
ENSBTAP00000049101-D1	4.72	1.90E-05	↓	Protein SZT2, partial [B. mutus]
ENSP00000320510-D1	4.72	1.90E-05	↓	Neurabin-2, partial [B. mutus]
ENSP00000310094-D1	4.72	1.90E-05	↓	Serine/threonine-protein kinase TAO2 [B. mutus]
ENSBTAP00000032295-D1	4.72	1.90E-05	↓	Popeye domain-containing protein 2, partial [B. mutus]
ENSP00000263097-D1	4.72	1.90E-05	↓	calponin-2 [B. taurus]
ENSBTAP00000000922-D1	4.72	1.90E-05	↓	acyl-coenzyme A thioesterase THEM5 [B. mutus]
ENSP00000396648-D2	4.72	1.90E-05	↓	proline-rich protein 23B-like [B. mutus]
ENSBTAP00000020024-D1	4.72	1.90E-05	↓	caspase recruitment domain-containing protein 10 isoform X4 [B. taurus]
ENSP00000358786-D1	4.72	1.90E-05	↓	potassium voltage-gated channel subfamily A member 10 [B. mutus]
ENSP00000362344-D1	4.72	1.90E-05	↓	Folylpolyglutamate synthase, mitochondrial [B. mutus]
ENSP00000402872-D2	4.72	1.90E-05	↓	Mamu class II histocompatibility antigen, DR alpha chain, partial [B. mutus]
ENSP00000381196-D1	4.72	1.90E-05	↓	Regulator of G-protein signaling 14, partial [B. mutus]
ENSP00000417928-D1	4.72	1.90E-05	↓	protein orai-2 [B. mutus]
ENSBTAP00000035161-D1	4.72	1.90E-05	↓	proton-coupled folate transporter isoform X1 [B. mutus]
ENSBTAP00000020982-D1	4.73	2.45E-30	↓	serine/threonine-protein phosphatase 4 regulatory subunit 1-like [Ceratotherium simum simum]
ENSBTAP00000019338-D1	4.73	2.66E-23	↓	Protein Niban, partial [B. mutus]
ENSP00000265104-D1	4.76	3.28E-09	↓	Dynein heavy chain 5, axonemal [B. mutus]
ENSP00000363298-D1	4.79	1.23E-31	↓	B box and SPRY domain-containing protein [B. mutus]
ENSP00000376337-D1	4.80	1.99E-09	↓	BTB/POZ domain-containing protein 6, partial [B. mutus]
ENSP00000338885-D1	4.80	1.15E-05	↓	C2 domain-containing protein 2-like protein, partial [B. mutus]
ENSP00000340578-D1	4.80	1.15E-05	↓	Ras association domain-containing protein 6, partial [B. mutus]
ENSP00000358655-D1	4.80	1.15E-05	↓	Protein FAM3A [B. mutus]
ENSP00000341165-D1	4.80	1.15E-05	↓	Zinc finger protein 772, partial [B. mutus]

ENSBTAP00000027909-D1	4.80	1.15E-05	↓	metallophosphoesterase MPPED2 isoform 1 [Homo sapiens]
ENSBTAP00000013671-D1	4.80	1.15E-05	↓	Ras-related protein Rab-12, partial [B. mutus]
ENSP00000358327-D1	4.80	1.15E-05	↓	Caspase-7, partial [B. mutus]
ENSBTAP00000053234-D1	4.80	1.15E-05	↓	Trinucleotide repeat-containing 6C protein, partial [B. mutus]
ENSP00000389415-D1	4.80	1.15E-05	↓	Coiled-coil domain-containing protein 17, partial [B. mutus]
ENSBTAP00000053512-D1	4.80	1.15E-05	↓	chromobox protein homolog 2 [B. mutus]
ENSBTAP00000043397-D6	4.80	1.15E-05	↓	hypothetical protein M91_13685, partial [B. mutus]
ENSBTAP00000037032-D1	4.84	3.18E-94	↓	zinc finger protein 3 [B. mutus]
ENSBTAP00000003267-D1	4.84	1.21E-09	↓	hypothetical protein M91_04011, partial [B. mutus]
ENSP00000333813-D1	4.87	7.31E-10	↓	dol-P-Man:Man(7)GlcNAc(2)-PP-Dol alpha-1,6-mannosyltransferase [B. mutus]
ENSBTAP00000011319-D1	4.87	6.91E-06	↓	Protein CLN8 [B. mutus]
ENSP00000404334-D1	4.87	6.91E-06	↓	HemK methyltransferase family member 1, partial [B. mutus]
ENSBTAP00000022253-D1	4.87	6.91E-06	↓	Protein deltex-1, partial [B. mutus]
ENSP00000241305-D1	4.87	6.91E-06	↓	inactive carboxypeptidase-like protein X2 precursor [B. taurus]
ENSBTAP00000047401-D1	4.87	6.91E-06	↓	Puratrophin-1, partial [B. mutus]
ENSBTAP00000028074-D1	4.87	6.91E-06	↓	type III endosome membrane protein TEMP [B. taurus]
ENSBTAP00000028498-D1	4.87	6.91E-06	↓	protein S100-A14 [B. taurus]
ENSP00000341214-D4	4.87	6.91E-06	↓	Testis-specific H1 histone, partial [B. mutus]
ENSBTAP00000005821-D1	4.87	6.91E-06	↓	1-acyl-sn-glycerol-3-phosphate acyltransferase alpha precursor [B. taurus]
ENSP00000217455-D1	4.87	6.91E-06	↓	acyl-coenzyme A thioesterase 8 [B. mutus]
ENSBTAP00000004133-D12	4.87	6.91E-06	↓	vomeronasal type-1 receptor 4-like [B. taurus]
ENSBTAP00000010762-D1	4.87	6.91E-06	↓	Protein fosB, partial [B. mutus]
ENSBTAP00000043168-D1	4.87	6.91E-06	↓	poly(U)-specific endoribonuclease precursor [B. taurus]
ENSP00000318585-D1	4.87	6.91E-06	↓	beta-secretase 1 isoform X1 [B. mutus]
ENSP00000363161-D1	4.87	6.91E-06	↓	transmembrane protein C9orf91 homolog [B. mutus]
ENSBTAP00000038432-D1	4.87	6.91E-06	↓	Relaxin-3 receptor 1, partial [B. mutus]
ENSBTAP00000052537-D1	4.91	4.45E-10	↓	Endothelin-3, partial [B. mutus]
ENSP00000361965-D1	4.92	1.99E-18	↓	Adenosine deaminase, partial [B. mutus]
ENSP00000336522-D1	4.94	1.21E-18	↓	myotubularin-related protein 5, partial [B. mutus]
ENSBTAP00000040374-D1	4.94	2.70E-10	↓	cat eye syndrome critical region protein 5 isoform X1 [Bison bison bison]
ENSP00000361245-D1	4.94	2.70E-10	↓	E3 ubiquitin-protein ligase HECTD3 [B. mutus]
ENSP00000372750-D1	4.94	4.19E-06	↓	muscarinic acetylcholine receptor M5 [B. mutus]
yakA07982	4.94	4.19E-06	↓	hypothetical protein M91_02255 [B. mutus]
ENSBTAP00000015303-D1	4.94	4.19E-06	↓	Coiled-coil domain-containing protein 106, partial [B. mutus]
ENSP00000415471-D1	4.94	4.19E-06	↓	PR domain zinc finger protein 15 isoform X1 [B. mutus]
ENSBTAP00000016278-D1	4.94	4.19E-06	↓	Protein FAM131A [B. mutus]
ENSP00000268711-D1	4.94	4.19E-06	↓	TPA: mediator of RNA polymerase II transcription subunit 9 [B. taurus]
ENSP00000330278-D1	4.94	4.19E-06	↓	pleckstrin homology domain-containing family H member 1 isoform X3 [B. taurus]
ENSBTAP00000016753-D1	4.94	4.19E-06	↓	protein CNPPD1 isoform X1 [Bubalus bubalis]
ENSBTAP00000003384-D1	4.94	4.19E-06	↓	LON peptidase N-terminal domain and RING finger protein 3 [B. taurus]
ENSBTAP00000000557-D1	4.94	4.19E-06	↓	tumor necrosis factor alpha-induced protein 3 [B. taurus]
ENSP00000416330-D1	4.94	4.19E-06	↓	transforming growth factor-beta-induced protein ig-h3 [B. mutus]
ENSBTAP00000006089-D1	4.94	4.19E-06	↓	hyaluronidase PH-20 isoform X1 [B. mutus]
ENSBTAP00000005378-D1	4.94	4.19E-06	↓	mitogen-activated protein kinase kinase kinase 11 isoform X2 [B. mutus]
ENSP00000257570-D1	4.94	4.19E-06	↓	59 kDa 2'-5'-oligoadenylate synthase-like protein, partial [B. mutus]
ENSBTAP00000025341-D1	4.94	4.19E-06	↓	aquaporin-4 isoform X1 [B. mutus]
ENSBTAP00000050534-D1	5.01	1.01E-10	↓	Forkhead box protein I3, partial [B. mutus]
ENSP00000355454-D1	5.01	1.01E-10	↓	Zinc finger protein 496 [B. mutus]
ENSBTAP00000050069-D45	5.01	1.01E-10	↓	zinc finger protein with KRAB and SCAN domains 5 isoform X1 [B. taurus]
ENSP00000304111-D1	5.01	2.54E-06	↓	ATP-binding cassette sub-family G member 4 [B. mutus]
ENSBTAP00000009528-D88	5.01	2.54E-06	↓	hypothetical protein M91_05995, partial [B. mutus]
ENSP00000366084-D2	5.01	2.54E-06	↓	multidrug resistance-associated protein 4-like [B. mutus]
ENSBTAP00000026283-D1	5.01	2.54E-06	↓	RNA exonuclease 4 [B. mutus]
ENSBTAP00000016120-D1	5.01	2.54E-06	↓	Serine/threonine-protein kinase PLK5, partial [B. mutus]
ENSP00000378312-D1	5.01	2.54E-06	↓	EF-hand calcium-binding domain-containing protein 5, partial [B. mutus]

ENSP00000349029-D3	5.01	2.54E-06	↓	inositol 1,4,5-trisphosphate receptor type 3 [B. mutus]
ENSP00000367459-D1	5.01	2.54E-06	↓	Putative protein KIAA0319, partial [B. mutus]
ENSBTAP00000048551-D1	5.01	2.54E-06	↓	protein FAM73B [Camelus dromedarius]
ENSBTAP00000031954-D1	5.01	2.54E-06	↓	pleckstrin homology domain-containing family G member 2 [B. mutus]
ENSBTAP00000002334-D1	5.04	6.14E-11	↓	serine--tRNA ligase, mitochondrial [B. mutus]
ENSP00000347088-D1	5.07	1.55E-06	↓	Glycosyltransferase-like protein LARGE1, partial [B. mutus]
ENSBTAP00000031485-D9	5.07	1.55E-06	↓	hypothetical protein M91_01698, partial [B. mutus]
ENSP00000386711-D1	5.07	1.55E-06	↓	Janus kinase and microtubule-interacting protein 1 [B. mutus]
ENSBTAP00000027731-D1	5.07	1.55E-06	↓	proton-coupled amino acid transporter 2 [B. mutus]
ENSP00000272521-D1	5.07	1.55E-06	↓	transmembrane protein 177 [B. mutus]
ENSBTAP00000041757-D1	5.07	1.55E-06	↓	2-5A-dependent ribonuclease, partial [B. mutus]
ENSP00000257414-D1	5.07	1.55E-06	↓	Semaphorin-6A [B. mutus]
ENSBTAP00000002469-D1	5.07	1.55E-06	↓	Potassium voltage-gated channel subfamily H member 7, partial [B. mutus]
ENSBTAP00000023138-D1	5.07	1.55E-06	↓	SEC14-like protein 2 [B. taurus]
ENSP00000304077-D18	5.07	1.55E-06	↓	hypothetical protein M91_08364, partial [B. mutus]
ENSP00000333633-D1	5.10	2.29E-11	↓	Metastasis-associated protein MTA1, partial [B. mutus]
ENSP00000386337-D1	5.11	2.13E-30	↓	Ankyrin repeat and zinc finger domain-containing protein 1 [B. mutus]
ENSP00000269886-D2	5.11	2.13E-30	↓	Endophilin-A1, partial [B. mutus]
ENSBTAP00000027147-D1	5.13	1.40E-11	↓	Transducin beta-like protein 3, partial [B. mutus]
ENSP00000265922-D1	5.13	9.41E-07	↓	deleted in bladder cancer protein 1 homolog [B. mutus]
ENSP00000222728-D2	5.13	9.41E-07	↓	homeobox protein Hox-B6 [Balaenoptera acutorostrata scammoni]
ENSBTAP00000029703-D1	5.13	9.41E-07	↓	hypothetical protein M91_11689, partial [B. mutus]
ENSP00000265537-D1	5.13	9.41E-07	↓	probable leucine--tRNA ligase, mitochondrial, partial [B. mutus]
ENSBTAP00000018876-D1	5.13	9.41E-07	↓	sodium-coupled neutral amino acid transporter 4 [B. taurus]
ENSP00000325123-D1	5.13	9.41E-07	↓	zinc finger and SCAN domain-containing protein 2 [B. mutus]
ENSP00000228468-D1	5.13	9.41E-07	↓	Amiloride-sensitive cation channel 2, neuronal, partial [B. mutus]
ENSP00000378431-D1	5.13	9.41E-07	↓	multimerin-1 isoform X1 [B. mutus]
ENSBTAP00000053810-D1	5.13	9.41E-07	↓	glucose-6-phosphate translocase [B. mutus]
ENSBTAP00000024549-D1	5.13	9.41E-07	↓	glutamate receptor ionotropic, NMDA 3A [B. mutus]
ENSP00000355173-D1	5.16	8.55E-12	↓	Autophagy-related protein 9A, partial [B. mutus]
ENSBTAP00000020474-D1	5.19	5.73E-07	↓	Cysteine protease ATG4B, partial [B. mutus]
ENSP00000368517-D1	5.19	5.73E-07	↓	Enoyl-CoA hydratase domain-containing protein 3, mitochondrial, partial [B. mutus]
ENSBTAP00000007760-D1	5.19	5.73E-07	↓	radial spoke head 10 homolog B [B. mutus]
ENSBTAP00000011667-D1	5.19	5.73E-07	↓	Protein FAM65A, partial [B. mutus]
ENSBTAP00000016893-D1	5.19	5.73E-07	↓	FYVE, RhoGEF and PH domain-containing protein 5, partial [B. mutus]
ENSBTAP00000020298-D1	5.19	5.73E-07	↓	P2X purinoceptor 5, partial [B. mutus]
ENSBTAP00000051942-D1	5.21	2.86E-22	↓	zinc finger C2HC domain-containing protein 1C [B. mutus]
ENSBTAP00000009103-D1	5.25	3.50E-07	↓	Kinesin-like protein KIF7, partial [B. mutus]
yakG015429	5.25	3.50E-07	↓	Myosin-14 [B. mutus]
ENSP00000002596-D1	5.25	3.50E-07	↓	heparan sulfate glucosamine 3-O-sulfotransferase 1 precursor [B. taurus]
ENSBTAP00000005621-D1	5.25	3.50E-07	↓	Ellis-van Creveld syndrome protein, partial [B. mutus]
ENSP00000371582-D1	5.25	3.50E-07	↓	Large neutral amino acids transporter small subunit 4 [B. mutus]
ENSBTAP00000017619-D1	5.25	3.50E-07	↓	GTPase ERas [B. mutus]
ENSBTAP00000052961-D1	5.25	3.50E-07	↓	hypothetical protein M91_00962, partial [B. mutus]
ENSP00000355010-D1	5.28	1.21E-12	↓	immunoglobulin superfamily member 1 isoform X2 [B. mutus]
ENSBTAP00000033821-D1	5.30	2.15E-07	↓	teashirt homolog 2-like [B. mutus]
ENSBTAP00000041580-D1	5.30	2.15E-07	↓	Glutathione S-transferase theta-1, partial [B. mutus]
ENSP00000250156-D1	5.33	4.59E-13	↓	rRNA methyltransferase 1, mitochondrial [Bison bison bison]
ENSP00000370770-D1	5.36	1.32E-07	↓	uncharacterized protein C17orf107 homolog [B. mutus]
ENSBTAP00000016573-D5	5.36	1.32E-07	↓	hypothetical protein M91_02832, partial [B. mutus]
ENSBTAP00000026577-D1	5.36	1.32E-07	↓	prenylcysteine oxidase-like [B. mutus]
ENSP00000397173-D1	5.36	1.32E-07	↓	Cytosolic carboxypeptidase 4, partial [B. mutus]
ENSP00000398560-D1	5.36	1.32E-07	↓	spermatogenesis-associated protein 13-like, partial [B. mutus]
ENSP00000391668-D1	5.36	1.32E-07	↓	myotubularin-related protein 11 isoform X1 [B. mutus]
ENSBTAP00000021043-D1	5.36	1.32E-07	↓	Homeobox protein Hox-D1, partial [B. mutus]

ENSP00000273784-D1	5.36	1.32E-07	↓	alpha-2-HS-glycoprotein [B. mutus]
ENSP00000338783-D1	5.38	1.75E-13	↓	Carbohydrate sulfotransferase 6, partial [B. mutus]
ENSBTAP00000008976-D1	5.41	8.15E-08	↓	Rap guanine nucleotide exchange factor 3, partial [B. mutus]
ENSBTAP00000016110-D1	5.41	8.15E-08	↓	tectonin beta-propeller repeat-containing protein 2 [B. mutus]
ENSBTAP00000036905-D1	5.41	8.15E-08	↓	RIMS-binding protein 2 [B. mutus]
ENSBTAP00000006986-D1	5.41	8.15E-08	↓	Mediator of RNA polymerase II transcription subunit 26, partial [B. mutus]
ENSBTAP00000047565-D1	5.41	8.15E-08	↓	hypothetical protein M91_06255, partial [B. mutus]
ENSBTAP00000005332-D1	5.43	6.63E-14	↓	Protocadherin Fat 3, partial [B. mutus]
ENSP00000391596-D1	5.46	5.02E-08	↓	Putative N-acetylglucosamine-6-phosphate deacetylase, partial [B. mutus]
ENSP00000312082-D1	5.46	5.02E-08	↓	Toll-like receptor 8, partial [B. mutus]
ENSP00000396160-D1	5.46	5.02E-08	↓	Fibrosin-1-like protein, partial [B. mutus]
ENSBTAP00000019966-D1	5.46	5.02E-08	↓	WD repeat-containing protein 62 [B. mutus]
ENSBTAP00000037790-D1	5.46	5.02E-08	↓	Tripartite motif-containing protein 47, partial [B. mutus]
ENSP00000373600-D1	5.46	5.02E-08	↓	leucine-rich repeat serine/threonine-protein kinase 1 [B. mutus]
ENSBTAP00000016586-D1	5.46	5.02E-08	↓	retinoic acid receptor alpha isoform X1 [Chrysochloris asiatica]
ENSP00000353766-D1	5.48	1.13E-26	↓	phosphatidylinositol 4,5-bisphosphate 3-kinase catalytic subunit delta isoform isoform X1 [Orcinus orca]
ENSBTAP00000046060-D1	5.50	1.57E-14	↓	SURP and G-patch domain-containing protein 1 [B. mutus]
ENSP00000384120-D1	5.50	3.11E-08	↓	neuropilin and tolloid-like protein 1 [Bison bison bison]
ENSP00000276410-D1	5.50	3.11E-08	↓	neuronal acetylcholine receptor subunit alpha-6 isoform X1 [B. taurus]
ENSBTAP00000023676-D1	5.50	3.11E-08	↓	zinc transporter ZIP9 isoform X1 [Bubalus bubalis]
ENSP00000321184-D1	5.50	3.11E-08	↓	Immunoglobulin superfamily member 3, partial [B. mutus]
ENSBTAP00000006356-D1	5.50	3.11E-08	↓	Iroquois-class homeodomain protein IRX-5, partial [B. mutus]
ENSP00000329452-D1	5.55	1.93E-08	↓	Mitochondrial 2-oxodicarboxylate carrier, partial [B. mutus]
ENSP00000398153-D1	5.55	1.93E-08	↓	ras-specific guanine nucleotide-releasing factor 1 isoform X1 [B. taurus]
ENSP00000386157-D1	5.55	1.93E-08	↓	tRNA (guanine-N(7)-)-methyltransferase, partial [B. mutus]
ENSP00000269391-D1	5.55	1.93E-08	↓	RING finger protein 157 [B. mutus]
ENSBTAP00000004579-D1	5.55	1.93E-08	↓	uncharacterized protein KIAA0895-like homolog [B. mutus]
ENSBTAP00000018213-D1	5.55	1.93E-08	↓	Sorting nexin-8, partial [B. mutus]
ENSBTAP00000003490-D1	5.57	3.75E-15	↓	Myosin-Ih, partial [B. mutus]
ENSBTAP00000024227-D1	5.59	1.20E-08	↓	Bifunctional ATP-dependent dihydroxyacetone kinase/FAD-AMP lyase (cyclizing), partial [B. mutus]
ENSP00000349705-D1	5.59	1.20E-08	↓	DNA topoisomerase 3-beta-1, partial [B. mutus]
ENSBTAP00000047454-D1	5.59	1.20E-08	↓	Putative 2-ketogluconate reductase [B. mutus]
ENSP00000230993-D1	5.59	1.20E-08	↓	anion exchange protein 4 [B. mutus]
ENSP00000269190-D1	5.59	1.20E-08	↓	dystrobrevin alpha isoform X5 [Bison bison bison]
yakG033556	5.59	1.20E-08	↓	TLD domain-containing protein KIAA1609 [B. mutus]
ENSP00000275820-D1	5.59	1.20E-08	↓	nucleolar protein with MIF4G domain 1 [B. mutus]
ENSP00000370242-D1	5.64	7.45E-09	↓	protein WWC3 isoform X5 [B. taurus]
ENSBTAP00000044500-D1	5.64	7.45E-09	↓	Homeobox protein SEBOX, partial [B. mutus]
ENSP00000366463-D1	5.64	7.45E-09	↓	Paxillin, partial [B. mutus]
ENSP00000268042-D1	5.64	7.45E-09	↓	arrestin domain-containing protein 4-like [Bubalus bubalis]
ENSBTAP00000047533-D1	5.66	5.64E-16	↓	Transmembrane protein 175 [B. mutus]
ENSP00000297146-D2	5.66	5.64E-16	↓	probable G-protein coupled receptor 173 [B. taurus]
ENSP00000366493-D1	5.68	4.64E-09	↓	lutropin subunit beta [B. mutus]
ENSP00000071281-D1	5.68	4.64E-09	↓	four and a half LIM domains protein 1 isoform X1 [B. mutus]
ENSBTAP00000008918-D7	5.68	4.64E-09	↓	Cytochrome P450 2C23, partial [B. mutus]
ENSBTAP00000025471-D1	5.68	4.64E-09	↓	hypothetical protein M91_07321, partial [B. mutus]
ENSBTAP00000025518-D1	5.72	2.90E-09	↓	zinc finger protein 710 [B. mutus]
ENSP00000367304-D1	5.72	2.90E-09	↓	carcinoembryonic antigen-related cell adhesion molecule 1-like [Ceratotherium simum simum]
ENSP00000231461-D1	5.72	2.90E-09	↓	CMP-N-acetylneuraminate-poly-alpha-2,8-sialyltransferase-like [B. mutus]
ENSBTAP00000046353-D1	5.76	1.81E-09	↓	protein SLX4IP [Capra hircus]
ENSBTAP00000023645-D1	5.76	1.81E-09	↓	pre-miRNA 5'-monophosphate methyltransferase [B. mutus]
ENSP00000385545-D1	5.80	1.13E-09	↓	Kinesin-like protein KIF26B, partial [B. mutus]
ENSBTAP00000006504-D1	5.80	1.13E-09	↓	Annexin A10, partial [B. mutus]
ENSBTAP00000007169-D1	5.84	7.09E-10	↓	Ras GTPase-activating protein 4, partial [B. mutus]
ENSBTAP00000026058-D1	5.84	7.09E-10	↓	migration and invasion-inhibitory protein [B. taurus]

ENSP00000416558-D1	5.84	7.09E-10	↓	F-box only protein 24, partial [B. mutus]
ENSBTAP00000009283-D1	5.84	7.09E-10	↓	autophagy-related protein 2 homolog A [Bison bison bison]
ENSP00000333456-D1	5.84	7.09E-10	↓	retinoic acid-induced protein 2 isoform X1 [B. mutus]
ENSBTAP00000020676-D1	5.85	5.21E-18	↓	Opioid growth factor receptor, partial [B. mutus]
ENSP00000351475-D1	5.87	4.46E-10	↓	PAS domain-containing serine/threonine-protein kinase, partial [B. mutus]
ENSP00000230671-D1	5.87	4.46E-10	↓	Sodium-dependent proline transporter, partial [B. mutus]
ENSBTAP00000012721-D1	5.91	2.80E-10	↓	lysophosphatidic acid receptor 1 [B. mutus]
ENSP00000313309-D1	5.91	2.80E-10	↓	protein fuzzy homolog isoform X1 [B. mutus]
ENSBTAP00000002276-D1	5.91	2.80E-10	↓	disintegrin and metalloproteinase domain-containing protein 11 [B. mutus]
ENSBTAP00000005307-D1	5.94	1.76E-10	↓	plasma serine protease inhibitor [B. mutus]
ENSP00000262605-D1	5.98	1.11E-10	↓	alpha-tocopherol transfer protein-like isoform X1 [B. mutus]
ENSBTAP00000018852-D2	6.01	7.05E-11	↓	Solute carrier family 12 member 7, partial [B. mutus]
ENSP00000337459-D1	6.04	4.46E-11	↓	Nitric oxide synthase, brain [B. mutus]
ENSBTAP00000027556-D1	6.06	2.09E-20	↓	matrix metalloproteinase-9 [B. mutus]
ENSP00000378174-D1	6.09	8.41E-21	↓	signal peptide, CUB and EGF-like domain-containing protein 3 isoform X6 [B. taurus]
ENSBTAP00000020385-D1	6.10	1.79E-11	↓	tetraspanin-12 [B. mutus]
ENSBTAP00000025164-D1	6.13	1.14E-11	↓	glucose-fructose oxidoreductase domain-containing protein 2 precursor [B. taurus]
ENSP00000409738-D1	6.13	1.14E-11	↓	protein lifeguard 3 [B. mutus]
ENSP00000363614-D1	6.19	4.59E-12	↓	Hydroxymethylglutaryl-CoA lyase, mitochondrial [B. mutus]
ENSBTAP00000022569-D1	6.22	2.92E-12	↓	carbohydrate sulfotransferase 4 [B. mutus]
ENSBTAP00000015925-D1	6.22	2.92E-12	↓	Transforming growth factor beta-3 [B. mutus]
ENSP00000400644-D1	6.25	1.87E-12	↓	unnamed protein product [Homo sapiens]
ENSBTAP00000018136-D1	6.25	1.87E-12	↓	E3 ubiquitin-protein ligase rififylin, partial [B. mutus]
ENSP00000361667-D1	6.28	2.46E-23	↓	dolichol kinase [B. taurus]
ENSP00000312834-D1	6.28	1.20E-12	↓	MLX-interacting protein, partial [B. mutus]
ENSP00000376615-D1	6.30	7.67E-13	↓	5'-nucleotidase domain-containing protein 3, partial [B. mutus]
ENSP00000203166-D1	6.30	7.67E-13	↓	HAUS augmin-like complex subunit 5 [B. mutus]
ENSBTAP00000017390-D1	6.33	4.93E-13	↓	Transcriptional repressor CTCFL [B. mutus]
ENSP00000396489-D1	6.46	5.42E-14	↓	E3 ubiquitin-protein ligase MARCH2 [B. taurus]
ENSBTAP00000006105-D1	6.46	5.42E-14	↓	lysophosphatidic acid receptor 2 [B. mutus]
ENSBTAP00000033424-D1	6.50	2.26E-14	↓	epithelial chloride channel protein-like isoform X1 [B. mutus]
ENSP00000337510-D1	6.53	1.46E-14	↓	F-box only protein 40 [B. mutus]
ENSBTAP00000025309-D1	6.53	1.46E-14	↓	melanoma-associated antigen F1-like [B. mutus]
ENSP00000403068-D1	6.53	1.46E-14	↓	Ras association domain-containing protein 7, partial [B. mutus]
ENSBTAP00000027155-D1	6.55	9.50E-15	↓	thrombospondin type-1 domain-containing protein 1 isoform X1 [B. mutus]
ENSBTAP00000026804-D1	6.55	9.50E-15	↓	serine protease HTRA2, mitochondrial precursor [B. taurus]
ENSBTAP00000043349-D1	6.59	4.00E-15	↓	Leucine-rich repeat-containing protein 29, partial [B. mutus]
ENSBTAP00000015669-D1	6.59	4.00E-15	↓	perilipin-3 isoform X1 [B. mutus]
ENSBTAP00000044620-D1	6.62	2.60E-15	↓	sphingosine-1-phosphate lyase 1 [B. mutus]
ENSBTAP00000002229-D1	6.68	7.20E-16	↓	arrestin domain-containing protein 2 isoform X1 [Bison bison bison]
ENSBTAP00000017844-D1	6.91	4.65E-18	↓	coiled-coil domain-containing protein 51 isoform X1 [B. mutus]
ENSP00000313258-D1	7.13	1.52E-20	↓	zinc finger protein 541 [B. mutus]
ENSP00000296604-D1	7.16	6.78E-21	↓	Ran-binding protein 3-like protein, partial [B. mutus]
ENSBTAP00000004507-D52	7.16	6.78E-21	↓	TPA: zinc finger protein 329 [B. taurus]
ENSP00000280527-D1	7.36	2.68E-23	↓	Cysteine-rich motor neuron 1 protein, partial [B. mutus]
ENSBTAP00000028766-D1	7.38	1.23E-23	↓	secretogranin-2-like [B. mutus]
8-cell vs Morula				
ENSBTAP00000007760-D1	-10.20	2.93E-109	↑	radial spoke head 10 homolog B [B. mutus]
ENSP00000386157-D1	-9.84	3.12E-90	↑	tRNA (guanine-N(7)-)-methyltransferase, partial [B. mutus]
ENSBTAP00000010982-D2	-9.67	1.69E-82	↑	uncharacterized protein LOC105610665 isoform X2 [Ovis aries musimon]
ENSBTAP00000004653-D1	-9.57	4.07E-78	↑	DNA polymerase lambda isoform X1 [B. mutus]
ENSP00000397380-D1	-9.20	4.92E-64	↑	lysosomal-associated transmembrane protein 5 [B. mutus]
ENSBTAP00000024227-D1	-9.13	8.62E-62	↑	Bifunctional ATP-dependent dihydroxyacetone kinase/FAD-AMP lyase (cyclizing), partial [B. mutus]
ENSBTAP00000015584-D1	-9.10	1.13E-118	↑	ankyrin repeat domain-containing protein 1 [B. mutus]
ENSBTAP00000029728-D23	-9.06	2.96E-59	↑	Zinc finger protein 81, partial [B. mutus]

ENSP00000071281-D1	-9.00	1.68E-57	↑	four and a half LIM domains protein 1 isoform X1 [B. mutus]
ENSBTAP00000021429-D1	-8.98	8.07E-57	↑	protein BRICK1 isoform X1 [Stegastes partitus]
ENSP00000360266-D1	-8.93	7.51E-108	↑	Transcription factor AP-1 [B. mutus]
ENSP00000356825-D1	-8.93	2.56E-55	↑	Adenylate cyclase type 10 [B. mutus]
ENSP00000346564-D1	-8.85	5.73E-53	↑	tumor protein p53-inducible protein 11 [Lipotes vexillifer]
ENSBTAP00000015315-D1	-8.82	3.93E-52	↑	Sodium-coupled monocarboxylate transporter 1 [B. mutus]
ENSBTAP00000053788-D1	-8.80	1.97E-51	↑	cyclin-dependent kinase 6 [B. mutus]
ENSP00000299345-D4	-8.78	5.19E-51	↑	Cadherin-8, partial [B. mutus]
ENSBTAP00000033424-D1	-8.76	1.90E-50	↑	epithelial chloride channel protein-like isoform X1 [B. mutus]
ENSBTAP00000046249-D1	-8.73	9.67E-50	↑	Response to complement 32 protein, partial [B. mutus]
ENSBTAP00000050331-D1	-8.73	9.67E-50	↑	C-type lectin domain family 1 member B [B. mutus]
ENSP00000363727-D1	-8.72	2.58E-49	↑	StAR-related lipid transfer protein 8, partial [B. mutus]
ENSP00000358803-D1	-8.69	1.33E-48	↑	calcium homeostasis modulator protein 2 [B. mutus]
ENSBTAP00000024068-D1	-8.66	9.65E-48	↑	ly6/PLAUR domain-containing protein 3 [B. mutus]
ENSBTAP00000032180-D1	-8.66	9.65E-48	↑	Geminin coiled-coil domain-containing protein 1, partial [B. mutus]
ENSBTAP00000026146-D1	-8.53	2.10E-44	↑	thy-1 membrane glycoprotein precursor [B. taurus]
ENSBTAP00000003384-D1	-8.53	2.10E-44	↑	LON peptidase N-terminal domain and RING finger protein 3 [B. taurus]
ENSP00000290510-D1	-8.40	1.92E-41	↑	Prolyl 3-hydroxylase 3, partial [B. mutus]
ENSP00000401313-D1	-8.35	5.31E-78	↑	cysteine and tyrosine-rich protein 1-like isoform X2 [Bubalus bubalis]
ENSP00000285419-D1	-8.35	2.16E-40	↑	type 2 phosphatidylinositol 4,5-bisphosphate 4-phosphatase [B. mutus]
ENSBTAP00000000549-D1	-8.30	2.48E-39	↑	Bcl-2-like protein antagonist/killer [B. mutus]
ENSBTAP00000027951-D1	-8.27	1.43E-38	↑	sushi domain-containing protein 4 [B. mutus]
ENSBTAP00000003315-D1	-8.23	8.34E-38	↑	inositol oxygenase [B. mutus]
ENSP00000326022-D1	-8.14	5.98E-36	↑	four and a half LIM domains protein 5 [B. mutus]
ENSP00000380299-D1	-8.14	5.98E-36	↑	Complement C1q tumor necrosis factor-related protein 6, partial [B. mutus]
ENSBTAP00000000752-D1	-8.11	1.76E-35	↑	Tenascin, partial [B. mutus]
ENSBTAP00000048068-D1	-8.06	2.21E-34	↑	hypothetical protein M91_10369, partial [B. mutus]
ENSP00000285928-D1	-8.04	4.58E-34	↑	Leucine-rich repeat and guanylate kinase domain-containing protein [B. mutus]
ENSBTAP00000043661-D4	-7.98	5.95E-33	↑	glutathione S-transferase subunit isoform I [B. taurus]
ENSBTAP00000023672-D1	-7.86	7.42E-31	↑	arylsulfatase J-like [B. mutus]
ENSP00000419361-D1	-7.84	1.57E-30	↑	Adenylate cyclase type 5, partial [B. mutus]
ENSBTAP00000053810-D1	-7.79	1.03E-29	↑	glucose-6-phosphate translocase [B. mutus]
ENSP00000396489-D1	-7.79	1.03E-29	↑	E3 ubiquitin-protein ligase MARCH2 [B. taurus]
ENSP00000312834-D1	-7.78	1.51E-29	↑	MLX-interacting protein, partial [B. mutus]
ENSBTAP00000015235-D1	-7.77	2.20E-29	↑	Myosin-binding protein H [B. mutus]
ENSP00000356257-D1	-7.76	6.71E-109	↑	leiomodin-1 [B. mutus]
ENSBTAP00000053058-D63	-7.76	3.21E-29	↑	Zinc finger protein 25, partial [B. mutus]
ENSP00000384177-D1	-7.75	4.69E-29	↑	Max-binding protein MNT [B. mutus]
ENSP00000356278-D1	-7.73	1.01E-28	↑	pleckstrin homology-like domain family A member 3, partial [B. mutus]
ENSP00000328274-D23	-7.70	3.17E-28	↑	hypothetical protein M91_03928, partial [B. mutus]
ENSP00000302166-D1	-7.70	3.17E-28	↑	potassium channel subfamily K member 9 [B. taurus]
ENSBTAP00000027155-D1	-7.67	6.85E-28	↑	thrombospondin type-1 domain-containing protein 1 isoform X1 [B. mutus]
ENSP00000359211-D1	-7.64	2.90E-101	↑	Dihydropyrimidine dehydrogenase [NADP+], partial [B. mutus]
ENSP00000254846-D1	-7.57	2.24E-26	↑	lysine-specific demethylase 6B, partial [B. mutus]
ENSP00000320849-D1	-7.56	3.32E-26	↑	coiled-coil domain-containing protein 184 [B. taurus]
ENSBTAP00000037291-D1	-7.54	1.58E-95	↑	ectoderm-neural cortex protein 1 isoform X1 [Odobenus rosmarus divergens]
ENSP00000315564-D1	-7.54	7.25E-26	↑	galectin-9 isoform 1 [B. taurus]
ENSP00000298687-D1	-7.49	3.50E-25	↑	protein NDRG2-like isoform X1 [Capra hircus]
ENSBTAP00000001420-D1	-7.49	3.50E-25	↑	cellular tumor antigen p53 [B. mutus]
ENSBTAP00000024617-D1	-7.48	5.20E-25	↑	Serum deprivation-response protein, partial [B. mutus]
ENSP00000325941-D1	-7.48	5.20E-25	↑	Synembryn-A [B. mutus]
ENSP00000332900-D1	-7.45	1.36E-46	↑	Putative G-protein coupled receptor 97, partial [B. mutus]
ENSBTAP00000017429-D1	-7.42	9.92E-46	↑	FERM, RhoGEF and pleckstrin domain-containing protein 2 isoform X3 [Bubalus bubalis]
ENSP00000299641-D1	-7.40	3.27E-45	↑	bifunctional heparan sulfate N-deacetylase/N-sulfotransferase 2 [B. taurus]
ENSP00000228841-D3	-7.37	1.25E-23	↑	myosin regulatory light chain 2, ventricular/cardiac muscle isoform isoform X1 [B. taurus]

ENSBTAP00000003490-D1	-7.32	2.68E-43	↑	Myosin-Ih, partial [B. mutus]
ENSP00000361965-D1	-7.29	3.14E-82	↑	Adenosine deaminase, partial [B. mutus]
ENSBTAP00000010648-D1	-7.26	3.19E-22	↑	Alkaline ceramidase 1, partial [B. mutus]
ENSP00000295256-D1	-7.26	3.19E-22	↑	hematopoietic prostaglandin D synthase isoform X1 [Bison bison bison]
ENSBTAP00000049973-D1	-7.25	1.54E-41	↑	BTB/POZ domain-containing protein 19 [Balaenoptera acutorostrata scammoni]
ENSP00000394338-D1	-7.25	2.32E-41	↑	Serine/threonine-protein phosphatase 2A activator, partial [B. mutus]
ENSBTAP00000017182-D1	-7.25	4.79E-22	↑	cardiac phospholamban [B. taurus]
ENSBTAP00000051767-D1	-7.20	1.63E-21	↑	armadillo repeat-containing X-linked protein 1 [B. mutus]
ENSP00000409007-D1	-7.18	2.46E-21	↑	Glial cell line-derived neurotrophic factor, partial [B. mutus]
ENSP00000274063-D1	-7.17	3.72E-21	↑	secreted frizzled-related protein 2 precursor [B. taurus]
ENSBTAP00000004963-D1	-7.14	8.48E-21	↑	EF-hand calcium-binding domain-containing protein 1, partial [B. mutus]
ENSP00000261402-D1	-7.14	8.48E-21	↑	NUAK family SNF1-like kinase 1, partial [B. mutus]
ENSP00000238994-D1	-7.13	8.71E-147	↑	Protein phosphatase 1 regulatory subunit 3C, partial [B. mutus]
ENSBTAP00000053425-D1	-7.12	1.64E-38	↑	Thrombospondin type-1 domain-containing protein 4, partial [B. mutus]
ENSBTAP00000043340-D1	-7.12	1.29E-20	↑	Krueppel-like factor 1, partial [B. mutus]
ENSP00000395225-D17	-7.12	1.29E-20	↑	testis-specific Y-encoded-like protein 5 [B. taurus]
ENSP00000335620-D3	-7.09	0.00E+00	↑	Glutathione S-transferase A1, partial [B. mutus]
ENSBTAP00000020758-D1	-7.07	1.99E-37	↑	Sphingomyelin phosphodiesterase, partial [B. mutus]
ENSBTAP00000011045-D1	-7.07	4.48E-20	↑	Cortexin-1, partial [B. mutus]
ENSBTAP00000047178-D1	-7.02	1.57E-19	↑	Tumor necrosis factor alpha-induced protein 2 [B. mutus]
ENSP00000215368-D1	-7.02	1.57E-19	↑	Ephrin-A2, partial [B. mutus]
ENSBTAP00000002878-D1	-7.02	1.57E-19	↑	F-box only protein 31, partial [B. mutus]
ENSP00000372410-D1	-7.01	2.40E-19	↑	probable inactive ribonuclease-like protein 13 precursor [B. taurus]
ENSBTAP00000006986-D1	-6.97	5.58E-19	↑	Mediator of RNA polymerase II transcription subunit 26, partial [B. mutus]
ENSP00000386022-D1	-6.93	1.30E-18	↑	membrane-spanning 4-domains subfamily A member 15 [B. mutus]
ENSBTAP00000046353-D1	-6.93	1.30E-18	↑	protein SLX4IP [Capra hircus]
ENSP00000323183-D1	-6.88	2.12E-33	↑	zinc finger and BTB domain-containing protein 2 [B. taurus]
ENSBTAP00000023138-D1	-6.88	4.68E-18	↑	SEC14-like protein 2 [B. taurus]
ENSP00000417824-D1	-6.86	7.17E-18	↑	Inter-alpha-trypsin inhibitor heavy chain H4 [B. mutus]
ENSP00000284274-D1	-6.85	4.26E-63	↑	Protein FAM105B, partial [B. mutus]
ENSBTAP00000005332-D1	-6.84	1.18E-32	↑	Protocadherin Fat 3, partial [B. mutus]
ENSP00000324775-D1	-6.84	1.10E-17	↑	Transmembrane inner ear expressed protein, partial [B. mutus]
ENSP00000300992-D6	-6.82	1.70E-17	↑	keratin, type I cytoskeletal 16 [B. mutus]
ENSBTAP00000022557-D1	-6.82	1.70E-17	↑	PHD finger protein 19, partial [B. mutus]
ENSP00000251776-D1	-6.81	3.11E-61	↑	ropporin-1-like [B. mutus]
ENSP00000316779-D1	-6.80	7.36E-61	↑	myc box-dependent-interacting protein 1-like isoform X1 [B. mutus]
ENSBTAP00000000580-D1	-6.78	4.03E-17	↑	carboxypeptidase B [B. mutus]
ENSP00000380318-D1	-6.76	6.22E-17	↑	3-mercaptopyruvate sulfurtransferase, partial [B. mutus]
ENSBTAP00000008304-D1	-6.74	8.45E-87	↑	nudC domain-containing protein 3 isoform X1 [B. mutus]
ENSP00000334665-D2	-6.74	8.83E-31	↑	Fascin, partial [B. mutus]
ENSP00000322323-D1	-6.74	9.59E-17	↑	Dentin matrix protein 4, partial [B. mutus]
ENSBTAP00000022569-D1	-6.74	9.59E-17	↑	carbohydrate sulfotransferase 4 [B. mutus]
ENSBTAP00000024727-D1	-6.73	3.11E-86	↑	docking protein 1 isoform X1 [B. mutus]
ENSP00000363832-D1	-6.73	1.36E-30	↑	Aldehyde oxidase [B. mutus]
ENSBTAP00000001222-D1	-6.67	3.58E-16	↑	Homeobox protein Hox-C13, partial [B. mutus]
ENSP00000371388-D1	-6.65	2.90E-29	↑	Sulfiredoxin-1, partial [B. mutus]
ENSP00000360806-D1	-6.63	8.59E-16	↑	Potassium voltage-gated channel subfamily B member 1, partial [B. mutus]
ENSP00000332834-D1	-6.60	2.15E-79	↑	GAS2-like protein 1 [Pongo abelii]
ENSP00000385545-D1	-6.58	2.08E-15	↑	Kinesin-like protein KIF26B, partial [B. mutus]
ENSBTAP00000049737-D52	-6.56	9.90E-28	↑	zinc finger protein 211-like [Orcinus orca]
ENSBTAP00000048973-D1	-6.54	3.95E-52	↑	Prostaglandin F2 receptor negative regulator, partial [B. mutus]
ENSBTAP00000004849-D1	-6.53	6.91E-174	↑	leucine-rich repeat-containing protein 37A-like [B. mutus]
ENSP00000343819-D4	-6.51	7.93E-15	↑	Cone-rod homeobox protein [B. mutus]
ENSP00000402831-D1	-6.49	2.16E-50	↑	Metal transporter CNNM4, partial [B. mutus]
ENSBTAP00000039132-D1	-6.45	7.80E-304	↑	zinc finger protein 512B [B. mutus]

ENSBTAP00000007338-D1	-6.41	2.53E-93	↑	Glycine amidinotransferase, mitochondrial, partial [B. mutus]
ENSP00000164133-D1	-6.41	2.13E-25	↑	serine/threonine-protein phosphatase 2A 56 kDa regulatory subunit beta isoform [B. taurus]
ENSP00000345133-D1	-6.39	7.57E-14	↑	serine/threonine-protein kinase MRCK gamma isoform X5 [B. taurus]
ENSP00000378492-D1	-6.38	4.49E-47	↑	metabotropic glutamate receptor 2 precursor [B. taurus]
ENSP00000377545-D1	-6.37	1.11E-46	↑	Complement C1q tumor necrosis factor-related protein 2, partial [B. mutus]
ENSP00000349297-D1	-6.36	1.30E-24	↑	Very long-chain specific acyl-CoA dehydrogenase, mitochondrial, partial [B. mutus]
ENSBTAP00000018959-D1	-6.33	6.98E-174	↑	Scavenger receptor class B member 1 [B. mutus]
ENSP00000346508-D1	-6.29	1.64E-44	↑	Platelet-derived growth factor subunit A, partial [B. mutus]
ENSBTAP00000025388-D1	-6.27	1.99E-23	↑	synaptogyrin-2 [Bison bison bison]
ENSBTAP00000000510-D2	-6.27	4.71E-13	↑	hypothetical protein M91_18032, partial [B. mutus]
ENSP00000313158-D1	-6.25	7.46E-13	↑	zinc finger protein 366 [B. mutus]
ENSP00000369003-D1	-6.25	7.46E-13	↑	short transient receptor potential channel 4 isoform X1 [B. mutus]
ENSBTAP00000009492-D1	-6.25	7.46E-13	↑	Arginyl aminopeptidase-like 1, partial [B. mutus]
ENSBTAP00000018573-D1	-6.22	1.55E-102	↑	low-density lipoprotein receptor-related protein 1B isoform X1, partial [B. taurus]
ENSP00000333633-D1	-6.22	1.25E-22	↑	Metastasis-associated protein MTA1, partial [B. mutus]
ENSP00000410938-D1	-6.22	1.18E-12	↑	catechol O-methyltransferase [B. mutus]
ENSBTAP00000018241-D1	-6.22	1.18E-12	↑	probable G-protein coupled receptor 37 [B. mutus]
ENSP00000349436-D1	-6.22	1.18E-12	↑	disintegrin and metalloproteinase domain-containing protein 15 isoform X1 [B. mutus]
ENSBTAP00000043771-D1	-6.20	1.75E-140	↑	Activated CDC42 kinase 1 [B. mutus]
ENSBTAP00000045884-D1	-6.18	3.16E-22	↑	Betaine--homocysteine S-methyltransferase 2, partial [B. mutus]
ENSBTAP00000030518-D1	-6.18	1.88E-12	↑	hypothetical protein M91_12207, partial [B. mutus]
ENSBTAP00000000944-D1	-6.15	3.36E-117	↑	A disintegrin and metalloproteinase with thrombospondin motifs 1 [B. mutus]
ENSP00000376337-D1	-6.15	7.97E-22	↑	BTB/POZ domain-containing protein 6, partial [B. mutus]
ENSP00000365198-D1	-6.09	7.73E-76	↑	kazrin isoform X4 [B. taurus]
ENSBTAP00000022907-D1	-6.09	7.64E-12	↑	Gamma-aminobutyric acid type B receptor subunit 1, partial [B. mutus]
ENSBTAP00000026223-D1	-6.09	7.64E-12	↑	melanocortin receptor 4-like [B. mutus]
ENSBTAP00000000510-D1	-6.09	7.64E-12	↑	Protein canopy-like protein 1, partial [B. mutus]
ENSP00000388548-D1	-6.07	5.76E-57	↑	Cbp/p300-interacting transactivator 1, partial [B. mutus]
ENSP00000342993-D2	-6.06	1.30E-20	↑	Synapsin-1, partial [B. mutus]
ENSBTAP00000024852-D1	-6.06	1.22E-11	↑	E3 ubiquitin-protein ligase TRIM11 [B. mutus]
ENSBTAP00000053815-D1	-6.05	2.68E-38	↑	Renalase, partial [B. mutus]
ENSP00000367384-D1	-6.04	1.40E-126	↑	probable G-protein coupled receptor 34 [B. taurus]
ENSBTAP00000025839-D10	-6.02	1.96E-11	↑	hypothetical protein M91_11413, partial [B. mutus]
yakG025338	-6.02	4.65E-159	↑	BEN domain-containing protein 2 [B. mutus]
ENSP00000360437-D1	-6.01	5.32E-20	↑	hypothetical protein M91_17506, partial [B. mutus]
ENSP00000216862-D1	-6.01	5.32E-20	↑	1,25-dihydroxyvitamin D(3) 24-hydroxylase, mitochondrial-like isoform X1 [B. mutus]
ENSP00000378472-D1	-5.99	2.05E-88	↑	transcription factor 7 isoform X1 [B. taurus]
ENSBTAP00000019413-D1	-5.99	3.16E-11	↑	prostacyclin receptor isoform X1 [B. taurus]
ENSBTAP00000026804-D1	-5.99	3.16E-11	↑	serine protease HTRA2, mitochondrial precursor [B. taurus]
ENSP00000351163-D1	-5.97	3.81E-70	↑	Collagen alpha-1(XI) chain [B. mutus]
ENSP00000387278-D1	-5.92	8.17E-11	↑	MAGUK p55 subfamily member 4 [B. mutus]
ENSP00000377854-D1	-5.90	9.10E-19	↑	LIM/homeobox protein Lhx6, partial [B. mutus]
ENSBTAP00000006003-D1	-5.86	3.03E-112	↑	paired mesoderm homeobox protein 1 [B. taurus]
ENSBTAP00000008080-D1	-5.85	5.27E-111	↑	WD repeat-containing protein 93 [B. mutus]
ENSBTAP00000015051-D2	-5.81	2.65E-123	↑	Homeodomain-interacting protein kinase 2 [B. mutus]
ENSBTAP00000008106-D1	-5.80	1.70E-92	↑	Rab11 family-interacting protein 5, partial [B. mutus]
ENSBTAP00000021731-D1	-5.79	1.26E-181	↑	acyl-coenzyme A thioesterase 13 [B. taurus]
ENSBTAP00000015161-D1	-5.79	7.37E-107	↑	dual specificity testis-specific protein kinase 1 [B. mutus]
ENSP00000345771-D1	-5.77	3.99E-32	↑	6-phosphofructokinase, muscle type isoform X1 [B. mutus]
ENSP00000419235-D1	-5.76	5.62E-10	↑	protein FAM115A-like isoform X2 [B. mutus]
ENSP00000352685-D1	-5.74	7.22E-46	↑	TNF receptor-associated factor 2 [B. mutus]
ENSP00000357362-D1	-5.74	4.19E-17	↑	thrombospondin-3 isoform X1 [B. mutus]
ENSP00000386538-D1	-5.73	8.20E-60	↑	TBC1 domain family member 10B, partial [B. mutus]
ENSBTAP00000018594-D1	-5.72	5.20E-116	↑	Nuclear receptor subfamily 2 group E member 1, partial [B. mutus]
ENSBTAP00000003637-D1	-5.72	6.79E-17	↑	calpain-10 [B. mutus]

ENSP00000318585-D1	-5.72	9.11E-10	↑	beta-secretase 1 isoform X1 [B. mutus]
ENSP00000386711-D1	-5.72	9.11E-10	↑	Janus kinase and microtubule-interacting protein 1 [B. mutus]
ENSBTAP00000025138-D1	-5.72	9.11E-10	↑	hypothetical protein M91_02566, partial [B. mutus]
ENSP00000353695-D1	-5.71	5.62E-59	↑	hypothetical protein M91_16426, partial [B. mutus]
ENSBTAP00000014144-D1	-5.71	7.16E-31	↑	Pro-neuregulin-2, membrane-bound isoform, partial [B. mutus]
ENSP00000326846-D1	-5.69	3.87E-58	↑	uncharacterized protein C14orf28 homolog isoform X1 [Ovis aries]
ENSP00000265340-D2	-5.67	1.48E-09	↑	Pituitary homeobox 1, partial [B. mutus]
ENSP00000280758-D1	-5.67	1.65E-57	↑	Ankyrin repeat and BTB/POZ domain-containing protein BTBD11, partial [B. mutus]
ENSP00000311291-D1	-5.64	1.31E-29	↑	Solute carrier organic anion transporter family member 2A1, partial [B. mutus]
ENSP00000416177-D1	-5.61	1.31E-81	↑	Putative E3 ubiquitin-protein ligase MARCH10, partial [B. mutus]
ENSBTAP00000037032-D1	-5.60	9.03E-159	↑	zinc finger protein 3 [B. mutus]
ENSP00000418668-D1	-5.59	2.84E-197	↑	muscle-related coiled-coil protein [B. mutus]
ENSBTAP00000020514-D1	-5.57	2.18E-155	↑	hypothetical protein M91_13526, partial [B. mutus]
ENSBTAP00000009437-D1	-5.56	1.75E-142	↑	P2X purinoceptor 1 [B. mutus]
ENSBTAP00000008613-D1	-5.56	8.00E-41	↑	vang-like protein 2 [B. mutus]
ENSP00000287152-D1	-5.55	5.96E-129	↑	Kinesin-like protein KIF6, partial [B. mutus]
ENSBTAP00000010022-D1	-5.54	1.11E-127	↑	Cathepsin D, partial [B. mutus]
ENSP00000317905-D1	-5.54	6.50E-09	↑	hypothetical protein M91_05252 [B. mutus]
ENSP00000207870-D1	-5.54	6.50E-09	↑	Xylulose kinase, partial [B. mutus]
ENSP00000363729-D1	-5.53	1.24E-89	↑	Transcription elongation factor A protein 3, partial [B. mutus]
ENSP00000294543-D1	-5.52	5.08E-52	↑	Transmembrane and coiled-coil domain-containing protein 4, partial [B. mutus]
ENSP00000397669-D1	-5.50	2.52E-136	↑	Phosphatase and actin regulator 1, partial [B. mutus]
ENSP00000393649-D1	-5.49	8.95E-15	↑	hypothetical protein M91_14019, partial [B. mutus]
ENSBTAP00000018307-D2	-5.49	1.06E-08	↑	keratin, type II cuticular Hb6 [B. taurus]
ENSP00000266671-D1	-5.49	1.06E-08	↑	PHLDA1 protein, partial [Homo sapiens]
ENSBTAP00000026477-D1	-5.49	1.06E-08	↑	Phosphoinositide 3-kinase adapter protein 1, partial [B. mutus]
ENSP00000357794-D1	-5.46	0.00E+00	↑	Trichohyalin, partial [B. mutus]
ENSP00000307598-D55	-5.46	2.24E-85	↑	hypothetical protein M91_06797, partial [B. mutus]
ENSBTAP00000023285-D1	-5.46	5.40E-97	↑	Zinc finger protein 236, partial [B. mutus]
ENSBTAP00000003033-D1	-5.44	1.75E-08	↑	Phosphatidylinositol 3-kinase regulatory subunit beta, partial [B. mutus]
ENSP00000166139-D1	-5.44	1.75E-08	↑	Follistatin-related protein 3, partial [B. mutus]
ENSP00000365395-D1	-5.44	1.75E-08	↑	arylacetamide deacetylase-like 4 [B. mutus]
ENSP00000307023-D1	-5.42	0.00E+00	↑	Krueppel-like factor 11, partial [B. mutus]
ENSBTAP00000008215-D1	-5.42	3.34E-37	↑	uncharacterized protein KIAA1614 homolog [B. mutus]
ENSP00000295559-D1	-5.42	1.86E-151	↑	kinesin-like protein KIF21B [Bubalus bubalis]
ENSP00000348897-D1	-5.41	5.47E-37	↑	gamma-aminobutyric acid receptor subunit alpha-2 isoform X2 [Ovis aries]
ENSBTAP00000000846-D1	-5.40	2.36E-25	↑	Protein S100-A6, partial [B. mutus]
ENSBTAP00000049559-D1	-5.39	2.44E-70	↑	TBC1 domain family member 24, partial [B. mutus]
ENSP00000303511-D1	-5.39	2.89E-08	↑	Peroxisome assembly factor 2, partial [B. mutus]
ENSP00000269554-D1	-5.37	1.88E-80	↑	phosphatidylinositol 5-phosphate 4-kinase type-2 beta [B. taurus]
ENSBTAP00000027623-D1	-5.37	1.03E-157	↑	dehydrogenase/reductase SDR family member 7 [B. mutus]
ENSBTAP00000015162-D1	-5.36	1.20E-57	↑	B-cell differentiation antigen CD72 [B. mutus]
ENSBTAP00000020285-D1	-5.27	7.94E-08	↑	Delta-1-pyrroline-5-carboxylate dehydrogenase, mitochondrial, partial [B. mutus]
ENSBTAP00000005033-D6	-5.27	7.94E-08	↑	hypothetical protein M91_01777, partial [B. mutus]
ENSBTAP00000018151-D1	-5.26	2.04E-74	↑	neuronal acetylcholine receptor subunit alpha-5 [B. mutus]
ENSP00000369833-D1	-5.25	5.58E-23	↑	Diamine acetyltransferase 2, partial [B. mutus]
ENSBTAP00000028111-D1	-5.24	1.92E-83	↑	Aldose 1-epimerase, partial [B. mutus]
ENSP00000216492-D1	-5.24	3.12E-103	↑	Chromogranin-A [B. mutus]
ENSBTAP00000018244-D1	-5.23	1.43E-62	↑	Glutamate receptor delta-2 subunit, partial [B. mutus]
ENSBTAP00000002979-D1	-5.22	2.97E-92	↑	chondroitin sulfate proteoglycan 5 precursor [B. taurus]
ENSP00000309689-D1	-5.22	1.31E-12	↑	Leucine-rich repeat and calponin-like protein domain-containing protein 4, partial [B. mutus]
ENSBTAP00000013316-D1	-5.19	2.10E-80	↑	Protocadherin Fat 4 [B. mutus]
ENSP00000233575-D2	-5.17	9.95E-51	↑	sorting nexin-17 [B. taurus]
ENSBTAP00000012705-D1	-5.17	1.50E-165	↑	A-kinase anchor protein 5 [B. mutus]
ENSP00000420659-D1	-5.15	1.13E-21	↑	kelch-like protein 29 [B. mutus]

ENSP00000357076-D1	-5.14	1.87E-21	↑	Transgelin-2, partial [B. mutus]
ENSP00000407950-D1	-5.13	2.57E-114	↑	Drebrin-like protein [B. mutus]
ENSBTAP00000042049-D1	-5.11	8.76E-58	↑	phospholemman precursor [B. taurus]
ENSBTAP00000024829-D1	-5.11	7.90E-76	↑	Serine/threonine-protein phosphatase 6 regulatory subunit 2, partial [B. mutus]
ENSP00000372445-D1	-5.11	1.45E-57	↑	methyltransferase-like protein 17, mitochondrial precursor [B. taurus]
ENSBTAP00000044707-D1	-5.11	2.64E-39	↑	La-related protein 6, partial [B. mutus]
ENSBTAP00000003272-D1	-5.09	3.59E-75	↑	synaptotagmin-5 [B. taurus]
ENSP00000294064-D1	-5.09	3.67E-07	↑	Sialidase-3, partial [B. mutus]
ENSBTAP00000029167-D1	-5.09	8.44E-102	↑	atypical kinase ADCK3, mitochondrial [B. taurus]
ENSBTAP00000043718-D1	-5.08	2.43E-92	↑	homeodomain leucine zipper protein [B. taurus]
ENSBTAP00000014920-D1	-5.06	0.00E+00	↑	Forkhead box protein O3, partial [B. mutus]
ENSBTAP00000005146-D1	-5.04	9.66E-64	↑	kinesin family member 5A [Camelus ferus]
ENSP00000355060-D1	-5.03	9.68E-29	↑	Rho guanine nucleotide exchange factor 10-like protein, partial [B. mutus]
ENSBTAP00000052456-D2	-5.03	9.68E-29	↑	peroxisomal membrane protein 11A isoform X1 [B. mutus]
ENSP00000392549-D1	-5.03	7.85E-89	↑	tubulin monoglycylase TTLL3 [B. mutus]
ENSBTAP00000053525-D1	-5.01	3.77E-79	↑	EVI5-like protein [B. mutus]
ENSP00000353766-D1	-5.01	1.07E-19	↑	phosphatidylinositol 4,5-bisphosphate 3-kinase catalytic subunit delta isoform isoform X1 [Orcinus orca]
ENSBTAP00000016791-D1	-5.00	4.43E-28	↑	Putative transcription factor Ovo-like 1, partial [B. mutus]
ENSP00000297146-D2	-4.99	4.56E-11	↑	probable G-protein coupled receptor 173 [B. taurus]
ENSP00000389770-D1	-4.99	4.56E-11	↑	FXYD domain-containing ion transport regulator 3, partial [B. mutus]
ENSBTAP00000051424-D1	-4.97	4.98E-69	↑	retrotransposon-derived protein PEG10 isoform 4 [B. taurus]
ENSBTAP00000027459-D1	-4.95	4.92E-19	↑	ATP-binding cassette sub-family B member 6, mitochondrial [B. taurus]
ENSBTAP00000006320-D1	-4.95	7.61E-11	↑	hypothetical protein M91_04687, partial [B. mutus]
ENSBTAP00000021753-D1	-4.95	1.04E-06	↑	Syntaxin-10 [B. mutus]
ENSP00000351255-D1	-4.94	3.61E-197	↑	Signal transducer and activator of transcription 4, partial [B. mutus]
ENSBTAP00000018862-D1	-4.91	5.89E-145	↑	Nuclear receptor coactivator 5, partial [B. mutus]
ENSBTAP00000031803-D2	-4.91	7.16E-216	↑	interferon alpha-inducible protein 27-like protein 2-like [B. mutus]
ENSBTAP00000040863-D2	-4.90	1.41E-57	↑	BTB/POZ domain-containing protein 2 isoform X2 [Ovis aries musimon]
ENSP00000200181-D1	-4.89	8.69E-104	↑	integrin beta-4 isoform X5 [Ovis aries musimon]
ENSP00000293328-D1	-4.88	4.75E-157	↑	Signal transducer and activator of transcription 5B [B. mutus]
ENSP00000336522-D1	-4.88	3.78E-18	↑	myotubularin-related protein 5, partial [B. mutus]
ENSP00000261937-D1	-4.88	1.75E-06	↑	Vascular endothelial growth factor receptor 3 [B. mutus]
ENSP00000377547-D1	-4.88	1.75E-06	↑	Cyclin-J-like protein [B. mutus]
ENSBTAP00000034555-D8	-4.88	1.75E-06	↑	hypothetical protein M91_15076, partial [B. mutus]
ENSBTAP00000022679-D1	-4.88	1.75E-06	↑	potassium/sodium hyperpolarization-activated cyclic nucleotide-gated channel 3 [B. mutus]
ENSBTAP00000007537-D1	-4.88	1.75E-06	↑	Transcription factor GATA-6 [B. mutus]
ENSP00000300176-D1	-4.85	4.59E-63	↑	Arf-GAP domain and FG repeats-containing protein 2 [B. mutus]
ENSP00000350136-D1	-4.84	3.02E-205	↑	beta-cityl-glutamate synthase B [B. mutus]
ENSP00000257013-D1	-4.80	2.95E-06	↑	protein FAM127 [B. taurus]
ENSBTAP00000009885-D1	-4.80	2.95E-06	↑	Peroxisomal membrane protein 2 [B. mutus]
ENSBTAP00000027421-D1	-4.79	4.13E-154	↑	putative histone-lysine N-methyltransferase PRDM6 isoform X6 [Ovis aries musimon]
ENSBTAP00000035716-D1	-4.78	2.75E-81	↑	Sorbitol dehydrogenase, partial [B. mutus]
ENSBTAP00000009698-D1	-4.78	9.78E-60	↑	lipoma HMGIC fusion partner-like 2 protein [B. taurus]
ENSP00000390849-D1	-4.75	1.49E-44	↑	1-acylglycerol-3-phosphate O-acyltransferase ABHD5 [B. mutus]
ENSBTAP00000015459-D1	-4.74	0.00E+00	↑	Serine/threonine-protein kinase 11 [B. mutus]
ENSBTAP00000025871-D1	-4.73	6.35E-51	↑	dapper homolog 1 [B. mutus]
ENSBTAP00000023676-D1	-4.72	4.99E-06	↑	zinc transporter ZIP9 isoform X1 [Bubalus bubalis]
ENSBTAP00000006005-D1	-4.72	4.99E-06	↑	RING finger protein 183 [B. mutus]
ENSP00000282499-D1	-4.72	4.99E-06	↑	glutamate receptor 4, partial [B. mutus]
ENSBTAP00000052498-D1	-4.70	3.85E-16	↑	Aspartate beta-hydroxylase domain-containing protein 2, partial [B. mutus]
ENSP00000346398-D1	-4.68	1.71E-95	↑	Parathyroid hormone-related protein, partial [B. mutus]
ENSBTAP00000019989-D1	-4.67	1.57E-22	↑	hypothetical protein M91_05948, partial [B. mutus]
ENSBTAP00000021833-D1	-4.67	1.46E-107	↑	leukotriene A-4 hydrolase [B. taurus]
ENSBTAP00000052206-D1	-4.63	8.04E-73	↑	retinol dehydrogenase 12-like [B. mutus]
ENSP00000377542-D1	-4.61	7.18E-103	↑	WW domain-binding protein 1 [B. mutus]

ENSP00000377435-D1	-4.61	3.03E-15	↑	T-box transcription factor TBX4 isoform X2 [B. taurus]
ENSP00000408288-D1	-4.58	7.99E-09	↑	unnamed protein product [Homo sapiens]
ENSBTAP00000053387-D1	-4.58	2.78E-76	↑	Zinc finger protein 335, partial [B. mutus]
ENSP00000323288-D1	-4.56	4.89E-51	↑	RNA pseudouridylate synthase domain-containing protein 2 [B. mutus]
ENSBTAP00000050407-D4	-4.56	6.81E-27	↑	Serine/threonine-protein phosphatase 6 regulatory ankyrin repeat subunit A, partial [B. mutus]
ENSBTAP00000015285-D1	-4.54	3.49E-80	↑	calsequestrin-2 [B. mutus]
ENSBTAP00000053211-D2	-4.54	1.46E-05	↑	MOSC domain-containing protein 2, mitochondrial [B. mutus]
ENSP00000367255-D1	-4.54	1.46E-05	↑	SCO-spondin, partial [B. mutus]
ENSP00000361423-D1	-4.53	0.00E+00	↑	Tyrosine-protein kinase ABL1, partial [B. mutus]
ENSBTAP00000008994-D1	-4.51	6.13E-55	↑	Acylphosphatase-2, partial [B. mutus]
ENSP00000376860-D1	-4.51	6.13E-55	↑	protein unc-79 homolog isoform X7 [Ovis aries musimon]
ENSBTAP00000018768-D1	-4.50	5.81E-83	↑	protein kinase C zeta type [B. taurus]
ENSBTAP00000042196-D1	-4.49	1.52E-25	↑	Serine protease 53, partial [B. mutus]
ENSBTAP00000027245-D1	-4.47	6.66E-177	↑	zinc finger protein 398 [B. taurus]
ENSP00000389128-D1	-4.46	2.06E-86	↑	Leucine-rich repeat-containing protein 15, partial [B. mutus]
ENSP00000367062-D17	-4.44	3.86E-08	↑	Histone H3.1 [Myotis brandtii]
ENSP00000348489-D1	-4.44	3.86E-08	↑	Putative fibrosin-1 long transcript protein, partial [B. mutus]
ENSBTAP00000005378-D1	-4.44	2.49E-05	↑	mitogen-activated protein kinase kinase kinase 11 isoform X2 [B. mutus]
ENSBTAP00000016087-D1	-4.44	2.49E-05	↑	Nasal embryonic luteinizing hormone-releasing hormone factor, partial [B. mutus]
ENSBTAP00000018580-D1	-4.44	2.49E-05	↑	extracellular superoxide dismutase [Cu-Zn] [B. mutus]
ENSBTAP00000007505-D1	-4.43	2.54E-46	↑	Protein fucU-like protein, partial [B. mutus]
ENSP00000318604-D1	-4.43	1.44E-241	↑	repressor of RNA polymerase III transcription MAF1 homolog isoform X1 [Balaenoptera acutorostrata scammoni]
ENSP00000404011-D2	-4.42	6.82E-262	↑	Keratin, type II cytoskeletal 8, partial [B. mutus]
ENSP00000310472-D5	-4.42	2.63E-67	↑	Zinc finger protein 383, partial [B. mutus]
ENSBTAP00000049268-D3	-4.41	1.93E-13	↑	hypothetical protein M91_09555, partial [B. mutus]
ENSBTAP00000016505-D1	-4.40	1.05E-34	↑	pyridoxal phosphate phosphatase, partial [B. mutus]
ENSP00000381652-D1	-4.40	6.45E-56	↑	Protein TSSC1, partial [B. mutus]
ENSBTAP00000000431-D1	-4.40	6.07E-278	↑	ENTH domain-containing protein 1 [B. mutus]
ENSP00000244751-D1	-4.39	1.08E-81	↑	copine-5 [B. taurus]
ENSBTAP00000045833-D1	-4.39	1.67E-39	↑	complement factor I [B. mutus]
ENSBTAP00000039906-D3	-4.39	6.53E-08	↑	epididymis-specific alpha-mannosidase-like, partial [B. mutus]
ENSBTAP00000041719-D1	-4.38	0.00E+00	↑	myosin light chain 6B [B. taurus]
ENSBTAP00000053836-D1	-4.38	5.12E-81	↑	run domain Beclin-1 interacting and cysteine-rich containing protein-like isoform X1 [B. mutus]
ENSBTAP00000052082-D1	-4.38	4.91E-60	↑	integrator complex subunit 11 [B. mutus]
ENSP00000360028-D5	-4.37	8.34E-34	↑	glutathione S-transferase A4 isoform X1 [B. taurus]
ENSBTAP00000000556-D1	-4.37	0.00E+00	↑	alpha-crystallin B chain [B. taurus]
ENSP00000353073-D1	-4.36	1.70E-162	↑	battenin isoform X1 [B. mutus]
ENSP00000253968-D1	-4.36	6.81E-80	↑	Homeobox protein BarH-like 1, partial [B. mutus]
ENSP00000257548-D1	-4.36	2.10E-69	↑	ubiquitin carboxyl-terminal hydrolase 30 [B. taurus]
ENSBTAP00000023578-D1	-4.35	1.92E-79	↑	Zinc finger protein ZFAT, partial [B. mutus]
ENSBTAP00000011949-D1	-4.34	2.02E-48	↑	Mitogen-activated protein kinase kinase kinase 5, partial [B. mutus]
ENSBTAP00000053381-D1	-4.33	7.66E-23	↑	Putative protein KIAA0556, partial [B. mutus]
ENSBTAP00000019149-D1	-4.33	1.10E-07	↑	Vinexin [B. mutus]
ENSBTAP00000050715-D1	-4.33	1.10E-07	↑	uncharacterized protein C15orf43 homolog, partial [B. mutus]
ENSBTAP00000025132-D1	-4.33	4.26E-05	↑	Synaptotagmin-6, partial [B. mutus]
ENSP00000333633-D3	-4.32	1.61E-152	↑	metastasis-associated protein MTA2 isoform X2 [Bubalus bubalis]
ENSBTAP00000022024-D1	-4.32	1.06E-37	↑	SPRY domain-containing SOCS box protein 3 [B. taurus]
ENSP00000316092-D1	-4.32	3.39E-132	↑	valine--tRNA ligase, mitochondrial [B. mutus]
ENSP00000350894-D1	-4.30	7.64E-47	↑	serpin H1 precursor [B. taurus]
ENSBTAP00000012863-D1	-4.30	0.00E+00	↑	Golgin subfamily A member 3 [B. mutus]
ENSP00000258415-D1	-4.30	1.96E-56	↑	sterol 26-hydroxylase, mitochondrial-like [B. mutus]
ENSBTAP00000002419-D1	-4.29	2.35E-17	↑	ubiquinone biosynthesis protein COQ9, mitochondrial [B. mutus]
ENSP00000389015-D1	-4.27	1.88E-07	↑	Serine/threonine-protein kinase SBK2, partial [B. mutus]
ENSBTAP00000000513-D1	-4.27	1.88E-07	↑	nanos homolog 3 isoform X1 [Bubalus bubalis]
ENSP00000268676-D1	-4.26	2.74E-50	↑	Differentially expressed in FDCP 8-like protein, partial [B. mutus]

ENSBTAP00000000561-D1	-4.26	1.90E-64	↑	heat shock protein beta-2 [B. mutus]
ENSBTAP00000007080-D1	-4.26	1.07E-40	↑	hepatitis delta antigen interacting protein A [Homo sapiens]
ENSP00000337140-D1	-4.25	1.13E-35	↑	unnamed protein product [Homo sapiens]
ENSP00000253458-D1	-4.25	2.67E-148	↑	genetic suppressor element 1 [B. mutus]
ENSP00000397269-D1	-4.25	4.44E-12	↑	protein FAM136A isoform 2 [Gorilla gorilla gorilla]
ENSP00000359001-D1	-4.24	0.00E+00	↑	Host cell factor 1 [B. mutus]
ENSBTAP00000002867-D1	-4.23	0.00E+00	↑	Glucosamine--fructose-6-phosphate aminotransferase [isomerizing] 2, partial [B. mutus]
ENSP00000339381-D1	-4.22	1.44E-39	↑	Regulator of G-protein signaling 12, partial [B. mutus]
ENSP00000251808-D1	-4.22	3.87E-44	↑	Grainyhead-like protein 2-like protein, partial [B. mutus]
ENSP00000372394-D1	-4.22	4.92E-21	↑	transmembrane protein 129 [B. mutus]
ENSP00000264895-D1	-4.21	4.08E-39	↑	extracellular matrix protein FRAS1 [B. mutus]
ENSP00000379078-D1	-4.18	1.38E-127	↑	Histone-lysine N-methyltransferase, H3 lysine-9 specific 3 [B. mutus]
ENSBTAP00000011906-D1	-4.18	1.05E-131	↑	AP-3 complex subunit delta-1 [B. mutus]
ENSBTAP00000051893-D1	-4.17	2.35E-20	↑	kynurenine--oxoglutarate transaminase 1-like, partial [B. mutus]
ENSBTAP00000018572-D1	-4.17	2.35E-20	↑	Phosphatidylinositol 4-kinase alpha, partial [B. mutus]
ENSBTAP00000041167-D1	-4.17	2.79E-64	↑	Extended synaptotagmin-2, partial [B. mutus]
ENSP00000290866-D1	-4.17	2.79E-64	↑	Angiotensin-converting enzyme, partial [B. mutus]
ENSP00000255641-D1	-4.17	1.04E-24	↑	casein kinase I isoform gamma-2 [B. taurus]
ENSBTAP00000018304-D1	-4.16	8.46E-51	↑	Heat shock 70 kDa protein 12A, partial [B. mutus]
ENSP00000371682-D1	-4.15	1.55E-37	↑	DDB1- and CUL4-associated factor 16 [Lipotes vexillifer]
ENSP00000366278-D1	-4.15	5.46E-07	↑	mediator of RNA polymerase II transcription subunit 25 isoform X1 [Bubalus bubalis]
ENSP00000307870-D1	-4.15	1.49E-201	↑	copper chaperone for superoxide dismutase isoform X2 [B. taurus]
ENSP00000404464-D1	-4.15	1.18E-41	↑	TPA: KIAA1983 protein-like [B. taurus]
ENSP00000216101-D1	-4.15	1.18E-41	↑	ras-like protein family member 10A [B. mutus]
ENSP00000347427-D1	-4.14	1.38E-179	↑	Misshapen-like kinase 1, partial [B. mutus]
ENSBTAP00000015210-D1	-4.14	8.30E-59	↑	Semaphorin-7A, partial [B. mutus]
ENSBTAP00000025703-D1	-4.14	2.85E-67	↑	BTB/POZ domain-containing protein 3, partial [B. mutus]
ENSP00000332530-D1	-4.14	2.94E-24	↑	hypothetical protein M91_00091, partial [B. mutus]
ENSP00000319104-D1	-4.13	0.00E+00	↑	Transcription elongation factor SPT6 [B. mutus]
ENSBTAP00000020995-D1	-4.13	9.68E-203	↑	Zinc finger protein 609, partial [B. mutus]
ENSP00000368880-D1	-4.12	0.00E+00	↑	RecName: Full=Forkhead box protein O1; AltName: Full=Forkhead box protein O1A; AltName: Full=Forkhead in rhabdomyosarcoma
ENSP00000354822-D1	-4.12	1.67E-129	↑	XIAP-associated factor 1 [B. mutus]
ENSP00000379769-D1	-4.12	0.00E+00	↑	Aconitate hydratase, mitochondrial, partial [B. mutus]
ENSBTAP00000023198-D1	-4.12	8.49E-62	↑	tetraspanin-17 isoform X1 [Pantholops hodgsonii]
ENSP00000350928-D1	-4.11	0.00E+00	↑	glutamate decarboxylase 1 [B. taurus]
ENSBTAP00000010294-D1	-4.10	2.58E-48	↑	REST corepressor 2 [Bison bison bison]
ENSP00000403941-D1	-4.09	5.72E-193	↑	myeloid leukemia factor 2 isoform X1 [Capra hircus]
ENSBTAP00000023734-D1	-4.09	9.30E-07	↑	Integral membrane protein 2C, partial [B. mutus]
yakG040728	-4.09	9.30E-07	↑	hypothetical protein M91_09212 [B. mutus]
ENSP00000382713-D1	-4.09	1.28E-04	↑	Ubiquitin-conjugating enzyme E2Q-like protein 1, partial [B. mutus]
ENSP00000392188-D40	-4.09	1.28E-04	↑	hypothetical protein M91_11961, partial [B. mutus]
ENSBTAP00000046239-D13	-4.09	1.28E-04	↑	hypothetical protein M91_01394, partial [B. mutus]
ENSBTAP00000013898-D1	-4.09	1.28E-04	↑	Envoplakin [B. mutus]
ENSP00000317534-D1	-4.09	1.28E-04	↑	zinc finger and BTB domain-containing protein 34 isoform X1 [B. taurus]
ENSBTAP00000027940-D1	-4.09	1.28E-04	↑	Tumor necrosis factor receptor superfamily member 16, partial [B. mutus]
ENSBTAP00000016890-D1	-4.08	3.97E-23	↑	neuroserpin isoform X1 [B. mutus]
ENSP00000364240-D1	-4.07	3.48E-47	↑	UBX domain-containing protein 10 [B. mutus]
ENSBTAP00000017759-D1	-4.04	2.17E-42	↑	myotonin-protein kinase isoform X7 [B. taurus]
ENSP00000335185-D1	-4.04	3.47E-58	↑	prickle-like protein 4 [B. mutus]
ENSBTAP00000002334-D1	-4.02	1.58E-06	↑	serine--tRNA ligase, mitochondrial [B. mutus]
ENSBTAP00000021265-D1	-4.01	4.50E-76	↑	nuclear distribution protein nudE homolog 1 [B. taurus]
ENSP00000317468-D1	-4.01	2.75E-68	↑	charged multivesicular body protein 6 [B. mutus]
ENSP00000365493-D1	-4.01	2.48E-191	↑	general transcription factor IIH subunit 4 [B. mutus]
ENSBTAP00000041074-D1	-4.00	2.49E-125	↑	probable ATP-dependent RNA helicase DDX41 [Ovis aries]

ENSP00000293677-D1	-4.00	4.77E-60	↑	ribonucleoprotein PTB-binding 1 isoform X1 [B. mutus]
ENSBTAP00000049387-D4	-4.00	3.71E-56	↑	Dimethylaniline monooxygenase [N-oxide-forming] 1, partial [B. mutus]
ENSP00000363794-D1	-4.00	1.83E-223	↑	Estradiol 17-beta-dehydrogenase 8, partial [B. mutus]
ENSP00000351650-D6	-3.98	5.92E-14	↑	diphosphoinositol polyphosphate phosphohydrolase 2 isoform X1 [Cavia porcellus]
ENSBTAP00000028663-D1	-3.97	0.00E+00	↑	Ubiquitin carboxyl-terminal hydrolase 36 [B. mutus]
ENSBTAP00000009480-D1	-3.97	2.96E-66	↑	striatin-interacting protein 2 [Bison bison bison]
ENSP00000006777-D1	-3.97	2.94E-77	↑	Rhomboid domain-containing protein 2 [B. mutus]
ENSBTAP00000001426-D1	-3.96	6.53E-51	↑	calretinin [Ovis aries]
ENSBTAP00000021516-D1	-3.95	2.06E-17	↑	keratin, type II cytoskeletal 7 [B. mutus]
ENSP00000332565-D1	-3.95	1.00E-13	↑	sulfotransferase 4A1 [B. taurus]
ENSP00000281523-D2	-3.95	2.22E-04	↑	Zinc finger protein 385D, partial [B. mutus]
ENSP00000007264-D1	-3.95	2.22E-04	↑	RNA pseudouridylate synthase domain-containing protein 1 [B. mutus]
ENSP00000242209-D3	-3.95	2.22E-04	↑	Peptidyl-prolyl cis-trans isomerase FKBP10, partial [B. mutus]
ENSP00000332875-D1	-3.95	2.22E-04	↑	UPF0573 protein C2orf70 homolog [B. mutus]
ENSP00000334437-D1	-3.95	2.22E-04	↑	Nucleoside diphosphate-linked moiety X motif 17 [B. mutus]
ENSP00000393308-D1	-3.95	2.22E-04	↑	von Willebrand factor A domain-containing protein 5B2 [B. mutus]
ENSP00000386869-D1	-3.92	9.23E-128	↑	Putative methylcytosine dioxygenase TET3, partial [B. mutus]
ENSP00000378529-D1	-3.92	5.38E-42	↑	fizzy-related protein homolog isoform X1 [B. taurus]
ENSP00000326110-D1	-3.91	9.06E-42	↑	UNC119-binding protein C5orf30 homolog [B. mutus]
ENSBTAP00000000697-D1	-3.90	9.25E-31	↑	zinc finger protein 395 [B. mutus]
ENSBTAP00000001970-D1	-3.90	3.40E-62	↑	G protein-coupled receptor kinase 4 [B. mutus]
ENSBTAP00000011195-D1	-3.88	1.66E-16	↑	Zinc finger MYM-type protein 3 [B. mutus]
ENSBTAP00000021234-D1	-3.88	4.79E-13	↑	acyl-CoA synthetase family member 3, mitochondrial [B. mutus]
ENSP00000381950-D2	-3.88	4.79E-13	↑	Transmembrane protein 216, partial [B. mutus]
ENSBTAP00000006080-D1	-3.88	4.60E-06	↑	Lethal(2) giant larvae protein-like protein 1, partial [B. mutus]
ENSP00000254480-D1	-3.88	0.00E+00	↑	SWI/SNF complex subunit SMARCC1, partial [B. mutus]
ENSBTAP00000009715-D1	-3.87	4.46E-47	↑	Synaptic vesicle membrane protein VAT-1-like protein, partial [B. mutus]
ENSP00000296318-D1	-3.86	1.09E-104	↑	Interleukin-17 receptor D [B. mutus]
ENSBTAP00000046715-D1	-3.86	8.82E-118	↑	BEN domain-containing protein 4, partial [B. mutus]
ENSBTAP00000034758-D1	-3.85	1.06E-76	↑	Post-GPI attachment to proteins factor 3 [B. mutus]
ENSBTAP00000021501-D1	-3.85	6.02E-258	↑	Disabled-like protein 2 [B. mutus]
ENSBTAP00000042463-D1	-3.84	2.41E-09	↑	proSAAS precursor [B. taurus]
ENSP00000361285-D1	-3.84	2.41E-09	↑	Uridine-cytidine kinase 1, partial [B. mutus]
ENSP00000005905-D1	-3.84	2.88E-59	↑	UPF0378 protein KIAA0100, partial [B. mutus]
ENSBTAP00000019774-D1	-3.82	1.68E-45	↑	GPN-loop GTPase 2, partial [B. mutus]
ENSBTAP00000011242-D1	-3.82	1.39E-61	↑	Tricarboxylate transport protein, mitochondrial, partial [B. mutus]
ENSP00000362888-D1	-3.80	3.85E-04	↑	2-aminoethanethiol dioxygenase, partial [B. mutus]
ENSBTAP00000051169-D1	-3.80	3.85E-04	↑	insulin-like growth factor-binding protein 6 isoform X2 [Bison bison bison]
ENSBTAP00000013382-D1	-3.80	3.85E-04	↑	Semaphorin-3B [B. mutus]
ENSP00000310193-D1	-3.80	3.85E-04	↑	carabin isoform X1 [B. taurus]
ENSP00000182527-D2	-3.78	0.00E+00	↑	hypothetical protein M91_11384, partial [B. mutus]
ENSP00000402140-D1	-3.77	7.03E-301	↑	Nostrin [B. mutus]
ENSBTAP00000045413-D1	-3.77	0.00E+00	↑	hypothetical protein M91_09218, partial [B. mutus]
ENSBTAP00000021778-D1	-3.77	2.57E-65	↑	opioid growth factor receptor-like protein 1-like [B. mutus]
ENSBTAP00000018985-D1	-3.77	1.44E-171	↑	protein Wnt-2b [B. mutus]
ENSP00000367265-D1	-3.76	2.67E-71	↑	Cytoskeleton-associated protein 4, partial [B. mutus]
ENSBTAP00000041430-D1	-3.76	2.99E-43	↑	proline-rich protein 19 [B. taurus]
ENSBTAP00000020376-D1	-3.76	1.49E-108	↑	transcriptional-regulating factor 1 isoform X4 [B. taurus]
ENSBTAP00000024112-D1	-3.75	5.13E-46	↑	mitochondrial fission process protein 1 [B. taurus]
ENSBTAP00000032874-D1	-3.75	2.03E-64	↑	arginine vasopressin-induced protein 1 [B. mutus]
ENSBTAP00000018252-D1	-3.75	2.16E-122	↑	Prominin-1, partial [B. mutus]
ENSP00000264659-D1	-3.74	3.75E-27	↑	SRC kinase signaling inhibitor 1 [Pteropus vampyrus]
ENSBTAP00000002327-D1	-3.74	1.08E-130	↑	NAD-dependent protein deacetylase sirtuin-2 [B. taurus]
ENSP00000319139-D1	-3.74	1.18E-148	↑	Dual specificity mitogen-activated protein kinase kinase 3, partial [B. mutus]
ENSP00000382163-D1	-3.73	6.26E-18	↑	SET domain-containing protein 4 [Bison bison bison]

ENSP00000293925-D1	-3.73	6.30E-27	↑	Protein cramped-like protein, partial [B. mutus]
ENSBTAP00000011818-D2	-3.72	0.00E+00	↑	ubiquitin carboxyl-terminal hydrolase 22 [B. mutus]
ENSBTAP00000001955-D1	-3.72	7.02E-51	↑	Acetolactate synthase-like protein, partial [B. mutus]
ENSP00000317891-D1	-3.72	7.19E-54	↑	TNFAIP3-interacting protein 1 [B. mutus]
ENSBTAP00000041215-D1	-3.72	1.16E-08	↑	TNF receptor-associated factor 1 [B. mutus]
ENSBTAP00000004411-D1	-3.72	1.16E-08	↑	protein-arginine deiminase type-2 isoform X4 [B. taurus]
ENSBTAP00000053503-D1	-3.71	4.03E-80	↑	uncharacterized protein C11orf93 homolog [B. mutus]
ENSBTAP00000010992-D1	-3.69	0.00E+00	↑	3 beta-hydroxysteroid dehydrogenase, steroid isomerase [cattle, Peptide, 372 aa]
ENSP00000379852-D97	-3.69	5.00E-29	↑	Zinc finger protein 271, partial [B. mutus]
ENSBTAP00000026034-D1	-3.69	2.96E-20	↑	microfibrillar-associated protein 3-like [B. mutus]
ENSBTAP00000019822-D1	-3.69	1.48E-113	↑	discoidin, CUB and LCCL domain-containing protein 2 [B. mutus]
ENSBTAP00000027003-D1	-3.69	4.68E-58	↑	Cadherin-like protein 26, partial [B. mutus]
ENSBTAP00000008112-D1	-3.69	2.57E-81	↑	Zinc finger protein 76 [B. mutus]
ENSP00000366702-D1	-3.69	7.94E-96	↑	ERBB receptor feedback inhibitor 1 [B. taurus]
ENSP00000320543-D1	-3.68	7.75E-93	↑	epsin-2 isoform X2 [B. mutus]
ENSP00000320081-D1	-3.68	3.51E-124	↑	hypothetical protein M91_10250, partial [B. mutus]
ENSP00000262367-D1	-3.68	0.00E+00	↑	CREB-binding protein [B. mutus]
ENSBTAP00000021579-D1	-3.67	7.80E-170	↑	RNA-binding protein 43, partial [B. mutus]
ENSP00000328998-D1	-3.67	3.53E-86	↑	trafficking kinesin-binding protein 1 isoform X1 [B. mutus]
ENSP00000261650-D1	-3.67	0.00E+00	↑	RNA demethylase ALKBH5 isoform X2 [Ovis aries musimon]
ENSBTAP00000012633-D1	-3.67	4.63E-123	↑	STAM-binding protein isoform X2 [B. mutus]
ENSP00000323858-D1	-3.66	2.23E-125	↑	ATP-dependent RNA helicase DDX54 isoform X1 [B. mutus]
ENSP00000313419-D1	-3.66	9.52E-80	↑	B-lymphocyte antigen CD19 [Bison bison bison]
ENSP00000367185-D1	-3.66	2.37E-28	↑	ly6/PLAUR domain-containing protein 5 [B. mutus]
ENSBTAP00000021634-D1	-3.66	3.13E-11	↑	Peptidyl-prolyl cis-trans isomerase FKBP8 [B. mutus]
ENSBTAP00000023199-D1	-3.65	6.75E-99	↑	serine/threonine-protein kinase 10 [B. mutus]
ENSP00000376827-D1	-3.65	7.38E-147	↑	Meckel syndrome type 1 protein [B. mutus]
ENSBTAP00000052975-D1	-3.65	1.51E-70	↑	Arf-GAP with dual PH domain-containing protein 1, partial [B. mutus]
ENSP00000294244-D1	-3.64	3.97E-25	↑	hypothetical protein M91_13022, partial [B. mutus]
ENSP00000352995-D1	-3.64	1.96E-212	↑	TPA: Rho guanine nucleotide exchange factor 18-like [B. taurus]
ENSP00000361823-D1	-3.64	1.51E-100	↑	Protein BHLHb9, partial [B. mutus]
ENSBTAP00000020325-D1	-3.64	0.00E+00	↑	outer dense fiber protein 2 isoform X1 [B. mutus]
ENSBTAP00000001851-D1	-3.63	5.96E-108	↑	hemK methyltransferase family member 2 [B. taurus]
ENSBTAP00000043881-D1	-3.63	6.71E-04	↑	growth hormone-inducible transmembrane protein [B. taurus]
ENSBTAP00000030954-D1	-3.63	6.71E-04	↑	Nose resistant to fluoxetine protein 6, partial [B. mutus]
ENSBTAP00000036275-D1	-3.63	6.71E-04	↑	Steroid hormone receptor ERR1, partial [B. mutus]
ENSP00000223210-D1	-3.63	6.71E-04	↑	Zinc finger protein 862, partial [B. mutus]
ENSBTAP00000003470-D1	-3.63	6.71E-04	↑	TPA: transmembrane protein 132E [B. taurus]
ENSBTAP00000022032-D1	-3.63	6.71E-04	↑	nitrogen permease regulator 3-like protein [B. mutus]
ENSBTAP00000026739-D7	-3.63	6.71E-04	↑	ankyrin repeat domain-containing protein 26 [B. taurus]
ENSP00000251535-D3	-3.63	6.71E-04	↑	arachidonate 12-lipoxygenase, epidermal-type-like [B. mutus]
ENSBTAP00000021009-D1	-3.63	6.71E-04	↑	properdin [B. mutus]
ENSBTAP00000034490-D6	-3.63	6.71E-04	↑	multidrug resistance-associated protein 4-like isoform X1 [B. taurus]
ENSP00000385865-D3	-3.62	2.28E-82	↑	Retinoic acid receptor beta, partial [B. mutus]
ENSP00000366190-D1	-3.62	0.00E+00	↑	Dr1-associated corepressor, partial [B. mutus]
ENSBTAP00000025438-D1	-3.62	1.29E-46	↑	Growth arrest-specific protein 7, partial [B. mutus]
ENSBTAP00000017672-D1	-3.62	3.83E-145	↑	SURP and G-patch domain-containing protein 2 [B. mutus]
ENSBTAP00000016977-D1	-3.62	1.09E-84	↑	Macrophage colony-stimulating factor 1 receptor, partial [B. mutus]
ENSBTAP00000043307-D1	-3.61	1.26E-43	↑	FH1/FH2 domain-containing protein 1 [Pantholops hodgsonii]
ENSBTAP00000009564-D1	-3.61	3.00E-141	↑	serotransferrin [Bison bison bison]
ENSP00000303999-D1	-3.61	2.52E-62	↑	.
ENSP00000233969-D1	-3.59	4.92E-51	↑	solute carrier family 9, subfamily A (NHE2, cation proton antiporter 2), member 2 [B. mutus]
ENSBTAP00000002247-D1	-3.59	8.17E-78	↑	major facilitator superfamily domain-containing protein 9 [B. mutus]
ENSP00000225512-D1	-3.59	1.49E-29	↑	proto-oncogene Wnt-3 precursor [B. taurus]
ENSBTAP00000015397-D1	-3.59	8.03E-236	↑	dual specificity protein phosphatase CDC14B isoform X3 [Bubalus bubalis]

ENSP00000280057-D1	-3.59	7.12E-35	↑	Protein FAM124A, partial [B. mutus]
ENSBTAP00000011732-D1	-3.59	1.08E-106	↑	transmembrane protein 98 [Ovis aries]
ENSP00000268603-D1	-3.58	2.50E-29	↑	cadherin-11 precursor [B. taurus]
ENSP00000360683-D1	-3.58	5.59E-08	↑	tyrosine-protein phosphatase non-receptor type 1 [B. mutus]
ENSP00000344582-D1	-3.58	0.00E+00	↑	NHS-like protein 1 isoform X2 [B. taurus]
ENSBTAP00000048347-D1	-3.58	8.68E-109	↑	Sulfhydryl oxidase 1, partial [B. mutus]
ENSP00000286067-D1	-3.57	2.20E-221	↑	uncharacterized protein C10orf12 homolog isoform X1 [B. mutus]
ENSBTAP00000004636-D1	-3.57	1.79E-76	↑	Tripartite motif-containing protein 3, partial [B. mutus]
ENSBTAP00000045723-D1	-3.57	9.94E-150	↑	Bromodomain-containing protein 9, partial [B. mutus]
ENSBTAP00000007129-D1	-3.57	1.80E-128	↑	Protein NLRC5 [B. mutus]
ENSBTAP00000050214-D22	-3.57	1.49E-10	↑	endogenous retrovirus group K member 25 Env polyprotein-like [Bison bison bison]
ENSBTAP00000026388-D1	-3.57	1.33E-44	↑	Rhomboid family member 1 [B. mutus]
ENSBTAP00000007589-D1	-3.57	1.44E-78	↑	Phosphoglucomutase-2, partial [B. mutus]
ENSP00000306129-D1	-3.56	0.00E+00	↑	D(1B) dopamine receptor [B. mutus]
ENSBTAP00000012142-D1	-3.56	1.01E-67	↑	mitochondrial import receptor subunit TOM40B [B. taurus]
ENSBTAP00000005345-D1	-3.56	1.59E-36	↑	adenylate kinase 8 [B. mutus]
ENSBTAP00000003325-D1	-3.55	1.89E-157	↑	G patch domain and KOW motifs-containing protein [B. mutus]
ENSBTAP00000050388-D1	-3.55	6.96E-26	↑	Coiled-coil domain-containing protein 97, partial [B. mutus]
ENSP00000268129-D1	-3.55	4.92E-95	↑	abhydrolase domain-containing protein 2 [B. taurus]
ENSP00000259737-D1	-3.54	5.25E-248	↑	protein YIPF3 [B. mutus]
ENSP00000362814-D1	-3.54	1.73E-43	↑	Serine incorporator 2, partial [B. mutus]
ENSBTAP00000009303-D1	-3.54	4.11E-23	↑	Abhydrolase domain-containing protein 1, partial [B. mutus]
ENSP00000304410-D1	-3.54	1.46E-20	↑	UPF0561 protein C2orf68 homolog isoform X1 [B. taurus]
ENSBTAP00000018455-D1	-3.54	3.93E-05	↑	Neuropeptide B, partial [B. mutus]
ENSBTAP00000041768-D1	-3.54	3.93E-05	↑	regulator of G-protein signaling protein-like [B. mutus]
ENSBTAP00000000005-D1	-3.53	1.82E-177	↑	Beta-adrenergic receptor kinase 2, partial [B. mutus]
ENSBTAP00000012611-D1	-3.53	4.30E-129	↑	M-phase inducer phosphatase 1, partial [B. mutus]
ENSBTAP00000003881-D1	-3.53	1.95E-25	↑	alanine aminotransferase 2 isoform X1 [B. taurus]
ENSP00000391056-D1	-3.53	2.93E-68	↑	synaptotagmin-1-like protein [Cricetulus griseus]
ENSBTAP00000018957-D1	-3.52	2.83E-256	↑	Zinc finger CCHC domain-containing protein 14 [B. mutus]
ENSBTAP00000024447-D1	-3.52	0.00E+00	↑	Wiskott-Aldrich syndrome protein family member 2, partial [B. mutus]
ENSP00000356809-D1	-3.52	7.38E-58	↑	G-protein coupled receptor 161 isoform X2 [Bison bison bison]
ENSBTAP00000025981-D1	-3.52	9.14E-78	↑	Alpha-1D adrenergic receptor [B. mutus]
ENSP00000344087-D1	-3.51	2.71E-187	↑	autism susceptibility candidate 2 [B. mutus]
ENSBTAP00000015883-D1	-3.51	0.00E+00	↑	Heat shock protein beta-1 [B. mutus]
ENSP00000177742-D1	-3.51	6.68E-120	↑	28S ribosomal protein S34, mitochondrial, partial [B. mutus]
ENSBTAP00000051202-D1	-3.50	1.46E-49	↑	Lambda-crystallin-like protein, partial [B. mutus]
ENSBTAP00000010854-D1	-3.50	5.24E-15	↑	Glypican-6, partial [B. mutus]
ENSP00000296328-D1	-3.50	0.00E+00	↑	UBX domain-containing protein 7 [B. mutus]
ENSP00000362942-D1	-3.50	2.93E-307	↑	phosphatase and actin regulator 4 isoform X1 [Bison bison bison]
ENSP00000398930-D1	-3.50	3.39E-206	↑	Epsilon-sarcoglycan [B. mutus]
ENSBTAP00000016559-D1	-3.49	1.90E-12	↑	TBC1 domain family member 13 [B. taurus]
ENSP00000391440-D1	-3.48	2.14E-58	↑	polyhomeotic-like protein 2 [B. mutus]
ENSBTAP00000024724-D1	-3.48	1.41E-135	↑	lysyl oxidase homolog 3 [B. mutus]
ENSP00000367991-D1	-3.48	1.16E-221	↑	Septin-8, partial [B. mutus]
ENSP00000288757-D1	-3.47	0.00E+00	↑	uncharacterized protein C12orf43 homolog isoform X1 [B. mutus]
ENSP00000398366-D1	-3.47	9.93E-58	↑	histone H4 transcription factor isoform X1 [Bison bison bison]
ENSBTAP00000011718-D1	-3.47	1.92E-19	↑	Chymotrypsin-like elastase family member 1 [B. mutus]
ENSP00000377473-D1	-3.46	2.01E-26	↑	protein KIBRA [B. mutus]
ENSBTAP00000011677-D1	-3.46	2.02E-52	↑	Calpain-3 [B. mutus]
ENSBTAP00000022364-D1	-3.46	1.43E-66	↑	Pyrroline-5-carboxylate reductase 3, partial [B. mutus]
ENSBTAP00000043531-D1	-3.46	3.42E-97	↑	presequence protease, mitochondrial [Bison bison bison]
ENSP00000366527-D1	-3.45	1.60E-54	↑	Zinc finger and SCAN domain-containing protein 16 [B. mutus]
ENSP00000413362-D1	-3.45	6.12E-59	↑	TPA: tubulin tyrosine ligase-like family, member 6 [B. taurus]
ENSBTAP00000000994-D1	-3.44	9.35E-52	↑	Histone acetyltransferase KAT2B, partial [B. mutus]

ENSP00000243389-D1	-3.44	5.49E-215	↑	proton-coupled amino acid transporter 1 isoform X2 [B. taurus]
ENSBTAP00000024795-D1	-3.44	2.46E-14	↑	Guanosine-3',5'-bis(diphosphate) 3'-pyrophosphohydrolase MESH1, partial [B. mutus]
ENSP00000267484-D1	-3.44	1.18E-09	↑	Reticulon-1, partial [B. mutus]
ENSP00000308022-D1	-3.44	6.78E-05	↑	Transcriptional adapter 2-beta [B. mutus]
yakA15205	-3.44	1.19E-03	↑	Fatty acid-binding protein, heart [B. mutus]
ENSP00000416979-D58	-3.44	1.19E-03	↑	.
ENSBTAP00000003073-D1	-3.44	8.80E-68	↑	nucleobindin-1 [B. mutus]
ENSBTAP00000020895-D1	-3.44	2.70E-07	↑	PERQ amino acid-rich with GYF domain-containing protein 1, partial [B. mutus]
ENSBTAP00000012917-D2	-3.42	5.89E-218	↑	Interferon-induced protein 44, partial [B. mutus]
ENSP00000328023-D1	-3.42	2.26E-199	↑	signal recognition particle receptor subunit alpha isoform X1 [Pantholops hodgsonii]
ENSBTAP00000011856-D1	-3.42	4.11E-14	↑	Dual specificity protein phosphatase 15, partial [B. mutus]
ENSP00000383958-D1	-3.42	6.76E-87	↑	Nuclear factor 1 X-type, partial [B. mutus]
ENSBTAP00000015973-D1	-3.41	5.85E-96	↑	ARF GTPase-activating protein GIT1, partial [B. mutus]
ENSBTAP00000000498-D1	-3.41	2.14E-166	↑	nicolin-1 [B. taurus]
ENSP00000270112-D1	-3.41	2.16E-34	↑	hormonally up-regulated neu tumor-associated kinase, partial [B. mutus]
ENSP00000356623-D1	-3.40	2.67E-52	↑	cbp/p300-interacting transactivator 2 [B. mutus]
ENSBTAP00000019489-D1	-3.40	3.32E-70	↑	Zinc finger homeobox protein 3, partial [B. mutus]
ENSBTAP00000011251-D1	-3.40	2.98E-173	↑	Suppressor of cytokine signaling 7, partial [B. mutus]
ENSBTAP00000002792-D1	-3.40	2.36E-175	↑	Zinc finger protein 771, partial [B. mutus]
ENSP00000396219-D2	-3.40	3.82E-81	↑	Myocyte-specific enhancer factor 2A [B. mutus]
ENSP00000396032-D1	-3.40	4.34E-25	↑	Putative protein KIAA0802, partial [B. mutus]
ENSBTAP00000044312-D1	-3.39	4.09E-45	↑	protein NOXP20 [B. mutus]
ENSBTAP00000049914-D1	-3.39	4.55E-07	↑	hypothetical protein M91_12293, partial [B. mutus]
ENSBTAP00000001982-D1	-3.38	2.38E-42	↑	Serine/threonine-protein kinase PDIK1L, partial [B. mutus]
ENSP00000262464-D1	-3.38	4.14E-18	↑	fibrillin-2 precursor [B. taurus]
ENSBTAP00000037502-D1	-3.38	4.99E-40	↑	Cation-independent mannose-6-phosphate receptor, partial [B. mutus]
ENSBTAP00000028227-D1	-3.37	8.61E-64	↑	Mitogen-activated protein kinase kinase kinase MLK4, partial [B. mutus]
ENSP00000300584-D1	-3.37	7.12E-90	↑	TBC1 domain family member 2B, partial [B. mutus]
ENSP00000307292-D1	-3.37	2.06E-72	↑	Protein kinase C delta-binding protein, partial [B. mutus]
ENSP00000418356-D1	-3.36	1.72E-78	↑	ras-related protein M-Ras [Myotis davidii]
ENSP00000375986-D1	-3.36	7.55E-74	↑	TPA: MTK1/MEKK4 homolog family member (mtk-1)-like [B. taurus]
ENSP00000292114-D1	-3.35	1.91E-13	↑	transmembrane protein 199 [B. mutus]
ENSBTAP00000052283-D1	-3.35	1.91E-13	↑	transmembrane protein 145 [B. mutus]
ENSP00000370532-D1	-3.35	2.90E-144	↑	transcription factor IIIA [B. mutus]
ENSBTAP00000015277-D1	-3.35	5.02E-172	↑	glycogen phosphorylase, liver form [B. taurus]
ENSBTAP00000009307-D1	-3.35	1.83E-62	↑	prolactin regulatory element-binding protein [B. taurus]
ENSP00000388566-D1	-3.34	1.92E-66	↑	Caspase-13, partial [B. mutus]
ENSP00000411197-D2	-3.34	5.56E-24	↑	TBC1 domain family member 9B, partial [B. mutus]
ENSP00000206380-D1	-3.34	1.33E-36	↑	transmembrane protein 101 [B. mutus]
ENSBTAP00000001461-D1	-3.33	1.18E-21	↑	CKLF-like MARVEL transmembrane domain-containing protein 4, partial [B. mutus]
ENSP00000222718-D1	-3.33	4.19E-11	↑	homeobox protein Hox-A2 [B. mutus]
ENSBTAP00000023648-D1	-3.31	1.25E-29	↑	testis-specific Y-encoded-like protein 2 [B. taurus]
ENSBTAP00000018722-D1	-3.31	1.95E-89	↑	amphoterin-induced protein 1 precursor [B. taurus]
ENSBTAP00000008532-D1	-3.31	4.21E-48	↑	epididymis-specific alpha-mannosidase [Bison bison bison]
ENSBTAP00000011549-D1	-3.30	2.90E-223	↑	sodium/hydrogen exchanger 1 [B. mutus]
ENSBTAP00000003936-D1	-3.30	1.02E-57	↑	Transmembrane protein 86A [B. mutus]
ENSBTAP00000017425-D1	-3.29	2.03E-51	↑	stromal interaction molecule 1 isoform X2 [Bison bison bison]
ENSP00000325355-D1	-3.29	2.44E-45	↑	caskin-2 [B. mutus]
ENSBTAP00000037196-D1	-3.29	6.83E-15	↑	rho GTPase-activating protein 39-like [B. mutus]
ENSBTAP00000026774-D1	-3.29	1.36E-81	↑	p53 apoptosis effector related to PMP-22 [B. taurus]
ENSBTAP00000009042-D1	-3.29	3.22E-47	↑	protein FAM117A [B. mutus]
ENSP00000360035-D1	-3.29	3.36E-51	↑	Protein phosphatase 1 regulatory subunit 3D, partial [B. mutus]
ENSBTAP00000025702-D2	-3.28	1.18E-70	↑	transcription factor MafG isoform X2 [Cavia porcellus]
ENSBTAP00000003424-D1	-3.28	1.11E-44	↑	probable small intestine urate exporter isoform X1 [B. taurus]
ENSP00000350881-D1	-3.27	1.55E-217	↑	GRAM domain-containing protein 1C isoform X2 [Ovis aries musimon]

ENSBTAP00000023201-D1	-3.27	9.42E-96	↑	Calcitonin receptor, partial [B. mutus]
ENSP00000376910-D1	-3.27	2.14E-123	↑	Leucine-rich repeat and IQ domain-containing protein 1, partial [B. mutus]
ENSP00000194118-D1	-3.26	1.42E-87	↑	death ligand signal enhancer [B. mutus]
ENSP00000337226-D1	-3.26	5.09E-44	↑	Cell division cycle-associated protein 4 [B. mutus]
ENSBTAP00000042659-D1	-3.26	1.77E-63	↑	Nucleoside diphosphate-linked moiety X motif 19, mitochondrial, partial [B. mutus]
ENSP00000356282-D1	-3.25	1.55E-08	↑	Ladinin-1, partial [B. mutus]
ENSP00000171887-D1	-3.25	1.07E-41	↑	tensin-1 isoform X1 [B. mutus]
ENSP00000254442-D1	-3.25	2.54E-51	↑	WD repeat-containing protein 7 [B. mutus]
ENSP00000401477-D1	-3.25	1.34E-107	↑	Transmembrane protein 232, partial [B. mutus]
ENSBTAP00000014779-D1	-3.25	1.34E-39	↑	Myosin-IXb, partial [B. mutus]
ENSP00000371175-D1	-3.25	3.17E-18	↑	ADP-ribosylation factor-binding protein GGA1, partial [B. mutus]
ENSP00000363298-D1	-3.25	1.94E-10	↑	B box and SPRY domain-containing protein [B. mutus]
ENSBTAP00000006353-D1	-3.25	1.94E-10	↑	homeobox protein Hox-D3 [B. mutus]
ENSBTAP00000005618-D1	-3.24	4.03E-47	↑	hypothetical protein M91_15871 [B. mutus]
ENSP00000358154-D7	-3.24	2.46E-12	↑	hypothetical protein M91_02497, partial [B. mutus]
ENSBTAP00000009255-D1	-3.24	9.17E-26	↑	vesicular glutamate transporter 1 [B. taurus]
ENSBTAP00000001367-D1	-3.23	1.06E-42	↑	homeobox protein Hox-A1 [B. taurus]
ENSP00000385099-D1	-3.22	3.44E-106	↑	Zinc finger protein 792, partial [B. mutus]
ENSP00000408176-D1	-3.22	3.81E-44	↑	A/G-specific adenine DNA glycosylase, partial [B. mutus]
ENSBTAP00000004368-D1	-3.22	3.29E-27	↑	patatin-like phospholipase domain-containing protein 3 isoform X3 [B. taurus]
ENSP00000293777-D1	-3.22	1.92E-23	↑	mediator of RNA polymerase II transcription subunit 11 [Ovis aries]
ENSBTAP00000029033-D1	-3.22	2.17E-06	↑	Selenoprotein N, partial [B. mutus]
ENSBTAP00000044812-D1	-3.22	1.98E-04	↑	uncharacterized protein C4orf46 homolog [B. mutus]
ENSBTAP00000020676-D1	-3.22	1.98E-04	↑	Opioid growth factor receptor, partial [B. mutus]
ENSBTAP00000021677-D1	-3.22	2.10E-03	↑	ribonuclease P protein subunit p25-like protein [B. taurus]
ENSBTAP00000040176-D1	-3.22	2.10E-03	↑	Beta-2-glycoprotein 1, partial [B. mutus]
ENSBTAP00000028569-D1	-3.22	2.10E-03	↑	Transmembrane epididymal protein 1, partial [B. mutus]
ENSBTAP00000005522-D1	-3.22	2.10E-03	↑	Tumor necrosis factor receptor superfamily member 1A, partial [B. mutus]
ENSP00000386456-D1	-3.21	2.39E-96	↑	AP2-associated protein kinase 1 isoform X3 [B. taurus]
ENSP00000354453-D1	-3.21	1.96E-103	↑	zinc finger protein 2 homolog [B. taurus]
ENSBTAP00000020785-D1	-3.20	2.45E-21	↑	Tenascin-N [B. mutus]
ENSBTAP00000049701-D1	-3.20	3.47E-36	↑	transmembrane protein 150B [B. mutus]
ENSBTAP00000023012-D1	-3.20	1.88E-19	↑	AT-hook-containing transcription factor [B. mutus]
ENSBTAP00000019773-D1	-3.20	2.64E-34	↑	G patch domain-containing protein 3 [B. mutus]
ENSP00000085068-D1	-3.20	1.12E-15	↑	Isochorismatase domain-containing protein 2, mitochondrial [B. mutus]
ENSP00000303192-D1	-3.20	2.31E-211	↑	ral guanine nucleotide dissociation stimulator-like 1 [B. taurus]
ENSP00000362979-D1	-3.19	7.14E-58	↑	homeobox protein TGIF2 [B. mutus]
ENSBTAP00000025006-D1	-3.19	1.55E-83	↑	Negative elongation factor D, partial [B. mutus]
ENSBTAP00000010660-D1	-3.19	4.72E-43	↑	RNA-binding protein 38 [B. taurus]
ENSBTAP00000021048-D1	-3.18	1.48E-55	↑	Protein FAM168B, partial [B. mutus]
ENSP00000385395-D1	-3.18	8.05E-154	↑	Glutamate receptor delta-1 subunit, partial [B. mutus]
ENSP00000390595-D1	-3.18	0.00E+00	↑	TPA: sprouty-related, EVH1 domain containing 2-like [B. taurus]
ENSP00000332624-D1	-3.18	6.09E-70	↑	molybdate-anion transporter [Bison bison bison]
ENSBTAP00000003176-D1	-3.18	4.51E-39	↑	hypothetical protein M91_13779 [B. mutus]
ENSP00000352956-D1	-3.18	6.72E-21	↑	probable RNA-binding protein 23 isoform X1 [Bison bison bison]
ENSP00000210444-D1	-3.17	1.54E-60	↑	sialic acid synthase [B. mutus]
ENSP00000342481-D1	-3.17	2.81E-44	↑	Putative phospholipid-transporting ATPase IIA, partial [B. mutus]
ENSBTAP00000042728-D1	-3.17	3.31E-179	↑	ras-GEF domain-containing family member 1B isoform X1 [Bubalus bubalis]
ENSP00000333367-D2	-3.17	0.00E+00	↑	DNA-binding protein SATB1, partial [B. mutus]
ENSBTAP00000015985-D1	-3.17	0.00E+00	↑	Histone-arginine methyltransferase CARM1, partial [B. mutus]
ENSP00000358865-D1	-3.16	4.09E-85	↑	Alpha-internexin, partial [B. mutus]
ENSP00000419153-D1	-3.16	5.85E-42	↑	Zinc finger and BTB domain-containing protein 20, partial [B. mutus]
ENSBTAP00000019348-D1	-3.16	4.79E-49	↑	Cas scaffolding protein family member 4, partial [B. mutus]
ENSBTAP00000028559-D1	-3.16	1.27E-43	↑	tyrosine-protein kinase CSK [B. taurus]
ENSP00000390724-D1	-3.15	9.94E-205	↑	Ribonuclease inhibitor, partial [B. mutus]

ENSP00000410735-D1	-3.15	3.67E-77	↑	Coiled-coil domain-containing protein 57 [B. mutus]
ENSP00000359337-D1	-3.15	4.51E-130	↑	gamma-aminobutyric acid receptor subunit alpha-3 isoform X1 [B. mutus]
ENSP00000305918-D1	-3.15	7.76E-247	↑	bromodomain-containing protein 3 isoform X2 [Balaenoptera acutorostrata scammoni]
ENSBTAP00000010155-D1	-3.15	6.12E-107	↑	3-ketodihydrosphingosine reductase, partial [B. mutus]
ENSBTAP00000022005-D1	-3.15	3.97E-22	↑	Laminin subunit beta-3 [B. mutus]
ENSP00000261888-D1	-3.15	1.41E-18	↑	mono [ADP-ribose] polymerase PARP16 [B. mutus]
ENSP00000384817-D1	-3.14	1.65E-216	↑	Paired box protein Pax-9, partial [B. mutus]
ENSBTAP00000035894-D1	-3.14	3.04E-20	↑	G1/S-specific cyclin-D3, partial [B. mutus]
ENSBTAP00000004174-D1	-3.14	1.51E-128	↑	Kinesin-like protein KIF3B, partial [B. mutus]
ENSBTAP00000027093-D1	-3.14	3.03E-201	↑	Ubiquinone biosynthesis monooxygenase COQ6, partial [B. mutus]
ENSP00000232975-D1	-3.14	2.45E-32	↑	troponin C, slow skeletal and cardiac muscles-like protein [Camelus ferus]
ENSP00000311713-D1	-3.13	3.69E-53	↑	serine/threonine-protein kinase OSR1 isoform X1 [Ovis aries]
ENSBTAP00000053695-D1	-3.13	2.33E-18	↑	ski oncogene, partial [B. mutus]
ENSBTAP00000012410-D1	-3.13	0.00E+00	↑	solute carrier family 25 member 44 [B. taurus]
ENSP00000307863-D1	-3.13	0.00E+00	↑	Splicing factor U2AF 65 kDa subunit, partial [B. mutus]
ENSP00000387292-D1	-3.13	0.00E+00	↑	transmembrane protein 150A precursor [B. taurus]
ENSP00000328083-D1	-3.13	5.42E-39	↑	rho guanine nucleotide exchange factor 37 [B. mutus]
ENSP00000381590-D1	-3.13	8.25E-258	↑	Ataxin-7, partial [B. mutus]
ENSP00000356357-D1	-3.12	1.18E-107	↑	LIM/homeobox protein Lhx9 isoform X1 [B. mutus]
ENSP00000264555-D1	-3.12	2.61E-78	↑	PHD and RING finger domain-containing protein 1 [B. mutus]
ENSBTAP00000019208-D1	-3.12	6.79E-37	↑	GTP-binding protein 5 [B. mutus]
ENSBTAP00000000927-D1	-3.12	1.66E-125	↑	integrin-linked protein kinase [B. taurus]
ENSP00000393248-D3	-3.11	7.32E-161	↑	Polyadenylate-binding protein 4, partial [B. mutus]
ENSBTAP00000019032-D1	-3.11	2.46E-111	↑	Atlastin-1, partial [B. mutus]
ENSBTAP00000021039-D1	-3.11	1.16E-263	↑	Piwi-like protein 2 [B. mutus]
ENSP00000380116-D1	-3.11	5.30E-40	↑	hypothetical protein M91_17395, partial [B. mutus]
ENSBTAP00000026474-D1	-3.10	6.36E-18	↑	microprocessor complex subunit DGCR8 [B. mutus]
ENSP00000367476-D1	-3.10	5.45E-82	↑	centrosomal protein of 104 kDa, partial [B. mutus]
ENSP00000331741-D1	-3.10	8.47E-35	↑	homeobox protein Hox-B2 [B. mutus]
ENSP00000361949-D2	-3.10	6.47E-33	↑	Polyadenylate-binding protein 1-like protein, partial [B. mutus]
ENSBTAP00000021469-D1	-3.10	1.65E-101	↑	ADP-ribosylation factor-binding protein GGA3, partial [B. mutus]
ENSP00000409259-D1	-3.09	0.00E+00	↑	Cyclin-dependent kinase inhibitor 1, partial [B. mutus]
ENSP00000416175-D1	-3.09	1.95E-89	↑	KAT8 regulatory NSL complex subunit 3 isoform X1 [B. mutus]
ENSBTAP00000026124-D1	-3.09	1.07E-32	↑	Retinoic acid-induced protein 3, partial [B. mutus]
ENSP00000295304-D1	-3.09	4.94E-31	↑	Cation transport regulator-like protein 2, partial [B. mutus]
ENSBTAP00000020600-D1	-3.09	1.05E-17	↑	Protein fat-free-like protein, partial [B. mutus]
ENSBTAP00000042087-D1	-3.09	5.15E-11	↑	UPF0669 protein C6orf120 homolog [Bubalus bubalis]
ENSBTAP00000024015-D1	-3.09	3.36E-04	↑	proteasome subunit beta type-10 [B. mutus]
ENSBTAP00000006923-D1	-3.08	0.00E+00	↑	osteopontin isoform X1 [B. taurus]
ENSBTAP00000019142-D1	-3.08	3.15E-138	↑	Krueppel-like factor 10, partial [B. mutus]
ENSP00000300835-D1	-3.08	0.00E+00	↑	proline-rich protein 14 [B. mutus]
ENSP00000276440-D1	-3.07	0.00E+00	↑	Dedicator of cytokinesis protein 5, partial [B. mutus]
ENSP00000350704-D1	-3.07	1.09E-43	↑	uncharacterized protein C1orf109 homolog [B. mutus]
ENSP00000387089-D1	-3.07	6.60E-232	↑	NGFI-A-binding protein 1 isoform X1 [B. mutus]
ENSP00000346490-D1	-3.07	1.53E-61	↑	cullin-9 [B. mutus]
ENSBTAP00000005002-D1	-3.07	2.24E-35	↑	R-spondin-1 [Bison bison bison]
ENSP00000356595-D1	-3.07	2.49E-110	↑	Abelson tyrosine-protein kinase 2 isoform X1 [Bison bison bison]
ENSP00000276681-D1	-3.07	8.99E-112	↑	Protein MAL2, partial [B. mutus]
ENSBTAP00000008078-D1	-3.07	1.79E-12	↑	peroxisomal membrane protein 11A [B. taurus]
ENSBTAP00000019460-D2	-3.07	6.57E-50	↑	hypothetical protein M91_20028, partial [B. mutus]
ENSP00000248673-D1	-3.07	2.86E-87	↑	ZFP36 protein [B. taurus]
ENSBTAP00000029107-D1	-3.06	5.15E-53	↑	Transcription elongation factor A protein 2, partial [B. mutus]
ENSP00000366387-D1	-3.06	3.31E-243	↑	MAM domain-containing protein 2 [B. mutus]
ENSP00000332407-D1	-3.06	1.78E-49	↑	short-chain dehydrogenase/reductase family 42E member 1 [B. mutus]
ENSP00000306625-D1	-3.06	1.12E-60	↑	tether containing UBX domain for GLUT4 [B. mutus]

ENSP00000264932-D1	-3.05	2.51E-79	↑	succinate dehydrogenase complex, subunit A, flavoprotein precursor [B. taurus]
ENSP00000383600-D1	-3.05	1.97E-82	↑	transmembrane protein 51 [B. taurus]
ENSP00000379531-D1	-3.04	7.93E-49	↑	Transmembrane channel-like protein 5, partial [B. mutus]
ENSBTAP00000001141-D1	-3.04	2.28E-64	↑	F-box/LRR-repeat protein 6 [B. mutus]
ENSP00000291577-D1	-3.04	2.76E-45	↑	ES1 protein-like protein, mitochondrial [B. mutus]
ENSBTAP00000028313-D1	-3.03	1.62E-29	↑	uncharacterized protein C7orf26 homolog [B. mutus]
ENSBTAP00000053653-D7	-3.03	1.25E-32	↑	Myosin-7, partial [B. mutus]
ENSBTAP00000012602-D1	-3.03	3.94E-92	↑	UBX domain-containing protein 11 [B. mutus]
ENSBTAP00000019080-D2	-3.03	1.62E-270	↑	RING finger protein 126, partial [B. mutus]
ENSBTAP00000011353-D1	-3.03	8.52E-106	↑	Exocyst complex component 3, partial [B. mutus]
ENSP00000361982-D1	-3.03	9.58E-42	↑	probable tRNA pseudouridine synthase 2 [B. mutus]
ENSBTAP00000053112-D1	-3.02	1.02E-05	↑	39S ribosomal protein L54, mitochondrial [B. mutus]
ENSP00000297375-D1	-3.02	1.02E-05	↑	Homeobox protein engrailed-2, partial [B. mutus]
ENSP00000339992-D1	-3.02	8.36E-130	↑	transcriptional activator Myb isoform X1 [B. mutus]
ENSP00000401371-D1	-3.02	1.69E-92	↑	nucleolysin TIA-1 isoform p40 isoform X2 [Ovis aries]
ENSBTAP00000018145-D1	-3.01	0.00E+00	↑	transcription initiation factor IIA subunit 1 [Condylura cristata]
ENSP00000360826-D1	-3.01	1.60E-70	↑	cyclin-dependent kinase 4 inhibitor C [B. taurus]
ENSP00000368450-D1	-3.01	2.63E-24	↑	CD83 antigen isoform X1 [B. mutus]
ENSBTAP00000042524-D1	-3.01	5.54E-55	↑	L-serine dehydratase/L-threonine deaminase [B. mutus]
ENSP00000406585-D1	-3.01	1.96E-33	↑	uncharacterized protein LOC102327815 [Pantholops hodgsonii]
ENSBTAP00000008101-D1	-3.01	1.51E-42	↑	sulfite oxidase, mitochondrial [B. taurus]
ENSBTAP00000006739-D2	-3.00	1.49E-101	↑	bone morphogenetic protein 4 precursor [Bubalus bubalis]
ENSBTAP00000052227-D1	-3.00	3.30E-07	↑	hypothetical protein M91_00465, partial [B. mutus]
ENSBTAP00000016866-D1	-3.00	1.90E-63	↑	hypothetical protein M91_01616, partial [B. mutus]
ENSP00000311399-D1	-3.00	8.64E-115	↑	Down syndrome critical region protein 3 isoform X1 [B. mutus]
ENSBTAP00000020819-D2	-2.99	8.38E-104	↑	Potassium voltage-gated channel subfamily C member 3, partial [B. mutus]
ENSBTAP00000003273-D1	-2.99	3.18E-209	↑	Methylthioribose-1-phosphate isomerase, partial [B. mutus]
ENSP00000311401-D1	-2.99	0.00E+00	↑	protein DENND6A [B. mutus]
ENSP00000315664-D1	-2.98	9.93E-103	↑	Zinc finger protein 18 [B. mutus]
ENSBTAP00000047824-D1	-2.98	2.82E-89	↑	ADP-ribosylation factor GTPase-activating protein 1 [B. mutus]
ENSP00000376127-D1	-2.98	2.84E-77	↑	Cohesin loading complex subunit SCC4-like protein, partial [B. mutus]
ENSBTAP00000001239-D1	-2.98	1.21E-17	↑	zinc finger protein 746 [B. taurus]
ENSBTAP00000013154-D1	-2.98	1.21E-17	↑	inhibin alpha chain [B. mutus]
yakG010302	-2.98	9.27E-134	↑	Phosphorylase b kinase regulatory subunit beta [B. mutus]
ENSBTAP00000052423-D1	-2.97	0.00E+00	↑	Leucine-rich repeat flightless-interacting protein 2, partial [B. mutus]
ENSBTAP00000020316-D1	-2.97	9.63E-147	↑	casein kinase I isoform epsilon-like isoform X1 [B. mutus]
ENSP00000408478-D2	-2.97	1.43E-105	↑	Mastermind-like protein 1, partial [B. mutus]
ENSBTAP00000007042-D1	-2.96	3.12E-23	↑	vacuolar protein sorting-associated protein 11 homolog isoform X1 [B. mutus]
ENSBTAP00000001491-D1	-2.95	5.62E-204	↑	Coronin-6, partial [B. mutus]
ENSBTAP00000010226-D1	-2.95	2.54E-56	↑	N-glycosylase/DNA lyase [B. taurus]
ENSP00000258381-D1	-2.95	2.54E-56	↑	sp110 nuclear body protein isoform X3 [Bison bison bison]
ENSBTAP00000020501-D1	-2.95	9.26E-16	↑	PDZ and LIM domain protein 4, partial [B. mutus]
ENSP00000335651-D1	-2.95	1.71E-05	↑	fibronectin type III and SPRY domain-containing protein 2 isoform X1 [B. mutus]
ENSP00000395818-D1	-2.95	3.76E-03	↑	Geminin [B. mutus]
ENSP00000382756-D4	-2.95	3.76E-03	↑	hypothetical protein M91_04266, partial [B. mutus]
ENSBTAP00000018213-D1	-2.95	3.76E-03	↑	Sorting nexin-8, partial [B. mutus]
ENSP00000231749-D1	-2.95	3.76E-03	↑	zinc finger MYND domain-containing protein 10 [B. mutus]
ENSBTAP00000010835-D1	-2.95	3.76E-03	↑	Toll-interacting protein, partial [B. mutus]
ENSBTAP00000050270-D1	-2.95	3.76E-03	↑	DDB1- and CUL4-associated factor 12-like protein 2-like [B. mutus]
ENSBTAP00000019293-D1	-2.95	3.76E-03	↑	galactoside 2-alpha-L-fucosyltransferase 2-like [B. mutus]
ENSBTAP00000023255-D1	-2.95	3.76E-03	↑	ATP synthase subunit e, mitochondrial, partial [B. mutus]
ENSBTAP00000019912-D1	-2.95	3.76E-03	↑	Muellerian-inhibiting factor [B. mutus]
ENSBTAP00000014038-D2	-2.94	7.85E-79	↑	calcineurin subunit B type 1 isoform X2 [Aotus nancymaae]
ENSBTAP00000011633-D1	-2.94	5.33E-53	↑	apolipoprotein M precursor [B. taurus]
ENSBTAP00000048313-D1	-2.94	4.15E-50	↑	Tripartite motif-containing protein 26 [B. mutus]

ENSBTAP00000020811-D1	-2.94	5.68E-212	↑	Transmembrane protein 194B, partial [B. mutus]
ENSBTAP00000041309-D1	-2.94	7.69E-166	↑	peroxisomal multifunctional enzyme type 2 isoform X2 [B. mutus]
ENSP00000404547-D1	-2.94	6.68E-20	↑	hypothetical protein M91_08272, partial [B. mutus]
ENSBTAP00000037474-D1	-2.94	1.52E-15	↑	lethal(3)malignant brain tumor-like protein 2 [B. taurus]
ENSBTAP00000015378-D1	-2.93	7.21E-37	↑	Beta-1,3-N-acetylglucosaminyltransferase radical fringe, partial [B. mutus]
ENSP00000342144-D1	-2.93	4.87E-24	↑	Limbin, partial [B. mutus]
ENSBTAP00000001918-D1	-2.93	7.92E-198	↑	probable G-protein coupled receptor 156 isoform X1 [B. mutus]
ENSP00000313875-D1	-2.93	1.58E-70	↑	Membrane cofactor protein, partial [B. mutus]
ENSP00000262891-D1	-2.93	3.57E-11	↑	MAP/microtubule affinity-regulating kinase 4, partial [B. mutus]
ENSBTAP00000013868-D1	-2.93	9.17E-178	↑	solute carrier family 40 member 1 isoform X1 [B. mutus]
ENSBTAP00000028016-D1	-2.92	2.08E-136	↑	Cathepsin K [B. mutus]
ENSP00000392204-D1	-2.92	8.75E-17	↑	Killin, partial [B. mutus]
ENSBTAP00000046675-D7	-2.92	5.82E-28	↑	putative ankyrin repeat domain-containing protein ENSP00000383069 [Ovis aries musimon]
ENSP00000367705-D1	-2.92	2.49E-259	↑	BCL-6 corepressor [B. mutus]
ENSP00000350556-D1	-2.92	4.63E-25	↑	hypothetical protein M91_14229, partial [B. mutus]
ENSP00000297596-D1	-2.91	2.03E-12	↑	GTP-binding protein GEM isoform X2 [Bison bison bison]
yakG027741	-2.91	2.01E-55	↑	Metalloproteinase inhibitor 2 [B. mutus]
ENSP00000354730-D1	-2.91	0.00E+00	↑	chromosome alignment-maintaining phosphoprotein 1 [B. mutus]
ENSBTAP00000013100-D1	-2.90	8.37E-36	↑	POLR2A protein [Homo sapiens]
ENSBTAP00000051195-D1	-2.90	6.23E-66	↑	RecName: Full=Progesterone receptor; Short=PR; AltName: Full=Nuclear receptor subfamily 3 group C member 3, partial [Ovis aries]
ENSBTAP00000023277-D1	-2.90	5.39E-81	↑	G1/S-specific cyclin-D1 [B. mutus]
ENSBTAP00000016763-D1	-2.90	0.00E+00	↑	Zinc finger protein 362, partial [B. mutus]
ENSBTAP00000041322-D1	-2.90	0.00E+00	↑	hypothetical protein M91_00923 [B. mutus]
ENSBTAP00000005681-D1	-2.90	2.45E-53	↑	coiled-coil domain-containing protein 87 [B. mutus]
ENSBTAP00000010247-D1	-2.90	2.57E-98	↑	GRIP1-associated protein 1 [B. mutus]
ENSBTAP00000029067-D1	-2.89	5.23E-30	↑	tachykinin-3 [B. mutus]
ENSBTAP00000015811-D1	-2.89	6.23E-60	↑	glycerol-3-phosphate acyltransferase 1, mitochondrial isoform X1 [Bison bison bison]
ENSBTAP00000015257-D1	-2.89	6.51E-79	↑	Inositol polyphosphate 5-phosphatase K, partial [B. mutus]
ENSBTAP00000002065-D1	-2.89	1.10E-123	↑	ADP-dependent glucokinase [B. mutus]
ENSP00000331305-D1	-2.89	4.78E-19	↑	protein Tob2 [B. mutus]
ENSP00000333602-D1	-2.89	6.25E-34	↑	E1A-binding protein p400 [B. mutus]
ENSP00000322016-D1	-2.89	4.56E-130	↑	Poly(U)-binding-splicing factor PUF60, partial [B. mutus]
ENSBTAP00000000301-D1	-2.89	0.00E+00	↑	Double-stranded RNA-binding protein Staufen-like protein 1, partial [B. mutus]
ENSP00000278951-D1	-2.88	1.91E-90	↑	SID1 transmembrane family member 2, partial [B. mutus]
ENSP00000359380-D1	-2.88	3.22E-129	↑	acyl-CoA desaturase [B. mutus]
ENSBTAP00000006413-D1	-2.88	3.28E-24	↑	caM kinase-like vesicle-associated protein isoform X3 [Ovis aries]
ENSBTAP00000018765-D1	-2.88	1.60E-21	↑	coiled-coil domain-containing protein 9 [B. taurus]
ENSBTAP00000028094-D1	-2.87	1.66E-33	↑	Multidrug resistance-associated protein 1, partial [B. mutus]
ENSBTAP00000045770-D1	-2.87	3.15E-137	↑	Leukemia NUP98 fusion partner 1-like protein [B. mutus]
ENSBTAP00000005489-D1	-2.86	0.00E+00	↑	vascular endothelial zinc finger 1 [B. taurus]
ENSBTAP00000019468-D1	-2.86	8.89E-267	↑	Wiskott-Aldrich syndrome protein family member 3 [B. mutus]
ENSP00000268624-D1	-2.86	2.78E-09	↑	adenosine deaminase domain-containing protein 2 isoform X2 [B. taurus]
ENSBTAP00000004644-D2	-2.86	1.11E-39	↑	ATP-dependent RNA helicase DDX19B, partial [B. mutus]
ENSP00000210633-D1	-2.86	5.33E-37	↑	Semaphorin-4G [B. mutus]
ENSP00000393170-D1	-2.86	1.84E-106	↑	probable G-protein coupled receptor 63 [B. mutus]
ENSBTAP00000013366-D1	-2.86	7.16E-193	↑	Protein CBFA2T2, partial [B. mutus]
ENSBTAP00000045877-D1	-2.85	3.61E-242	↑	SH3 domain-binding glutamic acid-rich-like protein 2, partial [B. mutus]
ENSBTAP00000008766-D1	-2.85	0.00E+00	↑	transcriptional repressor p66-beta [B. taurus]
ENSP00000354579-D1	-2.85	6.35E-55	↑	CKLF-like MARVEL transmembrane domain-containing protein 3, partial [B. mutus]
ENSBTAP00000003956-D1	-2.85	6.60E-82	↑	ral GTPase-activating protein subunit beta isoform X1 [B. mutus]
ENSBTAP00000000436-D1	-2.85	5.79E-43	↑	Aldehyde dehydrogenase family 16 member A1, partial [B. mutus]
ENSBTAP00000025389-D1	-2.85	6.92E-21	↑	peripheral myelin protein 22 [B. taurus]
ENSP00000376500-D1	-2.84	2.50E-62	↑	TNF receptor-associated factor 3 [B. mutus]
ENSBTAP00000003929-D1	-2.84	1.18E-227	↑	transcription factor Sp1 [B. taurus]

ENSP00000338352-D1	-2.84	1.32E-298	↑	diphosphoinositol polyphosphate phosphohydrolase 2 isoform X1 [B. mutus]
ENSBTAP00000043290-D1	-2.84	4.06E-62	↑	DCN1-like protein 3 [B. taurus]
ENSP00000308022-D3	-2.84	2.31E-23	↑	transcriptional adaptor 2B [B. mutus]
ENSP00000246914-D1	-2.83	2.59E-28	↑	Serine/threonine-protein kinase WNK4, partial [B. mutus]
ENSBTAP00000024450-D1	-2.83	1.66E-15	↑	Caytaxin, partial [B. mutus]
ENSBTAP00000015061-D1	-2.83	7.63E-156	↑	E3 ubiquitin-protein ligase TRIM68 [B. mutus]
ENSBTAP00000052877-D1	-2.83	2.18E-268	↑	GATS-like protein 3 [B. mutus]
ENSP00000335261-D1	-2.83	2.57E-10	↑	trimethyllysine dioxygenase, mitochondrial [B. taurus]
ENSBTAP00000008754-D1	-2.82	6.95E-165	↑	Endoplasmic reticulum resident protein 29, partial [B. mutus]
yakG045259	-2.82	1.46E-11	↑	UDP-N-acetylhexosamine pyrophosphorylase-like protein 1 [B. mutus]
ENSBTAP00000043096-D1	-2.82	1.01E-173	↑	Helicase ARIP4 [B. mutus]
ENSBTAP00000049463-D1	-2.81	8.10E-191	↑	Spermatogenesis-associated protein 6, partial [B. mutus]
ENSBTAP00000030838-D1	-2.81	2.60E-35	↑	neuroblastoma breakpoint family member 6-like protein-like isoform X1 [B. mutus]
ENSP00000415430-D1	-2.81	0.00E+00	↑	G2 and S phase-expressed protein 1, partial [B. mutus]
ENSP00000362904-D1	-2.81	0.00E+00	↑	protein 4.1 isoform X1 [B. mutus]
ENSP00000401831-D1	-2.81	8.79E-38	↑	Acyl-CoA synthetase family member 2, mitochondrial [B. mutus]
ENSBTAP00000045264-D1	-2.81	5.11E-39	↑	Putative E3 ubiquitin-protein ligase RNF217, partial [B. mutus]
ENSBTAP00000025681-D1	-2.81	7.26E-34	↑	F-box/LRR-repeat protein 17, partial [B. mutus]
ENSP00000368972-D1	-2.80	7.41E-160	↑	MAP7 domain-containing protein 2 [B. mutus]
ENSP00000330358-D1	-2.80	4.83E-20	↑	Zinc finger protein 623, partial [B. mutus]
ENSP00000381523-D1	-2.80	2.37E-11	↑	zinc finger and BTB domain-containing protein 21 [B. mutus]
yakG001560	-2.80	9.83E-04	↑	Vacuolar protein sorting-associated protein 35 [B. mutus]
ENSP00000216259-D1	-2.80	2.21E-59	↑	phosphomannomutase 1 [B. taurus]
ENSBTAP00000053438-D1	-2.80	2.50E-43	↑	Phosphatidylinositol-4-phosphate 5-kinase type-1 gamma, partial [B. mutus]
ENSBTAP00000008938-D1	-2.79	1.57E-50	↑	E3 ubiquitin-protein ligase MARCH3 [B. taurus]
ENSBTAP00000017828-D1	-2.79	7.86E-20	↑	CTD small phosphatase-like protein [B. taurus]
ENSBTAP00000019875-D1	-2.78	1.25E-13	↑	Myosin light chain kinase 2, skeletal/cardiac muscle [B. mutus]
ENSP00000420721-D1	-2.78	5.84E-45	↑	basic helix-loop-helix domain-containing protein KIAA2018 homolog [B. mutus]
ENSP00000303482-D1	-2.78	3.86E-11	↑	receptor expression-enhancing protein 4 [B. mutus]
ENSP00000296444-D1	-2.78	3.86E-11	↑	Protein shisa-5, partial [B. mutus]
ENSBTAP00000018459-D1	-2.78	2.21E-18	↑	E3 ubiquitin-protein ligase TRIM21, partial [B. mutus]
ENSBTAP00000002395-D1	-2.77	8.92E-82	↑	39S ribosomal protein L10, mitochondrial [B. mutus]
ENSP00000264758-D1	-2.77	3.40E-272	↑	Alpha-adducin [B. mutus]
ENSP00000380288-D1	-2.77	1.22E-08	↑	hypothetical protein M91_00213 [B. mutus]
ENSP00000379144-D1	-2.77	1.23E-183	↑	Trinucleotide repeat-containing 6A protein, partial [B. mutus]
ENSBTAP00000007814-D1	-2.76	4.80E-63	↑	radial spoke head protein 3 homolog [B. mutus]
ENSP00000384479-D1	-2.76	1.30E-112	↑	tetratricopeptide repeat protein 18 [B. mutus]
ENSBTAP00000044440-D1	-2.76	0.00E+00	↑	Rho guanine nucleotide exchange factor 12, partial [B. mutus]
ENSBTAP00000011393-D1	-2.76	4.08E-51	↑	Putative E3 ubiquitin-protein ligase TRIML1, partial [B. mutus]
ENSBTAP00000048482-D1	-2.76	0.00E+00	↑	cadherin-1 [B. mutus]
ENSP00000372924-D1	-2.75	0.00E+00	↑	ralA-binding protein 1 [B. taurus]
ENSP00000302777-D1	-2.75	2.59E-80	↑	Tubulin beta-3 chain [B. mutus]
ENSP00000335615-D1	-2.75	1.44E-189	↑	BTB/POZ domain-containing protein 7 [B. mutus]
ENSP00000384734-D2	-2.75	5.18E-227	↑	zinc finger protein GLIS3 [B. mutus]
ENSBTAP00000002250-D1	-2.75	0.00E+00	↑	aurora kinase B [B. taurus]
ENSP00000363640-D1	-2.75	4.07E-06	↑	PHD finger protein 1 isoform X1 [B. mutus]
ENSBTAP00000009379-D3	-2.74	0.00E+00	↑	alpha-endosulfine [Nannospalax galili]
ENSBTAP00000016462-D1	-2.74	5.26E-33	↑	Z-DNA-binding protein 1, partial [B. mutus]
ENSBTAP00000014573-D1	-2.74	3.23E-128	↑	Prospero homeobox protein 1 [B. mutus]
ENSP00000264712-D1	-2.74	3.09E-85	↑	kinesin-like protein KIF3C [B. mutus]
ENSP00000288065-D1	-2.74	1.57E-37	↑	hypothetical protein M91_19449 [B. mutus]
ENSBTAP00000006153-D1	-2.74	5.76E-12	↑	delta(24)-sterol reductase [B. mutus]
ENSBTAP00000026394-D1	-2.74	5.76E-12	↑	Protein FAM195B, partial [B. mutus]
ENSBTAP00000053253-D1	-2.74	0.00E+00	↑	golgi-specific brefeldin A-resistance guanine nucleotide exchange factor 1 isoform X1 [B. mutus]
ENSBTAP00000023373-D1	-2.74	8.52E-33	↑	prelamin-A/C isoform X1 [B. mutus]

ENSBTAP00000010347-D1	-2.74	0.00E+00	↑	glucosamine-6-phosphate isomerase 1 [Camelus ferus]
ENSBTAP00000027587-D1	-2.74	4.62E-35	↑	Chloride transport protein 6, partial [B. mutus]
ENSBTAP00000053032-D1	-2.74	2.32E-75	↑	zinc finger protein 391 [B. mutus]
ENSBTAP00000025752-D1	-2.73	5.34E-13	↑	kinesin light chain 2 [B. taurus]
ENSP00000387882-D1	-2.73	0.00E+00	↑	tandem C2 domains nuclear protein isoform X1 [B. mutus]
ENSBTAP00000043820-D1	-2.72	1.22E-27	↑	Ran-binding protein 10 [B. mutus]
ENSBTAP00000026265-D1	-2.72	7.78E-05	↑	endothelial transcription factor GATA-2 [B. taurus]
ENSP00000324842-D1	-2.72	1.53E-97	↑	acetoacetyl-CoA synthetase [B. mutus]
ENSP00000359290-D1	-2.72	1.85E-167	↑	protein Dr1 [Jaculus jaculus]
ENSP00000416255-D1	-2.71	6.26E-249	↑	eukaryotic translation initiation factor 4 gamma 1 isoform X1 [B. mutus]
ENSP00000235372-D1	-2.70	0.00E+00	↑	PR domain zinc finger protein 2, partial [B. mutus]
ENSP00000329117-D1	-2.70	1.72E-29	↑	APC membrane recruitment protein 1 [B. mutus]
ENSP00000366477-D1	-2.70	2.13E-107	↑	MAM and LDL-receptor class A domain-containing protein 1 [Bison bison bison]
ENSP00000406218-D1	-2.70	1.84E-276	↑	protein PROCA1 [B. mutus]
ENSBTAP00000000481-D1	-2.70	1.15E-168	↑	DIS3-like exonuclease 1, partial [B. mutus]
ENSP00000265968-D1	-2.70	0.00E+00	↑	cysteine and glycine-rich protein 3 [B. taurus]
ENSBTAP00000009921-D1	-2.70	1.41E-32	↑	peroxisomal biogenesis factor 19 isoform X1 [B. mutus]
ENSP00000312999-D5	-2.70	1.01E-23	↑	guanine nucleotide-binding protein G(o) subunit alpha isoform X1 [B. mutus]
ENSP00000412545-D1	-2.69	4.30E-74	↑	Phosphoacetylglucosamine mutase, partial [B. mutus]
ENSBTAP00000006407-D1	-2.69	7.66E-132	↑	Cyclin-L2, partial [B. mutus]
ENSP00000301396-D1	-2.69	4.19E-30	↑	PELP1 [Homo sapiens]
ENSBTAP00000013285-D1	-2.68	2.01E-131	↑	gasdermin-A [B. mutus]
ENSP00000369263-D1	-2.67	5.83E-62	↑	Transforming acidic coiled-coil-containing protein 1 [B. mutus]
ENSP00000276282-D1	-2.67	4.54E-66	↑	Malignant fibrous histiocytoma-amplified sequence 1, partial [B. mutus]
ENSP00000338711-D1	-2.67	0.00E+00	↑	Tripartite motif-containing protein 34, partial [B. mutus]
ENSP00000339030-D1	-2.67	0.00E+00	↑	E3 ubiquitin-protein ligase ZFP91, partial [B. mutus]
ENSP00000391579-D1	-2.67	0.00E+00	↑	R3H domain-containing protein 1 isoform X3 [Bison bison bison]
ENSBTAP00000007903-D1	-2.67	2.08E-13	↑	GTP-binding protein 2, partial [B. mutus]
ENSBTAP00000023551-D1	-2.66	1.53E-31	↑	zinc transporter ZIP3 isoform X1 [B. mutus]
ENSP00000232564-D3	-2.66	5.10E-139	↑	guanine nucleotide-binding protein G(I)/G(S)/G(T) subunit beta-1 [Mus musculus]
ENSP00000265978-D1	-2.66	2.74E-44	↑	FTS and Hook-interacting protein [B. mutus]
ENSP00000248879-D1	-2.66	1.25E-34	↑	Protein DGCR6, partial [B. mutus]
ENSBTAP00000048755-D1	-2.66	1.25E-34	↑	hypothetical protein M91_13771, partial [B. mutus]
ENSBTAP00000003629-D4	-2.66	2.81E-29	↑	Sulfotransferase 1A1, partial [B. mutus]
ENSBTAP00000017727-D1	-2.66	1.94E-102	↑	Carboxypeptidase A1, partial [B. mutus]
ENSBTAP00000043784-D1	-2.66	2.51E-83	↑	alanine and arginine-rich domain-containing protein [B. taurus]
ENSP00000417470-D1	-2.66	1.21E-72	↑	Zinc finger protein 786, partial [B. mutus]
yakA23459	-2.65	4.05E-98	↑	SAM and SH3 domain-containing protein 1 [B. mutus]
ENSP00000352834-D1	-2.65	2.67E-37	↑	unconventional myosin-Ic [B. taurus]
ENSBTAP00000016053-D1	-2.65	3.13E-154	↑	Butyrophilin-like protein 1 [B. mutus]
ENSBTAP00000011888-D1	-2.64	5.67E-55	↑	NADPH oxidase 5, partial [B. mutus]
ENSP00000387641-D1	-2.64	3.03E-65	↑	Cullin-associated NEDD8-dissociated protein 2, partial [B. mutus]
ENSP00000386649-D1	-2.64	4.02E-38	↑	small membrane A-kinase anchor protein [B. taurus]
ENSP00000338266-D1	-2.64	3.64E-137	↑	Rap guanine nucleotide exchange factor 1, partial [B. mutus]
ENSBTAP00000010530-D1	-2.64	2.66E-171	↑	Toll-like receptor 2, partial [B. mutus]
ENSP00000332698-D1	-2.63	3.76E-54	↑	Heat shock factor protein 1 [B. mutus]
ENSBTAP00000041271-D1	-2.63	4.30E-16	↑	membrane-spanning 4-domains subfamily A member 13 [B. taurus]
ENSP00000411197-D1	-2.63	6.38E-11	↑	TBC1 domain family member 9, partial [B. mutus]
ENSP00000406852-D1	-2.63	6.38E-11	↑	Protein FAM193B, partial [B. mutus]
ENSBTAP00000053483-D1	-2.63	1.66E-03	↑	Rho guanine nucleotide exchange factor 17 [B. mutus]
ENSP00000346291-D1	-2.62	4.21E-23	↑	unconventional myosin-XVIIIa [B. mutus]
ENSP00000368918-D1	-2.62	2.12E-108	↑	M-phase inducer phosphatase 2 isoform X2 [B. mutus]
ENSP00000414662-D1	-2.62	3.69E-32	↑	2-oxoglutarate dehydrogenase, mitochondrial isoform X1 [B. mutus]
ENSBTAP00000001000-D1	-2.62	1.93E-57	↑	E3 SUMO-protein ligase PIAS4, partial [B. mutus]
ENSP00000264866-D1	-2.61	1.10E-96	↑	TBC1 domain family member 19 isoform X1 [Ovis aries]

ENSP00000342859-D1	-2.61	0.00E+00	↑	Nuclear factor 1 C-type, partial [B. mutus]
ENSP00000293805-D1	-2.61	4.83E-35	↑	B-cell CLL/lymphoma 6 member B protein [B. mutus]
ENSP00000295666-D1	-2.60	0.00E+00	↑	Insulin-like growth factor-binding protein 7, partial [B. mutus]
ENSP00000263228-D2	-2.60	1.20E-42	↑	ubiquitin-conjugating enzyme E2 R1 [B. taurus]
ENSP00000280357-D1	-2.60	0.00E+00	↑	interleukin-18 isoform X2 [B. mutus]
ENSP00000352780-D1	-2.60	2.28E-158	↑	Glutamate receptor-interacting protein 1, partial [B. mutus]
ENSP00000310561-D1	-2.60	3.48E-144	↑	rho GTPase-activating protein 32 [B. mutus]
ENSBTAP00000020263-D3	-2.60	2.31E-39	↑	Group XIIB secretory phospholipase A2-like protein, partial [B. mutus]
ENSP00000378364-D3	-2.59	1.17E-87	↑	Natural resistance-associated macrophage protein 2 [B. mutus]
ENSBTAP00000009273-D1	-2.59	1.54E-17	↑	zinc finger homeobox protein 2 [Camelus bactrianus]
ENSP00000392466-D1	-2.59	4.43E-51	↑	LIM domain-binding protein 1 isoform X1 [Cricetulus griseus]
ENSP00000385899-D1	-2.59	2.12E-26	↑	Protein sidekick-1, partial [B. mutus]
ENSP00000289269-D1	-2.59	1.86E-21	↑	Protocadherin alpha-C2 [B. mutus]
ENSBTAP00000007621-D1	-2.59	8.73E-166	↑	DDB1- and CUL4-associated factor 8 [B. taurus]
yakG038406	-2.59	2.43E-24	↑	hypothetical protein M91_13008 [B. mutus]
ENSBTAP00000020194-D1	-2.59	6.34E-90	↑	protease, serine, 23 [B. mutus]
ENSP00000312054-D1	-2.58	4.42E-29	↑	hypothetical protein M91_08634, partial [B. mutus]
ENSP00000267023-D1	-2.58	5.42E-70	↑	SOSS complex subunit B1 [Vicugna pacos]
ENSBTAP00000024807-D1	-2.58	4.64E-65	↑	E3 ubiquitin-protein ligase PDZRN3, partial [B. mutus]
ENSP00000399646-D1	-2.58	7.59E-35	↑	PRKCA-binding protein isoform X1 [B. mutus]
ENSBTAP00000017554-D1	-2.57	2.49E-198	↑	Disks large-like protein 5, partial [B. mutus]
ENSP00000302413-D1	-2.57	0.00E+00	↑	protein-tyrosine sulfotransferase 1 [B. taurus]
ENSBTAP00000009844-D1	-2.57	6.63E-23	↑	Short-chain specific acyl-CoA dehydrogenase, mitochondrial, partial [B. mutus]
ENSBTAP00000017198-D1	-2.57	3.07E-14	↑	TPA: ubiquitin specific protease 35-like [B. taurus]
ENSP00000393664-D6	-2.57	1.78E-05	↑	hypothetical protein M91_07668, partial [B. mutus]
ENSP00000309186-D3	-2.57	8.00E-93	↑	Putative E3 ubiquitin-protein ligase SH3RF1 [B. mutus]
ENSP00000261692-D1	-2.56	8.32E-129	↑	cyclin-dependent kinase 2-associated protein 1 [Sus scrofa]
ENSBTAP00000027991-D1	-2.56	1.23E-63	↑	far upstream element-binding protein 2 isoform X2, partial [B. taurus]
ENSBTAP00000002602-D1	-2.56	2.65E-10	↑	Kidney mitochondrial carrier protein 1, partial [B. mutus]
ENSP00000194900-D1	-2.56	3.16E-85	↑	Disks large-like protein 3 [B. mutus]
ENSP00000369071-D1	-2.56	8.39E-55	↑	periostin isoform X1 [B. taurus]
ENSP00000360060-D1	-2.56	2.20E-07	↑	proto-oncogene FRAT1, partial [B. mutus]
ENSP00000295640-D1	-2.55	3.37E-113	↑	aminopeptidase B [B. taurus]
ENSP00000300850-D1	-2.55	4.04E-30	↑	zinc finger protein 646 [B. mutus]
ENSBTAP00000005656-D1	-2.55	0.00E+00	↑	Vigilin [B. mutus]
ENSBTAP00000013876-D1	-2.55	1.55E-132	↑	SERTA domain-containing protein 3 isoform X2 [Condylura cristata]
ENSP00000377272-D2	-2.55	3.57E-90	↑	protein FAM19A2-like [Tupaia chinensis]
ENSBTAP00000029886-D1	-2.55	2.20E-148	↑	follistatin-related protein 1 isoform X2 [Ovis aries musimon]
ENSBTAP00000003543-D1	-2.55	1.52E-52	↑	homeobox protein DBX1 [B. mutus]
ENSBTAP00000046883-D1	-2.55	0.00E+00	↑	Tryptophanyl-tRNA synthetase, cytoplasmic [B. mutus]
ENSP00000218348-D1	-2.55	1.80E-93	↑	ubiquitin specific peptidase 11 [B. mutus]
ENSBTAP00000020239-D1	-2.55	5.30E-13	↑	interferon alpha/beta receptor 2 [B. mutus]
ENSBTAP00000032197-D1	-2.55	0.00E+00	↑	DNA-directed RNA polymerase I subunit RPA34 [B. mutus]
ENSP00000400939-D1	-2.54	4.57E-93	↑	treacle protein isoform X2 [Ovis aries musimon]
ENSBTAP00000051129-D1	-2.54	1.19E-70	↑	Nuclear receptor subfamily 6 group A member 1, partial [B. mutus]
ENSBTAP00000035760-D1	-2.54	1.53E-124	↑	uncharacterized protein C6orf136 homolog [B. mutus]
ENSBTAP00000006917-D1	-2.54	6.77E-65	↑	Peroxisomal membrane protein PEX13, partial [B. mutus]
ENSBTAP00000040563-D1	-2.54	5.28E-76	↑	Solute carrier family 2, facilitated glucose transporter member 1, partial [B. mutus]
ENSP00000410257-D1	-2.54	1.40E-118	↑	Sodium channel protein type 5 subunit alpha [B. mutus]
ENSBTAP00000027893-D1	-2.54	3.00E-34	↑	Annexin A11 [B. mutus]
ENSP00000272433-D1	-2.54	2.88E-21	↑	Sideroflexin-5, partial [B. mutus]
ENSP00000417207-D1	-2.54	2.48E-06	↑	Homeobox protein Hox-B3 [B. mutus]
ENSBTAP00000008640-D1	-2.54	2.13E-04	↑	sperm flagellar protein 1 [B. mutus]
ENSBTAP00000044829-D3	-2.54	2.13E-04	↑	TPA: acyl-protein thioesterase 1-like [B. taurus]
ENSP00000258081-D1	-2.53	6.32E-37	↑	ancient ubiquitous protein 1 [Bubalus bubalis]

ENSP00000394530-D1	-2.53	0.00E+00	↑	rap guanine nucleotide exchange factor-like 1 isoform X7 [Ovis aries musimon]
ENSP00000340010-D1	-2.53	3.09E-20	↑	Insulin-induced 1 protein, partial [B. mutus]
ENSBTAP00000029190-D1	-2.53	0.00E+00	↑	Nuclear pore complex protein Nup214, partial [B. mutus]
ENSBTAP00000004881-D1	-2.53	2.61E-29	↑	calcium-activated potassium channel subunit beta-4 [Pteropus alecto]
ENSP00000221482-D1	-2.52	3.93E-51	↑	Histone-lysine N-methyltransferase, H3 lysine-79 specific, partial [B. mutus]
ENSP00000408828-D1	-2.52	6.86E-22	↑	Leucine-rich repeat-containing protein 63, partial [B. mutus]
ENSBTAP00000036035-D1	-2.52	1.72E-15	↑	Ankyrin repeat and SAM domain-containing protein 1A, partial [B. mutus]
ENSP00000359243-D1	-2.52	4.63E-09	↑	Leucine zipper putative tumor suppressor 2 [B. mutus]
ENSBTAP00000009611-D2	-2.52	1.03E-64	↑	Ras-related protein Rab-3B, partial [B. mutus]
ENSP00000371587-D1	-2.52	6.50E-72	↑	Kv channel-interacting protein 4, partial [B. mutus]
ENSBTAP00000016607-D1	-2.52	2.96E-48	↑	dual specificity tyrosine-phosphorylation-regulated kinase 1B [Pantholops hodgsonii]
ENSBTAP00000022514-D1	-2.52	3.16E-47	↑	heterogeneous nuclear ribonucleoprotein A0 [B. taurus]
yakG004246	-2.52	7.54E-41	↑	RNA polymerase-associated protein LEO1 [B. mutus]
ENSBTAP00000051318-D1	-2.52	2.31E-73	↑	E3 ubiquitin-protein ligase NRDP1 [B. taurus]
ENSBTAP00000013251-D1	-2.51	0.00E+00	↑	DNA-directed RNA polymerase III subunit RPC4 [B. taurus]
ENSBTAP00000026674-D2	-2.51	2.62E-71	↑	uncharacterized protein C1orf43 homolog [B. mutus]
ENSBTAP00000014206-D1	-2.51	1.80E-41	↑	Protein KRI1-like protein, partial [B. mutus]
ENSP00000371345-D1	-2.51	4.73E-27	↑	hypothetical protein M91_14892, partial [B. mutus]
ENSP00000299413-D1	-2.51	1.63E-22	↑	tripartite motif-containing protein 44 [B. taurus]
ENSBTAP00000018809-D1	-2.51	0.00E+00	↑	homeobox protein PKNOX1 [B. mutus]
ENSP00000322915-D1	-2.51	4.12E-162	↑	Zinc finger MYM-type protein 4 [B. mutus]
ENSP00000263062-D1	-2.51	0.00E+00	↑	enhancer of polycomb homolog 1 isoform X1 [B. mutus]
ENSP00000361981-D1	-2.50	8.39E-19	↑	cAMP-dependent protein kinase inhibitor gamma isoform X1 [B. taurus]
ENSP00000216160-D1	-2.50	8.39E-19	↑	TGF-beta-activated kinase 1 and MAP3K7-binding protein 1 [B. mutus]
ENSBTAP00000035638-D1	-2.50	1.25E-74	↑	ras-related protein Rab-15 [B. mutus]
ENSBTAP00000039021-D1	-2.50	0.00E+00	↑	3-phosphoinositide-dependent protein kinase 1, partial [B. mutus]
ENSBTAP00000014308-D1	-2.50	1.46E-11	↑	carboxymethylenebutenolidase homolog [B. mutus]
ENSP00000357175-D1	-2.50	0.00E+00	↑	ETS translocation variant 3 [B. mutus]
ENSP00000362909-D1	-2.50	1.29E-101	↑	Protein NDRG3, partial [B. mutus]
ENSBTAP00000013354-D1	-2.50	9.99E-71	↑	apolipoprotein E [B. mutus]
ENSBTAP00000028405-D1	-2.49	4.66E-165	↑	Solute carrier family 25 member 40, partial [B. mutus]
ENSBTAP00000018617-D1	-2.49	5.98E-127	↑	UbiA prenyltransferase domain-containing protein 1, partial [B. mutus]
ENSP00000340330-D1	-2.49	1.05E-83	↑	histone acetyltransferase KAT5 isoform X1 [B. taurus]
ENSBTAP00000010703-D1	-2.49	5.38E-45	↑	interferon-induced helicase C domain-containing protein 1 [B. mutus]
ENSP00000397123-D1	-2.49	1.88E-67	↑	Bifunctional 3'-phosphoadenosine 5'-phosphosulfate synthase 2, partial [B. mutus]
ENSP00000386183-D1	-2.48	4.69E-14	↑	hypothetical protein PANDA_004034, partial [Ailuropoda melanoleuca]
ENSP00000380711-D1	-2.48	2.16E-147	↑	nuclear factor 1 B-type [Physeter catodon]
ENSP00000367359-D1	-2.48	1.41E-64	↑	uncharacterized protein C6orf62 homolog [B. taurus]
ENSBTAP00000037593-D1	-2.48	1.59E-10	↑	endothelin-converting enzyme 1 [B. mutus]
ENSP00000377204-D1	-2.48	4.67E-20	↑	G protein-coupled receptor kinase 6, partial [B. mutus]
ENSP00000216268-D1	-2.48	2.33E-11	↑	zinc finger BED domain-containing protein 4 [B. mutus]
ENSP00000267064-D1	-2.48	1.43E-291	↑	SWI/SNF complex subunit SMARCC2, partial [B. mutus]
ENSBTAP00000004834-D1	-2.48	6.95E-21	↑	protein FAM89B isoform X2 [Ovis aries musimon]
ENSBTAP00000005558-D3	-2.48	1.98E-86	↑	Acyl-protein thioesterase 1, partial [B. mutus]
ENSP00000402760-D1	-2.48	4.79E-26	↑	Solute carrier family 22 member 5 [B. mutus]
ENSBTAP00000032241-D1	-2.47	0.00E+00	↑	kalirin, partial [B. mutus]
ENSBTAP00000020974-D1	-2.47	2.37E-256	↑	Nestin [B. mutus]
ENSP00000363257-D1	-2.47	5.05E-13	↑	Palmitoyltransferase ZDHHC18, partial [B. mutus]
ENSBTAP00000037356-D1	-2.47	0.00E+00	↑	Serine/arginine repetitive matrix protein 2 [B. mutus]
ENSP00000410996-D1	-2.47	9.70E-103	↑	similar to Six transmembrane epithelial antigen of prostate [Homo sapiens]
ENSBTAP00000017386-D1	-2.47	3.54E-70	↑	Sorting nexin-19 [B. mutus]
ENSP00000354919-D1	-2.47	2.71E-40	↑	Ventricular zone-expressed PH domain-containing protein-like protein 1 [B. mutus]
ENSBTAP00000026715-D1	-2.46	7.07E-134	↑	Malectin [B. mutus]
ENSP00000390948-D1	-2.46	0.00E+00	↑	cytoplasmic FMR1-interacting protein 2 isoform X1 [B. mutus]
ENSP00000303252-D1	-2.46	3.18E-51	↑	Zinc finger protein 804A, partial [B. mutus]

ENSP00000356540-D1	-2.46	4.29E-40	↑	syntaxin-11 isoform X2 [B. taurus]
ENSBTAP00000013140-D1	-2.46	1.74E-93	↑	MORC family CW-type zinc finger protein 2 isoform X2 [Bison bison bison]
ENSBTAP00000013121-D1	-2.46	1.20E-25	↑	E3 ubiquitin/ISG15 ligase TRIM25 [B. taurus]
ENSP00000402009-D1	-2.46	0.00E+00	↑	Nuclear pore complex protein Nup153, partial [B. mutus]
ENSP00000399075-D1	-2.46	3.70E-11	↑	hypothetical protein M91_08470, partial [B. mutus]
ENSBTAP00000002174-D4	-2.46	8.80E-30	↑	EH domain-containing protein 1, partial [B. mutus]
ENSBTAP00000005899-D1	-2.46	9.91E-68	↑	estrogen receptor beta [B. mutus]
ENSBTAP00000024278-D1	-2.46	1.03E-256	↑	dnaJ homolog subfamily C member 27 isoform X1 [B. mutus]
ENSP00000403954-D2	-2.45	3.13E-64	↑	Teneurin-4 [B. mutus]
ENSBTAP00000001376-D1	-2.45	1.17E-157	↑	splicing factor 1 isoform X1 [Bison bison bison]
ENSP00000418232-D1	-2.45	7.61E-202	↑	Nischarin, partial [B. mutus]
ENSBTAP00000026147-D2	-2.45	6.87E-33	↑	prostate tumor-overexpressed gene 1 protein isoform X2, partial [Ovis aries musimon]
ENSP00000384899-D1	-2.45	0.00E+00	↑	Transcriptional repressor p66-alpha, partial [B. mutus]
ENSBTAP00000020711-D1	-2.44	1.58E-53	↑	serine/threonine-protein kinase ICK isoform X2 [Bison bison bison]
ENSBTAP00000053560-D1	-2.44	0.00E+00	↑	Nuclear factor, partial [B. mutus]
ENSBTAP00000004318-D1	-2.44	7.41E-51	↑	Follistatin, partial [B. mutus]
ENSBTAP00000052348-D1	-2.44	1.17E-109	↑	translation initiation factor eIF-2B subunit beta [B. taurus]
ENSBTAP00000042791-D1	-2.44	1.71E-123	↑	trichoplein keratin filament-binding protein [B. taurus]
ENSP00000363288-D1	-2.44	2.55E-33	↑	delta-aminolevulinic acid dehydratase isoform X1 [Pongo abelii]
ENSBTAP00000002231-D1	-2.44	1.27E-12	↑	transmembrane protein 107 [B. taurus]
ENSBTAP00000012406-D1	-2.44	4.01E-10	↑	gigaxonin [B. taurus]
ENSP00000245304-D1	-2.44	4.63E-05	↑	ras-related protein Rap-2a [Equus caballus]
ENSP00000403323-D1	-2.44	4.63E-05	↑	rho GTPase-activating protein 27 isoform X3 [Ovis aries musimon]
ENSP00000372023-D1	-2.44	2.11E-42	↑	serine/threonine-protein kinase Chk2 [B. taurus]
ENSP00000297163-D1	-2.44	9.68E-22	↑	SMAD5 antisense gene protein 1-like [Mustela putorius furo]
ENSBTAP00000005857-D1	-2.44	1.96E-140	↑	ER lumen protein retaining receptor 1 [B. mutus]
ENSBTAP00000052213-D1	-2.44	8.81E-139	↑	Protein transport protein Sec16B [B. mutus]
ENSP00000290650-D1	-2.44	1.85E-102	↑	E3 ubiquitin-protein ligase UBR1, partial [B. mutus]
ENSP00000388725-D1	-2.43	0.00E+00	↑	ankycorbin isoform X1 [B. mutus]
ENSP00000398704-D1	-2.43	1.52E-72	↑	Short stature homeobox protein 2, partial [B. mutus]
ENSBTAP00000012504-D1	-2.43	1.17E-209	↑	leucine zipper protein 1 [B. mutus]
ENSP00000337313-D1	-2.43	1.05E-266	↑	Zinc finger CCHC domain-containing protein 8 [B. mutus]
ENSP00000239597-D1	-2.43	4.66E-19	↑	CMP-N-acetylneuraminate-beta-galactosamide-alpha-2, 3-sialyltransferase 4, partial [B. mutus]
ENSBTAP00000028744-D1	-2.43	0.00E+00	↑	DDB1- and CUL4-associated factor 12, partial [B. mutus]
ENSP00000359991-D1	-2.42	3.02E-169	↑	phosphoglycerate mutase 1 [B. taurus]
ENSP00000360108-D1	-2.42	8.46E-67	↑	Putative tyrosine-protein phosphatase auxilin, partial [B. mutus]
ENSBTAP00000004229-D1	-2.42	5.45E-296	↑	oxysterols receptor LXR-beta [B. taurus]
ENSP00000298923-D1	-2.42	2.01E-130	↑	Sodium- and chloride-dependent glycine transporter 2, partial [B. mutus]
ENSBTAP00000021939-D1	-2.42	1.18E-36	↑	Transmembrane channel-like protein 7, partial [B. mutus]
ENSP00000381102-D1	-2.41	7.18E-31	↑	type 1 phosphatidylinositol 4,5-bisphosphate 4-phosphatase isoform X1 [Vicugna pacos]
ENSP00000267569-D1	-2.41	4.82E-30	↑	Jun dimerization protein 2, partial [B. mutus]
ENSP00000369198-D1	-2.41	1.13E-55	↑	G-protein coupled receptor 64 [B. mutus]
ENSP00000355428-D1	-2.41	2.65E-87	↑	SH3 domain-binding protein 5-like [B. mutus]
ENSP00000416951-D1	-2.41	4.62E-92	↑	zinc finger CCCH domain-containing protein 18 isoform X2 [B. taurus]
yakA13557	-2.41	2.99E-08	↑	Protein FAM170A [B. mutus]
ENSBTAP00000018877-D1	-2.41	9.06E-46	↑	protein S100-A16 [B. taurus]
ENSP00000395535-D1	-2.41	2.96E-24	↑	methyl-CpG-binding protein 2 [B. mutus]
ENSBTAP00000023618-D1	-2.41	0.00E+00	↑	nuclear factor interleukin-3-regulated protein [B. mutus]
ENSBTAP00000029264-D1	-2.40	0.00E+00	↑	neurofilament light polypeptide isoform X2 [B. mutus]
ENSP00000367024-D1	-2.40	2.07E-07	↑	N-alpha-acetyltransferase 40, NatD catalytic subunit, partial [B. mutus]
ENSP00000265717-D1	-2.40	1.27E-40	↑	cAMP-dependent protein kinase type II-beta regulatory subunit [B. mutus]
ENSBTAP00000024485-D1	-2.40	1.50E-44	↑	TSC22 domain family protein 2, partial [B. mutus]
ENSBTAP00000036650-D1	-2.40	2.36E-201	↑	cathepsin B isoform X1 [B. mutus]
ENSP00000354703-D1	-2.40	1.93E-59	↑	zinc finger protein 358 [B. mutus]
ENSP00000356331-D1	-2.39	2.36E-44	↑	Nuclear receptor subfamily 5 group A member 2 [B. mutus]

ENSBTAP00000010165-D1	-2.39	0.00E+00	↑	zinc finger X-chromosomal protein isoform X1 [Ovis aries]
ENSBTAP00000002495-D1	-2.39	1.89E-254	↑	Zinc finger protein 652, partial [B. mutus]
ENSP00000343445-D1	-2.39	2.96E-29	↑	Serpin B4, partial [B. mutus]
ENSP00000233813-D1	-2.39	1.11E-41	↑	Insulin-like growth factor-binding protein 5 [B. mutus]
ENSBTAP00000017878-D1	-2.39	1.46E-139	↑	hypothetical protein M91_14072, partial [B. mutus]
ENSP00000394117-D1	-2.39	0.00E+00	↑	F-box only protein 34 [B. mutus]
ENSP00000381430-D1	-2.39	1.03E-05	↑	MAGUK p55 subfamily member 3, partial [B. mutus]
ENSP00000262518-D1	-2.38	1.47E-79	↑	hypothetical protein PANDA_014226, partial [Ailuropoda melanoleuca]
ENSBTAP00000006992-D1	-2.38	0.00E+00	↑	rab GDP dissociation inhibitor beta isoform X1 [B. mutus]
ENSBTAP00000018177-D1	-2.38	7.21E-136	↑	Oxidative stress-induced growth inhibitor 2, partial [B. mutus]
ENSBTAP00000050620-D1	-2.38	4.36E-59	↑	Guanine nucleotide-binding protein subunit alpha-11, partial [B. mutus]
ENSBTAP00000015787-D1	-2.38	1.47E-160	↑	ubiquitin carboxyl-terminal hydrolase 4 isoform X1 [B. mutus]
ENSBTAP00000002523-D1	-2.37	0.00E+00	↑	Selenocysteine insertion sequence-binding protein 2 [B. mutus]
ENSP00000351352-D1	-2.37	9.00E-160	↑	signal-induced proliferation-associated 1-like protein 1 isoform X1 [B. mutus]
ENSP00000241416-D2	-2.37	1.22E-22	↑	Activin receptor type-2B [Tupaia chinensis]
ENSBTAP00000005930-D1	-2.37	0.00E+00	↑	RAF proto-oncogene serine/threonine-protein kinase [B. taurus]
ENSP00000372335-D1	-2.37	1.72E-29	↑	negative elongation factor A [B. taurus]
ENSP00000338807-D1	-2.37	1.15E-28	↑	zinc finger and BTB domain-containing protein 49 [B. mutus]
ENSBTAP00000038136-D1	-2.37	9.38E-118	↑	Histone deacetylase 7, partial [B. mutus]
ENSBTAP00000053528-D1	-2.37	3.40E-84	↑	Myotubularin-related protein 12, partial [B. mutus]
ENSBTAP00000017306-D1	-2.37	4.02E-14	↑	AMP deaminase 2 isoform X4 [B. mutus]
ENSBTAP00000014537-D3	-2.37	7.45E-05	↑	Chloride intracellular channel protein 6, partial [B. mutus]
ENSP00000379566-D1	-2.37	7.45E-05	↑	Coiled-coil alpha-helical rod protein 1, partial [B. mutus]
ENSP00000274897-D1	-2.37	4.27E-52	↑	hypothetical protein M91_00211, partial [B. mutus]
ENSBTAP00000025896-D1	-2.36	3.31E-32	↑	CTD nuclear envelope phosphatase 1 [B. taurus]
ENSBTAP00000012393-D1	-2.36	0.00E+00	↑	Putative protein KIAA1704, partial [B. mutus]
ENSBTAP00000053355-D1	-2.36	5.18E-217	↑	Myosin-Ie, partial [B. mutus]
ENSBTAP00000026057-D1	-2.36	1.84E-12	↑	Zinc finger and SCAN domain-containing protein 20, partial [B. mutus]
ENSP00000387836-D83	-2.35	7.98E-83	↑	Zinc finger protein 248, partial [B. mutus]
ENSBTAP00000049224-D1	-2.35	2.32E-06	↑	protein FAM136A-like [Bubalus bubalis]
ENSP00000320176-D1	-2.35	2.32E-06	↑	hematopoietic lineage cell-specific protein [B. mutus]
ENSBTAP00000000541-D2	-2.35	9.15E-69	↑	RNA-binding protein 4B isoform X1 [Bubalus bubalis]
ENSBTAP00000047522-D1	-2.35	7.50E-08	↑	Elastin [B. mutus]
ENSBTAP00000051161-D1	-2.35	2.24E-52	↑	zinc finger protein 346 [B. taurus]
ENSP00000261007-D1	-2.35	5.77E-25	↑	acetylcholine receptor subunit alpha [Bison bison bison]
ENSBTAP00000019581-D1	-2.35	1.21E-48	↑	Myelin regulatory factor, partial [B. mutus]
ENSP00000393379-D1	-2.34	1.14E-303	↑	kinesin heavy chain isoform 5C isoform X1 [B. taurus]
ENSBTAP00000027092-D1	-2.34	2.31E-46	↑	Protein FAM161B [B. mutus]
ENSP00000354227-D1	-2.34	3.77E-182	↑	hypothetical protein M91_14016, partial [B. mutus]
ENSBTAP00000043674-D2	-2.34	2.28E-57	↑	glutathione S-transferase omega-1-like isoform X1 [B. mutus]
ENSP00000386420-D1	-2.34	9.04E-123	↑	Calmodulin-regulated spectrin-associated protein 1, partial [B. mutus]
ENSBTAP00000017535-D1	-2.34	0.00E+00	↑	uncharacterized protein KIAA0355 homolog [B. mutus]
ENSP00000360141-D1	-2.34	0.00E+00	↑	Guanine nucleotide-binding protein G(s) subunit alpha isoforms XLas [B. mutus]
ENSBTAP00000018239-D1	-2.34	1.59E-291	↑	Cysteine protease ATG4A, partial [B. mutus]
ENSBTAP00000034759-D1	-2.34	0.00E+00	↑	Integrator complex subunit 10, partial [B. mutus]
ENSBTAP00000007160-D1	-2.33	4.71E-37	↑	WD repeat-containing protein 78 [B. taurus]
ENSBTAP00000050902-D1	-2.33	4.75E-69	↑	Zinc finger protein 699, partial [B. mutus]
ENSBTAP00000031483-D1	-2.33	4.84E-117	↑	Tribbles-like protein 1 [B. mutus]
ENSBTAP00000014539-D1	-2.33	1.70E-118	↑	hypothetical protein M91_02361, partial [B. mutus]
ENSBTAP00000053437-D1	-2.33	0.00E+00	↑	Neuron navigator 3, partial [B. mutus]
ENSP00000371201-D1	-2.33	1.71E-91	↑	putative methyltransferase NSUN7 [B. mutus]
ENSBTAP00000011972-D1	-2.33	3.33E-20	↑	integrin beta-6 isoform X1 [B. mutus]
ENSP00000402861-D1	-2.33	5.69E-04	↑	Inactive phospholipase C-like protein 1, partial [B. mutus]
ENSP00000363590-D1	-2.33	0.00E+00	↑	chondroitin sulfate N-acetylgalactosaminyltransferase 2 [B. mutus]
ENSP00000395538-D1	-2.32	5.37E-15	↑	trinucleotide repeat-containing gene 18 protein [B. mutus]

ENSBTAP00000027271-D1	-2.32	2.05E-135	↑	fermitin family homolog 1 [B. taurus]
ENSP00000236040-D1	-2.32	6.33E-23	↑	prolyl 3-hydroxylase 1 [B. mutus]
ENSP00000340465-D1	-2.32	1.22E-20	↑	Sodium-dependent phosphate transporter 2 [B. mutus]
ENSBTAP00000027619-D1	-2.32	1.24E-45	↑	Rho guanine nucleotide exchange factor 7, partial [B. mutus]
ENSP00000316674-D10	-2.31	3.10E-11	↑	Actin-like protein 8, partial [B. mutus]
ENSP00000264128-D2	-2.31	2.93E-283	↑	calcium-binding mitochondrial carrier protein SCaMC-1-like [B. mutus]
ENSBTAP00000038304-D1	-2.31	9.47E-74	↑	serine/threonine-protein phosphatase 2A 56 kDa regulatory subunit alpha isoform [Bubalus bubalis]
ENSBTAP00000027659-D1	-2.31	3.11E-55	↑	Zinc finger protein 526 [B. mutus]
ENSP00000258526-D1	-2.31	3.36E-300	↑	Plexin-C1, partial [B. mutus]
ENSP00000417490-D1	-2.31	6.77E-17	↑	BTB/POZ domain-containing protein KCTD6 [Myotis brandtii]
ENSP00000268719-D1	-2.31	6.77E-17	↑	glucose-induced degradation protein 4 homolog [B. taurus]
ENSP00000006275-D1	-2.31	2.44E-13	↑	Trafficking protein particle complex subunit 6A [B. mutus]
ENSBTAP00000001940-D1	-2.31	3.67E-06	↑	Protein turtle-like protein A, partial [B. mutus]
ENSBTAP00000034638-D1	-2.31	0.00E+00	↑	MLL1/MLL complex subunit KIAA1267 [B. mutus]
ENSBTAP00000016245-D1	-2.31	1.69E-147	↑	biogenesis of lysosome-related organelles complex 1 subunit 6 [B. taurus]
ENSP00000369131-D1	-2.30	5.68E-14	↑	paired amphipathic helix protein Sin3b isoform X1 [B. taurus]
ENSBTAP00000046530-D1	-2.30	2.70E-08	↑	Sharpin, partial [B. mutus]
ENSBTAP00000015922-D1	-2.30	2.70E-08	↑	Breast cancer anti-estrogen resistance protein 1, partial [B. mutus]
ENSP00000384832-D1	-2.30	0.00E+00	↑	Protein FAM104A [B. mutus]
ENSP00000350314-D1	-2.30	2.54E-41	↑	Beta,beta-carotene 9',10'-oxygenase, partial [B. mutus]
ENSBTAP00000018227-D1	-2.30	7.89E-54	↑	protein FAM5B [B. mutus]
ENSBTAP00000007064-D1	-2.30	2.98E-64	↑	Transmembrane and TPR repeat-containing protein 1, partial [B. mutus]
ENSBTAP00000029559-D1	-2.29	3.82E-13	↑	acetyl-CoA carboxylase 2-like [B. mutus]
ENSBTAP00000049180-D1	-2.29	0.00E+00	↑	brain abundant, membrane attached signal protein 1 [Pantholops hodgsonii]
ENSP00000309230-D1	-2.29	3.08E-122	↑	Tyrosine-protein kinase SgK269 [B. mutus]
ENSBTAP00000002220-D1	-2.29	1.85E-78	↑	RNA polymerase II-associated protein 1 [B. mutus]
ENSBTAP00000020710-D1	-2.29	0.00E+00	↑	G-protein coupled receptor 84 [B. mutus]
ENSBTAP00000042008-D1	-2.29	1.67E-59	↑	prenylcysteine oxidase-like [B. mutus]
ENSP00000417980-D1	-2.29	4.76E-29	↑	Histone-lysine N-methyltransferase, H3 lysine-9 specific 5, partial [B. mutus]
ENSP00000361433-D1	-2.29	0.00E+00	↑	exosome complex component RRP4 [Ovis aries]
ENSP00000393952-D1	-2.29	1.34E-18	↑	tetratricopeptide repeat protein 39A [B. mutus]
ENSP00000368651-D1	-2.29	4.06E-35	↑	Folliculin-interacting protein 2 [B. mutus]
ENSBTAP00000014053-D1	-2.29	4.75E-130	↑	dynactin subunit 2 isoform X3 [B. mutus]
ENSBTAP00000020586-D1	-2.28	3.11E-19	↑	lysine-specific demethylase 4B isoform X1 [B. taurus]
ENSBTAP00000033249-D1	-2.28	2.38E-45	↑	Sterol regulatory element-binding protein cleavage-activating protein, partial [B. mutus]
ENSBTAP00000050194-D94	-2.28	1.86E-07	↑	hypothetical protein M91_08672 [B. mutus]
ENSP00000281243-D1	-2.28	5.21E-61	↑	Dihydropteridine reductase, partial [B. mutus]
ENSP00000385169-D1	-2.28	0.00E+00	↑	R3H domain-containing protein 2 [B. mutus]
ENSP00000318944-D1	-2.28	4.49E-162	↑	E3 ubiquitin-protein ligase TRAF7, partial [B. mutus]
ENSBTAP00000026838-D3	-2.28	6.21E-78	↑	Ribonuclease H2 subunit B [B. mutus]
ENSBTAP00000053835-D1	-2.28	1.11E-15	↑	Ubiquinone biosynthesis protein COQ4-like protein, mitochondrial, partial [B. mutus]
ENSP00000411825-D1	-2.28	2.24E-139	↑	programmed cell death 6-interacting protein-like isoform X1 [B. mutus]
ENSP00000355024-D1	-2.27	1.28E-102	↑	Myosin light chain kinase, smooth muscle [B. mutus]
yakG017578	-2.27	7.57E-11	↑	hypothetical protein M91_01220 [B. mutus]
ENSP00000266880-D1	-2.27	6.56E-34	↑	E3 ubiquitin-protein ligase CHFR isoform X1 [B. mutus]
ENSBTAP00000003078-D1	-2.27	4.03E-48	↑	Putative palmitoyltransferase ZDHHC5 [B. mutus]
yakG017947	-2.27	2.64E-20	↑	hypothetical protein M91_20063 [B. mutus]
ENSP00000277575-D1	-2.27	0.00E+00	↑	hypothetical protein M91_00197 [B. mutus]
ENSBTAP00000009850-D1	-2.27	0.00E+00	↑	nuclear receptor coactivator 3 [B. mutus]
ENSBTAP00000007065-D1	-2.27	9.17E-63	↑	UDP-N-acetylglucosamine--dolichyl-phosphate N-acetylglucosaminephosphotransferase [B. mutus]
ENSP00000261530-D1	-2.27	8.90E-132	↑	G patch domain-containing protein 2-like isoform X1 [B. mutus]
ENSBTAP00000044701-D1	-2.26	4.03E-16	↑	WD repeat-containing protein 49-like isoform X1 [B. taurus]
ENSBTAP00000022232-D1	-2.26	2.22E-09	↑	Putative aminopeptidase NPEPL1, partial [B. mutus]
ENSP00000206542-D1	-2.26	7.13E-37	↑	probable tRNA N6-adenosine threonylcarbamoyltransferase [B. taurus]
ENSP00000314407-D1	-2.26	6.83E-51	↑	carbonic anhydrase-related protein [B. taurus]

ENSP00000302441-D1	-2.26	3.08E-104	↑	N-acylneuraminate-9-phosphatase [B. taurus]
ENSBTAP00000013635-D1	-2.26	9.10E-39	↑	GTP-binding protein 1, partial [B. mutus]
ENSP00000366036-D1	-2.26	2.54E-137	↑	TBC1 domain family member 8 isoform X2 [Bison bison bison]
ENSBTAP00000004744-D1	-2.26	7.20E-206	↑	cyclin-J isoform X1 [B. mutus]
ENSP00000303740-D1	-2.25	1.29E-06	↑	beta-1,3-galactosyltransferase 1 [B. taurus]
ENSBTAP00000012812-D1	-2.25	0.00E+00	↑	replication protein A 70 kDa DNA-binding subunit [B. mutus]
ENSP00000359489-D1	-2.25	7.07E-32	↑	AF4/FMR2 family member 2, partial [B. mutus]
ENSP00000361418-D1	-2.25	1.65E-32	↑	importin-13 [B. mutus]
ENSP00000385888-D1	-2.25	1.30E-285	↑	Histone acetyltransferase MYST3 [B. mutus]
ENSBTAP00000006449-D1	-2.25	2.11E-65	↑	Actin-binding LIM protein 3, partial [B. mutus]
ENSP00000375820-D91	-2.25	1.90E-101	↑	Zinc finger protein 214, partial [B. mutus]
ENSBTAP00000030687-D1	-2.25	0.00E+00	↑	PX domain-containing protein kinase-like protein [B. taurus]
ENSBTAP00000007904-D1	-2.25	3.11E-61	↑	Nuclear factor NF-kappa-B p100 subunit [B. mutus]
ENSP00000332111-D1	-2.25	4.86E-183	↑	hypothetical protein M91_00474, partial [B. mutus]
ENSP00000269197-D1	-2.24	3.55E-68	↑	Putative Polycomb group protein ASXL3, partial [B. mutus]
ENSBTAP00000022801-D1	-2.24	1.78E-35	↑	E3 ubiquitin-protein ligase TRIM32 [B. mutus]
ENSP00000404121-D1	-2.24	3.84E-239	↑	Interleukin enhancer-binding factor 3, partial [B. mutus]
ENSBTAP00000002806-D1	-2.24	9.15E-46	↑	Podocin, partial [B. mutus]
ENSBTAP00000002512-D1	-2.24	3.48E-09	↑	TraB domain-containing protein, partial [B. mutus]
ENSBTAP00000019309-D1	-2.24	1.04E-52	↑	Mps one binder kinase activator-like 2A, partial [B. mutus]
ENSP00000237642-D1	-2.24	3.71E-92	↑	starch-binding domain-containing protein 1 [B. taurus]
ENSBTAP00000048631-D1	-2.24	0.00E+00	↑	protein Tob1 [B. taurus]
ENSBTAP00000001246-D3	-2.23	7.04E-128	↑	pleckstrin homology domain-containing family B member 2 [B. mutus]
ENSP00000243918-D1	-2.23	9.78E-12	↑	protein SYS1 homolog [B. taurus]
ENSBTAP00000012801-D1	-2.23	0.00E+00	↑	Cat eye syndrome critical region protein 2, partial [B. mutus]
ENSBTAP00000009173-D1	-2.23	9.55E-70	↑	Peroxisomal multifunctional enzyme type 2, partial [B. mutus]
ENSBTAP00000039205-D2	-2.23	4.57E-51	↑	sorting nexin 5 [B. mutus]
ENSP00000305632-D1	-2.23	3.23E-92	↑	Homer protein-like protein 2, partial [B. mutus]
ENSP00000412831-D1	-2.23	1.80E-118	↑	Cold-inducible RNA-binding protein, partial [Cathartes aura]
ENSBTAP00000010355-D1	-2.23	3.61E-36	↑	sorting nexin-12 isoform X1 [B. mutus]
ENSP00000263238-D1	-2.23	1.29E-49	↑	Actin-related protein 3B, partial [B. mutus]
ENSP00000334225-D1	-2.23	6.50E-15	↑	UPF0258 protein KIAA1024-like protein, partial [B. mutus]
ENSP00000381049-D1	-2.23	5.69E-61	↑	neuron-specific protein family member 1 [B. taurus]
ENSBTAP00000021768-D1	-2.23	1.98E-95	↑	TPA: ATP-binding cassette sub-family G member 5 [B. taurus]
ENSP00000376765-D1	-2.22	1.81E-29	↑	E3 SUMO-protein ligase PIAS3, partial [B. mutus]
ENSBTAP00000027359-D1	-2.22	6.93E-46	↑	apoptosis-associated speck-like protein containing a CARD [B. taurus]
ENSBTAP00000008733-D1	-2.22	1.29E-56	↑	RCC1 and BTB domain-containing protein 2, partial [B. mutus]
ENSP00000409466-D1	-2.22	3.18E-48	↑	Retrograde Golgi transport protein RGP1-like protein, partial [B. mutus]
ENSP00000324804-D2	-2.22	1.36E-47	↑	serine/threonine-protein phosphatase 2A 65 kDa regulatory subunit A alpha isoform-like isoform X1 [P. hodgsonii]
ENSBTAP00000023319-D1	-2.22	2.54E-22	↑	peroxisome proliferator-activated receptor delta [B. taurus]
ENSP00000300209-D1	-2.22	3.71E-19	↑	protein-lysine methyltransferase METTL21B [B. taurus]
ENSP00000400588-D1	-2.22	2.37E-08	↑	kynurenine/alpha-aminoadipate aminotransferase, mitochondrial-like [B. mutus]
ENSP00000407619-D3	-2.22	4.85E-03	↑	prohibitin isoform X1 [Tupaia chinensis]
ENSBTAP00000002616-D1	-2.21	7.28E-304	↑	eukaryotic translation initiation factor 5A-1 [Myotis brandtii]
ENSP00000337512-D1	-2.21	1.30E-75	↑	ftsJ methyltransferase domain-containing protein 1 [B. mutus]
ENSP00000256646-D1	-2.21	9.03E-118	↑	neurogenic locus notch homolog protein 2 [B. mutus]
ENSBTAP00000015645-D1	-2.21	2.47E-53	↑	Secretogranin-1, partial [B. mutus]
ENSBTAP00000022780-D1	-2.21	0.00E+00	↑	Syntaxin-7, partial [B. mutus]
ENSP00000365534-D1	-2.20	0.00E+00	↑	Threonine synthase-like 1, partial [B. mutus]
ENSBTAP00000008329-D1	-2.20	1.14E-94	↑	thioredoxin-like protein 4B [B. taurus]
yakG040703	-2.20	1.25E-115	↑	Programmed cell death protein 6 [B. mutus]
ENSP00000070846-D1	-2.20	3.80E-79	↑	Plakophilin-2 [B. mutus]
ENSP00000184956-D1	-2.19	1.11E-31	↑	HEAT repeat-containing protein 6-like [B. mutus]
ENSBTAP00000012427-D1	-2.19	2.13E-148	↑	beta-1,3-galactosyl-O-glycosyl-glycoprotein beta-1,6-N-acetylglucosaminyltransferase 3 [B. taurus]
ENSP00000261643-D1	-2.19	8.34E-57	↑	Protoheme IX farnesyltransferase, mitochondrial, partial [B. mutus]

ENSBTAP00000001360-D1	-2.19	6.36E-82	↑	AT-rich interactive domain-containing protein 1A [B. mutus]
ENSBTAP00000019842-D1	-2.19	2.77E-35	↑	NAD-dependent protein deacylase sirtuin-5, mitochondrial isoform X1 [B. mutus]
ENSP00000379503-D1	-2.19	2.98E-152	↑	pyruvate dehyrogenase phosphatase catalytic subunit 1 isoform X1 [Bison bison bison]
ENSBTAP00000004141-D1	-2.19	7.24E-175	↑	arf-GAP with SH3 domain, ANK repeat and PH domain-containing protein 1 [B. taurus]
ENSP00000371634-D1	-2.19	4.01E-70	↑	insulin-like growth factor 2 mRNA-binding protein 2 [B. taurus]
ENSBTAP00000031937-D2	-2.19	5.62E-15	↑	Delta(3,5)-Delta(2,4)-dienoyl-CoA isomerase, mitochondrial, partial [B. mutus]
ENSP00000267012-D1	-2.19	7.72E-300	↑	Bridging integrator 2, partial [B. mutus]
ENSBTAP00000053354-D2	-2.19	3.68E-08	↑	protein kinase C-binding protein NELL1 [Bison bison bison]
ENSP00000405387-D1	-2.19	0.00E+00	↑	protein SMG7 isoform X2 [Bison bison bison]
ENSP00000345856-D1	-2.19	9.74E-308	↑	cyclin-dependent kinase 11B-like isoform X2 [Pantholops hodgsonii]
ENSP00000280756-D1	-2.18	7.66E-105	↑	transmembrane protein 263 [B. taurus]
ENSBTAP00000005927-D1	-2.18	4.90E-112	↑	Putative E3 ubiquitin-protein ligase makorin-2, partial [B. mutus]
ENSP00000383295-D1	-2.18	0.00E+00	↑	Lipopolysaccharide-responsive and beige-like anchor protein, partial [B. mutus]
ENSBTAP00000013884-D1	-2.18	6.23E-21	↑	ankyrin repeat and SOCS box protein 7 isoform 2 [Homo sapiens]
ENSBTAP00000008609-D1	-2.18	2.08E-29	↑	HDGF protein [B. taurus]
ENSBTAP00000025762-D1	-2.18	0.00E+00	↑	RFK protein [B. taurus]
ENSP00000345268-D2	-2.18	1.15E-19	↑	Palmitoyltransferase ZDHHC3 [B. mutus]
ENSBTAP00000028418-D1	-2.18	8.26E-44	↑	collagen alpha-3(IV) chain precursor [B. taurus]
ENSBTAP00000031029-D1	-2.18	1.35E-258	↑	Interferon-induced protein 44-like protein [B. mutus]
ENSP00000395735-D2	-2.18	2.56E-38	↑	60S acidic ribosomal protein P1-like [B. mutus]
ENSP00000299466-D1	-2.18	5.24E-22	↑	Sal-like protein 3, partial [B. mutus]
ENSBTAP00000010424-D1	-2.18	1.76E-276	↑	Nuclear receptor coactivator 6, partial [B. mutus]
ENSBTAP00000037377-D1	-2.18	2.16E-39	↑	DNA-3-methyladenine glycosylase, partial [B. mutus]
ENSP00000297293-D1	-2.17	9.62E-21	↑	Serine/threonine-protein kinase LMTK2, partial [B. mutus]
ENSBTAP00000020327-D1	-2.17	0.00E+00	↑	golgin subfamily A member 1 [B. mutus]
ENSP00000230859-D1	-2.17	4.13E-20	↑	non-canonical poly(A) RNA polymerase PAPD7 isoform X1 [B. taurus]
ENSP00000284245-D1	-2.17	3.51E-190	↑	hypothetical protein M91_08315, partial [B. mutus]
yakG009020	-2.17	0.00E+00	↑	hypothetical protein M91_08913 [B. mutus]
ENSBTAP00000004272-D1	-2.17	1.96E-43	↑	lactadherin [B. mutus]
ENSP00000368102-D1	-2.17	2.88E-33	↑	coiled-coil domain-containing protein 3 isoform X1 [Ovis aries]
ENSBTAP00000053832-D1	-2.17	2.47E-52	↑	sterile alpha motif domain-containing protein 9 [B. mutus]
ENSBTAP00000001564-D1	-2.17	3.00E-09	↑	Telomerase protein component 1 [B. mutus]
ENSP00000256682-D1	-2.17	6.29E-195	↑	ADP-ribosylation factor 1 isoformX1 [Equus caballus]
ENSP00000354826-D1	-2.17	0.00E+00	↑	non-muscle caldesmon [B. mutus]
ENSBTAP00000025044-D1	-2.16	6.05E-69	↑	Isocitrate dehydrogenase [NAD] subunit beta, mitochondrial, partial [B. mutus]
ENSP00000370723-D1	-2.16	0.00E+00	↑	hypothetical protein M91_21411, partial [B. mutus]
ENSBTAP00000003242-D1	-2.16	7.91E-195	↑	bromodomain-containing protein 4 [Bubalus bubalis]
yakG008085	-2.16	4.16E-85	↑	hypothetical protein M91_19846 [B. mutus]
ENSP00000371462-D1	-2.16	2.58E-43	↑	Microtubule-associated protein 1A [B. mutus]
ENSP00000346560-D1	-2.16	5.10E-200	↑	filamin A-interacting protein 1-like isoform X1 [B. taurus]
ENSBTAP00000020550-D1	-2.16	1.06E-32	↑	Basic fibroblast growth factor receptor 1, partial [B. mutus]
ENSBTAP00000031873-D1	-2.16	1.97E-203	↑	fas apoptotic inhibitory molecule 1 [B. taurus]
ENSBTAP00000005747-D1	-2.16	5.16E-69	↑	Protein ETHE1, mitochondrial, partial [B. mutus]
ENSBTAP00000021927-D1	-2.16	1.97E-48	↑	Tumor necrosis factor receptor superfamily member 19L, partial [B. mutus]
yakA05610	-2.15	1.07E-09	↑	adenylate kinase 2, mitochondrial-like [B. mutus]
ENSBTAP00000006012-D1	-2.15	2.52E-07	↑	Fibroblast growth factor 14, partial [B. mutus]
ENSBTAP00000015698-D1	-2.15	7.40E-35	↑	AT-rich interactive domain-containing protein 3B isoform X1 [Bison bison bison]
ENSP00000258455-D1	-2.15	2.46E-94	↑	28S ribosomal protein S9, mitochondrial [B. mutus]
ENSP00000259939-D1	-2.15	1.52E-29	↑	E3 ubiquitin-protein ligase RNF144B [B. mutus]
ENSP00000259874-D1	-2.15	2.02E-11	↑	radiation-inducible immediate-early gene IEX-1 [B. taurus]
ENSBTAP00000004087-D1	-2.14	1.12E-06	↑	TBCC domain-containing protein 1 [B. mutus]
ENSBTAP00000032779-D3	-2.14	5.87E-251	↑	14-3-3 protein theta, partial [B. mutus]
ENSP00000395259-D1	-2.14	4.26E-51	↑	SCAN domain-containing protein 3 [B. mutus]
ENSP00000399831-D1	-2.14	9.41E-26	↑	Nucleolar protein 3, partial [B. mutus]
ENSBTAP00000020381-D1	-2.14	3.26E-56	↑	zinc fingers and homeoboxes protein 1 [B. mutus]

ENSBTAP00000003257-D1	-2.14	1.84E-27	↑	guanine nucleotide-binding protein G(I)/G(S)/G(O) subunit gamma-3-like protein [Camelus ferus]
ENSBTAP00000016261-D1	-2.14	9.22E-115	↑	peptide chain release factor 1, mitochondrial [B. mutus]
ENSBTAP00000053340-D3	-2.14	1.24E-36	↑	Transcription factor COE2, partial [B. mutus]
ENSBTAP00000014200-D1	-2.14	2.33E-19	↑	cysteine protease ATG4D [Bubalus bubalis]
ENSBTAP00000011817-D2	-2.14	1.04E-37	↑	mitochondrial import receptor subunit TOM6 homolog [B. mutus]
ENSBTAP00000002392-D1	-2.14	1.49E-64	↑	Leucine-rich repeat-containing protein 46, partial [B. mutus]
ENSBTAP00000024537-D10	-2.14	2.03E-75	↑	hypothetical protein M91_21171 [B. mutus]
yakA28237	-2.14	0.00E+00	↑	Mediator of RNA polymerase II transcription subunit 1 [B. mutus]
ENSBTAP00000013370-D1	-2.13	4.36E-79	↑	Osteopetrosis-associated transmembrane protein 1, partial [B. mutus]
ENSBTAP00000009887-D1	-2.13	1.22E-42	↑	Serine/threonine-protein phosphatase PGAM5, mitochondrial, partial [B. mutus]
ENSP00000303525-D1	-2.13	9.97E-221	↑	carbonyl reductase family member 4 [B. taurus]
ENSP00000355884-D4	-2.13	1.06E-65	↑	Serine/threonine-protein kinase MARK2 [B. mutus]
ENSBTAP00000050811-D1	-2.13	5.37E-64	↑	CD166 antigen, partial [B. mutus]
ENSBTAP00000027362-D1	-2.13	6.34E-34	↑	Putative E3 ubiquitin-protein ligase HERC6, partial [B. mutus]
ENSP00000364536-D3	-2.13	2.78E-185	↑	Sodium channel protein type 8 subunit alpha, partial [B. mutus]
ENSP00000365191-D1	-2.13	9.51E-25	↑	Golgi membrane protein 1 [B. mutus]
ENSP00000386921-D1	-2.13	2.11E-189	↑	TBC1 domain family member 14 isoform X1 [B. mutus]
ENSBTAP00000014447-D1	-2.13	1.03E-77	↑	homeobox protein MSX-1 [B. mutus]
ENSBTAP00000013009-D1	-2.12	7.97E-26	↑	Cell death activator CIDE-A, partial [B. mutus]
ENSBTAP00000002387-D1	-2.12	1.03E-42	↑	hypothetical protein M91_18932, partial [B. mutus]
ENSBTAP00000006057-D1	-2.12	4.64E-29	↑	kinesin family member 19, partial [B. mutus]
ENSP00000355648-D1	-2.12	9.55E-74	↑	hypothetical protein M91_02777 [B. mutus]
ENSP00000356621-D1	-2.12	5.07E-275	↑	Ras GTPase-activating protein nGAP, partial [B. mutus]
ENSBTAP00000041324-D1	-2.12	8.99E-99	↑	MAPK-interacting and spindle-stabilizing protein-like protein, partial [B. mutus]
ENSP00000334008-D1	-2.12	3.43E-74	↑	alpha-parvin [Camelus dromedarius]
ENSP00000376315-D3	-2.12	0.00E+00	↑	cysteine-rich protein 1 [B. taurus]
ENSBTAP00000043277-D1	-2.12	5.31E-102	↑	serine/threonine-protein kinase haspin, partial [B. mutus]
ENSBTAP00000041782-D1	-2.12	5.23E-25	↑	aromatase cytochrome P450 [Bubalus bubalis]
ENSP00000346128-D1	-2.12	1.44E-45	↑	protein MEF2BNB isoform X1 [Balaenoptera acutorostrata scammoni]
ENSBTAP00000020126-D1	-2.11	0.00E+00	↑	Arginine and glutamate-rich protein 1 [B. mutus]
ENSP00000407656-D1	-2.11	1.71E-11	↑	E3 ubiquitin-protein ligase RNF5 [B. mutus]
ENSP00000378554-D1	-2.10	1.35E-35	↑	armadillo repeat-containing X-linked protein 3 [B. mutus]
ENSBTAP00000052501-D1	-2.10	2.65E-102	↑	Caprin-1, partial [B. mutus]
ENSBTAP00000027498-D1	-2.10	4.56E-60	↑	F-box only protein 7 [B. mutus]
ENSP00000228928-D2	-2.10	4.34E-157	↑	2'-5'-oligoadenylate synthase 3 [Ceratotherium simum simum]
ENSBTAP00000051035-D78	-2.10	9.00E-10	↑	olfactory receptor 51G2-like [Galeopterus variegatus]
ENSBTAP00000007696-D2	-2.10	4.63E-86	↑	COP9 signalosome complex subunit 8-like [B. mutus]
ENSP00000390232-D1	-2.10	1.67E-48	↑	zinc finger B-box domain-containing protein 1 [B. mutus]
ENSBTAP00000016762-D1	-2.10	7.37E-11	↑	Excitatory amino acid transporter 2, partial [B. mutus]
ENSBTAP00000053583-D1	-2.10	1.37E-93	↑	rho GTPase-activating protein 26 isoform X2 [B. mutus]
ENSBTAP00000008028-D1	-2.10	2.56E-28	↑	exocyst complex component 8 [B. taurus]
ENSBTAP00000041598-D1	-2.10	1.81E-161	↑	dual specificity mitogen-activated protein kinase kinase 4 [Ursus maritimus]
ENSBTAP00000041513-D1	-2.10	3.20E-21	↑	transcription cofactor vestigial-like protein 4 [B. taurus]
ENSBTAP00000051403-D14	-2.10	4.13E-14	↑	uncharacterized protein LOC100076456 [Ornithorhynchus anatinus]
ENSP00000287996-D1	-2.10	2.84E-16	↑	Inositol-pentakisphosphate 2-kinase [B. mutus]
ENSBTAP00000051991-D1	-2.09	4.37E-203	↑	consortin [B. mutus]
ENSBTAP00000048702-D1	-2.09	6.47E-51	↑	AP-1 complex subunit sigma-3, partial [B. mutus]
ENSP00000341737-D1	-2.09	1.87E-38	↑	adaptin ear-binding coat-associated protein 1 [B. taurus]
ENSP00000341380-D1	-2.09	4.90E-21	↑	hypothetical protein M91_15523, partial [B. mutus]
ENSBTAP00000019943-D1	-2.09	2.15E-12	↑	Ubiquitin thioesterase OTUB2, partial [B. mutus]
ENSP00000241014-D1	-2.09	1.02E-04	↑	c-Jun-amino-terminal kinase-interacting protein 1 [Bubalus bubalis]
yakA20341	-2.09	1.47E-03	↑	hypothetical protein M91_21715 [B. mutus]
ENSP00000402918-D1	-2.09	5.02E-204	↑	oxidation resistance protein 1 isoform X3 [B. mutus]
ENSP00000293218-D1	-2.09	4.99E-29	↑	RING finger protein unkempt-like protein, partial [B. mutus]
ENSBTAP00000029020-D1	-2.09	2.24E-118	↑	pituitary tumor-transforming gene 1 protein-interacting protein [B. mutus]

ENSP00000365714-D1	-2.09	5.78E-59	↑	Protein tweety-like protein 1, partial [B. mutus]
ENSP00000228284-D1	-2.08	4.35E-16	↑	Squamous cell carcinoma antigen recognized by T-cells 3 [B. mutus]
ENSP00000406955-D1	-2.08	0.00E+00	↑	zinc finger and BTB domain-containing protein 38 isoform X1 [B. mutus]
ENSBTAP00000000071-D1	-2.08	6.32E-14	↑	flap endonuclease 1 [Ovis aries]
ENSP00000381821-D1	-2.08	1.05E-57	↑	Protein yippee-like 3, partial [B. mutus]
ENSP00000395910-D1	-2.08	6.67E-38	↑	protein tyrosine phosphatase domain-containing protein 1 isoform X1 [B. mutus]
ENSBTAP00000015344-D1	-2.08	1.00E-43	↑	short transient receptor potential channel 2-like [B. mutus]
ENSBTAP00000004535-D1	-2.08	1.13E-10	↑	Interactor protein for cytohesin exchange factors 1, partial [B. mutus]
ENSBTAP00000038493-D1	-2.08	1.57E-24	↑	homeobox protein SIX3 [Mesocricetus auratus]
ENSP00000378792-D59	-2.08	9.78E-30	↑	Zinc finger protein 599, partial [B. mutus]
ENSBTAP00000014030-D1	-2.08	7.82E-116	↑	Nucleoporin NUP188-like protein, partial [B. mutus]
ENSBTAP00000022442-D1	-2.08	0.00E+00	↑	DNA polymerase delta subunit 3, partial [B. mutus]
ENSP00000363135-D1	-2.08	0.00E+00	↑	uncharacterized protein C6orf106 homolog [Bison bison bison]
ENSBTAP00000051390-D1	-2.08	9.20E-41	↑	uncharacterized protein CCDC7 isoform X1 [B. taurus]
ENSBTAP00000017199-D1	-2.07	1.67E-94	↑	Rod cGMP-specific 3',5'-cyclic phosphodiesterase subunit alpha [B. mutus]
ENSP00000337697-D1	-2.07	4.98E-28	↑	Neuronal migration protein doublecortin, partial [B. mutus]
ENSBTAP00000029169-D2	-2.07	9.44E-84	↑	AP-3 complex subunit mu-2 isoform X2 [B. mutus]
ENSP00000419879-D1	-2.07	7.99E-15	↑	Phosphatidate cytidylyltransferase 2, partial [B. mutus]
ENSBTAP00000014878-D1	-2.07	1.78E-28	↑	Bcl10-interacting CARD protein, partial [B. mutus]
ENSP00000230124-D1	-2.07	5.30E-159	↑	polyphosphoinositide phosphatase [B. taurus]
yakG014895	-2.07	2.37E-37	↑	hypothetical protein M91_06192 [B. mutus]
ENSBTAP00000014120-D1	-2.07	5.99E-40	↑	Propionyl-CoA carboxylase alpha chain, mitochondrial, partial [B. mutus]
ENSBTAP00000008055-D1	-2.07	1.62E-92	↑	serine/threonine-protein kinase PLK3 [B. mutus]
ENSP00000400010-D1	-2.07	1.91E-152	↑	receptor-type tyrosine-protein phosphatase eta [B. mutus]
ENSP00000262241-D1	-2.06	2.51E-18	↑	REST corepressor 1, partial [B. mutus]
ENSP00000380785-D1	-2.06	7.39E-08	↑	Protein Hook-like protein 2, partial [B. mutus]
ENSBTAP00000038956-D1	-2.06	1.65E-159	↑	RING finger protein 114, partial [B. mutus]
ENSBTAP00000006514-D1	-2.06	5.14E-119	↑	ETS domain-containing protein Elk-4, partial [B. mutus]
ENSP00000305699-D1	-2.06	0.00E+00	↑	Protein Hook-like protein 3, partial [B. mutus]
ENSP00000261517-D1	-2.06	1.44E-268	↑	Vacuolar protein sorting-associated protein 13C, partial [B. mutus]
ENSBTAP00000018279-D1	-2.06	1.21E-112	↑	Transcriptional repressor CTCF [B. mutus]
ENSP00000008311-D1	-2.06	0.00E+00	↑	zinc finger protein 200 [B. taurus]
ENSP00000356792-D2	-2.05	0.00E+00	↑	lymphotactin [Capra hircus]
ENSP00000352011-D1	-2.05	3.81E-18	↑	Voltage-dependent T-type calcium channel subunit alpha-1G [B. mutus]
ENSBTAP00000014899-D1	-2.05	7.34E-45	↑	DnaJ-like protein subfamily C member 11, partial [B. mutus]
ENSP00000203407-D1	-2.05	1.15E-39	↑	Cytochrome b-c1 complex subunit 1, mitochondrial [B. mutus]
ENSBTAP00000053746-D1	-2.05	6.74E-218	↑	Tescalcin, partial [B. mutus]
ENSP00000370719-D1	-2.05	5.40E-36	↑	intersectin-1 isoform X1 [B. mutus]
ENSP00000308546-D1	-2.05	1.79E-69	↑	7SK snRNA methylphosphate capping enzyme [Bubalus bubalis]
ENSP00000381055-D1	-2.04	3.40E-40	↑	Dermatan-sulfate epimerase-like protein, partial [B. mutus]
ENSBTAP00000053827-D1	-2.04	0.00E+00	↑	Forkhead box protein J3, partial [B. mutus]
ENSBTAP00000007220-D1	-2.04	2.07E-18	↑	Cyclin-Y, partial [B. mutus]
ENSBTAP00000030064-D1	-2.04	2.60E-211	↑	zinc finger protein ubi-d4 isoform X1 [Loxodonta africana]
ENSBTAP00000045667-D1	-2.04	1.48E-34	↑	uncharacterized protein LOC777593 precursor [B. taurus]
ENSBTAP00000027922-D1	-2.04	5.85E-167	↑	striatin-interacting protein 1 [Bubalus bubalis]
ENSBTAP00000011581-D2	-2.04	2.09E-25	↑	high-mobility group 20B [Homo sapiens]
ENSBTAP00000053635-D1	-2.04	0.00E+00	↑	DENN domain-containing protein 5A, partial [B. mutus]
ENSBTAP00000003115-D1	-2.03	1.28E-29	↑	uncharacterized protein LOC102271444 [B. mutus]
ENSBTAP00000001096-D1	-2.03	7.33E-184	↑	Integral membrane protein GPR137B, partial [B. mutus]
ENSBTAP00000013304-D1	-2.03	3.38E-191	↑	Low affinity cationic amino acid transporter 2 [B. mutus]
ENSBTAP00000019665-D1	-2.02	5.74E-24	↑	5'-nucleotidase domain-containing protein 2 [B. mutus]
ENSBTAP00000024963-D1	-2.02	4.12E-142	↑	Protein FAM117B, partial [B. mutus]
ENSBTAP00000024072-D1	-2.02	2.05E-24	↑	nicotinate-nucleotide pyrophosphorylase [carboxylating] [Bison bison bison]
ENSP00000231061-D1	-2.02	1.54E-97	↑	SPARC isoform X1 [Callithrix jacchus]
ENSBTAP00000000872-D1	-2.02	1.01E-57	↑	transcription factor 20 [B. mutus]

ENSP00000248975-D2	-2.02	5.86E-111	↑	14-3-3 protein eta, partial [B. mutus]
ENSBTAP00000012242-D1	-2.02	5.04E-11	↑	Ena/VASP-like protein, partial [B. mutus]
ENSP00000416125-D1	-2.02	2.30E-85	↑	rho guanine nucleotide exchange factor 38 [Bison bison bison]
ENSP00000328813-D1	-2.02	4.46E-29	↑	Potassium voltage-gated channel subfamily H member 8, partial [B. mutus]
ENSBTAP00000018637-D1	-2.01	2.06E-116	↑	Rhotekin-2, partial [B. mutus]
ENSBTAP00000010675-D1	-2.01	7.20E-157	↑	polymerase delta-interacting protein 2 [Microcebus murinus]
ENSP00000288228-D1	-2.01	0.00E+00	↑	Protein FAM81A, partial [B. mutus]
ENSBTAP00000007404-D1	-2.01	3.08E-30	↑	Valyl-tRNA synthetase [B. mutus]
ENSP00000265165-D1	-2.01	9.78E-268	↑	lymphoid enhancer-binding factor 1 [B. taurus]
ENSP00000307854-D1	-2.01	7.54E-170	↑	protein-L-isoaspartate O-methyltransferase domain-containing protein 2 isoform X1 [B. mutus]
ENSBTAP00000046380-D1	-2.01	5.67E-36	↑	Histone H3-like centromeric protein A, partial [B. mutus]
ENSBTAP00000013741-D1	-2.01	1.49E-84	↑	transmembrane protein 169 [B. mutus]
ENSP00000413956-D1	-2.00	2.81E-13	↑	Zinc finger protein 575, partial [B. mutus]
ENSP00000388192-D6	-2.00	0.00E+00	↑	hypothetical protein M91_03048 [B. mutus]
ENSP00000335384-D1	-2.00	3.23E-49	↑	Zona pellucida-binding protein 2, partial [B. mutus]
ENSBTAP00000053242-D1	-2.00	7.32E-43	↑	Lipoma-preferred partner, partial [B. mutus]
ENSBTAP00000017259-D1	-2.00	9.00E-105	↑	Phosphopantothenate--cysteine ligase, partial [B. mutus]
ENSP00000363362-D1	-2.00	2.05E-70	↑	uncharacterized protein KIAA1958 homolog isoform X1 [B. taurus]
ENSP00000414088-D1	-2.00	5.25E-107	↑	disintegrin and metalloproteinase domain-containing protein 19 [B. mutus]
ENSP00000383433-D1	-2.00	1.05E-131	↑	probable palmitoyltransferase ZDHHC20 [B. taurus]
ENSBTAP00000003750-D2	-2.00	3.84E-24	↑	Protein DDI1-like protein 2 [B. mutus]
ENSBTAP00000053515-D1	-2.00	1.21E-33	↑	AT-rich interactive domain-containing protein 1B, partial [B. mutus]
ENSBTAP00000008416-D1	-2.00	4.44E-15	↑	Enhancer of mRNA-decapping protein 4 [B. mutus]
ENSBTAP00000005218-D1	-2.00	1.37E-24	↑	hydrocephalus-inducing protein homolog isoform X1 [B. taurus]
ENSP00000260187-D1	-2.00	2.71E-284	↑	ubiquitin carboxyl-terminal hydrolase 2 [B. taurus]
ENSBTAP00000023912-D1	-1.99	5.61E-16	↑	zyxin [B. mutus]
ENSBTAP00000023082-D1	-1.99	2.97E-80	↑	Protein diaphanous-like protein 1, partial [B. mutus]
ENSP00000216797-D1	-1.99	0.00E+00	↑	NF-kappa-B inhibitor alpha [B. taurus]
ENSBTAP00000006074-D1	-1.99	5.35E-14	↑	cartilage oligomeric matrix protein precursor [B. taurus]
ENSP00000384114-D1	-1.99	3.42E-101	↑	SET domain-containing protein 5 isoform X7 [B. taurus]
ENSP00000215587-D1	-1.99	1.16E-10	↑	DNA-directed RNA polymerases I, II, and III subunit RPABC1 [Homo sapiens]
ENSP00000323264-D1	-1.98	5.61E-92	↑	MARVEL domain-containing protein 2 isoform X1 [B. mutus]
ENSP00000355884-D2	-1.98	2.61E-43	↑	TPA: serine/threonine-protein kinase MARK1-like [B. taurus]
ENSBTAP00000032740-D1	-1.98	3.92E-31	↑	Zinc finger protein 79 [B. mutus]
ENSBTAP00000003283-D1	-1.98	0.00E+00	↑	Rho GTPase-activating protein 10, partial [B. mutus]
ENSP00000365002-D1	-1.98	6.38E-27	↑	abhydrolase domain-containing protein 16A [B. mutus]
ENSBTAP00000021613-D1	-1.98	3.70E-23	↑	Retinoblastoma-like protein 2 [B. mutus]
ENSP00000359598-D1	-1.98	0.00E+00	↑	outer dense fiber protein 2-like [Bubalus bubalis]
ENSP00000352119-D8	-1.98	5.06E-50	↑	histone H2A type 1-E-like [Leptonychotes weddellii]
ENSP00000310120-D1	-1.98	3.63E-35	↑	Alpha-1,2-glucosyltransferase ALG10-A [B. mutus]
ENSP00000335040-D1	-1.98	7.73E-20	↑	Niban-like protein 2, partial [B. mutus]
ENSP00000389841-D2	-1.98	6.12E-152	↑	zinc finger protein with KRAB and SCAN domains 7 [B. mutus]
ENSBTAP00000021723-D1	-1.97	1.14E-08	↑	protein rogdi homolog [Bison bison bison]
ENSBTAP00000020651-D1	-1.97	4.44E-52	↑	rho GTPase-activating protein 35 [B. taurus]
ENSBTAP00000034568-D1	-1.97	1.21E-33	↑	Na(+)/H(+) exchange regulatory cofactor NHE-RF1, partial [B. mutus]
ENSBTAP00000024880-D1	-1.97	1.44E-38	↑	Acyl-CoA synthetase short-chain family member 3, mitochondrial, partial [B. mutus]
ENSP00000282572-D1	-1.97	2.21E-50	↑	cyclin-O [B. mutus]
ENSBTAP00000053300-D1	-1.96	0.00E+00	↑	M-phase phosphoprotein 8 [B. mutus]
ENSP00000352262-D1	-1.96	3.54E-215	↑	Histone-lysine N-methyltransferase MLL, partial [B. mutus]
ENSP00000311202-D1	-1.96	5.45E-15	↑	BTB/POZ domain-containing adapter for CUL3-mediated RhoA degradation protein 1 [B. mutus]
ENSBTAP00000052927-D1	-1.96	2.44E-16	↑	ras-related protein Rap-2b [B. taurus]
ENSP00000374147-D1	-1.96	5.49E-210	↑	MBT domain-containing protein 1 [B. mutus]
ENSBTAP00000005255-D1	-1.96	9.34E-84	↑	UPF0577 protein KIAA1324-like protein, partial [B. mutus]
ENSBTAP00000003501-D1	-1.96	6.71E-58	↑	V-type proton ATPase catalytic subunit A [B. taurus]
ENSP00000382239-D1	-1.96	2.40E-156	↑	RNA-binding protein 12B [B. mutus]

ENSP00000381932-D1	-1.96	1.81E-239	↑	Dual specificity tyrosine-phosphorylation-regulated kinase 1A, partial [B. mutus]
ENSBTAP00000026137-D1	-1.96	1.58E-235	↑	src-like-adapter 2 [B. taurus]
ENSBTAP00000031225-D1	-1.96	3.58E-35	↑	poly(ADP-ribose) glycohydrolase isoform X1 [B. mutus]
ENSP00000256194-D1	-1.96	0.00E+00	↑	Protein MICAL-2 [B. mutus]
ENSP00000317442-D1	-1.96	3.76E-48	↑	kelch-like protein 36 [B. taurus]
ENSP00000380244-D1	-1.95	0.00E+00	↑	Regulator of nonsense transcripts 2, partial [B. mutus]
ENSP00000368363-D1	-1.95	1.30E-255	↑	serine/threonine-protein kinase Nek1 [B. mutus]
ENSP00000376268-D1	-1.95	2.58E-226	↑	SEC14-like protein 1 [B. mutus]
ENSBTAP00000011619-D1	-1.95	6.29E-27	↑	DNA-directed RNA polymerase III subunit RPC6 isoform X2 [Ovis aries]
ENSP00000410207-D1	-1.95	1.47E-12	↑	methionine-R-sulfoxide reductase B1 [Bubalus bubalis]
ENSP00000283632-D2	-1.95	4.14E-12	↑	protein RMD5 homolog B [B. mutus]
ENSP00000221543-D1	-1.95	4.01E-07	↑	TBC1 domain family member 17, partial [B. mutus]
ENSBTAP00000004504-D24	-1.95	8.25E-05	↑	hypothetical protein M91_02901, partial [B. mutus]
ENSBTAP00000023260-D1	-1.95	2.37E-03	↑	uncharacterized protein LOC102270295 [B. mutus]
ENSBTAP00000025049-D3	-1.95	8.21E-03	↑	POU domain, class 4, transcription factor 1 [B. mutus]
ENSBTAP00000006547-D1	-1.95	8.21E-03	↑	palmitoyltransferase ZDHHC23 [B. taurus]
ENSP00000268720-D2	-1.95	8.21E-03	↑	copine-6 isoform X1 [B. mutus]
ENSBTAP00000018368-D3	-1.95	8.21E-03	↑	ATM interactor [Bubalus bubalis]
ENSBTAP00000003544-D1	-1.95	6.13E-96	↑	hypothetical protein M91_08798, partial [B. mutus]
ENSP00000260283-D1	-1.95	1.11E-31	↑	Rho GTPase-activating protein 20, partial [B. mutus]
ENSBTAP00000008327-D1	-1.95	1.31E-220	↑	Death-associated protein 1, partial [B. mutus]
ENSP00000260372-D1	-1.94	0.00E+00	↑	HAUS augmin-like complex subunit 2 [B. taurus]
ENSP00000365398-D1	-1.94	0.00E+00	↑	Ras-related protein Ral-B, partial [B. mutus]
ENSP00000401197-D1	-1.94	3.18E-50	↑	Ubiquitin carboxyl-terminal hydrolase 19 [B. mutus]
ENSBTAP00000051149-D1	-1.94	0.00E+00	↑	hypothetical protein M91_05926, partial [B. mutus]
ENSP00000338360-D1	-1.94	4.50E-54	↑	transcription factor E3 [B. taurus]
ENSBTAP00000026621-D1	-1.94	2.78E-52	↑	Casein kinase I isoform delta [Fukomys damarensis]
ENSP00000309595-D1	-1.94	1.70E-50	↑	twinkle protein, mitochondrial [B. mutus]
ENSBTAP00000017304-D1	-1.94	1.41E-10	↑	Coiled-coil domain-containing protein 13 [B. mutus]
ENSBTAP00000025200-D1	-1.94	4.00E-10	↑	histone-lysine N-methyltransferase SETMAR [B. mutus]
ENSP00000408891-D1	-1.93	1.14E-09	↑	Pyruvate kinase isozymes R/L, partial [B. mutus]
ENSP00000229268-D2	-1.93	0.00E+00	↑	Ubiquitin carboxyl-terminal hydrolase 13, partial [B. mutus]
ENSBTAP00000036528-D1	-1.93	2.78E-64	↑	inactive ubiquitin thioesterase FAM105A [B. mutus]
ENSP00000419760-D1	-1.93	2.96E-16	↑	transcription initiation factor TFIID subunit 6 isoform X1 [Pantholops hodgsonii]
ENSP00000219548-D1	-1.93	8.31E-16	↑	E3 ubiquitin-protein ligase CHIP [Camelus bactrianus]
ENSP00000355343-D1	-1.93	0.00E+00	↑	Ubiquitin-associated protein 2-like protein [B. mutus]
ENSP00000217901-D1	-1.93	2.73E-46	↑	isocitrate dehydrogenase [NAD] subunit gamma, mitochondrial isoform X1 [B. taurus]
ENSBTAP00000004704-D1	-1.93	0.00E+00	↑	synaptonemal complex protein 2 [B. mutus]
ENSP00000369614-D1	-1.93	1.74E-247	↑	Centrobin [B. mutus]
ENSP00000261349-D1	-1.93	1.25E-180	↑	Low-density lipoprotein receptor-related protein 6, partial [B. mutus]
ENSBTAP00000002653-D1	-1.93	0.00E+00	↑	Tyrosine-protein phosphatase non-receptor type 11, partial [B. mutus]
ENSP00000262525-D1	-1.93	1.48E-13	↑	zinc finger protein 629 [B. mutus]
ENSBTAP00000053701-D1	-1.93	1.79E-40	↑	zinc transporter 4 [B. taurus]
ENSBTAP00000026945-D1	-1.93	3.69E-55	↑	RNA exonuclease 1-like protein, partial [B. mutus]
ENSP00000378405-D1	-1.93	3.48E-22	↑	[3-methyl-2-oxobutanoate dehydrogenase [lipoamide]] kinase, mitochondrial precursor [B. taurus]
ENSP00000264313-D1	-1.93	8.54E-272	↑	SLAIN motif-containing protein 2 [B. taurus]
ENSBTAP00000021018-D1	-1.93	7.72E-94	↑	AMP deaminase 3 [B. mutus]
ENSP00000379739-D1	-1.92	3.56E-132	↑	actin-binding protein IPP [B. taurus]
ENSBTAP00000016104-D1	-1.92	2.32E-54	↑	Homeobox protein SIX1, partial [B. mutus]
ENSBTAP00000021104-D1	-1.92	2.85E-36	↑	RAD9, HUS1, RAD1-interacting nuclear orphan protein 1 [B. mutus]
ENSBTAP00000014880-D1	-1.92	1.43E-76	↑	Ninjurin-1, partial [B. mutus]
ENSBTAP00000009708-D1	-1.92	9.80E-42	↑	Cytohesin-3, partial [B. mutus]
ENSP00000222726-D1	-1.92	2.13E-10	↑	Homeobox protein Hox-A5 [B. mutus]
ENSBTAP00000017356-D7	-1.92	1.75E-06	↑	Cathepsin G, partial [B. mutus]
ENSBTAP00000019540-D1	-1.92	8.25E-295	↑	putative ATP-dependent RNA helicase DHX57 [B. mutus]

ENSBTAP00000040815-D1	-1.92	7.37E-70	↑	SPARC-related modular calcium-binding protein 1 [B. mutus]
ENSP00000355621-D1	-1.92	1.69E-39	↑	Tetratricopeptide repeat protein 13, partial [B. mutus]
ENSBTAP00000003674-D1	-1.92	0.00E+00	↑	transcription initiation factor TFIID subunit 3 isoform X2 [B. taurus]
ENSBTAP00000019817-D1	-1.92	6.05E-10	↑	Myomesin-3 [B. mutus]
ENSBTAP00000053804-D1	-1.92	5.58E-86	↑	ADAMTS-like protein 1 [B. mutus]
ENSP00000254654-D1	-1.91	1.21E-48	↑	integrin-linked kinase-associated serine/threonine phosphatase 2C [B. mutus]
ENSP00000379474-D1	-1.91	1.17E-28	↑	Tyrosine-protein phosphatase non-receptor type 5, partial [B. mutus]
ENSBTAP00000006718-D1	-1.91	6.53E-61	↑	cell cycle control protein 50A isoform X1 [Bubalus bubalis]
ENSBTAP00000025898-D1	-1.91	9.10E-275	↑	uncharacterized protein C7orf31 homolog [B. mutus]
ENSP00000366280-D1	-1.91	8.50E-25	↑	VIP36-like protein precursor [B. taurus]
yakG013905	-1.91	5.01E-258	↑	Rab3 GTPase-activating protein non-catalytic subunit [B. mutus]
ENSP00000383954-D1	-1.91	3.56E-62	↑	CCR4-NOT transcription complex subunit 3, partial [B. mutus]
ENSBTAP00000008650-D1	-1.91	2.84E-74	↑	importin subunit beta-1 [B. taurus]
ENSP00000367715-D1	-1.91	1.43E-35	↑	Doublecortin domain-containing protein 2, partial [B. mutus]
ENSBTAP00000024017-D1	-1.91	0.00E+00	↑	short/branched chain specific acyl-CoA dehydrogenase, mitochondrial [B. mutus]
ENSP00000313420-D1	-1.91	9.63E-111	↑	DNA-dependent protein kinase catalytic subunit [B. mutus]
ENSBTAP00000023854-D1	-1.91	4.01E-35	↑	Nuclear envelope pore membrane protein POM 121C, partial [B. mutus]
ENSBTAP00000002637-D1	-1.91	0.00E+00	↑	ataxin-7-like protein 1 isoform X3 [Bison bison bison]
ENSP00000262442-D2	-1.91	3.20E-47	↑	dynein heavy chain 17, axonemal isoform X1 [B. taurus]
ENSP00000273980-D1	-1.90	2.31E-144	↑	TBC domain-containing protein kinase-like protein isoform X1 [B. mutus]
ENSBTAP00000028616-D1	-1.90	5.63E-58	↑	Mediator of RNA polymerase II transcription subunit 24 [B. mutus]
ENSBTAP00000009482-D1	-1.90	2.80E-15	↑	Histone deacetylase 11, partial [B. mutus]
ENSP00000338785-D1	-1.90	4.28E-05	↑	stAR-related lipid transfer protein 13 [B. mutus]
ENSP00000336888-D1	-1.90	4.28E-05	↑	choline transporter-like protein 2 [B. taurus]
ENSP00000228567-D1	-1.90	5.06E-55	↑	synaptotagmin-10 [B. mutus]
ENSBTAP00000039687-D1	-1.90	1.85E-37	↑	mannosyl-oligosaccharide 1,2-alpha-mannosidase IB isoform X2 [Ovis aries]
ENSP00000379861-D1	-1.90	1.96E-32	↑	MKL/myocardin-like protein 1, partial [B. mutus]
ENSBTAP00000053365-D1	-1.90	6.89E-268	↑	WD repeat and FYVE domain-containing protein 3, partial [B. mutus]
ENSBTAP00000013096-D46	-1.90	3.23E-67	↑	hypothetical protein M91_18033, partial [B. mutus]
ENSBTAP00000008644-D1	-1.90	2.51E-34	↑	prolyl 4-hydroxylase subunit alpha-3 precursor [B. taurus]
ENSBTAP00000024228-D1	-1.90	1.11E-41	↑	cytochrome b ascorbate-dependent protein 3 [B. taurus]
ENSP00000300896-D1	-1.90	5.06E-270	↑	ubiquitin carboxyl-terminal hydrolase 32 [B. mutus]
ENSBTAP00000046639-D1	-1.90	1.27E-17	↑	Calcyphosin-2, partial [B. mutus]
ENSBTAP00000022223-D1	-1.89	2.81E-38	↑	Centrosomal protein of 68 kDa, partial [B. mutus]
ENSBTAP00000008917-D1	-1.89	4.99E-13	↑	Napsin-A, partial [B. mutus]
ENSP00000232219-D1	-1.89	1.60E-289	↑	retinol-binding protein 1 isoform X2 [Ovis aries musimon]
ENSP00000415054-D1	-1.89	2.47E-131	↑	ecto-NOX disulfide-thiol exchanger 1 isoform X1 [Bubalus bubalis]
ENSP00000363397-D1	-1.89	1.66E-41	↑	ceramide glucosyltransferase [Canis lupus familiaris]
ENSP00000411242-D1	-1.89	0.00E+00	↑	Fibronectin type III domain-containing protein 3B [B. mutus]
ENSP00000398523-D1	-1.89	0.00E+00	↑	Calcium-binding and coiled-coil domain-containing protein 2 [B. mutus]
ENSP00000309558-D1	-1.89	2.77E-135	↑	TATA-binding protein-associated factor 2N isoform X4 [Ochotona princeps]
ENSP00000252487-D1	-1.89	3.32E-14	↑	mitochondrial import receptor subunit TOM40 homolog isoform X1 [Bubalus bubalis]
ENSP00000358727-D3	-1.89	1.19E-23	↑	glutathione S-transferase omega-2 [B. taurus]
ENSBTAP00000009046-D1	-1.89	1.06E-34	↑	ras GTPase-activating-like protein IQGAP3 [B. mutus]
ENSP00000343690-D1	-1.89	2.77E-45	↑	dihydropyrimidinase-related protein 3 isoform X1 [B. mutus]
ENSBTAP00000020055-D1	-1.89	2.22E-251	↑	LanC-like protein 1, partial [B. mutus]
ENSP00000323645-D1	-1.88	9.24E-154	↑	Tetratricopeptide repeat protein 39C, partial [B. mutus]
ENSBTAP00000004009-D1	-1.88	0.00E+00	↑	RWD domain-containing protein 4 isoform X1 [B. taurus]
ENSBTAP00000040681-D1	-1.88	1.63E-20	↑	apoptosis regulator BAX [B. taurus]
ENSP00000227524-D1	-1.88	1.37E-44	↑	Pre-mRNA-processing factor 19, partial [B. mutus]
ENSP00000221130-D2	-1.87	3.93E-93	↑	glutathione reductase, mitochondrial-like [B. mutus]
ENSP00000381549-D1	-1.87	0.00E+00	↑	FERM domain-containing protein 4B [B. mutus]
ENSBTAP00000027890-D1	-1.87	1.08E-71	↑	Placenta-specific protein 9, partial [B. mutus]
ENSBTAP00000015103-D3	-1.87	6.33E-111	↑	protein lin-7 homolog A isoform X1 [Cricetulus griseus]
ENSBTAP00000019573-D1	-1.87	4.40E-78	↑	Ubiquitin-like protein ISG15, partial [B. mutus]

ENSBTAP00000018888-D1	-1.87	2.22E-05	↑	60S ribosomal protein L35a-like [B. mutus]
ENSP00000377665-D1	-1.87	1.29E-20	↑	GTPase-activating protein and VPS9 domain-containing protein 1 [Capra hircus]
ENSBTAP00000011774-D5	-1.87	7.64E-41	↑	hypothetical protein M91_07167, partial [B. mutus]
ENSBTAP00000053353-D1	-1.86	1.99E-59	↑	Leucine-rich repeat serine/threonine-protein kinase 2, partial [B. mutus]
ENSBTAP00000027232-D1	-1.86	1.11E-82	↑	NACHT, LRR and PYD domains-containing protein 1 [B. mutus]
ENSBTAP00000009476-D2	-1.86	2.97E-155	↑	Etoposide-induced protein 2.4-like protein, partial [B. mutus]
ENSP00000298119-D1	-1.86	5.88E-13	↑	Leucine-rich repeat and fibronectin type-III domain-containing protein 5, partial [B. mutus]
ENSBTAP00000011826-D1	-1.86	3.38E-139	↑	suppressor of cytokine signaling 5 [B. mutus]
ENSBTAP00000030166-D1	-1.86	0.00E+00	↑	Protein FAM50A, partial [B. mutus]
ENSP00000255189-D1	-1.86	2.62E-15	↑	Dimethylglycine dehydrogenase, mitochondrial, partial [B. mutus]
ENSP00000384690-D3	-1.86	3.90E-137	↑	Monocyte to macrophage differentiation protein, partial [B. mutus]
ENSP00000397552-D14	-1.86	4.54E-231	↑	Actin, cytoplasmic 1 [B. mutus]
ENSP00000376344-D1	-1.86	1.66E-47	↑	Putative RNA-binding protein 19 [B. mutus]
ENSBTAP00000001861-D1	-1.86	4.96E-16	↑	39S ribosomal protein L4, mitochondrial [B. mutus]
ENSBTAP00000015332-D1	-1.86	0.00E+00	↑	maspardin [B. taurus]
ENSP00000343873-D1	-1.85	4.76E-132	↑	NAD(P) transhydrogenase, mitochondrial [B. mutus]
ENSBTAP00000053642-D1	-1.85	2.38E-61	↑	RING finger protein 31, partial [B. mutus]
ENSBTAP00000014625-D1	-1.85	0.00E+00	↑	Protein phosphatase Slingshot-like protein 2 [B. mutus]
ENSBTAP00000002972-D1	-1.85	8.77E-111	↑	golgi glycoprotein 1 [B. mutus]
ENSP00000346255-D1	-1.85	5.39E-21	↑	protein phosphatase PTC7 homolog [Trichechus manatus latirostris]
ENSBTAP00000033144-D2	-1.85	2.55E-42	↑	Thioredoxin, mitochondrial [B. mutus]
ENSP00000261991-D1	-1.85	1.28E-218	↑	Ribosomal protein S6 kinase alpha-5, partial [B. mutus]
ENSP00000211998-D1	-1.85	5.44E-151	↑	vinculin-like [B. mutus]
ENSBTAP00000013925-D1	-1.85	0.00E+00	↑	HMG domain-containing protein 4 [B. mutus]
ENSBTAP00000021771-D1	-1.85	3.31E-45	↑	tRNA-splicing endonuclease subunit Sen15 [B. mutus]
ENSP00000216039-D1	-1.84	1.26E-147	↑	josephin-1 [B. mutus]
ENSP00000381897-D1	-1.84	0.00E+00	↑	Putative Polycomb group protein ASXL2, partial [B. mutus]
ENSBTAP00000025590-D1	-1.84	8.18E-50	↑	Kinesin-like protein KIF13A, partial [B. mutus]
ENSBTAP00000007341-D1	-1.84	8.56E-09	↑	serine/threonine-protein kinase 19 isoform X3 [B. taurus]
ENSP00000035383-D1	-1.84	4.48E-34	↑	RecName: Full=Leucine-rich repeat-containing protein 7; AltName: Full=Densin-180; Short=Densin
ENSP00000265362-D1	-1.84	8.00E-188	↑	semaphorin-3A isoform X2 [B. taurus]
ENSBTAP00000009123-D1	-1.84	1.02E-23	↑	GTPase-activating Rap/Ran-GAP domain-like protein 3 [B. mutus]
ENSP00000403683-D1	-1.84	0.00E+00	↑	SOSS complex subunit B2 [B. taurus]
ENSP00000327349-D1	-1.84	1.96E-94	↑	transmembrane and coiled-coil domains protein 1 isoform X2 [B. taurus]
ENSBTAP00000041740-D1	-1.84	1.76E-73	↑	transcription elongation factor SPT5 isoform X2 [Pantholops hodgsonii]
ENSBTAP00000034423-D1	-1.84	8.32E-52	↑	ran-binding protein 17-like, partial [B. mutus]
ENSBTAP00000047840-D1	-1.84	2.07E-16	↑	NADH dehydrogenase [ubiquinone] iron-sulfur protein 6, mitochondrial, partial [B. mutus]
ENSP00000344847-D1	-1.83	8.53E-36	↑	A disintegrin and metalloproteinase with thrombospondin motifs 12 isoform X2 [B. mutus]
ENSBTAP00000006800-D1	-1.83	1.23E-51	↑	gamma-soluble NSF attachment protein [Bubalus bubalis]
ENSP00000350228-D1	-1.83	0.00E+00	↑	cytosolic carboxypeptidase 2 isoform X1 [Bison bison bison]
ENSBTAP00000025322-D1	-1.83	3.76E-07	↑	NADH dehydrogenase [ubiquinone] 1 alpha subcomplex subunit 11 [B. taurus]
ENSP00000413017-D1	-1.83	5.77E-17	↑	coiled-coil domain-containing protein 28B isoform X3 [Leptonychotes weddellii]
ENSP00000258301-D1	-1.82	1.52E-25	↑	Syntaxin-6 [B. mutus]
ENSBTAP00000003433-D1	-1.82	3.47E-196	↑	zinc finger protein ZPR1 [B. taurus]
ENSP00000324956-D1	-1.82	0.00E+00	↑	E3 ubiquitin-protein ligase RNF14 [B. taurus]
ENSP00000350945-D2	-1.82	2.16E-184	↑	Myosin-Vb, partial [B. mutus]
ENSBTAP00000006361-D1	-1.82	6.03E-93	↑	RING finger and SPRY domain-containing protein 1 [B. mutus]
ENSBTAP00000026725-D1	-1.82	1.15E-74	↑	collagen alpha-1(XII) chain [B. mutus]
ENSP00000383063-D1	-1.82	5.32E-39	↑	nuclear receptor-interacting protein 1-like [B. mutus]
ENSP00000359603-D1	-1.82	1.48E-48	↑	Collagen alpha-1(XXIV) chain, partial [B. mutus]
ENSP00000262919-D1	-1.82	3.04E-18	↑	attractin, partial [B. mutus]
ENSP00000354453-D12	-1.81	1.69E-45	↑	zinc finger protein 471 isoform X2 [B. taurus]
ENSBTAP00000014795-D1	-1.81	2.19E-37	↑	MORN repeat-containing protein 4 [Ovis aries]
ENSBTAP00000053096-D1	-1.81	4.73E-45	↑	.
ENSBTAP00000040922-D1	-1.81	2.88E-12	↑	Coiled-coil domain-containing protein 114, partial [B. mutus]

ENSP00000369739-D1	-1.81	2.25E-27	↑	valacyclovir hydrolase [B. mutus]
ENSBTAP00000046644-D1	-1.81	1.70E-36	↑	1-phosphatidylinositol-4,5-bisphosphate phosphodiesterase beta-1, partial [B. mutus]
ENSP00000403802-D1	-1.81	1.89E-14	↑	F-box only protein 10 isoform X1 [Bison bison bison]
ENSBTAP00000008072-D2	-1.81	9.90E-188	↑	Microtubule-associated proteins 1A/1B light chain 3A, partial [B. mutus]
ENSP00000307541-D1	-1.81	6.72E-09	↑	BCL-6 corepressor-like protein 1 [B. mutus]
ENSP00000286827-D1	-1.81	8.29E-122	↑	T-lymphoma invasion and metastasis-inducing protein 1-like isoform X1 [B. mutus]
ENSBTAP00000019692-D1	-1.81	5.70E-21	↑	armadillo repeat-containing protein 7 [B. taurus]
ENSP00000363708-D1	-1.81	4.97E-155	↑	bone morphogenetic protein receptor type-2 [B. mutus]
yakG028808	-1.80	2.24E-54	↑	Microtubule-associated serine/threonine-protein kinase 4 [B. mutus]
ENSBTAP00000037586-D2	-1.80	2.61E-74	↑	hypothetical protein M91_13163 [B. mutus]
ENSP00000339834-D1	-1.80	1.81E-89	↑	U11/U12 small nuclear ribonucleoprotein 48 kDa protein [B. mutus]
ENSP00000225972-D1	-1.80	1.53E-45	↑	leucine-rich repeat-containing protein 59 [B. mutus]
ENSP00000352541-D1	-1.80	5.02E-89	↑	ubiquitin-associated protein 1 isoform X1 [B. mutus]
ENSP00000324205-D1	-1.80	3.32E-29	↑	charged multivesicular body protein 4a [B. mutus]
ENSP00000387426-D1	-1.80	7.11E-51	↑	cysteine and histidine-rich protein 1 isoform X1 [B. taurus]
ENSBTAP00000013799-D1	-1.80	5.67E-23	↑	Podocalyxin, partial [B. mutus]
ENSP00000352447-D1	-1.80	2.81E-14	↑	specifically androgen-regulated gene protein [B. mutus]
ENSBTAP00000005810-D1	-1.80	4.25E-12	↑	Chordin-like protein 1, partial [B. mutus]
ENSP00000234739-D1	-1.80	3.52E-09	↑	B-cell CLL/lymphoma 9 protein isoform X1 [Bison bison bison]
ENSP00000370808-D2	-1.80	1.90E-08	↑	ADP/ATP translocase 3 [B. taurus]
ENSP00000352819-D1	-1.80	3.09E-06	↑	PR domain zinc finger protein 13 [B. mutus]
ENSP00000357431-D1	-1.80	2.01E-54	↑	cAMP-dependent protein kinase inhibitor beta, partial [B. mutus]
ENSP00000271277-D1	-1.80	8.34E-65	↑	CTTNBP2 N-terminal-like protein isoform X1 [B. mutus]
ENSBTAP00000014015-D1	-1.79	0.00E+00	↑	Signal recognition particle 72 kDa protein [B. mutus]
ENSP00000317382-D1	-1.79	2.97E-53	↑	Proton-coupled amino acid transporter 4, partial [B. mutus]
ENSP00000380386-D1	-1.79	2.97E-53	↑	ELKS/Rab6-interacting/CAST family member 1 [B. taurus]
ENSP00000244040-D1	-1.79	1.09E-70	↑	ras-related protein Rab-22A isoform X1 [Ovis aries]
ENSP00000309548-D1	-1.79	1.19E-35	↑	[Pyruvate dehydrogenase [acetyl-transferring]]-phosphatase 2, mitochondrial [B. mutus]
ENSBTAP00000053322-D1	-1.79	2.00E-42	↑	DNA-directed RNA polymerase III subunit RPC2 [B. mutus]
ENSBTAP00000017158-D2	-1.79	0.00E+00	↑	Cellular retinoic acid-binding protein 2, partial [B. mutus]
ENSP00000402584-D1	-1.79	1.37E-45	↑	MAM domain-containing glycosylphosphatidylinositol anchor protein 1 [B. mutus]
ENSP00000414964-D1	-1.79	1.24E-21	↑	coiled-coil domain-containing protein 107 [B. mutus]
ENSP00000358552-D1	-1.79	3.93E-62	↑	DENN domain-containing protein 2C isoform X1 [B. taurus]
ENSBTAP00000012252-D1	-1.79	1.70E-193	↑	hypothetical protein M91_05956, partial [B. mutus]
ENSP00000325002-D1	-1.79	2.02E-48	↑	coatomer subunit gamma-1 [B. taurus]
ENSBTAP00000039471-D1	-1.79	1.61E-06	↑	latrophilin-3-like isoform X3 [B. mutus]
ENSBTAP00000022419-D1	-1.79	3.30E-304	↑	BUD13 homolog isoform X1 [B. mutus]
ENSP00000402457-D1	-1.78	1.23E-24	↑	GRAM domain-containing protein 1B, partial [B. mutus]
ENSP00000358131-D1	-1.78	2.15E-15	↑	OTU domain-containing protein 7B [B. mutus]
ENSP00000379023-D1	-1.78	8.97E-06	↑	TPA: ATP synthase, H+ transporting, mitochondrial F1 complex, epsilon subunit-like [B. taurus]
ENSP00000358548-D1	-1.78	3.19E-89	↑	GTPase NRas [B. mutus]
ENSBTAP00000029211-D1	-1.78	0.00E+00	↑	F-box-like/WD repeat-containing protein TBL1XR1, partial [B. mutus]
ENSBTAP00000021955-D1	-1.78	5.60E-33	↑	Carboxypeptidase E, partial [B. mutus]
ENSBTAP00000019248-D1	-1.78	8.91E-89	↑	adenosine deaminase-like protein [B. mutus]
ENSBTAP00000052035-D10	-1.78	1.76E-11	↑	histone H2A type 1-like [Cavia porcellus]
ENSBTAP00000012348-D1	-1.78	2.80E-08	↑	Type-1 angiotensin II receptor-associated protein, partial [B. mutus]
ENSBTAP00000002634-D1	-1.78	7.99E-100	↑	UBX domain-containing protein 2A [B. taurus]
ENSBTAP00000007703-D1	-1.78	1.78E-177	↑	cyclin-T1 [B. taurus]
ENSBTAP00000012886-D1	-1.78	7.59E-19	↑	Glycerol-3-phosphate dehydrogenase, mitochondrial [B. mutus]
ENSP00000308847-D2	-1.78	0.00E+00	↑	tropomyosin alpha-3 chain-like, partial [B. mutus]
ENSBTAP00000017416-D1	-1.78	8.24E-33	↑	Fructose-bisphosphate aldolase C, partial [B. mutus]
ENSBTAP00000020690-D1	-1.78	3.19E-80	↑	glutamate--cysteine ligase catalytic subunit isoform X1 [B. taurus]
ENSBTAP00000027882-D1	-1.78	3.35E-215	↑	Host cell factor 2 [B. mutus]
ENSBTAP00000018522-D1	-1.78	2.30E-32	↑	Transmembrane and coiled-coil domain-containing protein 7, partial [B. mutus]
ENSP00000254454-D1	-1.78	2.11E-18	↑	hypothetical protein [Pongo abelii]

ENSP00000397900-D2	-1.77	2.30E-208	↑	Protein maestro, partial [B. mutus]
ENSBTAP00000041842-D1	-1.77	2.89E-95	↑	Apoptosis regulatory protein Siva, partial [B. mutus]
ENSBTAP00000008535-D1	-1.77	1.82E-133	↑	metal regulatory transcription factor 1 [B. taurus]
ENSP00000253925-D1	-1.77	1.40E-128	↑	Liprin-alpha-1 [B. mutus]
ENSBTAP00000035932-D1	-1.77	1.74E-100	↑	hypothetical protein M91_12753, partial [B. mutus]
ENSBTAP00000011476-D1	-1.77	6.39E-37	↑	KDEL motif-containing protein 2, partial [B. mutus]
ENSP00000373522-D1	-1.77	6.35E-108	↑	Lysine-specific histone demethylase 1B [B. mutus]
ENSP00000365682-D1	-1.77	2.87E-90	↑	transducin-like enhancer protein 1 isoform X1 [B. mutus]
ENSBTAP00000016102-D1	-1.77	2.32E-118	↑	la-related protein 1 isoform X2 [Ovis aries musimon]
ENSBTAP00000053593-D1	-1.77	3.01E-50	↑	Voltage-dependent R-type calcium channel subunit alpha-1E [B. mutus]
ENSP00000361776-D4	-1.77	6.43E-40	↑	protein BEX5 isoform X2 [B. taurus]
ENSBTAP00000020731-D1	-1.77	0.00E+00	↑	Endoplasmic reticulum metallopeptidase 1, partial [B. mutus]
ENSBTAP00000025659-D2	-1.77	0.00E+00	↑	asparagine--tRNA ligase, cytoplasmic [B. mutus]
ENSP00000394008-D1	-1.77	3.90E-26	↑	gamma-aminobutyric acid receptor-associated protein-like 1 isoform X1 [Zonotrichia albicollis]
ENSBTAP00000019878-D1	-1.77	1.22E-220	↑	Trafficking kinesin-binding protein 2 [B. mutus]
ENSBTAP00000050847-D1	-1.77	0.00E+00	↑	tetratricopeptide repeat protein 9C [B. taurus]
ENSP00000352040-D1	-1.77	1.40E-10	↑	hypothetical protein M91_18803, partial [B. mutus]
ENSP00000215909-D1	-1.76	0.00E+00	↑	Galectin-1, partial [B. mutus]
ENSBTAP00000013636-D1	-1.76	3.06E-21	↑	Hypoxia up-regulated protein 1 [B. mutus]
ENSP00000359222-D1	-1.76	2.04E-120	↑	transmembrane protein 56 [B. mutus]
ENSBTAP00000003140-D1	-1.76	2.37E-56	↑	GPI mannosyltransferase 1 [B. mutus]
ENSBTAP00000048942-D26	-1.76	1.14E-72	↑	zinc finger protein 850-like isoform X1 [Lipotes vexillifer]
ENSP00000262563-D1	-1.76	0.00E+00	↑	CCR4-NOT transcription complex subunit 4 [Camelus dromedarius]
ENSP00000347834-D1	-1.76	1.16E-80	↑	F-box/LRR-repeat protein 3 [Capra hircus]
ENSBTAP00000012237-D1	-1.76	2.58E-05	↑	Zinc finger CCCH domain-containing protein 4, partial [B. mutus]
ENSP00000287675-D1	-1.76	2.41E-18	↑	nuclease EXOG, mitochondrial [B. taurus]
ENSP00000300101-D1	-1.76	2.38E-20	↑	zinc finger and BTB domain-containing protein 39 [B. mutus]
ENSBTAP00000001541-D1	-1.76	2.38E-06	↑	Protein spire-like protein 2, partial [B. mutus]
ENSBTAP00000022271-D1	-1.76	0.00E+00	↑	N-acetyltransferase 10, partial [B. mutus]
ENSBTAP00000005043-D1	-1.75	1.35E-105	↑	Nuclear inhibitor of protein phosphatase 1 [Fukomys damarensis]
ENSP00000355094-D1	-1.75	6.76E-17	↑	QUAKING isoform 6 [Homo sapiens]
ENSP00000384164-D1	-1.75	5.71E-171	↑	Kinesin-like protein KIF16B, partial [B. mutus]
ENSBTAP00000011396-D1	-1.75	4.11E-80	↑	hypothetical protein M91_04433, partial [B. mutus]
ENSP00000320291-D1	-1.75	0.00E+00	↑	Oxysterol-binding protein-related protein 1 [B. mutus]
ENSP00000378807-D1	-1.75	0.00E+00	↑	casein kinase I isoform gamma-3-like isoform X3 [B. mutus]
ENSBTAP00000052461-D1	-1.75	1.92E-89	↑	interferon alpha responsive isoform 2 [B. taurus]
ENSBTAP00000013295-D1	-1.75	1.23E-06	↑	probable palmitoyltransferase ZDHHC1 isoform X4 [Macaca nemestrina]
ENSBTAP00000024505-D1	-1.74	4.91E-37	↑	Proteasome inhibitor PI31 subunit, partial [B. mutus]
ENSP00000221265-D1	-1.74	7.60E-73	↑	RNA polymerase II-associated factor 1 homolog [Fukomys damarensis]
ENSBTAP00000015764-D1	-1.74	5.25E-16	↑	chromatin assembly factor 1 subunit B [B. mutus]
ENSBTAP00000009771-D1	-1.74	7.33E-52	↑	Myb-binding protein 1A [B. mutus]
ENSP00000388124-D1	-1.74	3.10E-74	↑	AP-1 complex subunit mu-2 isoform X1 [B. taurus]
ENSP00000271628-D1	-1.74	8.39E-59	↑	splicing factor 3B subunit 4 [Oryzias latipes]
ENSP00000379032-D1	-1.74	2.01E-31	↑	Exostosin-2, partial [B. mutus]
ENSP00000378914-D1	-1.74	3.88E-25	↑	Serine/threonine-protein kinase ULK2, partial [B. mutus]
ENSP00000373937-D1	-1.74	1.05E-33	↑	Protogenin, partial [B. mutus]
yakG014377	-1.74	2.01E-28	↑	hypothetical protein M91_11177 [B. mutus]
ENSBTAP00000003035-D1	-1.74	1.48E-14	↑	ADP-ribosylation factor-binding protein GGA2, partial [B. mutus]
ENSBTAP00000009691-D1	-1.74	1.44E-80	↑	Eukaryotic translation initiation factor 2-alpha kinase 1, partial [B. mutus]
ENSBTAP00000018350-D1	-1.74	1.12E-92	↑	60 kDa SS-A/Ro ribonucleoprotein, partial [B. mutus]
ENSP00000230449-D1	-1.74	4.66E-106	↑	Exocyst complex component 2 [B. mutus]
ENSBTAP00000029272-D1	-1.73	4.29E-40	↑	Iron-sulfur cluster assembly 2-like protein, mitochondrial [B. mutus]
ENSBTAP00000042980-D1	-1.73	8.60E-86	↑	sororin-like isoform X1 [B. mutus]
ENSP00000365637-D1	-1.73	1.58E-10	↑	Sickle tail protein-like protein, partial [B. mutus]
ENSP00000364145-D4	-1.73	1.29E-124	↑	6-phosphofructo-2-kinase/fructose-2,6-biphosphatase 1, partial [B. mutus]

ENSP00000259698-D1	-1.73	3.01E-22	↑	protein FAM65B-like [B. mutus]
ENSBTAP00000002045-D1	-1.73	0.00E+00	↑	LDLR chaperone MESD [B. mutus]
ENSBTAP00000051958-D1	-1.73	2.19E-13	↑	hypothetical protein M91_15394, partial [B. mutus]
ENSP00000344285-D1	-1.72	9.26E-65	↑	Brain-specific homeobox protein-like protein [B. mutus]
ENSP00000274054-D1	-1.72	5.75E-109	↑	H/ACA ribonucleoprotein complex non-core subunit NAF1, partial [B. mutus]
ENSP00000401191-D1	-1.72	3.88E-103	↑	Putative methyltransferase WBSCR22 [B. mutus]
ENSBTAP00000015295-D1	-1.72	8.04E-52	↑	protein SERAC1 [B. mutus]
ENSBTAP00000036380-D1	-1.72	6.51E-142	↑	Calmodulin-like protein 4, partial [B. mutus]
ENSP00000318185-D1	-1.72	1.86E-194	↑	leucine-rich repeat-containing protein 42 isoform X1 [Bubalus bubalis]
ENSP00000389087-D1	-1.72	7.93E-44	↑	N-acetyl-D-glucosamine kinase, partial [B. mutus]
ENSBTAP00000031993-D1	-1.72	6.38E-24	↑	Heterogeneous nuclear ribonucleoprotein L, partial [B. mutus]
ENSP00000261721-D1	-1.72	6.39E-23	↑	TPA: BTB (POZ) domain containing 1 [B. taurus]
ENSP00000326630-D1	-1.72	8.81E-04	↑	zinc finger protein, FOG family member 1 [B. mutus]
ENSBTAP00000007184-D1	-1.72	8.81E-04	↑	protein SOX-15 [B. mutus]
ENSBTAP00000003059-D1	-1.72	1.72E-69	↑	host cell factor C1 regulator 1 isoform X2 [B. taurus]
ENSBTAP00000006875-D1	-1.72	2.48E-50	↑	serine/threonine-protein kinase WNK1, partial [Bison bison bison]
ENSP00000335193-D1	-1.72	2.52E-71	↑	vam6/Vps39-like protein isoform X1 [B. mutus]
ENSBTAP00000032931-D1	-1.72	0.00E+00	↑	rho GTPase-activating protein 25 isoform X1 [Bison bison bison]
ENSP00000186436-D1	-1.72	6.05E-150	↑	transmembrane protein 131 isoform X1 [B. taurus]
ENSP00000289166-D1	-1.72	8.63E-16	↑	protein FAM46B [B. mutus]
ENSP00000302640-D1	-1.72	1.69E-43	↑	nucleolar transcription factor 1 isoform X1 [B. mutus]
ENSBTAP00000004035-D1	-1.71	4.71E-104	↑	Sal-like protein 4, partial [B. mutus]
ENSP00000354526-D1	-1.71	0.00E+00	↑	lethal(3)malignant brain tumor-like protein 3 isoform X1 [B. mutus]
ENSP00000356355-D1	-1.71	2.58E-24	↑	serine/threonine-protein kinase Nek7 isoform X1 [B. taurus]
ENSP00000417354-D1	-1.71	4.67E-42	↑	Golgi integral membrane protein 4 isoform X1 [B. mutus]
ENSP00000378624-D2	-1.71	3.85E-277	↑	probable E3 ubiquitin-protein ligase HERC3 [B. mutus]
ENSBTAP00000023642-D1	-1.71	3.15E-196	↑	3-hydroxyacyl-CoA dehydrogenase type-2, partial [B. mutus]
ENSP00000357048-D1	-1.71	5.23E-245	↑	coatomer subunit alpha isoform X1 [B. mutus]
ENSBTAP00000000361-D1	-1.71	2.43E-112	↑	ankyrin repeat and SOCS box protein 8 [B. taurus]
ENSBTAP00000011370-D1	-1.71	7.70E-66	↑	testis-expressed protein 19.2 [B. taurus]
ENSP00000378917-D1	-1.71	8.76E-37	↑	ataxin-2-like protein isoform X1 [B. mutus]
ENSBTAP00000033789-D1	-1.71	1.88E-41	↑	Pogo transposable element with ZNF domain [B. mutus]
ENSP00000401645-D4	-1.70	8.33E-111	↑	Serpin B6 [B. mutus]
ENSP00000257934-D1	-1.70	3.77E-22	↑	Separin [B. mutus]
ENSBTAP00000026669-D1	-1.70	1.62E-45	↑	5-formyltetrahydrofolate cyclo-ligase [B. mutus]
ENSBTAP00000009526-D1	-1.70	3.91E-70	↑	zinc finger CCHC domain-containing protein 4 isoform X1 [B. mutus]
ENSBTAP00000051208-D3	-1.70	1.38E-19	↑	hypothetical protein M91_00154, partial [B. mutus]
ENSBTAP00000015248-D2	-1.70	2.63E-18	↑	Myosin regulatory light chain 2, smooth muscle major isoform, partial [B. mutus]
ENSBTAP00000024812-D1	-1.70	0.00E+00	↑	V-type proton ATPase subunit B, brain isoform, partial [B. mutus]
ENSBTAP00000026432-D1	-1.70	1.99E-20	↑	SHC-transforming protein 1 isoform X3 [B. taurus]
ENSP00000252992-D1	-1.70	1.98E-271	↑	Coiled-coil domain-containing protein 21, partial [B. mutus]
ENSBTAP00000026386-D1	-1.69	0.00E+00	↑	DNA-directed RNA polymerase III subunit RPC10 [Pantholops hodgsonii]
ENSBTAP00000027246-D1	-1.69	8.36E-39	↑	Thimet oligopeptidase, partial [B. mutus]
ENSBTAP00000021483-D1	-1.69	2.14E-152	↑	Liprin-beta-2, partial [B. mutus]
ENSBTAP00000013990-D1	-1.69	2.54E-11	↑	SH3 domain-binding glutamic acid-rich-like protein [B. taurus]
ENSP00000303802-D1	-1.69	6.09E-24	↑	GTP-binding protein 8 isoform X4 [B. taurus]
ENSP00000257899-D1	-1.69	1.71E-121	↑	biogenesis of lysosome-related organelles complex 1 subunit 1-like isoform X7 [Macaca fascicularis]
ENSBTAP00000041558-D1	-1.69	4.63E-74	↑	tRNA-splicing endonuclease subunit Sen2 isoform X1 [B. mutus]
ENSBTAP00000035516-D1	-1.69	4.73E-62	↑	Isochorismatase domain-containing protein 1, partial [B. mutus]
ENSP00000370430-D1	-1.69	6.85E-62	↑	patatin-like phospholipase domain-containing protein 4-like [B. mutus]
ENSBTAP00000013338-D1	-1.69	1.86E-08	↑	zinc finger and BTB domain-containing protein 24 isoform X1 [B. mutus]
ENSP00000263826-D1	-1.69	0.00E+00	↑	RAC-gamma serine/threonine-protein kinase, partial [Heterocephalus glaber]
ENSP00000356363-D1	-1.68	1.20E-34	↑	uncharacterized protein C1orf53-like [Ovis aries musimon]
ENSP00000374171-D1	-1.68	1.54E-17	↑	Protein TANC2, partial [B. mutus]
ENSP00000360425-D1	-1.68	2.20E-41	↑	Calcipressin-2, partial [B. mutus]

ENSP00000377914-D1	-1.68	6.73E-156	↑	anamorsin [B. mutus]
ENSP00000259569-D1	-1.68	5.83E-50	↑	TPA: karyopherin beta 3-like [B. taurus]
ENSBTAP00000014549-D1	-1.68	8.19E-139	↑	lysosome membrane protein 2 isoform X1 [B. mutus]
ENSBTAP00000018283-D9	-1.68	1.11E-48	↑	Protein BEX3, partial [B. mutus]
ENSBTAP00000047997-D1	-1.68	7.21E-132	↑	hypothetical protein M91_04367, partial [B. mutus]
ENSBTAP00000013663-D3	-1.67	5.33E-35	↑	Moesin, partial [B. mutus]
ENSP00000238018-D1	-1.67	7.71E-35	↑	guanine deaminase [B. mutus]
ENSP00000364699-D1	-1.67	3.17E-89	↑	Succinate dehydrogenase [ubiquinone] cytochrome b small subunit, mitochondrial [B. mutus]
ENSBTAP00000011643-D1	-1.67	6.77E-20	↑	junctophilin-1 isoform X2 [Ovis aries musimon]
ENSP00000348081-D1	-1.67	1.49E-05	↑	E3 ubiquitin-protein ligase MIB2 [B. mutus]
ENSP00000334974-D1	-1.66	7.67E-12	↑	hypothetical protein M91_13831, partial [B. mutus]
ENSBTAP00000009138-D1	-1.66	6.57E-62	↑	copine-1 [B. mutus]
ENSBTAP00000031486-D15	-1.66	9.10E-28	↑	hypothetical protein M91_13651, partial [B. mutus]
ENSBTAP00000005261-D1	-1.66	4.31E-67	↑	ligand-dependent corepressor isoform X1 [Camelus ferus]
ENSBTAP00000010477-D1	-1.66	3.85E-09	↑	Putative phospholipid-transporting ATPase IIB [B. mutus]
ENSP00000411132-D5	-1.66	5.75E-13	↑	hypothetical protein M91_15431, partial [B. mutus]
ENSP00000283628-D2	-1.66	9.10E-27	↑	Alpha-globin transcription factor CP2 [B. mutus]
ENSP00000309117-D1	-1.66	3.43E-243	↑	Fox-1-like protein A, partial [B. mutus]
ENSBTAP00000004559-D1	-1.66	1.71E-16	↑	glutathione synthetase [B. mutus]
ENSP00000355812-D1	-1.66	1.17E-245	↑	FGFR1 oncogene partner, partial [B. mutus]
ENSBTAP00000032458-D1	-1.65	3.26E-115	↑	protein BANP isoform X1 [Bubalus bubalis]
ENSBTAP00000021480-D1	-1.65	6.25E-55	↑	zinc finger protein 608-like [B. mutus]
ENSBTAP00000016976-D1	-1.65	3.91E-20	↑	Ras-related protein Rab-7b [B. mutus]
ENSBTAP00000053756-D1	-1.65	1.79E-108	↑	phosphodiesterase 3A, cGMP-inhibited [B. mutus]
ENSP00000332979-D1	-1.65	6.80E-17	↑	Beta-secretase 2, partial [B. mutus]
ENSBTAP00000028236-D1	-1.65	3.57E-17	↑	39S ribosomal protein L16, mitochondrial [B. mutus]
ENSP00000398026-D1	-1.65	8.62E-60	↑	Beta-hexosaminidase subunit alpha [B. mutus]
ENSP00000226440-D2	-1.65	1.42E-18	↑	ubiquitin carboxyl-terminal hydrolase 12 [Bubalus bubalis]
ENSP00000281527-D1	-1.65	7.47E-19	↑	Cathepsin O, partial [B. mutus]
ENSBTAP00000051032-D1	-1.65	8.93E-53	↑	Cullin-2, partial [B. mutus]
ENSBTAP00000027126-D1	-1.65	1.55E-224	↑	GON-4-like protein [B. mutus]
ENSP00000294053-D1	-1.65	6.23E-22	↑	caseinolytic peptidase B protein homolog isoform X1 [B. mutus]
ENSP00000295619-D1	-1.65	2.91E-09	↑	prokineticin-2 isoform X1 [B. mutus]
ENSP00000257264-D1	-1.65	5.87E-132	↑	transcobalamin-1 [B. mutus]
ENSBTAP00000022699-D1	-1.64	0.00E+00	↑	Band 4.1-like protein 4B, partial [B. mutus]
ENSP00000240731-D4	-1.64	4.37E-28	↑	zinc finger protein 211 [B. taurus]
yakG034077	-1.64	3.12E-48	↑	Deubiquitinating protein VCIP135 [B. mutus]
ENSP00000404853-D1	-1.64	1.64E-48	↑	Exportin-7, partial [B. mutus]
ENSBTAP00000009395-D1	-1.64	3.34E-69	↑	WD repeat and SOCS box-containing protein 2 isoform X1 [B. mutus]
ENSBTAP00000012526-D1	-1.64	1.11E-119	↑	E3 ubiquitin-protein ligase TRIM23 [B. taurus]
ENSP00000345895-D1	-1.64	1.52E-186	↑	nuclear pore complex protein Nup50 [B. mutus]
ENSP00000372689-D1	-1.64	1.30E-115	↑	Cohesin subunit SA-1, partial [B. mutus]
ENSBTAP00000014975-D1	-1.64	1.88E-23	↑	Sorting nexin-31 [B. mutus]
ENSP00000306522-D1	-1.64	1.88E-23	↑	Calmodulin-binding transcription activator 1, partial [B. mutus]
ENSP00000355924-D1	-1.64	3.66E-69	↑	SET and MYND domain-containing protein 2, partial [B. mutus]
ENSP00000328203-D1	-1.64	7.21E-37	↑	TRAF-interacting protein [B. mutus]
ENSBTAP00000052934-D1	-1.64	1.71E-20	↑	WAP four-disulfide core domain protein 3 [B. mutus]
ENSP00000389932-D1	-1.63	0.00E+00	↑	double-stranded RNA-binding protein Staufen homolog 2 [B. mutus]
ENSP00000350267-D1	-1.63	3.04E-297	↑	Bromodomain and PHD finger-containing protein 3 [B. mutus]
ENSBTAP00000023088-D1	-1.63	1.40E-115	↑	CCR4-NOT transcription complex subunit 6 [B. taurus]
ENSP00000292205-D1	-1.63	1.33E-120	↑	double-stranded RNA-specific adenosine deaminase isoform X1 [Bison bison bison]
ENSP00000258341-D1	-1.63	8.32E-81	↑	Laminin subunit gamma-1, partial [B. mutus]
ENSBTAP00000053741-D1	-1.63	2.70E-234	↑	ankyrin repeat and IBR domain-containing protein 1 isoform X1 [Bison bison bison]
ENSBTAP00000042066-D1	-1.63	8.60E-155	↑	protein FAM126B isoform X1 [Bison bison bison]
ENSP00000405943-D1	-1.63	1.02E-83	↑	protein prenyltransferase alpha subunit repeat-containing protein 1 [B. taurus]

ENSP00000347836-D1	-1.63	2.16E-61	↑	ubiquitin-conjugating enzyme E2 H isoform 1 [Homo sapiens]
ENSP00000368848-D1	-1.63	2.33E-32	↑	Synphilin-1, partial [B. mutus]
ENSBTAP00000040060-D1	-1.63	8.23E-25	↑	Adiponectin receptor protein 1, partial [B. mutus]
ENSP00000313600-D1	-1.63	6.87E-14	↑	probable phospholipid-transporting ATPase VB [B. mutus]
ENSBTAP00000025999-D1	-1.63	1.65E-04	↑	zinc finger protein 513 [B. taurus]
ENSP00000298130-D1	-1.63	5.95E-03	↑	serine palmitoyltransferase small subunit A-like [Myotis lucifugus]
ENSP00000393349-D36	-1.63	1.38E-02	↑	hypothetical protein M91_20944, partial [B. mutus]
ENSBTAP00000008370-D3	-1.63	1.38E-02	↑	uncharacterized protein LOC102273301 [B. mutus]
ENSP00000250156-D1	-1.63	1.38E-02	↑	rRNA methyltransferase 1, mitochondrial [Bison bison bison]
ENSBTAP00000041247-D4	-1.63	1.38E-02	↑	hypothetical protein M91_09280, partial [B. mutus]
ENSP00000361536-D1	-1.63	9.24E-33	↑	elongation of very long chain fatty acids protein 1 isoform X2 [B. taurus]
ENSP00000311449-D1	-1.63	3.35E-32	↑	ras-related protein Rab-6B isoform X1 [B. mutus]
ENSP00000301744-D1	-1.63	2.49E-26	↑	TPA: zinc finger and SCAN domain containing 2-like [B. taurus]
ENSP00000264515-D1	-1.63	0.00E+00	↑	retinoblastoma-binding protein 5 isoform X3 [Microcebus murinus]
ENSP00000362264-D1	-1.63	7.96E-65	↑	Apolipoprotein O-like protein, partial [B. mutus]
ENSBTAP00000033778-D1	-1.62	7.47E-151	↑	Peroxisome biogenesis factor 1, partial [B. mutus]
ENSP00000372035-D2	-1.62	4.51E-37	↑	serine/threonine-protein kinase LATS2 isoform X1 [B. taurus]
ENSBTAP00000040330-D1	-1.62	2.13E-35	↑	Dolichyl-diphosphooligosaccharide--protein glycosyltransferase 48 kDa subunit, partial [B. mutus]
ENSP00000388916-D8	-1.62	2.24E-17	↑	60S ribosomal protein L21-like isoform 1 [Macaca mulatta]
ENSBTAP00000001489-D2	-1.62	5.64E-16	↑	Ankyrin repeat domain-containing protein 13D, partial [B. mutus]
ENSBTAP00000028680-D1	-1.62	1.24E-145	↑	Calcium signal-modulating cyclophilin ligand, partial [B. mutus]
ENSBTAP00000026700-D1	-1.62	2.88E-91	↑	Protein FAM13B [B. mutus]
ENSBTAP00000002831-D1	-1.62	2.22E-120	↑	RING-box protein 2 isoform X1 [Otolemur garnettii]
ENSP00000352208-D1	-1.62	1.40E-151	↑	myoferlin [B. mutus]
ENSBTAP00000014794-D1	-1.62	4.45E-75	↑	cholinesterase [B. mutus]
ENSBTAP00000037030-D1	-1.62	1.79E-184	↑	Epithelial splicing regulatory protein 1, partial [B. mutus]
ENSP00000385478-D1	-1.62	8.30E-128	↑	isoprenoid synthase domain-containing protein [B. mutus]
ENSBTAP00000008902-D1	-1.62	2.89E-211	↑	Leucyl-tRNA synthetase, cytoplasmic, partial [B. mutus]
ENSBTAP00000041529-D1	-1.62	2.36E-10	↑	EP300-interacting inhibitor of differentiation 1-like [B. mutus]
ENSP00000256637-D1	-1.62	8.87E-18	↑	Sortilin, partial [B. mutus]
ENSP00000310180-D1	-1.62	2.46E-24	↑	Transmembrane protein 110 [B. mutus]
ENSP00000339145-D1	-1.62	1.83E-123	↑	Ribosomal RNA processing protein 1-like protein B, partial [B. mutus]
ENSP00000385122-D1	-1.62	8.66E-10	↑	synaptonemal complex central element protein 3 [B. taurus]
ENSP00000329127-D1	-1.62	2.15E-120	↑	protein kinase C eta type [B. mutus]
ENSP00000412500-D2	-1.62	6.18E-127	↑	unconventional myosin-VI isoform X1 [Bison bison bison]
ENSBTAP00000015441-D1	-1.61	0.00E+00	↑	calcium-transporting ATPase type 2C member 1 [B. mutus]
ENSP00000334918-D1	-1.61	8.30E-34	↑	Constitutive coactivator of PPAR-gamma-like protein 1, partial [B. mutus]
ENSBTAP00000007289-D1	-1.61	0.00E+00	↑	Epidermal growth factor receptor substrate 15, partial [B. mutus]
ENSBTAP00000028077-D1	-1.61	7.48E-14	↑	transmembrane protein 125 [B. mutus]
ENSP00000418803-D1	-1.61	1.28E-23	↑	translocase of inner mitochondrial membrane domain-containing protein 1 [B. mutus]
ENSBTAP00000018160-D1	-1.61	5.14E-30	↑	uncharacterized protein C3orf26 homolog [B. mutus]
ENSBTAP00000015199-D1	-1.61	9.53E-86	↑	puromycin-sensitive aminopeptidase [Bison bison bison]
ENSBTAP00000017579-D1	-1.61	6.82E-50	↑	Putative cation-transporting ATPase 13A3 [B. mutus]
ENSBTAP00000008132-D2	-1.61	0.00E+00	↑	actin, cytoplasmic 2 isoform X1 [Colobus angolensis palliatus]
ENSP00000221448-D1	-1.61	1.15E-06	↑	U1 small nuclear ribonucleoprotein 70 kDa-like [B. mutus]
ENSBTAP00000053475-D1	-1.61	5.53E-157	↑	scm-like with four MBT domains protein 1 [B. taurus]
ENSBTAP00000029938-D1	-1.60	1.32E-82	↑	nephronectin isoform X4 [B. mutus]
ENSP00000414859-D1	-1.60	7.03E-103	↑	CUGBP Elav-like family member 1 isoform X1 [B. mutus]
ENSBTAP00000018997-D1	-1.60	8.98E-85	↑	Putative helicase MOV-10, partial [B. mutus]
ENSP00000413971-D1	-1.60	9.96E-12	↑	Transmembrane protein 233, partial [B. mutus]
ENSP00000362424-D1	-1.60	7.09E-47	↑	TRAF3-interacting protein 1, partial [B. mutus]
ENSP00000386190-D1	-1.59	1.34E-10	↑	hypothetical protein M91_00186, partial [B. mutus]
ENSBTAP00000025870-D1	-1.59	1.73E-77	↑	NADH dehydrogenase [ubiquinone] iron-sulfur protein 7, mitochondrial, partial [B. mutus]
ENSP00000379796-D1	-1.59	0.00E+00	↑	Tripartite motif-containing protein 39 [B. mutus]
ENSBTAP00000052270-D1	-1.59	6.25E-05	↑	cyclin-dependent kinase 5 activator 1 [B. mutus]

ENSBTAP00000016866-D3	-1.59	7.94E-111	↑	Glutathione S-transferase Mu 1, partial [B. mutus]
ENSP00000396519-D1	-1.59	2.14E-23	↑	engulfment and cell motility protein 2 isoform X1 [Capra hircus]
ENSP00000265094-D1	-1.59	1.46E-50	↑	KIAA0696 protein [Homo sapiens]
ENSBTAP00000041817-D1	-1.59	7.16E-36	↑	Selenoprotein M [B. mutus]
ENSBTAP00000010169-D1	-1.59	1.31E-156	↑	Protein EFR3-like protein A, partial [B. mutus]
ENSBTAP00000052520-D51	-1.59	5.81E-91	↑	Zinc finger protein 624, partial [B. mutus]
ENSBTAP00000050302-D1	-1.59	6.47E-20	↑	39S ribosomal protein L38, mitochondrial [B. mutus]
ENSP00000371607-D1	-1.59	1.23E-19	↑	Mitochondrial intermediate peptidase, partial [B. mutus]
ENSP00000386331-D1	-1.58	8.60E-07	↑	Myosin-VIIa, partial [B. mutus]
ENSP00000359356-D1	-1.58	2.67E-128	↑	ecotropic viral integration site 5 protein homolog [B. mutus]
ENSP00000378669-D1	-1.58	3.30E-113	↑	fructose-bisphosphate aldolase A isoform X2 [B. taurus]
ENSP00000357600-D2	-1.58	3.70E-21	↑	Testis-specific Y-encoded-like protein 1, partial [B. mutus]
ENSBTAP00000003589-D1	-1.58	7.46E-111	↑	guanine nucleotide-binding protein subunit beta-4 isoform X1 [Oryctolagus cuniculus]
ENSBTAP00000053200-D1	-1.58	1.27E-08	↑	peroxisomal bifunctional enzyme [B. mutus]
yakG018989	-1.58	1.01E-56	↑	Ankyrin repeat and FYVE domain-containing protein 1 [B. mutus]
ENSP00000359073-D1	-1.58	1.25E-133	↑	guanine nucleotide exchange factor VAV3 [B. mutus]
ENSP00000364382-D1	-1.58	1.49E-83	↑	thioredoxin-like protein AAED1, partial [Bison bison bison]
ENSP00000383700-D1	-1.58	3.33E-33	↑	arginine-glutamic acid dipeptide repeats protein isoform X1 [B. taurus]
ENSBTAP00000000074-D1	-1.58	2.49E-55	↑	Stromal cell-derived factor 2-like protein 1 [B. mutus]
ENSBTAP00000013737-D1	-1.58	5.62E-12	↑	Myosin-9, partial [B. mutus]
ENSP00000307939-D1	-1.58	0.00E+00	↑	GRIP and coiled-coil domain-containing protein 2, partial [B. mutus]
ENSBTAP00000026905-D1	-1.58	1.35E-89	↑	corticotropin-releasing factor-binding protein precursor [B. taurus]
ENSP00000268138-D1	-1.58	2.85E-69	↑	Treslin [B. mutus]
ENSP00000365071-D1	-1.58	1.83E-97	↑	G patch domain and ankyrin repeat-containing protein 1 [B. taurus]
ENSP00000360200-D1	-1.58	0.00E+00	↑	inaD-like protein [B. mutus]
ENSBTAP00000012221-D1	-1.58	5.53E-52	↑	regulator of G-protein signaling 17 isoform X5 [Ovis aries]
ENSP00000345808-D1	-1.58	0.00E+00	↑	rho GTPase-activating protein 12 isoform X1 [Bison bison bison]
yakG021684	-1.58	6.05E-13	↑	Calpain small subunit 1 [B. mutus]
ENSP00000318177-D1	-1.58	6.05E-13	↑	far upstream element-binding protein 3 [B. taurus]
ENSP00000371546-D1	-1.57	1.33E-19	↑	DEP domain-containing protein 5, partial [B. mutus]
ENSP00000300013-D1	-1.57	4.41E-44	↑	Protein SAAL1 [B. mutus]
ENSP00000356319-D1	-1.57	8.30E-69	↑	Kinesin-like protein KIF14, partial [B. mutus]
ENSBTAP00000022411-D1	-1.57	0.00E+00	↑	14-3-3 protein beta/alpha, partial [B. mutus]
ENSP00000390342-D1	-1.57	6.12E-170	↑	hypothetical protein M91_18721, partial [B. mutus]
ENSBTAP00000027964-D1	-1.57	7.52E-11	↑	RuvB-like 1, partial [B. mutus]
ENSBTAP00000001474-D1	-1.57	3.65E-149	↑	serine/threonine-protein kinase D3 [B. mutus]
ENSP00000317614-D1	-1.57	9.45E-90	↑	zinc finger protein 518B [B. mutus]
ENSP00000216807-D1	-1.57	2.94E-153	↑	Breast cancer metastasis-suppressor 1-like protein [B. mutus]
ENSBTAP00000020053-D1	-1.57	5.60E-38	↑	Transmembrane protease serine 7 [B. mutus]
ENSBTAP00000048960-D14	-1.57	2.23E-71	↑	hypothetical protein M91_03925, partial [B. mutus]
ENSBTAP00000044680-D1	-1.57	1.21E-05	↑	Putative hexokinase HKDC1, partial [B. mutus]
ENSBTAP00000025683-D1	-1.57	6.49E-60	↑	protein kinase C and casein kinase substrate in neurons protein 2 [B. taurus]
ENSBTAP00000035395-D1	-1.57	5.69E-46	↑	Glia maturation factor beta, partial [B. mutus]
ENSBTAP00000023194-D1	-1.57	4.21E-111	↑	calcipressin-3 isoform X1 [B. mutus]
ENSBTAP00000003472-D1	-1.57	1.81E-08	↑	6-phosphofructokinase type C [B. mutus]
ENSP00000383911-D1	-1.57	6.73E-23	↑	ubiquitin-associated domain-containing protein 2 [B. mutus]
ENSP00000356814-D1	-1.56	3.74E-76	↑	DDB1- and CUL4-associated factor 6 isoform X1 [B. taurus]
yakG028929	-1.56	4.79E-63	↑	Serine/threonine-protein kinase Kist [B. mutus]
yakG044149	-1.56	2.36E-05	↑	Ras-related protein Rab-4A [B. mutus]
ENSBTAP00000003777-D1	-1.56	2.08E-68	↑	guanine nucleotide-binding protein G(I)/G(S)/G(O) subunit gamma-4 [B. taurus]
ENSBTAP00000000337-D1	-1.56	0.00E+00	↑	Meiosis-specific nuclear structural protein 1, partial [B. mutus]
ENSP00000384198-D1	-1.56	1.35E-184	↑	INO80 complex subunit D [B. mutus]
ENSP00000403265-D1	-1.56	1.22E-107	↑	Pyruvate kinase isozymes M1/M2, partial [B. mutus]
ENSP00000229270-D1	-1.56	1.17E-79	↑	triosephosphate isomerase [B. mutus]
ENSBTAP00000053340-D1	-1.56	6.85E-303	↑	Transcription factor COE1, partial [B. mutus]

ENSBTAP00000012819-D1	-1.56	1.36E-61	↑	Protein RRP5-like protein, partial [B. mutus]
ENSP00000072644-D1	-1.56	1.49E-66	↑	protein YIPF1 isoform X1 [B. mutus]
ENSP00000378518-D1	-1.56	2.78E-31	↑	RecName: Full=Ubiquitin-like protein 7 [B. taurus]
ENSP00000379795-D1	-1.56	4.60E-05	↑	CKLF-like MARVEL transmembrane domain-containing protein 7, partial [B. mutus]
ENSBTAP00000051282-D1	-1.55	4.30E-16	↑	Magnesium-dependent phosphatase 1, partial [B. mutus]
ENSBTAP00000016988-D1	-1.55	0.00E+00	↑	rac GTPase-activating protein 1 [B. mutus]
ENSBTAP00000002513-D1	-1.55	8.06E-11	↑	Carnitine O-acetyltransferase [B. mutus]
ENSP00000315334-D1	-1.55	3.52E-37	↑	Rho GTPase-activating protein 24, partial [B. mutus]
ENSBTAP00000018238-D1	-1.55	2.12E-23	↑	rabenosyn-5 [B. taurus]
ENSP00000223641-D3	-1.55	8.99E-29	↑	protein transport protein Sec61 subunit beta isoform X1 [Pantholops hodgsonii]
ENSP00000394510-D1	-1.55	2.59E-08	↑	proline-rich protein 12 [B. mutus]
ENSP00000350558-D1	-1.55	6.02E-95	↑	CDK5 regulatory subunit-associated protein 1 [B. mutus]
ENSP00000315949-D1	-1.55	1.07E-30	↑	Homeobox protein Hox-D8, partial [B. mutus]
yakG015983	-1.55	4.19E-35	↑	AMY-1-associating protein expressed in testis 1 [B. mutus]
ENSP00000324913-D24	-1.55	3.78E-18	↑	olfactory receptor 4C45-like [B. mutus]
ENSBTAP00000053705-D1	-1.55	2.75E-09	↑	Protein Daple, partial [B. mutus]
ENSBTAP00000052063-D1	-1.54	1.32E-54	↑	Biotin--protein ligase, partial [B. mutus]
ENSP00000345001-D1	-1.54	9.04E-06	↑	CREB-regulated transcription coactivator 1 [B. mutus]
ENSP00000261845-D1	-1.54	0.00E+00	↑	mitogen-activated protein kinase 6 isoform X2 [B. taurus]
ENSP00000356297-D1	-1.54	7.55E-262	↑	Pleckstrin-like protein domain-containing family G member 1 [B. mutus]
ENSBTAP00000040521-D1	-1.54	5.75E-45	↑	Putative ATP-dependent RNA helicase DDX31, partial [B. mutus]
ENSBTAP00000014005-D1	-1.54	0.00E+00	↑	Structural maintenance of chromosomes protein 1B, partial [B. mutus]
ENSP00000345964-D1	-1.54	1.42E-43	↑	VPS10 domain-containing receptor SorCS1, partial [B. mutus]
ENSBTAP00000023556-D1	-1.54	2.54E-252	↑	WASP homolog-associated protein with actin, membranes and microtubules [B. mutus]
ENSBTAP00000022529-D1	-1.54	3.22E-45	↑	uncharacterized protein C2orf42 homolog isoform X1 [B. mutus]
ENSP00000357801-D1	-1.54	1.78E-06	↑	S100 calcium binding protein A10, isoform CRA_b, partial [Homo sapiens]
ENSBTAP00000016000-D1	-1.54	1.32E-59	↑	zinc finger protein 473 [B. mutus]
ENSP00000246112-D1	-1.54	4.69E-66	↑	Transducin-like enhancer protein 6, partial [B. mutus]
ENSP00000300249-D1	-1.53	0.00E+00	↑	microtubule-associated protein RP/EB family member 2 isoform X1 [Heterocephalus glaber]
ENSP00000362803-D1	-1.53	2.80E-46	↑	peptidyl-prolyl cis-trans isomerase-like 1 [Homo sapiens]
ENSP00000314813-D1	-1.53	0.00E+00	↑	RecName: Full=Ornithine decarboxylase antizyme 1; Short=ODC-Az [B. taurus]
ENSP00000413645-D1	-1.53	1.45E-28	↑	hypothetical protein M91_14639 [B. mutus]
ENSP00000256682-D3	-1.53	1.24E-15	↑	ADP-ribosylation factor 3 isoform X1 [Chlorocebus sabaeus]
ENSP00000330813-D1	-1.53	2.98E-81	↑	BRCA1-associated protein [B. mutus]
ENSBTAP00000033002-D2	-1.53	2.18E-14	↑	hypothetical protein EGM_16376 [Macaca fascicularis]
ENSP00000330276-D1	-1.53	1.24E-147	↑	Tyrosine-protein phosphatase non-receptor type 21 [B. mutus]
ENSBTAP00000013131-D1	-1.53	1.31E-186	↑	Paired box protein Pax-3, partial [B. mutus]
ENSBTAP00000037915-D1	-1.53	0.00E+00	↑	RING finger protein 146 [B. mutus]
ENSP00000239882-D1	-1.53	1.19E-233	↑	ETS-related transcription factor Elf-1, partial [B. mutus]
ENSP00000370745-D1	-1.53	2.77E-103	↑	ATP-dependent RNA helicase DDX1 [B. taurus]
ENSBTAP00000027504-D1	-1.53	5.98E-22	↑	metalloproteinase inhibitor 3-like isoform X1 [B. mutus]
ENSBTAP00000030209-D1	-1.53	3.29E-40	↑	zinc finger and BTB domain-containing protein 44 isoform X1 [Bison bison bison]
ENSBTAP00000009985-D1	-1.53	2.69E-195	↑	inhibitor of nuclear factor kappa-B kinase subunit alpha isoform X1 [Bison bison bison]
ENSBTAP00000015998-D1	-1.53	0.00E+00	↑	1-aminocyclopropane-1-carboxylate synthase-like protein 2, partial [B. mutus]
ENSBTAP00000025152-D1	-1.53	6.21E-266	↑	26S proteasome non-ATPase regulatory subunit 11 [Homo sapiens]
ENSP00000299045-D1	-1.53	2.13E-89	↑	T-complex protein 11-like protein 2 [B. mutus]
ENSP00000346634-D1	-1.53	8.31E-127	↑	thyroid hormone receptor-associated protein 3 isoform X1 [Capra hircus]
ENSP00000371155-D1	-1.53	2.18E-150	↑	nucleoporin p58/p45 [Pantholops hodgsonii]
ENSP00000261441-D1	-1.53	8.21E-93	↑	round spermatid basic protein 1 [B. mutus]
ENSBTAP00000023484-D1	-1.52	3.52E-04	↑	Protein CDV3-like protein, partial [B. mutus]
ENSP00000355637-D1	-1.52	0.00E+00	↑	ATP-binding cassette sub-family B member 10, mitochondrial, partial [B. mutus]
ENSBTAP00000038128-D1	-1.52	6.33E-22	↑	caspase-8 [B. mutus]
ENSP00000303909-D1	-1.52	4.60E-49	↑	Active breakpoint cluster region-related protein, partial [B. mutus]
ENSBTAP00000006505-D1	-1.52	1.33E-39	↑	Bardet-Biedl syndrome 7 protein [B. taurus]
ENSP00000340510-D1	-1.52	1.12E-13	↑	periplakin [Pantholops hodgsonii]

ENSP00000376352-D1	-1.52	3.24E-58	↑	[Pyruvate dehydrogenase [lipoamide]] kinase isozyme 1, mitochondrial, partial [B. mutus]
ENSBTAP00000007945-D1	-1.52	5.10E-12	↑	dephospho-CoA kinase domain-containing protein [B. mutus]
yakG043161	-1.52	3.32E-94	↑	Zinc finger CCCH domain-containing protein 14 [B. mutus]
ENSP00000347325-D1	-1.52	2.57E-224	↑	Histone-lysine N-methyltransferase MLL3, partial [B. mutus]
ENSBTAP00000019607-D1	-1.52	1.98E-33	↑	Putative ribosomal RNA methyltransferase NOP2 [B. mutus]
ENSBTAP00000018490-D1	-1.52	0.00E+00	↑	WD repeat-containing protein 52, partial [B. mutus]
ENSBTAP00000024133-D1	-1.52	3.55E-29	↑	vacuolar protein sorting-associated protein 53 homolog [B. taurus]
ENSBTAP00000007218-D1	-1.51	6.92E-36	↑	WD repeat-containing protein 36, partial [B. mutus]
ENSP00000267859-D1	-1.51	5.29E-59	↑	BCL2/adenovirus E1B 19 kDa protein-interacting protein 2 [B. mutus]
ENSBTAP00000006219-D1	-1.51	2.17E-09	↑	Leucine-rich repeat-containing protein 47, partial [B. mutus]
ENSBTAP00000009631-D1	-1.51	7.93E-17	↑	protein jagged-1 [Bubalus bubalis]
ENSBTAP00000024107-D1	-1.51	1.70E-60	↑	phosphatidylethanolamine binding protein 1 [B. mutus]
ENSBTAP00000047883-D15	-1.51	4.45E-10	↑	hypothetical protein M91_11302, partial [B. mutus]
ENSBTAP00000024022-D2	-1.51	2.54E-06	↑	UDP-GlcNAc:betaGal beta-1,3-N-acetylglucosaminyltransferase 2 [B. mutus]
ENSBTAP00000008739-D1	-1.51	9.09E-11	↑	insulin-like peptide INSL6 [B. mutus]
ENSP00000382863-D1	-1.51	4.88E-32	↑	Chromodomain-helicase-DNA-binding protein 8 [B. mutus]
ENSP00000322300-D1	-1.51	0.00E+00	↑	Transmembrane and TPR repeat-containing protein 2, partial [B. mutus]
ENSBTAP00000021518-D1	-1.51	0.00E+00	↑	Putative lysosomal cobalamin transporter, partial [B. mutus]
ENSBTAP00000009058-D1	-1.51	0.00E+00	↑	HMG box transcription factor BBX [B. mutus]
ENSBTAP00000040456-D2	-1.51	1.55E-95	↑	Tubulin gamma-2 chain, partial [B. mutus]
ENSBTAP00000022956-D1	-1.51	1.96E-116	↑	Coilin, partial [B. mutus]
ENSBTAP00000027443-D1	-1.50	1.87E-18	↑	OCIA domain-containing protein 2 [B. taurus]
ENSBTAP00000015650-D1	-1.50	3.31E-10	↑	uncharacterized protein CXorf38 homolog [B. mutus]
ENSP00000367394-D1	-1.50	7.02E-04	↑	rho guanine nucleotide exchange factor 1 isoform X6 [B. taurus]
ENSP00000290075-D1	-1.50	4.20E-03	↑	mitoferrin-1 [B. mutus]
ENSBTAP00000053275-D1	-1.50	1.80E-40	↑	Cell division protein kinase 14, partial [B. mutus]
ENSBTAP00000033902-D1	-1.50	0.00E+00	↑	ras-specific guanine nucleotide-releasing factor RalGPS2 isoform X1 [Ovis aries]
ENSBTAP00000011469-D1	-1.50	1.88E-34	↑	interferon-induced, double-stranded RNA-activated protein kinase [B. mutus]
ENSBTAP00000034997-D1	-1.50	1.29E-10	↑	Protein spinster-like protein 3, partial [B. mutus]
ENSBTAP00000017428-D1	-1.50	7.47E-75	↑	ribonucleoside-diphosphate reductase large subunit [B. mutus]
ENSP00000320940-D1	-1.50	5.09E-108	↑	nuclear receptor coactivator 1 isoform X1 [B. mutus]
ENSBTAP00000003291-D1	-1.50	2.47E-58	↑	hypothetical protein M91_10077, partial [B. mutus]
ENSP00000360398-D1	-1.50	2.22E-66	↑	mitochondrial uncoupling protein 4 [B. taurus]
ENSBTAP00000013607-D2	-1.50	6.22E-17	↑	Choline/ethanolamine kinase, partial [B. mutus]
ENSBTAP00000015317-D1	-1.50	3.74E-07	↑	TPA: 5'(3')-deoxyribonucleotidase, cytosolic type [B. taurus]
ENSBTAP00000048073-D1	-1.50	1.88E-06	↑	brain protein I3, partial [B. mutus]
ENSBTAP00000045291-D1	-1.50	1.57E-53	↑	otogelin-like protein [Bison bison bison]
ENSP00000344504-D1	-1.49	1.03E-124	↑	histone H1.0-like [Tupaia chinensis]
ENSP00000383623-D1	-1.49	2.84E-28	↑	afadin-like [B. mutus]
ENSBTAP00000022116-D1	-1.49	5.69E-65	↑	wiskott-Aldrich syndrome protein family member 1 [Otolemur garnettii]
ENSBTAP00000004221-D1	-1.49	0.00E+00	↑	Serine/threonine-protein kinase 4, partial [B. mutus]
ENSP00000379709-D1	-1.49	5.97E-44	↑	rho GTPase-activating protein 21 isoform X2 [B. taurus]
ENSBTAP00000011104-D1	-1.49	3.32E-23	↑	AP-5 complex subunit sigma-1 [B. taurus]
ENSBTAP00000002827-D1	-1.49	4.89E-14	↑	KH domain-containing, RNA-binding, signal transduction-associated protein 3 [Capra hircus]
ENSBTAP00000020417-D1	-1.49	5.13E-51	↑	CDC42 small effector protein 1 [Homo sapiens]
ENSP00000254301-D1	-1.49	1.01E-59	↑	galectin-3 [Bison bison bison]
ENSBTAP00000004631-D1	-1.49	5.19E-27	↑	Apoptosis-stimulating of p53 protein 1, partial [B. mutus]
ENSBTAP00000002860-D1	-1.49	9.67E-33	↑	hematological and neurological expressed 1-like protein [B. mutus]
ENSBTAP00000027072-D1	-1.48	1.84E-05	↑	Formin-binding protein 1, partial [B. mutus]
ENSBTAP00000048501-D1	-1.48	1.84E-05	↑	hypothetical protein M91_14422, partial [B. mutus]
ENSBTAP00000004547-D1	-1.48	3.48E-23	↑	Ectonucleotide pyrophosphatase/phosphodiesterase family member 4, partial [B. mutus]
ENSP00000360583-D1	-1.48	1.45E-56	↑	Protein zyg-11-like protein A [B. mutus]
ENSBTAP00000000791-D1	-1.48	2.89E-135	↑	Cyclin-I, partial [B. mutus]
ENSBTAP00000043485-D8	-1.48	1.48E-120	↑	Rho-related GTP-binding protein RhoC, partial [B. mutus]
ENSBTAP00000027531-D1	-1.48	6.03E-19	↑	Ligand of Numb protein X 2, partial [B. mutus]

ENSBTAP00000043907-D1	-1.48	9.83E-297	↑	phosphatidylinositol 4-phosphate 3-kinase C2 domain-containing subunit alpha [B. mutus]
ENSP00000366280-D2	-1.48	3.54E-26	↑	Vesicular integral-membrane protein VIP36 [B. mutus]
ENSBTAP00000015070-D1	-1.48	8.34E-09	↑	kinetochore-associated protein NSL1 homolog [B. taurus]
ENSP00000362330-D1	-1.48	7.14E-73	↑	Chromodomain-helicase-DNA-binding protein 6, partial [B. mutus]
ENSP00000263346-D1	-1.48	3.99E-24	↑	transcription factor 25 [B. mutus]
ENSBTAP00000011112-D1	-1.48	1.51E-116	↑	tRNA wybutosine-synthesizing protein 1-like protein, partial [B. mutus]
ENSP00000329034-D1	-1.48	6.25E-252	↑	atlastin-3 [B. taurus]
ENSP00000265981-D1	-1.48	0.00E+00	↑	RING finger protein 141 [B. taurus]
ENSP00000270288-D1	-1.48	8.46E-16	↑	Wilms tumor protein 1-interacting protein, partial [B. mutus]
ENSBTAP00000007795-D1	-1.48	2.58E-10	↑	Putative Polycomb group protein ASXL1, partial [B. mutus]
yakG004253	-1.48	9.58E-05	↑	hypothetical protein M91_14096 [B. mutus]
ENSP00000386040-D1	-1.48	8.11E-108	↑	ATP-citrate synthase isoform X2 [B. mutus]
ENSBTAP00000028107-D1	-1.48	1.59E-08	↑	protein phosphatase 1, regulatory subunit 3F, partial [B. mutus]
ENSP00000345656-D1	-1.47	2.41E-254	↑	Vesicle-associated membrane protein-associated protein A, partial [B. mutus]
ENSBTAP00000021773-D1	-1.47	1.46E-177	↑	Leucine-rich PPR motif-containing protein, mitochondrial [B. mutus]
ENSP00000403362-D1	-1.47	8.63E-297	↑	AP-2 complex subunit mu-1 [B. mutus]
ENSP00000364416-D1	-1.47	1.84E-31	↑	E3 ubiquitin-protein ligase UBR4 [B. mutus]
ENSP00000352933-D1	-1.47	7.89E-08	↑	Sodium- and chloride-dependent creatine transporter 1, partial [B. mutus]
ENSP00000299001-D1	-1.47	7.89E-08	↑	Piwi-like protein 4, partial [B. mutus]
ENSBTAP00000028687-D1	-1.47	4.77E-238	↑	Signal transducer and activator of transcription 3 [B. mutus]
ENSP00000384369-D1	-1.47	6.48E-28	↑	probable methyltransferase-like protein 15 [B. mutus]
ENSBTAP00000003149-D1	-1.47	2.52E-25	↑	alpha/beta hydrolase domain-containing protein 13 [B. mutus]
ENSP00000299424-D1	-1.47	5.17E-99	↑	transcription initiation factor TFIID subunit 10 [B. mutus]
ENSP00000271526-D1	-1.47	8.09E-30	↑	proline-rich protein PRCC [B. mutus]
ENSBTAP00000025582-D1	-1.47	2.13E-35	↑	Adenylate cyclase type 2, partial [B. mutus]
ENSBTAP00000029271-D1	-1.47	7.42E-11	↑	Epididymal secretory protein E1, partial [B. mutus]
ENSP00000337240-D1	-1.47	4.03E-35	↑	UNC93-like protein MFSD11 isoform X1 [B. taurus]
ENSBTAP00000053708-D1	-1.47	2.26E-18	↑	chromosome unknown open reading frame, human C11orf31 [B. mutus]
ENSBTAP00000027250-D1	-1.47	9.32E-148	↑	Electron transfer flavoprotein subunit beta, partial [B. mutus]
yakG002396	-1.47	3.92E-07	↑	hypothetical protein M91_09874 [B. mutus]
ENSP00000403067-D1	-1.47	4.61E-36	↑	FERM domain-containing protein 5 isoform X1 [Bison bison bison]
ENSBTAP00000008076-D1	-1.47	5.79E-38	↑	Semaphorin-3C, partial [B. mutus]
ENSBTAP00000050185-D1	-1.47	1.37E-13	↑	Zinc finger protein 519, partial [B. mutus]
ENSBTAP00000044966-D1	-1.46	0.00E+00	↑	protein FAM154A [B. mutus]
ENSP00000297770-D1	-1.46	5.14E-06	↑	Carboxypeptidase A6, partial [B. mutus]
ENSP00000415682-D1	-1.46	0.00E+00	↑	RING finger protein 10 [B. taurus]
ENSBTAP00000013449-D2	-1.46	4.59E-41	↑	Serine/threonine-protein kinase PAK 1 [B. mutus]
ENSBTAP00000009038-D1	-1.46	1.35E-52	↑	Ras-GEF domain-containing family member 1A, partial [B. mutus]
ENSBTAP00000023150-D1	-1.46	1.10E-31	↑	Importin subunit alpha-4, partial [B. mutus]
ENSP00000381044-D1	-1.46	5.04E-107	↑	Ubiquitin carboxyl-terminal hydrolase 10, partial [B. mutus]
ENSP00000223145-D1	-1.46	1.76E-21	↑	Glucocorticoid-induced transcript 1 protein, partial [B. mutus]
ENSBTAP00000043518-D1	-1.46	2.65E-172	↑	RecName: Full=Protein phosphatase 1B; AltName: Full=Protein phosphatase 2C isoform beta; Short=PP2C-beta
ENSP00000369162-D1	-1.46	1.07E-233	↑	serine/threonine-protein kinase RIO1 [B. mutus]
ENSP00000324064-D75	-1.46	3.06E-64	↑	Zinc finger protein 436, partial [B. mutus]
ENSBTAP00000020094-D1	-1.46	1.31E-09	↑	V-type proton ATPase subunit e 1 [B. taurus]
ENSBTAP00000011217-D1	-1.46	7.04E-05	↑	F-box only protein 42 [B. mutus]
ENSBTAP00000039739-D1	-1.46	3.77E-63	↑	importin-7 [Heterocephalus glaber]
ENSBTAP00000026836-D1	-1.46	7.88E-80	↑	Angiopoietin-1 receptor, partial [B. mutus]
ENSBTAP00000020765-D1	-1.45	3.65E-13	↑	hypothetical protein M91_00779, partial [B. mutus]
ENSP00000353556-D1	-1.45	2.19E-201	↑	extracellular calcium-sensing receptor [B. mutus]
ENSBTAP00000026396-D1	-1.45	4.09E-31	↑	piggyBac transposable element-derived protein 2 [B. mutus]
ENSP00000313644-D1	-1.45	7.79E-50	↑	Mitogen-activated protein kinase kinase kinase kinase 4, partial [B. mutus]
ENSBTAP00000013238-D1	-1.45	4.60E-48	↑	Ephrin type-A receptor 4 [B. mutus]
ENSBTAP00000026274-D1	-1.45	5.06E-16	↑	DAZ-associated protein 1, partial [B. mutus]
ENSBTAP00000035073-D1	-1.45	5.06E-16	↑	Fanconi-associated nuclease 1, partial [B. mutus]

ENSBTAP00000021144-D1	-1.45	1.56E-169	↑	Nuclear receptor ROR-alpha, partial [B. mutus]
ENSP00000349140-D1	-1.45	8.00E-100	↑	myotrophin [Homo sapiens]
ENSP00000333836-D1	-1.45	1.45E-06	↑	fidgetin isoform X2 [B. taurus]
ENSP00000407944-D1	-1.45	0.00E+00	↑	Protein transport protein Sec31A [B. mutus]
ENSBTAP00000011264-D1	-1.45	5.57E-07	↑	Sal-like protein 1 [B. mutus]
ENSBTAP00000024134-D1	-1.45	2.70E-51	↑	Microtubule-associated protein 2 [B. mutus]
ENSP00000261739-D1	-1.45	1.54E-130	↑	Ankyrin repeat domain-containing protein 13A [B. mutus]
ENSBTAP00000038110-D2	-1.45	4.64E-241	↑	Actin-related protein 3, partial [B. mutus]
ENSBTAP00000017969-D1	-1.44	5.59E-62	↑	protein asunder homolog [B. taurus]
ENSBTAP00000042566-D1	-1.44	7.31E-38	↑	Putative phospholipid-transporting ATPase IH, partial [B. mutus]
ENSBTAP00000014951-D1	-1.44	0.00E+00	↑	unconventional myosin-Ib [B. mutus]
ENSBTAP00000010976-D1	-1.44	3.84E-43	↑	ATP-dependent RNA helicase DHX8 isoform 2 [Equus caballus]
ENSP00000352876-D1	-1.44	4.63E-99	↑	HBeAg-binding protein 2 binding protein A [Homo sapiens]
ENSP00000401596-D1	-1.44	0.00E+00	↑	vasculin isoform X1 [B. mutus]
ENSP00000359685-D1	-1.44	4.43E-249	↑	protein tyrosine phosphatase type IVA 1 [B. taurus]
ENSBTAP00000030993-D1	-1.44	2.95E-43	↑	AFG3-like protein 2 [B. mutus]
ENSP00000389924-D22	-1.44	9.28E-03	↑	MAP7 domain-containing protein 3 [B. mutus]
ENSP00000216027-D1	-1.44	0.00E+00	↑	iron-sulfur cluster co-chaperone protein HscB, mitochondrial [B. mutus]
ENSP00000256190-D1	-1.44	5.87E-136	↑	Myotubularin-related protein 13, partial [B. mutus]
ENSP00000223023-D1	-1.44	1.32E-131	↑	neural Wiskott-Aldrich syndrome protein isoform X2 [Ovis aries musimon]
ENSBTAP00000019656-D1	-1.43	5.40E-179	↑	Guanylate kinase, partial [B. mutus]
ENSBTAP00000003726-D1	-1.43	8.33E-46	↑	Transmembrane protein 183 [B. mutus]
ENSBTAP00000006234-D1	-1.43	3.61E-26	↑	Serine/threonine-protein kinase Nek4 [B. mutus]
ENSP00000366843-D1	-1.43	1.81E-203	↑	Ataxin-2, partial [B. mutus]
ENSP00000345216-D1	-1.43	4.23E-48	↑	Inactive ubiquitin carboxyl-terminal hydrolase 54 [B. mutus]
ENSBTAP00000013997-D1	-1.43	0.00E+00	↑	Membrane-associated guanylate kinase, WW and PDZ domain-containing protein 1 [B. mutus]
ENSP00000370938-D2	-1.43	1.92E-31	↑	Cell division protein kinase 19, partial [B. mutus]
ENSBTAP00000017710-D1	-1.43	7.85E-106	↑	ATP synthase subunit beta, mitochondrial, partial [B. mutus]
ENSBTAP00000032212-D1	-1.43	3.23E-53	↑	3-hydroxyacyl-CoA dehydratase 2, partial [B. mutus]
ENSP00000417651-D1	-1.43	8.00E-73	↑	Cytochrome c oxidase copper chaperone [B. mutus]
ENSP00000314768-D1	-1.43	1.73E-68	↑	Protein LSM14-like protein A, partial [B. mutus]
ENSP00000395389-D1	-1.43	7.83E-07	↑	lysophosphatidic acid receptor 3 [B. taurus]
ENSBTAP00000005166-D1	-1.43	0.00E+00	↑	centriolar coiled-coil protein of 110 kDa isoform X1 [B. mutus]
ENSP00000382529-D1	-1.43	7.14E-137	↑	PAB-dependent poly(A)-specific ribonuclease subunit PAN3 isoform X5 [Bison bison bison]
ENSBTAP00000041666-D1	-1.42	4.93E-09	↑	phospholipase D3 [B. mutus]
ENSBTAP00000020894-D1	-1.42	0.00E+00	↑	Importin subunit alpha-8, partial [B. mutus]
ENSP00000270162-D1	-1.42	1.41E-05	↑	serine/threonine-protein kinase SIK1 [B. mutus]
ENSBTAP00000003566-D1	-1.42	8.17E-165	↑	krev interaction trapped protein 1 isoform X1 [B. taurus]
ENSBTAP00000029616-D1	-1.42	3.72E-05	↑	SH2B adapter protein 3, partial [B. mutus]
ENSBTAP00000016021-D1	-1.42	4.98E-73	↑	transmembrane protein 176A [B. mutus]
ENSP00000262144-D1	-1.42	9.70E-18	↑	WD repeat-containing protein 59 [B. mutus]
ENSP00000396491-D1	-1.42	2.78E-83	↑	Formin-1 [B. mutus]
ENSBTAP00000021620-D1	-1.42	6.44E-184	↑	protein LSM12 homolog [Homo sapiens]
ENSBTAP00000053577-D1	-1.42	5.77E-76	↑	5'-AMP-activated protein kinase subunit gamma-2, partial [B. mutus]
ENSP00000369274-D1	-1.42	2.85E-152	↑	phosphorylase b kinase regulatory subunit alpha, liver isoform [B. mutus]
ENSP00000327647-D2	-1.42	8.35E-37	↑	death domain-containing protein CRADD [B. mutus]
ENSP00000010299-D1	-1.42	6.20E-74	↑	Protein FAM76A, partial [B. mutus]
ENSBTAP00000029226-D1	-1.42	2.77E-97	↑	Spermatid perinuclear RNA-binding protein, partial [B. mutus]
ENSP00000232978-D1	-1.41	7.30E-159	↑	NK-tumor recognition protein isoform X2 [Bison bison bison]
ENSBTAP00000002778-D1	-1.41	5.21E-92	↑	DTW domain-containing protein 2 [B. mutus]
ENSP00000383945-D3	-1.41	1.63E-07	↑	Zinc finger protein 169, partial [B. mutus]
ENSP00000358617-D2	-1.41	0.00E+00	↑	Putative homeodomain transcription factor 2, partial [B. mutus]
ENSBTAP00000018040-D1	-1.41	2.01E-116	↑	Ubiquitin-conjugating enzyme E2 variant 3, partial [B. mutus]
ENSP00000289371-D1	-1.41	0.00E+00	↑	eukaryotic translation initiation factor 5B isoform X1 [B. taurus]
ENSP00000381666-D1	-1.41	2.24E-41	↑	Glucoside xylosyltransferase 1, partial [B. mutus]

ENSP00000306788-D1	-1.41	0.00E+00	↑	ADP-ribosylation factor-like protein 6-interacting protein 1 [B. mutus]
ENSP00000276590-D1	-1.41	2.14E-65	↑	beta-lactamase-like protein 2 [B. mutus]
ENSP00000414921-D1	-1.41	1.19E-75	↑	polypyrimidine tract-binding protein 3 isoform X1 [B. taurus]
ENSBTAP00000025850-D1	-1.41	7.38E-18	↑	Coiled-coil domain-containing protein 37 [B. mutus]
ENSP00000358956-D1	-1.41	6.80E-35	↑	interleukin-1 receptor-associated kinase 1-binding protein 1 [B. mutus]
ENSP00000377370-D1	-1.41	1.09E-15	↑	immunoglobulin superfamily member 11 isoform X1 [B. taurus]
ENSBTAP00000028690-D1	-1.41	1.45E-25	↑	Insulin-like growth factor 1 receptor [B. mutus]
ENSP00000400787-D1	-1.41	5.59E-160	↑	NIF3-like protein 1 [B. mutus]
ENSP00000345494-D1	-1.41	1.86E-66	↑	Phospholipid scramblase 2 [B. mutus]
ENSP00000261623-D1	-1.41	1.98E-09	↑	Cytochrome b-245 light chain [B. mutus]
ENSP00000384666-D1	-1.41	2.73E-05	↑	peptidyl-prolyl cis-trans isomerase A-like [B. mutus]
ENSP00000324175-D1	-1.41	2.73E-05	↑	MEF2-activating motif and SAP domain-containing transcriptional regulator isoform X1 [B. mutus]
ENSP00000342254-D1	-1.41	7.33E-04	↑	uncharacterized protein C15orf61 homolog [B. taurus]
ENSBTAP00000040537-D1	-1.41	7.33E-04	↑	epsin-1 isoform X2 [B. taurus]
ENSBTAP00000023577-D1	-1.41	4.17E-229	↑	Nuclear receptor subfamily 2 group C member 1 [B. mutus]
ENSP00000311679-D1	-1.41	4.90E-122	↑	Zinc finger protein 483, partial [B. mutus]
ENSP00000343899-D1	-1.41	4.17E-13	↑	FERM domain-containing protein 6 isoform X1 [B. mutus]
yakG039592	-1.41	1.27E-70	↑	Eukaryotic translation initiation factor 3 subunit B [B. mutus]
ENSP00000414712-D1	-1.41	1.15E-28	↑	Ensconsin, partial [B. mutus]
ENSP00000411372-D1	-1.40	2.87E-06	↑	Protein FAM200A [B. mutus]
ENSBTAP00000009951-D1	-1.40	0.00E+00	↑	TPA: translocating chain-associated membrane protein 1 [B. taurus]
ENSP00000347555-D1	-1.40	4.47E-260	↑	Abl interactor 1 [B. mutus]
ENSBTAP00000008409-D1	-1.40	1.02E-56	↑	hypothetical protein M91_08375, partial [B. mutus]
ENSP00000375751-D1	-1.40	2.39E-186	↑	Apoptosis-stimulating of p53 protein 2, partial [B. mutus]
ENSBTAP00000053346-D1	-1.40	2.82E-30	↑	Aminopeptidase O, partial [B. mutus]
ENSBTAP00000004879-D1	-1.40	7.22E-05	↑	Non-specific lipid-transfer protein, partial [B. mutus]
ENSP00000417183-D2	-1.40	7.56E-06	↑	dual specificity protein phosphatase 7 [B. taurus]
ENSBTAP00000004472-D1	-1.40	5.08E-75	↑	Ubiquitin carboxyl-terminal hydrolase 46, partial [B. mutus]
ENSP00000228280-D1	-1.40	1.66E-102	↑	Kit ligand, partial [B. mutus]
ENSBTAP00000001949-D1	-1.40	0.00E+00	↑	Zinc finger protein 280D, partial [B. mutus]
ENSBTAP00000014336-D1	-1.40	8.84E-19	↑	calcium/calmodulin-dependent protein kinase kinase 2 isoform X1 [B. mutus]
ENSP00000321556-D1	-1.40	2.27E-07	↑	Dedicator of cytokinesis protein 6, partial [B. mutus]
ENSBTAP00000004658-D1	-1.40	2.08E-03	↑	TPA: KIAA1717 protein-like [B. taurus]
ENSP00000390155-D2	-1.39	8.78E-11	↑	liver carboxylesterase-like, partial [B. mutus]
ENSP00000361021-D1	-1.39	3.59E-93	↑	TPA_exp: mitochondrial PTENalpha [B. taurus]
ENSP00000344215-D1	-1.39	3.44E-304	↑	tumor protein p53-inducible nuclear protein 1 isoform X1 [B. mutus]
ENSBTAP00000014994-D1	-1.39	5.10E-143	↑	TRMT1-like protein isoform X2 [B. taurus]
ENSP00000377381-D1	-1.39	3.54E-14	↑	ubiquitin domain-containing protein 2 [Bison bison bison]
ENSP00000306991-D1	-1.39	0.00E+00	↑	hyaluronan synthase 2-like [B. mutus]
ENSBTAP00000027294-D1	-1.39	3.25E-211	↑	Activating signal cointegrator 1 complex subunit 3 [B. mutus]
ENSP00000359478-D1	-1.39	2.76E-255	↑	canalicular multispecific organic anion transporter 1 [B. mutus]
ENSBTAP00000005850-D1	-1.39	0.00E+00	↑	Oral-facial-digital syndrome 1 protein, partial [B. mutus]
ENSBTAP00000004549-D1	-1.39	3.15E-203	↑	Phosphatidylinositide phosphatase SAC1, partial [B. mutus]
ENSP00000363587-D1	-1.39	2.21E-77	↑	sushi, von Willebrand factor type A, EGF and pentraxin domain-containing protein 1, partial [B. mutus]
ENSBTAP00000018618-D1	-1.39	2.25E-110	↑	Tropomodulin-2 [B. mutus]
ENSBTAP00000005688-D1	-1.39	6.39E-66	↑	39S ribosomal protein L21, mitochondrial [B. mutus]
ENSBTAP00000007213-D3	-1.39	1.49E-09	↑	Calcitonin receptor-stimulating peptide 2 [B. mutus]
ENSP00000261723-D1	-1.39	0.00E+00	↑	cytoplasmic polyadenylation element-binding protein 1 isoform X4 [Bubalus bubalis]
ENSBTAP00000026452-D1	-1.39	1.31E-259	↑	OTU domain-containing protein 4, partial [B. mutus]
ENSBTAP00000027352-D1	-1.38	6.12E-63	↑	translation initiation factor IF-3, mitochondrial [B. mutus]
ENSP00000391402-D1	-1.38	1.42E-04	↑	CDGSH iron-sulfur domain-containing protein 3, mitochondrial, partial [B. mutus]
ENSP00000222382-D3	-1.38	5.12E-24	↑	cytochrome P450 3A24-like [B. mutus]
ENSBTAP00000004811-D1	-1.38	0.00E+00	↑	WD repeat domain phosphoinositide-interacting protein 3, partial [B. mutus]
ENSP00000371310-D1	-1.38	1.38E-261	↑	Ubiquitin carboxyl-terminal hydrolase 7, partial [B. mutus]
ENSP00000368464-D1	-1.38	7.78E-124	↑	Kinesin-like protein KIF24 [B. mutus]

ENSBTAP00000053594-D1	-1.38	1.56E-12	↑	Zinc finger ZZ-type and EF-hand domain-containing protein 1, partial [B. mutus]
ENSBTAP00000026639-D1	-1.37	2.28E-20	↑	Rap1 GTPase-activating protein 2, partial [B. mutus]
ENSP00000264954-D1	-1.37	0.00E+00	↑	GrpE protein-like protein 1, mitochondrial, partial [B. mutus]
yakG021538	-1.37	2.13E-52	↑	hypothetical protein M91_19301 [B. mutus]
ENSP00000230431-D2	-1.37	4.84E-21	↑	2'-deoxynucleoside 5'-phosphate N-hydrolase 1 [B. mutus]
ENSP00000402109-D1	-1.37	1.87E-08	↑	Protein GPR107, partial [B. mutus]
ENSBTAP00000007786-D3	-1.37	6.53E-16	↑	coiled-coil domain-containing protein 3-like isoform X1 [B. taurus]
ENSBTAP00000021529-D4	-1.37	0.00E+00	↑	nucleolin [Bubalus bubalis]
ENSBTAP00000015794-D1	-1.37	0.00E+00	↑	Ras-related protein R-Ras2, partial [B. mutus]
ENSBTAP00000031693-D1	-1.37	5.78E-15	↑	Zinc finger protein 322A, partial [B. mutus]
ENSP00000372024-D1	-1.37	2.01E-49	↑	hypothetical protein M91_10246, partial [B. mutus]
ENSBTAP00000013697-D1	-1.37	8.36E-25	↑	hypothetical protein M91_11992, partial [B. mutus]
ENSP00000388996-D1	-1.37	4.80E-08	↑	AP-1 complex subunit mu-1 [Capra hircus]
ENSP00000395886-D3	-1.37	1.02E-04	↑	40S ribosomal protein S28-like [B. mutus]
ENSBTAP00000004671-D1	-1.37	1.02E-04	↑	RIB43A-like with coiled-coils protein 2, partial [B. mutus]
ENSP00000371061-D1	-1.37	0.00E+00	↑	UPF0600 protein C5orf51 homolog isoform X1 [Bison bison bison]
ENSBTAP00000015716-D1	-1.37	2.76E-156	↑	elongation factor 1-gamma [B. mutus]
ENSBTAP00000053476-D1	-1.36	1.87E-23	↑	roquin-2 isoform X1 [Bison bison bison]
ENSBTAP00000017209-D1	-1.36	5.94E-34	↑	probable tRNA pseudouridine synthase 1 isoform X1 [B. taurus]
ENSBTAP00000029084-D1	-1.36	8.21E-10	↑	CD97 antigen isoform X1 [B. mutus]
ENSBTAP00000019948-D1	-1.36	3.51E-23	↑	regulation of nuclear pre-mRNA domain-containing protein 2 isoform X1 [B. mutus]
ENSBTAP00000026370-D1	-1.36	3.51E-23	↑	Grainyhead-like protein 3-like protein, partial [B. mutus]
ENSP00000354558-D1	-1.36	4.39E-27	↑	serine/threonine-protein kinase mTOR [B. mutus]
ENSBTAP00000046577-D1	-1.36	1.11E-42	↑	Torsin-1A-interacting protein 2, partial [B. mutus]
ENSP00000342422-D1	-1.36	5.77E-45	↑	discoidin, CUB and LCCL domain-containing protein 1 isoform X2 [B. taurus]
ENSBTAP00000045110-D1	-1.36	2.09E-19	↑	nucleoside diphosphate kinase, mitochondrial precursor [B. taurus]
ENSBTAP00000025851-D1	-1.36	5.88E-15	↑	mitochondrial assembly of ribosomal large subunit protein 1 [B. mutus]
ENSP00000411459-D1	-1.36	2.63E-150	↑	Rho-guanine nucleotide exchange factor, partial [B. mutus]
ENSBTAP00000013858-D1	-1.36	5.05E-44	↑	SERTA domain-containing protein 1 [B. taurus]
ENSP00000264331-D1	-1.36	0.00E+00	↑	DNA topoisomerase 2-beta, partial [B. mutus]
ENSBTAP00000004047-D1	-1.36	3.14E-222	↑	THUMP domain-containing protein 3 [B. mutus]
ENSP00000370949-D1	-1.36	6.26E-41	↑	NF-X1-type zinc finger protein NFXL1 isoform X1 [Bison bison bison]
ENSBTAP00000042931-D1	-1.36	1.08E-175	↑	lysM and putative peptidoglycan-binding domain-containing protein 4 [B. mutus]
ENSBTAP00000004955-D1	-1.36	1.68E-22	↑	cell differentiation protein RCD1 homolog, partial [Struthio camelus australis]
ENSP00000385467-D4	-1.36	1.93E-30	↑	TPA: hypothetical protein BOS_6159 [B. taurus]
ENSBTAP00000025978-D1	-1.36	0.00E+00	↑	probable 8-oxo-dGTP diphosphatase NUDT15 [B. taurus]
ENSBTAP00000041402-D1	-1.36	1.80E-126	↑	A-kinase anchor protein 10, mitochondrial, partial [B. mutus]
ENSBTAP00000017010-D1	-1.36	3.27E-178	↑	tetraspanin-13 [Eptesicus fuscus]
ENSBTAP00000004480-D3	-1.35	1.12E-35	↑	V-type proton ATPase subunit H isoform X1 [Bubalus bubalis]
ENSP00000262539-D1	-1.35	9.55E-46	↑	Tyrosine-protein phosphatase non-receptor type 3 [B. mutus]
ENSBTAP00000037932-D1	-1.35	5.66E-291	↑	Inhibitor of Bruton tyrosine kinase, partial [B. mutus]
ENSBTAP00000022196-D1	-1.35	5.92E-29	↑	Putative protein KIAA0319-like protein, partial [B. mutus]
ENSBTAP00000043649-D1	-1.35	2.32E-38	↑	hypothetical protein M91_20809, partial [B. mutus]
ENSP00000384179-D1	-1.35	6.14E-45	↑	Zinc finger protein ZFPM2, partial [B. mutus]
ENSBTAP00000013153-D3	-1.35	4.70E-52	↑	UV excision repair protein RAD23-like protein A [B. mutus]
ENSBTAP00000039756-D1	-1.35	2.67E-23	↑	Vitamin D 25-hydroxylase, partial [B. mutus]
ENSBTAP00000029302-D1	-1.35	2.16E-53	↑	protein PRRC1 [B. mutus]
ENSP00000238789-D1	-1.35	1.44E-79	↑	ATPase family AAA domain-containing protein 2B [B. taurus]
ENSBTAP00000027071-D1	-1.35	3.51E-130	↑	nuclear receptor coactivator 2 [B. mutus]
ENSBTAP00000032310-D1	-1.35	3.38E-45	↑	vitamin K-dependent protein S [B. mutus]
ENSP00000347134-D1	-1.35	2.22E-53	↑	neuroepithelial cell-transforming gene 1 protein isoform X1 [B. taurus]
ENSP00000253332-D1	-1.35	2.18E-44	↑	A-kinase anchor protein 12, partial [B. mutus]
ENSBTAP00000007704-D1	-1.35	5.64E-33	↑	Cyclic AMP-dependent transcription factor ATF-7, partial [B. mutus]
ENSBTAP00000004376-D1	-1.35	1.05E-03	↑	UDP-xylose and UDP-N-acetylglucosamine transporter [B. mutus]
ENSBTAP00000034933-D1	-1.35	1.06E-132	↑	Protein FAM18B1 [B. mutus]

ENSBTAP00000009971-D1	-1.35	1.88E-54	↑	Shootin-1, partial [B. mutus]
ENSP00000273612-D1	-1.34	0.00E+00	↑	Protein VPRBP, partial [B. mutus]
ENSP00000377528-D1	-1.34	0.00E+00	↑	F-box/WD repeat-containing protein 7 isoform X1 [B. taurus]
ENSP00000335632-D1	-1.34	0.00E+00	↑	calcineurin B homologous protein 1-like isoformX1 [Sus scrofa]
ENSP00000250173-D1	-1.34	2.10E-14	↑	Leucine-rich repeat-containing protein 6, partial [B. mutus]
ENSP00000371390-D1	-1.34	0.00E+00	↑	transmembrane protein 50B [Homo sapiens]
ENSP00000309644-D2	-1.34	1.07E-05	↑	biorientation of chromosomes in cell division protein 1-like [Balaenoptera acutorostrata scammoni]
ENSP00000287322-D3	-1.34	3.02E-26	↑	BAG family molecular chaperone regulator 4, partial [B. mutus]
ENSP00000366557-D1	-1.34	0.00E+00	↑	Crooked neck-like protein 1, partial [B. mutus]
ENSP00000336856-D1	-1.34	1.36E-78	↑	high mobility group protein 20A [B. mutus]
ENSP00000246505-D1	-1.34	4.26E-17	↑	PCI domain-containing protein 2, partial [B. mutus]
ENSBTAP00000011028-D1	-1.34	2.00E-32	↑	Protein fem-1-like protein B, partial [B. mutus]
ENSP00000362975-D1	-1.34	6.63E-18	↑	Protein FAM13C, partial [B. mutus]
ENSBTAP00000012233-D1	-1.34	6.57E-56	↑	Glutaminase liver isoform, mitochondrial [B. mutus]
ENSBTAP00000026878-D1	-1.34	1.41E-18	↑	aladin [B. taurus]
ENSBTAP00000015186-D4	-1.34	6.48E-13	↑	tropomyosin alpha-3 chain-like isoform X1 [B. mutus]
ENSBTAP00000048399-D1	-1.34	9.21E-08	↑	55 kDa erythrocyte membrane protein, partial [B. mutus]
ENSBTAP00000012565-D1	-1.34	9.88E-72	↑	Beta-galactoside alpha-2,6-sialyltransferase 1 [B. mutus]
ENSBTAP00000053762-D1	-1.33	0.00E+00	↑	ras-related protein Rab-2B [B. taurus]
ENSBTAP00000017191-D1	-1.33	1.55E-85	↑	Dysbindin, partial [B. mutus]
ENSBTAP00000028867-D1	-1.33	1.24E-221	↑	Apoptotic protease-activating factor 1, partial [B. mutus]
ENSP00000287156-D1	-1.33	8.45E-22	↑	Ubiquitin/ISG15-conjugating enzyme E2 L6, partial [B. mutus]
ENSP00000315212-D1	-1.33	6.91E-78	↑	E3 ubiquitin-protein ligase RNF4 isoform X1 [B. mutus]
ENSP00000279907-D1	-1.33	6.50E-159	↑	UHRF1-binding protein 1-like [B. mutus]
ENSBTAP00000024600-D1	-1.33	7.25E-52	↑	NADH dehydrogenase [ubiquinone] iron-sulfur protein 3, mitochondrial [B. mutus]
ENSBTAP00000007484-D1	-1.33	3.96E-30	↑	Protein DBF4-like protein B, partial [B. mutus]
ENSP00000358831-D1	-1.33	3.49E-38	↑	elongation of very long chain fatty acids protein 4 [B. mutus]
ENSBTAP00000015284-D1	-1.33	4.35E-03	↑	hypothetical protein M91_18522, partial [B. mutus]
ENSBTAP00000010179-D1	-1.33	4.35E-03	↑	Cytokine receptor-like factor 1, partial [B. mutus]
ENSP00000349259-D1	-1.33	2.00E-115	↑	Spectrin beta chain, brain 1 [B. mutus]
ENSBTAP00000029060-D1	-1.33	5.18E-67	↑	migration and invasion enhancer 1 [B. taurus]
ENSP00000383986-D1	-1.33	1.29E-294	↑	Cell division cycle-associated 7-like protein, partial [B. mutus]
ENSP00000350854-D1	-1.33	1.12E-43	↑	vacuolar protein sorting-associated protein 13D [B. mutus]
ENSBTAP00000009440-D1	-1.33	9.48E-69	↑	aspartate aminotransferase, mitochondrial isoform X1 [B. mutus]
ENSP00000261722-D1	-1.33	5.15E-11	↑	AP-3 complex subunit beta-2 isoform X2 [B. mutus]
ENSBTAP00000006465-D4	-1.33	1.16E-09	↑	Profilin-1, partial [B. mutus]
ENSBTAP00000024956-D1	-1.32	1.41E-99	↑	Mitochondrial carrier-like protein 2, partial [B. mutus]
ENSP00000265036-D1	-1.32	0.00E+00	↑	DEP domain-containing protein 1B isoform X1 [B. mutus]
ENSP00000373696-D1	-1.32	0.00E+00	↑	Putative E3 ubiquitin-protein ligase TRIP12 [B. mutus]
ENSP00000268184-D1	-1.32	3.09E-19	↑	CREB-regulated transcription coactivator 3 [B. mutus]
ENSP00000324948-D1	-1.32	2.90E-32	↑	transcription factor SOX-6 isoform X3 [B. taurus]
ENSP00000367081-D4	-1.32	1.59E-80	↑	Sodium-dependent phosphate transport protein 1, partial [B. mutus]
ENSBTAP00000036999-D1	-1.32	3.50E-53	↑	Copine-3 [B. mutus]
ENSP00000359172-D1	-1.32	1.34E-94	↑	UDP-N-acetylglucosamine transporter, partial [B. mutus]
ENSBTAP00000000143-D1	-1.32	9.29E-27	↑	sentrin-specific protease 3 isoform X1 [Capra hircus]
ENSP00000369461-D1	-1.32	4.17E-102	↑	Peroxisomal 3,2-trans-enoyl-CoA isomerase, partial [B. mutus]
ENSBTAP00000017580-D1	-1.32	1.07E-27	↑	ADP/ATP translocase 1 [B. taurus]
ENSP00000303507-D1	-1.32	5.41E-04	↑	Breakpoint cluster region protein, partial [B. mutus]
ENSP00000361160-D1	-1.32	5.04E-149	↑	serine-rich coiled-coil domain-containing protein 2 isoform X1 [B. mutus]
ENSP00000310520-D1	-1.31	0.00E+00	↑	DNA repair endonuclease XPF [B. mutus]
ENSP00000360275-D1	-1.31	1.90E-42	↑	Disabled-like protein 1, partial [B. mutus]
ENSP00000295414-D1	-1.31	0.00E+00	↑	cyclin-Y-like protein 1 isoform X1 [B. taurus]
ENSP00000384586-D1	-1.31	1.28E-26	↑	Protein Mpv17 [B. mutus]
ENSP00000410689-D1	-1.31	5.85E-12	↑	actin filament-associated protein 1 isoform X1 [B. mutus]
ENSBTAP00000023707-D1	-1.31	1.75E-17	↑	glutaredoxin-like protein C5orf63 homolog [B. taurus]

ENSP00000367991-D2	-1.31	0.00E+00	↑	Septin-11, partial [B. mutus]
ENSBTAP00000033878-D1	-1.31	0.00E+00	↑	cyclin-dependent kinases regulatory subunit 1-like [Cricetulus griseus]
ENSP00000338160-D1	-1.31	2.64E-133	↑	Peptidyl-prolyl cis-trans isomerase FKBP5 [B. mutus]
ENSBTAP00000001332-D1	-1.31	8.88E-245	↑	zinc finger protein 280B isoform X1 [B. mutus]
ENSBTAP00000006381-D1	-1.31	2.01E-38	↑	Pleckstrin-like protein domain-containing family O member 1, partial [B. mutus]
ENSBTAP00000042489-D1	-1.31	6.92E-18	↑	Protein phosphatase 1 regulatory subunit 11, partial [B. mutus]
ENSBTAP00000003493-D1	-1.31	1.69E-19	↑	ubiquitin-protein ligase E3B isoform X1 [B. mutus]
ENSBTAP00000002549-D1	-1.31	2.06E-36	↑	28S ribosomal protein S27, mitochondrial isoform X1 [Bison bison bison]
ENSBTAP00000009098-D1	-1.31	6.18E-115	↑	ATP-binding cassette sub-family F member 1 [B. taurus]
ENSP00000349929-D1	-1.31	1.24E-19	↑	Serologically defined colon cancer antigen 3, partial [B. mutus]
ENSP00000180173-D1	-1.31	1.67E-41	↑	Myotubularin-related protein 7, partial [B. mutus]
ENSP00000352925-D1	-1.31	7.47E-45	↑	transformation/transcription domain-associated protein [B. mutus]
ENSBTAP00000012949-D1	-1.31	1.20E-21	↑	Serine/threonine-protein phosphatase 4 regulatory subunit 1, partial [B. mutus]
ENSP00000413737-D1	-1.31	5.71E-30	↑	tafazzin isoform X4 [Bubalus bubalis]
ENSBTAP00000026571-D1	-1.31	0.00E+00	↑	ubiquitin carboxyl-terminal hydrolase 44 [B. mutus]
ENSP00000393518-D1	-1.30	1.92E-31	↑	Poly [ADP-ribose] polymerase 9, partial [B. mutus]
yakG028650	-1.30	9.85E-25	↑	Heterogeneous nuclear ribonucleoprotein U-like protein 1 [B. mutus]
ENSP00000393313-D1	-1.30	1.36E-61	↑	Epidermal growth factor receptor substrate 15-like 1, partial [B. mutus]
ENSBTAP00000023939-D1	-1.30	8.21E-80	↑	Protein max, partial [B. mutus]
ENSBTAP00000022338-D1	-1.30	8.10E-39	↑	peroxisomal biogenesis factor 7 [B. taurus]
ENSBTAP00000017032-D1	-1.30	4.91E-13	↑	Syntaxin-3, partial [B. mutus]
ENSP00000361027-D1	-1.30	2.86E-49	↑	angiomotin [B. mutus]
ENSP00000340823-D2	-1.30	0.00E+00	↑	general transcription factor IIF subunit 2 isoform X1 [Ovis aries]
ENSBTAP00000013522-D1	-1.30	6.98E-18	↑	dehydrogenase/reductase SDR family member 1 [B. mutus]
ENSP00000267396-D1	-1.30	3.77E-18	↑	GTP-binding protein REM 2 [B. taurus]
ENSP00000329933-D1	-1.30	1.04E-44	↑	PHD finger protein 12 [B. mutus]
ENSP00000367679-D1	-1.30	4.74E-33	↑	serine/threonine-protein kinase PAK 7 [B. mutus]
ENSP00000265838-D1	-1.29	4.99E-176	↑	Acetyl-CoA acetyltransferase, mitochondrial, partial [B. mutus]
ENSBTAP00000000160-D1	-1.29	2.68E-08	↑	N-lysine methyltransferase SETD8 [Bison bison bison]
ENSBTAP00000006162-D1	-1.29	5.36E-16	↑	Cell division protein kinase 9, partial [B. mutus]
ENSBTAP00000013726-D1	-1.29	1.56E-25	↑	dedicator of cytokinesis protein 9 isoform X4 [Bison bison bison]
ENSBTAP00000048587-D1	-1.29	2.02E-126	↑	AP-2 complex subunit sigma, partial [B. mutus]
ENSBTAP00000022939-D1	-1.29	6.42E-263	↑	long-chain-fatty-acid--CoA ligase 3 [B. taurus]
ENSBTAP00000004465-D1	-1.29	1.02E-61	↑	zinc finger and BTB domain-containing protein 43 [B. taurus]
ENSBTAP00000027570-D1	-1.29	1.51E-05	↑	RelA-associated inhibitor [B. mutus]
ENSP00000295797-D1	-1.29	1.50E-18	↑	protein kinase C iota type [B. taurus]
ENSBTAP00000005204-D1	-1.29	1.33E-10	↑	CXXC-type zinc finger protein 5 isoform X1 [Bubalus bubalis]
ENSP00000353401-D1	-1.29	5.30E-51	↑	Homeobox protein cut-like 1, partial [B. mutus]
ENSP00000390531-D1	-1.29	2.24E-103	↑	AP-5 complex subunit mu-1 [B. mutus]
ENSBTAP00000005065-D1	-1.29	9.38E-08	↑	Constitutive coactivator of peroxisome proliferator-activated receptor gamma, partial [B. mutus]
ENSBTAP00000022906-D1	-1.29	1.09E-05	↑	Fatty acid desaturase 6, partial [B. mutus]
ENSP00000319248-D1	-1.29	4.94E-13	↑	Zinc finger E-box-binding homeobox 1, partial [B. mutus]
ENSBTAP00000000718-D1	-1.28	2.12E-16	↑	kelch domain-containing protein 10 [Propithecus coquereli]
ENSP00000258428-D1	-1.28	1.49E-113	↑	DNA repair protein REV1 [B. mutus]
ENSP00000373657-D1	-1.28	3.28E-245	↑	uncharacterized protein C17orf85 homolog [B. taurus]
ENSBTAP00000021984-D1	-1.28	2.82E-11	↑	carbonic anhydrase 5B, mitochondrial [B. mutus]
ENSP00000259512-D1	-1.28	4.92E-31	↑	derlin-1 [B. taurus]
ENSBTAP00000020512-D4	-1.28	0.00E+00	↑	Cofilin-1 [Heterocephalus glaber]
ENSP00000312758-D1	-1.28	1.14E-109	↑	Hermansky-Pudlak syndrome 5 protein [B. mutus]
ENSP00000413684-D1	-1.28	6.18E-26	↑	dnaJ homolog subfamily B member 5 isoform X1 [Capra hircus]
ENSP00000410298-D1	-1.28	2.49E-136	↑	calcium/calmodulin-dependent protein kinase type II subunit gamma isoform X1 [Nannospalax galili]
ENSBTAP00000027190-D1	-1.28	3.61E-261	↑	Zinc finger MYM-type protein 2 [B. mutus]
ENSP00000381470-D1	-1.28	0.00E+00	↑	spindle and kinetochore-associated protein 1 [B. taurus]
ENSP00000295148-D1	-1.28	8.96E-22	↑	WD repeat-containing protein C2orf44 homolog [B. mutus]
ENSP00000349588-D1	-1.28	8.17E-84	↑	Ankyrin-2, partial [B. mutus]

yakA25976	-1.28	3.75E-152	↑	hypothetical protein M91_21720 [B. mutus]
ENSP00000246043-D1	-1.28	3.34E-37	↑	ribosome-binding protein 1 isoform X3 [Bubalus bubalis]
ENSP00000370055-D1	-1.28	2.72E-29	↑	ubiquitin-like protein 3 [Aotus nancymaae]
ENSP00000331105-D1	-1.28	5.24E-11	↑	thyroid adenoma-associated protein homolog [B. mutus]
ENSP00000276326-D2	-1.27	2.60E-22	↑	F-box only protein 32 [B. mutus]
ENSBTAP00000031373-D1	-1.27	3.98E-159	↑	Caprin-2, partial [B. mutus]
ENSBTAP00000005749-D1	-1.27	8.12E-28	↑	Signal transducer and activator of transcription 2 [B. mutus]
ENSBTAP00000017477-D1	-1.27	2.68E-20	↑	N-alpha-acetyltransferase 11 [B. taurus]
ENSP00000416371-D1	-1.27	0.00E+00	↑	TPA: ubiquitin specific peptidase 34-like [B. taurus]
ENSBTAP00000039541-D1	-1.27	1.07E-42	↑	netrin receptor UNC5C isoform X2 [B. taurus]
ENSBTAP00000043467-D1	-1.27	9.07E-87	↑	Reticulon-3 [B. mutus]
ENSBTAP00000053484-D1	-1.27	7.82E-114	↑	PRKC apoptosis WT1 regulator protein, partial [B. mutus]
ENSP00000352962-D1	-1.27	1.33E-10	↑	Lipid phosphate phosphatase-related protein type 3 [B. mutus]
ENSP00000313377-D1	-1.27	1.46E-29	↑	pantothenate kinase 2, mitochondrial [B. mutus]
ENSBTAP00000016760-D1	-1.27	6.50E-154	↑	Beta-1,4-galactosyltransferase 5, partial [B. mutus]
ENSP00000220562-D1	-1.27	3.51E-22	↑	exostosin-like 3-like [B. mutus]
ENSP00000359956-D1	-1.27	9.02E-31	↑	elongation of very long chain fatty acids protein 5-like [B. mutus]
ENSP00000278483-D1	-1.27	4.85E-198	↑	protein Hikeshi [B. taurus]
ENSP00000357330-D1	-1.27	1.74E-147	↑	histone-lysine N-methyltransferase ASH1L isoform X1 [Bison bison bison]
ENSP00000225688-D1	-1.27	2.06E-05	↑	dexamethasone-induced Ras-related protein 1 [B. taurus]
ENSBTAP00000008486-D1	-1.26	2.81E-11	↑	oxysterol-binding protein-related protein 11 [B. taurus]
ENSBTAP00000039862-D1	-1.26	9.15E-13	↑	basigin [B. mutus]
ENSP00000397094-D1	-1.26	7.18E-291	↑	Arf-GAP domain and FG repeats-containing protein 1, partial [B. mutus]
ENSBTAP00000001466-D1	-1.26	0.00E+00	↑	germ cell-less protein-like 1 isoform X1 [B. taurus]
ENSBTAP00000043672-D1	-1.26	2.30E-41	↑	DNA repair protein complementing XP-G cells [B. mutus]
ENSP00000381428-D1	-1.26	3.61E-271	↑	CGG triplet repeat-binding protein 1 [Homo sapiens]
ENSBTAP00000004093-D1	-1.26	0.00E+00	↑	Interferon alpha-inducible protein 27-like protein 1 [B. mutus]
ENSP00000297405-D3	-1.26	4.22E-41	↑	CUB and sushi domain-containing protein 2, partial [B. mutus]
ENSP00000271311-D1	-1.26	5.73E-06	↑	fibronectin type III domain-containing protein 7 [B. mutus]
ENSBTAP00000024458-D1	-1.26	1.60E-09	↑	Spermine synthase, partial [B. mutus]
ENSBTAP00000039437-D1	-1.26	6.75E-53	↑	amyloid beta A4 precursor protein-binding family B member 2 isoform X4 [B. taurus]
ENSBTAP00000027109-D1	-1.25	6.85E-08	↑	putative helicase Mov10l1 [B. mutus]
ENSP00000395220-D1	-1.25	1.26E-65	↑	synaptotagmin-like protein 5 isoform X1 [B. mutus]
ENSP00000366396-D1	-1.25	1.10E-116	↑	5'-3' exoribonuclease 2, partial [B. mutus]
ENSP00000331310-D1	-1.25	1.27E-174	↑	mortality factor 4-like protein 1 isoform X1 [B. taurus]
ENSBTAP00000009563-D1	-1.25	1.91E-42	↑	V-type proton ATPase 116 kDa subunit a isoform 2 [B. taurus]
ENSP00000378201-D1	-1.25	1.40E-42	↑	zinc finger and BTB domain-containing protein 1 [B. taurus]
ENSP00000354033-D1	-1.25	3.03E-06	↑	Polycomb group RING finger protein 2 [B. mutus]
ENSP00000367189-D1	-1.25	3.03E-06	↑	UPF0723 protein C11orf83 homolog [B. mutus]
ENSP00000403163-D1	-1.25	6.80E-103	↑	VPS33B-interacting protein, partial [B. mutus]
ENSP00000360017-D2	-1.25	1.72E-37	↑	Zinc finger protein 280C, partial [B. mutus]
ENSBTAP00000036386-D1	-1.25	2.62E-20	↑	sodium/potassium/calcium exchanger 1 isoform X1 [B. mutus]
ENSP00000410403-D1	-1.25	1.45E-04	↑	ras-related protein Rab-34 [B. mutus]
ENSBTAP00000010002-D1	-1.25	6.01E-17	↑	probable methyltransferase BTM2 homolog [Pantholops hodgsonii]
ENSP00000336607-D1	-1.25	1.45E-27	↑	P2X purinoceptor 4, partial [B. mutus]
ENSP00000416290-D1	-1.25	9.42E-229	↑	hypothetical protein M91_15832, partial [B. mutus]
ENSP00000263256-D1	-1.25	7.03E-25	↑	desumoylating isopeptidase 1 isoform X3 [B. taurus]
ENSP00000354952-D1	-1.25	2.17E-09	↑	TPA: GRB2-associated binding protein 2-like [B. taurus]
ENSP00000284202-D1	-1.24	1.92E-51	↑	protein IMPACT isoform X2 [B. taurus]
ENSBTAP00000002733-D1	-1.24	1.11E-16	↑	zinc finger CCHC domain-containing protein 3 [B. taurus]
ENSP00000334379-D1	-1.24	6.48E-125	↑	C2 domain-containing protein 3, partial [B. mutus]
ENSP00000378427-D1	-1.24	3.96E-54	↑	UPF0461 protein C5orf24 homolog [Capra hircus]
ENSP00000350377-D1	-1.24	2.87E-32	↑	multiple C2 and transmembrane domain-containing protein 2 [B. mutus]
ENSBTAP00000024328-D1	-1.24	1.28E-07	↑	Solute carrier family 28 member 3 [B. mutus]
ENSBTAP00000024607-D1	-1.24	4.01E-65	↑	AF4/FMR2 family member 1, partial [B. mutus]

ENSBTAP00000002916-D1	-1.24	6.80E-08	↑	betaine--homocysteine S-methyltransferase 1 isoform X1 [B. mutus]
ENSBTAP00000023407-D1	-1.24	1.76E-92	↑	Ras-related protein Rab-13, partial [B. mutus]
ENSP00000320340-D1	-1.24	2.84E-05	↑	diacylglycerol kinase zeta isoform X3 [Ovis aries musimon]
ENSBTAP00000048844-D1	-1.24	1.11E-80	↑	Peroxisome proliferator-activated receptor gamma coactivator 1-alpha, partial [B. mutus]
ENSBTAP00000004441-D1	-1.24	0.00E+00	↑	ATP-dependent RNA helicase DDX24 [B. mutus]
ENSBTAP00000037204-D1	-1.24	2.93E-14	↑	Protein argonaute-2, partial [B. mutus]
ENSP00000354091-D1	-1.24	1.70E-46	↑	guanine nucleotide-binding protein-like 3-like protein isoform X1 [B. mutus]
ENSBTAP00000006171-D1	-1.24	8.44E-15	↑	Zinc finger protein 211, partial [B. mutus]
ENSBTAP00000052846-D1	-1.24	3.99E-21	↑	Breast carcinoma-amplified sequence 4, partial [B. mutus]
ENSP00000349828-D1	-1.24	1.08E-05	↑	Transcriptional enhancer factor TEF-5, partial [B. mutus]
ENSBTAP00000001247-D1	-1.24	1.05E-18	↑	Zinc finger protein 286A, partial [B. mutus]
ENSBTAP00000052799-D2	-1.24	4.03E-09	↑	60S ribosomal protein L35a-like [B. mutus]
ENSBTAP00000027206-D1	-1.24	4.03E-09	↑	probable ATP-dependent RNA helicase DHX37 [B. mutus]
ENSBTAP00000016002-D1	-1.24	1.63E-19	↑	WD repeat domain phosphoinositide-interacting protein 2, partial [B. mutus]
ENSP00000370047-D1	-1.23	2.44E-51	↑	Zinc finger protein basonuclin-2 [B. mutus]
ENSBTAP00000028640-D1	-1.23	1.95E-67	↑	glutathione S-transferase A4 [B. taurus]
ENSBTAP00000025116-D1	-1.23	1.43E-45	↑	leucine-rich repeat and calponin homology domain-containing protein 3 [B. mutus]
ENSP00000380996-D1	-1.23	1.16E-06	↑	hypothetical protein M91_16729 [B. mutus]
ENSBTAP00000024887-D1	-1.23	5.24E-105	↑	Histone acetyltransferase MYST2 [B. mutus]
ENSBTAP00000007067-D3	-1.23	4.34E-53	↑	disks large-associated protein 4 [B. mutus]
ENSP00000298125-D1	-1.23	3.09E-159	↑	WD repeat and FYVE domain-containing protein 2 isoform X2 [B. taurus]
yakG034094	-1.23	1.68E-33	↑	High affinity cAMP-specific 3',5'-cyclic phosphodiesterase 7A [B. mutus]
ENSP00000311344-D1	-1.23	4.88E-34	↑	serine/threonine-protein phosphatase 2A 65 kDa regulatory subunit A beta isoform isoform X1 [B. mutus]
ENSBTAP00000007590-D1	-1.23	3.85E-30	↑	Speedy protein C, partial [B. mutus]
ENSP00000370480-D1	-1.23	6.06E-40	↑	hypothetical protein M91_00113 [B. mutus]
ENSBTAP00000003191-D1	-1.23	0.00E+00	↑	kelch-like protein 8 isoform X1 [B. mutus]
ENSP00000222339-D1	-1.23	3.59E-08	↑	Zinc finger protein 574, partial [B. mutus]
ENSBTAP00000042200-D1	-1.23	5.65E-104	↑	Agmatinase, mitochondrial, partial [B. mutus]
ENSBTAP00000030511-D1	-1.22	1.01E-94	↑	muscleblind-like protein 2 [B. mutus]
ENSP00000395703-D3	-1.22	7.01E-68	↑	60S ribosomal protein L36-like [Myotis brandtii]
ENSP00000358884-D2	-1.22	5.03E-11	↑	Cytochrome b561 domain-containing protein 2, partial [B. mutus]
ENSBTAP00000024631-D1	-1.22	7.72E-12	↑	LisH domain-containing protein ARMC9, partial [B. mutus]
ENSBTAP00000035705-D1	-1.22	0.00E+00	↑	rho GTPase-activating protein 11A [B. mutus]
ENSP00000355557-D2	-1.22	0.00E+00	↑	Chain A, Crystal Structure Of Human Neutrophil Peptide 2 (variant Gly16- >gi|75765495|pdb|1XHM|B Chain B, The Crystal Structure Of A Biologically Active Peptide (Sigk) Bound To A G Protein Beta:gamma Heterodimer
ENSBTAP00000018514-D1	-1.22	4.37E-29	↑	hypothetical protein M91_16219, partial [B. mutus]
ENSBTAP00000019107-D1	-1.22	5.70E-125	↑	TPA: neuropilin- and tolloid-like protein 2-like [B. taurus]
ENSP00000358233-D1	-1.22	1.83E-137	↑	Golgi pH regulator-like isoform X1 [B. mutus]
ENSP00000359520-D1	-1.22	2.65E-23	↑	Ectonucleoside triphosphate diphosphohydrolase 7, partial [B. mutus]
ENSP00000262061-D1	-1.22	2.65E-23	↑	Coatomer subunit zeta-1 [B. mutus]
ENSP00000368547-D1	-1.22	5.88E-92	↑	Fasciculation and elongation protein zeta-2, partial [B. mutus]
ENSBTAP00000012905-D1	-1.22	9.34E-55	↑	ras-related protein Rab-32, partial [B. mutus]
ENSBTAP00000004097-D1	-1.22	4.51E-61	↑	80 kDa MCM3-associated protein [B. mutus]
ENSP00000315357-D1	-1.22	1.29E-27	↑	ADP-ribosylation factor-like protein 6-interacting protein 6, partial [B. mutus]
ENSBTAP00000002274-D1	-1.22	4.33E-15	↑	ubinuclein-1 isoform X1 [Bison bison bison]
ENSP00000384250-D2	-1.22	2.59E-08	↑	SNRPN upstream reading frame protein, partial [B. mutus]
ENSP00000357004-D1	-1.22	1.25E-07	↑	Thiosulfate sulfurtransferase/rhodanese-like domain-containing protein 1 [B. mutus]
ENSBTAP00000006178-D1	-1.22	2.76E-04	↑	histone-lysine N-methyltransferase SUV39H1 isoform X2 [Orcinus orca]
yakA09460	-1.22	4.34E-03	↑	hypothetical protein M91_00902 [B. mutus]
yakG027577	-1.22	9.36E-03	↑	hypothetical protein M91_17768 [B. mutus]
ENSBTAP00000013205-D1	-1.22	9.36E-03	↑	ATP synthase subunit f, mitochondrial [B. mutus]
ENSBTAP00000030983-D1	-1.22	1.42E-02	↑	hypothetical protein M91_19668, partial [B. mutus]
ENSP00000268058-D1	-1.22	2.28E-02	↑	protein PML-like, partial [B. mutus]
ENSBTAP00000041866-D1	-1.22	2.28E-02	↑	hypothetical protein M91_01397 [B. mutus]
ENSBTAP00000014410-D1	-1.22	2.28E-02	↑	Sodium/calcium exchanger 2 [B. mutus]

ENSP00000265299-D1	-1.22	2.28E-02	↑	Protein FAM188B, partial [B. mutus]
ENSBTAP00000051083-D1	-1.22	2.28E-02	↑	Unknown (protein for IMAGE:8833257) [B. taurus]
ENSP00000323678-D1	-1.22	2.28E-02	↑	Zinc-binding alcohol dehydrogenase domain-containing protein 2, partial [B. mutus]
ENSBTAP00000007046-D1	-1.22	2.28E-02	↑	Tubulin polymerization-promoting protein [B. mutus]
ENSBTAP00000029021-D1	-1.21	0.00E+00	↑	Mothers against decapentaplegic-like protein 5, partial [B. mutus]
ENSP00000379334-D2	-1.21	2.15E-128	↑	hypothetical protein M91_01585, partial [B. mutus]
ENSP00000348429-D1	-1.21	9.34E-28	↑	Long-chain-fatty-acid--CoA ligase 5, partial [B. mutus]
ENSP00000164227-D1	-1.21	7.52E-12	↑	B-cell lymphoma 3 protein, partial [B. mutus]
ENSP00000358404-D1	-1.21	1.15E-17	↑	Transcription factor 7-like 2 [B. mutus]
ENSBTAP00000049804-D2	-1.20	1.17E-107	↑	nuclear ubiquitous casein and cyclin-dependent kinase substrate 1-like [B. mutus]
ENSBTAP00000016629-D1	-1.20	3.97E-29	↑	amyloid beta A4 precursor protein-binding family B member 1-interacting protein [B. mutus]
ENSP00000300870-D85	-1.20	6.13E-108	↑	Zinc finger protein 354A, partial [B. mutus]
ENSBTAP00000039151-D1	-1.20	7.95E-39	↑	solute carrier family 23 member 2 isoform X1 [Trichechus manatus latirostris]
ENSBTAP00000009062-D1	-1.20	4.37E-07	↑	Nitric oxide synthase, inducible [B. mutus]
ENSBTAP00000003151-D1	-1.20	7.13E-149	↑	Transcription intermediary factor 1-alpha, partial [B. mutus]
ENSP00000362900-D1	-1.20	8.26E-44	↑	serine/arginine-rich splicing factor 4 [Mesocricetus auratus]
ENSBTAP00000010949-D1	-1.20	1.39E-63	↑	succinate dehydrogenase [ubiquinone] iron-sulfur subunit, mitochondrial precursor [B. taurus]
ENSP00000058691-D1	-1.20	1.13E-06	↑	Heme-binding protein 2, partial [B. mutus]
ENSBTAP00000021534-D1	-1.20	1.74E-18	↑	fibrinogen-like protein 1 isoform X1 [B. mutus]
ENSBTAP00000018050-D1	-1.20	6.83E-14	↑	Neutral amino acid transporter B(0), partial [B. mutus]
ENSP00000377626-D1	-1.20	1.27E-13	↑	Sodium/bile acid cotransporter 7, partial [B. mutus]
ENSBTAP00000000447-D1	-1.20	1.74E-13	↑	procollagen-lysine,2-oxoglutarate 5-dioxygenase 3 [B. mutus]
yakG007079	-1.20	8.03E-10	↑	hypothetical protein M91_03489 [B. mutus]
ENSP00000352540-D1	-1.20	2.05E-09	↑	epithelial membrane protein 2 [B. mutus]
ENSP00000252677-D1	-1.20	0.00E+00	↑	bone morphogenetic protein 15 [B. mutus]
ENSP00000331719-D1	-1.20	7.66E-06	↑	ethanolamine-phosphate cytidylyltransferase [B. mutus]
ENSBTAP00000053572-D1	-1.20	1.26E-107	↑	Ankyrin-3, partial [B. mutus]
ENSBTAP00000027006-D1	-1.19	4.00E-53	↑	thiamine transporter 1 [B. mutus]
ENSBTAP00000019629-D1	-1.19	0.00E+00	↑	A-kinase anchor protein 11, partial [B. mutus]
ENSP00000212355-D1	-1.19	9.19E-57	↑	transforming growth factor beta receptor type 3 [B. mutus]
ENSP00000386543-D1	-1.19	1.47E-62	↑	Prolyl endopeptidase-like protein [B. mutus]
ENSBTAP00000043609-D1	-1.19	9.27E-29	↑	exocyst complex component 1-like [Pantholops hodgsonii]
ENSP00000349722-D10	-1.19	1.70E-23	↑	ubiquitin-conjugating enzyme E2 D1 [Sorex araneus]
ENSP00000284563-D1	-1.19	1.76E-87	↑	protein enabled homolog isoform X7 [Pan paniscus]
ENSP00000253457-D1	-1.19	6.12E-52	↑	ER membrane protein complex subunit 8 [Sus scrofa]
ENSBTAP00000045803-D1	-1.19	4.49E-16	↑	splicing regulator RBM11 [B. mutus]
ENSBTAP00000012582-D1	-1.19	5.12E-58	↑	nesprin-2 [B. mutus]
ENSBTAP00000050695-D1	-1.19	1.23E-13	↑	Mitogen-activated protein kinase 1, partial [B. mutus]
ENSP00000404384-D1	-1.19	5.91E-07	↑	sulfatase-modifying factor 1 precursor [B. taurus]
ENSP00000358633-D1	-1.19	1.01E-04	↑	glucose-6-phosphate 1-dehydrogenase isoform X1 [B. taurus]
ENSBTAP00000020305-D1	-1.19	1.53E-56	↑	Succinate dehydrogenase assembly factor 2, mitochondrial [B. mutus]
ENSBTAP00000048583-D1	-1.19	1.55E-15	↑	hypothetical protein M91_07351, partial [B. mutus]
ENSBTAP00000037931-D1	-1.19	5.16E-09	↑	Ubiquitin-conjugating enzyme E2C-binding protein, partial [B. mutus]
ENSP00000373347-D1	-1.19	8.09E-07	↑	SLIT-ROBO Rho GTPase-activating protein 3 [B. mutus]
ENSP00000347441-D1	-1.19	5.16E-56	↑	hypothetical protein M91_09684, partial [B. mutus]
ENSP00000360722-D1	-1.18	1.32E-08	↑	14 kDa phosphohistidine phosphatase [Pantholops hodgsonii]
ENSP00000355160-D1	-1.18	1.02E-89	↑	Molybdopterin synthase catalytic subunit, partial [B. mutus]
ENSP00000378260-D1	-1.18	0.00E+00	↑	putative bifunctional UDP-N-acetylglucosamine transferase and deubiquitinase ALG13 isoform X1 [B. taurus]
ENSP00000222286-D1	-1.18	3.04E-10	↑	glyceraldehyde-3-phosphate dehydrogenase, testis-specific isoform X1 [B. mutus]
ENSBTAP00000017463-D1	-1.18	2.82E-102	↑	Tyrosyl-DNA phosphodiesterase 1, partial [B. mutus]
ENSBTAP00000035783-D1	-1.18	1.21E-13	↑	Glycine N-acyltransferase-like protein 3, partial [B. mutus]
ENSP00000345629-D1	-1.18	2.33E-31	↑	Mitogen-activated protein kinase kinase kinase 15, partial [B. mutus]
ENSBTAP00000017229-D1	-1.18	1.81E-34	↑	Bifunctional 3'-phosphoadenosine 5'-phosphosulfate synthase 1, partial [B. mutus]
ENSP00000356984-D1	-1.18	3.67E-136	↑	Death effector domain-containing protein [B. mutus]
ENSP00000332287-D1	-1.18	2.78E-20	↑	Synaptogyrin-1, partial [B. mutus]

ENSBTAP00000004182-D1	-1.18	1.58E-50	↑	Activating transcription factor 7-interacting protein 1 [B. mutus]
ENSBTAP00000009702-D1	-1.18	1.24E-36	↑	CAP-Gly domain-containing linker protein 4 [B. mutus]
ENSP00000324806-D1	-1.18	8.48E-180	↑	Glycogen synthase kinase-3 beta, partial [B. mutus]
ENSP00000356685-D1	-1.18	5.77E-31	↑	ankyrin repeat domain-containing protein 45 isoform X2 [B. taurus]
ENSBTAP00000042309-D1	-1.18	3.75E-04	↑	Gametogenetin [B. mutus]
ENSP00000343890-D1	-1.18	3.75E-04	↑	ceramide-1-phosphate transfer protein [B. mutus]
ENSBTAP00000016909-D1	-1.18	8.68E-14	↑	4-hydroxy-2-oxoglutarate aldolase, mitochondrial [B. mutus]
ENSBTAP00000027169-D1	-1.18	0.00E+00	↑	OX-2 membrane glycoprotein, partial [B. mutus]
ENSP00000263574-D1	-1.18	3.05E-26	↑	amyloid-like protein 2 isoform X1 [B. mutus]
ENSBTAP00000043851-D1	-1.18	4.77E-59	↑	Sciellin [B. mutus]
ENSP00000221418-D1	-1.18	2.24E-21	↑	Delta(3,5)-Delta(2,4)-dienoyl-CoA isomerase, mitochondrial, partial [B. mutus]
ENSBTAP00000009899-D1	-1.18	3.79E-15	↑	syntaxin-4 [B. taurus]
ENSBTAP00000023186-D1	-1.18	3.54E-170	↑	Cysteine dioxygenase type 1 [B. mutus]
ENSP00000209728-D1	-1.17	4.40E-275	↑	cell division control protein 6 homolog [B. mutus]
ENSP00000357285-D1	-1.17	3.30E-113	↑	Mitogen-activated protein-binding protein-interacting protein, partial [B. mutus]
ENSP00000265085-D1	-1.17	0.00E+00	↑	cytoplasmic polyadenylation element-binding protein 4 [B. mutus]
ENSBTAP00000004805-D1	-1.17	8.58E-42	↑	Forkhead box protein K2, partial [B. mutus]
ENSP00000339051-D1	-1.17	1.96E-05	↑	Translationally-controlled tumor protein, partial [B. mutus]
ENSBTAP00000016884-D3	-1.17	0.00E+00	↑	Lactoylglutathione lyase, partial [B. mutus]
ENSBTAP00000017155-D1	-1.17	1.87E-50	↑	heat shock 70 kDa protein 13 precursor [B. taurus]
ENSP00000358147-D1	-1.17	4.55E-08	↑	CDK2-associated and cullin domain-containing protein 1 [B. mutus]
ENSP00000364004-D1	-1.17	1.09E-06	↑	rho guanine nucleotide exchange factor 9 isoform X1 [Bubalus bubalis]
ENSBTAP00000015290-D1	-1.17	1.42E-247	↑	Rab GTPase-binding effector protein 1, partial [B. mutus]
ENSBTAP00000028887-D1	-1.17	3.72E-05	↑	High affinity copper uptake protein 1, partial [B. mutus]
ENSBTAP00000029308-D1	-1.17	2.05E-243	↑	Ras association domain-containing protein 8, partial [B. mutus]
ENSBTAP00000008386-D1	-1.17	1.21E-77	↑	Glucose-6-phosphate isomerase, partial [B. mutus]
ENSP00000258198-D1	-1.17	9.83E-165	↑	cytoplasmic dynein 1 light intermediate chain 2 [Bison bison bison]
ENSP00000353410-D11	-1.17	1.03E-03	↑	zinc finger protein 347 isoform X1 [B. taurus]
ENSP00000246747-D1	-1.16	1.73E-08	↑	ADP-ribosylation factor-like protein 2 [B. taurus]
ENSBTAP00000002168-D1	-1.16	6.39E-132	↑	beta-2-syntrophin [B. taurus]
ENSP00000361803-D1	-1.16	4.01E-70	↑	stromal membrane-associated protein 2 [B. mutus]
ENSP00000331845-D1	-1.16	1.66E-101	↑	protein PRR14L [B. mutus]
ENSP00000368439-D1	-1.16	9.04E-12	↑	choline-phosphate cytidylyltransferase B [B. taurus]
ENSBTAP00000013086-D1	-1.16	8.79E-107	↑	protein N-terminal glutamine amidohydrolase isoform X1 [B. taurus]
ENSP00000220325-D1	-1.16	5.96E-14	↑	EH domain-containing protein 4 [B. taurus]
ENSBTAP00000007075-D1	-1.16	3.32E-117	↑	F-box only protein 30 [B. mutus]
ENSP00000371734-D1	-1.16	1.81E-28	↑	KN motif and ankyrin repeat domain-containing protein 1, partial [B. mutus]
ENSP00000366035-D1	-1.16	1.19E-40	↑	Alpha-actinin-1 [B. mutus]
ENSBTAP00000025155-D1	-1.16	0.00E+00	↑	AT-rich interactive domain-containing protein 4A, partial [B. mutus]
ENSP00000255977-D1	-1.16	1.80E-20	↑	E3 ubiquitin-protein ligase makorin-1, partial [B. mutus]
ENSP00000358266-D1	-1.16	2.07E-60	↑	Actin-binding LIM protein 1 [B. mutus]
ENSP00000397107-D1	-1.16	2.86E-10	↑	LETM1 domain-containing protein 1 [B. mutus]
ENSP00000356793-D1	-1.16	9.88E-05	↑	lymphotactin [B. taurus]
ENSBTAP00000053074-D1	-1.16	1.44E-03	↑	hypothetical protein M91_08196, partial [B. mutus]
ENSBTAP00000013069-D3	-1.16	1.44E-03	↑	Transcription factor Spi-C, partial [B. mutus]
ENSBTAP00000043465-D1	-1.16	2.67E-52	↑	Protein MRVI1 [B. mutus]
ENSBTAP00000046597-D1	-1.16	1.12E-91	↑	ubiquitin-protein ligase E3A [B. taurus]
ENSBTAP00000000541-D1	-1.16	1.17E-76	↑	RNA-binding protein 4 [B. taurus]
ENSBTAP00000028030-D1	-1.16	1.03E-18	↑	Transcription factor E2F3, partial [B. mutus]
ENSP00000407802-D1	-1.16	2.12E-63	↑	Pleckstrin-like protein domain-containing family A member 8, partial [B. mutus]
ENSP00000324419-D1	-1.16	5.83E-51	↑	TPA: synaptotagmin IX [B. taurus]
ENSP00000321507-D1	-1.16	3.19E-215	↑	Antizyme inhibitor 1 [B. mutus]
ENSBTAP00000024666-D1	-1.16	2.61E-18	↑	immunoglobulin J chain precursor [B. taurus]
ENSP00000374309-D1	-1.16	1.07E-24	↑	laminin subunit alpha-1 [B. mutus]
ENSBTAP00000021080-D1	-1.16	4.61E-47	↑	pre-rRNA-processing protein TSR2 homolog [B. mutus]

ENSBTAP00000025619-D2	-1.15	1.21E-99	↑	biogenesis of lysosome-related organelles complex 1 subunit 5 [B. mutus]
ENSBTAP00000011671-D1	-1.15	0.00E+00	↑	Transmembrane protein 87A, partial [B. mutus]
ENSP00000346890-D1	-1.15	1.21E-115	↑	Nardilysin [B. mutus]
ENSP00000363617-D1	-1.15	4.51E-42	↑	ras-associated and pleckstrin homology domains-containing protein 1 isoform X1 [Homo sapiens]
ENSBTAP00000041052-D1	-1.15	4.92E-24	↑	WASH complex subunit 7 [B. taurus]
ENSBTAP00000020111-D1	-1.15	5.00E-67	↑	calreticulin [B. mutus]
ENSP00000341351-D1	-1.15	5.17E-19	↑	DDB1- and CUL4-associated factor 5 isoform X2 [B. mutus]
ENSP00000367284-D2	-1.15	2.03E-78	↑	FUN14 domain-containing protein 1 [Ovis aries]
ENSBTAP00000001883-D1	-1.15	2.18E-60	↑	hypothetical protein M91_15495, partial [B. mutus]
ENSBTAP00000042444-D1	-1.15	1.83E-21	↑	high mobility group nucleosome-binding domain-containing protein 4 [B. mutus]
ENSP00000387262-D1	-1.15	7.66E-63	↑	Mitochondrial inner membrane protein, partial [B. mutus]
ENSP00000401740-D1	-1.15	5.48E-122	↑	Tudor domain-containing protein 3, partial [B. mutus]
ENSBTAP00000011038-D1	-1.15	0.00E+00	↑	ATP-dependent Clp protease ATP-binding subunit clpX-like, mitochondrial [Capra hircus]
ENSP00000386074-D1	-1.15	7.41E-55	↑	putative tRNA pseudouridine synthase Pus10 [B. mutus]
ENSP00000305899-D1	-1.15	0.00E+00	↑	Histone-lysine N-methyltransferase SUV420H1 [B. mutus]
ENSP00000307041-D1	-1.15	3.63E-05	↑	Zinc finger protein basonuclin-1, partial [B. mutus]
ENSBTAP00000024427-D1	-1.15	1.05E-19	↑	transmembrane protein 109 precursor [B. taurus]
ENSBTAP00000046195-D1	-1.15	1.28E-51	↑	angiogenic factor with G patch and FHA domains 1-like [B. mutus]
ENSBTAP00000009097-D1	-1.15	0.00E+00	↑	ornithine aminotransferase, mitochondrial isoform X1 [Capra hircus]
ENSBTAP00000020947-D1	-1.15	4.29E-40	↑	hippocampus abundant transcript 1 protein [B. mutus]
ENSP00000333938-D1	-1.15	3.38E-68	↑	Nexilin, partial [B. mutus]
ENSP00000385699-D1	-1.14	7.17E-06	↑	Fatty aldehyde dehydrogenase [B. mutus]
ENSP00000386800-D1	-1.14	5.84E-234	↑	pleckstrin homology domain-containing family A member 1 isoform X1 [B. taurus]
ENSP00000376117-D1	-1.14	3.55E-220	↑	protein boule-like isoform X2 [Cricetulus griseus]
ENSP00000342876-D1	-1.14	1.45E-31	↑	Zinc finger protein Helios [Tupaia chinensis]
ENSP00000359446-D1	-1.14	2.56E-13	↑	carboxypeptidase N catalytic chain isoform X1 [B. taurus]
ENSP00000262776-D1	-1.14	3.48E-13	↑	galectin-3-binding protein [B. mutus]
ENSP00000358823-D1	-1.14	1.36E-05	↑	deoxyribonuclease-1-like 1 [B. mutus]
ENSP00000225428-D1	-1.14	0.00E+00	↑	Pre-mRNA-splicing factor CWC25-like protein, partial [B. mutus]
ENSBTAP00000003816-D1	-1.14	2.55E-31	↑	hypothetical protein M91_04450, partial [B. mutus]
ENSP00000399632-D2	-1.14	0.00E+00	↑	hypothetical protein M91_09126, partial [B. mutus]
ENSBTAP00000023969-D1	-1.14	6.94E-20	↑	COUP transcription factor 2, partial [B. mutus]
ENSBTAP00000029470-D1	-1.14	5.09E-17	↑	Coiled-coil domain-containing protein 117, partial [B. mutus]
ENSBTAP00000034422-D1	-1.14	1.05E-26	↑	Sestrin-1 [B. mutus]
ENSP00000295087-D1	-1.14	2.37E-65	↑	ADP-ribosylation factor-like protein 5A, partial [B. mutus]
yakG037648	-1.13	6.15E-09	↑	hypothetical protein M91_14387 [B. mutus]
ENSBTAP00000020148-D1	-1.13	0.00E+00	↑	Protein S100-A11, partial [B. mutus]
ENSP00000377371-D1	-1.13	8.74E-46	↑	CREB3 regulatory factor [B. taurus]
ENSP00000312435-D1	-1.13	2.69E-14	↑	dystroglycan [B. mutus]
ENSBTAP00000016697-D1	-1.13	7.11E-04	↑	Putative protein KIAA1530, partial [B. mutus]
ENSBTAP00000016997-D2	-1.13	2.02E-111	↑	Serine/arginine-rich splicing factor 9, partial [B. mutus]
ENSP00000381167-D1	-1.13	1.57E-45	↑	Cytoplasmic dynein 2 heavy chain 1 [B. mutus]
ENSBTAP00000008944-D1	-1.13	2.09E-83	↑	transmembrane protein 106A [B. mutus]
ENSP00000344166-D1	-1.13	0.00E+00	↑	Transmembrane protein 189, partial [B. mutus]
ENSBTAP00000048203-D1	-1.13	1.87E-50	↑	selenophosphate synthetase 2 [B. mutus]
ENSP00000284273-D1	-1.13	2.03E-83	↑	ubiquitin-associated and SH3 domain-containing protein B [B. mutus]
ENSBTAP00000005076-D1	-1.13	4.82E-17	↑	Period circadian protein-like protein 1, partial [B. mutus]
ENSP00000316032-D1	-1.13	8.94E-56	↑	nuclear pore complex protein Nup98-Nup96 isoform X2 [B. taurus]
ENSP00000037243-D5	-1.13	8.61E-85	↑	Chain A, Crystal Structure Of The Gaba(A) Receptor Associated Protein, Gabarap
ENSBTAP00000001069-D1	-1.13	7.76E-28	↑	cytochrome c oxidase assembly protein COX14 isoform X2 [B. taurus]
ENSBTAP00000045864-D1	-1.13	2.98E-12	↑	uncharacterized protein LOC100126544 [B. taurus]
ENSBTAP00000050375-D1	-1.13	9.83E-78	↑	General transcription factor IIE subunit 1 [B. mutus]
ENSP00000386880-D1	-1.13	3.61E-48	↑	COP9 signalosome complex subunit 7b [B. mutus]
ENSBTAP00000006725-D1	-1.13	1.45E-21	↑	death domain-containing protein CRADD [B. taurus]
ENSBTAP00000041906-D1	-1.13	2.32E-202	↑	Malate dehydrogenase, mitochondrial, partial [B. mutus]

ENSBTAP00000002687-D1	-1.12	4.20E-35	↑	lysine-specific demethylase 4A isoform X1 [B. mutus]
ENSP00000322439-D1	-1.12	4.73E-30	↑	Elongation factor Tu, mitochondrial, partial [B. mutus]
ENSBTAP00000050369-D3	-1.12	7.28E-104	↑	hypothetical protein M91_21309, partial [B. mutus]
ENSBTAP00000027016-D1	-1.12	9.78E-15	↑	nuclear factor NF-kappa-B p105 subunit isoform X1 [B. mutus]
ENSBTAP00000002998-D1	-1.12	1.36E-222	↑	Ubiquitin carboxyl-terminal hydrolase 28, partial [B. mutus]
ENSP00000265070-D3	-1.12	1.75E-17	↑	Golgi phosphoprotein 3, partial [B. mutus]
ENSBTAP00000024313-D1	-1.12	0.00E+00	↑	WW domain-containing adapter protein with coiled-coil, partial [B. mutus]
ENSP00000415453-D31	-1.12	7.60E-106	↑	Zinc finger protein 82-like protein, partial [B. mutus]
ENSP00000303844-D1	-1.12	0.00E+00	↑	cellular nucleic acid-binding protein isoform X1 [Balaenoptera acutorostrata scammoni]
ENSBTAP00000053545-D1	-1.12	2.81E-97	↑	Msx2-interacting protein, partial [B. mutus]
ENSP00000343636-D1	-1.12	1.57E-16	↑	GDNF family receptor alpha-like protein, partial [B. mutus]
ENSP00000371221-D1	-1.12	1.70E-17	↑	Myotubularin-related protein 6, partial [B. mutus]
ENSP00000407477-D1	-1.12	3.86E-71	↑	cell division cycle 7-related protein kinase [Bison bison bison]
ENSP00000354718-D1	-1.12	1.40E-07	↑	A-kinase anchor protein 13 [B. mutus]
ENSBTAP00000040158-D1	-1.12	1.41E-61	↑	DEP domain-containing protein 1A-like isoform X1 [B. mutus]
ENSBTAP00000042502-D4	-1.11	1.09E-23	↑	Pancreatic progenitor cell differentiation and proliferation factor [B. mutus]
ENSBTAP00000032189-D1	-1.11	1.15E-09	↑	HRAS-like suppressor [B. mutus]
ENSP00000398748-D1	-1.11	6.57E-22	↑	metaxin-3-like isoform X2 [B. mutus]
ENSBTAP00000050584-D1	-1.11	1.91E-07	↑	NEDD4-binding protein 2-like 1, partial [B. mutus]
ENSP00000378920-D1	-1.11	9.26E-06	↑	Solute carrier family 26 member 6 [B. mutus]
ENSBTAP00000025560-D1	-1.11	2.02E-08	↑	ELL-associated factor 1, partial [B. mutus]
ENSBTAP00000006689-D1	-1.11	2.75E-08	↑	39S ribosomal protein L49, mitochondrial isoform X1 [B. mutus]
ENSP00000411822-D1	-1.11	3.14E-10	↑	Pre-mRNA-splicing factor ISY1-like protein, partial [B. mutus]
yakG034595	-1.11	0.00E+00	↑	Small EDRK-rich factor 2 [B. mutus]
ENSP00000410208-D1	-1.11	7.53E-79	↑	Golgi reassembly-stacking protein 2, partial [B. mutus]
ENSBTAP00000023778-D1	-1.11	2.45E-118	↑	T-complex protein 1 subunit epsilon, partial [B. mutus]
ENSBTAP00000002986-D1	-1.11	0.00E+00	↑	E3 ubiquitin-protein ligase RNF34, partial [B. mutus]
ENSP00000257290-D1	-1.10	1.12E-25	↑	platelet-derived growth factor receptor alpha [B. mutus]
ENSP00000377044-D1	-1.10	8.59E-23	↑	TPA: ring finger protein 130 [B. taurus]
ENSBTAP00000016504-D1	-1.10	9.12E-12	↑	Squalene synthase [B. mutus]
ENSBTAP00000019254-D1	-1.10	2.72E-46	↑	gamma-tubulin complex component 4 [B. mutus]
ENSP00000351596-D1	-1.10	1.42E-109	↑	tetratricopeptide repeat protein 37 [B. mutus]
ENSBTAP00000009083-D1	-1.10	1.25E-55	↑	phosphatidylinositol 4,5-bisphosphate 3-kinase catalytic subunit beta isoform [B. taurus]
ENSBTAP00000033568-D1	-1.10	2.83E-09	↑	ataxin-7-like protein 2 [B. taurus]
ENSP00000386759-D1	-1.10	2.16E-22	↑	Histone-lysine N-methyltransferase SETD2, partial [B. mutus]
ENSP00000401950-D1	-1.10	6.57E-05	↑	uncharacterized protein LOC101288956 [Orcinus orca]
ENSBTAP00000010937-D1	-1.10	6.24E-31	↑	1-phosphatidylinositol 4,5-bisphosphate phosphodiesterase eta-1 [B. mutus]
ENSBTAP00000000479-D1	-1.10	1.82E-46	↑	epidermal growth factor receptor kinase substrate 8 isoform X2 [Bubalus bubalis]
ENSP00000222008-D1	-1.10	1.36E-61	↑	prenylated Rab acceptor protein 1 [B. taurus]
ENSP00000390696-D1	-1.10	1.15E-112	↑	transmembrane protein 168 [B. taurus]
ENSBTAP00000023563-D1	-1.10	4.31E-39	↑	RNA-binding Raly-like protein, partial [B. mutus]
ENSP00000265428-D1	-1.10	3.14E-69	↑	NEDD4-like E3 ubiquitin-protein ligase WWP1 [B. mutus]
ENSP00000386311-D1	-1.10	1.12E-57	↑	Ly6/PLAUR domain-containing protein 1, partial [B. mutus]
ENSP00000347198-D1	-1.10	1.42E-306	↑	SLIT-ROBO Rho GTPase-activating protein 1 [B. taurus]
ENSBTAP00000048712-D1	-1.10	9.20E-13	↑	Nuclear receptor-binding factor 2 [B. mutus]
ENSBTAP00000029240-D1	-1.10	3.34E-05	↑	arachidonate 12-lipoxygenase, 12S-type [B. mutus]
ENSBTAP00000053751-D1	-1.09	0.00E+00	↑	STE20/SPS1-related proline-alanine-rich protein kinase [Bubalus bubalis]
ENSBTAP00000016449-D1	-1.09	1.64E-47	↑	Deoxycytidine kinase [B. mutus]
ENSBTAP00000010690-D1	-1.09	9.82E-09	↑	mediator of RNA polymerase II transcription subunit 10 [Homo sapiens]
ENSP00000360916-D1	-1.09	1.24E-05	↑	Guanine nucleotide exchange factor VAV2, partial [B. mutus]
ENSBTAP00000025170-D1	-1.09	7.34E-73	↑	Ras-related protein Rab-25, partial [B. mutus]
ENSBTAP00000006842-D1	-1.09	8.79E-157	↑	Hamartin [B. mutus]
ENSBTAP00000008425-D1	-1.09	5.02E-14	↑	Troponin I, cardiac muscle, partial [B. mutus]
ENSBTAP00000053279-D1	-1.09	2.66E-43	↑	small integral membrane protein 4 [B. taurus]
ENSBTAP00000021408-D1	-1.09	8.33E-172	↑	ras-related protein Rab-8A [B. taurus]

ENSBTAP00000010613-D1	-1.09	2.28E-188	↑	endoplasmic reticulum lectin 1 isoform X1 [B. taurus]
ENSBTAP00000029015-D1	-1.09	6.47E-149	↑	proheparin-binding EGF-like growth factor [B. mutus]
ENSP00000321195-D1	-1.09	1.18E-92	↑	probable phospholipid-transporting ATPase IF isoform X1 [B. taurus]
ENSP00000411524-D1	-1.09	0.00E+00	↑	chromobox protein homolog 5 [Homo sapiens]
ENSP00000305426-D1	-1.09	9.28E-23	↑	tubby protein homolog [B. mutus]
ENSBTAP00000020219-D1	-1.09	2.03E-20	↑	Niemann-Pick C1 protein, partial [B. mutus]
ENSBTAP00000003211-D1	-1.09	1.22E-06	↑	Coiled-coil domain-containing protein 78, partial [B. mutus]
ENSP00000320006-D1	-1.09	6.17E-44	↑	multiple PDZ domain protein isoform X3 [B. taurus]
ENSBTAP00000006918-D2	-1.08	2.78E-55	↑	Mitochondrial uncoupling protein 3, partial [B. mutus]
ENSBTAP00000047701-D1	-1.08	8.20E-244	↑	guanine nucleotide-binding protein G(I)/G(S)/G(O) subunit gamma-12 precursor [B. taurus]
ENSBTAP00000017860-D1	-1.08	1.67E-06	↑	glutaredoxin 5 [B. mutus]
ENSP00000341779-D1	-1.08	5.77E-70	↑	serine/threonine-protein phosphatase PP1-gamma catalytic subunit [Pantholops hodgsonii]
ENSP00000379654-D1	-1.08	2.42E-33	↑	meiosis arrest female protein 1 isoform X1 [B. mutus]
ENSBTAP00000020387-D1	-1.08	1.38E-10	↑	Brain-specific angiogenesis inhibitor 3, partial [B. mutus]
ENSP00000385328-D1	-1.08	3.22E-05	↑	E3 ubiquitin-protein ligase RNF43 [B. mutus]
ENSP00000361242-D1	-1.08	0.00E+00	↑	Putative helicase senataxin [B. mutus]
ENSBTAP00000000533-D1	-1.08	0.00E+00	↑	protein LAP2 isoform X3 [Bison bison bison]
ENSBTAP00000019999-D1	-1.08	2.30E-06	↑	trafficking protein particle complex subunit 6B [B. taurus]
ENSP00000411851-D1	-1.08	0.00E+00	↑	hypothetical protein M91_06863, partial [B. mutus]
ENSP00000260408-D1	-1.08	1.55E-139	↑	disintegrin and metalloproteinase domain-containing protein 10 [B. mutus]
ENSBTAP00000053245-D1	-1.08	1.74E-30	↑	PH domain leucine-rich repeat-containing protein phosphatase 2 [B. taurus]
ENSBTAP00000037188-D1	-1.08	1.96E-22	↑	Vacuolar protein sorting-associated protein 28-like protein [B. mutus]
ENSBTAP00000033613-D1	-1.08	2.50E-45	↑	Peptidyl-prolyl cis-trans isomerase FKBP9, partial [B. mutus]
ENSBTAP00000012776-D1	-1.08	1.83E-36	↑	F-box only protein 15 [B. mutus]
ENSP00000355601-D1	-1.07	1.18E-07	↑	egl nine homolog 1 isoform X1 [Ovis aries]
ENSBTAP00000009073-D1	-1.07	7.22E-63	↑	L-asparaginase, partial [B. mutus]
ENSBTAP00000011385-D1	-1.07	3.14E-07	↑	unnamed protein product [Mus musculus]
ENSP00000371735-D1	-1.07	9.22E-17	↑	Sacsin, partial [B. mutus]
ENSP00000281623-D1	-1.07	1.17E-191	↑	F-box only protein 4 [B. taurus]
ENSBTAP00000040188-D1	-1.07	4.07E-03	↑	BTB/POZ domain-containing protein KCTD2 [Ceratotherium simum simum]
ENSP00000253814-D1	-1.07	7.29E-51	↑	NEDD4 family-interacting protein 1 isoform X1 [Ovis aries]
ENSP00000348128-D1	-1.07	7.19E-52	↑	supervillin [B. mutus]
ENSP00000350667-D3	-1.07	3.09E-70	↑	Tropomyosin alpha-1 chain [B. mutus]
ENSBTAP00000045871-D1	-1.07	4.57E-10	↑	high mobility group AT-hook 1, isoform CRA_c, partial [Homo sapiens]
ENSP00000391106-D1	-1.07	5.77E-44	↑	Myosin-X, partial [B. mutus]
ENSP00000316490-D1	-1.07	8.22E-120	↑	histone RNA hairpin-binding protein [B. mutus]
ENSP00000362649-D2	-1.07	8.65E-41	↑	histone deacetylase 1 [Fukomys damarensis]
ENSP00000379838-D1	-1.06	6.65E-12	↑	calcium-regulated heat stable protein 1 isoform X1 [Pantholops hodgsonii]
ENSP00000327255-D1	-1.06	7.29E-83	↑	protein phosphatase 1A isoform X2 [Ovis aries musimon]
ENSP00000242576-D1	-1.06	1.98E-33	↑	uracil-DNA glycosylase [B. mutus]
ENSBTAP00000044574-D1	-1.06	2.91E-58	↑	Nuclear factor erythroid 2-related factor 2, partial [B. mutus]
ENSBTAP00000015301-D1	-1.06	1.14E-189	↑	39S ribosomal protein L44, mitochondrial, partial [B. mutus]
ENSP00000251507-D1	-1.06	2.07E-87	↑	rab GTPase-activating protein 1-like, isoform 10-like isoform X1 [B. mutus]
ENSP00000358081-D1	-1.06	7.99E-07	↑	BAG family molecular chaperone regulator 3 [B. mutus]
ENSBTAP00000014523-D1	-1.06	0.00E+00	↑	mRNA export factor isoform X1 [Capra hircus]
ENSP00000375503-D1	-1.06	8.92E-26	↑	Putative PIP5K1A and PSMD4-like protein, partial [B. mutus]
yakG038520	-1.06	1.91E-28	↑	calponin-3-like [B. mutus]
ENSP00000226319-D1	-1.06	1.79E-20	↑	Protein Jade-1 [B. mutus]
ENSP00000314193-D1	-1.06	0.00E+00	↑	WD repeat-containing protein 75 [B. mutus]
ENSBTAP00000010680-D1	-1.06	1.74E-12	↑	R-spondin-3 [Ovis aries]
ENSBTAP00000008682-D1	-1.06	1.09E-07	↑	hepatic leukemia factor [Ovis aries]
ENSBTAP00000012701-D1	-1.06	1.20E-76	↑	solute carrier family 12 member 2 isoform X1 [B. taurus]
ENSP00000310126-D1	-1.06	2.90E-56	↑	leucine-rich repeat and immunoglobulin-like domain-containing nogo receptor-interacting protein 2-like [B. mutus]
ENSBTAP00000011029-D1	-1.06	1.54E-69	↑	Calpastatin, partial [B. mutus]
ENSP00000341805-D1	-1.06	4.54E-12	↑	PHD finger protein 10, partial [B. mutus]

ENSP00000397054-D1	-1.06	1.56E-09	↑	hypothetical protein M91_04327, partial [B. mutus]
ENSBTAP00000006308-D1	-1.06	4.33E-26	↑	RNA polymerase I-specific transcription initiation factor RRN3 [B. mutus]
ENSBTAP00000014776-D1	-1.06	4.21E-39	↑	Serine/threonine-protein kinase 38 [B. mutus]
ENSP00000349892-D1	-1.06	6.39E-41	↑	E3 ubiquitin-protein ligase MYCBP2 [Bison bison bison]
ENSP00000408108-D1	-1.06	3.84E-141	↑	WD repeat domain 26 variant [Homo sapiens]
ENSP00000354964-D1	-1.06	7.34E-31	↑	Zinc finger protein 318, partial [B. mutus]
ENSBTAP00000037404-D1	-1.06	1.75E-13	↑	Protein phosphatase 1 regulatory subunit 14B, partial [B. mutus]
ENSP00000342307-D1	-1.06	4.61E-04	↑	Forkhead box protein M1 [B. mutus]
ENSBTAP00000001064-D1	-1.06	7.74E-08	↑	zinc finger protein 667 [B. taurus]
ENSBTAP00000041750-D1	-1.06	1.40E-72	↑	Cyclin-I2 [B. mutus]
ENSBTAP00000020987-D1	-1.05	5.77E-10	↑	cyclic AMP-responsive element-binding protein 3-like protein 2 [Capra hircus]
ENSBTAP00000021640-D1	-1.05	2.08E-08	↑	DNA repair protein XRCC1, partial [B. mutus]
ENSP00000286424-D1	-1.05	1.53E-191	↑	Transmembrane BAX inhibitor motif-containing protein 4 [B. mutus]
ENSP00000420716-D1	-1.05	1.16E-04	↑	uncharacterized protein C1orf228 homolog [B. taurus]
ENSP00000312734-D1	-1.05	2.25E-103	↑	ras-related protein Rab-8B [B. mutus]
ENSBTAP00000014026-D1	-1.05	1.52E-05	↑	DNA damage-inducible transcript 4-like protein [B. taurus]
ENSBTAP00000027473-D1	-1.05	5.55E-10	↑	diacylglycerol kinase delta [B. taurus]
ENSBTAP00000008655-D1	-1.05	1.41E-273	↑	Cystic fibrosis transmembrane conductance regulator, partial [B. mutus]
ENSP00000257821-D1	-1.05	1.31E-36	↑	PHD finger protein 21A, partial [B. mutus]
ENSP00000330965-D1	-1.05	6.35E-04	↑	uncharacterized protein LOC101125104 isoform 1 [Gorilla gorilla gorilla]
ENSP00000330307-D4	-1.05	5.89E-03	↑	histone H2A type 1-like [B. mutus]
ENSP00000405275-D1	-1.05	1.59E-04	↑	proline-rich protein 3 isoform X2 [Pantholops hodgsonii]
ENSP00000393641-D1	-1.04	0.00E+00	↑	E3 ubiquitin-protein ligase Arkadia isoform X1 [B. mutus]
ENSP00000351813-D1	-1.04	4.58E-31	↑	E3 ubiquitin-protein ligase MARCH5 [B. mutus]
ENSP00000242210-D2	-1.04	1.38E-10	↑	Cytosolic 5'-nucleotidase 3, partial [B. mutus]
ENSBTAP00000026265-D2	-1.04	7.45E-14	↑	trans-acting T-cell-specific transcription factor GATA-3 isoform X2 [B. taurus]
ENSP00000281950-D1	-1.04	5.77E-41	↑	gem-associated protein 6 [B. taurus]
ENSP00000299736-D1	-1.04	0.00E+00	↑	Centromere protein V, partial [B. mutus]
ENSP00000283131-D1	-1.04	0.00E+00	↑	SWI/SNF-related matrix-associated actin-dependent regulator of chromatin subfamily A member 5-like [B. mutus]
ENSP00000362768-D1	-1.04	1.45E-16	↑	retinoblastoma-like protein 1 [B. taurus]
ENSP00000328004-D2	-1.04	1.92E-98	↑	RING1 and YY1-binding protein isoform X2 [Latimeria chalumnae]
ENSBTAP00000009250-D1	-1.04	1.90E-41	↑	3-oxoacyl-[acyl-carrier-protein] synthase, mitochondrial [B. mutus]
ENSBTAP00000019438-D1	-1.04	1.67E-170	↑	vesicle-associated membrane protein 3 [Pantholops hodgsonii]
ENSBTAP00000047771-D1	-1.04	5.05E-13	↑	olfactory receptor 5M9-like [Bison bison bison]
ENSP00000310042-D1	-1.04	3.62E-29	↑	zinc finger protein 622 [Bison bison bison]
ENSBTAP00000032374-D1	-1.03	7.16E-106	↑	neuroblastoma-amplified sequence [B. mutus]
ENSBTAP00000019643-D1	-1.03	5.05E-79	↑	CD9 antigen, partial [B. mutus]
ENSBTAP00000043789-D1	-1.03	6.60E-11	↑	Apolipoprotein D, partial [B. mutus]
ENSP00000302239-D1	-1.03	0.00E+00	↑	ubiquitin carboxyl-terminal hydrolase 8 [B. mutus]
ENSBTAP00000041594-D1	-1.03	1.67E-35	↑	transient receptor potential cation channel subfamily M member 7 [B. mutus]
ENSBTAP00000012106-D1	-1.03	8.92E-13	↑	DnaJ-like protein subfamily C member 28, partial [B. mutus]
ENSBTAP00000012523-D2	-1.03	0.00E+00	↑	acyl-CoA-binding protein [B. taurus]
ENSP00000302108-D1	-1.03	1.71E-12	↑	pantothenate kinase 1 [Nomascus leucogenys]
ENSP00000229971-D1	-1.03	5.77E-299	↑	F-box/LRR-repeat protein 4 [B. taurus]
ENSBTAP00000017839-D1	-1.03	9.30E-183	↑	Alpha-enolase [B. mutus]
ENSP00000252603-D1	-1.03	2.52E-23	↑	6-phosphogluconolactonase [B. mutus]
ENSP00000330937-D1	-1.03	1.11E-36	↑	NADH dehydrogenase [ubiquinone] 1 alpha subcomplex subunit 6 [B. mutus]
ENSP00000378654-D1	-1.03	2.64E-78	↑	Rap guanine nucleotide exchange factor 6, partial [B. mutus]
ENSBTAP00000031555-D1	-1.03	4.80E-26	↑	Microcephalin, partial [B. mutus]
ENSP00000386987-D1	-1.03	3.29E-50	↑	FCH and double SH3 domains protein 2 isoform X1 [B. mutus]
ENSBTAP00000001303-D1	-1.03	2.60E-217	↑	ras-related protein Rab-10 [Mus musculus]
ENSBTAP00000029514-D1	-1.03	3.03E-04	↑	Ras-related protein Rab-35, partial [B. mutus]
ENSP00000387307-D1	-1.02	5.21E-77	↑	ras-related protein Rab-6C [Canis lupus familiaris]
ENSBTAP00000016535-D1	-1.02	3.34E-07	↑	zinc finger matrin-type protein 3 isoform X1 [Bubalus bubalis]
ENSP00000356696-D1	-1.02	2.08E-134	↑	Protein osteopotentia-like protein, partial [B. mutus]

ENSP00000362085-D1	-1.02	1.78E-49	↑	UPF0184 protein C9orf16 homolog [B. taurus]
ENSBTAP00000043415-D14	-1.02	0.00E+00	↑	ferritin light chain-like [Bubalus bubalis]
ENSBTAP00000022140-D1	-1.02	2.80E-16	↑	Aminopeptidase Q, partial [B. mutus]
ENSP00000358437-D1	-1.02	5.28E-39	↑	T-box transcription factor TBX15 isoform X1 [Ovis aries]
ENSBTAP00000028174-D1	-1.02	3.72E-23	↑	protein RMD5 homolog A [Homo sapiens]
ENSP00000254190-D1	-1.02	5.12E-37	↑	Chondroitin sulfate synthase 1, partial [B. mutus]
ENSBTAP00000003681-D1	-1.02	5.86E-149	↑	HEAT repeat-containing protein 4, partial [B. mutus]
ENSBTAP00000010041-D1	-1.02	7.20E-14	↑	Protein FAM151B [B. mutus]
ENSBTAP00000015625-D1	-1.02	0.00E+00	↑	MAGUK p55 subfamily member 5 [B. mutus]
ENSP00000351015-D1	-1.02	1.15E-69	↑	Neurofibromin, partial [B. mutus]
ENSP00000296591-D1	-1.02	1.74E-21	↑	EGF-like repeat and discoidin I-like domain-containing protein 3, partial [B. mutus]
ENSBTAP00000011134-D1	-1.02	7.06E-95	↑	Large subunit GTPase 1-like protein [B. mutus]
ENSP00000368540-D1	-1.02	1.48E-51	↑	Ubiquitin-associated protein 2 [B. mutus]
ENSP00000357484-D1	-1.01	7.11E-75	↑	golgi-associated PDZ and coiled-coil motif-containing protein isoform X1 [Bubalus bubalis]
ENSBTAP00000022114-D4	-1.01	4.70E-16	↑	ADP-ribosylation factor 6 [Homo sapiens]
ENSBTAP00000028964-D1	-1.01	5.73E-08	↑	2-oxoglutarate dehydrogenase-like, mitochondrial, partial [B. mutus]
ENSBTAP00000048673-D1	-1.01	8.35E-145	↑	Clusterin-associated protein 1 [B. mutus]
ENSP00000384273-D1	-1.01	2.10E-08	↑	Transcription factor p65, partial [B. mutus]
ENSBTAP00000011979-D3	-1.01	1.70E-44	↑	guanine nucleotide-binding protein G(I)/G(S)/G(O) subunit gamma-10 [Pantholops hodgsonii]
ENSBTAP00000021109-D1	-1.01	1.11E-47	↑	ubiquitin-conjugating enzyme E2 J2 isoform X2 [Capra hircus]
ENSBTAP00000033404-D1	-1.01	5.43E-44	↑	sugar phosphate exchanger 3 [B. mutus]
ENSP00000364310-D6	-1.01	2.59E-03	↑	histone H2A.x [B. taurus]
ENSP00000288670-D1	-1.01	2.87E-206	↑	Formin-like protein 2, partial [B. mutus]
ENSBTAP00000017658-D1	-1.01	1.05E-62	↑	Calcium-independent phospholipase A2-gamma [B. mutus]
ENSP00000349124-D1	-1.01	2.38E-275	↑	serine/threonine-protein phosphatase 4 regulatory subunit 2 [B. taurus]
ENSBTAP00000020923-D1	-1.01	3.34E-25	↑	U6 snRNA phosphodiesterase isoform X1 [B. mutus]
ENSP00000393369-D1	-1.01	3.19E-06	↑	transmembrane protein 138 isoform X2 [B. taurus]
ENSBTAP00000039852-D1	-1.01	0.00E+00	↑	peptidyl-prolyl cis-trans isomerase G [B. mutus]
ENSP00000418648-D1	-1.01	7.39E-71	↑	Ubinuclein-2, partial [B. mutus]
ENSP00000371532-D1	-1.01	4.13E-29	↑	very low-density lipoprotein receptor [B. mutus]
ENSBTAP00000022077-D1	-1.01	2.45E-38	↑	hypothetical protein H671_2g5148 [Cricetulus griseus]
ENSP00000387266-D1	-1.01	1.81E-21	↑	Protein spire-like protein 1, partial [B. mutus]
ENSBTAP00000027844-D1	-1.01	2.90E-201	↑	interferon-induced transmembrane protein 3 isoform X1 [B. mutus]
ENSP00000360613-D1	-1.00	2.48E-36	↑	ubiquitin-conjugating enzyme E2 A [Stegastes partitus]
ENSBTAP00000033682-D1	-1.00	3.94E-46	↑	28S ribosomal protein S21, mitochondrial [B. taurus]
ENSBTAP00000018554-D1	-1.00	1.20E-23	↑	phosphoserine aminotransferase [Bison bison bison]
ENSBTAP00000027295-D1	-1.00	6.40E-22	↑	uncharacterized aarF domain-containing protein kinase 2 [B. mutus]
ENSBTAP00000053517-D1	-1.00	3.35E-50	↑	Transmembrane protein 33, partial [B. mutus]
ENSP00000352936-D1	-1.00	1.22E-21	↑	Serine protease inhibitor Kazal-type 5, partial [B. mutus]
ENSBTAP00000016033-D1	-1.00	5.86E-04	↑	N-acetyllactosaminide alpha-1,3-galactosyltransferase isoform X4 [B. taurus]
ENSP00000263921-D1	-1.00	3.60E-52	↑	cysteine-rich hydrophobic domain-containing protein 2 [Propithecus coquereli]
ENSP00000345752-D1	-1.00	3.63E-174	↑	myotubularin-related protein 2 isoform X1 [B. mutus]
ENSBTAP00000003354-D1	-1.00	9.35E-17	↑	exosome component 10 [B. mutus]
ENSBTAP00000015901-D1	1.00	1.39E-04	↓	Feline leukemia virus subgroup C receptor-related protein 2 [B. mutus]
ENSBTAP00000010567-D1	1.00	9.76E-92	↓	28S ribosomal protein S35, mitochondrial isoform X1 [B. mutus]
ENSBTAP00000001256-D1	1.00	2.01E-139	↓	Protein ECT2 [B. mutus]
ENSBTAP00000025144-D1	1.00	4.68E-63	↓	V-type proton ATPase 21 kDa proteolipid subunit isoform X1 [Lipotes vexillifer]
ENSBTAP00000048365-D68	1.01	3.27E-06	↓	bucentaur [Cervus nippon]
ENSBTAP00000004550-D1	1.01	1.09E-03	↓	DDB1- and CUL4-associated factor 7 isoform X1 [Chinchilla lanigera]
ENSP00000311905-D1	1.01	5.33E-03	↓	Latent-transforming growth factor beta-binding protein 4, partial [B. mutus]
ENSP00000359321-D1	1.01	1.64E-205	↓	Metal-response element-binding transcription factor 2, partial [B. mutus]
ENSP00000265062-D1	1.01	4.84E-12	↓	ras-related protein Rab-7a isoform X1 [B. taurus]
ENSP00000307604-D1	1.01	4.34E-06	↓	zinc finger and BTB domain-containing protein 5 [B. mutus]
ENSBTAP00000009724-D1	1.01	1.09E-46	↓	28S ribosomal protein S24, mitochondrial [B. mutus]
ENSP00000419730-D1	1.01	1.65E-09	↓	SLAIN motif-containing protein 1, partial [B. mutus]

ENSBTAP00000027104-D1	1.01	7.12E-23	↓	T-complex protein 1 subunit zeta-2 isoform X1 [B. mutus]
ENSBTAP00000027060-D1	1.01	4.00E-29	↓	F-box only protein 38 isoform X1 [Bison bison bison]
ENSP00000247207-D1	1.01	3.29E-18	↓	heat shock-related 70 kDa protein 2 [Capra hircus]
ENSP00000373555-D1	1.01	1.14E-34	↓	Teneurin-2, partial [B. mutus]
ENSP00000196551-D1	1.01	3.65E-141	↓	40S ribosomal protein S5 [Homo sapiens]
ENSP00000274000-D1	1.01	6.08E-08	↓	AP-1 complex-associated regulatory protein, partial [B. mutus]
ENSP00000204549-D1	1.01	3.16E-05	↓	Programmed cell death protein 7, partial [B. mutus]
ENSBTAP00000017042-D1	1.02	2.40E-24	↓	ADP/ATP translocase 4 [B. mutus]
ENSBTAP00000035670-D3	1.02	2.89E-23	↓	growth arrest and DNA damage-inducible protein GADD45 alpha [B. taurus]
ENSBTAP00000026353-D1	1.02	1.03E-54	↓	cell division cycle-associated protein 3-like [B. mutus]
ENSBTAP00000029344-D1	1.02	1.23E-26	↓	voltage-dependent L-type calcium channel subunit beta-2 isoform X1 [B. taurus]
ENSP00000354621-D2	1.02	8.31E-32	↓	E3 ubiquitin-protein ligase SMURF2, partial [B. mutus]
ENSBTAP00000050903-D1	1.02	1.42E-15	↓	rho-related GTP-binding protein RhoE [Rhinopithecus roxellana]
ENSBTAP00000000959-D1	1.02	5.99E-38	↓	Contactin-associated protein-like 2, partial [B. mutus]
ENSBTAP00000028757-D13	1.02	8.88E-265	↓	heterogeneous nuclear ribonucleoproteins A2/B1 isoform X2 [Loxodonta africana]
ENSP00000233099-D2	1.02	3.28E-07	↓	HEAT repeat-containing protein 5A [B. mutus]
ENSBTAP00000026919-D1	1.02	1.05E-15	↓	leucine-rich repeat-containing protein 8B [B. mutus]
ENSBTAP00000028701-D1	1.02	1.25E-31	↓	RWD domain-containing protein 3 isoform X1 [B. mutus]
ENSBTAP00000036255-D1	1.02	1.98E-03	↓	synaptonemal complex protein SC65, partial [B. mutus]
ENSBTAP00000009864-D3	1.02	2.80E-66	↓	heterogeneous nuclear ribonucleoprotein F [B. taurus]
ENSBTAP00000020036-D1	1.02	8.81E-84	↓	hypothetical protein PANDA_011885, partial [Ailuropoda melanoleuca]
ENSP00000256658-D1	1.02	3.30E-08	↓	AP-4 complex subunit beta-1 isoform X1 [B. mutus]
ENSBTAP00000019602-D1	1.03	1.67E-93	↓	39S ribosomal protein L51, mitochondrial [B. mutus]
ENSBTAP00000053499-D1	1.03	2.49E-17	↓	Transcription initiation factor TFIID subunit 2, partial [B. mutus]
ENSBTAP00000016838-D1	1.03	7.77E-04	↓	connector enhancer of kinase suppressor of ras 3 isoform X1 [B. taurus]
ENSP00000322956-D1	1.03	5.93E-39	↓	microfibril-associated glycoprotein 3 precursor [B. taurus]
ENSP00000313007-D1	1.03	1.71E-75	↓	polyadenylate-binding protein 1 [Equus przewalskii]
ENSBTAP00000053840-D1	1.03	1.58E-74	↓	hypothetical protein M91_17184, partial [B. mutus]
ENSP00000315397-D1	1.03	0.00E+00	↓	28S ribosomal protein S31, mitochondrial isoform X2 [B. mutus]
yakG004678	1.03	5.88E-25	↓	Nogo-B receptor [B. mutus]
ENSBTAP00000022146-D1	1.03	2.42E-46	↓	Putative fructose-2,6-bisphosphatase TIGAR, partial [B. mutus]
ENSBTAP00000020623-D1	1.03	2.24E-39	↓	Ribosome-releasing factor 2, mitochondrial, partial [B. mutus]
ENSBTAP00000049383-D1	1.03	5.17E-169	↓	poly(rC)-binding protein 1 [B. mutus]
ENSP00000368605-D1	1.04	5.62E-145	↓	Acyl-coenzyme A thioesterase 9, mitochondrial, partial [B. mutus]
ENSP00000353850-D3	1.04	8.73E-37	↓	integrin beta-1-binding protein 1 isoform X1 [B. mutus]
ENSP00000397181-D1	1.04	2.21E-05	↓	regulator of G-protein signaling 4 [Orcinus orca]
ENSP00000260619-D1	1.04	3.16E-14	↓	THUMP domain-containing protein 2 [B. mutus]
ENSBTAP00000050442-D5	1.04	0.00E+00	↓	40S ribosomal protein S3a [Myotis brandtii]
ENSBTAP00000019938-D1	1.04	1.36E-105	↓	ALX homeobox protein 1 [B. mutus]
ENSBTAP00000000526-D1	1.04	3.44E-91	↓	pre-mRNA-processing factor 17 [B. taurus]
ENSP00000389125-D1	1.04	2.21E-128	↓	hypothetical protein M91_06043, partial [B. mutus]
ENSP00000365807-D4	1.04	8.81E-30	↓	guanine nucleotide-binding protein [Sus scrofa]
ENSBTAP00000012297-D1	1.04	2.00E-16	↓	Katanin p60 ATPase-containing subunit A-like 1 [B. mutus]
ENSP00000380033-D1	1.04	1.03E-09	↓	Putative ATP-dependent RNA helicase DDX17, partial [B. mutus]
ENSBTAP00000021305-D1	1.04	2.95E-07	↓	serine/threonine-protein phosphatase 2B catalytic subunit alpha isoform-like [Myotis brandtii]
ENSBTAP00000031299-D1	1.04	4.40E-42	↓	graves disease carrier protein [Pantholops hodgsonii]
ENSP00000369965-D6	1.04	0.00E+00	↓	transcription factor BTF3 isoform X1 [B. mutus]
ENSBTAP00000015592-D1	1.04	1.09E-11	↓	Ubiquitin carboxyl-terminal hydrolase 31, partial [B. mutus]
ENSP00000374490-D3	1.05	3.34E-78	↓	dynein heavy chain 11, axonemal isoform X2 [B. mutus]
ENSP00000379133-D1	1.05	6.90E-112	↓	Putative methylthioribulose-1-phosphate dehydratase, partial [B. mutus]
ENSBTAP00000043469-D1	1.05	1.48E-12	↓	DNA excision repair protein ERCC-6 [B. mutus]
ENSP00000366829-D1	1.05	2.28E-04	↓	RNA-binding protein 10, partial [B. mutus]
ENSBTAP00000030636-D1	1.05	5.64E-04	↓	T-cell leukemia translocation-altered gene protein homolog [B. taurus]
ENSP00000315299-D2	1.05	1.05E-02	↓	calmodulin-like [B. mutus]
ENSP00000412663-D1	1.05	1.05E-02	↓	glycogen synthase kinase-3 alpha [B. mutus]

ENSBTAP00000019738-D1	1.05	1.05E-02	↓	39S ribosomal protein L52, mitochondrial precursor [B. taurus]
ENSP00000389536-D1	1.05	5.14E-56	↓	Far upstream element-binding protein 1 [B. mutus]
ENSBTAP00000051754-D1	1.05	4.13E-21	↓	39S ribosomal protein L33, mitochondrial, partial [B. mutus]
ENSP00000353735-D1	1.05	1.29E-66	↓	E3 ubiquitin-protein ligase Topors, partial [B. mutus]
ENSP00000396377-D1	1.05	5.39E-21	↓	cAMP-regulated phosphoprotein 19 isoform 1 [B. taurus]
ENSBTAP00000024550-D1	1.05	0.00E+00	↓	Grancalcin, partial [B. mutus]
ENSBTAP00000011371-D1	1.05	1.50E-38	↓	cAMP-dependent protein kinase type I-alpha regulatory subunit, partial [B. mutus]
ENSP00000268854-D1	1.05	3.66E-104	↓	probable ATP-dependent RNA helicase DDX52 [B. taurus]
ENSBTAP00000026064-D1	1.05	2.13E-05	↓	MICAL-like protein 1, partial [B. mutus]
ENSBTAP00000044410-D1	1.05	2.80E-36	↓	transmembrane protein C5orf28 homolog [B. taurus]
ENSP00000328551-D1	1.05	4.49E-13	↓	ras-related protein Rab-28 [B. taurus]
ENSP00000376159-D1	1.05	1.16E-132	↓	Bcl-2-associated transcription factor 1, partial [B. mutus]
ENSBTAP00000017348-D1	1.06	1.03E-44	↓	zinc finger protein 277 [B. mutus]
ENSP00000295930-D1	1.06	5.52E-44	↓	Serine/Arginine-related protein 53, partial [B. mutus]
ENSP00000278200-D1	1.06	4.39E-217	↓	mitochondrial inner membrane protease subunit 1 isoform X1 [B. taurus]
ENSBTAP00000012929-D1	1.06	1.70E-115	↓	craniofacial development protein 2-like [B. mutus]
ENSBTAP00000044185-D36	1.06	2.82E-05	↓	craniofacial development protein 2 isoform X2 [Ovis aries musimon]
ENSBTAP00000053507-D1	1.06	0.00E+00	↓	coiled-coil domain-containing protein 73 [B. mutus]
ENSBTAP00000018595-D1	1.06	1.05E-37	↓	acylphosphatase-1 isoform X1 [Bison bison bison]
ENSP00000370624-D1	1.06	2.02E-22	↓	Mesoderm induction early response protein 3, partial [B. mutus]
ENSP00000384718-D1	1.06	1.90E-03	↓	40S ribosomal protein S25-like [B. mutus]
ENSBTAP00000025254-D1	1.06	6.63E-80	↓	Glycyl-tRNA synthetase [B. mutus]
ENSBTAP00000050548-D1	1.06	3.43E-40	↓	Protein L-Myc-1, partial [B. mutus]
yakG020512	1.06	8.62E-06	↓	Protein FAM96A [B. mutus]
ENSP00000351969-D1	1.06	4.03E-04	↓	Dynamin-2, partial [B. mutus]
ENSBTAP00000007036-D1	1.07	3.10E-122	↓	ORF [B. taurus]
ENSP00000419112-D1	1.07	1.70E-39	↓	Stress-associated endoplasmic reticulum protein 1, partial [B. mutus]
ENSP00000253329-D1	1.07	8.39E-301	↓	peptidyl-prolyl cis-trans isomerase-like 4 [B. taurus]
ENSP00000342564-D1	1.07	8.97E-05	↓	methylmalonate-semialdehyde dehydrogenase [acylating], mitochondrial isoform X1 [B. mutus]
ENSP00000369424-D2	1.07	0.00E+00	↓	histone-binding protein RBBP7 isoform X3 [Ovis aries]
ENSP00000216367-D1	1.07	1.83E-48	↓	DNA polymerase epsilon subunit 2 [B. mutus]
ENSP00000369857-D1	1.07	1.15E-105	↓	BRCA1-A complex subunit Abraxas, partial [B. mutus]
ENSBTAP00000020719-D2	1.07	6.29E-69	↓	40S ribosomal protein S10, partial [B. mutus]
ENSBTAP00000028708-D1	1.07	2.67E-65	↓	Putative rRNA-processing protein EBP2, partial [B. mutus]
ENSP00000344976-D1	1.07	5.10E-03	↓	X-linked interleukin-1 receptor accessory protein-like 2 [B. mutus]
ENSBTAP00000002448-D1	1.07	5.10E-03	↓	Period circadian protein-like protein 2, partial [B. mutus]
ENSP00000352581-D1	1.07	4.96E-21	↓	CLIP-associating protein 2 isoform X14 [B. taurus]
ENSBTAP00000029589-D1	1.07	1.83E-25	↓	Replication factor C subunit 5 [B. mutus]
ENSBTAP00000015829-D1	1.07	0.00E+00	↓	CD63 antigen, partial [B. mutus]
ENSP00000363925-D1	1.08	0.00E+00	↓	structural maintenance of chromosomes protein 2-like [B. mutus]
ENSBTAP00000022299-D1	1.08	1.09E-10	↓	ubiquitin-like modifier-activating enzyme 1 [B. taurus]
ENSBTAP00000011734-D1	1.08	3.43E-141	↓	splicing factor 3B subunit 1 isoform X1 [Pteropus alecto]
ENSBTAP00000000434-D1	1.08	9.69E-19	↓	Multiple epidermal growth factor-like domains protein 10 [B. mutus]
ENSBTAP00000001241-D1	1.08	1.70E-69	↓	sperm-specific antigen 2 isoform X1 [B. mutus]
ENSP00000263918-D1	1.08	1.73E-19	↓	Striatin, partial [B. mutus]
ENSBTAP00000035184-D3	1.08	0.00E+00	↓	S-phase kinase-associated protein 1 [Gorilla gorilla gorilla]
ENSP00000322304-D2	1.08	2.92E-04	↓	protein-cysteine N-palmitoyltransferase porcupine isoform X1 [B. taurus]
ENSP00000334836-D1	1.08	6.78E-18	↓	BMP-2-inducible protein kinase [B. mutus]
ENSBTAP00000019457-D1	1.08	1.16E-36	↓	sortilin-related receptor [B. mutus]
ENSP00000403400-D1	1.08	1.10E-78	↓	cell cycle progression protein 1 isoform X1 [B. mutus]
ENSP00000406103-D1	1.08	0.00E+00	↓	Nuclear pore complex protein Nup54 [B. mutus]
ENSBTAP00000008961-D1	1.08	1.90E-13	↓	E3 ubiquitin-protein ligase CBL, partial [B. mutus]
ENSBTAP00000038962-D1	1.08	1.37E-10	↓	Activator of 90 kDa heat shock protein ATPase-like protein 2, partial [B. mutus]
ENSP00000378086-D1	1.08	1.36E-08	↓	alpha-(1,6)-fucosyltransferase isoform X1 [B. taurus]
ENSBTAP00000052830-D58	1.08	8.47E-17	↓	Zinc finger protein 585A, partial [B. mutus]

ENSBTAP00000011811-D1	1.09	1.34E-03	↓	Histone H2A type 1-D, partial [B. mutus]
ENSBTAP00000050222-D22	1.09	1.58E-229	↓	60S ribosomal protein L17, partial [B. mutus]
ENSBTAP00000048529-D1	1.09	2.31E-24	↓	Neuronal acetylcholine receptor subunit alpha-3, partial [B. mutus]
ENSBTAP00000042757-D1	1.09	3.58E-43	↓	leucine-rich repeat-containing protein 57 isoform X1 [B. mutus]
yakG032579	1.09	7.93E-291	↓	hypothetical protein M91_09610 [B. mutus]
ENSP00000340662-D1	1.09	3.20E-82	↓	HSPC185 [Homo sapiens]
ENSP00000296782-D1	1.09	1.22E-36	↓	Rapamycin-insensitive companion of mTOR, partial [B. mutus]
ENSP00000384718-D9	1.09	0.00E+00	↓	40S ribosomal protein S25, partial [Charadrius vociferus]
ENSP00000339384-D1	1.09	2.55E-186	↓	uncharacterized protein C2orf66 homolog [B. mutus]
ENSP00000392353-D1	1.09	4.12E-11	↓	Proteasome assembly chaperone 4, partial [B. mutus]
ENSP00000339723-D1	1.09	0.00E+00	↓	hypothetical protein PANDA_008997, partial [Ailuropoda melanoleuca]
ENSP00000341610-D1	1.09	1.43E-05	↓	normal mucosa of esophagus-specific gene 1 protein [B. mutus]
ENSBTAP00000006491-D1	1.09	7.59E-223	↓	Serologically defined colon cancer antigen 1 [B. mutus]
ENSBTAP00000005648-D1	1.09	3.19E-35	↓	putative E3 ubiquitin-protein ligase UBR7 [B. mutus]
ENSP00000333277-D10	1.09	1.09E-239	↓	histone H3.3 [Felis catus]
ENSBTAP00000007840-D1	1.09	2.65E-304	↓	hypothetical protein M91_02430 [B. mutus]
ENSP00000365543-D1	1.09	3.04E-08	↓	WD repeat domain phosphoinositide-interacting protein 4 [B. mutus]
ENSBTAP00000022075-D1	1.09	2.09E-04	↓	Formin-like protein 3, partial [B. mutus]
ENSP00000406694-D1	1.09	9.41E-13	↓	Integrin alpha-6, partial [B. mutus]
ENSP00000353864-D1	1.09	2.56E-111	↓	serine/threonine-protein kinase PAK 3 [B. taurus]
ENSBTAP00000026218-D1	1.10	1.95E-26	↓	nucleoside-diphosphate kinase NBR-A [B. taurus]
ENSP00000291374-D1	1.10	2.96E-93	↓	Zinc finger and BTB domain-containing protein 8B, partial [B. mutus]
ENSBTAP00000005222-D1	1.10	1.96E-27	↓	histone deacetylase complex subunit SAP30L [B. taurus]
ENSBTAP00000024496-D1	1.10	2.25E-17	↓	Emopamil-binding protein-like protein, partial [B. mutus]
ENSBTAP00000010151-D1	1.10	1.65E-15	↓	Major facilitator superfamily domain-containing protein 6 [B. mutus]
ENSBTAP00000050787-D1	1.10	1.87E-26	↓	uncharacterized protein C8orf59 homolog [B. taurus]
ENSP00000264350-D1	1.10	6.97E-09	↓	E3 ISG15--protein ligase HERC5, partial [B. mutus]
ENSP00000381119-D8	1.10	1.30E-193	↓	Putative heterogeneous nuclear ribonucleoprotein A1-like protein 3 [Heterocephalus glaber]
ENSBTAP00000004526-D1	1.10	1.32E-45	↓	hypothetical protein M91_12349, partial [B. mutus]
ENSP00000376721-D1	1.10	5.70E-06	↓	Zinc finger and BTB domain-containing protein 16 [B. mutus]
yakG033019	1.10	1.28E-99	↓	E3 UFM1-protein ligase 1 [B. mutus]
ENSBTAP00000017187-D1	1.10	6.38E-39	↓	Thymocyte nuclear protein 1, partial [B. mutus]
ENSBTAP00000026132-D1	1.10	6.21E-41	↓	Putative homeodomain transcription factor 1 [B. mutus]
ENSBTAP00000018917-D2	1.10	3.20E-147	↓	T-complex protein 1 subunit theta, partial [B. mutus]
ENSBTAP00000051492-D13	1.10	0.00E+00	↓	histone H3.3 [Felis catus]
ENSP00000361524-D1	1.10	7.94E-39	↓	uncharacterized protein C9orf78 homolog isoform X1 [B. mutus]
ENSP00000387710-D1	1.10	2.24E-55	↓	threonine--tRNA ligase, cytoplasmic isoform X1 [B. mutus]
ENSP00000358590-D1	1.10	9.15E-55	↓	zinc finger protein 292 [B. mutus]
ENSP00000365668-D1	1.11	4.48E-05	↓	alpha-tubulin N-acetyltransferase 1 [B. taurus]
ENSBTAP00000008196-D1	1.11	2.79E-04	↓	WD repeat domain-containing protein 83 isoform X1 [B. mutus]
ENSP00000335076-D1	1.11	9.59E-04	↓	ectodysplasin-A receptor-associated adapter protein isoform X1 [B. mutus]
ENSBTAP00000005623-D1	1.11	7.08E-03	↓	cell division control protein 45 homolog isoform X1 [B. mutus]
ENSP00000393821-D1	1.11	1.52E-02	↓	GRAM domain-containing protein 1A [B. mutus]
ENSBTAP00000037794-D1	1.11	1.52E-02	↓	cytochrome c oxidase subunit 6B1-like [B. mutus]
ENSBTAP00000016108-D1	1.11	1.52E-02	↓	cell cycle checkpoint control protein RAD9A isoform X1 [B. mutus]
ENSBTAP00000047566-D2	1.11	5.14E-04	↓	TPA: ATP synthase, H+ transporting, mitochondrial F0 complex, subunit G-like [B. taurus]
ENSBTAP00000053277-D1	1.11	0.00E+00	↓	centromere protein K isoform X1 [Bubalus bubalis]
ENSBTAP00000023208-D1	1.11	8.12E-19	↓	prolyl-tRNA synthetase associated domain-containing protein 1 [B. taurus]
ENSBTAP00000020789-D92	1.11	4.57E-14	↓	mariner transposase [Homo sapiens]
ENSBTAP00000012472-D1	1.11	5.03E-49	↓	plexin domain-containing protein 2 isoform X1 [Capra hircus]
ENSP00000256443-D1	1.11	0.00E+00	↓	cyclin-dependent kinase 7 [B. taurus]
ENSBTAP00000018778-D1	1.11	1.49E-148	↓	cytochrome c oxidase subunit 6C [B. taurus]
ENSBTAP00000009104-D1	1.11	9.30E-07	↓	serine/threonine-protein phosphatase 1 regulatory subunit 10 isoform X2 [B. taurus]
ENSP00000228958-D1	1.11	1.01E-18	↓	Translation initiation factor eIF-2B subunit alpha, partial [B. mutus]
ENSBTAP00000012091-D1	1.11	1.75E-25	↓	ankyrin repeat domain-containing protein 49 [B. mutus]

ENSP00000281131-D1	1.11	1.28E-25	↓	ankyrin repeat domain-containing protein 50 [B. mutus]
ENSBTAP00000000722-D1	1.11	4.11E-209	↓	survival of motor neuron-related-splicing factor 30 [Otolemur garnettii]
ENSP00000259050-D1	1.12	5.27E-66	↓	E3 ubiquitin-protein ligase MARCH7 isoform X1 [B. mutus]
ENSP00000391432-D1	1.12	6.96E-191	↓	RNA-binding protein 40 isoform X1 [Bison bison bison]
ENSP00000260008-D1	1.12	2.64E-08	↓	FH2 domain-containing protein 1 isoform X2 [B. taurus]
ENSP00000357384-D1	1.12	2.39E-11	↓	Dolichol-phosphate mannosyltransferase subunit 3, partial [B. mutus]
ENSP00000281701-D1	1.12	8.37E-250	↓	Nuclear valosin-containing protein-like protein, partial [B. mutus]
ENSP00000257789-D1	1.12	0.00E+00	↓	origin recognition complex subunit 3 isoform X1 [B. mutus]
ENSBTAP00000006696-D1	1.12	0.00E+00	↓	mediator of RNA polymerase II transcription subunit 21 [B. taurus]
ENSP00000354622-D1	1.12	3.37E-09	↓	RUN domain-containing protein 1, partial [B. mutus]
ENSBTAP00000002532-D1	1.12	1.89E-26	↓	vesicle transport through interaction with t-SNAREs homolog 1B [Capra hircus]
ENSP00000217426-D1	1.12	6.77E-04	↓	Adenosylhomocysteinase, partial [B. mutus]
ENSBTAP00000052146-D1	1.13	4.88E-35	↓	ubiquitin-conjugating enzyme E2 U [B. mutus]
ENSP00000222693-D1	1.13	4.64E-18	↓	caveolin-2 [B. taurus]
ENSP00000359206-D1	1.13	2.99E-102	↓	F-box/WD repeat-containing protein 1A, partial [B. mutus]
ENSBTAP00000016043-D1	1.13	3.78E-13	↓	Transmembrane protein 87B, partial [B. mutus]
ENSP00000379457-D1	1.13	2.38E-20	↓	FAS-associated factor 1, partial [B. mutus]
ENSP00000380734-D1	1.13	3.56E-156	↓	protein FAM98B [B. taurus]
ENSBTAP00000010591-D1	1.13	1.66E-41	↓	GC-rich sequence DNA-binding factor, partial [B. mutus]
ENSP00000358265-D1	1.13	5.92E-32	↓	kelch-like protein 32 isoform X8 [B. taurus]
ENSBTAP00000010661-D1	1.13	2.45E-101	↓	retinal dehydrogenase 1 [B. mutus]
ENSBTAP00000008651-D1	1.13	5.84E-30	↓	protein chibby homolog 1 isoform X3 [Pantholops hodgsonii]
ENSBTAP00000015782-D1	1.13	6.21E-42	↓	Oral cancer-overexpressed protein 1, partial [B. mutus]
ENSBTAP00000012926-D1	1.13	6.39E-13	↓	RNA-binding protein Musashi-like protein 1, partial [B. mutus]
ENSBTAP00000045739-D1	1.13	4.16E-09	↓	probable phospholipid-transporting ATPase IA isoform X2 [B. mutus]
ENSP00000388311-D20	1.14	5.42E-44	↓	Zinc finger protein 845, partial [B. mutus]
ENSBTAP00000052237-D1	1.14	1.77E-56	↓	Biliverdin reductase A, partial [B. mutus]
ENSBTAP00000007189-D1	1.14	1.81E-39	↓	NADH dehydrogenase [ubiquinone] 1 alpha subcomplex subunit 9, mitochondrial [B. mutus]
ENSBTAP00000049230-D5	1.14	1.39E-25	↓	TPA: ribosomal protein L27a-like [B. taurus]
ENSP00000350750-D3	1.14	1.62E-43	↓	60S ribosomal protein L26-like 1 isoform X1 [Pan troglodytes]
ENSP00000323580-D1	1.14	8.17E-130	↓	Intraflagellar transport protein 88-like protein, partial [B. mutus]
ENSBTAP00000050946-D1	1.14	6.17E-60	↓	Transcription termination factor, mitochondrial, partial [B. mutus]
ENSP00000339299-D1	1.14	1.32E-15	↓	triple functional domain protein isoform X2 [Ovis aries musimon]
ENSBTAP00000018919-D1	1.14	5.62E-05	↓	hypothetical protein M91_16328 [B. mutus]
ENSP00000250498-D1	1.14	0.00E+00	↓	dolichyl-diphosphooligosaccharide--protein glycosyltransferase subunit DAD1 [B. taurus]
ENSP00000371973-D1	1.14	3.48E-161	↓	histone deacetylase complex subunit SAP18 [B. mutus]
ENSBTAP00000012481-D1	1.14	1.92E-172	↓	DNA-directed RNA polymerase II subunit RPB7 [B. mutus]
ENSP00000229251-D1	1.14	1.40E-13	↓	COP9 signalosome complex subunit 7a [B. taurus]
ENSBTAP00000001519-D1	1.14	1.02E-13	↓	disintegrin and metalloproteinase domain-containing protein 17 [B. mutus]
ENSBTAP00000006896-D1	1.14	1.03E-04	↓	zinc finger protein 605 isoform X3 [Bison bison bison]
ENSBTAP00000006184-D1	1.14	3.76E-26	↓	Hepatocyte nuclear factor 1-beta, partial [B. mutus]
ENSBTAP00000046975-D7	1.14	6.51E-70	↓	hypothetical protein M91_13240, partial [B. mutus]
ENSBTAP00000040527-D2	1.14	1.46E-71	↓	Beta-2-microglobulin [B. mutus]
ENSP00000257776-D1	1.14	2.20E-24	↓	melanocortin-2 receptor accessory protein 2 [B. mutus]
ENSP00000390181-D1	1.14	9.70E-149	↓	telomere-associated protein RIF1 [B. mutus]
ENSBTAP00000026889-D1	1.14	3.27E-29	↓	probable RNA-binding protein EIF1AD [B. mutus]
ENSP00000299705-D1	1.14	2.46E-07	↓	transmembrane emp24 domain-containing protein 3 [B. mutus]
ENSBTAP00000021092-D1	1.15	5.11E-62	↓	DNA ligase 4, partial [B. mutus]
ENSP00000373394-D1	1.15	4.24E-29	↓	eyes absent homolog 1-like isoform X3 [Tupaia chinensis]
ENSP00000418721-D1	1.15	1.98E-06	↓	Purine-rich element-binding protein gamma, partial [B. mutus]
ENSP00000377575-D1	1.15	1.88E-04	↓	basal body-orientation factor 1 isoform X1 [B. taurus]
ENSBTAP00000012742-D1	1.15	1.88E-04	↓	Transmembrane protein 184B, partial [B. mutus]
ENSP00000413632-D1	1.15	4.81E-24	↓	MCM domain-containing protein 2 isoform X1 [B. mutus]
ENSP00000273375-D1	1.15	8.52E-24	↓	Rab-like protein 3, partial [B. mutus]
ENSBTAP00000000830-D1	1.15	2.34E-186	↓	protein MMS22-like [B. mutus]

ENSBTAP00000001417-D1	1.15	9.31E-165	↓	Zinc finger CW-type PWWP domain protein 1 [B. mutus]
ENSP00000342305-D2	1.15	3.61E-75	↓	small nuclear ribonucleoprotein-associated proteins B and B', partial [Ursus maritimus]
ENSP00000347792-D1	1.15	2.91E-13	↓	Synaptojanin-2, partial [B. mutus]
ENSBTAP00000044459-D1	1.15	7.95E-30	↓	deoxyguanosine kinase, mitochondrial isoform X1 [B. mutus]
ENSBTAP00000029026-D1	1.15	3.46E-04	↓	NADH dehydrogenase [ubiquinone] flavoprotein 1, mitochondrial, partial [B. mutus]
ENSBTAP00000028179-D1	1.15	5.79E-07	↓	Proline-serine-threonine phosphatase-interacting protein 1, partial [B. mutus]
ENSBTAP00000008042-D1	1.16	0.00E+00	↓	arginine/serine-rich coiled-coil protein 2 isoform X1 [Microcebus murinus]
ENSBTAP00000051671-D53	1.16	1.71E-03	↓	hypothetical protein M91_20222, partial [B. mutus]
ENSBTAP00000018533-D1	1.16	3.65E-93	↓	U4/U6 small nuclear ribonucleoprotein Prp3 [Homo sapiens]
ENSBTAP00000041123-D31	1.16	3.83E-08	↓	p97Bcnt protein [Capra hircus]
ENSBTAP00000032361-D1	1.16	1.45E-09	↓	TPA: tetratricopeptide repeat domain 39B [B. taurus]
ENSBTAP00000017358-D1	1.16	2.32E-40	↓	E3 ubiquitin-protein ligase CBL-B isoform X1 [B. mutus]
ENSBTAP00000022477-D1	1.16	4.86E-18	↓	Mediator of RNA polymerase II transcription subunit 8, partial [B. mutus]
ENSBTAP00000002847-D1	1.16	4.53E-25	↓	atrial natriuretic peptide-converting enzyme [B. mutus]
ENSBTAP00000014520-D3	1.16	2.31E-96	↓	Single-stranded DNA-binding protein, mitochondrial, partial [B. mutus]
ENSP00000268206-D1	1.16	2.32E-15	↓	elongation factor Tu GTP-binding domain-containing protein 1 isoform X1 [B. mutus]
ENSBTAP00000021532-D1	1.16	6.29E-18	↓	PTB-containing, cubilin and LRP1-interacting protein, partial [B. mutus]
ENSP00000262094-D1	1.16	1.27E-117	↓	ras-related protein Rab-27B [B. taurus]
ENSBTAP00000006763-D1	1.17	1.80E-19	↓	centrosomal protein of 55 kDa [B. taurus]
ENSBTAP00000041398-D1	1.17	1.11E-93	↓	Tubulin alpha-1A chain, partial [B. mutus]
ENSBTAP00000008730-D1	1.17	1.05E-40	↓	lysophosphatidic acid receptor 6 [B. mutus]
ENSBTAP00000024413-D1	1.17	3.98E-219	↓	E3 ubiquitin-protein ligase Hakai, partial [B. mutus]
ENSP00000212015-D1	1.17	8.21E-09	↓	NAD-dependent deacetylase sirtuin-1, partial [B. mutus]
ENSBTAP00000035838-D1	1.17	3.37E-13	↓	Erythrocyte membrane protein band 4.2, partial [B. mutus]
ENSBTAP00000012636-D1	1.17	9.27E-25	↓	Transmembrane protein 120B, partial [B. mutus]
ENSP00000382423-D1	1.17	1.52E-05	↓	mitogen-activated protein kinase kinase kinase 1 [B. mutus]
ENSP00000304268-D1	1.17	8.37E-26	↓	zinc finger protein 33B isoform X1 [Bison bison bison]
ENSP00000302530-D1	1.17	3.44E-26	↓	mitotic checkpoint serine/threonine-protein kinase BUB1 [Bison bison bison]
ENSP00000263073-D1	1.17	1.13E-11	↓	Telomerase-binding protein EST1A, partial [B. mutus]
ENSBTAP00000012523-D4	1.17	1.55E-24	↓	acyl-CoA-binding domain-containing protein 7 [B. taurus]
ENSP00000348206-D2	1.17	1.44E-30	↓	Protein slowmo-like protein 2, partial [B. mutus]
ENSP00000382717-D1	1.18	1.73E-76	↓	WD repeat-containing protein 19, partial [B. mutus]
ENSBTAP00000024972-D1	1.18	6.16E-44	↓	transmembrane protein 163 isoform X2 [B. taurus]
ENSBTAP00000007241-D2	1.18	1.21E-03	↓	Small nuclear ribonucleoprotein G [B. mutus]
ENSP00000377027-D1	1.18	9.90E-03	↓	transforming growth factor-beta receptor-associated protein 1 [B. mutus]
ENSP00000307265-D1	1.18	9.90E-03	↓	interferon regulatory factor 2-binding protein 1-like [B. mutus]
ENSP00000377191-D1	1.18	1.43E-62	↓	Laminin subunit beta-1, partial [B. mutus]
ENSBTAP00000026896-D1	1.18	0.00E+00	↓	DNA cross-link repair 1A protein [B. mutus]
ENSBTAP00000041774-D1	1.18	1.33E-69	↓	hypothetical protein M91_09177, partial [B. mutus]
ENSBTAP00000016994-D1	1.18	7.78E-28	↓	TP53-regulated inhibitor of apoptosis 1 [Bubalus bubalis]
ENSBTAP00000044555-D1	1.18	7.88E-40	↓	Coiled-coil domain-containing protein 162, partial [B. mutus]
ENSBTAP00000008839-D1	1.18	1.98E-05	↓	Fibroblast growth factor 16, partial [B. mutus]
ENSP00000369257-D1	1.19	2.61E-109	↓	Protein fantom [B. mutus]
ENSP00000225719-D1	1.19	2.30E-93	↓	carboxypeptidase D [Bison bison bison]
ENSP00000370037-D1	1.19	6.76E-36	↓	Annexin A3, partial [B. mutus]
ENSBTAP00000053220-D1	1.19	1.14E-172	↓	Coiled-coil domain-containing protein 45, partial [B. mutus]
ENSP00000385552-D1	1.19	2.16E-09	↓	Calcium-activated potassium channel subunit alpha-1, partial [B. mutus]
ENSP00000264037-D1	1.19	8.50E-04	↓	Alpha-tectorin [B. mutus]
ENSP00000385021-D1	1.19	7.28E-44	↓	E3 ubiquitin-protein ligase FANCL [B. mutus]
ENSP00000418070-D1	1.19	2.92E-29	↓	Poliovirus receptor-related protein 3, partial [B. mutus]
ENSBTAP00000053448-D3	1.19	1.15E-09	↓	disks large homolog 1 isoform X14 [Condylura cristata]
ENSP00000409445-D1	1.19	2.33E-08	↓	integrator complex subunit 3 [Orcinus orca]
ENSBTAP00000018071-D1	1.20	1.54E-198	↓	charged multivesicular body protein 3 isoform X1 [B. mutus]
ENSBTAP00000013367-D1	1.20	0.00E+00	↓	NEDD8-activating enzyme E1 regulatory subunit [B. taurus]
ENSP00000309402-D1	1.20	3.63E-12	↓	translocating chain-associated membrane protein 1-like 1-like [B. mutus]

ENSBTAP00000003636-D1	1.20	6.27E-81	↓	Proteasome subunit alpha type-3, partial [B. mutus]
ENSBTAP00000020228-D1	1.20	1.59E-27	↓	ATP synthase subunit s, mitochondrial precursor [B. taurus]
ENSBTAP00000005061-D1	1.20	1.23E-54	↓	zinc finger with UFM1-specific peptidase domain protein isoform X1 [B. taurus]
ENSBTAP00000027339-D1	1.20	1.54E-226	↓	protein deglycase DJ-1 [B. taurus]
ENSBTAP00000052969-D2	1.20	5.32E-11	↓	40S ribosomal protein S4, partial [B. mutus]
ENSBTAP00000006175-D1	1.20	1.29E-16	↓	Immunoglobulin-like domain-containing receptor 1, partial [B. mutus]
ENSP00000323527-D1	1.20	2.36E-111	↓	muskelin isoform X1 [B. mutus]
ENSP00000219905-D1	1.20	3.86E-17	↓	MAX associated protein [B. mutus]
ENSBTAP00000019475-D1	1.20	0.00E+00	↓	arginine--tRNA ligase, cytoplasmic isoform X1 [B. mutus]
ENSBTAP00000018777-D1	1.20	4.69E-12	↓	Ras-related protein Rab-5B, partial [B. mutus]
ENSBTAP00000040957-D1	1.20	7.38E-106	↓	Synapsin-3, partial [B. mutus]
ENSP00000264344-D1	1.21	3.42E-21	↓	Protein FAM13A, partial [B. mutus]
ENSBTAP00000053567-D1	1.21	2.18E-08	↓	Palmitoyltransferase ZDHHC2, partial [B. mutus]
ENSP00000354519-D1	1.21	8.11E-79	↓	Phosphatidylinositide phosphatase SAC2, partial [B. mutus]
ENSBTAP00000001565-D1	1.21	1.31E-30	↓	Ribosomal protein S6 kinase delta-1 [B. mutus]
ENSBTAP00000012287-D1	1.21	2.00E-89	↓	NADH dehydrogenase [ubiquinone] 1 alpha subcomplex subunit 5, partial [B. mutus]
ENSBTAP00000011513-D1	1.21	0.00E+00	↓	cullin-1-like [B. mutus]
ENSBTAP00000044998-D1	1.21	0.00E+00	↓	ragulator complex protein LAMTOR3 [Pantholops hodgsonii]
ENSP00000360242-D1	1.21	3.37E-41	↓	E3 ubiquitin-protein ligase XIAP [B. mutus]
ENSBTAP00000029531-D1	1.21	3.87E-06	↓	Sodium/potassium/calcium exchanger 6 [B. mutus]
ENSBTAP00000033816-D1	1.21	0.00E+00	↓	Centromere protein F [B. mutus]
ENSBTAP00000026293-D1	1.22	0.00E+00	↓	Splicing factor, arginine/serine-rich 18 [B. mutus]
ENSBTAP00000049089-D1	1.22	4.28E-04	↓	zinc finger protein 62 homolog [Bubalus bubalis]
ENSBTAP00000014659-D1	1.22	4.28E-04	↓	RNA-binding protein with multiple splicing 2, partial [B. mutus]
ENSBTAP00000004181-D1	1.22	3.45E-84	↓	asparagine synthetase [glutamine-hydrolyzing] [B. taurus]
ENSBTAP00000015436-D1	1.22	1.27E-38	↓	UPF0711 protein C18orf21 homolog [B. mutus]
ENSP00000378324-D3	1.22	0.00E+00	↓	DnaJ-like protein subfamily A member 1, partial [B. mutus]
ENSBTAP00000019440-D1	1.22	1.04E-64	↓	Nucleosome-remodeling factor subunit BPTF, partial [B. mutus]
ENSP00000225916-D1	1.22	6.80E-10	↓	histone acetyltransferase KAT2A isoform X1 [Bubalus bubalis]
ENSP00000310260-D1	1.23	8.27E-53	↓	integrator complex subunit 6 [Bubalus bubalis]
ENSBTAP00000036040-D2	1.23	3.69E-123	↓	H/ACA ribonucleoprotein complex subunit 3 [B. taurus]
ENSP00000272839-D1	1.23	2.85E-22	↓	protein N-lysine methyltransferase METTL21A [B. mutus]
ENSBTAP00000004878-D2	1.23	2.35E-146	↓	prostaglandin reductase 2 [B. taurus]
ENSP00000354742-D1	1.23	3.03E-04	↓	Importin-9, partial [B. mutus]
ENSP00000399734-D1	1.23	5.64E-09	↓	unnamed protein product [Homo sapiens]
ENSBTAP00000010757-D1	1.23	0.00E+00	↓	protein Spindly isoform X1 [B. mutus]
ENSBTAP00000021170-D2	1.23	1.51E-19	↓	transmembrane protein 147 [B. taurus]
ENSP00000245539-D1	1.23	9.68E-39	↓	28S ribosomal protein S7, mitochondrial [B. mutus]
ENSBTAP00000003008-D1	1.23	0.00E+00	↓	disks large-associated protein 5 [B. mutus]
ENSBTAP00000001008-D1	1.23	5.52E-85	↓	N-alpha-acetyltransferase 35, NatC auxiliary subunit, partial [B. mutus]
ENSP00000348821-D1	1.23	6.10E-05	↓	Myomesin-1 [B. mutus]
ENSP00000341094-D2	1.23	6.10E-05	↓	histone H2A type 1-like [Cavia porcellus]
ENSBTAP00000015480-D1	1.24	3.51E-56	↓	Pre-mRNA-processing factor 6, partial [B. mutus]
ENSP00000295025-D1	1.24	1.10E-23	↓	proto-oncogene c-Rel [B. mutus]
ENSP00000330714-D7	1.24	1.68E-22	↓	Ras-related protein Rab-18, partial [B. mutus]
ENSP00000222823-D1	1.24	3.65E-23	↓	nucleotide-binding oligomerization domain-containing protein 1 [B. mutus]
ENSBTAP00000005894-D1	1.24	4.61E-12	↓	ubiquitin-like modifier-activating enzyme 5 [B. taurus]
ENSP00000388381-D1	1.24	1.15E-07	↓	A-kinase anchor protein 1, mitochondrial, partial [B. mutus]
ENSBTAP00000016072-D1	1.24	3.07E-24	↓	cytochrome c-type heme lyase [B. taurus]
ENSP00000266479-D1	1.24	5.23E-128	↓	dynamin-1-like protein isoform X1 [Pan paniscus]
ENSP00000375063-D1	1.24	3.30E-21	↓	NACHT, LRR and PYD domains-containing protein 5, partial [B. mutus]
ENSP00000288985-D1	1.24	5.05E-20	↓	putative DNA repair and recombination protein RAD26 isoform X1 [B. mutus]
ENSP00000399194-D1	1.24	1.29E-08	↓	polyhomeotic-like protein 1 [B. taurus]
ENSBTAP00000010129-D1	1.24	1.54E-04	↓	elongation of very long chain fatty acids protein 7 [B. taurus]
ENSBTAP00000022154-D1	1.24	1.54E-04	↓	abhydrolase domain-containing protein 4 [B. mutus]

ENSBTAP00000034926-D1	1.24	4.47E-03	↓	asporin [B. mutus]
ENSBTAP00000019351-D2	1.24	5.30E-204	↓	nucleolar RNA helicase 2 [Bison bison bison]
ENSP00000320251-D1	1.25	5.78E-50	↓	uncharacterized protein C3orf17 homolog [B. mutus]
ENSP00000379719-D1	1.25	0.00E+00	↓	occludin [Bison bison bison]
ENSBTAP00000013500-D1	1.25	5.69E-116	↓	cleavage and polyadenylation specificity factor subunit 2 [Propithecus coquereli]
ENSBTAP00000025858-D1	1.25	0.00E+00	↓	centrosomal protein CEP57L1 isoform X1 [B. mutus]
ENSBTAP00000012557-D1	1.25	4.86E-11	↓	Serine/threonine-protein phosphatase with EF-hands 1, partial [B. mutus]
ENSP00000364405-D1	1.25	2.24E-05	↓	Zinc finger protein 367 [B. mutus]
ENSBTAP00000017379-D1	1.25	1.13E-290	↓	IQ calmodulin-binding motif-containing protein 1 isoform X1 [B. mutus]
ENSP00000318024-D2	1.25	1.53E-83	↓	Regulator of microtubule dynamics protein 1, partial [B. mutus]
ENSP00000354888-D1	1.25	7.59E-10	↓	Coiled-coil domain-containing protein 152, partial [B. mutus]
ENSBTAP00000053613-D1	1.25	9.30E-150	↓	hypothetical protein M91_20519, partial [B. mutus]
ENSBTAP00000016001-D1	1.25	1.79E-65	↓	dnaJ homolog subfamily C member 3 [Bison bison bison]
ENSBTAP00000004632-D1	1.25	7.67E-24	↓	serine/threonine-protein kinase 35 isoform X2 [Ovis aries musimon]
ENSBTAP00000005796-D1	1.26	3.40E-25	↓	UDP-N-acetylglucosamine transferase subunit ALG14 homolog [B. taurus]
ENSBTAP00000000269-D1	1.26	6.02E-155	↓	RNA-binding protein 34 [B. mutus]
ENSP00000364811-D1	1.26	6.91E-111	↓	Isoleucyl-tRNA synthetase, cytoplasmic, partial [B. mutus]
ENSP00000406052-D1	1.26	1.57E-104	↓	Tudor domain-containing protein 5, partial [B. mutus]
ENSP00000276533-D1	1.26	5.76E-44	↓	DNA replication complex GINS protein SLD5 [B. mutus]
ENSBTAP00000026221-D1	1.26	1.16E-05	↓	CAP-Gly domain-containing linker protein 3 [B. taurus]
ENSBTAP00000026526-D2	1.26	6.92E-158	↓	syntenin-1 isoform X1 [B. mutus]
ENSBTAP00000029636-D1	1.26	1.66E-33	↓	26S proteasome non-ATPase regulatory subunit 9 [B. taurus]
ENSP00000369757-D1	1.26	7.74E-101	↓	40S ribosomal protein S6 [B. taurus]
ENSP00000337991-D1	1.26	4.37E-47	↓	protein SPT2 homolog [B. mutus]
ENSP00000396960-D1	1.26	4.92E-260	↓	26S proteasome complex subunit DSS1-like [B. mutus]
ENSP00000379366-D2	1.26	1.51E-99	↓	Mps one binder kinase activator-like 1B, partial [B. mutus]
ENSP00000261531-D1	1.26	1.72E-282	↓	SNW domain-containing protein 1, partial [B. mutus]
ENSP00000341082-D1	1.26	0.00E+00	↓	39S ribosomal protein L20, mitochondrial [B. mutus]
ENSP00000341213-D1	1.26	2.51E-42	↓	uncharacterized protein C20orf24 homolog isoform 2 [Canis lupus familiaris]
ENSBTAP00000022784-D1	1.26	0.00E+00	↓	protein CIP2A [B. mutus]
ENSP00000420487-D1	1.26	2.12E-08	↓	Aminoacylase-1, partial [B. mutus]
ENSP00000222388-D1	1.27	2.77E-41	↓	ATP-binding cassette sub-family F member 2 [B. taurus]
ENSBTAP00000000022-D2	1.27	1.68E-129	↓	WASH complex subunit CCDC53, partial [B. mutus]
ENSP00000367299-D1	1.27	1.75E-23	↓	Vacuolar protein-sorting-associated protein 36, partial [B. mutus]
ENSBTAP00000014517-D1	1.27	3.09E-06	↓	cysteine/serine-rich nuclear protein 2 [B. mutus]
ENSP00000292782-D1	1.28	7.24E-279	↓	uncharacterized LOC101866996 isoform X1 [Macaca fascicularis]
ENSP00000321746-D1	1.28	6.86E-46	↓	PDZ and LIM domain protein 5 isoform X1 [B. mutus]
ENSP00000336733-D1	1.28	2.11E-03	↓	hypothetical protein M91_12699, partial [B. mutus]
ENSBTAP00000002136-D1	1.28	2.11E-03	↓	Kinesin-like protein KIFC1, partial [B. mutus]
ENSP00000368017-D1	1.28	4.10E-80	↓	zinc finger C3H1 domain-containing protein [B. mutus]
ENSBTAP00000006331-D1	1.28	3.12E-61	↓	39S ribosomal protein L35, mitochondrial [B. taurus]
ENSBTAP00000044967-D1	1.28	1.49E-05	↓	DNA polymerase epsilon subunit 4, partial [B. mutus]
ENSP00000354891-D1	1.28	6.81E-11	↓	protein FAM163A [B. mutus]
ENSP00000384651-D1	1.29	4.26E-27	↓	myotubularin-related protein 3 isoform X4 [Bison bison bison]
ENSP00000413094-D1	1.29	5.31E-24	↓	probable ribonuclease ZC3H12C [B. mutus]
ENSBTAP00000004555-D1	1.29	7.82E-64	↓	UPF0547 protein C16orf87 homolog [B. taurus]
ENSBTAP00000014000-D1	1.29	1.46E-03	↓	protein ZNF365-like [B. mutus]
ENSBTAP00000009178-D1	1.29	2.38E-28	↓	complement decay-accelerating factor-like isoform X1 [B. mutus]
ENSBTAP00000037817-D2	1.29	1.09E-288	↓	Transcription initiation factor TFIID subunit 12, partial [B. mutus]
ENSP00000263331-D1	1.29	4.73E-34	↓	DNA-directed RNA polymerase I subunit RPA2 isoform X1 [B. mutus]
ENSP00000401946-D1	1.29	5.44E-77	↓	trinucleotide repeat-containing gene 6B protein isoform X1 [Bubalus bubalis]
ENSP00000359531-D1	1.29	9.04E-86	↓	transcription initiation factor IIB [B. mutus]
ENSBTAP00000042040-D1	1.30	4.70E-22	↓	LYR motif-containing protein 4 [Bison bison bison]
ENSP00000233607-D1	1.30	5.20E-05	↓	adenomatous polyposis coli protein 2 [B. mutus]
ENSP00000264245-D1	1.30	1.04E-03	↓	rho GTPase-activating protein 31 [B. mutus]

ENSBTAP00000000086-D1	1.30	1.23E-59	↓	Golgi-associated plant pathogenesis-related protein 1, partial [B. mutus]
ENSBTAP00000053667-D1	1.30	1.73E-42	↓	dymeclin [B. mutus]
ENSBTAP00000020461-D1	1.30	2.67E-15	↓	fructosamine-3-kinase isoform X1 [B. mutus]
ENSP00000376250-D1	1.30	0.00E+00	↓	coiled-coil domain-containing protein 50 isoform X1 [Bison bison bison]
ENSBTAP00000019527-D2	1.30	5.10E-34	↓	Polyadenylate-binding protein-interacting protein 2 [Apaloderma vittatum]
ENSP00000259873-D1	1.30	6.30E-77	↓	28S ribosomal protein S18b, mitochondrial [B. mutus]
ENSBTAP00000042915-D2	1.30	3.43E-12	↓	hypothetical protein M91_04874, partial [B. mutus]
ENSBTAP00000025863-D1	1.30	3.22E-185	↓	dual specificity protein kinase CLK4 [B. taurus]
ENSBTAP00000044477-D1	1.30	4.19E-67	↓	Protein lin-9-like protein, partial [B. mutus]
ENSP00000364301-D1	1.30	3.74E-48	↓	serine/threonine-protein kinase WNK3 isoform X1 [Ovis aries]
ENSBTAP00000017007-D1	1.30	0.00E+00	↓	signal recognition particle 19 kDa protein [B. taurus]
ENSP00000366982-D1	1.31	6.72E-67	↓	tRNA methyltransferase 10 homolog B [B. mutus]
ENSBTAP00000031853-D1	1.31	5.46E-60	↓	ruvB-like 2 isoform X1 [B. mutus]
ENSP00000281146-D1	1.31	4.76E-308	↓	UPF0462 protein C4orf33 homolog [B. mutus]
ENSBTAP00000023995-D1	1.31	1.09E-45	↓	IQ domain-containing protein G [B. mutus]
ENSBTAP00000013781-D1	1.31	1.94E-10	↓	A-kinase anchor protein 8-like isoform X2 [B. taurus]
ENSBTAP00000003652-D1	1.31	3.53E-76	↓	cyclic nucleotide-binding domain-containing protein 2-like [B. mutus]
ENSBTAP00000000742-D1	1.31	2.41E-70	↓	Transcription factor HES-1, partial [B. mutus]
ENSBTAP00000042141-D1	1.31	5.26E-66	↓	myb/SANT-like DNA-binding domain-containing protein 3-like isoform 4 [Ceratotherium simum simum]
ENSBTAP00000051366-D1	1.31	3.28E-09	↓	polycomb group RING finger protein 3 [B. taurus]
ENSP00000340083-D1	1.32	1.71E-09	↓	lysine-rich coiled-coil protein 1 isoform X1 [Bison bison bison]
ENSP00000346500-D1	1.32	3.92E-30	↓	Limb region 1 protein-like protein, partial [B. mutus]
ENSP00000260867-D1	1.32	5.84E-34	↓	mitochondrial import inner membrane translocase subunit Tim23 [Ovis aries]
ENSP00000365834-D1	1.32	5.65E-114	↓	vacuolar protein sorting-associated protein 13A isoform X1 [B. mutus]
ENSBTAP00000017765-D1	1.32	1.77E-121	↓	pumilio domain-containing protein KIAA0020 homolog isoform X1 [B. mutus]
ENSBTAP00000048165-D1	1.32	1.15E-40	↓	TRMT61B protein [B. taurus]
ENSBTAP00000007948-D1	1.32	0.00E+00	↓	Basic leucine zipper and W2 domain-containing protein 1, partial [B. mutus]
ENSP00000356322-D1	1.32	1.84E-04	↓	Zinc finger protein 281, partial [B. mutus]
ENSBTAP00000041885-D1	1.32	2.51E-14	↓	Protein deltex-2 [B. mutus]
ENSBTAP00000009419-D1	1.32	7.97E-82	↓	tetraspanin-31 [B. taurus]
ENSBTAP00000045378-D2	1.32	7.83E-23	↓	up-regulated during skeletal muscle growth protein 5 [B. taurus]
ENSP00000403172-D3	1.32	0.00E+00	↓	TPA: ribosomal protein L6-like [B. taurus]
ENSBTAP00000028855-D1	1.32	4.45E-27	↓	growth arrest-specific protein 2 [B. taurus]
ENSP00000325376-D1	1.32	1.99E-73	↓	heterogeneous nuclear ribonucleoprotein M isoform X1 [Sus scrofa]
ENSP00000385534-D2	1.33	6.70E-159	↓	mitochondrial import inner membrane translocase subunit Tim9 isoform a [Homo sapiens]
ENSP00000346874-D1	1.33	2.75E-77	↓	fatty acyl-CoA reductase 1-like [B. mutus]
ENSP00000311034-D1	1.33	4.02E-78	↓	Transmembrane protein 196, partial [B. mutus]
ENSP00000249750-D1	1.33	9.42E-42	↓	retinal dehydrogenase 2 isoform X2 [B. taurus]
ENSP00000386866-D1	1.33	2.15E-82	↓	coiled-coil domain-containing protein 14 [B. mutus]
ENSP00000279227-D1	1.33	3.82E-16	↓	fermitin family homolog 3 [B. taurus]
ENSBTAP00000014803-D1	1.33	1.10E-113	↓	E3 ubiquitin-protein ligase RNF13 precursor [B. taurus]
ENSBTAP00000047209-D1	1.33	1.64E-67	↓	Methylosome subunit pICln, partial [B. mutus]
ENSBTAP00000004831-D1	1.33	4.09E-74	↓	protein transport protein Sec23A [B. taurus]
ENSBTAP00000020704-D1	1.33	4.63E-129	↓	tetratricopeptide repeat protein 32 [B. taurus]
ENSBTAP00000003728-D1	1.34	1.24E-55	↓	DNA replication licensing factor MCM7 [B. taurus]
ENSBTAP00000017006-D1	1.34	3.21E-11	↓	uridine phosphorylase 2 [B. mutus]
ENSP00000278193-D1	1.34	0.00E+00	↓	hypothetical protein PANDA_009213, partial [Ailuropoda melanoleuca]
ENSBTAP00000050194-D85	1.34	3.92E-123	↓	hypothetical protein M91_06506 [B. mutus]
ENSP00000359610-D1	1.34	3.49E-289	↓	Proto-oncogene DBL, partial [B. mutus]
ENSP00000356879-D1	1.34	7.28E-09	↓	Regulator of G-protein signaling 5, partial [B. mutus]
ENSBTAP00000020876-D1	1.34	5.47E-38	↓	putative methyltransferase NSUN3 [B. mutus]
ENSBTAP00000024045-D1	1.34	4.03E-72	↓	protein lunapark isoform X1 [B. mutus]
ENSBTAP00000053796-D1	1.34	4.21E-43	↓	Eukaryotic translation initiation factor 4 gamma 3 [B. mutus]
ENSBTAP00000003099-D1	1.34	2.38E-06	↓	mediator of RNA polymerase II transcription subunit 19 [Canis lupus familiaris]
ENSBTAP00000014796-D1	1.34	3.65E-105	↓	Protein FAM162A, partial [B. mutus]

yakG036002	1.35	5.22E-127	↓	Transcriptional repressor protein YY1 [B. mutus]
ENSP00000266022-D1	1.35	1.36E-55	↓	RNA-binding protein 6 [B. mutus]
ENSBTAP00000000519-D1	1.35	5.27E-15	↓	vitamin K epoxide reductase complex subunit 1 isoform X1 [Camelus ferus]
ENSP00000337386-D1	1.35	5.62E-11	↓	zinc finger protein 36, C3H1 type-like 1 [B. mutus]
ENSP00000389702-D1	1.35	1.73E-63	↓	hexokinase-1 [B. taurus]
ENSP00000282003-D1	1.35	1.19E-228	↓	RING finger protein 219 [B. taurus]
ENSBTAP00000012256-D2	1.35	8.54E-115	↓	programmed cell death protein 5 [B. taurus]
ENSBTAP00000032777-D1	1.35	2.54E-83	↓	isoamyl acetate-hydrolyzing esterase 1 homolog [B. mutus]
ENSBTAP00000003285-D1	1.35	3.28E-45	↓	eukaryotic translation elongation factor 1 epsilon-1 [B. mutus]
ENSBTAP00000020011-D1	1.35	2.59E-272	↓	Protein Red, partial [B. mutus]
ENSBTAP00000006742-D1	1.35	6.61E-15	↓	Putative low affinity copper uptake protein 2 [B. mutus]
ENSBTAP00000002922-D1	1.35	9.14E-09	↓	Ankyrin repeat domain-containing protein 40, partial [B. mutus]
ENSBTAP00000015208-D1	1.36	5.71E-23	↓	histidine triad nucleotide-binding protein 2, mitochondrial [B. mutus]
ENSBTAP00000003585-D1	1.36	1.90E-71	↓	Transmembrane protein 149 [B. mutus]
ENSBTAP00000007032-D1	1.36	3.22E-115	↓	GPN-loop GTPase 3, partial [B. mutus]
ENSBTAP00000051260-D1	1.36	2.41E-39	↓	hypothetical protein M91_18953 [B. mutus]
yakG000052	1.36	3.54E-26	↓	hypothetical protein M91_21646 [B. mutus]
ENSP00000337194-D1	1.36	8.48E-20	↓	serine/threonine-protein kinase PRP4 homolog isoform X1 [Ovis aries]
ENSP00000379370-D6	1.36	8.40E-40	↓	Zinc finger protein 271, partial [B. mutus]
ENSP00000363142-D1	1.36	4.21E-25	↓	CD164 sialomucin-like 2 protein isoform X1 [Bison bison bison]
ENSP00000360132-D1	1.36	1.66E-111	↓	Snurportin-1, partial [B. mutus]
ENSP00000378294-D1	1.36	1.74E-16	↓	Nucleotide exchange factor SIL1, partial [B. mutus]
ENSP00000354884-D1	1.36	1.74E-16	↓	Ras association domain-containing protein 9, partial [B. mutus]
ENSP00000408283-D1	1.36	4.73E-54	↓	Profilin-2, partial [B. mutus]
ENSBTAP00000045674-D2	1.36	2.97E-57	↓	Small EDRK-rich factor 1, partial [B. mutus]
ENSP00000329858-D1	1.37	1.06E-25	↓	tRNA wybutosine-synthesizing protein 2-like protein, partial [B. mutus]
ENSBTAP00000034238-D1	1.37	8.71E-18	↓	hypothetical protein M91_04532, partial [B. mutus]
ENSP00000414921-D3	1.37	4.36E-17	↓	Polypyrimidine tract-binding protein 1, partial [B. mutus]
ENSP00000409204-D1	1.37	6.41E-11	↓	SUN domain-containing protein 2 [B. mutus]
ENSBTAP00000016944-D1	1.37	2.34E-10	↓	testis intracellular mediator protein [B. taurus]
ENSP00000343002-D1	1.37	3.01E-06	↓	Beta-1,3-glucosyltransferase, partial [B. mutus]
ENSP00000354684-D1	1.37	1.59E-05	↓	ubiquitin-conjugating enzyme E2 J1 [Bison bison bison]
ENSBTAP00000038073-D2	1.37	2.20E-05	↓	histone H2B type 1-like [Propithecus coquereli]
ENSP00000328521-D1	1.37	8.43E-05	↓	cysteine-rich protein 2 [B. taurus]
ENSP00000297239-D1	1.37	2.35E-04	↓	synaptotagmin-like protein 3 isoform X1 [B. mutus]
ENSBTAP00000026008-D1	1.37	1.91E-03	↓	integrin alpha-D [B. mutus]
ENSP00000366267-D1	1.37	2.77E-03	↓	Glypican-5, partial [B. mutus]
ENSBTAP00000044383-D1	1.37	4.05E-03	↓	SPRY domain-containing protein 3 [B. taurus]
ENSBTAP00000003883-D1	1.37	4.05E-03	↓	Membrane-associated phosphatidylinositol transfer protein 3, partial [B. mutus]
ENSP00000263314-D1	1.37	8.96E-03	↓	P2X purinoceptor 3 [B. mutus]
ENSBTAP00000028633-D1	1.37	8.96E-03	↓	S-arrestin, partial [B. mutus]
ENSP00000261797-D2	1.37	1.38E-02	↓	Bifunctional heparan sulfate N-deacetylase/N-sulfotransferase 1, partial [B. mutus]
ENSBTAP00000042479-D9	1.37	1.38E-02	↓	histone H2A type 1-like [Ursus maritimus]
ENSP00000381022-D1	1.37	1.38E-02	↓	Collagen alpha-1(XXVI) chain, partial [B. mutus]
ENSBTAP00000029870-D1	1.37	2.24E-02	↓	epidermal growth factor receptor kinase substrate 8-like protein 2 [B. mutus]
ENSP00000263707-D1	1.37	2.24E-02	↓	Transcription factor CP2-like protein 1, partial [B. mutus]
ENSBTAP00000037042-D1	1.37	2.24E-02	↓	Zinc-alpha-2-glycoprotein, partial [B. mutus]
ENSBTAP00000040253-D22	1.37	2.24E-02	↓	60S ribosomal protein L17-like [B. mutus]
ENSP00000253055-D1	1.37	2.24E-02	↓	mitogen-activated protein kinase kinase kinase 10 isoform X2 [Ovis aries musimon]
ENSBTAP00000004932-D7	1.37	2.24E-02	↓	Trace amine-associated receptor 3, partial [B. mutus]
ENSBTAP00000051671-D10	1.37	2.24E-02	↓	hypothetical protein M91_15199 [B. mutus]
ENSBTAP00000006831-D1	1.37	2.24E-02	↓	TP53-target gene 5 protein [B. mutus]
ENSBTAP00000053454-D2	1.37	2.24E-02	↓	fibroblast growth factor 9 [B. taurus]
ENSBTAP00000023515-D1	1.37	2.24E-02	↓	nitric oxide synthase, endothelial [B. mutus]
ENSBTAP00000052217-D9	1.37	2.24E-02	↓	dynein light chain 1, cytoplasmic-like [Struthio camelus australis]

yakG025494	1.37	2.24E-02	↓	Myosin light chain 5 [B. mutus]
yakG018621	1.37	2.24E-02	↓	hypothetical protein M91_08197 [B. mutus]
ENSP00000207457-D1	1.37	2.24E-02	↓	Tektin-2 [B. mutus]
ENSP00000386029-D1	1.37	2.24E-02	↓	Gamma-aminobutyric acid receptor subunit rho-2 [B. mutus]
ENSP00000408948-D1	1.37	2.24E-02	↓	uncharacterized protein ENSP00000365934-like [Capra hircus]
ENSBTAP00000051924-D7	1.37	1.07E-81	↓	histone H3.3-like [B. mutus]
ENSBTAP00000020512-D1	1.37	0.00E+00	↓	Chain A, Destrin, Nmr, Minimized Average Structure
ENSP00000229595-D2	1.37	0.00E+00	↓	histone chaperone ASF1A [Dipodomys ordii]
ENSBTAP00000030401-D1	1.37	0.00E+00	↓	TBC1 domain family member 15, partial [B. mutus]
ENSBTAP00000012363-D2	1.38	1.52E-225	↓	E3 SUMO-protein ligase NSE2 [B. mutus]
ENSBTAP00000018493-D1	1.38	2.29E-131	↓	glutaminyl-peptide cyclotransferase [B. mutus]
ENSBTAP00000053650-D1	1.38	3.35E-22	↓	ryanodine receptor 2 [Bison bison bison]
ENSBTAP00000010864-D1	1.38	4.19E-11	↓	Exophilin-5, partial [B. mutus]
ENSBTAP00000009815-D1	1.38	6.76E-240	↓	uncharacterized protein C5orf34 homolog [B. mutus]
ENSBTAP00000013200-D1	1.38	2.25E-89	↓	transmembrane and coiled-coil domains protein 1 precursor [Rattus norvegicus]
ENSBTAP00000019353-D1	1.38	2.03E-58	↓	KIF1-binding protein [Bubalus bubalis]
ENSP00000296595-D1	1.38	7.68E-37	↓	Transmembrane protein 161B, partial [B. mutus]
ENSP00000386464-D1	1.38	1.97E-43	↓	lymphocyte antigen 75 isoform X2 [Bison bison bison]
ENSP00000389277-D2	1.38	4.23E-231	↓	transmembrane BAX inhibitor motif containing 6 [B. mutus]
ENSP00000356836-D1	1.39	7.48E-09	↓	POU domain, class 2, transcription factor 1 isoform X1 [Bison bison bison]
ENSP00000368438-D1	1.39	0.00E+00	↓	proliferating cell nuclear antigen [B. taurus]
ENSBTAP00000053423-D1	1.39	2.83E-49	↓	Putative protein KIAA1328, partial [B. mutus]
ENSBTAP00000023877-D1	1.39	1.83E-56	↓	Jouberin, partial [B. mutus]
ENSBTAP00000041888-D1	1.40	7.24E-11	↓	Selenoprotein T [B. mutus]
ENSP00000296135-D1	1.40	4.85E-65	↓	Leucine zipper transcription factor-like protein 1, partial [B. mutus]
ENSBTAP00000004247-D1	1.40	1.85E-130	↓	DET1- and DDB1-associated protein 1-like [B. mutus]
ENSP00000309198-D1	1.40	2.71E-06	↓	Ubiquitin-protein ligase E3C, partial [B. mutus]
ENSP00000355541-D1	1.40	1.71E-78	↓	HEAT repeat-containing protein 1-like, partial [B. mutus]
ENSBTAP00000039388-D1	1.40	3.52E-184	↓	Centromere protein C 1, partial [B. mutus]
ENSBTAP00000001522-D1	1.40	3.47E-161	↓	N-acetyltransferase 5 (ARD1 homolog, S. cerevisiae), isoform CRA_f [Mus musculus]
ENSP00000381817-D1	1.40	2.23E-68	↓	torsin-1A-interacting protein 1 [B. mutus]
ENSP00000376829-D1	1.40	8.03E-20	↓	Kelch repeat and BTB domain-containing protein 3, partial [B. mutus]
ENSBTAP00000003800-D1	1.40	3.83E-16	↓	dual specificity phosphatase DUPD1 [B. mutus]
ENSP00000353655-D2	1.40	2.36E-51	↓	alpha-mannosidase 2 [B. mutus]
ENSBTAP00000043102-D1	1.40	6.79E-09	↓	DNA-directed RNA polymerase I subunit RPA12, partial [B. mutus]
ENSBTAP00000014279-D1	1.40	6.79E-09	↓	Protein phosphatase 1E, partial [B. mutus]
ENSP00000267620-D1	1.40	1.02E-05	↓	thyroid receptor-interacting protein 11-like protein [Camelus ferus]
ENSBTAP00000004442-D1	1.40	1.53E-76	↓	mitochondrial genome maintenance exonuclease 1 [B. mutus]
ENSBTAP00000051209-D1	1.41	6.40E-73	↓	dolichyl-diphosphooligosaccharide--protein glycosyltransferase subunit 2 isoform X1 [B. taurus]
ENSBTAP00000028063-D1	1.41	1.97E-138	↓	dystrobrevin beta isoform X1 [Myotis lucifugus]
yakG042214	1.41	0.00E+00	↓	Kinesin-like protein KIF20B [B. mutus]
ENSP00000398177-D1	1.41	2.01E-122	↓	Rab5 GDP/GTP exchange factor [B. mutus]
ENSP00000353375-D1	1.41	9.96E-22	↓	Microtubule-associated protein 4, partial [B. mutus]
yakG029799	1.41	2.07E-63	↓	hypothetical protein M91_15455 [B. mutus]
ENSBTAP00000005441-D1	1.41	3.86E-05	↓	spermatogenesis-associated protein 20 isoform X1 [B. mutus]
ENSP00000382328-D1	1.41	4.66E-100	↓	NEDD4-binding protein 2-like 2, partial [B. mutus]
ENSBTAP00000024502-D1	1.41	3.43E-24	↓	Tubulin polyglutamylase TTLL4 [B. mutus]
ENSBTAP00000008253-D1	1.42	1.03E-35	↓	hypothetical protein M91_06214 [B. mutus]
ENSBTAP00000018052-D1	1.42	3.09E-16	↓	Transmembrane protein 66, partial [B. mutus]
ENSP00000370964-D1	1.42	1.16E-11	↓	solute carrier organic anion transporter family member 1C1 isoform X1 [B. mutus]
ENSP00000262650-D1	1.42	3.17E-09	↓	E3 ubiquitin-protein ligase Itchy homolog [Bison bison bison]
ENSP00000361190-D1	1.42	6.29E-35	↓	28S ribosomal protein S18a, mitochondrial [B. mutus]
ENSP00000344424-D1	1.42	3.07E-11	↓	uncharacterized protein KIAA0247 homolog isoform X1 [B. taurus]
ENSBTAP00000026114-D1	1.42	0.00E+00	↓	oocyte-expressed protein homolog [B. taurus]
ENSP00000390750-D1	1.42	8.38E-09	↓	small integral membrane protein 19 [B. taurus]

ENSBTAP00000013507-D1	1.43	2.89E-123	↓	NADH dehydrogenase [ubiquinone] iron-sulfur protein 5 [B. taurus]
ENSBTAP00000051733-D1	1.43	2.75E-18	↓	hydroxyacyl-thioester dehydratase type 2, mitochondrial-like [Tursiops truncatus]
yakG034559	1.43	1.44E-39	↓	proteoglycan 4 [B. mutus]
ENSP00000327539-D2	1.43	1.99E-133	↓	heterogeneous nuclear ribonucleoprotein H isoform X1 [Camelus dromedarius]
ENSBTAP00000013675-D1	1.43	2.90E-58	↓	vacuolar protein sorting-associated protein 37A [B. taurus]
ENSBTAP00000003415-D1	1.43	1.90E-51	↓	Septin-9, partial [B. mutus]
ENSBTAP00000025800-D1	1.43	5.72E-157	↓	DNA-directed RNA polymerase II subunit RPB2 [B. mutus]
ENSBTAP00000020679-D1	1.43	2.10E-04	↓	DLA class II histocompatibility antigen, DR-1 beta chain-like isoform X1 [Bison bison bison]
ENSP00000297151-D1	1.43	3.48E-141	↓	pre-mRNA-splicing factor SLU7 [B. mutus]
ENSBTAP00000006857-D1	1.43	1.74E-55	↓	transmembrane protein 14A isoform X2 [B. taurus]
ENSP00000402890-D1	1.43	1.74E-55	↓	DPY30 domain-containing protein 1, partial [B. mutus]
ENSP00000216468-D1	1.43	4.27E-08	↓	Protein TMED8, partial [B. mutus]
ENSBTAP00000004701-D1	1.43	0.00E+00	↓	protein PBDC1 isoform X1 [B. taurus]
ENSBTAP00000013591-D1	1.43	2.21E-41	↓	WASH complex subunit strumpellin, partial [B. mutus]
ENSP00000408965-D48	1.43	7.50E-51	↓	Zinc finger protein 836, partial [B. mutus]
ENSBTAP00000021770-D1	1.43	1.48E-09	↓	ATP-binding cassette sub-family G member 8, partial [B. mutus]
ENSP00000384085-D1	1.43	8.19E-08	↓	hypothetical protein M91_02481 [B. mutus]
ENSP00000357055-D1	1.43	2.96E-04	↓	hypothetical protein PANDA_018453, partial [Ailuropoda melanoleuca]
ENSBTAP00000023552-D1	1.43	2.64E-55	↓	ATP-binding cassette sub-family G member 2 isoform X1 [B. mutus]
ENSBTAP00000019407-D1	1.44	2.06E-09	↓	hypothetical protein M91_14314, partial [B. mutus]
ENSP00000286835-D1	1.44	1.23E-15	↓	splicing factor, arginine/serine-rich 15 isoform X1 [B. taurus]
ENSP00000302768-D1	1.44	0.00E+00	↓	Polyadenylate-binding protein-interacting protein 1, partial [B. mutus]
ENSP00000257787-D1	1.44	0.00E+00	↓	akirin-2 [Callithrix jacchus]
ENSP00000364550-D1	1.44	4.16E-04	↓	lysine-specific demethylase 5C isoform X2 [B. mutus]
ENSP00000348234-D1	1.44	8.42E-17	↓	tyrosine aminotransferase isoform X1 [B. mutus]
ENSBTAP00000014735-D1	1.44	7.38E-80	↓	dipeptidyl peptidase 1 [B. mutus]
ENSP00000201023-D1	1.44	0.00E+00	↓	endoplasmic reticulum-Golgi intermediate compartment protein 2 [B. taurus]
ENSP00000274787-D1	1.44	3.53E-82	↓	HIG1 domain family member 2A, mitochondrial [B. taurus]
ENSP00000415433-D1	1.44	5.18E-273	↓	integrator complex subunit 12 [B. mutus]
ENSP00000315569-D1	1.44	2.39E-13	↓	DIS3-like exonuclease 2, partial [B. mutus]
ENSBTAP00000003532-D1	1.44	1.07E-40	↓	mRNA-capping enzyme [B. taurus]
ENSBTAP00000028055-D2	1.44	0.00E+00	↓	Lymphokine-activated killer T-cell-originated protein kinase [B. mutus]
ENSP00000355493-D1	1.44	9.36E-162	↓	adenylosuccinate synthetase isozyme 2 [B. taurus]
ENSP00000393785-D1	1.45	7.51E-19	↓	NF-kappa-B inhibitor-interacting Ras-like protein 1 isoform X2 [B. taurus]
ENSP00000350136-D2	1.45	1.04E-18	↓	Ribosomal protein S6 modification-like protein A, partial [B. mutus]
ENSBTAP00000052540-D2	1.45	0.00E+00	↓	OTU domain-containing protein 6B [B. mutus]
ENSBTAP00000003206-D1	1.45	1.78E-05	↓	Ras-related protein Rab-11B, partial [B. mutus]
ENSBTAP00000049540-D46	1.45	2.71E-34	↓	zinc finger protein 729-like isoform X1 [Leptonychotes weddellii]
ENSP00000295645-D1	1.45	2.09E-48	↓	phosducin-like protein 2 [B. mutus]
ENSP00000260563-D1	1.45	1.66E-25	↓	RNA 3'-terminal phosphate cyclase isoform X1 [B. mutus]
ENSP00000360492-D6	1.45	2.77E-84	↓	hypothetical protein M91_03235 [B. mutus]
ENSP00000350275-D16	1.45	8.09E-07	↓	Histone H3.2 [B. mutus]
ENSBTAP00000006855-D1	1.45	2.99E-26	↓	dermatan-sulfate epimerase [B. mutus]
ENSP00000229769-D1	1.45	2.47E-05	↓	Fanconi anemia group E protein, partial [B. mutus]
ENSBTAP00000009093-D1	1.45	2.47E-05	↓	Kelch domain-containing protein 4, partial [B. mutus]
ENSBTAP00000024514-D1	1.45	0.00E+00	↓	Putative ATP-dependent RNA helicase DDX5, partial [B. mutus]
ENSBTAP00000015090-D1	1.45	8.71E-22	↓	ADP-ribose pyrophosphatase, mitochondrial [B. mutus]
ENSBTAP00000026038-D1	1.45	3.31E-80	↓	39S ribosomal protein L39, mitochondrial, partial [B. mutus]
ENSP00000380747-D1	1.45	1.95E-58	↓	Transmembrane protein 209, partial [B. mutus]
ENSP00000264157-D1	1.45	4.37E-50	↓	cyclin-T2 isoform X1 [B. mutus]
ENSBTAP00000050205-D1	1.45	1.20E-21	↓	Oligosaccharyltransferase complex subunit OSTC, partial [B. mutus]
ENSP00000399801-D1	1.45	1.13E-06	↓	beta-crystallin B1 [B. mutus]
ENSP00000393538-D1	1.46	1.44E-14	↓	cAMP-responsive element modulator isoform X1 [B. mutus]
ENSBTAP00000017411-D1	1.46	1.11E-285	↓	Protein unc-119-like protein A, partial [B. mutus]
ENSP00000264596-D1	1.46	1.59E-214	↓	Endonuclease 8-like 3, partial [B. mutus]

ENSP00000298139-D1	1.46	8.03E-57	↓	Werner syndrome ATP-dependent helicase [B. mutus]
ENSP00000356853-D1	1.46	3.26E-28	↓	uridine-cytidine kinase 2 isoform X2 [Capra hircus]
yakG033319	1.46	2.17E-06	↓	hypothetical protein M91_19059 [B. mutus]
ENSBTAP00000010219-D1	1.46	2.02E-21	↓	Equilibrative nucleoside transporter 4, partial [B. mutus]
ENSBTAP00000011762-D1	1.46	0.00E+00	↓	Prefoldin subunit 5, partial [B. mutus]
ENSBTAP00000026236-D1	1.46	4.47E-10	↓	Large proline-rich protein BAT2 [B. mutus]
ENSP00000231509-D1	1.46	4.81E-05	↓	glucocorticoid receptor isoform X1 [B. taurus]
ENSBTAP00000012693-D1	1.46	0.00E+00	↓	la-related protein 7 isoform X2 [Bison bison bison]
ENSBTAP00000018075-D4	1.47	1.30E-31	↓	Zinc finger protein 345, partial [B. mutus]
ENSP00000358541-D1	1.47	3.34E-50	↓	suppressor of IKBKE 1 isoform X1 [B. taurus]
ENSBTAP00000052592-D18	1.47	1.19E-03	↓	hypothetical protein M91_12176, partial [B. mutus]
ENSP00000341422-D1	1.47	1.19E-03	↓	transmembrane prolyl 4-hydroxylase isoform X2 [Rhinopithecus roxellana]
ENSP00000365220-D1	1.47	1.44E-77	↓	Tripeptidyl-peptidase 2, partial [B. mutus]
ENSP00000402046-D1	1.47	9.29E-78	↓	Pleckstrin-like protein domain-containing family A member 6 [B. mutus]
ENSBTAP00000031689-D1	1.47	3.71E-07	↓	TPA: paternally expressed 3 [B. taurus]
ENSBTAP00000046261-D1	1.47	8.15E-137	↓	mediator of RNA polymerase II transcription subunit 7 isoform X1 [B. taurus]
ENSBTAP00000029234-D2	1.47	4.80E-34	↓	Protein AATF, partial [B. mutus]
ENSBTAP00000024297-D1	1.47	2.23E-09	↓	cyclin-dependent kinase inhibitor 1B [B. taurus]
ENSBTAP00000021123-D2	1.48	2.85E-22	↓	NADH dehydrogenase [ubiquinone] 1 beta subcomplex subunit 4, partial [B. mutus]
ENSBTAP00000042274-D1	1.48	1.78E-198	↓	T-complex protein 1 subunit delta [B. mutus]
ENSBTAP00000024373-D1	1.48	1.42E-107	↓	Serine/threonine-protein phosphatase 2A 55 kDa regulatory subunit B alpha isoform, partial [B. mutus]
ENSP00000409548-D1	1.48	2.64E-43	↓	solute carrier family 35 member B1 [B. mutus]
ENSBTAP00000045734-D1	1.48	1.26E-164	↓	ATP-binding cassette sub-family E member 1 [B. taurus]
ENSBTAP00000002277-D1	1.48	0.00E+00	↓	terminal uridylyltransferase 7 isoform X6 [Bison bison bison]
ENSP00000372612-D1	1.48	8.05E-96	↓	solute carrier family 25 member 51 [B. taurus]
ENSBTAP00000016610-D1	1.48	7.15E-07	↓	Bcl2 antagonist of cell death, partial [B. mutus]
ENSP00000404386-D1	1.48	7.98E-244	↓	LIM domain only protein 3 isoform X1 [Nomascus leucogenys]
ENSP00000270812-D1	1.49	8.94E-08	↓	hypothetical protein PANDA_001141, partial [Ailuropoda melanoleuca]
ENSP00000309823-D1	1.49	1.68E-03	↓	forkhead box protein P4 [B. taurus]
ENSP00000360113-D1	1.49	8.14E-09	↓	VWFA and cache domain-containing protein 1, partial [B. mutus]
ENSP00000364737-D1	1.49	1.59E-257	↓	centromere protein P [Bison bison bison]
ENSP00000260956-D1	1.49	0.00E+00	↓	lupus La protein homolog [B. mutus]
ENSBTAP00000021594-D1	1.49	9.59E-98	↓	60S ribosomal export protein NMD3 [B. mutus]
ENSP00000309432-D1	1.49	3.86E-18	↓	protein FAM134C [B. mutus]
ENSP00000412461-D1	1.50	1.85E-04	↓	rho GTPase-activating protein 22 isoform X4 [Bison bison bison]
ENSP00000378396-D1	1.50	1.85E-04	↓	60S ribosomal protein L23a, partial [B. mutus]
ENSP00000332791-D1	1.50	2.73E-09	↓	kelch-like protein 15 [B. taurus]
ENSP00000415847-D1	1.50	9.50E-19	↓	DENN domain-containing protein 1B, partial [B. mutus]
ENSP00000373420-D1	1.50	5.98E-175	↓	RUN and FYVE domain-containing protein 2 [B. mutus]
ENSP00000394031-D1	1.50	6.45E-29	↓	Forkhead box protein N2 [B. mutus]
ENSP00000356233-D1	1.50	2.97E-08	↓	Lysine-specific demethylase 5B, partial [B. mutus]
ENSBTAP00000047729-D1	1.50	0.00E+00	↓	L-lactate dehydrogenase A chain isoform X1 [B. mutus]
ENSP00000265421-D1	1.50	2.87E-87	↓	DNA polymerase beta [B. taurus]
ENSP00000351723-D1	1.50	0.00E+00	↓	Nucleolar protein 8 [B. mutus]
ENSBTAP00000011044-D1	1.50	2.14E-106	↓	Small nuclear ribonucleoprotein E, partial [B. mutus]
ENSP00000354774-D1	1.50	2.00E-34	↓	Chronic lymphocytic leukemia deletion region 6 protein-like protein, partial [B. mutus]
ENSP00000349748-D1	1.50	1.78E-79	↓	splicing factor, proline- and glutamine-rich [Ailuropoda melanoleuca]
ENSBTAP00000009536-D1	1.51	8.81E-260	↓	kinetochore protein Nuf2-like [B. mutus]
ENSBTAP00000044801-D1	1.51	2.43E-03	↓	RING finger protein 26 [B. mutus]
ENSBTAP00000008382-D2	1.51	0.00E+00	↓	Acyl carrier protein, mitochondrial, partial [B. mutus]
ENSP00000336606-D1	1.51	1.86E-117	↓	DNA repair and recombination protein RAD54B [B. mutus]
ENSBTAP00000021643-D2	1.51	0.00E+00	↓	Small nuclear ribonucleoprotein F, partial [B. mutus]
ENSP00000414321-D1	1.51	1.31E-172	↓	40S ribosomal protein S24, partial [B. mutus]
ENSP00000263672-D1	1.51	2.18E-115	↓	Signal peptidase complex subunit 2 [B. mutus]
ENSP00000384048-D1	1.51	8.29E-14	↓	PAX-interacting protein 1, partial [B. mutus]

ENSP00000282251-D1	1.51	4.43E-93	↓	CWF19-like protein 2, partial [B. mutus]
ENSBTAP00000013104-D1	1.52	4.21E-05	↓	target of rapamycin complex subunit LST8 [B. taurus]
ENSP00000373933-D1	1.52	4.21E-05	↓	TPA: Protein Wiz-like [B. taurus]
ENSP00000307491-D1	1.52	5.33E-282	↓	WD repeat-containing protein 48 [Capra hircus]
ENSP00000315410-D1	1.52	3.06E-61	↓	oxysterol-binding protein-related protein 3 [B. taurus]
ENSBTAP00000048468-D1	1.52	0.00E+00	↓	Zona pellucida sperm-binding protein 2 [B. mutus]
ENSBTAP00000022514-D5	1.52	8.71E-07	↓	heterogeneous nuclear ribonucleoprotein A1-like [B. mutus]
ENSP00000264926-D1	1.52	0.00E+00	↓	E3 ubiquitin-protein ligase RAD18, partial [B. mutus]
ENSP00000357625-D1	1.52	3.44E-136	↓	BCL2/adenovirus E1B 19 kDa protein-interacting protein 3, partial [B. mutus]
ENSBTAP00000012088-D6	1.52	0.00E+00	↓	60S ribosomal protein L7, partial [B. mutus]
ENSP00000282382-D1	1.52	1.02E-31	↓	TIR domain-containing adapter molecule 2, partial [B. mutus]
ENSBTAP00000018399-D1	1.53	2.07E-07	↓	zinc finger protein 689 [B. taurus]
ENSP00000334234-D1	1.53	2.28E-15	↓	Septin-10, partial [B. mutus]
ENSP00000337393-D1	1.53	8.75E-108	↓	Protein UXT, partial [B. mutus]
ENSBTAP00000007749-D1	1.53	2.31E-56	↓	Trimethylguanosine synthase [B. mutus]
ENSBTAP00000005311-D1	1.53	1.08E-110	↓	3'(2'),5'-bisphosphate nucleotidase 1 isoform X3 [B. taurus]
ENSBTAP00000026720-D1	1.53	6.26E-190	↓	Putative protein KIAA1712-like protein, partial [B. mutus]
ENSP00000359645-D1	1.53	1.99E-114	↓	RNA-binding motif protein, X chromosome isoform X1 [Ovis aries]
ENSP00000223208-D1	1.53	1.88E-13	↓	centrosomal protein of 41 kDa [B. taurus]
ENSP00000356076-D1	1.54	8.30E-12	↓	dual specificity tyrosine-phosphorylation-regulated kinase 3 isoform X1 [Bison bison bison]
ENSP00000417052-D1	1.54	1.88E-62	↓	3-beta-hydroxysteroid-Delta(8),Delta(7)-isomerase [B. mutus]
ENSP00000344818-D1	1.54	5.36E-115	↓	polyubiquitin-C precursor [Rattus norvegicus]
ENSBTAP00000027348-D1	1.54	2.02E-21	↓	Isocitrate dehydrogenase [NADP] cytoplasmic, partial [B. mutus]
ENSBTAP00000023446-D1	1.54	2.00E-18	↓	FAD-linked sulfhydryl oxidase ALR [B. taurus]
ENSBTAP00000020072-D1	1.54	8.20E-05	↓	metallothionein-4 [B. taurus]
ENSBTAP00000043551-D33	1.54	3.53E-03	↓	hypothetical protein M91_16257, partial [B. mutus]
ENSBTAP00000021336-D3	1.54	3.53E-03	↓	Homeobox protein Hox-B9 [B. mutus]
yakG024169	1.54	3.53E-03	↓	hypothetical protein M91_16460 [B. mutus]
ENSP00000236709-D1	1.54	3.53E-03	↓	alpha-1,4-N-acetylglucosaminyltransferase [B. mutus]
ENSBTAP00000019636-D1	1.54	9.79E-38	↓	Wnt inhibitory factor 1 [B. mutus]
ENSBTAP00000044469-D1	1.54	1.16E-48	↓	trafficking protein particle complex subunit 4 [B. taurus]
ENSBTAP00000015449-D1	1.54	0.00E+00	↓	microtubule-associated proteins 1A/1B light chain 3B [B. taurus]
ENSP00000395021-D1	1.54	2.88E-09	↓	Cytochrome c oxidase subunit 7A1, mitochondrial, partial [B. mutus]
ENSBTAP00000041096-D1	1.54	0.00E+00	↓	CDK-activating kinase assembly factor MAT1 [B. taurus]
ENSP00000359484-D1	1.55	9.24E-40	↓	stromal membrane-associated protein 1 isoform X6 [B. taurus]
ENSBTAP00000012842-D1	1.55	5.49E-07	↓	Putative tubulin polyglutamylase TTLL9, partial [B. mutus]
ENSP00000253810-D1	1.55	1.12E-31	↓	Ankyrin repeat and KH domain-containing protein 1, partial [B. mutus]
ENSP00000386259-D1	1.55	2.35E-97	↓	nebulin [B. mutus]
ENSBTAP00000006984-D1	1.55	2.42E-21	↓	dTDP-D-glucose 4,6-dehydratase [B. mutus]
ENSP00000348984-D1	1.55	1.74E-93	↓	protein SON isoform X2 [B. mutus]
ENSBTAP00000021310-D1	1.55	4.76E-196	↓	kelch-like protein 7 [B. mutus]
ENSP00000336931-D1	1.55	6.35E-24	↓	proline-rich nuclear receptor coactivator 1 [B. mutus]
ENSP00000296642-D1	1.56	2.10E-105	↓	geranylgeranyl transferase type-1 subunit beta [Orcinus orca]
ENSBTAP00000025685-D1	1.56	2.73E-22	↓	Integrin alpha-2, partial [B. mutus]
ENSBTAP00000001677-D1	1.56	3.19E-60	↓	Protein phosphatase 1L, partial [B. mutus]
ENSBTAP00000021791-D1	1.56	7.43E-121	↓	Ganglioside-induced differentiation-associated protein 2 [B. mutus]
ENSP00000307214-D1	1.56	5.85E-13	↓	leucine carboxyl methyltransferase 2 [B. mutus]
ENSBTAP00000017314-D1	1.56	2.20E-50	↓	HCLS1-associated protein X-1 [B. mutus]
yakG021436	1.56	2.63E-05	↓	Zinc finger protein 142 [B. mutus]
ENSP00000282606-D1	1.56	6.90E-185	↓	GTP-binding protein SAR1b [B. mutus]
ENSBTAP00000051795-D1	1.56	4.66E-87	↓	Hydrolethalus syndrome protein 1-like protein [B. mutus]
ENSP00000274382-D1	1.57	5.24E-104	↓	protein limb expression 1 homolog [B. taurus]
ENSP00000391903-D2	1.57	7.68E-31	↓	Lys-63-specific deubiquitinase BRCC36, partial [B. mutus]
ENSP00000351100-D1	1.57	1.06E-10	↓	WD repeat-containing protein 55 [B. taurus]
ENSP00000410059-D1	1.57	2.19E-17	↓	elongation factor 1-delta isoform X2 [B. mutus]

ENSBTAP00000006026-D2	1.57	0.00E+00	↓	peptidyl-prolyl cis-trans isomerase H isoform X1 [B. taurus]
ENSBTAP00000051539-D1	1.57	1.37E-51	↓	non-structural maintenance of chromosomes element 1 homolog, partial [B. mutus]
ENSP00000362744-D2	1.57	0.00E+00	↓	Protein DBF4 like protein B [Pteropus alecto]
ENSP00000331201-D1	1.57	1.60E-04	↓	hepatocyte growth factor-regulated tyrosine kinase substrate isoform X1 [B. taurus]
ENSBTAP00000039580-D1	1.57	3.33E-105	↓	CDGSH iron-sulfur domain-containing protein 2, partial [B. mutus]
ENSP00000361543-D2	1.58	3.37E-18	↓	Nuclear pore glycoprotein p62, partial [B. mutus]
ENSBTAP00000024914-D1	1.58	4.34E-87	↓	Putative ATP-dependent RNA helicase DDX20, partial [B. mutus]
ENSBTAP00000040632-D1	1.58	2.00E-06	↓	Junction plakoglobin, partial [B. mutus]
ENSP00000414303-D1	1.58	2.00E-06	↓	Brain-derived neurotrophic factor, partial [B. mutus]
ENSBTAP00000000112-D1	1.58	1.22E-31	↓	TPA: 39S ribosomal protein L23, mitochondrial-like [B. taurus]
ENSP00000250454-D1	1.58	0.00E+00	↓	E2F-associated phosphoprotein [B. mutus]
ENSBTAP00000008769-D1	1.58	2.69E-08	↓	Putative ATP-dependent RNA helicase DDX27, partial [B. mutus]
ENSP00000401856-D1	1.59	5.80E-117	↓	Coiled-coil domain-containing protein 80, partial [B. mutus]
ENSP00000388274-D15	1.59	4.30E-27	↓	hypothetical protein M91_18457, partial [B. mutus]
ENSBTAP00000011379-D1	1.59	6.01E-149	↓	mitochondrial fission regulator 1 [B. mutus]
ENSP00000396918-D1	1.59	5.21E-10	↓	Up-regulator of cell proliferation, partial [B. mutus]
ENSP00000381138-D1	1.59	1.55E-07	↓	1-acyl-sn-glycerol-3-phosphate acyltransferase gamma [B. mutus]
ENSBTAP00000048967-D86	1.59	2.24E-04	↓	hypothetical protein M91_20933 [B. mutus]
ENSP00000390475-D1	1.59	5.15E-03	↓	CpG-binding protein, partial [B. mutus]
ENSP00000323362-D1	1.59	5.75E-24	↓	E3 ubiquitin-protein ligase ZNRF1, partial [B. mutus]
ENSP00000398131-D3	1.59	1.52E-274	↓	Eukaryotic peptide chain release factor GTP-binding subunit ERF3A, partial [B. mutus]
ENSBTAP00000053495-D1	1.60	1.45E-264	↓	usherin-like [B. mutus]
ENSP00000354575-D1	1.60	5.08E-08	↓	Lipolysis-stimulated lipoprotein receptor, partial [B. mutus]
ENSP00000306524-D2	1.60	1.23E-39	↓	volume-regulated anion channel subunit LRRC8C [B. taurus]
ENSBTAP00000000357-D1	1.60	0.00E+00	↓	glucosamine-6-phosphate isomerase 2 isoform X1 [B. taurus]
ENSBTAP00000053409-D1	1.60	8.12E-51	↓	Putative E3 ubiquitin-protein ligase HERC1, partial [B. mutus]
ENSP00000305502-D1	1.60	2.13E-07	↓	Activating signal cointegrator 1 complex subunit 2 [B. mutus]
ENSP00000271588-D1	1.60	2.54E-99	↓	hemicentin-1 [B. mutus]
ENSP00000220849-D2	1.60	0.00E+00	↓	eukaryotic translation initiation factor 3 subunit E isoform X2 [Nomascus leucogenys]
ENSP00000386515-D1	1.61	0.00E+00	↓	tax1-binding protein 1 homolog [B. taurus]
ENSBTAP00000020362-D1	1.61	0.00E+00	↓	uncharacterized protein C8orf76 homolog [B. mutus]
ENSBTAP00000051470-D1	1.61	1.90E-11	↓	Ovochymase-2, partial [B. mutus]
ENSP00000357901-D1	1.61	1.83E-224	↓	sodium channel modifier 1 [B. taurus]
ENSBTAP00000005072-D1	1.61	7.58E-25	↓	ethylmalonyl-CoA decarboxylase isoform X1 [Bison bison bison]
ENSBTAP00000018538-D1	1.61	1.78E-92	↓	Protein ELYS, partial [B. mutus]
ENSBTAP00000019969-D1	1.61	4.89E-86	↓	mCG22814 [Mus musculus]
yakG001658	1.61	5.18E-97	↓	Exocyst complex component 5 [B. mutus]
ENSBTAP00000053720-D1	1.62	2.99E-53	↓	contactin-associated protein-like 2-like, partial [B. mutus]
ENSBTAP00000025255-D1	1.62	9.32E-98	↓	COP9 signalosome complex subunit 3 [B. taurus]
ENSP00000386213-D1	1.62	2.20E-21	↓	myosin-IIIb [B. mutus]
yakG019205	1.62	3.11E-04	↓	hypothetical protein M91_18079 [B. mutus]
ENSBTAP00000051337-D10	1.62	5.91E-31	↓	Hemoglobin subunit alpha-1, partial [B. mutus]
ENSP00000264312-D1	1.62	0.00E+00	↓	OCIA domain-containing protein 1 [B. taurus]
ENSP00000379505-D1	1.62	1.13E-15	↓	Sorting and assembly machinery component 50-like protein, partial [B. mutus]
ENSP00000309431-D15	1.62	8.64E-129	↓	Tubulin alpha-1C chain, partial [B. mutus]
ENSP00000251076-D1	1.62	6.91E-163	↓	dmX-like protein 2 [B. mutus]
ENSBTAP00000013139-D1	1.62	1.12E-75	↓	phosducin-like protein 3 [B. mutus]
ENSBTAP00000024467-D1	1.62	0.00E+00	↓	hypothetical protein M91_10435, partial [B. mutus]
ENSBTAP00000008975-D1	1.62	3.00E-21	↓	TPA: hypothetical protein BOS_13152 [B. taurus]
ENSBTAP00000015026-D1	1.62	6.67E-28	↓	coiled-coil domain-containing protein 12 [B. taurus]
ENSBTAP00000005280-D1	1.62	3.07E-17	↓	4-aminobutyrate aminotransferase, mitochondrial [B. mutus]
ENSBTAP00000008646-D1	1.63	9.11E-123	↓	protein BEX4 isoform X1 [Bison bison bison]
ENSP00000222124-D1	1.63	2.06E-30	↓	transmembrane protein 205 [B. mutus]
ENSP00000364107-D2	1.63	2.80E-19	↓	Putative G antigen family D member 1, partial [B. mutus]
ENSBTAP00000019339-D1	1.63	1.50E-84	↓	Guanine nucleotide-binding protein-like 3 [B. mutus]

ENSBTAP00000041428-D1	1.63	1.55E-120	↓	GPI-anchor transamidase [B. mutus]
ENSP00000387462-D1	1.63	1.40E-94	↓	zinc finger and BTB domain-containing protein 10 [Bison bison bison]
ENSP00000258888-D1	1.63	7.26E-06	↓	Alpha-protein kinase 3, partial [B. mutus]
ENSP00000378320-D1	1.63	9.79E-05	↓	Tetratricopeptide repeat protein 7A, partial [B. mutus]
ENSBTAP00000022950-D1	1.63	1.28E-157	↓	serine/threonine-protein kinase greatwall isoform X2 [B. mutus]
ENSBTAP00000042500-D1	1.64	5.67E-306	↓	mesencephalic astrocyte-derived neurotrophic factor precursor [B. taurus]
ENSBTAP00000006849-D1	1.64	1.42E-68	↓	tRNA wybutosine-synthesizing protein 3 homolog [B. taurus]
ENSP00000374855-D30	1.64	5.49E-127	↓	hypothetical protein M91_09155, partial [B. mutus]
ENSBTAP00000011542-D1	1.64	5.24E-120	↓	TRAF-type zinc finger domain-containing protein 1 [B. mutus]
ENSP00000281141-D1	1.64	8.47E-62	↓	Cell division cycle protein 123-like protein [B. mutus]
ENSBTAP00000010141-D1	1.64	9.52E-63	↓	vesicle-trafficking protein SEC22b-like isoform X1 [B. mutus]
ENSP00000307272-D1	1.64	3.12E-05	↓	regulatory-associated protein of mTOR isoform X1 [B. mutus]
ENSBTAP00000044311-D1	1.65	4.95E-20	↓	protein FAM36A [B. taurus]
ENSP00000357681-D1	1.65	0.00E+00	↓	Fibronectin type 3 and ankyrin repeat domains protein 1, partial [B. mutus]
yakG043431	1.65	3.13E-63	↓	Protein QN1-like protein [B. mutus]
ENSBTAP00000019191-D1	1.65	0.00E+00	↓	anaphase-promoting complex subunit CDC26 isoform X1 [B. taurus]
ENSP00000296273-D1	1.65	1.36E-101	↓	replication factor C subunit 4 isoform X1 [B. taurus]
ENSP00000362111-D1	1.65	0.00E+00	↓	tetraspanin-6 [B. taurus]
ENSP00000367330-D1	1.65	1.85E-56	↓	uncharacterized protein C12orf73 homolog precursor [B. taurus]
ENSBTAP00000024325-D1	1.65	1.70E-127	↓	TATA box-binding protein-like protein 2, partial [B. mutus]
ENSBTAP00000053067-D4	1.65	8.17E-08	↓	histone H3.3 [Felis catus]
ENSBTAP00000028759-D1	1.65	0.00E+00	↓	Condensin complex subunit 3 [B. mutus]
ENSP00000404465-D1	1.65	3.40E-36	↓	Intraflagellar transport protein 57-like protein [B. mutus]
ENSBTAP00000007028-D1	1.66	2.12E-48	↓	hypothetical protein PANDA_000890, partial [Ailuropoda melanoleuca]
ENSBTAP00000052414-D1	1.66	8.85E-09	↓	hypothetical protein M91_03971, partial [B. mutus]
ENSBTAP00000019340-D1	1.66	5.03E-30	↓	glycosyltransferase 8 domain-containing protein 1 [Bison bison bison]
ENSBTAP00000002369-D1	1.66	2.72E-265	↓	Histone H1oo [B. mutus]
ENSBTAP00000019702-D1	1.67	1.06E-10	↓	enolase-phosphatase E1 [B. mutus]
ENSBTAP00000011729-D1	1.67	3.54E-74	↓	Polyribonucleotide nucleotidyltransferase 1, mitochondrial [B. mutus]
ENSP00000367086-D1	1.67	3.68E-08	↓	Cytosolic acyl coenzyme A thioester hydrolase [B. mutus]
ENSBTAP00000007150-D1	1.67	1.16E-54	↓	Macrophage-capping protein, partial [B. mutus]
ENSBTAP00000037314-D1	1.67	5.63E-27	↓	hypothetical protein M91_05051, partial [B. mutus]
ENSP00000311827-D1	1.67	1.79E-21	↓	E3 ubiquitin-protein ligase MSL2 [B. taurus]
ENSBTAP00000009227-D1	1.67	1.44E-06	↓	hypothetical protein M91_11567, partial [B. mutus]
ENSBTAP00000023588-D2	1.68	2.94E-31	↓	tubulin alpha-1 chain-like [B. mutus]
ENSP00000234816-D1	1.68	4.69E-07	↓	angiopoietin-related protein 1 isoform X1 [B. mutus]
ENSBTAP00000025936-D1	1.68	2.37E-67	↓	Protein cornichon-like protein 4 [B. mutus]
yakG002104	1.68	2.36E-15	↓	TBC1 domain family member 1 [B. mutus]
ENSP00000379653-D1	1.68	5.33E-58	↓	apoptosis facilitator Bcl-2-like protein 14 isoform X2 [B. mutus]
ENSP00000310688-D1	1.68	6.54E-11	↓	ATP-binding cassette sub-family D member 2 [B. mutus]
ENSBTAP00000029284-D1	1.68	0.00E+00	↓	Activated RNA polymerase II transcriptional coactivator p15, partial [B. mutus]
ENSP00000325905-D1	1.69	0.00E+00	↓	unnamed protein product [Mus musculus]
ENSP00000319501-D1	1.69	3.11E-40	↓	UDP-glucose 6-dehydrogenase [B. mutus]
ENSBTAP00000036401-D1	1.69	4.35E-89	↓	3-hydroxyacyl-CoA dehydratase 3, partial [B. mutus]
ENSBTAP00000016642-D1	1.69	0.00E+00	↓	Caspase-6 [B. mutus]
ENSP00000402852-D1	1.69	0.00E+00	↓	Small nuclear ribonucleoprotein Sm D3, partial [Heterocephalus glaber]
ENSBTAP00000027378-D1	1.69	3.62E-108	↓	tRNA (cytosine-5-)-methyltransferase-like [B. mutus]
ENSP00000353427-D1	1.69	2.09E-07	↓	Nuclear receptor subfamily 4 group A member 1, partial [B. mutus]
ENSP00000413520-D1	1.69	6.39E-07	↓	hypothetical protein M91_03306, partial [B. mutus]
ENSBTAP00000034200-D1	1.69	1.97E-06	↓	protein Mdm4 isoform X2 [Pantholops hodgsonii]
ENSBTAP00000049865-D24	1.69	5.94E-05	↓	spermatid nuclear transition protein 3-like, partial [Bison bison bison]
ENSBTAP00000008501-D1	1.69	5.94E-05	↓	tRNA-splicing endonuclease subunit Sen34 [B. mutus]
ENSP00000414552-D1	1.69	2.04E-03	↓	mitogen-activated protein kinase kinase kinase 12 isoform X1 [B. taurus]
ENSBTAP00000029911-D2	1.69	2.04E-03	↓	motile sperm domain-containing protein 2 isoform X2 [Bubalus bubalis]
ENSP00000400168-D1	1.69	2.04E-03	↓	Pentatricopeptide repeat-containing protein 1, partial [B. mutus]

ENSP00000332258-D1	1.69	7.57E-03	↓	Diacylglycerol O-acyltransferase 1, partial [B. mutus]
ENSP00000258775-D1	1.69	7.57E-03	↓	NAC-alpha domain-containing protein 1, partial [B. mutus]
ENSBTAP00000024049-D1	1.69	7.57E-03	↓	Spermatogenesis-associated protein 2, partial [B. mutus]
ENSP00000252575-D1	1.69	7.57E-03	↓	Neurocan core protein [B. mutus]
ENSBTAP00000022003-D1	1.69	5.58E-82	↓	Multidrug and toxin extrusion protein 1, partial [B. mutus]
ENSBTAP00000012421-D1	1.69	3.04E-126	↓	erythroid differentiation-related factor 1 [B. mutus]
ENSP00000379978-D1	1.69	8.18E-28	↓	Beta-2-microglobulin [B. mutus]
ENSP00000415032-D1	1.69	1.59E-230	↓	General transcription factor IIH subunit 5 [B. mutus]
ENSBTAP00000043167-D1	1.70	2.08E-56	↓	hypothetical protein M91_18500 [B. mutus]
ENSBTAP00000047554-D1	1.70	0.00E+00	↓	nucleophosmin [B. taurus]
ENSP00000340736-D1	1.70	1.23E-35	↓	Glycogenin-1, partial [B. mutus]
ENSBTAP00000019802-D1	1.70	6.41E-81	↓	cytochrome c oxidase assembly factor 5 [B. taurus]
ENSP00000202788-D1	1.70	7.13E-45	↓	MAP kinase-activated protein kinase 5 [B. mutus]
ENSBTAP00000008903-D1	1.70	3.08E-72	↓	39S ribosomal protein L13, mitochondrial, partial [B. mutus]
ENSBTAP00000018540-D1	1.70	0.00E+00	↓	heterogeneous nuclear ribonucleoprotein D0-like [Equus caballus]
ENSP00000345079-D1	1.70	2.04E-274	↓	cysteine and glycine-rich protein 2 [Homo sapiens]
ENSP00000315568-D1	1.71	7.27E-51	↓	late secretory pathway protein AVL9 homolog isoform X1 [Bubalus bubalis]
ENSBTAP00000035648-D1	1.71	8.77E-61	↓	serine/threonine-protein kinase Nek3 [B. mutus]
ENSBTAP00000024191-D1	1.71	3.66E-15	↓	Nuclear prelamin A recognition factor [B. mutus]
ENSP00000358879-D1	1.71	9.98E-14	↓	Filamin-A [B. mutus]
ENSBTAP00000020214-D1	1.71	9.83E-72	↓	hypothetical protein M91_00537, partial [B. mutus]
ENSP00000358640-D1	1.71	2.69E-112	↓	monocarboxylate transporter 1-like [B. mutus]
ENSBTAP00000011481-D1	1.71	8.37E-06	↓	DDB1- and CUL4-associated factor 4 [B. mutus]
ENSBTAP00000019672-D1	1.71	3.07E-222	↓	protein polybromo-1 isoform X1 [B. taurus]
ENSBTAP00000042140-D1	1.71	0.00E+00	↓	kinesin-like protein KIF18A [B. mutus]
ENSP00000302111-D1	1.72	7.42E-11	↓	Methylated-DNA--protein-cysteine methyltransferase, partial [B. mutus]
ENSP00000252483-D1	1.72	2.60E-05	↓	Poliovirus receptor-related protein 2, partial [B. mutus]
ENSP00000332103-D1	1.72	6.36E-35	↓	N(alpha)-acetyltransferase 38, NatC auxiliary subunit isoform X2 [B. taurus]
ENSP00000253024-D1	1.72	1.22E-12	↓	Transcription intermediary factor 1-beta, partial [B. mutus]
ENSP00000369339-D1	1.72	2.54E-83	↓	protein C9orf72 homolog [B. taurus]
ENSBTAP00000053604-D1	1.72	1.29E-129	↓	remodeling and spacing factor 1 [B. mutus]
ENSP00000346762-D1	1.73	9.01E-37	↓	staphylococcal nuclease domain-containing protein 1 [B. taurus]
ENSBTAP00000030114-D2	1.73	0.00E+00	↓	mitochondrial import inner membrane translocase subunit Tim8 A [Myotis davidii]
yakG021238	1.73	6.72E-53	↓	Prohibitin-2 [B. mutus]
ENSP00000295113-D1	1.73	3.06E-270	↓	secreted frizzled-related protein 3 precursor [B. taurus]
ENSBTAP00000044228-D1	1.73	4.13E-147	↓	WD repeat-containing protein 70 [B. mutus]
ENSBTAP00000037395-D7	1.73	2.61E-04	↓	NKG2D ligand 1, partial [B. mutus]
ENSP00000339428-D1	1.73	2.61E-04	↓	suppressor of cytokine signaling 2 isoform X1 [B. mutus]
ENSP00000265872-D1	1.73	1.34E-186	↓	cell division cycle and apoptosis regulator protein 1 isoform X1 [B. mutus]
ENSBTAP00000042628-D1	1.74	0.00E+00	↓	nuclear pore complex protein Nup107 isoform X1 [Bison bison bison]
ENSP00000356780-D1	1.74	0.00E+00	↓	coiled-coil domain-containing protein 181 isoform X1 [B. mutus]
ENSBTAP00000003788-D1	1.74	4.54E-214	↓	Protein CASC5 [Tupaia chinensis]
ENSP00000366813-D1	1.74	0.00E+00	↓	Ubiquitin carboxyl-terminal hydrolase isozyme L3 [B. mutus]
ENSBTAP00000013356-D1	1.74	3.38E-30	↓	cysteine protease ATG4C isoform X1 [B. mutus]
ENSBTAP00000049747-D1	1.74	8.49E-43	↓	Cochlin, partial [B. mutus]
ENSP00000254799-D1	1.74	1.46E-102	↓	G-rich sequence factor 1, partial [B. mutus]
ENSBTAP00000003195-D1	1.75	0.00E+00	↓	39S ribosomal protein L47, mitochondrial isoform X1 [B. mutus]
yakG028134	1.75	4.81E-70	↓	hypothetical protein M91_08854 [B. mutus]
ENSP00000341280-D1	1.75	2.06E-36	↓	protein Jumonji isoform X1 [Physeter catodon]
ENSP00000317175-D1	1.75	3.57E-05	↓	pleckstrin homology domain-containing family D member 1 [B. taurus]
ENSBTAP00000050921-D50	1.75	3.57E-05	↓	Transposon TX1 uncharacterized 149 kDa protein, partial [B. mutus]
ENSP00000222139-D1	1.75	8.46E-04	↓	erythropoietin receptor [B. mutus]
ENSBTAP00000026786-D1	1.75	1.84E-29	↓	Heat shock protein 75 kDa, mitochondrial, partial [B. mutus]
ENSP00000281453-D1	1.75	0.00E+00	↓	Centromere protein U, partial [B. mutus]
ENSBTAP00000010103-D2	1.75	6.82E-14	↓	dihydrofolate reductase [B. taurus]

ENSBTAP00000021217-D1	1.75	1.37E-12	↓	Golgi SNAP receptor complex member 2 [B. mutus]
ENSBTAP00000019839-D1	1.75	9.05E-129	↓	calmodulin-like [B. mutus]
ENSP00000412582-D1	1.76	3.60E-80	↓	Spliceosome RNA helicase BAT1 [B. mutus]
ENSBTAP00000028878-D1	1.76	0.00E+00	↓	histidine protein methyltransferase 1 homolog [B. mutus]
ENSBTAP00000022848-D1	1.76	0.00E+00	↓	hypothetical protein M91_20945 [B. mutus]
ENSBTAP00000049118-D1	1.76	8.63E-148	↓	Centrin-1, partial [B. mutus]
ENSP00000378324-D5	1.76	2.04E-35	↓	DnaJ-like protein subfamily A member 4, partial [B. mutus]
ENSBTAP00000019691-D1	1.76	1.99E-106	↓	Vesicle-trafficking protein SEC22a [B. mutus]
ENSP00000387195-D1	1.76	1.12E-04	↓	Cysteine/serine-rich nuclear protein 3, partial [B. mutus]
ENSBTAP00000036520-D1	1.76	4.14E-14	↓	protein prune homolog 2 [B. mutus]
ENSBTAP00000039209-D1	1.77	0.00E+00	↓	Ras suppressor protein 1, partial [B. mutus]
ENSP00000324463-D1	1.77	1.91E-78	↓	YLP motif-containing protein 1, partial [B. mutus]
ENSP00000324551-D1	1.77	7.05E-18	↓	protein SSUH2 homolog [B. mutus]
ENSP00000261479-D1	1.77	0.00E+00	↓	proteasome subunit alpha type-6 [Rattus norvegicus]
ENSBTAP00000044492-D2	1.77	3.68E-76	↓	charged multivesicular body protein 1b [Capra hircus]
ENSBTAP00000002772-D1	1.77	1.00E-65	↓	Protein-arginine deiminase type-6, partial [B. mutus]
ENSP00000365625-D1	1.77	7.51E-12	↓	putative pre-mRNA-splicing factor ATP-dependent RNA helicase DHX16 [B. mutus]
ENSBTAP00000000786-D1	1.77	1.12E-12	↓	Ankyrin repeat and LEM domain-containing protein 2, partial [B. mutus]
ENSBTAP00000000606-D1	1.77	1.10E-127	↓	DNA-directed RNA polymerases I, II, and III subunit RPABC3 isoform 2 [Homo sapiens]
ENSBTAP00000025036-D1	1.78	1.68E-13	↓	Solute carrier family 25 member 35 [B. mutus]
ENSP00000420617-D1	1.78	0.00E+00	↓	mitofusin-1 [B. taurus]
ENSBTAP00000053069-D5	1.78	8.05E-34	↓	Melanoma-associated antigen B3, partial [B. mutus]
ENSP00000317618-D1	1.78	0.00E+00	↓	DNA polymerase subunit gamma-2, mitochondrial [B. mutus]
ENSBTAP00000016796-D1	1.78	3.83E-193	↓	hypothetical protein M91_08378, partial [B. mutus]
ENSBTAP00000020634-D1	1.78	1.69E-77	↓	Myosin-Id, partial [B. mutus]
ENSP00000380295-D1	1.78	0.00E+00	↓	ATP synthase subunit gamma, mitochondrial, partial [B. mutus]
ENSP00000284136-D1	1.78	2.28E-23	↓	semaphorin-3D [B. mutus]
ENSP00000414066-D1	1.78	0.00E+00	↓	Protein ACN9-like protein, mitochondrial [B. mutus]
ENSBTAP00000009641-D1	1.78	2.20E-34	↓	Histone-lysine N-methyltransferase SETDB2, partial [B. mutus]
ENSBTAP00000000335-D1	1.78	0.00E+00	↓	COBW domain-containing protein 2-like isoform X1 [B. mutus]
ENSBTAP00000022876-D1	1.78	0.00E+00	↓	LOC100138230 protein [B. taurus]
ENSBTAP00000007295-D1	1.78	1.35E-21	↓	hypothetical protein M91_12255, partial [B. mutus]
ENSBTAP00000049867-D3	1.78	1.51E-12	↓	Alpha-actinin-4, partial [B. mutus]
ENSP00000299192-D1	1.78	9.82E-07	↓	HEAT repeat-containing protein 3 [B. taurus]
yakA09846	1.78	6.83E-06	↓	U6 snRNA-associated Sm-like protein LSm6-like [B. mutus]
ENSP00000286353-D1	1.78	3.57E-04	↓	acid phosphatase-like protein 2 [B. mutus]
ENSP00000382213-D1	1.78	2.90E-03	↓	dentin sialophosphoprotein [B. mutus]
ENSBTAP00000007973-D1	1.78	2.90E-03	↓	Coiled-coil and C2 domain-containing protein 1A, partial [B. mutus]
ENSBTAP00000017303-D3	1.78	2.90E-03	↓	guanine nucleotide-binding protein G(i) subunit alpha-2 isoform X1 [Odobenus rosmarus divergens]
ENSBTAP00000015049-D1	1.78	2.90E-03	↓	EMILIN-1 isoform X1 [B. mutus]
ENSBTAP00000019228-D1	1.79	3.34E-186	↓	anaphase-promoting complex subunit 13 isoform X1 [Pantholops hodgsonii]
ENSP00000307701-D2	1.79	0.00E+00	↓	Tetraspanin-5, partial [B. mutus]
ENSBTAP00000046653-D1	1.79	0.00E+00	↓	mitotic spindle assembly checkpoint protein MAD2A-like [B. mutus]
ENSBTAP00000051888-D1	1.79	1.18E-143	↓	DNA mismatch repair protein Msh2 [B. mutus]
ENSP00000349156-D1	1.79	2.95E-268	↓	E3 ubiquitin-protein ligase SIAH1 [B. taurus]
ENSBTAP00000052997-D1	1.79	1.29E-292	↓	probable arginine--tRNA ligase, mitochondrial [B. mutus]
ENSP00000260361-D1	1.79	5.68E-58	↓	complex I intermediate-associated protein 30, mitochondrial [B. mutus]
ENSP00000403817-D1	1.80	0.00E+00	↓	Nascent polypeptide-associated complex subunit alpha, muscle-specific form, partial [B. mutus]
ENSBTAP00000020681-D1	1.80	4.19E-79	↓	mitochondrial import inner membrane translocase subunit TIM44 [B. mutus]
ENSBTAP00000049562-D43	1.80	4.34E-07	↓	Transposon TX1 uncharacterized 149 kDa protein, partial [B. mutus]
ENSP00000326417-D1	1.80	3.00E-06	↓	Krueppel-like factor 4, partial [B. mutus]
ENSBTAP00000011898-D1	1.80	0.00E+00	↓	centromere protein E, 312kDa [B. mutus]
ENSBTAP00000000493-D1	1.80	2.03E-52	↓	mediator of RNA polymerase II transcription subunit 27 [B. taurus]
ENSP00000413575-D1	1.80	0.00E+00	↓	Peroxisomal carnitine O-octanoyltransferase, partial [B. mutus]
ENSP00000343081-D1	1.80	7.34E-198	↓	pseudouridylate synthase 7 homolog-like protein-like [B. mutus]

ENSBTAP00000018363-D1	1.81	4.86E-180	↓	GAS2-like protein 3, partial [B. mutus]
ENSP00000386674-D1	1.81	3.07E-24	↓	Coiled-coil domain-containing protein 148, partial [B. mutus]
ENSP00000408263-D1	1.81	5.91E-62	↓	selenium-binding protein 1 [B. mutus]
ENSBTAP00000051671-D33	1.81	1.53E-04	↓	hypothetical protein M91_08065 [B. mutus]
yakA21566	1.81	1.53E-04	↓	hypothetical protein M91_11360 [B. mutus]
ENSP00000341684-D1	1.81	3.32E-58	↓	coiled-coil domain-containing protein 15 isoform X1 [Bison bison bison]
ENSBTAP00000005760-D1	1.81	3.96E-52	↓	poly(A) RNA polymerase, mitochondrial, partial [B. mutus]
ENSP00000359802-D1	1.82	5.46E-255	↓	zinc finger protein 75D-like [B. mutus]
ENSBTAP00000012301-D1	1.82	1.49E-42	↓	archaemetzincin-2 isoform X1 [B. mutus]
ENSBTAP00000021980-D1	1.82	2.48E-15	↓	nucleolar complex protein 2 homolog [B. mutus]
ENSP00000385089-D1	1.82	0.00E+00	↓	hypothetical protein M91_03007, partial [B. mutus]
ENSP00000346437-D1	1.82	7.90E-21	↓	Ubiquitin-like modifier-activating enzyme ATG7, partial [B. mutus]
ENSP00000365505-D1	1.82	4.84E-70	↓	proteolipid protein 2 [B. taurus]
ENSBTAP00000038340-D1	1.82	1.22E-49	↓	Mitogen-activated protein kinase kinase kinase MLT [B. mutus]
ENSP00000380397-D1	1.83	2.48E-50	↓	threonylcarbamoyladenosine tRNA methylthiotransferase [Bison bison bison]
ENSP00000398244-D1	1.83	3.34E-15	↓	hsp70-binding protein 1 [B. taurus]
yakG041173	1.83	4.85E-30	↓	Protein phosphatase 1 regulatory subunit 15A [B. mutus]
ENSP00000361531-D1	1.83	1.18E-03	↓	hypothetical protein M91_07524, partial [B. mutus]
ENSBTAP00000013714-D1	1.83	0.00E+00	↓	protein FAM188A [B. mutus]
ENSP00000341159-D1	1.83	9.26E-27	↓	Ceramide kinase-like protein, partial [B. mutus]
ENSP00000377738-D1	1.83	3.85E-42	↓	polypyrimidine tract-binding protein 2 isoform X8 [Ovis aries musimon]
ENSP00000405252-D1	1.84	5.95E-24	↓	A-kinase anchor protein 7 isoform gamma isoform X2 [B. taurus]
ENSP00000239906-D1	1.84	3.88E-91	↓	protein FAM53C [B. taurus]
ENSP00000315713-D1	1.84	1.79E-06	↓	Protein phosphatase Slingshot-like protein 1, partial [B. mutus]
ENSBTAP00000020727-D1	1.84	4.61E-114	↓	UPF0544 protein C5orf45 homolog [B. mutus]
ENSBTAP00000052020-D1	1.84	0.00E+00	↓	hypothetical protein M91_16189 [B. mutus]
ENSP00000262134-D1	1.84	8.33E-19	↓	lysophosphatidylcholine acyltransferase 2 [B. mutus]
ENSBTAP00000008956-D1	1.84	5.98E-15	↓	transmembrane protein 185A isoform X2 [Ovis aries musimon]
ENSBTAP00000048503-D1	1.85	1.02E-28	↓	pejvakin [B. mutus]
ENSP00000381293-D1	1.85	1.76E-187	↓	Vesicle-fusing ATPase, partial [B. mutus]
ENSBTAP00000003010-D4	1.85	1.24E-164	↓	U6 snRNA-associated Sm-like protein LSm5 isoform X1 [Cavia porcellus]
ENSBTAP00000008111-D1	1.85	8.50E-38	↓	RNA-binding protein 45, partial [B. mutus]
ENSBTAP00000028113-D1	1.85	3.03E-28	↓	solute carrier family 35 member F5 isoform X1 [B. taurus]
ENSP00000312355-D1	1.85	3.62E-41	↓	hypothetical protein M91_17088, partial [B. mutus]
ENSP00000216068-D1	1.85	2.27E-08	↓	dynein light chain 4, axonemal [Homo sapiens]
ENSBTAP00000006322-D1	1.85	6.65E-10	↓	Type I inositol-3,4-bisphosphate 4-phosphatase [B. mutus]
ENSP00000382450-D1	1.85	6.56E-73	↓	zinc finger protein 131 isoform X1 [B. mutus]
ENSBTAP00000012726-D1	1.85	4.14E-127	↓	Myotubularin-related protein 1, partial [B. mutus]
ENSP00000326806-D1	1.85	2.96E-48	↓	nuclear cap-binding protein subunit 2 isoform X2 [Chlorocebus sabaeus]
ENSBTAP00000003764-D2	1.85	4.83E-41	↓	Importin subunit alpha-1, partial [B. mutus]
ENSP00000264079-D1	1.86	2.98E-10	↓	mucolipin-1 [Pantholops hodgsonii]
ENSBTAP00000024029-D1	1.86	1.01E-08	↓	MAP kinase-interacting serine/threonine-protein kinase 2, partial [B. mutus]
ENSP00000415939-D1	1.86	2.86E-24	↓	Zinc finger BED domain-containing protein 5 [B. mutus]
ENSBTAP00000002071-D1	1.86	1.53E-152	↓	Calmegin, partial [B. mutus]
ENSBTAP00000033454-D1	1.86	1.20E-27	↓	Choline transporter-like protein 3, partial [B. mutus]
ENSBTAP00000016937-D1	1.86	3.95E-12	↓	NEDD8-conjugating enzyme Ubc12 [Cercocebus atys]
ENSBTAP00000024173-D2	1.86	4.47E-09	↓	Laminin subunit alpha-2, partial [B. mutus]
ENSP00000325161-D1	1.86	7.15E-186	↓	solute carrier family 35, member E3 [Balaenoptera acutorostrata scammoni]
ENSP00000363308-D1	1.86	1.52E-93	↓	WD repeat-containing protein 31 isoform X1 [B. mutus]
ENSP00000225698-D1	1.86	5.02E-207	↓	complement component 1 Q subcomponent-binding protein, mitochondrial [B. mutus]
ENSBTAP00000019583-D1	1.86	5.59E-23	↓	Cystatin-M [B. mutus]
ENSBTAP00000025324-D1	1.86	3.10E-89	↓	storkhead-box protein 1 isoform X1 [B. mutus]
ENSP00000350945-D1	1.87	4.94E-39	↓	Myosin-Va, partial [B. mutus]
ENSP00000339484-D1	1.87	1.45E-16	↓	AN1-type zinc finger and ubiquitin domain-containing protein 1 [B. mutus]
ENSP00000366636-D98	1.87	0.00E+00	↓	hypothetical protein M91_13120, partial [B. mutus]

ENSP00000369699-D1	1.87	5.08E-24	↓	Collectrin [B. mutus]
ENSBTAP00000033692-D1	1.87	3.62E-88	↓	Pendrin [B. mutus]
ENSBTAP00000025389-D4	1.87	1.18E-86	↓	epithelial membrane protein 3 [B. taurus]
ENSP00000178640-D1	1.87	3.97E-10	↓	dual specificity mitogen-activated protein kinase kinase 5 [B. taurus]
ENSP00000378110-D1	1.87	4.75E-68	↓	septin-6 isoform X1 [Trichechus manatus latirostris]
ENSBTAP00000044575-D1	1.88	7.64E-128	↓	Superkiller viralicidic activity 2-like 2, partial [B. mutus]
ENSBTAP00000025042-D1	1.88	1.48E-64	↓	Nucleolar protein 56, partial [B. mutus]
ENSP00000351737-D1	1.88	2.17E-221	↓	transmembrane protein 126B [B. mutus]
yakG041387	1.88	1.06E-06	↓	hypothetical protein M91_02485 [B. mutus]
ENSBTAP00000007175-D1	1.88	3.39E-82	↓	FYVE, RhoGEF and PH domain-containing protein 4 isoform X1 [B. mutus]
ENSP00000355961-D1	1.88	7.00E-127	↓	Integrator complex subunit 7 [B. mutus]
ENSBTAP00000008107-D1	1.88	1.07E-65	↓	peroxiredoxin-4 precursor [B. taurus]
ENSBTAP00000047871-D2	1.89	3.94E-76	↓	28S ribosomal protein S17, mitochondrial isoform X1 [B. mutus]
ENSBTAP00000012183-D1	1.89	8.71E-256	↓	Protein dpy-30-like protein, partial [B. mutus]
ENSP00000324587-D1	1.89	2.06E-07	↓	Protein FAM193A, partial [B. mutus]
ENSBTAP00000028238-D1	1.89	6.00E-46	↓	Cadherin-2, partial [B. mutus]
ENSBTAP00000014027-D1	1.89	1.54E-41	↓	transmembrane emp24 domain-containing protein 4 precursor [B. taurus]
ENSBTAP00000052701-D2	1.89	9.05E-08	↓	hypothetical protein M91_09636, partial [B. mutus]
ENSBTAP00000013429-D1	1.89	3.85E-05	↓	Vesicle transport protein GOT1A, partial [B. mutus]
ENSBTAP00000047500-D1	1.89	3.85E-05	↓	Coiled-coil domain-containing protein 160, partial [B. mutus]
ENSBTAP00000019823-D1	1.89	3.85E-05	↓	cytochrome P450 19A1-like [B. mutus]
ENSP00000245157-D1	1.90	3.78E-205	↓	Bardet-Biedl syndrome 2 protein [B. mutus]
ENSBTAP00000027791-D1	1.90	1.22E-41	↓	Regulator of nonsense transcripts 3A, partial [B. mutus]
ENSBTAP00000026871-D1	1.90	3.40E-84	↓	huntingtin interacting protein 2, isoform CRA_c [Mus musculus]
ENSP00000356520-D1	1.90	2.37E-69	↓	ATP-dependent RNA helicase A isoform X1 [Bison bison bison]
yakG013249	1.90	0.00E+00	↓	structural maintenance of chromosomes protein 4 isoform X4 [Leptonychotes weddellii]
ENSBTAP00000053633-D1	1.90	2.24E-15	↓	Ectonucleoside triphosphate diphosphohydrolase 4, partial [B. mutus]
ENSBTAP00000017427-D1	1.91	3.53E-52	↓	keratinocyte-associated transmembrane protein 2 [B. mutus]
ENSBTAP00000021022-D1	1.91	5.75E-296	↓	Coiled-coil domain-containing protein 65 [B. mutus]
ENSP00000342699-D1	1.91	1.19E-116	↓	Coiled-coil domain-containing protein 104 [B. mutus]
ENSBTAP00000042732-D1	1.91	3.21E-06	↓	hypothetical protein M91_00961, partial [B. mutus]
ENSP00000416015-D1	1.91	4.51E-21	↓	protein-methionine sulfoxide oxidase MICAL3 [B. mutus]
ENSBTAP00000013082-D2	1.91	0.00E+00	↓	Aldose reductase, partial [B. mutus]
yakA14331	1.91	9.10E-22	↓	T-complex protein 1 subunit theta-like [B. mutus]
ENSBTAP00000028203-D1	1.92	0.00E+00	↓	Cytoskeleton-associated protein 2, partial [B. mutus]
ENSBTAP00000017614-D1	1.92	3.47E-58	↓	4F2 cell-surface antigen heavy chain isoform X1 [B. mutus]
ENSBTAP00000047415-D1	1.92	1.07E-51	↓	LOC535754 protein [B. taurus]
ENSBTAP00000019203-D1	1.92	0.00E+00	↓	Proteasome subunit alpha type-4, partial [Heterocephalus glaber]
ENSP00000384796-D1	1.92	4.99E-60	↓	Pro-neuregulin-3, membrane-bound isoform, partial [B. mutus]
ENSP00000376472-D1	1.92	8.35E-133	↓	dolichyl-diphosphooligosaccharide--protein glycosyltransferase subunit STT3A [B. taurus]
ENSBTAP00000025183-D1	1.93	1.06E-08	↓	Glutamine-rich protein 1 [B. mutus]
ENSBTAP00000008082-D1	1.93	1.20E-203	↓	probable ATP-dependent RNA helicase DHX36 isoform X1 [B. mutus]
ENSP00000332444-D2	1.93	6.52E-28	↓	Cleavage stimulation factor subunit 2 [B. mutus]
ENSBTAP00000040572-D1	1.93	7.10E-17	↓	Tissue alpha-L-fucosidase, partial [B. mutus]
ENSP00000279206-D1	1.93	4.14E-10	↓	nucleoside diphosphate-linked moiety X motif 22 [B. mutus]
ENSBTAP00000042059-D1	1.94	0.00E+00	↓	Syntaxin-8, partial [B. mutus]
ENSP00000337122-D77	1.94	4.66E-21	↓	Zinc finger protein 75A, partial [B. mutus]
ENSBTAP00000053637-D1	1.94	1.89E-22	↓	PAB-dependent poly(A)-specific ribonuclease subunit 2, partial [B. mutus]
ENSP00000314153-D1	1.94	0.00E+00	↓	transcriptional regulator Kaiso [B. mutus]
ENSP00000405473-D1	1.94	4.58E-29	↓	Mitochondrial inner membrane protease subunit 2 [B. mutus]
ENSP00000390190-D1	1.94	4.19E-17	↓	Coiled-coil domain-containing protein 141, partial [B. mutus]
ENSP00000250416-D1	1.94	3.39E-32	↓	Poly [ADP-ribose] polymerase 2, partial [B. mutus]
ENSP00000262650-D2	1.94	2.37E-112	↓	E3 ubiquitin-protein ligase Itchy homolog [B. mutus]
ENSP00000365782-D1	1.94	3.19E-68	↓	Centrosomal protein of 78 kDa, partial [B. mutus]
ENSP00000339245-D1	1.95	6.05E-56	↓	YTH domain-containing protein 1 [B. taurus]

ENSBTAP00000006653-D1	1.95	0.00E+00	↓	metalloproteinase inhibitor 1 precursor [B. taurus]
ENSP00000378119-D1	1.95	1.85E-60	↓	metalloreductase STEAP2 [B. taurus]
ENSBTAP00000004418-D2	1.95	0.00E+00	↓	splicing factor 3B subunit 6 [Homo sapiens]
ENSBTAP00000017392-D1	1.95	0.00E+00	↓	Phosphoserine phosphatase, partial [B. mutus]
ENSP00000383298-D16	1.95	7.03E-101	↓	hypothetical protein M91_00743 [B. mutus]
ENSBTAP00000017298-D1	1.95	2.57E-206	↓	phospholipase A-2-activating protein isoform X1 [Bison bison bison]
ENSP00000319371-D1	1.95	7.94E-37	↓	G protein pathway suppressor 2 [Balaenoptera acutorostrata scammoni]
ENSP00000293273-D1	1.95	8.58E-13	↓	RAD52 motif-containing protein 1-like isoform X1 [B. mutus]
ENSBTAP00000031777-D1	1.95	2.24E-05	↓	Protein asteroid-like protein 1 [B. mutus]
ENSP00000412798-D1	1.95	6.61E-04	↓	Kinesin-like protein KIF18B [B. mutus]
ENSP00000319756-D1	1.95	1.60E-03	↓	tensin-like C1 domain-containing phosphatase isoform X4 [B. taurus]
ENSP00000309714-D1	1.95	4.06E-03	↓	SH3 and PX domain-containing protein 2B, partial [B. mutus]
ENSP00000367345-D7	1.95	4.06E-03	↓	Protocadherin gamma-A7, partial [B. mutus]
ENSBTAP00000028368-D1	1.95	4.06E-03	↓	Syntaxin-binding protein 6, partial [B. mutus]
ENSBTAP00000019304-D1	1.95	4.06E-03	↓	GTP-binding protein Di-Ras2 [B. taurus]
ENSP00000369976-D1	1.95	1.11E-02	↓	epidermal growth factor-like protein 6 isoform X1 [B. taurus]
ENSBTAP00000032431-D1	1.95	1.11E-02	↓	paraplegin, partial [B. mutus]
ENSP00000291890-D7	1.95	1.11E-02	↓	Platelet glycoprotein VI, partial [B. mutus]
ENSP00000412971-D27	1.95	1.11E-02	↓	tubulin alpha-1C chain-like [B. mutus]
ENSP00000375080-D1	1.95	1.11E-02	↓	serine/threonine-protein phosphatase 2A regulatory subunit B'' subunit beta [B. mutus]
ENSBTAP00000017670-D1	1.95	1.11E-02	↓	smoothened homolog precursor [B. taurus]
ENSBTAP00000002098-D1	1.95	1.11E-02	↓	interleukin-4 receptor subunit alpha [B. mutus]
ENSP00000204517-D1	1.95	1.11E-02	↓	transcription factor AP-4 [B. taurus]
ENSBTAP00000019311-D1	1.95	3.63E-07	↓	Carbonyl reductase [NADPH] 1, partial [B. mutus]
ENSP00000349351-D1	1.95	2.83E-04	↓	Protein bicaudal D-like protein 2 [B. mutus]
ENSBTAP00000005975-D1	1.96	0.00E+00	↓	oxidized low-density lipoprotein receptor 1 isoform X1 [B. mutus]
ENSP00000352254-D1	1.96	5.80E-43	↓	transportin-3 [Tursiops truncatus]
ENSP00000307833-D1	1.96	2.49E-71	↓	F-box only protein 22 [B. mutus]
ENSBTAP00000043009-D1	1.96	6.76E-80	↓	GTP-binding protein 10 [B. mutus]
ENSBTAP00000001995-D1	1.96	2.50E-38	↓	tyrosine-protein kinase Yes [B. taurus]
ENSP00000376339-D1	1.97	7.25E-17	↓	Adapter protein CIKS [B. mutus]
ENSP00000309020-D1	1.97	4.02E-15	↓	Glycogen debranching enzyme, partial [B. mutus]
ENSBTAP00000005628-D1	1.97	0.00E+00	↓	NADH dehydrogenase [ubiquinone] 1 alpha subcomplex subunit 8, partial [B. mutus]
ENSBTAP00000042959-D1	1.97	5.64E-12	↓	Cation channel sperm-associated protein 2 [B. mutus]
ENSBTAP00000005236-D1	1.97	1.03E-97	↓	N-acetylgalactosamine kinase, partial [B. mutus]
ENSBTAP00000042742-D1	1.97	2.85E-11	↓	cyclic AMP-dependent transcription factor ATF-6 beta isoform X2 [B. mutus]
ENSP00000359160-D1	1.97	1.79E-138	↓	tRNA:m(4)X modification enzyme TRM13 homolog [B. mutus]
ENSBTAP00000005990-D1	1.98	3.06E-94	↓	Elongator complex protein 4 [B. mutus]
ENSP00000384411-D1	1.98	1.68E-209	↓	mitochondrial import receptor subunit TOM5 homolog [Propithecus coquereli]
ENSBTAP00000020832-D1	1.98	7.75E-269	↓	cytochrome c oxidase assembly protein COX16 homolog, mitochondrial [B. taurus]
ENSP00000375773-D1	1.98	6.66E-23	↓	Protein-cysteine N-palmitoyltransferase HHAT, partial [B. mutus]
ENSBTAP00000027191-D1	1.98	0.00E+00	↓	NADH dehydrogenase [ubiquinone] 1 beta subcomplex subunit 9 [B. taurus]
ENSBTAP00000007060-D1	1.98	1.23E-40	↓	Ovochymase-1, partial [B. mutus]
ENSP00000234301-D1	1.98	0.00E+00	↓	cytochrome c oxidase subunit 7A-related protein, mitochondrial-like [B. mutus]
ENSP00000291458-D2	1.99	0.00E+00	↓	Coiled-coil domain-containing protein 58, partial [B. mutus]
ENSBTAP00000019577-D1	1.99	5.27E-47	↓	putative ATP-dependent Clp protease proteolytic subunit, mitochondrial [B. mutus]
ENSBTAP00000034848-D1	1.99	3.59E-142	↓	Chromodomain-helicase-DNA-binding protein 1, partial [B. mutus]
ENSP00000376598-D1	1.99	0.00E+00	↓	origin recognition complex subunit 4 [B. taurus]
ENSBTAP00000012034-D1	1.99	7.15E-154	↓	DNA polymerase iota, partial [B. mutus]
ENSBTAP00000007027-D1	1.99	7.31E-162	↓	glucosamine 6-phosphate N-acetyltransferase isoform X1 [Ovis aries]
ENSBTAP00000021202-D1	1.99	3.63E-29	↓	calcium uptake protein 2, mitochondrial [Bison bison bison]
ENSBTAP00000036858-D1	1.99	4.80E-07	↓	protein LBH [B. taurus]
ENSBTAP00000050555-D1	2.00	2.65E-19	↓	RAB6-interacting golgin [B. mutus]
ENSBTAP00000008321-D1	2.00	2.57E-118	↓	Solute carrier family 35 member F4, partial [B. mutus]
ENSBTAP00000044534-D1	2.00	1.75E-31	↓	Phosphatidylinositol 4-kinase type 2-beta, partial [B. mutus]

ENSP00000310453-D1	2.00	1.36E-39	↓	folliculin-interacting protein 1 isoform X1 [B. mutus]
ENSP00000267973-D2	2.00	0.00E+00	↓	WD repeat-containing protein 61 isoform X1 [Trichechus manatus latirostris]
ENSBTAP00000046417-D1	2.00	1.88E-10	↓	uncharacterized protein C7orf57 homolog [Pantholops hodgsonii]
ENSP00000415464-D1	2.00	4.69E-120	↓	la-related protein 4 [B. taurus]
ENSBTAP00000048398-D1	2.00	2.46E-06	↓	Monoacylglycerol lipase ABHD12, partial [B. mutus]
ENSBTAP00000010329-D1	2.00	2.46E-06	↓	V-set and immunoglobulin domain-containing protein 10 [B. mutus]
ENSP00000356846-D1	2.00	0.00E+00	↓	protein maelstrom homolog isoform X1 [B. taurus]
ENSBTAP00000017640-D1	2.01	1.55E-19	↓	pre-mRNA-splicing factor ATP-dependent RNA helicase PRP16 [B. mutus]
ENSP00000338034-D1	2.01	2.12E-09	↓	SPRY domain-containing protein 4 [Pantholops hodgsonii]
ENSBTAP00000005379-D1	2.01	2.70E-76	↓	5-azacytidine-induced protein 2 [B. mutus]
ENSBTAP00000013355-D1	2.01	4.05E-289	↓	Histone acetyltransferase type B catalytic subunit, partial [B. mutus]
ENSP00000369004-D1	2.01	7.64E-68	↓	hypothetical protein M91_10668 [B. mutus]
ENSP00000261813-D1	2.01	0.00E+00	↓	prefoldin subunit 1 [B. taurus]
ENSP00000364398-D1	2.01	4.75E-09	↓	intracellular hyaluronan-binding protein 4 [B. mutus]
ENSBTAP00000010234-D2	2.01	1.29E-05	↓	N-alpha-acetyltransferase 50-like [B. mutus]
ENSBTAP00000015408-D3	2.01	6.13E-255	↓	splicing factor, arginine/serine-rich 5, isoform CRA_e, partial [Homo sapiens]
ENSBTAP00000015172-D1	2.02	0.00E+00	↓	Stress-70 protein, mitochondrial, partial [B. mutus]
ENSBTAP00000018153-D13	2.02	4.77E-70	↓	60S ribosomal protein L22-like 1 [Ovis aries]
ENSBTAP00000012175-D1	2.02	4.60E-24	↓	ER lumen protein-retaining receptor 2, partial [Ursus maritimus]
ENSP00000381034-D3	2.02	2.97E-05	↓	suppressor of cytokine signaling 6 [Ovis aries]
ENSBTAP00000019928-D6	2.02	3.56E-244	↓	ragulator complex protein LAMTOR5 [Camelus bactrianus]
ENSP00000272091-D1	2.02	6.39E-111	↓	protein SDE2 homolog [B. mutus]
ENSBTAP00000047439-D1	2.02	0.00E+00	↓	thyroid transcription factor 1-associated protein 26-like [B. mutus]
ENSP00000334373-D2	2.02	6.47E-64	↓	importin subunit alpha-4 [Bison bison bison]
ENSP00000322609-D1	2.03	0.00E+00	↓	hypothetical protein M91_20100 [B. mutus]
ENSBTAP00000022607-D1	2.03	1.26E-78	↓	Tyrosine-protein kinase RYK, partial [B. mutus]
ENSBTAP00000026252-D1	2.03	1.11E-115	↓	zinc finger protein Rlf [B. mutus]
yakA09785	2.03	6.87E-05	↓	40S ribosomal protein S24 [B. mutus]
ENSP00000358272-D1	2.03	0.00E+00	↓	NADH dehydrogenase [ubiquinone] 1 alpha subcomplex assembly factor 4 [B. mutus]
ENSP00000385614-D1	2.04	9.96E-254	↓	Double-strand break repair protein MRE11A [B. mutus]
ENSP00000374856-D5	2.04	6.15E-16	↓	hypothetical protein M91_04407, partial [B. mutus]
ENSBTAP00000053264-D1	2.04	3.48E-24	↓	Voltage-dependent calcium channel subunit alpha-2/delta-1, partial [B. mutus]
ENSBTAP00000019620-D1	2.04	6.21E-09	↓	Thioredoxin domain-containing protein 15, partial [B. mutus]
ENSBTAP00000005622-D1	2.04	6.33E-11	↓	probable ATP-dependent RNA helicase DDX28 [B. mutus]
ENSBTAP00000018986-D1	2.04	1.41E-41	↓	Zinc finger FYVE domain-containing protein 9, partial [B. mutus]
ENSP00000378367-D1	2.05	2.31E-55	↓	pro-neuregulin-4, membrane-bound isoform [B. mutus]
ENSBTAP00000009709-D1	2.05	8.26E-183	↓	hypothetical protein M91_16488, partial [B. mutus]
ENSBTAP00000049765-D24	2.05	2.72E-124	↓	Zinc finger protein 239, partial [B. mutus]
ENSP00000416084-D1	2.05	2.64E-48	↓	tudor domain-containing protein 15-like [B. mutus]
ENSBTAP00000025112-D2	2.05	5.83E-178	↓	DNA-directed primase/polymerase protein isoform X6 [B. taurus]
ENSP00000345689-D1	2.06	7.27E-27	↓	ras-related protein Rab-5C [B. taurus]
ENSBTAP00000002462-D1	2.06	0.00E+00	↓	hypothetical protein M91_08640, partial [B. mutus]
ENSBTAP00000041966-D1	2.07	4.61E-16	↓	Calpain-13 [B. mutus]
ENSBTAP00000004504-D1	2.07	7.51E-24	↓	hypothetical protein M91_02902, partial [B. mutus]
ENSBTAP00000023655-D1	2.07	3.72E-04	↓	B9 domain-containing protein 1 precursor [B. taurus]
ENSP00000381461-D1	2.07	0.00E+00	↓	Protein phosphatase methylesterase 1 [B. mutus]
yakG027374	2.07	5.38E-17	↓	Syntaxin-binding protein 6 [B. mutus]
ENSP00000418086-D1	2.08	9.69E-37	↓	uncharacterized protein C3orf14 homolog [B. mutus]
ENSP00000350009-D1	2.08	3.12E-179	↓	Ubiquitin carboxyl-terminal hydrolase 33, partial [B. mutus]
yakA26379	2.08	3.29E-19	↓	Histone-arginine methyltransferase CARM1 [B. mutus]
ENSP00000347463-D1	2.08	1.76E-113	↓	Transcription factor COE3, partial [B. mutus]
ENSP00000359982-D1	2.08	5.71E-77	↓	ankyrin repeat domain-containing protein 13C isoform X1 [Pantholops hodgsonii]
ENSBTAP00000043944-D1	2.08	0.00E+00	↓	THO complex subunit 7-like protein, partial [B. mutus]
yakG032272	2.09	1.80E-08	↓	hypothetical protein M91_21689 [B. mutus]
ENSP00000376110-D55	2.09	1.69E-05	↓	Zinc finger protein 555, partial [B. mutus]

ENSP00000406318-D88	2.09	4.72E-60	↓	Putative zinc finger protein 724, partial [B. mutus]
ENSP00000397644-D1	2.09	1.41E-72	↓	DNA-binding protein RFX7 [B. mutus]
ENSBTAP00000002964-D1	2.09	2.05E-239	↓	cytoskeleton-associated protein 2-like [B. mutus]
yakG002790	2.10	2.40E-288	↓	Ribosome biogenesis protein NSA2-like protein [B. mutus]
ENSBTAP00000048093-D1	2.10	9.96E-52	↓	Coiled-coil domain-containing protein 130, partial [B. mutus]
ENSBTAP00000047104-D2	2.10	0.00E+00	↓	ATP-dependent RNA helicase DDX3X, partial [B. mutus]
ENSP00000347322-D1	2.10	3.50E-94	↓	hypothetical protein M91_20452, partial [B. mutus]
ENSBTAP00000046135-D1	2.10	9.70E-118	↓	tRNA guanosine-2'-O-methyltransferase TRM11-like protein, partial [B. mutus]
ENSP00000419242-D1	2.10	0.00E+00	↓	RNA-binding protein 48 [B. mutus]
ENSBTAP00000006502-D1	2.10	0.00E+00	↓	Exosome complex exonuclease RRP45 [B. mutus]
ENSBTAP00000049380-D4	2.11	3.26E-14	↓	T-complex protein 1 subunit beta, partial [B. mutus]
ENSBTAP00000048364-D3	2.11	8.85E-04	↓	vesicle transport protein SFT2A [B. taurus]
ENSP00000315476-D1	2.11	6.91E-42	↓	Exosome complex exonuclease RRP41 [B. mutus]
ENSP00000394741-D1	2.12	1.03E-08	↓	Target of Myb protein 1, partial [B. mutus]
ENSBTAP00000002876-D1	2.12	1.29E-208	↓	ribonuclease P protein subunit p29 isoform X1 [B. mutus]
ENSP00000359025-D1	2.12	0.00E+00	↓	Syntaxin-binding protein 3, partial [B. mutus]
ENSP00000288207-D1	2.12	0.00E+00	↓	G2/mitotic-specific cyclin-B2 [B. mutus]
ENSP00000261973-D1	2.12	0.00E+00	↓	RBM25 protein [Homo sapiens]
ENSP00000390428-D1	2.12	4.01E-34	↓	creatine kinase U-type, mitochondrial [Bison bison bison]
ENSP00000370889-D1	2.12	4.20E-06	↓	DENN domain-containing protein 1C, partial [B. mutus]
ENSP00000261416-D2	2.13	8.04E-13	↓	beta-hexosaminidase subunit beta-like [B. mutus]
ENSBTAP00000001019-D1	2.13	3.20E-90	↓	glucose 1,6-bisphosphate synthase [B. mutus]
ENSP00000349906-D1	2.13	5.32E-212	↓	protein RTF2 homolog isoform X1 [Pantholops hodgsonii]
ENSBTAP00000024424-D4	2.13	1.24E-81	↓	40S ribosomal protein S23, partial [B. mutus]
ENSBTAP00000026163-D1	2.14	0.00E+00	↓	sorting nexin-16 isoform X1 [B. mutus]
ENSBTAP00000007896-D1	2.14	8.90E-05	↓	stabilin-2 [B. mutus]
ENSBTAP00000000733-D1	2.14	6.10E-114	↓	Mitochondrial import receptor subunit TOM20-like protein, partial [B. mutus]
ENSBTAP00000039864-D1	2.14	2.75E-119	↓	Retinoic acid receptor responder protein 1, partial [B. mutus]
ENSBTAP00000010676-D1	2.14	0.00E+00	↓	ribosome production factor 1 [B. taurus]
ENSBTAP00000006987-D1	2.15	9.49E-06	↓	DNA-directed RNA polymerase III subunit RPC8 isoform X1 [Pantholops hodgsonii]
ENSP00000403117-D1	2.15	0.00E+00	↓	Major facilitator superfamily domain-containing protein 1, partial [B. mutus]
ENSP00000360182-D1	2.15	2.32E-280	↓	Dedicator of cytokinesis protein 7, partial [B. mutus]
ENSBTAP00000033540-D1	2.15	1.05E-06	↓	DENN domain-containing protein 2A [B. mutus]
ENSBTAP00000005496-D1	2.16	8.89E-12	↓	Diablo-like protein, mitochondrial, partial [B. mutus]
ENSP00000392892-D1	2.16	0.00E+00	↓	SAP domain-containing ribonucleoprotein, partial [B. mutus]
ENSP00000362144-D1	2.16	9.64E-18	↓	calcium uniporter protein, mitochondrial-like isoform X1 [Bubalus bubalis]
ENSP00000405890-D2	2.16	1.55E-49	↓	Pre-B-cell leukemia transcription factor 3, partial [B. mutus]
ENSP00000333374-D1	2.17	8.23E-36	↓	coiled-coil domain-containing protein 60 [Bison bison bison]
ENSBTAP00000001421-D1	2.17	2.90E-19	↓	telomerase Cajal body protein 1 [B. mutus]
ENSP00000251588-D1	2.17	2.27E-12	↓	cytosolic Fe-S cluster assembly factor NARFL [B. mutus]
ENSBTAP00000025572-D1	2.17	0.00E+00	↓	Ribosome biogenesis protein BRX1-like protein, partial [B. mutus]
ENSBTAP00000021300-D1	2.17	0.00E+00	↓	alpha-synuclein [B. taurus]
ENSBTAP00000005939-D1	2.17	1.98E-59	↓	U6 snRNA-associated Sm-like protein LSm7 [Lipotes vexillifer]
ENSP00000343785-D1	2.17	9.95E-27	↓	protein sprouty homolog 1 [B. taurus]
ENSBTAP00000014010-D1	2.17	0.00E+00	↓	gamma-glutamylcyclotransferase [B. taurus]
ENSP00000295452-D1	2.17	1.43E-37	↓	gamma-aminobutyric acid receptor subunit gamma-1 precursor [B. taurus]
ENSBTAP00000026373-D1	2.18	4.37E-125	↓	regulator of G-protein signaling 22 [B. mutus]
ENSP00000373594-D1	2.18	3.84E-10	↓	transmembrane protein C15orf27 homolog [B. mutus]
ENSP00000334314-D2	2.18	2.36E-06	↓	Echinoderm microtubule-associated protein-like 2 [B. mutus]
ENSP00000352252-D18	2.18	2.07E-04	↓	core histone H2A/H2B/H3/H4 [Ancylostoma ceylanicum]
ENSP00000257262-D1	2.18	2.31E-217	↓	putative nef1 variant 1 [Taeniopygia guttata]
ENSP00000251250-D1	2.18	6.86E-255	↓	DTW domain-containing protein 1 isoform X1 [B. taurus]
ENSBTAP00000005174-D1	2.18	8.12E-179	↓	ubiquitin thioesterase OTUB1 [B. taurus]
ENSBTAP00000015719-D1	2.18	1.81E-37	↓	ATPase ASNA1 [Chlorocebus sabaeus]
ENSP00000406490-D1	2.18	7.40E-141	↓	PMS1 protein homolog 1 [B. mutus]

ENSBTAP00000003795-D1	2.18	5.16E-30	↓	regulator of microtubule dynamics protein 3 [B. taurus]
ENSP00000356700-D1	2.18	2.33E-16	↓	uncharacterized protein C1orf105 homolog [B. mutus]
ENSBTAP00000053199-D3	2.18	2.33E-16	↓	Teneurin-1, partial [B. mutus]
ENSP00000340211-D2	2.18	8.84E-42	↓	Coronin-1C, partial [B. mutus]
ENSP00000394682-D1	2.18	2.00E-15	↓	Putative ATP-dependent RNA helicase DHX30 [B. mutus]
ENSP00000271308-D1	2.19	0.00E+00	↓	Proteasome subunit alpha type-5, partial [B. mutus]
ENSP00000328187-D1	2.19	1.12E-11	↓	F-box only protein 21 isoform X3 [Ovis aries musimon]
ENSP00000340671-D1	2.19	0.00E+00	↓	DNA damage-inducible transcript 3 protein [B. taurus]
ENSBTAP00000018338-D1	2.19	9.82E-11	↓	phosphoribosyl pyrophosphate synthase-associated protein 1 [B. taurus]
ENSP00000218432-D1	2.19	5.88E-72	↓	Peptidyl-prolyl cis-trans isomerase NIMA-interacting 4, partial [B. mutus]
ENSBTAP00000006760-D3	2.19	0.00E+00	↓	proteasome subunit alpha type-7 [Balaenoptera acutorostrata scammoni]
ENSBTAP00000029070-D1	2.20	0.00E+00	↓	DEP domain-containing protein 7, partial [B. mutus]
ENSBTAP00000011848-D1	2.20	5.92E-07	↓	phosphatidylserine decarboxylase proenzyme [B. mutus]
ENSBTAP00000051325-D1	2.20	2.18E-10	↓	Katanin p80 WD40-containing subunit B1, partial [B. mutus]
ENSP00000378784-D1	2.20	1.09E-43	↓	Tudor domain-containing protein PHF20L1 [B. mutus]
ENSP00000265997-D1	2.20	1.39E-20	↓	cytoplasmic polyadenylation element-binding protein 3 [B. mutus]
ENSP00000384996-D1	2.21	5.32E-06	↓	TBC1 domain family member 10A [B. mutus]
ENSBTAP00000034364-D5	2.21	0.00E+00	↓	ubiquitin [Acipenser persicus]
ENSP00000358042-D1	2.21	3.03E-48	↓	glutamyl-tRNA(Gln) amidotransferase subunit A, mitochondrial [B. mutus]
ENSBTAP00000053323-D1	2.21	1.74E-247	↓	Dyslexia susceptibility 1 candidate 1 protein-like protein, partial [B. mutus]
ENSBTAP00000020872-D2	2.21	5.56E-11	↓	cysteine-rich PDZ-binding protein [Homo sapiens]
yakG005737	2.21	1.13E-26	↓	hypothetical protein M91_08923 [B. mutus]
ENSP00000262059-D1	2.22	5.59E-15	↓	calcium-binding and coiled-coil domain-containing protein 1 isoform X1 [B. mutus]
ENSP00000286428-D1	2.22	0.00E+00	↓	prefoldin subunit 3 [Ovis aries]
ENSP00000420641-D1	2.22	0.00E+00	↓	hypothetical protein M91_11447 [B. mutus]
ENSBTAP00000051629-D1	2.23	1.28E-18	↓	homer protein homolog 1 [B. taurus]
ENSBTAP00000048343-D1	2.23	1.32E-06	↓	AP-2 complex subunit alpha-2, partial [B. mutus]
ENSP00000379999-D1	2.23	4.52E-71	↓	mitochondrial import inner membrane translocase subunit Tim17-B isoform X2 [Ovis aries]
ENSP00000274289-D1	2.23	8.89E-24	↓	Serine/threonine-protein kinase PLK2, partial [B. mutus]
ENSBTAP00000010035-D1	2.24	4.28E-126	↓	THAP domain-containing protein 1 [B. taurus]
ENSBTAP00000017525-D1	2.24	0.00E+00	↓	replication protein A 14 kDa subunit [B. mutus]
ENSP00000359018-D1	2.24	5.48E-73	↓	protein AKNAD1 [Bubalus bubalis]
ENSBTAP00000029148-D1	2.24	4.83E-04	↓	mediator of RNA polymerase II transcription subunit 31 isoform X1 [Papio anubis]
ENSBTAP00000025666-D1	2.24	0.00E+00	↓	SOSS complex subunit C [Dasypus novemcinctus]
ENSBTAP00000036829-D1	2.25	0.00E+00	↓	zona pellucida sperm-binding protein 4 [B. mutus]
ENSP00000328118-D1	2.25	1.30E-24	↓	probable guanine nucleotide exchange factor MCF2L2 isoform X5 [Bison bison bison]
ENSBTAP00000031685-D1	2.25	2.73E-31	↓	leukocyte receptor cluster member 1 homolog [B. mutus]
ENSP00000260047-D1	2.25	7.04E-32	↓	ankyrin repeat domain-containing protein 42 [B. mutus]
ENSBTAP00000042506-D1	2.25	3.95E-268	↓	39S ribosomal protein L48, mitochondrial, partial [B. mutus]
ENSBTAP00000051256-D1	2.25	6.31E-158	↓	histone H1.3-like [B. mutus]
ENSBTAP00000021717-D1	2.25	5.64E-74	↓	UPF0690 protein C1orf52 homolog [B. taurus]
ENSP00000379366-D1	2.25	1.04E-79	↓	MOB kinase activator 1B [Dipodomys ordii]
ENSP00000358770-D1	2.25	2.17E-46	↓	STE20-like serine/threonine-protein kinase isoform X1 [B. mutus]
ENSBTAP00000048457-D1	2.25	4.31E-132	↓	General transcription factor IIF subunit 1, partial [B. mutus]
ENSP00000303709-D1	2.26	1.54E-14	↓	ubiquitin-conjugating enzyme E2 E1 [B. taurus]
ENSP00000222803-D1	2.26	1.55E-31	↓	peptidyl-prolyl cis-trans isomerase FKBP14 precursor [B. taurus]
ENSP00000414273-D1	2.26	0.00E+00	↓	hypothetical protein M91_17887, partial [B. mutus]
yakG033476	2.26	1.76E-11	↓	Microtubule-associated proteins 1A/1B light chain 3B [B. mutus]
ENSP00000395986-D1	2.26	1.34E-43	↓	TBC1 domain family member 4, partial [B. mutus]
ENSP00000417841-D1	2.26	6.07E-10	↓	EF-hand calcium-binding domain-containing protein 10 [B. mutus]
ENSP00000380177-D1	2.26	6.07E-10	↓	histone H2B type 1-like [Propithecus coquereli]
ENSBTAP00000026288-D1	2.26	2.97E-06	↓	ankyrin repeat and EF-hand domain-containing protein 1 [B. mutus]
ENSBTAP00000016882-D3	2.26	2.97E-06	↓	Putative palmitoyltransferase ZDHHC16 [B. mutus]
ENSP00000285871-D1	2.26	9.44E-246	↓	coiled-coil domain-containing protein 146 isoform X1 [Bubalus bubalis]
ENSBTAP00000027471-D1	2.27	2.09E-08	↓	protein kinase C epsilon type, partial [B. mutus]

yakG027556	2.27	1.79E-62	↓	hypothetical protein M91_17749 [B. mutus]
ENSP00000161863-D1	2.27	7.66E-37	↓	probable ATP-dependent RNA helicase YTHDC2 [B. taurus]
ENSP00000281961-D1	2.27	5.03E-60	↓	transmembrane protein 178A [B. taurus]
ENSP00000318775-D1	2.27	7.33E-46	↓	anaphase-promoting complex subunit 4 [B. taurus]
ENSBTAP00000017811-D1	2.28	0.00E+00	↓	threonylcarbamoyladenosine tRNA methylthiotransferase [B. taurus]
ENSP00000295101-D1	2.28	1.14E-04	↓	G protein-activated inward rectifier potassium channel 1 [Bison bison bison]
ENSP00000342169-D1	2.28	0.00E+00	↓	Testis-expressed sequence 9 protein, partial [B. mutus]
ENSP00000331381-D1	2.28	7.49E-14	↓	ankyrin repeat and sterile alpha motif domain-containing protein 1B isoform X1 [Bison bison bison]
ENSBTAP00000052877-D3	2.29	1.91E-14	↓	GATS-like protein 2 [B. taurus]
ENSP00000378306-D1	2.29	3.41E-10	↓	Serine/threonine-protein phosphatase 2B catalytic subunit beta isoform, partial [B. mutus]
ENSBTAP00000026055-D1	2.29	9.10E-39	↓	A-kinase anchor protein 17B, partial [B. mutus]
yakG041221	2.30	8.63E-11	↓	hypothetical protein M91_11602 [B. mutus]
ENSP00000311493-D1	2.30	0.00E+00	↓	TFIIA-alpha and beta-like factor-like isoform 1 [Ceratotherium simum simum]
ENSBTAP00000022041-D1	2.30	4.24E-32	↓	unnamed protein product [Mus musculus]
ENSP00000355966-D1	2.30	3.92E-175	↓	serine/threonine-protein kinase Nek2 isoform X1 [B. taurus]
ENSBTAP00000004850-D1	2.30	6.95E-280	↓	Bromodomain testis-specific protein [B. mutus]
ENSP00000380911-D1	2.31	1.10E-305	↓	centrosomal protein of 83 kDa [B. taurus]
ENSP00000297423-D1	2.31	5.10E-93	↓	Putative protein KIAA0146, partial [B. mutus]
yakG043453	2.31	6.68E-06	↓	Hydroxyindole O-methyltransferase [B. mutus]
ENSP00000299300-D1	2.31	0.00E+00	↓	T-complex protein 1 subunit beta, partial [B. mutus]
ENSBTAP00000011723-D1	2.32	2.15E-51	↓	Factor in the germline alpha, partial [B. mutus]
ENSBTAP00000018789-D1	2.32	4.28E-153	↓	Myotubularin, partial [B. mutus]
ENSBTAP00000012770-D1	2.32	1.76E-165	↓	serine/threonine-protein phosphatase 2A regulatory subunit B'' subunit gamma [B. taurus]
ENSBTAP00000041982-D1	2.32	4.82E-11	↓	DNA-binding protein SMUBP-2 [B. mutus]
ENSP00000216252-D1	2.32	1.17E-45	↓	PHD finger-like domain-containing protein 5A-like [Pundamilia nyererei]
yakA01168	2.32	5.29E-93	↓	hypothetical protein M91_21448 [B. mutus]
ENSP00000377626-D2	2.32	5.69E-29	↓	sodium/bile acid cotransporter 7 [B. taurus]
ENSBTAP00000004003-D1	2.32	1.30E-20	↓	5'-nucleotidase domain-containing protein 1 [B. mutus]
ENSP00000389792-D1	2.32	3.94E-16	↓	Doublecortin domain-containing protein 1, partial [B. mutus]
ENSP00000368237-D1	2.32	0.00E+00	↓	atlastin-2 [B. taurus]
ENSP00000359719-D1	2.33	1.44E-137	↓	cAMP-dependent protein kinase catalytic subunit beta isoform X1 [B. taurus]
ENSP00000395302-D1	2.33	0.00E+00	↓	Cytoskeleton-associated protein 5 [B. mutus]
yakG020207	2.34	7.28E-21	↓	Type II iodothyronine deiodinase [B. mutus]
ENSBTAP00000027753-D1	2.34	1.89E-135	↓	Trans-Golgi network integral membrane protein 2, partial [B. mutus]
ENSBTAP00000002453-D1	2.34	5.29E-234	↓	Cooperator of PRMT5, partial [B. mutus]
ENSP00000312066-D1	2.34	8.09E-24	↓	signal recognition particle subunit SRP68 [B. taurus]
ENSP00000317786-D1	2.34	5.63E-17	↓	Myosin phosphatase Rho-interacting protein, partial [B. mutus]
ENSBTAP00000007228-D1	2.34	4.41E-86	↓	squalene monooxygenase [B. taurus]
ENSP00000413606-D1	2.34	1.06E-10	↓	Syntaxin-binding protein 2, partial [B. mutus]
ENSP00000235329-D1	2.35	4.36E-32	↓	Mitofusin-2 [B. mutus]
yakG037561	2.35	4.19E-127	↓	Splicing factor 3B subunit 3 [B. mutus]
ENSP00000386563-D1	2.35	1.16E-24	↓	MORN repeat-containing protein 2 isoform X1 [Physeter catodon]
ENSP00000412652-D1	2.35	7.34E-89	↓	Netrin receptor UNC5D, partial [B. mutus]
ENSBTAP00000053003-D4	2.36	0.00E+00	↓	polyubiquitin-B-like [B. mutus]
ENSP00000409016-D1	2.36	8.92E-89	↓	transforming growth factor beta regulator 1 [B. mutus]
ENSP00000292144-D1	2.37	1.17E-85	↓	T-cell surface glycoprotein CD3 gamma chain [B. mutus]
ENSBTAP00000004304-D1	2.37	1.50E-11	↓	tubulin polyglutamylase TTLL7 [B. mutus]
ENSBTAP00000051517-D2	2.37	9.11E-07	↓	multifunctional protein ADE2 isoform X1 [Bubalus bubalis]
ENSBTAP00000032032-D1	2.37	9.11E-07	↓	Mitogen-activated protein kinase-binding protein 1 [B. mutus]
ENSBTAP00000004413-D1	2.37	9.11E-07	↓	Quinone oxidoreductase PIG3, partial [B. mutus]
ENSBTAP00000001972-D1	2.37	6.19E-05	↓	Huntingtin, partial [B. mutus]
ENSBTAP00000001080-D1	2.37	6.19E-05	↓	ephrin type-A receptor 2 [B. mutus]
ENSP00000418458-D1	2.37	6.19E-05	↓	hypothetical protein M91_11903 [B. mutus]
ENSBTAP00000020243-D1	2.37	2.61E-04	↓	carbonic anhydrase 3 [B. mutus]
ENSP00000260570-D1	2.37	2.61E-04	↓	intraflagellar transport protein 172 homolog [B. mutus]

ENSP00000376260-D1	2.37	1.14E-03	↓	Transmembrane channel-like protein 6 [B. mutus]
ENSBTAP00000041788-D1	2.37	1.14E-03	↓	LIM domain kinase 2, partial [B. mutus]
ENSBTAP00000012415-D1	2.37	1.14E-03	↓	protein SMG5 [B. mutus]
ENSP00000254488-D1	2.37	5.45E-03	↓	sodium- and chloride-dependent GABA transporter 3 [B. taurus]
ENSBTAP00000004354-D1	2.37	5.45E-03	↓	Sodium-dependent noradrenaline transporter, partial [B. mutus]
ENSP00000392188-D1	2.37	5.45E-03	↓	hypothetical protein M91_08556, partial [B. mutus]
ENSBTAP00000027975-D1	2.37	5.45E-03	↓	Tripartite motif-containing protein 55 [B. mutus]
ENSBTAP00000005415-D1	2.37	5.45E-03	↓	glioma tumor suppressor candidate region gene 1 protein [B. mutus]
ENSBTAP00000037521-D1	2.37	5.45E-03	↓	85/88 kDa calcium-independent phospholipase A2 [B. mutus]
ENSP00000401466-D1	2.37	5.45E-03	↓	Tripartite motif-containing protein 66, partial [B. mutus]
ENSBTAP00000040758-D1	2.37	5.45E-03	↓	Pro-neuregulin-2, membrane-bound isoform [Pteropus alecto]
ENSP00000411998-D19	2.37	5.45E-03	↓	hypothetical protein N307_11227, partial [Picoides pubescens]
ENSBTAP00000006264-D1	2.37	5.45E-03	↓	retinoic acid-induced protein 1 [B. mutus]
ENSBTAP00000053749-D1	2.37	3.81E-76	↓	Zinc finger MYM-type protein 1 [B. mutus]
ENSP00000308897-D1	2.37	5.52E-240	↓	39S ribosomal protein L11, mitochondrial [B. mutus]
ENSBTAP00000005214-D2	2.38	1.32E-257	↓	60S ribosomal protein L7-like 1 [B. mutus]
ENSP00000370376-D1	2.38	6.91E-253	↓	deoxyuridine 5'-triphosphate nucleotidohydrolase, mitochondrial isoform X1 [B. mutus]
ENSBTAP00000005604-D1	2.38	0.00E+00	↓	sushi repeat-containing protein SRPX isoform X1 [Bubalus bubalis]
ENSBTAP00000036460-D1	2.38	5.36E-101	↓	Thrombospondin-4 [B. mutus]
ENSP00000364005-D1	2.38	8.67E-105	↓	lipid phosphate phosphatase-related protein type 1 [B. taurus]
yakG020864	2.39	1.49E-16	↓	Protein piccolo [B. mutus]
ENSBTAP00000004334-D1	2.39	0.00E+00	↓	HD domain-containing protein 2, partial [B. mutus]
ENSP00000385823-D1	2.39	0.00E+00	↓	Metastasis-associated protein MTA3, partial [B. mutus]
ENSBTAP00000027028-D1	2.39	5.89E-58	↓	Deoxycytidylate deaminase, partial [B. mutus]
ENSP00000377700-D1	2.39	1.91E-103	↓	TBC1 domain family member 23, partial [B. mutus]
ENSP00000399362-D1	2.40	7.91E-189	↓	Protein farnesyltransferase subunit beta [B. mutus]
ENSP00000305151-D1	2.40	2.16E-98	↓	PWWP domain-containing protein 2A [B. mutus]
ENSP00000358739-D1	2.40	2.63E-179	↓	RWD domain-containing protein 2A isoform X1 [B. mutus]
ENSBTAP00000038178-D1	2.40	5.07E-10	↓	dynein heavy chain 7, axonemal [B. taurus]
ENSBTAP00000010783-D1	2.40	2.00E-09	↓	Ral guanine nucleotide dissociation stimulator-like 3, partial [B. mutus]
ENSP00000401494-D1	2.40	2.00E-09	↓	autophagy-related protein 101 [B. taurus]
ENSP00000298281-D1	2.40	4.16E-102	↓	pre-mRNA cleavage complex 2 protein Pcf11 [B. mutus]
ENSBTAP00000022009-D1	2.41	5.42E-76	↓	Testis-expressed sequence 12 protein [B. mutus]
ENSBTAP00000027435-D1	2.41	6.37E-62	↓	Vesicle-associated membrane protein 4 [B. mutus]
ENSBTAP00000046338-D25	2.41	5.77E-35	↓	hypothetical protein M91_10439, partial [B. mutus]
ENSP00000314608-D1	2.41	8.33E-29	↓	kelch-like protein 11 [B. mutus]
ENSP00000384304-D1	2.41	2.24E-204	↓	Nuclear nucleic acid-binding protein C1D, partial [B. mutus]
ENSP00000298428-D2	2.41	3.11E-23	↓	protein transport protein Sec61 subunit alpha isoform 1-like protein [Cricetulus griseus]
ENSBTAP00000034654-D1	2.41	1.24E-07	↓	Intercellular adhesion molecule 5, partial [B. mutus]
ENSBTAP00000052233-D7	2.42	1.90E-40	↓	hypothetical protein M91_08630, partial [B. mutus]
yakG008256	2.43	1.22E-57	↓	hypothetical protein M91_12305 [B. mutus]
ENSP00000395147-D1	2.43	4.18E-171	↓	Ribonuclease P protein subunit p30, partial [B. mutus]
ENSBTAP00000018461-D1	2.43	6.06E-20	↓	Phosphatidylserine synthase 1, partial [B. mutus]
ENSP00000339823-D7	2.44	4.21E-141	↓	zinc finger protein 184 [B. mutus]
ENSBTAP00000048387-D1	2.44	2.53E-12	↓	Glycosyltransferase 8 domain-containing protein 2, partial [B. mutus]
ENSBTAP00000004132-D1	2.44	4.10E-196	↓	Arginyl-tRNA--protein transferase 1, partial [B. mutus]
ENSP00000349145-D1	2.44	1.35E-125	↓	Myosin-XVI [B. mutus]
ENSP00000256858-D1	2.45	3.34E-20	↓	LisH domain and HEAT repeat-containing protein KIAA1468 [B. mutus]
ENSP00000377655-D1	2.45	1.94E-89	↓	Sentrin-specific protease 7, partial [B. mutus]
ENSBTAP00000043974-D1	2.45	1.68E-36	↓	tyrosine-protein kinase Tec isoform X2 [Bison bison bison]
ENSBTAP00000044615-D1	2.45	2.14E-16	↓	Intraflagellar transport protein 140-like protein [B. mutus]
ENSBTAP00000002597-D2	2.45	9.46E-164	↓	uncharacterized protein C12orf31 homolog [B. mutus]
ENSBTAP00000052592-D54	2.46	3.35E-05	↓	hypothetical protein M91_06787, partial [B. mutus]
ENSBTAP00000024161-D3	2.46	4.41E-93	↓	28S ribosomal protein S18c, mitochondrial [B. taurus]
ENSP00000378837-D2	2.46	4.08E-289	↓	sorting nexin-24 [Bubalus bubalis]

ENSBTAP00000018838-D1	2.46	1.90E-45	↓	Galanin-like peptide, partial [B. mutus]
ENSP00000217446-D1	2.47	0.00E+00	↓	Phosphatidylinositol glycan anchor biosynthesis class U protein [B. mutus]
ENSBTAP00000028683-D1	2.47	2.44E-23	↓	bifunctional apoptosis regulator [B. mutus]
ENSBTAP00000033017-D1	2.47	6.97E-182	↓	Mitochondrial ribonuclease P protein 1 [B. mutus]
ENSBTAP00000039023-D2	2.47	3.08E-235	↓	Oocyte-secreted protein 1 [B. mutus]
ENSBTAP00000027878-D1	2.48	0.00E+00	↓	Survival of motor neuron protein-interacting protein 1, partial [B. mutus]
ENSBTAP00000053769-D1	2.48	1.95E-13	↓	arf-GAP with GTPase, ANK repeat and PH domain-containing protein 1 isoform X1 [B. taurus]
ENSBTAP00000016395-D1	2.48	1.95E-13	↓	E3 ubiquitin-protein ligase RNF220, partial [B. mutus]
ENSBTAP00000022381-D1	2.48	1.79E-15	↓	WD repeat-containing protein 46 isoform X1 [B. mutus]
ENSBTAP00000022762-D1	2.48	0.00E+00	↓	G-protein-signaling modulator 2, partial [B. mutus]
ENSBTAP00000015059-D1	2.49	2.35E-49	↓	5'-nucleotidase, partial [B. mutus]
ENSBTAP00000016073-D1	2.49	1.10E-06	↓	V-type proton ATPase subunit S1 [B. mutus]
ENSP00000360803-D1	2.49	1.40E-04	↓	DNL-type zinc finger protein, partial [B. mutus]
ENSP00000419475-D1	2.49	0.00E+00	↓	COMM domain-containing protein 2 [B. mutus]
ENSP00000344393-D2	2.49	5.40E-205	↓	rRNA-processing protein FCF1 homolog [Galeopterus variegatus]
ENSBTAP00000045022-D1	2.50	8.08E-33	↓	BRCA1-associated RING domain protein 1, partial [B. mutus]
ENSBTAP00000011800-D1	2.50	9.17E-29	↓	D-tyrosyl-tRNA(Tyr) deacylase 1 [B. taurus]
ENSBTAP00000009684-D1	2.50	1.40E-92	↓	U6 snRNA-associated Sm-like protein LSm3 [Clupea harengus]
ENSP00000264424-D1	2.50	4.37E-22	↓	Guanylate cyclase soluble subunit beta-1, partial [B. mutus]
ENSP00000380338-D1	2.51	5.02E-29	↓	protein MANBAL [B. taurus]
ENSBTAP00000016475-D1	2.51	4.19E-13	↓	Zinc finger protein 511, partial [B. mutus]
ENSBTAP00000051625-D1	2.51	1.45E-134	↓	hypothetical protein M91_15965 [B. mutus]
ENSBTAP00000006382-D1	2.51	5.78E-25	↓	sodium/potassium/calcium exchanger 5 [B. mutus]
yakG038243	2.51	4.62E-11	↓	lactoylglutathione lyase-like [B. mutus]
ENSP00000376703-D2	2.51	1.69E-70	↓	anoctamin-4 isoform X1 [Ovis aries]
ENSBTAP00000002705-D1	2.52	1.64E-12	↓	Menin, partial [B. mutus]
ENSBTAP00000011202-D1	2.52	4.57E-56	↓	THO complex subunit 4, partial [B. mutus]
ENSBTAP00000015401-D1	2.52	0.00E+00	↓	KRR1 small subunit processome component homolog [B. mutus]
ENSBTAP00000050644-D2	2.52	1.87E-72	↓	GTP-binding protein Rheb, partial [Heterocephalus glaber]
ENSBTAP00000053408-D4	2.52	4.18E-28	↓	myosin-2 [B. mutus]
ENSBTAP00000042334-D2	2.52	9.33E-22	↓	transcription initiation factor TFIID subunit 13 [Cariama cristata]
ENSBTAP00000050471-D1	2.52	0.00E+00	↓	Putative TRAF4-associated factor 1 [B. mutus]
ENSP00000273666-D2	2.52	9.23E-39	↓	Syntaxin-binding protein 5 [B. mutus]
ENSP00000385776-D2	2.52	1.24E-41	↓	Casein kinase I isoform alpha, partial [B. mutus]
ENSBTAP00000001319-D1	2.52	2.75E-18	↓	Protein FAM46A [Myotis brandtii]
ENSBTAP00000027728-D1	2.52	0.00E+00	↓	Ubiquitin-like protein FUBI, partial [B. mutus]
ENSBTAP00000041886-D1	2.53	0.00E+00	↓	hypothetical protein M91_18198 [B. mutus]
ENSP00000227758-D2	2.53	1.96E-38	↓	Baculoviral IAP repeat-containing protein 3, partial [B. mutus]
ENSBTAP00000015797-D1	2.53	2.63E-41	↓	activin receptor type-1 [B. mutus]
yakG044243	2.54	4.13E-226	↓	Importin subunit alpha-6 [B. mutus]
ENSP00000358925-D1	2.54	7.11E-10	↓	Proline/serine-rich coiled-coil protein 1 [B. mutus]
ENSBTAP00000053609-D1	2.54	7.11E-10	↓	nuclear receptor coactivator 7 isoform X1 [B. mutus]
ENSBTAP00000050301-D1	2.54	5.95E-04	↓	Hermansky-Pudlak syndrome 4 protein [B. mutus]
ENSBTAP00000053809-D1	2.54	5.95E-04	↓	disintegrin and metalloproteinase domain-containing protein 7 isoform X2 [Ovis aries]
ENSBTAP00000012047-D1	2.54	0.00E+00	↓	mRNA cap guanine-N7 methyltransferase isoform X1 [B. mutus]
ENSBTAP00000002064-D1	2.55	0.00E+00	↓	G patch domain-containing protein 2 [B. taurus]
ENSBTAP00000010668-D1	2.55	0.00E+00	↓	intraflagellar transport protein 20 homolog [B. mutus]
ENSBTAP00000022459-D1	2.55	3.50E-12	↓	leucine-rich repeat transmembrane neuronal protein 3-like isoform X2 [B. mutus]
ENSBTAP00000008363-D1	2.56	0.00E+00	↓	H/ACA ribonucleoprotein complex subunit 2, partial [B. mutus]
ENSBTAP00000030490-D1	2.56	3.75E-29	↓	DNA-directed RNA polymerases I, II, and III subunit RPABC4 [Fukomys damarensis]
ENSBTAP00000020547-D1	2.56	3.06E-92	↓	TELO2-interacting protein 2 [B. mutus]
ENSBTAP00000026237-D1	2.56	4.20E-21	↓	Ly6/PLAUR domain-containing protein 6B, partial [B. mutus]
ENSBTAP00000003058-D1	2.56	5.51E-143	↓	probable ATP-dependent RNA helicase DDX47 isoform X1 [B. mutus]
ENSP00000300026-D1	2.56	1.82E-205	↓	peptidyl-prolyl cis-trans isomerase B [B. mutus]
ENSBTAP00000043233-D1	2.56	8.02E-08	↓	major prion protein precursor [B. taurus]

ENSBTAP00000022460-D1	2.56	1.08E-70	↓	.
ENSP00000225726-D1	2.56	3.11E-157	↓	coiled-coil domain-containing protein 47 [B. mutus]
ENSBTAP00000007889-D1	2.57	1.10E-25	↓	MPN domain-containing protein isoform X1 [B. taurus]
ENSBTAP00000010311-D1	2.57	0.00E+00	↓	Inorganic pyrophosphatase, partial [B. mutus]
ENSBTAP00000028046-D1	2.57	6.22E-69	↓	ATP-dependent RNA helicase DDX42 [B. mutus]
ENSBTAP00000005354-D1	2.57	7.32E-47	↓	UPF0364 protein C6orf211 homolog [B. mutus]
ENSBTAP00000044174-D1	2.57	3.44E-16	↓	Cytotoxic and regulatory T-cell molecule, partial [B. mutus]
ENSP00000398064-D7	2.58	0.00E+00	↓	HIG1 domain family member 1A, partial [B. mutus]
ENSBTAP00000028197-D1	2.58	2.40E-222	↓	TBC1 domain family member 31 isoform X1 [B. mutus]
ENSBTAP00000013463-D1	2.58	4.64E-301	↓	Activating transcription factor 7-interacting protein 2 [B. mutus]
ENSP00000295902-D2	2.58	6.40E-105	↓	prickle-like protein 1 isoform X1 [B. mutus]
ENSBTAP00000009529-D1	2.58	7.88E-39	↓	Coiled-coil domain-containing protein 147, partial [B. mutus]
ENSP00000285518-D1	2.58	2.38E-116	↓	1-acyl-sn-glycerol-3-phosphate acyltransferase epsilon, partial [B. mutus]
ENSP00000257627-D1	2.58	5.88E-36	↓	oncomodulin-like [B. mutus]
ENSP00000360541-D1	2.58	2.45E-19	↓	carnitine O-palmitoyltransferase 2, mitochondrial isoform X1 [B. mutus]
ENSBTAP00000053428-D1	2.58	1.25E-21	↓	Short transient receptor putative channel 6, partial [B. mutus]
ENSBTAP00000020159-D1	2.58	7.68E-77	↓	induced myeloid leukemia cell differentiation protein Mcl-1 homolog [B. mutus]
ENSBTAP00000018626-D1	2.59	0.00E+00	↓	proteasome maturation protein [B. taurus]
yakA22750	2.59	7.44E-12	↓	hypothetical protein M91_07326 [B. mutus]
ENSP00000263301-D1	2.59	7.32E-05	↓	mth938 domain-containing protein [B. mutus]
ENSP00000383042-D1	2.59	8.81E-77	↓	lysine-specific histone demethylase 1A isoform X2 [Chrysochloris asiatica]
ENSP00000348828-D2	2.60	3.43E-60	↓	rho guanine nucleotide exchange factor 26 [Bison bison bison]
ENSBTAP00000050581-D2	2.60	0.00E+00	↓	Ribose-phosphate pyrophosphokinase 1, partial [B. mutus]
ENSP00000258302-D1	2.60	1.29E-29	↓	Regulator of G-protein signaling 8, partial [B. mutus]
ENSBTAP00000052925-D1	2.60	5.18E-15	↓	BH3-interacting domain death agonist, partial [B. mutus]
ENSBTAP00000048527-D1	2.60	0.00E+00	↓	WD repeat-containing protein 41 [B. mutus]
ENSBTAP00000052602-D85	2.60	2.10E-10	↓	hypothetical protein M91_13633, partial [B. mutus]
ENSBTAP00000019111-D1	2.61	0.00E+00	↓	centrin-3 [Ovis aries]
ENSP00000297273-D1	2.61	4.80E-110	↓	CAS1 domain-containing protein 1 isoform X2 [Bison bison bison]
ENSBTAP00000046387-D4	2.61	3.21E-222	↓	hypothetical protein M91_21441, partial [B. mutus]
ENSBTAP00000027817-D1	2.61	1.16E-135	↓	hypothetical protein M91_12086, partial [B. mutus]
ENSP00000352833-D1	2.62	1.45E-25	↓	tyrosine-protein phosphatase non-receptor type 22 [B. taurus]
ENSP00000336655-D1	2.62	7.70E-36	↓	STE20-related kinase adapter protein alpha isoform X2 [B. taurus]
ENSBTAP00000015548-D1	2.62	2.04E-26	↓	Inositol monophosphatase 1, partial [B. mutus]
ENSP00000343676-D1	2.63	1.02E-171	↓	gap junction alpha-4 protein [B. mutus]
ENSBTAP00000049146-D1	2.63	2.45E-54	↓	uncharacterized protein LOC101404271 [Ceratotherium simum simum]
ENSP00000269214-D1	2.63	6.71E-214	↓	N-acetyltransferase ESCO1 [B. mutus]
ENSBTAP00000025568-D1	2.63	2.55E-58	↓	cell cycle checkpoint protein RAD1 [B. mutus]
ENSP00000216484-D1	2.63	4.18E-36	↓	Serine palmitoyltransferase 2, partial [B. mutus]
ENSP00000353942-D1	2.63	1.10E-122	↓	coiled-coil domain-containing protein lobo homolog [Bison bison bison]
ENSBTAP00000027497-D1	2.63	4.64E-91	↓	RNA-binding protein NOB1 [B. mutus]
ENSBTAP00000025259-D1	2.63	1.13E-10	↓	Cysteine-rich protein 2-binding protein [B. mutus]
ENSBTAP00000002055-D4	2.63	0.00E+00	↓	Chain A, Crystal Structure Of Calmodulin Complexed With A Peptide
ENSBTAP00000052857-D1	2.64	8.30E-71	↓	zinc finger protein 616-like isoform X1 [Bison bison bison]
ENSBTAP00000010639-D1	2.64	1.52E-15	↓	AMMECR1-like protein [B. taurus]
ENSP00000327072-D1	2.64	0.00E+00	↓	TATA box-binding protein-associated factor RNA polymerase I subunit A [B. mutus]
ENSBTAP00000002557-D1	2.64	2.14E-16	↓	Coiled-coil domain-containing protein 39 [B. mutus]
ENSBTAP00000001440-D1	2.64	1.72E-07	↓	Four and a half LIM domains protein 2, partial [B. mutus]
ENSBTAP00000006115-D1	2.65	2.36E-197	↓	coenzyme Q-binding protein COQ10 homolog B, mitochondrial [B. mutus]
ENSBTAP00000042771-D1	2.65	2.34E-08	↓	E3 ubiquitin-protein ligase RNF123 [B. mutus]
ENSBTAP00000024562-D1	2.65	8.31E-82	↓	ufm1-specific protease 2 [B. mutus]
ENSBTAP00000053542-D1	2.66	1.30E-61	↓	Putative protein KIAA1797 [B. mutus]
ENSP00000356581-D1	2.66	3.20E-09	↓	G-protein coupled receptor 126 isoform X1 [B. mutus]
ENSBTAP00000017420-D1	2.66	3.20E-09	↓	Collagen alpha-1(I) chain [B. mutus]
ENSP00000408309-D1	2.66	1.27E-30	↓	protein lin-54 homolog isoform X2 [B. taurus]

ENSBTAP00000012033-D1	2.66	4.42E-10	↓	Pituitary-specific positive transcription factor 1 [B. mutus]
ENSP00000321874-D1	2.66	1.25E-26	↓	UDP-GlcNAc:betaGal beta-1,3-N-acetylglucosaminyltransferase 3 [B. mutus]
ENSBTAP00000006593-D1	2.66	5.24E-86	↓	uncharacterized protein LOC102972240 [Panthera tigris altaica]
ENSBTAP00000012029-D1	2.67	2.75E-82	↓	Cohesin subunit SA-2, partial [B. mutus]
ENSP00000381968-D1	2.67	6.23E-17	↓	Serine palmitoyltransferase 3, partial [B. mutus]
ENSP00000382204-D1	2.68	0.00E+00	↓	Putative JmjC domain-containing histone demethylation protein 2C [B. mutus]
ENSP00000324173-D1	2.68	0.00E+00	↓	78 kDa glucose-regulated protein [Ovis aries]
ENSBTAP00000003668-D1	2.68	1.12E-56	↓	Mothers against decapentaplegic-like protein 1, partial [B. mutus]
ENSBTAP00000010106-D1	2.68	2.01E-31	↓	Thrombospondin type-1 domain-containing protein 7A, partial [B. mutus]
ENSBTAP00000030232-D1	2.69	8.74E-41	↓	Histone H4 [Heterocephalus glaber]
ENSBTAP00000010696-D1	2.69	1.48E-87	↓	SRA stem-loop-interacting RNA-binding protein, mitochondrial [B. taurus]
ENSP00000202816-D1	2.69	0.00E+00	↓	ESF1 homolog [B. mutus]
ENSBTAP00000012443-D1	2.69	1.53E-32	↓	methyltransferase-like protein 2-like [B. mutus]
ENSBTAP00000022416-D1	2.69	6.62E-19	↓	smad nuclear-interacting protein 1 [B. mutus]
ENSBTAP00000019517-D1	2.69	8.83E-14	↓	Protein SIX6OS1 [B. mutus]
ENSP00000401258-D1	2.69	3.86E-05	↓	Beta-soluble NSF attachment protein [B. mutus]
ENSBTAP00000031460-D1	2.69	3.86E-05	↓	Collagen alpha-1(XXII) chain, partial [B. mutus]
ENSBTAP00000007890-D1	2.69	3.86E-05	↓	endophilin-A2 isoform X2 [B. mutus]
ENSBTAP00000052619-D23	2.69	3.05E-04	↓	hypothetical protein M91_04067, partial [B. mutus]
ENSP00000332444-D1	2.69	3.05E-04	↓	cleavage stimulation factor, 3' pre-RNA, subunit 2, 64kDa, tau variant [B. mutus]
ENSBTAP00000050520-D73	2.69	3.05E-04	↓	hypothetical protein M91_07727, partial [B. mutus]
ENSP00000407238-D1	2.69	3.05E-04	↓	centrosomal protein of 170 kDa protein B [B. mutus]
yakG010089	2.69	2.66E-03	↓	hypothetical protein M91_11573 [B. mutus]
ENSBTAP00000019400-D1	2.69	2.66E-03	↓	collagen alpha-5(IV) chain-like, partial [Tursiops truncatus]
ENSP00000375809-D1	2.69	2.66E-03	↓	TFIIH basal transcription factor complex helicase XPD subunit isoform X1 [Ovis aries]
ENSBTAP00000042304-D2	2.69	2.66E-03	↓	signal transducer CD24 [B. taurus]
ENSP00000372649-D6	2.69	2.66E-03	↓	hypothetical protein M91_14285, partial [B. mutus]
ENSBTAP00000014236-D1	2.69	2.66E-03	↓	thyroid hormone receptor alpha [B. taurus]
ENSBTAP00000002174-D3	2.69	2.66E-03	↓	EH domain-containing protein 2, partial [B. mutus]
ENSBTAP00000049349-D1	2.69	2.66E-03	↓	actin-related protein 2 isoform b [Homo sapiens]
ENSBTAP00000023023-D1	2.69	2.66E-03	↓	DNA polymerase nu [B. mutus]
ENSP00000229758-D1	2.70	0.00E+00	↓	F-box only protein 5 isoform X1 [B. mutus]
ENSBTAP00000016652-D1	2.70	2.41E-54	↓	Protein CASC4 [B. mutus]
ENSP00000400932-D1	2.71	3.16E-32	↓	solute carrier family 35 member G1 isoform X1 [B. taurus]
ENSP00000311697-D1	2.71	1.29E-16	↓	fibroblast growth factor 5 [B. mutus]
ENSBTAP00000004852-D1	2.71	1.29E-16	↓	myotubularin-related protein 9 [B. mutus]
ENSP00000397881-D1	2.71	9.23E-16	↓	protein PET117 homolog, mitochondrial-like [B. mutus]
ENSBTAP00000047700-D1	2.72	2.16E-74	↓	inner nuclear membrane protein Man1 [B. taurus]
ENSBTAP00000036979-D1	2.72	0.00E+00	↓	Protein BUD31-like protein, partial [B. mutus]
ENSBTAP00000044604-D1	2.72	2.45E-12	↓	Charged multivesicular body protein 1a, partial [B. mutus]
ENSP00000413625-D4	2.72	1.76E-11	↓	Cdc42-interacting protein 4 [B. mutus]
ENSBTAP00000015174-D1	2.73	0.00E+00	↓	carbonic anhydrase 9 isoform X3 [B. taurus]
ENSBTAP00000033059-D1	2.73	9.22E-10	↓	cyclin-dependent kinase-like 4 [B. mutus]
ENSBTAP00000016476-D2	2.73	0.00E+00	↓	ARL14 effector protein [B. taurus]
ENSP00000216727-D1	2.73	0.00E+00	↓	Polyadenylate-binding protein 2, partial [B. mutus]
ENSBTAP00000020932-D1	2.73	0.00E+00	↓	spermatogenesis-associated protein 22 isoform X1 [B. mutus]
ENSBTAP00000044543-D1	2.73	8.42E-27	↓	homeobox protein notochord isoform X1 [B. taurus]
ENSBTAP00000025130-D1	2.74	3.04E-149	↓	Zinc finger protein 451, partial [B. mutus]
ENSBTAP00000030505-D3	2.74	4.85E-08	↓	kelch-like protein 7 isoform X4 [Cercocebus atys]
ENSP00000400333-D1	2.74	9.33E-98	↓	sorting nexin-27 [B. mutus]
ENSP00000328547-D1	2.74	5.23E-18	↓	DNA (cytosine-5)-methyltransferase 3B [B. mutus]
ENSBTAP00000004426-D1	2.75	1.63E-24	↓	Protein FAM154B, partial [B. mutus]
ENSBTAP00000005988-D1	2.76	2.79E-120	↓	low-density lipoprotein receptor-related protein 2 [B. mutus]
ENSBTAP00000009775-D1	2.76	1.06E-95	↓	Nucleolar and coiled-body phosphoprotein 1 [B. mutus]
ENSP00000341637-D1	2.76	7.63E-34	↓	Putative Ig-like domain-containing protein DKFZp686O24166/DKFZp686I21167-like protein, partial [B. mutus]

ENSBTAP00000014253-D1	2.76	2.86E-157	↓	hypothetical protein M91_14826, partial [B. mutus]
ENSP00000348132-D1	2.76	5.67E-291	↓	Serine/threonine-protein kinase 31 [B. mutus]
ENSP00000295851-D1	2.76	1.65E-61	↓	abl interactor 2 isoform X4 [B. taurus]
ENSP00000317333-D1	2.76	2.67E-06	↓	neurogenin-2 [B. taurus]
ENSP00000203001-D1	2.76	0.00E+00	↓	tRNA (adenine(58)-N(1))-methyltransferase non-catalytic subunit TRM6 isoform X1 [B. mutus]
ENSBTAP00000046522-D2	2.77	2.43E-84	↓	Serpin B8 [B. mutus]
ENSBTAP00000041953-D1	2.77	3.14E-22	↓	leucine-rich repeat-containing protein 2 [B. mutus]
ENSBTAP00000008388-D1	2.77	0.00E+00	↓	mitochondrial import receptor subunit TOM7 homolog isoform X1 [Capra hircus]
ENSBTAP00000053003-D3	2.78	0.00E+00	↓	ubiquitin [Pan paniscus]
ENSP00000366221-D1	2.78	2.03E-05	↓	zinc finger protein castor homolog 1 isoform X2 [Ovis aries musimon]
ENSP00000337332-D1	2.78	2.03E-05	↓	NAD-dependent protein deacetylase sirtuin-6 isoform X1 [B. mutus]
ENSBTAP00000046795-D1	2.78	2.03E-05	↓	prostaglandin reductase 2 [Ovis aries]
ENSBTAP00000016741-D2	2.78	2.03E-05	↓	Condensin-2 complex subunit H2, partial [B. mutus]
ENSP00000261669-D1	2.79	4.80E-69	↓	calcium-binding protein 39-like isoform X1 [Bison bison bison]
ENSBTAP00000047094-D1	2.79	5.45E-16	↓	hypothetical protein M91_02584, partial [B. mutus]
ENSBTAP00000027177-D1	2.79	3.74E-13	↓	ADP-ribosylation factor-like protein 13A, partial [B. mutus]
ENSBTAP00000009665-D1	2.80	5.09E-38	↓	COMM domain-containing protein 4, partial [B. mutus]
ENSP00000310071-D1	2.80	0.00E+00	↓	anaphase-promoting complex subunit 10 isoform X1 [Colobus angolensis palliatus]
ENSP00000383901-D2	2.80	8.81E-45	↓	Lysosomal acid lipase/cholesteryl ester hydrolase, partial [B. mutus]
ENSBTAP00000014020-D1	2.80	0.00E+00	↓	UPF0538 protein C2orf76 homolog isoform X1 [B. mutus]
ENSP00000315017-D1	2.81	0.00E+00	↓	39S ribosomal protein L1, mitochondrial [B. mutus]
ENSBTAP00000012372-D1	2.81	6.58E-55	↓	Methionine synthase reductase, partial [B. mutus]
ENSBTAP00000001172-D1	2.81	5.02E-138	↓	Katanin p60 ATPase-containing subunit A1 [B. mutus]
ENSBTAP00000001725-D1	2.81	2.91E-16	↓	Frataxin, mitochondrial [B. mutus]
ENSBTAP00000041674-D1	2.81	2.34E-275	↓	Apoptosis-related protein 3, partial [B. mutus]
ENSP00000225805-D1	2.81	2.70E-47	↓	Protein Njmu-R1, partial [B. mutus]
ENSBTAP00000016757-D1	2.81	9.07E-34	↓	norrin [B. mutus]
ENSP00000262265-D1	2.81	1.13E-70	↓	PIH1 domain-containing protein 1 [B. mutus]
ENSBTAP00000012308-D1	2.82	2.41E-123	↓	TPA: ataxin-10 [B. taurus]
ENSBTAP00000002865-D1	2.82	1.99E-13	↓	C-Jun-amino-terminal kinase-interacting protein 3 isoform X13 [B. taurus]
ENSBTAP00000012896-D1	2.82	0.00E+00	↓	nucleoporin NUP53 [Bubalus bubalis]
ENSBTAP00000000099-D1	2.82	2.07E-15	↓	Protein transport protein Sec31B [B. mutus]
ENSP00000342011-D1	2.82	0.00E+00	↓	DNA repair protein XRCC4 isoform X1 [B. mutus]
ENSP00000299518-D1	2.83	3.56E-102	↓	Isocitrate dehydrogenase [NAD] subunit alpha, mitochondrial, partial [B. mutus]
ENSBTAP00000020222-D1	2.83	1.41E-06	↓	Zinc finger protein DZIP1L [B. mutus]
ENSBTAP00000026877-D1	2.83	1.41E-06	↓	Arylsulfatase K, partial [B. mutus]
ENSP00000283243-D1	2.83	1.41E-06	↓	secretory phospholipase A2 receptor [B. mutus]
ENSP00000249499-D1	2.83	1.58E-04	↓	homeobox protein Hox-C9 [Homo sapiens]
ENSBTAP00000014034-D1	2.83	1.58E-04	↓	methyl-CpG-binding domain protein 6 [Bison bison bison]
ENSP00000262126-D1	2.83	1.36E-181	↓	ankyrin repeat domain-containing protein 12 isoform X1 [B. mutus]
ENSBTAP00000040386-D41	2.84	1.57E-38	↓	p97Bcnt protein [Capra hircus]
ENSP00000367405-D1	2.84	1.16E-17	↓	Peripheral plasma membrane protein CASK, partial [B. mutus]
ENSP00000394794-D1	2.84	2.60E-237	↓	Tyrosine-protein phosphatase non-receptor type 13 [B. mutus]
ENSBTAP00000027600-D1	2.84	0.00E+00	↓	centromere protein Q isoform X1 [B. mutus]
ENSP00000356832-D1	2.84	8.28E-48	↓	serine/threonine-protein kinase Sgk1 isoform X1 [B. taurus]
ENSP00000306296-D1	2.85	4.28E-122	↓	PHD finger protein 14 isoform X1 [B. taurus]
ENSP00000269724-D1	2.85	1.01E-09	↓	atherin, partial [B. mutus]
ENSBTAP00000009487-D1	2.86	5.48E-99	↓	RNA polymerase II elongation factor ELL2, partial [B. mutus]
ENSP00000329638-D1	2.86	8.09E-147	↓	zinc finger protein 804B-like [B. mutus]
ENSP00000323046-D1	2.86	5.22E-278	↓	exosome complex component RRP40 [B. mutus]
ENSBTAP00000012993-D1	2.86	2.92E-99	↓	TPA: M-phase phosphoprotein 10 [B. taurus]
ENSP00000281455-D1	2.86	7.39E-11	↓	long-chain-fatty-acid--CoA ligase 1 isoform X1 [Bison bison bison]
ENSP00000283109-D1	2.86	0.00E+00	↓	serine/threonine-protein kinase RIO2 [B. mutus]
ENSP00000317156-D1	2.86	2.53E-263	↓	Centrosomal protein of 192 kDa, partial [B. mutus]
ENSBTAP00000013946-D1	2.86	3.67E-22	↓	centrosomal protein of 76 kDa [B. taurus]

ENSBTAP00000034197-D1	2.86	4.06E-62	↓	Pleckstrin-like protein domain-containing family A member 7, partial [B. mutus]
ENSBTAP00000049353-D1	2.87	3.46E-20	↓	fucose-1-phosphate guanylyltransferase [B. mutus]
ENSP00000398536-D1	2.87	1.39E-32	↓	39S ribosomal protein L3, mitochondrial [B. mutus]
ENSP00000344242-D1	2.87	2.36E-257	↓	leucine-rich repeat-containing protein 17 [B. mutus]
yakG001076	2.87	1.06E-05	↓	hypothetical protein M91_12772 [B. mutus]
ENSBTAP00000001767-D1	2.87	1.83E-305	↓	probable ribosome biogenesis protein RLP24 [B. taurus]
ENSBTAP00000038069-D1	2.88	5.33E-10	↓	serine/threonine-protein kinase 36 [B. mutus]
ENSBTAP00000042130-D1	2.89	4.34E-53	↓	cytoplasmic dynein 1 heavy chain 1 [B. mutus]
ENSBTAP00000025038-D1	2.89	3.55E-133	↓	ran guanine nucleotide release factor [B. mutus]
ENSBTAP00000010673-D1	2.89	7.34E-225	↓	E3 ubiquitin-protein ligase SHPRH [B. mutus]
ENSBTAP00000015224-D1	2.90	3.22E-61	↓	Cytosolic 5'-nucleotidase III-like protein, partial [B. mutus]
ENSP00000262455-D1	2.90	2.41E-297	↓	Endoplasmic reticulum resident protein 44, partial [B. mutus]
ENSP00000294016-D1	2.91	5.25E-08	↓	adenylate cyclase type 9 [B. mutus]
ENSBTAP00000008572-D1	2.91	2.69E-39	↓	bcl-2-like protein 1 [B. taurus]
ENSP00000319831-D1	2.92	2.91E-23	↓	differentially expressed in FDCP 6 homolog isoform X1 [Bubalus bubalis]
ENSP00000359124-D1	2.92	8.75E-110	↓	Diphthine synthase [B. mutus]
ENSP00000361140-D1	2.92	1.43E-39	↓	alcohol dehydrogenase [NADP(+)] isoform X1 [B. mutus]
ENSBTAP00000005625-D2	2.92	6.54E-18	↓	Dihydropyrimidinase-related protein 2 [B. mutus]
ENSBTAP00000039319-D1	2.92	0.00E+00	↓	U2 small nuclear ribonucleoprotein B'', partial [B. mutus]
ENSBTAP00000009988-D1	2.93	6.51E-77	↓	CWF19-like protein 1, partial [B. mutus]
ENSBTAP00000018584-D1	2.94	0.00E+00	↓	TPA: uveal autoantigen with coiled-coil domains and ankyrin repeats protein [B. taurus]
ENSBTAP00000008067-D1	2.95	1.13E-120	↓	programmed cell death 1 ligand 1 [B. mutus]
ENSP00000299714-D1	2.95	6.26E-205	↓	signal peptidase complex catalytic subunit SEC11C [B. mutus]
ENSP00000324444-D1	2.95	4.32E-279	↓	MAP3K12-binding inhibitory protein 1 isoform X1 [B. mutus]
ENSBTAP00000002554-D1	2.95	4.63E-106	↓	tetratricopeptide repeat protein 14 isoform X1 [B. mutus]
ENSP00000253233-D1	2.95	6.96E-132	↓	hypothetical protein M91_19035, partial [B. mutus]
ENSBTAP00000021981-D1	2.95	1.20E-65	↓	Kidney mitochondrial carrier protein 1, partial [B. mutus]
ENSBTAP00000053000-D1	2.95	2.81E-62	↓	mitochondrial inner membrane protease subunit 2 isoform X2 [B. taurus]
ENSBTAP00000012550-D1	2.95	2.02E-09	↓	Proliferation-associated protein 2G4 [Pteropus alecto]
ENSBTAP00000000184-D1	2.95	5.48E-06	↓	cystathionine beta-synthase [B. taurus]
ENSP00000261800-D1	2.95	5.48E-06	↓	protocadherin Fat 2 [B. mutus]
ENSP00000267202-D1	2.95	8.09E-05	↓	Vacuolar protein sorting-associated protein 37B, partial [B. mutus]
ENSP00000299441-D1	2.95	1.29E-03	↓	Protocadherin-16 [B. mutus]
ENSBTAP00000014152-D1	2.95	1.29E-03	↓	Segment polarity protein dishevelled-like protein DVL-1, partial [B. mutus]
ENSBTAP00000029353-D1	2.95	1.29E-03	↓	Tripartite motif-containing protein 65, partial [B. mutus]
ENSBTAP00000013887-D1	2.95	1.29E-03	↓	F-box/WD repeat-containing protein 5 [B. taurus]
ENSBTAP00000023866-D1	2.95	1.29E-03	↓	DNA polymerase delta subunit 4 [B. mutus]
ENSP00000401792-D1	2.95	1.29E-03	↓	branched-chain-amino-acid aminotransferase, cytosolic isoform X1 [Bubalus bubalis]
ENSP00000365899-D1	2.95	1.29E-03	↓	visual system homeobox 1 [B. mutus]
ENSP00000347755-D89	2.95	1.29E-03	↓	hypothetical protein M91_06004, partial [B. mutus]
ENSBTAP00000015586-D1	2.95	1.29E-03	↓	insulin-like growth factor 2 mRNA-binding protein 1 isoform X1 [B. mutus]
ENSP00000416753-D1	2.96	3.91E-191	↓	mucin-15 [B. mutus]
ENSP00000356632-D1	2.96	0.00E+00	↓	costars family protein ABRACL [B. taurus]
ENSBTAP00000018956-D1	2.97	3.04E-23	↓	sterol regulatory element-binding protein 2 [B. mutus]
ENSBTAP00000013696-D1	2.97	6.31E-267	↓	transmembrane protein 184C [B. mutus]
ENSP00000344996-D1	2.98	3.68E-52	↓	Paralemmin-3 [B. mutus]
ENSP00000316840-D1	2.98	4.83E-74	↓	TNF receptor-associated factor 6 [B. mutus]
ENSBTAP00000035168-D2	2.98	2.87E-295	↓	ankyrin repeat domain-containing protein 40-like [B. mutus]
ENSP00000256246-D1	2.98	1.61E-23	↓	testis-expressed sequence 15 protein [B. mutus]
ENSP00000242067-D1	2.98	2.23E-125	↓	protein PTHB1 isoform X1 [B. mutus]
ENSP00000364721-D1	2.99	0.00E+00	↓	microtubule-associated protein RP/EB family member 1 [B. mutus]
ENSBTAP00000022060-D1	2.99	0.00E+00	↓	ribosome production factor 2 homolog isoform X1 [Ovis aries]
ENSP00000418194-D1	2.99	1.67E-71	↓	poly [ADP-ribose] polymerase 14 [B. mutus]
ENSBTAP00000053533-D1	2.99	9.13E-17	↓	gamma-tubulin complex component 3 [B. mutus]
ENSBTAP00000029040-D1	2.99	1.06E-09	↓	membrane progestin receptor alpha isoform X1 [B. mutus]

ENSBTAP00000019734-D1	3.00	1.76E-206	↓	myosin light chain kinase 3 [B. mutus]
ENSBTAP00000021658-D1	3.00	3.04E-39	↓	prolyl endopeptidase [B. mutus]
ENSP00000331720-D2	3.00	1.65E-14	↓	UPF0472 protein C16orf72-like [Physeter catodon]
ENSBTAP00000008193-D1	3.00	1.44E-08	↓	Lysosomal alpha-mannosidase [B. mutus]
ENSP00000358554-D1	3.02	0.00E+00	↓	pre-mRNA-splicing factor SPF27 [Felis catus]
ENSP00000263733-D1	3.02	4.07E-11	↓	glycosaminoglycan xylosylkinase isoform X1 [B. taurus]
ENSBTAP00000021732-D1	3.02	5.90E-39	↓	Signal peptide peptidase-like 3, partial [B. mutus]
ENSBTAP00000030521-D1	3.03	0.00E+00	↓	immunoglobulin M heavy chain secretory form [B. taurus]
ENSP00000412326-D1	3.03	2.93E-113	↓	cAMP-regulated phosphoprotein 21-like isoform X2 [B. mutus]
ENSBTAP00000027116-D1	3.03	2.84E-06	↓	Coiled-coil domain-containing protein 92, partial [B. mutus]
ENSBTAP00000028751-D1	3.04	3.97E-105	↓	zinc finger FYVE domain-containing protein 16 [B. mutus]
ENSP00000260327-D1	3.05	1.14E-167	↓	CTD small phosphatase-like protein 2 [B. taurus]
ENSP00000395886-D1	3.05	0.00E+00	↓	40S ribosomal protein S28-like [B. mutus]
ENSP00000362824-D1	3.05	6.63E-124	↓	UDP-N-acetylglucosamine--peptide N-acetylglucosaminyltransferase 110 kDa subunit, partial [B. mutus]
ENSBTAP00000038799-D1	3.05	1.07E-59	↓	apolipoprotein B-100 [B. mutus]
ENSP00000357844-D1	3.05	2.13E-11	↓	Monocarboxylate transporter 10, partial [B. mutus]
ENSBTAP00000042237-D1	3.06	0.00E+00	↓	Shugoshin-like 2, partial [B. mutus]
ENSBTAP00000012628-D1	3.06	4.10E-300	↓	Neuroguidin, partial [B. mutus]
ENSBTAP00000002494-D1	3.07	3.16E-39	↓	hypothetical protein M91_05378, partial [B. mutus]
ENSBTAP00000011040-D2	3.07	3.04E-53	↓	THO complex subunit 2 [Bison bison bison]
ENSBTAP00000013879-D1	3.07	6.95E-18	↓	cohesin subunit SA-3 [B. taurus]
ENSP00000273261-D1	3.07	2.37E-15	↓	leucine-rich repeats and immunoglobulin-like domains protein 1 [B. mutus]
ENSP00000376400-D1	3.07	1.04E-07	↓	protein S100-A13 isoform X1 [B. taurus]
ENSBTAP00000047102-D1	3.07	1.04E-07	↓	Acyl-CoA dehydrogenase family member 9, mitochondrial, partial [B. mutus]
ENSP00000327889-D1	3.07	4.13E-05	↓	integrator complex subunit 5 [B. mutus]
ENSBTAP00000014649-D1	3.07	4.13E-05	↓	prostaglandin reductase 1 [B. mutus]
ENSP00000331258-D1	3.07	4.13E-05	↓	protein O-mannose kinase-like [B. mutus]
ENSBTAP00000017898-D1	3.07	3.87E-66	↓	Speckle-type POZ protein-like protein, partial [B. mutus]
ENSBTAP00000026815-D2	3.08	0.00E+00	↓	hypothetical protein M91_11000, partial [B. mutus]
ENSBTAP00000021337-D1	3.08	3.20E-14	↓	Sulfotransferase 1C4, partial [B. mutus]
ENSBTAP00000053000-D2	3.09	0.00E+00	↓	hypothetical protein M91_16624, partial [B. mutus]
ENSP00000420213-D1	3.09	1.24E-15	↓	filamin-B isoform X1 [B. mutus]
ENSBTAP00000018496-D1	3.09	7.67E-52	↓	E3 ubiquitin-protein ligase HERC2 [B. mutus]
ENSP00000194530-D1	3.10	9.98E-95	↓	STE20-related kinase adapter protein beta, partial [B. mutus]
yakG012084	3.10	4.87E-17	↓	hypothetical protein M91_12843 [B. mutus]
yakG040301	3.11	1.47E-06	↓	SH3-containing GRB2-like protein 3-interacting protein 1 [B. mutus]
ENSP00000359892-D1	3.11	2.31E-167	↓	Choline transporter-like protein 5, partial [B. mutus]
yakG045207	3.11	3.34E-145	↓	Leucine-rich repeat-containing protein 40 [B. mutus]
ENSBTAP00000012404-D1	3.12	1.36E-79	↓	Protein phosphatase 1D, partial [B. mutus]
ENSBTAP00000005802-D1	3.12	0.00E+00	↓	histone H2A.Z-like [Falco cherrug]
ENSP00000383599-D1	3.12	0.00E+00	↓	WD repeat-containing protein 47 [B. mutus]
ENSBTAP00000015105-D1	3.12	2.03E-266	↓	Nucleolar complex protein 3-like protein, partial [B. mutus]
ENSBTAP00000019527-D1	3.12	1.00E-18	↓	Polyadenylate-binding protein-interacting protein 2B, partial [B. mutus]
ENSP00000360133-D1	3.14	2.05E-09	↓	hypothetical protein M91_00092, partial [B. mutus]
ENSBTAP00000028280-D2	3.14	0.00E+00	↓	GTP-binding protein SAR1a [Sus scrofa]
ENSBTAP00000011521-D1	3.14	1.33E-17	↓	Aldehyde dehydrogenase, mitochondrial, partial [B. mutus]
ENSP00000016171-D1	3.15	3.03E-12	↓	cytochrome c oxidase assembly protein COX15 homolog [B. mutus]
ENSP00000274276-D1	3.15	1.67E-99	↓	oncostatin-M-specific receptor subunit beta [B. mutus]
ENSBTAP00000023743-D1	3.15	0.00E+00	↓	Serglycin, partial [B. mutus]
ENSP00000349696-D1	3.16	1.38E-148	↓	choline/ethanolaminephosphotransferase 1 isoform X1 [Physeter catodon]
ENSBTAP00000001495-D1	3.16	1.30E-56	↓	galactocerebrosidase isoform X1 [Bison bison bison]
ENSBTAP00000033320-D1	3.16	0.00E+00	↓	Complement component C8 beta chain [B. mutus]
ENSBTAP00000040605-D6	3.16	0.00E+00	↓	Zinc finger protein 729, partial [B. mutus]
ENSP00000369860-D1	3.17	9.26E-258	↓	Motile sperm domain-containing protein 2, partial [B. mutus]
ENSBTAP00000029458-D2	3.17	9.78E-141	↓	D-dopachrome decarboxylase [B. taurus]

ENSBTAP00000012720-D1	3.18	2.80E-08	↓	T-cell leukemia/lymphoma protein 1B [B. mutus]
ENSP00000356959-D1	3.18	7.55E-07	↓	Nuclear receptor subfamily 1 group I member 3 [B. mutus]
ENSBTAP00000016996-D1	3.18	7.55E-07	↓	glutamyl-tRNA(Gln) amidotransferase subunit C, mitochondrial [B. mutus]
ENSP00000383145-D1	3.18	2.11E-05	↓	Protein SFI1-like protein [B. mutus]
yakG040511	3.18	2.11E-05	↓	ras-related protein Rab-27A-like [B. mutus]
ENSBTAP00000037843-D2	3.18	2.11E-05	↓	hypothetical protein M91_07312, partial [B. mutus]
ENSBTAP00000020034-D1	3.18	2.11E-05	↓	DNA-directed RNA polymerase, mitochondrial [B. mutus]
ENSP00000305976-D1	3.18	2.11E-05	↓	Serine/threonine-protein kinase SIK2, partial [B. mutus]
ENSP00000366977-D1	3.18	6.41E-04	↓	Pleckstrin-like protein domain-containing family G member 5, partial [B. mutus]
ENSBTAP00000050468-D1	3.18	6.41E-04	↓	.
ENSBTAP00000049946-D89	3.18	6.41E-04	↓	hypothetical protein M91_10420, partial [B. mutus]
ENSBTAP00000036113-D1	3.18	6.41E-04	↓	latrophilin-1 isoform X1 [B. mutus]
ENSBTAP00000016342-D1	3.18	6.41E-04	↓	Low-density lipoprotein receptor, partial [B. mutus]
ENSP00000379915-D1	3.18	6.41E-04	↓	Atrophin-1, partial [B. mutus]
ENSP00000392188-D32	3.18	6.41E-04	↓	hypothetical protein M91_04770, partial [B. mutus]
ENSP00000348216-D1	3.18	6.41E-04	↓	Tripartite motif-containing protein 4, partial [B. mutus]
ENSBTAP00000047385-D4	3.18	6.41E-04	↓	hypothetical protein M91_14596, partial [B. mutus]
ENSP00000361229-D1	3.18	4.73E-79	↓	tetraspanin-14 [B. taurus]
ENSBTAP00000012561-D1	3.19	3.17E-62	↓	MAD2L1-binding protein [B. mutus]
ENSBTAP00000015733-D1	3.19	0.00E+00	↓	Exosome complex exonuclease RRP43, partial [B. mutus]
ENSBTAP00000012884-D1	3.20	6.77E-50	↓	interferon-induced protein with tetratricopeptide repeats 3 [B. mutus]
ENSBTAP00000053020-D5	3.20	1.34E-95	↓	histone H3.1-like [B. mutus]
ENSP00000296043-D1	3.20	3.17E-14	↓	protein Shroom3 [B. mutus]
ENSBTAP00000049167-D1	3.21	1.07E-121	↓	Succinate receptor 1, partial [B. mutus]
ENSP00000260147-D1	3.21	3.83E-52	↓	protocadherin Fat 1 [B. mutus]
ENSP00000367440-D1	3.22	6.83E-151	↓	tyrosyl-DNA phosphodiesterase 2 [B. mutus]
ENSP00000320147-D1	3.22	7.91E-22	↓	histone-lysine N-methyltransferase EZH2 isoform X1 [Bison bison bison]
ENSP00000363015-D2	3.22	0.00E+00	↓	replication protein A 32 kDa subunit isoform X1 [Bison bison bison]
ENSBTAP00000053441-D1	3.23	4.36E-63	↓	FH1/FH2 domain-containing protein 3, partial [B. mutus]
ENSBTAP00000010105-D1	3.23	7.00E-59	↓	DDB1- and CUL4-associated factor 17, partial [B. mutus]
ENSP00000338235-D1	3.23	1.52E-211	↓	Protein LYRIC [B. mutus]
ENSP00000308927-D1	3.23	8.28E-40	↓	transcription factor SOX-5-like [B. mutus]
ENSP00000250263-D1	3.23	1.12E-54	↓	3'-5' exoribonuclease 1 [B. mutus]
ENSBTAP00000005079-D1	3.23	1.61E-23	↓	Cytidine monophosphate-N-acetylneuraminic acid hydroxylase, partial [B. mutus]
ENSBTAP00000015353-D1	3.23	4.23E-13	↓	surfeit locus protein 6 [B. mutus]
ENSP00000358714-D1	3.24	1.30E-17	↓	inactive serine protease 35 [B. mutus]
ENSP00000250377-D1	3.24	0.00E+00	↓	mitochondrial ribonuclease P protein 3 isoform X1 [B. mutus]
ENSBTAP00000053019-D1	3.24	3.01E-156	↓	plastin-1 [B. taurus]
ENSBTAP00000044190-D85	3.24	1.09E-11	↓	hypothetical protein M91_07993, partial [B. mutus]
ENSP00000317817-D1	3.24	3.87E-07	↓	SH2 domain-containing protein 3C isoform X1 [B. mutus]
ENSP00000382267-D6	3.24	3.87E-07	↓	cytochrome c-like [B. mutus]
ENSP00000336747-D1	3.25	2.66E-28	↓	Huntingtin-interacting protein 1, partial [B. mutus]
ENSBTAP00000000356-D1	3.27	1.11E-22	↓	translation factor GUF1, mitochondrial [B. mutus]
ENSP00000335628-D1	3.27	2.90E-258	↓	speedy protein A isoform X1 [B. mutus]
ENSP00000320924-D1	3.28	1.48E-30	↓	WAS/WASL-interacting protein family member 2, partial [B. mutus]
ENSP00000386170-D1	3.28	1.07E-05	↓	T-box transcription factor TBX20 [B. mutus]
ENSBTAP00000019311-D3	3.28	0.00E+00	↓	Carbonyl reductase [NADPH] 3, partial [B. mutus]
ENSBTAP00000022090-D1	3.29	0.00E+00	↓	polycomb group RING finger protein 1 [Ovis aries]
ENSBTAP00000001083-D1	3.29	1.41E-183	↓	Pleckstrin-like protein domain-containing family G member 7, partial [B. mutus]
ENSP00000377486-D1	3.29	4.52E-172	↓	39S ribosomal protein L19, mitochondrial [B. mutus]
ENSBTAP00000016407-D1	3.29	1.46E-21	↓	Rho guanine nucleotide exchange factor 11, partial [B. mutus]
ENSP00000229088-D1	3.30	1.47E-10	↓	TBC1 domain family member 30, partial [B. mutus]
ENSBTAP00000044662-D1	3.30	3.78E-86	↓	Collectin-12, partial [B. mutus]
ENSP00000367878-D1	3.31	7.59E-22	↓	hypothetical protein M91_11657, partial [B. mutus]
ENSP00000365806-D1	3.31	1.98E-07	↓	guanine nucleotide-binding protein-like 1 [B. mutus]

ENSBTAP00000004443-D1	3.31	6.99E-54	↓	E3 ubiquitin-protein ligase BRE1B [B. mutus]
ENSBTAP00000003986-D1	3.32	1.63E-175	↓	PAX3- and PAX7-binding protein 1 [B. taurus]
ENSBTAP00000012182-D2	3.32	3.93E-22	↓	serine/threonine-protein phosphatase 2A catalytic subunit beta isoform [Heterocephalus glaber]
ENSBTAP00000000737-D1	3.32	3.85E-09	↓	neuron-specific protein family member 2 [B. taurus]
ENSP00000417132-D1	3.32	3.85E-09	↓	ubiquitin carboxyl-terminal hydrolase BAP1 isoform X1 [B. mutus]
ENSP00000367308-D1	3.32	1.42E-146	↓	solute carrier family 41 member 2 [B. mutus]
ENSBTAP00000044202-D8	3.33	2.43E-85	↓	bucentaur [Cervus nippon]
ENSBTAP00000044540-D1	3.33	8.01E-24	↓	acylpyruvase FAHD1, mitochondrial [B. taurus]
ENSP00000412453-D1	3.33	0.00E+00	↓	Dolichol-phosphate mannosyltransferase [B. mutus]
ENSP00000387841-D1	3.33	1.27E-69	↓	ribosome-recycling factor, mitochondrial precursor [B. taurus]
ENSBTAP00000028134-D1	3.34	2.51E-137	↓	Exocyst complex component 6 [B. mutus]
ENSP00000362208-D2	3.34	5.75E-177	↓	28S ribosomal protein S15, mitochondrial [B. mutus]
ENSP00000358335-D1	3.34	1.98E-106	↓	mitogen-activated protein kinase kinase kinase 7 isoform X1 [Ovis aries]
ENSP00000392852-D1	3.36	1.38E-39	↓	hypothetical protein M91_16290 [B. mutus]
ENSBTAP00000019652-D1	3.37	0.00E+00	↓	Hyaluronan mediated motility receptor, partial [B. mutus]
ENSBTAP00000023587-D1	3.37	1.47E-41	↓	3-hydroxyacyl-CoA dehydratase 4, partial [B. mutus]
ENSBTAP00000052854-D1	3.37	1.31E-19	↓	Beta-taxilin [B. mutus]
ENSP00000316649-D1	3.37	7.79E-13	↓	oxysterol-binding protein-related protein 2 isoform X1 [B. mutus]
ENSP00000373194-D68	3.37	5.43E-06	↓	olfactory receptor 8H1-like [B. mutus]
ENSBTAP00000050211-D1	3.37	5.43E-06	↓	hypothetical protein M91_13164, partial [B. mutus]
ENSP00000360103-D1	3.37	3.17E-04	↓	Palmitoyltransferase ZDHHC9 [B. mutus]
ENSP00000333329-D1	3.37	3.17E-04	↓	Ubiquitin carboxyl-terminal hydrolase 17, partial [B. mutus]
ENSP00000359563-D1	3.37	3.17E-04	↓	cerebellar degeneration-related antigen 1 [Pantholops hodgsonii]
ENSBTAP00000029684-D1	3.37	3.17E-04	↓	Glycosyltransferase 1 domain-containing protein 1, partial [B. mutus]
ENSP00000265348-D2	3.37	3.17E-04	↓	cullin-7 [B. taurus]
yakA00220	3.37	3.17E-04	↓	NADH dehydrogenase [ubiquinone] 1 alpha subcomplex subunit 1-like [B. mutus]
ENSP00000381854-D1	3.37	3.17E-04	↓	Tensin-3 [B. mutus]
ENSBTAP00000023728-D1	3.38	9.44E-134	↓	hypothetical protein M91_05440, partial [B. mutus]
ENSP00000261556-D1	3.39	2.54E-30	↓	hypothetical protein M91_11980, partial [B. mutus]
ENSP00000263956-D1	3.40	1.20E-244	↓	general transcription factor 3C polypeptide 3 [B. mutus]
ENSP00000347206-D1	3.40	4.01E-13	↓	glycosylphosphatidylinositol anchor attachment 1 protein isoform X1 [B. taurus]
ENSP00000160382-D2	3.40	1.96E-112	↓	actin-like protein 6A isoform X1 [Balaenoptera acutorostrata scammoni]
ENSBTAP00000008406-D1	3.41	3.48E-20	↓	THAP domain-containing protein 11 [B. mutus]
ENSP00000313854-D1	3.41	1.53E-25	↓	Cleft lip and palate transmembrane protein 1-like protein, partial [B. mutus]
ENSBTAP00000029970-D1	3.41	1.02E-09	↓	nudix (nucleoside diphosphate linked moiety X)-type motif 11 [B. taurus]
ENSBTAP00000009556-D1	3.43	4.49E-120	↓	ATP-dependent RNA helicase DDX55, partial [B. mutus]
ENSBTAP00000035035-D1	3.43	3.16E-56	↓	HMG domain-containing protein 3, partial [B. mutus]
ENSBTAP00000034967-D1	3.43	5.17E-08	↓	Arf-GAP with coiled-coil, ANK repeat and PH domain-containing protein 1, partial [B. mutus]
ENSP00000269577-D1	3.43	0.00E+00	↓	DNA topoisomerase 2-alpha, partial [B. mutus]
ENSP00000313243-D1	3.43	1.52E-72	↓	Heat shock factor protein 5, partial [B. mutus]
ENSP00000256429-D1	3.43	3.64E-42	↓	Methyl-CpG-binding domain protein 2, partial [B. mutus]
ENSP00000229268-D1	3.44	1.03E-11	↓	ubiquitin carboxyl-terminal hydrolase 5 [B. mutus]
ENSP00000389913-D1	3.44	1.03E-11	↓	Pleckstrin-like protein domain-containing family M member 1, partial [B. mutus]
ENSP00000265168-D2	3.45	2.22E-84	↓	hydroxyacyl-coenzyme A dehydrogenase, mitochondrial isoform X2 [B. mutus]
ENSBTAP00000012347-D1	3.45	1.25E-124	↓	kinesin-like protein KIF11 [B. mutus]
ENSP00000380293-D13	3.46	3.57E-156	↓	Zinc finger protein 420, partial [B. mutus]
ENSP00000359361-D1	3.46	9.46E-206	↓	Putative ATP-dependent RNA helicase DDX43 [B. mutus]
ENSBTAP00000027398-D1	3.46	5.00E-43	↓	probable ATP-dependent RNA helicase DDX59 [B. mutus]
ENSP00000035307-D1	3.46	2.75E-06	↓	Chondroitin sulfate glucuronyltransferase [B. mutus]
ENSP00000345412-D1	3.46	2.75E-06	↓	Cleavage and polyadenylation specificity factor subunit 7, partial [B. mutus]
ENSBTAP00000009521-D1	3.46	3.38E-216	↓	Mitotic checkpoint serine/threonine-protein kinase BUB1 beta, partial [B. mutus]
ENSBTAP00000029739-D1	3.47	5.38E-36	↓	rap guanine nucleotide exchange factor 2 isoform X8 [B. taurus]
ENSP00000411096-D1	3.47	6.02E-49	↓	ATP-binding cassette sub-family A member 13-like [B. mutus]
ENSP00000358314-D1	3.49	5.46E-14	↓	LIX1-like protein [B. taurus]
ENSP00000205890-D1	3.50	1.33E-23	↓	unconventional myosin-XV [B. mutus]

ENSP00000381684-D7	3.50	1.48E-27	↓	hypothetical protein M91_12106, partial [B. mutus]
ENSBTAP00000022518-D1	3.51	1.65E-31	↓	Transcription factor E2F7 [B. mutus]
ENSBTAP00000010212-D1	3.51	0.00E+00	↓	Cell division protein kinase 5 [B. mutus]
ENSP00000417175-D2	3.51	2.80E-14	↓	Vesicle-associated membrane protein-associated protein B, partial [B. mutus]
ENSBTAP00000000194-D1	3.51	0.00E+00	↓	Ankyrin repeat, SAM and basic leucine zipper domain-containing protein 1, partial [B. mutus]
ENSBTAP00000017757-D1	3.52	2.91E-16	↓	Homeobox protein Nkx-2.8, partial [B. mutus]
ENSP00000360497-D1	3.52	1.16E-72	↓	RING finger protein 113A [B. mutus]
ENSP00000237858-D3	3.52	0.00E+00	↓	glutaredoxin-1 [B. mutus]
ENSBTAP00000024390-D1	3.52	4.03E-209	↓	exosome complex component RRP42 [B. taurus]
ENSP00000261908-D1	3.53	3.92E-28	↓	Neogenin, partial [B. mutus]
ENSP00000344936-D1	3.53	0.00E+00	↓	securin isoform X1 [B. mutus]
ENSBTAP00000043896-D1	3.53	8.19E-105	↓	Zygote arrest protein 1, partial [B. mutus]
ENSP00000410083-D1	3.53	9.64E-58	↓	vezatin-like, partial [B. mutus]
ENSP00000336673-D1	3.54	2.67E-38	↓	transmembrane protein 106B [B. taurus]
ENSP00000351137-D1	3.54	1.49E-16	↓	pre-mRNA-splicing factor SYF1 [B. mutus]
ENSP00000397807-D6	3.54	1.44E-14	↓	Acyl-protein thioesterase 2, partial [B. mutus]
ENSBTAP00000017908-D1	3.54	1.39E-12	↓	Protein FAM55C, partial [B. mutus]
ENSP00000407460-D1	3.54	1.39E-06	↓	Secernin-1, partial [B. mutus]
ENSP00000311489-D1	3.54	1.39E-06	↓	Spectrin beta chain, brain 2 [B. mutus]
ENSBTAP00000040958-D1	3.54	1.39E-06	↓	Glioma tumor suppressor candidate region 2 protein [B. mutus]
yakA15909	3.54	1.57E-04	↓	hypothetical protein M91_21668 [B. mutus]
ENSBTAP00000007796-D1	3.54	1.57E-04	↓	BarH-like 2 homeobox protein, partial [B. mutus]
ENSP00000382659-D1	3.54	1.57E-04	↓	transient receptor potential cation channel subfamily V member 1 [B. mutus]
ENSP00000375693-D1	3.54	1.57E-04	↓	DNA polymerase delta catalytic subunit [B. mutus]
ENSBTAP00000042187-D4	3.54	1.57E-04	↓	60S ribosomal protein L39 [Jaculus jaculus]
ENSBTAP00000016228-D1	3.54	1.57E-04	↓	Cell division protein kinase 10, partial [B. mutus]
ENSBTAP00000040666-D1	3.55	1.02E-50	↓	target of EGR1 protein 1 [B. taurus]
ENSP00000261015-D1	3.56	6.47E-287	↓	Ribosome biogenesis protein WDR12, partial [B. mutus]
ENSP00000412693-D1	3.56	1.01E-105	↓	Kinesin light chain 1, partial [B. mutus]
ENSBTAP00000012305-D1	3.57	3.43E-37	↓	oncoprotein-induced transcript 3 protein [B. mutus]
ENSP00000364813-D1	3.57	7.29E-295	↓	uncharacterized protein LOC474849, partial [Canis lupus familiaris]
ENSBTAP00000027029-D1	3.57	4.32E-21	↓	dual specificity phosphatase 5 [B. taurus]
ENSBTAP00000002309-D1	3.58	7.63E-54	↓	DNA-binding protein RFXANK isoform X1 [B. mutus]
ENSBTAP00000028036-D1	3.58	4.13E-19	↓	ADM isoform X1 [B. mutus]
ENSP00000419457-D1	3.58	6.97E-11	↓	Homeobox protein NOBOX, partial [B. mutus]
ENSBTAP00000023758-D53	3.59	3.76E-15	↓	hypothetical protein M91_14149, partial [B. mutus]
ENSBTAP00000044395-D1	3.59	6.91E-09	↓	hypothetical protein M91_03817, partial [B. mutus]
ENSP00000392188-D14	3.59	6.91E-09	↓	hypothetical protein M91_16901, partial [B. mutus]
ENSBTAP00000005670-D1	3.59	6.91E-09	↓	pygopus homolog 2 [B. taurus]
ENSBTAP00000027564-D2	3.59	2.62E-75	↓	Actin-related protein 2/3 complex subunit 1B [B. mutus]
ENSBTAP00000035133-D1	3.60	1.49E-125	↓	eukaryotic peptide chain release factor subunit 1 [Anolis carolinensis]
ENSP00000367385-D1	3.60	2.55E-40	↓	glycylpeptide N-tetradecanoyltransferase 2 isoform X1 [B. taurus]
ENSP00000395574-D1	3.60	1.37E-88	↓	transcription elongation factor B polypeptide 3 isoform X1 [B. taurus]
ENSP00000265814-D1	3.60	5.01E-53	↓	protein CBFA2T1 isoform X1 [Bison bison bison]
ENSBTAP00000018375-D1	3.62	1.09E-19	↓	coiled-coil domain-containing protein 170-like [B. mutus]
ENSBTAP00000040846-D1	3.62	3.55E-11	↓	Zinc finger FYVE domain-containing protein 26 [B. mutus]
ENSBTAP00000014486-D1	3.63	5.85E-22	↓	Protein phosphatase 1 regulatory subunit 1A, partial [B. mutus]
ENSBTAP00000007524-D1	3.63	1.86E-13	↓	synaptotagmin-like protein 4 [B. mutus]
ENSP00000333125-D1	3.63	1.86E-13	↓	mediator of RNA polymerase II transcription subunit 12 [B. taurus]
ENSBTAP00000015388-D1	3.63	1.86E-13	↓	Acylamino-acid-releasing enzyme, partial [B. mutus]
ENSBTAP00000025179-D1	3.63	1.86E-13	↓	Inosine-5'-monophosphate dehydrogenase 2, partial [B. mutus]
ENSBTAP00000042908-D1	3.64	0.00E+00	↓	E3 SUMO-protein ligase RanBP2 [B. mutus]
ENSP00000408068-D1	3.64	3.00E-22	↓	MMS19 nucleotide excision repair protein-like protein, partial [B. mutus]
ENSP00000343552-D1	3.64	3.51E-09	↓	protein-S-isoprenylcysteine O-methyltransferase [B. mutus]
ENSP00000361158-D1	3.64	3.51E-09	↓	dual specificity testis-specific protein kinase 2 isoform X4 [B. taurus]

ENSP00000266269-D1	3.64	3.51E-09	↓	POZ-, AT hook-, and zinc finger-containing protein 1 [B. taurus]
ENSBTAP00000007481-D1	3.65	1.57E-37	↓	Transmembrane emp24 domain-containing protein 10, partial [B. mutus]
ENSBTAP00000027276-D1	3.65	1.45E-85	↓	sterile alpha motif domain-containing protein 15 [B. mutus]
ENSBTAP00000001184-D1	3.66	2.40E-42	↓	hypothetical protein M91_08917, partial [B. mutus]
ENSP00000332359-D1	3.67	1.17E-53	↓	transcription elongation factor A protein-like 4 [B. mutus]
ENSP00000299563-D1	3.67	3.88E-36	↓	RING finger protein 169, partial [B. mutus]
ENSBTAP00000019582-D1	3.69	7.52E-120	↓	gamma-tubulin complex component 5 [B. mutus]
ENSP00000299339-D1	3.69	4.35E-199	↓	Claudin-10, partial [B. mutus]
ENSP00000238561-D1	3.69	9.26E-12	↓	Putative aarF domain-containing protein kinase 1, partial [B. mutus]
ENSBTAP00000042466-D4	3.69	9.26E-12	↓	unnamed protein product [Tetraodon nigroviridis]
ENSBTAP00000009776-D1	3.69	3.55E-07	↓	Protein KIAA1199 [B. mutus]
ENSBTAP00000016955-D1	3.69	3.55E-07	↓	Kinesin light chain 4, partial [B. mutus]
ENSP00000377374-D1	3.69	7.75E-05	↓	Endoplasmic reticulum-Golgi intermediate compartment protein 1, partial [B. mutus]
ENSBTAP00000018404-D1	3.69	7.75E-05	↓	single-stranded DNA-binding protein 2 isoform X2 [Chrysochloris asiatica]
ENSP00000367635-D1	3.69	7.75E-05	↓	cytoplasmic tRNA 2-thiolation protein 2 [B. mutus]
ENSBTAP00000041432-D1	3.69	7.75E-05	↓	Hormone-sensitive lipase, partial [B. mutus]
ENSBTAP00000021507-D1	3.69	7.75E-05	↓	Mitogen-activated protein kinase 3, partial [B. mutus]
ENSP00000263125-D2	3.69	7.75E-05	↓	Protein kinase C delta type [B. mutus]
ENSP00000262375-D7	3.69	7.75E-05	↓	hypothetical protein M91_10296, partial [B. mutus]
ENSBTAP00000047643-D68	3.69	7.75E-05	↓	uncharacterized protein LOC105605376 [Ovis aries]
yakG011015	3.69	7.75E-05	↓	hypothetical protein M91_07947 [B. mutus]
ENSBTAP00000004002-D1	3.69	7.75E-05	↓	segment polarity protein dishevelled homolog DVL-2 isoform X2 [B. mutus]
ENSBTAP00000002179-D1	3.69	7.75E-05	↓	conserved oligomeric Golgi complex subunit 8 [B. mutus]
ENSBTAP00000045681-D1	3.69	1.27E-03	↓	Immunoglobulin superfamily DCC subclass member 3, partial [B. mutus]
ENSP00000274764-D9	3.69	1.27E-03	↓	histone H2B type 1-like [B. mutus]
ENSP00000408239-D1	3.69	1.27E-03	↓	Polyadenylate-binding protein 4-like protein [B. mutus]
ENSP00000344244-D1	3.69	1.27E-03	↓	N-acetylated-alpha-linked acidic dipeptidase-like protein [B. mutus]
ENSBTAP00000010598-D1	3.69	1.27E-03	↓	glutamate decarboxylase 2 [B. mutus]
ENSP00000292476-D1	3.69	1.27E-03	↓	cleavage and polyadenylation specificity factor subunit 4 isoform X2 [Chinchilla lanigera]
ENSP00000360261-D1	3.69	1.27E-03	↓	Tectonic-3, partial [B. mutus]
ENSBTAP00000013120-D1	3.69	1.27E-03	↓	BRICHOS domain-containing protein 5 [B. mutus]
ENSBTAP00000017690-D1	3.69	1.27E-03	↓	Zinc finger and BTB domain-containing protein 7C, partial [B. mutus]
ENSP00000251472-D1	3.69	1.27E-03	↓	microtubule-associated serine/threonine-protein kinase 1 [B. mutus]
ENSBTAP00000040256-D1	3.69	1.27E-03	↓	ADP-ribosylation factor-like protein 16 [B. mutus]
ENSBTAP00000047813-D1	3.69	1.27E-03	↓	Putative threonine protease PRSS50 [B. mutus]
ENSBTAP00000025873-D2	3.69	1.27E-03	↓	hsc70-interacting protein isoform X3 [Ovis aries musimon]
ENSBTAP00000043333-D1	3.69	1.27E-03	↓	glutaryl-CoA dehydrogenase, mitochondrial [B. mutus]
ENSBTAP00000018930-D2	3.69	1.27E-03	↓	Tracheal antimicrobial peptide [B. mutus]
ENSP00000301924-D1	3.69	1.27E-03	↓	tripartite motif-containing protein 35 [B. taurus]
ENSBTAP00000001547-D1	3.69	1.27E-03	↓	core histone macro-H2A.2-like isoform X1 [Ictidomys tridecemlineatus]
ENSP00000262731-D1	3.69	1.27E-03	↓	Rho GTPase-activating protein 8, partial [B. mutus]
ENSP00000301327-D1	3.69	1.27E-03	↓	major facilitator superfamily domain-containing protein 3 [B. mutus]
ENSBTAP00000017527-D1	3.69	1.27E-03	↓	cytotoxic T-lymphocyte protein 4 precursor [B. taurus]
ENSP00000402050-D2	3.69	1.27E-03	↓	hypothetical protein M91_12177, partial [B. mutus]
ENSBTAP00000017274-D1	3.69	1.27E-03	↓	cadherin-6 precursor [B. taurus]
ENSP00000323901-D1	3.69	1.27E-03	↓	Frizzled-2, partial [B. mutus]
ENSP00000412696-D1	3.69	1.27E-03	↓	Protein YIPF7, partial [B. mutus]
ENSBTAP00000015173-D1	3.69	1.27E-03	↓	leukocyte antigen CD37 [B. mutus]
ENSBTAP00000040882-D1	3.69	1.27E-03	↓	uncharacterized protein KIAA1522 homolog [B. mutus]
ENSP00000316786-D1	3.69	1.27E-03	↓	corticosteroid 11-beta-dehydrogenase isozyme 2 [Bison bison bison]
ENSBTAP00000028056-D1	3.69	1.27E-03	↓	Suppressor of fused-like protein [B. mutus]
ENSBTAP00000031858-D1	3.69	1.27E-03	↓	branched-chain-amino-acid aminotransferase, mitochondrial isoform X1 [B. mutus]
ENSP00000284601-D1	3.69	1.27E-03	↓	protein phosphatase 1 regulatory subunit 3A-like [B. mutus]
ENSP00000322273-D1	3.69	1.27E-03	↓	Olfactomedin-like protein 3 [B. mutus]
ENSP00000262219-D1	3.69	1.27E-03	↓	Annexin A13, partial [B. mutus]

ENSBTAP00000022431-D1	3.69	1.27E-03	↓	testosterone 17-beta-dehydrogenase 3 [B. mutus]
ENSBTAP00000007855-D2	3.69	1.27E-03	↓	disheveled-associated activator of morphogenesis 2 [B. mutus]
ENSBTAP00000021752-D1	3.69	1.27E-03	↓	Transcriptional enhancer factor TEF-4 [B. mutus]
ENSBTAP00000034715-D1	3.69	1.27E-03	↓	Signal transducer and activator of transcription 5A [B. mutus]
yakG029468	3.69	1.27E-03	↓	Calcium-activated potassium channel subunit beta-3 [B. mutus]
ENSP00000414215-D1	3.69	1.27E-03	↓	Transmembrane channel-like protein 3 [B. mutus]
ENSBTAP00000011048-D1	3.69	1.27E-03	↓	amyloid beta A4 precursor protein-binding family A member 3 [B. mutus]
ENSP00000288048-D1	3.69	1.27E-03	↓	uncharacterized protein C1orf158 homolog [B. mutus]
ENSP00000392188-D60	3.69	1.27E-03	↓	hypothetical protein M91_08847, partial [B. mutus]
ENSBTAP00000053241-D1	3.69	1.27E-03	↓	glucose-fructose oxidoreductase domain-containing protein 1 isoform X1 [B. mutus]
ENSBTAP00000019336-D1	3.69	1.27E-03	↓	Phosphoglycerate mutase 2, partial [B. mutus]
ENSP00000250160-D1	3.69	1.27E-03	↓	WNT1-inducible-signaling pathway protein 1 [B. mutus]
ENSP00000377298-D1	3.69	1.27E-03	↓	Nuclear mitotic apparatus protein 1, partial [B. mutus]
ENSP00000276055-D1	3.69	1.27E-03	↓	carbohydrate sulfotransferase 7 [Ailuropoda melanoleuca]
ENSP00000413540-D1	3.69	1.27E-03	↓	uncharacterized protein LOC104972129 isoform X2 [B. taurus]
ENSBTAP00000018130-D1	3.69	1.27E-03	↓	killer cell lectin-like receptor subfamily G member 1 [B. mutus]
ENSP00000262577-D1	3.69	1.27E-03	↓	Zinc finger CCCH domain-containing protein 3 [B. mutus]
ENSBTAP00000050273-D21	3.69	1.27E-03	↓	Retrovirus-related Pol polyprotein from type-1 retrotransposable element R2, partial [B. mutus]
ENSP00000361942-D1	3.69	1.27E-03	↓	Regulating synaptic membrane exocytosis protein 4, partial [B. mutus]
ENSP00000384983-D1	3.69	1.27E-03	↓	formin-2-like, partial [B. mutus]
ENSBTAP00000032532-D1	3.69	1.27E-03	↓	apical endosomal glycoprotein [Vicugna pacos]
ENSBTAP00000019861-D1	3.69	1.27E-03	↓	hypothetical protein M91_01792, partial [B. mutus]
ENSP00000364298-D1	3.69	1.27E-03	↓	FK506-binding protein-like protein [B. mutus]
ENSBTAP00000032574-D1	3.69	1.27E-03	↓	Protein VAC14-like protein [B. mutus]
ENSP00000321744-D1	3.69	1.27E-03	↓	histone H2B type 1-like [Propithecus coquereli]
ENSBTAP00000049624-D1	3.69	1.27E-03	↓	transcription factor RelB [Bison bison bison]
ENSBTAP00000000263-D1	3.69	1.27E-03	↓	glutamate receptor ionotropic, NMDA 2B [B. mutus]
ENSBTAP00000011438-D1	3.69	1.27E-03	↓	serine/arginine repetitive matrix protein 4, partial [B. mutus]
ENSBTAP00000016860-D82	3.69	1.27E-03	↓	zinc finger protein 485, partial [Capra hircus]
ENSP00000317904-D1	3.69	1.27E-03	↓	glycogen [starch] synthase, muscle isoform X1 [B. mutus]
ENSP00000400796-D1	3.69	1.27E-03	↓	hypothetical protein EGM_20057, partial [Macaca fascicularis]
ENSBTAP00000038686-D1	3.69	1.27E-03	↓	[Pyruvate dehydrogenase [lipoamide]] kinase isozyme 4, mitochondrial [B. mutus]
ENSBTAP00000020978-D18	3.69	1.27E-03	↓	mariner transposase [Homo sapiens]
ENSP00000380401-D1	3.69	1.27E-03	↓	FAD-dependent oxidoreductase domain-containing protein 2 [B. mutus]
ENSP00000042931-D1	3.69	1.27E-03	↓	bestrophin-2 [B. mutus]
ENSBTAP00000012416-D1	3.69	1.27E-03	↓	transmembrane protein 79 [B. mutus]
ENSBTAP00000011449-D1	3.69	1.27E-03	↓	Tetratricopeptide repeat protein 15, partial [B. mutus]
ENSP00000392593-D91	3.69	1.27E-03	↓	zinc finger protein 33B-like [Ovis aries musimon]
ENSP00000397695-D1	3.69	1.27E-03	↓	Zinc finger protein ZIC 4, partial [B. mutus]
ENSBTAP00000003928-D1	3.69	1.27E-03	↓	Scavenger receptor cysteine-rich domain-containing group B protein, partial [B. mutus]
ENSBTAP00000024720-D1	3.69	1.27E-03	↓	dihydropyrimidinase-related protein 5 isoform X2 [Ovis aries musimon]
ENSBTAP00000028921-D1	3.69	1.27E-03	↓	Nuclear receptor ROR-beta, partial [B. mutus]
ENSP00000407306-D1	3.69	1.27E-03	↓	high mobility group protein HMGI-C [Ailuropoda melanoleuca]
ENSBTAP00000049632-D1	3.69	1.27E-03	↓	olfactory receptor 52K1 [B. taurus]
ENSP00000393562-D4	3.69	1.27E-03	↓	Leucine-rich repeat transmembrane neuronal protein 4 [B. mutus]
ENSBTAP00000002699-D1	3.69	1.27E-03	↓	protein FAM217A [B. mutus]
ENSP00000371846-D1	3.69	1.27E-03	↓	Deformed epidermal autoregulatory factor 1-like protein, partial [B. mutus]
ENSP00000225973-D1	3.69	1.27E-03	↓	Rho-related GTP-binding protein RhoN, partial [B. mutus]
ENSBTAP00000036408-D1	3.69	1.27E-03	↓	serine protease 38 [B. mutus]
ENSBTAP00000052438-D1	3.69	1.27E-03	↓	Mas-related G-protein coupled receptor member D, partial [B. mutus]
ENSBTAP00000028547-D1	3.69	1.27E-03	↓	transmembrane and coiled-coil domain-containing protein 5B-like [B. mutus]
ENSBTAP00000010671-D1	3.69	1.27E-03	↓	BTB/POZ domain-containing adapter for CUL3-mediated RhoA degradation protein 2 [B. mutus]
ENSBTAP00000038597-D1	3.69	1.27E-03	↓	Synaptic vesicle glycoprotein 2B, partial [B. mutus]
ENSBTAP00000049352-D1	3.69	1.27E-03	↓	uncharacterized protein LOC616319 [B. taurus]
ENSBTAP00000039941-D1	3.69	1.27E-03	↓	Rho GDP-dissociation inhibitor 3, partial [B. mutus]

ENSP00000249330-D1	3.69	1.27E-03	↓	Neurosecretory protein VGF, partial [B. mutus]
ENSP00000335147-D1	3.69	1.27E-03	↓	ankyrin repeat domain-containing protein 37 [B. taurus]
ENSP00000330808-D1	3.69	1.27E-03	↓	otolin-1 [B. mutus]
ENSBTAP00000016726-D1	3.69	1.27E-03	↓	tektin-4-like [B. mutus]
ENSBTAP00000039202-D3	3.69	1.27E-03	↓	Nuclear factor 1 B-type, partial [Fukomys damarensis]
ENSP00000385899-D2	3.69	1.27E-03	↓	Protein sidekick-2, partial [B. mutus]
ENSBTAP00000018382-D1	3.69	1.27E-03	↓	desmoglein-1 [B. mutus]
ENSP00000299092-D1	3.69	1.27E-03	↓	Putative G-protein coupled receptor 176, partial [B. mutus]
ENSP00000330812-D1	3.69	1.27E-03	↓	Putative short-chain dehydrogenase/reductase family 42E member 2, partial [B. mutus]
ENSBTAP00000048897-D1	3.69	1.27E-03	↓	Sodium/hydrogen exchanger 5, partial [B. mutus]
ENSBTAP00000009611-D1	3.69	1.27E-03	↓	Ras-related protein Rab-3A, partial [B. mutus]
ENSP00000274979-D1	3.69	1.27E-03	↓	anoctamin-7 isoform X4 [Macaca nemestrina]
ENSP00000290871-D1	3.69	1.27E-03	↓	Testis, prostate and placenta-expressed protein, partial [B. mutus]
ENSBTAP00000022753-D1	3.69	1.27E-03	↓	GTP-binding protein Rhes, partial [B. mutus]
ENSBTAP00000019695-D1	3.69	1.27E-03	↓	Carboxypeptidase Z, partial [B. mutus]
ENSBTAP00000015695-D2	3.69	1.27E-03	↓	uncharacterized protein C19orf43 homolog [Camelus bactrianus]
ENSP00000362638-D1	3.69	1.27E-03	↓	MARCKS-related protein [B. taurus]
ENSBTAP00000006418-D1	3.69	1.27E-03	↓	forkhead box protein O4 [B. taurus]
ENSBTAP00000011569-D1	3.69	1.27E-03	↓	Potassium-transporting ATPase alpha chain 1, partial [B. mutus]
ENSP00000342557-D1	3.69	1.27E-03	↓	interleukin 4 induced 1 [B. mutus]
ENSP00000338607-D1	3.69	1.27E-03	↓	protein THEM6 precursor [B. taurus]
ENSBTAP00000015994-D1	3.69	1.27E-03	↓	Diphosphomevalonate decarboxylase, partial [B. mutus]
ENSBTAP00000041372-D1	3.69	1.27E-03	↓	Sushi domain-containing protein 1, partial [B. mutus]
ENSBTAP00000050857-D1	3.69	1.27E-03	↓	Double C2-like domain-containing protein gamma [B. mutus]
ENSBTAP00000018863-D2	3.69	1.27E-03	↓	Catenin alpha-2, partial [B. mutus]
ENSBTAP00000016194-D1	3.69	1.27E-03	↓	BOLA class I histocompatibility antigen, alpha chain BL3-6, partial [B. mutus]
ENSBTAP00000052590-D4	3.69	1.27E-03	↓	hypothetical protein M91_03888, partial [B. mutus]
ENSP00000264036-D1	3.69	1.27E-03	↓	Cell surface glycoprotein MUC18, partial [B. mutus]
ENSP00000318557-D1	3.69	1.27E-03	↓	Solute carrier family 12 member 4, partial [B. mutus]
ENSP00000351975-D1	3.69	1.27E-03	↓	chromosome 16 open reading frame 5 [Homo sapiens]
ENSBTAP00000004717-D1	3.69	1.27E-03	↓	Putative alpha-ketoglutarate-dependent dioxygenase ABH4, partial [B. mutus]
ENSP00000264651-D2	3.69	1.27E-03	↓	keratin, type I cytoskeletal 24 [B. mutus]
ENSBTAP00000034009-D1	3.69	1.27E-03	↓	protein S100-A12 isoform X1 [B. mutus]
ENSBTAP00000021720-D1	3.69	1.27E-03	↓	Sodium/nucleoside cotransporter 2 [B. mutus]
yakA24149	3.69	1.27E-03	↓	Collagen alpha-1(XXV) chain [B. mutus]
ENSP00000408903-D1	3.69	1.27E-03	↓	Immunoglobulin superfamily member 2, partial [B. mutus]
ENSBTAP00000001278-D1	3.69	1.27E-03	↓	serine/threonine-protein kinase TNNI3K [B. taurus]
ENSP00000314145-D1	3.69	1.27E-03	↓	Carboxylesterase 8, partial [B. mutus]
ENSBTAP00000000407-D1	3.69	1.27E-03	↓	nucleoside diphosphate-linked moiety X motif 13 [B. taurus]
ENSBTAP00000011592-D1	3.69	1.27E-03	↓	XK-related protein 8 [B. mutus]
ENSP00000356370-D1	3.69	1.27E-03	↓	Crumbs-like protein 1, partial [B. mutus]
ENSP00000204279-D4	3.69	1.27E-03	↓	Protein yippee-like 1 [B. mutus]
ENSP00000360158-D1	3.69	1.27E-03	↓	polycystic kidney and hepatic disease 1 (autosomal recessive), partial [B. mutus]
ENSBTAP00000016647-D1	3.69	1.27E-03	↓	Calcineurin B-like protein 2, partial [B. mutus]
ENSBTAP00000053057-D3	3.69	1.27E-03	↓	Complement factor H-related protein 3, partial [B. mutus]
ENSP00000411354-D1	3.69	1.27E-03	↓	hypothetical protein M91_16574 [B. mutus]
ENSBTAP00000029525-D1	3.69	1.27E-03	↓	RBPJ-interacting and tubulin-associated protein [B. mutus]
ENSBTAP00000041865-D1	3.69	1.27E-03	↓	proline-rich protein 5-like [Capra hircus]
ENSP00000387907-D1	3.69	1.27E-03	↓	protein ANKUB1 [B. mutus]
ENSBTAP00000006958-D1	3.69	1.27E-03	↓	GDNF family receptor alpha-3, partial [B. mutus]
ENSBTAP00000016090-D1	3.69	1.27E-03	↓	alpha-aminoadipic semialdehyde synthase, mitochondrial isoform X1 [B. mutus]
ENSBTAP00000044190-D33	3.69	1.27E-03	↓	hypothetical protein M91_17522, partial [B. mutus]
ENSP00000230588-D1	3.69	1.27E-03	↓	Meprin A subunit alpha, partial [B. mutus]
ENSBTAP00000041536-D57	3.69	1.27E-03	↓	olfactory receptor 6C1-like [B. taurus]
ENSP00000316222-D1	3.69	1.27E-03	↓	glycoprotein hormone beta-5 [B. taurus]

ENSP00000349486-D1	3.69	1.27E-03	↓	protein scribble homolog [B. mutus]
ENSP00000300061-D1	3.69	1.27E-03	↓	amiloride-sensitive sodium channel subunit gamma isoform X2 [B. taurus]
ENSP00000419194-D1	3.69	1.27E-03	↓	prostate-associated microseminoprotein [B. taurus]
ENSBTAP00000052650-D1	3.69	1.27E-03	↓	Alkylglycerol monooxygenase, partial [B. mutus]
ENSBTAP00000040053-D1	3.69	1.27E-03	↓	sorting nexin-18 [B. mutus]
ENSP00000411015-D1	3.69	1.27E-03	↓	rho GTPase-activating protein 1 [B. taurus]
ENSBTAP00000001595-D1	3.69	1.27E-03	↓	Putative protein KIAA1462-like protein [B. mutus]
ENSBTAP00000034529-D1	3.69	1.27E-03	↓	hypothetical protein M91_18808, partial [B. mutus]
ENSP00000343348-D1	3.69	1.27E-03	↓	vimentin-type intermediate filament-associated coiled-coil protein [B. mutus]
ENSBTAP00000010194-D1	3.69	1.27E-03	↓	Kinesin-like protein KIFC2 [B. mutus]
ENSBTAP00000019482-D1	3.69	1.27E-03	↓	ATP-binding cassette sub-family D member 4 [B. mutus]
ENSBTAP00000028105-D1	3.69	1.27E-03	↓	clavesin-2 [B. taurus]
ENSP00000226279-D1	3.69	1.27E-03	↓	ADP-ribosyl cyclase 1, partial [B. mutus]
ENSBTAP00000035832-D1	3.69	1.27E-03	↓	NF-kappa-B inhibitor epsilon, partial [B. mutus]
ENSBTAP00000022973-D1	3.69	1.27E-03	↓	fibroblast growth factor 19 [B. taurus]
ENSP00000386794-D1	3.69	1.27E-03	↓	hypothetical protein M91_07915, partial [B. mutus]
yakA06234	3.69	1.27E-03	↓	Growth hormone-regulated TBC protein 1 [B. mutus]
ENSBTAP00000006736-D3	3.69	1.27E-03	↓	slit homolog 1 protein [B. mutus]
ENSP00000386167-D15	3.69	1.27E-03	↓	hypothetical protein M91_02341 [B. mutus]
ENSBTAP00000005212-D1	3.69	1.27E-03	↓	inositol 1,4,5-trisphosphate receptor-interacting protein [B. mutus]
ENSBTAP00000015578-D1	3.69	1.27E-03	↓	stAR-related lipid transfer protein 3 isoform X1 [B. mutus]
ENSBTAP00000023232-D1	3.69	1.27E-03	↓	Krueppel-like factor 3 isoform X1 [Physeter catodon]
ENSP00000225823-D1	3.69	1.27E-03	↓	Amiloride-sensitive cation channel 1, neuronal, partial [B. mutus]
ENSP00000249883-D1	3.69	1.27E-03	↓	angiomotin-like protein 2 isoform X2 [B. mutus]
ENSBTAP00000037863-D1	3.69	1.27E-03	↓	Protein FAM26F [B. mutus]
ENSBTAP00000039748-D1	3.69	1.27E-03	↓	hypothetical protein M91_07905, partial [B. mutus]
ENSP00000308361-D1	3.69	1.27E-03	↓	P2Y purinoceptor 14 [Bison bison bison]
ENSP00000333603-D8	3.69	1.27E-03	↓	hypothetical protein M91_00740, partial [B. mutus]
ENSP00000309591-D1	3.69	1.27E-03	↓	cAMP-dependent protein kinase catalytic subunit alpha [Cercocebus atys]
ENSBTAP00000006372-D1	3.69	1.27E-03	↓	Olfactomedin-like protein 2A, partial [B. mutus]
ENSP00000320083-D1	3.69	1.27E-03	↓	N-acetylated-alpha-linked acidic dipeptidase 2, partial [B. mutus]
ENSBTAP00000002336-D1	3.69	1.27E-03	↓	F-box only protein 17 [B. mutus]
ENSBTAP00000032393-D2	3.69	1.27E-03	↓	liver carboxylesterase-like [B. mutus]
ENSP00000349770-D1	3.69	1.27E-03	↓	interferon regulatory factor 5 isoform X1 [B. mutus]
ENSBTAP00000005713-D1	3.69	1.27E-03	↓	antithrombin-III [B. mutus]
ENSP00000352232-D1	3.69	1.27E-03	↓	SH2B adapter protein 1 isoform X1 [Bubalus bubalis]
ENSBTAP00000052350-D1	3.69	1.27E-03	↓	mitochondrial import inner membrane translocase subunit TIM14-like [B. mutus]
ENSBTAP00000043655-D1	3.70	1.56E-125	↓	transcription factor A, mitochondrial-like [B. mutus]
ENSBTAP00000005281-D1	3.71	2.40E-46	↓	Putative ATP-dependent RNA helicase DHX35, partial [B. mutus]
ENSP00000286688-D1	3.71	2.06E-23	↓	protein C8orf37 homolog [B. taurus]
ENSBTAP00000045797-D1	3.71	0.00E+00	↓	dual specificity protein kinase TTK [B. mutus]
ENSBTAP00000044884-D1	3.71	0.00E+00	↓	early endosome antigen 1 isoform X2 [Bison bison bison]
ENSBTAP00000033996-D1	3.72	7.53E-40	↓	4-trimethylaminobutyraldehyde dehydrogenase, partial [B. mutus]
ENSP00000356563-D1	3.72	4.70E-63	↓	peroxisomal biogenesis factor 3 [B. mutus]
ENSP00000348010-D1	3.72	3.77E-158	↓	pre-mRNA-processing factor 39 isoform X2 [B. taurus]
ENSBTAP00000007865-D1	3.73	5.65E-73	↓	sperm equatorial segment protein 1 precursor [B. taurus]
ENSP00000263026-D1	3.74	9.10E-10	↓	eukaryotic elongation factor 2 kinase [Bison bison bison]
ENSP00000227520-D1	3.74	9.10E-10	↓	coiled-coil domain-containing protein 86 [B. mutus]
ENSP00000359958-D1	3.75	0.00E+00	↓	zinc finger protein 265, isoform CRA_c [Homo sapiens]
ENSBTAP00000002406-D1	3.76	0.00E+00	↓	Glutathione S-transferase Mu 5 [B. mutus]
ENSBTAP00000002563-D1	3.77	1.13E-48	↓	G-substrate, partial [B. mutus]
ENSBTAP00000023750-D1	3.77	2.76E-293	↓	platelet glycoprotein 4-like isoform X1 [B. mutus]
ENSBTAP00000020116-D1	3.78	6.47E-15	↓	serine/threonine-protein kinase SIK3 isoform X1 [B. taurus]
ENSBTAP00000051331-D74	3.78	4.63E-10	↓	Transposon TX1 uncharacterized 149 kDa protein, partial [B. mutus]
ENSP00000349029-D1	3.79	3.38E-74	↓	inositol 1,4,5-trisphosphate receptor type 1 isoform X3 [Ovis aries]

ENSBTAP00000044021-D1	3.79	2.00E-27	↓	Tetratricopeptide repeat protein 23-like protein, partial [B. mutus]
ENSBTAP00000023273-D1	3.80	0.00E+00	↓	Myopalladin [B. mutus]
ENSP00000306482-D1	3.81	1.90E-25	↓	DENN domain-containing protein 5B, partial [B. mutus]
ENSBTAP00000007823-D1	3.82	1.70E-78	↓	band 4.1-like protein 2 isoform X10 [B. taurus]
ENSP00000368516-D1	3.82	9.97E-211	↓	Leucine-rich repeat-containing G-protein coupled receptor 4, partial [B. mutus]
yakG007946	3.83	3.49E-23	↓	Protein transport protein Sec24C [B. mutus]
ENSP00000360886-D1	3.83	3.85E-05	↓	ELAV (embryonic lethal, abnormal vision, Drosophila)-like 4 isoform X5 [Equus caballus]
ENSP00000265334-D1	3.83	3.85E-05	↓	cyclin-dependent kinase-like 3 [B. mutus]
ENSBTAP00000016300-D1	3.83	3.85E-05	↓	Aflatoxin B1 aldehyde reductase member 2, partial [B. mutus]
ENSP00000264249-D1	3.83	3.85E-05	↓	Carbohydrate sulfotransferase 10 [B. mutus]
ENSP00000358160-D3	3.83	3.85E-05	↓	histone H3.1-like [B. mutus]
ENSBTAP00000051025-D1	3.83	3.85E-05	↓	protein-cysteine N-palmitoyltransferase HHAT-like protein [B. mutus]
ENSP00000266987-D1	3.84	2.93E-36	↓	RISC-loading complex subunit TARBP2 [B. taurus]
ENSP00000379839-D1	3.84	4.96E-26	↓	Bifunctional UDP-N-acetylglucosamine 2-epimerase/N-acetylmannosamine kinase, partial [B. mutus]
ENSP00000362166-D1	3.84	2.67E-181	↓	chromatin modification-related protein MEAF6 isoform X1 [Trichechus manatus latirostris]
ENSBTAP00000008528-D1	3.86	7.90E-246	↓	Putative protein KIAA0586 [B. mutus]
ENSBTAP00000017653-D1	3.86	2.53E-26	↓	N-acetylglucosaminyl-phosphatidylinositol de-N-acetylase [B. mutus]
ENSP00000262384-D1	3.86	2.53E-26	↓	NEDD4-binding protein 1, partial [B. mutus]
ENSP00000376458-D1	3.86	2.53E-26	↓	Cell adhesion molecule-related/down-regulated by oncogenes [B. mutus]
ENSP00000374118-D1	3.87	9.08E-164	↓	Serine/threonine-protein kinase SMG1, partial [B. mutus]
ENSP00000382237-D1	3.87	1.19E-10	↓	transmembrane protein 26-like [B. mutus]
ENSP00000360382-D1	3.87	1.13E-87	↓	hypothetical protein M91_07816, partial [B. mutus]
ENSBTAP00000008412-D1	3.88	0.00E+00	↓	Leucine-rich repeat and IQ domain-containing protein 4, partial [B. mutus]
ENSBTAP00000039130-D1	3.88	4.39E-16	↓	DnaJ (Hsp40) homolog, subfamily C, member 5 [Capra hircus]
ENSBTAP00000023322-D1	3.89	1.85E-56	↓	FCH domain only protein 2, partial [B. mutus]
ENSBTAP00000015941-D1	3.89	1.03E-37	↓	Fibrous sheath-interacting protein 1, partial [B. mutus]
ENSP00000413073-D1	3.89	1.62E-13	↓	zinc finger protein 445 [B. mutus]
ENSBTAP00000053192-D1	3.90	4.85E-57	↓	frizzled-3 [B. mutus]
ENSP00000356713-D1	3.91	0.00E+00	↓	TPA: interferon gamma receptor 1 [B. taurus]
ENSP00000254029-D1	3.91	7.27E-66	↓	WD repeat-containing protein 44 isoform X1 [B. mutus]
ENSBTAP00000022552-D1	3.92	1.01E-93	↓	WD repeat and FYVE domain-containing protein 1, partial [B. mutus]
ENSBTAP00000014960-D1	3.92	3.61E-246	↓	Acid ceramidase, partial [B. mutus]
yakG025773	3.92	2.25E-80	↓	Neurexin-3-alpha [B. mutus]
ENSP00000308957-D1	3.92	8.25E-14	↓	Multiple C2 and transmembrane domain-containing protein 1, partial [B. mutus]
ENSBTAP00000006959-D1	3.93	1.14E-16	↓	calcium homeostasis endoplasmic reticulum protein [Ceratotherium simum simum]
ENSBTAP00000017907-D1	3.94	1.28E-36	↓	centrosomal protein of 97 kDa [B. mutus]
ENSBTAP00000041678-D1	3.95	2.30E-90	↓	LYR motif-containing protein 7, partial [B. mutus]
ENSBTAP00000002323-D1	3.95	5.82E-17	↓	modulator of retrovirus infection homolog [B. mutus]
ENSP00000354855-D1	3.95	4.19E-14	↓	Nuclear factor erythroid 2-related factor 1, partial [B. mutus]
ENSP00000222345-D1	3.95	2.33E-08	↓	Signal-induced proliferation-associated 1-like protein 3 [B. mutus]
ENSBTAP00000030443-D1	3.95	1.91E-05	↓	Receptor-type tyrosine-protein phosphatase O, partial [B. mutus]
ENSP00000337014-D1	3.95	1.91E-05	↓	Hemojuvelin [B. mutus]
ENSP00000355385-D1	3.95	1.91E-05	↓	hypothetical protein M91_15535, partial [B. mutus]
ENSP00000362235-D1	3.95	1.91E-05	↓	alpha-N-acetylgalactosaminide alpha-2,6-sialyltransferase 6 [B. taurus]
ENSP00000376946-D1	3.95	6.09E-04	↓	Bardet-Biedl syndrome 10 protein, partial [B. mutus]
ENSBTAP00000045122-D1	3.95	6.09E-04	↓	tissue factor pathway inhibitor [B. mutus]
ENSP00000282356-D1	3.95	6.09E-04	↓	calcium/calmodulin-dependent protein kinase type IV [Bison bison bison]
ENSBTAP00000052690-D1	3.95	6.09E-04	↓	Interleukin-23 receptor [B. mutus]
ENSBTAP00000031486-D11	3.95	6.09E-04	↓	hypothetical protein M91_01311, partial [B. mutus]
ENSBTAP00000002092-D2	3.95	6.09E-04	↓	.
ENSP00000283025-D1	3.95	6.09E-04	↓	tektin-5 [B. mutus]
ENSP00000362076-D1	3.95	6.09E-04	↓	Rhomboid-related protein 2, partial [B. mutus]
ENSBTAP00000011474-D1	3.95	6.09E-04	↓	Sulfotransferase 6B1, partial [B. mutus]
ENSP00000413405-D1	3.95	6.09E-04	↓	GPI mannosyltransferase 4, partial [B. mutus]
ENSP00000302251-D1	3.95	6.09E-04	↓	Homeobox protein GBX-2, partial [B. mutus]

ENSBTAP00000031488-D1	3.95	6.09E-04	↓	nucleoredoxin-like protein 2 [B. mutus]
ENSP00000389066-D1	3.95	6.09E-04	↓	Putative ATP-dependent RNA helicase DHX33, partial [B. mutus]
ENSP00000401653-D1	3.95	6.09E-04	↓	Dorsal root ganglia homeobox protein, partial [B. mutus]
ENSBTAP00000011978-D1	3.95	6.09E-04	↓	Lysyl oxidase-like protein 1 [B. mutus]
ENSBTAP00000006662-D1	3.95	6.09E-04	↓	glucagon-like peptide 2 receptor [B. mutus]
ENSBTAP00000045314-D1	3.95	6.09E-04	↓	neuropeptide Y receptor type 5 [B. taurus]
ENSBTAP00000035721-D65	3.95	6.09E-04	↓	hypothetical protein M91_16788, partial [B. mutus]
ENSP00000268699-D1	3.95	6.09E-04	↓	growth arrest-specific protein 8 [B. mutus]
ENSP00000264198-D1	3.95	6.09E-04	↓	mitochondrial ubiquitin ligase activator of NFKB 1 [B. mutus]
ENSP00000392188-D8	3.95	6.09E-04	↓	hypothetical protein M91_18372, partial [B. mutus]
ENSBTAP00000003394-D2	3.95	6.09E-04	↓	SAM domain-containing protein SAMSN-1, partial [B. mutus]
ENSP00000398350-D1	3.95	6.09E-04	↓	UPF0696 protein C11orf68 homolog [B. mutus]
ENSBTAP00000007806-D1	3.95	6.09E-04	↓	urokinase-type plasminogen activator [B. mutus]
ENSBTAP00000036358-D1	3.95	6.09E-04	↓	abhydrolase domain-containing protein 8 [B. taurus]
ENSP00000410076-D1	3.95	6.09E-04	↓	caspase-1-like [B. mutus]
ENSP00000340608-D1	3.95	6.09E-04	↓	Serine/threonine-protein kinase Sgk2, partial [B. mutus]
ENSP00000309087-D1	3.95	6.09E-04	↓	PLAC8-like protein 1 [B. taurus]
ENSBTAP00000046628-D1	3.95	6.09E-04	↓	pogo transposable element with KRAB domain [B. mutus]
ENSP00000312397-D1	3.95	6.09E-04	↓	Kelch-like protein 3, partial [B. mutus]
ENSP00000375413-D3	3.95	6.09E-04	↓	hypothetical protein M91_12914, partial [B. mutus]
ENSP00000339952-D1	3.95	6.09E-04	↓	Kinase suppressor of Ras 2, partial [B. mutus]
ENSBTAP00000008629-D1	3.95	6.09E-04	↓	seizure 6-like protein [B. mutus]
ENSBTAP00000021995-D1	3.95	6.09E-04	↓	Calcium/calmodulin-dependent protein kinase type 1G [B. mutus]
ENSBTAP00000006543-D1	3.95	6.09E-04	↓	Leucine-rich repeat-containing protein 24 [B. mutus]
ENSBTAP00000019129-D2	3.95	6.09E-04	↓	5'-AMP-activated protein kinase subunit beta-1 [B. mutus]
ENSBTAP00000023971-D1	3.95	6.09E-04	↓	leucine-, glutamate- and lysine-rich protein 1 [B. mutus]
ENSP00000322453-D8	3.95	6.09E-04	↓	Tripartite motif-containing protein 7 [B. mutus]
ENSBTAP00000016482-D1	3.95	6.09E-04	↓	Bcl-2-like protein 15, partial [B. mutus]
ENSBTAP00000023605-D1	3.95	6.09E-04	↓	Regulator of G-protein signaling 9 [B. mutus]
ENSBTAP00000020078-D1	3.95	6.09E-04	↓	corticosteroid 11-beta-dehydrogenase isozyme 1 [B. taurus]
ENSP00000401306-D1	3.95	6.09E-04	↓	fibrous sheath-interacting protein 2-like, partial [B. mutus]
ENSP00000388723-D1	3.95	6.09E-04	↓	kelch repeat and BTB domain-containing protein 13 [Bubalus bubalis]
ENSBTAP00000026019-D1	3.95	6.09E-04	↓	CKLF-like MARVEL transmembrane domain-containing protein 6, partial [B. mutus]
ENSBTAP00000023772-D1	3.95	6.09E-04	↓	Protein FAM173B, partial [B. mutus]
ENSBTAP00000040020-D1	3.95	6.09E-04	↓	Tetratricopeptide repeat protein GNN, partial [B. mutus]
ENSBTAP00000036815-D1	3.95	6.09E-04	↓	Multiple epidermal growth factor-like domains protein 11, partial [B. mutus]
ENSBTAP00000001845-D1	3.95	6.09E-04	↓	hypothetical protein M91_18164, partial [B. mutus]
ENSP00000210187-D1	3.95	6.09E-04	↓	Ras-related protein Rab-26, partial [B. mutus]
ENSBTAP00000023120-D1	3.95	6.09E-04	↓	LOC531467 protein [B. taurus]
ENSP00000289228-D1	3.95	6.09E-04	↓	beta-centractin [B. taurus]
ENSP00000217909-D1	3.95	6.09E-04	↓	Solute carrier family 25 member 43, partial [B. mutus]
ENSP00000309762-D1	3.95	6.09E-04	↓	Collagen alpha-5(VI) chain, partial [B. mutus]
ENSP00000376419-D1	3.95	6.09E-04	↓	kelch-like protein 23 [Bubalus bubalis]
ENSP00000420560-D1	3.95	6.09E-04	↓	leucine-rich repeat-containing protein 61 [B. mutus]
ENSP00000365392-D2	3.95	6.09E-04	↓	major histocompatibility class I related protein isoform X2 [B. taurus]
ENSBTAP00000016106-D1	3.95	6.09E-04	↓	probable G-protein coupled receptor 128 [B. mutus]
ENSBTAP00000019834-D1	3.95	6.09E-04	↓	hexokinase-3 [B. mutus]
ENSBTAP00000016639-D1	3.95	6.09E-04	↓	BEN domain-containing protein 3 [B. mutus]
ENSBTAP00000028257-D1	3.95	6.09E-04	↓	probable lipid phosphate phosphatase PPAPDC3 [B. mutus]
ENSP00000256324-D1	3.95	6.09E-04	↓	UPF0317 protein C14orf159 homolog, mitochondrial [Balaenoptera acutorostrata scammoni]
ENSBTAP00000030474-D27	3.95	6.09E-04	↓	Protocadherin gamma-A4, partial [B. mutus]
ENSP00000355089-D2	3.95	6.09E-04	↓	CUGBP Elav-like family member 3, partial [B. mutus]
ENSBTAP00000047510-D1	3.95	6.09E-04	↓	transmembrane protein 245 [Elephantulus edwardii]
ENSBTAP00000029126-D1	3.95	6.09E-04	↓	cadherin EGF LAG seven-pass G-type receptor 3 isoform X2 [B. taurus]
ENSP00000381597-D1	3.95	6.09E-04	↓	Putative G-protein coupled receptor 179 [B. mutus]

ENSBTAP00000050773-D76	3.95	6.09E-04	↓	hypothetical protein M91_00245, partial [B. mutus]
ENSBTAP00000003600-D1	3.95	6.09E-04	↓	Tyrosine-protein phosphatase non-receptor type 23 [B. mutus]
ENSBTAP00000018628-D1	3.95	6.09E-04	↓	Phosphoprotein associated with glycosphingolipid-enriched microdomains 1, partial [B. mutus]
ENSBTAP00000040546-D1	3.95	6.09E-04	↓	Putative beta-lactamase-like 1, partial [B. mutus]
ENSBTAP00000022295-D1	3.95	6.09E-04	↓	uncharacterized protein C7orf61 homolog [B. mutus]
ENSBTAP00000044787-D1	3.95	6.09E-04	↓	Protein FAM162B, partial [B. mutus]
ENSP00000404672-D1	3.95	6.09E-04	↓	Doublecortin domain-containing protein 5, partial [B. mutus]
ENSP00000328854-D1	3.95	6.09E-04	↓	nucleolar complex protein 4 homolog [B. mutus]
ENSP00000375952-D1	3.95	6.09E-04	↓	SPARC-related modular calcium-binding protein 2, partial [B. mutus]
ENSP00000363136-D1	3.95	6.09E-04	↓	G-protein coupled receptor 3, partial [B. mutus]
ENSBTAP00000001990-D1	3.95	6.09E-04	↓	protein RRNAD1 [B. mutus]
ENSBTAP00000004024-D1	3.95	6.09E-04	↓	testis-specific protein 10-interacting protein [B. mutus]
ENSP00000360635-D1	3.95	6.09E-04	↓	Dipeptidyl peptidase 2, partial [B. mutus]
ENSBTAP00000047305-D1	3.95	6.09E-04	↓	hypothetical protein M91_11706, partial [B. mutus]
ENSP00000382548-D1	3.95	6.09E-04	↓	hypothetical protein M91_19085, partial [B. mutus]
ENSBTAP00000053109-D3	3.95	6.09E-04	↓	Protocadherin beta-16, partial [B. mutus]
ENSBTAP00000006808-D1	3.95	6.09E-04	↓	Putative phospholipid-transporting ATPase ID [B. mutus]
ENSP00000340595-D1	3.95	6.09E-04	↓	G-protein coupled receptor family C group 5 member C, partial [B. mutus]
ENSP00000415299-D1	3.95	6.09E-04	↓	hypothetical protein M91_01548 [B. mutus]
ENSBTAP00000029309-D1	3.95	6.09E-04	↓	Transmembrane protein 18, partial [B. mutus]
ENSBTAP00000025071-D1	3.95	6.09E-04	↓	Leucine-rich repeat-containing protein 68, partial [B. mutus]
ENSBTAP00000051598-D38	3.95	6.09E-04	↓	Interferon tau-1, partial [B. mutus]
ENSBTAP00000051494-D1	3.95	6.09E-04	↓	hypothetical protein M91_17997, partial [B. mutus]
ENSBTAP00000045384-D1	3.95	6.09E-04	↓	TPA: melanoma antigen family B, 4 [B. taurus]
ENSBTAP00000027804-D1	3.95	6.09E-04	↓	Aromatic-L-amino-acid decarboxylase, partial [B. mutus]
ENSBTAP00000001648-D1	3.95	6.09E-04	↓	leukocyte cell-derived chemotaxin-2 [B. mutus]
ENSP00000278886-D1	3.95	6.09E-04	↓	ninein-like protein isoform X1 [B. taurus]
ENSP00000386435-D1	3.95	6.09E-04	↓	Protein FAM176A, partial [B. mutus]
ENSP00000282928-D1	3.95	6.09E-04	↓	zinc finger protein ZIC 1 [Trichechus manatus latirostris]
ENSBTAP00000046733-D5	3.95	6.09E-04	↓	histone H4-like [Oryctolagus cuniculus]
ENSBTAP00000011594-D1	3.95	6.09E-04	↓	ATP synthase mitochondrial F1 complex assembly factor 2 isoform X1 [Bison bison bison]
ENSBTAP00000011190-D1	3.95	6.09E-04	↓	hypothetical protein M91_10367, partial [B. mutus]
ENSBTAP00000008335-D1	3.95	6.09E-04	↓	Haptoglobin, partial [B. mutus]
ENSP00000215730-D1	3.95	6.09E-04	↓	Synaptosomal-associated protein 29, partial [B. mutus]
ENSBTAP00000023500-D2	3.95	6.09E-04	↓	Interferon-induced guanylate-binding protein 1, partial [B. mutus]
ENSP00000391968-D2	3.95	6.09E-04	↓	hypothetical protein LOC100428091 isoform 1 [Macaca mulatta]
ENSBTAP00000026744-D1	3.95	6.09E-04	↓	Canalicular multispecific organic anion transporter 2, partial [B. mutus]
ENSP00000311030-D1	3.95	6.09E-04	↓	Multidrug resistance-associated protein 9 [B. mutus]
ENSP00000362160-D2	3.95	6.09E-04	↓	calcium-binding mitochondrial carrier protein SCaMC-3 [B. mutus]
ENSP00000273600-D1	3.95	6.09E-04	↓	Dynein heavy chain 1, axonemal [B. mutus]
ENSBTAP00000013455-D12	3.95	6.09E-04	↓	histone H4-like [Bison bison bison]
ENSP00000405932-D1	3.95	6.09E-04	↓	Prospero homeobox protein 2, partial [B. mutus]
ENSBTAP00000049695-D1	3.95	6.09E-04	↓	Tumor necrosis factor receptor superfamily member 26, partial [B. mutus]
ENSBTAP00000007726-D1	3.95	6.09E-04	↓	protein FAM83A [B. mutus]
ENSP00000407097-D1	3.95	6.09E-04	↓	uncharacterized protein LOC105607708 [Ovis aries musimon]
ENSP00000309338-D1	3.95	6.09E-04	↓	Cardiotrophin-like cytokine factor 1, partial [B. mutus]
ENSBTAP00000025343-D1	3.95	6.09E-04	↓	Putative phospholipase B-like 2, partial [B. mutus]
ENSBTAP00000002385-D1	3.95	6.09E-04	↓	Solute carrier family 2, facilitated glucose transporter member 6 [B. mutus]
ENSP00000264833-D3	3.95	6.09E-04	↓	Noelin [B. mutus]
ENSP00000413517-D1	3.95	6.09E-04	↓	putative uncharacterized protein FLJ13197-like [Tursiops truncatus]
ENSBTAP00000051671-D48	3.95	6.09E-04	↓	hypothetical protein M91_03766 [B. mutus]
ENSP00000274853-D1	3.95	6.09E-04	↓	Phostensin [B. mutus]
ENSBTAP00000025829-D8	3.95	6.09E-04	↓	Sodium channel protein type 7 subunit alpha, partial [B. mutus]
ENSP00000307911-D1	3.95	6.09E-04	↓	Carbohydrate sulfotransferase 2 [Mus musculus]
ENSP00000366918-D1	3.95	6.09E-04	↓	protein FAM220A [Bubalus bubalis]

ENSP00000266068-D1	3.95	6.09E-04	↓	glucocorticoid modulatory element-binding protein 2 [B. mutus]
ENSBTAP00000043988-D1	3.95	6.09E-04	↓	hypothetical protein M91_12651 [B. mutus]
ENSP00000360727-D1	3.95	6.09E-04	↓	rab-like protein 6 isoform X2 [Ovis aries musimon]
ENSBTAP00000044019-D1	3.95	6.09E-04	↓	Cadherin-related family member 3, partial [B. mutus]
ENSP00000367059-D1	3.95	6.09E-04	↓	Espin, partial [B. mutus]
ENSP00000396157-D3	3.95	6.09E-04	↓	sialic acid-binding Ig-like lectin 8 [B. mutus]
ENSP00000371594-D1	3.95	6.09E-04	↓	guanine nucleotide-binding protein G(I)/G(S)/G(O) subunit gamma-7 isoform 1 [Canis lupus familiaris]
ENSBTAP00000002297-D1	3.95	6.09E-04	↓	Plasma alpha-L-fucosidase, partial [B. mutus]
ENSP00000393631-D1	3.95	6.09E-04	↓	putative adrenomedullin-5-like protein [Pantholops hodgsonii]
ENSBTAP00000048404-D1	3.95	6.09E-04	↓	THAP domain-containing protein 10 [B. mutus]
ENSP00000264613-D2	3.95	6.09E-04	↓	Ceruloplasmin [B. mutus]
ENSBTAP00000039689-D1	3.95	6.09E-04	↓	pleckstrin homology domain-containing family G member 3 isoform X2 [B. taurus]
ENSP00000396145-D1	3.95	6.09E-04	↓	Serine hydrolase-like protein, partial [B. mutus]
ENSBTAP00000010748-D1	3.95	6.09E-04	↓	Putative potassium channel regulatory protein [B. mutus]
ENSBTAP00000019352-D1	3.95	6.09E-04	↓	H2.0-like homeobox [B. mutus]
ENSP00000343507-D1	3.95	6.09E-04	↓	transmembrane protein 198 isoform X1 [B. mutus]
ENSBTAP00000029925-D9	3.95	6.09E-04	↓	ankyrin repeat domain-containing protein 26-like isoform X1 [B. taurus]
ENSP00000372295-D1	3.95	6.09E-04	↓	gap junction beta-2 protein [B. mutus]
ENSP00000329632-D1	3.95	6.09E-04	↓	F-box/LRR-repeat protein 7 isoform X2 [B. taurus]
ENSBTAP00000035843-D1	3.95	6.09E-04	↓	Tight junction-associated protein 1 [B. mutus]
ENSP00000359019-D1	3.95	6.09E-04	↓	pituitary homeobox 3 [B. mutus]
ENSBTAP00000005411-D1	3.95	6.09E-04	↓	hypothetical protein M91_04936, partial [B. mutus]
ENSBTAP00000024319-D1	3.95	6.09E-04	↓	putative sodium-coupled neutral amino acid transporter 10 [B. mutus]
yakG017852	3.95	6.09E-04	↓	Histone H4 [Heterocephalus glaber]
ENSBTAP00000050430-D88	3.95	6.09E-04	↓	hypothetical protein M91_10569, partial [B. mutus]
ENSBTAP00000020613-D33	3.95	6.09E-04	↓	hypothetical protein M91_04723, partial [B. mutus]
ENSP00000285402-D1	3.95	6.09E-04	↓	outer dense fiber protein 1 [B. mutus]
ENSBTAP00000034269-D2	3.95	6.09E-04	↓	hypothetical protein CB1_001402132 [Camelus ferus]
ENSBTAP00000022347-D1	3.95	6.09E-04	↓	Serine/threonine-protein kinase/endoribonuclease IRE1, partial [B. mutus]
ENSP00000393043-D1	3.95	6.09E-04	↓	ankyrin and armadillo repeat-containing protein isoform X1 [B. mutus]
ENSBTAP00000000626-D1	3.95	6.09E-04	↓	hypothetical protein M91_16804, partial [B. mutus]
ENSBTAP00000004038-D7	3.95	6.09E-04	↓	hypothetical protein M91_12045, partial [B. mutus]
ENSP00000407396-D1	3.95	6.09E-04	↓	Transmembrane protein 40, partial [B. mutus]
ENSP00000395882-D1	3.95	6.09E-04	↓	UPF0488 protein C8orf33 homolog isoform X2 [B. taurus]
yakG002415	3.95	6.09E-04	↓	hypothetical protein M91_01478 [B. mutus]
ENSP00000347140-D1	3.95	6.09E-04	↓	Asialoglycoprotein receptor 2 [B. mutus]
ENSP00000290953-D1	3.95	6.09E-04	↓	agouti-related protein isoform X1 [B. taurus]
ENSP00000313921-D1	3.95	6.09E-04	↓	Peptide methionine sulfoxide reductase, partial [B. mutus]
ENSP00000266718-D1	3.96	0.00E+00	↓	lumican [B. mutus]
ENSP00000347087-D1	3.96	1.80E-140	↓	Putative ATP-dependent RNA helicase DDX4, partial [B. mutus]
ENSBTAP00000044097-D1	3.97	0.00E+00	↓	TATA element modulatory factor, partial [B. mutus]
ENSBTAP00000052447-D1	3.97	3.49E-43	↓	MAP4K1 protein [B. taurus]
ENSP00000271657-D1	3.98	8.46E-32	↓	Phosphatidylinositol 4-kinase beta, partial [B. mutus]
ENSP00000380692-D1	3.98	1.87E-84	↓	Lysine-specific demethylase 7, partial [B. mutus]
ENSBTAP00000032331-D1	3.99	0.00E+00	↓	Soma ferritin [B. mutus]
ENSP00000317872-D1	3.99	0.00E+00	↓	E3 ubiquitin-protein ligase RBBP6 isoform X1 [B. mutus]
ENSP00000300291-D1	3.99	0.00E+00	↓	cleavage and polyadenylation specificity factor subunit 5 [Homo sapiens]
ENSP00000354611-D1	3.99	1.57E-11	↓	pleckstrin homology-like domain family B member 1 [B. mutus]
ENSBTAP00000042180-D1	4.00	3.74E-94	↓	scaffold attachment factor B2 [Orcinus orca]
ENSBTAP00000007115-D1	4.00	0.00E+00	↓	dual specificity protein kinase CLK1 isoform X1 [B. taurus]
ENSP00000378945-D1	4.01	1.08E-20	↓	cGMP-dependent protein kinase 2-like, partial [B. mutus]
ENSP00000379140-D1	4.01	1.17E-08	↓	stromal cell-derived factor 1 isoform X1 [Odobenus rosmarus divergens]
yakG001435	4.02	0.00E+00	↓	Testicular haploid expressed protein [B. mutus]
ENSP00000304713-D1	4.03	0.00E+00	↓	myb/SANT-like DNA-binding domain-containing protein 4 [B. taurus]
ENSBTAP00000003210-D1	4.03	3.19E-45	↓	Mitotic spindle assembly checkpoint protein MAD1 [B. mutus]

ENSP00000217971-D1	4.03	7.94E-12	↓	membrane-associated progesterone receptor component 1-like isoform X1 [B. mutus]
ENSP00000344426-D1	4.04	5.29E-125	↓	hypothetical protein M91_19343, partial [B. mutus]
ENSBTAP00000034574-D1	4.04	2.13E-36	↓	Nuclear pore complex protein Nup85, partial [B. mutus]
ENSP00000253382-D1	4.04	5.53E-15	↓	Acetyl-coenzyme A synthetase, cytoplasmic [B. mutus]
ENSBTAP00000012520-D1	4.04	1.64E-226	↓	peptidylprolyl isomerase domain and WD repeat-containing protein 1 isoform X1 [B. mutus]
ENSBTAP00000008920-D1	4.05	7.40E-132	↓	coiled-coil domain-containing protein 174 [B. mutus]
ENSBTAP00000009732-D1	4.06	2.18E-46	↓	AN1-type zinc finger protein 2A, partial [B. mutus]
ENSP00000373214-D1	4.07	2.00E-18	↓	Calcium-dependent secretion activator 1, partial [Heterocephalus glaber]
ENSP00000379108-D1	4.07	5.96E-09	↓	Putative bifunctional methylenetetrahydrofolate dehydrogenase/cyclohydrolase 2, partial [B. mutus]
ENSP00000348551-D1	4.07	5.96E-09	↓	nuclear receptor corepressor 2, partial [B. mutus]
ENSBTAP00000023600-D1	4.07	5.96E-09	↓	Xin actin-binding repeat-containing protein 2 [B. mutus]
ENSBTAP00000019092-D1	4.07	9.52E-06	↓	Draxin, partial [B. mutus]
ENSP00000351280-D100	4.07	9.52E-06	↓	zinc finger protein 546 [B. mutus]
ENSBTAP00000000179-D1	4.07	9.52E-06	↓	galectin-related protein [Panthera tigris altaica]
ENSBTAP00000001004-D1	4.07	9.52E-06	↓	Zinc finger RNA-binding protein 2, partial [B. mutus]
ENSP00000320821-D1	4.07	9.52E-06	↓	kinesin-like protein KIF1C [Bubalus bubalis]
ENSBTAP00000020395-D1	4.07	5.26E-94	↓	Spectrin alpha chain, brain [B. mutus]
ENSBTAP00000010802-D1	4.08	1.98E-182	↓	Ribonucleoside-diphosphate reductase subunit M2 [B. mutus]
ENSBTAP00000000024-D1	4.08	0.00E+00	↓	nucleoporin Nup37 [B. mutus]
ENSP00000398181-D1	4.09	5.29E-25	↓	Alpha-ketoglutarate-dependent dioxygenase alkB-like protein 2 [B. mutus]
ENSP00000357453-D1	4.09	3.95E-44	↓	mannosyl-oligosaccharide 1,2-alpha-mannosidase IA [Bison bison bison]
ENSP00000347672-D1	4.09	7.33E-22	↓	vang-like protein 1 [Capra hircus]
ENSBTAP00000011675-D1	4.10	1.43E-15	↓	neutral alpha-glucosidase C [B. mutus]
ENSBTAP00000002852-D1	4.10	8.11E-64	↓	NADH dehydrogenase [ubiquinone] iron-sulfur protein 2, mitochondrial, partial [B. mutus]
ENSBTAP00000001589-D1	4.10	2.12E-147	↓	28S ribosomal protein S29, mitochondrial, partial [B. mutus]
ENSBTAP00000000096-D1	4.10	0.00E+00	↓	estradiol 17-beta-dehydrogenase 12 [B. mutus]
ENSP00000420502-D17	4.11	2.05E-12	↓	hypothetical protein M91_21146 [B. mutus]
ENSP00000409153-D1	4.12	1.38E-25	↓	AP-1 complex subunit gamma-1, partial [B. mutus]
ENSBTAP00000019358-D1	4.12	7.29E-16	↓	xyloside xylosyltransferase 1 [Pantholops hodgsonii]
ENSBTAP00000017150-D1	4.12	3.01E-09	↓	ADP-ribosylation factor-like protein 8B [Homo sapiens]
ENSP00000388613-D1	4.12	3.01E-09	↓	gamma-parvin [B. mutus]
ENSBTAP00000049725-D1	4.13	0.00E+00	↓	Cone cGMP-specific 3',5'-cyclic phosphodiesterase subunit alpha' [B. mutus]
ENSP00000327538-D85	4.14	1.65E-125	↓	Zinc finger protein 283, partial [B. mutus]
ENSP00000377823-D1	4.14	2.58E-29	↓	Protein NDRG4-A, partial [B. mutus]
ENSP00000405963-D1	4.14	1.04E-12	↓	ras-specific guanine nucleotide-releasing factor 1 isoform X1 [B. mutus]
ENSP00000339328-D1	4.14	0.00E+00	↓	urokinase plasminogen activator surface receptor [B. mutus]
ENSBTAP00000003529-D1	4.15	1.38E-59	↓	HORMA domain-containing protein 1 isoform X1 [B. mutus]
ENSBTAP00000010275-D1	4.15	6.05E-207	↓	cx9C motif-containing protein 4 isoform X1 [Capra hircus]
ENSP00000323271-D11	4.15	1.04E-86	↓	hypothetical protein M91_20748, partial [B. mutus]
ENSBTAP00000040311-D1	4.16	4.93E-57	↓	Holliday junction recognition protein isoform X2 [B. taurus]
ENSP00000399258-D1	4.18	9.13E-51	↓	Laminin subunit alpha-3, partial [B. mutus]
ENSBTAP00000032097-D1	4.18	1.90E-16	↓	Transmembrane emp24 domain-containing protein 5, partial [B. mutus]
ENSBTAP00000013323-D1	4.18	1.90E-16	↓	Mediator of RNA polymerase II transcription subunit 20, partial [B. mutus]
ENSP00000406027-D1	4.18	1.53E-09	↓	EPM2A-interacting protein 1 [B. taurus]
ENSBTAP00000014770-D1	4.18	1.53E-09	↓	H(+)/Cl(-) exchange transporter 4, partial [B. mutus]
ENSBTAP00000024595-D1	4.18	4.75E-06	↓	kelch repeat and BTB domain-containing protein 4 isoform X1 [B. mutus]
ENSBTAP00000027076-D3	4.18	4.75E-06	↓	AP-1 complex subunit beta-1 [B. mutus]
ENSP00000307479-D1	4.18	4.75E-06	↓	aryl hydrocarbon receptor nuclear translocator 2 isoform X1 [Bison bison bison]
ENSP00000358155-D1	4.18	4.75E-06	↓	histone H2A type 1-like [Cavia porcellus]
ENSBTAP00000034991-D3	4.18	4.75E-06	↓	Zinc finger protein 322A, partial [B. mutus]
ENSBTAP00000010506-D1	4.18	4.75E-06	↓	hyaluronan synthase 3 [B. taurus]
ENSBTAP00000032393-D1	4.18	4.75E-06	↓	Liver carboxylesterase, partial [B. mutus]
ENSP00000220509-D1	4.18	2.96E-04	↓	vacuolar protein sorting-associated protein 18 homolog [B. taurus]
ENSBTAP00000042213-D5	4.18	2.96E-04	↓	hypothetical protein M91_00741, partial [B. mutus]
ENSBTAP00000031486-D10	4.18	2.96E-04	↓	hypothetical protein M91_16502, partial [B. mutus]

ENSP00000407031-D1	4.18	2.96E-04	↓	NUT family member 1 [B. mutus]
ENSP00000320869-D1	4.18	2.96E-04	↓	E3 ubiquitin-protein ligase TRIM41 [Bubalus bubalis]
ENSP00000351040-D1	4.18	2.96E-04	↓	protein FAM217B [B. mutus]
ENSBTAP00000001766-D1	4.18	2.96E-04	↓	protein Wnt-5b isoform X1 [B. taurus]
ENSP00000362238-D1	4.18	2.96E-04	↓	Protein POF1B, partial [B. mutus]
ENSP00000382434-D1	4.18	2.96E-04	↓	cytosolic phospholipase A2 epsilon [B. mutus]
ENSP00000403701-D1	4.18	2.96E-04	↓	beta-arrestin-2 isoform X1 [Bubalus bubalis]
ENSP00000317579-D1	4.18	2.96E-04	↓	UPF0420 protein C16orf58 homolog, partial [B. mutus]
ENSP00000260599-D1	4.18	2.96E-04	↓	Ketohexokinase [B. mutus]
ENSP00000377567-D3	4.18	2.96E-04	↓	keratin, type I microfibrillar, 47.6 kDa-like [B. mutus]
ENSP00000300134-D1	4.18	2.96E-04	↓	Signal transducer and activator of transcription 6 [B. mutus]
ENSP00000412272-D1	4.18	2.96E-04	↓	TPA: Putative uncharacterized protein C10orf41-like [B. taurus]
ENSP00000040584-D1	4.18	2.96E-04	↓	homeobox protein Hox-C8 [Trichechus manatus latirostris]
ENSP00000332706-D1	4.18	2.96E-04	↓	transcriptional activator protein Pur-alpha isoform X3 [Sus scrofa]
ENSBTAP00000021621-D1	4.18	2.96E-04	↓	Glucose-6-phosphatase 3, partial [B. mutus]
ENSP00000378001-D1	4.18	2.96E-04	↓	bifunctional heparan sulfate N-deacetylase/N-sulfotransferase 3-like [B. mutus]
ENSBTAP00000017105-D59	4.18	2.96E-04	↓	zinc finger protein 83-like [B. mutus]
ENSP00000389716-D1	4.18	2.96E-04	↓	unnamed protein product [Homo sapiens]
ENSP00000392188-D5	4.18	2.96E-04	↓	hypothetical protein M91_20590, partial [B. mutus]
ENSP00000334996-D1	4.18	2.96E-04	↓	calicin [B. mutus]
ENSBTAP00000017244-D1	4.18	2.96E-04	↓	E3 ubiquitin-protein ligase RNF167, partial [B. mutus]
ENSP00000338283-D1	4.18	2.96E-04	↓	neuronal PAS domain-containing protein 2 [B. mutus]
ENSP00000284425-D2	4.18	2.96E-04	↓	ATP-binding cassette sub-family A member 10 [B. mutus]
ENSBTAP00000013207-D1	4.18	2.96E-04	↓	DnaJ-like protein subfamily A member 3, mitochondrial, partial [B. mutus]
ENSP00000311648-D1	4.18	2.96E-04	↓	2-hydroxyacylsphingosine 1-beta-galactosyltransferase-like [B. mutus]
ENSP00000334547-D1	4.18	2.96E-04	↓	T-cell acute lymphocytic leukemia protein 2 [B. mutus]
ENSBTAP00000002745-D1	4.18	2.96E-04	↓	myosin-IIIa [B. mutus]
ENSP00000354571-D2	4.18	2.96E-04	↓	RING finger protein 175, partial [B. mutus]
ENSBTAP00000040444-D1	4.18	2.96E-04	↓	Retina-specific copper amine oxidase [B. mutus]
ENSBTAP00000004485-D1	4.18	2.96E-04	↓	Cyclic AMP-dependent transcription factor ATF-5 [B. mutus]
yakA25805	4.18	2.96E-04	↓	Lipopolysaccharide-binding protein [B. mutus]
ENSP00000379326-D1	4.18	2.96E-04	↓	sodium-coupled monocarboxylate transporter 2 [B. mutus]
ENSBTAP00000045382-D1	4.18	2.96E-04	↓	agouti-signaling protein-like [B. mutus]
ENSP00000284770-D1	4.18	2.96E-04	↓	PDZ and LIM domain protein 3 isoform X1 [B. taurus]
ENSP00000245323-D1	4.18	2.96E-04	↓	ephrin-B2, partial [B. mutus]
ENSBTAP00000050194-D3	4.18	2.96E-04	↓	hypothetical protein M91_17975 [B. mutus]
ENSBTAP00000001727-D1	4.18	2.96E-04	↓	myeloid-associated differentiation marker-like [B. mutus]
ENSBTAP00000001704-D2	4.18	2.96E-04	↓	Inhibitor of carbonic anhydrase, partial [B. mutus]
ENSP00000356939-D1	4.18	2.96E-04	↓	Low affinity immunoglobulin gamma Fc region receptor II [B. mutus]
ENSP00000408285-D1	4.18	2.96E-04	↓	Serine/threonine-protein kinase D2 [B. mutus]
ENSP00000393444-D1	4.18	2.96E-04	↓	protein PAPPAS-like [Macaca mulatta]
ENSBTAP00000013931-D1	4.18	2.96E-04	↓	Thioredoxin domain-containing protein 8 [B. mutus]
ENSBTAP00000018383-D1	4.18	2.96E-04	↓	Serine/threonine-protein kinase receptor R3 [B. mutus]
ENSP00000410106-D1	4.18	2.96E-04	↓	Tenascin-R, partial [B. mutus]
ENSBTAP00000047173-D19	4.18	2.96E-04	↓	PRAME family member 12 isoform X2 [B. taurus]
ENSBTAP00000008159-D1	4.18	2.96E-04	↓	Lysyl oxidase-like protein 2 [B. mutus]
ENSBTAP00000029144-D1	4.18	2.96E-04	↓	Negative elongation factor B, partial [B. mutus]
ENSP00000416515-D1	4.18	2.96E-04	↓	NADPH:adrenodoxin oxidoreductase, mitochondrial, partial [B. mutus]
ENSP00000354612-D1	4.18	2.96E-04	↓	Prostaglandin G/H synthase 1, partial [B. mutus]
ENSBTAP00000051439-D1	4.18	2.96E-04	↓	interferon-induced very large GTPase 1-like isoform X2 [B. taurus]
ENSBTAP00000028636-D1	4.18	2.96E-04	↓	tetraspanin-18 [B. mutus]
ENSP00000369252-D1	4.18	2.96E-04	↓	leucine-rich repeat, immunoglobulin-like domain and transmembrane domain-containing protein 3 [B. mutus]
ENSBTAP00000047450-D1	4.18	2.96E-04	↓	putative ribosome-binding factor A, mitochondrial [B. mutus]
ENSBTAP00000007333-D1	4.18	2.96E-04	↓	Protein SZT2, partial [B. mutus]
ENSBTAP00000035049-D1	4.18	2.96E-04	↓	Serine/threonine-protein kinase 32A, partial [B. mutus]

ENSBTAP00000002156-D1	4.18	2.96E-04	↓	protein AAR2 homolog isoform X1 [B. taurus]
ENSBTAP00000006787-D6	4.18	2.96E-04	↓	hypothetical protein M91_20047, partial [B. mutus]
ENSBTAP00000014630-D45	4.18	2.96E-04	↓	mariner transposase [Homo sapiens]
ENSP00000317818-D1	4.18	2.96E-04	↓	PILR alpha-associated neural protein precursor [B. taurus]
ENSBTAP00000048923-D1	4.18	2.96E-04	↓	hypothetical protein M91_18609, partial [B. mutus]
ENSBTAP00000008662-D1	4.18	2.96E-04	↓	olfactory receptor 5W2 [B. taurus]
ENSBTAP00000002589-D1	4.18	2.96E-04	↓	HEAT repeat-containing protein 7B2, partial [B. mutus]
ENSP00000389912-D11	4.18	2.96E-04	↓	hypothetical protein M91_13311, partial [B. mutus]
ENSBTAP00000030100-D1	4.18	2.96E-04	↓	Integral membrane protein GPR137, partial [B. mutus]
ENSBTAP00000044692-D2	4.18	2.96E-04	↓	Solute carrier family 35 member F2, partial [B. mutus]
ENSBTAP00000017815-D1	4.18	2.96E-04	↓	Protein FAM57A, partial [B. mutus]
ENSP00000381797-D1	4.18	2.96E-04	↓	zinc finger protein DPF3 isoform X1 [B. taurus]
ENSBTAP00000016978-D1	4.18	2.96E-04	↓	histone H2B subacrosomal variant-like [B. mutus]
ENSBTAP00000034822-D2	4.18	2.96E-04	↓	solute carrier family 22 member 9 [B. mutus]
ENSP00000325827-D1	4.18	2.96E-04	↓	protein transport protein Sec16A [B. mutus]
ENSP00000365343-D15	4.18	2.96E-04	↓	uncharacterized protein LOC102265782 [B. mutus]
ENSP00000377347-D1	4.18	2.96E-04	↓	unnamed protein product [Homo sapiens]
ENSP00000290776-D1	4.18	2.96E-04	↓	copine-2 [B. mutus]
ENSP00000369121-D1	4.18	2.96E-04	↓	tectonin beta-propeller repeat-containing protein 1 [B. mutus]
ENSP00000357835-D1	4.18	2.96E-04	↓	Phospholysine phosphohistidine inorganic pyrophosphate phosphatase, partial [B. mutus]
ENSBTAP00000040897-D1	4.18	2.96E-04	↓	Fibronectin type III domain-containing protein 5, partial [B. mutus]
ENSP00000351657-D16	4.18	2.96E-04	↓	Zinc finger protein 256 [B. mutus]
ENSBTAP00000029367-D1	4.18	2.96E-04	↓	guanine nucleotide-binding protein subunit beta-like protein 1 [B. mutus]
ENSBTAP00000034409-D3	4.18	2.96E-04	↓	hypothetical protein M91_13901, partial [B. mutus]
ENSBTAP00000033741-D1	4.18	2.96E-04	↓	Rap guanine nucleotide exchange factor 5, partial [B. mutus]
ENSBTAP00000038763-D1	4.18	2.96E-04	↓	hypothetical protein M91_03153, partial [B. mutus]
yakG044571	4.18	2.96E-04	↓	hypothetical protein M91_15201 [B. mutus]
ENSBTAP00000023918-D3	4.18	2.96E-04	↓	histone H2B type F-M-like [B. mutus]
ENSP00000320232-D1	4.18	2.96E-04	↓	hypothetical protein M91_15732 [B. mutus]
ENSBTAP00000051584-D52	4.18	2.96E-04	↓	endogenous retrovirus group K member 11 Pol protein-like [Bison bison bison]
ENSBTAP00000028950-D1	4.18	2.96E-04	↓	Poly(ADP-ribose) glycohydrolase ARH3, partial [B. mutus]
ENSP00000240587-D1	4.18	2.96E-04	↓	Teashirt-like protein 3, partial [B. mutus]
ENSP00000378350-D1	4.18	2.96E-04	↓	cell division cycle protein 23 homolog isoform X1 [Ovis aries]
ENSP00000350265-D1	4.18	2.96E-04	↓	ectonucleotide pyrophosphatase/phosphodiesterase family member 3 [B. mutus]
ENSBTAP00000042880-D2	4.18	2.96E-04	↓	RNA-binding motif protein, X chromosome-like [B. mutus]
ENSBTAP00000006851-D1	4.18	2.96E-04	↓	fibroblast growth factor 1 [B. taurus]
ENSBTAP00000005525-D1	4.18	2.96E-04	↓	Mitogen-activated protein kinase kinase kinase 2, partial [B. mutus]
ENSP00000365838-D1	4.18	2.96E-04	↓	divergent paired-related homeobox-like [B. taurus]
ENSBTAP00000019510-D1	4.18	2.96E-04	↓	Protein spinster-like protein 1, partial [B. mutus]
ENSP00000332204-D1	4.18	2.96E-04	↓	semaphorin-4B [B. mutus]
ENSBTAP00000045355-D1	4.18	2.96E-04	↓	fragile X mental retardation 1 neighbor protein [B. mutus]
ENSBTAP00000020308-D1	4.18	2.96E-04	↓	N-sulphoglucosamine sulphohydrolase [B. mutus]
ENSBTAP00000031650-D9	4.18	2.96E-04	↓	multidrug resistance-associated protein 4-like isoform X1 [B. taurus]
ENSP00000419739-D1	4.18	2.96E-04	↓	Mitochondrial carrier-like protein 1, partial [B. mutus]
yakG035477	4.18	2.96E-04	↓	General transcription factor II-I repeat domain-containing protein 1 [B. mutus]
ENSP00000407761-D1	4.18	2.96E-04	↓	acyl-coenzyme A oxidase-like protein isoform X3 [Bison bison bison]
ENSBTAP00000036773-D3	4.18	2.96E-04	↓	40S ribosomal protein S29-like [B. mutus]
ENSP00000358795-D1	4.18	2.96E-04	↓	neuralized-like protein 1A [B. mutus]
ENSBTAP00000010607-D1	4.18	2.96E-04	↓	vacuolar protein sorting 37 homolog C (S. cerevisiae) [B. mutus]
ENSP00000406145-D1	4.18	2.96E-04	↓	cortexin-2 [B. taurus]
ENSBTAP00000007733-D1	4.18	2.96E-04	↓	zinc finger protein 333 isoform X1 [B. taurus]
ENSP00000392188-D15	4.18	2.96E-04	↓	hypothetical protein M91_04027, partial [B. mutus]
ENSP00000307235-D1	4.18	2.96E-04	↓	Eukaryotic translation initiation factor 2-alpha kinase 3, partial [B. mutus]
ENSBTAP00000047176-D1	4.18	2.96E-04	↓	FCH domain only protein 1, partial [B. mutus]
ENSP00000404456-D1	4.18	2.96E-04	↓	MAP7 domain-containing protein 1-like [Bubalus bubalis]

ENSBTAP00000030479-D16	4.18	2.96E-04	↓	Chain A, Crystal Structure Of Up1 Complexed With D(Ttagggttag(6-Mi) G); A Human Telomeric Repeat Containing 6-Methyl-8-(2- Deoxy-Beta-Ribofuranosyl)isoxanthopteridine (6-Mi)
ENSBTAP00000012208-D1	4.18	2.96E-04	↓	CD226 antigen, partial [B. mutus]
ENSP00000381364-D1	4.18	2.96E-04	↓	calcineurin-binding protein cabin-1 isoform X6 [B. taurus]
ENSBTAP00000015657-D11	4.18	2.96E-04	↓	hypothetical protein M91_02768, partial [B. mutus]
ENSP00000294664-D1	4.18	2.96E-04	↓	WD repeat-containing protein 63 [B. mutus]
ENSBTAP00000009779-D1	4.18	2.96E-04	↓	tektin-1-like [B. mutus]
ENSP00000359629-D1	4.18	2.96E-04	↓	Cartilage acidic protein 1, partial [B. mutus]
ENSP00000379065-D1	4.18	2.96E-04	↓	Zinc finger transcription factor Trps1, partial [B. mutus]
ENSP00000339971-D1	4.18	2.96E-04	↓	protein MROH8 isoform X2 [B. taurus]
ENSBTAP00000020990-D1	4.18	2.96E-04	↓	sigma non-opioid intracellular receptor 1 isoform X2 [Ovis aries musimon]
ENSBTAP00000017835-D2	4.18	2.96E-04	↓	cysteine and glycine-rich protein 1 [B. taurus]
ENSBTAP00000038737-D1	4.18	2.96E-04	↓	Janus kinase and microtubule-interacting protein 3, partial [B. mutus]
ENSP00000299824-D1	4.18	2.96E-04	↓	protein phosphatase 1 regulatory inhibitor subunit 16B [B. taurus]
ENSP00000402649-D1	4.18	2.96E-04	↓	hypothetical protein M91_07472, partial [B. mutus]
ENSP00000399221-D2	4.18	2.96E-04	↓	Rpl13 [B. taurus]
ENSBTAP00000000491-D1	4.18	2.96E-04	↓	Spexin, partial [B. mutus]
ENSBTAP00000006601-D1	4.18	2.96E-04	↓	Sideroflexin-3, partial [B. mutus]
ENSBTAP00000001635-D1	4.18	2.96E-04	↓	cell surface glycoprotein CD200 receptor 1-like [B. mutus]
ENSBTAP00000009888-D1	4.18	2.96E-04	↓	group XV phospholipase A2 [B. mutus]
ENSP00000372199-D1	4.18	2.96E-04	↓	nucleolar pre-ribosomal-associated protein 1 [B. mutus]
ENSP00000345031-D1	4.18	2.96E-04	↓	nuclear GTPase SLIP-GC [B. mutus]
ENSBTAP00000052162-D1	4.18	2.96E-04	↓	Interleukin-2 receptor subunit alpha, partial [B. mutus]
ENSBTAP00000051204-D1	4.18	2.96E-04	↓	calcium/calmodulin-dependent 3',5'-cyclic nucleotide phosphodiesterase 1A isoform X1 [B. mutus]
yakG038584	4.18	2.96E-04	↓	hypothetical protein M91_04970 [B. mutus]
ENSP00000375470-D1	4.18	2.96E-04	↓	hypothetical protein M91_11983, partial [B. mutus]
ENSBTAP00000051011-D15	4.18	2.96E-04	↓	histone H2B type 1-N-like [B. mutus]
ENSP00000353654-D1	4.18	2.96E-04	↓	Collagen alpha-2(IV) chain, partial [B. mutus]
ENSBTAP00000033956-D1	4.18	2.96E-04	↓	high affinity immunoglobulin epsilon receptor subunit gamma precursor [B. taurus]
ENSBTAP00000019925-D1	4.18	2.96E-04	↓	yjeF N-terminal domain-containing protein 3 [B. mutus]
ENSBTAP00000013292-D1	4.18	2.96E-04	↓	Collagen alpha-1(XV) chain, partial [B. mutus]
ENSBTAP00000008986-D1	4.18	2.96E-04	↓	Putative L-aspartate dehydrogenase, partial [B. mutus]
ENSBTAP00000045034-D1	4.18	2.96E-04	↓	interferon beta-2-like [B. mutus]
ENSBTAP00000018632-D1	4.18	2.96E-04	↓	DNA-binding protein Ikaros [B. mutus]
ENSP00000358798-D1	4.18	2.96E-04	↓	calcium homeostasis modulator protein 3 [B. taurus]
yakA22442	4.18	2.96E-04	↓	Serine/threonine-protein kinase PRKX [B. mutus]
yakG030560	4.18	2.96E-04	↓	BTB/POZ domain-containing protein KCTD12 [B. mutus]
ENSP00000286794-D2	4.19	4.75E-27	↓	N-alpha-acetyltransferase 10, NatA catalytic subunit, partial [B. mutus]
ENSBTAP00000003376-D1	4.20	3.27E-48	↓	septin-2 [B. taurus]
ENSBTAP00000027119-D1	4.20	2.34E-69	↓	Astacin-like metalloendopeptidase, partial [B. mutus]
ENSBTAP00000015763-D1	4.22	1.80E-20	↓	MAL-like protein [B. mutus]
ENSP00000416621-D1	4.23	2.33E-31	↓	DNA replication licensing factor MCM5 [B. mutus]
ENSP00000376663-D2	4.23	7.75E-10	↓	Histone H4 [Heterocephalus glaber]
ENSBTAP00000012345-D1	4.23	7.75E-10	↓	Lymphocyte cytosolic protein 2 [B. mutus]
ENSP00000339750-D1	4.24	2.94E-96	↓	Glycosyltransferase-like domain-containing protein 1, partial [B. mutus]
ENSBTAP00000013093-D1	4.24	1.37E-13	↓	Putative protein KIAA1737-like protein, partial [B. mutus]
ENSBTAP00000010863-D1	4.25	2.52E-17	↓	Dystrophin, partial [B. mutus]
ENSP00000338844-D1	4.25	2.05E-79	↓	protein transport protein Sec23B [B. taurus]
ENSBTAP00000029325-D1	4.26	3.72E-105	↓	tomoregulin-1 isoform X1 [Ovis aries]
ENSP00000264444-D1	4.26	5.72E-54	↓	max dimerization protein 1 [B. taurus]
ENSBTAP00000051624-D1	4.28	4.50E-25	↓	hypothetical protein M91_01465, partial [B. mutus]
ENSP00000355310-D1	4.28	1.28E-17	↓	Hermansky-Pudlak syndrome 1 protein [B. mutus]
ENSBTAP00000022870-D1	4.28	7.00E-14	↓	glycine receptor subunit alpha-4 isoform X1 [B. taurus]
ENSBTAP00000045373-D8	4.28	7.00E-14	↓	40S ribosomal protein S6 [B. taurus]
ENSP00000305403-D28	4.28	2.38E-06	↓	hypothetical protein M91_02988, partial [B. mutus]

ENSBTAP00000016861-D26	4.28	5.43E-163	↓	hypothetical protein M91_04445, partial [B. mutus]
ENSP00000296513-D1	4.30	0.00E+00	↓	adenosine deaminase domain-containing protein 1 isoform X1 [B. mutus]
ENSBTAP00000052433-D24	4.31	4.02E-37	↓	p97Bcnt protein [Capra hircus]
ENSBTAP00000048703-D2	4.31	2.11E-33	↓	protein phosphatase 1 regulatory subunit 14B-like, partial [Bison bison bison]
ENSBTAP00000018920-D1	4.31	3.92E-41	↓	small integral membrane protein 12 isoform X1 [Bubalus bubalis]
ENSP00000264839-D1	4.32	3.34E-18	↓	Regulating synaptic membrane exocytosis protein 1, partial [B. mutus]
ENSBTAP00000021879-D1	4.32	2.00E-10	↓	Serine/threonine-protein kinase MRCK beta [B. mutus]
ENSBTAP00000017926-D1	4.34	0.00E+00	↓	dnaJ homolog subfamily C member 17 [B. mutus]
ENSBTAP00000012735-D1	4.34	1.84E-57	↓	DNA-binding protein A, partial [B. mutus]
ENSBTAP00000007079-D1	4.34	0.00E+00	↓	zinc finger CCHC domain-containing protein 10 [Oryctolagus cuniculus]
ENSBTAP00000011448-D1	4.34	1.82E-14	↓	Solute carrier family 46 member 3, partial [B. mutus]
ENSP00000382004-D1	4.34	2.93E-203	↓	catenin delta-1 [B. mutus]
ENSP00000372319-D1	4.35	1.56E-26	↓	Chromodomain Y-like protein, partial [B. mutus]
ENSP00000391551-D1	4.36	1.44E-34	↓	hypothetical protein M91_07872, partial [B. mutus]
ENSBTAP00000048880-D1	4.36	0.00E+00	↓	disintegrin and metalloproteinase domain-containing protein 28 isoform X1 [B. taurus]
ENSBTAP00000008483-D1	4.36	1.16E-74	↓	tau-tubulin kinase 2 [B. mutus]
ENSP00000271751-D2	4.37	5.92E-75	↓	potassium voltage-gated channel subfamily H member 1 isoform X1 [Bubalus bubalis]
ENSP00000265018-D1	4.37	6.07E-71	↓	protein FAM184B isoform X1 [Bison bison bison]
ENSP00000306361-D1	4.37	6.72E-51	↓	fibrinogen alpha chain [B. mutus]
ENSBTAP00000020661-D1	4.37	7.38E-35	↓	promotilin [B. mutus]
ENSBTAP00000051551-D1	4.37	1.02E-10	↓	Krueppel-like factor 16, partial [B. mutus]
ENSP00000384040-D1	4.37	1.02E-10	↓	Coiled-coil domain-containing protein 85A, partial [B. mutus]
ENSP00000391601-D1	4.37	1.21E-06	↓	Peroxisomal targeting signal 1 receptor, partial [B. mutus]
ENSBTAP00000033117-D1	4.37	1.21E-06	↓	sodium/hydrogen exchanger 4 [B. mutus]
ENSP00000261503-D1	4.37	1.21E-06	↓	Cytospin-B [B. mutus]
ENSBTAP00000041163-D1	4.37	1.45E-04	↓	UPF0449 protein C19orf25 homolog [B. taurus]
ENSP00000395340-D1	4.37	1.45E-04	↓	Protein FAM185A [B. mutus]
ENSBTAP00000049290-D1	4.37	1.45E-04	↓	SLIT and NTRK-like protein 1, partial [B. mutus]
ENSBTAP00000028224-D1	4.37	1.45E-04	↓	A disintegrin and metalloproteinase with thrombospondin motifs 14, partial [B. mutus]
ENSBTAP00000042192-D1	4.37	1.45E-04	↓	Major facilitator superfamily domain-containing protein 7, partial [B. mutus]
ENSP00000279146-D1	4.37	1.45E-04	↓	AH receptor-interacting protein [B. taurus]
ENSP00000359663-D1	4.37	1.45E-04	↓	CD40 ligand [B. taurus]
ENSP00000380903-D1	4.37	1.45E-04	↓	roundabout homolog 3 [B. mutus]
ENSP00000350718-D1	4.37	1.45E-04	↓	Transmembrane and coiled-coil domains protein 2 [B. mutus]
ENSP00000344129-D1	4.37	1.45E-04	↓	zinc finger protein 648-like [B. mutus]
ENSP00000349612-D1	4.37	1.45E-04	↓	Ankyrin repeat domain-containing protein 7, partial [B. mutus]
ENSBTAP00000041364-D1	4.37	1.45E-04	↓	selenocysteine-specific elongation factor [B. mutus]
ENSBTAP00000052774-D5	4.37	1.45E-04	↓	hCG1645834 [Homo sapiens]
ENSP00000368818-D1	4.37	1.45E-04	↓	TOM1-like protein 2, partial [B. mutus]
ENSP00000392490-D1	4.37	1.45E-04	↓	Fibrocystin-L [B. mutus]
ENSBTAP00000018697-D1	4.37	1.45E-04	↓	Neurexin-1-alpha, partial [B. mutus]
ENSP00000400899-D1	4.37	1.45E-04	↓	leucine-rich repeat-containing protein 9-like [B. mutus]
ENSBTAP00000018773-D1	4.37	1.45E-04	↓	putative E3 ubiquitin-protein ligase UNKL isoform X1 [B. taurus]
ENSBTAP00000028542-D2	4.37	1.45E-04	↓	C1GALT1-specific chaperone 1-like [B. mutus]
ENSP00000263088-D1	4.37	1.45E-04	↓	phospholipase D2 [Bison bison bison]
ENSBTAP00000051910-D87	4.37	1.45E-04	↓	LINE-1 reverse transcriptase-like protein [B. mutus]
ENSP00000238699-D1	4.37	1.45E-04	↓	ovarian cancer G-protein coupled receptor 1 isoform X1 [B. taurus]
yakG014813	4.37	1.45E-04	↓	Fibrous sheath CABYR-binding protein [B. mutus]
ENSBTAP00000007434-D1	4.37	1.45E-04	↓	spectrin, beta, non-erythrocytic 4 [B. mutus]
ENSBTAP00000040974-D1	4.37	1.45E-04	↓	mucosal addressin cell adhesion molecule 1 [B. mutus]
ENSP00000334042-D1	4.37	1.45E-04	↓	Tachykinin-4, partial [B. mutus]
ENSBTAP00000021570-D1	4.37	1.45E-04	↓	amine oxidase [flavin-containing] A [B. taurus]
ENSP00000258180-D1	4.37	1.45E-04	↓	uncharacterized protein KIAA0513 homolog isoform X1 [B. taurus]
ENSBTAP00000027836-D1	4.37	1.45E-04	↓	Matrilin-3, partial [B. mutus]
ENSP00000347710-D1	4.37	1.45E-04	↓	Oligophrenin-1, partial [B. mutus]

ENSBTAP00000005370-D1	4.37	1.45E-04	↓	hypothetical protein M91_16566, partial [B. mutus]
ENSBTAP00000034425-D1	4.37	1.45E-04	↓	Endonuclease domain-containing 1 protein, partial [B. mutus]
ENSBTAP00000010124-D1	4.37	1.45E-04	↓	transmembrane protein 59-like precursor [B. taurus]
ENSP00000271889-D1	4.37	1.45E-04	↓	cyclic AMP-responsive element-binding protein 3-like protein 4 isoform X1 [B. mutus]
ENSP00000355785-D2	4.37	1.45E-04	↓	left-right determination factor 2 [B. mutus]
ENSP00000368207-D6	4.37	1.45E-04	↓	Ferritin heavy chain [B. mutus]
ENSBTAP00000013156-D1	4.37	1.45E-04	↓	t-SNARE domain-containing protein 1 [B. mutus]
yakG040942	4.37	1.45E-04	↓	D(4) dopamine receptor [B. mutus]
ENSBTAP00000000607-D1	4.37	1.45E-04	↓	Interferon-related developmental regulator 2, partial [B. mutus]
ENSBTAP00000047683-D1	4.37	1.45E-04	↓	Putative carboxypeptidase PM20D1 [B. mutus]
ENSBTAP00000049713-D4	4.37	1.45E-04	↓	protocadherin beta-6 [B. mutus]
ENSP00000373354-D32	4.37	1.45E-04	↓	mariner transposase [Homo sapiens]
yakG032601	4.37	1.45E-04	↓	ALK tyrosine kinase receptor [B. mutus]
ENSP00000338171-D1	4.37	1.45E-04	↓	Src kinase-associated phosphoprotein 1, partial [B. mutus]
ENSBTAP00000002404-D1	4.37	1.45E-04	↓	INO80 complex subunit B [B. taurus]
ENSBTAP00000025092-D1	4.37	1.45E-04	↓	Dead end protein-like protein 1 [B. mutus]
ENSP00000358532-D1	4.37	1.45E-04	↓	Putative RNA-binding protein 20, partial [B. mutus]
ENSBTAP00000013230-D3	4.37	1.45E-04	↓	hypothetical protein M91_15035, partial [B. mutus]
ENSBTAP00000039405-D1	4.37	1.45E-04	↓	RE1-silencing transcription factor [B. mutus]
ENSBTAP00000007812-D1	4.37	1.45E-04	↓	ESX homeobox 1 [B. mutus]
ENSBTAP00000028319-D1	4.37	1.45E-04	↓	transmembrane protein 35 [Ovis aries]
ENSBTAP00000026200-D1	4.37	1.45E-04	↓	AT-rich interactive domain-containing protein 5A, partial [B. mutus]
ENSBTAP00000049401-D1	4.37	1.45E-04	↓	Claudin-17, partial [B. mutus]
ENSBTAP00000016154-D1	4.37	1.45E-04	↓	Nuclear receptor subfamily 1 group D member 1 [B. mutus]
ENSBTAP00000018110-D1	4.37	1.45E-04	↓	lamin-B2 [B. mutus]
ENSBTAP00000021041-D1	4.37	1.45E-04	↓	uncharacterized protein LOC102395856 isoform X3 [Bubalus bubalis]
ENSP00000370713-D2	4.37	1.45E-04	↓	Double-stranded RNA-specific editase 1, partial [B. mutus]
ENSP00000331625-D1	4.37	1.45E-04	↓	AP20 region protein 1, partial [B. mutus]
ENSP00000298440-D1	4.37	1.45E-04	↓	2-oxoglutarate receptor 1 [B. mutus]
ENSP00000392093-D1	4.37	1.45E-04	↓	rhomboid domain-containing protein 3 [B. mutus]
ENSBTAP00000026423-D1	4.37	1.45E-04	↓	TGF-beta receptor type-2, partial [B. mutus]
ENSP00000268296-D1	4.37	1.45E-04	↓	integrin alpha-X [B. mutus]
ENSBTAP00000025728-D1	4.37	1.45E-04	↓	COP9 signalosome complex subunit 1, partial [B. mutus]
ENSP00000354608-D1	4.37	1.45E-04	↓	lysosomal thioesterase PPT2 precursor [B. taurus]
ENSBTAP00000025032-D1	4.37	1.45E-04	↓	ankyrin repeat and SOCS box protein 13 [B. taurus]
ENSBTAP00000009334-D1	4.37	1.45E-04	↓	thiamine-triphosphatase [B. mutus]
ENSP00000335306-D1	4.37	1.45E-04	↓	Developmental pluripotency-associated protein 4, partial [B. mutus]
ENSBTAP00000026214-D1	4.37	1.45E-04	↓	ADP-ribosylation factor-like protein 4C-like protein [Cricetulus griseus]
ENSP00000264029-D1	4.37	1.45E-04	↓	Trehalase, partial [B. mutus]
ENSP00000329499-D1	4.37	1.45E-04	↓	Ig-like V-type domain-containing protein FAM187A-like [B. mutus]
ENSBTAP00000022318-D1	4.37	1.45E-04	↓	5-hydroxytryptamine receptor 5B [B. mutus]
ENSP00000296327-D1	4.37	1.45E-04	↓	organic solute transporter subunit alpha isoform X1 [B. taurus]
ENSBTAP00000044190-D5	4.37	1.45E-04	↓	hypothetical protein M91_11590, partial [B. mutus]
ENSBTAP00000003831-D1	4.37	1.45E-04	↓	probable D-tyrosyl-tRNA(Tyr) deacylase 2 [B. taurus]
ENSBTAP00000009438-D1	4.37	1.45E-04	↓	Putative sodium-coupled neutral amino acid transporter 7, partial [B. mutus]
ENSBTAP00000025912-D1	4.37	1.45E-04	↓	Prostaglandin E synthase, partial [B. mutus]
yakG003209	4.37	1.45E-04	↓	Huntingtin-associated protein 1 [B. mutus]
ENSBTAP00000028562-D1	4.37	1.45E-04	↓	complexin-3 [B. mutus]
ENSP00000385285-D1	4.37	1.45E-04	↓	RING finger protein 32 [B. mutus]
ENSP00000369738-D70	4.37	1.45E-04	↓	hypothetical protein M91_01591, partial [B. mutus]
ENSBTAP00000025090-D1	4.37	1.45E-04	↓	Serpin A11, partial [B. mutus]
ENSBTAP00000001356-D1	4.37	1.45E-04	↓	zinc finger protein 598, partial [B. mutus]
ENSP00000368298-D1	4.37	1.45E-04	↓	Uroplakin-1a [B. mutus]
ENSBTAP00000048025-D1	4.37	1.45E-04	↓	NKG2-D type II integral membrane protein [B. mutus]
ENSP00000382770-D1	4.37	1.45E-04	↓	Potassium channel subfamily U member 1, partial [B. mutus]

ENSP00000295213-D1	4.37	1.45E-04	↓	Spermatogenesis-associated protein 18, partial [B. mutus]
ENSP00000306138-D1	4.37	1.45E-04	↓	metabotropic glutamate receptor 5 isoform X2 [Ovis aries musimon]
ENSP00000316329-D1	4.37	1.45E-04	↓	stearoyl-CoA desaturase 5 [Pantholops hodgsonii]
ENSBTAP00000036314-D1	4.37	1.45E-04	↓	tumor necrosis factor ligand superfamily member 8 [B. taurus]
ENSP00000419260-D1	4.37	1.45E-04	↓	Phosphatidylinositol-4,5-bisphosphate 3-kinase catalytic subunit gamma isoform [B. mutus]
ENSBTAP00000009823-D1	4.37	1.45E-04	↓	gremlin-1 isoform X2 [Ovis aries musimon]
ENSP00000360659-D1	4.37	1.45E-04	↓	neural proliferation differentiation and control protein 1 [B. mutus]
ENSP00000402929-D1	4.37	1.45E-04	↓	Tumor necrosis factor receptor superfamily member 27 [B. mutus]
ENSP00000312099-D1	4.37	1.45E-04	↓	Beta-crystallin S, partial [B. mutus]
ENSP00000346693-D1	4.37	1.45E-04	↓	elongation of very long chain fatty acids protein 2 [B. taurus]
ENSBTAP00000026897-D1	4.37	1.45E-04	↓	fibroblast growth factor 13 isoform X1 [Orcinus orca]
ENSP00000311984-D1	4.37	1.45E-04	↓	ATP-dependent (S)-NAD(P)H-hydrate dehydratase isoform X5 [Ovis aries musimon]
ENSBTAP00000043756-D2	4.37	1.45E-04	↓	.
ENSBTAP00000009880-D1	4.37	1.45E-04	↓	Fatty-acid amide hydrolase 1 [B. mutus]
ENSBTAP00000032154-D17	4.37	1.45E-04	↓	Zinc finger protein 550, partial [B. mutus]
ENSP00000378204-D1	4.37	1.45E-04	↓	zinc finger and BTB domain-containing protein 25 [B. mutus]
ENSBTAP00000013951-D1	4.37	1.45E-04	↓	sodium/potassium-transporting ATPase subunit alpha-4 [B. mutus]
ENSP00000352288-D1	4.37	1.45E-04	↓	plexin-B2 precursor [B. taurus]
ENSBTAP00000011575-D1	4.37	1.45E-04	↓	5-hydroxytryptamine receptor 7 isoform X3 [B. taurus]
ENSBTAP00000050176-D13	4.37	1.45E-04	↓	histone H2B type 1-J-like [Camelus ferus]
ENSP00000263196-D1	4.37	1.45E-04	↓	Integral membrane protein DGCR2/IDD, partial [B. mutus]
ENSBTAP00000025510-D1	4.37	1.45E-04	↓	phosphatidylinositol 3,4,5-trisphosphate 5-phosphatase 2 [B. mutus]
ENSP00000391093-D1	4.37	1.45E-04	↓	Neurobeachin, partial [B. mutus]
ENSBTAP00000017302-D1	4.37	1.45E-04	↓	probable G-protein coupled receptor 61 [B. mutus]
ENSBTAP00000024453-D8	4.37	1.45E-04	↓	hypothetical protein M91_20816, partial [B. mutus]
ENSP00000353806-D1	4.37	1.45E-04	↓	core histone macro-H2A.1 isoform X1 [B. taurus]
ENSBTAP00000020343-D1	4.37	1.45E-04	↓	testis-expressed protein 19.2-like [B. mutus]
ENSP00000339801-D1	4.37	1.45E-04	↓	Iduronate 2-sulfatase, partial [B. mutus]
ENSP00000218008-D1	4.37	1.45E-04	↓	protein ATP1B4 [B. taurus]
ENSP00000373288-D1	4.37	1.45E-04	↓	biotinidase isoform X1 [B. mutus]
ENSP00000406305-D1	4.37	1.45E-04	↓	galactokinase [B. mutus]
ENSBTAP00000011392-D1	4.37	1.45E-04	↓	protein FAM110A [B. mutus]
ENSBTAP00000049117-D1	4.38	3.79E-35	↓	Mas-related G-protein coupled receptor member X2, partial [B. mutus]
ENSBTAP00000045974-D1	4.39	1.94E-35	↓	hypothetical protein M91_11428, partial [B. mutus]
ENSP00000228955-D1	4.41	0.00E+00	↓	general transcription factor IIH subunit 3 [B. taurus]
ENSBTAP00000053697-D1	4.41	5.69E-69	↓	enhancer of polycomb homolog 2 [B. mutus]
ENSP00000261783-D1	4.41	7.89E-119	↓	arginase-2, mitochondrial [B. mutus]
ENSBTAP00000000662-D1	4.41	5.17E-11	↓	Alpha-1-syntrophin, partial [B. mutus]
ENSP00000323687-D1	4.43	2.40E-15	↓	ubiquitin-conjugating enzyme E2 O [B. mutus]
ENSBTAP00000041902-D2	4.43	2.70E-32	↓	BRCA1-A complex subunit BRE [Galeopterus variegatus]
ENSP00000324821-D1	4.44	1.16E-19	↓	Chitinase domain-containing protein 1, partial [B. mutus]
ENSP00000256117-D1	4.44	2.06E-66	↓	transcription factor E2F5 isoform X1 [B. taurus]
ENSP00000387020-D1	4.45	1.03E-113	↓	rho guanine nucleotide exchange factor 33 isoform X1 [B. taurus]
ENSBTAP00000023190-D1	4.45	8.52E-50	↓	Transcription factor E2F8, partial [B. mutus]
ENSBTAP00000018417-D1	4.46	6.07E-07	↓	homeobox protein MSX-2 [B. taurus]
ENSP00000365318-D1	4.46	6.07E-07	↓	NF-kappa-B inhibitor-like protein 1 [B. mutus]
ENSBTAP00000024626-D1	4.46	6.07E-07	↓	little elongation complex subunit 1 [Bison bison bison]
ENSBTAP00000043604-D1	4.46	1.41E-110	↓	neuroendocrine protein 7B2 isoform X1 [B. taurus]
ENSBTAP00000043450-D1	4.47	3.60E-33	↓	hypothetical protein M91_11909, partial [B. mutus]
ENSBTAP00000011598-D1	4.47	3.60E-33	↓	Ubiquitin carboxyl-terminal hydrolase 3, partial [B. mutus]
ENSP00000356436-D1	4.47	1.06E-272	↓	cytosolic phospholipase A2 [B. mutus]
ENSBTAP00000002628-D1	4.49	4.71E-38	↓	protein FAM69A [B. taurus]
ENSP00000335463-D1	4.49	0.00E+00	↓	Protein CASC5, partial [B. mutus]
ENSBTAP00000028244-D1	4.49	4.66E-246	↓	uncharacterized protein C12orf45 homolog [Bubalus bubalis]
ENSBTAP00000051850-D1	4.49	1.38E-104	↓	Platelet-activating factor acetylhydrolase IB subunit alpha, partial [B. mutus]

ENSBTAP00000003193-D1	4.51	3.29E-150	↓	Nuclear pore complex protein Nup155, partial [B. mutus]
ENSP00000353866-D1	4.51	3.92E-25	↓	Eyes absent-like protein 2, partial [B. mutus]
ENSBTAP00000021029-D1	4.52	6.98E-66	↓	Ectonucleotide pyrophosphatase/phosphodiesterase family member 5, partial [B. mutus]
ENSP00000244537-D6	4.52	5.96E-84	↓	unnamed protein product [Tetraodon nigroviridis]
ENSP00000352608-D2	4.52	7.98E-21	↓	ryanodine receptor 3 [B. mutus]
ENSP00000379279-D1	4.54	7.78E-122	↓	dnaJ homolog subfamily C member 24 [B. taurus]
ENSP00000343741-D1	4.54	1.25E-71	↓	serine/threonine-protein kinase ATR [B. mutus]
ENSP00000262259-D94	4.54	1.65E-16	↓	zinc finger protein 7 isoform X2 [B. taurus]
ENSP00000358576-D1	4.54	6.83E-12	↓	5' exonuclease Apollo, partial [B. mutus]
ENSP00000384932-D1	4.54	3.06E-07	↓	G-protein coupled receptor 12 [B. taurus]
ENSP00000350989-D1	4.54	3.06E-07	↓	Thioredoxin domain-containing protein 5, partial [B. mutus]
ENSP00000369111-D1	4.54	3.06E-07	↓	6-phosphofructo-2-kinase/fructose-2,6-bisphosphatase 3 isoform X4 [B. taurus]
ENSBTAP00000040327-D1	4.54	3.06E-07	↓	Platelet endothelial cell adhesion molecule, partial [B. mutus]
ENSBTAP00000008743-D1	4.54	3.06E-07	↓	prolactin-inducible protein homolog [B. mutus]
ENSBTAP00000017991-D1	4.54	3.06E-07	↓	N(G),N(G)-dimethylarginine dimethylaminohydrolase 2, partial [B. mutus]
ENSP00000357392-D1	4.54	7.16E-05	↓	Ephrin-A1, partial [B. mutus]
ENSP00000368353-D1	4.54	7.16E-05	↓	sodium/bile acid cotransporter 5 [B. taurus]
ENSBTAP00000024638-D1	4.54	7.16E-05	↓	Phakinin, partial [B. mutus]
ENSP00000399384-D1	4.54	7.16E-05	↓	AP-2 complex subunit alpha-1, partial [B. mutus]
ENSBTAP00000004288-D1	4.54	7.16E-05	↓	Tumor necrosis factor receptor superfamily member 9, partial [B. mutus]
ENSP00000291823-D1	4.54	7.16E-05	↓	homeodomain-interacting protein kinase 4 [B. mutus]
ENSBTAP00000013535-D1	4.54	7.16E-05	↓	Growth hormone-regulated TBC protein 1, partial [B. mutus]
ENSBTAP00000039011-D1	4.54	7.16E-05	↓	Trypsin, partial [B. mutus]
ENSP00000356906-D1	4.54	7.16E-05	↓	SH2 domain-containing protein 1B [B. taurus]
ENSP00000358160-D2	4.54	7.16E-05	↓	histone H3.1-like [B. mutus]
ENSP00000305288-D2	4.54	7.16E-05	↓	Neuroligin-2, partial [B. mutus]
ENSP00000341223-D7	4.54	7.16E-05	↓	protein FAM90A27P-like [B. taurus]
ENSBTAP00000001331-D1	4.54	7.16E-05	↓	Endothelial cell-selective adhesion molecule [B. mutus]
ENSBTAP00000049500-D14	4.54	7.16E-05	↓	hypothetical protein M91_19872, partial [B. mutus]
ENSBTAP00000012247-D1	4.54	7.16E-05	↓	Xylosyltransferase 2, partial [B. mutus]
ENSBTAP00000025512-D1	4.54	7.16E-05	↓	Paired mesoderm homeobox protein 2A, partial [B. mutus]
ENSBTAP00000020079-D1	4.54	7.16E-05	↓	Glycerophosphodiester phosphodiesterase domain-containing protein 5 [B. mutus]
ENSP00000374205-D1	4.54	7.16E-05	↓	T-box brain protein 1 [B. mutus]
ENSBTAP00000026911-D1	4.54	7.16E-05	↓	Zinc finger protein 263, partial [B. mutus]
ENSBTAP00000011966-D2	4.54	7.16E-05	↓	Beta-adducin, partial [B. mutus]
ENSP00000376390-D1	4.54	7.16E-05	↓	Ras-related protein Rab-37, partial [B. mutus]
ENSBTAP00000048832-D1	4.54	7.16E-05	↓	hypothetical protein M91_21269, partial [B. mutus]
ENSBTAP00000040177-D1	4.54	7.16E-05	↓	Axin-2 [B. mutus]
ENSBTAP00000032976-D1	4.54	7.16E-05	↓	SH2 domain-containing protein 6, partial [B. mutus]
ENSBTAP00000002390-D1	4.54	7.16E-05	↓	Secernin-2, partial [B. mutus]
ENSBTAP00000015375-D1	4.54	7.16E-05	↓	ectonucleotide pyrophosphatase/phosphodiesterase family member 6 [B. mutus]
ENSP00000394472-D1	4.54	7.16E-05	↓	BTB/POZ domain-containing protein 18 [B. mutus]
ENSBTAP00000001978-D1	4.54	7.16E-05	↓	B-cell lymphoma 6 protein [B. mutus]
ENSP00000270824-D1	4.54	7.16E-05	↓	Protein FAM176B, partial [B. mutus]
ENSBTAP00000027514-D1	4.54	7.16E-05	↓	Zinc finger CCCH domain-containing protein 6, partial [B. mutus]
ENSP00000278426-D1	4.54	7.16E-05	↓	large neutral amino acids transporter small subunit 3 [B. taurus]
ENSP00000414068-D1	4.54	7.16E-05	↓	Pyrroline-5-carboxylate reductase 1, mitochondrial, partial [B. mutus]
ENSBTAP00000005303-D1	4.54	7.16E-05	↓	hypothetical protein M91_19816, partial [B. mutus]
ENSBTAP00000045934-D1	4.54	7.16E-05	↓	Antigen peptide transporter 1, partial [B. mutus]
yakG014383	4.54	7.16E-05	↓	hypothetical protein M91_06300 [B. mutus]
ENSP00000296098-D1	4.54	7.16E-05	↓	Tripartite motif-containing protein 54, partial [B. mutus]
ENSBTAP00000002094-D2	4.54	7.16E-05	↓	Membrane-associated phosphatidylinositol transfer protein 1, partial [B. mutus]
ENSBTAP00000013203-D1	4.54	7.16E-05	↓	solute carrier family 22 member 4 isoform X1 [B. mutus]
ENSP00000263379-D1	4.54	7.16E-05	↓	interleukin-27 receptor subunit alpha [B. mutus]
ENSP00000361569-D1	4.54	7.16E-05	↓	Immediate early response 5-like protein, partial [B. mutus]

ENSP00000353656-D1	4.54	7.16E-05	↓	Cadherin-4, partial [B. mutus]
ENSP00000328137-D1	4.54	7.16E-05	↓	hypothetical protein M91_00034, partial [B. mutus]
ENSBTAP00000045721-D1	4.54	7.16E-05	↓	angiotensin-converting enzyme 2 [B. mutus]
ENSBTAP00000041545-D1	4.54	7.16E-05	↓	1-phosphatidylinositol 4,5-bisphosphate phosphodiesterase delta-4 [B. mutus]
ENSBTAP00000003434-D1	4.54	7.16E-05	↓	methyltransferase-like protein 7A-like [B. mutus]
ENSP00000350818-D1	4.54	7.16E-05	↓	hypothetical protein M91_20409, partial [B. mutus]
ENSBTAP00000011256-D1	4.54	7.16E-05	↓	Solute carrier family 22 member 3, partial [B. mutus]
ENSP00000382146-D1	4.54	7.16E-05	↓	C-type lectin domain family 19 member A-like [B. mutus]
ENSBTAP00000014725-D1	4.54	7.16E-05	↓	fatty acyl-CoA reductase 2 [B. mutus]
ENSP00000392637-D1	4.54	7.16E-05	↓	CD99 antigen-like protein 2, partial [B. mutus]
ENSP00000364218-D1	4.54	7.16E-05	↓	coronin-2A [B. taurus]
ENSP00000383053-D1	4.54	7.16E-05	↓	EPH receptor B2, isoform CRA_c [Homo sapiens]
ENSP00000231004-D1	4.54	7.16E-05	↓	protein-lysine 6-oxidase isoform X1 [B. taurus]
ENSBTAP00000010385-D1	4.54	7.16E-05	↓	N-acetylglucosamine-1-phosphodiester alpha-N-acetylglucosaminidase [B. mutus]
ENSP00000325612-D1	4.54	7.16E-05	↓	Mediator of RNA polymerase II transcription subunit 16, partial [B. mutus]
yakG022501	4.54	7.16E-05	↓	E3 ubiquitin-protein ligase MARCH1 [B. mutus]
ENSP00000406534-D1	4.54	7.16E-05	↓	Transmembrane protein 178 [Tupaia chinensis]
ENSBTAP00000041951-D1	4.54	7.16E-05	↓	Zinc finger FYVE domain-containing protein 21, partial [B. mutus]
ENSBTAP00000001467-D1	4.54	7.16E-05	↓	monocarboxylate transporter 6 [B. mutus]
ENSBTAP00000000139-D1	4.54	7.16E-05	↓	Fibroblast growth factor 18, partial [B. mutus]
ENSP00000342576-D1	4.54	7.16E-05	↓	hypothetical protein M91_01497, partial [B. mutus]
ENSBTAP00000028930-D1	4.54	7.16E-05	↓	T-box transcription factor TBX3-like [Balaenoptera acutorostrata scammoni]
ENSBTAP00000026619-D1	4.54	7.16E-05	↓	DNA replication complex GINS protein PSF1, partial [B. mutus]
ENSP00000358765-D1	4.54	7.16E-05	↓	trophoblast glycoprotein [B. taurus]
ENSP00000333917-D1	4.54	7.16E-05	↓	Dual specificity protein phosphatase 18 [B. mutus]
ENSBTAP00000036828-D1	4.54	7.16E-05	↓	sodium/hydrogen exchanger 2-like [B. mutus]
yakA16470	4.54	7.16E-05	↓	hypothetical protein M91_21672 [B. mutus]
ENSBTAP00000016573-D2	4.54	7.16E-05	↓	hypothetical protein M91_02834, partial [B. mutus]
ENSBTAP00000047628-D1	4.54	7.16E-05	↓	Homeobox protein Hox-A9, partial [B. mutus]
ENSP00000342162-D1	4.54	7.16E-05	↓	Transmembrane protein 221, partial [B. mutus]
ENSBTAP00000030207-D2	4.54	7.16E-05	↓	hypothetical protein M91_08980, partial [B. mutus]
ENSBTAP00000041561-D1	4.54	7.16E-05	↓	interferon-induced transmembrane protein 5 [B. mutus]
ENSP00000374376-D1	4.54	7.16E-05	↓	hypothetical protein M91_01232 [B. mutus]
ENSBTAP00000016063-D1	4.54	7.16E-05	↓	mitoferrin-2 [B. taurus]
ENSBTAP00000040470-D1	4.54	7.16E-05	↓	Collagen alpha-1(V) chain, partial [B. mutus]
ENSP00000371085-D1	4.54	7.16E-05	↓	brain-specific angiogenesis inhibitor 1-associated protein 2-like protein 2 [B. mutus]
yakG032875	4.54	7.16E-05	↓	hypothetical protein M91_09516 [B. mutus]
ENSBTAP00000016301-D1	4.54	7.16E-05	↓	Amiloride-sensitive sodium channel subunit beta, partial [B. mutus]
ENSBTAP00000008736-D1	4.54	7.16E-05	↓	zinc finger protein 668 [B. mutus]
ENSBTAP00000004726-D1	4.54	7.16E-05	↓	Putative DBH-like monooxygenase protein 2, partial [B. mutus]
ENSBTAP00000026635-D1	4.54	7.16E-05	↓	Hydroxyacylglutathione hydrolase, mitochondrial, partial [B. mutus]
ENSP00000392257-D1	4.54	7.16E-05	↓	peptidyl-prolyl cis-trans isomerase-like 6 isoform X6 [Bubalus bubalis]
ENSP00000367815-D1	4.54	7.16E-05	↓	Leucine-rich repeat-containing protein 50, partial [B. mutus]
ENSP00000366091-D1	4.54	7.16E-05	↓	myelin-oligodendrocyte glycoprotein isoform X1 [Pantholops hodgsonii]
ENSP00000381861-D1	4.54	7.16E-05	↓	tetratricopeptide repeat protein 29 isoform X1 [B. taurus]
ENSBTAP00000002617-D7	4.54	7.16E-05	↓	hypothetical protein M91_09584, partial [B. mutus]
ENSP00000347930-D1	4.54	7.16E-05	↓	cell cycle control protein 50B [B. taurus]
ENSBTAP00000021364-D1	4.54	7.16E-05	↓	Arylsulfatase A, partial [B. mutus]
ENSP00000370299-D1	4.54	7.16E-05	↓	Protein Shroom2, partial [B. mutus]
ENSP00000356155-D1	4.54	7.16E-05	↓	phosphatidylinositol 4-phosphate 3-kinase C2 domain-containing subunit beta [B. mutus]
ENSBTAP00000027417-D1	4.54	7.16E-05	↓	Serine protease 27, partial [B. mutus]
ENSP00000394849-D1	4.54	7.16E-05	↓	RHBDL3 protein, partial [Homo sapiens]
ENSP00000370483-D2	4.54	7.16E-05	↓	Neuroligin-4, X-linked, partial [B. mutus]
ENSP00000376234-D1	4.54	7.16E-05	↓	ATP-binding cassette sub-family B member 9 isoform X2 [B. mutus]
ENSBTAP00000016176-D1	4.54	7.16E-05	↓	Putative protein KIAA0415, partial [B. mutus]

ENSP00000346300-D1	4.54	7.16E-05	↓	crk-like protein [B. mutus]
ENSBTAP00000050586-D1	4.54	7.16E-05	↓	Metallo-beta-lactamase domain-containing protein 1 [B. mutus]
ENSP00000264051-D1	4.54	7.16E-05	↓	ephexin-1 [Pantholops hodgsonii]
ENSBTAP00000024042-D1	4.54	7.16E-05	↓	T-lymphocyte activation antigen CD80 [B. mutus]
ENSP00000404438-D1	4.54	7.16E-05	↓	Spermatogenesis- and oogenesis-specific basic helix-loop-helix-containing protein 1 [B. mutus]
ENSP00000362014-D1	4.54	7.16E-05	↓	Dynamin-1 [B. mutus]
ENSP00000366402-D1	4.54	7.16E-05	↓	uncharacterized protein C9orf135 homolog [B. mutus]
ENSP00000291481-D2	4.54	7.16E-05	↓	Hyaluronan and proteoglycan link protein 4, partial [B. mutus]
ENSP00000357575-D1	4.54	7.16E-05	↓	tetratricopeptide repeat protein 40-like [B. mutus]
ENSBTAP00000046309-D1	4.54	7.16E-05	↓	Butyrophilin-like protein 2 [B. mutus]
ENSBTAP00000016263-D1	4.54	7.16E-05	↓	pyridoxine-5'-phosphate oxidase [B. taurus]
ENSP00000295927-D1	4.54	7.16E-05	↓	pentraxin-related protein PTX3 [Bison bison bison]
ENSP00000284878-D1	4.54	7.16E-05	↓	Coxsackievirus and adenovirus receptor-like protein, partial [B. mutus]
ENSP00000339161-D1	4.55	8.41E-77	↓	Axin interactor, dorsalization-associated protein [B. mutus]
ENSBTAP00000023705-D1	4.56	1.73E-35	↓	RNA-binding motif, single-stranded-interacting protein 2 [B. mutus]
ENSP00000332225-D1	4.56	1.03E-180	↓	pro-MCH [B. mutus]
ENSBTAP00000000463-D1	4.57	8.42E-17	↓	Tyrosine-protein kinase SgK223 [B. mutus]
ENSBTAP00000008936-D7	4.57	1.52E-82	↓	Glycine cleavage system H protein, mitochondrial, partial [B. mutus]
ENSP00000292180-D1	4.57	2.27E-40	↓	FAD synthase, partial [B. mutus]
ENSBTAP00000041027-D1	4.57	2.71E-26	↓	F-box only protein 36 [B. mutus]
ENSP00000344666-D1	4.58	3.49E-12	↓	merlin isoform X1 [Bubalus bubalis]
ENSP00000372348-D1	4.58	3.49E-12	↓	histone-lysine N-methyltransferase NSD2 isoform X1 [B. taurus]
ENSP00000250617-D1	4.59	4.33E-17	↓	Rho guanine nucleotide exchange factor 6 [B. mutus]
ENSBTAP00000049304-D3	4.59	9.45E-94	↓	Serine incorporator 3 [B. mutus]
ENSBTAP00000019217-D1	4.62	1.87E-56	↓	RecName: Full=Serine/threonine-protein kinase PLK1; AltName: Full=Polo-like kinase 1; Short=PLK-1
ENSP00000358200-D1	4.62	4.74E-32	↓	Rab11 family-interacting protein 2 [B. mutus]
ENSBTAP00000050721-D43	4.62	2.22E-17	↓	hypothetical protein M91_10256, partial [B. mutus]
ENSBTAP00000001432-D1	4.62	1.78E-12	↓	palladin isoform X2 [B. taurus]
yakG016156	4.62	1.78E-12	↓	Synaptonemal complex central element protein 1-like protein [B. mutus]
ENSP00000354677-D1	4.62	1.55E-07	↓	Glutathione peroxidase 7, partial [B. mutus]
ENSBTAP00000050174-D14	4.63	3.18E-37	↓	hypothetical protein M91_02532, partial [B. mutus]
ENSBTAP00000052226-D1	4.64	0.00E+00	↓	Protein DBF4 like protein B [Pteropus alecto]
ENSBTAP00000021761-D1	4.64	1.31E-57	↓	solute carrier organic anion transporter family member 4C1 [B. mutus]
ENSBTAP00000048378-D1	4.65	0.00E+00	↓	Synaptonemal complex central element protein 2, partial [B. mutus]
ENSBTAP00000033657-D1	4.67	3.29E-33	↓	ataxin-7-like protein 1 isoform 2 [B. taurus]
ENSBTAP00000024699-D2	4.68	4.10E-131	↓	YTH domain-containing family protein 2 [B. taurus]
ENSP00000322238-D1	4.69	0.00E+00	↓	uncharacterized protein C14orf119 homolog [Ovis aries]
ENSBTAP00000018899-D1	4.69	2.65E-111	↓	Hematopoietically-expressed homeobox protein HHEX, partial [B. mutus]
ENSBTAP00000043782-D1	4.69	7.85E-08	↓	CAV1 protein [B. taurus]
ENSP00000310170-D1	4.69	7.85E-08	↓	fos-related antigen 1 [B. taurus]
ENSBTAP00000048486-D1	4.69	7.85E-08	↓	DNA replication factor Cdt1, partial [B. mutus]
ENSBTAP00000053018-D1	4.69	3.57E-05	↓	5-hydroxytryptamine receptor 4 isoform X1 [B. mutus]
ENSBTAP00000001770-D1	4.69	3.57E-05	↓	protein-lysine methyltransferase METTL21E-like [B. mutus]
ENSP00000335307-D1	4.69	3.57E-05	↓	beta-defensin 15 isoform X2 [B. taurus]
ENSP00000287713-D1	4.69	3.57E-05	↓	nicotinamide mononucleotide adenylyltransferase 2 isoform X1 [Capra hircus]
ENSBTAP00000004546-D2	4.69	3.57E-05	↓	Androgen-induced 1 protein, partial [B. mutus]
ENSP00000414232-D1	4.69	3.57E-05	↓	gamma-aminobutyric acid receptor subunit alpha-1 precursor [B. taurus]
ENSP00000374027-D1	4.69	3.57E-05	↓	tryptophan 5-hydroxylase 2 [B. mutus]
ENSBTAP00000004639-D1	4.69	3.57E-05	↓	hypothetical protein M91_19703, partial [B. mutus]
ENSP00000300406-D1	4.69	3.57E-05	↓	guanine nucleotide-binding protein G(I)/G(S)/G(O) subunit gamma-T2 precursor [B. taurus]
ENSBTAP00000010444-D1	4.69	3.57E-05	↓	peroxisomal sarcosine oxidase [B. mutus]
ENSP00000358016-D1	4.69	3.57E-05	↓	sex comb on midleg-like protein 4 isoform X2 [Bison bison bison]
ENSBTAP00000044518-D1	4.69	3.57E-05	↓	RING finger protein 151, partial [B. mutus]
ENSBTAP00000007315-D1	4.69	3.57E-05	↓	ceroid-lipofuscinosis neuronal protein 6 isoform X2 [Bison bison bison]
ENSP00000345436-D1	4.69	3.57E-05	↓	CASK interacting protein 1 [B. mutus]

ENSBTAP00000005250-D1	4.69	3.57E-05	↓	melanocyte protein PMEL isoform X2 [B. mutus]
ENSBTAP00000015001-D1	4.69	3.57E-05	↓	leucine-zipper-like transcriptional regulator 1 [Equus przewalskii]
yakA09427	4.69	3.57E-05	↓	hypothetical protein M91_05662 [B. mutus]
ENSBTAP00000013784-D1	4.69	3.57E-05	↓	Deoxyribonuclease-2-beta, partial [B. mutus]
ENSBTAP00000043710-D1	4.69	3.57E-05	↓	tuftelin isoform X1 [B. mutus]
ENSBTAP00000050413-D1	4.69	3.57E-05	↓	hypothetical protein LOC91289 [B. taurus]
ENSBTAP00000006118-D1	4.69	3.57E-05	↓	Monocarboxylate transporter 12 [B. mutus]
ENSP00000355614-D1	4.69	3.57E-05	↓	Protein FAM89A, partial [B. mutus]
ENSBTAP00000003576-D1	4.69	3.57E-05	↓	Cytosolic phospholipase A2 delta, partial [B. mutus]
ENSP00000297203-D1	4.69	3.57E-05	↓	uncharacterized protein C7orf62-like [B. mutus]
ENSBTAP00000007340-D1	4.69	3.57E-05	↓	decapping and exoribonuclease protein [B. taurus]
ENSBTAP00000019991-D1	4.69	3.57E-05	↓	galactose-3-O-sulfotransferase 4 [B. mutus]
ENSP00000372326-D1	4.69	3.57E-05	↓	ferrochelatase, mitochondrial [Bubalus bubalis]
ENSP00000329411-D1	4.69	3.57E-05	↓	Interferon regulatory factor 7, partial [B. mutus]
ENSBTAP00000010892-D1	4.69	3.57E-05	↓	hepatocyte nuclear factor 4-gamma [B. mutus]
ENSP00000357292-D1	4.69	3.57E-05	↓	ubiquilin-4 [B. mutus]
ENSBTAP00000047165-D3	4.69	3.57E-05	↓	hypothetical protein M91_00797, partial [B. mutus]
ENSBTAP00000047512-D1	4.69	3.57E-05	↓	proteasome (prosome, macropain) 26S subunit, ATPase, 6, partial [Danio rerio]
yakG000700	4.69	3.57E-05	↓	Zinc finger FYVE domain-containing protein 1 [B. mutus]
ENSP00000312753-D1	4.69	3.57E-05	↓	Protein FAM109B [B. mutus]
ENSBTAP00000025471-D2	4.69	3.57E-05	↓	Serine/threonine-protein kinase Nek5, partial [B. mutus]
ENSP00000374121-D1	4.69	3.57E-05	↓	TPA: integrator complex subunit 1-like [B. taurus]
ENSP00000322628-D1	4.69	3.57E-05	↓	ADP-ribosylation factor-like protein 4D [B. taurus]
ENSP00000299642-D1	4.69	3.57E-05	↓	C-type lectin domain family 3 member A [B. mutus]
ENSBTAP00000044658-D1	4.69	3.57E-05	↓	methyltransferase-like protein 24 precursor [B. taurus]
ENSP00000364324-D1	4.69	3.57E-05	↓	constitutive coactivator of PPAR-gamma-like protein 2 [B. mutus]
ENSBTAP00000004136-D1	4.69	3.57E-05	↓	netrin-4 isoform X1 [B. mutus]
ENSBTAP00000017013-D1	4.69	3.57E-05	↓	mannan-binding lectin serine protease 2 [B. mutus]
ENSBTAP00000025375-D1	4.69	3.57E-05	↓	Autophagy-related protein 16-2 [B. mutus]
ENSBTAP00000051321-D1	4.69	3.57E-05	↓	tetratricopeptide repeat protein 39B-like [B. mutus]
ENSBTAP00000003767-D1	4.69	3.57E-05	↓	protein FAM111B [B. mutus]
ENSBTAP00000050623-D44	4.69	3.57E-05	↓	hypothetical protein M91_11336, partial [B. mutus]
ENSBTAP00000009510-D1	4.69	3.57E-05	↓	spermatogenesis-associated protein 9 [B. mutus]
ENSBTAP00000048593-D12	4.69	3.57E-05	↓	endogenous retrovirus group K member 7 Gag polyprotein-like [B. taurus]
ENSP00000376799-D1	4.69	3.57E-05	↓	uncharacterized protein C11orf65 homolog [B. mutus]
ENSP00000351657-D25	4.69	3.57E-05	↓	putative uncharacterized zinc finger protein 814-like [B. mutus]
ENSP00000301057-D1	4.69	3.57E-05	↓	Tumor protein p53-inducible protein 13, partial [B. mutus]
ENSBTAP00000015608-D1	4.69	3.57E-05	↓	Williams-Beuren syndrome chromosomal region 16 protein-like protein, partial [B. mutus]
ENSBTAP00000024803-D2	4.69	3.57E-05	↓	RCC1 domain-containing protein 1, partial [B. mutus]
ENSP00000389046-D92	4.69	3.57E-05	↓	hypothetical protein M91_01429, partial [B. mutus]
ENSBTAP00000026454-D1	4.69	3.57E-05	↓	EGF-like module-containing mucin-like hormone receptor-like 4, partial [B. mutus]
ENSBTAP00000036381-D1	4.69	3.57E-05	↓	LAG1 longevity assurance-like protein 1, partial [B. mutus]
ENSBTAP00000045767-D2	4.69	3.57E-05	↓	melanoma-associated antigen B1-like [B. mutus]
ENSP00000403663-D1	4.69	3.57E-05	↓	AP-4 complex subunit mu-1 [B. mutus]
yakA01362	4.69	3.57E-05	↓	mucin-associated surface protein (MASP) [Trypanosoma cruzi strain CL Brener]
ENSBTAP00000006236-D1	4.69	3.57E-05	↓	B-lymphocyte antigen CD20 [B. mutus]
ENSBTAP00000053344-D1	4.69	3.57E-05	↓	Armadillo repeat-containing protein 3, partial [B. mutus]
ENSBTAP00000052334-D1	4.69	3.57E-05	↓	hypothetical protein M91_15088, partial [B. mutus]
ENSP00000382941-D1	4.69	3.57E-05	↓	Tyrosine-protein phosphatase non-receptor type substrate 1, partial [B. mutus]
ENSBTAP00000037296-D1	4.69	3.57E-05	↓	RecQ-mediated genome instability protein 2, partial [B. mutus]
ENSP00000388762-D1	4.69	3.57E-05	↓	tripeptidyl-peptidase 1 [B. mutus]
ENSBTAP00000006962-D1	4.69	3.57E-05	↓	fms-related tyrosine kinase 3 ligand isoform X1 [B. mutus]
ENSBTAP00000002023-D1	4.69	3.57E-05	↓	sodium-dependent phosphate transport protein 2B [B. mutus]
yakG029257	4.69	3.57E-05	↓	hypothetical protein M91_15713 [B. mutus]
ENSBTAP00000028221-D1	4.69	3.57E-05	↓	cysteine-rich secretory protein LCCL domain-containing 2 [B. mutus]

ENSP00000361993-D2	4.69	3.57E-05	↓	mitochondrial import inner membrane translocase subunit Tim8 A-like [B. mutus]
ENSP00000320445-D1	4.69	3.57E-05	↓	CUB and sushi domain-containing protein 1 [B. mutus]
ENSBTAP00000025295-D1	4.69	3.57E-05	↓	PR domain zinc finger protein 10 [B. mutus]
yakG019336	4.69	3.57E-05	↓	Potassium voltage-gated channel subfamily G member 2 [B. mutus]
ENSBTAP00000007819-D1	4.69	3.57E-05	↓	protein-tyrosine kinase 2-beta isoform X1 [B. mutus]
ENSP00000367486-D1	4.69	3.57E-05	↓	meiosis expressed gene 1 protein homolog isoform 1 [B. taurus]
ENSBTAP00000027515-D1	4.69	3.57E-05	↓	ras-like protein family member 11B [B. taurus]
ENSP00000258200-D1	4.69	3.57E-05	↓	F-box/LRR-repeat protein 8 [B. mutus]
ENSBTAP00000013440-D1	4.69	3.57E-05	↓	collagen alpha-3(V) chain [Bison bison bison]
ENSBTAP00000001347-D1	4.69	3.57E-05	↓	Mitogen-activated protein kinase 7 [B. mutus]
ENSBTAP00000005418-D1	4.69	3.57E-05	↓	probable inactive protein kinase-like protein SgK071 [B. mutus]
ENSBTAP00000025417-D1	4.69	3.57E-05	↓	fibronectin type III domain-containing protein 8 [B. mutus]
yakG001720	4.69	3.57E-05	↓	hypothetical protein M91_05466 [B. mutus]
ENSP00000323555-D18	4.69	3.57E-05	↓	olfactory receptor 8K3-like isoform X2 [Ovis aries musimon]
ENSP00000357354-D1	4.69	3.57E-05	↓	Protein FAM189B [B. mutus]
ENSP00000361048-D1	4.69	3.57E-05	↓	leucine rich adaptor protein 1 [B. mutus]
ENSBTAP00000018203-D1	4.69	3.57E-05	↓	tetratricopeptide repeat protein 25 [B. mutus]
ENSP00000415034-D1	4.69	3.57E-05	↓	neurobeachin-like protein 2 [B. mutus]
ENSBTAP00000020791-D1	4.69	3.57E-05	↓	Amyloid beta A4 precursor protein-binding family B member 1 [B. mutus]
ENSP00000359090-D1	4.69	3.57E-05	↓	netrin-G1 isoform X1 [B. taurus]
ENSP00000251203-D1	4.69	3.57E-05	↓	Pre-B-cell leukemia transcription factor 4, partial [B. mutus]
ENSBTAP00000051904-D1	4.69	3.57E-05	↓	uncharacterized protein C16orf52 homolog [Jaculus jaculus]
ENSBTAP00000024181-D1	4.69	3.57E-05	↓	2-oxoglutarate and iron-dependent oxygenase domain-containing protein 3 [B. mutus]
ENSP00000221561-D1	4.69	3.57E-05	↓	amino-terminal enhancer of split [B. mutus]
ENSBTAP00000027366-D1	4.69	3.57E-05	↓	chondroitin sulfate synthase 2 [B. mutus]
ENSBTAP00000005487-D1	4.69	3.57E-05	↓	jmjC domain-containing protein 4 [B. mutus]
ENSBTAP00000045461-D2	4.69	3.57E-05	↓	POTE ankyrin domain family member A, partial [B. mutus]
ENSBTAP00000027432-D1	4.69	3.57E-05	↓	coiled-coil and C2 domain-containing protein 2A-like [B. mutus]
ENSBTAP00000015658-D1	4.70	6.38E-91	↓	tRNA (guanine-N(1)-)-methyltransferase, partial [B. mutus]
ENSBTAP00000027858-D1	4.70	2.06E-44	↓	Formin-binding protein 4, partial [B. mutus]
ENSP00000387555-D8	4.71	1.02E-23	↓	Guanine nucleotide-binding protein subunit alpha-14, partial [B. mutus]
ENSBTAP00000019450-D1	4.71	0.00E+00	↓	Forkhead box protein R1, partial [B. mutus]
ENSP00000377279-D1	4.73	6.17E-93	↓	DNA polymerase theta [B. mutus]
ENSBTAP00000048967-D99	4.73	2.40E-13	↓	hypothetical protein M91_01403, partial [B. mutus]
ENSP00000326432-D1	4.75	1.35E-51	↓	c-C chemokine receptor type 8 [B. mutus]
ENSBTAP00000015168-D1	4.76	4.75E-30	↓	translation initiation factor eIF-2B subunit epsilon [Bison bison bison]
ENSP00000323194-D3	4.76	4.00E-08	↓	plexin-A4 isoform X1 [B. taurus]
ENSBTAP00000053800-D1	4.76	4.00E-08	↓	testis development-related protein-like [B. mutus]
ENSBTAP00000002361-D1	4.76	4.00E-08	↓	Twisted gastrulation protein-like protein 1, partial [B. mutus]
ENSBTAP00000018207-D1	4.76	4.00E-08	↓	zinc finger protein 296 [B. mutus]
ENSBTAP00000050430-D85	4.77	2.95E-41	↓	hypothetical protein M91_09415, partial [B. mutus]
ENSP00000359245-D1	4.78	4.14E-69	↓	Retinal-specific ATP-binding cassette transporter, partial [B. mutus]
ENSBTAP00000031525-D1	4.78	0.00E+00	↓	Cyclin-dependent kinase-like 2, partial [B. mutus]
ENSBTAP00000025655-D1	4.79	2.75E-47	↓	ER degradation-enhancing alpha-mannosidase-like 3 [B. mutus]
ENSP00000307318-D1	4.79	4.98E-53	↓	prolyl 4-hydroxylase subunit alpha-1 precursor [B. taurus]
ENSBTAP00000018426-D1	4.80	6.34E-14	↓	protein phosphatase Slingshot homolog 3 isoform X1 [Bison bison bison]
ENSBTAP00000014650-D1	4.80	1.50E-205	↓	ATP-binding cassette sub-family B member 7, mitochondrial isoform X2 [B. mutus]
ENSP00000329930-D1	4.81	2.07E-206	↓	nitric oxide-inducible gene protein [B. mutus]
ENSBTAP00000025153-D1	4.81	2.41E-59	↓	leucine-rich repeat-containing protein 16C [Bison bison bison]
ENSP00000377385-D1	4.81	1.43E-184	↓	Suppressor of SWI4 1-like protein, partial [B. mutus]
ENSP00000260045-D1	4.83	1.01E-48	↓	protein-kinase, interferon-inducible double stranded RNA dependent inhibitor, repressor of [B. mutus]
ENSBTAP00000001615-D1	4.83	3.26E-14	↓	Homeobox protein Nkx-3.1 [B. mutus]
ENSBTAP00000043722-D1	4.83	2.05E-08	↓	Protein FAM134B, partial [B. mutus]
ENSP00000361009-D1	4.83	2.05E-08	↓	phosphatidylinositol 3,4,5-trisphosphate-dependent Rac exchanger 1 protein [B. mutus]
ENSBTAP00000009604-D1	4.83	2.05E-08	↓	four and a half LIM domains protein 3 [B. taurus]

ENSBTAP00000026650-D1	4.83	1.79E-05	↓	coiled-coil domain-containing protein 81 [B. mutus]
ENSP00000395738-D1	4.83	1.79E-05	↓	tRNA (uracil-5-)-methyltransferase-like protein A, partial [B. mutus]
ENSBTAP00000004332-D1	4.83	1.79E-05	↓	Tumor protein D53, partial [B. mutus]
ENSP00000317980-D1	4.83	1.79E-05	↓	Protein turtle-like protein B, partial [B. mutus]
ENSBTAP00000048408-D1	4.83	1.79E-05	↓	hypothetical protein M91_05048, partial [B. mutus]
ENSP00000411948-D1	4.83	1.79E-05	↓	suppressor APC domain-containing protein 1 [B. mutus]
ENSBTAP00000018498-D1	4.83	1.79E-05	↓	WAP four-disulfide core domain protein 2-like [B. mutus]
ENSBTAP00000048682-D1	4.83	1.79E-05	↓	interleukin-12 receptor subunit beta-2 isoform X1 [B. mutus]
ENSBTAP00000024623-D1	4.83	1.79E-05	↓	Tubby-related protein 1 [B. mutus]
ENSP00000260983-D1	4.83	1.79E-05	↓	E3 ubiquitin-protein ligase HECW2 [B. mutus]
ENSP00000264144-D1	4.83	1.79E-05	↓	Laminin subunit gamma-2, partial [B. mutus]
ENSP00000361219-D1	4.83	1.79E-05	↓	general transcription factor 3C polypeptide 4 [B. mutus]
ENSBTAP00000008748-D1	4.83	1.79E-05	↓	hypothetical protein M91_00263, partial [B. mutus]
ENSP00000366995-D1	4.83	1.79E-05	↓	FERM and PDZ domain-containing protein 1 [B. mutus]
ENSBTAP00000024433-D1	4.83	1.79E-05	↓	Transmembrane protein 132A, partial [B. mutus]
ENSBTAP00000045610-D1	4.83	1.79E-05	↓	leucine-rich glioma-inactivated protein 1 isoform X1 [Ovis aries]
ENSBTAP00000033278-D3	4.83	1.79E-05	↓	histone H2A type 1-like [Ursus maritimus]
ENSP00000325285-D1	4.83	1.79E-05	↓	Myotubularin-related protein 4, partial [B. mutus]
ENSP00000376148-D1	4.83	1.79E-05	↓	Armadillo repeat-containing protein 6, partial [B. mutus]
ENSBTAP00000053704-D2	4.83	1.79E-05	↓	BDNF/NT-3 growth factors receptor-like isoform X1 [B. mutus]
ENSBTAP00000031584-D1	4.83	1.79E-05	↓	Calcium-binding tyrosine phosphorylation-regulated protein [B. mutus]
ENSBTAP00000046130-D1	4.83	1.79E-05	↓	PR domain zinc finger protein 8 [B. mutus]
ENSBTAP00000020843-D1	4.83	1.79E-05	↓	Vacuolar fusion protein MON1-like protein B, partial [B. mutus]
ENSBTAP00000015491-D1	4.83	1.79E-05	↓	BTB/POZ domain-containing protein KCTD14, partial [B. mutus]
ENSP00000302756-D1	4.83	1.79E-05	↓	forkhead box protein D4-like 1 isoform X2, partial [B. taurus]
ENSP00000331897-D1	4.83	1.79E-05	↓	RecName: Full=Isocitrate dehydrogenase, mitochondrial; Short=IDH; AltName: Full=ICD-M; AltName: Full=IDP; AltName: Full=NADP(+)-specific ICDH; AltName: Full=Oxalosuccinate decarboxylase; Flags: Precursor
ENSBTAP00000051307-D1	4.83	1.79E-05	↓	Septin-4, partial [B. mutus]
ENSP00000395040-D1	4.83	1.79E-05	↓	Transcription elongation factor SPT5, partial [B. mutus]
ENSBTAP00000048584-D28	4.83	1.79E-05	↓	hypothetical protein M91_09415, partial [B. mutus]
ENSP00000392996-D1	4.83	1.79E-05	↓	Ribonuclease-like protein 10, partial [B. mutus]
ENSP00000254584-D1	4.83	1.79E-05	↓	arfaptin-2 isoform 1 [Ceratotherium simum simum]
ENSBTAP00000003061-D1	4.83	1.79E-05	↓	Tubby-related protein 2, partial [B. mutus]
ENSBTAP00000053410-D1	4.83	1.79E-05	↓	ribosomal protein S6 kinase alpha-2 [B. taurus]
ENSP00000302077-D1	4.83	1.79E-05	↓	Schlafen family member 12, partial [B. mutus]
ENSP00000321326-D1	4.83	1.79E-05	↓	Proteinase-activated receptor 1, partial [B. mutus]
ENSBTAP00000030094-D1	4.83	1.79E-05	↓	hypothetical protein M91_20772 [B. mutus]
ENSP00000252137-D1	4.83	1.79E-05	↓	protein DGCR14 [B. taurus]
ENSBTAP00000005112-D1	4.83	1.79E-05	↓	SH2 domain-containing protein 4B [B. mutus]
ENSP00000296473-D1	4.83	1.79E-05	↓	vacuolar fusion protein MON1 homolog A [B. mutus]
ENSP00000314543-D1	4.83	1.79E-05	↓	melanoma-associated antigen B10-like [B. mutus]
ENSBTAP00000007187-D1	4.83	1.79E-05	↓	adenylate cyclase type 6 [B. mutus]
yakG033567	4.83	1.79E-05	↓	hypothetical protein M91_03724 [B. mutus]
ENSP00000360120-D1	4.83	1.79E-05	↓	tyrosine-protein kinase transmembrane receptor ROR1 isoform X1 [Bison bison bison]
ENSBTAP00000017637-D1	4.83	1.79E-05	↓	gamma-aminobutyric acid receptor subunit alpha-6 [B. taurus]
ENSP00000380523-D1	4.83	1.79E-05	↓	calcyphosin-like protein isoform X2 [Ovis aries]
ENSBTAP00000047568-D1	4.83	1.79E-05	↓	uncharacterized protein C11orf63 homolog isoform X1 [B. mutus]
ENSBTAP00000036833-D1	4.83	1.79E-05	↓	A disintegrin and metalloproteinase with thrombospondin motifs 20-like [B. mutus]
ENSP00000361818-D1	4.83	1.79E-05	↓	Syndecan-4, partial [B. mutus]
ENSBTAP00000008890-D1	4.83	1.79E-05	↓	Carbonic anhydrase-related protein 11 [B. mutus]
ENSBTAP00000042923-D2	4.83	1.79E-05	↓	Phosphatidate phosphatase PPAPDC1B, partial [B. mutus]
ENSP00000371711-D1	4.83	1.79E-05	↓	Doublesex- and mab-3-related transcription factor 1 [B. mutus]
ENSBTAP00000034537-D1	4.83	1.79E-05	↓	N-acetyllactosaminide beta-1,6-N-acetylglucosaminyl-transferase, isoform B-like [B. mutus]
yakG004837	4.83	1.79E-05	↓	Fer-1-like protein 4 [B. mutus]
ENSP00000314380-D1	4.83	1.79E-05	↓	protein lifeguard 1, partial [Capra hircus]

ENSBTAP00000053750-D2	4.83	1.79E-05	↓	neurabin-1 [B. mutus]
ENSP00000220659-D1	4.83	1.79E-05	↓	transcription factor IIIB 50 kDa subunit [B. mutus]
ENSP00000350630-D1	4.83	1.79E-05	↓	tolloid-like protein 2 [B. mutus]
ENSP00000324343-D1	4.83	1.79E-05	↓	beta-ureidopropionase [B. mutus]
ENSBTAP00000021451-D2	4.83	1.79E-05	↓	hypothetical protein M91_10368, partial [B. mutus]
ENSP00000355014-D1	4.83	1.79E-05	↓	E3 ubiquitin-protein ligase Praja-1 isoform X1 [B. mutus]
ENSP00000255390-D1	4.83	1.79E-05	↓	Protein SCO1-like protein, mitochondrial [B. mutus]
ENSBTAP00000049140-D5	4.83	1.79E-05	↓	acyl-protein thioesterase 1 isoform 1 [Ceratotherium simum simum]
ENSBTAP00000025033-D1	4.83	1.79E-05	↓	cadherin EGF LAG seven-pass G-type receptor 2 [B. mutus]
ENSP00000292672-D1	4.83	1.79E-05	↓	CUGBP Elav-like family member 5 isoform X1 [Odobenus rosmarus divergens]
ENSBTAP00000004411-D3	4.83	1.79E-05	↓	protein-arginine deiminase type-1 [B. mutus]
ENSBTAP00000032783-D1	4.83	1.79E-05	↓	collagen alpha-1(XVII) chain [B. mutus]
ENSP00000371512-D1	4.83	1.79E-05	↓	Zeta-sarcoglycan, partial [B. mutus]
ENSBTAP00000006500-D1	4.83	1.79E-05	↓	testis-expressed sequence 35 protein [B. mutus]
ENSBTAP00000029677-D1	4.83	1.79E-05	↓	transmembrane protein 132B [Bubalus bubalis]
ENSBTAP00000014128-D1	4.83	1.79E-05	↓	leucine-rich repeat-containing protein 31 [B. mutus]
ENSP00000246117-D1	4.83	1.79E-05	↓	nicalin [B. taurus]
ENSBTAP00000023784-D1	4.83	1.79E-05	↓	Alpha-2,8-sialyltransferase 8B, partial [B. mutus]
ENSBTAP00000015167-D1	4.83	1.79E-05	↓	hypothetical protein M91_01537 [B. mutus]
ENSBTAP00000003860-D1	4.83	1.79E-05	↓	Protein phosphatase 1M, partial [B. mutus]
ENSP00000303129-D1	4.83	1.79E-05	↓	synaptic vesicle membrane protein VAT-1 homolog-like [B. taurus]
ENSP00000311502-D1	4.83	1.79E-05	↓	Protein HEG-like protein 1, partial [B. mutus]
ENSBTAP00000025292-D1	4.83	1.79E-05	↓	spermatid nuclear transition protein 1 [B. mutus]
ENSBTAP00000024226-D1	4.83	1.79E-05	↓	interleukin-15 precursor [B. taurus]
ENSBTAP00000005144-D2	4.83	1.79E-05	↓	hypothetical protein M91_07077, partial [B. mutus]
ENSBTAP00000031114-D11	4.83	1.79E-05	↓	putative RNA polymerase II subunit A C-terminal domain phosphatase SSU72-like protein 1-like [L. vexillifer]
ENSP00000333980-D11	4.83	1.79E-05	↓	Zinc finger protein 416, partial [B. mutus]
ENSBTAP00000013892-D1	4.83	1.79E-05	↓	D-aspartate oxidase, partial [B. mutus]
ENSP00000400153-D2	4.83	1.79E-05	↓	Interleukin-32, partial [B. mutus]
ENSP00000360576-D1	4.83	1.79E-05	↓	NADPH-dependent diflavin oxidoreductase 1 [B. mutus]
ENSP00000306662-D1	4.83	1.79E-05	↓	Alpha-1B adrenergic receptor, partial [B. mutus]
ENSP00000324617-D1	4.83	1.79E-05	↓	Heparan-sulfate 6-O-sulfotransferase 2, partial [B. mutus]
ENSP00000310241-D3	4.83	1.79E-05	↓	Solute carrier family 22 member 20, partial [B. mutus]
ENSBTAP00000025446-D1	4.83	1.79E-05	↓	Cdc42 effector protein 4 [B. mutus]
ENSBTAP00000039441-D1	4.83	1.79E-05	↓	Neuronal acetylcholine receptor subunit alpha-9, partial [B. mutus]
ENSBTAP00000028359-D1	4.83	1.79E-05	↓	radial spoke head 1 homolog isoform X1 [B. taurus]
ENSP00000368347-D1	4.83	1.79E-05	↓	Heat shock protein HSP 90-beta, partial [B. mutus]
ENSBTAP00000000032-D1	4.83	1.79E-05	↓	Lysozyme-like protein 6, partial [B. mutus]
ENSBTAP00000051022-D1	4.83	1.79E-05	↓	Proproteinase E, partial [B. mutus]
ENSP00000189978-D1	4.83	1.79E-05	↓	muscle, skeletal receptor tyrosine-protein kinase isoform X3 [B. mutus]
ENSP00000410312-D1	4.83	1.79E-05	↓	ATP-dependent DNA helicase Q4 [B. mutus]
ENSP00000243189-D1	4.83	1.02E-77	↓	UPF0471 protein C1orf63 homolog [B. mutus]
ENSP00000358939-D1	4.83	3.55E-141	↓	Seryl-tRNA synthetase, cytoplasmic [B. mutus]
ENSP00000311665-D1	4.86	4.64E-32	↓	Proteasomal ATPase-associated factor 1, partial [B. mutus]
ENSBTAP00000005874-D1	4.86	8.04E-156	↓	hydroxyacid-oxoacid transhydrogenase, mitochondrial isoform X1 [B. mutus]
ENSP00000316237-D1	4.86	1.68E-14	↓	coiled-coil domain-containing protein 102B [B. mutus]
ENSP00000254262-D1	4.87	2.33E-150	↓	Fanconi anemia-associated protein of 24 kDa [B. mutus]
ENSP00000409797-D1	4.87	3.31E-62	↓	Leucine-rich repeat-containing protein 67, partial [B. mutus]
ENSP00000251296-D1	4.88	2.55E-86	↓	Immunoglobulin superfamily member 21, partial [B. mutus]
ENSBTAP00000009150-D1	4.88	4.03E-212	↓	Mediator of RNA polymerase II transcription subunit 30 [B. mutus]
ENSP00000380875-D1	4.88	3.03E-182	↓	dedicator of cytokinesis protein 4 [Lipotes vexillifer]
ENSP00000360672-D1	4.88	1.15E-38	↓	Partitioning defective 6-like protein beta, partial [B. mutus]
ENSP00000400459-D1	4.89	7.71E-21	↓	Clathrin coat assembly protein AP180 [B. mutus]
ENSP00000306410-D1	4.89	8.67E-15	↓	Armadillo repeat-containing protein 4 [B. mutus]
ENSP00000369674-D1	4.89	1.05E-08	↓	B-cell CLL/lymphoma 7 protein family member C, partial [B. mutus]

ENSBTAP00000006683-D1	4.89	1.05E-08	↓	Delta(14)-sterol reductase, partial [B. mutus]
ENSBTAP00000039875-D1	4.89	1.05E-08	↓	Dixin, partial [B. mutus]
ENSBTAP00000008762-D1	4.89	1.05E-08	↓	transmembrane protein 179 [B. mutus]
ENSBTAP00000048644-D1	4.89	1.05E-08	↓	hypothetical protein M91_08429, partial [B. mutus]
ENSP00000196489-D14	4.90	3.08E-39	↓	hypothetical protein M91_05127, partial [B. mutus]
ENSP00000363027-D1	4.93	2.07E-21	↓	mannose-1-phosphate guanyltransferase alpha isoform X2 [Pan paniscus]
ENSBTAP00000002976-D1	4.94	0.00E+00	↓	probable U3 small nucleolar RNA-associated protein 11 [B. mutus]
ENSP00000390728-D1	4.95	5.39E-09	↓	Disintegrin and metalloproteinase domain-containing protein 8, partial [B. mutus]
ENSBTAP00000027999-D1	4.95	5.39E-09	↓	Prolyl 3-hydroxylase 2, partial [B. mutus]
ENSBTAP00000019752-D1	4.95	5.39E-09	↓	voltage-dependent P/Q-type calcium channel subunit alpha-1A [B. mutus]
ENSP00000366035-D3	4.95	9.05E-06	↓	Alpha-actinin-2, partial [B. mutus]
ENSBTAP00000006939-D1	4.95	9.05E-06	↓	Wee1-like protein kinase, partial [B. mutus]
ENSBTAP00000017255-D1	4.95	9.05E-06	↓	catechol O-methyltransferase domain-containing protein 1 [B. taurus]
ENSBTAP00000029455-D1	4.95	9.05E-06	↓	Serine/threonine-protein kinase PINK1, mitochondrial, partial [B. mutus]
ENSBTAP00000032677-D1	4.95	9.05E-06	↓	hypothetical protein M91_05896 [B. mutus]
ENSP00000336775-D1	4.95	9.05E-06	↓	Synemin, partial [B. mutus]
ENSP00000295588-D1	4.95	9.05E-06	↓	protein O-glucosyltransferase 1 [B. mutus]
ENSBTAP00000001624-D1	4.95	9.05E-06	↓	mitochondrial glutamate carrier 1 [B. mutus]
ENSBTAP00000017188-D1	4.95	9.05E-06	↓	Isobutyryl-CoA dehydrogenase, mitochondrial, partial [B. mutus]
ENSBTAP00000015434-D1	4.95	9.05E-06	↓	izumo sperm-egg fusion protein 1 isoform X1 [B. taurus]
ENSBTAP00000028643-D1	4.95	9.05E-06	↓	tRNA (guanine-N(7)-)-methyltransferase subunit WDR4 [B. mutus]
ENSBTAP00000001298-D1	4.95	9.05E-06	↓	kelch domain-containing protein 8B [B. mutus]
ENSBTAP00000027992-D1	4.95	9.05E-06	↓	hypothetical protein M91_06832 [B. mutus]
ENSBTAP00000028966-D1	4.95	9.05E-06	↓	gap junction delta-2 protein [B. taurus]
ENSP00000283309-D1	4.95	9.05E-06	↓	FERM domain-containing protein 1, partial [B. mutus]
ENSP00000301873-D1	4.95	9.05E-06	↓	Latent-transforming growth factor beta-binding protein 3, partial [B. mutus]
ENSBTAP00000042806-D28	4.95	9.05E-06	↓	hypothetical protein M91_09013, partial [B. mutus]
ENSP00000377372-D1	4.95	9.05E-06	↓	Neuromodulin, partial [B. mutus]
ENSBTAP00000049051-D1	4.95	9.05E-06	↓	surfactant-associated protein 2 precursor [B. taurus]
ENSBTAP00000010350-D1	4.95	9.05E-06	↓	heparan sulfate glucosamine 3-O-sulfotransferase 3B1 [B. mutus]
ENSBTAP00000002760-D1	4.95	9.05E-06	↓	protein FAM196B [B. mutus]
ENSBTAP00000050580-D1	4.95	9.05E-06	↓	hypothetical protein M91_05510, partial [B. mutus]
ENSBTAP00000023559-D1	4.95	9.05E-06	↓	attractin-like protein 1 isoform X4 [Ovis aries]
yakG022852	4.95	9.05E-06	↓	hypothetical protein M91_06050 [B. mutus]
ENSBTAP00000047526-D1	4.95	9.05E-06	↓	Tumor necrosis factor alpha-induced protein 8-like protein 1 [B. mutus]
ENSP00000165698-D1	4.95	9.05E-06	↓	receptor expression-enhancing protein 1 [Tarsius syrichta]
ENSP00000362287-D4	4.95	9.05E-06	↓	hypothetical protein PANDA_005372, partial [Ailuropoda melanoleuca]
ENSBTAP00000015978-D1	4.95	9.05E-06	↓	protein-arginine deiminase type-3 isoform X1 [B. taurus]
ENSP00000365012-D1	4.95	9.05E-06	↓	Tyrosine-protein kinase HCK, partial [B. mutus]
ENSBTAP00000040454-D1	4.95	9.05E-06	↓	Coiled-coil domain-containing protein 69, partial [B. mutus]
ENSP00000264705-D1	4.95	9.05E-06	↓	CAD protein [B. mutus]
yakA05801	4.95	9.05E-06	↓	hypothetical protein M91_03567 [B. mutus]
ENSBTAP00000020131-D1	4.95	9.05E-06	↓	hepatoma-derived growth factor-like [B. mutus]
ENSBTAP00000031960-D1	4.95	9.05E-06	↓	Putative cationic amino acid transporter, partial [B. mutus]
ENSBTAP00000023781-D4	4.95	9.05E-06	↓	Bone morphogenetic protein 6, partial [B. mutus]
ENSP00000388165-D1	4.95	9.05E-06	↓	coiled-coil domain-containing protein C16orf93 homolog [B. mutus]
ENSBTAP00000003353-D1	4.95	9.05E-06	↓	mRNA-decapping enzyme 1B [B. mutus]
ENSP00000384716-D1	4.95	9.05E-06	↓	probable palmitoyltransferase ZDHHC8 [B. mutus]
ENSBTAP00000024327-D1	4.95	9.05E-06	↓	spermatogenesis-associated protein 5-like protein 1 [B. mutus]
yakA19103	4.95	9.05E-06	↓	hypothetical protein M91_13117 [B. mutus]
ENSP00000306997-D1	4.95	9.05E-06	↓	Myozenin-2 [B. mutus]
ENSBTAP00000053812-D1	4.95	9.05E-06	↓	Matrix metalloproteinase-21, partial [B. mutus]
ENSBTAP00000050441-D1	4.95	9.05E-06	↓	hypothetical protein M91_01920, partial [B. mutus]
ENSBTAP00000052592-D80	4.95	9.05E-06	↓	hypothetical protein M91_02702, partial [B. mutus]
ENSP00000375647-D1	4.95	9.05E-06	↓	voltage-dependent calcium channel gamma-7 subunit [Homo sapiens]

ENSP00000363241-D1	4.95	9.05E-06	↓	Zinc finger protein 618, partial [B. mutus]
ENSBTAP00000000051-D1	4.95	9.05E-06	↓	hypothetical protein M91_19667, partial [B. mutus]
ENSBTAP00000020428-D2	4.95	9.05E-06	↓	hypothetical protein M91_16259, partial [B. mutus]
ENSBTAP00000029247-D1	4.95	9.05E-06	↓	SH3 domain-binding protein 1, partial [B. mutus]
yakG017263	4.95	9.05E-06	↓	Leukemia NUP98 fusion partner 1 [B. mutus]
ENSBTAP00000003503-D1	4.95	9.05E-06	↓	Maleylacetoacetate isomerase, partial [B. mutus]
ENSP00000254667-D1	4.95	9.05E-06	↓	Receptor-type tyrosine-protein phosphatase epsilon [B. mutus]
ENSBTAP00000034061-D1	4.95	9.05E-06	↓	kynurenine 3-monooxygenase [B. mutus]
ENSP00000362601-D1	4.95	9.05E-06	↓	cysteine-rich hydrophobic domain-containing protein 1 isoform X3 [Trichechus manatus latirostris]
ENSBTAP00000011564-D1	4.95	9.05E-06	↓	Retinal guanylyl cyclase 1, partial [B. mutus]
ENSP00000216445-D1	4.95	9.05E-06	↓	uncharacterized protein C14orf105 homolog isoform X1 [B. mutus]
ENSBTAP00000034540-D1	4.95	9.05E-06	↓	serine/threonine-protein kinase MAK isoform X2 [B. mutus]
ENSBTAP00000025599-D1	4.95	9.05E-06	↓	Zinc transporter ZIP14, partial [B. mutus]
ENSBTAP00000046047-D1	4.95	9.05E-06	↓	Carbonic anhydrase-related protein 10, partial [B. mutus]
ENSBTAP00000043123-D1	4.95	9.05E-06	↓	Receptor-transporting protein 4 [B. mutus]
ENSBTAP00000022108-D1	4.95	9.05E-06	↓	Dual serine/threonine and tyrosine protein kinase [B. mutus]
ENSP00000409610-D1	4.95	9.05E-06	↓	Protein TSSC4, partial [B. mutus]
ENSBTAP00000027868-D1	4.95	9.05E-06	↓	Regulator of nonsense transcripts 1 [B. mutus]
ENSP00000377141-D1	4.95	9.05E-06	↓	beta-arrestin-1 isoform X3 [Ovis aries]
ENSBTAP00000041294-D2	4.95	9.05E-06	↓	fatty acid desaturase 2-like protein FADS2P1-like, partial [B. mutus]
ENSP00000332613-D1	4.95	9.05E-06	↓	Out at first protein-like protein, partial [B. mutus]
ENSP00000362222-D1	4.95	9.05E-06	↓	Serine/threonine-protein kinase 40 [B. mutus]
ENSBTAP00000050423-D1	4.95	9.05E-06	↓	succinate dehydrogenase assembly factor 1, mitochondrial [B. mutus]
ENSP00000403181-D1	4.95	9.05E-06	↓	hypothetical protein M91_00185, partial [B. mutus]
ENSBTAP00000039977-D1	4.95	9.05E-06	↓	hypothetical protein M91_15761, partial [B. mutus]
yakG042006	4.95	9.05E-06	↓	hypothetical protein M91_01569 [B. mutus]
ENSBTAP00000051692-D9	4.95	9.05E-06	↓	hypothetical protein M91_03879, partial [B. mutus]
ENSBTAP00000014674-D1	4.95	9.05E-06	↓	presqualene diphosphate phosphatase [B. mutus]
ENSBTAP00000005603-D1	4.95	9.05E-06	↓	Adipocyte plasma membrane-associated protein, partial [B. mutus]
ENSBTAP00000053302-D1	4.95	9.05E-06	↓	GPI ethanolamine phosphate transferase 3 isoform X1 [B. taurus]
ENSBTAP00000014893-D1	4.95	9.05E-06	↓	THAP domain-containing protein 3 [Bison bison bison]
ENSBTAP00000025945-D1	4.95	9.05E-06	↓	N-acylethanolamine-hydrolyzing acid amidase, partial [B. mutus]
ENSP00000308926-D1	4.95	9.05E-06	↓	protein phosphatase 1J isoform X1 [Bison bison bison]
ENSBTAP00000022045-D1	4.95	9.05E-06	↓	Protein ITFG3, partial [B. mutus]
ENSP00000347310-D1	4.95	9.05E-06	↓	arylsulfatase G [B. mutus]
ENSP00000363157-D1	4.95	9.05E-06	↓	tumor necrosis factor ligand superfamily member 15 [B. mutus]
ENSBTAP00000008149-D1	4.95	9.05E-06	↓	probable Xaa-Pro aminopeptidase 3 [B. mutus]
ENSBTAP00000021035-D1	4.95	9.05E-06	↓	exocyst complex component 3-like protein 2 isoform X7 [Ovis aries musimon]
ENSP00000377582-D23	4.95	9.05E-06	↓	hypothetical protein M91_00511, partial [B. mutus]
ENSBTAP00000050194-D98	4.95	9.05E-06	↓	hypothetical protein M91_04059 [B. mutus]
ENSBTAP00000021495-D1	4.95	9.05E-06	↓	Complement component C9, partial [B. mutus]
ENSBTAP00000024705-D1	4.95	9.05E-06	↓	Zinc finger FYVE domain-containing protein 27 [B. mutus]
yakA11407	4.95	9.05E-06	↓	FXYD domain-containing ion transport regulator 4 [B. mutus]
ENSP00000348478-D1	4.95	9.05E-06	↓	Katanin p60 ATPase-containing subunit A-like 2, partial [B. mutus]
ENSP00000271331-D1	4.95	9.05E-06	↓	prokineticin-1 [B. mutus]
ENSBTAP00000024358-D1	4.95	9.05E-06	↓	pappalysin-2 [B. mutus]
ENSP00000310668-D1	4.97	1.66E-136	↓	Nuclear pore complex protein Nup93 [B. mutus]
yakG021104	4.97	2.61E-28	↓	Lethal(2) giant larvae protein-like protein 2 [B. mutus]
ENSP00000334564-D1	4.97	5.35E-150	↓	DNA-directed RNA polymerase III subunit RPC3 [B. mutus]
ENSBTAP00000003666-D1	4.98	5.12E-125	↓	Porimin, partial [B. mutus]
ENSBTAP00000005822-D1	5.00	7.02E-29	↓	Solute carrier family 15 member 1, partial [B. mutus]
ENSP00000278360-D1	5.00	3.41E-68	↓	Inactive serine protease PAMR1, partial [B. mutus]
ENSBTAP00000007077-D1	5.00	4.39E-42	↓	XK-related protein 2, partial [B. mutus]
ENSP00000361924-D1	5.01	7.06E-62	↓	Peptidyl-prolyl cis-trans isomerase E, partial [B. mutus]
ENSP00000357000-D1	5.01	6.23E-16	↓	upstream transcription factor 1 [Pantholops hodgsonii]

ENSBTAP00000049055-D1	5.01	6.23E-16	↓	inhibitor of nuclear factor kappa-B kinase subunit beta [B. taurus]
ENSBTAP00000002024-D1	5.01	2.78E-09	↓	alpha-1,3-mannosyl-glycoprotein 2-beta-N-acetylglucosaminyltransferase isoform X1 [Pantholops hodgsonii]
ENSP00000296218-D1	5.01	2.78E-09	↓	Axonemal dynein light intermediate polypeptide 1, partial [B. mutus]
ENSBTAP00000051259-D76	5.01	2.78E-09	↓	hypothetical protein M91_05014, partial [B. mutus]
ENSP00000359263-D1	5.02	6.31E-76	↓	breast cancer anti-estrogen resistance protein 3 isoform X1 [B. mutus]
ENSBTAP00000032948-D1	5.03	7.77E-23	↓	T-complex protein 11-like protein 1 [B. mutus]
ENSP00000370744-D1	5.04	5.71E-90	↓	Inositol 1,4,5-trisphosphate receptor type 2 [B. mutus]
ENSP00000319739-D1	5.05	4.18E-91	↓	hypothetical protein M91_16792, partial [B. mutus]
ENSP00000083182-D1	5.06	9.52E-112	↓	TPA: amyloid beta precursor protein (cytoplasmic tail) binding protein 2 [B. taurus]
ENSP00000234296-D1	5.06	3.81E-173	↓	origin recognition complex subunit 2 isoform X1 [B. mutus]
ENSBTAP00000020656-D1	5.07	1.34E-215	↓	bifunctional lysine-specific demethylase and histidyl-hydroxylase MINA isoform X3 [Bison bison bison]
ENSBTAP00000007029-D1	5.07	2.69E-30	↓	Transmembrane protein 92, partial [B. mutus]
ENSP00000378248-D1	5.07	1.43E-09	↓	hypothetical protein EGM_14585, partial [Macaca fascicularis]
ENSBTAP00000052922-D1	5.07	1.43E-09	↓	Kelch repeat and BTB domain-containing protein 5, partial [B. mutus]
ENSP00000396577-D1	5.07	1.43E-09	↓	WD repeat-containing protein 37 isoform X2 [B. taurus]
ENSBTAP00000007652-D1	5.07	1.43E-09	↓	Coiled-coil domain-containing protein 61 [B. mutus]
ENSBTAP00000053653-D5	5.07	1.43E-09	↓	Myosin-13, partial [B. mutus]
yakG008778	5.07	1.43E-09	↓	Aryl hydrocarbon receptor [B. mutus]
ENSP00000306760-D1	5.07	4.60E-06	↓	Leucine-rich repeat-containing protein 45 [B. mutus]
ENSBTAP00000046985-D1	5.07	4.60E-06	↓	TPA: ankyrin repeat domain 26-like [B. taurus]
ENSP00000380988-D1	5.07	4.60E-06	↓	Protein FAM188B2, partial [B. mutus]
ENSBTAP00000020019-D1	5.07	4.60E-06	↓	Brain protein 16, partial [B. mutus]
ENSBTAP00000009404-D1	5.07	4.60E-06	↓	probable serine carboxypeptidase CPVL [B. mutus]
ENSP00000272771-D1	5.07	4.60E-06	↓	Tomoregulin-2, partial [B. mutus]
ENSP00000415822-D1	5.07	4.60E-06	↓	ataxin-1-like [B. taurus]
ENSBTAP00000007623-D1	5.07	4.60E-06	↓	E3 ubiquitin-protein ligase TRIM50 isoform X1 [B. mutus]
ENSP00000358817-D1	5.07	4.60E-06	↓	Macrophage colony-stimulating factor 1, partial [B. mutus]
ENSBTAP00000039787-D1	5.07	4.60E-06	↓	uncharacterized protein LOC536190 precursor [B. taurus]
ENSBTAP00000039142-D1	5.07	4.60E-06	↓	uncharacterized protein C20orf196 homolog [B. mutus]
ENSP00000358884-D1	5.07	4.60E-06	↓	Cytochrome b561 domain-containing protein 1 [B. mutus]
ENSP00000413501-D1	5.07	4.60E-06	↓	Somatotropin, partial [B. mutus]
ENSBTAP00000024202-D1	5.07	4.60E-06	↓	ankyrin repeat and SOCS box protein 4 [B. mutus]
ENSP00000392188-D7	5.07	4.60E-06	↓	hypothetical protein M91_05887, partial [B. mutus]
ENSBTAP00000010364-D1	5.07	4.60E-06	↓	ras-related protein Rab-40C isoform X1 [Orcinus orca]
ENSP00000409384-D1	5.07	4.60E-06	↓	sperm-associated antigen 11-like [B. taurus]
ENSBTAP00000046780-D2	5.07	4.60E-06	↓	hypothetical protein M91_13119, partial [B. mutus]
ENSP00000259006-D1	5.07	4.60E-06	↓	LIM domain-containing protein 2 [B. mutus]
ENSP00000259351-D2	5.07	4.60E-06	↓	ras-specific guanine nucleotide-releasing factor RalGPS1 isoform X2 [Pantholops hodgsonii]
ENSP00000409075-D1	5.07	4.60E-06	↓	NADH-cytochrome b5 reductase-like protein, partial [B. mutus]
ENSP00000360967-D1	5.07	4.60E-06	↓	Sodium- and chloride-dependent neutral and basic amino acid transporter B(0+) [B. mutus]
ENSBTAP00000033782-D1	5.07	4.60E-06	↓	leucine-rich repeat and death domain-containing protein 1 [B. mutus]
ENSP00000253303-D1	5.07	4.60E-06	↓	regucalcin isoform X2 [B. taurus]
ENSP00000248929-D1	5.07	4.60E-06	↓	Small G protein signaling modulator 3, partial [B. mutus]
ENSBTAP00000021935-D1	5.07	4.60E-06	↓	zinc finger protein 740 [B. mutus]
ENSP00000302569-D1	5.07	4.60E-06	↓	C-type lectin domain family 7 member A [B. mutus]
ENSBTAP00000020121-D1	5.07	4.60E-06	↓	Kallikrein-6 [B. mutus]
ENSBTAP00000016904-D1	5.07	4.60E-06	↓	Membrane transport protein XK [B. mutus]
ENSP00000391753-D1	5.07	4.60E-06	↓	Myeloid differentiation primary response protein MyD88, partial [B. mutus]
ENSBTAP00000006983-D1	5.07	4.60E-06	↓	angiopoietin-related protein 5 [B. mutus]
ENSP00000294947-D1	5.07	4.60E-06	↓	uncharacterized protein C2orf61 homolog isoform X2 [B. taurus]
ENSP00000368124-D1	5.07	4.60E-06	↓	Calcium/calmodulin-dependent protein kinase type 1D, partial [B. mutus]
ENSP00000327145-D1	5.07	4.60E-06	↓	Filamin-C [B. mutus]
ENSBTAP00000053602-D2	5.07	4.60E-06	↓	zinc finger protein 41 homolog [Bison bison bison]
ENSP00000345065-D1	5.07	4.60E-06	↓	ankyrin repeat and death domain-containing protein 1B isoform X1 [B. taurus]
ENSP00000407118-D1	5.07	4.60E-06	↓	Syntabulin [B. mutus]

ENSBTAP00000009810-D1	5.07	4.60E-06	↓	Guanylate cyclase soluble subunit beta-2, partial [B. mutus]
ENSP00000385026-D1	5.07	4.60E-06	↓	Neuronal acetylcholine receptor subunit alpha-2 [B. mutus]
ENSBTAP00000028854-D1	5.07	4.60E-06	↓	PDZ domain-containing protein 9 [B. taurus]
ENSP00000317691-D1	5.07	4.60E-06	↓	ribonuclease P protein subunit p25 [B. mutus]
ENSP00000228289-D1	5.07	4.60E-06	↓	Zinc finger protein 268, partial [B. mutus]
ENSP00000418397-D1	5.07	4.60E-06	↓	Isovaleryl-CoA dehydrogenase, mitochondrial, partial [B. mutus]
ENSP00000349087-D1	5.07	4.60E-06	↓	Membralin, partial [B. mutus]
ENSBTAP00000048201-D5	5.07	4.60E-06	↓	histone H2B type 1-J-like [Sorex araneus]
ENSP00000274773-D15	5.07	4.60E-06	↓	E3 ubiquitin-protein ligase TRIM31, partial [B. mutus]
ENSBTAP00000002478-D1	5.07	4.60E-06	↓	Fanconi anemia group A protein [B. mutus]
ENSBTAP00000007294-D1	5.07	4.60E-06	↓	hyaluronidase-4 [B. mutus]
ENSP00000374989-D1	5.07	4.60E-06	↓	Ig alpha-1 chain C region, partial [B. mutus]
ENSBTAP00000049943-D1	5.07	4.60E-06	↓	UPF0452 protein C7orf41 homolog [Ceratotherium simum simum]
ENSP00000321346-D1	5.07	4.60E-06	↓	Tubulin polyglutamylase TTLL11, partial [B. mutus]
ENSP00000365005-D1	5.07	4.60E-06	↓	mitogen-activated protein kinase kinase kinase 19 isoform X1 [B. mutus]
ENSBTAP00000053373-D1	5.07	4.60E-06	↓	low-density lipoprotein receptor-related protein 1B [Bison bison bison]
ENSBTAP00000002568-D1	5.07	4.60E-06	↓	Transcription initiation factor TFIID subunit 7-like protein, partial [B. mutus]
ENSBTAP00000006928-D1	5.07	4.60E-06	↓	Kelch-like protein 2, partial [B. mutus]
ENSP00000359693-D2	5.07	4.60E-06	↓	peptidyl-prolyl cis-trans isomerase FKBP1A-like [B. mutus]
ENSP00000414669-D1	5.07	4.60E-06	↓	putative uncharacterized protein PNAS-138-like [Ceratotherium simum simum]
ENSBTAP00000017999-D1	5.07	4.60E-06	↓	DNA fragmentation factor subunit alpha [B. mutus]
ENSBTAP00000013144-D1	5.07	4.60E-06	↓	3,2-trans-enoyl-CoA isomerase, mitochondrial, partial [B. mutus]
ENSBTAP00000002335-D1	5.07	4.60E-06	↓	28S ribosomal protein S12, mitochondrial [B. mutus]
ENSBTAP00000011810-D1	5.07	4.60E-06	↓	Ankyrin repeat and SOCS box protein 6, partial [B. mutus]
ENSBTAP00000028806-D1	5.07	4.60E-06	↓	Zinc finger CCCH-type antiviral protein 1 [B. mutus]
ENSP00000309163-D1	5.07	4.60E-06	↓	CAAX prenyl protease 2-like isoform X1 [B. mutus]
ENSBTAP00000038873-D2	5.07	4.60E-06	↓	hypothetical protein M91_08020, partial [B. mutus]
ENSBTAP00000025281-D1	5.07	4.60E-06	↓	tumor necrosis factor ligand superfamily member 10 [B. mutus]
ENSBTAP00000023918-D2	5.07	4.60E-06	↓	Histone H2B type F-M, partial [B. mutus]
yakG013051	5.07	4.60E-06	↓	hypothetical protein M91_06530 [B. mutus]
ENSP00000360366-D1	5.07	4.60E-06	↓	acyl-coenzyme A thioesterase 11 [B. mutus]
ENSBTAP00000030051-D1	5.07	4.60E-06	↓	casein kinase I isoform beta-like [B. mutus]
ENSBTAP00000014661-D1	5.07	4.60E-06	↓	adrenocorticotropic hormone receptor [B. mutus]
ENSP00000290399-D1	5.07	4.60E-06	↓	Single-minded-like protein 2, partial [B. mutus]
ENSP00000324560-D1	5.07	4.60E-06	↓	Serine/threonine-protein kinase ULK1, partial [B. mutus]
yakG040722	5.07	4.60E-06	↓	Mastermind-like protein 3 [B. mutus]
ENSP00000337452-D1	5.07	4.60E-06	↓	magnesium transporter NIPA1 [B. taurus]
ENSP00000367462-D1	5.07	4.60E-06	↓	S-acyl fatty acid synthase thioesterase, medium chain, partial [B. mutus]
ENSBTAP00000023182-D1	5.07	4.60E-06	↓	hypothetical protein M91_00499, partial [B. mutus]
ENSP00000366509-D10	5.07	4.60E-06	↓	zinc finger protein 160-like isoform X1 [Ovis aries]
yakG044517	5.07	4.60E-06	↓	Putative overexpressed in colon carcinoma 1 protein-like protein [B. mutus]
ENSBTAP00000022924-D1	5.07	4.60E-06	↓	RPE-spondin, partial [B. mutus]
ENSP00000368312-D1	5.07	4.60E-06	↓	ETS translocation variant 2 [B. mutus]
ENSP00000361598-D1	5.07	4.60E-06	↓	Zinc finger SWIM domain-containing protein 1, partial [B. mutus]
ENSBTAP00000039660-D1	5.07	4.60E-06	↓	Short transient receptor putative channel 3, partial [B. mutus]
ENSBTAP00000006828-D1	5.07	4.60E-06	↓	mevalonate kinase [B. taurus]
ENSP00000358932-D1	5.07	4.60E-06	↓	hypothetical protein M91_04669, partial [B. mutus]
ENSBTAP00000039733-D1	5.07	4.60E-06	↓	interferon beta-2 [B. taurus]
ENSBTAP00000010047-D1	5.07	4.60E-06	↓	ATP-dependent DNA helicase PIF1, partial [B. mutus]
ENSBTAP00000044315-D1	5.07	4.60E-06	↓	leucine-rich repeat-containing protein 3B precursor [B. taurus]
ENSBTAP00000019595-D1	5.07	4.60E-06	↓	CD27 antigen [B. mutus]
ENSBTAP00000047862-D1	5.07	4.60E-06	↓	ribosyldihydronicotinamide dehydrogenase [quinone] [B. mutus]
ENSP00000358784-D1	5.07	4.60E-06	↓	potassium voltage-gated channel, shaker-related subfamily, member 3 [B. mutus]
ENSBTAP00000048532-D1	5.07	4.60E-06	↓	Zinc finger and BTB domain-containing protein 9, partial [B. mutus]
ENSP00000364080-D2	5.07	4.60E-06	↓	hypothetical protein M91_05095 [B. mutus]

ENSBTAP00000031126-D6	5.07	4.60E-06	↓	hypothetical protein M91_05882, partial [B. mutus]
ENSBTAP00000036200-D1	5.07	4.60E-06	↓	collagen alpha-1(XXI) chain isoform X2 [B. taurus]
ENSBTAP00000013674-D1	5.07	4.60E-06	↓	Copper-transporting ATPase 2, partial [B. mutus]
ENSP00000324633-D2	5.07	4.60E-06	↓	beta-defensin 103A-like [B. mutus]
ENSP00000333122-D1	5.07	4.60E-06	↓	Nuclear receptor subfamily 4 group A member 3 [B. mutus]
ENSP00000265175-D1	5.09	2.53E-79	↓	Protein transport protein Sec24B, partial [B. mutus]
ENSBTAP00000048356-D1	5.10	4.40E-52	↓	dynactin subunit 5 [B. taurus]
ENSP00000377612-D1	5.11	3.80E-31	↓	Extended synaptotagmin-1 [B. mutus]
ENSP00000396586-D3	5.12	7.43E-10	↓	hypothetical protein M91_07654 [B. mutus]
ENSBTAP00000011298-D1	5.12	7.43E-10	↓	deleted in lung and esophageal cancer protein 1 [B. mutus]
ENSBTAP00000017423-D1	5.12	7.43E-10	↓	hypothetical protein M91_05472, partial [B. mutus]
ENSP00000349364-D1	5.12	7.43E-10	↓	Intermediate filament family orphan 1, partial [B. mutus]
ENSBTAP00000024208-D1	5.13	1.60E-110	↓	stefin-C-like [B. mutus]
ENSBTAP00000034581-D19	5.13	3.28E-53	↓	histone H3.3 [Mytilus galloprovincialis]
ENSP00000282753-D1	5.14	1.56E-24	↓	metabotropic glutamate receptor 1 [B. taurus]
ENSBTAP00000002665-D1	5.15	2.37E-17	↓	DNA replication licensing factor MCM9, partial [B. mutus]
ENSBTAP00000015850-D1	5.15	2.37E-17	↓	Microsomal triglyceride transfer protein large subunit, partial [B. mutus]
ENSBTAP00000026937-D1	5.16	2.15E-135	↓	protein FAM72A [B. taurus]
ENSBTAP00000053117-D1	5.18	3.86E-10	↓	crossover junction endonuclease MUS81 [B. mutus]
ENSBTAP00000028783-D1	5.18	3.86E-10	↓	Huntingtin-interacting protein 1-related protein, partial [B. mutus]
ENSBTAP00000018681-D1	5.18	3.86E-10	↓	U6 snRNA-associated Sm-like protein LSm6-like [B. mutus]
yakA23725	5.18	2.36E-06	↓	AP-5 complex subunit beta-1 [B. mutus]
ENSBTAP00000049437-D1	5.18	2.36E-06	↓	vomeronasal type-1 receptor 2-like [B. mutus]
ENSP00000369962-D1	5.18	2.36E-06	↓	Immunoglobulin superfamily member 5, partial [B. mutus]
ENSBTAP00000007444-D1	5.18	2.36E-06	↓	leucine-rich repeat-containing protein 20 [B. mutus]
ENSP00000303325-D1	5.18	2.36E-06	↓	neuromedin-K receptor [B. mutus]
ENSP00000341988-D1	5.18	2.36E-06	↓	Protein FAM75D1 [B. mutus]
ENSP00000304895-D1	5.18	2.36E-06	↓	insulin receptor substrate 1 [B. mutus]
ENSBTAP00000005162-D1	5.18	2.36E-06	↓	Transmembrane protein 220, partial [B. mutus]
ENSP00000378227-D1	5.18	2.36E-06	↓	Rab11 family-interacting protein 4, partial [B. mutus]
ENSBTAP00000040741-D1	5.18	2.36E-06	↓	Cytosolic carboxypeptidase 6, partial [B. mutus]
ENSBTAP00000016926-D1	5.18	2.36E-06	↓	C-C motif chemokine 26 [Bison bison bison]
ENSP00000303515-D1	5.18	2.36E-06	↓	tRNA-dihydrouridine(16/17) synthase [NAD(P)(+)]-like [B. mutus]
ENSBTAP00000021898-D1	5.18	2.36E-06	↓	arylamine N-acetyltransferase 1 [B. taurus]
ENSP00000380309-D1	5.18	2.36E-06	↓	AP-1 complex subunit gamma-like 2 isoform X1 [B. mutus]
ENSBTAP00000053531-D8	5.18	2.36E-06	↓	Ephrin type-A receptor 5, partial [B. mutus]
ENSP00000317272-D1	5.18	2.36E-06	↓	Hepatocyte growth factor receptor [B. mutus]
ENSBTAP00000040290-D1	5.18	2.36E-06	↓	Ras-related protein Rab-17, partial [B. mutus]
ENSBTAP00000053414-D1	5.18	2.36E-06	↓	glycine receptor subunit alpha-3 isoform X1 [B. mutus]
ENSBTAP00000026602-D1	5.18	2.36E-06	↓	hypothetical protein M91_17360 [B. mutus]
ENSBTAP00000015064-D1	5.18	2.36E-06	↓	Ankyrin repeat domain-containing protein 33B, partial [B. mutus]
ENSP00000371497-D1	5.18	2.36E-06	↓	complement C1q tumor necrosis factor-related protein 3 isoform X1 [B. taurus]
ENSP00000416922-D1	5.18	2.36E-06	↓	Putative glutamyl-tRNA synthetase, mitochondrial [B. mutus]
ENSP00000254101-D1	5.18	2.36E-06	↓	5'-AMP-activated protein kinase subunit beta-2 [B. taurus]
ENSBTAP00000010198-D1	5.18	2.36E-06	↓	U4/U6 small nuclear ribonucleoprotein Prp31 [B. mutus]
ENSP00000263686-D1	5.18	2.36E-06	↓	P-selectin [Bubalus bubalis]
ENSP00000383760-D1	5.18	2.36E-06	↓	Quinone oxidoreductase-like protein 2, partial [B. mutus]
ENSBTAP00000028327-D1	5.18	2.36E-06	↓	C2 domain-containing protein 2, partial [B. mutus]
ENSBTAP00000021521-D1	5.18	2.36E-06	↓	DNA-binding protein inhibitor ID-1 [B. taurus]
ENSP00000365147-D1	5.18	2.36E-06	↓	EF-hand domain-containing protein D2 [B. mutus]
ENSBTAP00000017829-D1	5.18	2.36E-06	↓	Rho guanine nucleotide exchange factor 40, partial [B. mutus]
yakG015969	5.18	2.36E-06	↓	hypothetical protein M91_21659 [B. mutus]
ENSP00000414649-D1	5.18	2.36E-06	↓	Taste receptor type 2 member 39, partial [B. mutus]
ENSP00000223366-D1	5.18	2.36E-06	↓	Glucokinase, partial [B. mutus]
ENSP00000356140-D2	5.18	2.36E-06	↓	neuronal cell adhesion molecule isoform X8 [Bison bison bison]

ENSP00000395892-D1	5.18	2.36E-06	↓	protein FAM118A isoform X1 [B. mutus]
ENSP00000379255-D1	5.18	2.36E-06	↓	transcriptional activator GLI3 isoform X6 [B. taurus]
ENSP00000375745-D1	5.18	2.36E-06	↓	Presenilin-2, partial [B. mutus]
ENSBTAP00000028637-D1	5.18	2.36E-06	↓	Olfactomedin-like protein 1 [B. mutus]
ENSBTAP00000012210-D1	5.18	2.36E-06	↓	UHRF1-binding protein 1, partial [B. mutus]
ENSBTAP00000022720-D1	5.18	2.36E-06	↓	Growth factor receptor-bound protein 10, partial [B. mutus]
ENSBTAP00000052128-D1	5.18	2.36E-06	↓	Retrotransposon gag domain-containing protein 1, partial [B. mutus]
yakG029648	5.18	2.36E-06	↓	Nuclear factor 1 A-type [B. mutus]
ENSP00000405798-D1	5.18	2.36E-06	↓	Cell division protein kinase 16, partial [B. mutus]
ENSBTAP00000052960-D5	5.18	2.36E-06	↓	interferon omega-1-like [B. mutus]
ENSBTAP00000022366-D1	5.18	2.36E-06	↓	growth-regulated protein homolog alpha precursor [B. taurus]
ENSP00000372649-D3	5.18	2.36E-06	↓	proline-rich protein 23C [B. taurus]
ENSP00000367345-D5	5.18	2.36E-06	↓	Protocadherin gamma-A10, partial [B. mutus]
ENSBTAP00000026199-D1	5.18	2.36E-06	↓	transmembrane and ubiquitin-like domain-containing protein 2 isoform X2 [B. taurus]
ENSBTAP00000004948-D1	5.18	2.36E-06	↓	Threonyl-tRNA synthetase, mitochondrial [B. mutus]
ENSBTAP00000037596-D17	5.18	2.36E-06	↓	Trace amine-associated receptor 6, partial [B. mutus]
ENSBTAP00000013662-D1	5.18	2.36E-06	↓	Parkinson disease 7 domain-containing protein 1, partial [B. mutus]
ENSBTAP00000006339-D1	5.18	2.36E-06	↓	Tartrate-resistant acid phosphatase type 5, partial [B. mutus]
ENSBTAP00000009013-D1	5.18	2.36E-06	↓	Homeobox protein Meis3, partial [B. mutus]
ENSP00000396857-D1	5.18	2.36E-06	↓	Zinc finger protein 697 [B. mutus]
ENSP00000297313-D1	5.18	2.36E-06	↓	Regulator of G-protein signaling 20 [B. mutus]
ENSP00000246229-D1	5.18	2.36E-06	↓	zinc finger protein PLAGL2 isoform X1 [B. taurus]
ENSBTAP00000001633-D1	5.18	2.36E-06	↓	myelin protein zero-like protein 2 precursor [B. taurus]
ENSBTAP00000008061-D1	5.18	2.36E-06	↓	General transcription factor 3C polypeptide 1, partial [B. mutus]
ENSBTAP00000022832-D2	5.18	2.36E-06	↓	MACRO domain-containing protein 1, partial [B. mutus]
ENSBTAP00000034579-D1	5.18	2.36E-06	↓	Alpha-N-acetylgalactosaminide alpha-2,6-sialyltransferase 1, partial [B. mutus]
ENSBTAP00000018336-D1	5.18	2.36E-06	↓	receptor-type tyrosine-protein phosphatase-like N isoform X1 [B. mutus]
ENSP00000282588-D1	5.18	2.36E-06	↓	integrin alpha-1 [B. mutus]
ENSP00000270223-D1	5.18	2.36E-06	↓	dystrophia myotonica WD repeat-containing protein isoform X2 [B. taurus]
ENSBTAP00000004012-D1	5.18	2.36E-06	↓	RAG1-activating protein 1 [B. mutus]
ENSBTAP00000051552-D1	5.18	2.36E-06	↓	Trypsin-X3 [B. mutus]
ENSP00000293760-D1	5.18	2.36E-06	↓	LEM domain-containing protein 2, partial [B. mutus]
ENSP00000231173-D8	5.18	2.36E-06	↓	Protocadherin gamma-A9, partial [B. mutus]
ENSP00000330523-D1	5.18	2.36E-06	↓	Collagen triple helix repeat-containing protein 1, partial [B. mutus]
ENSP00000385118-D1	5.18	2.36E-06	↓	Proline-rich protein 16, partial [B. mutus]
ENSBTAP00000014593-D1	5.18	2.36E-06	↓	Cathepsin H, partial [B. mutus]
ENSBTAP00000026613-D1	5.18	2.36E-06	↓	UMP-CMP kinase 2, mitochondrial, partial [B. mutus]
ENSP00000368405-D1	5.18	2.36E-06	↓	hypothetical protein M91_16071, partial [B. mutus]
ENSBTAP00000020860-D1	5.18	2.36E-06	↓	Protein BTG2, partial [B. mutus]
ENSP00000245564-D1	5.18	2.36E-06	↓	protein misato homolog 1-like isoform X1 [B. mutus]
ENSP00000274487-D1	5.18	2.36E-06	↓	A disintegrin and metalloproteinase with thrombospondin motifs 19, partial [B. mutus]
ENSBTAP00000050080-D12	5.18	2.36E-06	↓	endogenous retrovirus group K member 11 Pol protein-like [Bison bison bison]
ENSP00000317027-D1	5.18	2.36E-06	↓	beta-1,3-galactosyl-O-glycosyl-glycoprotein beta-1,6-N-acetylglucosaminyltransferase 4 [B. mutus]
ENSP00000256398-D2	5.18	5.85E-85	↓	Elongator complex protein 3, partial [B. mutus]
ENSBTAP00000021952-D1	5.18	6.47E-130	↓	Polymerase delta-interacting protein 3, partial [B. mutus]
ENSBTAP00000046275-D1	5.20	2.13E-161	↓	uncharacterized protein C4orf21 homolog [B. mutus]
ENSP00000394331-D1	5.20	6.44E-18	↓	COMM domain-containing protein 5 [B. taurus]
ENSP00000265023-D1	5.21	1.16E-25	↓	Kininogen-1 [B. mutus]
ENSP00000317123-D1	5.21	1.16E-25	↓	U5 small nuclear ribonucleoprotein 200 kDa helicase [B. mutus]
ENSBTAP00000026189-D1	5.22	2.11E-33	↓	serine/threonine-protein kinase 26 isoform X1 [Nomascus leucogenys]
ENSBTAP00000015828-D1	5.23	2.01E-10	↓	plectin [B. mutus]
ENSBTAP00000050657-D1	5.23	2.01E-10	↓	Ubiquitin carboxyl-terminal hydrolase 43, partial [B. mutus]
ENSP00000312399-D1	5.23	2.01E-10	↓	Coiled-coil domain-containing protein 63, partial [B. mutus]
ENSBTAP00000006600-D1	5.24	0.00E+00	↓	Heat shock protein 105 kDa [B. mutus]
ENSP00000267970-D3	5.25	8.30E-136	↓	tetraspanin-3 [B. taurus]

ENSBTAP00000015942-D1	5.25	1.77E-18	↓	lemur tyrosine kinase 3 [B. mutus]
ENSP00000284073-D1	5.25	1.77E-18	↓	RNA-binding protein Musashi-like protein 2, partial [B. mutus]
ENSP00000356057-D1	5.28	9.29E-19	↓	Transmembrane protein 181, partial [B. mutus]
ENSP00000384903-D1	5.28	9.29E-19	↓	Lysine-specific demethylase 2A [B. mutus]
ENSP00000361725-D1	5.28	1.05E-10	↓	endonuclease G [B. mutus]
ENSBTAP00000002020-D1	5.28	1.05E-10	↓	BRI3-binding protein, partial [B. mutus]
ENSBTAP00000004317-D1	5.28	1.22E-06	↓	Baculoviral IAP repeat-containing protein 1 [B. mutus]
ENSP00000391121-D1	5.28	1.22E-06	↓	Retinol dehydrogenase 13 [B. mutus]
ENSBTAP00000001896-D1	5.28	1.22E-06	↓	histamine H1 receptor isoform X1 [B. mutus]
ENSP00000336854-D1	5.28	1.22E-06	↓	epimerase family protein SDR39U1 [B. mutus]
ENSP00000377112-D1	5.28	1.22E-06	↓	Solute carrier family 12 member 8, partial [B. mutus]
ENSBTAP00000030588-D1	5.28	1.22E-06	↓	death domain-containing membrane protein NRADD-like [B. mutus]
yakG003570	5.28	1.22E-06	↓	hypothetical protein M91_15334 [B. mutus]
ENSBTAP00000022612-D1	5.28	1.22E-06	↓	ranBP-type and C3HC4-type zinc finger-containing protein 1 isoform X1 [B. mutus]
ENSP00000396732-D3	5.28	1.22E-06	↓	histone-lysine N-methyltransferase PRDM9-like [Bubalus bubalis]
ENSP00000303540-D1	5.28	1.22E-06	↓	sodium channel protein type 3 subunit alpha [B. mutus]
ENSBTAP00000053268-D1	5.28	1.22E-06	↓	Astrotactin-1, partial [B. mutus]
ENSBTAP00000001142-D1	5.28	1.22E-06	↓	solute carrier family 52, riboflavin transporter, member 2 [B. mutus]
ENSP00000281525-D1	5.28	1.22E-06	↓	tryptophan 2,3-dioxygenase [B. taurus]
ENSBTAP00000011922-D1	5.28	1.22E-06	↓	transmembrane protein 198-like isoform X1 [B. mutus]
ENSBTAP00000003504-D1	5.28	1.22E-06	↓	Meiotic recombination protein REC8-like protein [B. mutus]
ENSP00000267085-D1	5.28	1.22E-06	↓	Cysteine sulfinic acid decarboxylase, partial [B. mutus]
ENSBTAP00000008930-D1	5.28	1.22E-06	↓	hypothetical protein M91_13886 [B. mutus]
yakA21558	5.28	1.22E-06	↓	Histone H4 [Heterocephalus glaber]
ENSBTAP00000018815-D1	5.28	1.22E-06	↓	Tubulin--tyrosine ligase, partial [B. mutus]
ENSP00000228922-D1	5.28	1.22E-06	↓	2-oxoglutarate and iron-dependent oxygenase domain-containing protein 2 [B. mutus]
ENSBTAP00000014031-D1	5.28	1.22E-06	↓	ADP-ribosylation factor-like protein 9, partial [B. mutus]
ENSP00000324633-D4	5.28	1.22E-06	↓	Beta-defensin 103A, partial [B. mutus]
ENSP00000357973-D1	5.28	1.22E-06	↓	lactation elevated protein 1, partial [B. mutus]
ENSP00000380144-D1	5.28	1.22E-06	↓	Zinc fingers and homeoboxes protein 3, partial [B. mutus]
ENSP00000365548-D1	5.28	1.22E-06	↓	procollagen-lysine,2-oxoglutarate 5-dioxygenase 1-like [Capra hircus]
ENSBTAP00000004192-D1	5.28	1.22E-06	↓	Ras-related protein Rab-7L1, partial [B. mutus]
ENSP00000351363-D1	5.28	1.22E-06	↓	Beta-microseminoprotein, partial [B. mutus]
ENSBTAP00000038143-D1	5.28	1.22E-06	↓	Aldehyde oxidase, partial [B. mutus]
ENSBTAP00000027857-D1	5.28	1.22E-06	↓	tyrosine-protein kinase JAK3 [B. mutus]
ENSBTAP00000009466-D1	5.28	1.22E-06	↓	guanylin precursor [B. taurus]
ENSBTAP00000027446-D1	5.28	1.22E-06	↓	Rho-related GTP-binding protein RhoD, partial [B. mutus]
ENSBTAP00000013600-D1	5.28	1.22E-06	↓	Dehydrogenase/reductase SDR family member 11, partial [B. mutus]
ENSBTAP00000001120-D1	5.28	1.22E-06	↓	Myosin-3 [B. mutus]
ENSBTAP00000005055-D1	5.28	1.22E-06	↓	syntaxin-16 isoform X3 [B. taurus]
ENSP00000382178-D1	5.28	1.22E-06	↓	MAM domain-containing glycosylphosphatidylinositol anchor protein 2 isoform X2 [B. taurus]
ENSP00000294811-D1	5.28	1.22E-06	↓	hypothetical protein M91_06962, partial [B. mutus]
ENSP00000230732-D1	5.28	1.22E-06	↓	POU domain, class 4, transcription factor 3 [B. taurus]
ENSP00000258883-D1	5.28	1.22E-06	↓	A disintegrin and metalloproteinase with thrombospondin motifs 7-like [B. mutus]
ENSBTAP00000035333-D9	5.28	1.22E-06	↓	hypothetical protein M91_04557, partial [B. mutus]
ENSP00000409937-D1	5.28	1.22E-06	↓	Amyotrophic lateral sclerosis 2 chromosomal region candidate 11 protein [B. mutus]
ENSP00000239830-D1	5.28	1.22E-06	↓	Coiled-coil domain-containing protein 77 [B. mutus]
ENSBTAP00000024714-D1	5.28	1.22E-06	↓	Epidermal retinol dehydrogenase 2 [B. mutus]
ENSBTAP00000009611-D3	5.28	1.22E-06	↓	Ras-related protein Rab-3D, partial [B. mutus]
ENSP00000368077-D1	5.28	1.22E-06	↓	C-C motif chemokine 19, partial [B. mutus]
ENSP00000346283-D1	5.28	1.22E-06	↓	histidine-rich carboxyl terminus protein 1 [B. taurus]
ENSP00000333711-D2	5.28	1.22E-06	↓	hypothetical protein M91_15279 [B. mutus]
ENSBTAP00000008128-D1	5.28	1.22E-06	↓	Kinetochore protein Spc24, partial [B. mutus]
ENSP00000376910-D2	5.28	1.22E-06	↓	leucine-rich repeat and IQ domain-containing protein 1-like, partial [B. mutus]
ENSBTAP00000026757-D1	5.28	1.22E-06	↓	Protein FAM71E1, partial [B. mutus]

ENSP00000304401-D1	5.28	1.22E-06	↓	Schlafen-like protein 1, partial [B. mutus]
ENSP00000304151-D1	5.28	1.22E-06	↓	Ribosome biogenesis protein BOP1, partial [B. mutus]
ENSP00000320025-D2	5.28	1.22E-06	↓	Voltage-dependent L-type calcium channel subunit beta-1, partial [B. mutus]
ENSP00000420778-D1	5.28	1.22E-06	↓	lactosylceramide 1,3-N-acetyl-beta-D-glucosaminyltransferase [B. mutus]
ENSBTAP00000040764-D1	5.28	1.22E-06	↓	Peroxidasin-like protein, partial [B. mutus]
ENSBTAP00000050518-D4	5.28	1.22E-06	↓	hypothetical protein M91_11552, partial [B. mutus]
ENSP00000268864-D1	5.28	1.22E-06	↓	ras-like protein family member 10B [B. taurus]
ENSBTAP00000021191-D1	5.28	1.22E-06	↓	iodotyrosine dehalogenase 1 [B. mutus]
yakG012614	5.28	1.22E-06	↓	hypothetical protein M91_12206 [B. mutus]
ENSP00000414276-D1	5.28	1.22E-06	↓	cysteine-rich DPF motif domain-containing protein 1 isoform X1 [Bison bison bison]
ENSBTAP00000034433-D1	5.28	1.22E-06	↓	B(0,+)-type amino acid transporter 1 [B. mutus]
ENSBTAP00000012044-D1	5.28	1.22E-06	↓	low-density lipoprotein receptor class A domain-containing protein 4 [B. mutus]
ENSP00000393252-D1	5.28	1.22E-06	↓	LOC401385 [Homo sapiens]
ENSP00000352336-D1	5.28	1.22E-06	↓	1-phosphatidylinositol-4,5-bisphosphate phosphodiesterase gamma-2, partial [B. mutus]
ENSBTAP00000018029-D1	5.28	1.22E-06	↓	Guanine nucleotide exchange factor MSS4, partial [B. mutus]
ENSBTAP00000013855-D1	5.28	1.22E-06	↓	small integral membrane protein 3 [B. mutus]
ENSBTAP00000053450-D1	5.28	1.22E-06	↓	Histone deacetylase 5, partial [B. mutus]
ENSP00000360642-D1	5.28	1.22E-06	↓	Coiled-coil and C2 domain-containing protein 1B [B. mutus]
ENSBTAP00000050033-D1	5.28	1.22E-06	↓	Putative prolyl-tRNA synthetase, mitochondrial, partial [B. mutus]
ENSBTAP00000034629-D1	5.28	1.22E-06	↓	zinc finger protein 750 [B. mutus]
ENSP00000368758-D1	5.28	1.22E-06	↓	cortexin-3 [B. taurus]
ENSBTAP00000043456-D1	5.28	1.22E-06	↓	estradiol 17-beta-dehydrogenase 1 [B. taurus]
ENSP00000334040-D1	5.30	1.51E-124	↓	Round spermatid basic protein 1-like protein [B. mutus]
ENSBTAP00000032900-D1	5.32	1.72E-52	↓	rhomboid-related protein 4 [B. mutus]
ENSP00000358105-D1	5.32	2.55E-19	↓	gamma-secretase subunit APH-1A isoform X1 [B. mutus]
ENSBTAP00000018791-D2	5.32	2.55E-19	↓	WD repeat-containing protein 17 isoform X1 [B. taurus]
ENSP00000304108-D1	5.32	5.48E-11	↓	protein FAM171B [Bison bison bison]
ENSBTAP00000000437-D1	5.32	5.48E-11	↓	ETS domain-containing transcription factor ERF [Ictidomys tridecemlineatus]
ENSP00000357088-D1	5.32	5.48E-11	↓	dual specificity protein phosphatase 23 [B. taurus]
ENSP00000407163-D1	5.32	5.48E-11	↓	pleckstrin homology domain-containing family M member 2 [B. mutus]
ENSP00000264977-D1	5.32	5.48E-11	↓	serine/threonine-protein phosphatase 2A regulatory subunit B'' subunit alpha isoform X1 [B. mutus]
ENSBTAP00000005179-D1	5.33	0.00E+00	↓	MIT domain-containing protein 1 [B. mutus]
ENSP00000262498-D1	5.34	0.00E+00	↓	UPF0468 protein C16orf80 homolog [Chrysochloris asiatica]
ENSP00000402092-D1	5.34	9.20E-45	↓	Sterile alpha motif domain-containing protein 3, partial [B. mutus]
ENSBTAP00000051910-D1	5.35	1.35E-19	↓	hypothetical protein M91_01141 [B. mutus]
ENSBTAP00000025456-D1	5.35	1.35E-19	↓	excitatory amino acid transporter 3 [B. mutus]
ENSP00000263401-D1	5.35	1.35E-19	↓	COMM domain-containing protein 9 [B. mutus]
ENSBTAP00000002496-D1	5.36	9.36E-37	↓	syntaxin-binding protein 5-like [B. taurus]
ENSP00000364476-D1	5.37	7.08E-20	↓	RIB43A-like with coiled-coils protein 1 [B. mutus]
ENSP00000262764-D1	5.37	2.88E-11	↓	CDP-diacylglycerol--glycerol-3-phosphate 3-phosphatidyltransferase, mitochondrial, partial [B. mutus]
ENSBTAP00000010538-D1	5.37	2.88E-11	↓	Serine/threonine-protein phosphatase 5 [B. mutus]
ENSBTAP00000013576-D1	5.37	6.30E-07	↓	O(6)-methylguanine-induced apoptosis 2 [B. mutus]
ENSBTAP00000017410-D1	5.37	6.30E-07	↓	forkhead box protein N1 isoform X1 [B. taurus]
ENSP00000357789-D3	5.37	6.30E-07	↓	Filaggrin, partial [B. mutus]
ENSP00000397297-D1	5.37	6.30E-07	↓	Neurotrophin-3, partial [B. mutus]
ENSBTAP00000049636-D2	5.37	6.30E-07	↓	myosin light chain 4 [Pantholops hodgsonii]
yakA08541	5.37	6.30E-07	↓	hypothetical protein M91_14536 [B. mutus]
ENSP00000333534-D1	5.37	6.30E-07	↓	cytochrome P450 2F3-like [B. mutus]
ENSBTAP00000005861-D1	5.37	6.30E-07	↓	hypothetical protein M91_07977, partial [B. mutus]
ENSP00000303992-D1	5.37	6.30E-07	↓	Transmembrane protein 43, partial [B. mutus]
ENSP00000379689-D96	5.37	6.30E-07	↓	hypothetical protein M91_11867, partial [B. mutus]
ENSBTAP00000037325-D1	5.37	6.30E-07	↓	ATP-binding cassette sub-family A member 3, partial [B. mutus]
ENSBTAP00000029241-D1	5.37	6.30E-07	↓	all-trans-retinol 13,14-reductase [B. mutus]
ENSP00000347538-D1	5.37	6.30E-07	↓	Synaptotagmin-17, partial [B. mutus]
ENSBTAP00000038868-D82	5.37	6.30E-07	↓	mariner transposase [Homo sapiens]

ENSBTAP00000053793-D1	5.37	6.30E-07	↓	methionine sulfoxide reductase B2 [B. mutus]
yakA00814	5.37	6.30E-07	↓	Exocyst complex component 1 [B. mutus]
ENSP00000343819-D1	5.37	6.30E-07	↓	homeobox protein OTX2 isoformX1 [Sus scrofa]
ENSP00000307183-D2	5.37	6.30E-07	↓	Inhibitor of growth protein 1, partial [B. mutus]
ENSBTAP00000004260-D1	5.37	6.30E-07	↓	Sodium/hydrogen exchanger 7, partial [B. mutus]
ENSP00000417515-D1	5.37	6.30E-07	↓	maltase-glucoamylase, intestinal isoform X4 [B. taurus]
ENSBTAP00000001513-D1	5.37	6.30E-07	↓	hypothetical protein M91_05344, partial [B. mutus]
ENSBTAP00000028218-D1	5.37	6.30E-07	↓	interphotoreceptor matrix proteoglycan 1 [Bison bison bison]
ENSBTAP00000042513-D1	5.37	6.30E-07	↓	WD repeat-containing protein 88, partial [B. mutus]
ENSP00000411465-D1	5.37	6.30E-07	↓	cystinosin isoform X2 [B. taurus]
ENSP00000408119-D3	5.37	6.30E-07	↓	putative RNA polymerase II subunit A C-terminal domain phosphatase SSU72-like protein 2-like [B. mutus]
ENSP00000356218-D1	5.37	6.30E-07	↓	NADH-cytochrome b5 reductase 1, partial [B. mutus]
ENSBTAP00000038104-D1	5.37	6.30E-07	↓	apomucin-like, partial [Bison bison bison]
ENSP00000319303-D1	5.37	6.30E-07	↓	X-ray radiation resistance-associated protein 1 isoform X1 [B. mutus]
ENSBTAP00000018269-D1	5.37	6.30E-07	↓	rho-related GTP-binding protein RhoQ isoform X1 [Condylura cristata]
ENSBTAP00000012835-D1	5.37	6.30E-07	↓	Fructose-1,6-bisphosphatase 1, partial [B. mutus]
ENSBTAP00000023397-D1	5.37	6.30E-07	↓	Triggering receptor expressed on myeloid cells 1, partial [B. mutus]
ENSP00000378804-D1	5.37	6.30E-07	↓	Spermidine/spermine N(1)-acetyltransferase-like protein 1, partial [B. mutus]
ENSP00000257632-D1	5.37	6.30E-07	↓	uroplakin-3b isoform X3 [Bubalus bubalis]
ENSBTAP00000048971-D1	5.37	6.30E-07	↓	Follicle-stimulating hormone receptor, partial [B. mutus]
ENSP00000348069-D1	5.37	6.30E-07	↓	sterol regulatory element-binding protein 1 [B. mutus]
ENSBTAP00000041770-D8	5.37	6.30E-07	↓	Tubulin beta-4 chain [B. mutus]
ENSBTAP00000035220-D1	5.37	6.30E-07	↓	Oligodendrocyte myelin glycoprotein [B. taurus]
ENSP00000360916-D2	5.37	6.30E-07	↓	Proto-oncogene vav [B. mutus]
ENSP00000396551-D1	5.37	6.30E-07	↓	hypothetical protein M91_03751, partial [B. mutus]
ENSBTAP00000051287-D1	5.37	6.30E-07	↓	Ornithine decarboxylase 2, partial [B. mutus]
ENSBTAP00000007678-D1	5.37	6.30E-07	↓	serine/threonine-protein kinase ULK3 [B. mutus]
ENSBTAP00000004815-D1	5.37	6.30E-07	↓	Bicaudal D-related protein 1 [B. mutus]
ENSBTAP00000031070-D1	5.37	6.30E-07	↓	Cytosolic non-specific dipeptidase, partial [B. mutus]
ENSBTAP00000026158-D1	5.37	6.30E-07	↓	Scavenger receptor class A member 5 [B. mutus]
yakG035046	5.37	6.30E-07	↓	Alpha-N-acetylgalactosaminide alpha-2,6-sialyltransferase 3 [B. mutus]
ENSBTAP00000004059-D1	5.37	6.30E-07	↓	Solute carrier family 22 member 1, partial [B. mutus]
ENSP00000263665-D1	5.37	6.30E-07	↓	Contactin-3, partial [B. mutus]
ENSBTAP00000002785-D1	5.37	6.30E-07	↓	cytochrome P450 4V2-like [B. mutus]
ENSP00000362285-D1	5.37	6.30E-07	↓	equilibrative nucleoside transporter 3 [B. mutus]
ENSBTAP00000044861-D1	5.37	6.30E-07	↓	hypothetical protein M91_05965 [B. mutus]
ENSBTAP00000047675-D2	5.37	6.30E-07	↓	Solute carrier family 7 member 13, partial [B. mutus]
ENSBTAP00000034182-D1	5.37	6.30E-07	↓	SH2 domain-containing adapter protein B [B. taurus]
ENSP00000335332-D1	5.37	6.30E-07	↓	Transmembrane protease serine 12 [B. mutus]
ENSP00000331327-D1	5.37	6.30E-07	↓	Wilms tumor protein isoform X1 [Otolemur garnettii]
ENSP00000378871-D1	5.37	6.30E-07	↓	KN motif and ankyrin repeat domain-containing protein 4 [B. mutus]
ENSBTAP00000002583-D1	5.37	6.30E-07	↓	MAP7 domain-containing protein 1, partial [B. mutus]
yakA21555	5.37	6.30E-07	↓	histone H2B type 1-like [B. mutus]
ENSBTAP00000004887-D1	5.37	6.30E-07	↓	StAR-related lipid transfer protein 9, partial [B. mutus]
ENSP00000392855-D1	5.37	6.30E-07	↓	Membrane-associated tyrosine- and threonine-specific cdc2-inhibitory kinase, partial [B. mutus]
ENSP00000357298-D1	5.37	6.30E-07	↓	rho guanine nucleotide exchange factor 2 isoform X5 [Bubalus bubalis]
ENSBTAP00000024993-D1	5.37	6.30E-07	↓	inactive hydroxysteroid dehydrogenase-like protein 1 [B. taurus]
ENSP00000419740-D1	5.37	6.30E-07	↓	5-methylcytosine rRNA methyltransferase NSUN4 [B. taurus]
ENSBTAP00000028109-D1	5.37	6.30E-07	↓	tumor necrosis factor receptor superfamily member 6B precursor [B. taurus]
ENSP00000377303-D1	5.37	6.30E-07	↓	N-acylglucosamine 2-epimerase, partial [B. mutus]
ENSP00000319052-D1	5.37	6.30E-07	↓	centrin-1 [B. taurus]
ENSP00000297504-D1	5.37	6.30E-07	↓	ATP-binding cassette sub-family B member 8, mitochondrial [B. mutus]
ENSP00000256460-D1	5.37	6.30E-07	↓	Calcium/calmodulin-dependent protein kinase type 1 [B. mutus]
ENSP00000341083-D1	5.37	6.30E-07	↓	atrial natriuretic peptide receptor 2 [B. mutus]
ENSBTAP00000045361-D1	5.37	6.30E-07	↓	hypothetical protein M91_20371, partial [B. mutus]

ENSBTAP00000011866-D1	5.37	6.30E-07	↓	Uroplakin-1b, partial [B. mutus]
ENSP00000343395-D1	5.37	6.30E-07	↓	Pro-neuregulin-1, membrane-bound isoform, partial [B. mutus]
ENSBTAP00000001322-D1	5.38	1.47E-71	↓	zinc transporter ZIP6 isoform X1 [B. taurus]
ENSP00000376616-D14	5.38	4.08E-297	↓	Zinc finger protein 135 [B. mutus]
ENSP00000297459-D1	5.38	9.75E-29	↓	transmembrane protein 74 [B. mutus]
ENSBTAP00000019089-D1	5.41	1.97E-20	↓	MYCBP-associated protein, partial [B. mutus]
ENSP00000323926-D1	5.41	1.51E-11	↓	Transient receptor putative cation channel subfamily M member 8, partial [B. mutus]
ENSBTAP00000043560-D1	5.43	8.10E-208	↓	intraflagellar transport protein 81 homolog [B. taurus]
ENSBTAP00000005789-D1	5.44	9.12E-66	↓	Nuclear migration protein nudC, partial [B. mutus]
ENSBTAP00000009149-D1	5.46	5.49E-21	↓	Cysteine desulfurase, mitochondrial, partial [B. mutus]
ENSP00000258960-D3	5.46	5.49E-21	↓	TPA: glycylpeptide N-tetradecanoyltransferase 1 [B. taurus]
ENSP00000261534-D1	5.46	3.28E-07	↓	Protein O-mannosyl-transferase 2, partial [B. mutus]
ENSP00000321406-D76	5.46	3.28E-07	↓	hypothetical protein M91_10832, partial [B. mutus]
ENSBTAP00000047422-D1	5.46	3.28E-07	↓	general transcription factor IIF subunit 2-like [B. mutus]
ENSBTAP00000012030-D1	5.46	3.28E-07	↓	Aldehyde dehydrogenase family 1 member A3, partial [B. mutus]
ENSBTAP00000028023-D1	5.46	3.28E-07	↓	aryl hydrocarbon receptor nuclear translocator isoform X1 [Bubalus bubalis]
ENSBTAP00000012190-D1	5.46	3.28E-07	↓	killer cell lectin-like receptor 2-like [B. mutus]
ENSP00000385253-D1	5.46	3.28E-07	↓	egl nine homolog 2 isoform X2 [B. taurus]
ENSBTAP00000023241-D1	5.46	3.28E-07	↓	Inositol-3-phosphate synthase 1, partial [B. mutus]
ENSP00000343418-D1	5.46	3.28E-07	↓	semaphorin-4D isoform X1 [B. mutus]
ENSBTAP00000011391-D1	5.46	3.28E-07	↓	Putative E3 ubiquitin-protein ligase TRIML2, partial [B. mutus]
ENSBTAP00000050906-D7	5.46	3.28E-07	↓	Putative PRAME family member 24, partial [B. mutus]
ENSBTAP00000004943-D1	5.46	3.28E-07	↓	Leucine-rich repeat-containing protein 27 [B. mutus]
ENSBTAP00000044748-D1	5.46	3.28E-07	↓	gamma-1-syntrophin [B. mutus]
ENSBTAP00000005226-D1	5.46	3.28E-07	↓	cold shock domain-containing protein C2 [B. taurus]
ENSP00000301891-D1	5.46	3.28E-07	↓	solute carrier family 22 (organic anion/urate transporter), member 11 [B. mutus]
ENSBTAP00000038062-D4	5.46	3.28E-07	↓	NADH dehydrogenase [ubiquinone] 1 alpha subcomplex subunit 4-like 2 [B. mutus]
ENSBTAP00000002339-D1	5.46	3.28E-07	↓	hydroxylysine kinase [B. mutus]
ENSBTAP00000028426-D1	5.46	3.28E-07	↓	Transmembrane 4 L6 family member 20, partial [B. mutus]
ENSP00000368119-D1	5.46	3.28E-07	↓	Galactose-1-phosphate uridylyltransferase, partial [B. mutus]
ENSBTAP00000027772-D1	5.46	3.28E-07	↓	Kallikrein-5 [B. mutus]
ENSBTAP00000004994-D1	5.46	3.28E-07	↓	Microfibrillar-associated protein 2, partial [B. mutus]
ENSP00000397197-D1	5.46	3.28E-07	↓	Inhibin beta A chain [B. mutus]
ENSBTAP00000025111-D1	5.46	3.28E-07	↓	Eukaryotic translation initiation factor 4E type 3, partial [B. mutus]
ENSP00000223214-D1	5.46	3.28E-07	↓	Secreted frizzled-related protein 4, partial [B. mutus]
ENSP00000323071-D1	5.46	3.28E-07	↓	Acyl-coenzyme A thioesterase 4, partial [B. mutus]
ENSP00000394587-D1	5.46	3.28E-07	↓	Vacuolar ATPase assembly integral membrane protein VMA21, partial [B. mutus]
ENSBTAP00000049429-D1	5.46	3.28E-07	↓	Serine protease inhibitor Kazal-type 4, partial [B. mutus]
ENSP00000388446-D1	5.46	3.28E-07	↓	reelin isoform X1 [B. taurus]
ENSP00000370673-D1	5.46	3.28E-07	↓	MANSC domain-containing protein ENSP00000370673, partial [B. mutus]
ENSBTAP00000007488-D1	5.46	3.28E-07	↓	Mitogen-activated protein kinase kinase kinase 9, partial [B. mutus]
ENSP00000353099-D6	5.46	3.28E-07	↓	DLA class II histocompatibility antigen, DR-1 beta chain [B. mutus]
ENSP00000252813-D1	5.46	3.28E-07	↓	Pyroglutamyl-peptidase 1, partial [B. mutus]
ENSBTAP00000050581-D1	5.46	3.28E-07	↓	ribose-phosphate pyrophosphokinase 1-like [B. mutus]
yakG026351	5.46	3.28E-07	↓	hypothetical protein M91_13896 [B. mutus]
ENSBTAP00000052032-D1	5.46	3.28E-07	↓	natural cytotoxicity triggering receptor 3 [B. mutus]
ENSBTAP00000041556-D12	5.46	3.28E-07	↓	hypothetical protein M91_05137, partial [B. mutus]
ENSP00000355082-D1	5.46	3.28E-07	↓	Protocadherin-18, partial [B. mutus]
ENSP00000358238-D1	5.46	3.28E-07	↓	gap junction alpha-8 protein [B. mutus]
ENSBTAP00000007857-D1	5.46	3.28E-07	↓	vesicle transport protein SFT2C [Ovis aries]
ENSBTAP00000015499-D1	5.46	3.28E-07	↓	histone H1.4-like [B. mutus]
ENSP00000368686-D1	5.46	3.28E-07	↓	Transcription factor E2F4 [B. mutus]
ENSBTAP00000045189-D1	5.46	3.28E-07	↓	putative protein TPRXL [Bison bison bison]
ENSBTAP00000005986-D1	5.46	3.28E-07	↓	Dehydrogenase/reductase SDR family member 9, partial [B. mutus]
ENSBTAP00000026311-D1	5.46	3.28E-07	↓	chromosome transmission fidelity protein 18 homolog [B. mutus]

ENSBTAP00000023059-D1	5.46	3.28E-07	↓	kinesin-like protein KIF2B-like [B. mutus]
ENSP00000337653-D1	5.46	3.28E-07	↓	tetratricopeptide repeat protein 8 isoform X2 [B. taurus]
ENSBTAP00000009938-D1	5.46	3.28E-07	↓	potassium voltage-gated channel subfamily A member 5 [B. mutus]
ENSBTAP00000017069-D1	5.46	3.28E-07	↓	Niban-like protein 1, partial [B. mutus]
ENSBTAP00000018635-D1	5.46	3.28E-07	↓	Inositol hexakisphosphate and diphosphoinositol-pentakisphosphate kinase 1, partial [B. mutus]
ENSP00000376564-D1	5.46	3.28E-07	↓	Na(+)/H(+) exchange regulatory cofactor NHE-RF4 [B. mutus]
ENSBTAP00000014143-D1	5.46	3.28E-07	↓	epithelial discoidin domain-containing receptor 1 isoform X2 [B. mutus]
ENSP00000321468-D1	5.46	3.28E-07	↓	hypothetical protein M91_20309, partial [B. mutus]
ENSBTAP00000021782-D1	5.46	3.28E-07	↓	Low-density lipoprotein receptor-related protein 10, partial [B. mutus]
ENSBTAP00000006187-D1	5.46	3.28E-07	↓	Mast cell-expressed membrane protein 1, partial [B. mutus]
ENSP00000264218-D1	5.46	3.28E-07	↓	Neuromedin-U, partial [B. mutus]
ENSP00000236495-D1	5.46	3.28E-07	↓	Sodium/glucose cotransporter 4 [B. mutus]
ENSBTAP00000041573-D5	5.46	3.28E-07	↓	hypothetical protein M91_17490, partial [B. mutus]
ENSP00000365840-D1	5.46	3.28E-07	↓	ectonucleoside triphosphate diphosphohydrolase 6 [B. mutus]
ENSP00000351632-D1	5.46	3.28E-07	↓	Protein FAM3D, partial [B. mutus]
ENSBTAP00000025331-D1	5.46	3.28E-07	↓	ras-associating and dilute domain-containing protein [B. mutus]
ENSBTAP00000040365-D1	5.46	3.28E-07	↓	hypothetical protein M91_04484, partial [B. mutus]
ENSBTAP00000009302-D1	5.46	3.28E-07	↓	large neutral amino acids transporter small subunit 2 [B. mutus]
ENSP00000362258-D1	5.46	3.28E-07	↓	UNC5C-like protein [B. mutus]
ENSP00000307449-D1	5.46	3.28E-07	↓	hypothetical protein M91_02616, partial [B. mutus]
ENSBTAP00000010078-D1	5.46	3.28E-07	↓	Zinc finger protein 414, partial [B. mutus]
ENSBTAP00000053246-D1	5.46	3.28E-07	↓	bone morphogenetic protein 7 precursor [B. taurus]
ENSP00000382767-D1	5.46	3.28E-07	↓	Protein sel-1-like protein 3, partial [B. mutus]
ENSBTAP00000045773-D1	5.46	3.28E-07	↓	MACRO domain-containing protein 2 [B. mutus]
ENSP00000386310-D1	5.46	3.28E-07	↓	SPATS2-like protein, partial [B. mutus]
ENSP00000413218-D1	5.50	4.18E-12	↓	hypothetical protein M91_10893, partial [B. mutus]
ENSBTAP00000028655-D1	5.50	4.18E-12	↓	Proactivator polypeptide [B. mutus]
ENSP00000369318-D1	5.50	4.18E-12	↓	rho-related BTB domain-containing protein 3 [B. mutus]
ENSP00000345886-D1	5.50	4.18E-12	↓	bromodomain-containing protein 1 isoform X1 [B. mutus]
ENSP00000304592-D1	5.50	4.18E-12	↓	fatty acid synthase [B. mutus]
ENSP00000257430-D1	5.51	1.85E-230	↓	adenomatous polyposis coli protein isoform X1 [B. mutus]
ENSP00000366844-D66	5.51	3.18E-31	↓	hypothetical protein M91_20272, partial [B. mutus]
ENSP00000351664-D1	5.52	2.72E-50	↓	NEDD9-interacting protein with calponin-like protein and LIM domains [B. mutus]
ENSBTAP00000030177-D1	5.53	3.58E-41	↓	emerin [B. mutus]
ENSBTAP00000013031-D1	5.54	4.34E-22	↓	translational activator of cytochrome c oxidase 1-like [B. mutus]
ENSBTAP00000044190-D45	5.54	4.34E-22	↓	hypothetical protein M91_15880, partial [B. mutus]
ENSBTAP00000053208-D4	5.54	4.34E-22	↓	Sodium channel protein type 2 subunit alpha, partial [B. mutus]
ENSBTAP00000027111-D1	5.54	2.21E-12	↓	Myocilin, partial [B. mutus]
ENSBTAP00000041055-D2	5.54	2.21E-12	↓	heterogeneous nuclear ribonucleoprotein A/B isoform X2 [Dasypus novemcinctus]
ENSBTAP00000018739-D1	5.54	2.21E-12	↓	HAUS augmin-like complex subunit 7 [B. mutus]
ENSP00000317971-D1	5.54	2.21E-12	↓	WD and tetratricopeptide repeats protein 1 [B. taurus]
ENSBTAP00000002720-D1	5.54	1.72E-07	↓	frizzled-1-like [Physeter catodon]
ENSBTAP00000012983-D1	5.54	1.72E-07	↓	probable G-protein coupled receptor 171 [B. mutus]
ENSBTAP00000026999-D1	5.54	1.72E-07	↓	metalloproteinase inhibitor 4 [B. mutus]
ENSBTAP00000001550-D1	5.54	1.72E-07	↓	apoptosis-inducing factor 2 isoform X1 [B. mutus]
ENSP00000410890-D1	5.54	1.72E-07	↓	protein ZNF783 isoform X2 [B. taurus]
ENSBTAP00000021161-D1	5.54	1.72E-07	↓	Dermokine [B. mutus]
ENSBTAP00000029961-D1	5.54	1.72E-07	↓	transmembrane protein 134 isoform X3 [B. taurus]
ENSP00000416002-D1	5.54	1.72E-07	↓	hypothetical protein M91_09855 [B. mutus]
ENSP00000349962-D1	5.54	1.72E-07	↓	growth arrest-specific protein 6 isoform X1 [B. taurus]
ENSP00000312773-D1	5.54	1.72E-07	↓	NF-kappa-B inhibitor-interacting Ras-like protein 2 [B. mutus]
ENSBTAP00000025396-D1	5.54	1.72E-07	↓	serine/threonine-protein kinase PAK 6 isoform X1 [B. mutus]
ENSBTAP00000025847-D1	5.54	1.72E-07	↓	Connector enhancer of kinase suppressor of ras 1 [B. mutus]
ENSBTAP00000030281-D1	5.54	1.72E-07	↓	transmembrane protein 218 [B. mutus]
ENSP00000245503-D3	5.54	1.72E-07	↓	myosin-1 [B. mutus]

ENSBTAP00000003797-D1	5.54	1.72E-07	↓	CX3C chemokine receptor 1 [B. mutus]
ENSBTAP00000019591-D1	5.54	1.72E-07	↓	Mucin-4, partial [B. mutus]
ENSP00000265825-D1	5.54	1.72E-07	↓	Fascin-3 [B. mutus]
ENSBTAP00000007794-D1	5.54	1.72E-07	↓	Meiotic recombination protein DMC1/LIM15-like protein, partial [B. mutus]
ENSBTAP00000026668-D1	5.54	1.72E-07	↓	interleukin-13 receptor subunit alpha-2 [B. mutus]
ENSBTAP00000016363-D1	5.54	1.72E-07	↓	Zinc finger protein 524, partial [B. mutus]
ENSBTAP00000015255-D1	5.54	1.72E-07	↓	hypothetical protein M91_13692 [B. mutus]
ENSBTAP00000021194-D1	5.54	1.72E-07	↓	FCH and double SH3 domains protein 1, partial [B. mutus]
ENSP00000326424-D1	5.54	1.72E-07	↓	glutamine-dependent NAD(+) synthetase [B. mutus]
ENSP00000395774-D1	5.54	1.72E-07	↓	kelch-like protein 35 isoform X1 [B. taurus]
ENSBTAP00000026275-D1	5.54	1.72E-07	↓	interleukin-8 [Pantholops hodgsonii]
ENSP00000216513-D1	5.54	1.72E-07	↓	Homeobox protein SIX4, partial [B. mutus]
ENSP00000410007-D1	5.54	1.72E-07	↓	F-box protein 46 [B. mutus]
ENSBTAP00000003661-D1	5.54	1.72E-07	↓	E3 ubiquitin-protein ligase RNF185 [Otolemur garnettii]
ENSBTAP00000048711-D2	5.54	1.72E-07	↓	Tyrosine-protein kinase ZAP-70, partial [B. mutus]
ENSP00000155840-D1	5.54	1.72E-07	↓	potassium voltage-gated channel subfamily KQT member 1 [B. mutus]
ENSP00000362207-D1	5.54	1.72E-07	↓	carbohydrate sulfotransferase 3 [B. mutus]
ENSBTAP00000009917-D1	5.54	1.72E-07	↓	Gamma-glutamyl hydrolase, partial [B. mutus]
ENSBTAP00000052397-D1	5.54	1.72E-07	↓	Interferon-induced guanylate-binding protein 2, partial [B. mutus]
ENSP00000363105-D1	5.54	1.72E-07	↓	APOBEC1 complementation factor isoform X1 [B. mutus]
ENSBTAP00000049316-D1	5.54	1.72E-07	↓	cerebellar degeneration-related protein 2 [B. mutus]
ENSP00000245810-D1	5.54	1.72E-07	↓	persephin [B. mutus]
ENSP00000369816-D1	5.54	1.72E-07	↓	Sex hormone-binding globulin [B. mutus]
ENSP00000342216-D3	5.54	1.72E-07	↓	ATP-binding cassette sub-family A member 6 [B. mutus]
ENSBTAP00000023517-D1	5.54	1.72E-07	↓	methylcytosine dioxygenase TET2 isoform X1 [Bison bison bison]
ENSBTAP00000009852-D1	5.54	1.72E-07	↓	extracellular sulfatase Sulf-2 isoform X1 [B. mutus]
ENSBTAP00000045301-D1	5.54	1.72E-07	↓	Zinc finger protein 521, partial [B. mutus]
ENSP00000333212-D1	5.54	1.72E-07	↓	cytochrome P450 2U1-like [B. mutus]
ENSBTAP00000022184-D1	5.54	1.72E-07	↓	zinc finger protein 202 [B. mutus]
ENSBTAP00000018774-D1	5.54	1.72E-07	↓	prostaglandin G/H synthase 2 isoform X1 [B. mutus]
ENSBTAP00000037755-D1	5.54	1.72E-07	↓	Absent in melanoma 1-like protein [B. mutus]
ENSP00000298295-D1	5.54	1.72E-07	↓	protein DEPP [B. mutus]
ENSBTAP00000011924-D1	5.54	1.72E-07	↓	matrix metalloproteinase-19 [B. mutus]
ENSBTAP00000003608-D1	5.54	1.72E-07	↓	zinc finger CCCH domain-containing protein 7B isoform X2 [Bison bison bison]
ENSBTAP00000022103-D1	5.54	1.72E-07	↓	Fc receptor-like protein 2, partial [B. mutus]
ENSP00000318868-D1	5.54	1.72E-07	↓	serine hydroxymethyltransferase, cytosolic isoform X1 [B. mutus]
ENSBTAP00000016291-D1	5.54	1.72E-07	↓	Chloride channel protein 2, partial [B. mutus]
ENSBTAP00000045034-D4	5.54	1.72E-07	↓	interferon beta-2-like [B. mutus]
ENSP00000401704-D1	5.54	1.72E-07	↓	PH and SEC7 domain-containing protein 3, partial [B. mutus]
ENSBTAP00000042778-D1	5.54	1.72E-07	↓	Endothelin-converting enzyme 2 [B. mutus]
ENSBTAP00000008883-D1	5.54	1.72E-07	↓	D site-binding protein [Bison bison bison]
ENSP00000376444-D1	5.56	1.54E-100	↓	ATP-binding cassette sub-family A member 5 [B. mutus]
ENSBTAP00000044267-D1	5.58	1.23E-22	↓	interferon regulatory factor 9 [B. mutus]
ENSBTAP00000053648-D1	5.58	1.17E-12	↓	junction-mediating and -regulatory protein [B. mutus]
ENSBTAP00000002844-D1	5.58	1.17E-12	↓	RRP12-like protein [B. mutus]
ENSP00000387546-D1	5.58	1.17E-12	↓	zinc finger protein 438 [B. mutus]
ENSP00000301178-D1	5.62	6.22E-13	↓	Tyrosine-protein kinase receptor UFO, partial [B. mutus]
ENSP00000308122-D1	5.62	6.22E-13	↓	V-type proton ATPase 116 kDa subunit a isoform 4 [B. mutus]
ENSBTAP00000050692-D1	5.62	9.02E-08	↓	hypothetical protein M91_15058, partial [B. mutus]
ENSP00000382084-D1	5.62	9.02E-08	↓	THAP domain-containing protein 7 [B. taurus]
ENSBTAP00000018564-D1	5.62	9.02E-08	↓	uncharacterized protein C19orf52 homolog [B. taurus]
ENSP00000363116-D1	5.62	9.02E-08	↓	tyrosine-protein kinase Fgr [B. taurus]
ENSP00000385862-D1	5.62	9.02E-08	↓	hypothetical protein M91_10965, partial [B. mutus]
ENSBTAP00000010191-D1	5.62	9.02E-08	↓	NF-kappa-B inhibitor-like protein 2 [B. mutus]
ENSP00000190165-D1	5.62	9.02E-08	↓	Doublesex- and mab-3-related transcription factor 3, partial [B. mutus]

ENSP00000321636-D1	5.62	9.02E-08	↓	DNA topoisomerase 3-alpha [B. mutus]
ENSBTAP00000051130-D1	5.62	9.02E-08	↓	laminin subunit beta-2-like [B. mutus]
ENSP00000322276-D2	5.62	9.02E-08	↓	Pantetheinase [B. mutus]
ENSBTAP00000053765-D1	5.62	9.02E-08	↓	Tyrosine-protein kinase transmembrane receptor ROR2, partial [B. mutus]
ENSP00000239440-D1	5.62	9.02E-08	↓	arf-GAP with Rho-GAP domain, ANK repeat and PH domain-containing protein 3 [B. mutus]
ENSBTAP00000007473-D1	5.62	9.02E-08	↓	28S ribosomal protein S2, mitochondrial, partial [B. mutus]
ENSP00000409223-D1	5.62	9.02E-08	↓	hypothetical protein M91_19169, partial [B. mutus]
ENSBTAP00000041753-D1	5.62	9.02E-08	↓	Oxysterol-binding protein-related protein 5 [B. mutus]
ENSP00000244051-D1	5.62	9.02E-08	↓	adenylyltransferase and sulfurtransferase MOCS3 [B. mutus]
ENSBTAP00000049790-D39	5.62	9.02E-08	↓	TPA: hypothetical protein BOS_26048 [B. taurus]
ENSP00000384611-D1	5.62	9.02E-08	↓	Potassium voltage-gated channel subfamily G member 3, partial [B. mutus]
ENSP00000215838-D1	5.62	9.02E-08	↓	transcobalamin-2 [B. mutus]
ENSP00000276737-D1	5.62	9.02E-08	↓	protein FAM135B [B. mutus]
ENSBTAP00000007766-D1	5.62	9.02E-08	↓	DNA methyltransferase 1-associated protein 1 [B. mutus]
ENSBTAP00000020444-D1	5.62	9.02E-08	↓	Monocarboxylate transporter 5, partial [B. mutus]
ENSP00000314004-D1	5.62	9.02E-08	↓	Anaphase-promoting complex subunit 2, partial [B. mutus]
ENSP00000332643-D1	5.62	9.02E-08	↓	necdin [B. taurus]
ENSP00000260264-D1	5.62	9.02E-08	↓	POU domain, class 2, transcription factor 3, partial [B. mutus]
ENSBTAP00000012781-D1	5.62	9.02E-08	↓	SH2B adapter protein 2 isoform X3 [B. taurus]
ENSP00000232888-D1	5.62	9.02E-08	↓	U3 small nucleolar RNA-interacting protein 2 [B. mutus]
ENSP00000364220-D1	5.62	9.02E-08	↓	von Willebrand factor A domain-containing protein 5B1 [B. mutus]
ENSBTAP00000016093-D1	5.62	9.02E-08	↓	Rab GDP dissociation inhibitor alpha, partial [B. mutus]
ENSP00000368798-D1	5.62	9.02E-08	↓	Membrane-bound transcription factor site-2 protease, partial [B. mutus]
ENSBTAP00000049647-D1	5.62	9.02E-08	↓	Protein FAM92B [B. mutus]
ENSP00000287957-D1	5.62	9.02E-08	↓	GATA zinc finger domain-containing protein 1, partial [B. mutus]
ENSBTAP00000030408-D1	5.62	9.02E-08	↓	Alpha-tocopherol transfer protein, partial [B. mutus]
ENSP00000270593-D1	5.62	9.02E-08	↓	testicular acid phosphatase [B. mutus]
ENSP00000384712-D1	5.62	9.02E-08	↓	CUE domain-containing protein 1, partial [B. mutus]
ENSBTAP00000001107-D1	5.62	9.02E-08	↓	Breast carcinoma-amplified sequence 1-like protein [B. mutus]
ENSBTAP00000033875-D1	5.62	9.02E-08	↓	ephrin-A4 [B. mutus]
ENSP00000347498-D1	5.62	9.02E-08	↓	Zinc finger FYVE domain-containing protein 19, partial [B. mutus]
ENSBTAP00000024520-D1	5.62	9.02E-08	↓	Protein pigeon-like protein, partial [B. mutus]
ENSP00000320509-D2	5.62	9.02E-08	↓	B-cell scaffold protein with ankyrin repeats, partial [B. mutus]
ENSP00000268835-D1	5.62	9.02E-08	↓	phosphoribosyl pyrophosphate synthase-associated protein 2 [B. taurus]
ENSBTAP00000006996-D1	5.62	9.02E-08	↓	Nuclear RNA export factor 2, partial [B. mutus]
ENSP00000406035-D1	5.62	9.02E-08	↓	Antigen peptide transporter 2, partial [B. mutus]
ENSBTAP00000025201-D1	5.62	9.02E-08	↓	Lanosterol synthase [B. mutus]
ENSP00000270233-D1	5.62	9.02E-08	↓	basal cell adhesion molecule [B. mutus]
ENSP00000376952-D1	5.62	9.02E-08	↓	crossover junction endonuclease EME1 [B. mutus]
ENSBTAP00000052860-D1	5.62	9.02E-08	↓	Thyrotropin receptor, partial [B. mutus]
ENSBTAP00000004942-D1	5.62	9.02E-08	↓	tektin-3 [B. mutus]
yakA20087	5.62	9.02E-08	↓	Protein FAM160A1 [B. mutus]
ENSBTAP00000039446-D1	5.62	9.02E-08	↓	V(D)J recombination-activating protein 2 [B. taurus]
ENSP00000314103-D1	5.62	9.02E-08	↓	ankyrin repeat domain-containing protein 34A [B. mutus]
ENSBTAP00000000078-D1	5.62	9.02E-08	↓	hypothetical protein M91_03590, partial [B. mutus]
ENSP00000261861-D1	5.62	9.02E-08	↓	Coronin-2B, partial [B. mutus]
ENSBTAP00000049930-D1	5.62	9.02E-08	↓	Transmembrane 4 L6 family member 19, partial [B. mutus]
ENSBTAP00000047551-D1	5.62	9.02E-08	↓	thymus-specific serine protease [B. mutus]
ENSP00000337443-D1	5.62	9.02E-08	↓	nucleoredoxin [Sus scrofa]
ENSBTAP00000053307-D1	5.62	9.02E-08	↓	Limbic system-associated membrane protein, partial [B. mutus]
ENSP00000349932-D1	5.62	9.02E-08	↓	Receptor-type tyrosine-protein phosphatase S, partial [B. mutus]
ENSP00000295190-D1	5.62	9.02E-08	↓	monocarboxylate transporter 14 [B. mutus]
ENSBTAP00000014357-D1	5.62	9.02E-08	↓	PI-PLC X domain-containing protein 3, partial [B. mutus]
ENSP00000302855-D1	5.62	9.02E-08	↓	Zinc finger protein 280A, partial [B. mutus]
ENSBTAP00000011327-D1	5.62	9.02E-08	↓	Rho guanine nucleotide exchange factor 10, partial [B. mutus]

ENSBTAP00000013751-D1	5.62	9.02E-08	↓	protocadherin-17-like [B. mutus]
ENSP00000262795-D1	5.62	9.02E-08	↓	SH3 and multiple ankyrin repeat domains protein 3, partial [B. mutus]
ENSP00000355865-D1	5.62	9.02E-08	↓	hypothetical protein M91_14086, partial [B. mutus]
ENSP00000273395-D1	5.62	9.02E-08	↓	Brother of CDO [B. mutus]
ENSBTAP00000033721-D1	5.62	9.02E-08	↓	Multidrug resistance protein 3, partial [B. mutus]
ENSBTAP00000052791-D8	5.62	9.02E-08	↓	Hemoglobin fetal subunit beta, partial [B. mutus]
ENSBTAP00000048591-D2	5.62	9.02E-08	↓	zinc finger protein LOC768229 [B. taurus]
ENSBTAP00000049529-D1	5.62	2.49E-64	↓	testicular haploid expressed gene protein [B. mutus]
ENSP00000258886-D2	5.63	8.17E-75	↓	Cytoplasmic aconitate hydratase [B. mutus]
ENSP00000238651-D1	5.63	1.33E-64	↓	Acyl-coenzyme A thioesterase 2, mitochondrial, partial [B. mutus]
ENSP00000261726-D1	5.64	3.54E-44	↓	Homeobox protein cut-like 2, partial [B. mutus]
ENSBTAP00000010237-D1	5.66	9.92E-24	↓	F-box and leucine-rich repeat protein 14, partial [B. mutus]
ENSP00000304292-D1	5.66	3.31E-13	↓	ankyrin repeat domain-containing protein 27 isoform X1 [B. mutus]
ENSBTAP00000038002-D1	5.66	3.31E-13	↓	diacylglycerol kinase alpha isoform X1 [Bubalus bubalis]
ENSP00000368842-D1	5.66	3.31E-13	↓	motile sperm domain-containing protein 3 [B. taurus]
ENSP00000385848-D1	5.66	0.00E+00	↓	Partitioning defective 3-like protein B, partial [B. mutus]
ENSBTAP00000043815-D1	5.67	1.67E-66	↓	acyl-coenzyme A thioesterase THEM4 [B. mutus]
ENSBTAP00000026285-D1	5.67	5.31E-24	↓	peroxisome assembly protein 12 [B. mutus]
ENSBTAP00000008586-D1	5.68	8.95E-67	↓	CDK5 regulatory subunit-associated protein 2, partial [B. mutus]
ENSP00000359042-D1	5.69	6.60E-175	↓	pre-mRNA-splicing factor 38B [Pantholops hodgsonii]
ENSP00000377813-D1	5.69	1.77E-13	↓	Protein phosphatase 1 regulatory subunit 1B, partial [B. mutus]
ENSBTAP00000024417-D1	5.69	4.77E-08	↓	actin-binding Rho-activating protein [B. mutus]
ENSP00000245796-D1	5.69	4.77E-08	↓	PH and SEC7 domain-containing protein 4, partial [B. mutus]
ENSBTAP00000033750-D1	5.69	4.77E-08	↓	Anterior gradient protein 2-like protein, partial [B. mutus]
ENSBTAP00000021595-D1	5.69	4.77E-08	↓	GDP-fucose protein O-fucosyltransferase 1 isoform a precursor [B. taurus]
ENSBTAP00000026111-D1	5.69	4.77E-08	↓	Pikachurin, partial [B. mutus]
ENSP00000245932-D1	5.69	4.77E-08	↓	Vasodilator-stimulated phosphoprotein, partial [B. mutus]
ENSP00000339175-D1	5.69	4.77E-08	↓	Uridine diphosphate glucose pyrophosphatase, partial [B. mutus]
ENSBTAP00000053659-D1	5.69	4.77E-08	↓	protein cornichon homolog 3 [B. taurus]
ENSP00000368698-D1	5.69	4.77E-08	↓	zinc finger protein 40 [B. mutus]
ENSBTAP00000043397-D11	5.69	4.77E-08	↓	hypothetical protein M91_02194 [B. mutus]
yakG034321	5.69	4.77E-08	↓	hypothetical protein M91_04058 [B. mutus]
ENSBTAP00000009383-D1	5.69	4.77E-08	↓	Putative cation-transporting ATPase 13A1 [B. mutus]
ENSP00000306320-D2	5.69	4.77E-08	↓	chromosome unknown open reading frame, human C14orf142 [B. mutus]
ENSP00000265686-D1	5.69	4.77E-08	↓	V-type proton ATPase 116 kDa subunit a isoform 3 [B. mutus]
ENSP00000305138-D1	5.69	4.77E-08	↓	Protein FAM195A, partial [B. mutus]
ENSBTAP00000024448-D3	5.69	4.77E-08	↓	Dihydropyrimidinase-related protein 1, partial [B. mutus]
ENSP00000319635-D2	5.69	4.77E-08	↓	c-X-C chemokine receptor type 2-like [B. mutus]
ENSBTAP00000012262-D1	5.69	4.77E-08	↓	Peroxisome biogenesis factor 10, partial [B. mutus]
ENSP00000216840-D1	5.69	4.77E-08	↓	Geranylgeranyl transferase type-2 subunit alpha, partial [B. mutus]
ENSP00000382390-D1	5.69	4.77E-08	↓	Neuralized-like protein 4, partial [B. mutus]
ENSP00000386439-D1	5.69	4.77E-08	↓	Syncoilin, partial [B. mutus]
ENSP00000376838-D1	5.69	4.77E-08	↓	Transmembrane protein 108, partial [B. mutus]
ENSBTAP00000048101-D1	5.69	4.77E-08	↓	chemokine (C-X-C motif) receptor 2 [B. mutus]
ENSP00000380855-D1	5.69	4.77E-08	↓	programmed cell death 1 ligand 2 [B. mutus]
ENSBTAP00000041434-D1	5.69	4.77E-08	↓	Fatty acid desaturase 2-like protein, partial [B. mutus]
ENSBTAP00000028684-D1	5.69	4.77E-08	↓	group 10 secretory phospholipase A2 [B. mutus]
ENSBTAP00000019481-D1	5.69	4.77E-08	↓	Visual system homeobox 2, partial [B. mutus]
ENSP00000342235-D1	5.69	4.77E-08	↓	Receptor tyrosine-protein kinase erbB-4, partial [B. mutus]
ENSBTAP00000011785-D1	5.69	4.77E-08	↓	Rap1 GTPase-activating protein 1, partial [B. mutus]
ENSBTAP00000049998-D1	5.69	4.77E-08	↓	ARL14 effector protein-like [B. mutus]
ENSBTAP00000046769-D1	5.69	4.77E-08	↓	synaptonemal complex central element protein 1 [B. mutus]
ENSBTAP00000016562-D1	5.69	4.77E-08	↓	Ran GTPase-activating protein 1, partial [B. mutus]
ENSBTAP00000001445-D1	5.69	4.77E-08	↓	vesicular glutamate transporter 3 isoform X1 [B. mutus]
ENSP00000360899-D1	5.69	4.77E-08	↓	BEN domain-containing protein 5, partial [B. mutus]

ENSBTAP00000035660-D1	5.69	4.77E-08	↓	Signal peptide peptidase-like 2B, partial [B. mutus]
ENSP00000359729-D1	5.69	4.77E-08	↓	Sodium/hydrogen exchanger 6, partial [B. mutus]
ENSP00000370936-D3	5.69	4.77E-08	↓	Transcription factor E2F6, partial [B. mutus]
ENSBTAP00000023277-D2	5.69	4.77E-08	↓	G1/S-specific cyclin-D2, partial [B. mutus]
ENSBTAP00000014478-D1	5.69	4.77E-08	↓	PAXIP1-associated glutamate-rich protein 1 [B. mutus]
ENSBTAP00000011390-D1	5.69	4.77E-08	↓	cAMP-specific 3',5'-cyclic phosphodiesterase 4B, partial [B. mutus]
ENSP00000385096-D1	5.69	4.77E-08	↓	BTB/POZ domain-containing protein KCTD17 [B. mutus]
ENSP00000418259-D1	5.69	4.77E-08	↓	Zinc finger protein 57-like protein, partial [B. mutus]
ENSBTAP00000015703-D1	5.69	4.77E-08	↓	delta-1-pyrroline-5-carboxylate synthase isoform X1 [B. mutus]
ENSP00000378414-D3	5.69	4.77E-08	↓	SWI/SNF-related matrix-associated actin-dependent regulator of chromatin subfamily D member 2, partial [B. mutus]
ENSBTAP00000032984-D1	5.69	4.77E-08	↓	hypothetical protein M91_19275, partial [B. mutus]
ENSBTAP00000040185-D1	5.69	4.77E-08	↓	Glutamate [NMDA] receptor subunit epsilon-4, partial [B. mutus]
ENSP00000251127-D1	5.69	4.77E-08	↓	sodium leak channel non-selective protein [B. mutus]
ENSP00000222462-D1	5.69	4.77E-08	↓	protein Wnt-16 [B. mutus]
ENSBTAP00000000375-D1	5.69	4.77E-08	↓	protein KIAA2022 homolog isoform X1 [B. taurus]
ENSBTAP00000048785-D1	5.69	4.77E-08	↓	Olfactory receptor 52I2, partial [B. mutus]
ENSP00000369979-D1	5.69	4.77E-08	↓	probable carboxypeptidase X1 precursor [B. taurus]
ENSP00000334294-D1	5.69	4.77E-08	↓	transcription factor-like 5 protein, partial [B. mutus]
ENSBTAP00000010785-D1	5.69	4.77E-08	↓	coiled-coil domain-containing protein 151 isoform X1 [B. mutus]
ENSBTAP00000007993-D1	5.69	4.77E-08	↓	Deoxyribonuclease-2-alpha, partial [B. mutus]
ENSP00000384982-D14	5.69	4.77E-08	↓	hypothetical protein M91_08008, partial [B. mutus]
ENSP00000384193-D1	5.69	4.77E-08	↓	cell adhesion molecule 2 isoform X1 [B. taurus]
ENSP00000268171-D1	5.69	4.77E-08	↓	Furin (paired basic amino acid cleaving enzyme) [B. taurus]
ENSBTAP00000049093-D62	5.69	4.77E-08	↓	Olfactory receptor 4S1, partial [B. mutus]
ENSBTAP00000026585-D1	5.69	4.77E-08	↓	Transmembrane 4 L6 family member 18, partial [B. mutus]
ENSP00000309501-D1	5.69	4.77E-08	↓	leucine-rich repeat and WD repeat-containing protein KIAA1239 homolog [B. mutus]
ENSP00000276654-D1	5.69	4.77E-08	↓	low-density lipoprotein receptor-related protein 12 isoform X1 [B. mutus]
ENSBTAP00000025159-D1	5.69	4.77E-08	↓	Adrenocortical dysplasia protein-like protein, partial [B. mutus]
ENSBTAP00000021222-D1	5.69	4.77E-08	↓	Tetraspanin-2, partial [B. mutus]
ENSP00000394485-D1	5.69	4.77E-08	↓	Na(+)/H(+) exchange regulatory cofactor NHE-RF3 [B. mutus]
ENSBTAP00000001037-D1	5.69	4.77E-08	↓	protransforming growth factor alpha [Balaenoptera acutorostrata scammoni]
yakG039836	5.69	4.77E-08	↓	Stress-induced-phosphoprotein 1 [B. mutus]
ENSBTAP00000008808-D1	5.69	4.77E-08	↓	prostaglandin D2 receptor-like [B. mutus]
ENSP00000385213-D1	5.69	4.77E-08	↓	piggyBac transposable element-derived protein 1 [Bison bison bison]
ENSP00000341963-D1	5.70	2.56E-35	↓	regulatory solute carrier protein family 1 member 1-like [B. mutus]
ENSP00000354688-D1	5.70	2.56E-35	↓	protein FAM45A isoform X1 [B. mutus]
ENSP00000382780-D2	5.71	1.52E-24	↓	60S ribosomal protein L10a isoform X1 [Colobus angolensis palliatus]
ENSP00000415436-D1	5.72	1.93E-287	↓	KAT8 regulatory NSL complex subunit 2 isoform X1 [B. mutus]
ENSBTAP00000023595-D1	5.72	7.91E-135	↓	Interleukin-6 receptor subunit beta [B. mutus]
ENSP00000294923-D1	5.73	8.16E-25	↓	desumoylating isopeptidase 2 [Aotus nancymaae]
ENSP00000288025-D1	5.74	1.77E-69	↓	transmembrane emp24 domain-containing protein 6 [B. mutus]
ENSP00000262719-D1	5.75	2.12E-36	↓	PH domain leucine-rich repeat-containing protein phosphatase 1, partial [B. mutus]
ENSP00000363036-D1	5.76	5.06E-14	↓	F-box/WD repeat-containing protein 2 [Pteropus alecto]
ENSBTAP00000011420-D1	5.76	2.55E-08	↓	hypothetical protein M91_13074 [B. mutus]
ENSP00000265634-D1	5.76	2.55E-08	↓	neuronal pentraxin-2 [Bubalus bubalis]
ENSP00000330074-D1	5.76	2.55E-08	↓	histone H1.5-like [Ceratotherium simum simum]
ENSBTAP00000050085-D1	5.76	2.55E-08	↓	regulator of G-protein signaling 21 [Bison bison bison]
ENSBTAP00000046384-D1	5.76	2.55E-08	↓	zinc finger CCHC domain-containing protein 12-like [B. mutus]
ENSBTAP00000041859-D1	5.76	2.55E-08	↓	glucokinase regulatory protein [B. mutus]
ENSP00000389813-D1	5.76	2.55E-08	↓	Acyl-CoA dehydrogenase family member 10 [B. mutus]
ENSBTAP00000015194-D1	5.76	2.55E-08	↓	Non-lysosomal glucosylceramidase, partial [B. mutus]
ENSP00000313731-D1	5.76	2.55E-08	↓	1-phosphatidylinositol-4,5-bisphosphate phosphodiesterase delta-3 [B. mutus]
ENSP00000370194-D1	5.76	2.55E-08	↓	GDP-mannose 4,6 dehydratase, partial [B. mutus]
yakG038008	5.76	2.55E-08	↓	Cytosolic beta-glucosidase [B. mutus]

ENSBTAP00000004621-D1	5.76	2.55E-08	↓	butyrate response factor 2 [B. taurus]
ENSP00000377910-D1	5.76	2.55E-08	↓	dual specificity protein phosphatase 14 [B. taurus]
ENSP00000306776-D1	5.76	2.55E-08	↓	Coiled-coil domain-containing protein 110, partial [B. mutus]
ENSBTAP00000010620-D1	5.76	2.55E-08	↓	Adenosine 3'-phospho 5'-phosphosulfate transporter 1, partial [B. mutus]
ENSBTAP00000049197-D1	5.76	2.55E-08	↓	Chemokine-like factor [B. mutus]
ENSP00000385312-D1	5.76	2.55E-08	↓	mitochondrial thiamine pyrophosphate carrier [B. mutus]
ENSBTAP00000023204-D1	5.76	2.55E-08	↓	mitochondrial dynamics protein MID51 [B. taurus]
ENSP00000300181-D1	5.76	2.55E-08	↓	TSC22 domain family protein 4, partial [B. mutus]
ENSBTAP00000034763-D1	5.76	2.55E-08	↓	methyltransferase-like protein 7A-like [B. mutus]
ENSBTAP00000016098-D1	5.76	2.55E-08	↓	diphthamide biosynthesis protein 7 [B. mutus]
ENSBTAP00000031013-D1	5.76	2.55E-08	↓	mitochondrial enolase superfamily member 1 [B. mutus]
ENSP00000261592-D2	5.76	2.55E-08	↓	hypothetical protein M91_18094, partial [B. mutus]
ENSBTAP00000052792-D1	5.76	2.55E-08	↓	ARL14 effector protein-like [B. mutus]
ENSP00000323511-D1	5.76	2.55E-08	↓	bifunctional polynucleotide phosphatase/kinase [B. taurus]
ENSP00000258711-D1	5.76	2.55E-08	↓	carbohydrate sulfotransferase 12 [B. mutus]
ENSP00000325313-D1	5.76	2.55E-08	↓	Microtubule-associated protein 1S [B. mutus]
yakG005699	5.76	2.55E-08	↓	E3 ubiquitin-protein ligase RNF149 [B. mutus]
ENSBTAP00000049108-D1	5.76	2.55E-08	↓	Ubiquitin carboxyl-terminal hydrolase 22-A, partial [B. mutus]
ENSBTAP00000014563-D1	5.76	2.55E-08	↓	Transmembrane emp24 domain-containing protein 1, partial [B. mutus]
ENSBTAP00000050426-D1	5.76	2.55E-08	↓	solute carrier family 10 member 6 [B. mutus]
ENSBTAP00000020664-D1	5.76	2.55E-08	↓	rho GTPase-activating protein 7 isoform X1 [B. mutus]
ENSP00000248598-D1	5.76	2.55E-08	↓	fibroleukin [Bison bison bison]
ENSBTAP00000025160-D1	5.76	2.55E-08	↓	enkurin domain-containing protein 1 [B. mutus]
ENSP00000306015-D1	5.76	2.55E-08	↓	Synaptopodin-2, partial [B. mutus]
ENSP00000405812-D1	5.76	2.55E-08	↓	Electroneutral sodium bicarbonate exchanger 1, partial [B. mutus]
ENSBTAP00000007564-D1	5.76	2.55E-08	↓	probable palmitoyltransferase ZDHHC24 [B. taurus]
ENSBTAP00000004673-D1	5.76	2.55E-08	↓	protein FAM216B [B. taurus]
ENSP00000378366-D1	5.76	2.55E-08	↓	Flotillin-2, partial [B. mutus]
ENSBTAP00000014839-D1	5.76	2.55E-08	↓	Protein FAM189A2, partial [B. mutus]
yakA12524	5.76	2.55E-08	↓	F-box only protein 6 [B. mutus]
ENSP00000264187-D1	5.76	2.55E-08	↓	nidogen-1 [B. mutus]
ENSBTAP00000026784-D1	5.76	2.55E-08	↓	deoxyribonuclease-1 [B. mutus]
ENSBTAP00000017854-D1	5.76	2.55E-08	↓	glycosylation-dependent cell adhesion molecule 1-like [B. mutus]
ENSBTAP00000005069-D1	5.76	2.55E-08	↓	seminal plasma protein A3-like [B. mutus]
ENSP00000337445-D1	5.76	2.55E-08	↓	Zinc phosphodiesterase ELAC protein 2, partial [B. mutus]
ENSBTAP00000007071-D1	5.76	2.55E-08	↓	Calcineurin-like phosphoesterase domain-containing protein 1 [B. mutus]
ENSBTAP00000014885-D1	5.76	2.55E-08	↓	Synaptotagmin-11, partial [B. mutus]
ENSP00000281631-D1	5.77	4.06E-82	↓	Poly [ADP-ribose] polymerase 8, partial [B. mutus]
ENSBTAP00000022240-D1	5.80	2.71E-14	↓	polynucleotide 5'-hydroxyl-kinase NOL9 [Bison bison bison]
ENSP00000400799-D1	5.81	3.67E-26	↓	Solute carrier family 45 member 4, partial [B. mutus]
ENSP00000317110-D1	5.82	0.00E+00	↓	Centromere protein S, partial [B. mutus]
ENSBTAP00000026641-D1	5.82	6.65E-97	↓	zinc finger protein 800 isoform X1 [B. taurus]
ENSBTAP00000037154-D1	5.82	2.44E-85	↓	transmembrane protein 68 [B. taurus]
ENSP00000246785-D1	5.83	8.92E-74	↓	Bcl-2-like protein 12, partial [B. mutus]
ENSP00000263468-D1	5.83	1.98E-26	↓	Putative protein KIAA1377, partial [B. mutus]
ENSP00000217026-D1	5.83	1.46E-14	↓	Myb-related protein B [B. mutus]
yakG006911	5.83	1.46E-14	↓	hypothetical protein M91_14676 [B. mutus]
ENSP00000298877-D1	5.83	1.36E-08	↓	Sodium/potassium/calcium exchanger 4, partial [B. mutus]
ENSP00000236925-D1	5.83	1.36E-08	↓	Calmodulin-regulated spectrin-associated protein 2 [B. mutus]
ENSBTAP00000016878-D1	5.83	1.36E-08	↓	T-cell surface glycoprotein CD3 zeta chain, partial [B. mutus]
ENSBTAP00000010726-D1	5.83	1.36E-08	↓	Protein FAM164B, partial [B. mutus]
ENSP00000355623-D1	5.83	1.36E-08	↓	hypothetical protein M91_14831, partial [B. mutus]
ENSBTAP00000023554-D1	5.83	1.36E-08	↓	Transcription factor E2F2, partial [B. mutus]
ENSP00000357265-D1	5.83	1.36E-08	↓	semaphorin-4A [B. mutus]
ENSBTAP00000013549-D1	5.83	1.36E-08	↓	Mitogen-activated protein kinase kinase kinase 6, partial [B. mutus]

ENSP00000389989-D1	5.83	1.36E-08	↓	jerky protein homolog-like [B. mutus]
ENSBTAP00000015642-D1	5.83	1.36E-08	↓	mitochondrial pyruvate carrier-like protein-like [B. mutus]
ENSBTAP00000020579-D1	5.83	1.36E-08	↓	CD2 antigen cytoplasmic tail-binding protein 2, partial [B. mutus]
ENSP00000412673-D1	5.83	1.36E-08	↓	Gamma-aminobutyric acid receptor subunit rho-1, partial [B. mutus]
ENSP00000248142-D1	5.83	1.36E-08	↓	WD repeat-containing protein 24, partial [B. mutus]
ENSBTAP00000007770-D1	5.83	1.36E-08	↓	Scm-like with four MBT domains protein 2 [B. mutus]
ENSBTAP00000021683-D1	5.83	1.36E-08	↓	Glycerol-3-phosphate dehydrogenase [NAD+], cytoplasmic, partial [B. mutus]
yakG039804	5.83	1.36E-08	↓	Rho GTPase-activating protein 15 [B. mutus]
ENSP00000355142-D1	5.83	1.36E-08	↓	Membrane progestin receptor beta, partial [B. mutus]
ENSP00000221891-D1	5.83	1.36E-08	↓	Amyloid-like protein 1, partial [B. mutus]
ENSP00000311257-D1	5.83	1.36E-08	↓	storkhead-box protein 2 [B. mutus]
ENSBTAP00000009798-D1	5.83	1.36E-08	↓	Complement factor B, partial [B. mutus]
ENSP00000361705-D1	5.83	1.36E-08	↓	deoxynucleotidyltransferase terminal-interacting protein 1 [B. taurus]
ENSP00000364683-D1	5.83	1.36E-08	↓	BMP and activin membrane-bound inhibitor homolog [Bubalus bubalis]
ENSBTAP00000002199-D1	5.83	1.36E-08	↓	leucine-rich repeat-containing protein 52 [Bubalus bubalis]
ENSBTAP00000000069-D4	5.83	1.36E-08	↓	Protocadherin-11 X-linked, partial [B. mutus]
ENSBTAP00000007879-D1	5.83	1.36E-08	↓	Death-inducer obliterator 1 [B. mutus]
ENSBTAP00000032818-D1	5.83	1.36E-08	↓	Putative G-protein coupled receptor 158, partial [B. mutus]
ENSBTAP00000021794-D1	5.83	1.36E-08	↓	Zinc finger protein 28-like protein, partial [B. mutus]
ENSP00000278829-D3	5.83	1.36E-08	↓	Fatty acid desaturase 3, partial [B. mutus]
ENSBTAP00000000691-D1	5.83	1.36E-08	↓	Zinc finger protein 684, partial [B. mutus]
ENSBTAP00000026449-D1	5.83	1.36E-08	↓	hypothetical protein M91_13196, partial [B. mutus]
ENSBTAP00000013791-D1	5.83	1.36E-08	↓	copine-4 isoform X1 [Ovis aries]
ENSP00000355510-D1	5.83	1.36E-08	↓	WD repeat-containing protein 64 [B. mutus]
ENSBTAP00000002485-D1	5.83	1.36E-08	↓	Transient receptor putative cation channel subfamily V member 5 [B. mutus]
ENSBTAP00000003031-D1	5.83	1.36E-08	↓	Syntaxin-1A, partial [B. mutus]
ENSBTAP00000045511-D1	5.83	1.36E-08	↓	Endomucin [B. mutus]
ENSP00000395590-D1	5.83	1.36E-08	↓	interferon-induced 35 kDa protein [B. mutus]
ENSP00000403392-D1	5.83	1.36E-08	↓	Integrin alpha-11, partial [B. mutus]
ENSP00000392188-D52	5.83	1.36E-08	↓	hypothetical protein M91_05496, partial [B. mutus]
ENSP00000260191-D1	5.83	1.36E-08	↓	5-hydroxytryptamine receptor 3B, partial [B. mutus]
ENSBTAP00000029877-D1	5.83	1.36E-08	↓	Matrix-remodeling-associated protein 5, partial [B. mutus]
ENSBTAP00000005228-D1	5.83	1.36E-08	↓	protein FAM199X [B. taurus]
ENSP00000410940-D5	5.83	1.36E-08	↓	hypothetical protein M91_21297, partial [B. mutus]
ENSBTAP00000019345-D1	5.83	1.36E-08	↓	zinc finger protein SNAI1 [B. taurus]
ENSP00000310933-D1	5.83	1.36E-08	↓	MAP kinase-activating death domain protein isoform X1 [B. mutus]
ENSBTAP00000006079-D4	5.83	1.36E-08	↓	Arylacetyl-CoA N-acyltransferase, partial [B. mutus]
ENSP00000343290-D1	5.83	1.36E-08	↓	hypothetical protein M91_19112, partial [B. mutus]
ENSBTAP00000026322-D1	5.83	1.36E-08	↓	hypothetical protein M91_16323 [B. mutus]
ENSBTAP00000029459-D1	5.83	1.36E-08	↓	Derlin-3, partial [B. mutus]
ENSBTAP00000004002-D2	5.83	1.36E-08	↓	Segment polarity protein dishevelled-like protein DVL-3, partial [B. mutus]
ENSP00000359521-D1	5.83	1.36E-08	↓	SLIT and NTRK-like protein 2 isoform X1 [B. mutus]
ENSBTAP00000015359-D1	5.83	1.36E-08	↓	Glycerol-3-phosphate acyltransferase 2, mitochondrial, partial [B. mutus]
ENSP00000310490-D1	5.83	1.36E-08	↓	monocarboxylate transporter 11 [B. mutus]
ENSP00000367629-D1	5.83	1.36E-08	↓	rho guanine nucleotide exchange factor 16 [B. mutus]
ENSBTAP00000011820-D2	5.83	1.36E-08	↓	Ubiquitin carboxyl-terminal hydrolase 49, partial [B. mutus]
ENSBTAP00000049914-D6	5.83	1.36E-08	↓	hypothetical protein M91_12293, partial [B. mutus]
ENSBTAP00000003943-D1	5.83	1.36E-08	↓	growth arrest and DNA damage-inducible protein GADD45 gamma [B. taurus]
ENSBTAP00000043981-D1	5.83	1.36E-08	↓	regulator of microtubule dynamics protein 2 isoform X1 [B. mutus]
ENSP00000384420-D1	5.85	1.07E-26	↓	Factor XIIa inhibitor, partial [B. mutus]
ENSBTAP00000006410-D1	5.86	7.85E-15	↓	N-acetyltransferase 15 [B. mutus]
ENSP00000376923-D1	5.86	7.85E-15	↓	Nuclear respiratory factor 1 [B. mutus]
ENSBTAP00000043833-D1	5.87	7.96E-234	↓	adipogenesis regulatory factor isoform X1 [B. mutus]
ENSP00000377725-D1	5.88	1.30E-39	↓	zinc finger protein with KRAB and SCAN domains 5 [B. mutus]
ENSBTAP00000018204-D1	5.89	7.28E-09	↓	Splicing factor, arginine/serine-rich 16 [B. mutus]

ENSP00000394981-D1	5.89	7.28E-09	↓	Zinc finger protein neuro-d4, partial [B. mutus]
ENSP00000310260-D2	5.89	7.28E-09	↓	protein DDX26B-like isoform X1 [B. mutus]
ENSBTAP00000027520-D1	5.89	7.28E-09	↓	testis-specific serine/threonine-protein kinase 3 [B. taurus]
yakA15795	5.89	7.28E-09	↓	hypothetical protein M91_03621 [B. mutus]
ENSP00000412315-D1	5.89	7.28E-09	↓	GSK3-beta interaction protein [B. taurus]
ENSBTAP00000009697-D1	5.89	7.28E-09	↓	PDZ and LIM domain protein 2 [B. mutus]
ENSBTAP00000000088-D1	5.89	7.28E-09	↓	Glutamate-rich WD repeat-containing protein 1, partial [B. mutus]
ENSP00000282041-D1	5.89	7.28E-09	↓	UPF0493 protein KIAA1632 [B. mutus]
ENSBTAP00000006255-D1	5.89	7.28E-09	↓	olfactory receptor 51I1-like [B. mutus]
ENSP00000386125-D1	5.89	7.28E-09	↓	armadillo repeat-containing protein 5 isoform X2 [Ovis aries musimon]
ENSBTAP00000024412-D1	5.89	7.28E-09	↓	interleukin-33 [B. mutus]
ENSBTAP00000035767-D1	5.89	7.28E-09	↓	mediator of DNA damage checkpoint protein 1 [B. mutus]
ENSBTAP00000019617-D1	5.89	7.28E-09	↓	Leucine-rich repeat and WD repeat-containing protein 1, partial [B. mutus]
ENSP00000407509-D1	5.89	7.28E-09	↓	Neuropathy target esterase [B. mutus]
ENSP00000278243-D1	5.89	7.28E-09	↓	post-GPI attachment to proteins factor 2 isoform X3 [B. mutus]
ENSBTAP00000021843-D1	5.89	7.28E-09	↓	matrix metalloproteinase-25 [B. mutus]
ENSP00000410533-D1	5.89	7.28E-09	↓	RING finger protein 223 [B. taurus]
ENSBTAP00000027390-D1	5.89	7.28E-09	↓	beta-defensin 123 precursor [B. taurus]
ENSP00000356988-D1	5.89	7.28E-09	↓	nitrilase homolog 1 isoform X1 [B. mutus]
ENSP00000311684-D1	5.89	7.28E-09	↓	Striated muscle preferentially expressed protein kinase [B. mutus]
ENSBTAP00000018649-D1	5.89	7.28E-09	↓	collectin-46-like [B. mutus]
ENSP00000219197-D1	5.89	7.28E-09	↓	cerebellin-1 [Octodon degus]
ENSP00000310832-D1	5.89	7.28E-09	↓	cathepsin F isoform X1 [B. mutus]
ENSBTAP00000010973-D1	5.89	7.28E-09	↓	ETS translocation variant 4 isoform X2 [B. taurus]
ENSP00000297001-D1	5.89	7.28E-09	↓	uncharacterized protein C7orf72 homolog [B. taurus]
ENSP00000296358-D1	5.89	7.28E-09	↓	otopetrin-1 [B. mutus]
ENSP00000268053-D1	5.89	7.28E-09	↓	Cholesterol side-chain cleavage enzyme, mitochondrial [B. mutus]
ENSP00000403290-D1	5.89	7.28E-09	↓	Putative gluconokinase, partial [B. mutus]
ENSBTAP00000022068-D1	5.89	7.28E-09	↓	rab11 family-interacting protein 3 isoform X5 [B. taurus]
ENSBTAP00000019713-D1	5.89	7.28E-09	↓	Williams-Beuren syndrome chromosomal region 28 protein homolog [B. mutus]
ENSBTAP00000006956-D1	5.89	7.28E-09	↓	serpin E3 [B. mutus]
ENSBTAP00000004917-D1	5.89	7.28E-09	↓	Tyrosine-protein kinase receptor Tie-1, partial [B. mutus]
ENSBTAP00000016778-D1	5.89	7.28E-09	↓	uroporphyrinogen decarboxylase [B. taurus]
ENSP00000420140-D1	5.89	7.28E-09	↓	Protein FAM71F2 [B. mutus]
ENSP00000325663-D1	5.89	7.28E-09	↓	NF-kappa-B inhibitor zeta, partial [B. mutus]
ENSP00000360262-D1	5.89	7.28E-09	↓	FGGY carbohydrate kinase domain-containing protein, partial [B. mutus]
ENSP00000228705-D1	5.89	7.28E-09	↓	Protein phosphatase 1H, partial [B. mutus]
ENSBTAP00000013015-D1	5.89	7.28E-09	↓	Protein FAM71F1, partial [B. mutus]
ENSBTAP00000021983-D1	5.89	7.28E-09	↓	MAP kinase-activated protein kinase 3 [B. mutus]
ENSP00000389891-D1	5.89	7.28E-09	↓	TNF receptor-associated factor 5 [B. mutus]
ENSBTAP00000005477-D1	5.89	7.28E-09	↓	WD repeat-containing protein 66, partial [B. mutus]
ENSP00000379011-D2	5.89	7.28E-09	↓	Carnitine O-palmitoyltransferase 1, liver isoform [B. mutus]
ENSP00000403888-D1	5.89	7.28E-09	↓	disrupted in schizophrenia 1 protein [B. mutus]
ENSP00000344468-D1	5.89	7.28E-09	↓	syndecan 3 [B. mutus]
ENSP00000252453-D1	5.89	7.28E-09	↓	betatrophin [B. mutus]
ENSBTAP00000004724-D51	5.89	7.28E-09	↓	zinc finger protein 501 [Lipotes vexillifer]
ENSP00000315106-D1	5.89	7.28E-09	↓	integral membrane protein GPR137C [B. taurus]
ENSBTAP00000030131-D1	5.89	7.28E-09	↓	Fatty acid desaturase 1, partial [B. mutus]
ENSP00000360676-D1	5.89	7.28E-09	↓	protein KTI12 homolog isoform X1 [B. mutus]
ENSP00000334314-D1	5.89	7.28E-09	↓	Echinoderm microtubule-associated protein-like 1, partial [B. mutus]
ENSBTAP00000023660-D1	5.89	7.28E-09	↓	coiled-coil domain-containing protein 153 [B. mutus]
ENSP00000397121-D1	5.89	7.28E-09	↓	TPA: rRNA/tRNA 2-O-methyltransferase fibrillarin-like protein 1-like [B. taurus]
ENSP00000321627-D1	5.89	7.28E-09	↓	Ankyrin repeat and fibronectin type-III domain-containing protein 1, partial [B. mutus]
ENSBTAP00000000573-D1	5.89	7.28E-09	↓	D-beta-hydroxybutyrate dehydrogenase, mitochondrial [B. mutus]
ENSBTAP00000002542-D1	5.89	7.28E-09	↓	histidine triad nucleotide-binding protein 3 [B. mutus]

ENSBTAP00000004913-D1	5.89	7.28E-09	↓	Complement component C8 alpha chain [B. mutus]
ENSP00000391200-D1	5.89	7.28E-09	↓	Zinc finger protein 692, partial [B. mutus]
ENSP00000385636-D1	5.89	7.28E-09	↓	obscurin-like protein 1, partial [B. mutus]
ENSP00000218006-D1	5.89	7.28E-09	↓	retinal guanylyl cyclase 2 [B. mutus]
ENSBTAP00000000271-D1	5.89	7.28E-09	↓	Serine/threonine-protein kinase Nek10, partial [B. mutus]
ENSP00000399681-D3	5.89	7.28E-09	↓	dynamin-3-like [Bubalus bubalis]
ENSBTAP00000053424-D1	5.89	7.28E-09	↓	inactive heparanase-2 [Bubalus bubalis]
ENSP00000364087-D1	5.89	7.28E-09	↓	SLA class II histocompatibility antigen, DQ haplotype D alpha chain-like [B. mutus]
ENSBTAP00000007603-D1	5.89	7.28E-09	↓	di-N-acetylchitobiase [B. mutus]
ENSP00000295324-D1	5.89	7.28E-09	↓	cdc42 effector protein 3 [B. mutus]
ENSP00000395668-D1	5.89	7.28E-09	↓	Coiled-coil domain-containing protein 149, partial [B. mutus]
ENSP00000319062-D1	5.90	1.64E-52	↓	Bardet-Biedl syndrome 12 protein-like protein, partial [B. mutus]
ENSP00000370503-D1	5.92	2.29E-15	↓	malcavernin [B. taurus]
ENSP00000262713-D1	5.92	2.29E-15	↓	LIM domain-containing protein ajuba [B. taurus]
ENSP00000355865-D2	5.92	2.29E-15	↓	E3 ubiquitin-protein ligase parkin-like [B. mutus]
ENSP00000226578-D1	5.93	4.98E-231	↓	beta-mannosidase [B. mutus]
ENSP00000262414-D1	5.94	3.31E-41	↓	116 kDa U5 small nuclear ribonucleoprotein component [Dipodomys ordii]
ENSP00000343435-D1	5.95	1.24E-15	↓	Protein YIF1B, partial [B. mutus]
ENSP00000408994-D1	5.95	1.24E-15	↓	DET1-like protein, partial [B. mutus]
ENSP00000267540-D1	5.95	3.92E-09	↓	TPA: stonin 2 [B. taurus]
ENSP00000262188-D3	5.95	3.92E-09	↓	SWI/SNF-related matrix-associated actin-dependent regulator of chromatin subfamily D member 3 [B. mutus]
ENSBTAP00000023771-D1	5.95	3.92E-09	↓	leucine-rich repeat-containing protein 38 isoform X1 [B. taurus]
ENSBTAP00000050368-D69	5.95	3.92E-09	↓	zinc finger protein 606 [Jaculus jaculus]
ENSBTAP00000049917-D1	5.95	3.92E-09	↓	Glypican-6, partial [B. mutus]
ENSP00000309007-D1	5.95	3.92E-09	↓	THAP domain-containing protein 6, partial [B. mutus]
ENSBTAP00000042640-D1	5.95	3.92E-09	↓	Metabotropic glutamate receptor 8, partial [B. mutus]
ENSP00000380315-D28	5.95	3.92E-09	↓	zinc finger protein 850-like isoform X4 [Physeter catodon]
ENSBTAP00000000639-D1	5.95	3.92E-09	↓	kelch-like protein 13 isoform X3 [Ailuropoda melanoleuca]
ENSBTAP00000023994-D1	5.95	3.92E-09	↓	Nuclear receptor subfamily 1 group D member 2, partial [B. mutus]
ENSBTAP00000013968-D1	5.95	3.92E-09	↓	Dehydrogenase/reductase SDR family member 7B, partial [B. mutus]
ENSBTAP00000029287-D1	5.95	3.92E-09	↓	Putative E3 ubiquitin-protein ligase MID2, partial [B. mutus]
ENSBTAP00000010606-D1	5.95	3.92E-09	↓	peroxisome proliferator-activated receptor alpha [B. taurus]
ENSP00000415203-D1	5.95	3.92E-09	↓	GPI ethanolamine phosphate transferase 2 isoform X1 [Bison bison bison]
ENSBTAP00000006032-D1	5.95	3.92E-09	↓	TPA: pim-2 oncogene [B. taurus]
ENSP00000325917-D1	5.95	3.92E-09	↓	proprotein convertase subtilisin/kexin type 7 [B. mutus]
ENSBTAP00000043279-D1	5.95	3.92E-09	↓	Protein HIDE1 [B. mutus]
ENSP00000385724-D1	5.95	3.92E-09	↓	voltage-dependent L-type calcium channel subunit alpha-1C isoform X16 [Ovis aries musimon]
ENSP00000302289-D1	5.95	3.92E-09	↓	Embigin, partial [B. mutus]
ENSP00000377437-D1	5.95	3.92E-09	↓	ELMO domain-containing protein 3 [B. mutus]
ENSP00000376332-D1	5.95	3.92E-09	↓	RING finger and transmembrane domain-containing protein 2, partial [B. mutus]
ENSP00000353074-D37	5.95	3.92E-09	↓	hypothetical protein M91_19007, partial [B. mutus]
ENSBTAP00000046543-D4	5.95	3.92E-09	↓	hypothetical protein M91_16213 [B. mutus]
ENSP00000404833-D1	5.95	3.92E-09	↓	tapasin isoform X1 [Capra hircus]
ENSP00000368260-D1	5.95	3.92E-09	↓	glutamate-rich protein 6B [Bison bison bison]
ENSP00000221166-D1	5.95	3.92E-09	↓	neurofilament medium polypeptide [B. mutus]
ENSP00000420211-D1	5.95	3.92E-09	↓	Ral guanine nucleotide dissociation stimulator-like 2, partial [B. mutus]
ENSP00000355802-D1	5.95	3.92E-09	↓	epoxide hydrolase 1 isoform X1 [B. mutus]
ENSP00000264360-D1	5.95	3.92E-09	↓	Protocadherin-10, partial [B. mutus]
ENSP00000370526-D3	5.95	3.92E-09	↓	Arylsulfatase D, partial [B. mutus]
ENSP00000358202-D1	5.95	3.92E-09	↓	homeobox protein EMX2 [B. mutus]
ENSP00000300127-D22	5.95	3.92E-09	↓	Olfactory receptor 4B1 [B. mutus]
ENSBTAP00000015242-D1	5.95	3.92E-09	↓	Cysteinyl-tRNA synthetase, mitochondrial, partial [B. mutus]
ENSP00000407487-D1	5.95	3.92E-09	↓	protein unc-45 homolog A [B. mutus]
ENSP00000416993-D2	5.95	3.92E-09	↓	Glutathione S-transferase theta-4, partial [B. mutus]
ENSBTAP00000023029-D2	5.95	3.92E-09	↓	Small ubiquitin-related modifier 3 [B. mutus]

ENSBTAP00000024978-D1	5.95	3.92E-09	↓	solute carrier family 35 member F6 precursor [B. taurus]
ENSP00000334113-D1	5.95	3.92E-09	↓	Dehydrogenase/reductase SDR family member on chromosome X, partial [B. mutus]
ENSP00000361302-D1	5.95	3.92E-09	↓	protein O-mannosyl-transferase 1 isoform X1 [B. mutus]
ENSP00000361459-D1	5.95	3.92E-09	↓	TSC22 domain family protein 3 isoform X1 [Condylura cristata]
ENSP00000361186-D1	5.95	3.92E-09	↓	TP53-regulating kinase [B. mutus]
ENSP00000331556-D1	5.95	3.92E-09	↓	Cylicin-1, partial [B. mutus]
ENSBTAP00000007273-D1	5.95	3.92E-09	↓	glycine N-methyltransferase [B. mutus]
ENSBTAP00000050773-D48	5.95	3.92E-09	↓	hypothetical protein M91_00245, partial [B. mutus]
ENSBTAP00000006985-D1	5.95	3.92E-09	↓	integral membrane protein GPR180 precursor [B. taurus]
ENSP00000403333-D2	5.95	3.92E-09	↓	Hepatitis A virus cellular receptor 1, partial [B. mutus]
ENSBTAP00000018541-D1	5.95	3.92E-09	↓	polypeptide N-acetylgalactosaminyltransferase 3 isoform X1 [B. taurus]
yakG013325	5.96	9.80E-42	↓	hypothetical protein M91_16189 [B. mutus]
ENSBTAP00000035742-D1	5.98	1.76E-81	↓	myelin expression factor 2 [B. mutus]
ENSBTAP00000018091-D2	5.98	1.22E-173	↓	Peptidyl-prolyl cis-trans isomerase FKBP2, partial [B. mutus]
ENSBTAP00000006301-D1	5.98	6.75E-16	↓	protein Smaug homolog 2 isoform X1 [Bubalus bubalis]
ENSP00000380108-D1	5.98	6.75E-16	↓	BEN domain-containing protein 7 isoform X13 [Ovis aries musimon]
yakG038480	6.00	2.34E-29	↓	histone H2A.J-like [B. mutus]
ENSP00000348314-D1	6.00	2.34E-29	↓	Sperm flagellar protein 2 [B. mutus]
ENSP00000312709-D1	6.00	6.19E-243	↓	transcription initiation factor TFIID subunit 7 [Ovis aries]
ENSP00000354574-D1	6.01	1.77E-56	↓	mitochondrial sodium/hydrogen exchanger 9B2 [B. mutus]
ENSBTAP00000000929-D1	6.01	3.68E-16	↓	dual specificity protein phosphatase 16 isoform X1 [Ovis aries]
ENSP00000321706-D1	6.01	3.68E-16	↓	gem-associated protein 4 [B. mutus]
ENSP00000296266-D1	6.01	2.12E-09	↓	TPA: intraflagellar transport 122 homolog isoform 2 [B. taurus]
ENSP00000409199-D1	6.01	2.12E-09	↓	transmembrane glycoprotein NMB [B. mutus]
ENSBTAP00000026679-D1	6.01	2.12E-09	↓	Fat storage-inducing transmembrane protein 2 [B. mutus]
ENSP00000399013-D3	6.01	2.12E-09	↓	Contactin-associated protein-like 5, partial [B. mutus]
ENSBTAP00000028634-D1	6.01	2.12E-09	↓	carbonic anhydrase 14 [B. mutus]
yakA22965	6.01	2.12E-09	↓	transmembrane protein 230-like [B. mutus]
ENSBTAP00000010277-D1	6.01	2.12E-09	↓	WW domain-containing transcription regulator protein 1 isoform X1 [Bison bison bison]
ENSBTAP00000015648-D2	6.01	2.12E-09	↓	Iron-sulfur cluster assembly enzyme ISCU, mitochondrial, partial [B. mutus]
ENSP00000363391-D1	6.01	2.12E-09	↓	sperm-associated antigen 4 protein isoform X1 [B. mutus]
ENSP00000362469-D1	6.01	2.12E-09	↓	Solute carrier family 2, facilitated glucose transporter member 8 [B. mutus]
ENSP00000368266-D13	6.01	2.12E-09	↓	melanoma-associated antigen B10-like [B. mutus]
ENSBTAP00000053295-D1	6.01	2.12E-09	↓	Homogentisate 1,2-dioxygenase, partial [B. mutus]
ENSBTAP00000021893-D1	6.01	2.12E-09	↓	TPA: tankyrase 1 binding protein 1, 182kDa [B. taurus]
ENSBTAP00000006291-D1	6.01	2.12E-09	↓	charged multivesicular body protein 4c [Ovis aries]
ENSP00000386770-D1	6.01	2.12E-09	↓	dynein heavy chain 10, axonemal [B. mutus]
ENSBTAP00000009765-D1	6.01	2.12E-09	↓	Protein spinster-like protein 2, partial [B. mutus]
ENSP00000283228-D1	6.01	2.12E-09	↓	receptor-type tyrosine-protein phosphatase R isoform X1 [B. mutus]
ENSBTAP00000011023-D1	6.01	2.12E-09	↓	integrin alpha-10 isoform X1 [B. mutus]
ENSP00000314499-D1	6.01	2.12E-09	↓	Cyclin-G-associated kinase, partial [B. mutus]
ENSP00000367356-D1	6.01	2.12E-09	↓	Protein FAM171A1, partial [B. mutus]
ENSBTAP00000010281-D1	6.01	2.12E-09	↓	GDP-fucose protein O-fucosyltransferase 2, partial [B. mutus]
ENSP00000343023-D1	6.01	2.12E-09	↓	nuclear body protein SP140-like protein isoform X2 [Bison bison bison]
ENSP00000350332-D1	6.01	2.12E-09	↓	myosin-binding protein C, fast-type [B. mutus]
ENSBTAP00000033611-D1	6.01	2.12E-09	↓	interleukin-1 receptor-associated kinase 3 [Bison bison bison]
ENSBTAP00000020388-D1	6.01	2.12E-09	↓	protein prune homolog isoform X1 [B. mutus]
ENSBTAP00000005969-D1	6.01	2.12E-09	↓	Protein FAM78A, partial [B. mutus]
ENSP00000383022-D1	6.01	2.12E-09	↓	hypothetical protein M91_08020, partial [B. mutus]
ENSBTAP00000022398-D1	6.01	2.12E-09	↓	protein MTO1 homolog, mitochondrial [B. mutus]
ENSP00000233997-D1	6.01	2.12E-09	↓	azurocidin [B. taurus]
ENSP00000377771-D1	6.01	2.12E-09	↓	signal-induced proliferation-associated protein 1 [B. mutus]
ENSBTAP00000033286-D7	6.01	2.12E-09	↓	histone H2A type 2-B [Homo sapiens]
ENSBTAP00000000146-D1	6.01	2.12E-09	↓	Macrosialin, partial [B. mutus]
ENSBTAP00000022755-D1	6.01	2.12E-09	↓	Protein FAM8A1, partial [B. mutus]

ENSP00000404328-D10	6.01	2.12E-09	↓	hypothetical protein M91_18968 [B. mutus]
ENSP00000351657-D5	6.01	2.12E-09	↓	hypothetical protein M91_02900, partial [B. mutus]
ENSBTAP00000003691-D1	6.01	2.12E-09	↓	TRAF3-interacting JNK-activating modulator [B. mutus]
ENSP00000262320-D1	6.01	2.12E-09	↓	Axin-1 [B. mutus]
ENSP00000382790-D1	6.01	2.12E-09	↓	Putative uncharacterized protein ENSP00000382790, partial [B. mutus]
ENSBTAP00000007690-D1	6.01	2.12E-09	↓	Protein delta-like protein 2, partial [B. mutus]
ENSBTAP00000051834-D1	6.01	2.12E-09	↓	ETS homologous factor isoform X1 [B. mutus]
ENSP00000316933-D95	6.01	2.12E-09	↓	zinc finger protein 175-like isoform X1 [Bison bison bison]
ENSP00000265965-D1	6.01	2.12E-09	↓	Secretion-regulating guanine nucleotide exchange factor, partial [B. mutus]
ENSP00000377360-D1	6.05	2.11E-154	↓	beta-1,4-galactosyltransferase 4 [B. mutus]
ENSBTAP00000019683-D1	6.05	4.60E-113	↓	hypothetical protein M91_03735, partial [B. mutus]
ENSP00000338769-D1	6.07	1.09E-16	↓	arf-GAP with SH3 domain, ANK repeat and PH domain-containing protein 3 [Bison bison bison]
ENSBTAP00000012241-D1	6.07	1.15E-09	↓	transmembrane protein 100 [B. taurus]
ENSBTAP00000000767-D1	6.07	1.15E-09	↓	lymphocyte antigen 6 complex locus protein G6c precursor [B. taurus]
ENSBTAP00000040707-D4	6.07	1.15E-09	↓	keratin, type I cytoskeletal 25 [Pantholops hodgsonii]
ENSBTAP00000035589-D1	6.07	1.15E-09	↓	Putative phospholipid-transporting ATPase IB, partial [B. mutus]
ENSP00000215631-D1	6.07	1.15E-09	↓	growth arrest and DNA damage-inducible protein GADD45 beta [B. taurus]
ENSBTAP00000009231-D1	6.07	1.15E-09	↓	Cytochrome c oxidase assembly protein COX11, mitochondrial [B. mutus]
ENSBTAP00000001710-D1	6.07	1.15E-09	↓	25-hydroxycholesterol 7-alpha-hydroxylase, partial [B. mutus]
ENSP00000311596-D9	6.07	1.15E-09	↓	zinc finger protein 420 isoform X3 [Ovis aries musimon]
ENSP00000288840-D1	6.07	1.15E-09	↓	mothers against decapentaplegic homolog 6 [B. mutus]
ENSBTAP00000035880-D5	6.07	1.15E-09	↓	toll-like receptor 1 [B. mutus]
ENSBTAP00000050519-D1	6.07	1.15E-09	↓	Histone deacetylase 6, partial [B. mutus]
yakA18166	6.07	1.15E-09	↓	hypothetical protein M91_00317 [B. mutus]
ENSBTAP00000052476-D1	6.07	1.15E-09	↓	Serpin B7 [B. mutus]
ENSP00000330161-D1	6.07	1.15E-09	↓	Receptor-interacting serine/threonine-protein kinase 4, partial [B. mutus]
ENSBTAP00000015461-D1	6.07	1.15E-09	↓	zinc finger and SCAN domain-containing protein 5B-like [Bison bison bison]
ENSBTAP00000043387-D1	6.07	1.15E-09	↓	AP-4 complex subunit sigma-1 [B. mutus]
ENSBTAP00000020398-D1	6.07	1.15E-09	↓	WD repeat-containing protein 34 [B. mutus]
ENSP00000339207-D1	6.07	1.15E-09	↓	protein FAM122B isoform X4 [Bubalus bubalis]
ENSBTAP00000002315-D1	6.07	1.15E-09	↓	Beta-1,4-galactosyltransferase 7, partial [B. mutus]
ENSP00000353013-D1	6.07	1.15E-09	↓	C-type lectin domain family 14 member A, partial [B. mutus]
ENSBTAP00000053328-D1	6.07	1.15E-09	↓	protoporphyrinogen oxidase [B. mutus]
ENSBTAP00000049878-D1	6.07	1.15E-09	↓	T-cell-specific surface glycoprotein CD28, partial [B. mutus]
ENSBTAP00000023851-D1	6.07	1.15E-09	↓	DOMON domain-containing protein FRRS1L [Capra hircus]
ENSP00000331242-D1	6.07	1.15E-09	↓	Kremen protein 1, partial [B. mutus]
ENSP00000309096-D1	6.07	1.15E-09	↓	N-acetyllactosaminide beta-1,3-N-acetylglucosaminyltransferase [B. mutus]
ENSBTAP00000028723-D1	6.07	1.15E-09	↓	Protein LSM14-like protein B, partial [B. mutus]
ENSP00000226432-D1	6.07	1.15E-09	↓	PGAP2-interacting protein [B. mutus]
ENSP00000246801-D1	6.07	1.15E-09	↓	testis-specific serine kinase substrate isoform X1 [B. mutus]
ENSBTAP00000014872-D1	6.07	1.15E-09	↓	inositol polyphosphate multikinase [B. mutus]
ENSP00000374782-D82	6.07	1.15E-09	↓	hypothetical protein M91_06182, partial [B. mutus]
ENSBTAP00000029373-D1	6.07	1.15E-09	↓	Sphingomyelin phosphodiesterase 4, partial [B. mutus]
ENSP00000405133-D1	6.07	1.15E-09	↓	ATP-binding cassette sub-family A member 12 [B. mutus]
ENSP00000320560-D92	6.07	1.15E-09	↓	hypothetical protein M91_01389, partial [B. mutus]
ENSBTAP00000042051-D1	6.07	1.15E-09	↓	ras-responsive element-binding protein 1 [B. mutus]
ENSP00000334849-D1	6.07	1.15E-09	↓	transmembrane protein 104 [B. mutus]
ENSP00000396452-D1	6.07	1.15E-09	↓	Haloacid dehalogenase-like hydrolase domain-containing protein 1, partial [B. mutus]
ENSBTAP00000000073-D1	6.07	1.15E-09	↓	leucine-rich repeat neuronal protein 4 [B. mutus]
ENSP00000365678-D1	6.07	1.15E-09	↓	OTU domain-containing protein 1, partial [B. mutus]
ENSP00000341139-D1	6.07	1.15E-09	↓	uncharacterized protein C3orf62 homolog isoform X1 [B. taurus]
ENSBTAP00000050535-D1	6.07	1.15E-09	↓	ADP-ribosylation factor-like protein 10 [B. mutus]
ENSBTAP00000025597-D25	6.07	1.15E-09	↓	Pepsin A, partial [B. mutus]
ENSP00000388743-D1	6.07	1.15E-09	↓	SUN domain-containing protein 1, partial [B. mutus]
ENSBTAP00000047796-D1	6.07	9.82E-87	↓	DNA excision repair protein ERCC-8 [B. mutus]

ENSP00000348730-D1	6.08	6.21E-31	↓	dual specificity protein phosphatase 13 isoform X2 [Bison bison bison]
ENSP00000290949-D1	6.10	1.27E-59	↓	V-type proton ATPase subunit d 1 [Oryctolagus cuniculus]
ENSP00000414993-D1	6.10	3.40E-31	↓	Nesprin-4, partial [B. mutus]
yakA21591	6.10	5.96E-17	↓	Histone H4 [Heterocephalus glaber]
ENSBTAP00000023380-D1	6.12	0.00E+00	↓	serine/threonine-protein kinase Chk1 [Bison bison bison]
ENSP00000355963-D1	6.12	3.26E-17	↓	acyl-CoA:lysophosphatidylglycerol acyltransferase 1 isoform X1 [Bubalus bubalis]
ENSBTAP00000036503-D1	6.12	6.28E-10	↓	transmembrane protein ENSP00000340100 homolog [B. mutus]
yakA08242	6.12	6.28E-10	↓	hypothetical protein M91_18051 [B. mutus]
ENSP00000244799-D1	6.12	6.28E-10	↓	opsin-5 [B. mutus]
ENSBTAP00000007094-D1	6.12	6.28E-10	↓	Mitochondrial inner membrane protein COX18, partial [B. mutus]
ENSBTAP00000013193-D1	6.12	6.28E-10	↓	interferon regulatory factor 2 [B. mutus]
ENSBTAP00000003220-D1	6.12	6.28E-10	↓	hydroxyacylglutathione hydrolase-like protein [B. mutus]
ENSP00000336693-D1	6.12	6.28E-10	↓	abhydrolase domain-containing protein 12B [B. mutus]
ENSBTAP00000019975-D1	6.12	6.28E-10	↓	Fanconi anemia group G protein [B. mutus]
ENSP00000275954-D1	6.12	6.28E-10	↓	transmembrane protein 47 [Homo sapiens]
ENSBTAP00000037387-D1	6.12	6.28E-10	↓	Peroxisomal 2,4-dienoyl-CoA reductase, partial [B. mutus]
ENSP00000399147-D1	6.12	6.28E-10	↓	Melanoma-associated antigen D4 [B. mutus]
ENSBTAP00000040134-D1	6.12	6.28E-10	↓	hypothetical protein M91_00819, partial [B. mutus]
ENSBTAP00000046348-D1	6.12	6.28E-10	↓	mcKusick-Kaufman/Bardet-Biedl syndromes putative chaperonin [B. mutus]
yakG032084	6.12	6.28E-10	↓	60S ribosomal protein L4 [B. mutus]
ENSBTAP00000048623-D1	6.12	6.28E-10	↓	hypothetical protein M91_12283 [B. mutus]
ENSP00000347041-D1	6.12	6.28E-10	↓	fibromodulin [B. mutus]
ENSP00000380679-D1	6.12	6.28E-10	↓	Coiled-coil domain-containing protein 40, partial [B. mutus]
ENSBTAP00000008887-D1	6.12	6.28E-10	↓	6-phosphofructo-2-kinase/fructose-2,6-biphosphatase 4, partial [B. mutus]
ENSBTAP00000020069-D1	6.12	6.28E-10	↓	hypothetical protein M91_20563, partial [B. mutus]
ENSBTAP00000002115-D1	6.12	6.28E-10	↓	dual specificity mitogen-activated protein kinase kinase 6 isoform X1 [Bison bison bison]
ENSP00000376609-D1	6.12	6.28E-10	↓	G protein-coupled receptor kinase 5 [B. mutus]
ENSP00000394225-D1	6.12	6.28E-10	↓	FERM and PDZ domain-containing protein 4, partial [B. mutus]
ENSBTAP00000001989-D1	6.12	6.28E-10	↓	interferon-stimulated 20 kDa exonuclease-like 2 [B. taurus]
ENSBTAP00000000876-D1	6.12	6.28E-10	↓	GH3 domain-containing protein isoform X1 [B. mutus]
ENSBTAP00000007924-D1	6.12	6.28E-10	↓	Lysozyme C-1 [B. mutus]
ENSBTAP00000013524-D1	6.12	6.28E-10	↓	cell death activator CIDE-B [B. taurus]
ENSBTAP00000049569-D6	6.12	6.28E-10	↓	melanoma-associated antigen B2-like [B. taurus]
yakG015170	6.12	6.28E-10	↓	Kielin/chordin-like protein [B. mutus]
ENSP00000378089-D1	6.12	6.28E-10	↓	neuromedin-B isoform X1 [B. taurus]
ENSBTAP00000000213-D1	6.12	6.28E-10	↓	heparan sulfate glucosamine 3-O-sulfotransferase 5 [B. taurus]
ENSBTAP00000010033-D1	6.12	6.28E-10	↓	cytokine receptor common subunit gamma precursor [B. taurus]
ENSP00000353868-D1	6.12	6.28E-10	↓	Protein FAM113A [B. mutus]
ENSBTAP00000010182-D4	6.12	6.28E-10	↓	hypothetical protein M91_08472, partial [B. mutus]
ENSBTAP00000010972-D1	6.12	6.28E-10	↓	glutamyl aminopeptidase [B. mutus]
ENSBTAP00000023689-D1	6.12	6.28E-10	↓	phosphomevalonate kinase [B. mutus]
ENSBTAP00000047904-D1	6.12	6.28E-10	↓	hypothetical protein M91_09836, partial [B. mutus]
ENSBTAP00000002572-D2	6.12	6.28E-10	↓	Small cell adhesion glycoprotein, partial [B. mutus]
ENSBTAP00000022672-D1	6.12	6.28E-10	↓	olfactory receptor 51G1-like [B. mutus]
ENSBTAP00000025618-D1	6.12	6.28E-10	↓	nef-associated protein 1 [B. mutus]
ENSBTAP00000001074-D1	6.12	6.28E-10	↓	Putative ATP-dependent RNA helicase DDX49, partial [B. mutus]
ENSP00000275364-D2	6.12	6.28E-10	↓	Guanine nucleotide-binding protein subunit alpha-12, partial [B. mutus]
ENSBTAP00000009796-D1	6.12	6.28E-10	↓	beta-nerve growth factor-like [B. mutus]
ENSBTAP00000013175-D1	6.12	6.28E-10	↓	calreticulin-3 [B. mutus]
ENSP00000384667-D1	6.12	6.28E-10	↓	UPF0193 protein EVG1 isoform X1 [B. mutus]
ENSP00000405068-D1	6.12	6.28E-10	↓	Nucleolar protein 14, partial [B. mutus]
ENSP00000295984-D1	6.12	6.28E-10	↓	proline-rich transmembrane protein 3 [B. mutus]
ENSBTAP00000027347-D1	6.13	1.88E-46	↓	Procollagen C-endopeptidase enhancer 1, partial [B. mutus]
ENSP00000419005-D1	6.15	3.55E-76	↓	digestive organ expansion factor homolog [B. mutus]
ENSP00000356375-D1	6.15	5.64E-47	↓	Zinc finger and BTB domain-containing protein 41 [B. mutus]

ENSBTAP00000025926-D1	6.15	1.79E-17	↓	solute carrier family 25 member 39 isoform X1 [B. taurus]
ENSBTAP00000053550-D1	6.15	1.79E-17	↓	RNA-binding protein 33 [B. mutus]
ENSBTAP00000005462-D1	6.16	3.87E-151	↓	UBX domain-containing protein 8, partial [B. mutus]
ENSBTAP00000008069-D1	6.18	3.44E-10	↓	DENN domain-containing protein 3, partial [B. mutus]
ENSBTAP00000011212-D1	6.18	3.44E-10	↓	sphingosine kinase 1 [B. mutus]
ENSP00000371710-D1	6.18	3.44E-10	↓	Cadherin-18, partial [B. mutus]
ENSBTAP00000019398-D1	6.18	3.44E-10	↓	guanylate cyclase soluble subunit alpha-1-like [B. mutus]
ENSP00000400843-D1	6.18	3.44E-10	↓	cytosolic phospholipase A2 zeta [B. mutus]
ENSP00000306099-D1	6.18	3.44E-10	↓	Fibrinogen beta chain, partial [B. mutus]
ENSP00000368544-D1	6.18	3.44E-10	↓	Vitrin, partial [B. mutus]
ENSP00000362684-D1	6.18	3.44E-10	↓	protein FAM167B [B. mutus]
ENSP00000331691-D1	6.18	3.44E-10	↓	uncharacterized protein C12orf60 homolog [B. taurus]
ENSBTAP00000011776-D1	6.18	3.44E-10	↓	serine dehydratase-like [B. mutus]
ENSP00000360582-D1	6.18	3.44E-10	↓	NF-kappa-B-repressing factor [B. taurus]
ENSP00000307260-D1	6.18	3.44E-10	↓	Transducin beta-like protein 2 [B. mutus]
ENSBTAP00000032422-D53	6.18	3.44E-10	↓	mariner transposase [Homo sapiens]
ENSP00000358312-D1	6.18	3.44E-10	↓	peroxisomal membrane protein 11B isoform X1 [Bubalus bubalis]
ENSBTAP00000020025-D2	6.18	3.44E-10	↓	Putative serine protease 41, partial [B. mutus]
ENSP00000301061-D1	6.18	3.44E-10	↓	protein Wnt-10b [B. mutus]
ENSBTAP00000016314-D1	6.18	3.44E-10	↓	TBC1 domain family member 22A [B. mutus]
ENSP00000388599-D1	6.18	3.44E-10	↓	Prickle-like protein 3 [B. mutus]
ENSP00000340211-D1	6.18	3.44E-10	↓	Coronin-1B [B. mutus]
ENSP00000387909-D1	6.18	3.44E-10	↓	Secretory carrier-associated membrane protein 4, partial [B. mutus]
ENSBTAP00000012280-D1	6.18	3.44E-10	↓	septin-14 [B. mutus]
ENSP00000254260-D1	6.18	3.44E-10	↓	rhophilin-2 [B. mutus]
ENSBTAP00000014385-D1	6.18	3.44E-10	↓	dimethylaniline monooxygenase [N-oxide-forming] 5-like [B. mutus]
ENSBTAP00000005217-D1	6.18	3.44E-10	↓	insulin-like growth factor-binding protein 3, partial [Bison bison bison]
ENSBTAP00000052271-D1	6.18	3.44E-10	↓	transketolase-like protein 2 isoform X1 [B. mutus]
ENSBTAP00000049746-D1	6.18	3.44E-10	↓	Serine/threonine-protein kinase 11-interacting protein, partial [B. mutus]
ENSP00000383958-D2	6.18	3.44E-10	↓	nuclear factor 1 A-type-like, partial [Leptonychotes weddellii]
ENSP00000371230-D1	6.18	3.44E-10	↓	GTP:AMP phosphotransferase AK3, mitochondrial [B. taurus]
ENSP00000326671-D1	6.18	3.44E-10	↓	Multidrug and toxin extrusion protein 2 [B. mutus]
ENSBTAP00000030023-D1	6.18	3.44E-10	↓	mariner transposase [Homo sapiens]
ENSP00000377530-D1	6.18	3.44E-10	↓	Pyruvate carboxylase, mitochondrial [B. mutus]
ENSP00000265689-D1	6.18	3.44E-10	↓	Choline kinase alpha, partial [B. mutus]
ENSBTAP00000052631-D1	6.18	3.44E-10	↓	hypothetical protein M91_04763, partial [B. mutus]
ENSBTAP00000012245-D1	6.18	3.44E-10	↓	Prestin [B. mutus]
ENSBTAP00000040260-D1	6.18	3.44E-10	↓	Ephrin-A5, partial [B. mutus]
ENSBTAP00000027374-D1	6.20	5.40E-18	↓	arginine decarboxylase isoform X1 [B. mutus]
ENSP00000256186-D1	6.22	8.51E-80	↓	hypothetical protein M91_01350 [B. mutus]
ENSP00000309430-D1	6.23	2.98E-18	↓	TPA: GPI transamidase component PIG-S [B. taurus]
ENSP00000234256-D1	6.23	2.98E-18	↓	neutral amino acid transporter A isoform X1 [B. mutus]
ENSBTAP00000050952-D8	6.23	2.98E-18	↓	histone H3.1-like [B. mutus]
ENSBTAP00000016280-D1	6.23	1.88E-10	↓	SRR1-like protein, partial [B. mutus]
ENSBTAP00000022065-D1	6.23	1.88E-10	↓	Transmembrane protein 8A, partial [B. mutus]
ENSP00000304290-D1	6.23	1.88E-10	↓	acetylcholine receptor subunit beta isoform X1 [B. mutus]
ENSBTAP00000020299-D2	6.23	1.88E-10	↓	galectin-16 [B. mutus]
ENSP00000374268-D1	6.23	1.88E-10	↓	Glucoside xylosyltransferase 2, partial [B. mutus]
ENSP00000222584-D1	6.23	1.88E-10	↓	Transcription factor Sp4, partial [B. mutus]
ENSBTAP00000020641-D1	6.23	1.88E-10	↓	nuclear receptor subfamily 0 group B member 1 [B. taurus]
ENSP00000258229-D1	6.23	1.88E-10	↓	pecanex-like protein 2 [B. mutus]
ENSBTAP00000024345-D2	6.23	1.88E-10	↓	motile sperm domain-containing protein 2 [B. taurus]
ENSP00000301886-D1	6.23	1.88E-10	↓	sorting nexin-15 isoform X1 [Bison bison bison]
ENSBTAP00000036397-D1	6.23	1.88E-10	↓	R3H and coiled-coil domain-containing protein 1, partial [B. mutus]
ENSBTAP00000040583-D73	6.23	1.88E-10	↓	hypothetical protein M91_05670, partial [B. mutus]

ENSP00000319979-D1	6.23	1.88E-10	↓	UDP-GlcNAc:betaGal beta-1,3-N-acetylglucosaminyltransferase-like protein 1, partial [B. mutus]
ENSP00000330381-D1	6.23	1.88E-10	↓	WD repeat-containing protein 5B [B. mutus]
ENSBTAP00000030173-D3	6.23	1.88E-10	↓	Pregnancy-associated glycoprotein 2, partial [B. mutus]
ENSP00000389524-D1	6.23	1.88E-10	↓	heparan-alpha-glucosaminide N-acetyltransferase [B. mutus]
ENSP00000251289-D1	6.23	1.88E-10	↓	WD repeat-containing protein 18 [B. mutus]
ENSBTAP00000012258-D1	6.23	1.88E-10	↓	MORN repeat-containing protein 1, partial [B. mutus]
ENSBTAP00000018545-D1	6.23	1.88E-10	↓	Chain A, Crystal Structure Of The Human P21-Activated Kinase 4
ENSBTAP00000002346-D1	6.23	1.88E-10	↓	hydroxysteroid 11-beta-dehydrogenase 1-like protein isoform X1 [B. mutus]
ENSBTAP00000027579-D2	6.23	1.88E-10	↓	hypothetical protein M91_16352, partial [B. mutus]
ENSBTAP00000036565-D23	6.23	1.88E-10	↓	PRAME family member 20/21, partial [B. mutus]
ENSBTAP00000017826-D1	6.23	1.88E-10	↓	Sodium channel subunit beta-4 [B. mutus]
ENSBTAP00000031988-D1	6.23	1.88E-10	↓	Non-specific cytotoxic cell receptor protein 1-like protein, partial [B. mutus]
ENSBTAP00000000693-D1	6.23	1.88E-10	↓	Glutamate decarboxylase 1, partial [B. mutus]
ENSP00000375651-D1	6.23	1.88E-10	↓	myeloid-associated differentiation marker [B. mutus]
ENSBTAP00000023166-D1	6.23	1.88E-10	↓	protein FAM180A [B. taurus]
ENSP00000377355-D3	6.23	1.88E-10	↓	PDZ domain-containing RING finger protein 4, partial [B. mutus]
ENSP00000360748-D1	6.23	1.88E-10	↓	transmembrane protein 141 [B. taurus]
ENSBTAP00000035695-D2	6.23	1.88E-10	↓	Glutathione S-transferase A2, partial [B. mutus]
ENSBTAP00000007955-D1	6.23	1.88E-10	↓	protein HEXIM1 [B. taurus]
ENSBTAP00000037975-D1	6.23	1.88E-10	↓	thiamine transporter 2-like [B. mutus]
ENSP00000286307-D1	6.23	1.88E-10	↓	U7 snRNA-associated Sm-like protein LSm11 [B. mutus]
ENSP00000367890-D1	6.23	1.88E-10	↓	NAD kinase isoform 2 [Homo sapiens]
ENSP00000297904-D1	6.23	1.88E-10	↓	vascular endothelial growth factor D precursor [B. taurus]
ENSP00000367197-D1	6.23	1.88E-10	↓	secretagogin [B. mutus]
ENSBTAP00000006703-D1	6.23	1.88E-10	↓	regulation of nuclear pre-mRNA domain-containing protein 1B isoform X1 [Cricetulus griseus]
ENSP00000333926-D1	6.23	1.88E-10	↓	Multidrug resistance-associated protein 5 [B. mutus]
ENSP00000384672-D1	6.24	8.09E-50	↓	Methyl-CpG-binding domain protein 5 [B. mutus]
ENSBTAP00000004065-D1	6.25	1.65E-18	↓	Peroxisomal membrane protein PEX16, partial [B. mutus]
ENSP00000361967-D1	6.26	9.96E-177	↓	tubulin-specific chaperone C [B. taurus]
ENSBTAP00000003425-D1	6.27	2.29E-82	↓	Regulator of G-protein signaling 10, partial [B. mutus]
ENSP00000379638-D1	6.27	2.18E-98	↓	MANSC domain-containing protein 1 [B. mutus]
ENSBTAP00000028671-D1	6.28	8.03E-35	↓	Aspartyl aminopeptidase, partial [B. mutus]
ENSP00000310303-D1	6.28	9.11E-19	↓	hypothetical protein M91_15456, partial [B. mutus]
ENSBTAP00000017556-D1	6.28	9.11E-19	↓	prolow-density lipoprotein receptor-related protein 1 [B. mutus]
ENSBTAP00000023658-D1	6.28	9.11E-19	↓	Gamma-glutamyltranspeptidase 1, partial [B. mutus]
ENSP00000357904-D1	6.28	1.04E-10	↓	lysM and putative peptidoglycan-binding domain-containing protein 1 [B. mutus]
ENSBTAP00000049395-D1	6.28	1.04E-10	↓	1-phosphatidylinositol 4,5-bisphosphate phosphodiesterase delta-1-like [B. mutus]
ENSP00000294168-D1	6.28	1.04E-10	↓	TAF6-like RNA polymerase II p300/CBP-associated factor-associated factor 65 kDa subunit 6L isoform X2 [B. mutus]
ENSBTAP00000051908-D1	6.28	1.04E-10	↓	peroxisomal membrane protein 4 [B. mutus]
ENSBTAP00000030308-D1	6.28	1.04E-10	↓	ras-related protein Rab-33A [Sus scrofa]
ENSP00000399229-D1	6.28	1.04E-10	↓	matrix-remodeling-associated protein 8 [Tursiops truncatus]
ENSP00000331470-D1	6.28	1.04E-10	↓	homeobox protein DBX2 [Bison bison bison]
ENSBTAP00000010004-D1	6.28	1.04E-10	↓	Integrin-alpha FG-GAP repeat-containing protein 2 [B. mutus]
ENSBTAP00000053158-D1	6.28	1.04E-10	↓	Thioredoxin reductase 2, mitochondrial, partial [B. mutus]
ENSP00000362640-D1	6.28	1.04E-10	↓	Phosphorylase b kinase regulatory subunit alpha, skeletal muscle isoform [B. mutus]
ENSP00000246794-D1	6.28	1.04E-10	↓	Transmembrane gamma-carboxyglutamic acid protein 2 [B. mutus]
ENSP00000353846-D2	6.28	1.04E-10	↓	serine/threonine-protein kinase DCLK1 isoform X4 [Canis lupus familiaris]
ENSP00000338814-D1	6.28	1.04E-10	↓	BAG family molecular chaperone regulator 5, partial [B. mutus]
ENSBTAP00000017344-D1	6.28	1.04E-10	↓	NIPA-like protein 3 [B. taurus]
ENSP00000228887-D1	6.28	1.04E-10	↓	G-protein coupled receptor family C group 5 member D, partial [B. mutus]
ENSP00000378090-D2	6.28	1.04E-10	↓	DNA repair protein RAD51-like protein 4, partial [B. mutus]
ENSP00000350583-D1	6.28	1.04E-10	↓	Dipeptidyl peptidase 9, partial [B. mutus]
ENSP00000327487-D1	6.28	1.04E-10	↓	tRNA-splicing endonuclease subunit Sen54 [B. mutus]
yakG007721	6.28	1.04E-10	↓	Cyclin-dependent kinase inhibitor 3 [B. mutus]

ENSP00000361180-D1	6.28	1.04E-10	↓	general transcription factor 3C polypeptide 5 [B. mutus]
ENSBTAP00000037662-D1	6.28	1.04E-10	↓	5-hydroxytryptamine receptor 1E, partial [B. mutus]
ENSBTAP00000037133-D1	6.28	1.04E-10	↓	putative methyltransferase NSUN5 isoform X1 [B. mutus]
ENSBTAP00000013284-D1	6.28	1.04E-10	↓	early growth response protein 1 [B. mutus]
ENSP00000335618-D1	6.28	1.04E-10	↓	cytoplasmic phosphatidylinositol transfer protein 1 isoform X2 [B. taurus]
ENSBTAP00000041190-D1	6.28	1.04E-10	↓	Coiled-coil-helix-coiled-coil-helix domain-containing protein 6, partial [B. mutus]
ENSBTAP00000001667-D1	6.28	1.04E-10	↓	histidine-rich glycoprotein [Bubalus bubalis]
ENSP00000297440-D1	6.28	1.04E-10	↓	HEAT repeat-containing protein 2, partial [B. mutus]
ENSP00000377195-D1	6.28	1.04E-10	↓	drebrin isoform X1 [B. taurus]
ENSBTAP00000042029-D1	6.28	1.04E-10	↓	vascular cell adhesion protein 1-like isoform X1 [B. mutus]
ENSP00000370150-D1	6.28	1.04E-10	↓	ATPase WRNIP1, partial [B. mutus]
ENSP00000301738-D1	6.28	1.04E-10	↓	BTB/POZ domain-containing protein KCTD5, partial [B. mutus]
ENSP00000363799-D1	6.28	1.04E-10	↓	actin-like protein 7B [B. mutus]
ENSBTAP00000004604-D1	6.28	1.04E-10	↓	Kelch-like ECH-associated protein 1, partial [B. mutus]
ENSP00000312002-D2	6.28	1.04E-10	↓	hepatitis A virus cellular receptor 2-like, partial [B. mutus]
ENSBTAP00000003238-D1	6.30	0.00E+00	↓	Synaptonemal complex protein 3, partial [B. mutus]
ENSP00000377102-D1	6.30	1.30E-51	↓	protein EMSY isoform X4 [B. taurus]
ENSP00000338093-D1	6.30	5.04E-19	↓	Putative threonyl-tRNA synthetase 2, cytoplasmic, partial [B. mutus]
ENSBTAP00000002188-D1	6.32	5.72E-11	↓	Protein Wnt-7a, partial [B. mutus]
ENSP00000354361-D1	6.32	5.72E-11	↓	E3 ubiquitin-protein ligase RFWD3 [B. mutus]
ENSP00000229332-D1	6.32	5.72E-11	↓	C-type lectin domain family 4 member A [B. mutus]
ENSBTAP00000020682-D1	6.32	5.72E-11	↓	enkurin [B. taurus]
ENSBTAP00000016137-D1	6.32	5.72E-11	↓	Ceruloplasmin, partial [B. mutus]
ENSP00000358595-D1	6.32	5.72E-11	↓	glycoprotein hormones alpha chain precursor [B. taurus]
ENSBTAP00000006586-D1	6.32	5.72E-11	↓	UDP-glucose 4-epimerase, partial [B. mutus]
ENSBTAP00000017171-D1	6.32	5.72E-11	↓	lysine-specific demethylase PHF2-like [Bubalus bubalis]
ENSP00000282541-D1	6.32	5.72E-11	↓	glycerol-3-phosphate dehydrogenase 1-like protein-like [B. mutus]
ENSP00000396896-D1	6.32	5.72E-11	↓	dnaJ homolog subfamily C member 4 isoform X4 [B. taurus]
ENSP00000369889-D1	6.32	5.72E-11	↓	Collagen alpha-1(II) chain, partial [B. mutus]
ENSBTAP00000000920-D1	6.32	5.72E-11	↓	zinc finger and SCAN domain-containing protein 5B-like, partial [Bison bison bison]
ENSP00000351310-D1	6.32	5.72E-11	↓	collagen alpha-6(VI) chain isoform X2 [B. taurus]
ENSBTAP00000047776-D1	6.32	5.72E-11	↓	granulins [B. mutus]
ENSBTAP00000042366-D7	6.32	5.72E-11	↓	zinc finger protein 181-like [Orcinus orca]
ENSBTAP00000009562-D1	6.32	5.72E-11	↓	tectonic-2 [B. mutus]
ENSBTAP00000011757-D1	6.32	5.72E-11	↓	Protein CREG1, partial [B. mutus]
ENSP00000358814-D1	6.32	5.72E-11	↓	putative adenosylhomocysteinase 2 isoform X2 [Orcinus orca]
ENSBTAP00000023151-D1	6.32	5.72E-11	↓	Adenylate kinase isoenzyme 5, partial [B. mutus]
ENSBTAP00000018791-D1	6.32	5.72E-11	↓	WD repeat-containing protein 17 isoform X2 [B. taurus]
ENSP00000328016-D1	6.33	1.25E-52	↓	Tumor necrosis factor alpha-induced protein 8-like protein 3, partial [B. mutus]
ENSBTAP00000012699-D1	6.34	4.24E-36	↓	protein arginine N-methyltransferase 7 [B. mutus]
ENSBTAP00000020114-D1	6.35	3.86E-53	↓	growth arrest and DNA damage-inducible proteins-interacting protein 1 [B. mutus]
ENSBTAP00000001081-D1	6.37	3.16E-11	↓	PR domain zinc finger protein 1 [B. mutus]
ENSBTAP00000048094-D1	6.37	3.16E-11	↓	Neurochondrin, partial [B. mutus]
yakG039136	6.37	3.16E-11	↓	Laminin subunit alpha-5 [B. mutus]
ENSP00000361827-D5	6.37	3.16E-11	↓	G-protein coupled receptor-associated sorting protein 1 [B. taurus]
ENSBTAP00000025923-D1	6.37	3.16E-11	↓	DBH-like monooxygenase protein 1 [B. mutus]
ENSP00000311010-D1	6.37	3.16E-11	↓	ETS-related transcription factor Elf-5, partial [B. mutus]
ENSP00000304308-D1	6.37	3.16E-11	↓	Zinc finger protein 217, partial [B. mutus]
ENSP00000296414-D1	6.37	3.16E-11	↓	dual adapter for phosphotyrosine and 3-phosphotyrosine and 3-phosphoinositide [B. taurus]
ENSBTAP00000026562-D1	6.37	3.16E-11	↓	UDP-N-acetylglucosamine/UDP-glucose/GDP-mannose transporter, partial [B. mutus]
ENSP00000288368-D1	6.37	3.16E-11	↓	phosphatidylinositol 3,4,5-trisphosphate-dependent Rac exchanger 2 protein [B. taurus]
ENSBTAP00000015445-D1	6.37	3.16E-11	↓	epidermal growth factor receptor [Bison bison bison]
ENSBTAP00000043848-D1	6.37	3.16E-11	↓	syntaxin-5 [B. taurus]
ENSBTAP00000016859-D1	6.37	3.16E-11	↓	zinc finger protein 454 [B. mutus]
ENSP00000356037-D2	6.37	3.16E-11	↓	hypothetical protein M91_02271 [B. mutus]

ENSP00000382708-D1	6.37	3.16E-11	↓	hypothetical protein M91_06987, partial [B. mutus]
ENSBTAP00000016444-D1	6.37	3.16E-11	↓	protein FAM63B isoform X2 [B. mutus]
ENSP00000381120-D1	6.37	3.16E-11	↓	C-Maf-inducing protein isoform X1 [B. taurus]
ENSBTAP00000010260-D1	6.37	3.16E-11	↓	DNA polymerase mu [B. mutus]
ENSBTAP00000005929-D1	6.37	3.16E-11	↓	potassium channel subfamily K member 1 [B. mutus]
ENSP00000321449-D1	6.37	3.16E-11	↓	ribosomal RNA-processing protein 7 homolog A [Bubalus bubalis]
ENSP00000299201-D1	6.37	3.16E-11	↓	tRNA (adenine-N(1)-)-methyltransferase catalytic subunit TRMT61A, partial [B. mutus]
ENSBTAP00000008351-D1	6.37	3.16E-11	↓	Lysosomal protein NCU-G1, partial [B. mutus]
ENSBTAP00000049323-D1	6.37	3.16E-11	↓	P2Y purinoceptor 3-like [Bison bison bison]
ENSBTAP00000001496-D1	6.37	3.16E-11	↓	ADAM DEC1 protein, partial [B. mutus]
ENSBTAP00000029915-D3	6.37	3.16E-11	↓	histone-binding protein RBBP4 [Anolis carolinensis]
ENSP00000367764-D1	6.37	3.16E-11	↓	FERM domain-containing protein 4A [B. mutus]
ENSP00000390600-D7	6.37	3.16E-11	↓	Sodium channel protein type 11 subunit alpha, partial [B. mutus]
ENSBTAP00000040594-D1	6.37	3.16E-11	↓	dual specificity protein phosphatase 3 [B. mutus]
ENSP00000287773-D1	6.37	3.16E-11	↓	transmembrane protein 171 isoform X1 [B. mutus]
ENSBTAP00000043112-D1	6.37	3.16E-11	↓	low-density lipoprotein receptor-related protein 1B [Bison bison bison]
ENSP00000366024-D4	6.37	3.16E-11	↓	BOLA class I histocompatibility antigen, alpha chain BL3-7-like [Bubalus bubalis]
ENSBTAP00000013173-D1	6.37	3.16E-11	↓	Integrin beta-3, partial [B. mutus]
ENSP00000350415-D1	6.37	3.16E-11	↓	gap junction alpha-9 protein [B. mutus]
ENSBTAP00000021081-D1	6.37	3.16E-11	↓	WD repeat-containing protein WRAP73 [B. mutus]
ENSP00000334181-D1	6.37	3.16E-11	↓	lysine-specific demethylase 4D [B. mutus]
ENSBTAP00000040490-D1	6.37	3.16E-11	↓	sestrin-2 isoform X1 [B. taurus]
ENSBTAP00000023928-D1	6.37	3.16E-11	↓	Zinc finger protein GLIS1, partial [B. mutus]
ENSBTAP00000011793-D1	6.38	1.12E-157	↓	Protein FAN, partial [B. mutus]
ENSP00000280612-D1	6.39	1.16E-54	↓	Cystine/glutamate transporter, partial [B. mutus]
ENSP00000317159-D1	6.39	2.28E-37	↓	cytochrome c1, heme protein, mitochondrial [Equus przewalskii]
ENSP00000395281-D1	6.39	4.80E-20	↓	UPF0691 protein C9orf116 homolog [B. mutus]
ENSBTAP00000000187-D1	6.41	2.68E-20	↓	DNA damage-inducible transcript 4 protein [B. taurus]
ENSBTAP00000000329-D1	6.41	2.68E-20	↓	DNA-binding protein RFX6 [B. mutus]
ENSBTAP00000013270-D1	6.41	2.68E-20	↓	short transient receptor potential channel 5 [B. mutus]
ENSBTAP00000024503-D1	6.41	1.76E-11	↓	coiled-coil domain-containing protein 42A isoform X1 [B. mutus]
ENSBTAP00000047712-D1	6.41	1.76E-11	↓	Synaptosomal-associated protein 47, partial [B. mutus]
ENSBTAP00000031240-D1	6.41	1.76E-11	↓	Anthrax toxin receptor-like protein, partial [B. mutus]
ENSBTAP00000039004-D1	6.41	1.76E-11	↓	Lysosomal protective protein, partial [B. mutus]
ENSBTAP00000018026-D1	6.41	1.76E-11	↓	Endoplasmic reticulum aminopeptidase 1, partial [B. mutus]
ENSBTAP00000043209-D1	6.41	1.76E-11	↓	Chordin, partial [B. mutus]
ENSBTAP00000049324-D1	6.41	1.76E-11	↓	endothelin-1 receptor [Bison bison bison]
ENSP00000384806-D1	6.41	1.76E-11	↓	cAMP-specific 3',5'-cyclic phosphodiesterase 4D isoform X8 [Bison bison bison]
ENSBTAP00000011067-D1	6.41	1.76E-11	↓	Transcription factor Ovo-like 2 [B. mutus]
ENSBTAP00000045918-D1	6.41	1.76E-11	↓	Nucleosome assembly protein 1-like 3, partial [B. mutus]
ENSP00000399808-D1	6.41	1.76E-11	↓	homeobox protein Hox-C4 [Mus musculus]
ENSBTAP00000040579-D10	6.41	1.76E-11	↓	Putative protein ZNF720, partial [B. mutus]
ENSBTAP00000001642-D1	6.41	1.76E-11	↓	tissue-type plasminogen activator isoform X1 [B. mutus]
ENSBTAP00000025143-D1	6.41	1.76E-11	↓	diphthamide biosynthesis protein 2 [B. mutus]
ENSP00000417038-D1	6.41	1.76E-11	↓	phosphatase and actin regulator 2 isoform X3 [Bison bison bison]
ENSBTAP00000010741-D1	6.41	1.76E-11	↓	integrin alpha-IIb isoform X1 [Bison bison bison]
ENSP00000380594-D1	6.41	1.76E-11	↓	BPI fold-containing family C protein [B. mutus]
ENSP00000356259-D1	6.41	1.76E-11	↓	hypothetical protein M91_14731, partial [B. mutus]
yakG039444	6.41	1.76E-11	↓	Disks large-like protein 2 [B. mutus]
ENSP00000312770-D1	6.41	1.76E-11	↓	UPF0565 protein C2orf69 homolog [B. mutus]
ENSBTAP00000050234-D1	6.41	1.76E-11	↓	adenylate kinase isoenzyme 1 isoform X1 [B. mutus]
ENSBTAP00000042083-D1	6.41	1.76E-11	↓	renin receptor isoform X5 [Ovis aries musimon]
ENSP00000350576-D49	6.41	1.76E-11	↓	Zinc finger protein 709, partial [B. mutus]
ENSBTAP00000007834-D1	6.41	1.76E-11	↓	Tolloid-like protein 1, partial [B. mutus]
ENSBTAP00000013262-D1	6.41	1.76E-11	↓	Granzyme B, partial [B. mutus]

ENSP00000387303-D1	6.41	1.76E-11	↓	Putative ATP-dependent RNA helicase TDRD9 [B. mutus]
ENSBTAP00000009843-D1	6.41	1.76E-11	↓	protein unc-119 homolog B isoform X1 [Ovis aries]
ENSBTAP00000038553-D1	6.41	1.76E-11	↓	hypothetical protein M91_03828 [B. mutus]
ENSBTAP00000015935-D1	6.41	1.76E-11	↓	Rieske domain-containing protein [B. taurus]
ENSBTAP00000028761-D1	6.41	1.76E-11	↓	tubulin polyglutamylase complex subunit 2 [B. mutus]
ENSP00000262873-D1	6.41	1.76E-11	↓	myosin-7B [Ceratotherium simum simum]
ENSP00000257408-D1	6.41	1.76E-11	↓	Beta-klotho, partial [B. mutus]
ENSBTAP00000020271-D1	6.41	1.76E-11	↓	MFS-type transporter SLC18B1 [B. mutus]
ENSBTAP00000005660-D1	6.41	1.76E-11	↓	proto-oncogene c-Fos [B. taurus]
ENSBTAP00000023929-D1	6.42	1.80E-73	↓	Transmembrane and TPR repeat-containing protein 4 [B. mutus]
ENSP00000311657-D1	6.44	1.50E-20	↓	GRAM domain-containing protein 2 [Pantholops hodgsonii]
ENSBTAP00000020692-D1	6.44	1.98E-56	↓	histone deacetylase complex subunit SAP30 [Bubalus bubalis]
ENSP00000291634-D1	6.46	8.36E-21	↓	hypothetical protein M91_03260 [B. mutus]
ENSBTAP00000051730-D98	6.46	9.78E-12	↓	hypothetical protein M91_00313, partial [B. mutus]
ENSBTAP00000014753-D1	6.46	9.78E-12	↓	Acyl-coenzyme A thioesterase 12, partial [B. mutus]
ENSBTAP00000018406-D1	6.46	9.78E-12	↓	UPF0472 protein C16orf72-like [B. mutus]
ENSP00000318318-D1	6.46	9.78E-12	↓	Mannose-6-phosphate isomerase, partial [B. mutus]
ENSP00000355896-D1	6.46	9.78E-12	↓	transforming growth factor beta-2 isoform X1 [Ovis aries]
ENSBTAP00000026537-D1	6.46	9.78E-12	↓	bicaudal D-related protein 2 [B. mutus]
ENSBTAP00000030163-D1	6.46	9.78E-12	↓	Synaptotagmin-7, partial [B. mutus]
ENSP00000297990-D1	6.46	9.78E-12	↓	nucleolar protein 6 isoform X1 [B. mutus]
ENSBTAP00000043485-D1	6.46	9.78E-12	↓	Rho-related GTP-binding protein RhoF, partial [B. mutus]
ENSP00000333551-D1	6.46	9.78E-12	↓	Proline synthase co-transcribed bacterial-like protein, partial [B. mutus]
ENSP00000328553-D1	6.46	9.78E-12	↓	collagen alpha-4(IV) chain [B. mutus]
ENSBTAP00000022588-D1	6.46	9.78E-12	↓	Tyrosine-protein phosphatase non-receptor type 9, partial [B. mutus]
ENSP00000407726-D1	6.46	9.78E-12	↓	von Willebrand factor A domain-containing protein 5A, partial [B. mutus]
ENSP00000233121-D1	6.46	9.78E-12	↓	Microtubule-associated protein RP/EB family member 3 [Fukomys damarensis]
ENSP00000295454-D2	6.46	9.78E-12	↓	Gamma-aminobutyric acid receptor subunit beta-1, partial [B. mutus]
ENSBTAP00000010081-D1	6.46	9.78E-12	↓	Myosin-If, partial [B. mutus]
ENSBTAP00000010554-D1	6.46	9.78E-12	↓	oxytocin-neurophysin 1 [B. mutus]
ENSP00000384701-D1	6.46	9.78E-12	↓	V-set and transmembrane domain-containing protein 2A [B. mutus]
ENSP00000012049-D1	6.46	9.78E-12	↓	glutaminyl-peptide cyclotransferase-like protein [B. taurus]
ENSP00000407270-D1	6.46	9.78E-12	↓	TELO2-interacting protein 1 homolog [B. mutus]
ENSBTAP00000004187-D1	6.46	9.78E-12	↓	coiled-coil domain-containing protein 115 [B. mutus]
ENSP00000362095-D1	6.46	9.78E-12	↓	sushi repeat-containing protein SRPX2 precursor [B. taurus]
ENSBTAP00000026930-D1	6.46	9.78E-12	↓	Protein Wnt-5a, partial [B. mutus]
ENSBTAP00000016415-D1	6.46	9.78E-12	↓	transmembrane protein 53 [B. taurus]
ENSBTAP00000007448-D1	6.46	9.78E-12	↓	zinc transporter ZIP8 precursor [B. taurus]
yakG045754	6.46	9.78E-12	↓	hypothetical protein M91_01702 [B. mutus]
ENSP00000348501-D27	6.46	9.78E-12	↓	zinc finger protein 565 [B. mutus]
ENSP00000265018-D2	6.46	9.78E-12	↓	hypothetical protein M91_14807, partial [B. mutus]
ENSBTAP00000022098-D1	6.46	9.78E-12	↓	Serine/threonine-protein kinase Nek9, partial [B. mutus]
ENSBTAP00000027987-D1	6.46	9.78E-12	↓	protein FAM196A isoform X1 [B. taurus]
ENSP00000346550-D1	6.48	4.68E-21	↓	Annexin A6, partial [B. mutus]
ENSBTAP00000006241-D1	6.48	4.68E-21	↓	SH3KBP1-binding protein 1 [B. mutus]
ENSP00000363458-D1	6.50	2.63E-21	↓	Low density lipoprotein receptor adapter protein 1, partial [B. mutus]
ENSBTAP00000002742-D2	6.50	5.46E-12	↓	Protein FAM109A, partial [B. mutus]
ENSBTAP00000022621-D1	6.50	5.46E-12	↓	Coiled-coil domain-containing protein 125 [B. mutus]
ENSBTAP00000041566-D1	6.50	5.46E-12	↓	RNA-binding protein Nova-1 [B. taurus]
ENSBTAP00000016260-D1	6.50	5.46E-12	↓	anoctamin-2-like isoform X1 [Bubalus bubalis]
ENSBTAP00000048801-D2	6.50	5.46E-12	↓	hypothetical protein M91_06760, partial [B. mutus]
ENSBTAP00000018411-D1	6.50	5.46E-12	↓	Dual specificity protein phosphatase 1, partial [B. mutus]
ENSP00000416268-D2	6.50	5.46E-12	↓	hypothetical protein M91_15086, partial [B. mutus]
ENSP00000314099-D1	6.50	5.46E-12	↓	carbonic anhydrase 5B, mitochondrial [B. mutus]
ENSBTAP00000047693-D1	6.50	5.46E-12	↓	RAD51-associated protein 2 [B. mutus]

ENSP00000336127-D1	6.50	5.46E-12	↓	Tetratricopeptide repeat protein 7B, partial [B. mutus]
ENSP00000202677-D1	6.50	5.46E-12	↓	Ral GTPase-activating protein subunit alpha-2, partial [B. mutus]
ENSP00000219315-D1	6.50	5.46E-12	↓	N-lysine methyltransferase SETD6 [B. mutus]
ENSBTAP00000028955-D1	6.50	5.46E-12	↓	corneodesmosin [Bison bison bison]
ENSBTAP00000020296-D1	6.50	5.46E-12	↓	Beta-galactosidase, partial [B. mutus]
ENSP00000262370-D1	6.50	5.46E-12	↓	E3 ubiquitin-protein ligase MGRN1 isoform X1 [B. mutus]
ENSBTAP00000052681-D72	6.50	5.46E-12	↓	zinc finger protein 607-like [B. mutus]
ENSBTAP00000047282-D1	6.50	5.46E-12	↓	hypothetical protein M91_19927 [B. mutus]
ENSBTAP00000037365-D1	6.50	5.46E-12	↓	hypothetical protein M91_08744, partial [B. mutus]
ENSBTAP00000019429-D1	6.50	5.46E-12	↓	EF-hand domain-containing protein D1 isoform X1 [Bubalus bubalis]
ENSBTAP00000010911-D1	6.50	5.46E-12	↓	Endothelial protein C receptor [B. mutus]
ENSBTAP00000052590-D2	6.50	5.46E-12	↓	protein FAM127 [B. taurus]
ENSP00000400184-D1	6.50	5.46E-12	↓	hypothetical protein M91_17359 [B. mutus]
ENSBTAP00000053616-D1	6.50	5.46E-12	↓	Pappalysin-1, partial [B. mutus]
ENSBTAP00000003864-D1	6.50	5.46E-12	↓	leucine-rich repeat transmembrane neuronal protein 1 [B. mutus]
ENSP00000412574-D2	6.50	5.46E-12	↓	Torsin-1B, partial [B. mutus]
ENSP00000363852-D1	6.50	5.46E-12	↓	choline transporter-like protein 1 isoform X1 [Bison bison bison]
ENSBTAP00000028210-D1	6.50	5.46E-12	↓	NAD-dependent protein deacetylase sirtuin-4 isoform X1 [Bison bison bison]
ENSBTAP00000016560-D1	6.50	5.46E-12	↓	CUB domain-containing protein 1, partial [B. mutus]
yakG004195	6.54	3.06E-12	↓	hypothetical protein M91_01302 [B. mutus]
ENSP00000325819-D1	6.54	3.06E-12	↓	COUP transcription factor 1, partial [B. mutus]
ENSP00000327650-D1	6.54	3.06E-12	↓	vacuolar protein sorting-associated protein 33B isoform X1 [B. mutus]
ENSBTAP00000021517-D1	6.54	3.06E-12	↓	Protein flightless-1-like protein, partial [B. mutus]
ENSP00000368278-D1	6.54	3.06E-12	↓	interleukin-1 receptor accessory protein-like 1 isoform X2 [B. taurus]
ENSBTAP00000042269-D1	6.54	3.06E-12	↓	hypothetical protein M91_11455, partial [B. mutus]
ENSBTAP00000024193-D1	6.54	3.06E-12	↓	uncharacterized protein C12orf50 homolog isoform X1 [B. mutus]
ENSP00000397939-D1	6.54	3.06E-12	↓	Alcohol dehydrogenase 4, partial [B. mutus]
ENSBTAP00000025642-D1	6.54	3.06E-12	↓	GLIPR1-like protein 2, partial [B. mutus]
ENSP00000276708-D1	6.54	3.06E-12	↓	Gasdermin-C [B. mutus]
ENSBTAP00000051820-D2	6.54	3.06E-12	↓	serum amyloid A protein [B. taurus]
ENSP00000411217-D1	6.54	3.06E-12	↓	protein CC2D2B isoform X31 [B. taurus]
ENSBTAP00000016414-D1	6.54	3.06E-12	↓	hypothetical protein M91_04351, partial [B. mutus]
ENSP00000371376-D1	6.54	3.06E-12	↓	toll-like receptor 6 [B. mutus]
ENSP00000390436-D1	6.54	3.06E-12	↓	zinc finger protein GLI2 [B. mutus]
ENSBTAP00000053478-D1	6.54	3.06E-12	↓	Contactin-5, partial [B. mutus]
ENSP00000323500-D1	6.54	3.06E-12	↓	Plasma glutamate carboxypeptidase, partial [B. mutus]
ENSP00000385610-D1	6.54	3.06E-12	↓	RNA-binding E3 ubiquitin-protein ligase MEX3C isoform X2, partial [B. taurus]
ENSP00000371886-D1	6.54	3.06E-12	↓	Cytosolic phospholipase A2 beta [B. mutus]
ENSP00000278198-D1	6.54	3.06E-12	↓	leucine-rich repeat-containing protein 4C-like isoform X1 [B. mutus]
ENSP00000408162-D1	6.54	3.06E-12	↓	hCG2042951 [Homo sapiens]
ENSP00000416418-D1	6.54	3.06E-12	↓	Sodium/hydrogen exchanger 8 [B. mutus]
ENSBTAP00000008383-D1	6.54	3.06E-12	↓	gamma-tubulin complex component 2 isoform X2 [B. mutus]
ENSBTAP00000049567-D1	6.54	3.06E-12	↓	FLYWCH family member 2 [B. taurus]
ENSP00000225235-D1	6.54	3.06E-12	↓	TBC1 domain family member 12, partial [B. mutus]
ENSP00000298953-D1	6.54	3.06E-12	↓	hypothetical protein M91_04339, partial [B. mutus]
ENSBTAP00000027511-D4	6.54	3.06E-12	↓	guanine nucleotide-binding protein G(t) subunit alpha-3 [B. mutus]
ENSP00000349640-D1	6.54	3.06E-12	↓	microspherule protein 1 isoform X1 [Otolemur garnettii]
ENSP00000402537-D1	6.54	3.06E-12	↓	myotubularin-related protein 10 [B. taurus]
ENSBTAP00000049393-D1	6.54	3.06E-12	↓	DNA-binding death effector domain-containing protein 2 [Ovis aries musimon]
ENSBTAP00000010060-D1	6.54	3.06E-12	↓	phosphatidylinositol N-acetylglucosaminyltransferase subunit A [B. taurus]
ENSP00000261180-D1	6.56	1.24E-137	↓	thyrotropin-releasing hormone-degrading ectoenzyme [Bison bison bison]
ENSP00000270281-D1	6.56	4.68E-22	↓	cleft lip and palate transmembrane protein 1 homolog [B. taurus]
ENSP00000287196-D1	6.58	2.64E-22	↓	Poly [ADP-ribose] polymerase 6, partial [B. mutus]
ENSP00000314897-D1	6.58	1.72E-12	↓	angiopoietin-2 precursor [B. taurus]
ENSBTAP00000028416-D1	6.58	1.72E-12	↓	Secretory carrier-associated membrane protein 5, partial [B. mutus]

ENSP00000361791-D2	6.58	1.72E-12	↓	Zinc finger protein 643, partial [B. mutus]
ENSP00000409597-D73	6.58	1.72E-12	↓	zinc finger protein 12 isoform X2 [B. mutus]
ENSBTAP00000016010-D1	6.58	1.72E-12	↓	asialoglycoprotein receptor 1 [B. mutus]
ENSBTAP00000001891-D1	6.58	1.72E-12	↓	Solute carrier family 15 member 2, partial [B. mutus]
ENSBTAP00000017956-D1	6.58	1.72E-12	↓	IQ and AAA domain-containing protein 1, partial [B. mutus]
ENSP00000356954-D1	6.58	1.72E-12	↓	connective tissue growth factor [Bison bison bison]
ENSBTAP00000023987-D2	6.58	1.72E-12	↓	B-cell lymphoma/leukemia 11A [B. mutus]
ENSBTAP00000006186-D1	6.58	1.72E-12	↓	Akirin-1, partial [B. mutus]
ENSP00000361634-D1	6.58	1.72E-12	↓	Endophilin-B2, partial [B. mutus]
ENSBTAP00000022087-D1	6.58	1.72E-12	↓	uncharacterized protein LOC786914 [B. taurus]
ENSP00000381227-D1	6.58	1.72E-12	↓	Triadin, partial [B. mutus]
ENSBTAP00000016524-D1	6.58	1.72E-12	↓	Disintegrin and metalloproteinase domain-containing protein 12, partial [B. mutus]
ENSBTAP00000053170-D1	6.58	1.72E-12	↓	Pleckstrin-like protein domain-containing family A member 4 [B. mutus]
ENSBTAP00000051871-D3	6.58	1.72E-12	↓	ankyrin repeat domain-containing protein 26-like isoform X1 [B. taurus]
ENSP00000369129-D1	6.58	1.72E-12	↓	Desmoplakin, partial [B. mutus]
yakG029197	6.58	1.72E-12	↓	hypothetical protein M91_00751 [B. mutus]
ENSBTAP00000001378-D1	6.58	1.72E-12	↓	Mitogen-activated protein kinase kinase kinase kinase 2, partial [B. mutus]
ENSBTAP00000015785-D1	6.58	1.72E-12	↓	Glutamate receptor-interacting protein 2, partial [B. mutus]
ENSBTAP00000018609-D1	6.58	1.72E-12	↓	torsin-3A [B. mutus]
ENSP00000297532-D1	6.58	1.72E-12	↓	Fas-activated serine/threonine kinase [B. mutus]
ENSBTAP00000001526-D1	6.58	1.72E-12	↓	Transcription factor HIVEP2 [B. mutus]
ENSBTAP00000030128-D13	6.58	1.72E-12	↓	hypothetical protein M91_07623, partial [B. mutus]
ENSBTAP00000043868-D1	6.58	1.72E-12	↓	Solute carrier family 12 member 1 [B. mutus]
ENSBTAP00000013095-D1	6.58	1.72E-12	↓	Major histocompatibility complex class I-related protein, partial [B. mutus]
ENSBTAP00000001619-D1	6.60	1.49E-22	↓	RNA-binding protein 14 isoform X1 [B. mutus]
ENSBTAP00000026420-D1	6.61	8.58E-123	↓	39S ribosomal protein L46, mitochondrial [B. mutus]
ENSBTAP00000043064-D1	6.62	8.41E-23	↓	Calsyntenin-1, partial [B. mutus]
ENSBTAP00000014883-D1	6.62	9.69E-13	↓	calponin-1 [Camelus ferus]
ENSBTAP00000008725-D1	6.62	9.69E-13	↓	uncharacterized protein C4orf19 homolog [B. mutus]
ENSBTAP00000052746-D1	6.62	9.69E-13	↓	methylenetetrahydrofolate reductase isoform X1 [B. mutus]
ENSBTAP00000006686-D1	6.62	9.69E-13	↓	Zinc finger HIT domain-containing protein 2 [B. mutus]
ENSP00000334100-D1	6.62	9.69E-13	↓	exocyst complex component 7 isoformX2 [Equus caballus]
ENSBTAP00000015489-D1	6.62	9.69E-13	↓	SHC-transforming protein 4 [B. mutus]
ENSP00000269209-D1	6.62	9.69E-13	↓	GRB2-associated and regulator of MAPK protein isoform X1 [B. taurus]
ENSP00000357600-D1	6.62	9.69E-13	↓	Testis-specific Y-encoded-like protein 4, partial [B. mutus]
ENSBTAP00000047910-D1	6.62	9.69E-13	↓	gonadotropin-releasing hormone II receptor-like [B. mutus]
ENSP00000386167-D18	6.62	9.69E-13	↓	hypothetical protein M91_01396 [B. mutus]
ENSP00000362639-D1	6.62	9.69E-13	↓	rab9 effector protein with kelch motifs isoform X1 [B. mutus]
ENSBTAP00000047419-D1	6.62	9.69E-13	↓	Zona pellucida-binding protein 1, partial [B. mutus]
ENSBTAP00000050891-D1	6.62	9.69E-13	↓	WD repeat-containing protein 38, partial [B. mutus]
ENSBTAP00000010787-D1	6.62	9.69E-13	↓	glucosidase 2 subunit beta precursor [B. taurus]
ENSBTAP00000046933-D1	6.62	9.69E-13	↓	Myotubularin-related protein 8, partial [B. mutus]
ENSP00000282406-D1	6.62	9.69E-13	↓	pleckstrin homology domain-containing family H member 2, partial [B. mutus]
ENSBTAP00000037982-D1	6.62	9.69E-13	↓	Dedicator of cytokinesis protein 10, partial [B. mutus]
ENSP00000403894-D11	6.62	9.69E-13	↓	tubulin alpha-3 chain-like [Eptesicus fuscus]
ENSP00000344816-D1	6.62	9.69E-13	↓	BSD domain-containing protein 1, partial [B. mutus]
ENSBTAP00000034554-D1	6.62	9.69E-13	↓	TPA: hypothetical protein BOS_18285 [B. taurus]
ENSP00000256010-D1	6.62	9.69E-13	↓	neurotensin/neuromedin N precursor [B. taurus]
ENSBTAP00000033686-D1	6.62	9.69E-13	↓	Chloride anion exchanger, partial [B. mutus]
ENSP00000322427-D19	6.65	1.13E-84	↓	zinc finger protein 160-like isoform X1 [B. taurus]
ENSBTAP00000002176-D1	6.66	5.47E-13	↓	Peptide deformylase, mitochondrial, partial [B. mutus]
ENSP00000366384-D1	6.66	5.47E-13	↓	Zinc finger protein 311, partial [B. mutus]
ENSP00000393633-D1	6.66	5.47E-13	↓	H(+)/Cl(-) exchange transporter 5, partial [B. mutus]
ENSBTAP00000010971-D2	6.66	5.47E-13	↓	hippocalcin-like protein 1 [B. taurus]
ENSP00000398365-D1	6.66	5.47E-13	↓	Dickkopf-related protein 3, partial [B. mutus]

ENSBTAP00000015493-D1	6.66	5.47E-13	↓	peptidase M20 domain-containing protein 2 [B. mutus]
ENSBTAP00000006513-D1	6.66	5.47E-13	↓	major facilitator superfamily domain-containing protein 4 isoform X1 [B. mutus]
ENSP00000371322-D2	6.66	5.47E-13	↓	60S ribosomal protein L13a, partial [B. mutus]
ENSBTAP00000028215-D1	6.66	5.47E-13	↓	coiled-coil domain-containing protein 134 isoform X1 [Bubalus bubalis]
ENSP00000391053-D1	6.66	5.47E-13	↓	V-set and immunoglobulin domain-containing protein 1, partial [B. mutus]
ENSBTAP00000013619-D1	6.66	5.47E-13	↓	intercellular adhesion molecule 4 [B. mutus]
ENSP00000306185-D1	6.66	5.47E-13	↓	Anthrax toxin receptor 2, partial [B. mutus]
ENSBTAP00000011546-D1	6.66	5.47E-13	↓	protease-associated domain-containing protein 1 [Sus scrofa]
ENSBTAP00000050674-D1	6.66	5.47E-13	↓	zinc transporter SLC39A7 precursor [B. taurus]
ENSBTAP00000018616-D1	6.66	5.47E-13	↓	Clusterin-like protein 1, partial [B. mutus]
ENSBTAP00000043056-D1	6.66	5.47E-13	↓	Conserved oligomeric Golgi complex subunit 7 [B. mutus]
ENSP00000265191-D1	6.66	5.47E-13	↓	nucleoside diphosphate kinase homolog 5 isoform X1 [Ovis aries]
ENSP00000378524-D1	6.66	5.47E-13	↓	leucine-rich repeat and IQ domain-containing protein 3 [B. mutus]
ENSBTAP00000012968-D1	6.66	5.47E-13	↓	cyclic nucleotide-gated cation channel alpha-3 [B. mutus]
ENSP00000412775-D1	6.66	5.47E-13	↓	Cell division protein kinase 15 [B. mutus]
ENSBTAP00000001917-D1	6.66	4.24E-44	↓	Tankyrase-1 [B. mutus]
ENSP00000400909-D1	6.66	4.24E-44	↓	Lysocardiolipin acyltransferase 1, partial [B. mutus]
ENSBTAP00000021600-D1	6.67	2.41E-44	↓	Ribonuclease H1, partial [B. mutus]
ENSBTAP00000021078-D1	6.69	8.71E-24	↓	RecName: Full=Tumor protein p63-regulated gene 1-like protein; AltName: Full=Mossy fiber terminal-associated vertebrate-specific presynaptic protein; AltName: Full=Protein FAM79A [B. taurus]
ENSP00000349687-D1	6.69	3.10E-13	↓	ganglioside GM2 activator [B. mutus]
ENSBTAP00000009389-D2	6.69	3.10E-13	↓	Lithostathine, partial [B. mutus]
ENSP00000326813-D1	6.69	3.10E-13	↓	ubiquitin thioesterase OTU1 [B. mutus]
ENSBTAP00000024371-D1	6.69	3.10E-13	↓	c-X-C motif chemokine 16 [B. mutus]
ENSBTAP00000053306-D1	6.69	3.10E-13	↓	protein phosphatase 1 regulatory subunit 1C isoform X1 [B. mutus]
ENSP00000237536-D1	6.69	3.10E-13	↓	protein SOGA1 [B. mutus]
ENSBTAP00000028460-D1	6.69	3.10E-13	↓	Acidic repeat-containing protein, partial [B. mutus]
ENSBTAP00000007048-D1	6.69	3.10E-13	↓	suppression of tumorigenicity 5 protein isoform X1 [B. mutus]
ENSBTAP00000048801-D1	6.69	3.10E-13	↓	Developmental pluripotency-associated protein 2, partial [B. mutus]
ENSP00000263160-D1	6.69	3.10E-13	↓	vesicular glutamate transporter 2 [B. taurus]
ENSP00000302088-D1	6.69	3.10E-13	↓	protein RER1 [Ovis aries]
ENSP00000358320-D1	6.69	3.10E-13	↓	DNA-directed RNA polymerase III subunit RPC7-like [B. taurus]
ENSP00000344223-D1	6.69	3.10E-13	↓	Membrane-bound transcription factor site-1 protease [B. mutus]
ENSBTAP00000022174-D1	6.69	3.10E-13	↓	phosphotriesterase-related protein [B. taurus]
ENSBTAP00000025337-D1	6.69	3.10E-13	↓	Rho GTPase-activating protein 28, partial [B. mutus]
ENSP00000256999-D1	6.69	3.10E-13	↓	Glutamate carboxypeptidase 2, partial [B. mutus]
ENSBTAP00000020365-D1	6.69	3.10E-13	↓	Argininosuccinate lyase, partial [B. mutus]
ENSBTAP00000026248-D1	6.69	3.10E-13	↓	BCL2L2 [Felis catus]
ENSP00000379818-D1	6.69	3.10E-13	↓	glutamate receptor ionotropic, NMDA 2A isoform X1 [Bison bison bison]
ENSP00000384360-D1	6.69	3.10E-13	↓	ubiquitin carboxyl-terminal hydrolase 26 [B. taurus]
ENSP00000296869-D1	6.69	3.10E-13	↓	Long-chain-fatty-acid--CoA ligase 6, partial [B. mutus]
ENSP00000385025-D1	6.69	3.10E-13	↓	smith-Magenis syndrome chromosomal region candidate gene 8 protein [B. mutus]
ENSP00000287202-D1	6.69	3.10E-13	↓	CUGBP Elav-like family member 6 [B. taurus]
ENSP00000243052-D1	6.69	3.10E-13	↓	calcium/calmodulin-dependent 3',5'-cyclic nucleotide phosphodiesterase 1B isoform X1 [B. taurus]
ENSBTAP00000024333-D1	6.69	3.10E-13	↓	vesicle-associated membrane protein 5 [B. taurus]
ENSBTAP00000028292-D1	6.69	3.10E-13	↓	DLA class II histocompatibility antigen, DR-1 beta chain-like [Bison bison bison]
ENSBTAP00000045387-D1	6.69	3.10E-13	↓	desmocollin-3 isoform X1 [B. mutus]
ENSBTAP00000045240-D1	6.70	4.45E-45	↓	phosphatidylinositol phosphatase PTPRQ isoform X1 [B. taurus]
ENSBTAP00000045070-D1	6.70	4.45E-45	↓	Interleukin-18 receptor accessory protein, partial [B. mutus]
ENSBTAP00000011113-D85	6.71	2.54E-45	↓	hypothetical protein M91_05198, partial [B. mutus]
ENSBTAP00000010778-D1	6.71	4.96E-24	↓	ATPase SWSAP1 [Bubalus bubalis]
ENSP00000361359-D1	6.73	2.82E-24	↓	Tumor necrosis factor receptor superfamily member 5 [B. mutus]
ENSBTAP00000007149-D1	6.73	2.82E-24	↓	LIM and cysteine-rich domains protein 1, partial [B. mutus]
ENSP00000360942-D1	6.73	1.76E-13	↓	Tumor necrosis factor receptor superfamily member 6, partial [B. mutus]
ENSBTAP00000003094-D1	6.73	1.76E-13	↓	transforming growth factor beta-1-induced transcript 1 protein isoform X1 [Bubalus bubalis]

ENSBTAP00000053857-D2	6.73	1.76E-13	↓	ankyrin repeat and BTB/POZ domain-containing protein 2 [B. taurus]
ENSP00000353393-D1	6.73	1.76E-13	↓	coagulation factor VIII [B. mutus]
ENSP00000357304-D1	6.73	1.76E-13	↓	UPF0469 protein KIAA0907 [B. mutus]
ENSBTAP00000012334-D1	6.73	1.76E-13	↓	Coiled-coil domain-containing protein 36, partial [B. mutus]
ENSBTAP00000039420-D1	6.73	1.76E-13	↓	Zinc finger protein 408 [B. mutus]
ENSP00000396052-D1	6.73	1.76E-13	↓	Breast cancer metastasis-suppressor 1-like protein, partial [B. mutus]
ENSP00000355846-D1	6.73	1.76E-13	↓	toll-like receptor 5 [B. mutus]
ENSBTAP00000003645-D1	6.73	1.76E-13	↓	Sterile alpha and TIR motif-containing protein 1, partial [B. mutus]
ENSP00000243578-D1	6.73	1.76E-13	↓	B9 domain-containing protein 2 [B. taurus]
ENSP00000349824-D1	6.73	1.76E-13	↓	ralBP1-associated Eps domain-containing protein 2 isoform X5 [B. taurus]
ENSP00000404826-D1	6.73	1.76E-13	↓	hypothetical protein M91_21383, partial [B. mutus]
ENSP00000390984-D1	6.73	1.76E-13	↓	Nuclear protein 1 [B. mutus]
ENSBTAP00000035972-D1	6.73	1.76E-13	↓	Protein YIPF2 [B. mutus]
ENSBTAP00000002731-D1	6.73	1.76E-13	↓	hypothetical protein M91_18129, partial [B. mutus]
ENSP00000261822-D1	6.73	1.76E-13	↓	B-cell CLL/lymphoma 7 protein family member A, partial [B. mutus]
ENSP00000306396-D1	6.73	1.76E-13	↓	Protein RNF187, partial [B. mutus]
ENSP00000401214-D1	6.73	1.76E-13	↓	EF-hand calcium-binding domain-containing protein 2 [B. mutus]
ENSBTAP00000029666-D1	6.73	1.76E-13	↓	RILP-like protein 1 isoform X1 [B. mutus]
ENSBTAP00000022675-D1	6.74	2.69E-46	↓	lysophosphatidic acid phosphatase type 6 isoform X1 [B. mutus]
ENSP00000416040-D1	6.74	1.61E-24	↓	UPF0606 protein KIAA1549, partial [B. mutus]
ENSP00000289968-D1	6.76	1.00E-13	↓	Rho GTPase-activating protein 17, partial [B. mutus]
ENSBTAP00000006167-D1	6.76	1.00E-13	↓	alpha-2-macroglobulin [B. mutus]
ENSBTAP00000038660-D2	6.76	1.00E-13	↓	protein FAM32A-like [B. mutus]
ENSBTAP00000022815-D1	6.76	1.00E-13	↓	Matrilin-2 [B. mutus]
ENSBTAP00000041555-D1	6.76	1.00E-13	↓	peroxisome proliferator-activated receptor gamma [B. taurus]
ENSP00000296325-D1	6.76	1.00E-13	↓	Alpha-2-macroglobulin receptor-associated protein [B. mutus]
ENSBTAP00000015357-D1	6.76	1.00E-13	↓	mediator of RNA polymerase II transcription subunit 22 [B. taurus]
ENSBTAP00000008393-D1	6.76	1.00E-13	↓	leucine-rich repeat and calponin homology domain-containing protein 2 isoform X1 [B. mutus]
ENSP00000371110-D1	6.76	1.00E-13	↓	2-amino-3-ketobutyrate coenzyme A ligase, mitochondrial [B. mutus]
ENSBTAP00000003618-D1	6.76	1.00E-13	↓	leucine-rich alpha-2-glycoprotein [B. mutus]
ENSBTAP00000038777-D1	6.76	1.00E-13	↓	putative inactive neutral ceramidase B isoform X2 [B. taurus]
ENSBTAP00000011702-D1	6.76	1.00E-13	↓	Progestin and adipoQ receptor family member 3, partial [B. mutus]
ENSP00000286234-D1	6.76	1.00E-13	↓	DEP domain-containing mTOR-interacting protein, partial [B. mutus]
ENSBTAP00000002838-D1	6.76	1.00E-13	↓	hypothetical protein M91_16539, partial [B. mutus]
ENSP00000352329-D2	6.76	1.00E-13	↓	fibroblast growth factor 2 precursor [B. taurus]
ENSBTAP00000043442-D1	6.76	1.00E-13	↓	sprouty-related, EVH1 domain-containing protein 1 [Pantholops hodgsonii]
ENSBTAP00000023909-D1	6.76	1.00E-13	↓	carbonic anhydrase 4 [B. mutus]
ENSP00000221459-D1	6.76	1.00E-13	↓	Protein lin-7-like protein B, partial [B. mutus]
ENSBTAP00000011523-D1	6.76	1.00E-13	↓	[Pyruvate dehydrogenase [lipoamide]] kinase isozyme 2, mitochondrial, partial [B. mutus]
yakA13537	6.76	1.00E-13	↓	Protein phosphatase 1 regulatory subunit 14A [B. mutus]
ENSBTAP00000007891-D1	6.76	1.00E-13	↓	Gamma-2-syntrophin, partial [B. mutus]
ENSBTAP00000019112-D1	6.76	1.00E-13	↓	caspase recruitment domain-containing protein 6 [B. mutus]
ENSBTAP00000028431-D1	6.76	1.00E-13	↓	dynein assembly factor with WDR repeat domains 1 [B. mutus]
ENSP00000267838-D1	6.76	1.00E-13	↓	lysM and putative peptidoglycan-binding domain-containing protein 2 [B. mutus]
ENSBTAP00000001758-D1	6.76	1.00E-13	↓	growth hormone receptor [B. mutus]
ENSBTAP00000001736-D1	6.76	1.00E-13	↓	doublesex- and mab-3-related transcription factor A1 [B. taurus]
ENSP00000406751-D1	6.76	1.00E-13	↓	Cryptochrome-2, partial [B. mutus]
ENSP00000296202-D1	6.76	1.00E-13	↓	Nicotinamide mononucleotide adenylyltransferase 3, partial [B. mutus]
ENSBTAP00000001651-D3	6.76	1.00E-13	↓	transcription factor AP-2 gamma isoform X3 [Ovis aries musimon]
ENSP00000318770-D1	6.76	1.00E-13	↓	aquaporin-11 isoform X1 [B. taurus]
ENSBTAP00000046053-D2	6.76	1.00E-13	↓	Guanine nucleotide-binding protein G(I)/G(S)/G(O) subunit gamma-11 [B. mutus]
ENSP00000346065-D1	6.76	1.00E-13	↓	Tudor domain-containing protein 6, partial [B. mutus]
ENSP00000256497-D1	6.76	1.00E-13	↓	ER degradation-enhancing alpha-mannosidase-like 1, partial [B. mutus]
ENSP00000350708-D1	6.78	5.26E-25	↓	UV excision repair protein RAD23-like protein B, partial [B. mutus]
ENSBTAP00000005167-D1	6.80	5.72E-14	↓	Zinc finger protein 316, partial [B. mutus]

ENSBTAP00000039269-D1	6.80	5.72E-14	↓	hypothetical protein M91_09989, partial [B. mutus]
ENSP00000358964-D1	6.80	5.72E-14	↓	uncharacterized protein C1orf194 homolog [B. mutus]
ENSBTAP00000043140-D1	6.80	5.72E-14	↓	Transmembrane protein 64, partial [B. mutus]
ENSP00000344820-D1	6.80	5.72E-14	↓	potassium channel subfamily K member 7 [B. mutus]
ENSP00000264080-D1	6.80	5.72E-14	↓	Protein GPR108, partial [B. mutus]
ENSBTAP00000027863-D2	6.80	5.72E-14	↓	homeobox protein NANOG [B. mutus]
ENSP00000410257-D6	6.80	5.72E-14	↓	sodium channel protein type 10 subunit alpha isoform X2 [B. taurus]
ENSP00000268459-D1	6.80	5.72E-14	↓	protein naked cuticle homolog 1 [B. mutus]
ENSBTAP00000025946-D1	6.80	5.72E-14	↓	Putative deoxyribonuclease TATDN2, partial [B. mutus]
ENSBTAP00000010532-D1	6.80	5.72E-14	↓	Folliculin [B. mutus]
ENSP00000409912-D1	6.80	5.72E-14	↓	hypothetical protein M91_09062, partial [B. mutus]
ENSBTAP00000040894-D1	6.80	5.72E-14	↓	Thiosulfate sulfurtransferase [B. mutus]
ENSBTAP00000014377-D1	6.80	5.72E-14	↓	cysteine-rich with EGF-like domain protein 1 precursor [B. taurus]
ENSP00000353249-D1	6.80	5.72E-14	↓	cytosolic carboxypeptidase-like protein 5 isoform X1 [B. mutus]
ENSP00000234396-D1	6.80	5.72E-14	↓	V-type proton ATPase subunit B, kidney isoform isoform X1 [B. mutus]
ENSBTAP00000023803-D1	6.80	5.72E-14	↓	Conserved oligomeric Golgi complex subunit 3, partial [B. mutus]
ENSBTAP00000011203-D1	6.81	1.02E-70	↓	tastin [B. mutus]
ENSP00000394085-D1	6.81	1.72E-25	↓	bifunctional arginine demethylase and lysyl-hydroxylase JMJD6 [B. mutus]
ENSP00000269385-D1	6.83	1.02E-48	↓	chromobox protein homolog 8 [B. mutus]
ENSBTAP00000018677-D1	6.83	3.26E-14	↓	hypothetical protein M91_07407 [B. mutus]
ENSBTAP00000025607-D1	6.83	3.26E-14	↓	prostaglandin E2 receptor EP3 subtype isoform X1 [B. mutus]
ENSBTAP00000051821-D1	6.83	3.26E-14	↓	hypothetical protein M91_05551, partial [B. mutus]
ENSBTAP00000053776-D1	6.83	3.26E-14	↓	Receptor-type tyrosine-protein phosphatase zeta, partial [B. mutus]
ENSP00000307134-D1	6.83	3.26E-14	↓	Catenin delta-2, partial [B. mutus]
ENSP00000368401-D1	6.83	3.26E-14	↓	Paired box protein Pax-6, partial [B. mutus]
ENSBTAP00000016912-D1	6.83	3.26E-14	↓	HAUS augmin-like complex subunit 4 isoform X1 [B. mutus]
ENSBTAP00000034599-D1	6.83	3.26E-14	↓	thymidine kinase, cytosolic [B. mutus]
ENSP00000391203-D2	6.83	3.26E-14	↓	hypothetical protein M91_04266, partial [B. mutus]
ENSBTAP00000019150-D1	6.83	3.26E-14	↓	GTPase IMAP family member 8, partial [B. mutus]
ENSP00000064778-D1	6.83	3.26E-14	↓	protein FAM168A isoform X2 [Canis lupus familiaris]
ENSP00000368727-D1	6.83	3.26E-14	↓	Xanthine dehydrogenase/oxidase, partial [B. mutus]
ENSBTAP00000028205-D1	6.83	3.26E-14	↓	VPS9 domain-containing protein 1 [B. mutus]
ENSBTAP00000006685-D1	6.83	3.26E-14	↓	Disrupted in renal carcinoma protein 2-like protein [B. mutus]
ENSBTAP00000013074-D1	6.83	3.26E-14	↓	Protein patched-like protein 1, partial [B. mutus]
ENSP00000344361-D18	6.83	3.26E-14	↓	ras-related protein Rap-1b, partial [Ictidomys tridecemlineatus]
yakA11113	6.83	3.26E-14	↓	Histone H4 [Heterocephalus glaber]
ENSP00000269886-D3	6.83	3.26E-14	↓	Endophilin-A3, partial [B. mutus]
ENSBTAP00000017158-D1	6.83	3.26E-14	↓	Cellular retinoic acid-binding protein 1, partial [B. mutus]
ENSBTAP00000014852-D1	6.83	3.26E-14	↓	Tetratricopeptide repeat protein 23, partial [B. mutus]
ENSP00000369895-D1	6.83	3.26E-14	↓	Gem-associated protein 8, partial [B. mutus]
ENSP00000336724-D2	6.84	5.85E-49	↓	TBC1 domain family member 22B [B. taurus]
ENSBTAP00000005252-D29	6.85	5.68E-26	↓	Cell division protein kinase 4, partial [B. mutus]
ENSP00000310796-D1	6.86	3.26E-26	↓	THAP domain-containing protein 2 [Capra hircus]
ENSBTAP00000015626-D1	6.86	1.87E-14	↓	serine/threonine-protein phosphatase 6 regulatory ankyrin repeat subunit C isoform X1 [Bubalus bubalis]
ENSBTAP00000042804-D1	6.86	1.87E-14	↓	Acetylcholine receptor subunit epsilon [B. mutus]
yakA21441	6.86	1.87E-14	↓	Serine incorporator 4 [B. mutus]
ENSP00000385687-D1	6.86	1.87E-14	↓	Serine/threonine-protein kinase 25, partial [B. mutus]
ENSBTAP00000006397-D72	6.86	1.87E-14	↓	zinc finger protein 514 [B. mutus]
ENSP00000329748-D1	6.86	1.87E-14	↓	copine-8 [B. mutus]
ENSBTAP00000020468-D1	6.86	1.87E-14	↓	GREB1-like protein [Tursiops truncatus]
ENSP00000336627-D1	6.86	1.87E-14	↓	SLIT and NTRK-like protein 4-like isoform X1 [B. mutus]
ENSP00000333037-D1	6.86	1.87E-14	↓	actin-related protein T3 [B. mutus]
ENSBTAP00000041851-D1	6.86	1.87E-14	↓	Tetratricopeptide repeat protein 26, partial [B. mutus]
ENSBTAP00000052719-D1	6.86	1.87E-14	↓	uncharacterized protein C12orf56 homolog [B. mutus]
ENSBTAP00000053453-D2	6.86	1.87E-14	↓	Homeobox protein Meis1, partial [B. mutus]

ENSP00000369335-D1	6.86	1.87E-14	↓	F-box only protein 18, partial [B. mutus]
ENSBTAP00000036517-D1	6.86	1.87E-14	↓	ropporin-1-like protein [B. mutus]
ENSP00000272367-D1	6.86	1.87E-14	↓	C-type lectin domain family 4 member F, partial [B. mutus]
ENSBTAP00000039681-D2	6.86	1.87E-14	↓	tetratricopeptide repeat protein 30B [B. mutus]
ENSBTAP00000053313-D1	6.86	1.87E-14	↓	major facilitator superfamily domain-containing protein 8 [B. mutus]
ENSBTAP00000016721-D1	6.86	1.87E-14	↓	ribonucleases P/MRP protein subunit POP1 [B. mutus]
ENSBTAP00000011980-D1	6.86	1.87E-14	↓	dnaJ homolog subfamily C member 25 precursor [B. taurus]
ENSBTAP00000016040-D1	6.86	1.87E-14	↓	Fibulin-1, partial [B. mutus]
ENSBTAP00000042078-D1	6.86	1.87E-14	↓	Thrombospondin-2, partial [B. mutus]
ENSP00000327890-D1	6.86	1.87E-14	↓	Interleukin-3 receptor subunit alpha, partial [B. mutus]
ENSP00000402938-D1	6.86	1.87E-14	↓	2-oxoisovalerate dehydrogenase subunit alpha, mitochondrial [B. mutus]
ENSBTAP00000053216-D1	6.86	1.87E-14	↓	protein kinase C theta type isoform X2 [B. mutus]
ENSBTAP00000001558-D1	6.86	1.87E-14	↓	Plexin-A2, partial [B. mutus]
ENSBTAP00000046068-D1	6.86	1.87E-14	↓	GDP-L-fucose synthase, partial [B. mutus]
ENSBTAP00000050583-D2	6.86	1.87E-14	↓	endogenous retrovirus group K member 11 Pol protein-like [Bison bison bison]
ENSBTAP00000007454-D1	6.88	1.88E-26	↓	sialidase-1 [B. mutus]
ENSP00000418778-D1	6.89	1.08E-26	↓	Schwannomin-interacting protein 1, partial [B. mutus]
ENSP00000347802-D1	6.89	1.08E-14	↓	Ankyrin repeat domain-containing protein 35, partial [B. mutus]
ENSP00000263579-D1	6.89	1.08E-14	↓	m7GpppX diphosphatase [B. mutus]
ENSP00000333664-D1	6.89	1.08E-14	↓	3-ketoacyl-CoA thiolase B, peroxisomal isoform X1 [B. mutus]
ENSP00000310749-D3	6.89	1.08E-14	↓	neurocalcin-delta [B. taurus]
ENSP00000267102-D1	6.89	1.08E-14	↓	protein LMBR1L [B. taurus]
ENSBTAP00000025066-D1	6.89	1.08E-14	↓	CKLF-like MARVEL transmembrane domain-containing protein 2-like [B. mutus]
ENSBTAP00000016536-D1	6.89	1.08E-14	↓	fibrous sheath-interacting protein 2-like isoform X2 [Ovis aries musimon]
ENSP00000245923-D1	6.89	1.08E-14	↓	reticulon-2 isoform X1 [Eptesicus fuscus]
ENSBTAP00000010601-D1	6.89	1.08E-14	↓	muscarinic acetylcholine receptor M3-like [B. mutus]
ENSBTAP00000045709-D1	6.89	1.08E-14	↓	Acrosome formation-associated factor, partial [B. mutus]
ENSP00000255674-D1	6.89	1.08E-14	↓	rotatin [B. mutus]
ENSBTAP00000044044-D1	6.89	1.08E-14	↓	alpha/beta hydrolase domain-containing protein 17C [Ursus maritimus]
ENSP00000363260-D1	6.89	1.08E-14	↓	GPI mannosyltransferase 2 [B. mutus]
ENSBTAP00000025264-D1	6.89	1.08E-14	↓	transmembrane protein 236 [B. mutus]
ENSP00000385006-D1	6.89	1.08E-14	↓	THAP domain-containing protein 4, partial [B. mutus]
ENSP00000251648-D2	6.89	1.08E-14	↓	keratin, type I cuticular Ha4-like [B. mutus]
ENSP00000289407-D1	6.89	1.08E-14	↓	Tetraspanin-33, partial [B. mutus]
ENSP00000307423-D1	6.89	1.08E-14	↓	alpha-1,6-mannosyl-glycoprotein 2-beta-N-acetylglucosaminyltransferase [Bison bison bison]
ENSBTAP00000027917-D1	6.89	1.08E-14	↓	tumor suppressor candidate 2 [B. taurus]
ENSP00000332963-D1	6.90	8.43E-75	↓	Putative folate receptor delta [B. mutus]
ENSBTAP00000019435-D1	6.90	0.00E+00	↓	Beta-sarcoglycan, partial [B. mutus]
ENSBTAP00000026529-D1	6.91	6.22E-27	↓	hypothetical protein M91_09470, partial [B. mutus]
ENSBTAP00000016668-D1	6.92	2.36E-51	↓	Putative RNA-binding protein 15, partial [B. mutus]
ENSP00000337572-D1	6.92	5.61E-149	↓	TPA: diacylglycerol kinase, eta-like [B. taurus]
ENSP00000340466-D1	6.92	6.19E-15	↓	Neutral alpha-glucosidase AB, partial [B. mutus]
ENSBTAP00000052020-D2	6.92	6.19E-15	↓	hypothetical protein M91_16189 [B. mutus]
ENSBTAP00000025796-D1	6.92	6.19E-15	↓	von Willebrand factor A domain-containing protein 3B [B. mutus]
ENSP00000309570-D2	6.92	6.19E-15	↓	Krueppel-like factor 6 isoform X1 [Capra hircus]
ENSBTAP00000010163-D1	6.92	6.19E-15	↓	Pescadillo-like protein, partial [B. mutus]
ENSBTAP00000040669-D1	6.92	6.19E-15	↓	hypothetical protein M91_21619 [B. mutus]
ENSP00000337555-D12	6.92	6.19E-15	↓	zinc finger protein 154-like [B. mutus]
ENSBTAP00000009602-D1	6.92	6.19E-15	↓	testis-expressed sequence 29 protein [B. mutus]
ENSBTAP00000026091-D1	6.92	6.19E-15	↓	Peptidase inhibitor 15, partial [B. mutus]
ENSBTAP00000025374-D1	6.92	6.19E-15	↓	claudin-11 [B. mutus]
ENSP00000380759-D1	6.92	6.19E-15	↓	Protein arginine N-methyltransferase 2, partial [B. mutus]
ENSP00000414147-D1	6.92	6.19E-15	↓	spartin [B. mutus]
ENSBTAP00000012731-D1	6.92	6.19E-15	↓	ribonuclease H2 subunit A [B. taurus]
ENSBTAP00000053745-D1	6.92	6.19E-15	↓	anthrax toxin receptor 1 isoform X1 [Odobenus rosmarus divergens]

ENSP00000396648-D3	6.92	6.19E-15	↓	hypothetical protein M91_14284 [B. mutus]
ENSBTAP00000009718-D1	6.92	6.19E-15	↓	uterine milk protein-like [B. mutus]
ENSP00000363533-D1	6.92	6.19E-15	↓	Putative malate dehydrogenase 1B, partial [B. mutus]
ENSBTAP00000024059-D1	6.92	6.19E-15	↓	phenylalanine--tRNA ligase, mitochondrial [B. mutus]
ENSBTAP00000017295-D1	6.93	7.91E-52	↓	coiled-coil domain-containing protein 126 precursor [B. taurus]
ENSBTAP00000023162-D1	6.95	1.20E-27	↓	Transmembrane protein 214, partial [B. mutus]
ENSBTAP00000013201-D1	6.95	1.20E-27	↓	Tetratricopeptide repeat protein 12, partial [B. mutus]
ENSBTAP00000001628-D1	6.95	3.57E-15	↓	Cadherin-related family member 1, partial [B. mutus]
ENSP00000364126-D1	6.95	3.57E-15	↓	DNA-(apurinic or apyrimidinic site) lyase 2 isoform X1 [B. mutus]
ENSP00000381970-D2	6.95	3.57E-15	↓	gypsy retrotransposon integrase-like protein 1 isoform X1 [B. mutus]
ENSP00000359504-D4	6.95	3.57E-15	↓	interferon-induced guanylate-binding protein 2-like, partial [B. mutus]
ENSP00000311224-D5	6.95	3.57E-15	↓	bile acid-CoA:amino acid N-acyltransferase [Pantholops hodgsonii]
ENSBTAP00000041350-D1	6.95	3.57E-15	↓	CD177 antigen [B. mutus]
ENSBTAP00000041321-D1	6.95	3.57E-15	↓	prostaglandin F2-alpha receptor isoform X1 [B. taurus]
ENSP00000259467-D1	6.95	3.57E-15	↓	Phosducin-like protein [B. mutus]
ENSBTAP00000010916-D2	6.95	3.57E-15	↓	N(6)-adenine-specific DNA methyltransferase 2 [B. mutus]
ENSBTAP00000020188-D1	6.95	3.57E-15	↓	cyclin-dependent kinase 20-like isoform X1 [B. mutus]
ENSP00000408992-D1	6.95	3.57E-15	↓	protein O-linked-mannose beta-1,4-N-acetylglucosaminyltransferase 2-like [B. mutus]
ENSP00000341289-D3	6.95	3.57E-15	↓	tubulin beta-4B chain [Heterocephalus glaber]
ENSP00000370736-D1	6.95	3.57E-15	↓	Transmembrane protein 165, partial [B. mutus]
ENSBTAP00000011960-D1	6.95	3.57E-15	↓	Cystatin-B, partial [B. mutus]
yakG032781	6.95	3.57E-15	↓	hypothetical protein M91_21691 [B. mutus]
ENSP00000266719-D1	6.95	3.57E-15	↓	keratocan [B. mutus]
ENSBTAP00000027051-D1	6.95	3.57E-15	↓	PQ-loop repeat-containing protein 3, partial [B. mutus]
ENSP00000217173-D1	6.95	3.57E-15	↓	RING finger protein 37 isoform X1 [B. mutus]
ENSP00000378191-D1	6.96	0.00E+00	↓	Aminoacyl tRNA synthase complex-interacting multifunctional protein 1, partial [B. mutus]
ENSBTAP00000045968-D9	6.98	2.06E-15	↓	hypothetical protein M91_02087, partial [B. mutus]
ENSP00000246077-D1	6.98	2.06E-15	↓	TBC1 domain family member 20 [Bubalus bubalis]
ENSP00000363349-D1	6.98	2.06E-15	↓	Sorting nexin-30, partial [B. mutus]
ENSP00000284116-D1	6.98	2.06E-15	↓	Glycerophosphodiester phosphodiesterase domain-containing protein 1 [B. mutus]
ENSBTAP00000007469-D1	6.98	2.06E-15	↓	N-arachidonyl glycine receptor [Capra hircus]
ENSBTAP00000017481-D1	6.98	2.06E-15	↓	protein NipSnap homolog 1 isoform X1 [B. mutus]
ENSBTAP00000011612-D1	6.98	2.06E-15	↓	laminin subunit alpha-4 isoform X1 [B. mutus]
ENSBTAP00000022702-D1	6.98	2.06E-15	↓	Zinc finger protein 384 [B. mutus]
ENSBTAP00000008891-D1	6.98	2.06E-15	↓	EH domain-binding protein 1-like protein 1 [B. mutus]
ENSP00000400997-D47	6.98	2.06E-15	↓	zinc finger protein 582 [Bubalus bubalis]
ENSBTAP00000020202-D1	6.98	2.06E-15	↓	PCTP-like protein, partial [B. mutus]
ENSBTAP00000008947-D1	6.98	2.06E-15	↓	zinc finger protein 180 [B. mutus]
ENSBTAP00000028268-D1	6.98	2.06E-15	↓	E3 ubiquitin-protein ligase HECW1, partial [B. mutus]
ENSBTAP00000020429-D1	6.98	2.06E-15	↓	GA-binding protein subunit beta-2 [B. mutus]
ENSP00000215980-D1	6.98	2.06E-15	↓	centromere protein M [B. taurus]
ENSP00000364519-D1	6.98	2.06E-15	↓	Transcription factor Dp-1, partial [B. mutus]
ENSP00000266503-D1	6.98	2.06E-15	↓	hypothetical protein M91_13238 [B. mutus]
ENSBTAP00000041677-D1	7.01	1.35E-28	↓	Chondroitin sulfate synthase 3, partial [B. mutus]
ENSP00000284981-D1	7.01	1.35E-28	↓	Amyloid beta A4 protein, partial [B. mutus]
ENSBTAP00000007216-D1	7.01	1.35E-28	↓	E3 ubiquitin-protein ligase ARIH2 [B. taurus]
ENSP00000412962-D1	7.01	1.35E-28	↓	E3 ubiquitin-protein ligase synoviolin [Tupaia chinensis]
ENSP00000235090-D1	7.01	1.20E-15	↓	methylosome protein 50 isoform X1 [B. mutus]
ENSP00000230771-D1	7.01	1.20E-15	↓	probable histidine--tRNA ligase, mitochondrial precursor [B. taurus]
ENSP00000343706-D1	7.01	1.20E-15	↓	melanoma-associated antigen H1 [B. taurus]
yakA13750	7.01	1.20E-15	↓	hypothetical protein M91_01211 [B. mutus]
ENSBTAP00000006978-D1	7.01	1.20E-15	↓	spermidine synthase [B. taurus]
ENSP00000367564-D1	7.01	1.20E-15	↓	UPF0692 protein C19orf54 homolog [B. taurus]
ENSBTAP00000042277-D1	7.01	1.20E-15	↓	Transmembrane and immunoglobulin domain-containing protein 2 [B. mutus]
ENSP00000311122-D1	7.01	1.20E-15	↓	transmembrane protein 52 [B. mutus]

ENSP00000359827-D1	7.01	1.20E-15	↓	protein FAM73A [Bison bison bison]
ENSBTAP00000039591-D1	7.01	1.20E-15	↓	erlin-2 isoform X2 [B. taurus]
ENSBTAP00000020456-D1	7.01	1.20E-15	↓	Ketosamine-3-kinase, partial [B. mutus]
yakG029112	7.01	1.20E-15	↓	hypothetical protein M91_15025 [B. mutus]
ENSBTAP00000047583-D1	7.01	1.20E-15	↓	complement component C7 [B. mutus]
ENSBTAP00000024222-D1	7.01	1.20E-15	↓	zinc finger and BTB domain-containing protein 8A [B. mutus]
ENSP00000355649-D1	7.01	1.20E-15	↓	S-phase response (cyclin related) [Equus caballus]
ENSP00000298705-D1	7.01	1.20E-15	↓	hypothetical protein M91_07555, partial [B. mutus]
ENSP00000377902-D1	7.01	1.20E-15	↓	Protein FAM19A4, partial [B. mutus]
ENSBTAP00000051299-D2	7.01	1.20E-15	↓	Coatomer subunit zeta-2, partial [B. mutus]
ENSBTAP00000053706-D1	7.04	2.27E-55	↓	beta-1,4-galactosyltransferase 6 [B. taurus]
ENSBTAP00000007607-D1	7.04	4.57E-29	↓	Protein dpy-19-like protein 1, partial [B. mutus]
ENSBTAP00000023206-D1	7.04	4.57E-29	↓	EGF-containing fibulin-like extracellular matrix protein 1 [B. mutus]
ENSP00000353094-D1	7.04	6.95E-16	↓	45 kDa calcium-binding protein, partial [B. mutus]
ENSP00000311121-D1	7.04	6.95E-16	↓	proteasome subunit alpha type-7-like isoform X3 [B. taurus]
ENSP00000283628-D1	7.04	6.95E-16	↓	Upstream-binding protein 1, partial [B. mutus]
ENSBTAP00000041626-D1	7.04	6.95E-16	↓	long-chain fatty acid transport protein 6 [B. mutus]
ENSBTAP00000007391-D1	7.04	6.95E-16	↓	lipopolysaccharide-induced tumor necrosis factor-alpha factor isoform X1 [Bison bison bison]
ENSBTAP00000012400-D1	7.04	6.95E-16	↓	Magnesium transporter NIPA3, partial [B. mutus]
ENSP00000265342-D1	7.04	6.95E-16	↓	Follistatin-related protein 4, partial [B. mutus]
ENSP00000377616-D1	7.04	6.95E-16	↓	keratin-like protein KRT222 [B. taurus]
ENSP00000350230-D1	7.04	6.95E-16	↓	transmembrane protein 170A isoform X1 [Propithecus coquereli]
ENSP00000216780-D1	7.04	6.95E-16	↓	phosphoenolpyruvate carboxykinase [GTP], mitochondrial [B. mutus]
ENSP00000286713-D1	7.04	6.95E-16	↓	Erythrocyte band 7 integral membrane protein, partial [B. mutus]
ENSBTAP00000025168-D1	7.04	6.95E-16	↓	DALR anticodon-binding domain-containing protein 3 [B. mutus]
ENSP00000384428-D1	7.04	6.95E-16	↓	Regulator of G-protein signaling 7 [B. mutus]
ENSBTAP00000002681-D1	7.04	6.95E-16	↓	neprilysin [B. taurus]
ENSBTAP00000002929-D1	7.04	6.95E-16	↓	BTB/POZ domain-containing protein KCTD18 [B. mutus]
ENSBTAP00000003240-D6	7.04	6.95E-16	↓	cadherin-7-like, partial [B. mutus]
ENSBTAP00000021257-D1	7.04	6.95E-16	↓	Seizure protein 6-like protein, partial [B. mutus]
ENSBTAP00000050477-D1	7.04	6.95E-16	↓	ras-related GTP-binding protein B-like [B. mutus]
ENSBTAP00000052380-D1	7.04	6.95E-16	↓	hypothetical protein M91_13899, partial [B. mutus]
ENSP00000361383-D1	7.04	6.95E-16	↓	Allograft inflammatory factor 1-like protein, partial [B. mutus]
ENSBTAP00000043941-D1	7.04	6.95E-16	↓	Activin receptor type-1B, partial [B. mutus]
ENSP00000360730-D1	7.04	6.95E-16	↓	Interleukin-13 receptor subunit alpha-1, partial [B. mutus]
ENSP00000307954-D1	7.04	6.95E-16	↓	membrane protein FAM174A [Cavia porcellus]
ENSBTAP00000041125-D1	7.04	6.95E-16	↓	trefoil factor 2-like [B. mutus]
ENSBTAP00000004650-D1	7.05	1.17E-108	↓	Protein FAM149A, partial [B. mutus]
ENSBTAP00000025394-D1	7.07	1.56E-29	↓	methionine aminopeptidase 1D, mitochondrial isoform X2 [Ovis aries musimon]
ENSBTAP00000005640-D1	7.07	4.05E-16	↓	Very long-chain acyl-CoA synthetase, partial [B. mutus]
ENSP00000386105-D1	7.07	4.05E-16	↓	DNA polymerase delta subunit 2 [B. mutus]
ENSBTAP00000001166-D1	7.07	4.05E-16	↓	centrosomal protein of 131 kDa isoform X1 [Bison bison bison]
ENSP00000260403-D1	7.07	4.05E-16	↓	transmembrane protein 62 [B. mutus]
ENSBTAP00000051734-D1	7.07	4.05E-16	↓	hypothetical protein M91_18687, partial [B. mutus]
ENSP00000356444-D1	7.07	4.05E-16	↓	Sterile alpha motif domain-containing protein 5, partial [B. mutus]
ENSBTAP00000000364-D1	7.07	4.05E-16	↓	Putative 2-oxoglutarate dehydrogenase E1 component DHKTD1, mitochondrial, partial [B. mutus]
ENSP00000262947-D1	7.07	4.05E-16	↓	UPF0556 protein C19orf10 homolog [Bubalus bubalis]
ENSBTAP00000012987-D1	7.07	4.05E-16	↓	placenta-specific gene 8 protein-like [B. mutus]
ENSBTAP00000043499-D1	7.07	4.05E-16	↓	Sperm-associated antigen 1 [B. mutus]
ENSBTAP00000014219-D1	7.07	4.05E-16	↓	Kelch repeat and BTB domain-containing protein 10, partial [B. mutus]
ENSBTAP00000051854-D2	7.07	4.05E-16	↓	Leucine-rich repeat transmembrane protein FLRT3, partial [B. mutus]
ENSBTAP00000029529-D1	7.07	4.05E-16	↓	Two pore calcium channel protein 1, partial [B. mutus]
ENSBTAP00000005745-D1	7.07	4.05E-16	↓	interleukin-23 subunit alpha [B. mutus]
ENSP00000417867-D1	7.07	4.05E-16	↓	hypothetical protein EGM_13600, partial [Macaca fascicularis]
ENSP00000358622-D1	7.07	4.05E-16	↓	NF-kappa-B essential modulator, partial [B. mutus]

ENSP00000324598-D52	7.07	4.05E-16	↓	Zinc finger protein 189, partial [B. mutus]
ENSP00000400664-D1	7.07	4.05E-16	↓	Receptor activity-modifying protein 2, partial [B. mutus]
ENSP00000226382-D1	7.07	4.05E-16	↓	paired mesoderm homeobox protein 2B [Camelus ferus]
ENSBTAP00000036318-D1	7.07	4.05E-16	↓	Importin-4 [B. mutus]
ENSBTAP00000011445-D1	7.07	4.05E-16	↓	toll-like receptor 3 [B. mutus]
ENSP00000345229-D1	7.07	4.05E-16	↓	Delta and Notch-like epidermal growth factor-related receptor, partial [B. mutus]
ENSBTAP00000035273-D1	7.08	1.57E-83	↓	RNA polymerase-associated protein CTR9-like protein [B. mutus]
ENSP00000252744-D1	7.10	2.36E-16	↓	Zinc finger SWIM domain-containing protein 6, partial [B. mutus]
ENSBTAP00000026765-D1	7.10	2.36E-16	↓	epoxide hydrolase 3 [B. taurus]
ENSBTAP00000023823-D2	7.10	2.36E-16	↓	cationic amino acid transporter 3-like [B. mutus]
ENSBTAP00000020766-D1	7.10	2.36E-16	↓	Inositol monophosphatase 3, partial [B. mutus]
ENSBTAP00000028608-D1	7.10	2.36E-16	↓	26S proteasome non-ATPase regulatory subunit 3 [B. taurus]
ENSBTAP00000045377-D2	7.10	2.36E-16	↓	hypothetical protein M91_12935 [B. mutus]
ENSP00000416247-D1	7.10	2.36E-16	↓	Desmocollin-2 [B. mutus]
ENSP00000364805-D5	7.10	2.36E-16	↓	RecName: Full=Heat shock 70 kDa protein 1A; AltName: Full=Heat shock 70 kDa protein 1; Short=HSP70.1
ENSBTAP00000008595-D1	7.10	2.36E-16	↓	Rab GTPase-binding effector protein 2, partial [B. mutus]
ENSP00000322218-D1	7.10	2.36E-16	↓	Membrane-associated phosphatidylinositol transfer protein 2, partial [B. mutus]
ENSBTAP00000028307-D2	7.10	2.36E-16	↓	protein sprouty homolog 2 [B. taurus]
ENSP00000370883-D1	7.10	2.36E-16	↓	nodal modulator 1 [B. taurus]
ENSBTAP00000024352-D1	7.10	2.36E-16	↓	Phosphatidylinositol N-acetylglucosaminyltransferase subunit H [B. mutus]
ENSP00000416142-D2	7.10	2.36E-16	↓	alpha-amylase 2B [B. mutus]
ENSP00000363668-D1	7.10	2.36E-16	↓	Death domain-associated protein 6 [B. mutus]
ENSBTAP00000017657-D1	7.10	2.36E-16	↓	coiled-coil domain-containing protein 22 isoform X1 [B. taurus]
ENSBTAP00000001409-D1	7.10	2.36E-16	↓	Homeobox protein Hox-A4, partial [B. mutus]
ENSBTAP00000009355-D1	7.10	2.36E-16	↓	REST corepressor 3, partial [B. mutus]
ENSBTAP00000009974-D1	7.12	1.38E-16	↓	Adenosine receptor A2b, partial [B. mutus]
ENSBTAP00000019855-D1	7.12	1.38E-16	↓	polyprenol reductase [B. mutus]
ENSP00000395277-D1	7.12	1.38E-16	↓	zinc finger SWIM domain-containing protein 8 isoform X1 [B. mutus]
ENSBTAP00000039583-D1	7.12	1.38E-16	↓	ribosomal RNA-processing protein 8 [B. mutus]
ENSBTAP00000003218-D1	7.12	1.38E-16	↓	7,8-dihydro-8-oxoguanine triphosphatase, partial [B. mutus]
ENSBTAP00000002373-D1	7.12	1.38E-16	↓	Rho guanine nucleotide exchange factor 5, partial [B. mutus]
ENSP00000281156-D2	7.12	1.38E-16	↓	KH domain-containing, RNA-binding, signal transduction-associated protein 2, partial [B. mutus]
ENSBTAP00000007033-D1	7.12	1.38E-16	↓	Regulatory factor X-associated protein, partial [B. mutus]
ENSP00000408464-D1	7.12	1.38E-16	↓	Dedicator of cytokinesis protein 8, partial [B. mutus]
ENSP00000380536-D1	7.12	1.38E-16	↓	uncharacterized protein C14orf93 homolog [B. taurus]
ENSBTAP00000022236-D1	7.12	1.38E-16	↓	Transmembrane protein 42, partial [B. mutus]
ENSBTAP00000003200-D1	7.12	1.38E-16	↓	Protein FAM173A, partial [B. mutus]
ENSP00000313869-D1	7.12	1.38E-16	↓	Golgi reassembly-stacking protein 1, partial [B. mutus]
ENSBTAP00000000005-D2	7.12	1.38E-16	↓	Beta-adrenergic receptor kinase 1, partial [B. mutus]
ENSP00000346635-D1	7.14	1.07E-30	↓	ERO1-like protein beta precursor [B. taurus]
ENSBTAP00000046705-D1	7.14	1.07E-30	↓	aspartate--tRNA ligase, mitochondrial [B. mutus]
ENSP00000263915-D1	7.14	1.07E-30	↓	growth factor receptor-bound protein 14 [B. taurus]
ENSP00000337281-D1	7.14	7.31E-59	↓	Ras guanyl-releasing protein 3, partial [B. mutus]
ENSBTAP00000020989-D79	7.15	8.07E-17	↓	hypothetical protein M91_20985, partial [B. mutus]
ENSP00000349856-D1	7.15	8.07E-17	↓	protein PRRC2B [B. mutus]
ENSP00000354916-D1	7.15	8.07E-17	↓	dual specificity protein phosphatase CDC14A isoform X5 [Bison bison bison]
ENSP00000398721-D1	7.15	8.07E-17	↓	Phosphorylated CTD-interacting factor 1 [B. mutus]
ENSBTAP00000001503-D1	7.15	8.07E-17	↓	thyroid hormone receptor interactor 6 [B. mutus]
ENSBTAP00000048935-D1	7.15	8.07E-17	↓	LYR motif-containing protein 9 [B. taurus]
ENSP00000255465-D1	7.15	8.07E-17	↓	Cyclin-A1, partial [B. mutus]
ENSP00000362650-D1	7.15	8.07E-17	↓	Protein SCAI, partial [B. mutus]
ENSP00000352358-D1	7.15	8.07E-17	↓	transient receptor potential cation channel subfamily V member 6 [B. mutus]
ENSP00000282185-D1	7.15	8.07E-17	↓	Ubiquitin-like-conjugating enzyme ATG10, partial [B. mutus]
ENSP00000295992-D1	7.15	8.07E-17	↓	Procollagen C-endopeptidase enhancer 2 [B. mutus]
ENSBTAP00000026834-D1	7.15	8.07E-17	↓	dipeptidyl peptidase 3 isoform X1 [B. mutus]

ENSBTAP00000007339-D1	7.15	8.07E-17	↓	Helicase SKI2W, partial [B. mutus]
ENSP00000399257-D1	7.15	8.07E-17	↓	PHD finger protein 7 isoform X2 [Bison bison bison]
ENSBTAP00000028108-D1	7.15	8.07E-17	↓	Regulator of telomere elongation helicase 1 [B. mutus]
ENSP00000245615-D1	7.15	8.07E-17	↓	Lysophospholipid acyltransferase 7 [B. mutus]
ENSP00000264316-D1	7.15	8.07E-17	↓	tyrosine-protein kinase TXK [B. mutus]
ENSP00000209884-D1	7.16	2.52E-59	↓	kelch-like protein 20 [Mus musculus]
ENSP00000308901-D1	7.17	2.28E-173	↓	39S ribosomal protein L45, mitochondrial isoform X1 [B. mutus]
ENSBTAP00000009545-D1	7.18	2.16E-31	↓	Dual specificity tyrosine-phosphorylation-regulated kinase 2, partial [B. mutus]
ENSP00000310244-D1	7.18	4.73E-17	↓	RAS guanyl-releasing protein 1, partial [B. mutus]
ENSBTAP00000005097-D95	7.18	4.73E-17	↓	uncharacterized protein LOC103282962 [Anolis carolinensis]
ENSBTAP00000017943-D1	7.18	4.73E-17	↓	5-hydroxytryptamine receptor 2A [B. mutus]
ENSBTAP00000007307-D4	7.18	4.73E-17	↓	chromobox protein homolog 1 [Cavia porcellus]
ENSBTAP00000027693-D1	7.18	4.73E-17	↓	protein strawberry notch homolog 2 [B. mutus]
ENSBTAP00000007176-D1	7.18	4.73E-17	↓	alpha-(1,3)-fucosyltransferase 10 [B. mutus]
ENSP00000297632-D1	7.18	4.73E-17	↓	transmembrane protein 65 [Pantholops hodgsonii]
ENSP00000322191-D1	7.18	4.73E-17	↓	beta-defensin 104A isoform X1 [B. taurus]
ENSBTAP00000053647-D1	7.18	4.73E-17	↓	Iron-sulfur protein NUBPL, partial [B. mutus]
ENSBTAP00000004075-D1	7.18	4.73E-17	↓	IQ domain-containing protein J, partial [B. mutus]
ENSP00000396552-D1	7.18	4.73E-17	↓	Inositol 1,4,5-triphosphate receptor-interacting protein-like 1, partial [B. mutus]
ENSBTAP00000011607-D1	7.19	1.27E-31	↓	WD repeat-containing protein 89 [B. taurus]
ENSP00000355568-D1	7.19	1.27E-31	↓	interferon regulatory factor 2-binding protein 2, partial [B. mutus]
ENSP00000368130-D1	7.20	5.14E-90	↓	aurora kinase A-interacting protein [B. mutus]
ENSBTAP00000015193-D1	7.20	2.78E-17	↓	cyclic AMP-responsive element-binding protein 3 isoform X1 [B. mutus]
ENSP00000392763-D1	7.20	2.78E-17	↓	solute carrier organic anion transporter family member 1B3 [B. mutus]
ENSBTAP00000020161-D1	7.20	2.78E-17	↓	GRB2-related adapter protein 2 [B. taurus]
ENSP00000360170-D1	7.20	2.78E-17	↓	angiopoietin-related protein 3 [B. mutus]
ENSBTAP00000006709-D1	7.20	2.78E-17	↓	Transmembrane protein 2, partial [B. mutus]
ENSBTAP00000012083-D1	7.20	2.78E-17	↓	solute carrier family 15 member 4 [B. taurus]
ENSP00000362937-D1	7.20	2.78E-17	↓	regulator of chromosome condensation isoform X2 [B. taurus]
ENSP00000289865-D1	7.20	2.78E-17	↓	ubiquitin carboxyl-terminal hydrolase 21 isoform X1 [B. mutus]
yakG041951	7.20	2.78E-17	↓	hypothetical protein M91_01555 [B. mutus]
ENSBTAP00000014138-D1	7.20	2.78E-17	↓	Cystatin-A, partial [B. mutus]
ENSBTAP00000022853-D1	7.20	2.78E-17	↓	Fanconi anemia group C protein homolog, partial [B. mutus]
ENSP00000368824-D1	7.20	2.78E-17	↓	connector enhancer of kinase suppressor of ras 2 [B. taurus]
ENSP00000326948-D3	7.20	2.78E-17	↓	putative tRNA (cytidine(32)/guanosine(34)-2'-O)-methyltransferase isoform X1 [B. taurus]
ENSP00000263726-D1	7.20	2.78E-17	↓	LIM/homeobox protein Lhx4 [B. taurus]
ENSBTAP00000006404-D1	7.20	2.78E-17	↓	uncharacterized protein LOC102282341 [B. mutus]
ENSP00000401574-D1	7.20	2.78E-17	↓	hypothetical protein M91_14931 [B. mutus]
ENSBTAP00000004758-D1	7.20	2.78E-17	↓	nuclear receptor subfamily 4 group A member 2 isoform X1 [Condylura cristata]
yakG029385	7.22	4.39E-32	↓	TBC1 domain family member 1 [B. mutus]
ENSP00000334052-D1	7.23	2.59E-32	↓	legumain [B. mutus]
ENSP00000314396-D1	7.23	1.64E-17	↓	putative protein arginine N-methyltransferase 10 isoform X1 [B. mutus]
ENSP00000020945-D1	7.23	1.64E-17	↓	zinc finger protein SNAI2 [B. taurus]
ENSBTAP00000005265-D1	7.23	1.64E-17	↓	DNA polymerase kappa [B. mutus]
ENSP00000318883-D1	7.23	1.64E-17	↓	bcl-2-like protein 13 isoform X1 [B. taurus]
ENSBTAP00000005111-D1	7.23	1.64E-17	↓	Multivesicular body subunit 12A [B. mutus]
ENSP00000320309-D1	7.23	1.64E-17	↓	phenylalanine--tRNA ligase alpha subunit isoform X1 [B. mutus]
yakG038680	7.23	1.64E-17	↓	hypothetical protein M91_14977 [B. mutus]
ENSP00000346627-D1	7.23	1.64E-17	↓	tRNA wybutosine-synthesizing protein 5 [B. mutus]
ENSP00000347170-D1	7.23	1.64E-17	↓	endonuclease 8-like 1 [B. mutus]
ENSBTAP00000035058-D1	7.23	1.64E-17	↓	double C2-like domain-containing protein beta-like [Bubalus bubalis]
ENSP00000386898-D1	7.23	1.64E-17	↓	AN1-type zinc finger protein 2B isoform X1 [B. mutus]
ENSBTAP00000021708-D1	7.23	1.64E-17	↓	Neuferricin [B. mutus]
ENSBTAP00000042467-D5	7.25	9.64E-18	↓	histone H4-like [Alligator sinensis]
ENSP00000349923-D1	7.25	9.64E-18	↓	L antigen family member 3, partial [B. mutus]

ENSBTAP00000024392-D1	7.25	9.64E-18	↓	Cell cycle checkpoint control protein RAD9B [B. mutus]
ENSP00000378298-D1	7.25	9.64E-18	↓	Sodium-dependent serotonin transporter, partial [B. mutus]
ENSBTAP00000010180-D1	7.25	9.64E-18	↓	pseudouridylate synthase 7 homolog [B. mutus]
ENSBTAP00000029508-D1	7.25	9.64E-18	↓	Neurofilament heavy polypeptide, partial [B. mutus]
ENSBTAP00000018730-D1	7.25	9.64E-18	↓	phosphopantothenoylcysteine decarboxylase [B. mutus]
ENSBTAP00000035840-D1	7.25	9.64E-18	↓	snRNA-activating protein complex subunit 2 [B. mutus]
ENSBTAP00000023779-D1	7.25	9.64E-18	↓	zinc finger matrin-type protein 5 [B. taurus]
ENSBTAP00000012963-D1	7.25	9.64E-18	↓	Ankyrin repeat and SAM domain-containing protein 4B, partial [B. mutus]
ENSBTAP00000001560-D1	7.25	9.64E-18	↓	leucine-rich repeat neuronal protein 1 precursor [B. taurus]
ENSBTAP00000017599-D1	7.26	1.91E-123	↓	checkpoint protein HUS1 isoform X1 [B. taurus]
ENSBTAP00000020419-D1	7.28	5.69E-18	↓	protachykinin-1 isoform X1 [B. mutus]
ENSP00000309751-D1	7.28	5.69E-18	↓	monocarboxylate transporter 13 [B. mutus]
ENSBTAP00000021956-D1	7.28	5.69E-18	↓	NADH-cytochrome b5 reductase 3, partial [B. mutus]
ENSP00000384406-D1	7.28	5.69E-18	↓	interferon regulatory factor 1 [B. taurus]
ENSBTAP00000053784-D1	7.28	5.69E-18	↓	Dynein heavy chain 14, axonemal, partial [B. mutus]
ENSP00000319152-D1	7.28	5.69E-18	↓	Equilibrative nucleoside transporter 1, partial [B. mutus]
ENSP00000360891-D1	7.28	5.69E-18	↓	interferon-induced protein with tetratricopeptide repeats 2 [B. mutus]
ENSP00000226760-D1	7.28	5.69E-18	↓	Wolframin [B. mutus]
ENSBTAP00000018777-D3	7.28	5.69E-18	↓	Ras-related protein Rab-5A, partial [B. mutus]
ENSBTAP00000003701-D1	7.28	5.69E-18	↓	Transmembrane protein 63A, partial [B. mutus]
ENSBTAP00000007083-D1	7.28	5.69E-18	↓	Ribonuclease P/MRP protein subunit POP5 [B. mutus]
ENSBTAP00000047808-D1	7.28	5.69E-18	↓	Low-density lipoprotein receptor-related protein 11, partial [B. mutus]
ENSBTAP00000012137-D1	7.29	5.87E-156	↓	tectonic-1 [Bison bison bison]
ENSBTAP00000030898-D3	7.30	3.37E-18	↓	Glycerol kinase, partial [B. mutus]
ENSP00000294390-D1	7.30	3.37E-18	↓	lipid phosphate phosphohydrolase 3 [B. taurus]
ENSBTAP00000051398-D1	7.30	3.37E-18	↓	Fatty-acid amide hydrolase 1, partial [B. mutus]
ENSBTAP00000051420-D1	7.30	3.37E-18	↓	Transmembrane emp24 domain-containing protein 9, partial [B. mutus]
ENSBTAP00000021338-D1	7.30	3.37E-18	↓	exosome complex component RRP46 [B. mutus]
ENSP00000374226-D1	7.30	3.37E-18	↓	Patched domain-containing protein 2 [B. mutus]
ENSP00000217402-D1	7.30	3.37E-18	↓	charged multivesicular body protein 4b [Bubalus bubalis]
ENSBTAP00000026859-D1	7.30	3.37E-18	↓	NFX1-type zinc finger-containing protein 1 [B. mutus]
ENSBTAP00000017880-D1	7.31	8.11E-65	↓	RNA-binding protein 42 isoform X2 [Ovis aries musimon]
ENSBTAP00000008962-D1	7.31	6.54E-34	↓	peroxisomal trans-2-enoyl-CoA reductase [B. taurus]
ENSBTAP00000001344-D1	7.32	2.00E-18	↓	Transmembrane 9 superfamily member 4, partial [B. mutus]
ENSP00000283357-D1	7.32	2.00E-18	↓	protein FAM81B [B. mutus]
ENSBTAP00000051869-D1	7.32	2.00E-18	↓	unnamed protein product [Mus musculus]
ENSP00000349931-D8	7.32	2.00E-18	↓	putative PRAME family member 24 [B. taurus]
ENSP00000264552-D1	7.32	2.00E-18	↓	Ubiquitin-conjugating enzyme E2 S [B. mutus]
ENSBTAP00000005375-D1	7.32	2.00E-18	↓	guanidinoacetate N-methyltransferase [B. taurus]
ENSBTAP00000040952-D1	7.32	2.00E-18	↓	ribosomal RNA processing protein 1 homolog A isoform X1 [B. mutus]
ENSBTAP00000010721-D1	7.32	2.00E-18	↓	SUN domain-containing protein 3 [B. mutus]
ENSP00000265643-D1	7.32	2.00E-18	↓	galanin peptides preproprotein [B. taurus]
ENSBTAP00000042855-D1	7.32	2.00E-18	↓	N-terminal kinase-like protein isoform X1 [B. mutus]
ENSP00000364621-D1	7.32	2.00E-18	↓	Sushi domain-containing protein 3, partial [B. mutus]
ENSBTAP00000002421-D1	7.32	2.00E-18	↓	Docking protein 4 [B. mutus]
ENSBTAP00000023383-D1	7.32	2.00E-18	↓	1-phosphatidylinositol-4,5-bisphosphate phosphodiesterase gamma-1, partial [B. mutus]
ENSBTAP00000007366-D3	7.35	1.19E-18	↓	tripartite motif-containing protein 5 [B. taurus]
ENSP00000222573-D1	7.35	1.19E-18	↓	integrin beta-8 [B. mutus]
ENSBTAP00000006862-D1	7.35	1.19E-18	↓	snRNA-activating protein complex subunit 5 [B. mutus]
ENSP00000359052-D1	7.35	1.19E-18	↓	protein FAM102B isoform X2 [Ovis aries]
ENSBTAP00000001212-D1	7.35	1.19E-18	↓	hypothetical protein M91_03906, partial [B. mutus]
ENSBTAP00000044409-D1	7.35	1.19E-18	↓	nucleolar protein 9 [B. mutus]
ENSBTAP00000001300-D1	7.35	1.19E-18	↓	hypothetical protein M91_16969 [B. mutus]
ENSBTAP00000052463-D1	7.35	1.19E-18	↓	transcription factor 21 [B. taurus]
ENSBTAP00000053454-D1	7.35	1.19E-18	↓	fibroblast growth factor 20 [B. mutus]

ENSBTAP00000043120-D1	7.35	1.19E-18	↓	hypothetical protein M91_21442, partial [B. mutus]
ENSP00000298050-D1	7.35	1.19E-18	↓	Coiled-coil domain-containing protein 67, partial [B. mutus]
ENSP00000223167-D2	7.35	1.19E-18	↓	myosin regulatory light chain 2, skeletal muscle isoform [B. mutus]
ENSBTAP00000046743-D1	7.35	1.19E-18	↓	fibroblast growth factor-binding protein 1 [B. mutus]
ENSP00000366915-D1	7.35	1.19E-18	↓	Progesterone-induced-blocking factor 1 [Pteropus alecto]
ENSBTAP00000042158-D1	7.35	1.19E-18	↓	protein TMEPAI [B. taurus]
ENSP00000311825-D1	7.36	8.15E-35	↓	C-terminal-binding protein 2 isoform X1 [B. taurus]
ENSBTAP00000003764-D1	7.37	1.48E-99	↓	importin subunit alpha-7 isoform X1 [Ovis aries]
ENSP00000296003-D1	7.37	4.86E-35	↓	myotubularin-related protein 14 isoform X1 [B. taurus]
ENSP00000262992-D1	7.37	7.08E-19	↓	Type II inositol-3,4-bisphosphate 4-phosphatase, partial [B. mutus]
ENSP00000349699-D1	7.37	7.08E-19	↓	PWWP domain-containing protein MUM1L1 [Bison bison bison]
ENSBTAP00000025068-D1	7.37	7.08E-19	↓	D-amino-acid oxidase, partial [B. mutus]
ENSBTAP00000048738-D1	7.37	7.08E-19	↓	Phosphatidylcholine transfer protein, partial [B. mutus]
ENSBTAP00000001158-D1	7.37	7.08E-19	↓	Zinc finger protein RFP, partial [B. mutus]
ENSP00000247207-D4	7.37	7.08E-19	↓	Heat shock 70 kDa protein 1B [B. mutus]
ENSP00000327536-D1	7.37	7.08E-19	↓	Roundabout-like protein 2, partial [B. mutus]
ENSBTAP00000028599-D1	7.37	7.08E-19	↓	Transcription factor E2-alpha, partial [B. mutus]
ENSBTAP00000023485-D1	7.37	7.08E-19	↓	Palmdelphin, partial [B. mutus]
ENSP00000389928-D6	7.37	7.08E-19	↓	tripartite motif-containing protein 43-like [B. mutus]
ENSBTAP00000009890-D3	7.39	1.73E-35	↓	Copine-9, partial [B. mutus]
ENSBTAP00000053257-D1	7.39	4.20E-19	↓	alpha-protein kinase 1 [B. mutus]
ENSP00000361681-D1	7.39	4.20E-19	↓	solute carrier family 25 member 53 [B. mutus]
ENSBTAP00000020801-D1	7.39	4.20E-19	↓	interphotoreceptor matrix proteoglycan 2 [B. mutus]
ENSBTAP00000019763-D1	7.39	4.20E-19	↓	metaxin-1 [B. mutus]
ENSBTAP00000006277-D1	7.39	4.20E-19	↓	Dynein heavy chain 2, axonemal, partial [B. mutus]
ENSBTAP00000039131-D1	7.39	4.20E-19	↓	Uridine-cytidine kinase-like 1, partial [B. mutus]
ENSP00000270059-D3	7.39	4.20E-19	↓	Pregnancy-specific beta-1-glycoprotein 6, partial [B. mutus]
ENSP00000375899-D1	7.39	4.20E-19	↓	nuclear body protein SP140 isoform X29 [B. taurus]
ENSP00000231721-D1	7.39	4.20E-19	↓	semaphorin-3G [B. mutus]
ENSP00000250863-D2	7.39	4.20E-19	↓	Deleted in azoospermia-like protein, partial [B. mutus]
ENSBTAP00000048820-D1	7.39	4.20E-19	↓	protocadherin-9 isoform X3 [Ovis aries]
ENSP00000386867-D1	7.39	4.20E-19	↓	PTB domain-containing engulfment adapter protein 1 isoform X1 [B. mutus]
ENSBTAP00000007349-D1	7.39	4.20E-19	↓	Insulin-like growth factor-binding protein 2, partial [B. mutus]
ENSBTAP00000014209-D1	7.39	4.20E-19	↓	Cyclin-dependent kinase 4 inhibitor D, partial [B. mutus]
ENSP00000359454-D1	7.39	4.20E-19	↓	probable ATP-dependent DNA helicase HFM1-like, partial [B. mutus]
ENSP00000414068-D2	7.39	4.20E-19	↓	Pyrroline-5-carboxylate reductase 2, partial [B. mutus]
ENSBTAP00000013255-D1	7.39	4.20E-19	↓	exonuclease 3'-5' domain-containing protein 2 [B. mutus]
ENSP00000390628-D1	7.41	2.51E-19	↓	Tyrosine-protein phosphatase non-receptor type 6, partial [B. mutus]
ENSP00000330302-D1	7.41	2.51E-19	↓	Protocadherin-7, partial [B. mutus]
ENSBTAP00000001006-D1	7.41	2.51E-19	↓	transmembrane protein 206 [B. mutus]
ENSBTAP00000043129-D1	7.41	2.51E-19	↓	kynureninase isoform X1 [Bison bison bison]
ENSBTAP00000042601-D1	7.41	2.51E-19	↓	NKAP-like protein [B. mutus]
ENSP00000403343-D1	7.41	2.51E-19	↓	angio-associated migratory cell protein isoform X1 [B. taurus]
ENSP00000285141-D1	7.41	2.51E-19	↓	Protein FAM38B [B. mutus]
ENSBTAP00000016355-D1	7.41	2.51E-19	↓	protein mab-21-like 1 [Corvus cornix cornix]
ENSBTAP00000002158-D1	7.41	2.51E-19	↓	pleckstrin homology domain-containing family J member 1 [B. mutus]
ENSP00000156084-D1	7.41	2.51E-19	↓	OTU domain-containing protein 5, partial [B. mutus]
ENSBTAP00000044349-D1	7.41	2.51E-19	↓	Leucine-rich repeat-containing protein 58, partial [B. mutus]
ENSBTAP00000042527-D1	7.41	2.51E-19	↓	Inner centromere protein [B. mutus]
ENSP00000338711-D2	7.41	2.51E-19	↓	Tripartite motif-containing protein 5, partial [B. mutus]
ENSBTAP00000046684-D1	7.41	2.51E-19	↓	Ankyrin repeat domain-containing protein 16, partial [B. mutus]
ENSBTAP00000011789-D1	7.41	2.51E-19	↓	proteasome subunit beta type-9 [B. mutus]
ENSP00000398480-D2	7.42	1.55E-69	↓	Mitogen-activated protein kinase 9, partial [B. mutus]
ENSBTAP00000025094-D1	7.42	3.69E-36	↓	Histidyl-tRNA synthetase, cytoplasmic, partial [B. mutus]
ENSBTAP00000007054-D1	7.44	2.21E-36	↓	inactive serine/threonine-protein kinase VRK3 [B. mutus]

ENSBTAP00000046225-D1	7.44	1.50E-19	↓	Proteinase-activated receptor 2, partial [B. mutus]
ENSP00000301608-D1	7.44	1.50E-19	↓	peroxisomal acyl-coenzyme A oxidase 1 isoform X1 [B. mutus]
ENSBTAP00000035807-D1	7.44	1.50E-19	↓	hypothetical protein LOC100423469 [Macaca mulatta]
ENSBTAP00000007468-D1	7.44	1.50E-19	↓	zinc phosphodiesterase ELAC protein 1 [B. taurus]
ENSBTAP00000030774-D1	7.44	1.50E-19	↓	Von Hippel-Lindau disease tumor suppressor, partial [B. mutus]
ENSP00000389039-D2	7.44	1.50E-19	↓	synaptotagmin-16 isoform X3 [B. taurus]
ENSBTAP00000037026-D1	7.44	1.50E-19	↓	protein canopy homolog 4 [B. mutus]
ENSP00000410221-D1	7.44	1.50E-19	↓	Stimulated by retinoic acid 6 protein-like protein, partial [B. mutus]
ENSBTAP00000032877-D1	7.44	1.50E-19	↓	Adenylate cyclase type 3, partial [B. mutus]
ENSP00000384582-D1	7.46	7.94E-37	↓	G-protein coupled receptor 98 [B. mutus]
ENSP00000370488-D1	7.46	8.97E-20	↓	protein lin-52 homolog isoform X1 [Bison bison bison]
ENSBTAP00000053693-D1	7.46	8.97E-20	↓	Microphthalmia-associated transcription factor, partial [B. mutus]
ENSP00000352444-D64	7.46	8.97E-20	↓	hypothetical protein M91_12902, partial [B. mutus]
ENSP00000342267-D2	7.46	8.97E-20	↓	mitochondrial ornithine transporter 1 [B. taurus]
ENSBTAP00000053539-D3	7.46	8.97E-20	↓	Mannosyl-oligosaccharide 1,2-alpha-mannosidase IC, partial [B. mutus]
ENSP00000332164-D2	7.46	8.97E-20	↓	slit homolog 2 protein isoform X3 [B. taurus]
ENSP00000376224-D1	7.46	8.97E-20	↓	transmembrane protein 200A [B. mutus]
ENSP00000258772-D1	7.46	8.97E-20	↓	probable ATP-dependent RNA helicase DDX56 [B. taurus]
ENSBTAP00000001164-D1	7.46	8.97E-20	↓	Endoplasmic reticulum aminopeptidase 2, partial [B. mutus]
ENSP00000403879-D20	7.46	8.97E-20	↓	Zinc finger protein 616, partial [B. mutus]
ENSP00000341247-D2	7.48	2.86E-37	↓	Eukaryotic peptide chain release factor GTP-binding subunit ERF3B, partial [B. mutus]
ENSP00000333181-D1	7.48	5.37E-20	↓	E3 ubiquitin-protein ligase MARCH11, partial [B. mutus]
ENSP00000327154-D1	7.48	5.37E-20	↓	Transmembrane protein 63B [B. mutus]
ENSP00000374152-D1	7.48	5.37E-20	↓	SET and MYND domain-containing protein 5 [B. mutus]
ENSP00000320346-D1	7.48	5.37E-20	↓	RNA polymerase II elongation factor ELL3 [B. mutus]
ENSBTAP00000021091-D1	7.48	5.37E-20	↓	Calpain-14, partial [B. mutus]
ENSP00000368104-D1	7.48	5.37E-20	↓	Bone morphogenetic protein 2 [B. mutus]
ENSP00000338218-D1	7.48	5.37E-20	↓	E3 ubiquitin-protein ligase RNF213 isoform X1, partial [B. taurus]
ENSBTAP00000026574-D1	7.49	2.09E-245	↓	lariat debranching enzyme isoform X1 [B. mutus]
ENSBTAP00000024965-D1	7.50	1.21E-72	↓	5'-AMP-activated protein kinase catalytic subunit alpha-2, partial [B. mutus]
ENSP00000364632-D1	7.50	3.22E-20	↓	BPI fold-containing family B member 4 [Vicugna pacos]
ENSP00000374218-D1	7.50	3.22E-20	↓	extended synaptotagmin-3 [B. mutus]
ENSBTAP00000029451-D1	7.50	3.22E-20	↓	HORMA domain-containing protein 2 [B. mutus]
ENSBTAP00000015740-D1	7.50	3.22E-20	↓	Sodium-coupled neutral amino acid transporter 5, partial [B. mutus]
ENSBTAP00000021025-D1	7.50	3.22E-20	↓	peptidyl-prolyl cis-trans isomerase FKBP11 precursor [B. taurus]
ENSP00000306080-D1	7.50	3.22E-20	↓	RNA methyltransferase-like protein 1, partial [B. mutus]
ENSBTAP00000048405-D60	7.50	3.22E-20	↓	zinc finger protein 790 [Bison bison bison]
ENSBTAP00000015965-D1	7.50	3.22E-20	↓	exocyst complex component 3-like protein [B. mutus]
ENSP00000365019-D4	7.50	3.22E-20	↓	spindlin-1 isoform X1 [Ovis aries]
ENSP00000376700-D1	7.52	1.94E-20	↓	Actin filament-associated protein 1-like 2, partial [B. mutus]
ENSP00000377725-D2	7.52	1.94E-20	↓	Zinc finger protein 192, partial [B. mutus]
ENSBTAP00000010531-D1	7.52	1.94E-20	↓	mitochondrial cardiolipin hydrolase [B. mutus]
ENSBTAP00000000677-D4	7.52	1.94E-20	↓	plasma membrane calcium-transporting ATPase 4 isoform X1 [B. mutus]
ENSBTAP00000019461-D1	7.52	1.94E-20	↓	alpha 2 actin [B. taurus]
ENSP00000263187-D1	7.52	1.94E-20	↓	MutS protein-like protein 4 [B. mutus]
ENSP00000340883-D1	7.52	1.94E-20	↓	SWI/SNF-related matrix-associated actin-dependent regulator of chromatin subfamily B member 1 [B. mutus]
ENSBTAP00000019500-D1	7.52	1.94E-20	↓	tRNA-dihydrouridine(20) synthase [NAD(P)+]-like [B. taurus]
ENSP00000352005-D81	7.52	1.94E-20	↓	zinc finger protein 665-like [Bubalus bubalis]
ENSBTAP00000047795-D1	7.54	1.17E-20	↓	Germ cell-less protein-like 1-like protein, partial [B. mutus]
ENSP00000398321-D1	7.54	1.17E-20	↓	Ankyrin repeat domain-containing protein 39, partial [B. mutus]
ENSP00000266682-D1	7.54	1.17E-20	↓	sodium-dependent neutral amino acid transporter B(0)AT2 [B. taurus]
ENSBTAP00000007090-D1	7.54	1.17E-20	↓	Glia maturation factor gamma, partial [B. mutus]
ENSP00000419057-D1	7.54	1.17E-20	↓	hypothetical protein M91_08248, partial [B. mutus]
ENSBTAP00000013956-D1	7.54	1.17E-20	↓	leucine-rich single-pass membrane protein 1 [B. mutus]
ENSP00000354842-D1	7.54	1.17E-20	↓	Thymocyte selection-associated high mobility group box protein TOX, partial [B. mutus]

ENSP00000380176-D1	7.54	1.17E-20	↓	rho-related GTP-binding protein RhoG [Leptonychotes weddellii]
ENSBTAP00000014056-D1	7.54	1.17E-20	↓	Blood vessel epicardial substance, partial [B. mutus]
ENSP00000383392-D1	7.54	1.17E-20	↓	neural cell adhesion molecule 2-like isoform X1 [B. mutus]
ENSBTAP00000007877-D1	7.55	8.20E-39	↓	Fibronectin type III and SPRY domain-containing protein 1 [B. mutus]
ENSP00000337518-D1	7.56	4.95E-39	↓	pre-rRNA processing protein FTSJ3 [B. mutus]
ENSBTAP00000053852-D1	7.56	7.03E-21	↓	platelet-derived growth factor C isoform X1 [Bubalus bubalis]
ENSBTAP00000025911-D1	7.56	7.03E-21	↓	transmembrane anterior posterior transformation 1 [B. mutus]
ENSBTAP00000035278-D1	7.56	7.03E-21	↓	uncharacterized protein C17orf78 homolog [Bison bison bison]
ENSBTAP00000025736-D1	7.56	7.03E-21	↓	N(2),N(2)-dimethylguanosine tRNA methyltransferase, partial [B. mutus]
ENSP00000278544-D1	7.56	7.03E-21	↓	alkaline ceramidase 3 [B. mutus]
ENSBTAP00000034409-D1	7.56	7.03E-21	↓	Melanoma antigen preferentially expressed in tumors, partial [B. mutus]
ENSBTAP00000037080-D1	7.56	7.03E-21	↓	calcipressin-1 isoform X2 [Orycteropus afer afer]
ENSP00000367263-D1	7.58	1.81E-39	↓	neuroblast differentiation-associated protein AHNAK [B. mutus]
ENSP00000385063-D1	7.58	4.24E-21	↓	protein TBRG4 isoform X1 [B. mutus]
ENSP00000265080-D1	7.58	4.24E-21	↓	ras-specific guanine nucleotide-releasing factor 2 [B. mutus]
ENSBTAP00000026239-D4	7.58	4.24E-21	↓	Diphosphoinositol polyphosphate phosphohydrolase 1, partial [B. mutus]
ENSP00000361628-D1	7.58	4.24E-21	↓	uncharacterized protein CXorf57 homolog [B. mutus]
ENSBTAP00000006746-D1	7.58	4.24E-21	↓	FK506-binding protein 15, partial [B. mutus]
ENSP00000417003-D1	7.58	4.24E-21	↓	Pleckstrin-like protein domain-containing family M member 3 [B. mutus]
ENSP00000367193-D1	7.58	4.24E-21	↓	F-box only protein 48 [B. taurus]
ENSBTAP00000001709-D1	7.58	4.24E-21	↓	AMSH-like protease [B. mutus]
ENSBTAP00000024865-D1	7.58	4.24E-21	↓	brachyury protein isoform X1 [B. taurus]
ENSBTAP00000033443-D1	7.58	4.24E-21	↓	Cytosolic carboxypeptidase 3 [B. mutus]
ENSP00000360519-D1	7.58	4.24E-21	↓	Retinol-binding protein 4, partial [B. mutus]
ENSP00000370724-D1	7.58	4.24E-21	↓	protein FAM49A [Homo sapiens]
ENSP00000266581-D1	7.58	4.24E-21	↓	amphoterin-induced protein 2 isoform X1 [B. mutus]
ENSP00000337224-D1	7.58	4.24E-21	↓	lecithin retinol acyltransferase isoform X1 [B. mutus]
ENSBTAP00000000967-D1	7.60	2.57E-21	↓	Ubiquitin carboxyl-terminal hydrolase 42, partial [B. mutus]
ENSP00000262878-D1	7.60	2.57E-21	↓	SAM domain and HD domain-containing protein 1 [B. mutus]
ENSP00000368797-D1	7.60	2.57E-21	↓	kelch repeat and BTB domain-containing protein 7 [B. taurus]
ENSBTAP00000032546-D1	7.60	2.57E-21	↓	Arrestin domain-containing protein 1, partial [B. mutus]
ENSP00000381377-D1	7.60	2.57E-21	↓	hypothetical protein M91_16065, partial [B. mutus]
ENSBTAP00000021140-D1	7.60	2.57E-21	↓	Zinc finger protein 354C, partial [B. mutus]
ENSBTAP00000041185-D1	7.60	2.57E-21	↓	hypothetical protein M91_04650, partial [B. mutus]
ENSBTAP00000003468-D1	7.62	1.29E-190	↓	mediator of RNA polymerase II transcription subunit 18 [B. taurus]
ENSBTAP00000004324-D22	7.62	1.55E-21	↓	zinc finger protein 551-like [B. mutus]
ENSP00000221852-D1	7.62	1.55E-21	↓	SH2 domain-containing adapter protein D [Pantholops hodgsonii]
ENSBTAP00000051656-D1	7.62	1.55E-21	↓	Hematopoietic SH2 domain-containing protein [B. mutus]
ENSBTAP00000040690-D2	7.62	1.55E-21	↓	RecName: Full=Protein O-linked-mannose beta-1,2-N-acetylglucosaminyltransferase 1; Short=POMGnT1
ENSP00000401574-D2	7.62	1.55E-21	↓	glycerol kinase 2 [B. taurus]
ENSP00000375586-D1	7.62	1.55E-21	↓	Zinc finger protein 304, partial [B. mutus]
ENSP00000327417-D1	7.62	1.55E-21	↓	G-protein coupled receptor 39, partial [B. mutus]
ENSBTAP00000027439-D1	7.62	1.55E-21	↓	BTB/POZ domain-containing protein KCTD20 [B. mutus]
ENSBTAP00000016048-D1	7.62	1.55E-21	↓	symplekin [B. taurus]
ENSP00000237172-D1	7.62	1.55E-21	↓	filamin-A-interacting protein 1 [B. mutus]
ENSBTAP00000011499-D1	7.62	1.55E-21	↓	MKL/myocardin-like protein 2 [B. mutus]
ENSBTAP00000025186-D1	7.64	9.43E-22	↓	liver-expressed antimicrobial peptide 2 [B. mutus]
ENSP00000370424-D1	7.64	9.43E-22	↓	CD81 antigen, partial [B. mutus]
ENSP00000263354-D1	7.64	9.43E-22	↓	alpha-soluble NSF attachment protein [B. taurus]
ENSBTAP00000002566-D1	7.64	9.43E-22	↓	protein YIPF6 [B. taurus]
ENSBTAP00000030514-D1	7.64	9.43E-22	↓	C-type lectin domain family 12 member B [B. mutus]
ENSBTAP00000002350-D1	7.64	9.43E-22	↓	Lon protease-like protein, mitochondrial, partial [B. mutus]
ENSP00000342680-D1	7.64	9.43E-22	↓	uncharacterized protein CXorf67 isoform X2 [B. taurus]
ENSBTAP00000009568-D1	7.64	9.43E-22	↓	triggering receptor expressed on myeloid cells 2 [B. mutus]
ENSBTAP00000049112-D1	7.64	9.43E-22	↓	U8 snoRNA-decapping enzyme [B. mutus]

ENSP00000262041-D1	7.64	9.43E-22	↓	homeobox protein MOX-2-like [Sus scrofa]
ENSBTAP00000010454-D1	7.64	9.43E-22	↓	CDP-diacylglycerol--inositol 3-phosphatidyltransferase [B. taurus]
ENSP00000404296-D1	7.64	9.43E-22	↓	Pleckstrin-like protein domain-containing family A member 5, partial [B. mutus]
ENSBTAP00000020582-D1	7.64	9.43E-22	↓	uncharacterized protein C6orf118 homolog [B. mutus]
ENSP00000260356-D1	7.64	9.43E-22	↓	Thrombospondin-1, partial [B. mutus]
ENSBTAP00000008907-D1	7.66	3.31E-41	↓	D-3-phosphoglycerate dehydrogenase [B. mutus]
ENSP00000350848-D1	7.66	5.73E-22	↓	High mobility group nucleosome-binding domain-containing protein 5, partial [B. mutus]
ENSP00000326305-D1	7.66	5.73E-22	↓	Mitochondrial carnitine/acylcarnitine carrier protein [B. mutus]
ENSP00000381543-D1	7.66	5.73E-22	↓	Male-specific lethal 1-like protein, partial [B. mutus]
ENSP00000378230-D1	7.66	5.73E-22	↓	ATP-binding cassette sub-family C member 11 [B. mutus]
ENSBTAP00000004433-D1	7.66	5.73E-22	↓	phosphorylase b kinase gamma catalytic chain, liver/testis isoform [B. taurus]
ENSP00000299687-D1	7.66	5.73E-22	↓	zinc finger protein 407 [B. mutus]
ENSP00000351933-D1	7.66	5.73E-22	↓	kelch-like protein 9 [Ailuropoda melanoleuca]
ENSBTAP00000023221-D1	7.67	0.00E+00	↓	Nucleobindin-2 [B. mutus]
ENSBTAP00000007628-D1	7.67	3.48E-22	↓	Glutamate receptor 1, partial [B. mutus]
ENSP00000375909-D1	7.67	3.48E-22	↓	A-kinase anchor protein SPHKAP [B. mutus]
ENSBTAP00000002402-D1	7.67	3.48E-22	↓	Leucine-rich repeat-containing protein 36 [B. mutus]
ENSBTAP00000014112-D1	7.67	3.48E-22	↓	gem-associated protein 7 isoform X1 [B. taurus]
ENSBTAP00000048632-D1	7.67	3.48E-22	↓	zinc finger protein 177 isoform X1 [B. taurus]
ENSP00000332601-D1	7.67	3.48E-22	↓	hypothetical protein M91_07424, partial [B. mutus]
ENSBTAP00000032337-D1	7.67	3.48E-22	↓	uncharacterized protein LOC102275278 [B. mutus]
ENSBTAP00000029103-D2	7.69	2.12E-22	↓	Putative 3-hydroxymethyl-3-methylglutaryl-CoA lyase 2, partial [B. mutus]
ENSP00000405400-D1	7.69	2.12E-22	↓	hypothetical protein M91_10116, partial [B. mutus]
ENSBTAP00000027024-D1	7.69	2.12E-22	↓	15-hydroxyprostaglandin dehydrogenase [NAD(+)] isoform X1 [B. mutus]
ENSP00000343657-D1	7.69	2.12E-22	↓	methylcrotonoyl-CoA carboxylase beta chain, mitochondrial [Bison bison bison]
ENSBTAP00000002134-D1	7.69	2.12E-22	↓	Polycomb protein SCMH1 [B. mutus]
ENSBTAP00000001530-D1	7.71	1.29E-22	↓	potassium voltage-gated channel subfamily E member 1 [B. taurus]
ENSP00000332164-D1	7.71	1.29E-22	↓	Slit-like protein 3 protein, partial [B. mutus]
ENSBTAP00000018749-D1	7.71	1.29E-22	↓	hypothetical protein M91_08764, partial [B. mutus]
ENSBTAP00000027371-D1	7.71	1.29E-22	↓	B-cell CLL/lymphoma 7 protein family member B, partial [B. mutus]
ENSBTAP00000018075-D99	7.71	1.29E-22	↓	Zinc finger protein 300, partial [B. mutus]
ENSBTAP00000017147-D1	7.71	1.29E-22	↓	Interferon gamma receptor 2, partial [B. mutus]
ENSBTAP00000053224-D1	7.71	1.29E-22	↓	Voltage-dependent calcium channel subunit alpha-2/delta-2, partial [B. mutus]
ENSBTAP00000003187-D1	7.71	1.29E-22	↓	alpha-1,3/1,6-mannosyltransferase ALG2 [B. mutus]
ENSP00000261170-D1	7.73	7.86E-23	↓	heat-stable enterotoxin receptor [B. mutus]
ENSBTAP00000036892-D1	7.73	7.86E-23	↓	hypothetical protein M91_18898, partial [B. mutus]
ENSP00000361238-D1	7.74	4.80E-23	↓	Radial spoke head protein 9-like protein, partial [B. mutus]
ENSP00000266025-D1	7.74	4.80E-23	↓	Transmembrane protein 115 [B. mutus]
ENSBTAP00000020193-D1	7.74	4.80E-23	↓	hypothetical protein M91_17283, partial [B. mutus]
ENSBTAP00000023149-D1	7.74	4.80E-23	↓	Heat shock factor 2-binding protein, partial [B. mutus]
ENSBTAP00000032888-D1	7.74	4.80E-23	↓	sodium-dependent multivitamin transporter [B. mutus]
ENSP00000401512-D55	7.74	4.80E-23	↓	Zinc finger protein 37A, partial [B. mutus]
ENSP00000358210-D1	7.74	4.80E-23	↓	single-minded homolog 1 [B. mutus]
ENSP00000261377-D1	7.74	4.80E-23	↓	Putative RNA exonuclease NEF-sp, partial [B. mutus]
ENSP00000309934-D1	7.74	4.80E-23	↓	OS-4 protein [Homo sapiens]
ENSBTAP00000016693-D1	7.74	4.80E-23	↓	Macrophage erythroblast attacher, partial [B. mutus]
ENSBTAP00000014826-D1	7.74	4.80E-23	↓	zinc finger protein with KRAB and SCAN domains 2 [B. mutus]
ENSBTAP00000053188-D1	7.76	2.94E-23	↓	adenylate kinase 7 [B. mutus]
ENSP00000251642-D1	7.76	2.94E-23	↓	Putative ATP-dependent RNA helicase DHX58 [B. mutus]
ENSP00000308575-D1	7.76	2.94E-23	↓	protein FAM46D [B. mutus]
ENSP00000282466-D1	7.76	2.94E-23	↓	immunoglobulin superfamily member 10 [B. mutus]
ENSP00000242719-D1	7.78	3.33E-44	↓	RING finger protein 11 [Ceratotherium simum simum]
ENSP00000316861-D1	7.78	1.80E-23	↓	neuronal membrane glycoprotein M6-b isoform X1 [B. taurus]
ENSP00000402050-D8	7.78	1.80E-23	↓	hypothetical protein M91_20981, partial [B. mutus]
ENSBTAP00000021448-D1	7.78	1.80E-23	↓	epididymal sperm-binding protein 1 [B. mutus]

ENSP00000385114-D1	7.78	1.80E-23	↓	TPA: chromosome 17 open reading frame 48 ortholog [B. taurus]
ENSP00000240050-D1	7.78	1.80E-23	↓	mTERF domain-containing protein 3, mitochondrial isoform X1 [B. mutus]
ENSP00000273371-D1	7.78	1.80E-23	↓	phospholipase A1 member A isoform X1 [B. mutus]
ENSBTAP00000051854-D1	7.78	1.80E-23	↓	Leucine-rich repeat transmembrane protein FLRT2, partial [B. mutus]
ENSBTAP00000004079-D1	7.78	1.80E-23	↓	Creatine kinase B-type, partial [B. mutus]
ENSP00000376436-D1	7.78	1.80E-23	↓	Transforming protein p68/c-ets-1 [B. mutus]
ENSP00000301226-D1	7.80	1.10E-23	↓	Trafficking protein particle complex subunit 9, partial [B. mutus]
ENSBTAP00000007584-D1	7.80	1.10E-23	↓	Nephrocystin-3 [B. mutus]
ENSP00000312141-D1	7.80	1.10E-23	↓	Zinc finger protein 654, partial [B. mutus]
ENSP00000378495-D1	7.80	1.10E-23	↓	hypothetical protein M91_19090, partial [B. mutus]
ENSBTAP00000037316-D1	7.80	1.10E-23	↓	protein FAM86A [B. mutus]
ENSBTAP00000017865-D2	7.80	1.10E-23	↓	tetratricopeptide repeat protein 30A [B. mutus]
ENSP00000295049-D1	7.81	6.78E-24	↓	Raftlin-2 [B. mutus]
ENSBTAP00000027669-D1	7.81	6.78E-24	↓	hypothetical protein M91_07076, partial [B. mutus]
ENSP00000357927-D1	7.81	6.78E-24	↓	bcl-2/adenovirus E1B 19 kDa-interacting protein 2-like protein [B. mutus]
ENSP00000283684-D1	7.81	6.78E-24	↓	probable phospholipid-transporting ATPase IC [B. mutus]
ENSBTAP00000028828-D1	7.81	6.78E-24	↓	G/T mismatch-specific thymine DNA glycosylase, partial [B. mutus]
ENSBTAP00000046877-D1	7.81	6.78E-24	↓	Protein FAM149B1 [B. mutus]
ENSBTAP00000015701-D1	7.81	6.78E-24	↓	signal peptide peptidase-like 2A [B. mutus]
ENSBTAP00000018126-D1	7.83	4.17E-24	↓	IQ domain-containing protein H, partial [B. mutus]
ENSBTAP00000045454-D1	7.83	4.17E-24	↓	Ankyrin repeat domain-containing protein 31 [B. mutus]
ENSP00000359730-D3	7.83	4.17E-24	↓	Sodium/hydrogen exchanger 9, partial [B. mutus]
ENSBTAP00000015413-D1	7.83	4.17E-24	↓	mitogen-activated protein kinase kinase kinase 8 isoform X2 [B. taurus]
ENSP00000332592-D1	7.83	4.17E-24	↓	Sperm-associated antigen 16 protein, partial [B. mutus]
ENSBTAP00000014525-D1	7.83	0.00E+00	↓	Apolipoprotein O, partial [B. mutus]
ENSBTAP00000020705-D1	7.84	1.11E-45	↓	polycomb complex protein BMI-1 [B. taurus]
ENSP00000416248-D1	7.85	2.57E-24	↓	Tudor domain-containing protein 12 [B. mutus]
ENSBTAP00000026493-D1	7.85	2.57E-24	↓	Polycystic kidney disease protein 1-like 3, partial [B. mutus]
ENSP00000290573-D1	7.85	2.57E-24	↓	hexokinase-2 isoform X3 [B. taurus]
ENSP00000403095-D3	7.85	2.57E-24	↓	putative upstream-binding factor 1-like protein 1 [Bison bison bison]
ENSBTAP00000008074-D1	7.85	2.57E-24	↓	cytidine deaminase [B. taurus]
ENSP00000369014-D1	7.85	2.57E-24	↓	protein NDNF precursor [B. taurus]
ENSP00000340568-D1	7.85	2.57E-24	↓	Cytochrome c oxidase subunit 8C, mitochondrial, partial [B. mutus]
ENSP00000257336-D1	7.86	1.58E-24	↓	basic immunoglobulin-like variable motif-containing protein-like [B. mutus]
ENSBTAP00000003332-D1	7.86	1.58E-24	↓	KLRAQ motif-containing protein 1, partial [B. mutus]
ENSBTAP00000025591-D13	7.86	1.58E-24	↓	putative RNA polymerase II subunit A C-terminal domain phosphatase SSU72-like protein 1-like [B. mutus]
ENSP00000328672-D1	7.86	1.58E-24	↓	G protein-regulated inducer of neurite outgrowth 3 [B. mutus]
ENSP00000420736-D1	7.86	1.58E-24	↓	forkhead box protein P1-like [Ceratotherium simum simum]
ENSP00000381047-D1	7.88	9.75E-25	↓	hypothetical protein M91_12155 [B. mutus]
ENSBTAP00000053622-D1	7.88	9.75E-25	↓	Echinoderm microtubule-associated protein-like 5, partial [B. mutus]
ENSBTAP00000028278-D2	7.88	9.75E-25	↓	Zinc finger SWIM domain-containing protein 5, partial [B. mutus]
ENSBTAP00000027341-D1	7.88	9.75E-25	↓	T-complex protein 11-like protein, partial [B. mutus]
ENSBTAP00000031823-D1	7.88	9.75E-25	↓	reticulocalbin-3 [B. mutus]
ENSP00000365363-D15	7.88	9.75E-25	↓	TPA: preferentially expressed antigen in melanoma-like [B. taurus]
ENSBTAP00000000695-D1	7.89	6.02E-25	↓	prepronociceptin [B. mutus]
ENSBTAP00000021623-D1	7.89	6.02E-25	↓	Pleckstrin-2, partial [B. mutus]
ENSBTAP00000006571-D1	7.89	6.02E-25	↓	glycoprotein-N-acetylgalactosamine 3-beta-galactosyltransferase 1 [B. taurus]
ENSP00000365118-D3	7.89	6.02E-25	↓	Putative tripartite motif-containing protein 43C, partial [B. mutus]
ENSBTAP00000011959-D1	7.89	6.02E-25	↓	zinc finger protein 572 [B. mutus]
ENSP00000335008-D3	7.89	6.02E-25	↓	spindlin-2 [B. taurus]
ENSP00000370128-D1	7.89	6.02E-25	↓	high affinity cationic amino acid transporter 1 [B. mutus]
ENSP00000384551-D1	7.89	6.02E-25	↓	hypothetical protein PANDA_009209, partial [Ailuropoda melanoleuca]
ENSBTAP00000027394-D1	7.89	6.02E-25	↓	stathmin domain-containing protein 1 [B. mutus]
ENSP00000339659-D1	7.89	6.02E-25	↓	Tripartite motif-containing protein 2, partial [B. mutus]
ENSP00000386884-D1	7.89	6.02E-25	↓	C-X-C chemokine receptor type 4 [B. mutus]

ENSBTAP00000016131-D1	7.89	6.02E-25	↓	Coiled-coil domain-containing protein 158 [B. mutus]
ENSP00000367882-D1	7.89	6.02E-25	↓	lanC-like protein 3 [Canis lupus familiaris]
ENSBTAP00000017780-D2	7.89	6.02E-25	↓	Palmitoyl-protein thioesterase 1, partial [B. mutus]
ENSP00000306817-D1	7.89	6.02E-25	↓	ribokinase-like [B. mutus]
ENSBTAP00000002357-D1	7.89	6.02E-25	↓	Synaptotagmin-4, partial [B. mutus]
ENSBTAP00000047466-D2	7.91	3.73E-25	↓	ARL14 effector protein-like [B. mutus]
ENSP00000415049-D1	7.91	3.73E-25	↓	Gasdermin-B [B. mutus]
ENSBTAP00000002120-D1	7.91	3.73E-25	↓	zinc finger protein 592 [B. mutus]
ENSBTAP00000042923-D1	7.91	3.73E-25	↓	Phosphatidate phosphatase PPAPDC1A, partial [B. mutus]
ENSP00000361708-D1	7.91	3.73E-25	↓	transcription elongation factor A protein-like 1 [Camelus ferus]
ENSP00000356655-D1	7.91	3.73E-25	↓	Epithelial cell-transforming sequence 2 oncogene-like protein [B. mutus]
ENSBTAP00000024845-D1	7.91	3.73E-25	↓	Phospholipase B1, membrane-associated, partial [B. mutus]
ENSBTAP00000048643-D19	7.91	3.73E-25	↓	zinc finger protein 404 [Bison bison bison]
ENSBTAP00000020700-D1	7.91	1.47E-92	↓	Retinoid-binding protein 7, partial [B. mutus]
ENSBTAP00000020870-D1	7.92	2.31E-25	↓	acrosin inhibitor 1 [B. taurus]
ENSBTAP00000052646-D3	7.92	2.31E-25	↓	hypothetical protein M91_08006, partial [B. mutus]
ENSBTAP00000000066-D1	7.92	2.31E-25	↓	PR domain zinc finger protein 14 [B. mutus]
ENSBTAP00000048392-D1	7.92	2.31E-25	↓	Bromodomain-containing protein 8, partial [B. mutus]
ENSBTAP00000017794-D1	7.92	2.31E-25	↓	hypothetical protein M91_03667, partial [B. mutus]
ENSP00000361927-D1	7.92	2.31E-25	↓	heterogeneous nuclear ribonucleoprotein H2 [Ovis aries]
ENSBTAP00000038178-D2	7.92	2.31E-25	↓	Dynein heavy chain 7, axonemal, partial [B. mutus]
ENSBTAP00000023886-D1	7.93	8.41E-139	↓	leucine-rich repeat-containing protein 51-like isoform X1 [B. mutus]
ENSBTAP00000047109-D2	7.94	5.20E-94	↓	Tripartite motif-containing protein 64, partial [B. mutus]
ENSP00000407978-D3	7.94	2.12E-48	↓	Homeobox protein Hox-D4, partial [B. mutus]
ENSP00000328423-D1	7.94	1.43E-25	↓	hypothetical protein M91_07495 [B. mutus]
ENSBTAP00000021466-D1	7.94	1.43E-25	↓	colostrum trypsin inhibitor-like [B. mutus]
ENSBTAP00000039717-D1	7.94	1.43E-25	↓	lysM and putative peptidoglycan-binding domain-containing protein 3 [B. mutus]
ENSBTAP00000015861-D1	7.94	1.43E-25	↓	mRNA turnover protein 4 homolog [B. taurus]
ENSBTAP00000010065-D1	7.94	3.23E-94	↓	putative sodium-coupled neutral amino acid transporter 11 isoform X1 [B. mutus]
ENSP00000350311-D1	7.95	8.19E-49	↓	bcl-2-like protein 11 isoform X1 [Bubalus bubalis]
ENSBTAP00000006854-D1	7.95	8.86E-26	↓	hypothetical protein M91_11452, partial [B. mutus]
ENSBTAP00000017205-D1	7.95	8.86E-26	↓	protein prune homolog 2 [B. mutus]
ENSBTAP00000027892-D1	7.95	8.86E-26	↓	RING finger protein 166, partial [B. mutus]
ENSP00000357057-D1	7.95	8.86E-26	↓	calsequestrin-1 [B. mutus]
ENSP00000295220-D1	7.95	8.86E-26	↓	Primary ciliary dyskinesia protein 1, partial [B. mutus]
ENSBTAP00000002376-D1	7.95	8.86E-26	↓	Transmembrane protein 161A, partial [B. mutus]
ENSP00000412361-D2	7.95	8.86E-26	↓	sialic acid-binding Ig-like lectin 5-like [B. mutus]
ENSBTAP00000007841-D1	7.95	8.86E-26	↓	3-keto-steroid reductase [B. taurus]
ENSBTAP00000041415-D1	7.97	3.16E-49	↓	formin-2 isoform X2 [B. taurus]
ENSP00000355396-D1	7.97	5.50E-26	↓	leucine-rich repeats and immunoglobulin-like domains protein 2 [B. mutus]
ENSP00000344041-D1	7.97	5.50E-26	↓	synaptogyrin-4 [Bison bison bison]
yakG010192	7.97	5.50E-26	↓	hypothetical protein M91_12735 [B. mutus]
ENSP00000398079-D3	7.97	5.50E-26	↓	putative RNA polymerase II subunit A C-terminal domain phosphatase SSU72-like protein 1-like [B. mutus]
ENSBTAP00000030119-D1	7.97	5.50E-26	↓	methyltransferase-like protein 12, mitochondrial [B. taurus]
ENSP00000262482-D1	7.97	5.50E-26	↓	E3 ubiquitin-protein ligase RNF167 isoform X1 [Physeter catodon]
ENSP00000343116-D72	7.98	1.22E-49	↓	Zinc finger protein 91, partial [B. mutus]
ENSBTAP00000043977-D1	7.98	3.42E-26	↓	Bile salt export pump [B. mutus]
ENSBTAP00000000445-D1	7.98	3.42E-26	↓	activity-dependent neuroprotector homeobox protein [B. mutus]
ENSBTAP00000021647-D1	7.99	1.07E-96	↓	Putative imidazolonepropionase, partial [B. mutus]
ENSBTAP00000000821-D1	8.00	2.13E-26	↓	Cation channel sperm-associated protein subunit gamma, partial [B. mutus]
ENSP00000390428-D2	8.00	2.13E-26	↓	Creatine kinase S-type, mitochondrial [B. mutus]
ENSP00000400717-D1	8.00	2.13E-26	↓	Guanine nucleotide-binding protein subunit alpha-13 [B. mutus]
ENSBTAP00000026785-D1	8.00	2.13E-26	↓	LETM1 and EF-hand domain-containing protein 1, mitochondrial, partial [B. mutus]
ENSBTAP00000028704-D1	8.01	1.32E-26	↓	WD repeat-containing protein 11 [B. mutus]
ENSP00000333539-D2	8.01	1.32E-26	↓	Mirror-image polydactyly 1 protein, partial [B. mutus]

ENSBTAP00000008455-D1	8.01	1.32E-26	↓	Acyl-coenzyme A synthetase ACSM3, mitochondrial, partial [B. mutus]
ENSP00000389998-D1	8.03	8.25E-27	↓	meckelin [B. mutus]
ENSP00000361752-D1	8.03	8.25E-27	↓	Putative palmitoyltransferase ZDHHC12 [B. mutus]
ENSP00000367752-D1	8.04	5.15E-27	↓	neurensin-1 isoform X1 [B. mutus]
ENSBTAP00000025636-D1	8.04	5.15E-27	↓	alpha-galactosidase A [B. mutus]
ENSBTAP00000050117-D1	8.04	5.15E-27	↓	hypothetical protein M91_17747 [B. mutus]
ENSP00000160827-D1	8.06	1.37E-100	↓	KIF22 protein [B. taurus]
ENSBTAP00000009821-D1	8.06	3.21E-27	↓	UDP-GalNAc:beta-1,3-N-acetylgalactosaminyltransferase 1 [B. taurus]
ENSP00000388311-D100	8.06	3.21E-27	↓	hypothetical protein M91_09463, partial [B. mutus]
ENSP00000370013-D1	8.06	3.21E-27	↓	Pentatricopeptide repeat-containing protein 2, partial [B. mutus]
ENSBTAP00000035305-D1	8.06	3.21E-27	↓	C-C motif chemokine 3 [Bison bison bison]
ENSP00000396593-D1	8.06	3.21E-27	↓	Dystrotelin [B. mutus]
ENSBTAP00000013035-D1	8.06	3.21E-27	↓	ER lumen protein retaining receptor 3, partial [B. mutus]
ENSBTAP00000027122-D1	8.06	3.21E-27	↓	membrane progestin receptor gamma [Bison bison bison]
ENSP00000181383-D1	8.07	2.01E-27	↓	carboxypeptidase B2 precursor [B. taurus]
ENSBTAP00000051902-D1	8.07	2.01E-27	↓	Ras-related protein Rab-24 [B. mutus]
ENSP00000324857-D1	8.07	2.01E-27	↓	semaphorin-6D isoform X1 [B. mutus]
ENSBTAP00000049212-D4	8.07	2.01E-27	↓	hypothetical protein M91_02395, partial [B. mutus]
ENSP00000418684-D1	8.07	2.01E-27	↓	Zinc finger protein Aiolos, partial [B. mutus]
ENSP00000377055-D1	8.07	2.01E-27	↓	lysosomal Pro-X carboxypeptidase [B. mutus]
ENSBTAP00000012908-D1	8.07	2.01E-27	↓	general transcription factor II-I repeat domain-containing protein 2-like [B. mutus]
ENSBTAP00000019041-D1	8.08	1.26E-27	↓	Acetyl-coenzyme A transporter 1, partial [B. mutus]
ENSP00000350353-D1	8.08	1.26E-27	↓	methyltransferase-like protein 20 [B. mutus]
ENSP00000349052-D1	8.08	1.26E-27	↓	Protein OSCP1 [B. mutus]
yakG042295	8.08	1.26E-27	↓	hypothetical protein M91_19973 [B. mutus]
ENSP00000320754-D1	8.08	1.26E-27	↓	serine/threonine-protein kinase 33 isoform X1 [Bison bison bison]
ENSBTAP00000018766-D1	8.08	1.26E-27	↓	N-acetylglucosamine-1-phosphotransferase subunit gamma, partial [B. mutus]
ENSP00000251363-D1	8.10	7.88E-28	↓	ceramide synthase 4 [B. mutus]
ENSBTAP00000027625-D1	8.10	7.88E-28	↓	transmembrane gamma-carboxyglutamic acid protein 4 [Bison bison bison]
ENSBTAP00000005416-D1	8.10	7.88E-28	↓	transcription regulator protein BACH1 [Bison bison bison]
ENSBTAP00000014256-D1	8.10	7.88E-28	↓	Opa interacting protein 5 [B. mutus]
ENSBTAP00000001830-D1	8.11	4.94E-28	↓	transmembrane emp24 domain-containing protein 9 precursor [B. taurus]
ENSBTAP00000023634-D1	8.11	4.94E-28	↓	serologically defined colon cancer antigen 8 homolog [B. mutus]
ENSBTAP00000012484-D1	8.11	4.94E-28	↓	transmembrane protein 179B [B. mutus]
ENSP00000364493-D1	8.11	4.94E-28	↓	hippocampus abundant transcript-like protein 1-like [B. mutus]
ENSBTAP00000013665-D1	8.11	4.94E-28	↓	interleukin-1 alpha [B. mutus]
ENSBTAP00000045061-D1	8.11	4.94E-28	↓	Gamma-interferon-inducible lysosomal thiol reductase, partial [B. mutus]
ENSP00000206595-D1	8.12	1.65E-53	↓	G2/M phase-specific E3 ubiquitin-protein ligase [B. mutus]
ENSBTAP00000004611-D1	8.12	6.22E-257	↓	TATA box-binding protein-associated factor RNA polymerase I subunit D, partial [B. mutus]
ENSP00000354927-D1	8.12	3.10E-28	↓	mitogen-activated protein kinase kinase kinase 3 isoform X1 [B. taurus]
ENSBTAP00000014941-D1	8.14	4.08E-54	↓	nuclear-interacting partner of ALK [B. mutus]
ENSP00000246912-D1	8.14	1.94E-28	↓	max-like protein X isoform X1 [Bubalus bubalis]
ENSP00000342970-D1	8.14	1.94E-28	↓	E3 ubiquitin-protein ligase TRIM9, partial [B. mutus]
ENSP00000261789-D1	8.14	1.94E-28	↓	transmembrane 9 superfamily member 1 isoform X2 [Ovis aries]
ENSBTAP00000030276-D1	8.14	1.94E-28	↓	sialate O-acetylesterase isoform X2 [B. mutus]
ENSP00000408186-D1	8.14	1.94E-28	↓	testis-expressed sequence 264 protein isoform X1 [B. mutus]
ENSP00000378401-D1	8.15	1.22E-28	↓	testican-1 isoform X1 [Ovis aries]
ENSBTAP00000024453-D7	8.15	1.22E-28	↓	hypothetical protein M91_01964, partial [B. mutus]
ENSBTAP00000017783-D1	8.15	1.22E-28	↓	Collagen alpha-1(XIV) chain, partial [B. mutus]
ENSBTAP00000016739-D1	8.15	1.22E-28	↓	Ganglioside-induced differentiation-associated protein 1, partial [B. mutus]
ENSBTAP00000002955-D1	8.15	1.89E-106	↓	Chromodomain-helicase-DNA-binding protein 9 [B. mutus]
ENSBTAP00000009769-D1	8.16	6.41E-55	↓	trafficking protein particle complex subunit 11 isoform X2 [B. mutus]
ENSP00000367207-D1	8.16	7.69E-29	↓	myc proto-oncogene protein [B. mutus]
ENSBTAP00000006511-D1	8.18	4.84E-29	↓	UPF0686 protein C11orf1 homolog [B. taurus]
ENSBTAP00000015745-D1	8.18	4.84E-29	↓	protocadherin-20-like [B. mutus]

ENSBTAP00000015221-D1	8.18	4.84E-29	↓	DNA polymerase alpha subunit B isoform X1 [B. mutus]
ENSP00000294428-D1	8.18	4.84E-29	↓	Ribonucleoprotein PTB-binding 2, partial [B. mutus]
ENSBTAP00000010113-D1	8.18	4.84E-29	↓	alanine--glyoxylate aminotransferase 2, mitochondrial [B. mutus]
ENSP00000052754-D1	8.18	4.84E-29	↓	decorin precursor [B. taurus]
ENSBTAP00000004969-D1	8.18	4.84E-29	↓	ER degradation-enhancing alpha-mannosidase-like protein 2 isoform X1 [B. mutus]
ENSBTAP00000012489-D1	8.19	3.06E-29	↓	WD repeat-containing protein 74 [B. taurus]
ENSP00000337852-D1	8.19	3.06E-29	↓	zinc finger protein 576 isoform X1 [Bison bison bison]
ENSP00000256257-D1	8.19	3.06E-29	↓	RING finger protein 122, partial [B. mutus]
ENSBTAP00000009882-D1	8.19	3.06E-29	↓	DNA polymerase epsilon catalytic subunit A [B. mutus]
ENSBTAP00000016164-D1	8.19	3.06E-29	↓	Dickkopf-like protein 1, partial [B. mutus]
ENSBTAP00000027058-D1	8.19	3.06E-29	↓	Mitochondrial GTPase 1 [B. mutus]
ENSP00000308938-D1	8.20	1.94E-29	↓	Plasminogen, partial [B. mutus]
ENSBTAP00000011613-D1	8.20	1.94E-29	↓	selenide, water dikinase 1-like isoform X1 [B. mutus]
ENSBTAP00000048474-D1	8.20	1.94E-29	↓	Ras-related protein Rab-4B, partial [B. mutus]
ENSBTAP00000029137-D1	8.20	1.94E-29	↓	anoctamin-3 isoform X1 [B. taurus]
ENSBTAP00000006262-D1	8.22	1.23E-29	↓	endonuclease 8-like 2 [B. mutus]
ENSBTAP00000037616-D1	8.23	6.53E-57	↓	lysophospholipid acyltransferase 5 [B. mutus]
ENSP00000322775-D1	8.23	7.74E-30	↓	Rhomboid family member 2 [B. mutus]
ENSP00000404365-D1	8.23	7.74E-30	↓	uncharacterized protein C4orf21 homolog [B. mutus]
ENSBTAP00000005777-D1	8.23	7.74E-30	↓	CD320 antigen, partial [B. mutus]
ENSP00000398079-D1	8.23	7.74E-30	↓	putative RNA polymerase II subunit A C-terminal domain phosphatase SSU72-like protein 1-like [B. mutus]
ENSP00000262288-D1	8.24	2.62E-57	↓	retinoid-inducible serine carboxypeptidase [B. mutus]
ENSBTAP00000017638-D1	8.24	4.90E-30	↓	DNA-directed RNA polymerase III subunit RPC1 [B. taurus]
ENSBTAP00000050872-D1	8.24	4.90E-30	↓	CHD2 protein [B. taurus]
ENSP00000401362-D1	8.24	4.90E-30	↓	uncharacterized protein C15orf41 homolog isoform X4 [B. taurus]
ENSP00000296486-D1	8.25	3.11E-30	↓	ethanolamine-phosphate phospho-lyase isoform X1 [B. mutus]
ENSP00000370781-D1	8.25	3.11E-30	↓	cell division cycle protein 20 homolog B [B. mutus]
ENSBTAP00000051852-D2	8.25	3.11E-30	↓	homeodomain-interacting protein kinase 1 isoform 1 [Homo sapiens]
ENSP00000341551-D1	8.25	3.11E-30	↓	mothers against decapentaplegic homolog 4 [Ovis aries]
ENSBTAP00000023697-D1	8.25	3.11E-30	↓	WD repeat-containing protein 16 [B. mutus]
ENSP00000300917-D1	8.25	3.11E-30	↓	Protein SMG8 [B. mutus]
ENSP00000403967-D4	8.25	3.11E-30	↓	putative upstream-binding factor 1-like protein 1 [B. taurus]
ENSBTAP00000008487-D1	8.25	3.11E-30	↓	epithelial cell adhesion molecule precursor [B. taurus]
ENSP00000415054-D2	8.26	1.97E-30	↓	Ecto-NOX disulfide-thiol exchanger 2, partial [B. mutus]
ENSBTAP00000010790-D1	8.26	1.97E-30	↓	UPF0586 protein C9orf41 homolog [Bison bison bison]
ENSP00000263245-D1	8.26	1.97E-30	↓	ADP-ribosylation factor GTPase-activating protein 3 isoform X1 [B. mutus]
ENSP00000223095-D1	8.26	1.97E-30	↓	plasminogen activator inhibitor 1 isoform X1 [B. mutus]
ENSP00000355170-D1	8.26	1.97E-30	↓	Copper-transporting ATPase 1 [B. mutus]
ENSBTAP00000003214-D1	8.28	1.25E-30	↓	Putative ribosomal RNA methyltransferase 2, partial [B. mutus]
ENSBTAP00000007911-D1	8.28	1.25E-30	↓	Centrosome-associated protein CEP250 [B. mutus]
ENSBTAP00000018760-D1	8.28	1.25E-30	↓	importin-8 [B. taurus]
ENSP00000056233-D1	8.28	1.25E-30	↓	Nuclear factor erythroid 2-related factor 3, partial [B. mutus]
ENSBTAP00000020482-D1	8.28	1.25E-30	↓	Protein-glutamine gamma-glutamyltransferase Z, partial [B. mutus]
ENSBTAP00000048235-D3	8.29	7.94E-31	↓	hypothetical protein M91_04682, partial [B. mutus]
ENSP00000339057-D1	8.29	7.94E-31	↓	G-protein coupled receptor-associated sorting protein 2-like [B. mutus]
ENSBTAP00000012078-D1	8.29	7.94E-31	↓	transmembrane protein 234 precursor [B. taurus]
ENSBTAP00000006227-D1	8.30	5.05E-31	↓	runt-related transcription factor 2 isoform X3 [Bison bison bison]
ENSBTAP00000006422-D1	8.30	5.05E-31	↓	discoidin domain-containing receptor 2 isoform X1 [B. taurus]
ENSBTAP00000041132-D1	8.30	5.05E-31	↓	flap endonuclease GEN homolog 1 [B. mutus]
ENSBTAP00000017047-D1	8.30	5.05E-31	↓	coiled-coil domain-containing protein 94 [B. mutus]
ENSBTAP00000000795-D1	8.30	5.05E-31	↓	Junctional adhesion molecule B, partial [B. mutus]
ENSBTAP00000031886-D2	8.31	3.21E-31	↓	Putative protein ENSP00000244321-like protein [B. mutus]
ENSBTAP00000022022-D1	8.32	9.27E-117	↓	tRNA 2'-phosphotransferase 1 [B. mutus]
ENSP00000405712-D1	8.32	7.23E-60	↓	Hemogen [B. mutus]
ENSBTAP00000053754-D1	8.32	2.04E-31	↓	WD repeat-containing protein 72, partial [B. mutus]

ENSBTAP00000014068-D1	8.32	2.04E-31	↓	Alkylated DNA repair protein alkB-like protein 8, partial [B. mutus]
ENSBTAP00000023996-D1	8.34	1.30E-31	↓	lanC-like protein 2 [B. mutus]
ENSP00000378897-D1	8.34	1.30E-31	↓	Neutral ceramidase, partial [B. mutus]
ENSBTAP00000049710-D1	8.34	1.30E-31	↓	GATA-type zinc finger protein 1 [B. mutus]
ENSBTAP00000039412-D1	8.35	8.32E-32	↓	Protein-tyrosine phosphatase mitochondrial 1, partial [B. mutus]
ENSBTAP00000012525-D1	8.36	5.30E-32	↓	carbonic anhydrase 6 [B. mutus]
ENSBTAP00000006570-D1	8.36	5.30E-32	↓	Elongator complex protein 1 [B. mutus]
ENSBTAP00000007784-D1	8.36	5.30E-32	↓	Multiple inositol polyphosphate phosphatase 1 [B. mutus]
ENSBTAP00000047294-D1	8.36	5.30E-32	↓	Arsenite methyltransferase, partial [B. mutus]
ENSBTAP00000019318-D29	8.37	3.39E-32	↓	Eukaryotic peptide chain release factor GTP-binding subunit ERF3B, partial [B. mutus]
ENSP00000357259-D1	8.37	3.39E-32	↓	polyamine-modulated factor 1 isoform X1 [Bubalus bubalis]
ENSBTAP00000032144-D1	8.37	3.39E-32	↓	dol-P-Man:Man(5)GlcNAc(2)-PP-Dol alpha-1,3-mannosyltransferase [B. taurus]
ENSP00000308012-D1	8.37	3.39E-32	↓	polyadenylate-binding protein 5 [B. taurus]
ENSP00000259486-D1	8.38	2.17E-32	↓	Ectonucleotide pyrophosphatase/phosphodiesterase family member 2, partial [B. mutus]
ENSP00000350676-D1	8.38	2.17E-32	↓	Histone lysine demethylase PHF8, partial [B. mutus]
ENSP00000350687-D1	8.38	2.17E-32	↓	fukutin [B. mutus]
ENSBTAP00000037736-D1	8.38	2.17E-32	↓	LYR motif-containing protein 5 [B. mutus]
ENSBTAP00000011678-D2	8.38	2.17E-32	↓	calpain-2 catalytic subunit precursor [B. taurus]
ENSP00000298386-D1	8.39	1.39E-32	↓	relaxin receptor 2 isoform X1 [B. mutus]
ENSBTAP00000007953-D1	8.39	1.39E-32	↓	acyl-CoA-binding domain-containing protein 4 [B. mutus]
ENSBTAP00000012447-D1	8.39	1.39E-32	↓	Serine/threonine/tyrosine-interacting-like protein 1 [B. mutus]
ENSBTAP00000040643-D1	8.40	8.87E-33	↓	Dehydrodolichyl diphosphate synthase [B. mutus]
ENSBTAP00000001425-D1	8.41	5.67E-33	↓	SAYSvFN domain-containing protein 1 [B. mutus]
ENSBTAP00000001562-D1	8.41	5.67E-33	↓	SUMO-specific isopeptidase USPL1 [B. mutus]
ENSBTAP00000001744-D1	8.41	5.67E-33	↓	vacuolar protein sorting-associated protein 16 homolog isoform X1 [B. mutus]
ENSP00000278385-D1	8.42	3.85E-124	↓	CD44 antigen [B. mutus]
ENSP00000382349-D1	8.42	3.63E-33	↓	Acyl-coenzyme A synthetase ACSM4, mitochondrial [B. mutus]
ENSP00000317327-D1	8.42	3.63E-33	↓	Ubiquitin-associated and SH3 domain-containing protein A [B. mutus]
ENSP00000229201-D1	8.42	3.63E-33	↓	Protein timeless-like protein, partial [B. mutus]
ENSBTAP00000014845-D1	8.44	6.10E-64	↓	Programmed cell death protein 2 [B. mutus]
ENSP00000410146-D1	8.44	2.33E-33	↓	Glutamate receptor 2, partial [B. mutus]
ENSBTAP00000009186-D1	8.44	2.33E-33	↓	Semaphorin-4F [B. mutus]
ENSBTAP00000016441-D2	8.44	2.33E-33	↓	hypothetical protein M91_17770, partial [B. mutus]
yakG014689	8.44	2.33E-33	↓	hypothetical protein M91_05409 [B. mutus]
ENSP00000358309-D1	8.45	1.50E-33	↓	ephrin type-A receptor 7-like isoform X2 [B. mutus]
ENSBTAP00000026443-D1	8.45	1.50E-33	↓	cytoplasmic polyadenylation element-binding protein 2 isoform X4 [Monodelphis domestica]
ENSBTAP00000023725-D1	8.45	1.50E-33	↓	junctional adhesion molecule A precursor [B. taurus]
ENSBTAP00000003398-D1	8.45	1.50E-33	↓	Proline-serine-threonine phosphatase-interacting protein 2, partial [B. mutus]
ENSP00000288071-D1	8.46	9.63E-34	↓	ATP-dependent RNA helicase DDX19A [B. taurus]
ENSBTAP00000005239-D1	8.46	9.63E-34	↓	fibroblast growth factor 7 precursor [B. taurus]
ENSP00000305785-D1	8.46	9.63E-34	↓	Doublesex- and mab-3-related transcription factor 2, partial [B. mutus]
ENSP00000353098-D1	8.47	6.20E-34	↓	hypothetical protein M91_15051, partial [B. mutus]
ENSBTAP00000007399-D1	8.47	6.20E-34	↓	CAMPATH-1 antigen precursor [B. taurus]
ENSP00000373575-D1	8.47	6.20E-34	↓	uncharacterized protein C6orf163 homolog [B. mutus]
ENSBTAP00000029890-D1	8.47	6.20E-34	↓	toll-like receptor 7 [B. taurus]
ENSP00000328030-D3	8.47	6.20E-34	↓	hypothetical protein M91_02917, partial [B. mutus]
ENSP00000368358-D1	8.47	2.78E-65	↓	DNA polymerase alpha catalytic subunit, partial [B. mutus]
ENSBTAP00000005677-D1	8.48	3.99E-34	↓	nicastrin isoform X1 [B. mutus]
ENSP00000229022-D1	8.48	3.99E-34	↓	vitamin D3 receptor [B. mutus]
ENSBTAP00000015396-D1	8.49	2.57E-34	↓	glioma pathogenesis-related protein 1 [B. mutus]
ENSBTAP00000053813-D1	8.49	2.57E-34	↓	hypothetical protein M91_18338, partial [B. mutus]
ENSP00000350961-D1	8.49	2.57E-34	↓	hypothetical protein M91_02012 [B. mutus]
ENSBTAP00000048704-D2	8.49	2.57E-34	↓	Inhibitor of growth protein 5, partial [B. mutus]
ENSP00000259470-D1	8.50	1.66E-34	↓	cathepsin L1-like [B. mutus]
ENSBTAP00000019639-D1	8.50	1.66E-34	↓	interferon-stimulated gene 20 kDa protein [B. mutus]

ENSP00000265431-D1	8.50	1.66E-34	↓	Calbindin [B. mutus]
ENSP00000374328-D1	8.50	1.66E-34	↓	hypothetical protein M91_01290 [B. mutus]
ENSBTAP00000006568-D1	8.50	2.00E-66	↓	NTF2-related export protein 1 [B. mutus]
ENSP00000361740-D1	8.51	1.07E-34	↓	WW domain-binding protein 5 [Bubalus bubalis]
ENSBTAP00000006002-D1	8.51	1.07E-34	↓	GLIPR1-like protein 1 precursor [B. taurus]
yakG018674	8.52	6.89E-35	↓	hypothetical protein M91_03880 [B. mutus]
ENSBTAP00000035354-D9	8.52	6.89E-35	↓	hypothetical protein M91_14136, partial [B. mutus]
ENSBTAP00000019177-D1	8.53	2.25E-67	↓	Bis(5'-adenosyl)-triphosphatase, partial [B. mutus]
ENSP00000356230-D1	8.53	4.46E-35	↓	Kelch-like protein 12 [B. mutus]
ENSP00000360124-D2	8.53	4.46E-35	↓	phosphoglucomutase-1 isoform X1 [B. taurus]
ENSP00000302811-D1	8.53	4.46E-35	↓	Melatonin receptor type 1A, partial [B. mutus]
ENSBTAP00000027612-D1	8.53	1.46E-67	↓	Multiple myeloma tumor-associated protein 2-like protein [B. mutus]
ENSP00000379773-D1	8.54	2.89E-35	↓	zinc finger CCCH domain-containing protein 7A [B. taurus]
ENSP00000377059-D1	8.54	2.89E-35	↓	sorting nexin-11 [B. taurus]
ENSBTAP00000024487-D1	8.54	2.89E-35	↓	methionine--tRNA ligase, cytoplasmic [B. mutus]
ENSP00000279488-D1	8.54	2.89E-35	↓	dual specificity protein phosphatase 6 [B. taurus]
ENSBTAP00000008135-D1	8.55	1.87E-35	↓	interleukin-1 receptor-associated kinase-like 2 [B. mutus]
ENSBTAP00000021443-D1	8.55	1.87E-35	↓	Dehydrogenase/reductase SDR family member 12, partial [B. mutus]
ENSP00000294409-D1	8.56	1.21E-35	↓	Glucocorticoid modulatory element-binding protein 1 [B. mutus]
ENSBTAP00000006879-D1	8.56	1.21E-35	↓	hypothetical protein M91_13902, partial [B. mutus]
ENSBTAP00000006428-D1	8.57	7.84E-36	↓	sodium/bile acid cotransporter 4 [B. taurus]
ENSBTAP00000010295-D1	8.57	7.84E-36	↓	thyroglobulin [B. mutus]
ENSBTAP00000025792-D1	8.57	7.84E-36	↓	TPA: hypothetical protein LOC539773 [B. taurus]
ENSP00000298717-D1	8.58	5.08E-36	↓	N6-adenosine-methyltransferase 70 kDa subunit [B. mutus]
ENSP00000407860-D1	8.59	3.30E-36	↓	cytochrome P450 20A1-like isoform X1 [B. mutus]
ENSBTAP00000005845-D1	8.59	3.30E-36	↓	transmembrane protein 45A-like [B. mutus]
ENSP00000393316-D1	8.59	1.38E-203	↓	thioredoxin-related transmembrane protein 1 [B. mutus]
ENSBTAP00000004918-D1	8.60	2.14E-36	↓	Zinc finger C4H2 domain-containing protein, partial [B. mutus]
ENSP00000301068-D2	8.60	2.14E-36	↓	GTPase RhebL1 [Capra hircus]
ENSP00000254663-D1	8.61	1.39E-36	↓	Selenocysteine lyase, partial [B. mutus]
ENSP00000385470-D1	8.62	9.05E-37	↓	tumor necrosis factor ligand superfamily member 18 [Bison bison bison]
ENSBTAP00000022716-D1	8.62	9.05E-37	↓	N(4)-(beta-N-acetylglucosaminyl)-L-asparaginase, partial [B. mutus]
ENSP00000366416-D1	8.63	5.90E-37	↓	endothelin B receptor isoform X2 [Ovis aries musimon]
ENSBTAP00000041351-D1	8.63	5.90E-37	↓	hypothetical protein M91_04744, partial [B. mutus]
ENSP00000355511-D1	8.64	3.84E-37	↓	rab proteins geranylgeranyltransferase component A 2 [B. mutus]
ENSP00000288139-D1	8.64	3.84E-37	↓	voltage-dependent L-type calcium channel subunit alpha-1D isoform X1 [B. mutus]
ENSP00000364000-D1	8.64	3.84E-37	↓	collagen alpha-2(V) chain-like, partial [Leptonychotes weddellii]
ENSBTAP00000018634-D1	8.64	3.84E-37	↓	E3 ubiquitin-protein ligase TRIM36 [B. mutus]
ENSP00000411051-D87	8.65	2.50E-37	↓	Zinc finger protein 713, partial [B. mutus]
ENSBTAP00000024611-D1	8.65	2.50E-37	↓	uncharacterized protein C8orf47 homolog [B. mutus]
ENSBTAP00000037698-D1	8.65	2.50E-37	↓	LYR motif-containing protein 2 [B. taurus]
ENSBTAP00000041952-D1	8.65	4.64E-72	↓	synaptonemal complex protein 2-like isoform X1 [B. taurus]
ENSP00000368872-D1	8.66	1.63E-37	↓	protein inscuteable homolog isoform X1 [B. mutus]
ENSBTAP00000012865-D10	8.66	1.63E-37	↓	melanoma antigen preferentially expressed in tumors-like [Bison bison bison]
ENSBTAP00000002753-D1	8.66	1.06E-37	↓	6-phosphofructo-2-kinase/fructose-2,6-bisphosphatase 2 isoform X1 [B. mutus]
ENSP00000368612-D1	8.66	1.06E-37	↓	Putative protein KIAA0564, partial [B. mutus]
ENSBTAP00000028874-D1	8.66	1.06E-37	↓	Methyltransferase-like protein 4 [B. mutus]
ENSP00000335008-D2	8.67	6.94E-38	↓	spindlin-2B-like [B. mutus]
ENSP00000365167-D1	8.67	6.94E-38	↓	forkhead-associated domain-containing protein 1 [B. mutus]
ENSBTAP00000009509-D1	8.67	6.94E-38	↓	Fanconi anemia, complementation group F [B. mutus]
ENSP00000366746-D1	8.68	4.54E-38	↓	signal transducing adapter molecule 1 [B. taurus]
ENSBTAP00000017239-D1	8.68	4.54E-38	↓	secernin-3 [B. mutus]
ENSP00000305334-D1	8.68	4.54E-38	↓	Follistatin-related protein 5, partial [B. mutus]
ENSP00000250457-D1	8.68	4.54E-38	↓	egl nine homolog 3 [B. taurus]
ENSP00000329482-D1	8.69	2.96E-38	↓	serine-rich coiled-coil domain-containing protein 1-like, partial [B. mutus]

ENSP00000309772-D1	8.69	2.96E-38	↓	cadherin-like and PC-esterase domain-containing protein 1 [B. mutus]
ENSBTAP00000007030-D1	8.70	1.94E-38	↓	tryptophan 5-hydroxylase 1 isoform X1 [B. taurus]
ENSBTAP00000046126-D1	8.70	1.94E-38	↓	C1H21ORF7 protein [B. taurus]
ENSP00000404232-D1	8.70	1.94E-38	↓	EF-hand domain-containing family member C2, partial [B. mutus]
ENSBTAP00000007563-D1	8.71	1.27E-38	↓	protein phosphatase 1K, mitochondrial isoform X1 [B. mutus]
ENSP00000415860-D1	8.71	1.27E-38	↓	protocadherin-15 isoform X3 [Capra hircus]
ENSBTAP00000020478-D1	8.72	8.32E-39	↓	thymidylate kinase [B. mutus]
ENSBTAP00000022947-D1	8.72	8.32E-39	↓	Ras-related protein Rab-31, partial [B. mutus]
ENSP00000300035-D1	8.73	5.45E-39	↓	PCNA-associated factor, partial [B. mutus]
ENSP00000263437-D1	8.73	8.26E-148	↓	NACHT, LRR and PYD domains-containing protein 2 [B. mutus]
ENSP00000304268-D91	8.74	3.57E-39	↓	Zinc finger protein 26, partial [B. mutus]
ENSP00000419124-D1	8.74	3.57E-39	↓	Protein FAM107A, partial [B. mutus]
ENSBTAP00000020585-D1	8.74	3.57E-39	↓	zinc finger protein-like 1 [B. taurus]
ENSP00000247970-D1	8.74	2.35E-39	↓	peptidyl-prolyl cis-trans isomerase NIMA-interacting 1 [B. taurus]
ENSP00000364946-D1	8.74	2.35E-39	↓	homeobox protein Mohawk [B. taurus]
ENSBTAP00000037810-D1	8.75	1.54E-39	↓	Intraflagellar transport protein 27-like protein, partial [B. mutus]
ENSP00000417610-D1	8.75	1.54E-39	↓	ras-related protein Rab-23 [B. taurus]
ENSP00000301073-D36	8.75	1.54E-39	↓	Zinc finger protein 64-like protein, isoforms 3 and 4, partial [B. mutus]
ENSP00000394419-D1	8.75	1.54E-39	↓	Apolipoprotein A-I-binding protein, partial [B. mutus]
ENSP00000365433-D1	8.75	1.54E-39	↓	transcription factor 19 [B. mutus]
ENSBTAP00000036700-D1	8.76	1.01E-39	↓	SH3 domain-binding glutamic acid-rich protein, partial [B. mutus]
ENSBTAP00000007067-D1	8.77	6.66E-40	↓	disks large-associated protein 1 isoform X4 [B. taurus]
ENSP00000378056-D2	8.77	6.66E-40	↓	High affinity cAMP-specific and IBMX-insensitive 3',5'-cyclic phosphodiesterase 8B, partial [B. mutus]
ENSBTAP00000017903-D1	8.77	6.66E-40	↓	zinc finger and BTB domain-containing protein 11 [B. mutus]
ENSP00000386516-D1	8.78	4.38E-40	↓	Lysine-specific demethylase 3A [B. mutus]
ENSBTAP00000050866-D1	8.78	4.38E-40	↓	transmembrane protein 185B [B. taurus]
ENSP00000383143-D1	8.78	4.38E-40	↓	SLIT and NTRK-like protein 6 isoform X1 [B. taurus]
ENSP00000286614-D1	8.78	4.38E-40	↓	Matrix metalloproteinase-16, partial [B. mutus]
ENSP00000399851-D1	8.78	4.38E-40	↓	DNA polymerase subunit gamma-1, partial [B. mutus]
ENSP00000368696-D1	8.79	2.88E-40	↓	transmembrane 6 superfamily member 1 isoform X5 [Ovis aries musimon]
ENSBTAP00000044597-D1	8.79	2.88E-40	↓	hypothetical protein M91_01312 [B. mutus]
ENSBTAP00000006215-D1	8.80	1.90E-40	↓	G1/S-specific cyclin-E1 [B. taurus]
ENSP00000376855-D1	8.80	1.90E-40	↓	inactive dipeptidyl peptidase 10 isoform X1 [B. mutus]
ENSBTAP00000007039-D1	8.80	1.25E-40	↓	BAG family molecular chaperone regulator 2, partial [B. mutus]
ENSBTAP00000002898-D1	8.80	1.25E-40	↓	RING finger protein 212, partial [B. mutus]
ENSBTAP00000029749-D1	8.81	8.23E-41	↓	TPA: hypothetical protein BOS_16394 [B. taurus]
ENSBTAP00000039961-D1	8.81	8.23E-41	↓	hypothetical protein M91_08377, partial [B. mutus]
ENSP00000263576-D1	8.82	4.03E-232	↓	ATP-dependent RNA helicase DDX25, partial [B. mutus]
ENSP00000400142-D1	8.82	5.42E-41	↓	Ewing sarcoma breakpoint region 1, isoform CRA_d [Homo sapiens]
ENSP00000392341-D1	8.82	5.42E-41	↓	Endoplasmic reticulum-Golgi intermediate compartment protein 3 [B. mutus]
ENSBTAP00000009011-D1	8.83	3.58E-41	↓	von Willebrand factor A domain-containing protein 2 [B. mutus]
ENSBTAP00000027226-D1	8.83	3.58E-41	↓	Mediator of RNA polymerase II transcription subunit 29, partial [B. mutus]
ENSBTAP00000028178-D1	8.83	3.58E-41	↓	DNA (cytosine-5)-methyltransferase 3A, partial [B. mutus]
ENSP00000356567-D2	8.84	2.37E-41	↓	acyl-CoA-binding domain-containing protein 6 [B. taurus]
ENSBTAP00000023197-D1	8.84	2.37E-41	↓	Disintegrin and metalloproteinase domain-containing protein 9 [B. mutus]
ENSP00000260382-D1	8.85	1.57E-41	↓	leucine-rich repeat-containing protein 49 isoform X2 [B. mutus]
ENSBTAP00000018878-D1	8.85	1.57E-41	↓	CDK5 regulatory subunit-associated protein 3 [B. mutus]
ENSBTAP00000026122-D1	8.87	5.96E-160	↓	intraflagellar transport protein 52 homolog [B. taurus]
ENSBTAP00000038660-D1	8.87	4.53E-42	↓	TPA: hypothetical protein BOS_18285 [B. taurus]
ENSBTAP00000024740-D1	8.88	3.00E-42	↓	Replication factor C subunit 2 [B. mutus]
ENSBTAP00000044412-D1	8.89	1.99E-42	↓	E3 ubiquitin-protein ligase RNF170 isoform X1 [Ovis aries]
ENSP00000415243-D1	8.89	1.99E-42	↓	ski-like protein isoform X1 [B. mutus]
ENSBTAP00000041475-D1	8.89	1.99E-42	↓	non-homologous end-joining factor 1 [B. mutus]
ENSBTAP00000032231-D1	8.89	1.99E-42	↓	transducin-like enhancer protein 4 [B. taurus]
ENSP00000409452-D99	8.89	1.99E-42	↓	Zinc finger protein 1-like protein, partial [B. mutus]

ENSBTAP00000004561-D1	8.90	8.72E-43	↓	hypothetical protein M91_18713 [B. mutus]
ENSP00000228437-D1	8.90	8.72E-43	↓	PR domain zinc finger protein 4, partial [B. mutus]
ENSP00000324882-D1	8.91	5.78E-43	↓	IQ and ubiquitin-like domain-containing protein [B. mutus]
ENSP00000306344-D1	8.92	3.83E-43	↓	transmembrane protein 135, partial [Bison bison bison]
ENSP00000403438-D1	8.92	3.83E-43	↓	transmembrane protein 150C [B. mutus]
ENSBTAP00000035103-D1	8.92	3.83E-43	↓	mitochondrial 2-oxoglutarate/malate carrier protein [B. taurus]
ENSP00000292090-D1	8.92	3.83E-43	↓	calfacilitin [B. taurus]
ENSBTAP00000017667-D1	8.92	3.83E-43	↓	Serine/threonine-protein kinase Sgk3, partial [B. mutus]
ENSBTAP00000005823-D1	8.92	3.83E-43	↓	xaa-Pro aminopeptidase 1 isoform X2 [B. taurus]
ENSBTAP00000045082-D6	8.92	2.55E-43	↓	Ubiquitin-conjugating enzyme E2 D3, partial [B. mutus]
ENSP00000261416-D1	8.93	1.69E-43	↓	Beta-hexosaminidase subunit beta, partial [B. mutus]
ENSBTAP00000053178-D1	8.93	1.69E-43	↓	F-box only protein 11, partial [B. mutus]
ENSBTAP00000027762-D1	8.94	1.13E-43	↓	Chromodomain-helicase-DNA-binding protein 1-like protein [B. mutus]
ENSBTAP00000023493-D1	8.95	4.99E-44	↓	dehydrogenase/reductase SDR family member 4-like isoform X1 [B. mutus]
ENSBTAP00000002051-D1	8.96	3.32E-44	↓	peptidyl-prolyl cis-trans isomerase C precursor [B. taurus]
ENSP00000384330-D1	8.96	3.32E-44	↓	myosin-8 [B. mutus]
ENSBTAP00000018911-D1	8.96	3.32E-44	↓	NEDD4 family-interacting protein 2 [B. mutus]
ENSP00000317684-D1	8.96	3.32E-44	↓	sperm acrosomal protein FSA-ACR.1 isoform X1 [B. mutus]
ENSBTAP00000007170-D3	8.96	3.32E-44	↓	hypothetical protein M91_05930, partial [B. mutus]
ENSP00000406235-D1	8.97	2.22E-44	↓	uncharacterized protein C2orf91 homolog [Balaenoptera acutorostrata scammoni]
ENSP00000357547-D1	8.98	1.48E-44	↓	nuclear pore membrane glycoprotein 210-like isoform X1 [B. mutus]
ENSBTAP00000049813-D1	8.98	0.00E+00	↓	Double homeobox protein A, partial [B. mutus]
ENSP00000315182-D1	8.98	9.85E-45	↓	Protein SHQ1-like protein [B. mutus]
ENSP00000357037-D1	8.98	9.85E-45	↓	Mediator of RNA polymerase II transcription subunit 23 [B. mutus]
ENSBTAP00000026875-D1	8.98	9.85E-45	↓	RecName: Full=UPF0160 protein MYG1, mitochondrial; Flags: Precursor
ENSBTAP00000022457-D1	8.98	9.85E-45	↓	SAGA-associated factor 29-like protein [B. mutus]
ENSBTAP00000009016-D1	8.99	6.58E-45	↓	C-type lectin domain family 2 member H, partial [B. mutus]
ENSP00000314444-D1	9.00	2.21E-87	↓	WD repeat-containing protein 35 [B. mutus]
ENSBTAP00000042336-D1	9.00	4.39E-45	↓	Leucine-rich repeat-containing protein 34, partial [B. mutus]
ENSP00000384653-D1	9.00	4.39E-45	↓	L-seryl-tRNA(Sec) kinase [B. mutus]
ENSBTAP00000040833-D1	9.01	2.94E-45	↓	Nucleoporin NDC1, partial [B. mutus]
ENSBTAP00000044840-D1	9.01	2.94E-45	↓	C-C motif chemokine 1 [B. taurus]
ENSP00000402020-D1	9.01	2.94E-45	↓	UPF0450 protein C17orf58 homolog isoform X1 [Ovis aries]
ENSBTAP00000016441-D1	9.01	1.96E-45	↓	hypothetical protein M91_13898, partial [B. mutus]
ENSP00000401343-D1	9.02	1.31E-45	↓	enhancer of mRNA-decapping protein 3 isoform X1 [B. taurus]
ENSBTAP00000025503-D1	9.02	1.31E-45	↓	Nitrogen permease regulator 2-like protein, partial [B. mutus]
ENSBTAP00000001147-D1	9.02	1.31E-45	↓	sodium-coupled neutral amino acid transporter 1 isoform X1 [B. mutus]
ENSBTAP00000012118-D1	9.03	8.80E-46	↓	ankyrin repeat and SOCS box protein 9 isoform X1 [B. mutus]
ENSBTAP00000036320-D2	9.03	8.80E-46	↓	Mpv17-like protein 2, partial [B. mutus]
ENSBTAP00000045756-D1	9.03	8.80E-46	↓	UPF0602 protein C4orf47 homolog isoform X1 [Bison bison bison]
ENSBTAP00000035023-D42	9.03	8.80E-46	↓	Zinc finger protein 674, partial [B. mutus]
ENSBTAP00000016879-D1	9.03	5.89E-46	↓	exosome complex component CSL4 isoform X1 [Bison bison bison]
ENSP00000282397-D1	9.04	2.63E-89	↓	Vascular endothelial growth factor receptor 1, partial [B. mutus]
ENSP00000267199-D1	9.04	3.94E-46	↓	vacuolar protein sorting-associated protein 33A [B. mutus]
ENSBTAP00000027559-D1	9.04	3.94E-46	↓	Contactin-1, partial [B. mutus]
ENSBTAP00000000595-D1	9.05	2.64E-46	↓	bone sialoprotein 2 [B. mutus]
ENSP00000292853-D2	9.05	2.64E-46	↓	F-box only protein 27, partial [B. mutus]
ENSBTAP00000015643-D1	9.05	2.64E-46	↓	Protein KIAA0664, partial [B. mutus]
ENSP00000363868-D1	9.05	2.64E-46	↓	ATP-binding cassette sub-family A member 1, partial [B. mutus]
ENSBTAP00000021396-D1	9.06	1.60E-90	↓	Zinc finger protein 317 [B. mutus]
ENSP00000372857-D1	9.07	7.94E-47	↓	Serine/threonine-protein kinase Nek11, partial [B. mutus]
ENSBTAP00000042058-D1	9.08	5.32E-47	↓	Sorting nexin-7, partial [B. mutus]
ENSBTAP00000015682-D1	9.08	5.32E-47	↓	Transportin-2, partial [B. mutus]
ENSBTAP00000019858-D1	9.08	5.32E-47	↓	hypothetical protein M91_05072 [B. mutus]
ENSBTAP00000053592-D1	9.08	5.32E-47	↓	frizzled-6 isoform X1 [B. mutus]

ENSBTAP00000044913-D22	9.08	3.58E-47	↓	spermatid nuclear transition protein 3-like [B. mutus]
ENSP00000263405-D1	9.08	3.58E-47	↓	FYN-binding protein, partial [B. mutus]
ENSP00000390625-D1	9.10	1.09E-47	↓	heterogeneous nuclear ribonucleoprotein L-like isoform X1 [B. mutus]
ENSBTAP00000003974-D1	9.10	1.09E-47	↓	proteasomal ubiquitin receptor ADRM1 [Orcinus orca]
ENSBTAP00000008322-D1	9.11	7.30E-48	↓	Interleukin-1 receptor type 2 [B. mutus]
ENSP00000316224-D1	9.12	2.77E-93	↓	calcium-responsive transcription factor isoform X1 [B. mutus]
ENSP00000298288-D1	9.12	4.91E-48	↓	leucine-rich repeat protein 1 isoform X1 [B. mutus]
ENSBTAP00000004817-D1	9.14	1.50E-48	↓	zinc finger protein 3 homolog [B. mutus]
ENSP00000326737-D1	9.14	1.50E-48	↓	tumor necrosis factor receptor superfamily member 12A [Ovis aries]
ENSP00000304553-D1	9.15	6.83E-49	↓	M-phase-specific PLK1-interacting protein [B. mutus]
ENSP00000315775-D1	9.16	3.10E-49	↓	Conserved oligomeric Golgi complex subunit 4 [B. mutus]
ENSBTAP00000042136-D2	9.17	2.10E-49	↓	spindlin-like [B. taurus]
ENSP00000408856-D1	9.19	6.47E-50	↓	A-kinase anchor protein 17B-like [B. mutus]
ENSBTAP00000036478-D1	9.20	4.38E-50	↓	synaptic vesicle glycoprotein 2C [B. taurus]
ENSP00000261234-D1	9.20	4.38E-50	↓	TPA: transmembrane protein 5 [B. taurus]
ENSBTAP00000047663-D2	9.20	2.97E-50	↓	cob(I)yrinic acid a,c-diamide adenosyltransferase, mitochondrial precursor [B. taurus]
yakG010543	9.22	1.36E-50	↓	hypothetical protein M91_03459 [B. mutus]
ENSBTAP00000017476-D1	9.23	6.27E-51	↓	claudin-1 [B. taurus]
ENSP00000417740-D1	9.23	6.27E-51	↓	zinc finger protein 639 isoform X1 [B. mutus]
ENSP00000349768-D1	9.23	6.27E-51	↓	protocadherin-23-like, partial [B. mutus]
ENSP00000399585-D1	9.23	4.26E-51	↓	Magnesium transporter MRS2-like protein, mitochondrial, partial [B. mutus]
ENSBTAP00000048510-D1	9.24	2.89E-51	↓	negative elongation factor C/D-like [B. mutus]
ENSP00000225171-D1	9.26	9.04E-52	↓	dnaJ homolog subfamily C member 12 isoform X1 [B. mutus]
ENSP00000257700-D1	9.26	9.04E-52	↓	RAD50-interacting protein 1, partial [B. mutus]
ENSP00000230792-D1	9.27	4.18E-52	↓	peroxisomal NADH pyrophosphatase NUDT12 isoform X1 [B. taurus]
ENSBTAP00000037099-D1	9.27	4.18E-52	↓	Tumor protein D52 [B. mutus]
ENSP00000380779-D1	9.27	4.18E-52	↓	Tectonic-1, partial [B. mutus]
ENSBTAP00000011257-D1	9.27	4.18E-52	↓	microsomal glutathione S-transferase 1 [B. mutus]
ENSP00000360961-D1	9.28	2.85E-52	↓	Kita-kyushu lung cancer antigen 1-like protein, partial [B. mutus]
ENSBTAP00000027308-D4	9.28	2.85E-52	↓	histone H2B type 1-like [B. mutus]
ENSBTAP00000053629-D1	9.28	1.94E-52	↓	zinc finger protein 42 homolog [Bison bison bison]
ENSP00000362748-D1	9.29	1.32E-52	↓	methylcytosine dioxygenase TET1 [B. mutus]
ENSBTAP00000007876-D1	9.30	6.13E-53	↓	Nocturnin, partial [B. mutus]
ENSBTAP00000003327-D1	9.30	6.13E-53	↓	programmed cell death protein 2-like [B. mutus]
ENSP00000391457-D1	9.30	6.13E-53	↓	INO80 complex subunit C [B. mutus]
ENSP00000346635-D2	9.32	1.95E-53	↓	ERO1-like protein alpha, partial [B. mutus]
ENSBTAP00000008298-D1	9.32	1.95E-53	↓	mitochondrial 10-formyltetrahydrofolate dehydrogenase [B. mutus]
ENSBTAP00000012944-D2	9.32	1.95E-53	↓	hypothetical protein M91_13894, partial [B. mutus]
ENSP00000362125-D1	9.32	1.95E-53	↓	Protocadherin-19, partial [B. mutus]
ENSBTAP00000035234-D1	9.34	6.18E-54	↓	hypothetical protein M91_15734, partial [B. mutus]
ENSBTAP00000000201-D1	9.34	6.18E-54	↓	transcription factor Sp3, partial [B. taurus]
ENSP00000387264-D1	9.35	2.88E-54	↓	LIM and senescent cell antigen-like-containing domain protein 1 isoform X4 [B. taurus]
ENSBTAP00000047624-D1	9.35	1.97E-54	↓	marginal zone B- and B1-cell-specific protein [B. mutus]
ENSP00000257724-D1	9.36	1.35E-54	↓	myoD family inhibitor domain-containing protein [B. mutus]
ENSBTAP00000005817-D1	9.36	1.35E-54	↓	epidermal growth factor-like protein 8 precursor [B. taurus]
ENSP00000369513-D1	9.37	6.30E-55	↓	V-type proton ATPase subunit S1-like protein, partial [B. mutus]
ENSP00000386141-D1	9.37	6.30E-55	↓	XK-related protein 9 [B. mutus]
ENSP00000372527-D1	9.38	4.32E-55	↓	Transmembrane 4 L6 family member 1 [B. mutus]
ENSBTAP00000021249-D1	9.38	4.32E-55	↓	glutaredoxin-2, mitochondrial [B. mutus]
ENSP00000370443-D1	9.38	2.96E-55	↓	A disintegrin and metalloproteinase with thrombospondin motifs 6 precursor [B. taurus]
ENSP00000322170-D1	9.38	2.96E-55	↓	profilin-4 [B. taurus]
ENSP00000376161-D1	9.39	2.03E-55	↓	ORM1-like protein 1 [B. taurus]
ENSBTAP00000001479-D1	9.39	1.39E-55	↓	Prolyl 4-hydroxylase subunit alpha-2, partial [B. mutus]
ENSBTAP00000000230-D1	9.40	9.54E-56	↓	RNA (guanine-9-)-methyltransferase domain-containing protein 2, partial [B. mutus]
ENSBTAP00000048938-D1	9.40	6.54E-56	↓	protein FAM47E [B. mutus]

ENSBTAP00000050877-D1	9.41	4.49E-56	↓	pyridine nucleotide-disulfide oxidoreductase domain-containing protein 1 [B. mutus]
ENSP00000412383-D54	9.41	4.49E-56	↓	hypothetical protein M91_07168, partial [B. mutus]
ENSBTAP00000017380-D1	9.41	4.49E-56	↓	ELL-associated factor 2 [B. taurus]
ENSBTAP00000012845-D1	9.41	3.08E-56	↓	p53 and DNA damage-regulated protein 1 [B. taurus]
ENSP00000228641-D1	9.43	1.00E-56	↓	myogenic factor 6 [B. taurus]
ENSBTAP00000001962-D1	9.43	1.00E-56	↓	Fidgetin-like protein 1, partial [B. mutus]
ENSP00000312981-D1	9.43	1.00E-56	↓	U4/U6.U5 tri-snRNP-associated protein 2 [Oryctolagus cuniculus]
ENSBTAP00000008492-D1	9.44	6.90E-57	↓	UPF0553 protein C9orf64 homolog [B. mutus]
ENSBTAP00000037960-D1	9.45	3.26E-57	↓	Chain A, Crystallographic Refinement Of Bovine Pro-Phospholipase A2 At 1.6 Angstroms Resolution
ENSP00000368164-D1	9.45	3.26E-57	↓	DNA replication licensing factor MCM8 [B. mutus]
ENSBTAP00000004233-D1	9.47	5.06E-58	↓	protein pelota homolog [B. taurus]
ENSBTAP00000009687-D1	9.48	3.48E-58	↓	aminoacyl tRNA synthase complex-interacting multifunctional protein 2 [B. mutus]
ENSP00000243346-D1	9.48	3.48E-58	↓	N-myc-interactor [B. mutus]
ENSBTAP00000013685-D1	9.48	2.41E-58	↓	Protein-tyrosine sulfotransferase 2, partial [B. mutus]
ENSP00000330031-D1	9.49	1.66E-58	↓	Piwi-like protein 3, partial [B. mutus]
ENSP00000269829-D55	9.50	7.04E-115	↓	Zinc finger protein 789, partial [B. mutus]
ENSBTAP00000053646-D1	9.50	7.89E-59	↓	zinc finger E-box-binding homeobox 2-like isoform X2 [B. mutus]
ENSP00000343155-D2	9.50	7.89E-59	↓	X-box-binding protein 1, partial [B. mutus]
ENSP00000386957-D1	9.50	7.89E-59	↓	Putative protein KIAA1107 [B. mutus]
ENSBTAP00000042390-D1	9.51	3.76E-59	↓	protein kintoun isoform X1 [B. mutus]
ENSBTAP00000005171-D1	9.51	3.76E-59	↓	lipoyltransferase 1, mitochondrial isoform X1 [B. mutus]
ENSBTAP00000053247-D1	9.51	2.60E-59	↓	Methionine-R-sulfoxide reductase B3, mitochondrial, partial [B. mutus]
ENSBTAP00000053663-D1	9.51	2.60E-59	↓	acetyl-CoA carboxylase 1 isoform X2 [B. taurus]
ENSBTAP00000024453-D12	9.51	2.60E-59	↓	Prostaglandin F synthase 1, partial [B. mutus]
ENSP00000334105-D1	9.52	1.80E-59	↓	1-phosphatidylinositol-4,5-bisphosphate phosphodiesterase beta-4, partial [B. mutus]
ENSBTAP00000006679-D1	9.52	1.24E-59	↓	HSPB1-associated protein 1 [B. mutus]
ENSBTAP00000036194-D2	9.52	1.24E-59	↓	Krueppel-like factor 17, partial [B. mutus]
ENSBTAP00000002311-D1	9.53	5.96E-60	↓	Nuclear receptor 2C2-associated protein [B. mutus]
ENSBTAP00000017963-D1	9.54	2.78E-117	↓	Torsin-2A, partial [B. mutus]
ENSBTAP00000020046-D1	9.54	2.85E-60	↓	Potassium channel subfamily K member 18, partial [B. mutus]
ENSBTAP00000016202-D1	9.55	1.98E-60	↓	Mixed lineage kinase domain-like protein, partial [B. mutus]
ENSBTAP00000005903-D1	9.55	1.98E-60	↓	pro-neuropeptide Y precursor [B. taurus]
ENSP00000378238-D1	9.55	1.98E-60	↓	protein EVI2A [B. mutus]
ENSBTAP00000001053-D3	9.55	1.37E-60	↓	folate receptor alpha precursor [B. taurus]
ENSP00000358374-D1	9.56	6.57E-61	↓	protein SCAF11 [B. mutus]
ENSBTAP00000042937-D2	9.57	3.16E-61	↓	rhombotin-1 [B. taurus]
ENSBTAP00000013299-D1	9.57	3.16E-61	↓	Fanconi anemia group D2 protein isoform X1 [Bison bison bison]
ENSBTAP00000033621-D1	9.58	1.52E-61	↓	endonuclease/exonuclease/phosphatase family domain-containing protein 1 [B. mutus]
ENSBTAP00000009822-D1	9.60	5.11E-62	↓	E3 ubiquitin-protein ligase MIB1 [B. taurus]
ENSBTAP00000029203-D1	9.60	5.11E-62	↓	succinate-semialdehyde dehydrogenase, mitochondrial [B. taurus]
ENSBTAP00000051799-D1	9.60	5.11E-62	↓	uncharacterized protein LOC613444 [B. taurus]
ENSP00000249356-D1	9.60	3.56E-62	↓	dnaJ homolog subfamily B member 9 precursor [B. taurus]
ENSP00000303788-D1	9.63	4.03E-63	↓	CDKN2A-interacting protein isoform X1 [Bison bison bison]
ENSBTAP00000038078-D1	9.68	7.64E-65	↓	epidermal growth factor [B. taurus]
ENSBTAP00000029091-D1	9.68	7.64E-65	↓	protein ARV1 [B. mutus]
ENSP00000230914-D1	9.69	3.73E-65	↓	28S ribosomal protein S30, mitochondrial [B. mutus]
ENSBTAP00000017941-D1	9.69	3.73E-65	↓	carboxypeptidase M [B. mutus]
ENSBTAP00000004892-D1	9.71	1.28E-65	↓	Transketolase, partial [B. mutus]
ENSP00000256319-D2	9.71	8.94E-66	↓	probable ergosterol biosynthetic protein 28-like [B. mutus]
ENSP00000340995-D1	9.71	8.94E-66	↓	Testis-expressed sequence 11 protein, partial [B. mutus]
ENSBTAP00000001343-D1	9.71	6.26E-66	↓	zinc transporter 5 [B. mutus]
ENSBTAP00000011963-D1	9.72	4.39E-66	↓	U1 small nuclear ribonucleoprotein A, partial [B. mutus]
ENSP00000343274-D1	9.72	3.07E-66	↓	Integrator complex subunit 8, partial [B. mutus]
ENSBTAP00000043394-D1	9.72	3.07E-66	↓	FAST kinase domain-containing protein 3 [B. mutus]
ENSBTAP00000048358-D1	9.73	2.15E-66	↓	dystrophin isoform X5 [B. taurus]

ENSBTAP00000008580-D1	9.73	2.15E-66	↓	NSFL1 cofactor p47 [B. taurus]
ENSBTAP00000012108-D1	9.74	7.40E-67	↓	Trifunctional purine biosynthetic protein adenosine-3, partial [B. mutus]
ENSBTAP00000008957-D1	9.74	5.18E-67	↓	Abhydrolase domain-containing protein FAM108B1 [Pteropus alecto]
ENSP00000365141-D9	9.76	1.26E-67	↓	Putative tripartite motif-containing protein 64B, partial [B. mutus]
ENSBTAP00000042698-D5	9.76	1.26E-67	↓	putative RNA polymerase II subunit A C-terminal domain phosphatase SSU72-like protein 1 [Bison bison bison]
ENSBTAP00000001867-D1	9.78	2.15E-68	↓	DNA mismatch repair protein Msh6 [B. mutus]
ENSBTAP00000040146-D1	9.80	7.48E-69	↓	anaphase-promoting complex subunit 1 isoform X1 [Bison bison bison]
ENSBTAP00000029136-D1	9.80	3.71E-69	↓	R3H and coiled-coil domain-containing protein 1-like isoform X1 [B. mutus]
ENSP00000359262-D1	9.81	1.84E-69	↓	39S ribosomal protein L43, mitochondrial, partial [B. mutus]
ENSBTAP00000043366-D1	9.82	6.46E-70	↓	Src kinase-associated phosphoprotein 2 [B. mutus]
ENSP00000311486-D1	9.84	2.27E-70	↓	UPF0536 protein C12orf66 homolog [B. mutus]
ENSBTAP00000031831-D1	9.84	1.60E-70	↓	cytochrome c 2 [B. taurus]
ENSBTAP00000024802-D1	9.85	5.64E-71	↓	Coiled-coil and C2 domain-containing protein 2A, partial [B. mutus]
ENSBTAP00000000013-D1	9.88	7.03E-72	↓	tetratricopeptide repeat protein 33 [B. taurus]
ENSP00000385642-D1	9.90	1.25E-72	↓	Aryl hydrocarbon receptor [B. mutus]
ENSBTAP00000014847-D1	9.92	2.23E-73	↓	actin-related protein 8 [B. taurus]
ENSP00000366901-D1	9.92	1.58E-73	↓	hypothetical protein M91_09758, partial [B. mutus]
ENSBTAP00000050906-D5	9.93	7.94E-74	↓	PRAME family member 9/15, partial [B. mutus]
ENSP00000319210-D1	9.93	7.94E-74	↓	rab effector Noc2 [Bison bison bison]
ENSP00000348064-D1	9.93	5.64E-74	↓	Ecto-ADP-ribosyltransferase 3, partial [B. mutus]
ENSP00000365788-D1	9.94	2.02E-74	↓	Sperm-associated antigen 6 [B. mutus]
ENSP00000393527-D1	9.99	2.41E-76	↓	NADPH--cytochrome P450 reductase [B. mutus]
ENSBTAP00000001821-D7	9.99	4.56E-150	↓	hypothetical protein M91_12973, partial [B. mutus]
ENSP00000261839-D4	10.00	1.22E-76	↓	Myosin-Vc, partial [B. mutus]
ENSBTAP00000052981-D1	10.01	4.43E-77	↓	dynein regulatory complex protein 1 [B. taurus]
ENSBTAP00000011539-D1	10.04	9.79E-154	↓	kinesin-like protein KIF20A [B. mutus]
ENSBTAP00000032595-D1	10.06	5.58E-79	↓	hypothetical protein M91_03891 [B. mutus]
ENSBTAP00000024824-D1	10.06	4.00E-79	↓	probable cation-transporting ATPase 13A5 [B. mutus]
ENSP00000357076-D4	10.06	4.00E-79	↓	Transgelin, partial [B. mutus]
ENSP00000313752-D1	10.08	5.39E-80	↓	Sjoegren syndrome nuclear autoantigen 1 homolog, partial [Equus caballus]
ENSP00000399571-D1	10.10	1.02E-80	↓	Sclerostin domain-containing protein 1 [B. mutus]
ENSBTAP00000024658-D1	10.10	1.02E-80	↓	tripartite motif-containing protein 38 [B. mutus]
ENSP00000347919-D1	10.11	3.78E-81	↓	coiled-coil-helix-coiled-coil-helix domain-containing protein 8 [B. taurus]
ENSBTAP00000012238-D1	10.14	9.94E-83	↓	Membrane-spanning 4-domains subfamily A member 8A, partial [B. mutus]
ENSBTAP00000051999-D1	10.18	1.92E-84	↓	ankyrin repeat domain-containing protein 26-like isoform X2 [B. taurus]
ENSBTAP00000051959-D1	10.23	2.00E-86	↓	hypothetical protein M91_16889 [B. mutus]
ENSP00000286371-D6	10.23	1.45E-86	↓	Sodium/potassium-transporting ATPase subunit beta-3, partial [B. mutus]
ENSBTAP00000044076-D1	10.25	2.86E-87	↓	telomeric repeat-binding factor 1 isoform X1 [B. mutus]
ENSP00000385165-D1	10.27	2.16E-88	↓	NAD kinase 2, mitochondrial isoform X2 [Bubalus bubalis]
ENSP00000336817-D2	10.27	1.57E-88	↓	Leucine zipper protein 2, partial [B. mutus]
ENSBTAP00000030565-D1	10.33	4.90E-91	↓	zinc finger protein 596-like isoform X1 [Bubalus bubalis]
ENSBTAP00000010428-D1	10.33	2.59E-91	↓	probable sodium-coupled neutral amino acid transporter 6 isoform X1 [B. taurus]
ENSBTAP00000018534-D1	10.34	1.88E-91	↓	Aprataxin, partial [B. mutus]
yakG038882	10.34	1.88E-91	↓	Protein FAM190A [B. mutus]
ENSBTAP00000053625-D1	10.36	4.32E-182	↓	Fanconi anemia group M protein [B. mutus]
ENSP00000368884-D1	10.36	2.04E-92	↓	Ribosomal protein S6 kinase alpha-3, partial [B. mutus]
ENSBTAP00000038164-D1	10.39	4.63E-94	↓	Voltage-dependent calcium channel gamma-like subunit [B. mutus]
ENSBTAP00000027650-D1	10.39	4.63E-94	↓	protein FAM76B [Chlorocebus sabaeus]
ENSBTAP00000027635-D1	10.39	2.47E-94	↓	NTF2-related export protein 2 [B. mutus]
ENSBTAP00000015934-D1	10.39	2.47E-94	↓	Homeobox protein CDX-1, partial [B. mutus]
ENSBTAP00000050537-D2	10.40	1.80E-94	↓	zinc finger and SCAN domain-containing protein 4 [Bison bison bison]
ENSP00000309181-D1	10.41	7.04E-95	↓	G1/S-specific cyclin-E2 [B. taurus]
ENSP00000374323-D12	10.42	1.46E-95	↓	Ephrin type-A receptor 6, partial [B. mutus]
ENSP00000372221-D1	10.44	1.20E-96	↓	Serpin B5 [B. mutus]
ENSBTAP00000017417-D1	10.45	2.53E-97	↓	sperm-associated antigen 5 [B. mutus]

ENSBTAP00000026578-D1	10.46	1.36E-97	↓	armadillo repeat-containing protein 8 [B. taurus]
ENSP00000403095-D1	10.46	9.95E-98	↓	putative upstream-binding factor 1-like protein 1-like [B. mutus]
ENSBTAP00000041352-D1	10.47	7.30E-98	↓	thioredoxin-related transmembrane protein 2 [Ovis aries]
ENSP00000370557-D1	10.47	5.35E-98	↓	protein MIS12 homolog [B. taurus]
ENSP00000239891-D1	10.47	2.87E-98	↓	dolichyl-phosphate beta-glucosyltransferase isoform 1 [B. taurus]
ENSBTAP00000014774-D1	10.49	3.28E-99	↓	heterogeneous nuclear ribonucleoproteins A2/B1-like isoform X2 [B. taurus]
ENSBTAP00000044179-D1	10.49	2.41E-99	↓	Transient receptor putative cation channel subfamily M member 6, partial [B. mutus]
ENSBTAP00000006703-D2	10.51	1.96E-197	↓	regulation of nuclear pre-mRNA domain-containing protein 1A [B. mutus]
ENSP00000364429-D1	10.52	8.10E-101	↓	glutamate-rich protein 2 [Propithecus coquereli]
ENSBTAP00000003296-D1	10.52	8.10E-101	↓	TATA-box-binding protein, partial [B. mutus]
ENSBTAP00000023885-D5	10.58	5.32E-104	↓	hypothetical protein M91_21298, partial [B. mutus]
ENSP00000354846-D1	10.58	3.93E-104	↓	LRP2-binding protein [B. mutus]
ENSP00000327104-D1	10.59	1.17E-104	↓	phospholipase DDHD1 [B. mutus]
ENSBTAP00000048590-D2	10.59	1.17E-104	↓	transcription elongation factor B polypeptide 2-like [B. mutus]
ENSBTAP00000042576-D1	10.60	6.38E-105	↓	protein SSX2-like, partial [Bison bison bison]
ENSP00000395465-D1	10.63	1.71E-106	↓	Nuclear receptor coactivator 4, partial [B. mutus]
ENSP00000384477-D1	10.67	7.74E-109	↓	interferon-related developmental regulator 1 isoform X1 [B. mutus]
ENSP00000224784-D2	10.69	3.92E-110	↓	hypothetical protein CB1_000252031 [Camelus ferus]
ENSP00000393599-D1	10.70	1.60E-110	↓	Metastasis-associated lung adenocarcinoma transcript 1, partial [B. mutus]
ENSP00000340083-D3	10.70	8.88E-111	↓	hypothetical protein M91_04855 [B. mutus]
ENSBTAP00000047788-D1	10.71	4.92E-111	↓	amidophosphoribosyltransferase isoform X1 [B. mutus]
ENSBTAP00000015870-D1	10.71	4.92E-111	↓	probable G-protein coupled receptor 19 [B. mutus]
ENSBTAP00000024903-D1	10.83	2.06E-118	↓	Cation channel sperm-associated protein subunit beta, partial [B. mutus]
ENSBTAP00000049986-D1	10.84	8.65E-119	↓	hypothetical protein M91_19384, partial [B. mutus]
ENSP00000354649-D1	10.87	8.58E-121	↓	magnesium transporter protein 1 [Camelus ferus]
ENSBTAP00000044811-D1	10.93	1.62E-124	↓	DNA-directed RNA polymerase I subunit RPA43 [B. mutus]
ENSP00000388373-D1	10.95	4.09E-126	↓	Liprin-alpha-2, partial [B. mutus]
ENSP00000360363-D1	10.98	7.90E-128	↓	C1GALT1-specific chaperone 1 [B. taurus]
ENSP00000259470-D2	11.12	1.06E-137	↓	cathepsin L1-like isoform X1 [B. mutus]
ENSP00000380074-D1	11.13	2.69E-138	↓	putative sodium-coupled neutral amino acid transporter 9 isoform X1 [Bison bison bison]
ENSBTAP00000023885-D2	11.17	3.00E-141	↓	hypothetical protein M91_19385, partial [B. mutus]
ENSP00000407583-D1	11.18	4.51E-142	↓	Putative leucine-twenty homeobox, partial [B. mutus]
ENSBTAP00000010324-D1	11.22	4.09E-145	↓	Polycomb protein EED, partial [B. mutus]
ENSP00000369411-D1	11.35	1.12E-307	↓	replication factor C subunit 3 [B. taurus]
yakA26736	11.41	1.65E-159	↓	beclin-2-like [B. mutus]
ENSBTAP00000026851-D2	11.48	1.58E-165	↓	E3 ubiquitin-protein ligase RNF138 isoform X2 [B. mutus]
ENSP00000389140-D1	11.50	1.63E-167	↓	Netrin receptor DCC, partial [B. mutus]
yakG029673	11.53	2.19E-169	↓	hypothetical protein M91_15046 [B. mutus]
ENSP00000220853-D1	11.53	1.70E-169	↓	Tetratricopeptide repeat protein 35, partial [B. mutus]
ENSBTAP00000028079-D1	11.59	2.26E-175	↓	ATP-dependent DNA helicase Q1, partial [B. mutus]
ENSBTAP00000052443-D1	11.70	8.64E-185	↓	vacuole membrane protein 1 [B. taurus]
ENSP00000383643-D14	11.75	1.23E-189	↓	Zinc finger protein 41, partial [B. mutus]
ENSP00000401475-D1	11.95	2.92E-210	↓	nucleoporin-like protein 2 [B. mutus]
ENSBTAP00000043967-D1	12.12	3.19E-229	↓	Spermatogenesis-associated serine-rich protein 1, partial [B. mutus]
ENSBTAP00000026904-D1	12.14	1.51E-231	↓	Protein S100-Z, partial [B. mutus]
ENSP00000292853-D5	12.36	9.78E-259	↓	hypothetical protein M91_07075 [B. mutus]
ENSBTAP00000009523-D1	12.45	8.42E-271	↓	tumor necrosis factor-inducible gene 6 protein precursor [B. taurus]
ENSBTAP00000050011-D1	12.98	0.00E+00	↓	zinc finger protein 135-like [B. mutus]
ENSP00000335578-D1	13.00	0.00E+00	↓	arginine-fifty homeobox [Bison bison bison]
ENSP00000371553-D1	13.05	0.00E+00	↓	NANOG neighbor homeobox, partial [B. mutus]
ENSBTAP00000050537-D1	13.05	0.00E+00	↓	hypothetical protein M91_02062, partial [B. mutus]
ENSP00000360389-D2	13.12	0.00E+00	↓	transmembrane protein 59 isoform X2 [B. taurus]
ENSP00000363344-D74	13.64	0.00E+00	↓	Zinc finger imprinted 3, partial [B. mutus]
ENSBTAP00000049936-D1	13.65	0.00E+00	↓	hypothetical protein M91_04889, partial [B. mutus]
ENSP00000351254-D1	13.74	0.00E+00	↓	putative methyl-CpG-binding domain protein 3-like 3 [B. mutus]

Morula vs Blastocyst				
ENSP00000259470-D2	-16.86	0.00E+00	↑	cathepsin L1-like isoform X1 [B. mutus]
ENSBTAP00000012987-D1	-16.43	0.00E+00	↑	placenta-specific gene 8 protein-like [B. mutus]
ENSBTAP00000001988-D1	-15.30	0.00E+00	↑	Keratin, type I cytoskeletal 18, partial [B. mutus]
ENSBTAP00000053629-D1	-15.25	0.00E+00	↑	zinc finger protein 42 homolog [Bison bison bison]
ENSP00000286371-D6	-15.15	0.00E+00	↑	Sodium/potassium-transporting ATPase subunit beta-3, partial [B. mutus]
ENSBTAP00000004079-D1	-14.94	0.00E+00	↑	Creatine kinase B-type, partial [B. mutus]
ENSBTAP00000010972-D1	-14.81	0.00E+00	↑	glutamyl aminopeptidase [B. mutus]
ENSP00000403333-D2	-14.80	0.00E+00	↑	Hepatitis A virus cellular receptor 1, partial [B. mutus]
ENSBTAP00000001867-D1	-14.73	0.00E+00	↑	DNA mismatch repair protein Msh6 [B. mutus]
ENSP00000259470-D1	-14.63	0.00E+00	↑	cathepsin L1-like [B. mutus]
ENSP00000358763-D1	-14.23	0.00E+00	↑	Chitinase-3-like protein 2 [B. mutus]
ENSBTAP00000004892-D1	-14.19	0.00E+00	↑	Transketolase, partial [B. mutus]
ENSBTAP00000029418-D3	-14.11	0.00E+00	↑	claudin-6 [B. mutus]
ENSBTAP00000008487-D1	-14.09	0.00E+00	↑	epithelial cell adhesion molecule precursor [B. taurus]
ENSBTAP00000023823-D2	-14.01	0.00E+00	↑	cationic amino acid transporter 3-like [B. mutus]
ENSP00000360389-D2	-13.91	0.00E+00	↑	transmembrane protein 59 isoform X2 [B. taurus]
ENSBTAP00000030173-D3	-13.84	0.00E+00	↑	Pregnancy-associated glycoprotein 2, partial [B. mutus]
ENSBTAP00000028178-D1	-13.66	0.00E+00	↑	DNA (cytosine-5)-methyltransferase 3A, partial [B. mutus]
ENSP00000220853-D1	-13.62	0.00E+00	↑	Tetratricopeptide repeat protein 35, partial [B. mutus]
ENSBTAP00000052791-D8	-13.62	0.00E+00	↑	Hemoglobin fetal subunit beta, partial [B. mutus]
ENSBTAP00000050011-D1	-13.55	0.00E+00	↑	zinc finger protein 135-like [B. mutus]
ENSP00000362748-D1	-13.54	0.00E+00	↑	methylcytosine dioxygenase TET1 [B. mutus]
ENSBTAP00000001633-D1	-13.48	0.00E+00	↑	myelin protein zero-like protein 2 precursor [B. taurus]
ENSBTAP00000011391-D1	-13.47	0.00E+00	↑	Putative E3 ubiquitin-protein ligase TRIML2, partial [B. mutus]
ENSBTAP00000046053-D2	-13.44	0.00E+00	↑	Guanine nucleotide-binding protein G(I)/G(S)/G(O) subunit gamma-11 [B. mutus]
ENSBTAP00000001053-D3	-13.31	0.00E+00	↑	folate receptor alpha precursor [B. taurus]
ENSP00000343274-D1	-13.27	0.00E+00	↑	Integrator complex subunit 8, partial [B. mutus]
ENSBTAP00000022806-D1	-13.16	0.00E+00	↑	hypothetical protein M91_15997, partial [B. mutus]
ENSBTAP00000023725-D1	-13.15	0.00E+00	↑	junctional adhesion molecule A precursor [B. taurus]
ENSBTAP00000009606-D1	-13.11	0.00E+00	↑	solute carrier family 2, facilitated glucose transporter member 5 [B. taurus]
ENSBTAP00000028498-D1	-12.83	7.93E-298	↑	protein S100-A14 [B. taurus]
ENSP00000308122-D1	-12.74	0.00E+00	↑	V-type proton ATPase 116 kDa subunit a isoform 4 [B. mutus]
ENSP00000239891-D1	-12.69	1.52E-278	↑	dolichyl-phosphate beta-glucosyltransferase isoform 1 [B. taurus]
yakA13537	-12.65	2.76E-273	↑	Protein phosphatase 1 regulatory subunit 14A [B. mutus]
ENSBTAP00000007273-D1	-12.55	8.63E-260	↑	glycine N-methyltransferase [B. mutus]
ENSP00000380074-D1	-12.54	1.19E-257	↑	putative sodium-coupled neutral amino acid transporter 9 isoform X1 [Bison bison bison]
ENSBTAP00000010324-D1	-12.52	1.33E-255	↑	Polycomb protein EED, partial [B. mutus]
ENSBTAP00000008936-D7	-12.51	0.00E+00	↑	Glycine cleavage system H protein, mitochondrial, partial [B. mutus]
ENSBTAP00000052443-D1	-12.50	4.91E-253	↑	vacuole membrane protein 1 [B. taurus]
ENSBTAP00000028091-D1	-12.45	1.01E-246	↑	V-type proton ATPase subunit d 2, partial [B. mutus]
ENSBTAP00000048801-D2	-12.45	1.29E-246	↑	hypothetical protein M91_06760, partial [B. mutus]
ENSBTAP00000018541-D1	-12.43	1.56E-244	↑	polypeptide N-acetylgalactosaminyltransferase 3 isoform X1 [B. taurus]
ENSP00000346635-D2	-12.42	1.07E-243	↑	ERO1-like protein alpha, partial [B. mutus]
ENSBTAP00000035309-D2	-12.42	1.19E-242	↑	WAP four-disulfide core domain protein 18-like [B. mutus]
ENSBTAP00000012238-D1	-12.41	1.92E-242	↑	Membrane-spanning 4-domains subfamily A member 8A, partial [B. mutus]
ENSP00000320083-D1	-12.38	1.92E-238	↑	N-acetylated-alpha-linked acidic dipeptidase 2, partial [B. mutus]
ENSBTAP00000020478-D1	-12.36	2.51E-236	↑	thymidylate kinase [B. mutus]
ENSP00000256319-D2	-12.35	1.25E-234	↑	probable ergosterol biosynthetic protein 28-like [B. mutus]
ENSP00000230914-D1	-12.34	5.42E-234	↑	28S ribosomal protein S30, mitochondrial [B. mutus]
ENSP00000358374-D1	-12.33	5.75E-232	↑	protein SCAF11 [B. mutus]
ENSP00000354649-D1	-12.32	1.54E-231	↑	magnesium transporter protein 1 [Camelus ferus]
ENSBTAP00000008128-D1	-12.31	2.96E-230	↑	Kinetochore protein Spc24, partial [B. mutus]
ENSBTAP00000045122-D1	-12.30	2.73E-229	↑	tissue factor pathway inhibitor [B. mutus]
ENSBTAP00000011539-D1	-12.19	0.00E+00	↑	kinesin-like protein KIF20A [B. mutus]

ENSP00000278829-D3	-12.17	4.82E-215	↑	Fatty acid desaturase 3, partial [B. mutus]
ENSBTAP00000044811-D1	-12.14	2.16E-211	↑	DNA-directed RNA polymerase I subunit RPA43 [B. mutus]
ENSP00000369411-D1	-12.13	0.00E+00	↑	replication factor C subunit 3 [B. taurus]
ENSBTAP00000006978-D1	-12.10	1.72E-207	↑	spermidine synthase [B. taurus]
ENSBTAP00000018774-D1	-12.10	8.06E-207	↑	prostaglandin G/H synthase 2 isoform X1 [B. mutus]
ENSBTAP00000028765-D1	-12.08	8.45E-205	↑	acid sphingomyelinase-like phosphodiesterase 3a [B. mutus]
ENSBTAP00000008580-D1	-12.05	7.27E-202	↑	NSFL1 cofactor p47 [B. taurus]
ENSP00000353098-D1	-12.01	8.95E-198	↑	hypothetical protein M91_15051, partial [B. mutus]
ENSP00000296869-D1	-11.99	2.11E-196	↑	Long-chain-fatty-acid--CoA ligase 6, partial [B. mutus]
ENSP00000262288-D1	-11.99	0.00E+00	↑	retinoid-inducible serine carboxypeptidase [B. mutus]
ENSBTAP00000042058-D1	-11.91	1.93E-188	↑	Sorting nexin-7, partial [B. mutus]
ENSBTAP00000027863-D2	-11.90	6.30E-187	↑	homeobox protein NANOG [B. mutus]
ENSP00000374268-D1	-11.88	1.60E-185	↑	Glucoside xylosyltransferase 2, partial [B. mutus]
ENSP00000347919-D1	-11.86	2.71E-183	↑	coiled-coil-helix-coiled-coil-helix domain-containing protein 8 [B. taurus]
ENSBTAP00000027762-D1	-11.86	4.66E-183	↑	Chromodomain-helicase-DNA-binding protein 1-like protein [B. mutus]
ENSBTAP00000018539-D2	-11.85	3.11E-182	↑	hypothetical protein M91_20172, partial [B. mutus]
ENSBTAP00000024611-D1	-11.83	6.20E-181	↑	uncharacterized protein C8orf47 homolog [B. mutus]
ENSP00000352284-D1	-11.81	3.72E-179	↑	transmembrane protein 139 [B. taurus]
ENSBTAP00000019865-D1	-11.79	1.73E-177	↑	interleukin-6 [B. mutus]
ENSP00000390625-D1	-11.79	1.18E-176	↑	heterogeneous nuclear ribonucleoprotein L-like isoform X1 [B. mutus]
ENSP00000372221-D1	-11.78	6.15E-176	↑	Serpin B5 [B. mutus]
ENSP00000350230-D1	-11.78	8.10E-176	↑	transmembrane protein 170A isoform X1 [Propithecus coquereli]
ENSBTAP00000044076-D1	-11.76	3.87E-174	↑	telomeric repeat-binding factor 1 isoform X1 [B. mutus]
ENSP00000263245-D1	-11.68	1.01E-167	↑	ADP-ribosylation factor GTPase-activating protein 3 isoform X1 [B. mutus]
ENSBTAP00000024487-D1	-11.64	2.04E-164	↑	methionine--tRNA ligase, cytoplasmic [B. mutus]
ENSBTAP00000021249-D1	-11.61	2.49E-161	↑	glutaredoxin-2, mitochondrial [B. mutus]
ENSBTAP00000018403-D1	-11.60	4.41E-161	↑	calmodulin-like protein 5 [B. taurus]
ENSBTAP00000024740-D1	-11.60	5.86E-161	↑	Replication factor C subunit 2 [B. mutus]
ENSP00000260403-D1	-11.57	2.40E-158	↑	transmembrane protein 62 [B. mutus]
yakA11407	-11.56	3.20E-157	↑	FXYD domain-containing ion transport regulator 4 [B. mutus]
ENSP00000402050-D8	-11.54	7.67E-156	↑	hypothetical protein M91_20981, partial [B. mutus]
ENSBTAP00000010428-D1	-11.53	1.82E-155	↑	probable sodium-coupled neutral amino acid transporter 6 isoform X1 [B. taurus]
ENSBTAP00000013299-D1	-11.50	1.95E-152	↑	Fanconi anemia group D2 protein isoform X1 [Bison bison bison]
ENSBTAP00000016778-D1	-11.47	1.17E-150	↑	uroporphyrinogen decarboxylase [B. taurus]
ENSP00000278544-D1	-11.47	1.17E-150	↑	alkaline ceramidase 3 [B. mutus]
ENSBTAP00000002023-D1	-11.46	2.20E-149	↑	sodium-dependent phosphate transport protein 2B [B. mutus]
ENSBTAP00000028693-D1	-11.45	7.15E-149	↑	Pyroglutamyl-peptidase 1-like protein, partial [B. mutus]
ENSBTAP00000001343-D1	-11.43	3.32E-147	↑	zinc transporter 5 [B. mutus]
ENSP00000304553-D1	-11.40	1.56E-145	↑	M-phase-specific PLK1-interacting protein [B. mutus]
ENSP00000401475-D1	-11.40	3.82E-145	↑	nucleoporin-like protein 2 [B. mutus]
ENSBTAP00000020664-D1	-11.38	1.84E-143	↑	rho GTPase-activating protein 7 isoform X1 [B. mutus]
ENSP00000357547-D1	-11.37	2.49E-143	↑	nuclear pore membrane glycoprotein 210-like isoform X1 [B. mutus]
ENSBTAP00000015586-D1	-11.37	3.77E-283	↑	insulin-like growth factor 2 mRNA-binding protein 1 isoform X1 [B. mutus]
ENSBTAP00000004969-D1	-11.37	8.23E-143	↑	ER degradation-enhancing alpha-mannosidase-like protein 2 isoform X1 [B. mutus]
ENSBTAP00000012255-D1	-11.36	4.97E-142	↑	Zinc transporter ZIP4 [B. mutus]
ENSBTAP00000007563-D1	-11.35	1.65E-141	↑	protein phosphatase 1K, mitochondrial isoform X1 [B. mutus]
ENSBTAP00000039004-D1	-11.35	3.01E-141	↑	Lysosomal protective protein, partial [B. mutus]
ENSP00000393599-D1	-11.34	4.06E-141	↑	Metastasis-associated lung adenocarcinoma transcript 1, partial [B. mutus]
ENSP00000416247-D1	-11.33	3.34E-140	↑	Desmocollin-2 [B. mutus]
ENSBTAP00000036928-D1	-11.32	3.75E-139	↑	UDP-glucuronosyltransferase 1-1, partial [B. mutus]
ENSP00000378856-D4	-11.32	3.75E-139	↑	TPA: galectin-14-like [B. taurus]
ENSP00000249356-D1	-11.31	9.28E-139	↑	dnaJ homolog subfamily B member 9 precursor [B. taurus]
ENSBTAP00000012050-D1	-11.30	1.05E-137	↑	cationic amino acid transporter 4 [B. mutus]
ENSBTAP00000038865-D1	-11.29	3.54E-137	↑	PC-esterase domain-containing protein 1B [B. mutus]
ENSP00000256857-D1	-11.28	8.80E-137	↑	Gastrin-releasing peptide, partial [B. mutus]

ENSBTAP00000012108-D1	-11.27	1.37E-135	↑	Trifunctional purine biosynthetic protein adenosine-3, partial [B. mutus]
ENSBTAP00000040833-D1	-11.26	4.66E-135	↑	Nucleoporin NDC1, partial [B. mutus]
ENSP00000368119-D1	-11.24	7.35E-134	↑	Galactose-1-phosphate uridylyltransferase, partial [B. mutus]
ENSBTAP00000018911-D1	-11.23	3.42E-133	↑	NEDD4 family-interacting protein 2 [B. mutus]
ENSBTAP00000005823-D1	-11.21	5.48E-132	↑	xaa-Pro aminopeptidase 1 isoform X2 [B. taurus]
ENSBTAP00000042937-D2	-11.20	8.85E-131	↑	rhombotin-1 [B. taurus]
ENSP00000350576-D49	-11.20	8.85E-131	↑	Zinc finger protein 709, partial [B. mutus]
ENSBTAP00000011257-D1	-11.19	3.06E-130	↑	microsomal glutathione S-transferase 1 [B. mutus]
ENSBTAP00000011613-D1	-11.18	7.76E-130	↑	selenide, water dikinase 1-like isoform X1 [B. mutus]
ENSBTAP00000012484-D1	-11.18	1.97E-129	↑	transmembrane protein 179B [B. mutus]
ENSP00000384477-D1	-11.18	1.97E-129	↑	interferon-related developmental regulator 1 isoform X1 [B. mutus]
ENSP00000349687-D1	-11.17	6.84E-129	↑	ganglioside GM2 activator [B. mutus]
ENSP00000399851-D1	-11.17	6.84E-129	↑	DNA polymerase subunit gamma-1, partial [B. mutus]
ENSP00000367263-D1	-11.16	2.60E-253	↑	neuroblast differentiation-associated protein AHNAK [B. mutus]
ENSBTAP00000028079-D1	-11.15	6.06E-128	↑	ATP-dependent DNA helicase Q1, partial [B. mutus]
ENSBTAP00000015396-D1	-11.15	6.06E-128	↑	glioma pathogenesis-related protein 1 [B. mutus]
ENSBTAP00000040146-D1	-11.11	3.23E-125	↑	anaphase-promoting complex subunit 1 isoform X1 [Bison bison bison]
ENSBTAP00000049936-D1	-11.08	1.96E-123	↑	hypothetical protein M91_04889, partial [B. mutus]
ENSP00000386516-D1	-11.08	3.70E-123	↑	Lysine-specific demethylase 3A [B. mutus]
ENSBTAP00000007083-D1	-11.07	6.97E-123	↑	Ribonuclease P/MRP protein subunit POP5 [B. mutus]
ENSP00000262482-D1	-11.07	1.81E-122	↑	E3 ubiquitin-protein ligase RNF167 isoform X1 [Physeter catodon]
yakA19103	-11.05	4.37E-121	↑	hypothetical protein M91_13117 [B. mutus]
ENSBTAP00000015701-D1	-11.04	8.26E-121	↑	signal peptide peptidase-like 2A [B. mutus]
ENSP00000352148-D1	-11.02	2.80E-119	↑	Protein S100-A5, partial [B. mutus]
ENSBTAP00000050877-D1	-11.01	5.32E-119	↑	pyridine nucleotide-disulfide oxidoreductase domain-containing protein 1 [B. mutus]
ENSBTAP00000011678-D2	-11.01	1.01E-118	↑	calpain-2 catalytic subunit precursor [B. taurus]
ENSP00000318770-D1	-11.01	1.01E-118	↑	aquaporin-11 isoform X1 [B. taurus]
ENSP00000350848-D1	-11.00	1.93E-118	↑	High mobility group nucleosome-binding domain-containing protein 5, partial [B. mutus]
ENSBTAP00000047788-D1	-10.99	1.33E-117	↑	amidophosphoribosyltransferase isoform X1 [B. mutus]
ENSBTAP00000010277-D1	-10.98	3.50E-117	↑	WW domain-containing transcription regulator protein 1 isoform X1 [Bison bison bison]
ENSBTAP00000014924-D1	-10.96	1.23E-115	↑	CD48 antigen [B. mutus]
ENSBTAP00000006215-D1	-10.94	6.27E-115	↑	G1/S-specific cyclin-E1 [B. taurus]
ENSBTAP00000014112-D1	-10.94	8.67E-115	↑	gem-associated protein 7 isoform X1 [B. taurus]
ENSBTAP00000006510-D1	-10.91	6.04E-113	↑	brain ribonuclease precursor [B. taurus]
ENSP00000343155-D2	-10.90	4.33E-112	↑	X-box-binding protein 1, partial [B. mutus]
ENSP00000368164-D1	-10.88	2.25E-111	↑	DNA replication licensing factor MCM8 [B. mutus]
ENSP00000269829-D55	-10.88	2.39E-219	↑	Zinc finger protein 789, partial [B. mutus]
ENSP00000296325-D1	-10.87	2.26E-110	↑	Alpha-2-macroglobulin receptor-associated protein [B. mutus]
ENSP00000369129-D1	-10.86	8.48E-110	↑	Desmoplakin, partial [B. mutus]
ENSBTAP00000020365-D1	-10.86	8.48E-110	↑	Argininosuccinate lyase, partial [B. mutus]
ENSP00000394419-D1	-10.86	8.48E-110	↑	Apolipoprotein A-I-binding protein, partial [B. mutus]
ENSBTAP00000005171-D1	-10.84	4.45E-109	↑	lipoyltransferase 1, mitochondrial isoform X1 [B. mutus]
ENSP00000370531-D4	-10.84	6.20E-109	↑	hypothetical protein M91_00655, partial [B. mutus]
ENSP00000341551-D1	-10.83	1.68E-108	↑	mothers against decapentaplegic homolog 4 [Ovis aries]
ENSBTAP00000009687-D1	-10.83	3.26E-108	↑	aminoacyl tRNA synthase complex-interacting multifunctional protein 2 [B. mutus]
ENSBTAP00000014256-D1	-10.83	3.26E-108	↑	Opa interacting protein 5 [B. mutus]
ENSP00000360363-D1	-10.82	8.88E-108	↑	C1GALT1-specific chaperone 1 [B. taurus]
ENSP00000366915-D1	-10.81	4.71E-107	↑	Progesterone-induced-blocking factor 1 [Pteropus alecto]
ENSBTAP00000011980-D1	-10.80	6.58E-107	↑	dnaJ homolog subfamily C member 25 precursor [B. taurus]
ENSBTAP00000003218-D1	-10.80	9.19E-107	↑	7,8-dihydro-8-oxoguanine triphosphatase, partial [B. mutus]
ENSBTAP00000012731-D1	-10.79	3.51E-106	↑	ribonuclease H2 subunit A [B. taurus]
ENSP00000261377-D1	-10.79	6.87E-106	↑	Putative RNA exonuclease NEF-sp, partial [B. mutus]
ENSBTAP00000035309-D3	-10.78	9.61E-106	↑	WAP four-disulfide core domain protein 18-like [B. taurus]
ENSBTAP00000016201-D1	-10.78	1.34E-105	↑	Dipeptidase 1 [B. mutus]
ENSBTAP00000022174-D1	-10.77	2.63E-105	↑	phosphotriesterase-related protein [B. taurus]

ENSBTAP00000018766-D1	-10.77	5.16E-105	↑	N-acetylglucosamine-1-phosphotransferase subunit gamma, partial [B. mutus]
ENSP00000403343-D1	-10.76	1.99E-104	↑	angio-associated migratory cell protein isoform X1 [B. taurus]
ENSBTAP00000024824-D1	-10.76	2.79E-104	↑	probable cation-transporting ATPase 13A5 [B. mutus]
ENSP00000292090-D1	-10.75	3.91E-104	↑	calfacilitin [B. taurus]
ENSBTAP00000014847-D1	-10.75	7.68E-104	↑	actin-related protein 8 [B. taurus]
ENSP00000333926-D1	-10.75	1.08E-103	↑	Multidrug resistance-associated protein 5 [B. mutus]
ENSBTAP00000011883-D1	-10.73	8.23E-103	↑	Macrophage metalloelastase, partial [B. mutus]
ENSP00000364126-D1	-10.72	2.28E-102	↑	DNA-(apurinic or apyrimidinic site) lyase 2 isoform X1 [B. mutus]
ENSBTAP00000029749-D1	-10.72	3.20E-102	↑	TPA: hypothetical protein BOS_16394 [B. taurus]
ENSP00000340811-D1	-10.71	4.49E-102	↑	Gap junction beta-5 protein [B. mutus]
ENSBTAP00000000364-D1	-10.71	8.88E-102	↑	Putative 2-oxoglutarate dehydrogenase E1 component DHKTD1, mitochondrial, partial [B. mutus]
ENSP00000371230-D1	-10.70	1.76E-101	↑	GTP:AMP phosphotransferase AK3, mitochondrial [B. taurus]
ENSBTAP00000003200-D1	-10.70	2.47E-101	↑	Protein FAM173A, partial [B. mutus]
ENSBTAP00000019763-D1	-10.70	2.47E-101	↑	metaxin-1 [B. mutus]
ENSBTAP00000032144-D1	-10.69	4.88E-101	↑	dol-P-Man:Man(5)GlcNAc(2)-PP-Dol alpha-1,3-mannosyltransferase [B. taurus]
ENSP00000391457-D1	-10.66	2.99E-99	↑	INO80 complex subunit C [B. mutus]
ENSBTAP00000011607-D1	-10.65	8.87E-195	↑	WD repeat-containing protein 89 [B. taurus]
ENSBTAP00000005252-D29	-10.65	8.87E-195	↑	Cell division protein kinase 4, partial [B. mutus]
ENSBTAP00000025456-D1	-10.65	0.00E+00	↑	excitatory amino acid transporter 3 [B. mutus]
ENSP00000370557-D1	-10.65	1.18E-98	↑	protein MIS12 homolog [B. taurus]
ENSP00000355649-D1	-10.64	4.72E-98	↑	S-phase response (cyclin related) [Equus caballus]
ENSBTAP00000009822-D1	-10.62	2.66E-97	↑	E3 ubiquitin-protein ligase MIB1 [B. taurus]
ENSBTAP00000038164-D1	-10.62	5.32E-97	↑	Voltage-dependent calcium channel gamma-like subunit [B. mutus]
ENSP00000262878-D1	-10.61	7.53E-97	↑	SAM domain and HD domain-containing protein 1 [B. mutus]
ENSP00000370424-D1	-10.60	3.02E-96	↑	CD81 antigen, partial [B. mutus]
ENSBTAP00000000230-D1	-10.59	8.57E-96	↑	RNA (guanine-9-)-methyltransferase domain-containing protein 2, partial [B. mutus]
ENSBTAP00000048643-D19	-10.59	8.57E-96	↑	zinc finger protein 404 [Bison bison bison]
ENSP00000288071-D1	-10.59	1.72E-95	↑	ATP-dependent RNA helicase DDX19A [B. taurus]
ENSBTAP00000041475-D1	-10.57	6.97E-95	↑	non-homologous end-joining factor 1 [B. mutus]
ENSBTAP00000006571-D1	-10.56	2.00E-94	↑	glycoprotein-N-acetylgalactosamine 3-beta-galactosyltransferase 1 [B. taurus]
ENSP00000206595-D1	-10.56	2.85E-185	↑	G2/M phase-specific E3 ubiquitin-protein ligase [B. mutus]
ENSBTAP00000051799-D1	-10.56	4.02E-94	↑	uncharacterized protein LOC613444 [B. taurus]
ENSBTAP00000006746-D1	-10.56	4.02E-94	↑	FK506-binding protein 15, partial [B. mutus]
ENSP00000317159-D1	-10.56	0.00E+00	↑	cytochrome c1, heme protein, mitochondrial [Equus przewalskii]
ENSBTAP00000020296-D1	-10.55	8.12E-94	↑	Beta-galactosidase, partial [B. mutus]
ENSBTAP00000035234-D1	-10.55	1.64E-93	↑	hypothetical protein M91_15734, partial [B. mutus]
ENSP00000340595-D1	-10.54	2.33E-93	↑	G-protein coupled receptor family C group 5 member C, partial [B. mutus]
ENSBTAP00000006167-D1	-10.54	2.33E-93	↑	alpha-2-macroglobulin [B. mutus]
ENSBTAP00000027226-D1	-10.54	3.32E-93	↑	Mediator of RNA polymerase II transcription subunit 29, partial [B. mutus]
ENSP00000356567-D2	-10.53	6.71E-93	↑	acyl-CoA-binding domain-containing protein 6 [B. taurus]
ENSP00000386105-D1	-10.53	6.71E-93	↑	DNA polymerase delta subunit 2 [B. mutus]
ENSBTAP00000041132-D1	-10.53	6.71E-93	↑	flap endonuclease GEN homolog 1 [B. mutus]
ENSBTAP00000034269-D2	-10.53	1.36E-92	↑	hypothetical protein CB1_001402132 [Camelus ferus]
ENSP00000298717-D1	-10.52	3.92E-92	↑	N6-adenosine-methyltransferase 70 kDa subunit [B. mutus]
ENSBTAP00000001651-D3	-10.52	3.92E-92	↑	transcription factor AP-2 gamma isoform X3 [Ovis aries musimon]
ENSBTAP00000023309-D1	-10.51	5.59E-92	↑	fetuin-B [B. mutus]
ENSBTAP00000002566-D1	-10.51	7.96E-92	↑	protein YIPF6 [B. taurus]
ENSBTAP00000053178-D1	-10.51	1.13E-91	↑	F-box only protein 11, partial [B. mutus]
ENSP00000364218-D1	-10.51	1.13E-91	↑	coronin-2A [B. taurus]
ENSBTAP00000017780-D2	-10.50	1.62E-91	↑	Palmitoyl-protein thioesterase 1, partial [B. mutus]
ENSP00000395465-D1	-10.50	3.28E-91	↑	Nuclear receptor coactivator 4, partial [B. mutus]
ENSBTAP00000026578-D1	-10.49	6.68E-91	↑	armadillo repeat-containing protein 8 [B. taurus]
ENSBTAP00000011963-D1	-10.49	9.52E-91	↑	U1 small nuclear ribonucleoprotein A, partial [B. mutus]
ENSP00000319152-D1	-10.48	1.94E-90	↑	Equilibrative nucleoside transporter 1, partial [B. mutus]
ENSBTAP00000049790-D39	-10.47	3.95E-90	↑	TPA: hypothetical protein BOS_26048 [B. taurus]

ENSBTAP00000012489-D1	-10.46	2.36E-89	↑	WD repeat-containing protein 74 [B. taurus]
ENSBTAP00000052710-D1	-10.45	3.37E-89	↑	Xaa-Pro dipeptidase, partial [B. mutus]
ENSBTAP00000026851-D2	-10.45	3.37E-89	↑	E3 ubiquitin-protein ligase RNF138 isoform X2 [B. mutus]
ENSP00000343819-D1	-10.45	6.90E-89	↑	homeobox protein OTX2 isoformX1 [Sus scrofa]
ENSP00000350961-D1	-10.44	2.02E-88	↑	hypothetical protein M91_02012 [B. mutus]
ENSP00000370736-D1	-10.43	4.14E-88	↑	Transmembrane protein 165, partial [B. mutus]
ENSBTAP00000045382-D1	-10.41	3.60E-87	↑	agouti-signaling protein-like [B. mutus]
ENSBTAP00000016926-D1	-10.41	3.60E-87	↑	C-C motif chemokine 26 [Bison bison bison]
ENSP00000389202-D1	-10.40	7.39E-87	↑	Myosin regulatory light chain 2, atrial isoform, partial [B. mutus]
ENSBTAP00000025912-D1	-10.40	1.06E-86	↑	Prostaglandin E synthase, partial [B. mutus]
ENSP00000216780-D1	-10.40	1.52E-86	↑	phosphoenolpyruvate carboxykinase [GTP], mitochondrial [B. mutus]
ENSP00000257700-D1	-10.40	1.52E-86	↑	RAD50-interacting protein 1, partial [B. mutus]
ENSP00000404365-D1	-10.39	2.19E-86	↑	uncharacterized protein C4orf21 homolog [B. mutus]
ENSBTAP00000023197-D1	-10.39	2.19E-86	↑	Disintegrin and metalloproteinase domain-containing protein 9 [B. mutus]
ENSBTAP00000037099-D1	-10.39	3.14E-86	↑	Tumor protein D52 [B. mutus]
ENSP00000304268-D91	-10.38	6.47E-86	↑	Zinc finger protein 26, partial [B. mutus]
ENSBTAP00000041352-D1	-10.38	6.47E-86	↑	thioredoxin-related transmembrane protein 2 [Ovis aries]
ENSBTAP00000008383-D1	-10.38	6.47E-86	↑	gamma-tubulin complex component 2 isoform X2 [B. mutus]
ENSBTAP00000002572-D2	-10.38	9.30E-86	↑	Small cell adhesion glycoprotein, partial [B. mutus]
ENSP00000356939-D1	-10.38	9.30E-86	↑	Low affinity immunoglobulin gamma Fc region receptor II [B. mutus]
ENSBTAP00000025792-D1	-10.36	8.26E-85	↑	TPA: hypothetical protein LOC539773 [B. taurus]
ENSBTAP00000026443-D1	-10.35	2.47E-84	↑	cytoplasmic polyadenylation element-binding protein 2 isoform X4 [Monodelphis domestica]
ENSP00000338218-D1	-10.34	3.55E-84	↑	E3 ubiquitin-protein ligase RNF213 isoform X1, partial [B. taurus]
ENSBTAP00000036320-D2	-10.34	3.55E-84	↑	Mpv17-like protein 2, partial [B. mutus]
ENSP00000365019-D4	-10.34	5.12E-84	↑	spindlin-1 isoform X1 [Ovis aries]
ENSP00000312981-D1	-10.34	5.12E-84	↑	U4/U6.U5 tri-snRNP-associated protein 2 [Oryctolagus cuniculus]
ENSBTAP00000016090-D1	-10.32	3.19E-83	↑	alpha-aminoadipic semialdehyde synthase, mitochondrial isoform X1 [B. mutus]
ENSBTAP00000000445-D1	-10.32	3.19E-83	↑	activity-dependent neuroprotector homeobox protein [B. mutus]
ENSBTAP00000022602-D1	-10.32	4.60E-83	↑	uncharacterized protein C12orf54 homolog [B. mutus]
ENSP00000290573-D1	-10.31	9.59E-83	↑	hexokinase-2 isoform X3 [B. taurus]
ENSBTAP00000003974-D1	-10.29	4.19E-82	↑	proteasomal ubiquitin receptor ADRM1 [Orcinus orca]
ENSP00000230792-D1	-10.29	6.05E-82	↑	peroxisomal NADH pyrophosphatase NUDT12 isoform X1 [B. taurus]
ENSBTAP00000001744-D1	-10.29	6.05E-82	↑	vacuolar protein sorting-associated protein 16 homolog isoform X1 [B. mutus]
ENSBTAP00000024598-D1	-10.29	8.75E-82	↑	major allergen Equ c 1-like [B. mutus]
ENSBTAP00000006002-D1	-10.28	1.83E-81	↑	GLIPR1-like protein 1 precursor [B. taurus]
ENSP00000359521-D1	-10.26	1.17E-80	↑	SLIT and NTRK-like protein 2 isoform X1 [B. mutus]
ENSBTAP00000015669-D1	-10.26	1.69E-80	↑	perilipin-3 isoform X1 [B. mutus]
ENSBTAP00000018534-D1	-10.26	1.69E-80	↑	Aprataxin, partial [B. mutus]
ENSBTAP00000012983-D1	-10.25	2.46E-80	↑	probable G-protein coupled receptor 171 [B. mutus]
ENSBTAP00000053625-D1	-10.25	3.15E-157	↑	Fanconi anemia group M protein [B. mutus]
ENSBTAP00000051999-D1	-10.24	7.51E-80	↑	ankyrin repeat domain-containing protein 26-like isoform X2 [B. taurus]
ENSBTAP00000024717-D1	-10.24	1.09E-79	↑	FL cytokine receptor, partial [B. mutus]
ENSP00000355511-D1	-10.24	1.09E-79	↑	rab proteins geranylgeranyltransferase component A 2 [B. mutus]
yakG008335	-10.23	3.34E-79	↑	glycine cleavage system H protein, mitochondrial-like [Pantholops hodgsonii]
ENSBTAP00000030898-D3	-10.21	1.49E-78	↑	Glycerol kinase, partial [B. mutus]
ENSBTAP00000007724-D26	-10.21	2.17E-78	↑	TPA: envelope glycoprotein-like [B. taurus]
ENSP00000356351-D1	-10.21	2.17E-78	↑	V-type proton ATPase subunit G 3 [B. mutus]
ENSP00000400184-D1	-10.19	6.67E-78	↑	hypothetical protein M91_17359 [B. mutus]
yakG029673	-10.19	9.71E-78	↑	hypothetical protein M91_15046 [B. mutus]
ENSBTAP00000044412-D1	-10.19	1.41E-77	↑	E3 ubiquitin-protein ligase RNF170 isoform X1 [Ovis aries]
ENSBTAP00000002311-D1	-10.18	2.06E-77	↑	Nuclear receptor 2C2-associated protein [B. mutus]
ENSBTAP00000006532-D3	-10.18	2.06E-77	↑	Actin, alpha cardiac muscle 1 [B. mutus]
ENSP00000340883-D1	-10.17	9.32E-77	↑	SWI/SNF-related matrix-associated actin-dependent regulator of chromatin subfamily B member 1 [B. mutus]
ENSBTAP00000025636-D1	-10.16	1.98E-76	↑	alpha-galactosidase A [B. mutus]
ENSBTAP00000042390-D1	-10.15	2.90E-76	↑	protein kintoun isoform X1 [B. mutus]

ENSP00000317272-D1	-10.15	2.90E-76	↑	Hepatocyte growth factor receptor [B. mutus]
ENSBTAP00000007993-D1	-10.15	2.90E-76	↑	Deoxyribonuclease-2-alpha, partial [B. mutus]
ENSBTAP00000016879-D1	-10.15	2.90E-76	↑	exosome complex component CSL4 isoform X1 [Bison bison bison]
ENSBTAP00000002542-D1	-10.15	4.23E-76	↑	histidine triad nucleotide-binding protein 3 [B. mutus]
ENSP00000417740-D1	-10.13	1.93E-75	↑	zinc finger protein 639 isoform X1 [B. mutus]
ENSP00000409452-D99	-10.13	2.82E-75	↑	Zinc finger protein 1-like protein, partial [B. mutus]
ENSBTAP00000049998-D1	-10.13	2.82E-75	↑	ARL14 effector protein-like [B. mutus]
ENSP00000306817-D1	-10.13	2.82E-75	↑	ribokinase-like [B. mutus]
ENSP00000298288-D1	-10.12	4.12E-75	↑	leucine-rich repeat protein 1 isoform X1 [B. mutus]
[truncated: 1,047,349 more chars]
